# Differential Ecological Specificity of Protist and Bacterial Microbiomes across a Set of Termite Species

**DOI:** 10.3389/fmicb.2017.02518

**Published:** 2017-12-19

**Authors:** Lena Waidele, Judith Korb, Christian R. Voolstra, Sven Künzel, Franck Dedeine, Fabian Staubach

**Affiliations:** ^1^Evolutionary Biology and Animal Ecology, Albert Ludwigs University of Freiburg, Freiburg, Germany; ^2^Division of Biological and Environmental Science and Engineering, Red Sea Research Center, King Abdullah University of Science and Technology (KAUST), Thuwal, Saudi Arabia; ^3^Max Planck Institute for Evolutionary Biology, Plön, Germany; ^4^Institut de Recherche sur la Biologie de l'Insecte, UMR 7261, Centre National de la Recherche Scientifique - Université de Tours, Tours, France

**Keywords:** termite, 16S rRNA gene sequencing, 18S rRNA gene sequencing, microbial ecology, evolution, host and microbe, symbiosis

## Abstract

The gut microbiome of lower termites comprises protists and bacteria that help these insects to digest cellulose and to thrive on wood. The composition of the termite gut microbiome correlates with phylogenetic distance of the animal host and host ecology (diet) in termites collected from their natural environment. However, carryover of transient microbes from host collection sites are an experimental concern and might contribute to the ecological imprints on the termite gut microbiome. Here, we set out to test whether an ecological imprint on the termite gut microbiome remains, when focusing on the persistent microbiome. Therefore, we kept five termite species under strictly controlled dietary conditions and subsequently profiled their protist and bacterial gut microbial communities using 18S and 16S rRNA gene amplicon sequencing. The species differed in their ecology; while three of the investigated species were wood-dwellers that feed on the piece of wood they live in and never leave except for the mating flight, the other two species were foragers that regularly leave their nests to forage for food. Despite these prominent ecological differences, protist microbiome structure aligned with phylogenetic relatedness of termite host species. Conversely, bacterial communities seemed more flexible, suggesting that microbiome structure aligned more strongly with the foraging and wood-dwelling ecologies. Interestingly, protist and bacterial community alpha-diversity correlated, suggesting either putative interactions between protists and bacteria, or that both types of microbes in the termite gut follow shared structuring principles. Taken together, our results add to the notion that bacterial communities are more variable over evolutionary time than protist communities and might react more flexibly to changes in host ecology.

## Introduction

Gut microbial communities of wood-feeding insects, such as roaches and termites, facilitate their ability to thrive on a wood diet. For the breakdown of lignocellulose from wood, lower termites depend on protists in their gut (Cleveland, 1923, 1925). With few exceptions, these protists belong to the order Oxymonadida (phylum Preaxostyla), which are specific to lower termite and wood-feeding roach guts, and the phylum Parabasalia. These protists are transmitted between termite colony members as well as from parents to offspring via proctodeal trophallaxis. This latter vertical transmission contributes to patterns of co-speciation between protist and the respective termite host (Bauer et al., 2000; Noda et al., 2007). Furthermore, most of these protists live in symbioses with ecto- and endosymbiotic bacteria (Brune and Dietrich, 2015). The bacterial microbiome of the termite gut is comprised of these protist-associated symbionts as well as free-living bacteria (Bauer et al., 2000; Breznak, 2002). In contrast to anaerobic protists, vertical transmission of bacteria between termites is not strict (Noda et al., 2009), although co-speciation can occur (Noda et al., 2007; Ohkuma, 2008; Ikeda-Ohtsubo and Brune, 2009; Desai et al., 2010).

The microbial communities (i.e., protists and bacteria) of termites share a common evolutionary origin with the microbial communities of cockroaches (Ohkuma et al., 2009; Schauer et al., 2012; Dietrich et al., 2014). Since the split of termites from cockroaches, lower termites have diversified and adapted to a variety of environments (Eggleton and Tayasu, 2001), life types (Abe, 1987), and diets (Donovan et al., 2001). Both, ecological factors like diet as well as termite phylogeny shape the gut microbiome (Boucias et al., 2013; Dietrich et al., 2014; Abdul Rahman et al., 2015; Tai et al., 2015). The contribution of diet to gut microbiome structure is larger in higher termites that have lost the protist part of their gut microbiome but adapted to more diverse diets (He et al., 2013; Mikaelyan et al., 2015a; Rossmassler et al., 2015).

The studies in which an effect of termite ecology in particular on the gut microbiome was found, largely focus on termites directly collected from the natural environment. Thus, transient microbes that reflect the microbial community of the termite collection site or food substrate but do not specifically interact with the termites, might contribute to the observed patterns. This includes in particular microbes that are ingested, but only passage the gut with the food without establishing a persistent population. The association with these transient microbes is less likely to involve the evolution of host-microbe interactions but rather an ecological side-effect that results from ingestion of diets that are habitats of microbial communities. In contrast, the microbiota that persist in the termite gut without frequent environmental replenishment is likely to be enriched for resident microbes that form long term and repeated host-microbe interactions (Sachs, 2006). An experiment in which the environment including host diet and the influx of new microbes into the gut is controlled, could help to remove many transient microbes. The microbes that persist would be enriched for resident microbes, and it would be possible to assess ecological imprints on the persistent microbiota. Yet, controlling diet for a set of termite host species that are adapted to different diets can lead to an artificial experimental setup that favors one host species over another. Therefore, an experimental setup with termites that primarily feed on the same diet under natural conditions but differ in another host ecological trait, lends itself more readily for this type of controlled experiments.

One such trait is termite life type; Abe (1987) coined this term suggesting that termite life types can be grouped into “one-piece types” and “separate-pieces types.” The one-piece type, also called wood-dwelling type, lives in the food resource, a piece of wood, and never leaves the nest except for the mating flight. The separate-pieces type, also called foraging type, leaves its nest to forage food. Leaving the nest to forage exposes the termites to a larger diversity of environmental microbes. Also, the soil on which foraging termites search for food and tunnel into can contain 5,000 times more microbes than even the nests of damp-wood-dwelling termites (800 colony forming units; Rosengaus et al., 2003; Vieira and Nahas, 2005). These microbes could challenge the resident microbiota potentially resulting in changes of the host-associated microbiota over evolutionary time. At the same time, exposure to a diversity of environmental microbes during foraging suggests contact with potential new symbiotic partners. This greater influx of environmental microbes recapitulates larger immigration rates in ecological models (e.g., MacArthur and Wilson, 1967; Shugart and Hett, 1973) and is expected to lead to faster turnover of ecological communities.

In this study, we sought to assess whether potential effects of termite ecology on the microbiome exist when focusing on the microbes that persist when environmental influx of microbes is controlled. To do this, we profiled protist and bacterial community structure of five termite species that all feed on wood under natural conditions, but differ with regard to life type (i.e., wood-dwelling and foraging types). In order to control for transient microbe contribution, all termites were kept with sterilized *Pinus* wood as the only food source for at least several weeks before the start of the experiment. Our assumption was that, when exposed to a common wood diet, phylogenetic distance should be the major remaining factor related to microbial community differences in gut microbiome structure. Conversely, if ecological factors were more important for the composition of the persistent termite gut microbiome, microbiomes should align more strongly with ecology than host phylogeny.

## Results

**Figure 1:**
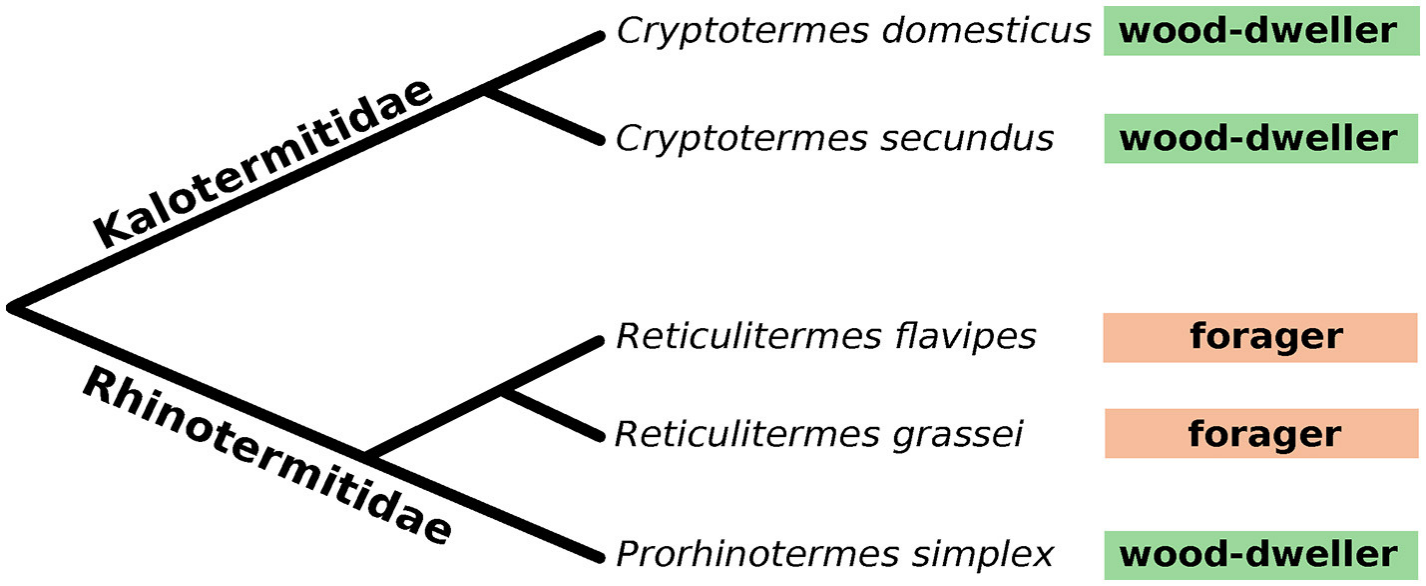
Phylogeny of the five lower termite species used in this study. Boxes following the species names indicate life type. Wood-dwellers never leave their nest except for the mating flight, foragers leave their nests to forage for food. Branch lengths not drawn to scale. See also Figure S5 for a cytochrome-c-oxidase sequence-based phylogenetic tree.

We profiled the protist and bacterial communities of two wood-dwelling species from the Kalotermitidae (*Cryptotermes secundus, Cryptotermes domesticus*), a wood-dwelling species from the Rhinotermitidae (*Prorhinotermes simplex*) that dwells in dead logs in coastal areas, and two Rhinotermitidae species that switched from the ancestral wood-dwelling life type to foraging (*Reticulitermes flavipes, Reticulitermes grassei*) (Figure 1). Given the substantial variation of microbial communities between different colonies reported previously (Hongoh et al., 2005, 2006; Benjamino and Graf, 2016), we analyzed five *C. domesticus*, eight *C. secundus*, seven *P. simplex*, five *R. flavipes*, and four *R. grassei* replicate colonies (Table 1). To ensure even coverage across samples, bacterial and protist sequence data was subsampled (rarefied) to the number of sequences that were observed in the sample with lowest coverage resulting in 32,824 and 22,285 sequences per sample for bacteria and protists, respectively (see also Table S1).

**Table 1:** Overview of sampling effort and alpha-diversity estimates.

	***C. domesticus***	***C. secundus***	***P. simplex***	***R. flavipes***	***R.grassei***	**wood-dweller**	**forager**	***P***
No. of replicate colonies	5	8	7	5	4	20	9	
Mean # of sequences per sample18S	107,394	108,253	103,180	85,504	48,781	113,955	96,994	
Mean # of sequences per sample 16S	54,031	60,026	63,172	59,487	43,534	83,773	74,538	
**PARABASALID PROTIST COMMUNITY**								
# OTUs	31 ± 5	20 ± 3	21 ± 2	35 ± 3	36 ± 4	23 ± 2	36 ± 2	0.001
Shannon's H	1.18 ± 0.2	1.35 ± 0.1	0.7 ± 0.01	1.63 ± 0.04	1.23 ± 0.2	1.08 ± 0.1	1.45 ± 0.1	0.03
Inequality #OTUs/e^H^ (Jost, 2010)	10.5 ± 2.3	5.1 ± 0.4	10.3 ± 0.9	7.0 ± 0.5	10.5 ± 1.1	8.3 ± 0.9	8.5 ± 0.8	0.47
**BACTERIAL COMMUNITY**								
# OTUs	450 ± 20	502 ± 33	490 ± 27	697 ± 53	559 ± 124	485 ± 17	636 ± 63	0.04
Shannon's H	3.53 ± 0.1	3.44 ± 0.1	3.14 ± 0.2	4.29 ± 0.1	3.71 ± 0.4	3.36 ± 0.1	4.03 ± 0.2	0.005
Inequality #OTUs/e^H^ (Jost, 2010)	13.4 ± 1.5	15.9 ± 1.1	22.3 ± 3.1	9.5 ± 0.9	13.6 ± 3.3	17.5 ± 1.4	11.3 ± 1.6	0.002

*First row contains total number of colonies. All other rows represent means and standard error of the mean. P-values designate the comparison of alpha-diversity measures of the protist and bacterial microbiomes between wood-dwelling and foraging termite host species using Mann-Whitney-U tests. Sequencing depth was subsampled to the same number of sequences for each sample to ensure consistent alpha-diversity estimates. green, wood-dweller; orange, forager*.

### Diversity of microbial communities

OTU (Operational Taxonomic Unit based on 97% sequence identity) based analysis of sequences revealed that the termite samples harbored between 10 and 59 different protists from the Parabasalia phylum per sample. Note that we had to exclude protists from the order Oxymonadida because their 18S rRNA gene escaped reliable amplification. In comparison, bacterial diversity was more than 10 times higher with 342 to 855 different bacterial OTUs per sample. A difference in alpha-diversity between protist and bacterial communities was also reflected by Shannon alpha-diversity indices that ranged from 0.67 to 2.0 for protist communities and from 2.63 to 4.61 for bacterial communities.

Bacterial as well as protist diversity was higher in *Reticulitermes* hosts (Mann-Whitney-*U*-test on Shannon diversity: *P* = 0.005, and *P* = 0.03 respectively, Table 1, Figure S1 for rarefaction curves) when compared to the wood-dwelling species *C. domesticus, C. secundus*, and *P. simplex*. Members of the genus *Reticulitermes* are foraging species. Higher microbial diversity was expected in foraging termites, assuming that species, frequently leaving their nests to forage, have a higher probability of encountering and acquiring new microorganisms. Environmental acquisition should not only increase alpha-diversity, but the inherent stochasticity in the acquisition process should also add to its variance. In fact, the variance in the number of bacterial (*P* = 0.001, Levene's test), but not protist OTUs (*P* = 0.55) was increased in the foraging termites in our study.

Next, we wanted to test if the increased alpha-diversity in *Reticulitermes* might be confounded by inefficient removal of transient microbes. Assuming that transient microbes should be at low frequencies, they should add to inequality in the distribution of OTUs in *Reticulitermes*. In contrast, using the inequality factor (S/eH) proposed by Jost (2010), we found that inequality was increased in the bacterial communities of the wood-dwelling species in our study (*P* = 0.0003, Mann-Whitney *U*-test). A possible explanation is that the bacterial communities of the wood-dwelling species were often dominated by individual OTUs (OTU1 in *Cryptotermes* and OTU18 in *P. simplex*, Table S1). No difference in inequality was found for protists.

**Figure 2:**
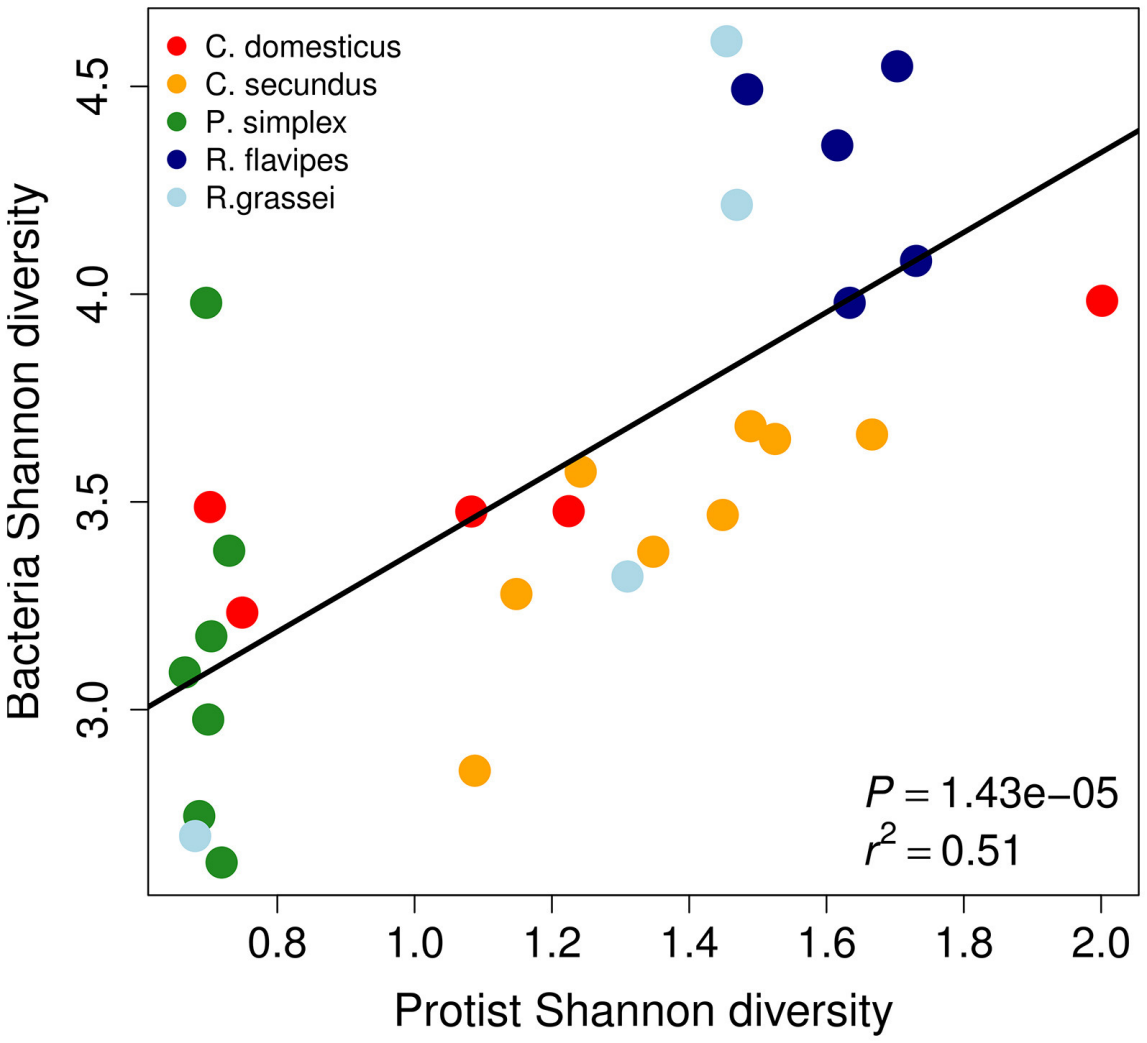
Shannon (H) diversities of protists and bacteria of the termite gut microbiome are correlated across termite species. Each dot represents a sample from a different termite colony. *P*-value and *r*^2^ were determined using Pearson's product-moment correlation.

Because protists in the termite gut often carry bacterial symbionts, we were interested to assess whether protist and bacterial diversities correlated across termite species. We found a highly significant correlation between protist and bacterial alpha-diversity measures across termite host species using Shannon's H (Figure 2, Pearson's product-moment correlation *P* = 1.43 × 10^−5^, *r*^2^ = 0.51) as well as between the total number of protist and bacterial taxa (OTUs Figure S2A, Pearson's product-moment correlation: *P* < 0.05, *r*^2^ = 0.19). Strongly positive correlation coefficients were also found within host species except for *P. simplex* and *R. flavipes* that showed only little variation in protist diversity (Figure S2B).

### Composition of microbial communities

**Figure 3:**
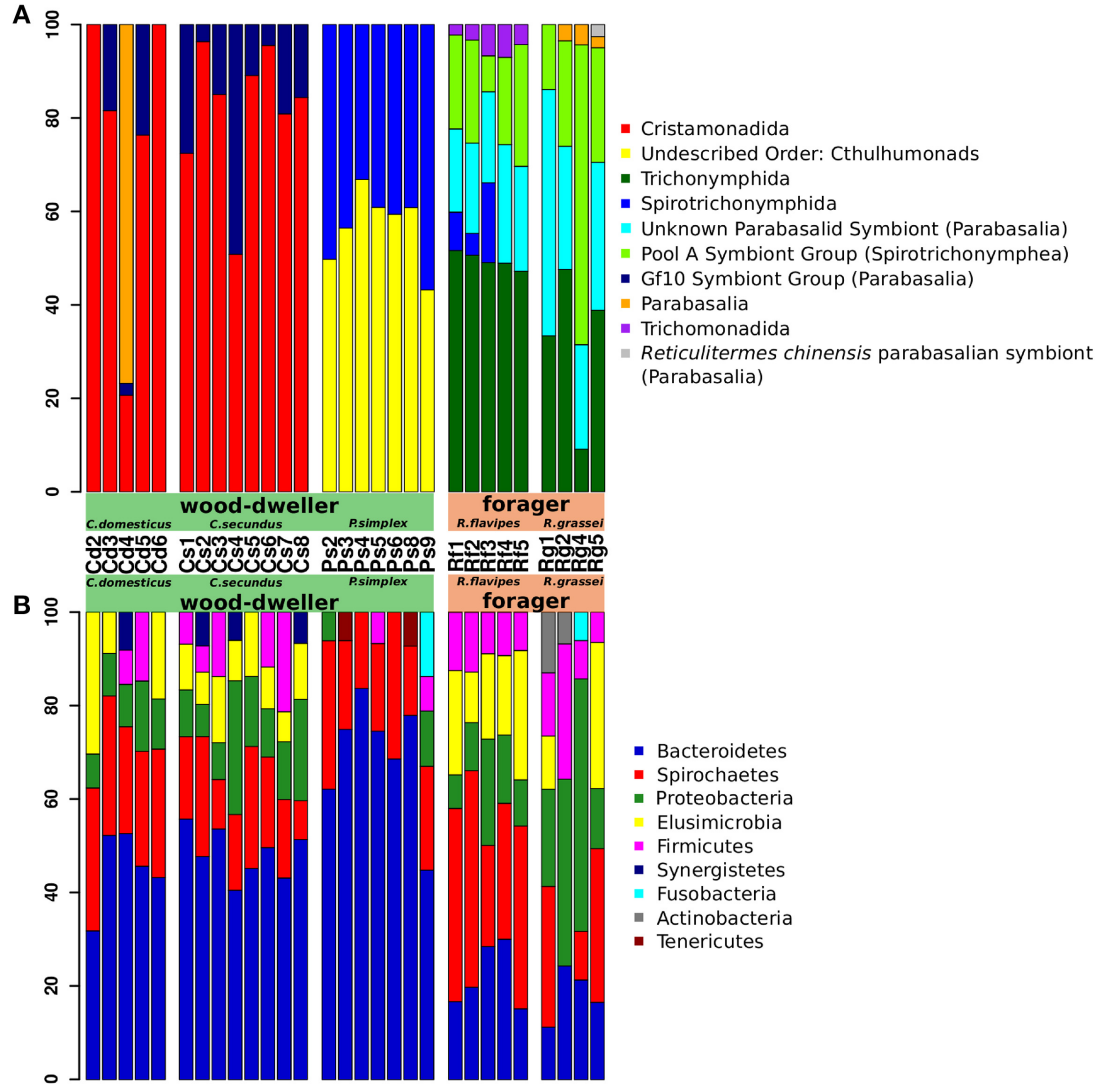
Termite gut microbiome community composition. Relative abundance of protist and bacterial taxa was assessed using taxonomic profiling of 18S rRNA and 16S rRNA gene amplicon sequences. Taxa are sorted by abundance. **(A)** Protist community profiles at the order level. Sequences that could not be classified to order with sufficient bootstrap support were labeled with the next higher taxonomic rank with sufficient support. Only orders with an abundance of >2% are shown. Taxa with unconventional names (e.g., “Gf10 Symbiont group”) are followed by the next higher classification in parentheses. “Unknown Parabasalid Symbiont” refers to reference sequences of symbionts isolated from *Reticulitermes speratus* with yet unidentified taxonomy (Ohkuma et al., 2000). Sequences of the unidentified “Gf 10 symbiont group” were first isolated from *Glyptotermes fuscus* (Ohkuma et al., 2000) and were also found in other Kalotermitids (Strassert et al., 2009). Note that the taxon “*Reticulitermes chinensis* parabasalian symbiont” was introduced here for clarification. Both are misleadingly classified as “Cryptotermes symbiont group” in the SILVA taxonomy, although being isolated from Rhinotermitid hosts (SILVA JX975351: isolated from *Heterotermes tenuis* (James et al., 2013); SILVA GU461593: isolated from *R. chinensis*, unpublished). **(B)** Bacterial community composition profiles at the phylum level. Only phyla with a relative abundance of >5% are shown. Cd, colonies of *C. domesticus;* Cs, *C. secundus;* Ps, *P. simplex;* Rf, *R. flavipes;* Rg, *R. grassei*.

The four most common protist orders in our dataset were Cristamonadida (40.3%), Cthulumonads (14.8%), Trichonymphida (13.4%), and Spirotrichonymphida (12.7%). Members from all of these taxa carry bacterial symbionts (Dolan, 2001; Noda et al., 2003; Ohkuma et al., 2007; Ikeda-Ohtsubo and Brune, 2009; Desai et al., 2010; James et al., 2013) (Figure 3A). The most common bacterial phyla were Bacteroidetes (40.6%), Spirochaetes (21.9%), Proteobacteria (12.4%), and Elusimicrobia (9.1%). Except for the Proteobacteria, all of these bacterial phyla contain symbionts of lower termite-associated protists (e.g., *Treponema* and *Endomicrobium*) (Noda et al., 2005, 2006; Strassert et al., 2010; He et al., 2013) (Figure 3B). The prevalence of protist-associated bacteria among the persistent microbiota could help explain the correlation of protist and bacterial alpha-diversity shown in Figure 2.

Of particular note is that many bacterial genera contained host-specific bacterial taxa. For instance, sequences from the spirochaete *Treponema Ia* fell into several distinct termite host-specific OTUs (e.g., OTUs 2, 3, and 33, see File S1). This was also true for sequences from *Endomicrobium* (OTUs 7, 12, and 20), or sequences from *Candidatus Armantifilum* (OTUs 1, 31, and 48). The prevalence of host-specific bacterial species strongly suggested that the microbial communities of the termite hosts were distinct. However, at the same time, the relatedness of these bacteria, as reflected by their common classification into genera, supported the notion that microbial communities shared a phylogenetic history (Schauer et al., 2012; Dietrich et al., 2014; Tai et al., 2015), and differences may have been the result of co-diversification of hosts and associated bacteria.

### Factors contributing to termite gut microbiome structure

We incorporated the phylogenetic relationship between bacteria from different termite species community distances by analyzing environment-specific phylogenetic branch lengths of all community members with the UniFrac metric (Lozupone and Knight, 2005). As our assumption was that ecologically relevant microbes should not be rare, we applied the weighted UniFrac distance metric (Lozupone et al., 2007) that takes abundance of microbes into account. We were especially interested in community clustering under controlled dietary and environmental conditions.

**Figure 4:**
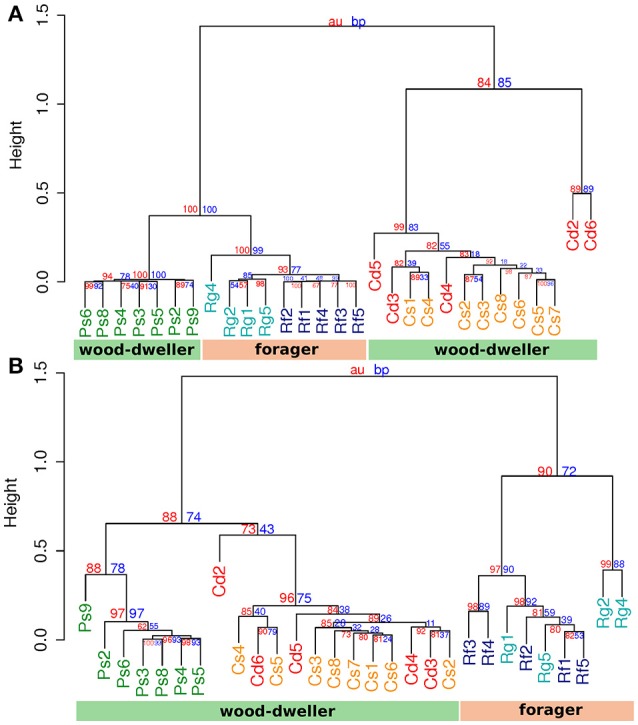
Cluster analysis by protist and bacterial community distances. **(A)** Cluster dendrogram based on protist community distances. **(B)** Cluster dendrogram based on bacterial community distance. Cd, colonies of *C. domesticus*; Cs, *C. secundus*; Ps, *P. simplex*; Rf, *R. flavipes*; Rg, *R. grassei*. Blue numbers, bootstrap probability; red numbers, approximate unbiased probability (Suzuki and Shimodaira, 2006). The weighted UniFrac metric was used. Clustering was performed using the UPGMA algorithm.

Protist communities clustered according to termite host family (Figure 4A). The protist communities of *R. flavipes* and *R. grassei* were distinct, while there was no consistent difference between the protist communities of *C. secundus* and *C. domesticus*, potentially due to the high variability of protist communities within *C. domesticus*. In contrast, bacterial communities of the wood-dwelling species *C. domesticus, C. secundus*, and *P. simplex* clustered separately from the bacterial communities of the foraging *Reticulitermes* (Figure 4B) and together with the bacterial communities of the wood-dwelling sub-social cockroaches of the genus *Cryptocercus* (Figure S3). The latter constitutes the sister lineage of termites. Hence, the clustering of the microbiome of the wood-dwelling species in our study with that of the wood-dwelling sister lineage confirmed the potentially ancestral state of the microbiome of the wood-dwelling host species in our study (Nalepa, 2015). Despite the clustering of bacterial communities according to life type and not host family, *Prorhinotermes* and *Cryptotermes* clustered separately. This suggested that a phylogenetic effect also contributed to differentiation of the bacterial microbiomes.

**Figure 5:**
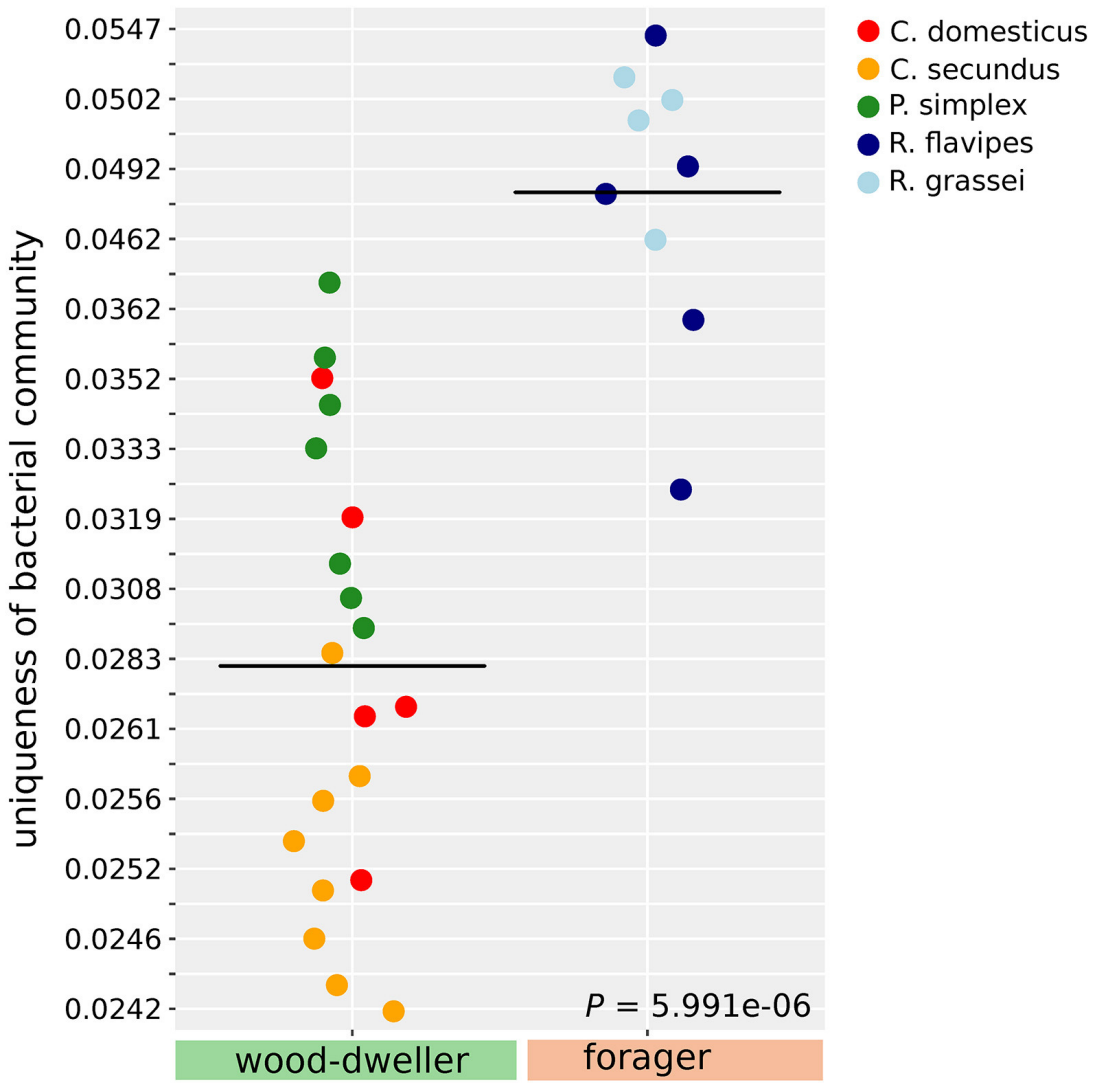
Uniqueness of bacterial community composition measured as LCBD (Legendre and De Cáceres, 2013). Uniqueness of communities was compared using the Mann-Whitney *U*-test.

The separate clustering of *Reticulitermes* bacterial microbiomes could have been driven by increased exposure to environmental microbes due to foraging behavior of the termite host that lead to increased independent acquisition of bacteria from the environment. If this was the case, independent acquisition should have led to more private microbes, and hence, increased uniqueness of *Reticulitermes* bacterial microbiomes. We used LCBD (Local contribution to Beta-Diversity, Legendre and De Cáceres, 2013), a composition-based measure for the uniqueness of communities, to test whether the uniqueness of microbiomes differed between foraging and wood-dwelling species in our study. We found no significant difference for protist communities. However, the bacterial communities of *Reticulitermes* were significantly more unique (*P* < 6 × 10^−6^, Mann-Whitney *U*-test, Figure 5).

### Bacteria specific to *Reticulitermes*

The distinct clustering of *Reticulitermes* bacterial communities suggests that they align with differences in the ecology between this termite host and all the others. Hence, identification of the bacteria that have contributed to the separate clustering of *Reticulitermes* communities might provide insight into the factors that have driven bacterial community differentiation between termite hosts. In order to identify *Reticulitermes* specific bacteria, we determined bacterial indicator taxa that were significantly associated with *Reticulitermes*. A full list of indicator taxa (97% identity OTUs) that were indicative of *Reticulitermes* and correlated with the difference in life type between this genus and the other termites in the study is available in Table S2. From this set of indicator taxa, we were in particular interested in those taxa that were related to environmental bacteria or bacteria found in non-dictyopteran hosts. Our reasoning was that these bacteria that were not shared with other termites might have been acquired more recently on the *Reticulitermes* lineage and were potentially connected to the evolutionary switch in life type from wood-dwelling to foraging. We were also interested in bacterial taxa that were closely related to bacteria found in other foraging Dictyoptera, but not in wood-dwelling species. Association with such bacteria might reflect the foraging life type.

In order to identify bacteria among the *Reticulitermes* specific indicator taxa that have close relatives in the environment, non-dictyopteran hosts, or other foraging Dictyoptera, we performed a phylogenetic analysis. This analysis included the *Reticulitermes* indicator taxa from Table S2, for which we wanted to find the close relatives. Furthermore, the analysis included, as potential origin of our indicator taxa, all bacteria for which we found at least 100 sequences in all other termites, as well as all sequences in the DictDB database (Mikaelyan et al., 2015b) (clades relevant for the indicator species are drawn in Figure S4, the full tree as Newick file can be found in File S2). This allowed us to detect several indicator OTUs that are most closely related to bacteria from the environment and non-dictyopteran hosts, suggesting acquisition of these bacteria on the *Reticulitermes* lineage. In some cases the closest relatives were found exclusively in other foraging termites (e.g., OTU 58), suggesting independent acquisition by other foraging termites. OTUs that match these criteria and comprised at least 1000 sequences are shown in Table 2.

**Table 2:** Bacterial Indicator taxa of *Reticulitermes* termites.

**Indicator OTU**	**#seqs**	**Taxonomy**	**Closest relative from (DictDB ID)**	**Representative close relatives from (F = foragers, E = environment)**	**DictDB IDs of rep. close relatives**
OTU56	5,936	Desulfovibrionaceae; Gut_cluster_1	Several equally close	*Reticulitermes santonensis* (F) *Homo sapiens* Microbial fuel cell (E)	UltB4523 UltB4524 Ultpro53
OTU77	2,726	Lachnospiraceae; Incertae_Sedis_34	Hind gut wall of *R. santonensis* (Ult21521)	*Coptotermes formosanus* (F) *Mus musculus* cecum *Rattus* gut	UltFir26 Ult21516-19 Ult21520
OTU58	2,505	Lactococcus_3	Mid gut of *R. santonensis* (Ult24789)	Electro active biofilm (E) anoxic zone of waste water (E) *Homo sapiens* stool defaunated *R. santonensis* (F) *Odontotermes yunnanensis* (F)	LatPisci latraff2 Ult24787 Ultlac87 UncUn200
OTU182	2,157	Desulfovibrionaceae; Gut_cluster_1	Mid gut of *R. santonensis* (UltB7663)	*Odontotermes yuannensis* (F)*Panesthia angustipennis* (F) hind gut *Salganea esakii* (F)	UncUn450 PahggY35 S0114542
OTU120	1,979	Treponema_I	*R. flavipes* (Ulttr346)	*Coptotermes formosanus* (F) *Neotermes koshunensis* (F)	UltSpi95 UltSpi16
OTU307	1,552	Stenoxybacter	*R. flavipes* (StnAceti)	*Microcerotermes* sp. (F)	UltTrB168

*Shown are indicator bacterial taxa (OTUs) of the host termite genus Reticulitermes with an indicator value larger than 99.9 and P < 0.001 that comprise at least 1,000 sequences and have no known close bacterial relatives in wood-dwellers. #seqs, number of sequences in the corresponding indicator OTU; DictDb ID refers to the label set in the DictDb_v3 database. See Figure S4 and File S2 for phylogenetic position of the indicator OTUs*.

## Discussion

Studies that analyze protist and bacterial communities across termite species are still rare, in particular studies monitoring environmental and dietary conditions to control for transient microbes. In this study we found that (i) protist and bacterial diversities correlated, and (ii) the clustering of protist communities was different from that of bacterial communities.

### Importance of study setups that control for environmental microbes for evolutionary studies and their applicability to termite systems

For a deeper understanding of how ecology shapes host-microbe interaction in the course of evolution, it is crucial to focus on the resident host-associated microbiome. Members of the resident microbiome are expected to form evolutionary relevant relationships with the host through long term and repeated interactions (Sachs, 2006). Transient gut microbes, by comparison, might simply be ingested with the food and pass the gut without significant host interaction, potentially obscuring the overall signal in previous studies.

While a phylogenetic signal remains visible in many studies (Boucias et al., 2013; Dietrich et al., 2014; Abdul Rahman et al., 2015; Tai et al., 2015), more subtle ecological imprints on the microbiota might be obscured. In some cases, environmental microbes might correlate with host ecology, host phylogenetic relatedness, or both. As a consequence, the transient microbiota can generate a signal that can misleadingly be interpreted as an evolutionary imprint on the microbiome. In fact, such a signal would only represent ecological side effects such as the microbial community composition at the collection site or the host's last meal.

Under that premise, and given that a significant proportion of the termite gut microbiota might be transient (Tai et al., 2015), it seems plausible to assume that termites collected from different natural environments and food substrates carry different transient microbes, and thus, analyses of termite microbiomes might suffer from the above-mentioned effects. Therefore, more studies that focus on the resident termite microbiome are required to formulate and test evolutionary and ecological hypotheses.

In this study, we took a first step toward focusing on the resident microbiome by controlling environment, diet, and the influx of microbes into the intestinal tract under laboratory conditions. In order to control for the varying contributions of transient microbes to the gut community due to different diet sources, we developed a setup to study diverse termite species under common conditions in the laboratory by keeping all termites on *Pinus* wood for at least several weeks before the start of the experiment. While we could not exclude that microbes that were acquired by the termites from their natural environment, i.e., during their lifetime before collection, persisted in the gut, the average gut retention time of 24–26 h in termites (Breznak, 1984; Li et al., 2017) is small in comparison to the here-applied 6-week treatment. Hence, we would assume that our approach is effective in removing the majority of transient microbes. In addition, Huang et al. (2013) found that significant adjustments of the lower termite gut microbiome take place in a 6-week time frame. Considering these time frames, our assumption is that the majority of excretable material, including remaining food, microbes, and microbial DNA was excreted, and that adjustments of the gut microbiota took place. The microbiome that persisted after the treatment should be enriched for microbes that share an evolutionary history with the host. This can be resident microbes that have shared a longer evolutionary history with the host, but also microbes that were more recently acquired along the different host lineages and that might reflect host ecology.

One of the limitations of such a setup is that the termite host species range is limited in comparison to relatively straightforward *in situ* collection studies. The reason is that this experimental approach requires keeping live specimens in the laboratory under previously tested conditions to ensure that all termites investigated fare well under the chosen conditions. For the same reasons the geographical range spanned by the sampling can be limited to locations from which an acquisition of intact colonies of live animals is feasible. This might limit the degree to which variation within a species can be represented in such experiments. While some degree of within-species variation may be overlooked, replicate colonies of the different termite species from their typical environment can help to increase representativeness.

Despite these limitations, experiments that control the environment in the laboratory are essential, especially for non-model organisms where it is difficult to culture hosts and microbes independently, and hence to perform controlled infection experiments with defined inocula on sterile hosts, like in lower termites. Such a study setup allows focusing on resident microbes and as such provides a tool to study the role of the microbiome in host function and in particular in host evolution.

### Protist communities cluster differently than bacterial communities

Under controlled environmental and dietary conditions we found that protist communities showed a stronger phylogenetic imprint than bacterial communities. While a phylogenetic imprint on the bacterial microbiome was still visible from the clustering according to host genus, higher level clustering aligned with life type. The wood-dwellers in our study had more similar bacterial communities, leaving foraging *Reticulitermes* communities distinct.

Most foraging termite species are exposed to microbe rich soil (4 × 10^6^ colony forming units, Vieira and Nahas, 2005), carrying on average 5,000 times more microbes than even the nests of damp-wood-dwelling termites (800 colony forming units, Rosengaus et al., 2003). This exposure to a large number of potential new members of the microbiome could, over evolutionary time, have fueled a higher bacterial species turnover in foraging *Reticulitermes* and driven differentiation between their bacterial communities and that of wood-dwellers. This effect of the number of new colonizers or immigrants on species turnover has been long known in ecological theory (e.g., MacArthur and Wilson, 1967; Shugart and Hett, 1973). A model of higher random influx was supported by the increased uniqueness of the bacterial microbiomes of foragers in our study (Figure 5). An increased influx of microbes into the microbiome of foragers could be seen akin to an increased influx of new mutations into a genome. A higher mutation rate leads to faster evolution as should a higher influx of microbes lead to faster divergence of microbiomes. Assuming that the removal of transient microbes was sufficiently effective (see our considerations above), the distinct *Reticulitermes* microbiomes in our study could be indicative of this faster microbiome divergence.

Interestingly, the ancestors of the wood-dwelling *Prorhinotermes* might have been exposed to similarly diverse microbes because there is some evidence that *Prorhinotermes* reverted back from foraging to wood-dwelling (Legendre et al., 2008; Bourguignon et al., 2015). If the ancestors of *Prorhinotermes* were indeed foragers, the propensity for random change of microbial communities over evolutionary time for *Reticulitermes* and *Prorhinotermes* would be similar. This notion was supported by beta-diversity analysis (Figure 5) that indicated that the uniqueness of *Prorhinotermes* bacterial microbiomes is higher than that of the wood-dwelling *Cryptotermes* (*P* = 0.0017, Mann-Whitney *U*-test). Given similar potential for random change of the microbiota in both genera, invoking life type-related selection, either driving the divergence of the *Reticulitermes* microbiota or the convergence of the *Prorhinotermes* microbiota back to that of wood-dwelling species or both, appears plausible to explain the distinct bacterial communities of *Reticulitermes*.

Although our results were consistent with life type-related changes in the microbiome, other ecological differences specific to *Reticulitermes* could potentially determine their distinct bacterial gut microbiota. For example, differences in colony size, climate, or subtle differences in diet under natural conditions (all species investigated here primarily feed on wood) could add to the divergence of *Reticulitermes* bacterial communities. It could also be argued that being kept in the laboratory on a *Pinus* wood diet would have different effects on the microbiota of the different termite species. While we cannot completely rule out this possibility, it should be considered that all species in our study fare well on this food source over extended periods of time. Hence, it appears unlikely that diet-related effects severely impaired the gut microbiota. *Pinus* is a natural food substrate for *P. simplex* and *Reticulitermes* and has extensively been tested as substrate for *C. secundus* (Korb and Lenz, 2004). It should also be considered that close relatives to all abundant indicator OTUs for *Reticulitermes* were identified in *Reticulitermes* in earlier studies (see bacterial phylogenetic trees in Figure S4 and File S2). This supports the notion that differences between *Reticulitermes* bacterial communities and those of the other species are driven by bacteria that also occurr in *Reticulitermes* under natural conditions, and hence, are unlikely to result from laboratory artifacts. Finally, ineffective transient removal in *Reticulitermes* might have confounded the divergence of bacterial microbiomes. If transients were not removed effectively in *Reticulitermes*, we would have expected an overreprensentation of low frequency microbes leading to higher inequality of microbe abundance. However, we found no evidence for this. Instead, inequality was increased in wood-dwelling species, probably due to the high abundance of protist-associated bacteria (Candidatus *Armantifilum* in Cryptotermes, Candidatus *Azobacteroides* in *P. simplex*, Table S1) that dominated the communities of wood-dwellers in our study.

The limitations of host taxon sampling, for any controlled experimental approach, calls for testing the impact of additional life type switches across the termite phylogeny to further generalize our results. The limited sampling might also explain that we found no evidence for the potential horizontal acquisition of protists along the *Reticulitermes* lineage as discussed by Kitade (2004) and Radek et al. (2017).

### Bacteria suggestive of termite ecology

Knowing the identity of bacteria underpinning the difference of bacterial communities of foraging *Reticulitermes* and the wood-dwelling species in our study could provide clues as to how the persistent bacterial microbiome has responded to ecological changes. Indicator OTU 120 was classified as *Treponema I*, a member of the Spirochaetaceae family. Spirochaetes, including *Treponema I*, strongly correlate with diet in higher termites (Mikaelyan et al., 2015a; Rossmassler et al., 2015). Since Treponema I is related to diet, the presence of this OTU might reflect more subtle changes in diet along the *Reticulitermes* lineage that exist although all species investigated are primarily wood-feeders. Indicator OTU 307 was classified as *Stenoxybacter. Stenoxybacter* colonizes the hind gut wall of *R. flavipes* and contributes to acetate metabolism as well as oxygen consumption (Wertz and Breznak, 2007). Another interesting candidate is Indicator OTU 58 (*Lactococcus*). The genus *Lactococcus* has, amongst termites, so far only been reported from *Reticulitermes* and higher termites (Bauer et al., 2000; König et al., 2006; Mathew et al., 2012; Boucias et al., 2013; Yan Yang et al., 2016). Lactococci are powerful probiotics in mammals (Ballal et al., 2015), fish (Heo et al., 2013), and arthropods (Maeda et al., 2014). The acquisition of probiotics would be an obvious evolutionary response to the increased pathogen exposure that is connected to the foraging life type. Furthermore, *Lactococcus* might be metabolically important in *R. flavipes*, as it contributes to acetate production in termite guts (Bauer et al., 2000).

### Correlation of protist and bacterial diversity

We found that protist and bacterial alpha-diversity were correlated across termite species by controlling the transient microbiota. Because bacteria are much more easily acquired from the environment than the mostly anaerobic protists that inhabit the lower termite gut, environmental microbes will primarily increase or add variation to bacterial but not protist diversity. Therefore, we believe that this result can easily be obscured if the transient microbiota is not controlled. This, and the fact that few studies exist that assess both protist and bacterial diversity, might explain why this correlation has, to our knowledge, not been reported before.

This correlation could be explained by prevalent association of bacteria with protists in the termite gut. Whenever protists are lost or gained over evolutionary time, the same will be true for their associated bacteria, leading to a correlation like the one we observed. While we do find a highly significant correlation of bacterial and protist diversity, it seems that different termite species harbor different strengths of protist diversity conservation. While *P. simplex* and *R. flavipes* seem to harbor highly conserved protist diversities, the effect is less strong in *C. domesticus, C. secundus*, or *R. grassei*. This highlights the importance of conducting multispecies studies. At this point, the influence of genotypic variation within distinct termite species and how it contributes to gut microbiome diversity remain to be investigated.

## Conclusion

We assessed termite gut protist and bacterial community composition across a set of termite hosts in a laboratory based setup controlling environment and diet. This allowed us to detect a correlation between protist and bacterial alpha-diversity in the termite gut. We also found evidence for ecological determinants of the termite gut microbiome. In contrast to protist communities, bacterial communities co-aligned with host ecology, even after removing differences that are present in natural termite environments. As such, our results suggest differential protist and bacterial microbiome host specificity and argue either for increased bacterial microbiome flexibility and/or distinct factors structuring protist and bacterial termite gut communities. This result adds to the notion that bacterial communities are more variable over evolutionary time than protist communities. Further studies that address the effect of a controlled environment on the termite gut microbiota in more detail (e.g., by including termites sampled from more diverse habitats and encompassing more life type switches) will be invaluable to quantify the contribution of phylogeny and ecology to termite microbiome structure and to disentangle these contributions from transient effects.

## Materials and methods

### Termite samples

All termites were collected from typical natural habitats. *C. domesticus* and *C. secundus* were collected from mangroves at several locations close to Darwin, Australia. Colonies were collected 2–5 km apart. *P. simplex* was collected from decaying pine wood in Soroa, Cuba, and colonies of *R, grassei* and *R. flavipes* used in this study were collected in three forests of the department of Charente-Maritime in France. Two of these forests are localized on the Oléron island (i.e., St Trojan forest in the south of the island, and Les Saumonards forest in the north of the island). These forests are mostly composed of pines (*Pinus pinaster*), and all colonies were collected from logs or dead branches. The average distances separating colonies was ~1,200 m (see also Table S1). Morphological species identification was confirmed by sequencing of the mitochondrial cytochrome c oxidase subunit II (Figure S5 and Supplementary Methods) for all species. Of note, neither the two *Reticulitermes* species nor the *Cryptotermes* species are sister species (Thompson et al., 2000; Casalla et al., 2016; Dedeine et al., 2016). Aiming at a balanced study design with regards to evolutionary divergence that might be reflected in microbiome divergence we chose *C. domesticus* and *C. secundus*. Although from the same genus their divergence is comparable to that between the rhinotermitid genera *Prorhinotermes* and *Reticulitermes* in our study. Because the two *Reticulitermes* species in our study were also selected to largely span the evolutionary distance within the genus, a rather representative picture of the variation in the genus is expected. All termites used in the experiments were kept under constant conditions (27°C, 70% humidity) and on the same food substrate (*Pinus radiata* wood) from the same source for at least 6 weeks prior to the experiment. *Pinus radiata* wood was sterilized by autoclaving before offering it to the termite colonies to prevent the uptake of new microbes through the food substrate. The acclimation period was chosen following Huang et al. (2013), who showed that 6 weeks are sufficient for the microbiota to adjust to a new diet. The chosen acclimation period lies well beyond the gut passage time of 24 h in lower termites (Breznak, 1984; Li et al., 2017), ensuring excretion of excretable material like remaining food, environmental microbes that have no mechanisms to persist in the gut, and microbial DNA that was taken up before the experiment. The foraging species *R. flavipes* and *R. grassei* were additionally provided with sand to build their nests. In order to exclude a contribution of microbes or microbial DNA in the sand to the *Reticulitermes*-associated microbiota, we applied the same DNA extraction, amplification, and sequencing procedure as for the termite guts to the sand. However, we failed to amplify the targeted fragments from the sand samples. This led us to conclude that the sand did not contribute to the bacterial nor protist community profiles.

### DNA extraction, primer design and library preparation

DNA was extracted from a pool of three worker guts per colony using bead beating, chloroform extraction and isopropanol precipitation (also see Supplementary Material and Methods). This way, each colony was treated as an independent sample. We amplified the v4 region of the 16S rRNA gene with the bacteria specific primerset 515f (5′- GTGCCAGCMGCCGCGGTAA−3′) and 806r (5′- GGACTACHVGGGTWTCTAAT−3′) developed by Caporaso et al. (2012). Specific barcode combinations were added to the primer sequences following Kozich et al. (2013).

In order to amplify a region of the 18S rRNA gene of parabasalid protists, we developed custom designed primers. Protists from the second large taxonomic group of termite symbionts, the order Oxymonadida, were excluded from the analysis because they are difficult to target (Heiss and Keeling, 2006) and escaped reliable Illumina sequencing compatible amplification (data not shown). Primer development was based on parabasalid sequences from SILVA SSU Ref Nr 123 that was extended by 18S rRNA gene sequences from protist taxa that we expected to occur in the termite species we analyzed according to Yamin (1979). These additional sequences were downloaded from ncbi and aligned using ClustalW (Larkin et al., 2007) in the BioEdit Sequence Alignment Editor version 7.2.5 (Hall, 1999). The resulting alignment was manually curated and searched for potential forward and reverse primers that would match as many protist sequences as possible but not termite 18S rRNA genes. The 18S rRNA gene alignment as well as the corresponding taxonomy reference—file are provided in the supplement (Files S4, S5). The resulting primerset Par18SF: 5′-AAACTGCGAATAGCTC-3′; Par18SR: 5′-CTCTCAGGCGCCTTCTCCGGCAGT-3′, amplified ~250 bp. A detailed PCR protocol can be found in the Supplementary material and methods section. Libraries were sequenced on an Illumina MiSeq reading 2 × 250 bp.

### Analysis

Data analysis was performed using MOTHUR version 1.33.3 (Schloss et al., 2009), following the MiSeq SOP (available at https://www.mothur.org/wiki/MiSeq_SOP, see also Kozich et al., 2013). Main data processing steps were quality filtering of raw sequence reads, denoising, removal of chimeric sequences, taxonomic classification, and OTU clustering at 97% sequence similarity. Our detailed MOTHUR script can be found in File S6. For taxonomic classification of the bacterial 16S OTUs a representative sequence of each OTU was aligned to DictDb_v3 (Mikaelyan et al., 2015b). The DictDB database is the SILVA (Quast et al., 2013) database extended by the most comprehensive collection of termite associated bacteria and allowed us to span a large set of termites and other Dictyoptera with our analysis. 18S protist OTUs were aligned and classified using all parabasalid sequences from the SILVA SSU Ref Nr 123 database that was extended by our custom made alignment and taxonomy reference (Files S7, S8). In order to avoid coverage bias, bacterial and protist datasets were rarefied resulting in 32,824 and 22,285 sequences per sample, respectively. Statistical tests and data visualization were performed in R version 3.1.2 (R Core Team, 2014). Phylogenetic trees necessary to apply UniFrac as well as the trees including OTU representative sequences were generated with FastTree version 2.1.7 (Price et al., 2010). The resulting trees were visualized with Dendroscope 3.3.4 (Huson and Scornavacca, 2012). Assuming that microbes that are ecologically relevant for the host should not be rare, we used the weighted Unifrac metric (Lozupone and Knight, 2005) for our analysis. A community distance measure that does not take abundance into account might put too much emphasis on rare microbes. By taking into account abundance, the use of weighted Unifrac also allowed us to avoid setting arbitrary thresholds (e.g., a minimum number of sequences) to call microbes present or absent. It is worth noting that Unifrac is not OTU based, but takes phylogenetic relatedness between microbes into account even if they would end up in termite species specific OTUs. Dendrograms based on weighted Unifrac distances were generated using the UPGMA algorithm as implemented in the R PvClust package version 2.0.0 (Suzuki and Shimodaira, 2006) and bootstrapped 10 000 times with standard settings. Indicator species were obtained following the method by Dufrêne and Legendre (1997), as included in MOTHUR and in the R Indicspecies package version 1.7.6 (De Cáceres et al., 2010). LCBD analysis was performed using the “LCBD.comp” command from the adespatial R package 0.0–9 (Dray et al., 2017) on weighted Unifrac distances.

## Data availability

Sequence data is available at MGRAST (http://metagenomics.anl.gov/) under the IDs 4711469.3–4711607.3. Supplementary information is available at the journal website.

## Ethics statement

The study was performed on unendangered arthropods.

## Author contributions

Conceived experiments: FS, JK, and LW. Performed experiments: LW and SK. Wrote the manuscript: FS, LW, JK, CRV, and FD. Provided samples: JK and FD.

### Conflict of interest statement

The authors declare that the research was conducted in the absence of any commercial or financial relationships that could be construed as a potential conflict of interest.
