# Supplementary material for: Differential Ecological Specificity of Protist and Bacterial Microbiomes across a Set of Termite Species
Source: Front Microbiol. 2017 Dec 19;8:2518. doi: 10.3389/fmicb.2017.02518 (PMC5742190; doi:10.3389/fmicb.2017.02518)
Supplement: Supplementary file 1 [file DataSheet1.pdf]

Figures and Tables Supplement

Table S1: Number of 16S and 18S sequences per sample after quality-filtering and before subsampling to 32824 and 22285 per sample respectively.

Sample	Termite Host	Origin	Collection Year	n (16S)	n (18S)
**Cd2**	*C. domesticus*	Darwin, Australia	2007	48739	140854
**Cd3**	*C. domesticus*	Darwin, Australia	2003	49782	118761
**Cd4**	*C. domesticus*	Darwin, Australia	2007	53126	123848
**Cd5**	*C. domesticus*	Darwin, Australia	2011	64954	64636
**Cd6**	*C. domesticus*	Darwin, Australia	2003-2011	53555	88935
**Cs1**	*C. secundus*	Darwin, Australia	2007	52153	100882
**Cs2**	*C. secundus*	Darwin, Australia	2011	68275	98556
**Cs3**	*C. secundus*	Darwin, Australia	2011	52626	127522
**Cs4**	*C. secundus*	Darwin, Australia	2011	68072	107700
**Cs5**	*C. secundus*	Darwin, Australia	2011	80497	114502
**Cs6**	*C. secundus*	Darwin, Australia	2011	66969	150807
**Cs7**	*C. secundus*	Darwin, Australia	2011	49659	88094
**Cs8**	*C. secundus*	Darwin, Australia	2011	41960	77778
**Ps2**	*P. simplex*	Soroa, Cuba	1964	80182	92012
**Ps3**	*P. simplex*	Soroa, Cuba	1964	60336	111154
**Ps4**	*P. simplex*	Soroa, Cuba	1964	33814	78020
**Ps5**	*P. simplex*	Soroa, Cuba	1964	54064	107191
**Ps6**	*P. simplex*	Soroa, Cuba	1964	75636	136586
**Ps8**	*P. simplex*	Soroa, Cuba	1964	71577	82049
**Ps9**	*P. simplex*	Soroa, Cuba	1964	66598	115255
**Rf1**	*R. flavipes*	Île d’ Oléron, France	2014	61106	122318
**Rf2**	*R. flavipes*	Île d’ Oléron, France	2014	44860	67995
**Rf3**	*R. flavipes*	Île d’ Oléron, France	2014	70343	86250
**Rf4**	*R. flavipes*	Île d’ Oléron, France	2014	72438	108646
**Rf5**	*R. flavipes*	Île d’ Oléron, France	2014	48689	131560
**Rg1**	*R. grassei*	Île d’ Oléron, France	2014	51076	39456
**Rg2**	*R. grassei*	Île d’ Oléron, France	2014	32824	68683
**Rg4**	*R. grassei*	Île d’ Oléron, France	2014	52844	22285
**Rg5**	*R. grassei*	Île d’ Oléron, France	2014	37390	64764

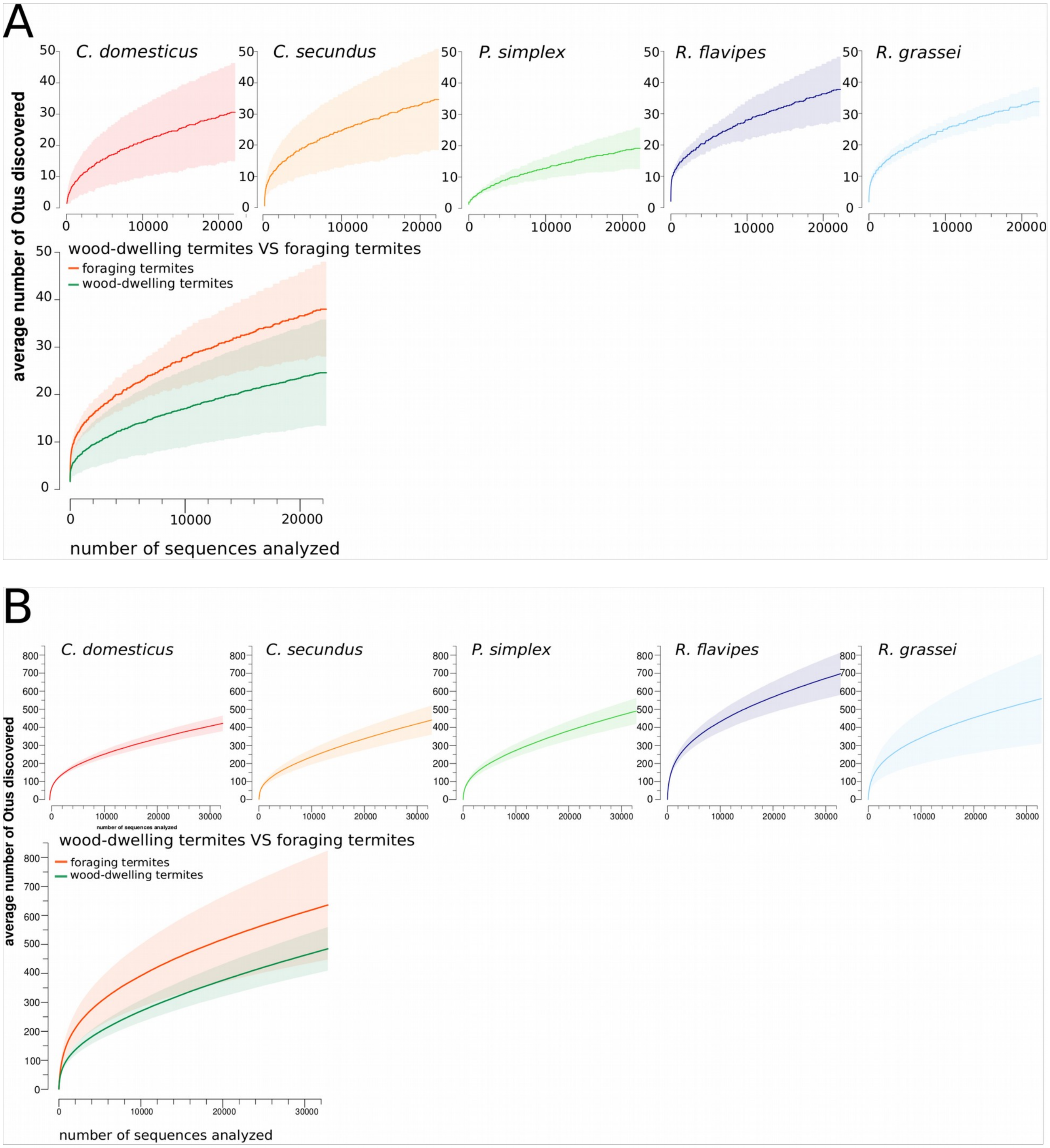

Figure S1: Average Rarefaction Curves of 97% identity OTUs. (A) Rarefaction curves of protist communities (B) Rarefaction curves of bacterial communities. The shaded area shows the standard deviation. Upper part shows individual termite species, lower part shows comparison of wood-dwellers (green) and foragers (orange). Protist – as well as bacterial communities were more diverse in foragers (P = 0.03 (protist communities); P = 0.005 (bacterial communities); Wilcoxon Rank Sum Test on Shannon – diversity indices).

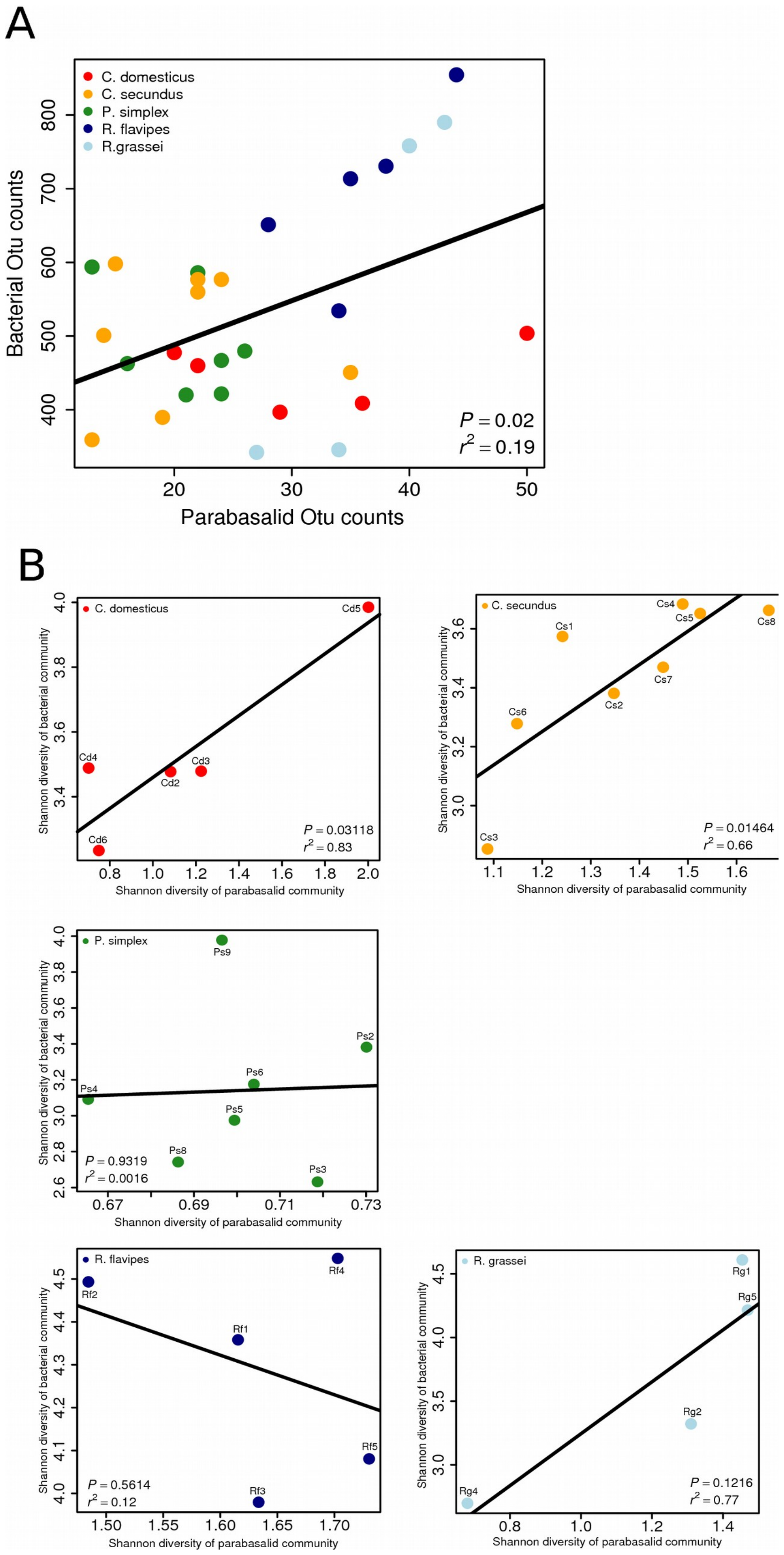
A
C. domesticus
C. secundus
P. simplex
R. flavipes
R.grassei
Bacterial Otu counts
Parabasalid Otu counts
P = 0.02
r2 = 0.19
B
C. domesticus
P = 0.03118
r2 = 0.83
C. secundus
P = 0.01464
r2 = 0.66
P. simplex
P = 0.9319
r2 = 0.0016
R. flavipes
P = 0.5614
r2 = 0.12
R. grassei
P = 0.1216
r2 = 0.77
Shannon diversity of bacterial community
Shannon diversity of parabasalid community

Figure S2: Correlation of bacterial and protist alpha diversity. (A) OTU counts are correlated across termite species. Each dot represents a termite colony. The regression line is based on all samples. P-value and r-squared were calculated using Pearson's product – moment correlation. (B) Scatterplots of parabasalid against bacterial Shannon diversities (Shannon's H) for termite host species. Each dot represents a termite colony.

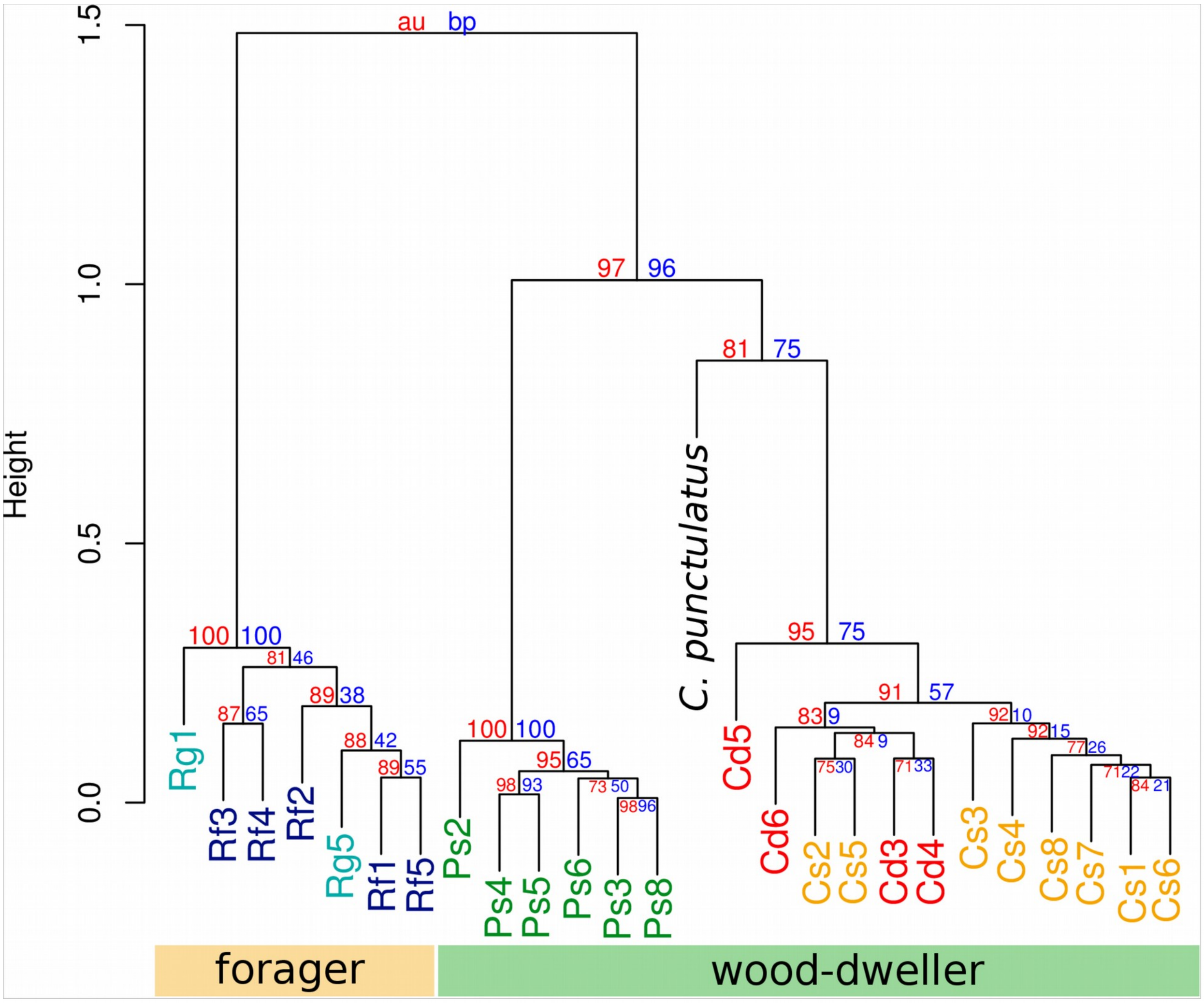

Figure S3: Cluster dendrogram based on weighted UniFrac bacterial community distances including the woodroach Cryptocercus punctulatus as an outgroup. Cd = C. domesticus, Cs = C. secundus, Ps = P. simplex, Rf = R. flavipes, Rg = R. grassei. Outgroup C. punctulatus in black. Blue numbers = bootstrap probability, red numbers=approximate unbiased probability.

For this analysis, we extracted a set of 3537 16S rRNA gene sequences from Cryptocercus punctulatus from Dietrich et al. (Dietrich et al., 2014) and analyzed them together with our data. More than 200 base pairs that overlap between the region we sequenced and the region Dietrich et al. (Dietrich et al., 2014) sequenced were analyzed. We had to rarefy the number of sequences in our samples by a factor of ~10 (32,000 sequences as opposed to 3537) to make our dataset comparable to the data from Dietrich et al. (Dietrich et al., 2014). In order to counter potential sampling effects that result from the rarefication, the 3537 sequences were resampled 1000 times from all samples using the respective commands in mothur. Shown is the average dendrogram over these re-samplings. Individual samples that sit on long branches in Figure 4 (Ps9, Rg2, Rg4, Cd2) were removed from the analysis because the long branches tended to attract clustering with the Cryptocercus community that is also more distant from the other samples (data not shown). This is an error known as long-branch attraction that is inherent to the clustering process (Felsenstein, 1978).

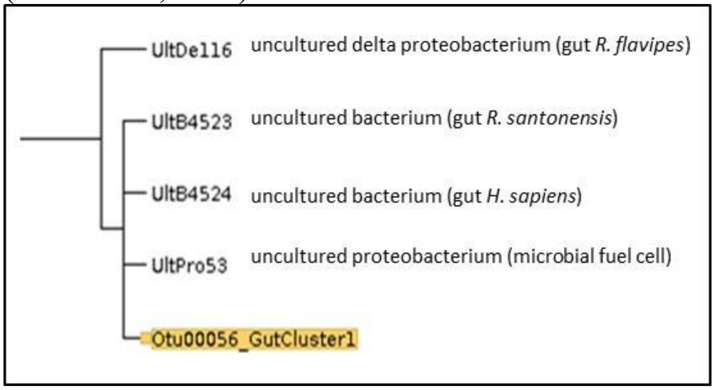

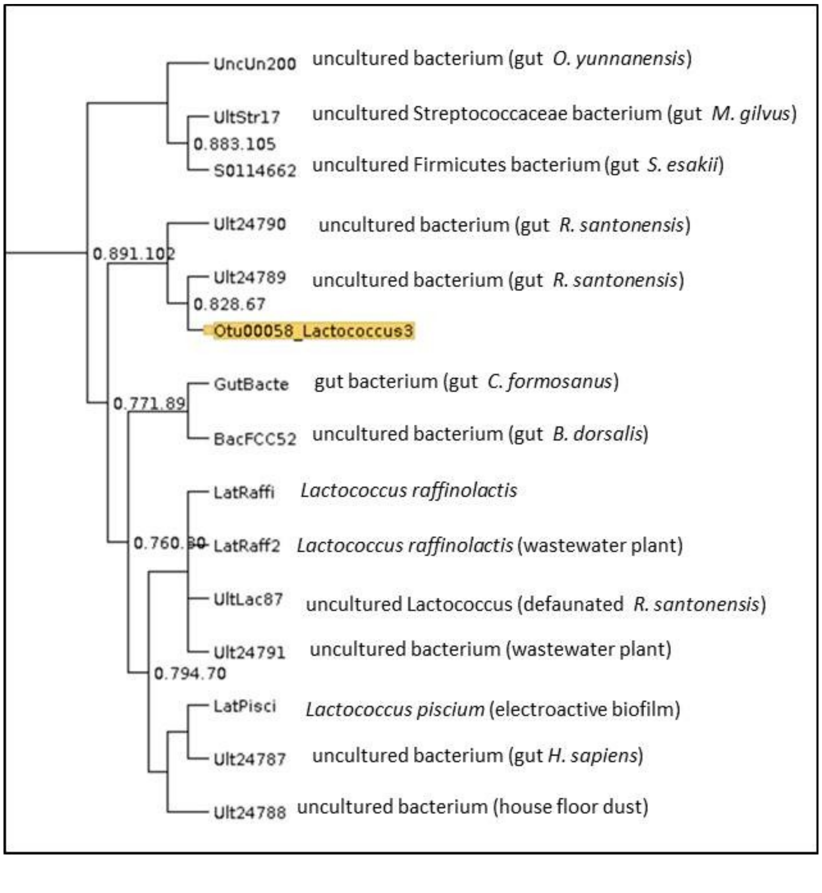

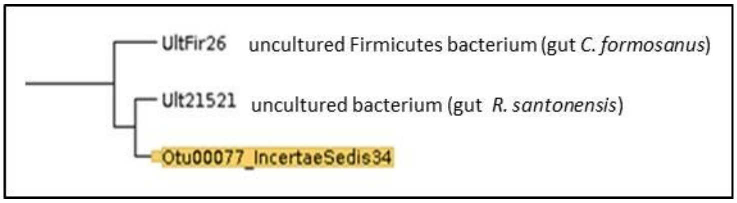

UltFir26 uncultured Firmicutes bacterium (gut C. formosanus)
Ult21521 uncultured bacterium (gut R. santonensis)
Otu00077_IncertaeSedis34

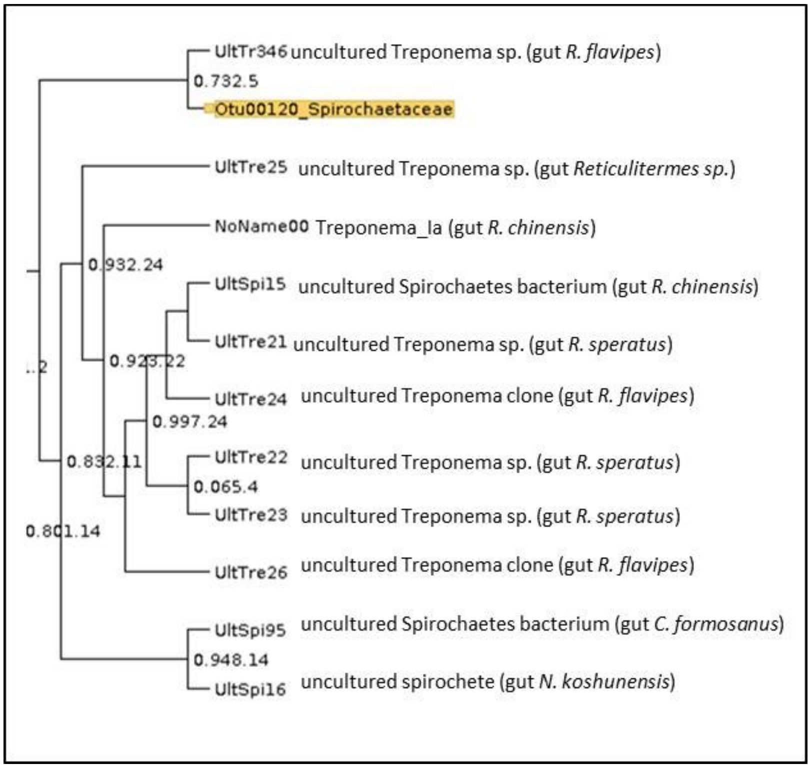

UltTr346 uncultured Treponema sp. (gut R. flavipes)
0.732.5
Otu00120_Spirochaetaceae
UltTre25 uncultured Treponema sp. (gut Reticulitermes sp.)
NoName00 Treponema_Ia (gut R. chinensis)
0.932.24
UltSpi15 uncultured Spirochaetes bacterium (gut R. chinensis)
UltTre21 uncultured Treponema sp. (gut R. speratus)
0.923.22
UltTre24 uncultured Treponema clone (gut R. flavipes)
0.997.24
UltTre22 uncultured Treponema sp. (gut R. speratus)
0.832.11
0.065.4
UltTre23 uncultured Treponema sp. (gut R. speratus)
0.801.14
UltTre26 uncultured Treponema clone (gut R. flavipes)
UltSpi95 uncultured Spirochaetes bacterium (gut C. formosanus)
0.948.14
UltSpi16 uncultured spirochete (gut N. koshunensis)

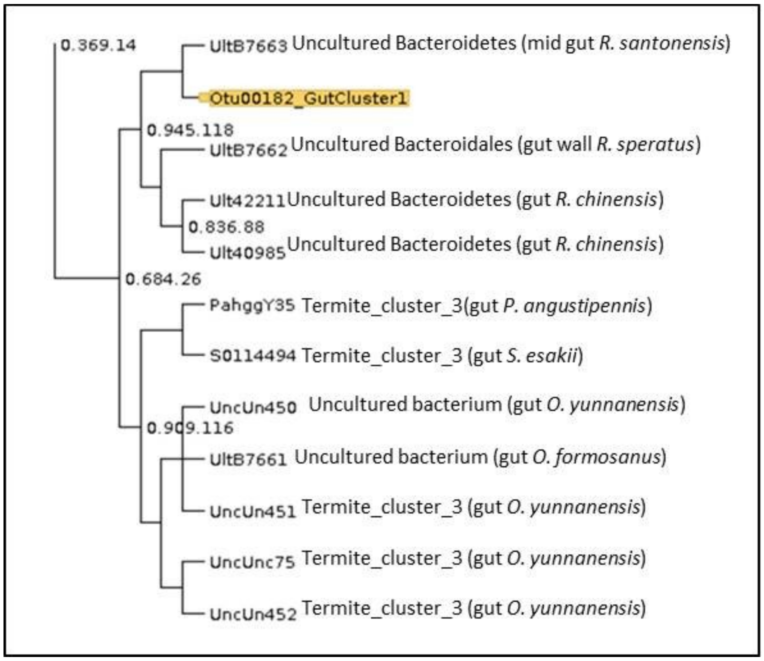

0.369.14
UltB7663 Uncultured Bacteroidetes (mid gut R. santonensis)
Otu00182_GutCluster1
0.945.118
UltB7662 Uncultured Bacteroidales (gut wall R. speratus)
Ult42211 Uncultured Bacteroidetes (gut R. chinensis)
0.836.88
Ult40985 Uncultured Bacteroidetes (gut R. chinensis)
0.684.26
PahggY35 Termite_cluster_3(gut P. angustipennis)
S0114494 Termite_cluster_3 (gut S. esakii)
UncUn450 Uncultured bacterium (gut O. yunnanensis)
0.909.116
UltB7661 Uncultured bacterium (gut O. formosanus)
UncUn451 Termite_cluster_3 (gut O. yunnanensis)
UncUnc75 Termite_cluster_3 (gut O. yunnanensis)
UncUn452 Termite_cluster_3 (gut O. yunnanensis)

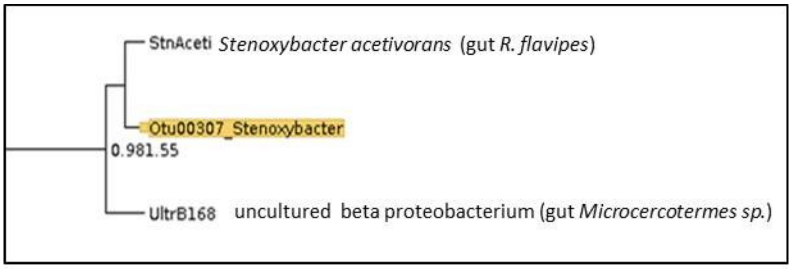

Figure S4: Phylogenetic tree of candidate OTUs from Table 2. Representative sequences of candidate OTUs affiliated with foraging Reticulitermes were aligned with DictDB and all OTUs with more than 100 sequences in Prorhinotermes or Cryptotermes. Shown are excerpts of the full tree. All indicator OTUs from Table 2 are represented as well as all indicator OTUs that contained more than 3000 sequences and had an indicator value >99.9 . The full newick-formatted tree can be found in Supplementary file S7.

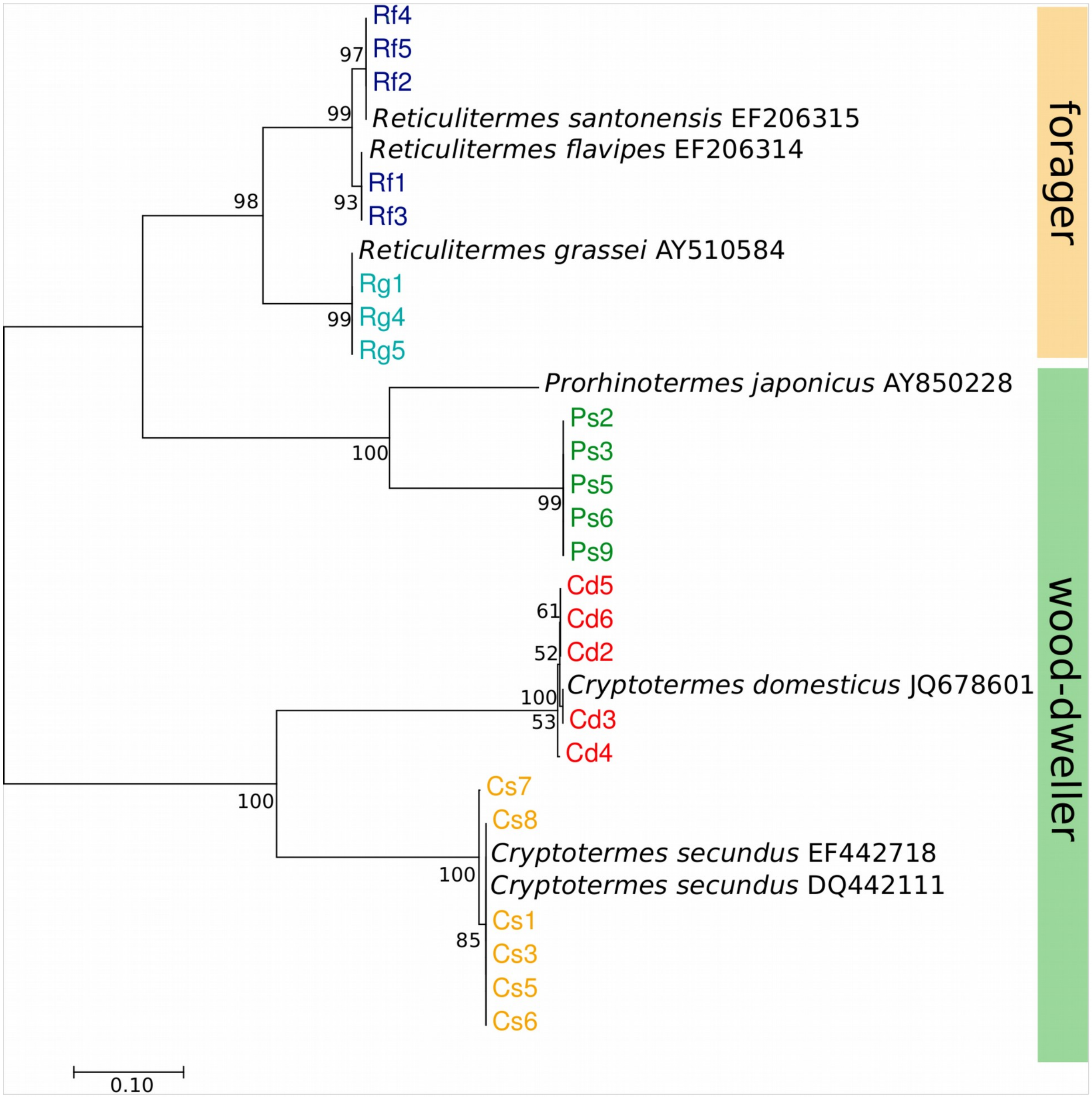

Figure S5: Phylogenetic tree of termite colonies in this study based on sequence data from cytochrome c oxidase subunit II (COII). The highest identity blast hits for each species were used as reference sequences in the sequence alignment (references in black, accession numbers included). If more than one reference per termite species is given, they had the same blast coverage and Identity-value. Cd = *C. domesticus*, Cs = *C. secundus*, Ps = *P. simplex*, Rf = *R. flavipes*, Rg = *R. grassei*; orange = foraging termite species; green = wood dwelling species; black = reference sequences based on blast search. Sequences were generated from the same samples that were used for microbial community profiling (see supplementary material and methods). Unfortunately, the PCR failed for extracts from colonies Cs2, Cs4, Ps4, Ps8, and Rg2. COII sequences were edited by hand using BioEdit (version 7.2.5 (Hall, 1999)). The edited sequences were subject to blast search against the nr database (NCBI nucleotide blast). Hits with the highest sequence identity were downloaded and used as reference sequences in the alignment. The alignment was generated using ClustalW with standard settings as implemented in BioEdit and manually curated resulting in a total alignment length of ~660 bp. The phylogenetic tree was

built in MEGA (version 7.0.18 (Kumar et al., 2016)) using the Neighbor-Joining tool with standard settings and 3000 bootstraps. Branch lengths were computed using the Maximum Composite Likelihood method [3] and are in units of base substitutions per site.

Supplementary materials and methods

DNA extraction

Entire guts of three workers per colony were extracted and immediately transferred to 100 µl of CTAB solution (0.75 M NaCl, 0.05 M Tris, 0.01 M EDTA, 2% CTAB). Guts were homogenized (zirconia and glass beats, 3 min at 25*1/s), using a Tissue Lyser II (Qiagen). Additional 400 µl of CTAB solution were added and samples were incubated for 1h at 65°C and 800 rpm on a Thermomixer Comfort (Eppendorf). After addition of 2 µl Proteinase K (Thermoscientific, concentration: ~20 mg/mL) samples were incubated for 2h at 55°C and 800 rpm. The proteinase K reaction was terminated by heating to 98°C for 15 min. DNA was extracted with 500 µl of chloroform:isoamylic alcohol (24:1) followed by centrifugation for 15 min at 10000 rpm. DNA, was precipitated with 325 µl of ice cold isopropanol and overnight incubation at -20°C. Samples were washed with 300 µl of 100% ethanol and twice with 300 µl 70% ethanol, centrifuging for 15 min at 4°C at 14.000 rpm between each washing step. The DNA pellet was air dried and resuspended in 50µl of water.

16S and 18S PCR protocol

PCR was run in 10 µl triplicates (6.7 µl PCR water, 2 µl HF buffer, 0.2 µl dNTPs, 0.1 µl enzyme (Phusion Hot Start II High Fidelity DNA Polymerase Kit, Thermoscientific), 0.25 µl forward/reverse primer, 0.5 µl DNA template). PCR conditions were 98°C for 30 s, 30X (98°C for 9s, 50°C(16S primer annealing)/52.8°C(18S primer annealing) for 60 s, 72°C for 90 s), 72°C for 10 min. PCR products were quantified on an agarose gel and pooled at equal amounts before purification using the MinElute Gel Extraction Kit (Qiagen).

PCR with COII markers and PEG mix purification

PCR with COII primerset (COII_A-tLeu_for: 5'-CAGATAAGTGCATTGGATTT-3'; COII_B-tLys_rev: 5'-GTTTAAGAGACCAGTACTTG-3') was run in 20 µl reactions (8.5 µl water, 4 µl enhancer (MP Biomedicals), 2 µl buffer, 1.6 µl MgCl2, 0.4 µl 10µM dNTPs, 0.5 µl enzyme (TaqDNA Polymerase, 10x250U TaqDNA Pol 5U/µl Kit, Cat# EPTQA925,MP Biomedicals), 1 µl forward/reverse primer (10µM), 1 µl DNA template. PCR conditions were 94°C for 2 min, 35X (94°C for 60 s, 50°C for 60 s, 72°C for 75 s), 72°C for 7 min. PCR products were purified using a Polyethylene glycol (PEG) mix (26.2 g PEG 800, 20 ml NaAc (pH 5.2, 3M), 660 µl MgCl2 (1M), added to 100 ml aqua bidest). For purification, the PCR product was transferred into a new tube (20µl) and the same volume of PEG mix was added. Samples were vortexed and incubated at room temperature for 10 min. After centrifuging for 10 min at 14.000 rpm and room temperature, the supernatant was discarded. The pellet was washed in 200µl 100% EtOH, followed by centrifugation for 10 min at 14.000 rpm and room temperature. The supernatant was discarded and the pellet was vacuum dried for approximately 10 min. After resuspending the pellet in 11µl Millipore water, purified DNA concentration was quantified on a Nanodrop and adjusted to 50ng/µl. PCR products were sequenced at Macrogen, NL.

Supplementary Literature

Dietrich, C., Köhler, T., Brune, A., Kohler, T., and Brune, A. (2014). The cockroach origin of the termite gut microbiota: Patterns in bacterial community structure reflect major evolutionary events. *Appl. Environ. Microbiol.* 80, 2261–2269. doi:10.1128/AEM.04206-13.

Felsenstein, J. (1978). Cases in which Parsimony or Compatibility Methods Will be Positively Misleading. *Syst. Zool.* 27, 401. doi:10.2307/2412923.

Hall, T. A. (1999). BioEdit: a user-friendly biological sequence alignment editor and analysis program for Windows 95/98/NT. *Nucleic Acids Symp Ser*, 95–98.

Kumar, S., Stecher, G., and Tamura, K. (2016). MEGA7: Molecular Evolutionary Genetics Analysis Version 7.0 for Bigger Datasets. *Mol. Biol. Evol.* 33, 1870–1874. doi:10.1093/molbev/msw054.
