# Supplementary material for: Differential Ecological Specificity of Protist and Bacterial Microbiomes across a Set of Termite Species
Source: Front Microbiol. 2017 Dec 19;8:2518. doi: 10.3389/fmicb.2017.02518 (PMC5742190; doi:10.3389/fmicb.2017.02518)
Supplement: Supplementary file 2 [file DataSheet2.PDF]

((((((Ult17884:0.02301,(((Ult19288:0.0439,Ult18593:0.01921)0.737.1:0.003
3,(Ult17665:0.00743,((((Ult18024:5.5E-4,(Ult18575:0.00369,(Ult18159:5.5E-
4,(Ult18574:0.00744,(Ult18572:0.0,Ult18573:0.0):0.00369)0.718.1:5.5E-
4)0.901.1:5.5E-4)0.850.1:0.00369)0.873.1:5.4E-4,(UltB7204:5.4E-
4,Ult17865:0.04734)0.833.1:0.00365)0.994.1:0.0228,(Ult17700:5.4E-
4,((((Ult17532:0.0,Ult17534:0.0):5.5E-
4,Ult17533:0.00369)0.963.1:0.01129,(Ult17535:5.3E-4,((Ult17556:5.5E-
4,(((Ult17552:0.05912,(((Ult17558:0.01113,Ult17927:0.02303)0.884.1:5.4E-
4,Ult17561:0.0037)0.915.1:5.4E-
4,Ult17555:0.0351)0.824.1:0.00361)0.974.1:5.4E-
4,((Ult21411:0.00369,((Ult18158:5.5E-
4,Ult18563:0.07523)0.827.1:0.0038,(Ult17526:0.0,Ult17548:0.0,Ult17547:0.0
,Ult17549:0.0,Ult17554:0.0,Ult17557:0.0,Ult17559:0.0,Ult17562:0.0,Ult1775
9:0.0,Ult18107:0.0,Ult21386:0.0):5.5E-4)0.000.1:5.5E-4)0.000.2:5.5E-
4,Ult18155:5.5E-4)0.546.1:5.5E-4)0.156.1:5.5E-
4,Ult17553:0.00369)0.859.1:0.00381)0.782.1:5.3E-4,(Ult17551:5.4E-
4,Ult17536:0.01892)0.396.1:0.00368)0.857.1:0.00378)0.155.1:5.5E-
4)0.801.1:0.00381,Ult17697:0.01121)0.780.1:0.0038)0.724.1:5.4E-
4)0.835.1:0.00376,((Ult17520:0.0,Ult17521:0.0,Ult17689:0.0,Ult17685:0.0,U
lt17686:0.0,Ult17687:0.0,Ult17691:0.0,Ult17692:0.0,Ult17695:0.0,Ult17703:
0.0,Ult17704:0.0,Ult17935:0.0,Ult18157:0.0,Ult21384:0.0):5.5E-
4,((((Ult24431:0.02645,(Ult19453:5.4E-
4,Ult22675:0.01888)0.433.1:0.01004)0.778.1:0.00538,(Ult17688:0.01119,(Ult
17696:0.00714,Ult17702:0.04819)0.923.1:5.5E-4)0.358.1:5.5E-
4)0.000.3:5.5E-4,Ult17699:0.0074)0.455.1:5.5E-
4,Ult17698:0.00369)0.458.1:5.4E-4)0.000.4:5.0E-4)0.151.1:5.5E-
4)0.520.1:5.4E-4)0.869.1:0.00382,(Ult17701:5.5E-
4,(Ult17693:0.08393,Ult17694:0.01031)0.761.1:0.00468)0.000.5:5.4E-
4)0.884.2:5.4E-4)0.837.1:0.00785,((((Ult19469:0.0,Ult21385:0.0):5.5E-
4,(Ult17574:0.00369,Ult17570:0.00369)0.669.1:5.5E-4)0.989.1:5.5E-
4,((Ult17565:0.01473,((Ult17571:0.00368,((Ult17566:0.0,Ult17568:0.0,Ult17
569:0.0,Ult17575:0.0,Ult17577:0.0):5.5E-
4,(Ult17573:0.00742,Ult19319:0.0074)0.665.1:5.5E-4)0.738.1:5.5E-
4)0.819.1:5.5E-4,(Ult17567:0.01109,Ult17578:0.00742)0.459.1:5.5E-
4)0.947.1:5.5E-4)1.000.1:5.5E-
4,(Ult17656:0.0228,Ult17854:0.01156)0.628.1:0.00752)0.928.1:0.00335)0.801
.2:5.4E-4,(((Ult17593:0.00369,(Ult17589:5.5E-
4,(Ult17585:0.02264,((((Ult17580:0.0,Ult17582:0.0,Ult17583:0.0,Ult17584:0
.0,Ult17587:0.0,Ult17586:0.0,Ult17588:0.0,Ult17595:0.0,Ult17596:0.0,Ult17
811:0.0):5.5E-4,((Ult17604:0.00369,Ult17590:0.00369)0.000.6:5.5E-
4,((Ult17603:0.00369,(((Ult17531:0.01131,(((UltB7193:0.0,Ult17600:0.0,Ult
20892:0.0):5.5E-
4,Ult17599:0.00742)0.831.1:0.00369,(Ult17550:0.00779,Ult17602:5.5E-
4)0.035.1:5.5E-4)0.861.1:0.00387)0.468.1:5.5E-
4,UltB7202:0.00368)0.570.1:5.5E-
4,(Ult17530:0.0,Ult17597:0.0,Ult17601:0.0,Ult17605:0.0,Ult17606:0.0):5.5E
-4)0.374.1:5.4E-4)0.430.1:5.5E-
4,Ult17598:0.01115)0.867.1:0.00387)0.264.1:5.5E-4)0.000.7:5.5E-
4,(Ult17591:0.00369,(Ult17581:0.00741,Ult19452:0.00369)0.865.1:5.5E-
4)0.000.8:5.5E-4)0.601.1:5.5E-
4,(Ult17592:0.03023,Ult17594:0.02724)0.763.1:0.00462)0.303.1:5.5E-
4)0.844.1:0.00381)0.275.1:5.4E-
4)0.963.2:0.01535,(Ult17837:0.01398,((Ult17715:0.0,Ult17716:0.0):5.4E-
4,(((Ult17712:5.4E-

4,Ult17717:0.04447)0.943.1:0.01984,Ult17711:0.01203)0.737.2:0.0032,Ult177
10:0.01549)0.889.1:0.01189)0.955.1:0.01881)0.690.1:0.00972)0.910.1:5.3E-
4,(UltClost:0.0037,Ult17576:5.5E-
4)0.852.1:0.00378)0.824.2:0.00771)0.885.1:0.00774)0.765.1:0.00356,(Ult191
18:0.0317,((((Ult17655:0.0,Ult17669:0.0,Ult21383:0.0):5.5E-
4,Ult17664:5.5E-4)0.487.1:5.5E-
4,((Ult17648:0.00212,Ult17663:0.01067)0.934.1:0.00184,(UltB7192:0.00369,(
Ult17660:0.00369,((Ult17652:0.0,Ult17654:0.0,Ult17642:0.0,Ult17643:0.0,Ul
t17649:0.0,Ult17651:0.0,Ult17668:0.0):5.5E-
4,(Ult17519:0.00369,((Ult17653:0.01864,(Ult17647:0.07634,(Ult17641:0.0512
,Ult17650:0.0568)0.560.1:0.00833)0.837.2:0.00602)0.966.1:5.5E-
4,(Ult17644:0.00743,(Ult17563:0.01116,(((UltB7180:0.0,Ult17657:0.0,Ult176
59:0.0,Ult17661:0.0,Ult17666:0.0,Ult17667:0.0,Ult17877:0.0):5.5E-
4,((Ult17646:0.00368,Ult17645:0.04811)0.452.1:5.5E-
4,Ult17662:0.00741)0.439.1:5.5E-4)0.000.9:5.5E-4,Ult18156:5.5E-
4)0.419.1:5.5E-4)0.171.1:5.5E-4)0.844.2:0.00372)0.339.1:5.3E-
4)0.408.1:5.5E-4)0.000.10:5.5E-4)0.214.1:5.5E-4)0.686.1:5.3E-
4)0.864.1:0.00369)0.823.1:0.00369,(Ult17564:5.5E-
4,Ult18108:0.00742)0.000.11:5.5E-
4)0.881.1:0.01069)0.778.2:0.00481)0.782.2:0.0039,Ult17629:0.00747)0.890.1
:0.00774,(UltClo36:0.01138,(((Ult19631:5.5E-
4,(Ult19629:0.04967,Ult19630:0.00743)0.746.1:0.00342)0.951.1:0.00713,(Ult
21476:0.02689,(Ult19579:0.01145,((((Ult19558:0.0,Ult19559:0.0):0.00402,((
((Ult17672:0.0,Ult18040:0.0,Ult19547:0.0,Ult19551:0.0,Ult19552:0.0,Ult195
54:0.0,Ult19555:0.0):5.5E-
4,(Ult19556:0.01502,Ult19546:0.00369)0.620.1:5.5E-4)0.991.1:5.5E-
4,(Ult19548:0.14718,(Ult19549:0.09917,Ult19553:0.00956)0.878.1:0.01233)0.
879.1:0.01109)0.835.2:0.00352,(((Ult19561:0.00369,Ult19562:0.0037)0.692.1
:5.0E-4,Ult19563:5.5E-4)0.907.1:5.5E-4,((Ult17861:5.5E-
4,(Ult19560:0.0037,Ult19564:0.00369)0.747.1:5.5E-4)0.809.1:5.5E-
4,(Ult19569:0.01303,Ult19632:0.03056)0.787.1:0.00688)0.925.1:0.00742)1.00
0.2:5.4E-
4)0.221.1:0.00735)0.771.1:0.00973,(Ult19565:0.00956,(Ult19557:0.02037,Ult
19550:0.0479)0.730.1:0.00496)0.901.2:0.01355)0.302.1:0.00399,(Ult19566:0.
01117,Ult19567:5.5E-
4)0.801.3:0.00535)0.886.1:0.01011)0.584.1:0.0037)0.910.2:5.3E-
4)0.925.2:0.00726,(Ult18354:0.01507,((Ult18351:0.0,Ult18353:0.0):5.5E-
4,Ult18352:0.01116)0.972.1:0.01513)0.050.1:5.4E-
4)0.908.1:0.00722)0.186.1:5.5E-
4)0.181.1:0.00364,Ult17572:0.01926)0.808.1:0.00198,(((Ult18162:0.02354,(U
lt21406:0.0324,Ult28166:0.07284)0.671.1:0.02782)0.950.1:0.03228,((((UltCl
o31:0.01309,(Ult17820:0.01518,(Ult17750:0.05074,(Ult17751:5.5E-
4,(Ult17752:0.0,Ult21264:0.0):5.5E-4)0.589.1:5.5E-
4)0.833.2:0.00865)0.924.1:0.01662)0.796.1:0.0077,((((((((Ult18017:0.0110
7,((Ult18018:0.0,Ult18019:0.0,Ult18020:0.0,Ult18022:0.0,Ult20093:0.0,Ult2
1342:0.0):5.5E-4,(Ult18023:0.00369,Ult18021:0.00369)0.662.1:5.5E-
4)0.941.1:5.5E-4)0.689.1:5.4E-
4,Ult17503:0.02296)0.810.1:0.00368,((Ult18012:0.01117,((((((Ult17882:0.0,
Ult17904:0.0):5.5E-
4,((Ult17923:0.00367,((Ult17898:0.0,Ult17903:0.0):5.5E-
4,(((((Ult17736:0.00546,Ult17727:0.01808)0.797.1:0.01014,(Ult17744:0.0036
9,((Ult17738:0.0,Ult17742:0.0):5.5E-
4,(Ult17730:0.02242,((((Ult17741:0.00369,(Ult17731:5.5E-
4,(Ult17745:0.0,Ult17843:0.0):5.5E-4)0.700.1:5.5E-

4)0.242.1:0.00194,Ult17728:0.03468)0.196.1:0.00181,Ult17729:0.10125)0.331
.1:5.5E-4,Ult21256:0.00742)0.865.2:0.00421)0.703.1:5.4E-4)0.168.1:5.5E-
4)1.000.3:5.4E-
4)0.880.1:0.00365,((Ult17732:0.01857,Ult17739:0.03023)0.955.2:5.4E-
4,(Ult17743:5.5E-4,Ult17746:0.0074)0.129.1:5.5E-4)1.000.4:5.4E-
4)0.591.1:0.0112,Ult18601:0.0179)0.875.1:0.01157,Ult17896:5.5E-
4)0.952.1:0.01263)0.586.1:5.5E-
4)0.848.1:0.00402,(Ult17883:0.00133,Ult17905:0.05362)0.985.1:0.00256)0.79
8.1:5.3E-4)0.139.1:5.5E-4,Ult17901:0.02656)0.492.1:5.4E-
4,Ult17900:0.03431)0.934.2:5.4E-
4,(Ult17996:0.07475,(Ult17902:0.03977,Ult17926:0.17975)0.924.2:0.0327)0.4
63.1:0.01673)0.926.1:0.00386,(Ult17899:0.00385,(Ult18602:0.01507,(Ult1859
5:0.00369,((Ult18599:0.0,Ult18598:0.0,Ult18600:0.0):5.5E-
4,(Ult18597:0.01492,Ult18596:0.01121)0.756.1:5.5E-4)0.667.1:5.5E-
4)0.893.1:5.2E-
4)0.827.2:0.01136)0.776.1:0.00359)0.859.2:0.00805)0.924.3:5.5E-
4,(((Ult18013:0.01912,(Ult17740:0.01519,((Ult17757:0.00744,Ult17758:0.003
72)0.784.1:0.00371,(Ult17755:0.00371,Ult17756:5.5E-
4)0.786.1:0.00368)0.733.1:0.00384)0.852.2:0.00738)0.797.2:0.00784,(Ult179
74:0.0037,Ult17975:5.5E-4)0.924.4:0.00349)1.000.5:5.5E-4,Ult18027:5.3E-
4)0.832.1:0.00374)0.801.4:0.00417)0.991.2:5.5E-
4,((Ult17767:0.00742,(Ult17765:0.01502,(RnsPeori:0.01918,((Ult17760:0.0,U
lt17764:0.0,Ult17763:0.0):5.5E-
4,(Ult17762:0.01116,Ult17761:0.00369)0.871.1:5.5E-4)0.926.2:5.5E-
4)0.277.1:5.5E-
4)0.831.2:0.00766)0.865.3:0.00726,((UltClos5:0.00354,(((Ult17912:5.4E-
4,Ult17897:0.09704)0.198.1:0.00778,(Ult17914:0.0079,(((Ult17895:0.00207,U
lt17948:0.00209)0.848.2:0.00711,((Ult17943:0.00367,Ult21387:0.00369)0.995
.1:5.4E-4,((((Ult17910:0.0,Ult17916:0.0,Ult17917:0.0):5.5E-
4,Ult18474:0.03514)0.906.1:0.00802,(Ult17918:0.01139,((Ult17920:0.00764,(
(Ult17915:0.00706,Ult17933:0.03365)0.916.1:5.5E-
4,((Ult17945:0.0,Ult17946:0.0,Ult17947:0.0):0.00211,((((Ult17932:0.0,Ult1
7938:0.0):5.4E-
4,Ult18547:0.01125)0.918.1:0.00247,(Ult17925:0.03039,(((((Ult17921:0.0,Ul
t17928:0.0,Ult17930:0.0,Ult17931:0.0,Ult17940:0.0,Ult17942:0.0):5.5E-
4,Ult17934:5.5E-4)0.000.12:5.5E-
4,(Ult17929:0.0074,Ult17941:0.00369)0.629.1:5.5E-4)0.743.1:5.5E-
4,(Ult17919:0.00371,Ult17922:0.00367)0.678.1:5.5E-4)0.225.1:5.5E-
4,Ult17924:0.01899)0.805.1:5.4E-
4)0.918.2:0.0024)0.918.3:0.00245,Ult21381:5.4E-
4)0.874.1:0.00203)0.915.2:0.00713)0.744.1:0.00363)0.474.1:5.4E-
4,Ult17944:0.01103)0.745.1:0.00387)0.777.1:0.00364)0.833.3:0.00734,(((Ult
17957:0.0,Ult17958:0.0):0.00221,(UltClos4:0.00369,Ult17959:5.5E-
4)0.916.2:0.00696)0.833.4:0.00195,((((Ult17949:5.5E-
4,((Ult17885:0.0,Ult17952:0.0,Ult17953:0.0,CstBacte:0.0,Ult17988:0.0):5.5
E-4,(Ult17951:0.00369,Ult17950:0.01495)0.621.1:5.5E-4)0.728.1:5.5E-
4)0.910.3:5.5E-
4,Ult17954:0.00737)0.766.1:0.00368,Ult20933:0.01922)0.800.1:0.00369,(Ult1
7956:5.5E-4,Ult17955:0.00368)0.808.2:5.5E-4)1.000.6:5.5E-
4)0.783.1:0.00749)0.751.1:0.00374)0.777.2:0.00346)0.915.3:0.0109,((((((Ul
t17966:0.00707,Ult17967:5.5E-
4)0.615.1:0.00387,(Ult17968:0.02144,Ult21449:0.02636)0.829.1:0.01055)0.86
3.1:0.01104,(((Ult17939:0.0,Ult17969:0.0,Ult17970:0.0,Ult17972:0.0,Ult179
73:0.0,Ult22597:0.0):5.5E-4,Ult17971:0.00369)0.954.1:5.5E-

4,((Ult17963:0.0,Ult17964:0.0,Ult17965:0.0):0.00197,(Ult17961:0.00369,(Ul
t17960:0.00369,Ult17962:5.5E-4)0.458.2:5.5E-
4)0.979.1:0.0019)0.988.1:0.01907)0.768.1:0.00396)0.290.1:0.01173,Ult18560
:0.02246)0.792.1:0.00831,((((Ult17879:0.00369,(UltB7200:0.0,Ult17887:0.0,
Ult17878:0.0,Ult17889:0.0,Ult17890:0.0,Ult17891:0.0,Ult17892:0.0,Ult17894
:0.0,Ult21343:0.0):5.5E-4)0.000.13:5.5E-
4,((Ult18008:0.00369,Ult18161:0.01118)0.781.1:0.00369,Ult17886:0.00369)0.
935.1:5.5E-4)0.861.2:5.5E-4,Ult17979:0.01114)0.971.1:5.5E-
4,(Ult17888:0.03111,Ult17893:0.03415)0.722.1:0.00306)1.000.7:5.4E-
4)0.793.1:0.00326,(Ult17880:0.01928,Ult17881:0.09243)0.873.2:0.01077)1.00
0.8:5.4E-
4)0.772.1:0.00315)0.769.1:0.00359)0.873.3:0.00728,(Ult18005:0.00754,((((U
lt17722:0.00376,(Ult17725:0.00752,(Ult17723:0.00369,Ult17724:5.5E-
4)0.856.1:5.5E-
4)0.794.1:0.00364)0.881.2:0.00759,Ult17913:0.00751)0.872.1:0.00715,Ult177
66:0.03028)0.793.2:5.5E-
4,(Ult18016:0.01514,((Ult18011:0.01935,(Ult18014:0.0,Ult18015:0.0):5.5E-
4)0.898.1:0.00745,(Ult17984:0.00398,(Ult18009:0.01966,Ult18010:0.05191)0.
935.2:0.01765)0.318.1:0.00356)0.816.1:5.3E-
4)0.798.2:0.00374)0.805.2:0.00412)0.656.1:0.00347)0.846.1:0.00761)0.868.1
:0.00743,((Ult18002:0.00732,((Ult17999:0.0,Ult18004:0.0):5.5E-
4,((Ult18001:0.10037,Ult18562:0.02657)0.994.2:5.4E-
4,Ult18000:0.00369)0.166.1:5.5E-4)0.714.1:5.4E-
4)0.888.1:0.00803,(Ult18006:0.00778,Ult17994:0.03203)0.758.1:0.00331)0.90
7.2:0.01194)0.901.3:0.00233)0.905.1:0.0015)0.866.1:0.00777,((Ult17718:0.0
1162,Ult17911:0.01145)0.910.4:0.01125,(((Ult18603:0.00945,((Ult17737:0.01
266,(Ult18689:0.0,Ult18701:0.0):0.05325)0.972.2:0.04553,((Ult18550:5.4E-
4,(Ult18549:0.02286,(Ult17726:5.5E-
4,Ult18548:0.00369)0.963.3:0.02719)0.908.2:0.01788)0.933.1:0.00741,(Ult18
553:0.01881,((Ult17864:0.0,Ult18551:0.0,Ult18554:0.0,Ult18555:0.0):5.5E-
4,Ult18552:0.0074)0.219.1:5.5E-4)0.945.1:5.4E-
4)0.977.1:0.04039)0.346.1:0.01445)0.953.1:0.02103,(Ult18583:5.4E-
4,(Ult18580:0.00371,((((UltB7199:0.0,UltB7198:0.0,Ult17501:0.0,Ult17733:0
.0,Ult18577:0.0,Ult18579:0.0,Ult18581:0.0,Ult18582:0.0,Ult18585:0.0,Ult18
586:0.0):5.5E-4,(Ult18590:0.00744,Ult18584:0.00369)0.700.2:5.5E-
4)0.000.14:5.5E-4,(Ult18578:0.0074,Ult18588:0.00369)0.687.1:5.5E-
4)0.834.1:5.5E-4,(Ult18587:0.00368,Ult18589:0.07394)0.774.1:5.5E-
4)0.764.1:5.5E-4)0.826.1:0.00369)0.977.2:0.01912)0.416.1:5.4E-
4,UltClos3:0.01143)0.946.1:0.01116)0.615.2:5.5E-
4)0.897.1:0.01113,((((((Ult19449:0.0,Ult19450:0.0):0.00352,Ult19451:0.003
92)0.960.1:0.02173,((((Ult17502:5.4E-4,((((Ult18691:5.4E-
4,(Ult18672:0.01909,(Ult18687:0.00369,((Ult18670:0.01115,Ult18686:0.00369
)0.666.1:5.5E-
4,(Ult18681:0.0,Ult18669:0.0,Ult18671:0.0,Ult18673:0.0):5.5E-
4)0.910.5:5.5E-4)0.979.2:5.4E-4)0.663.1:0.00369)0.955.3:5.4E-
4,(UltEub38:0.0,Otu00356:0.0):0.06642)0.811.1:0.00763,((Ult18692:0.00743,
(Ult18698:0.00369,(Ult18695:5.5E-
4,(Ult18694:0.01116,Ult18697:0.00369)0.763.2:5.5E-4)0.995.2:5.5E-
4)0.968.1:0.01958)0.868.2:0.00809,Ult18682:0.00358)0.763.3:0.00377)0.881.
3:0.0075,((((Ult18674:0.00369,(Ult18679:0.02343,Ult18677:0.03142)0.873.4:
5.5E-4)0.177.1:5.5E-4,UltClo11:0.00369)0.453.1:5.5E-
4,(Ult18675:0.0,Ult18676:0.0,Ult18668:0.0,Ult18690:0.0,Ult18688:0.0,Ult18
693:0.0):5.5E-4)0.237.1:5.5E-4,Ult18680:5.5E-4)0.946.2:5.5E-
4)0.931.1:0.01981)0.344.1:0.01864,(UltEub39:0.04119,(UltClo57:0.02071,Ult

Clo58:0.0116)0.964.1:0.03321)0.933.2:0.02219)0.898.2:0.01674,((Ult18464:0
.019,(Ult18700:0.0,Ult18699:0.0):0.13222)0.889.2:0.02181,Ult17540:0.01534
)0.762.1:0.00725)0.431.1:0.00801,(((Ult18718:0.00713,(UltB7248:0.00369,Ul
t18719:0.00367)0.940.1:5.4E-
4)0.351.1:0.01223,(Ult23634:0.03522,(((Ult18705:0.00366,Ult18707:0.00737)
0.926.3:5.4E-
4,(Ult18713:0.0,Ult18715:0.0,Ult18716:0.0,Ult18706:0.0,Ult18708:0.0,Ult18
717:0.0):5.5E-4)0.552.1:5.5E-4,(Ult18703:0.05042,(Ult18704:5.4E-
4,Ult18702:0.11551)0.921.1:0.01502)0.741.1:0.00339)0.731.1:0.00357)0.932.
1:0.02814)0.679.1:0.0389,((Ult18723:0.00363,((Ult18721:0.00729,((Ult18720
:0.00366,Ult18722:0.00734)0.792.2:0.00361,Ult18728:0.02245)0.910.6:5.5E-
4)0.035.2:5.5E-4,(Ult18724:0.0,Ult18725:0.0):5.5E-4)0.949.1:5.2E-
4)0.887.1:0.01227,(Ult18726:0.02373,Ult18727:0.02728)0.958.1:0.02834)0.76
0.1:0.01589)0.970.1:0.05303)0.898.3:0.02187)0.363.1:0.00396,(Ult20867:0.0
3785,UltFir13:0.03169)0.962.1:0.03634)0.891.1:0.01267,((Ult18566:0.06809,
(Ult18567:0.0,Ult18568:0.0):5.5E-4)0.989.2:0.01988,(Ult18569:5.4E-
4,Ult18571:0.01138)0.749.1:0.00299)0.873.5:0.00807)0.746.2:0.00365,((Ult1
8592:0.06384,Ult18591:5.5E-
4)0.983.1:0.02756,((Ult17682:0.04879,Ult20450:0.02701)0.856.2:0.01387,(Ul
t19568:0.07321,(Ult18611:0.12446,Ult19590:0.06603)0.895.1:0.04387)0.762.2
:0.01519)0.851.1:0.01045)0.854.1:0.01338)0.785.1:0.00422)0.891.2:5.4E-
4,(UltClo55:0.00495,UltClo56:0.03989)0.944.1:0.01855)0.950.2:0.01618,((Un
cUn127:0.0,UncUn128:0.0,UncUn129:0.0):0.01106,(Ult18082:0.02359,((Ult1948
2:0.0326,(Ult20795:0.02711,(Ult19483:0.00377,Ult20747:0.02303)0.871.2:5.4
E-4)0.900.1:0.01096)0.285.1:5.4E-
4,(Ult18085:0.00771,Ult18084:0.0035)0.860.1:0.00748)0.432.1:0.00785)0.925
.3:0.0162)0.872.2:5.5E-
4)0.786.2:0.00424,((CsrSpec3:0.0,Otu00005:0.0):5.5E-4,((Ult19425:5.5E-
4,(BacNL215:5.5E-
4,(BacNL216:0.00749,Ult19426:0.00376)1.000.9:0.00169)1.000.10:0.00193)0.9
32.2:0.00745,(CsrIndol:5.5E-
4,(CsrMetho:0.0,CsrSpec5:0.0):0.0037)0.501.1:5.5E-
4)0.835.3:0.00367)0.748.1:0.00359)0.842.1:0.0077)0.237.2:5.5E-
4,(((Cuiii23:0.04865,Ult21287:0.00986)0.995.3:0.04861,(((UltClo10:0.0201
4,(UltClos2:0.01651,((Ult17813:0.01283,Ult17814:0.01849)0.810.2:0.01259,(
Ult19007:0.03225,(UltRu310:0.03785,(Ult17675:5.5E-
4,((Ult17676:0.0,Ult17677:0.0,Ult17678:0.0):0.00549,(Ult17679:5.4E-
4,(Ult17680:0.02812,Ult18711:0.04116)0.742.1:0.00201)0.884.3:0.01008)0.92
6.4:0.01808)0.894.1:0.02079)0.392.1:0.01306)0.848.3:0.02549)0.889.3:0.019
59)0.847.1:0.00767)0.875.2:0.01339,((((Ult21286:0.04301,Ult17673:0.00195)
0.832.2:0.00855,Ult17674:0.02757)0.830.1:0.00918,(UltClo59:0.01935,Ult197
63:0.06209)0.820.1:5.5E-
4)0.936.1:0.01635,((Ult17624:0.01554,Ult19486:0.01556)0.875.3:0.01265,(((
(((Ult17510:0.00368,((Ult17513:0.00369,Ult17514:0.00369)0.934.3:5.5E-
4,((Ult17505:0.00369,Ult18678:0.00369)0.900.2:5.5E-
4,((Ult17506:0.0,Ult17507:0.0,Ult17508:0.0,Ult17509:0.0,Ult17511:0.0,Ult1
7515:0.0,Ult17516:0.0,Ult17522:0.0,Ult17523:0.0,Ult17527:0.0,Ult17528:0.0
,Ult17856:0.0):5.5E-4,(Ult17504:0.00369,Ult17512:0.00369)0.898.4:5.5E-
4)0.000.15:5.5E-4)0.000.16:5.5E-4)0.902.1:5.5E-4)0.839.1:5.3E-
4,(Ult17683:0.00756,Ult17500:0.04374)0.752.1:0.00361)0.962.2:0.01125,((Ul
t18576:0.00752,Ult22386:0.00365)0.928.2:0.01112,(Ult17517:0.00369,Ult1751
8:5.5E-4)0.932.3:0.011)0.870.1:5.5E-
4)0.951.2:0.01847,Ult17684:0.0185)0.795.1:0.00833,((Ult19628:0.0112,(((((
((Ult18520:0.00369,Ult18521:0.00369)0.921.2:5.3E-

4,(((Ult18514:0.00372,(Ult18513:0.00371,((((Ult18526:0.00356,Ult19380:0.0
1547)0.844.3:0.01943,Ult18511:0.01121)1.000.11:5.5E-
4,((Ult18457:0.0,Ult18519:0.0,Ult18518:0.0,Ult18523:0.0,Ult18527:0.0,Ult1
8528:0.0,Ult18529:0.0,Ult18531:0.0,Ult18532:0.0,Ult21464:0.0):5.5E-
4,(Ult18522:0.01508,Ult18530:0.01496)0.845.1:5.5E-4)0.969.1:5.5E-
4)0.868.3:0.0037,Ult18524:0.0037)0.391.1:5.5E-
4)0.838.1:0.00371)0.830.2:5.5E-
4,Ult18533:0.02283)0.846.2:0.00759,Ult18512:0.00763)0.896.1:0.00742)0.954
.2:0.01834,(((Ult17658:0.04354,((Ult18456:0.0,Ult18451:0.0,Ult18454:0.0,U
lt18460:0.0):5.5E-4,Ult18453:0.00369)0.395.1:5.4E-4)0.821.1:5.4E-
4,((Ult18459:0.0,Ult18450:0.0,Ult18452:0.0,Ult18455:0.0,Ult18461:0.0,Ult1
8525:0.0):5.5E-
4,Ult21341:0.05129)0.853.1:0.00339)0.901.4:0.01703,(((Ult18468:0.0,Ult184
69:0.0):5.4E-
4,((Ult18436:0.00609,Ult18449:0.00636)0.800.2:0.00613,(Ult18467:0.01547,U
lt18462:0.00734)0.291.1:0.00363)0.872.3:5.4E-4)0.877.1:5.5E-
4,(Ult18472:0.03123,(((Ult18447:0.01492,(((((Ult18432:0.0,Ult18431:0.0,Ul
t18433:0.0,Ult18440:0.0,Ult18441:0.0,Ult18442:0.0,Ult18444:0.0):5.5E-
4,((Ult18434:5.4E-
4,Ult18446:0.01131)0.840.1:0.0037,Ult18465:0.00369)0.743.2:5.5E-
4)0.868.4:5.5E-4,Ult21339:5.5E-4)0.529.1:5.5E-4,Ult18466:5.5E-
4)1.000.12:5.4E-
4,Ult18437:0.05497)0.682.1:0.00572)0.798.3:0.00592,((Ult18438:5.5E-
4,(Ult18445:0.00741,Ult18463:0.00369)0.695.1:5.5E-4)0.878.2:5.5E-
4,(Ult18439:0.01121,Ult18443:0.00745)0.811.2:5.4E-4)0.997.1:5.4E-
4)0.923.2:0.00744,(Ult18448:0.05363,Ult18470:0.00347)0.360.1:5.3E-
4)0.920.1:0.01124)0.761.2:0.00362)0.952.2:0.02391)0.826.2:0.00961)0.787.2
:0.00833,((Ult18501:0.00739,((UltB6921:0.0,Ult18489:0.0,Ult18492:0.0,Ult1
8493:0.0,Ult18494:0.0,Ult18495:0.0,Ult18502:0.0):5.5E-
4,(Ult18490:0.03425,(Ult18496:0.03623,((Ult18485:0.01154,(Ult18478:0.0112
3,(Ult18484:0.0,Ult18491:0.0):5.5E-
4)0.797.3:0.00361)0.770.1:0.00389,(Ult18486:5.3E-
4,(Ult18483:0.01148,((Ult18482:5.5E-
4,Ult18487:0.00753)0.792.3:0.00361,((Ult18479:0.0,Ult18480:0.0,Ult18481:0
.0):5.5E-
4,Ult17539:0.01523)0.905.2:0.00773)0.744.2:0.00383)0.894.2:0.01547)0.864.
2:0.00725)0.777.3:0.00453)0.959.1:0.01909)0.900.3:5.4E-4)0.759.1:5.5E-
4)0.937.1:0.01154,((UltClo12:0.08511,((Ult18498:5.4E-
4,(Ult18488:0.00369,Ult18650:5.5E-
4)0.997.2:0.03145)0.774.2:0.00433,(Ult18475:0.00144,Ult18476:0.03866)0.94
7.2:0.01776)0.725.1:0.00594)0.704.1:0.0048,((BactCTm2:0.0,Ult18499:0.0):5
.5E-4,Ult18497:5.5E-4)0.933.3:0.01122)0.770.2:5.5E-
4)0.875.4:0.01238)0.897.2:0.01505,Ult18561:0.02598)0.776.2:0.01192,Ult184
73:0.01237)0.915.4:0.01459,(((Ult19336:0.00369,Ult19335:0.00741)0.772.2:5
.5E-
4,((UltB7244:0.0,Ult19339:0.0,Ult19340:0.0,Ult19342:0.0,Ult19343:0.0,Ult1
9344:0.0):5.5E-4,(Ult19341:0.02729,Ult19338:5.3E-
4)0.847.2:0.00369)0.747.2:5.5E-4)0.752.2:5.4E-
4,(Ult19337:0.04642,Ult20094:0.01471)0.978.1:5.4E-4)0.898.5:5.5E-
4)0.923.3:0.01128)0.760.2:0.00416,Ult17525:0.03094)0.770.3:0.00947)0.918.
4:0.01703,((UltClo25:0.03273,(Ult18083:0.03592,(Ult17713:0.06231,Ult17714
:0.00632)0.991.3:0.05553)0.498.1:0.01295)0.804.1:0.01363,((((Ult18826:0.0
1808,(Ult21126:0.03158,Ult21350:0.01301)0.959.2:0.03718)0.469.1:0.0161,((
((UltEub37:0.08441,(((Ult20413:0.03537,(Ult20409:0.00742,((UltB6919:0.0,U

lt20410:0.0,Ult20411:0.0):5.5E-4,Ult20412:0.00369)0.185.1:5.5E-
4)0.745.2:0.00331)0.894.3:0.01456,((Ult18683:0.00652,(Ult18684:0.00369,Ul
t18685:5.5E-
4)0.847.3:0.00888)0.966.2:0.03158,Ult18570:0.01207)0.969.2:0.02879)0.867.
2:0.01388,Ult18730:0.05395)0.471.1:0.00972)0.706.1:0.00719,(UltLac12:0.02
476,(((Ult18828:0.00367,(Ult18829:5.5E-
4,(Ult21309:0.00745,(Ult18827:0.00369,BcrPecti:5.5E-
4)0.855.1:0.0037)0.883.1:5.5E-4)0.846.3:5.4E-
4)0.994.3:0.03548,(UdnRum24:0.04838,UltRu317:0.00413)0.753.1:0.00421)0.92
7.1:0.02073,(BtvSpeci:0.05606,(Ult18227:0.00725,AbiDefec:5.5E-
4)0.999.1:0.08823)0.935.3:0.03712)0.785.2:0.00818)0.793.3:0.02118)0.875.5
:0.01895,(((((UltFir11:0.04573,(UltLac10:0.03142,Otu00349:0.01829)0.911.1
:0.02454)0.872.4:0.01615,(Otu00418:0.01885,(UltLach9:0.01747,(UltEub35:5.
4E-
4,Otu00220:0.02307)0.925.4:0.0216)0.855.2:0.00821)0.730.2:0.00412)0.972.3
:0.02519,(((Ult20458:0.02976,Ult20457:0.02062)0.764.2:0.00829,(Ult20459:0
.00762,Ult20483:0.03552)0.890.2:0.01186)0.758.2:0.00401,(Ult20455:0.01532
,Ult20456:0.00731)0.924.5:0.01161)0.750.1:0.00393)0.873.6:5.5E-
4,(((((((Ult18613:0.02666,((Ult18615:0.023,(Ult18612:0.0,Ult18616:0.0):5.
5E-4)0.668.1:5.3E-
4,(Ult18614:0.02659,Ult21408:0.02701)0.744.3:0.00377)0.758.3:0.0037)0.754
.1:0.00515,(Ult18187:0.01878,(Ult18619:0.00369,(Ult18617:0.0,Ult18618:0.0
,Ult18620:0.0):5.5E-4)0.857.2:5.4E-
4)0.967.1:0.02182)0.109.1:0.00843,(Ult18621:0.01251,Ult18622:0.02648)0.95
0.3:0.02622)0.945.2:0.02316,(((Ult18633:0.02235,Ult18636:0.03043)0.809.2:
5.4E-
4,(Ult18627:0.01796,Ult18630:0.04961)0.417.1:0.0089)0.842.2:0.00333,(((Ul
t18626:0.0,Ult18628:0.0,Ult18632:0.0,Ult18635:0.0,Ult18803:0.0):5.5E-
4,(Ult18629:0.01774,Ult18637:0.01102)0.418.1:5.5E-4)0.440.1:5.5E-
4,Ult18631:0.00729)0.899.1:5.5E-
4)0.907.3:0.01588)0.827.3:0.01403,(((Ult24428:0.00365,(((Ult22390:0.00369
,(((((Ult18638:0.0,Ult18640:0.0,Ult18641:0.0,Ult24427:0.0):5.5E-
4,Ult18639:0.00368)0.621.2:5.5E-
4,(Ult18661:0.0,Ult18662:0.0):0.01119)0.846.4:0.00364,(Ult18435:0.00738,U
lt18665:5.5E-4)0.260.1:0.00369)0.685.1:5.5E-
4,((Ult18651:0.0,Ult18652:0.0,Ult18655:0.0,Ult18659:0.0,Ult18657:0.0,Ult1
8658:0.0,Ult19545:0.0):5.5E-
4,(Ult18654:0.00741,(Ult18644:0.0222,Ult18656:0.0631)0.768.2:0.00471)0.23
2.1:5.5E-4)0.000.17:5.5E-4)0.000.18:5.5E-4)0.413.1:5.5E-
4,Ult18653:0.00369)0.367.1:5.5E-4,Ult18643:0.00367)0.204.1:5.5E-
4)0.852.3:5.5E-4,Ult18646:0.05155)0.428.1:5.4E-
4,((Ult18648:0.06507,Ult18663:0.05501)0.347.1:0.00715,Ult18660:0.01502)0.
754.2:5.4E-
4)0.888.2:0.00994)0.716.1:0.00633,Ult18236:0.04048)0.604.1:0.01056,(Ult18
235:0.04241,((Ult20452:0.0079,((Ult20442:0.0343,((Ult20440:0.0,Ult20443:0
.0):0.00369,(Ult20444:0.0,Ult20445:0.0):5.5E-4)0.919.1:5.4E-
4)0.945.3:0.02364,((Ult20451:0.0,UltClo43:0.0):0.02337,((Ult18625:0.0,Ult
18624:0.0):0.04334,Ult20453:0.01939)0.630.1:0.01412)0.769.2:0.00782)0.920
.2:0.01679)0.786.3:5.4E-4,(Ult18734:0.00741,Ult20454:5.5E-
4)0.890.3:0.01044)0.905.3:0.01649)0.714.2:0.0047)0.892.1:0.0126)0.881.4:0
.01387,((Ult20717:0.04052,Ult20781:0.18049)0.889.4:0.0501,(Ult19726:0.232
39,Ult20238:0.25414)0.649.1:0.06584)0.806.1:0.02052)0.759.2:0.00688)0.955
.4:0.0221,(((Ult18740:5.5E-
4,((UltClo19:0.00366,Ult18738:0.00375)0.926.5:0.01098,Ult18739:0.00201)0.

926.6:0.00199)0.952.3:5.5E-
4,Ult19290:0.02278)0.914.1:0.00682,(((Ult18349:0.00367,Ult18825:0.00372)0
.929.1:0.00763,CsrPolys:0.02318)0.068.1:5.4E-
4,Ult21267:0.02732)0.383.1:5.3E-
4)0.093.1:0.00398)0.763.4:0.00776)0.913.1:0.0179,UncUn134:0.01984)0.742.2
:0.00598,UltClo62:0.00893)0.888.3:0.01392)0.717.1:0.00539)0.701.1:0.00389
)0.872.5:0.00868)0.839.2:0.00769)0.746.3:0.00489,((((Ult19390:0.01664,((U
lt18813:0.02949,((Ult18812:0.06147,((((Ult18817:0.05199,(Ult18284:0.03073
,UltRu321:0.02272)0.120.1:0.0053)0.381.1:0.01502,Ult18811:0.01417)0.806.2
:0.01125,(Ult18808:0.02967,(Ult18809:0.01666,Ult18810:0.0179)0.769.3:0.00
706)0.603.1:0.00612)0.914.2:0.02126,Ult18806:0.02582)0.107.1:0.01767)0.56
6.1:0.00167,(UltRu320:0.02913,Ult18807:0.01025)0.839.3:0.00939)0.751.2:0.
00602)0.868.5:0.02291,((Ult18535:0.00835,(Ult18951:0.04218,(Ult18534:5.5E
-
4,((Ult18712:0.00367,(Ult18543:0.00369,(Ult18541:0.0,Ult18540:0.0,Ult1854
2:0.0,Ult18544:0.0):5.5E-4)0.402.1:5.5E-
4)1.000.13:0.05258,(Ult21354:0.0427,(Ult17538:0.00369,(Ult17537:0.0,Ult17
735:0.0):5.5E-4)0.397.1:5.5E-4)0.065.1:5.4E-
4)0.302.2:0.00364)0.953.2:0.02288)0.771.2:0.01326)0.302.3:0.00444,Ult1853
6:0.05519)0.960.2:0.02524)0.870.2:5.3E-
4)0.758.4:0.00396,(((((Ult18995:0.00223,(UltRu340:0.0111,(Ult18994:0.0073
2,((Ult18993:0.0,Ult18992:0.0,Ult22498:0.0):5.5E-
4,(Ult18996:0.00366,UltB7247:5.5E-4)0.770.4:5.5E-4)0.804.2:5.5E-
4)0.739.1:5.5E-
4)0.880.2:0.00891)0.920.3:0.01913,(Ult18997:0.06864,((Ult19000:0.03608,(U
lt18999:0.01505,Ult18998:0.00942)0.952.4:0.03302)0.928.3:0.02368,((Ult189
50:0.06111,Ult19001:0.00647)0.876.1:0.02434,(Ult19034:0.03605,(Ult19033:0
.0171,UltRu353:0.02696)0.024.1:0.00767)0.871.3:0.01915)0.880.3:0.01347)0.
769.4:0.00459)0.762.3:0.00617)0.886.2:0.01767,((Ult20692:0.01201,((Ult206
91:0.00908,(Ult20690:5.4E-
4,(UltRu370:0.01138,UltRu369:0.0114)0.776.3:0.00365)0.774.3:0.00595)0.991
.4:0.04976,(Ult20693:0.03217,(Ult20694:0.00367,Ult20695:5.5E-
4)0.747.3:0.00327)0.545.1:0.01171)0.700.3:0.00421)0.992.1:0.05107,((UltRu
347:0.03554,UltRu346:0.04166)0.852.4:0.03222,((Ult19004:0.00424,Ult19005:
0.00307)0.994.4:0.06223,(Ult19002:0.01905,Ult19003:0.08895)0.843.1:0.0179
2)0.488.1:0.02103)0.844.4:0.01574)0.205.1:0.0167)0.725.2:5.4E-
4,(((Ult18986:0.00731,((LchBact2:0.00367,(Ult18989:0.00368,(((Ult18982:0.
0,Ult18985:0.0,Ult18988:0.0):5.5E-
4,(Ult21365:0.00369,Ult18983:0.00369)0.896.2:5.5E-4)0.000.19:5.5E-
4,(Ult18981:0.01489,Ult18984:0.03958)0.922.1:5.5E-4)0.202.1:5.5E-
4)0.209.1:5.5E-4)0.323.1:5.5E-4,Ult18987:5.5E-4)0.668.2:5.5E-
4)0.720.1:0.0048,(Ult18816:0.03933,(Ult18814:0.01428,Ult18815:0.00526)0.8
79.2:0.01838)0.997.3:0.07348)0.887.2:0.02378,(Ult18991:0.05505,UltRu348:0
.03879)0.893.2:5.4E-
4)0.824.3:0.01123)0.949.2:0.02921,((Ult18735:0.03151,((Ult21451:0.00773,(
Ult21452:5.3E-
4,Ult21453:0.04505)0.195.1:0.01514)0.939.1:0.03162,(Ult21455:0.04085,Ult2
1454:0.01646)0.894.4:0.01861)0.720.2:0.02455)0.960.3:0.039,(((UltB7225:0.
07726,UltB7228:0.07522)0.990.1:0.08688,(UltB6977:0.13064,(UltB6978:0.0696
6,UltB6979:0.15454)0.174.1:0.00137)0.913.2:0.05051)0.951.3:0.04628,((UltB
1988:5.4E-
4,Ult21431:0.07219)0.985.2:0.05346,(Ult21402:0.09401,Hmyy0040:0.14753)0.7
48.2:0.03127)0.703.2:0.00908)0.898.6:0.02579)0.462.1:0.01288)0.766.2:0.00
563)0.895.2:0.01337,((UltRu356:0.03941,(Ult19466:0.01977,UltRu355:0.01654

)0.961.1:0.03581)0.957.1:0.03853,((Ult19391:0.07331,((Ult19346:0.02641,Ul
t19347:0.01002)0.921.3:0.02849,((Ult18517:0.03877,(Ult18515:5.5E-
4,Ult18516:0.00371)0.973.1:5.4E-
4)0.985.3:0.05749,(Ult18955:0.04519,Ult19465:0.04938)0.338.1:0.00751)0.87
4.2:0.02464)0.444.1:0.00659)0.919.2:0.02648,(Ult19392:0.00732,(Ult19388:0
.00284,Ult19389:0.00463)0.971.2:0.0311)0.763.5:0.01263)0.857.3:5.4E-
4)0.872.6:0.01749)0.504.1:0.00888,(FirmOral:0.02906,((((Ult18939:5.5E-
4,(((BtvFibri:5.4E-4,(UltRu334:5.4E-4,(UltRu333:0.00743,BtvFibr3:5.4E-
4)0.272.1:0.01141)0.970.2:0.01523)0.069.1:5.4E-4,(((BtvHunga:5.5E-
4,Ult18941:5.5E-4)0.795.2:0.00356,((BtvFibr2:5.5E-
4,(((Ult18940:0.0,Ult18947:0.0):5.5E-4,UltRu337:5.5E-
4)0.925.5:0.00742,BtvSpec2:0.00371)0.871.4:5.5E-4)0.803.1:5.5E-
4,(UltRu336:5.5E-
4,Ult18949:0.0037)0.857.4:0.00369)0.771.3:0.00395)0.882.1:0.00777,(Ult189
44:0.01891,(UltRu335:0.00739,((((UltRu332:0.0,BtvFibr4:0.0):5.5E-
4,Ult18946:5.5E-4)0.000.20:5.5E-4,Ult18948:5.5E-4)0.739.2:5.5E-
4,BtvProte:5.5E-4)0.888.4:5.5E-4)0.984.1:5.5E-
4)0.788.1:0.00348)0.870.3:0.00779)0.914.3:0.00745,UdnRum25:0.00371)0.761.
3:5.3E-4)0.937.2:5.4E-4,((BtvFibr5:0.03137,(Ult18943:5.5E-
4,Ult18945:5.5E-
4)0.988.2:0.03549)0.926.7:0.02576,UdnRum26:0.00346)0.786.4:0.00392)0.892.
2:0.01538,UdnRum27:0.01962)0.908.3:0.01897,(UltRu350:0.03929,(Ult18979:0.
00419,Ult18978:0.01501)0.997.4:0.07545)0.894.5:0.02398)0.198.2:0.01744)0.
913.3:0.02209)0.871.5:0.00978)1.000.14:0.00169)0.952.5:0.00198,((((((Ult1
9043:0.02064,(Ult17810:0.00633,Ult19044:0.03386)0.952.6:0.02862)0.842.3:0
.01569,(Ult19036:5.5E-4,(Ult19035:0.00741,UltRu352:5.5E-
4)0.924.6:0.00745)0.952.7:0.02139)0.905.4:0.01718,((UltRu354:0.02105,(Ult
19055:0.02294,Ult19054:5.3E-
4)0.899.2:0.01721)0.555.1:0.01234,(EubCellu:0.0284,Ult19032:0.0242)0.780.
2:0.00702)0.485.1:5.4E-4)0.902.2:0.00971,(((Ult21358:5.4E-
4,(Ult19037:0.00369,((Ult19038:0.0,Ult19040:0.0,Ult19041:0.0):5.5E-
4,Ult19039:5.5E-4)0.267.1:5.5E-
4)0.838.2:0.00363)0.964.2:0.02623,(Ult19222:0.01593,(Ult21257:0.02863,(((
((Ult19372:0.0,Ult19373:0.0):5.5E-
4,(Ult19379:0.00745,Ult19378:0.00369)0.752.3:5.5E-4)0.809.3:5.5E-
4,(Ult18556:0.01888,(Ult19375:0.00369,Ult19374:5.5E-4)0.998.1:5.5E-
4)0.948.1:0.00746)0.866.2:0.0037,Ult19377:5.4E-4)0.698.1:5.4E-
4,Ult19376:0.01113)0.713.1:0.00314)0.955.5:0.01986)0.773.1:0.01032)0.798.
4:0.00713,((Ult19217:0.00722,((Ult19218:0.0,Ult19219:0.0):5.5E-
4,Ult19220:0.01523)0.463.2:5.4E-
4)0.925.6:0.01351,(Ult19046:0.02812,(Ult19050:0.00372,((Ult19045:0.0,Ult1
9047:0.0,Ult19049:0.0):5.5E-
4,Ult21182:0.0037)0.799.1:0.0037)0.788.2:0.00478)0.612.1:0.00723)0.798.5:
0.0075)0.763.6:0.00585)0.765.2:0.00394,((Ult20141:0.0,Ult20142:0.0):0.014
15,(((Ult19028:0.0,Ult19029:0.0):5.5E-
4,Ult19030:0.0519)0.965.1:0.01546,((Ult19051:0.0,Ult19053:0.0):0.01927,Ul
t19052:5.4E-
4)0.898.7:0.0114)0.776.4:0.00502)0.782.3:0.00378)0.727.1:0.00416,((((Ult1
9250:0.00751,((Ult18061:0.0294,(((Ult18059:0.0375,(((Ult17497:0.15766,Ult
20265:0.03714)0.807.1:0.07392,Ult18140:0.11516)0.915.5:0.04092,(Ult18054:
0.23415,Ult19149:0.07664)0.591.2:0.02328)0.910.7:0.02456)0.651.1:0.01401,
((Ult18292:0.04587,((Ult19285:0.0,Ult19294:0.0):5.4E-
4,(Ult21322:0.02315,(Ult21189:0.01131,(Ult21190:0.0038,(Ult21149:0.04682,
Ult22384:0.01812)0.762.4:0.00483)0.866.3:0.00751)0.992.2:0.02759)0.808.3:

0.0036)0.400.1:0.01361)0.876.2:0.01392,((Ult19889:0.0285,(Ult19145:0.0073
7,(Ult19204:5.5E-4,((((Ult19144:0.00371,(Ult19156:0.0037,Ult19182:5.5E-
4)0.078.1:5.5E-
4)0.859.3:0.00372,(((((Ult19756:0.00371,Ult19140:0.00372)0.000.21:5.5E-
4,((Ult19143:0.01122,((((UltB7187:0.0,Ult18304:0.0,Ult19292:0.0,Ult19141:
0.0,Ult19135:0.0,Ult19146:0.0,Ult19148:0.0,Ult19151:0.0,Ult19154:0.0,Ult1
9155:0.0,Ult19157:0.0,Ult19158:0.0,Ult19169:0.0,Ult19170:0.0,Ult19171:0.0
,Ult19180:0.0,Ult19181:0.0,Ult19184:0.0,Ult19188:0.0,Ult19191:0.0,Ult1919
3:0.0,Ult19192:0.0,Ult19199:0.0,Ult19208:0.0,Ult19207:0.0,Ult19927:0.0,Ul
t20075:0.0,Ult20074:0.0,Ult20076:0.0,Ult20111:0.0,Ult20633:0.0,Ult21366:0
.0,Ult21885:0.0,Ult22391:0.0):5.5E-
4,(Ult21374:0.0037,(((UltB7188:0.06086,Ult19200:0.00722)0.741.2:0.0042,((
Ult19167:0.0,Ult19187:0.0,Ult20696:0.0,Ult21372:0.0):0.00357,((Ult21307:0
.0734,(Ult19186:0.0299,UltB7215:0.02446)0.100.1:0.00685)0.741.3:5.4E-
4,Ult20185:0.07817)0.524.1:0.01124)1.000.15:5.4E-4)0.778.3:5.5E-
4,(Ult18306:0.0021,(Ult19196:5.5E-
4,Ult19209:0.01141)0.920.4:0.00696)0.884.4:0.00208)0.000.22:5.5E-
4)0.028.1:5.5E-4)0.000.23:5.5E-4,Ult19190:0.00371)0.000.24:5.4E-
4,(Ult19137:0.01924,Ult19152:0.01137)0.778.4:0.00357)0.251.1:5.5E-
4)0.458.3:5.2E-
4,(((Ult19168:0.0,Ult19189:0.0):0.0037,(Ult19147:0.00371,Ult21373:0.00371
)0.274.1:5.5E-4)0.937.3:5.5E-
4,(Ult19164:0.0037,Ult19197:0.0037)0.959.3:5.5E-4)0.000.25:5.5E-
4)0.218.1:5.5E-4)0.000.26:5.5E-
4,(Ult19205:0.0037,Ult19153:0.00371)0.000.27:5.5E-4)0.000.28:5.5E-
4,Ult19150:0.01129)0.000.29:5.5E-
4,(Ult18069:0.00747,(Ult18305:0.00196,Ult19713:0.00371)1.000.16:0.00173)0
.000.30:5.5E-4)0.000.31:5.5E-4)0.172.1:5.4E-4,Ult19142:5.5E-
4)0.391.2:5.5E-4,Ult19183:5.5E-4)0.276.1:5.5E-4)0.241.1:5.5E-
4)0.852.5:5.4E-4)0.235.1:5.4E-
4,(Ult20789:0.00529,Ult19136:0.06357)0.759.3:0.0058)0.576.1:0.00447)0.910
.8:0.0243)0.768.3:0.00944,(Ult18051:0.08576,((((((Ult18049:0.00369,((Ult1
8095:0.01117,(Ult18090:0.00742,(Ult18094:5.5E-
4,(Ult18092:0.00369,Ult18091:0.00371)0.736.1:5.5E-4)0.887.3:5.5E-
4)0.849.1:5.5E-4)0.897.3:0.01515,Ult18093:0.02693)0.994.5:5.4E-
4)0.962.3:5.5E-4,(Ult18062:0.00369,Ult20202:0.0037)0.757.1:5.5E-
4)0.000.32:5.5E-
4,(Ult18041:0.0,Ult18064:0.0,Ult18068:0.0,Ult19293:0.0,Ult21353:0.0):5.5E
-4)0.587.1:5.4E-4,Ult18067:5.5E-
4)0.997.5:0.0022,(Ult18088:0.00368,((Ult18086:0.0,Ult18087:0.0):5.5E-
4,Ult18089:5.5E-4)0.961.2:5.4E-
4)1.000.17:0.0398)0.989.3:0.00158,((Ult18066:0.00738,(((((Ult18097:0.0037
2,(Ult18098:0.0,Ult18099:0.0,Ult18100:0.0):0.01526)0.801.5:0.00371,(Ult20
748:0.00369,Ult18070:0.00467)0.000.33:5.5E-4)0.123.1:5.5E-
4,((Ult18045:0.00747,Ult18046:0.00371)0.789.1:5.4E-
4,(Ult18047:0.0,Ult18048:0.0):5.5E-4)0.919.3:0.00369)0.300.1:5.5E-
4,Ult19828:0.00369)0.489.1:5.5E-
4,(Ult18055:0.0,Ult18057:0.0,Ult18058:0.0,Ult18042:0.0,Ult18050:0.0,Ult18
060:0.0,Ult18063:0.0,Ult18065:0.0):5.5E-4)0.535.1:5.3E-4)0.125.1:5.4E-
4,(Ult18043:0.01883,Ult18044:5.5E-4)0.903.1:0.00733)0.308.1:5.5E-
4)0.782.4:0.00445)0.756.2:0.00458)0.415.1:5.4E-
4)0.581.1:0.00242,((UltFir14:0.03643,(Ult19471:0.02479,(Ult20537:0.01504,
(Ult20872:0.00369,(Ult21351:0.00369,Ult20871:5.5E-4)0.004.1:5.5E-
4)0.917.1:5.4E-

4)0.865.4:0.0098)0.831.3:0.01077)0.685.2:0.01636,Ult20710:0.0385)0.822.1:
0.02362)0.752.4:0.0083)0.805.3:0.00528,((((Ult21462:0.00396,Ult21461:0.00
353)0.969.3:0.02029,(Ult18868:0.00743,Ult20140:5.5E-
4)0.827.4:0.00735)0.915.6:0.01172,(((Ult21025:0.0235,(Ult20978:5.3E-
4,((((((((Ult21028:0.00369,Ult21034:0.00369)0.867.3:5.5E-
4,(Ult21041:0.00744,Ult20215:0.00369)0.645.1:5.5E-4)0.000.34:5.5E-
4,(Ult20214:0.0074,Ult21027:0.00368)0.651.2:5.5E-4)0.000.35:5.5E-
4,Ult21020:0.00369)0.000.36:5.5E-
4,((Ult20986:0.0,Ult21024:0.0,Ult21019:0.0,Ult21021:0.0,Ult21018:0.0,Ult2
1032:0.0,Ult21033:0.0,Ult21035:0.0,Ult21042:0.0,Ult21045:0.0,Ult21046:0.0
,Ult21047:0.0,Ult21395:0.0):5.5E-4,Ult22385:0.00369)0.000.37:5.5E-
4)0.885.2:5.5E-4,UltB7184:5.5E-4)0.416.2:5.5E-4,Ult18742:5.5E-
4)0.571.1:5.3E-4,((Ult21048:5.5E-
4,Ult23781:0.00739)0.842.4:0.00366,(Ult21026:0.01137,(Ult21031:0.00369,((
(Ult20216:0.0,Ult21039:0.0):5.5E-
4,((Ult21049:0.00372,(Ult20842:0.0037,Ult21030:0.00371)0.789.2:0.00371)0.
773.2:0.0037,(Ult18746:0.01493,Ult24024:5.3E-
4)0.963.4:0.0149)0.948.2:5.5E-4)0.000.38:5.5E-
4,(Ult21038:0.00369,Ult21029:0.00369)0.916.3:5.5E-4)0.915.7:5.5E-
4)0.966.3:5.5E-4)0.852.6:0.00369)0.107.2:5.5E-
4)0.949.3:0.0073)0.774.4:0.00345)0.840.2:0.00757,((((Ult20975:0.00757,((U
lt19807:0.01126,(((Ult19990:5.5E-
4,((((UltRumi3:0.00245,Ult20008:0.04629)0.997.6:0.00131,((((((((UltLac18:
0.01501,Ult20012:0.00369)0.684.1:5.5E-4,Ult21357:0.00369)0.969.4:5.5E-
4,(Ult19967:0.00741,Ult19999:0.00369)0.938.1:5.5E-4)0.000.39:5.5E-
4,((((Ult19997:0.00369,((Ult19772:0.01857,Ult22668:0.05202)0.740.1:0.0041
8,(Ult19994:0.00363,(Ult19978:0.0074,Ult19992:0.00369)1.000.18:5.4E-
4)0.431.2:0.00369)1.000.19:5.5E-4)0.000.40:5.4E-
4,(((Ult20007:0.0074,(((Ult19172:0.0,Ult19194:0.0,Ult19806:0.0,Ult19867:0
.0,Ult19866:0.0,Ult19899:0.0,Ult19900:0.0,Ult19961:0.0,Ult19962:0.0,Ult19
964:0.0,Ult19965:0.0,Ult19966:0.0,Ult19968:0.0,Ult19969:0.0,Ult19970:0.0,
Ult19971:0.0,Ult19972:0.0,Ult19975:0.0,Ult19974:0.0,Ult19976:0.0,Ult19983
:0.0,Ult19981:0.0,Ult19985:0.0,Ult19986:0.0,Ult19989:0.0,Ult19991:0.0,Ult
19995:0.0,Ult20000:0.0,Ult20004:0.0,Ult20006:0.0,Ult20009:0.0,Ult20011:0.
0,Ult20010:0.0,Ult20013:0.0,Ult20015:0.0,Ult20014:0.0,Ult20016:0.0,Ult200
17:0.0,Ult20099:0.0,Ult20197:0.0,Ult20198:0.0,Ult20199:0.0,Ult20377:0.0,U
lt20515:0.0,Ult20983:0.0,Ult20976:0.0,Ult21398:0.0,Ult21435:0.0,Ult22373:
0.0,Ult24018:0.0):5.5E-4,(((Ult19998:0.015,(((Ult20227:5.4E-
4,Ult18277:0.02333)0.782.5:0.00375,(Ult20974:5.4E-
4,Ult18884:0.02742)0.694.1:0.00365)0.976.1:0.01896,(Ult19174:5.5E-
4,Ult19980:0.04602)0.817.1:0.00393)0.009.1:5.4E-4)0.441.1:5.5E-
4,(Ult20472:0.00198,Ult20684:5.5E-4)1.000.20:0.0017)0.097.1:5.5E-
4,(Ult19173:0.00368,Ult19996:0.02655)0.000.41:5.4E-4)0.466.1:5.5E-
4)0.000.42:5.5E-4,(Ult19901:0.00369,Ult19993:0.00369)0.000.43:5.5E-
4)0.202.2:5.4E-4)0.000.44:5.5E-4,Ult19963:0.00371)0.433.2:5.5E-
4,Ult19979:5.5E-4)0.422.1:5.3E-4)0.132.1:5.5E-
4,Ult19984:0.0037)0.466.2:5.5E-
4,((Ult20513:0.0074,(UltB6725:0.01498,Ult19768:5.5E-
4)0.427.1:0.00369)0.928.4:5.5E-4,Ult19988:0.00369)0.000.45:5.5E-
4)0.466.3:5.5E-4)0.000.46:5.5E-4,Ult19982:0.00368)0.393.1:5.5E-
4,(Ult19854:0.02325,Ult19781:0.02674)0.710.1:0.00366)0.216.1:5.5E-
4,Ult19977:5.5E-4)0.177.2:5.4E-4,BlutLut2:5.5E-4)0.257.1:5.5E-
4)0.393.2:5.5E-
4,(Ult19973:0.01943,Ult20473:0.02704)0.844.5:0.00694)0.844.6:0.00383,Ult2

1419:0.00369)0.230.1:5.5E-4)0.000.47:5.5E-
4,Ult19960:0.00369)0.431.3:5.5E-4,Ult19987:0.00746)0.000.48:5.3E-
4)0.889.5:0.00893,(Ult18269:0.03633,Ult20005:0.00957)0.761.4:0.00577)0.75
1.3:0.00289)0.789.3:0.00631,((Ult19537:0.00744,(Ult19198:0.00369,(Ult1953
8:0.0,Ult20477:0.0):5.5E-4)0.811.3:5.3E-
4)0.794.2:0.00379,(((((Ult18255:0.02505,(Ult19178:0.04299,(Ult19766:0.016
08,((Ult20149:0.0,Ult20150:0.0,Ult20151:0.0,Ult22678:0.0):5.4E-
4,(((BacNL263:5.5E-
4,BacNL267:0.00209)1.000.21:0.00155,(BacNL265:0.00367,(BacNL268:5.5E-
4,(BluProdu:0.0,Ult20143:0.0,BacNL264:0.0,BacNL266:0.0,BacNL269:0.0):5.5E
-4)0.212.1:5.5E-4)0.267.2:5.5E-
4)0.863.2:0.00364,(((Ult20810:0.00369,Ult20969:0.00741)0.717.2:5.5E-
4,(Ult20148:0.00368,(((Ult20002:0.00369,Ult20127:0.00369)0.901.5:5.5E-
4,((Ult19296:0.0,Ult20001:0.0,Ult20123:0.0,Ult20124:0.0,Ult20125:0.0,Ult2
0126:0.0,BluHanse:0.0,Ult20130:0.0,Ult20131:0.0,Ult20132:0.0,Ult20133:0.0
,BacNL271:0.0,Ult20144:0.0,Ult20146:0.0,Ult20145:0.0,Ult20147:0.0,Ult2018
7:0.0,Ult20196:0.0,Ult20704:0.0,Ult20703:0.0,Ult21416:0.0):5.5E-
4,(Ult20129:0.00369,Ult20972:0.0074)0.919.4:5.5E-4)0.000.49:5.5E-
4)0.000.50:5.5E-4,Ult20134:5.5E-4)0.724.2:5.5E-4)0.415.2:5.5E-
4)0.953.3:5.5E-4,(BacNL270:0.011,BacNL272:5.5E-
4)0.852.7:0.00362)0.432.2:5.4E-
4)0.922.2:0.00325)0.476.1:0.01062)0.224.1:0.00418)0.763.7:0.00428)0.819.2
:0.0061,(((Ult19511:0.01921,(Ult19524:0.03121,Ult20023:5.5E-
4)0.870.4:0.00752)0.781.2:0.00364,((Ult19533:0.0,Ult19534:0.0):0.00355,((
Ult20465:0.0074,(Ult20464:0.0,Ult20466:0.0):5.5E-
4)0.950.4:0.01889,(Ult20768:0.01161,(Ult20462:5.5E-
4,Ult20463:0.00744)0.760.3:0.00348)0.791.1:0.00915)0.939.2:0.01863)0.773.
3:0.01164)0.548.1:5.3E-
4,(((Ult19860:0.0037,((Ult20050:0.0,Ult20051:0.0):5.5E-
4,Ult20052:0.0074)0.491.1:5.4E-
4)0.936.2:0.0075,((Ult18411:0.0,Ult19852:0.0,Ult20020:0.0,Ult20981:0.0):5
.5E-4,Ult20019:0.0037)0.988.3:0.01538)0.144.1:5.5E-
4,((Ult20174:0.02268,((Ult19948:5.5E-
4,Ult20113:0.00742)0.990.2:0.02003,BluSchin:0.00324)0.464.1:0.00747)0.841
.1:5.5E-
4,(((Ult19769:0.0,Ult20021:0.0,Ult20107:0.0,Ult20121:0.0,Ult20137:0.0):5.
5E-4,Ult20106:0.0075)0.847.4:0.0037,(((Ult20072:5.5E-
4,Ult20073:0.00368)0.984.2:0.02336,((Ult19940:0.0,Ult19941:0.0,Ult19942:0
.0,Ult19943:0.0,Ult20982:0.0):0.00745,((Ult20109:0.00387,(UltB7207:0.0340
3,Ult18883:0.02581)0.755.1:0.00604)0.781.3:0.00348,(Ult19510:5.5E-
4,Ult20067:0.00743)0.920.5:0.00746)0.183.1:5.4E-
4)0.756.3:0.00397)0.295.1:0.00777,((Ult19947:5.5E-
4,Ult19946:0.01124)0.990.3:5.4E-4,(((UltRumi2:5.5E-
4,(((((Ult18053:0.0,Ult18294:0.0,Ult18206:0.0,Ult19179:0.0,Ult19691:0.0,U
lt19944:0.0,Ult19945:0.0,Ult19933:0.0,Ult19934:0.0,Ult19935:0.0,Ult19936:
0.0,Ult19950:0.0,Ult19951:0.0,Ult19953:0.0,Ult19958:0.0,Ult20018:0.0,Ult2
0105:0.0,Ult21430:0.0,Ult22364:0.0):5.5E-
4,(Ult19954:0.00368,(Ult18205:0.0,Ult19690:0.0):0.00369)0.626.1:5.5E-
4)0.000.51:5.5E-4,((Ult19861:0.0037,Ult20644:0.00742)0.859.4:5.5E-
4,(Ult18052:0.0037,(((Ult19959:0.0,Ult21375:0.0):5.5E-4,((Ult19689:5.5E-
4,(Ult21323:5.4E-
4,Ult21349:0.03091)0.803.2:0.00368)0.805.4:0.00369,Ult19853:0.0037)0.808.
4:0.0037)0.108.1:5.4E-4,Ult21421:0.00369)0.827.5:0.0037)0.731.2:5.5E-
4)0.000.52:5.5E-4)0.000.53:5.5E-4,Ult19937:5.5E-4)0.000.54:5.5E-

4,(((Ult19865:0.01118,Ult20160:5.5E-
4)0.816.2:0.00369,Ult20022:0.00742)0.719.1:5.5E-
4,(Ult20645:0.00369,Ult19956:0.01886)0.923.4:5.5E-4)0.000.55:5.5E-
4)0.735.1:5.4E-4)0.739.3:5.5E-4,Ult19952:5.5E-4)0.882.2:5.5E-
4,(Ult19938:5.4E-
4,(Ult19955:0.01375,Ult22767:0.02225)0.943.2:0.01807)0.844.7:0.00366)0.92
2.3:0.00744)0.835.4:0.01107)0.787.3:0.00407)0.700.4:5.5E-
4)0.742.3:0.00734)0.754.3:0.00358)0.961.3:0.01484)0.478.1:5.4E-
4,((Ult19496:0.01557,(Ult19499:0.00369,((Ult19497:0.0,Ult20743:0.0):5.5E-
4,Ult19498:0.00369)0.230.2:5.5E-
4)0.873.7:0.00766)0.854.2:0.00766,((Ult19163:5.5E-
4,((((UltLac15:0.0,Ult19543:0.0):5.5E-
4,(Ult19540:0.00362,Ult19544:0.00362)0.898.8:5.5E-4)0.759.4:5.5E-
4,(Ult19542:0.0036,Ult20193:0.01496)0.782.6:0.00369)0.683.1:5.4E-
4,Ult19541:0.02265)0.828.1:0.00368)0.030.1:5.4E-
4,Ult20559:0.00729)0.964.3:0.01543)0.872.7:0.00753)0.967.2:0.00137,((((Ul
t19512:0.01913,Ult19949:5.4E-
4)0.364.1:0.00744,(Ult20112:0.03469,(Ult19509:0.00374,UltRu358:0.0152)0.3
71.1:5.5E-
4)0.917.2:0.01101)0.782.7:0.00379,((Ult19520:0.00845,(Ult19931:0.00643,(U
lt19518:5.5E-
4,Ult19517:0.00362)0.773.4:0.00462)0.904.1:0.01523)0.911.2:0.01582,((((Ul
tBac23:5.5E-
4,((Ult20037:0.00745,((Ult20057:0.0,Ult20058:0.0,Ult20061:0.0,Ult20191:0.
0,Ult20192:0.0,Ult20984:0.0):5.5E-
4,(Ult20056:0.00369,Ult20060:0.00371)0.749.2:5.5E-4)0.424.1:5.5E-
4)0.991.5:5.4E-
4,(Ult20189:0.01515,Ult24021:0.01124)0.980.1:0.02306)0.831.4:0.00366)0.79
5.3:0.00372,Ult20122:0.00372)0.791.2:0.00603,((Ult20108:0.0,Ult20153:0.0)
:0.00744,(Ult19529:5.5E-
4,((Ult19526:0.00369,((Ult19528:0.0,Ult21335:0.0):5.5E-
4,Ult19527:0.00369)0.077.1:5.5E-
4)0.852.8:0.00371,Ult20677:0.00369)0.743.3:5.5E-4)0.893.3:5.3E-
4)0.774.5:0.00596)0.869.2:0.01265,Ult20650:0.01812)0.010.1:0.00732)0.843.
2:5.5E-4)0.558.1:5.4E-
4,((MvnForma:0.0188,((Ult19514:0.0,Ult19513:0.0,Ult22375:0.0):5.5E-
4,Ult20740:0.03505)0.962.4:0.01483)0.972.4:5.4E-
4,(Ult19508:0.006,Ult19539:0.01473)0.810.3:0.006)0.003.1:0.00749)0.963.5:
0.01626)0.872.8:0.00231,(UltFir17:0.03496,((Ult21356:0.03562,(Ult19495:0.
00974,Ult19523:0.02182)0.318.2:0.01439)0.736.2:0.00445,((UltRu297:0.00694
,UltRu298:0.02361)0.989.4:0.03169,((Ult17802:5.4E-
4,(Ult17801:0.00392,Ult25411:0.03925)0.984.3:0.02745)0.889.6:0.0075,((Ult
Eub36:0.00489,UltFir12:0.01048)0.997.7:0.04038,((((UltRu303:5.5E-
4,(Ult18374:0.00369,Ult18375:0.0037)0.819.3:5.5E-4)0.963.6:5.3E-
4,((((Ult18368:0.0103,((Ult18372:0.00368,(Ult18366:5.5E-4,UltRu304:5.5E-
4)0.950.5:5.4E-4)0.864.3:0.00725,(Ult18376:0.00376,((Ult18371:5.5E-
4,(Ult18369:0.00371,(Ult18377:5.5E-4,(Ult18379:0.00374,Ult18378:5.5E-
4)0.757.2:5.5E-4)0.862.1:5.5E-4)0.844.8:0.00372)0.770.5:5.5E-
4,(UltRu301:0.0154,Ult18367:0.00361)0.902.3:0.00763)0.891.3:0.0075)0.753.
2:0.00405)0.858.1:0.00986)0.935.4:0.01732,UltRu302:0.01767)0.737.3:0.0025
7,Ult18373:0.00387)0.964.4:0.01519,Ult18364:5.5E-
4)0.989.5:0.01524)0.882.3:0.01026,Ult18370:0.00544)0.914.4:0.01447,((Ult1
8385:0.011,(Ult18386:0.0114,Ult21043:0.01519)0.888.5:5.4E-
4)0.969.5:0.0274,((((Ult18365:0.01447,Ult18380:0.00541)0.880.4:0.01033,Ul

t18382:5.5E-4)0.825.1:0.00369,Ult18381:5.5E-
4)0.950.6:0.01129,UltRu305:5.4E-
4)0.649.2:0.00426)0.959.4:0.03262)0.725.3:0.00386)0.867.4:0.00784)0.447.1
:5.5E-
4)0.987.1:0.02304)0.087.1:0.00531)0.861.3:0.01033)0.760.4:0.00357)0.778.5
:0.00571)0.933.4:0.01634,(((Ult20062:0.00377,(Ult19519:0.01948,Ult19895:0
.00349)0.876.3:0.00775)0.858.2:0.00738,((((Ult19906:5.4E-
4,(Ult19522:0.0074,Ult20516:0.01541)0.887.4:0.0077)0.831.5:0.00368,(Ult20
053:0.01147,((Ult18408:0.00364,UltRu359:0.01514)0.757.3:0.00377,(((Ult198
14:0.00739,Ult21422:5.4E-
4)0.433.3:0.00369,((Ult19177:0.0,Ult19892:0.0,Ult19894:0.0,Ult20470:0.0):
5.5E-4,Ult19893:0.00369)1.000.22:5.5E-
4)0.739.4:0.00363,(Ult21426:0.01901,Ult20471:0.00369)1.000.23:5.4E-
4)0.741.4:0.00372)0.891.4:0.00764)0.769.5:0.00364)1.000.24:5.4E-
4,(Ult19925:5.4E-
4,(Ult19914:0.00359,(((Ult19905:0.01495,Ult19917:0.01476)0.792.4:5.4E-
4,(Ult19923:5.5E-4,(((Ult19472:0.00739,Ult20973:0.00367)0.934.4:5.5E-
4,(Ult19916:0.00367,Ult19915:0.00367)0.609.1:5.5E-4)0.000.56:5.5E-
4,((((Ult21294:0.00368,Ult19928:0.00367)0.667.2:5.5E-
4,(Ult19932:0.00367,Ult20077:0.00367)0.877.2:5.5E-4)0.000.57:5.5E-
4,(Ult18249:0.0,Ult19138:0.0,Ult19773:0.0,Ult19907:0.0,Ult19908:0.0,Ult19
909:0.0,Ult19910:0.0,Ult19912:0.0,Ult19913:0.0,Ult19918:0.0,Ult19919:0.0,
Ult19920:0.0,Ult19921:0.0,Ult19896:0.0,Ult19888:0.0,UltLac17:0.0,Ult19884
:0.0,Ult19890:0.0,Ult19898:0.0,Ult20135:0.0,Ult20224:0.0,Ult20682:0.0,Ult
20681:0.0,Ult20977:0.0,Ult22679:0.0):5.5E-4)0.000.58:5.5E-
4,(Ult19897:0.00367,Ult19885:0.00741)0.683.2:5.5E-4)0.000.59:5.5E-
4)0.735.2:5.5E-4)0.533.1:5.5E-4)0.726.1:5.5E-
4,((Ult11284:0.0792,Ult19911:0.01973)0.743.4:0.00245,(Ult19926:5.4E-
4,Ult20048:0.00752)0.833.5:0.00368)0.905.5:5.5E-4)0.837.3:5.5E-
4)0.871.6:0.00352)0.858.3:0.00353)0.855.3:0.00352,((Ult19515:5.5E-
4,((Ult19957:0.0111,((Ult20071:0.0,Ult21438:0.0):5.5E-
4,Ult24019:0.01503)0.890.4:5.5E-
4)0.778.6:0.00758,Ult19516:0.00775)0.866.4:0.00731)0.334.1:0.00727,(((Blu
Hydro:0.0,Ult20173:0.0):0.01633,((Ult19803:0.00357,Ult20172:0.05015)0.772
.3:0.00373,(((Ult19732:5.5E-4,Ult19848:0.0037)0.172.2:5.5E-
4,((CsrSpec8:0.01873,Ult19829:5.5E-4)0.832.3:0.00367,((Ult19855:5.5E-
4,((((Ult19682:0.00368,Ult19725:0.03886)0.153.1:5.5E-
4,(Ult21429:0.00368,((((Ult18358:0.00369,((Ult19696:0.00741,(((Ult19720:0
.00369,((Ult20169:0.00369,((Ult19721:5.5E-4,((((Ult19688:5.5E-
4,Ult19703:0.00369)0.860.2:0.00369,((((((Ult18426:0.0,Ult19849:0.0):0.00
356,(Ult21393:0.01492,Ult21298:0.00385)0.066.1:0.00368)1.000.25:5.5E-
4,Ult19801:0.0074)0.000.60:5.5E-
4,(((((Ult19809:0.00369,((((((Ult19782:0.00369,Ult19804:5.5E-
4)0.859.5:0.00369,(((Ult19139:0.0,Ult19836:0.0,Ult19837:0.0,Ult21399:0.0)
:5.5E-4,(((((((Ult20205:5.5E-
4,((((Ult21291:0.01894,(UltB7186:0.01132,Ult22673:0.00741)0.746.4:0.00377
)0.873.8:0.01025,(Ult22671:0.01992,Ult21392:0.05551)0.746.5:0.00703)0.576
.2:0.00568,UltB8607:0.01497)0.765.3:0.00772,(Ult21427:0.03963,(Ult19856:0
.01128,Ult21163:0.00747)0.755.2:0.0037)0.916.4:0.00741)1.000.26:5.5E-
4)1.000.27:5.5E-4,(Ult20204:0.00369,(Ult20206:5.5E-
4,(Ult18209:0.00741,Ult20155:0.00369)0.653.1:5.5E-4)0.655.1:5.0E-
4)0.877.3:0.00361)0.424.2:0.00375,Ult19850:0.0036)1.000.28:5.4E-
4,(Ult19787:5.5E-4,Ult21368:0.0112)0.836.1:0.00369)0.001.1:5.5E-
4,Ult19707:0.00368)0.426.1:5.1E-4,Ult19709:0.00369)0.176.1:5.5E-

4,Ult19716:0.00369)0.445.1:5.5E-4,((Ult19685:5.5E-
4,(Ult19819:0.01087,((Ult19820:0.0,Ult21036:0.0):5.5E-
4,Ult19939:0.00369)0.933.5:0.00199)0.930.1:0.00198)0.450.1:0.00369,((Ult1
9775:0.00591,(Ult19777:0.00364,Ult22031:0.00749)0.906.2:0.01044)0.775.1:0
.00585,(Ult18263:5.3E-
4,((Ult19812:0.00896,(Ult19759:0.01421,Ult25686:0.02241)0.672.1:0.01622)0
.766.3:0.00592,(Ult18285:0.0,Ult18288:0.0):0.0037)0.772.4:0.00369)0.911.3
:0.00716)0.916.5:5.5E-4)0.765.4:5.4E-4)0.000.61:5.5E-4)0.243.1:5.5E-
4,Ult21337:0.00369)0.062.1:5.4E-4)0.378.1:5.5E-
4,Ult19686:0.00369)0.000.62:5.5E-4,Ult19796:0.00368)0.000.63:5.5E-
4,Ult19876:0.01119)0.392.2:5.5E-4,Ult19833:0.00369)0.000.64:5.3E-
4)0.381.2:5.5E-4,Ult19731:0.00369)0.000.65:5.5E-
4,Ult19840:0.00369)0.000.66:5.5E-4,Ult19166:0.00369)0.442.1:5.5E-
4,Ult19704:0.00369)0.469.2:5.5E-4)0.034.1:5.5E-
4,((Ult19702:0.0764,Ult20162:0.00595)0.455.2:0.01346,Ult20159:0.00364)0.9
95.4:5.4E-4)0.000.67:5.4E-4,Ult19681:0.00369)0.293.1:5.5E-
4,(Ult19767:5.5E-4,Ult19789:0.00369)0.873.9:0.00369)0.000.68:5.5E-
4)0.385.1:5.5E-4,Ult19800:0.00369)0.426.2:5.5E-
4,Ult20200:0.00369)0.044.1:5.5E-4)0.000.69:5.5E-
4,Ult20209:0.00369)0.000.70:5.5E-4)0.221.2:5.5E-
4,Ult19699:0.00369)0.000.71:5.5E-4)0.378.2:5.5E-
4,Ult19838:0.00369)0.173.1:5.5E-
4,(Ult19729:0.00692,Ult19780:0.0021)0.904.2:0.00206)0.000.72:5.5E-
4)0.000.73:5.5E-
4,(Ult19811:0.00245,Ult20165:0.00245)0.929.2:0.00245)0.000.74:5.5E-
4)0.431.4:5.5E-4,Ult19786:0.00369)0.355.1:5.5E-4,Ult19693:5.5E-
4)0.000.75:5.5E-
4,(UltB7210:0.0,Ult18262:0.0,Ult18899:0.0,Ult19765:0.0,Ult19774:0.0,Ult19
779:0.0,Ult19783:0.0,Ult19687:0.0,Ult19680:0.0,Ult19683:0.0,Ult19684:0.0,
Ult19695:0.0,Ult19694:0.0,Ult19697:0.0,Ult19698:0.0,Ult19701:0.0,Ult19706
:0.0,Ult19708:0.0,Ult19711:0.0,Ult19714:0.0,Ult19715:0.0,Ult19718:0.0,Ult
19719:0.0,Ult19723:0.0,Ult19724:0.0,Ult19727:0.0,Ult19728:0.0,Ult19730:0.
0,Ult19788:0.0,Ult19793:0.0,Ult19795:0.0,Ult19797:0.0,Ult19799:0.0,Ult198
02:0.0,Ult19805:0.0,Ult19808:0.0,Ult19810:0.0,Ult19822:0.0,Ult19831:0.0,U
lt19830:0.0,Ult19832:0.0,Ult19834:0.0,Ult19835:0.0,Ult19839:0.0,Ult19841:
0.0,Ult19843:0.0,Ult19844:0.0,Ult19846:0.0,Ult19847:0.0,Ult19862:0.0,Ult1
9859:0.0,Ult19870:0.0,Ult19871:0.0,Ult19872:0.0,Ult19873:0.0,Ult19874:0.0
,Ult19875:0.0,Ult19877:0.0,Ult19878:0.0,Ult19879:0.0,Ult19880:0.0,Ult1988
1:0.0,Ult19882:0.0,Ult19883:0.0,Ult19904:0.0,Ult19903:0.0,Ult19902:0.0,Ul
t20157:0.0,Ult20158:0.0,Ult20154:0.0,Ult20156:0.0,Ult20161:0.0,Ult20164:0
.0,Ult20166:0.0,Ult20167:0.0,Ult20168:0.0,Ult20170:0.0,Ult20171:0.0,Ult20
208:0.0,Ult20207:0.0,Ult20686:0.0,Ult21290:0.0,Ult21317:0.0,Ult21332:0.0,
Ult21428:0.0,Ult21423:0.0):5.5E-4)0.406.1:5.5E-4)0.060.1:5.5E-
4)0.000.76:5.5E-4,(Ult19165:0.00369,Ult20100:5.5E-
4)0.848.4:0.0037)0.430.2:5.5E-4,((BlutLuti:5.5E-
4,((Ult19700:0.01529,Ult19824:0.00377)0.556.1:0.00764,Ult19845:5.4E-
4)1.000.29:5.9E-4)1.000.30:0.00297,Ult19717:0.00371)0.476.2:5.5E-
4)0.429.1:5.5E-4)0.147.1:5.5E-
4,((Ult19792:0.00369,((Ult19785:0.0,Ult19791:0.0,Ult24017:0.0):5.5E-
4,Ult19790:0.00369)0.064.1:5.5E-4)0.906.3:0.00361,(Ult19705:5.4E-
4,(Ult19798:5.5E-4,(Ult19710:5.5E-
4,Ult19794:0.07694)0.836.2:0.00363)0.906.4:0.00358)0.076.1:0.00366)1.000.
31:5.1E-4)0.134.1:5.5E-4)0.072.1:5.5E-4)0.248.1:5.5E-
4,((Ult19851:0.01124,(Ult19784:5.5E-4,((Ult19778:0.0,Ult19857:0.0):5.5E-

4,Ult19858:0.00369)0.752.5:5.5E-4)0.717.3:5.5E-
4)0.841.2:0.00367,(Ult19722:0.02249,Ult19842:0.00369)0.425.1:5.5E-
4)0.110.1:5.5E-4)0.582.1:5.5E-4)0.872.9:0.01003)0.138.1:5.4E-
4,Ult20627:0.02376)0.966.4:0.01655)0.774.6:0.00422)0.895.3:5.5E-
4)0.764.3:0.00456,((((((Ult20080:5.5E-4,Ult20081:5.5E-
4)0.897.4:0.01056,Ult20195:0.0106)0.777.4:0.00563,(((Ult20032:0.0,Ult2008
3:0.0,Ult20085:0.0,Ult20087:0.0,Ult20089:0.0,Ult20090:0.0,Ult20091:0.0,Ul
t20190:0.0):5.5E-4,(Ult20088:0.00743,Ult19770:0.00369)0.757.4:5.5E-
4)0.000.77:5.5E-4,((Ult20086:0.0037,Ult20082:0.00747)0.667.3:5.5E-
4,Ult18340:0.0037)0.740.2:5.5E-4)0.778.7:5.5E-
4)0.969.6:0.00127,(Ult20084:0.0,Ult21433:0.0):0.00259)0.942.1:0.01754,(((
Ult20065:0.00744,(Ult20064:5.5E-4,(Ult20066:5.5E-
4,(Ult18342:0.00369,Ult19930:0.00369)0.706.2:5.5E-4)0.740.3:5.5E-
4)0.936.3:5.4E-4)0.901.6:0.01107,(Ult20063:0.00341,(Ult20070:5.5E-
4,(((Ult20068:0.0,Ult20069:0.0):0.00328,Ult21437:0.01198)0.212.2:0.00392,
Ult19929:0.01887)0.822.2:0.0079)0.792.5:0.00769)0.782.8:0.00624)0.749.3:0
.00566,Ult19891:0.02744)0.192.1:0.0041)0.807.2:0.00382,(((((Ult20041:5.5E
-4,Ult21436:5.5E-4)0.886.3:5.4E-4,((Ult20059:5.5E-
4,(UltB7217:0.01117,Ult22672:0.01502)0.000.78:5.5E-
4)0.845.2:0.00369,((Ult20055:0.0,Ult20679:0.0):5.5E-
4,(Ult18248:0.02271,Ult20054:0.00369)0.954.3:5.5E-4)0.645.2:5.5E-
4)0.819.4:0.00369)0.827.6:5.4E-
4,(((Ult20039:0.0,Ult20044:0.0,Ult20046:0.0,Ult20049:0.0):5.5E-
4,(Ult20034:0.00369,Ult19692:0.00369)0.718.2:5.5E-4)0.941.2:5.5E-
4,((Ult18743:0.01898,Ult19195:5.3E-
4)0.802.1:0.00742,(Ult20047:0.00367,((((Ult20033:0.00369,(Ult20026:0.0036
9,Ult20029:0.00369)0.000.79:5.5E-4)0.000.80:5.5E-
4,(Ult22374:0.00742,((Ult20030:0.00369,Ult21439:0.0037)0.263.1:5.5E-
4,Ult20514:0.00369)0.000.81:5.5E-4)0.999.2:5.5E-4)0.000.82:5.5E-
4,(Ult19924:0.0,Ult20025:0.0,Ult20027:0.0,Ult20028:0.0,Ult20036:0.0,Ult20
040:0.0,Ult20043:0.0,Ult20045:0.0,BluSpeci:0.0,Ult20980:0.0,Ult20979:0.0)
:5.5E-4)0.000.83:5.5E-4,(Ult20038:0.00369,Ult20042:0.00369)0.926.8:5.5E-
4)0.865.5:5.5E-4)0.989.6:5.4E-
4)0.825.2:0.00366)0.864.4:0.00368)0.783.2:0.00386,((Ult19535:0.00747,(Ult
19525:0.02281,(Ult19532:0.01032,(Ult19531:0.01506,(Ult19536:0.0117,(Ult18
080:0.01155,Ult19530:0.00727)0.840.3:0.0076)0.727.2:0.00567)0.823.2:0.008
8)0.829.2:0.00963)0.965.2:6.7E-
4)0.929.3:0.00754,(((Ult20958:0.0,Ult21432:0.0):5.4E-
4,Ult20959:0.00369)0.990.4:0.02292,((UltLac20:5.5E-4,Ult20181:5.5E-
4)0.873.10:0.00746,(PshggY81:0.00743,UltLac19:5.4E-
4)0.890.5:0.0075)0.916.6:0.0112)0.354.1:5.5E-
4)0.774.7:0.00355)0.597.1:0.00759,((Ult19922:0.00744,Ult20035:5.5E-
4)0.915.8:0.00748,Ult19665:0.00745)0.872.10:5.4E-
4)0.938.2:0.01108)0.945.4:5.4E-
4)0.293.2:0.00592)0.799.2:0.00609,(((Ult20667:0.0,Ult20689:0.0,Ult20985:0
.0):5.5E-
4,(Ult18316:0.00738,((Ult20663:0.02263,(Ult23328:0.00555,(Ult18792:0.0477
1,Ult20672:0.00983)0.784.2:0.0046)0.916.7:0.01018)0.706.3:5.1E-
4,((Ult20668:0.00369,((Ult18302:0.0,Ult20003:0.0,Ult20031:0.0,Ult20078:0.
0,Ult20669:0.0,Ult20670:0.0,Ult20673:0.0,Ult20662:0.0,Ult20666:0.0,UltClo
47:0.0,Ult20697:0.0,Ult20707:0.0):5.5E-
4,((Ult20685:0.00369,Ult20664:5.5E-
4)0.838.3:0.00369,(Ult20671:0.06418,Ult20665:0.00367)0.961.4:5.5E-
4)0.483.1:5.5E-4)0.000.84:5.5E-4)0.000.85:5.5E-

4,(Ult20225:0.00747,Ult21334:0.00371)0.778.8:5.5E-4)0.436.1:5.5E-
4)0.829.3:5.5E-
4)0.872.11:0.00368)0.747.4:0.00351,(Ult19485:0.0076,(Ult19185:0.01928,(Ul
t20024:0.03072,(Ult19504:0.01925,(Ult19506:0.00362,((Ult19505:0.0,Ult1950
3:0.0):5.5E-4,(Ult19502:0.00362,Ult19507:0.00362)0.877.4:5.5E-
4)0.867.5:5.5E-
4)0.773.5:0.0034)0.908.4:0.0144)0.847.5:0.00934)0.853.2:0.00736)0.921.4:0
.01143)0.726.2:0.00375)0.768.4:0.00397)0.917.3:0.01279,((Ult20709:0.01934
,Ult19006:0.01935)0.824.4:0.00748,((Ult19286:5.5E-
4,(Ult18311:0.01504,(Ult20873:0.00369,Ult20874:5.5E-4)0.939.3:5.5E-
4)0.828.2:0.00367)0.989.7:0.0191,(Ult20120:5.4E-4,((Ult20118:5.5E-
4,Ult20119:0.00743)0.988.4:0.01528,(Ult20114:0.00369,((Ult20117:0.0,Ult20
115:0.0):5.5E-4,(Ult20116:0.00369,Ult21417:0.00369)0.901.7:5.5E-
4)0.663.2:5.5E-4)0.991.6:5.4E-
4)0.802.2:0.00749)0.895.4:0.00721)0.850.2:5.5E-
4)0.754.4:0.00373)0.902.4:0.00848)0.454.1:5.4E-
4,(Ult20092:0.01523,((Ult21105:5.5E-
4,((Ult21081:0.0,Ult21108:0.0,Ult21109:0.0,Ult21107:0.0,Ult21110:0.0):5.5
E-4,(Ult21104:0.00369,Ult21106:5.5E-
4)0.953.4:0.01494)0.970.3:0.0191)0.907.4:0.00747,(Ult20194:5.4E-
4,(((Ult20966:0.00369,((Ult21415:0.06996,Ult20967:0.0074)0.762.5:5.5E-
4,Ult20965:0.00368)0.357.1:5.5E-4)0.000.86:5.4E-
4,(Ult19664:0.0,Ult20962:0.0,Ult20963:0.0,Ult20964:0.0,Ult20968:0.0,Ult21
037:0.0,Ult22669:0.0):5.5E-4)0.186.2:5.5E-
4,Ult20229:0.00369)0.859.6:0.0037)0.867.6:0.00374)0.919.5:5.4E-
4)0.949.4:0.01127)0.943.3:0.01098)0.449.1:5.4E-
4,((((Ult19064:0.00379,((Ult19063:0.04052,(Ult19065:0.01935,((Ult19058:5.
3E-
4,((Ult19062:0.01229,Ult19061:0.02275)0.431.5:0.00737,((Ult19056:0.0,Ult1
9060:0.0):5.5E-4,Ult19057:5.5E-
4)0.896.3:0.01072)0.784.3:0.00431)0.933.6:0.00746,Ult19059:5.4E-
4)0.863.3:0.00757)0.826.3:0.00762)0.784.4:0.00381,(Ult19464:0.03323,Ult19
467:0.04352)0.912.1:0.01969)0.772.5:0.00405)0.958.2:0.01557,(((BacNL219:0
.00363,BacNL220:0.00743)0.809.4:0.00368,(UltClo30:0.00369,(Ult19588:5.5E-
4,Ult19589:5.5E-4)0.899.3:5.5E-4)0.938.3:5.5E-
4)0.983.2:0.01508,Ult19501:0.00748)0.092.1:5.5E-
4)0.968.2:0.01576,((((((UltB7206:0.0,Ult20360:0.0):0.0319,(((Ult20508:0.0
1089,Ult20509:5.4E-
4)0.892.3:0.02,((Ult21409:0.04864,((Ult20438:0.0,Ult20437:0.0):0.00329,(U
lt20235:5.5E-4,Ult20648:0.00368)0.985.4:0.01961)0.492.2:5.4E-
4)0.791.3:0.01255,Ult20439:0.04438)0.481.1:0.02685)0.800.3:0.01767,ShuSat
el:0.04128)0.576.3:0.00819)0.947.3:0.02207,(UltRu341:0.02061,(((Ult18510:
0.01053,Ult18509:0.02347)0.781.4:0.0052,(Ult18505:5.5E-
4,((Ult18503:0.0,Ult18504:0.0,Ult18506:0.0):5.5E-
4,(Ult18508:0.02,Ult18507:0.00369)0.441.2:5.5E-4)0.192.2:5.4E-
4)0.381.3:5.5E-4)0.830.3:0.00389,Ult19289:5.5E-
4)0.994.6:0.04784)0.902.5:0.02466)0.830.4:0.0249,((Ult19042:0.02757,Ult21
345:0.04596)0.824.5:0.01156,((UltRu351:0.01988,(Ult19031:0.00804,Ult12229
:0.07085)0.811.4:0.00748)0.798.6:0.00632,(SnsSucro:0.01954,((UdnRum29:0.0
0226,(UltRu345:0.04821,UltRu344:0.00776)0.773.6:0.0075)0.671.2:0.00158,(U
ltRu343:0.01099,(UltRu342:0.0,Ult22367:0.0):5.5E-
4)0.735.3:0.00371)0.933.7:0.01161)0.764.4:0.00505)0.666.2:0.00722)0.288.1
:5.4E-
4)0.931.2:0.02255,((((UltRu349:0.03455,(Ult19008:0.00369,(Ult19009:0.0,Ul

t19010:0.0):5.5E-
4)0.931.3:0.01901)0.905.6:0.01423,(Ult19011:0.0277,OrcSinus:0.01163)0.851
.2:0.0084)0.939.4:0.0241,(OrcSpeci:0.02676,Ult19012:0.01778)0.815.1:0.012
78)0.952.8:0.02311,Ult19067:0.03931)0.942.2:5.3E-
4)0.880.5:0.00893,((((Ult21127:0.00747,((Ult20711:0.0,Ult20712:0.0,Ult207
13:0.0,Ult20714:0.0):0.01504,Ult20698:0.01925)0.362.1:5.4E-
4)0.777.5:0.0039,(((((Ult21130:0.0,Ult21131:0.0):5.3E-
4,(Ult20911:0.02698,Ult21369:0.00225)0.960.4:0.00162)0.705.1:5.5E-
4,(Ult20910:5.4E-4,(((Ult20912:5.5E-
4,(Ult20903:0.00993,Ult20896:0.07755)0.770.6:0.0051)0.890.6:0.01319,((Ult
20897:0.01534,((Ult20901:0.01119,(Ult20637:0.03499,((Ult20898:0.01117,Ult
20900:0.00742)0.842.5:5.5E-4,Ult20899:5.5E-4)0.988.5:5.5E-
4)0.855.4:0.00365)1.000.32:5.4E-4,(Ult20895:0.0,Ult20904:0.0):5.5E-
4)0.962.5:5.5E-
4)0.777.6:0.0072,(UltB7249:0.0,Ult20894:0.0,Ult20902:0.0):5.4E-
4)0.799.3:0.00732)0.944.2:0.01751,(Ult20908:0.00367,((Ult20905:0.0,Ult209
07:0.0):5.5E-4,(Ult20909:0.00368,Ult20906:0.00367)0.744.4:5.5E-
4)0.736.3:5.5E-4)0.888.6:5.4E-
4)0.967.3:0.01492)0.952.9:0.01491)0.640.1:0.00712,((((Ult21050:0.0,CsrGly
cy:0.0,Ult21051:0.0):5.5E-4,Ult21052:0.01122)0.959.5:5.4E-
4,(Ult20886:0.015,(Ult20998:5.5E-4,Ult20997:0.00741)0.951.4:5.4E-
4)0.993.1:0.02733)0.875.6:0.00751,(Ult20961:0.051,(RmcSpeci:5.5E-
4,Ult20960:0.00373)0.993.2:5.4E-
4)0.758.5:0.00368)0.958.3:0.01505)0.642.1:5.4E-
4,(((Ult20705:0.0212,(UltRu313:5.5E-
4,(Ult18416:0.01122,(UltRu311:0.01143,((UltRu147:0.0,Ult18415:0.0):5.5E-
4,UltRu312:0.0037)0.835.5:5.5E-
4)0.893.4:0.01148)0.944.3:0.01529)0.937.4:0.01771)0.511.1:0.00743,UltFir1
6:0.00524)0.781.5:0.00765,UltRu314:0.01859)0.945.5:0.01855)0.799.4:0.0037
3)0.925.7:0.00371,UltRu316:0.02667)0.839.4:0.00422,((((((((Ult21137:0.011
17,((Ult21134:0.00369,((Ult18286:0.0,Ult20275:0.0,Ult20785:0.0):5.5E-
4,(Ult21136:0.0074,UltLac30:0.01946)0.842.6:5.5E-4)0.083.1:5.4E-
4)0.938.4:5.4E-4,(Ult21135:5.4E-
4,UltClo51:0.02343)0.790.1:0.00368)0.890.7:0.00785)0.964.5:0.02137,(((Ult
Clo18:0.06226,Ult21391:0.01791)0.923.5:0.03226,(((Ult20642:0.00756,((Ult2
1211:0.01229,(((LccBovis:0.0,UltRu365:0.0):0.01755,(UltClos8:0.03121,((Ul
t21367:0.01851,Ult22387:0.02255)0.782.9:0.0049,(Ult20486:0.0151,(((Ult183
47:0.00369,Ult21325:0.01907)0.238.1:5.5E-
4,(Ult18346:0.0,Ult20487:0.0,Ult20485:0.0,Ult20489:0.0,Ult20490:0.0,Ult21
324:0.0,Ult22496:0.0):5.5E-4)0.476.3:5.5E-
4,Ult20488:0.00369)0.975.1:5.4E-
4)0.756.4:0.00369)0.946.3:0.01798)0.749.4:0.00632)0.299.1:0.00897,((Ult20
511:0.10023,Otu00537:0.027)0.922.4:0.02573,(UltRu366:0.03477,((UltClo17:0
.09535,((Ult18215:0.01872,(Ult18216:0.0074,Ult18224:0.00369)0.385.2:5.5E-
4)0.881.5:0.01475,(Ult18217:0.00369,((Ult18220:0.0074,Ult18218:0.03836)0.
586.2:5.5E-
4,((UltB7245:0.0,Ult18219:0.0,Ult18221:0.0,Ult18222:0.0,Ult18225:0.0):5.5
E-4,Ult18223:0.00369)0.752.6:5.5E-4)0.582.2:5.4E-
4)0.769.6:0.00813)0.947.4:0.04025)0.234.1:0.0246,(Ult20373:0.04152,Ult203
74:0.04117)0.754.5:0.01323)0.901.8:0.03192)0.381.4:5.5E-
4)0.948.3:0.02417)0.874.3:0.01998)0.976.2:0.0348,Ult21212:0.01468)0.954.4
:5.5E-
4)0.940.2:0.01215,Ult18350:0.01505)0.711.1:0.00145,Ult21263:0.04362)0.716
.2:0.01269)0.756.5:0.0117,((Ult18732:0.0,UltClo15:0.0):0.0188,(UltClo14:0

.00365,(Ult18731:0.00369,UltClo16:5.5E-
4)0.779.1:0.00371)0.864.5:0.00947)0.864.6:0.01235)0.780.3:0.01021)0.868.6
:0.01346,(Ult19864:0.01181,Ult21295:0.00786)0.440.2:0.00954)0.911.4:0.018
54,((((Ult21261:0.01504,(((Ult20222:0.00369,Ult20579:0.00369)0.671.3:5.5E
-4,(Ult20577:0.00369,Ult20575:0.00369)0.898.9:5.5E-4)0.000.87:5.5E-
4,((Ult20223:0.0,Ult20576:0.0):5.5E-4,Ult20578:5.5E-4)0.000.88:5.5E-
4)0.583.1:5.5E-4)0.954.5:5.5E-4,(Ult20274:5.3E-
4,(Ult20375:0.00753,((Ult20838:0.01371,(Ult18185:0.00754,((Ult18184:0.003
72,Ult18183:0.00374)0.880.6:0.00721,(((Ult18174:5.5E-
4,(((((Ult18176:0.02299,Ult18177:0.022)0.886.4:0.00973,Ult18178:0.0596)0.
797.4:5.5E-4,Ult18181:0.00745)0.176.2:5.5E-
4,Ult18175:0.01494)0.388.1:5.5E-4,Ult18179:5.5E-4)0.396.2:5.5E-
4)0.834.2:0.00389,Ult18182:0.00753)0.290.2:5.4E-4,Ult18180:5.5E-
4)0.930.2:0.01194)0.917.4:5.5E-
4)0.873.11:0.00979)0.507.1:0.00812,(Ult20499:0.0685,(((Ult20498:0.0,Ult20
503:0.0,Ult20505:0.0):5.5E-4,(Ult20500:0.00369,((Ult18545:5.5E-
4,Ult18604:0.01124)0.824.6:0.00368,Ult20502:0.0074)0.466.4:5.5E-
4)0.000.89:5.5E-4)0.926.9:5.4E-4,((Ult17670:0.00369,Ult20501:5.5E-
4)0.994.7:5.4E-4,Ult20504:0.0188)0.836.3:0.00365)0.869.3:5.4E-
4)0.822.3:0.00592)0.774.8:0.00519)0.895.5:0.00755)0.329.1:0.00746)0.893.5
:0.00748,(((Ult19297:0.00372,((Ult18201:0.0,Ult18202:0.0,Ult18203:0.0):5.
5E-4,(Ult18204:0.01499,(Ult20970:0.0,Ult21206:0.0):0.00369)0.630.2:5.4E-
4)0.799.5:0.00369)0.876.4:0.00755,((Ult18733:0.00843,Ult20661:0.01334)0.8
33.6:0.00865,Ult21210:0.03978)0.755.3:0.00379)0.936.4:0.01119,((((((Ult20
261:5.3E-4,(Ult20388:0.00369,Ult20387:5.5E-
4)0.815.2:0.00367)0.717.4:0.00368,(Ult20535:0.01483,((Ult20272:0.00369,((
(Ult20273:0.00369,(((Ult20313:0.00369,(((((Ult18747:0.00486,(Ult20521:0.0
1964,Ult20297:0.02126)0.399.1:0.00914)0.898.10:0.01013,Ult20239:5.5E-
4)0.838.4:0.00369,(Ult20296:0.01125,((((((((Ult20560:0.00364,Ult20236:0.0
1527)0.847.6:0.00703,(Ult20248:0.02714,(Ult20243:0.0686,(Ult20263:0.0,Ult
20279:0.0):5.4E-4)0.698.2:0.00187)0.087.2:5.1E-
4)0.841.3:0.00206,(Ult18974:0.02351,((Ult20314:0.0,Ult20315:0.0):5.5E-
4,Ult20839:0.00746)0.877.5:0.00767)0.829.4:0.01137)1.000.33:5.5E-
4,Ult22372:0.00743)0.371.2:5.5E-
4,(Ult20219:0.00369,Ult20309:0.00369)0.798.7:0.00368)0.294.1:5.5E-
4,Ult20242:0.00369)0.000.90:5.5E-4,Ult21316:0.00369)0.000.91:5.5E-
4,UltClo41:0.00368)0.041.1:5.5E-4)0.467.1:5.5E-4)0.000.92:5.5E-
4,(Ult20277:0.00245,(Ult20237:0.00209,Ult20270:0.00691)0.009.2:8.4E-
4)0.919.6:0.00245)0.000.93:5.5E-4,Ult20288:0.0074)0.448.1:5.5E-
4)0.344.2:5.5E-4,(Ult20258:5.5E-
4,Ult21306:0.00197)1.000.34:0.00171)0.068.2:5.5E-
4,Ult20290:0.00369)0.474.2:5.5E-4)0.000.94:5.5E-
4,(Ult18251:0.0,Ult18748:0.0,Ult19176:0.0,Ult19815:0.0,Ult19887:0.0,Ult20
213:0.0,Ult20233:0.0,Ult20241:0.0,Ult20244:0.0,Ult20245:0.0,Ult20246:0.0,
Ult20247:0.0,Ult20249:0.0,Ult20250:0.0,Ult20251:0.0,Ult20252:0.0,Ult20253
:0.0,Ult20254:0.0,Ult20255:0.0,Ult20256:0.0,Ult20257:0.0,Ult20259:0.0,Ult
20260:0.0,Ult20262:0.0,Ult20264:0.0,Ult20269:0.0,Ult20276:0.0,Ult20278:0.
0,Ult20280:0.0,Ult20286:0.0,Ult20284:0.0,Ult20285:0.0,Ult20287:0.0,Ult202
89:0.0,Ult20291:0.0,Ult20294:0.0,Ult20312:0.0,Ult20316:0.0,Ult20318:0.0,U
lt20323:0.0,Ult20324:0.0,Ult20325:0.0,Ult20326:0.0,Ult20328:0.0,Ult20327:
0.0,Ult20382:0.0,Ult20381:0.0,Ult20383:0.0,Ult20378:0.0,Ult20379:0.0,Ult2
0519:0.0,Ult21312:0.0,Ult21315:0.0,Ult21400:0.0,Ult22596:0.0):5.5E-
4)0.318.3:5.5E-4,Ult21313:0.00369)0.389.1:5.5E-4)0.227.1:5.5E-
4,(Ult20232:5.5E-4,Ult20240:0.0074)0.862.2:0.00368)0.549.1:5.5E-

4)0.298.1:5.5E-4)0.810.4:5.3E-
4,(((Ult20548:0.00374,(Ult20520:0.0224,((((((Ult20552:0.00742,(((Ult19863
:0.00765,Ult20546:5.4E-4)0.917.5:0.00765,Ult19813:0.00369)0.471.2:5.4E-
4,Ult20543:0.00369)0.000.95:5.5E-4)0.286.1:5.5E-
4,BtvFib10:0.0074)0.637.1:5.5E-
4,(Ult20308:0.0,Ult20517:0.0,Ult20540:0.0,Ult20541:0.0,Ult20544:0.0,Ult20
545:0.0,UltClo44:0.0,Ult20547:0.0,Ult20551:0.0,Ult20553:0.0,Ult20554:0.0,
Ult20569:0.0,Ult20837:0.0,Ult21310:0.0):5.5E-4)0.587.2:5.5E-
4,(UltLac22:0.0074,((Ult20292:0.0074,Ult20557:0.00369)0.918.5:5.5E-
4,((Ult20527:0.0,Ult20528:0.0,Ult20529:0.0,Ult20836:0.0):5.5E-
4,(Ult20384:0.00369,Ult20556:0.00369)0.659.1:5.5E-4)0.000.96:5.5E-
4)0.944.4:5.5E-4)0.833.7:0.00368)0.420.1:5.5E-
4,(((Ult20307:0.00745,UltLac21:5.5E-4)0.921.5:0.00242,((Ult20538:5.5E-
4,(Ult20523:0.0037,((Ult20571:0.00369,(UltClo45:0.00369,UltClo46:5.5E-
4)0.113.1:5.5E-
4)0.874.4:0.00763,Ult20572:0.01151)0.784.5:0.00363)0.783.3:5.4E-
4)1.000.35:5.5E-
4,(((Ult20281:0.00377,Ult20539:0.04802)0.787.4:0.00364,UltRu361:0.0037)1.
000.36:5.4E-
4,(Ult20563:0.00369,(((Ult21311:0.00369,Ult20295:0.01127)0.641.1:5.5E-
4,(UltLac23:0.00369,(Ult20220:0.00746,Ult21361:0.00743)0.756.6:5.5E-
4)0.931.4:5.5E-4)0.000.97:5.5E-
4,(Ult20282:0.0,Ult20306:0.0,Ult20562:0.0):5.5E-4)0.727.3:5.5E-
4)0.814.1:0.00366)0.224.2:0.0037)0.921.6:0.00241)0.921.7:0.00247,Ult20549
:0.03103)0.564.1:5.5E-4)0.519.1:5.5E-
4,(Ult20550:0.00213,Ult20555:0.02214)0.982.1:0.00168)0.863.4:5.3E-
4)0.826.4:0.00367)0.780.4:0.00369,(Ult20558:0.0,Ult20561:0.0):5.3E-
4)0.850.3:0.00368,(((((Ult18296:0.00369,Ult20395:0.00369)0.903.2:5.5E-
4,(UltB7189:0.0,Ult20319:0.0,Ult20390:0.0,Ult20391:0.0,Ult20392:0.0,Ult20
393:0.0,Ult20394:0.0,Ult20385:0.0,BtPBact3:0.0,Ult20396:0.0,Ult20397:0.0,
Ult20398:0.0,Ult20525:0.0,Ult20526:0.0,Ult20565:0.0,Ult20840:0.0,Ult21401
:0.0):5.5E-4)0.000.98:5.5E-4,((Ult20999:0.01123,((Ult20320:5.5E-
4,((Ult20522:5.5E-
4,Ult21319:0.05502)0.916.8:0.00743,Ult20566:0.00369)0.773.7:5.4E-
4)0.708.1:5.5E-4,(((((Ult20329:0.00369,Ult20564:0.00369)0.933.8:5.5E-
4,(Ult19206:0.00369,Ult20533:0.00369)0.694.2:5.5E-4)0.000.99:5.5E-
4,Ult20311:0.00369)0.000.100:5.5E-4,Ult20518:5.5E-4)0.690.2:5.5E-
4,(Ult20293:5.4E-4,(((Ult20531:5.3E-4,(UltB7216:0.02312,(Ult20567:5.4E-
4,Ult22684:0.0036)0.883.2:0.01526)0.739.5:0.00372)0.963.7:0.01517,((Ult21
403:0.04724,Ult23329:0.00543)0.821.2:0.00851,Ult22495:0.01446)0.761.5:0.0
0578)0.760.5:0.00361,((Ult20568:0.00745,Ult20570:0.00378)0.952.10:0.01523
,((Ult20271:0.03008,Ult24025:0.00437)0.367.2:0.00743,(Ult20532:0.01132,(U
lt21153:0.0,Ult21448:0.0):0.00744)0.764.5:0.00436)0.802.3:0.00315)0.798.8
:0.00373)0.792.6:0.00377)0.853.3:0.00368)0.836.4:0.00369)0.867.7:0.00369)
0.738.2:5.5E-4,Ult21314:5.4E-4)0.934.5:0.00368)0.000.101:5.5E-
4,((UltRoseb:0.01118,Ult20574:0.00369)0.424.3:5.5E-
4,Ult20354:0.00369)0.000.102:5.5E-4)0.907.5:5.3E-
4,(Ult20386:0.00369,Ult20266:0.0227)0.931.5:5.5E-4)0.402.2:5.5E-
4)0.825.3:0.00356)0.772.6:0.00722,(Ult20380:5.4E-
4,(((Ult20268:0.02007,(Ult20310:0.02913,Ult21425:0.01207)0.686.2:0.0023)0
.791.4:0.00469,(((((Ult20211:5.5E-
4,Ult21164:0.02286)0.901.9:0.01163,(Ult21040:0.00411,Ult22392:0.03917)0.9
03.3:0.01152)0.061.1:0.00143,Ult19175:0.01475)0.927.2:0.00249,Ult20212:5.
4E-

4)0.788.3:0.00377,(Ult19816:0.0192,Ult21177:0.00377)0.922.5:0.01134)0.787
.5:0.00445)0.765.5:0.00324,((Ult20626:0.0075,Ult21884:0.0037)0.457.1:5.4E
-
4,(Ult18261:0.00196,Ult18250:0.01861)0.978.2:0.00191)0.862.3:0.00369)0.38
3.2:5.5E-4)1.000.37:5.3E-4)0.886.5:0.0036,Ult20389:5.4E-
4)0.781.6:0.00378,LchBact3:0.01125)0.559.1:0.00371)0.893.6:5.3E-
4)0.891.5:0.00759,(((UltLac11:0.02309,(Ult18171:5.5E-
4,(Ult20887:0.02272,(Ult18190:0.03442,((Ult18172:0.0,Ult18173:0.0,Ult1818
9:0.0):5.5E-4,Ult18188:0.00741)0.811.5:0.00373)0.862.4:5.3E-
4)0.949.5:0.0146)0.840.4:0.01456)0.987.2:5.4E-
4,((UltB6920:0.00369,Ult20583:5.5E-4)0.785.3:0.00382,(Ult20582:5.4E-
4,Ult20581:0.02322)0.898.11:0.01492)0.713.2:0.00755)0.756.7:0.01098,((Ult
20497:0.00766,((Ult20492:0.01876,((Ult20494:0.0,Ult20495:0.0,Ult20496:0.0
):5.5E-4,UltCl200:0.00369)0.955.6:5.5E-
4)0.803.3:0.00359,Ult20491:0.00385)0.773.8:0.00372)0.783.4:0.00367,Ult206
56:0.00758)0.749.5:0.00362)0.597.2:5.4E-
4)0.865.6:0.01303)0.565.1:0.00615,(Ult18301:0.04649,(Ult18338:0.0574,Ult1
8343:0.02481)0.816.3:0.02004)0.153.2:0.01056)0.437.1:5.4E-
4,Ult21472:0.02684)0.832.4:5.4E-
4,((((((Ult18736:0.0,Ult18737:0.0):0.0201,Ult20305:0.01138)0.541.1:0.0074
8,(((Ult18194:0.00369,(Ult18195:0.0,Ult18196:0.0):5.5E-4)0.906.5:5.5E-
4,((Ult20461:0.01888,(Ult20460:0.00369,Ult20482:5.5E-4)1.000.38:5.5E-
4)0.951.5:0.01445,((((((Ult20367:0.00369,(Ult20368:0.0,Ult20369:0.0,Ult2
0370:0.0,Ult20372:0.0):5.5E-
4)0.771.4:0.00366,(Ult19823:0.00369,Ult19868:5.5E-
4)0.943.4:0.01141)0.760.6:0.00378,((Ult18407:0.07206,((Ult20506:0.0,Ult22
395:0.0):5.5E-4,Ult20507:5.5E-
4)0.881.6:0.01175)0.782.10:0.00732,((CsrSpec9:0.01228,Ult20362:0.02332)0.
904.3:0.01596,Ult19886:0.01963)0.739.6:0.00391)0.855.5:0.0076)0.898.12:5.
5E-4,(Ult19295:0.00749,((Ult20366:0.0,Ult20355:0.0,Ult20358:0.0):5.5E-
4,(Ult20357:0.00369,Ult20356:0.00741)0.637.2:5.5E-4)0.969.7:5.5E-
4)0.043.1:0.00717)0.149.1:0.00707,(((Ult20302:5.5E-
4,Ult20303:0.00369)0.788.4:0.00373,(Ult20300:0.03163,(Ult20301:5.5E-
4,(((((BtvFibr6:0.0,RmnBact3:0.0,UltRu362:0.0,UltRu363:0.0,UltRu364:0.0,U
dnRum30:0.0):5.5E-4,(PbvXylan:5.5E-4,UltPse48:5.5E-4)0.000.103:5.5E-
4)0.613.1:5.5E-4,(Ult20304:0.01514,(BtvFibr7:5.5E-
4,BtvFibr9:0.01117)0.966.5:0.01123)0.651.3:5.3E-4)0.879.3:5.4E-
4,(Ult20655:0.03633,UltRu367:0.01539)0.880.7:0.01145)0.793.4:0.00376,(Btv
Fibr8:0.0,PbvRumin:0.0):0.0075)0.791.5:0.00376)0.844.9:5.5E-
4)0.912.2:0.00763)0.911.5:0.00761,(Ult20298:0.00369,Ult20299:5.5E-
4)0.904.4:5.4E-
4)0.949.6:0.01598)0.743.5:0.01065,((Ult20706:0.0467,Ult20715:0.01116)0.45
2.2:0.00758,(((Ult21000:0.0177,(Ult18234:5.5E-
4,(Ult18232:0.00739,Ult18233:0.00774)0.987.3:0.02357)0.996.1:0.04546)0.96
9.8:0.02669,Ult20542:5.4E-
4)0.828.3:0.00935,(Ult20467:0.00741,(Ult20468:0.0,Ult20469:0.0):5.5E-
4)0.949.7:0.01833)0.915.9:0.01989)0.420.2:0.01488)0.137.1:0.00823,((Ult20
365:0.0,Ult20364:0.0):5.3E-
4,Ult20363:0.00744)0.957.2:0.01847)0.239.1:0.00425)0.824.7:0.00451)0.840.
5:0.00382,(Ult18208:0.01128,(Ult18197:0.00368,(Ult18198:5.5E-
4,Ult21305:0.00742)0.703.3:5.5E-
4)0.886.6:0.00752)0.797.5:0.00366)0.876.5:5.4E-
4)0.906.6:0.01188,Ult18192:0.0183)0.139.2:5.4E-
4,((Ult20359:0.01826,Ult20510:0.01799)0.890.8:0.01333,Ult18193:0.00449)0.

495.1:0.00372)0.931.6:0.01218,(((Ult20653:0.0,Ult22821:0.0):0.01487,((Ult
20580:0.01038,(Ult26845:5.5E-4,(Ult20361:0.01885,(((Ult20524:5.5E-
4,((Ult20347:0.00369,(Ult20376:0.01912,(Ult20349:0.00741,Ult20335:0.00369
)0.000.104:5.5E-4)0.000.105:5.5E-4)0.000.106:5.5E-
4,(Ult20350:0.00369,(Ult20332:5.5E-
4,(Ult20321:0.0,Ult20330:0.0,Ult20331:0.0,Ult20333:0.0,Ult20334:0.0,Ult20
336:0.0,Ult20337:0.0,Ult20338:0.0,Ult20339:0.0,Ult20340:0.0,Ult20341:0.0,
RoeInuli:0.0,Ult20342:0.0,Ult20343:0.0,Ult20344:0.0,Ult20346:0.0,Ult20351
:0.0,Ult20352:0.0,Ult20353:0.0,Ult20530:0.0,Ult20573:0.0):5.5E-
4)0.000.107:5.5E-4)0.000.108:5.5E-4)0.748.3:5.5E-4)0.415.3:5.4E-
4,Ult20348:5.5E-4)0.840.6:5.5E-4,Ult20345:0.03777)0.914.5:5.4E-
4)0.540.1:0.00695)0.879.4:0.01301)0.797.6:0.0156,(Ult20654:0.0038,(Ult188
85:0.05343,UdnRum31:0.00361)0.964.6:0.02402)0.677.1:0.00907)0.851.3:0.024
91)0.893.7:5.5E-
4,Ult20741:0.02779)0.855.6:0.0072)0.848.5:0.00745)0.937.5:0.01082,(((((((
(Ult20178:0.01519,Ult20179:5.5E-
4)0.948.4:0.01514,((Ult20176:0.00369,Ult20177:5.4E-
4)0.948.5:0.02284,(Ult21123:0.00898,(UltLac28:5.5E-
4,UltLac29:0.01491)0.924.7:0.01876)0.981.1:0.03197)0.641.2:5.4E-
4)0.928.5:0.01556,(((((Ult20180:0.01807,(Cuiiii22:0.02739,Ult20175:0.0353
1)0.900.4:0.02562)0.545.2:0.01376,Ult21133:0.01298)0.792.7:0.01077,UltEub
42:5.5E-
4)0.798.9:0.00369,Ult21125:0.00371)0.815.3:0.00371,UltRu315:0.02752)1.000
.39:5.4E-
4)0.811.6:0.00379,(((Ult21128:0.00151,Ult21129:0.01897)0.974.2:0.00167,((
((Ult20885:0.077,(CsrHylem:0.01504,CstBact4:5.5E-
4)0.784.6:0.0037)0.769.7:0.00386,((UltClo38:0.02484,((UltRu368:0.01051,Ul
t20478:0.02453)0.953.5:0.02659,Ult18324:0.01894)0.720.3:0.01241)0.785.4:0
.01787,(Ult20956:5.5E-
4,Ult20957:0.01527)0.889.7:0.01447)0.749.6:0.00477)0.979.3:9.9E-
4,(((((HesPorci:0.0041,HesSterc:0.02711)0.837.4:0.00778,((Ult21122:0.0210
6,(Ult21121:0.01509,UltLac27:0.00884)0.835.6:0.01241)0.987.4:0.03739,(Ult
19291:0.00747,(Ult21141:0.02704,(Ult21142:0.0,Ult21143:0.0):5.4E-
4)0.921.8:0.00751)0.238.2:5.4E-
4)0.824.8:0.01255)0.938.5:0.01577,Ult21103:5.4E-
4)0.882.4:0.00798,(Ult20880:0.0,Ult20881:0.0,Ult20882:0.0,Ult20883:0.0):0
.01142)0.873.12:0.00796,(Ult20884:0.0,Ult20893:0.0):5.4E-
4)0.918.6:0.00286)0.913.4:0.01439,(Ult21111:0.00894,(Ult17803:0.00744,Ult
26392:5.5E-
4)0.910.9:0.0141)0.830.5:0.00788)0.872.12:0.01036)0.736.4:0.0042,Ult21132
:0.01669)0.742.4:0.00304)0.871.7:0.00803,((Ult20870:0.0,Ult20869:0.0):0.0
2294,(BacNL281:5.5E-4,(BacNL280:5.5E-4,((BacNL279:0.0,BacNL282:0.0):5.5E-
4,Ult21124:0.02021)0.197.1:5.5E-4)0.068.3:5.5E-
4)0.798.10:0.00648)0.771.5:0.00612)0.872.13:0.0071,(((((CsdBacte:0.0,LcfL
ongo:0.0):0.00369,BacNL273:5.5E-
4)0.903.4:0.00759,((Ult20138:0.00371,(Ult20188:0.00372,(Ult20877:0.01853,
(((Ult20136:0.0,Ult20678:0.0,Ult21119:0.0):5.5E-
4,((Ult20876:0.00369,Ult20875:0.00746)0.086.1:5.5E-
4,Ult20878:0.00369)0.890.9:5.5E-4)0.895.6:5.5E-
4,Ult21120:0.00744)0.918.7:5.4E-
4)0.766.4:0.00364)0.886.7:0.00743)0.883.3:5.3E-
4,((Ult21413:0.00371,((Ult21056:0.00368,(Ult21044:5.5E-
4,(((((Ult21095:0.01518,(Ult21096:5.5E-
4,(Ult21094:0.00369,Ult21101:0.0037)0.764.6:0.0037)0.945.6:5.4E-

4)0.900.5:0.00745,(Ult21058:0.01542,(Ult21068:5.5E-
4,((Ult19825:0.0,Ult21063:0.0,Ult21064:0.0,Ult21065:0.0,Ult21066:0.0,Ult2
1067:0.0,Ult21070:0.0,Ult21162:0.0):5.5E-
4,(Ult21071:0.00369,Ult21069:0.00369)0.893.8:5.5E-4)0.739.7:5.5E-
4)0.861.4:0.0076)0.842.7:0.00747)0.294.2:5.4E-4,Ult21057:5.5E-
4)0.853.4:0.0037,((Ult21055:0.0,Ult21061:0.0):5.5E-
4,(Ult21062:0.0037,Ult21060:0.00369)0.743.6:5.5E-4)0.849.2:5.3E-
4)0.916.9:0.00744,(UltLac25:0.00744,UltLac26:0.00372)0.887.5:0.00744)0.95
6.1:5.4E-4)0.725.4:5.5E-4)0.915.10:5.4E-
4,Ult21059:0.02658)0.864.7:0.0071)0.773.9:0.00364,((((Ult20879:0.01074,Ul
t21053:0.04839)0.929.4:0.02114,((Ult18101:0.0,Ult20992:0.0):0.0268,((Ult2
1099:0.01279,(Ult21100:0.01156,Ult21054:0.00763)0.835.7:0.00885)0.764.7:0
.0079,(Ult17806:0.02459,Ult20221:0.0157)0.733.2:0.00351)0.482.1:0.00408)0
.143.1:0.00424)0.756.8:5.4E-4,Ult21102:0.0037)0.584.2:5.4E-
4,(Ult21097:0.0,Ult21098:0.0):5.1E-4)0.844.10:5.4E-
4)0.941.3:0.01097)0.416.3:0.00378)0.795.4:0.00739,(((Ult21091:5.4E-
4,((((Ult18744:0.04594,(Ult18973:0.00743,Ult21077:5.5E-
4)0.996.2:0.00226)1.000.40:0.00148,((((Ult20228:0.0,Ult20994:0.0,Ult21082
:0.0,Ult21075:0.0,Ult21078:0.0,Ult21079:0.0,Ult21086:0.0,Ult21088:0.0,Ult
21089:0.0,Ult21090:0.0,Ult21446:0.0,Ult22378:0.0,Ult22670:0.0,Ult26844:0.
0):5.5E-4,((Ult20987:0.00718,Ult21087:0.01101)0.917.6:5.4E-
4,((Ult21085:0.00369,Ult21084:0.01891)0.974.3:5.5E-
4,(Ult21072:0.00369,Ult21076:0.00369)0.893.9:5.5E-4)0.000.109:5.5E-
4)0.057.1:5.5E-4)0.000.110:5.5E-
4,(Ult21092:0.00369,Ult21083:0.00369)0.674.1:5.5E-4)0.749.7:5.5E-
4,Ult21074:0.00367)0.795.5:5.4E-4)0.416.4:5.5E-4,((BacNL277:5.5E-
4,(BacNL278:0.00366,Ult18252:0.01497)0.843.3:5.5E-
4)0.838.5:0.00363,(Ult19826:5.5E-4,(Ult20988:0.00369,(Ult20744:5.5E-
4,Ult21080:5.5E-4)0.755.4:5.5E-4)0.850.4:0.0037)0.896.4:5.3E-
4)0.863.5:0.00363)0.176.3:5.5E-
4,(((Ult20742:0.00369,(Ult20991:0.0,Ult20993:0.0,Ult21181:0.0):5.5E-
4)0.890.10:0.00749,((Ult20989:0.0,Ult20990:0.0):5.4E-
4,Ult20699:0.01117)0.771.6:0.00369)0.775.2:0.00372,(Ult20995:0.01063,((Ul
t21003:0.04182,Ult22368:0.00843)0.898.13:0.01527,(Ult21093:5.5E-
4,(Ult20996:0.0074,CsrSpe10:0.02307)0.668.3:5.5E-
4)0.762.6:0.00245)0.626.2:0.00265)0.912.3:0.0027)0.620.2:5.5E-
4)0.836.5:0.00349)0.898.14:0.00769,Ult21424:0.03071)0.413.2:0.00355,Ult21
073:0.01074)0.886.8:0.00792)0.823.3:0.00848,Ult23174:0.02704)0.881.7:0.00
896)0.946.4:5.5E-4,(Ult20139:0.0,Ult21112:0.0):5.4E-
4)0.761.6:0.00341,Ult18056:0.00375)0.912.4:0.01023)0.950.7:5.4E-
4)0.932.4:0.01433)0.764.8:0.0038)0.774.9:0.00605,((Ult20414:0.02071,Ult20
415:0.12166)1.000.41:0.13765,(Ult20371:0.04995,(Ult19323:0.08556,Ult22228
:0.31557)0.918.8:0.08243)0.920.6:0.04792)0.734.1:0.012)0.767.1:0.0047)0.8
84.5:5.4E-
4,((((Ult19066:0.03773,JohIgnav:0.02886)0.989.8:0.04549,((Ult19161:5.5E-
4,(Ult18096:0.00743,(Ult19159:0.0,Ult19162:0.0):5.5E-
4)0.913.5:0.01508)0.914.6:0.01804,(Ult18078:0.01577,Ult19160:0.02044)0.23
7.3:0.00746)0.248.2:0.00508)0.930.3:0.02259,((Ult18312:0.05091,Ult20716:0
.02377)0.678.2:0.01165,((Ult20708:5.5E-
4,Ult21441:0.00369)0.952.11:0.02374,((Ult19490:0.0,Ult19491:0.0):0.01114,
(Ult19494:5.5E-4,(Ult19493:0.00369,Ult19492:0.0037)0.166.2:5.5E-
4)0.930.4:5.4E-
4)0.967.4:0.02729)0.516.1:0.00662)0.851.4:0.01273)0.754.6:0.00382,((Ult19
771:0.06444,(Ult19014:0.04496,(Ult19015:0.02488,(((UltB5566:0.16025,Ult223

69:0.00205)0.957.3:0.037,(Ult19016:0.00361,((Ult19024:0.01575,(((((Ult190
22:5.5E-4,Ult19020:0.00364)0.911.6:5.5E-
4,(Ult19017:0.00765,(Ult21442:0.01126,(Ult19019:0.00369,Ult19282:5.5E-
4)0.889.8:5.4E-
4)0.743.7:0.00359)0.970.4:0.01941)0.845.3:0.00999,Ult19018:0.03603)0.758.
6:0.00499,Ult19023:0.01168)0.848.6:0.00753,Ult19021:0.01942)0.979.4:0.024
15)0.756.9:0.00329,Ult19013:0.01518)0.767.2:0.0039)0.826.5:0.00904)0.825.
4:0.01639)0.814.2:0.01028)0.893.10:0.02212)0.811.7:0.01317,(((CstBact2:0.
00392,(UltClo21:0.0151,EubSpeci:0.01173)0.398.1:0.00396)0.977.3:0.02698,(
Ult20647:5.5E-
4,(((Ult18401:0.00384,(Ult20806:0.04462,Ult20796:0.03941)0.717.5:0.00472)
0.943.5:0.01411,(((Ult20797:0.0,Ult20799:0.0):5.5E-
4,(Ult17804:0.00369,Ult20801:0.00743)0.730.3:5.3E-4)0.958.4:5.4E-
4,(Ult17805:0.00767,(Ult20803:0.00748,(Ult20798:5.4E-
4,(Ult20800:0.00752,Ult20802:5.5E-4)0.895.7:0.0074)0.781.7:5.4E-
4)0.754.7:0.00369)0.951.6:0.01157)0.780.5:0.00484)0.819.5:0.01116,((Ult19
645:0.0,Ult19646:0.0):0.0234,(((UltB7201:0.0,Ult20915:0.0):0.00334,((Ult2
0916:0.07456,(Ult20917:5.5E-4,Ult18667:0.00369)0.622.1:5.4E-
4)0.867.8:0.00795,(Ult20918:5.5E-
4,Ult20919:0.01076)0.234.2:0.00758)0.828.4:0.00868)0.733.3:0.00403,(((Ult
18565:0.01419,Ult19345:0.03459)0.854.3:0.00885,(Ult20920:5.5E-
4,((Ult17937:0.0,Ult20913:0.0):5.5E-4,Ult20914:0.00372)0.322.1:5.5E-
4)0.936.5:5.4E-
4)0.771.7:0.00465,(Ult20921:0.00491,(Ult20809:0.00337,(UltLac24:0.00369,(
(UltB6403:0.0,Ult20794:0.0,Ult20792:0.0,DorFormi:0.0,Ult20804:0.0,Ult2129
6:0.0):5.5E-4,((Ult20808:0.00369,Ult20807:0.0037)0.729.1:5.5E-
4,(Ult20805:0.00369,Ult20793:0.00369)0.685.3:5.5E-4)0.000.111:5.5E-
4)0.705.2:5.5E-4)0.988.6:5.3E-
4)0.788.5:0.00612)0.763.8:0.00783)0.474.3:0.00661)0.620.3:0.00124)0.830.6
:0.01303)0.874.5:0.01137)0.979.5:0.02248)0.362.2:0.00302,Ult19647:0.04393
)0.958.5:0.02689)0.866.5:0.0144)0.911.7:0.01781)0.858.4:0.01012)0.903.5:0
.01182)0.726.3:0.00816)0.690.3:5.4E-
4,(((((Ult19424:0.01901,(((Ult17608:5.5E-
4,UltRu296:0.01138)0.922.6:0.0075,(Ult17622:0.01144,(Ult19678:0.00455,Ult
Lac46:0.00782)0.956.2:0.01868)0.747.5:0.00357)0.902.6:0.00723,(Ult19428:0
.00741,(Ult19616:0.00753,Ult19429:0.00371)0.560.2:5.4E-4)0.928.6:5.5E-
4)0.905.7:0.01117)0.777.7:0.00357,(((Ult19418:0.00369,((BacNL209:0.00726,
(BacNL210:0.00369,BacNL212:0.00368)0.819.6:0.00362)0.049.1:5.5E-
4,(((((Ult19421:0.00369,(CsrSpec2:0.0037,BacNL214:0.00387)0.315.1:5.0E-
4)0.208.1:5.5E-4,BacNL208:0.00369)0.492.3:5.5E-4,Ult19422:5.5E-
4)0.492.4:5.5E-4,Ult19419:5.5E-4)0.492.5:5.5E-
4,(Ult19420:0.0,BacNL207:0.0,BacNL213:0.0,Ult20687:0.0,Ult21328:0.0):5.5E
-4)0.000.112:5.5E-4)0.379.1:5.5E-4)0.280.1:5.5E-
4,Ult19423:0.01124)0.419.2:5.5E-4,BacNL211:5.5E-4)0.134.2:5.5E-
4)0.218.2:5.5E-
4,(Ult19621:0.01124,(BacNL218:0.00368,(CsrCitro:0.0,Ult19427:0.0,BacNL217
:0.0):5.5E-4)0.768.5:5.5E-
4)0.849.3:0.00367)0.993.3:0.02051,Ult19679:5.4E-
4)0.951.7:0.01879,((UltFir15:0.01153,UltClo32:0.00366)0.789.4:0.00637,(Ul
tClo61:0.00743,UltEub43:0.02736)0.779.2:0.00632)0.797.7:0.00644)0.874.6:0
.00864)0.886.9:0.00539)0.088.1:5.5E-
4,(((((Ult19462:0.01524,((((Ult19408:5.4E-
4,((Ult19399:0.00369,Ult19411:0.01872)0.000.113:5.5E-4,(Ult19409:5.5E-
4,((Ult19395:0.0,Ult19410:0.0,Ult19414:0.0,Ult21336:0.0):5.5E-

4,Ult19396:0.00368)0.000.114:5.5E-4)0.737.4:5.5E-
4)0.840.7:0.00371)0.785.5:0.00369,(Ult19401:5.3E-4,((((Ult19407:5.5E-
4,Ult19412:0.00742)0.827.7:0.0037,(Ult19398:0.08489,Ult19413:5.5E-
4)0.794.3:0.00374)0.538.1:5.4E-
4,(Ult19402:0.0,Ult19403:0.0,Ult19397:0.0,Ult19404:0.0,Ult19405:0.0,Ult19
406:0.0):5.5E-4)0.244.1:5.5E-
4,Ult19400:0.0037)0.861.5:0.00379)0.873.13:0.00783)0.749.8:0.004,(Ult1767
1:0.01895,Ult19457:5.5E-
4)0.935.5:0.01137)0.849.4:0.00767,((((Ult19227:0.01877,(((Ult19230:5.3E-
4,(Ult17524:5.5E-
4,Ult19229:0.00369)0.847.7:0.00369)0.785.6:0.006,(Ult18226:0.03196,(Ult18
477:0.01891,((Ult19223:0.0,Ult19224:0.0,Ult19225:0.0):5.5E-
4,Ult19226:0.00369)0.956.3:5.5E-
4)0.758.7:0.00318)0.888.7:0.01128)0.791.6:0.00609,Ult19228:0.00377)0.793.
5:0.00366)0.959.6:5.4E-4,(UltClo24:0.0,Ult19232:0.0,Ult19456:0.0):5.5E-
4)1.000.42:5.4E-4,((Ult19394:0.0,Ult20226:0.0):0.0153,Ult19231:5.4E-
4)0.798.11:0.00368)0.899.4:0.01101,Ult19233:0.0192)0.854.4:5.5E-
4)0.573.1:0.00814)0.856.3:0.00788,((Ult19253:0.01886,((((Ult18664:0.01145
,Ult18645:0.03601)0.842.8:0.00749,((Ult19351:0.05065,Ult19352:0.02235)0.9
53.6:5.4E-4,((Ult19349:0.0,Ult19350:0.0,Ult19353:0.0,Ult19354:0.0):5.5E-
4,(Ult19348:0.00369,((Ult19361:0.01516,(MouseGu2:0.00369,(Ult19360:0.0,Ul
t19362:0.0):5.5E-4)0.456.1:5.5E-
4)0.946.5:0.0115,Ult19355:0.00742)0.566.2:5.5E-4)0.377.1:5.5E-
4)0.176.4:5.4E-4)0.591.3:5.4E-
4)0.725.5:0.00355,Ult20493:0.01158)0.970.5:0.0281,((Ult19357:0.0112,Ult19
358:5.5E-4)1.000.43:5.4E-4,(Ult19359:5.4E-
4,Ult19356:0.01912)0.892.4:0.00349)0.741.5:0.00587)0.885.3:0.01738)0.923.
6:0.01824,(Ult21118:0.01618,Ult21117:0.01134)0.346.2:0.01067)0.325.1:5.3E
-
4)0.910.10:0.00784,(Ult20790:0.02309,((((((Ult19442:0.0,Ult19444:0.0):5.5
E-4,((Ult19443:0.0,Ult21463:0.0):5.5E-
4,(Ult19463:0.00743,Ult21434:0.00745)0.151.2:5.5E-
4)0.917.7:0.0037)0.996.3:5.4E-
4,(((Ult18077:0.0,Ult19448:0.0):0.00766,(((Ult17747:0.0037,Ult18109:0.003
71)0.783.5:0.0037,((Ult19434:0.00369,Ult19436:0.00369)0.683.3:5.5E-
4,(Ult19320:0.0,Ult19435:0.0,Ult19433:0.0,Ult19437:0.0):5.5E-
4)0.916.10:5.5E-4)0.638.1:5.5E-
4,Ult17859:0.00369)0.974.4:0.01534)0.734.2:0.00262,(Ult19447:0.01639,(Ult
19445:0.00369,Ult19446:5.5E-
4)0.723.1:0.00236)0.947.5:0.01659)0.793.6:0.00475)0.949.8:0.01126,((Ult19
247:0.03468,(Ult19243:0.00722,(Ult17495:0.01029,Ult19246:0.00578)0.891.6:
0.01007)0.909.1:5.5E-4)0.768.6:5.5E-
4,(Ult19244:0.0,Ult19245:0.0,Ult19248:0.0,Ult20932:0.0):5.5E-
4)0.957.4:5.4E-
4)0.861.6:0.00361,(((((Ult19210:0.00369,(Ult19216:0.04262,(Ult19214:0.001
93,HumanG11:0.03101)0.996.4:0.00184)0.896.5:5.5E-4)0.249.1:5.5E-
4,(Ult19212:0.0,Ult19213:0.0,Ult19215:0.0):5.5E-4)0.860.3:5.5E-
4,Ult19211:0.0075)0.913.6:0.00752,Ult19620:0.00378)0.901.10:0.00753,((Ult
19430:0.0,Ult21347:0.0):5.5E-4,(((Ult18972:0.0,BtPBact2:0.0):5.5E-
4,(Ult19613:0.00369,Ult19612:0.00394)0.152.1:5.5E-
4)0.917.8:0.00393,((((Ult19461:0.00366,Ult19458:0.07006)0.668.4:5.5E-
4,(Ult19459:0.0,Ult19460:0.0):5.5E-4)0.506.1:5.5E-
4,Ult19073:0.00369)0.913.7:0.00761,Ult19431:0.05947)0.848.7:5.5E-
4)0.381.5:5.5E-4)0.385.3:5.4E-4)0.315.2:5.5E-

4)0.851.5:0.00393,Ult19251:0.00749)0.568.1:5.5E-
4)0.840.8:0.00419)0.804.3:5.4E-4,((((((Ult17860:0.00368,Ult19239:5.5E-
4)0.983.3:0.03389,(Ult19455:0.01598,((Ult19440:0.00369,Ult19441:0.01121)0
.007.1:5.5E-4,(Ult19595:0.00739,(Ult19439:5.5E-
4,Ult21388:0.00369)0.972.5:5.5E-
4)0.855.7:0.00367)0.784.7:0.0069)0.388.2:0.0137)0.765.6:0.00768,(Ult19432
:0.0037,Ult21414:0.01877)0.987.5:5.3E-
4)0.769.8:0.00324,UltClo27:0.00406)0.252.1:0.00366,(((Ult19474:0.00369,(U
lt20680:0.0,Ult21114:0.0):5.5E-
4)0.865.7:0.00369,(Ult19611:0.00746,(Ult21113:0.00746,Ult23324:0.00373)0.
802.4:0.00367)0.572.1:5.5E-4)0.891.7:5.3E-
4,(Ult19242:0.00756,Ult19619:0.0114)0.880.8:0.00755)0.941.4:0.01104)0.721
.1:5.5E-
4,((((Ult19609:0.01135,((Ult19608:0.00369,Ult19610:0.01491)0.603.2:5.5E-
4,((Ult22376:0.00369,Ult19607:0.01492)0.633.1:5.5E-4,Ult19606:5.5E-
4)0.744.5:5.5E-4)0.908.5:5.4E-
4)0.853.5:0.0037,((((Ult19386:0.0,Ult19387:0.0):0.00847,((Ult19381:0.0120
7,(Ult19382:0.0,Ult19383:0.0,Ult19384:0.0,Ult19385:0.0):0.01212)0.979.6:0
.03083,UltClo26:0.01843)0.742.5:0.00515)0.822.4:0.01261,(UltBa929:0.01852
,(Ult19365:0.07893,(Ult19363:0.0387,(((Ult19369:0.0,Ult19370:0.0,Ult19371
:0.0):5.5E-
4,((Ult19364:0.00748,Ult19366:0.01915)0.773.10:0.00367,Ult19368:0.0074)0.
335.1:5.3E-4)0.552.2:5.4E-4,Ult19367:0.00369)0.835.8:6.1E-
4)0.687.2:0.0029)0.965.3:0.03599)0.710.2:0.01514)0.801.6:0.00734,((Ult207
36:0.02746,((UltClo29:0.00371,(Ult19583:0.00753,(Ult19585:0.00374,Ult1958
6:0.00746)0.891.8:0.00749)0.774.10:0.00373)0.804.4:0.00369,(Ult19582:5.5E
-4,Ult19584:5.5E-4)0.841.4:5.5E-
4)0.753.3:0.00343)0.985.5:0.02349,(Ult24022:0.01889,(UltBa753:0.0074,Ult1
9605:5.5E-4)0.870.5:5.4E-4)0.975.2:0.00189)0.966.6:0.00177)1.000.44:5.4E-
4)0.863.6:0.00375,((Ult19234:0.09597,(((Ult17496:0.0,Ult19237:0.0,Ult1923
6:0.0,Ult19235:0.0,Ult20934:0.0):5.4E-4,Ult17936:0.01122)0.816.4:5.4E-
4,Ult19238:0.09863)0.284.1:0.01019)0.821.3:0.00939,((Ult19593:0.00369,((U
lt19470:0.0,Ult19591:0.0):5.5E-
4,(Ult17907:0.00741,Ult19594:0.00369)0.654.1:5.5E-4)0.796.2:5.5E-
4)0.925.8:0.01559,(Ult18117:0.00742,(Ult17494:5.5E-
4,Ult17986:0.00369)0.039.1:5.5E-
4)0.904.5:0.01163)0.970.6:0.01966)0.904.6:5.4E-4)0.408.2:5.3E-
4,((Ult19312:0.00785,(Ult18341:0.01529,(((Ult19325:0.0,Ult19328:0.0,Ult19
329:0.0):5.5E-4,(Ult19326:0.00369,Ult19327:0.00369)0.707.1:5.5E-
4)0.910.11:5.5E-4,(Ult11297:0.04246,Ult21204:0.08213)0.867.9:5.4E-
4)0.599.1:5.3E-4)0.298.2:0.00716)0.960.5:0.01642,((Ult19307:5.4E-
4,(Ult19308:0.01145,(Ult19310:5.5E-
4,Ult19311:0.00369)0.989.9:0.03236)0.632.1:0.01168)0.958.6:0.01469,((Ult1
9332:0.01755,(Ult19309:5.5E-4,(Ult19334:0.01902,Ult19500:5.4E-
4)0.548.2:0.00369)0.781.8:0.00561)0.348.1:0.00799,(Ult19306:0.00374,((Ult
19322:0.01155,((Ult19324:0.00369,(Ult18557:0.01876,(Ult19317:5.4E-
4,Ult19321:0.04353)0.833.8:0.00369)0.486.1:5.5E-4)0.677.2:5.4E-
4,Ult19318:0.01501)0.778.9:0.00369)0.945.7:0.01157,((((Ult19298:0.0,Ult19
300:0.0):5.5E-
4,Ult19301:0.00741)0.782.11:0.00372,((Ult19303:0.0,Ult19304:0.0,Ult19305:
0.0):5.2E-4,((Ult19299:0.0037,Ult19302:5.5E-
4)0.856.4:0.0037,(BtPBacte:5.5E-4,Ult20110:5.5E-4)0.381.6:5.5E-
4)0.864.8:0.00394)0.909.2:0.00798)0.787.6:0.00396,((Ult19315:5.4E-
4,(Ult21348:0.01118,(Ult19313:0.00743,(Ult18079:0.00368,Ult19314:0.03506)

0.624.1:5.5E-4)0.177.3:5.5E-
4)0.983.4:0.01544)0.775.3:0.00364,Ult19316:0.01153)0.885.4:0.00772)0.918.
9:5.5E-4)0.846.5:0.00817)0.874.7:0.01457)0.180.1:0.01436)0.242.2:5.5E-
4)0.971.3:0.01707)0.715.1:0.00425)0.891.9:0.00812)0.916.11:0.01177,((((Ul
t19614:0.00727,(Ult19615:5.5E-4,Ult21389:0.00369)0.950.8:5.5E-
4)0.946.6:0.00169,(MouseGu3:0.00182,(Ult19617:0.0,Ult19618:0.0):0.02336)0
.215.1:5.1E-4)0.948.6:0.01457,(((Ult19475:0.0,Ult21115:0.0):5.4E-
4,Ult21116:0.00741)0.835.9:0.00371,((Ult20628:0.04535,(((Ult21016:5.4E-
4,(((Ult21010:0.0,Ult21012:0.0):5.5E-
4,Ult21013:0.0074)0.823.4:0.00369,(Ult21017:0.00745,Ult21015:0.01137)0.37
8.3:5.0E-
4)0.863.7:0.0037)0.901.11:0.01114,(UltClo50:0.00376,Ult21011:0.00372)0.92
0.7:0.01144)0.834.3:5.4E-
4,(Ult21009:0.00769,(Ult21014:0.02735,(Ult19762:0.01118,(((Ult19666:0.0,U
lt20186:0.0,Ult21001:0.0,Ult21004:0.0,Ult21006:0.0,Ult21007:0.0,UltRumi4:
0.0,RmcTorq2:0.0,Ult21008:0.0):5.5E-
4,(Ult21002:0.00369,RmcTorqu:0.00369)0.910.12:5.5E-4)0.902.7:5.5E-
4,(Ult20217:0.00369,Ult21005:5.5E-4)0.866.6:0.00369)0.600.1:5.3E-
4)0.965.4:5.3E-
4)0.962.6:0.01916)0.788.6:0.0037)0.737.5:0.0111)0.649.3:0.00396,((Ult1761
7:0.0,Ult17616:0.0):5.5E-
4,Ult20595:0.01876)0.793.7:0.01204)0.752.7:0.00327)0.897.5:5.4E-
4)0.641.3:0.00457,Ult18207:0.01939)0.165.1:5.4E-
4)0.898.15:0.01268)0.836.6:0.00348,((((CsrAlgid:0.0,CsrXyla2:0.0,CsrXylan
:0.0):5.3E-
4,(CsrSpec7:0.00743,((Ult19670:0.00712,(Ult18869:0.01899,(Ult18937:0.0036
9,UltLac13:5.5E-4)0.943.6:5.4E-4)0.928.7:0.00721)0.922.7:5.4E-
4,Ult19671:5.5E-4)0.260.2:5.5E-
4)0.934.6:0.00737)0.794.4:0.00369,((BacNL260:0.0144,BacNL261:5.5E-
4)0.829.5:5.4E-4,(BacNL256:5.5E-
4,(((((BacNL254:0.00368,Tryy0096:0.00746)0.728.2:5.5E-
4,(CsrSpec4:0.0,CsrSacch:0.0,BacNL255:0.0):5.5E-4)0.695.2:5.5E-
4,BacNL257:5.5E-4)1.000.45:0.00191,BacNL258:5.5E-
4)1.000.46:0.00176,(BacNL259:5.5E-
4,((CsrCeler:0.0,UltClo37:0.0,CsrSpec6:0.0):5.5E-
4,BacNL262:0.00749)0.060.2:5.5E-4)0.455.3:5.5E-4)0.139.3:5.5E-
4)0.962.7:5.5E-
4)0.768.7:0.00357)0.877.6:0.00975,((Ult18322:0.03753,((Ult17822:5.5E-
4,Ult17823:0.00369)0.858.5:0.0037,(((((((Ult19333:0.02775,Ult19521:0.0030
6)0.990.5:0.04024,(Ult18422:5.5E-4,(Ult18395:0.04042,(UltRu309:5.5E-
4,(UdnRum23:0.00369,((Ult18393:0.0,UltRu306:0.0,UltRu307:0.0,UltRu308:0.0
):5.5E-4,(Ult18394:5.5E-4,(Ult18396:5.5E-4,Ult18397:0.00369)0.743.8:5.5E-
4)0.000.115:5.5E-4)0.834.4:5.5E-
4)0.930.5:0.00336)0.271.1:0.01082)0.966.7:0.0241)0.310.1:0.02148)0.794.5:
0.00885,((Ult17796:0.0,Ult17797:0.0):5.4E-4,(Ult17795:5.5E-
4,Ult17794:0.02311)0.895.8:0.00745)0.737.6:0.00127)0.231.1:0.00728,Ult178
16:0.03716)0.854.5:0.00945,((Ult18344:0.00748,(Ult18363:0.00202,((Ult1836
1:0.0,Ult18362:0.0,Ult21370:0.0):5.5E-
4,(Ult18360:0.03533,Ult17990:0.00368)0.336.1:5.5E-
4)0.927.3:0.00218)0.847.8:0.00711)0.967.5:0.01929,(Ult17798:0.0,Ult18303:
0.0):0.01066)0.717.6:0.00324)0.801.7:0.00635,(Ult18423:5.5E-
4,Ult18424:0.06715)0.968.3:0.01967)0.996.5:5.4E-4,Ult17821:5.3E-
4)0.496.1:5.4E-
4)0.847.9:0.00761)0.754.8:0.00484,(((Ult18425:0.00752,((Ult19473:0.00741,

(Ult19070:0.01125,(Ult18870:0.00367,((Ult19069:0.0,Ult19072:0.0):5.5E-
4,(Ult19071:0.00369,Ult19068:0.00369)0.708.2:5.5E-4)0.862.5:5.5E-
4)0.107.3:5.5E-
4)0.760.7:0.00374)0.907.6:0.00749,(Ult21180:0.02104,(Ult17638:0.00974,Ult
17639:0.00919)0.398.2:0.00343)0.965.5:0.01679)0.739.8:0.00356)0.773.11:0.
00354,(((((Ult19677:5.4E-
4,UltClo39:0.00753)0.948.7:0.01124,((((Ult19668:0.00748,(((((UltB7195:0.0
201,Ult17768:0.00745)0.818.1:0.00764,((Ult17817:0.0,UltLach7:0.0,Ult17815
:0.0,Ult17818:0.0):5.4E-
4,Ult18243:0.01504)0.943.7:0.01179)0.779.3:0.00344,LchBacte:0.00749)0.776
.5:0.00377,(UltClo40:0.02125,(Ult17866:0.00369,Ult21410:5.5E-
4)0.789.5:0.00367)0.765.7:0.00413)0.795.6:0.00409,Ult18399:0.00753)0.474.
4:5.5E-4)0.063.1:5.4E-4,UltB7986:0.00369)0.729.2:5.3E-
4,UltLach2:0.01459)0.162.1:0.00391,((((((Ult19575:0.01126,((UltClo28:5.5E
-4,(Ult17840:0.00369,(Ult19577:0.01885,Ult19578:0.03055)0.986.1:5.5E-
4)0.962.8:5.5E-4)0.963.8:5.5E-4,Ult21340:0.00369)0.916.12:5.4E-
4)0.784.8:0.00533,(((Ult18074:0.00369,(UltB7190:5.5E-4,BacNL205:5.5E-
4)0.627.1:5.5E-4)0.371.3:5.3E-
4,(Ult18073:0.02273,((Ult18081:0.0,BacNL204:0.0):5.5E-
4,(Ult18072:0.0037,BacNL203:0.01153)0.723.2:5.4E-4)0.868.7:5.5E-
4)0.954.6:0.01154)0.972.6:0.02495,(((Ult20646:0.00369,(Ult19581:5.5E-
4,Ult17579:5.5E-4)0.982.2:5.5E-4)0.683.4:5.5E-
4,Ult19580:0.00741)0.911.8:0.01889,(Ult21258:0.03358,(Ult18356:0.00369,(U
lt18357:0.0,BtvCross:0.0,Ult18359:0.0):5.5E-
4)0.881.8:0.01526)0.473.1:0.01027)0.815.4:0.01257)0.295.2:0.00665)0.945.8
:0.02004,((Ult18833:0.00368,Ult18834:5.5E-
4)0.996.6:0.03624,RmnBact4:0.0157)0.738.3:0.00391)0.740.4:0.00326,(((Ult1
9623:0.00367,Ult19622:5.4E-
4)0.999.3:0.03218,(((UltEub41:0.04756,(Ult19626:0.00368,(Ult19627:5.5E-
4,(Ult19625:0.00369,Ult19624:0.00742)0.913.8:5.5E-4)0.737.7:5.5E-
4)0.940.3:0.03068)0.681.1:0.01508,((UncUn130:0.0,UncUn131:0.0):0.01752,Ul
tClo53:0.02685)0.969.9:0.03278)0.564.2:0.00865,((Ult17607:0.01805,((Ult18
160:0.00369,(Ult17720:0.00369,(Ult17721:0.0,Ult17719:0.0):5.5E-
4)0.471.3:5.5E-
4)0.795.7:0.00504,(Ult18106:0.01538,Ult17619:0.02699)0.227.2:0.00425)0.58
0.1:0.00554)0.956.4:0.02392,((Ult19573:0.0038,((Ult19572:0.0,Ult19574:0.0
):5.4E-
4,Ult19571:0.01919)0.370.1:0.00367)0.958.7:0.01962,((Ult18413:0.0,Ult1841
4:0.0):0.03945,Ult19570:5.5E-
4)0.852.9:0.01204)0.830.7:0.00857)0.780.6:0.00684)0.775.4:0.00447)0.034.2
:5.5E-
4,((Ult18831:0.0,Ult18832:0.0):0.04216,(Ult18729:0.00612,(Ult19115:0.0149
4,Ult19116:5.5E-
4)0.965.6:0.02056)0.996.7:0.04564)0.585.1:0.02257)0.767.3:0.00717)0.927.4
:0.01237,((UltClo23:0.0194,(((((Ult18836:0.04707,Ult18882:0.04293)0.842.9
:0.01768,(Ult18871:0.03213,Ult18893:0.01252)0.814.3:0.0093)0.684.2:0.0152
8,(((CsrSpeci:0.00371,Ult18890:0.00744)0.881.9:0.00759,((((Pshggg11:0.047
6,Ult18866:0.01356)0.927.5:0.02249,(((Ult17499:0.0,Ult18842:0.0):5.4E-
4,(Ult18841:0.02319,(Ult18837:5.5E-
4,Ult18838:0.01117)0.864.9:0.01011)0.849.5:0.00965)0.862.6:0.00765,((Ult1
8853:0.0037,((Ult18844:0.0,Ult18852:0.0,Ult18860:0.0,Ult18865:0.0):5.5E-
4,(Ult17862:0.01901,Ult18843:0.00762)0.661.1:5.5E-4)0.651.4:5.4E-
4)0.779.4:0.00399,((Ult18858:0.0,Ult18859:0.0):5.5E-4,(Ult18846:5.4E-
4,Ult18845:0.06095)0.886.10:0.01104)0.911.9:0.0114)0.750.2:0.00363)0.771.

8:0.0041)0.684.3:0.00412,((Ult18895:0.00567,(Ult18894:0.00742,Ult24601:5.5E-
4)0.995.5:0.04)0.007.2:0.01453,(((((Ult21318:0.00368,Ult18564:0.02715)0.795.8:0.00372,Ult18851:0.00369)0.581.2:5.4E-
4,(Ult18848:0.0,Ult18849:0.0,Ult18850:0.0):5.5E-4)0.968.4:5.4E-
4,((((Ult18856:0.0,Ult18857:0.0):5.5E-
4,(Ult18854:0.05541,Ult18855:0.00365)0.003.2:5.5E-
4)0.932.5:0.00371,Ult18847:5.5E-4)0.364.2:5.4E-
4,Ult18500:0.00741)0.857.5:0.00368)0.907.7:0.01455,((Ult18862:5.5E-
4,Ult18863:0.00369)0.936.6:0.00749,((Ult18867:5.4E-
4,(Ult18839:0.02183,Ult19487:0.05477)0.925.9:0.02215)0.866.7:0.00719,(Ult18861:5.5E-4,(Ult18864:0.03889,Ult18323:0.00367)1.000.47:5.5E-
4)0.642.2:5.4E-4)0.957.5:5.4E-
4)0.919.7:0.01458)0.845.4:0.0014)0.406.2:0.01199)0.961.5:0.01581,Ult20096:0.02989)0.955.7:5.4E-4)0.763.9:0.00385,(Ult21352:0.00246,(Ult18888:5.5E-
4,Ult18889:0.03299)0.921.9:0.00246)0.841.5:0.00267)0.800.4:0.01178)0.943.8:0.02258,Ult18963:0.04555)0.469.3:0.00416,(((Ult22366:0.08089,(UltB7209:0.20249,Ult18942:0.04879)0.438.1:0.01539)0.825.5:0.01662,((Ult21459:0.10687,Ult21460:5.5E-4)0.230.3:0.01544,(Ult18231:0.0237,(Ult18229:5.5E-
4,(Ult18230:0.00762,Ult18228:0.0389)0.772.7:0.00333)0.946.7:0.02815)1.000.48:0.07831)0.826.6:0.00959)0.756.10:0.00485,(Ult18835:0.01347,(((((Ult18880:0.00744,Ult18876:0.00369)0.700.5:5.5E-
4,((Ult18874:0.0,Ult18875:0.0,Ult18872:0.0,Ult18877:0.0,UltCopro:0.0,Ult18881:0.0):5.5E-4,CopCatus:5.5E-4)0.000.116:5.5E-4)0.931.7:5.4E-
4,(Ult20841:0.02298,Ult18878:0.00375)0.804.5:0.00365)0.849.6:5.5E-
4,(Ult18879:5.4E-
4,Ult20317:0.01916)0.873.14:0.00742)0.847.10:0.00882,Ult18873:0.01401)0.925.10:0.02226)0.902.8:0.01713)0.747.6:0.00485)0.746.6:0.00613)0.837.5:0.00872,(((CsrFimet:0.01124,((((UltLac14:0.01857,(Ult18886:0.04254,(Ult21219:0.00746,Ult21220:0.00372)0.927.6:5.4E-
4)0.887.6:0.00927)0.893.11:0.00992,(((Ult21456:5.4E-
4,Ult21457:0.00368)0.996.8:0.0394,(UltClo52:0.01948,Ult21458:0.02373)0.472.1:0.0153)0.993.4:5.3E-4,((AsbMobil:0.0,Ult21218:0.0):5.5E-
4,(Ult21216:0.00741,Ult21217:0.00799)0.276.2:5.4E-4)0.540.2:5.5E-
4)0.615.3:5.4E-
4)0.767.4:0.00389,BacNL288:0.01391)0.792.8:0.00485,(((Ult21140:0.0074,(Ult21138:0.00369,Ult21139:5.5E-4)0.675.1:5.5E-
4)0.992.3:0.02813,(Ult18071:0.0445,((Ult18840:0.057,PahggY46:0.01866)0.914.7:0.01823,((((Bfhggg42:0.0227,(Bfhggg44:0.0,Bfhggg43:0.0,Bfhggg45:0.0):5.4E-4)0.805.5:5.4E-
4,((((HlmSpe14:0.26975,AtPYy144:0.1981)0.963.9:0.10724,(UltOr110:0.2798,Pshggg54:0.14114)0.123.2:0.02251)1.000.49:0.28385,Bfhggg46:5.4E-
4)0.912.5:0.0442,(UltRu416:0.45196,AtPYy142:0.17677)1.000.50:0.38213)0.962.9:0.06139)0.953.7:0.01881,((((AtPYy115:1.23973,(Bfhggg17:0.11866,EchggYy5:0.20005)0.875.7:0.118)0.181.2:0.06154,(AtPYy113:0.34209,EchggYy7:0.01233)0.995.6:0.19561)0.764.9:0.06677,(UdnRum17:0.32651,AtPYy109:1.61727)0.798.12:0.1557)0.999.4:0.37365,((((PsdSpe56:0.52971,Ult17379:0.65172)0.979.7:0.42565,(UltHol29:0.44046,(((UltB2762:0.43776,(UltB9361:0.28537,Ult25026:0.84032)0.948.8:0.27808)0.862.7:0.14259,Ult30657:1.11386)0.232.2:0.14131,((EchggYy3:0.12777,(UltOrga5:0.29568,UltOrg87:0.28105)1.000.51:0.42129)0.911.10:0.13647,UltOr244:0.23747)0.315.3:0.07028)0.723.3:0.02971)0.052.1:0.05954)0.254.1:0.01084,((((BrkSpec2:0.18306,((((UdnBact8:0.59342,UdnBact9:0.53434)0.772.8:0.16121,(UltFungu:0.36478,AtPYy087:0.744)0.695.3:0.12229)0.897.6:0.14942,AtPYy081:0.25165)0.488.2:0.10702,CmmAquat:0.2641)0.454

.2:0.04845)0.943.9:0.14443,(SalEnter:0.30196,UltOrgan:0.22627)0.889.9:0.0
5397)0.899.5:0.06652,((((AtPYy096:0.82265,(AtPYy094:0.7386,(AtPYy092:0.51
608,AtPYy095:0.59002)0.952.12:0.31028)0.517.1:0.10086)0.863.8:0.18963,(At
PYy083:0.65794,Ult11490:0.87275)0.913.9:0.27466)0.830.8:0.09467,(Ult14793
:0.90782,UltB1944:0.49768)0.669.2:0.12858)0.803.4:0.04961,(Ult14381:1.612
82,(ClpSpec3:1.74855,UltOr144:1.12777)0.408.3:0.26523)0.493.1:0.14373)0.3
41.1:0.0466)0.523.1:0.05443,((UltOrga4:0.24484,AciCal14:0.31317)0.971.4:0
.13419,(((UltB1133:0.07308,AztVine2:0.17886)0.999.5:0.14237,(((PsdSpe68:0
.05539,(UltSpon2:0.16753,UltAc852:0.29521)0.997.8:0.21857)0.869.4:0.0559,
(PsdSpe37:0.29626,PsdPuti5:0.12525)0.754.9:0.05824)0.600.2:0.05509,PsdSpe
57:0.05227)0.857.6:0.0822)0.661.2:0.04852,(UltB2748:0.16796,UltThiot:0.12
14)0.834.5:0.03822)0.857.7:0.04113)0.883.4:0.04544)0.045.1:0.03475)0.861.
7:0.05599,(AtPYy152:0.66397,(EtcSpeci:0.56804,(Ult30922:0.50568,(UltCellu
:5.5E-4,UltGlaci:5.5E-
4)0.992.4:0.39702)0.815.5:0.14843)0.943.10:0.37727)0.965.7:0.29118)0.335.
2:0.06474)0.690.4:0.02546)0.860.4:5.5E-
4,Ult21214:0.058)0.899.6:0.00905)0.761.7:0.00539)0.861.8:0.01534)0.970.7:
5.5E-
4,(Ult19092:0.02277,Ult21205:0.01767)0.363.2:0.0116)0.728.3:0.01719)0.487
.2:5.4E-4)0.935.6:5.4E-
4,((UltLac32:0.03133,(((((Ult21213:0.00481,Ult25908:0.07988)0.862.8:0.012
88,CsrPhyto:0.01733)0.405.1:0.00596,PpbPauci:0.00793)0.847.11:0.00874,((U
lt21215:0.01538,(UltBacil:5.4E-
4,CsdBact2:0.01514)0.774.11:0.00755)0.771.9:0.00379,CsrPopul:0.00754)0.79
3.8:0.00375)0.925.11:0.00246,((Ult21266:0.0,Ult21475:0.0):0.00374,Ult2147
3:0.00775)0.925.12:0.00247)0.925.13:0.00234)0.132.2:5.4E-
4,(Ult21237:0.00376,(BacNL290:0.01462,((BacNL295:5.5E-
4,(BacNL294:0.00365,BacNL296:5.5E-4)0.129.2:5.5E-
4)0.923.7:0.00361,((BacNL289:0.0,BacNL291:0.0,BacNL293:0.0):5.5E-
4,BacNL292:5.5E-4)0.976.3:5.5E-4)0.616.1:5.5E-
4)0.884.6:0.00719)0.894.6:0.00718)0.924.8:0.007)0.705.3:5.4E-
4,((Ult19085:0.0068,(((Ult19086:0.01781,(Ult21208:0.02224,Ult21209:0.0207
6)0.921.10:0.01808)0.773.12:0.00782,(((Ult19110:5.5E-4,Ult19111:5.5E-
4)0.886.11:0.00734,(((Ult19097:0.0,Ult19101:0.0,Ult19102:0.0,Ult19103:0.0
,Ult19109:0.0):5.5E-4,(Ult19104:0.00369,Ult19106:0.00369)0.665.2:5.5E-
4)0.962.10:5.4E-4,((HumanGu8:5.5E-
4,(Ult19108:0.00369,Ult19105:0.00369)0.086.2:5.5E-4)1.000.52:5.4E-
4,(EubElige:5.4E-4,(Ult19107:0.00741,Ult22370:5.5E-
4)0.851.6:0.00362)0.185.2:0.00371)0.748.4:0.00361)0.919.8:0.01161)0.857.8
:0.00888,(Ult19075:0.01356,(Ult19074:0.02692,((Ult19078:0.0,Ult19079:0.0,
Ult19081:0.0,Ult19082:0.0,Ult19083:0.0,Ult19084:0.0,Ult19098:0.0,Ult19117
:0.0):5.5E-4,(HumanGu7:0.00366,Ult19076:0.05563)0.998.2:5.5E-
4)0.992.5:5.4E-
4)0.877.7:0.01072)0.853.6:0.01024)0.886.12:0.01488)0.672.2:0.01485,(Ult18
300:0.01925,(((Ult19094:0.0,Ult19095:0.0):5.5E-
4,Ult21450:0.015)0.893.12:0.00761,(Ult19114:0.00369,(((Ult19112:0.0,Ult19
113:0.0):5.3E-4,((Ult19077:0.00742,Ult19080:5.5E-4)0.815.6:5.5E-
4,Ult19087:0.01508)0.924.9:0.00747)0.902.9:0.00745,((Ult18749:0.0,Ult1909
6:0.0,Ult19088:0.0,Ult19091:0.0):5.5E-
4,((Ult18260:0.01872,Ult19587:0.00369)0.643.1:5.5E-
4,(Ult19089:0.00369,Ult19090:0.00394)0.905.8:5.5E-4)0.929.5:5.5E-
4)0.902.10:5.5E-4)0.994.8:5.4E-
4)0.885.5:0.00761)0.763.10:0.00382)0.341.2:0.00502)0.138.2:5.4E-
4)0.961.6:0.01877,((Ult20079:0.01457,(ApsCacca:0.00379,ApsSpeci:0.01136)0

.736.5:0.00431)0.898.16:0.01538,(((((((Ult21202:0.0075,(Ult21178:0.01104,
((((Ult21159:5.5E-4,Ult21169:5.5E-4)0.909.3:0.00369,(((((Ult21152:5.4E-
4,(Ult21144:0.00352,Ult21175:0.03483)0.782.12:0.00388)0.870.6:0.00369,Ult
18287:0.0112)0.718.3:5.4E-
4,(Ult21151:0.01124,Ult21145:0.00369)0.717.7:5.5E-4)0.186.3:5.5E-
4,(Ult21171:0.00369,Ult21150:0.00741)0.690.5:5.5E-4)0.000.117:5.5E-
4,(Ult19099:0.0,Ult21161:0.0,Ult21166:0.0,Ult21172:0.0,Ult21148:0.0,Ult21
155:0.0,Ult21176:0.0,Ult21179:0.0,Ult21184:0.0,Ult21186:0.0,Ult21192:0.0,
Ult21194:0.0,Ult21292:0.0,Ult22623:0.0):5.5E-4)0.000.118:5.5E-
4)0.816.5:5.5E-4,(Ult21160:0.00369,Ult21173:0.01494)0.229.1:5.5E-
4)0.983.5:5.5E-4,((UltAna12:5.5E-4,((Ult20688:5.5E-
4,(Ult20649:0.01116,(((Ult21154:5.5E-4,Ult21203:5.4E-
4)0.842.10:0.00369,Ult21146:0.01118)0.812.1:5.4E-
4,(Ult21191:0.00369,Ult21193:0.0037)1.000.53:5.5E-4)0.000.119:5.5E-
4)1.000.54:5.5E-4)0.000.120:5.5E-
4,(Ult21167:0.0,Ult21147:0.0,Ult21174:0.0,Ult21183:0.0,Ult21185:0.0,Ult21
187:0.0):5.5E-4)0.739.9:5.5E-4)0.969.10:5.4E-
4,(Ult19776:0.05946,Ult21170:0.01099)0.878.3:5.4E-
4)0.922.8:0.00364)0.916.13:5.4E-4)0.881.10:0.00708)0.938.6:5.4E-
4,(Ult21201:0.00761,Ult23262:0.01959)0.819.7:0.01147)0.755.5:0.01084,(Ult
21165:0.02281,(Ult18934:0.03661,Ult21168:0.0155)0.934.7:0.02051)0.883.5:0
.0127)0.760.8:0.00545,(((Ult18933:5.4E-
4,Ult21222:0.02633)0.896.6:0.00735,((((((Ult18912:0.0,Ult18914:0.0):5.5E-
4,Ult18935:5.5E-4)0.881.11:0.00752,(Ult18913:0.01879,(Ult18916:5.5E-
4,Ult18915:0.00742)0.800.5:5.5E-
4)0.771.10:0.00364)0.918.10:0.01645,(Ult18348:0.03756,UltRu300:0.03459)0.
810.5:0.01606)0.792.9:0.00907,Ult21200:0.01555)0.019.1:0.00895,UltRu331:0
.03437)0.878.4:0.00887)0.328.1:5.4E-4,((Ult18930:0.0,Ult18936:0.0):5.4E-
4,(Ult18932:0.0112,Ult18931:0.0037)0.783.6:0.00367)0.957.6:0.01079)0.298.
3:0.00641)0.744.6:0.00325,(Ult21199:0.01517,((Ult18753:0.01212,Ult18779:0
.0105)0.879.5:0.01455,(Ult21195:0.01117,(Ult21196:0.0,Ult21197:0.0,Ult211
98:0.0,Ult26846:0.0):5.5E-
4)0.861.9:0.0133)0.044.2:0.01197)0.919.9:0.01892)0.793.9:5.4E-
4,(UltB7194:0.02651,Ult21382:0.04234)0.886.13:0.01537)0.829.6:0.00701,((U
lt18900:5.4E-
4,(((Ult18901:0.0,Ult18896:0.0,Ult18897:0.0,Ult18902:0.0,Ult18903:0.0,Ult
18904:0.0,Ult18905:0.0,Ult18906:0.0,Ult18907:0.0,Ult18908:0.0,Ult21321:0.
0,Ult21344:0.0):5.4E-
4,(Ult18909:0.00728,(Ult22371:0.01554,Ult20534:0.02711)0.527.1:0.00726)0.
931.8:5.4E-4)0.998.3:5.4E-
4,(UltRu322:0.03967,(EubRumin:0.01945,((Ult18911:0.00899,(UltRu329:0.0237
1,(UltRu330:0.0251,((UltRu326:5.4E-
4,(Ult18917:0.00366,(UltRu327:0.01122,(UltRu323:0.00366,(UltRu324:0.00745
,UltRu325:0.00369)1.000.55:5.4E-4)0.578.1:0.00746)0.932.6:5.5E-
4)0.858.6:0.00367)0.885.6:0.00797,(Ult18918:0.0071,UltRu328:0.02411)0.869
.5:0.01095)0.907.8:0.01731)0.895.9:0.01603)0.799.6:0.00982)0.906.7:0.0149
6,Ult18910:0.00498)0.771.11:0.00588)0.933.9:0.01105)0.800.6:5.4E-
4)0.953.8:0.00707)0.941.5:5.5E-4,(UltRu379:0.02492,(Ult21558:5.5E-
4,Ult21559:0.00369)0.927.7:0.02495)0.973.2:0.03456)0.969.11:0.02535)0.844
.11:0.00947)0.885.7:0.01125)0.762.7:0.00323)0.875.8:0.00788)0.877.8:0.008
91)0.925.14:5.3E-
4,((Ult18355:0.04053,Ult18938:0.0226)0.520.2:0.00887,Ult18398:0.0033)0.85
2.10:0.008)0.903.6:0.00915)0.325.2:5.3E-4)0.172.3:5.5E-
4,(((Ult18928:0.01331,(((((CopEutac:5.4E-

4,(Ult21320:0.03249,((Ult18924:0.00369,(Ult18923:5.5E-4,Ult18926:5.5E-4)0.924.10:5.5E-4)0.960.6:5.4E-4,(Ult18925:0.01042,Ult18927:0.01882)0.760.9:0.00528)0.743.9:0.00309)0.976.4:0.0287)0.839.5:0.00368,(Ult18920:0.00193,Ult21299:0.0474)0.998.4:0.00183)0.089.1:5.5E-4,(UltB7185:0.0,Ult18919:0.0,Ult18922:0.0,Ult20475:0.0):5.5E-4)0.919.10:0.00206,Ult18921:0.00209)0.866.8:0.0071,Ult20476:0.00358)0.965.8:0.03127)0.975.3:0.03536,Ult19093:0.01198)0.918.11:0.02033,(((Ult19667:0.01184,(Ult17623:0.01223,UltLach3:0.01993)0.756.11:0.00314)0.840.9:0.00722,((((Ult17635:0.00825,(Ult17632:0.00369,(Ult17633:0.00371,((Ult17631:0.0,Ult17634:0.0):5.5E-4,(Ult17630:0.0074,Ult19712:0.02658)0.987.6:5.5E-4)0.950.9:5.5E-4)0.761.8:5.5E-4)0.907.9:0.01325)0.773.13:0.00776,((Ult18430:0.01275,Ult19027:0.03961)0.882.5:0.01919,((Ult19393:0.00369,(Ult18471:0.00369,(Ult18037:0.0,Ult18036:0.0):5.5E-4)0.137.2:5.5E-4)0.943.11:0.01538,(((Ult18025:0.00431,(Ult18026:0.00378,Ult18115:0.01131)0.921.11:0.0044)0.929.6:0.00985,Ult18035:5.4E-4)0.925.15:0.01148,(((Ult18031:0.0,Ult18032:0.0,Ult18033:0.0):5.5E-4,(Ult18594:0.00741,Ult21380:0.00369)0.605.1:5.5E-4)0.894.7:5.5E-4,(Ult18034:0.12646,(Ult20152:0.0266,(Ult18029:0.00369,Ult18030:5.5E-4)0.794.6:0.00535)0.757.5:0.00492)0.799.7:0.00554)0.936.7:0.0117)0.747.7:0.00366)0.792.10:0.00707)0.786.5:0.00663)0.926.10:0.01484,((((((Ult19674:5.4E-4,(Ult21207:0.01845,((((CsrAmino:5.5E-4,BacNL287:9.9E-4)0.998.5:0.00298,(BacNL283:5.4E-4,BacNL286:0.01524)0.832.5:0.00369)0.119.1:5.5E-4,BacNL285:0.00734)0.093.2:5.4E-4,BacNL284:0.01115)0.997.9:0.03829)0.279.1:0.00974)0.780.7:0.01192,UltLac34:0.01112)0.146.1:5.5E-4,Ult21285:0.00787)0.919.11:0.01176,(Ult19675:0.04867,((Ult22361:0.0275,(Ult17636:0.01887,Ult21346:0.00384)0.782.13:0.00329)0.749.9:0.00655,(Ult18980:0.05106,UltRu357:0.04025)0.156.2:0.00719)0.394.1:0.00977)0.258.1:0.00375)0.785.7:0.00444,UltLac33:5.5E-4)0.779.5:0.00392,(Ult18428:0.00476,((Ult18384:0.03265,Ult19604:0.01796)0.912.6:0.02043,Ult18429:0.01312)0.777.8:0.00595)0.904.7:0.01121)0.412.1:0.00432)0.817.2:5.5E-4,(((Ult18421:5.5E-4,Ult21444:0.00745)0.938.7:0.01109,(Ult17637:0.01514,Ult18427:0.0076)0.206.1:5.4E-4)0.952.13:0.01124,(((((UltB7205:0.0,Ult18417:0.0,Ult18420:0.0,Ult23533:0.0):5.5E-4,Ult20182:0.00366)0.000.121:5.4E-4,(Ult18419:0.0074,Ult21221:5.5E-4)0.846.6:0.00366)0.998.6:5.5E-4,(Ult18116:0.018,Ult19026:0.00985)0.901.12:0.01815)0.914.8:0.00696,Ult19676:0.00369)0.319.1:5.3E-4)0.863.9:0.00709)0.925.16:0.00681)0.834.6:5.4E-4,(Ult21467:5.3E-4,((((Ult21471:0.00718,((Ult21279:0.0041,(Ult17906:0.00781,((Ult21277:0.00368,((Ult21272:0.0,Ult21273:0.0):5.5E-4,Ult21390:0.00366)0.909.4:5.5E-4)0.949.9:0.01172,(((Ult21274:0.0,Ult21275:0.0,Ult21276:0.0,Ult21268:0.0,Ult21269:0.0,Ult21379:0.0):5.5E-4,(Ult21278:0.01831,(Ult18170:0.00374,Ult20449:0.02275)0.785.8:0.00571)0.774.12:0.00583)0.954.7:5.5E-4,(Ult21270:0.01505,Ult21271:0.0587)0.751.4:0.00329)0.747.8:0.00325)0.759.5:0.00361)0.917.9:0.01113)0.858.7:0.00137,(Ult21280:0.00369,(Ult21281:5.5E-4,Ult21282:5.5E-4)0.742.6:5.5E-4)0.965.9:0.00205)0.981.2:0.01813)0.888.8:5.4E-4,(Ult19454:0.01275,(((Ult18199:0.00749,Ult18200:0.00748)0.939.5:5.5E-

4,((Ult21225:0.00366,((Ult21224:0.01188,(UltRu372:5.5E-4,(Ult20643:5.5E-
4,Ult21223:0.00369)0.850.5:0.00369)0.766.5:0.00315)0.852.11:0.00779,(((Ul
t21228:0.01496,(Ult21227:5.5E-4,Ult21229:0.00369)0.813.1:5.5E-
4)0.944.5:0.01126,Ult21230:5.3E-4)0.603.3:5.4E-
4,Ult21231:0.0112)0.951.8:0.01896)0.962.11:0.01991)0.935.7:0.01134,Ult212
26:5.4E-
4)0.998.7:0.03477)0.692.2:0.01944,((Ult18165:0.00376,(Ult18666:0.16106,(U
lt18649:0.00744,(Ult18642:5.4E-
4,Ult18634:0.00742)0.802.5:0.00369)0.741.6:0.0024)0.713.3:0.01246)0.983.6
:0.02622,((UltClo13:0.04069,(UltB7246:0.00748,(((((Ult17858:0.00369,Ult20
419:0.00369)0.857.9:5.5E-
4,((Ult20420:0.0,Ult20421:0.0,Ult20424:0.0,Ult20480:0.0):5.5E-
4,(Ult20423:0.00369,Ult20425:0.00743)0.718.4:5.5E-4)0.000.122:5.5E-
4)0.917.10:5.4E-
4,(Ult20422:0.01932,Ult20441:0.01138)0.906.8:0.0114)0.822.5:0.00703,(((Ul
t20416:0.02295,Ult20417:0.01909)0.858.8:0.00726,(Ult20418:0.02275,((Ult20
405:0.00691,((Ult20401:0.00369,((Ult20400:0.0,Ult20402:0.0,Ult20403:0.0,U
lt20404:0.0,Ult20406:0.0,Ult22388:0.0):5.5E-
4,(Ult20399:0.00369,UltB7211:0.00369)0.683.5:5.5E-4)0.000.123:5.5E-
4)0.609.2:5.5E-
4,Ult20407:0.0517)0.916.14:0.00201)0.914.9:0.00214,Ult20408:0.00743)0.990
.6:0.02291)0.753.4:0.00361)0.949.10:0.01514,((Ult20429:0.01123,(Ult20430:
5.2E-4,UltClo42:0.00743)0.586.3:5.5E-
4)0.925.17:0.00232,((Ult20433:0.02264,(Ult20484:0.00368,((Ult18186:0.0,Ul
t20434:0.0,Ult20435:0.0,Ult20436:0.0,Ult21407:0.0):5.5E-
4,(Ult20431:0.00743,Ult20432:0.00369)0.714.3:5.5E-4)0.888.9:5.5E-
4)0.893.13:5.4E-
4)0.803.5:0.00376,(Ult20427:0.04838,(Ult20426:0.04283,Ult20428:5.5E-
4)0.735.4:0.00248)0.960.7:0.02054)0.923.8:0.00231)0.800.7:0.00255)0.933.1
0:0.0154)0.891.10:0.00386,(Ult18647:0.019,(Ult20479:0.00716,Ult20481:0.00
403)0.887.7:0.00757)0.741.7:0.00371)0.817.3:5.4E-
4)0.979.8:0.01931)0.000.124:5.5E-
4,((Ult18623:0.05338,((((Ult18606:0.0,Ult18608:0.0,Ult22389:0.0):5.5E-
4,Ult18609:5.5E-4)0.581.3:5.4E-4,((Ult18605:5.5E-4,Ult18607:5.5E-
4)1.000.56:5.5E-
4,Ult18610:0.09203)0.677.3:0.00363)0.938.8:0.02019,(Ult20446:0.06272,Ult2
0448:0.00807)0.890.11:0.01771)0.071.1:0.00764)0.964.7:0.02752,((((Ult1813
2:0.01472,Ult18134:5.5E-
4)0.872.14:0.00364,((Ult18146:0.02643,Ult18153:0.00368)0.358.2:5.5E-
4,(((((((Ult18121:0.00369,(Ult18147:0.00371,Ult18130:0.01913)0.372.1:5.5E
-4)0.405.2:5.5E-4,UltB7250:0.00369)0.000.125:5.5E-
4,Ult18154:0.00369)0.451.1:5.5E-4,(Ult18120:5.4E-
4,(Ult18119:0.0112,Ult18138:0.00742)0.764.10:0.00372)0.871.8:0.00369)0.45
5.4:5.5E-
4,(Ult18123:0.0,Ult18124:0.0,Ult18125:0.0,Ult18127:0.0,Ult18129:0.0,Ult18
144:0.0,Ult18149:0.0,Ult18150:0.0):5.5E-4)0.000.126:5.5E-
4,((Ult18122:0.0,Ult18126:0.0,Ult18128:0.0,Ult18141:0.0,Ult18142:0.0,Ult1
8148:0.0):5.5E-4,(Ult18118:0.01119,Ult18164:0.02303)0.373.1:5.5E-
4)0.929.7:0.00369)0.408.4:5.5E-
4,(Ult18143:0.0074,(Ult18151:0.00368,Ult18131:5.5E-4)0.205.2:5.5E-
4)0.831.6:0.00368)0.438.2:5.4E-4)0.295.3:5.4E-
4)0.869.6:0.00367,Ult18145:5.4E-
4)0.796.3:0.00375,(((Ult18135:0.0,Ult18136:0.0):5.4E-
4,((Ult18137:0.01505,Ult23633:5.5E-

4)0.862.9:0.00369,(UltB2007:0.01884,Ult18139:0.0269)0.613.2:5.4E-
4)0.845.5:0.00369)0.988.7:0.0192,(Ult17841:0.0238,((Ult18169:0.0,Ult21412
:0.0):5.5E-4,(Ult18168:0.0037,(Ult18167:5.5E-
4,(Ult18166:0.00369,UltClos7:0.00369)0.879.6:5.5E-4)0.892.5:5.5E-
4)0.956.5:0.0112)0.317.1:5.5E-
4)0.774.13:0.00372)0.749.10:0.00374)0.929.8:0.01179)0.746.7:0.003)0.000.1
27:5.5E-
4)0.784.9:0.01362)0.883.6:0.01469)0.766.6:0.0057)0.804.6:0.00336,(((BacNL
206:5.5E-4,(Ult21327:0.00369,(Ult19415:0.0,Ult19416:0.0):5.5E-
4)0.727.4:5.5E-
4)0.930.6:0.0074,((Ult21465:0.00719,(Ult18320:0.0111,(Ult21469:0.0,Ult214
70:0.0):5.5E-4)0.910.13:5.5E-
4)0.951.9:0.01108,(Ult21265:0.00278,Ult21371:0.03138)0.955.8:0.01594)0.41
3.3:5.5E-
4)0.912.7:0.00728,(Ult21466:0.00741,Ult21468:0.00371)0.904.8:5.4E-
4)0.994.9:5.4E-
4)0.832.6:0.00326,((((((Ult24103:0.02857,((Ult20230:0.01522,(Ult18775:0.0
037,(Ult19202:0.0,Ult19203:0.0):5.5E-4)0.821.4:0.00378)0.862.10:5.5E-
4,(Ult19201:0.00778,((Ult20218:0.03357,Ult22683:0.02781)0.749.11:0.0134,(
Ult18793:0.0271,(((((Ult18795:0.00201,Ult18310:0.01087)0.935.8:0.00199,((
Ult18752:0.00369,Ult18771:0.01115)0.597.3:5.5E-
4,((Ult18800:0.00369,Ult22382:0.00369)0.920.8:5.5E-
4,(Ult18760:0.0,Ult18759:0.0,Ult18773:0.0,Ult18804:0.0):5.5E-
4)0.000.128:5.5E-4)0.888.10:5.5E-
4)0.854.6:0.0037,((Ult18756:0.0,UltEub40:0.0,Ult18766:0.0,Ult18768:0.0,Ul
t18798:0.0,Ult18805:0.0,Ult20474:0.0,Ult20621:0.0):5.5E-
4,(Ult18801:0.00741,Ult22379:5.5E-4)0.847.12:0.00369)0.172.4:5.5E-
4)0.842.11:0.00371,Ult18769:5.5E-
4)0.827.8:0.00366,(((Ult18245:0.01296,((Ult18782:0.01339,(Ult18272:0.0,Ul
t20622:0.0):0.02586)0.797.8:0.00961,((Ult18790:0.04762,(Ult18796:0.0,Ult1
8797:0.0):5.4E-4)0.833.9:0.00367,Ult18784:5.4E-
4)0.797.9:0.00671)0.925.18:0.0182)0.869.7:0.01412,(Ult18786:0.03377,(Ult1
8785:0.01867,(UltRu319:0.01951,(Ult18781:0.06382,(UltRu318:0.01654,Ult223
81:0.03108)0.838.6:0.01892)0.821.5:0.01597)0.809.5:0.01519)0.694.3:0.0027
8)0.696.1:0.00914)0.838.7:0.0084,(((Ult18783:0.0037,((Ult18780:0.00369,(U
lt18802:0.00369,((Ult18754:0.0,Ult18757:0.0,Ult18758:0.0,Ult18761:0.0,Ult
18762:0.0,Ult18763:0.0,Ult18764:0.0,Ult18767:0.0,Ult18772:0.0,Ult18774:0.
0,Ult18777:0.0,Ult18791:0.0,Ult18788:0.0,Ult18787:0.0,Ult18789:0.0,Ult187
94:0.0,Ult19284:0.0):5.5E-
4,(Ult18765:0.0037,(Ult18776:0.00383,Ult18755:0.0038)0.584.3:5.5E-
4)0.295.4:5.5E-4)0.000.129:5.5E-4)0.357.2:5.5E-4)0.462.2:5.5E-
4,(Ult18751:0.0037,(Ult18253:0.01499,Ult18799:0.0037)0.778.10:5.4E-
4)0.852.12:0.00369)0.000.130:5.5E-4)0.234.3:5.5E-
4,Ult18750:0.00368)0.900.6:5.5E-
4,(Ult18770:0.02883,Ult18778:0.00571)0.756.12:0.00548)0.813.2:0.00377)0.7
71.12:5.4E-
4)0.728.4:0.00359)0.867.10:0.00972)0.916.15:0.01451)0.819.8:0.00704)0.819
.9:0.01307)0.966.8:0.03277,((Ult18214:0.01119,(Ult18213:0.03816,((Ult1821
1:0.0,Ult18212:0.0):5.5E-4,(Ult18210:0.00716,UltLach8:5.5E-
4)0.932.7:0.01104)0.861.10:5.5E-4)0.890.12:5.4E-
4)0.990.7:0.03594,((Ult18237:0.00369,UltRu299:5.5E-4)0.725.6:5.3E-
4,(Ult18238:5.5E-
4,((((((((Ult17498:0.01507,((Ult18163:0.00363,(Ult19121:0.0,Ult19126:0.0,
Ult19127:0.0):5.5E-4)0.958.8:5.5E-4,(Ult19120:0.00569,(Ult19119:5.4E-

4,(Ult19122:0.00368,((Ult17681:0.0,Ult19123:0.0,Ult19124:0.0,UltClo22:0.0
,Ult19125:0.0,Ult19128:0.0):5.5E-4,Ult19129:0.00367)0.275.2:5.4E-
4)0.845.6:0.00368)0.898.17:0.01031)0.884.7:0.0103)0.865.8:0.00728)0.915.1
1:0.01125,(Ult18325:0.01988,(Ult18830:0.03394,((((Ult18819:0.0,Ult21355:0
.0):5.5E-4,(Ult18818:0.00362,Ult18820:5.5E-
4)0.951.10:0.01875)0.991.7:0.03324,(Ult18821:0.00776,Ult18822:0.01512)0.7
39.10:0.00653)0.988.8:0.0323,(Ult18275:0.03032,(Ult19480:5.4E-
4,(Ult19479:5.3E-4,((Ult19477:0.0,Ult19478:0.0):5.5E-
4,Ult19476:0.00363)0.918.12:0.00741)0.848.8:0.00364)0.925.19:5.3E-
4)0.712.1:0.00556)0.780.8:0.00735)0.882.6:0.01659)0.912.8:0.01407)0.894.8
:0.00758,(((Ult18962:5.4E-
4,(Ult18961:0.01383,(Ult18959:0.00366,Ult18960:5.5E-
4)0.976.5:0.03129)0.902.11:0.01379)0.924.11:0.01905,UltClo20:0.00775)0.96
3.10:0.01938,((((Ult18956:0.0,Ult19131:0.0):5.5E-
4,Ult18966:0.00369)0.912.9:5.4E-
4,(Ult18967:0.01114,(Ult18958:0.00742,((Ult18957:0.00369,Ult18975:0.00369
)0.688.1:5.5E-
4,((Ult18964:0.0,Ult18969:0.0,Ult18968:0.0,Ult18970:0.0,Ult18971:0.0,Ult1
8976:0.0,Ult18977:0.0,Ult19130:0.0,Ult19133:0.0):5.5E-
4,(Ult19134:0.0037,Ult19132:0.00369)0.690.6:5.5E-4)0.000.131:5.5E-
4)0.918.13:5.5E-4)0.613.3:5.4E-
4)0.831.7:0.00367)0.866.9:0.00752,(Ult21301:0.01521,Ult18965:0.01129)0.86
5.9:0.00759)0.873.15:0.00763)0.845.7:0.00773)0.760.10:0.00321,((Ult20924:
0.02217,(((Ult20922:0.0,Ult20923:0.0):0.00668,Ult20926:0.0157)0.720.4:0.0
0362,(Ult21443:0.03229,Ult20928:0.01479)0.880.9:0.01097)0.959.7:0.01574)0
.869.8:5.4E-
4,(Ult20925:0.01492,Ult20927:0.00365)0.912.10:0.00756)0.949.11:0.01554)0.
863.10:0.01483,Ult19481:0.06747)0.286.2:0.01036,Ult20512:0.03139)0.945.9:
0.0195,(Ult20231:0.01454,(Ult20659:0.00369,((HumanGu9:0.0,Ult20657:0.0,Ul
t20658:0.0):5.5E-4,Ult20660:0.00369)0.874.8:5.5E-
4)0.935.9:0.01808)0.841.6:0.00993)0.071.2:0.01155,(((Ult20128:0.00343,(((
(((((((Ult20942:0.00369,(Ult20947:0.01158,Ult22681:0.00741)0.411.1:5.5E-
4)0.482.2:5.5E-4,Ult20951:0.00369)0.000.132:5.5E-
4,Ult20811:0.00369)0.133.1:5.5E-
4,(UltB7196:0.0,Ult20952:0.0):0.0037)0.000.133:5.5E-
4,Ult19827:0.00741)0.499.1:5.5E-
4,(Ult20938:0.0,Ult20943:0.0,RmcGnavu:0.0,Ult20944:0.0,Ult20945:0.0,Ult20
946:0.0,Ult20948:0.0,Ult20950:0.0,Ult20953:0.0):5.5E-4)0.622.2:5.5E-
4,(Ult20941:0.0548,Ult20940:0.00714)0.890.13:5.5E-4)0.176.5:5.5E-
4,Ult20939:0.00361)0.931.9:0.00169,Ult20954:0.01089)0.924.12:0.00247,((Ul
t20759:0.05951,((((Ult20783:0.00369,(Ult20730:0.00369,Ult20782:5.5E-
4)0.934.8:5.5E-
4)0.985.6:0.01945,(UltClo48:0.0084,(((Ult20890:0.05154,(Ult18110:5.5E-
4,((Ult20720:0.0,Ult20721:0.0,Ult20722:0.0,Ult20784:0.0):5.5E-
4,(Ult20718:0.03082,Ult20719:0.01114)0.980.2:5.5E-4)0.813.3:5.3E-
4)0.954.8:5.4E-
4)0.874.9:0.00761,Ult20859:0.01155)0.895.10:0.01085,((((Ult20866:0.00369,
(((Ult20762:0.09985,(Ult20865:0.00369,((Ult20763:0.0,Ult20861:0.0):5.5E-
4,Ult20860:0.00369)0.027.1:5.5E-4)0.999.6:5.4E-
4)0.854.7:0.00367,(Ult20775:0.00701,(Ult20857:0.01501,(Ult20856:0.00369,C
stBact3:5.5E-4)0.948.9:5.3E-
4)0.911.11:0.002)0.908.6:0.00219)0.809.6:0.00383,Ult20868:0.00742)0.403.1
:5.4E-4)0.415.4:5.5E-4,((BacNL275:5.5E-4,(BacNL274:5.5E-
4,((UltB7208:0.0,Ult20754:0.0,Ult20734:0.0,CsrScin3:0.0,Ult20891:0.0):5.5

E-4,((CsrScind:0.00369,CsrScin2:0.0074)0.662.2:5.5E-
4,(Ult20755:0.00369,Ult20737:0.00369)0.880.10:5.5E-4)0.000.134:5.5E-
4)0.739.11:5.5E-4)0.733.4:5.4E-
4)0.863.11:0.00364,(((Ult19821:0.00371,Ult20735:0.00371)0.779.6:0.00526,(
Ult17857:0.0122,Ult18714:0.01599)0.860.5:0.01424)0.863.12:0.00993,Ult2076
0:0.01926)0.682.2:0.00373)0.162.2:5.4E-4)0.928.8:5.4E-
4,Ult20862:0.00731)0.791.7:0.00443,(((Ult20726:0.00369,(Ult20727:5.5E-
4,((Ult20723:0.00369,(Ult20728:0.00741,(Ult20724:0.04274,((Ult20858:0.048
15,Ult20776:5.5E-4)0.816.6:0.00373,Ult20725:0.00369)0.636.1:5.5E-
4)0.820.2:0.00369)0.610.1:5.4E-4)0.207.1:5.5E-
4,(Ult20447:0.0,Ult20729:0.0,Ult20731:0.0,Ult20889:0.0):5.5E-
4)0.471.4:5.5E-4)0.318.4:5.5E-4)0.483.2:5.4E-
4,((Ult20183:0.0037,(UltClo49:5.5E-
4,(Ult20732:0.0,Ult20761:0.0,Ult20888:0.0):5.5E-4)0.939.6:5.5E-
4)0.893.14:0.00723,(Ult20778:5.4E-
4,(Ult20766:0.03876,((((Ult20733:0.0,Ult20765:0.0,Ult20770:0.0,Ult21360:0
.0):5.5E-
4,(Ult20767:0.00368,(Ult20772:0.0112,Ult20758:0.00369)0.374.2:5.5E-
4)0.000.135:5.5E-4)0.000.136:5.5E-
4,(Ult20756:0.00369,(Ult20764:0.01116,Ult20769:0.00369)0.138.3:5.5E-
4)0.785.9:5.5E-4)0.895.11:5.5E-
4,(Ult20771:0.00368,Ult20777:0.01136)0.624.2:5.5E-4)1.000.57:5.4E-
4)0.824.9:0.00362)0.956.6:0.0111)0.906.9:5.3E-
4)0.864.10:0.0037,(Ult20773:0.0037,Ult20774:0.00741)0.759.6:5.4E-
4)0.645.3:0.00307)0.915.12:0.0118)0.807.3:5.4E-
4)0.301.1:0.00396)0.358.3:5.4E-
4,((Ult20757:0.0,Ult20780:0.0):0.00758,((Ult19761:0.0,Ult20779:0.0,Ult208
64:0.0):5.5E-
4,Ult20863:0.01518)0.857.10:0.00756)0.874.10:0.01018)0.850.6:0.00757,(((U
lt19283:0.02271,((Ult20184:0.0,Ult21445:0.0):0.00369,Ult20955:5.5E-
4)0.948.10:5.5E-4)0.986.2:5.5E-
4,(Ult20852:0.00993,Ult20851:0.01013)0.880.11:0.01402)0.172.5:0.00854,(Ba
cNL276:5.4E-
4,(((Ult20850:0.0037,((((((((((Ult20749:0.0,Ult20750:0.0,Ult20751:0.0,Ul
t20752:0.0,Ult20753:0.0,Ult20786:0.0,Ult20787:0.0,Ult20788:0.0,Ult20791:0
.0,Ult20812:0.0,Ult20814:0.0,Ult20816:0.0,Ult20818:0.0,Ult20820:0.0,Ult20
822:0.0,Ult20823:0.0,HumIntes:0.0,Ult20826:0.0,Ult20833:0.0,Ult20835:0.0,
Ult20845:0.0,Ult20847:0.0,Ult20848:0.0,Ult20849:0.0,Ult21420:0.0):5.5E-
4,((Ult20825:0.05142,((((Ult20817:5.5E-
4,Ult20824:0.02258)0.805.6:0.00376,((Ult21300:0.03512,(Ult18745:5.5E-
4,Ult18892:0.00746)0.933.11:0.01756)0.546.2:0.00922,(Ult20700:0.01116,(Ul
t20815:5.5E-4,Ult19468:0.00369)0.308.2:5.5E-4)0.921.12:5.3E-
4)0.901.13:0.01091)0.132.3:5.5E-
4,(Ult20830:0.03486,Ult20829:0.011)0.888.11:5.5E-4)0.535.2:5.5E-
4,(Ult20844:0.00245,Ult20846:0.00245)0.920.9:0.00245)0.272.2:5.5E-
4)0.000.137:5.5E-4,Ult20834:5.5E-4)0.421.1:5.5E-4)0.000.138:5.5E-
4,(Ult20322:0.01105,(Ult18741:0.02301,Ult20819:0.01523)0.555.2:0.00769)0.
876.6:5.4E-4)0.000.139:5.5E-4,Ult20828:0.00369)0.418.2:5.5E-
4,Ult20831:0.00369)0.011.1:5.5E-4,Ult20832:0.01114)0.418.3:5.5E-
4,Ult20813:0.00369)0.333.1:5.5E-4,Ult20821:5.5E-4)0.479.1:5.5E-
4,Ult20827:0.01856)0.589.2:5.3E-
4,Ult20843:0.00368)0.940.4:0.01161,Ult20853:0.01126)0.881.12:5.4E-
4)0.837.6:0.00377,Ult20855:5.5E-
4)0.748.5:0.00326,Ult20854:0.03547)0.795.9:0.00776)0.833.10:0.01065)0.823

.5:0.00747)0.750.3:0.00423)0.848.9:0.00658,(Ult20936:5.5E-
4,(Ult20937:0.00371,Ult20935:0.00369)0.231.2:5.5E-
4)0.935.10:0.00216)0.907.10:0.00194)0.862.11:0.00761,Ult20949:0.00762)0.7
45.3:0.00402)0.762.8:0.0039,Ult24090:0.04427)0.861.11:0.00732,(Ult18823:0
.01912,(Ult18824:0.00301,(Ult18383:0.02746,(Ult18389:0.00855,(((Ult18387:
0.0,Ult18388:0.0):5.5E-
4,Ult18391:0.00369)0.952.14:0.01404,(Ult18390:5.5E-
4,Ult18392:0.00742)0.772.9:0.00477)0.020.1:0.0037)0.799.8:0.00647)0.986.3
:0.02864)0.782.14:0.01635)0.759.7:0.00376)0.972.7:5.5E-
4)0.989.10:0.01831)0.824.10:0.00722)0.933.12:0.01689)0.321.1:0.00652)0.78
2.15:0.00626,UltRu371:0.02509)0.856.5:0.00945,Ult18406:0.01519)0.874.11:0
.00758,((((Ult21474:0.03044,Ult19634:0.02733)0.132.4:5.4E-
4,(Ult21249:0.00951,((((((Ult18559:0.0,Ult21248:0.0,Ult21259:0.0,Ult21260
:0.0,Ult21252:0.0,Ult21253:0.0,Ult21254:0.0):5.5E-
4,(Ult21255:0.00746,(Ult21245:0.00209,Ult21250:0.00694)0.905.9:0.00207)0.
726.4:5.4E-4)0.953.9:5.5E-4,((Ult21246:0.0074,Ult21244:5.5E-
4)0.776.6:0.0035,((Ult21242:0.06398,Ult21241:5.5E-
4)0.805.7:0.00396,(Ult21240:0.00367,Ult21243:5.4E-
4)0.771.13:0.00346)0.931.10:0.01149)0.879.7:0.00766)0.844.12:0.00713,Ult2
1251:0.01487)0.109.2:0.00355,Ult18558:0.01866)0.877.9:5.5E-
4,(Ult17839:0.011,(Ult17863:0.00715,(Ult21359:0.02692,((Ult21235:0.01143,
((Ult18887:0.08782,(Ult22380:0.02695,Ult21238:0.00372)0.846.7:5.4E-
4)0.939.7:0.01101,(Ult21239:0.00369,((Ult21234:0.0,Ult21233:0.0):5.5E-
4,Ult21232:0.00369)0.000.140:5.5E-4)0.940.5:5.4E-
4)0.662.3:0.00757)0.782.16:0.0038,(Ult21236:5.5E-
4,Ult21247:0.00368)0.345.1:5.5E-
4)0.770.7:0.00372)0.892.6:0.00784)0.924.13:0.01526)0.140.1:5.5E-
4)0.770.8:0.00681)0.000.141:0.00617)0.950.10:0.0189,(Ult19576:0.02271,(Ul
t17690:0.0311,Ult21262:0.04027)0.011.2:0.00186)0.922.9:0.01351)0.769.9:5.
5E-
4,(Ult18318:0.04928,(((Ult21479:0.02424,CsdBact3:0.06224)0.893.15:0.02419
,(Ult21564:0.02911,((Ult21561:0.01419,(Ult21562:0.08104,(Ult21363:0.02942
,Ult21560:0.01671)0.745.4:0.00937)0.738.4:0.01802)0.994.10:0.04653,((((Ul
t21556:0.02631,(UltRu378:0.01916,Ult21555:0.017)0.946.8:0.02679)0.878.5:0
.01914,(Ult21912:0.04859,Ult21557:0.04955)0.783.7:0.01391)0.536.1:0.00436
,(UltClo81:0.01218,Ult21563:0.03096)0.936.8:0.02643)0.107.4:0.01378,(Ult2
1551:5.5E-
4,((Ult21539:0.01553,Ult21550:0.00381)0.752.8:0.00377,((((BacNL302:0.0036
9,(((Ult21526:0.01556,(UltRu377:0.03238,((UncUn140:0.00368,UncUn141:5.5E-
4)0.999.7:0.07675,UltEub85:0.02065)0.216.2:0.01313)0.816.7:0.01268)0.942.
3:0.01839,(CsrNeopr:0.00369,(BacNL303:5.5E-
4,(Ult21525:0.00743,BacNL304:0.0112)0.797.10:5.5E-4)0.895.12:5.5E-
4)0.857.11:0.00884)0.920.10:0.01801,(BacNL300:0.00368,BacNL301:5.5E-
4)0.963.11:5.4E-4)0.802.6:0.00729)0.927.8:0.01408,((Ult21536:5.3E-
4,(Ult21522:0.02627,((UltFir26:0.00348,(Ult21521:0.0,Otu00077:0.0):0.0115
1)0.983.7:0.01942,(((((((Ult21548:0.00675,((Ult21544:0.01859,(Ult21543:5.
5E-4,Ult21545:0.00369)0.842.12:5.5E-
4)0.897.7:0.00738,((Ult21546:0.0,Ult21547:0.0):5.5E-4,(Ult21542:5.5E-
4,(Ult21541:0.0074,UltClo79:0.00369)0.230.4:5.2E-
4)0.907.11:0.00369)0.994.11:5.5E-
4)0.933.13:0.01743)0.958.9:0.02168,(Ult21519:0.0036,(Ult21517:0.02613,((U
lt21518:0.0,Ult21516:0.0):5.5E-4,Ult21520:0.00368)0.962.12:5.5E-
4)1.000.58:5.4E-
4)0.742.7:0.00739)0.749.12:0.00372,(Ult21523:0.01485,Ult21524:5.4E-

4)0.937.6:0.01096)0.801.8:0.00362,(Ult21478:5.5E-
4,((Ult21394:0.0,Ult21527:0.0,Ult21528:0.0,Ult21553:0.0):5.5E-
4,Ult21529:0.00369)0.759.8:5.5E-4)0.967.6:5.3E-
4)0.910.14:0.0019,Ult21530:0.00221)0.911.12:0.00672,(Ult21534:0.00558,((U
lt21549:5.5E-4,(Ult21540:0.00737,(Ult21535:5.5E-
4,(Ult23525:0.00739,Ult21538:0.00368)0.739.12:5.5E-4)0.924.14:5.4E-
4)0.948.11:0.00744)0.834.7:5.4E-4,(Ult21533:5.4E-
4,(Ult21532:0.01095,Ult21554:0.002)0.923.9:0.00195)0.844.13:0.00713)0.872
.15:0.00984)0.958.10:0.01842)0.758.8:5.4E-4,(Ult21531:5.5E-
4,(Ult23524:5.5E-4,UltClo78:0.00369)0.742.8:5.5E-4)0.970.8:5.4E-
4)0.763.11:0.00344)0.947.6:0.01814)0.609.3:5.4E-4)0.824.11:5.5E-
4,Ult21537:0.03947)0.918.14:0.01519)0.827.9:0.00735,BacEnr42:0.02199)0.83
3.11:0.00851,Ult21552:0.03142)0.630.3:0.0039)0.942.4:0.01431)0.959.8:0.02
432)0.766.7:0.01121)0.116.1:0.00762)0.886.14:0.01595)0.852.13:0.01302,(((
(Ult21156:0.0,Ult21157:0.0):5.5E-
4,Ult21158:0.00368)0.973.3:0.03596,(CstBact5:0.07927,(Ult21493:0.02776,((
Ult21490:0.00622,(Ult21491:0.03053,(Ult21492:5.5E-
4,Ult23863:0.00369)0.984.4:5.5E-
4)0.917.11:0.02101)0.155.2:0.02112,(Ult21489:0.01583,((EpuSpec6:0.00365,(
EpuSpec4:0.0075,EpuSpec5:0.00369)0.878.6:0.00759)0.898.18:0.00751,(EpuSpe
c2:5.5E-4,(EpuSpeci:0.00369,EpuSpec3:0.0037)0.686.3:5.4E-4)0.945.10:5.4E-
4)0.978.3:0.0271)0.959.9:0.03118)0.959.10:0.02647)0.714.4:0.00386)0.798.1
3:0.01838)0.926.11:0.02828,((((((UltB7772:0.04068,(UltClo63:0.07175,(((U
lt21484:0.0,Ult21485:0.0):0.01931,(Ult21480:5.5E-
4,((Ult21481:0.0,Ult21483:0.0):5.5E-
4,Ult21482:0.00369)0.911.13:0.00742)0.850.7:0.01127)0.962.13:0.02563,((Ba
cNL297:0.0,Ult21488:0.0):0.02599,((Ult21486:0.0,Ult23526:0.0):0.00377,Ult
21487:0.03132)0.592.1:0.01052)0.739.13:0.00633)0.436.2:0.01025)0.985.7:0.
05537)0.850.8:0.02565,UltB7359:0.14946)0.659.2:0.02232,(UltB6922:0.10302,
(((Ult12222:0.15454,UltB7349:0.15025)0.851.7:0.04377,((UltB6726:0.04923,(
UltB6718:0.0552,UltB7354:0.10336)0.906.10:0.05148)0.592.2:0.02658,UltB735
6:0.06564)0.888.12:0.04229)0.210.1:0.01358,(((((BfhggY12:0.12365,Ult19272
:0.03108)0.121.1:0.01175,(UltrSo69:0.56078,UltrSo70:0.26137)0.832.7:0.037
19)0.694.4:0.01232,(Pshggg12:0.06159,((Ult19261:0.04097,Ult19266:0.05078)
0.711.2:0.01151,((UltB7341:0.03374,(Ult19258:0.04107,(Ult19256:0.01532,(U
ltB7339:0.0367,UltB7342:0.00768)0.977.4:0.03888)0.914.10:0.0205)0.713.4:0
.00548)0.878.7:0.01781,((UltB5859:0.20894,Ult19275:0.02247)0.910.15:0.044
23,(UltB5723:0.0591,UltB5730:0.03244)0.992.6:0.08808)0.979.9:0.06226)0.22
9.2:0.00721)0.842.13:0.01577)0.919.12:0.02486)0.804.7:0.01375,((((((((((
((CuiiiYy2:0.00367,CbPB0000:5.5E-4)0.991.8:0.04345,((UltB7674:5.5E-
4,(Ult31824:0.08221,Ult31823:5.5E-
4)0.995.7:0.03622)1.000.59:0.07664,(((((((BacNL200:0.00367,(BacNL197:5.5E
-4,(BacNL199:5.5E-4,(BacNL196:0.00371,(BacNL198:5.5E-4,BacNL201:5.5E-
4)0.900.7:0.00766)0.410.1:5.5E-4)0.197.2:5.5E-4)0.196.2:5.5E-
4)0.980.3:0.0212,(BacNL193:5.5E-4,((BcrSpec4:0.0,BacNL194:0.0):5.5E-
4,BacNL192:5.5E-4)0.819.10:5.5E-
4)0.763.12:0.00366)0.781.9:0.00379,((UltB7666:0.0075,BacNL202:5.5E-
4)0.884.8:0.00761,(BacNL195:0.0072,Otu00815:0.01115)0.918.15:5.4E-
4)0.903.7:0.00774)0.899.7:0.01072,(UltrSo61:0.03361,UltB7678:0.02715)0.24
1.2:5.4E-
4)0.955.9:0.01465,UltB7676:0.00378)0.780.9:0.00356,UltB7675:0.00369)0.999
.8:5.3E-4,(UltB7668:0.02601,((UltB7679:0.01653,((UltB7669:5.5E-
4,(UltB7673:0.0,UltB7672:0.0):5.5E-4)0.902.12:5.3E-
4,(UltB7671:0.01097,Ult12368:0.11452)0.889.10:5.5E-

4)0.944.6:0.01827)0.912.11:0.01758,((BcrSpec5:0.0,PtmSulfu:0.0):0.00408,U
ltB7677:0.01514)0.876.7:0.01177)0.711.3:0.00499)0.426.3:0.01691)0.449.2:0
.0095)0.735.5:0.00618)0.451.2:0.0226,UltB7667:0.05868)0.936.9:0.02698,(((
UltB7491:0.04148,((((UltB7489:0.01975,UltB7490:0.04142)0.842.14:0.0187,Ul
tB7471:0.07773)0.877.10:0.01963,Bfhggg18:0.02906)0.811.8:0.01329,((((((((
(UltB7466:0.01896,(((UncUnc50:0.0,UncUnc51:0.0,UncUnc53:0.0,UncUnc52:0.0,
Otu00069:0.0):5.5E-4,DysGadei:0.00369)0.501.2:5.4E-
4,(((UltPor23:0.00696,(UltB7469:0.03469,(UltB7468:0.02262,UltB7467:0.0042
6)0.227.3:0.00801)0.873.16:0.01232)0.891.11:0.0121,((UltB7465:0.00369,(Ul
tB7464:0.0088,UncUnc57:0.03544)0.946.9:0.01885)0.772.10:0.00352,UltB7472:
0.00778)0.774.14:0.00383)0.506.2:0.00388,UltB7457:0.0311)0.860.6:5.4E-
4)0.896.7:0.01439)0.753.5:0.00698,UltB7463:0.02601)0.348.2:0.01144,Otu007
55:0.01163)0.892.7:0.0114,UltB7456:5.4E-
4)0.785.10:0.0031,(UltB7473:0.04612,((UltB7455:0.0,Otu00070:0.0):0.0081,(
(UltB7454:0.0,Tryy0089:0.0,Tryyy033:0.0,Tryyy092:0.0):5.5E-
4,(Try00025:0.0,Try00026:0.0):5.5E-
4)0.763.13:0.0031)0.809.7:0.00736)0.920.11:0.01573)0.759.9:0.00446,(UltB7
470:0.0,Otu00409:0.0):0.0271)0.789.6:0.00359,(Bfhggg16:0.07429,PahggY25:0
.0225)0.859.7:0.00845)0.941.6:0.01494,((UncUnc58:0.01149,UncUnc59:0.01141
)0.774.15:0.00378,(((UltB7462:5.5E-4,(UltB7460:0.01125,UltB7461:5.4E-
4)0.960.8:0.01515)0.827.10:0.00725,(((BacNL179:0.00368,(BacNL182:0.0,BacN
L185:0.0,DysMossi:0.0):5.5E-4)0.885.8:5.4E-4,((BacNL180:5.4E-
4,BacNL181:0.00742)0.428.2:0.00367,((BacNL183:0.0,BacNL184:0.0):5.5E-
4,BacNL178:5.5E-4)1.000.60:5.5E-
4)0.845.8:0.00362)0.986.4:0.02694,(UltB7458:0.01108,((UncUnc54:0.0,UncUnc
56:0.0,UncUnc55:0.0):0.00928,UltB7459:0.0294)0.323.2:0.00406)0.869.9:0.01
176)0.747.9:0.00387)0.912.12:0.01117,(UltB7474:5.3E-
4,Otu00218:0.00725)0.997.10:0.0311)0.886.15:5.3E-4)0.778.11:5.5E-
4)0.891.12:0.02267,UltB7492:0.09252)0.921.13:0.03037)0.071.3:7.5E-
4)0.985.8:0.03406,(((UltB7478:0.00369,Otu01295:5.5E-
4)0.965.10:0.03646,(((Otu01292:0.00767,(UltB7475:0.00885,UltB7476:0.01407
)0.941.7:0.01562)0.742.9:0.00393,UncUnc60:0.00882)0.787.7:0.00819,UltB747
7:0.00949)0.843.4:0.02205)0.995.8:0.0611,(UltB7387:0.04228,(PahggY23:0.02
626,UltB7386:0.10251)0.431.6:0.02214)0.990.8:0.0598)0.451.3:5.5E-
4)0.898.19:0.01557,((UltPor27:0.00387,(UltPor25:0.00528,(UltB7481:0.03517
,(UncUnc61:0.00372,UncUnc62:0.00372)0.974.5:0.03521)0.847.13:0.01596)0.99
2.7:0.03829)0.780.10:0.00334,(UltPor26:0.01122,((UltB7482:0.03113,Bfhggg1
9:0.01857)0.862.12:0.01291,(((UltB7486:0.0,UncUnc63:0.0,UncUnc64:0.0):0.0
2185,(DysCapno:0.00378,UltB7488:0.00744)0.889.11:0.01267)0.770.9:0.00397,
((UltB7480:0.01148,(UltPor24:0.00778,(UltB7479:0.00392,Pshggg21:0.0277)0.
829.7:0.00723)0.975.4:0.02808)0.958.11:0.02379,(((UltB7484:5.5E-
4,(UltB7485:0.0037,UltB7483:0.00369)0.603.4:5.5E-4)0.931.11:5.4E-
4,(Otu00420:5.4E-
4,UltPor28:0.01927)0.910.16:0.00741)0.926.12:0.01229,UltB7487:0.02849)0.7
05.4:0.00266)0.449.3:0.00815)0.907.12:0.01158)0.769.10:0.00383)0.872.16:0
.00804)0.760.11:0.00427)0.840.10:0.01088)0.965.11:0.03152,(((UltB7450:0.0
3296,(Bfhggg11:0.03999,P0114483:0.03988)0.986.5:0.05067)0.882.7:0.01952,(
(((Z0114520:5.5E-4,(UltB6233:5.4E-
4,Z0114490:0.01497)0.995.9:0.03071)0.737.8:0.00944,(UltB6234:0.03283,Sgff
ff07:0.07866)0.948.12:0.03762)0.989.11:0.07258,(UltB6235:5.4E-
4,Z0114492:0.01507)0.906.11:0.02613)0.976.6:0.04541,(((SwnFeca4:0.04229,(
BcrPropi:0.00186,BtrBacte:0.07941)0.934.9:0.02797)0.979.10:0.04041,(IroBa
c14:0.03532,(Pshggg10:0.02063,((UltB6247:0.00413,UltB6246:0.00333)0.703.4
:0.00342,((BacNLA85:0.00366,((BacNLA82:0.0,BcrGrami:0.0,UltB6245:0.0):5.5

E-4,BacNLA83:0.00368)0.743.10:5.5E-4)0.947.7:5.5E-
4,(BacNLA81:0.0104,BacNLA84:0.00549)0.793.10:0.01467)0.972.8:0.02797)0.87
8.8:0.02607)0.925.20:0.02281)0.707.2:0.00281)0.918.16:0.02621,(((BfhgggY0
:0.0313,(PshgggY8:0.06165,((UltB6230:0.0,UltB6231:0.0):0.01536,UltB6232:0
.02923)0.879.8:0.02893)0.922.10:0.03489)0.355.2:0.00913,(UltB6244:0.01411
,(((((UltB6228:0.00558,((Otu00015:5.5E-
4,Otu01368:0.00336)0.952.15:0.03248,(UltB6227:0.05478,UltB6226:0.05837)0.
807.4:0.01342)0.899.8:0.01982)0.973.4:0.03052,(UltB6225:0.02242,(UncUnc49
:0.02297,UltB6224:0.01629)0.995.10:0.06724)0.904.9:0.02098)0.831.8:0.0132
1,(UltB6229:0.03411,UltB6237:0.01066)0.609.4:0.00987)0.885.9:0.01037,((Ul
tB6238:0.00772,((PshgggY9:0.0,PshggYy8:0.0):0.00744,UltB6242:0.00373)0.84
3.5:0.00732)0.810.6:0.01119,UltB6243:0.01136)0.821.6:5.4E-
4)0.803.6:0.00717,UltB6240:0.03572)0.921.14:0.01316)0.878.9:0.01628)0.701
.2:0.01221,(UltB6241:0.02513,(((((BacNLA91:5.5E-
4,(BacNLA88:0.00367,((((UltB6251:0.00369,((BacNLA87:0.0,BcrCellu:0.0,BcrS
peci:0.0,BcrCell2:0.0,UltB6253:0.0,BacNLA93:0.0,BcrSpec2:0.0,UltB6254:0.0
,UltB6385:0.0,UltB6400:0.0):5.5E-
4,(UltB6252:0.01123,BacNLA90:0.00424)0.180.2:5.5E-4)0.000.142:5.5E-
4)0.225.2:5.5E-4,BacNLA92:5.5E-4)0.143.2:5.5E-4,BacNLA94:5.5E-
4)0.471.5:5.5E-4,BacNLA89:5.5E-4)0.479.2:5.5E-4)0.335.3:5.4E-
4)0.975.5:0.01695,((((((UltB6392:0.00741,(UltB6360:5.5E-4,UltB6363:5.5E-
4)0.394.2:5.5E-
4)0.794.7:0.00371,((UltB6361:0.0,UltB6368:0.0):0.00369,(UltB6362:0.0,BcrE
gger:0.0,BcrEgge2:0.0):5.5E-
4)0.791.8:0.0037)0.779.7:0.00364,(BacNL176:5.5E-
4,BacNL177:0.00364)1.000.61:0.03085)0.762.9:0.00389,((UltB6248:5.5E-
4,BcrSterc:5.5E-4)0.901.14:0.00747,(((UltB6011:0.0,UltB6365:0.0):5.5E-
4,(UltB6364:0.00369,UltB6391:0.02271)0.986.6:5.5E-
4)0.776.7:0.00371,((((((UltB6387:0.07353,UltB6415:0.01119)0.941.8:5.5E-
4,UltB6418:0.01114)0.508.1:5.5E-4,UltB6389:5.5E-4)0.493.2:5.5E-
4,(UltB6388:0.0,UltB6390:0.0):5.5E-4)0.997.11:5.3E-4,((((BacNL175:5.5E-
4,((UltB6218:0.0,BacNL165:0.0,BacNL171:0.0,UltB6409:0.0,UltB6410:0.0,UltB
6414:0.0,UltB6416:0.0,UltB6424:0.0,BcrUnifo:0.0,Ult19260:0.0):5.5E-
4,(UltB6425:0.00366,(((((UltB6405:0.01885,UltB6269:0.00741)0.682.3:5.5E-
4,(((UltB6107:0.0037,UltB6419:0.01481)0.874.12:5.3E-
4,Ult19271:0.00369)0.909.5:5.5E-
4,(UltB6024:0.0,UltB6109:0.0,UltB6263:0.0,UltB6367:0.0,UltB6386:0.0,BacNL
164:0.0,BacNL172:0.0,UltB6420:0.0,UltB6421:0.0,UltB6422:0.0):5.5E-
4)0.000.143:5.5E-4)0.727.5:5.5E-4,UltB6108:0.00369)0.792.11:5.5E-
4,UltB6427:0.08273)0.201.1:5.4E-
4,UltB6260:0.00739)0.387.1:0.00368)1.000.62:5.3E-4)0.761.9:5.5E-
4)0.804.8:5.5E-4,BacNL173:5.5E-4)0.854.8:5.5E-4,((BacNL168:5.5E-
4,(UltB6428:0.00369,((UltB6294:0.01129,UltB7558:0.0151)0.873.17:0.00347,(
UltB6217:0.00767,(UltB6366:0.01127,UltB7332:0.0038)0.956.7:0.01888)0.821.
7:0.01126)1.000.63:5.4E-4)0.935.11:5.5E-4)0.835.10:5.4E-
4,(UltB6423:0.05189,(BacNL174:0.00376,UltB6426:0.04331)0.794.8:0.00353)1.
000.64:5.4E-4)0.787.8:5.4E-4)0.771.14:5.5E-4,((BacNL167:5.5E-
4,BacNL166:5.5E-4)0.748.6:5.5E-4,(BacNL169:5.5E-
4,BacNL170:0.0036)0.425.2:5.4E-
4)0.853.7:0.00354)0.868.8:0.00348)0.781.10:0.00337,(UltB6429:0.01503,UltB
6413:5.5E-
4)0.182.1:0.00371)0.792.12:0.00363)0.775.5:0.00352)0.873.18:0.00709)0.911
.14:0.01319,((UltB6411:0.01519,(UltB6412:0.00774,UltB6432:0.01958)0.763.1
4:0.0036)0.782.17:0.00392,(UltB6384:5.3E-

4,(((UltB6378:0.00748,(UltB6093:5.5E-
4,((((UltB6381:0.05179,UltB6092:0.01899)0.921.15:5.5E-
4,(UltB6408:0.00369,Ult19270:0.00741)0.000.144:5.5E-4)0.936.10:5.5E-
4,(UltB6369:0.0,UltB6370:0.0,UltB6371:0.0,UltB6372:0.0,UltB6376:0.0,UltB6
380:0.0,BcrSter4:0.0,BcrSter5:0.0,UltB7333:0.0):5.5E-4)0.000.145:5.5E-
4,(((BcrSter2:0.0,UltB6382:0.0):5.5E-
4,BcrSter3:0.00385)0.835.11:0.00386,(UltB6377:0.00369,UltB6091:0.00369)0.
000.146:5.5E-4)0.469.4:5.5E-4)0.348.3:5.5E-4)0.545.3:5.5E-
4)0.916.16:0.00199,UltB6379:0.01081)0.905.10:0.00209,UltB6296:0.02006)0.8
87.8:0.00808)0.878.10:0.00813)0.670.1:0.00263)0.883.7:0.00996,((HmZZZZ02:
0.01977,((Hmyy0012:5.5E-
4,Hmyy0015:0.00369)0.803.7:0.00228,((HmZZZZ00:0.0,Hmyy0013:0.0,Hmyyy057:0
.0):5.3E-
4,Hmyy0014:0.01125)0.889.12:0.01014)0.830.9:0.00984)0.964.8:0.01948,(UltB
6373:0.0049,BcrGalli:0.01475)0.859.8:0.01456)0.744.7:5.4E-
4)0.779.8:0.00743,((BcrIntes:0.0,BacNLA86:0.0,UltB6406:0.0):5.5E-
4,(UltB6250:0.00743,UltB6249:0.00742)0.736.6:5.4E-4)0.621.3:5.5E-
4)0.385.4:5.4E-
4)0.202.3:0.00363,UltB7346:0.00758)0.858.9:0.00832,((((UltB6335:0.00369,U
ltB6319:0.00741)0.661.3:5.5E-
4,((UltB6048:0.00369,UltB6330:0.00369)0.904.10:5.5E-
4,(UltB6010:0.0,UltB6220:0.0,UltB6264:0.0,UltB6267:0.0,UltB6308:0.0,UltB6
329:0.0,BcrCacca:0.0,UltB6331:0.0,UltB6332:0.0,UltB6333:0.0,UltB6336:0.0,
UltB6337:0.0,UltB6338:0.0,UltB6339:0.0,UltB7343:0.0,UltB7345:0.0):5.5E-
4)0.000.147:5.5E-4)0.940.6:5.5E-
4,(Ult20163:0.0188,UltB6334:0.00369)0.596.1:5.5E-4)0.844.14:5.4E-
4,((((((((UltB6345:0.0,UltB6346:0.0):5.3E-4,((((BacNL113:5.5E-
4,((UltB6313:0.00372,(((UltB6265:0.0,UltB6268:0.0,BacNL111:0.0,BacNL112:0
.0,BcrOvat2:0.0,BcrOvatu:0.0,BcrOvat3:0.0,UltB6306:0.0,BacNL114:0.0,UltB6
320:0.0,UltB6325:0.0,UltB6327:0.0,BacNL116:0.0,BacNL117:0.0):5.5E-
4,(UltB6323:0.00369,((Ult19263:0.00369,(UltB6324:0.00369,BacNL115:0.01004
)0.420.3:5.5E-4)0.357.3:5.5E-4,UltB6311:0.00369)0.411.2:5.5E-
4)0.430.3:5.5E-4)0.000.148:5.5E-4,UltB6326:0.00369)0.275.3:5.5E-
4)0.253.1:5.5E-4,UltB6307:0.01492)0.405.3:5.5E-4)0.847.14:5.4E-
4,UltB6321:5.5E-
4)0.921.16:0.00247,UltB6302:0.00246)0.921.17:0.00245,((UltB5983:0.0,UltB6
304:0.0,UltB6310:0.0):0.00372,((UltB6351:0.00369,UltB6309:0.00369)0.851.8
:5.5E-
4,((UltB6303:0.0,UltB6305:0.0,UltB6315:0.0,UltB6316:0.0,UltB7344:0.0):5.5
E-4,(UltB6301:0.00369,(UltB6312:0.0037,UltB6314:0.0037)0.389.2:5.5E-
4)0.864.11:5.5E-4)0.000.149:5.5E-4)0.298.4:5.5E-4)0.785.11:5.2E-
4)0.655.2:0.00369)0.907.13:0.00685,UltB6291:0.00219)0.894.9:0.00195,(((Ul
tB6328:5.3E-4,((BacNL132:0.00362,(BacNL145:5.5E-4,(BacNL134:5.5E-
4,BacNL140:5.5E-4)0.912.13:0.00364)0.753.6:5.5E-4)0.557.1:5.5E-
4,((UltB6353:0.00369,BacNL151:0.0037)0.792.13:0.00367,(((BacNL147:0.00361
,(BacNL163:0.00366,BacNL156:5.5E-4)0.812.2:5.5E-
4)0.859.9:0.00358,(BacNL130:5.5E-4,(BacNL129:5.5E-4,(BacNL153:5.5E-
4,((((UltB6358:0.00369,(BacNL149:0.00369,(UltB6357:0.0074,((UltB5986:0.0,
BacNL119:0.0,BacNL121:0.0,BacNL122:0.0,BacNL124:0.0,UltB6354:0.0,UltB6356
:0.0,BacNL127:0.0,BacNL133:0.0,BacNL141:0.0,BacNL142:0.0,BacNL146:0.0,Bac
NL148:0.0,BacNL152:0.0,BacNL158:0.0,UltB6359:0.0):5.5E-4,BacNL118:5.5E-
4)0.000.150:5.5E-4)0.301.2:5.5E-4)0.000.151:5.5E-4)0.545.4:5.5E-
4,BacNL154:5.5E-4)0.441.3:5.1E-4,BacNL136:5.5E-4)0.536.2:5.3E-
4,(UltB6300:0.00369,((((((((BacNL155:0.01475,(BacNL137:0.00725,BacNL161:

0.00371)0.476.4:0.00362)0.887.9:5.3E-4,(BacNL135:5.5E-4,BacNL162:5.5E-
4)0.668.5:5.4E-4)0.920.12:0.00354,(BacNL139:5.5E-4,(BacNL120:5.5E-
4,(UltB6355:0.00369,BacNL143:5.5E-4)0.790.2:5.5E-4)0.750.4:5.5E-
4)0.988.9:5.5E-4)0.797.11:5.5E-4,BacNL128:0.00363)0.427.2:5.5E-
4,(BacNL123:5.4E-4,BacNL159:0.00735)0.831.9:0.00365)0.204.2:5.5E-
4,BacNL125:0.01491)0.370.2:5.5E-4,BacNL126:5.5E-4)0.078.2:5.4E-
4,(BacNL131:5.5E-4,(BacNL138:5.5E-4,(BacNL157:5.5E-
4,BacNL144:0.00193)0.138.4:5.7E-4)0.468.2:6.8E-4)0.656.2:5.1E-
4)0.228.1:5.5E-4,BacNL160:0.00737)0.277.2:5.4E-4)0.477.1:5.5E-
4)0.642.3:5.4E-4)0.163.1:5.5E-4)0.208.2:5.5E-4)0.177.4:5.4E-
4)0.496.2:5.5E-
4,(BacNL150:0.0032,(UltB6257:0.03688,UltB6352:0.07737)0.760.12:0.00623)0.
788.7:0.00404)0.391.3:5.5E-4)0.866.10:5.5E-
4)0.780.11:0.00347)0.408.5:0.00355,((UltB6347:0.01824,((UltB6013:0.0,UltB
6049:0.0,BcrFineg:0.0,UltB7347:0.0):5.5E-
4,UltB6278:0.00368)0.962.14:0.00205)0.978.4:0.00177,((UltB6322:0.0,UltB63
49:0.0):5.5E-4,UltB6348:0.00369)0.961.7:5.5E-
4)0.831.10:0.00354)0.641.4:5.5E-4,(UltB6290:5.5E-
4,(BcrAcidi:0.0,UltB6340:0.0,UltB6341:0.0,UltB6343:0.0,UltB6344:0.0,UltB7
348:0.0):5.5E-4)0.633.2:5.4E-
4)0.798.14:0.00384)0.703.5:0.00384,(UltB5780:0.01518,UltB6350:0.00743)0.9
49.12:0.01508)1.000.65:5.4E-
4,(UltB6239:0.0538,((UltB6081:0.01125,((((((UltB6089:0.00369,UltB6104:0.0
1116)0.770.10:5.4E-4,(UltB6085:0.00369,(UltB6404:0.01883,UltB7366:5.5E-
4)0.829.8:0.00369)0.866.11:5.5E-4)0.000.152:5.5E-
4,(UltB6087:0.0,UltB6090:0.0,UltB6096:0.0,UltB6099:0.0,UltB6100:0.0,UltB6
101:0.0,UltB6102:0.0,UltB6103:0.0,UltB6106:0.0,UltB6397:0.0,Ult19262:0.0,
Ult19264:0.0,Ult19280:0.0,Ult24023:0.0):5.5E-4)0.000.153:5.5E-
4,UltB6219:5.5E-4)0.724.3:5.5E-
4,(UltB6088:0.00368,UltB6095:0.03905)0.999.9:5.5E-4)0.767.5:5.5E-
4,(UltB6097:0.0,UltB6098:0.0):5.5E-
4)0.783.8:0.00366)0.723.4:0.00342,((UltB6082:0.0,UltB6083:0.0):0.02364,((
UltB6261:0.00741,(UltB6018:0.00369,UltB6084:0.00369)0.659.3:5.5E-
4)0.883.8:0.00777,(UltB6075:5.5E-
4,(((BacNLA80:0.0,UltB6077:0.0,UltB6105:0.0):5.5E-
4,UltB6074:0.04009)0.258.2:5.5E-
4,(UltB6076:0.00235,UltB6073:0.03726)1.000.66:0.00137)0.833.12:0.0037)0.8
42.15:0.0072)0.720.5:0.00383)0.665.3:0.00363)0.893.16:0.01134)0.956.8:0.0
2336)0.802.7:0.00361,((((BacNL100:5.5E-
4,(((UltB6279:0.01134,UltB6281:0.16172)0.994.12:0.05822,(((((UltB6266:0.0
0741,(((BcrNord2:0.0,BcrNordi:0.0,UltB6276:0.0):0.01505,(BacNL108:5.5E-
4,BcrSalye:0.00369)0.910.17:5.4E-
4)0.957.7:0.01519,(((((UltB6272:0.0037,BacNLA96:5.5E-4)0.000.154:5.4E-
4,UltB6399:0.00369)0.000.155:5.5E-4,UltB6298:0.00369)0.500.1:5.2E-
4,(UltB6012:0.0,UltB6047:0.0,UltB6055:0.0,UltB6271:0.0,BacNLA95:0.0,UltB6
255:0.0,UltB6256:0.0,BacNLA97:0.0,BacNLA99:0.0,BacNL101:0.0,BcrThet2:0.0,
UltB6259:0.0,BcrSpec3:0.0,UltB6277:0.0,UltB6292:0.0,UltB6293:0.0,UltB6297
:0.0,UltB6383:0.0,UltB6398:0.0):5.5E-4)0.488.3:5.5E-4,(UltB6258:5.5E-
4,UltB6317:0.00372)0.855.8:0.00371)0.026:5.5E-4)0.000.156:5.2E-
4)0.389.3:5.5E-4,UltB5984:0.00369)0.609.5:5.5E-
4,(BacNL104:0.0,BacNL105:0.0):5.5E-4)0.551.1:5.5E-
4,(BcrTheta:0.00741,BacNL102:5.5E-4)0.834.8:0.00368)0.226.1:5.4E-
4,BacNLA98:0.00368)0.078.3:5.5E-4)0.074.1:5.5E-
4,BacNL103:0.00367)0.796.4:5.4E-

4)0.801.9:0.00364,UltB6275:0.00374)0.772.11:0.00359,(BcrFrag2:0.00751,(Ul
tB6280:0.00394,((((UltB6284:0.0,UltB6285:0.0,UltB6287:0.0):5.5E-
4,(UltB6289:5.5E-
4,(BacNL109:0.0,BacNL110:0.0,BcrFragi:0.0,UltB6282:0.0,UltB6288:0.0):5.5E
-4)0.416.5:5.5E-4)0.785.12:5.5E-4,UltB6286:0.00363)0.736.7:5.5E-
4,((BacIc122:0.02254,UltB6283:5.5E-
4)0.838.8:0.00363,BacIc125:0.00364)0.200.1:5.5E-
4)0.879.9:0.00161)0.913.10:0.00363)0.799.9:0.00487)0.771.15:0.00506,UltB6
274:0.06725)0.779.9:0.00443)0.762.10:0.00491,((UltB6222:0.00364,(UltB6295
:5.5E-4,(GutMeta5:0.00365,UltB6221:0.00735)0.772.12:5.5E-4)0.810.7:5.5E-
4)0.875.9:0.01283,(UltB6318:0.03035,(BcrRetic:5.5E-4,UltB6270:5.5E-
4)0.986.7:0.034)0.400.2:0.00409)0.876.8:0.0141)0.830.10:0.00806,UltB6299:
0.01106)0.313.1:0.00436)0.875.10:0.01085)0.758.9:0.00947,(UltB6236:5.5E-
4,Otu00761:0.00367)1.000.67:0.09131)0.831.11:0.00929)0.854.9:0.01282)0.51
0.1:0.01076)0.813.4:0.00811)0.893.17:0.01432)0.870.7:0.01505,(UltB6273:0.
04359,(UltB7452:0.08104,UltB7451:0.00914)0.990.9:0.06337)0.877.11:0.02674
)0.135.1:0.00674)0.972.9:5.4E-
4,(UltB7546:0.03447,(UltB7363:0.00742,UltB7362:0.05446)0.967.7:0.03986)0.
735.6:0.02401)0.865.10:0.01102,(((((UncUn446:5.5E-
4,UncUnc73:0.00368)0.994.13:0.06386,(CanVesti:0.0206,UltB7572:0.04168)0.9
22.11:0.03338)0.915.13:0.03147,(Otu00922:0.06559,(PahggY24:0.04351,(UltB7
381:0.04241,UltB7382:0.06478)0.234.4:0.01481)0.088.2:0.03046)0.977.5:0.04
29)0.852.14:0.02219,((((((((((Otu00387:0.0183,(Kty00019:0.0397,Otu00487:
0.02581)0.388.3:0.00528)0.938.9:0.018,(Otu00125:0.00501,Otu00175:0.0139)0
.949.13:0.01543)0.908.7:5.4E-
4,Otu00670:0.0205)0.711.4:0.0093,(((UltPor43:0.004,(UltB7600:0.008,UltB75
99:0.01931)0.375.1:0.00354)0.983.8:0.00112,(PahggY28:0.01949,UltB7601:0.0
0775)0.894.10:0.0024)0.972.10:0.01934,((UltB7589:0.00791,UltB7590:0.01474
)0.767.6:0.00381,(UltB7591:0.01132,Cuiiii15:0.04811)0.652.1:5.5E-
4)0.899.9:0.01161)0.795.10:0.00829)0.871.9:0.01066,(MsGtttG2:0.04736,Otu0
0601:0.04917)0.987.7:0.04182)0.357.4:0.00887,UltB7583:0.08753)0.746.8:0.0
0423,((PpmGingi:0.0346,PpmSpeci:0.01052)1.000.68:0.08605,(UltB7592:0.0449
1,UltB7575:0.10507)0.876.9:0.03671)0.872.17:0.02846)0.794.9:0.01141,(((Pp
mCanor:0.02975,((UltPor35:0.04417,(UltB7507:5.4E-
4,(UltB7506:0.00745,PpmBenno:5.5E-
4)0.904.11:0.01111)0.990.10:0.07125)0.964.9:0.05213,(PpmSomer:0.01182,((P
pmLevii:0.00363,UltPor33:5.4E-
4)0.758.10:0.01761,(UltPor34:0.04057,((UltPor32:0.04156,PpmAsacc:0.08587)
0.935.12:0.04286,(UltB7505:0.07826,(UltB7504:0.02306,(PpmCanis:0.02388,(P
pmEndod:0.02694,(UdnEubac:5.5E-4,PpmSpec2:5.5E-4)0.966.9:5.4E-
4)0.809.8:0.00893)0.769.11:0.01195)0.849.7:0.02505)0.465.1:0.02526)0.963.
12:0.04815)0.961.8:0.04112)0.853.8:0.01364)0.699.1:0.00234)0.856.6:0.0191
1)0.396.3:0.00997,PpmCangi:0.02969)0.991.9:0.05977,(((((UltPor31:0.023,Ul
tB7495:0.01169)0.850.9:0.00865,(UltB7503:0.01389,UltB7502:0.05661)0.759.1
0:0.00838)0.906.12:0.01541,((UltB7494:0.0,UltPor30:0.0,UltB7493:0.0):5.5E
-
4,UltPor29:0.00368)0.984.5:0.03052)0.851.9:0.01059,(UltB7501:0.03036,((Ul
tB7500:0.02111,UltB7499:0.0103)0.324.1:0.00935,(UltB7498:0.03528,(UltB749
6:5.5E-
4,UltB7497:0.00369)0.898.20:0.0176)0.947.8:0.03317)0.895.13:0.02676)0.608
.1:0.00585)0.815.7:0.01572,(UltB7318:0.19405,PpmCrevi:0.10587)0.006.1:0.0
2459)0.828.5:0.02392)0.780.12:0.01665)0.845.9:0.01351,UltB7562:0.03427)0.
833.13:0.01709,((((((UltB7552:0.00693,UltPor38:0.00823)0.945.11:0.0159,((
(PbdSpec2:0.01577,(UltB7571:0.0351,(UltPor40:0.01847,UltPor39:0.0162)0.74

4.8:0.0036)0.884.9:0.00855)0.928.9:5.3E-
4,UltPor37:0.01092)0.819.11:0.00766,((Pshggg23:0.01959,UltB7542:0.0113)0.
867.11:0.0066,((((UltB7549:0.00375,UltB7550:0.00747)0.879.10:0.00737,UltB
7547:0.0038)0.895.14:0.00748,((PbdGolds:0.0,UltB7548:0.0):5.5E-
4,UltParab:0.01121)0.847.15:5.4E-
4)0.889.13:0.01157,(((((UltB7559:0.00369,(UltB7357:0.0,PbdMerda:0.0):5.5E
-4)0.737.9:5.5E-4,(UltB7554:0.01491,UltB7212:0.01888)0.664.1:5.5E-
4)0.974.6:5.5E-
4,(UltB6033:0.03133,UltB6199:0.02727)0.961.9:0.02297)0.922.12:0.00196,(Ul
tB7560:0.00756,(PbdJohns:5.4E-
4,UltB7553:0.01127)0.923.10:0.00757)0.908.8:0.00201)0.875.11:0.00916,(Ult
B7557:0.0191,((SwnFeca5:0.0,UltB7555:0.0):0.00749,UltB7556:5.4E-
4)0.877.12:0.00778)0.867.12:0.00969)0.740.5:0.00907)0.749.13:0.01175)0.88
1.13:0.0064)0.755.6:0.00378)0.749.14:0.00804,UltPor36:0.0435)0.000.157:5.
4E-
4,UltB7551:0.01929)0.767.7:0.00515,(((((((KtYyyyy8:0.0,Kty00012:0.0,Ktyyy
002:0.0,Kty00013:0.0,Kty00015:0.0,Kty00016:0.0):5.5E-
4,Kty00014:0.00369)0.724.4:0.00279,(Otu00247:0.02039,Otu00135:0.02776)0.9
19.13:0.01589)0.931.12:0.01617,(Z0114522:0.00772,((UltB7577:0.0,Otu00161:
0.0):0.03137,(UltB7576:0.01908,Otu00288:5.3E-
4)0.770.11:0.01002)0.780.13:0.00523)0.664.2:0.00473)0.730.4:0.00424,(((Kt
y00018:0.00368,(Kty00017:0.0,Ktyy0040:0.0):5.5E-
4)0.974.7:0.04203,(UltB7582:0.03786,(Otu00119:0.01003,Otu00346:0.04718)0.
992.8:0.04842)0.909.6:0.02342)0.956.9:0.02573,((((UltB7596:0.02142,(TanFo
rsy:0.00381,(((Bfhggg23:0.01144,(((Pshggg24:5.5E-4,(P0114473:5.5E-
4,PahggY27:0.00366)0.999.10:0.02307)0.946.10:0.01111,(Bfhggg20:0.01112,(B
fhggg21:5.5E-4,Bfhggg22:0.00366)0.813.5:5.5E-4)0.911.15:5.5E-
4)0.943.12:0.01089,UltPor42:5.4E-
4)0.814.4:0.01135)0.770.12:0.00395,(((UltB7598:0.0146,Otu00484:0.01356)0.
985.9:0.03679,((UltB7595:0.01899,Tryyy035:0.19942)0.518.1:5.3E-
4,(UltB7588:0.00893,UltB7587:0.03447)0.893.18:0.01548)0.932.8:0.01416)0.8
44.15:6.7E-4,(MsHtH004:0.02779,(UltB7584:5.5E-
4,(UltB7585:0.00743,(UltB7586:0.00394,Otu00386:0.06177)0.860.7:0.00758)0.
988.10:0.0274)0.940.7:0.01186)0.756.13:0.00334)0.927.9:0.01146)0.874.13:5
.5E-
4,((Otu00486:0.00767,Otu00199:0.03089)0.734.3:0.01366,UltB7594:0.01414)0.
624.3:0.00807)0.774.16:0.00671)0.781.11:0.00544)0.755.7:0.01198,Otu00956:
0.05718)0.790.3:0.00983,((UltB7593:0.01917,Otu00519:0.01728)0.894.11:0.01
454,Otu00495:0.02504)0.668.6:0.00248)0.930.7:0.01794,(MsAtAtA0:0.0,RodSym
b2:0.0):0.04006)0.000.158:0.00931)0.853.9:0.01181)0.964.10:0.01535,((BacN
L190:5.5E-4,((BacNL187:0.0,BacNL188:0.0,BacNL189:0.0):5.5E-
4,BacNL191:5.5E-4)0.757.6:5.5E-
4)0.851.10:0.00954,(UltB7543:0.02435,Otu01984:0.01507)0.879.11:0.01585)0.
949.14:0.02152)0.000.159:5.5E-4,(((((UltB7578:0.01888,UltB7579:5.5E-
4)0.965.12:0.02638,((Otu00117:0.03441,Otu00285:0.01372)0.883.9:0.0174,(Ul
tB7580:0.02329,UltB7581:0.04301)0.000.160:0.0072)0.859.10:0.01238)0.618.1
:0.01036,Hmyy0017:0.07538)0.769.12:0.01546,(UltB7597:0.01307,UncUnc72:0.0
0635)0.997.12:0.05722)0.816.8:0.01687,((UncUnc70:0.0,UncUnc71:0.0):0.0193
6,UltB7566:0.03473)0.841.7:0.01383)0.392.3:0.02295)0.937.7:0.01919)0.785.
13:0.00522,((((((((((UltB7530:0.00369,(UltB7512:0.0,UltB7532:0.0,PbdDist
a:0.0):5.5E-4)0.617.1:5.5E-
4,UltB7517:0.00742)0.827.11:0.0037,((UltB7511:0.0,UltB7522:0.0,UltB7513:0
.0,UltB7521:0.0,Ult19273:0.0):5.5E-
4,(UltB7535:0.00369,UltB7518:0.0037)0.675.2:5.5E-4)0.844.16:5.5E-

4)0.831.12:0.00366,((UltB7520:0.01524,(UltB7515:0.00742,((UltB7531:0.0149
6,(PbdSpeci:5.5E-
4,((UltB7508:0.00744,BacNL186:0.00379)0.787.9:0.00369,((UltB7519:5.5E-
4,(UltB7523:0.00369,UltB7534:0.01117)0.698.3:5.5E-4)0.729.3:5.5E-
4,(UltB7510:0.02288,UltB7514:0.00369)0.868.9:5.5E-4)0.977.6:5.5E-
4)1.000.69:0.00266)1.000.70:0.001)0.476.5:5.5E-
4,UltB7533:0.01121)0.658.1:5.5E-4)0.600.3:0.00368)0.889.14:5.4E-
4,UltB7509:5.4E-4)0.782.18:5.5E-
4)0.954.9:0.01793,((UltB7529:0.06729,UltB7536:0.00805)0.754.10:0.00383,Gu
tMeta7:0.03558)0.875.12:0.01154)0.738.5:0.00408,(((UltB7528:0.00746,UltB7
524:0.00385)0.763.15:0.00893,(UltB7526:5.4E-
4,(UltB7525:0.01565,UltB7527:0.00374)0.800.8:0.00349)0.737.10:0.01031)0.9
91.10:0.04807,(UltB7540:5.5E-4,(UltB7539:0.00369,(UltB7538:5.5E-
4,UltB7537:0.01534)0.065.2:5.5E-
4)0.823.6:0.00369)0.952.16:0.03287)0.822.6:0.01712)0.955.10:0.01752,(UltB
7561:0.04776,UltB7541:0.01655)0.872.18:0.01556)0.686.4:0.00324,(UltB7564:
5.5E-
4,UltB7565:0.01115)0.840.11:0.01333)0.791.9:0.01689,((PahggY26:0.05952,(P
0114504:5.5E-4,P0114513:5.5E-
4)0.993.5:0.05469)0.818.2:0.0174,(((UltB7569:0.01583,(UltB7570:0.02262,(U
ltB7568:0.023,(UltB7567:0.00651,(UncUnc69:0.00605,((UncUnc65:0.0,UncUnc68
:0.0):5.5E-4,(UncUnc66:0.0037,UncUnc67:5.5E-
4)0.892.8:0.00358)0.339.2:0.00909)0.965.13:0.02612)0.233.1:0.0124)0.699.2
:0.00967)0.737.11:0.00417)0.922.13:0.01756,Otu00671:0.0217)0.906.13:0.014
85,PshggYy9:0.02164)0.774.17:0.00452)0.838.9:0.01071)0.819.12:0.0088,Pshg
gg22:0.01074)0.756.14:0.00354,(UltPor41:0.00765,Otu00047:0.04065)0.910.18
:0.01231)0.858.10:5.4E-
4)0.877.13:0.01554)0.949.15:0.02128,(((UltB7574:0.08203,(UltB7150:0.07085
,(UltB8550:0.07018,UltB8549:0.00125)0.995.11:0.08506)0.853.10:0.02811)0.4
51.4:0.00837,UltB7573:0.1541)0.826.7:0.01499,(UltB7563:0.0118,(UltB7327:0
.09375,((((UltB7322:0.00333,(UltB7319:0.03642,(BnsVisce:0.02101,(((((UltB
7315:5.5E-4,UltB7317:0.00375)0.947.9:5.5E-
4,UltB7316:0.05232)0.691.1:0.00377,(UltB7311:0.0114,UltB7312:5.5E-
4)0.779.10:0.00379)0.936.11:0.01154,(UltB7308:0.00754,((UltB7309:0.0,UltB
7320:0.0):5.5E-4,(UltB7307:0.00374,(UltB7314:0.01137,UltB7313:5.3E-
4)0.217.1:0.00376)1.000.71:5.4E-4)0.699.3:5.5E-4)0.928.10:5.4E-
4)0.426.4:0.00401,UltB7310:0.00358)0.869.10:0.01974)0.809.9:0.04143)0.964
.11:0.04478)0.875.13:0.00797,UltB7321:5.3E-
4)0.328.2:0.01485,UltB7323:0.00889)0.831.13:0.01968,(UltB7325:0.0,UltB732
6:0.0):0.07181)0.921.18:0.04467)0.937.8:0.04328)0.880.12:0.01919)0.941.9:
0.02666)0.745.5:0.01069)0.646.1:0.00606,((UltB6608:0.09898,(((((PahgYy17:
0.01295,UltPorp6:0.02537)0.709.1:0.00642,(((((((Pshggg19:5.4E-
4,Otu01373:0.06687)0.911.16:0.02331,Pshggg13:0.02808)0.923.11:0.01608,((P
shggg15:0.01033,((PahgYy22:5.3E-
4,S0114423:0.01531)0.951.11:0.01158,UltPor20:0.01095)0.649.4:0.00441)0.81
1.9:5.5E-
4,((((UltB7446:0.00749,Z0114382:0.00752)0.807.5:0.0037,((Z0114360:5.4E-
4,(UltB7445:0.02311,(UltB7447:0.00747,(Otu00193:0.00742,(UltB7448:0.01521
,UltB7443:0.00371)0.784.10:0.01145)0.146.2:5.3E-
4)0.967.8:0.01518)0.187.1:5.5E-
4)0.916.17:0.01485,(Otu00314:0.01594,Sgffff09:0.0102)0.979.11:0.0156)0.96
4.12:5.5E-4)0.921.19:5.4E-4,(UltB7441:0.02296,(UltB7442:5.4E-
4,(UltPor14:0.00761,(UltB7439:0.0,Otu00061:0.0):0.02747)0.933.14:0.01527)
0.943.13:0.01451)0.327.1:0.00478)0.756.15:0.01278,(Z0114380:0.00647,Otu00

630:0.03015)0.509.1:0.00556)0.890.14:0.01054)0.803.8:0.00355)0.775.6:0.00
429,((((PahYyyy9:0.0,PahggY21:0.0,PahgYy13:0.0):0.00753,(P0114410:0.02766
,Pshggg16:0.01142)0.898.21:0.01148)0.864.12:0.00774,PahggY22:0.0155)0.768
.8:0.00448,P0114503:0.02309)0.888.13:0.00979)0.259.1:0.00361,((((Pshggg17
:0.01535,(((Bfhggg13:0.02732,(PahgYy08:0.01137,S0114390:5.4E-
4)0.865.11:0.00864)0.905.11:0.01172,S0114443:0.01173)0.354.2:5.4E-
4,(P0114443:0.01537,UltPor22:0.01164)0.949.16:0.01866)0.816.9:0.0075)0.66
7.4:0.00373,PahggY17:5.4E-
4)0.841.8:0.00912,UltB7449:0.00601)0.479.3:0.00761,Bfhggg14:0.02553)0.785
.14:0.00436)0.875.14:0.0118,PahggY15:0.00622)0.770.13:0.00546,((((((Bfhgg
g15:0.18614,Pshggg20:0.01217)0.755.8:0.00719,PshggY00:0.02909)0.306.1:0.0
0659,UltPor19:0.0678)0.699.4:0.00442,(PahgYy21:0.03377,PahgYyyy:0.02132)0
.961.10:0.03199)0.775.7:0.00406,((Z0114390:0.0035,((((Cuiiii13:0.06179,Ul
tB7394:0.03148)0.756.16:0.00976,(((Otu00228:0.02727,(Otu00021:0.01606,Otu
00032:0.01623)0.964.13:0.02391)0.774.18:0.00856,Otu00133:0.01221)0.834.9:
0.01133,(Otu00826:0.02239,(UltB7401:0.11602,Cuiiii14:0.04029)0.877.14:0.0
2736)0.946.11:0.03424)0.881.14:0.01784)0.918.17:0.01985,((UltB7399:0.0453
,(((UltB7417:0.01887,(UltB7416:0.00359,UltB7415:0.03519)0.931.13:5.5E-
4)0.843.6:0.01535,(UltB7413:0.03078,UltB7414:0.01961)0.850.10:0.0153)0.96
6.10:0.02925,(((Otu00018:0.00396,Otu00642:0.03868)0.846.8:0.0235,((UltB74
11:0.00707,UltB7412:0.01274)0.785.15:0.01025,((UltB7408:0.00377,UltB7409:
0.00736)0.872.19:0.00781,(CanAzoba:0.00333,UltB7410:0.03623)0.399.2:0.008
01)0.929.9:0.01804)0.900.8:0.01735)0.980.4:0.03156,UltB7418:0.04239)0.110
.2:0.00337)0.870.8:0.0159)0.785.16:0.00815,(UltB7419:0.03697,(UltB7420:0.
02882,(OphiYyyy:0.03956,UltB7421:0.02616)0.954.10:5.4E-
4)0.960.9:0.03321)0.424.4:0.00663)0.752.9:0.01465)0.922.14:0.02714,Otu014
08:0.03396)0.900.9:0.02093)0.914.11:0.02553,(Sgffff08:0.06094,(UltPor13:5
.5E-4,Otu00739:5.5E-
4)0.997.13:0.08304)0.761.10:0.01565)0.758.11:0.0102)0.745.6:0.00421,(((((
(UltB7367:0.00763,UltPor12:0.04144)0.937.9:0.02413,((((Ma0sttt6:5.5E-
4,(UltB7395:0.02815,UltB7396:0.00667)0.998.8:0.04576)0.917.12:0.02378,((U
ltB7402:0.00884,(Otu00725:0.03608,(UltB7403:0.01688,UltB7422:0.05031)0.19
8.3:0.01485)0.841.9:0.01879)0.935.13:0.02236,Otu00147:0.00925)0.913.11:0.
01498)0.809.10:0.00657,(((UltB7368:0.01608,UltB7369:0.02103)0.967.9:0.029
67,((((UltB7440:0.00958,((UltB7374:0.02516,Otu00095:0.04334)0.795.11:0.01
011,(UltB7371:0.03992,(UltB7377:0.00975,Otu00223:0.0036)0.887.10:0.00719)
0.092.2:5.5E-
4)0.572.2:0.00746)0.787.10:0.00654,(UltB7400:0.035,UltB7370:0.02822)0.837
.7:0.01284)0.848.10:0.00181,((Otu00031:0.02188,(UltB7376:5.5E-
4,(UltB7375:0.02571,Otu00106:0.0164)0.896.8:0.01602)0.896.9:0.01955)0.867
.13:0.02153,(((UltB7391:0.03142,((UltB7388:0.01533,((CanArma3:0.00806,(Ca
nArma6:5.5E-4,(CanArma4:0.00369,CanArma5:5.5E-
4)0.836.7:0.00369)0.975.6:0.02703)0.611.1:0.00796,Otu00074:0.00828)0.893.
19:0.01089)0.932.9:0.01532,(UltB7390:0.01892,((UltB7389:0.00387,Otu00027:
0.00355)0.994.14:0.02327,(CanArma2:0.0037,(CanArman:0.0,Otu00001:0.0):5.5
E-4)0.937.10:0.01138)0.839.6:5.4E-
4)0.276.3:0.00369)0.923.12:0.01555)0.814.5:5.5E-
4,Otu00178:0.04392)0.804.9:0.00765,UltB7373:0.07606)0.705.5:5.4E-
4)0.880.13:0.01327)0.915.14:0.00651,(UltB7398:0.03428,UltB7372:0.02557)0.
861.12:0.01126)0.765.8:0.00554)0.829.9:0.01158,(UltB7404:0.00885,MsGtttt2
:0.03532)0.932.10:0.02099)0.804.10:0.00883)0.923.13:5.4E-
4,((UltB7383:0.03393,(UltB7385:0.04742,(UltB7384:0.02293,Otu00591:0.00832
)0.753.7:0.0199)0.537.1:0.00816)0.988.11:0.04983,(UltB7423:0.05117,(UltB7
433:0.01854,(Otu00457:0.01117,(ZnvYyy00:0.01555,(UltB7434:0.00369,Otu0031

5:5.5E-
4)0.964.14:0.02348)0.629.2:0.01352)0.047.1:0.01651)0.637.3:0.02421)0.675.
3:0.0081)0.888.14:0.01777)0.925.21:0.00661)0.748.7:5.4E-
4,((Otu00162:0.01283,(((UltB7424:0.01942,Otu00046:0.017)0.589.3:0.02133,O
tu00924:0.04549)0.830.11:0.02055,UltB7397:0.04089)0.742.10:0.0103)0.716.3
:0.00235,(Otu00200:0.02298,((UltPor21:0.03394,((UltPor15:0.0,UltPor17:0.0
):0.00369,((UltB7437:0.0,UltB7438:0.0,UltB7444:0.0,Otu00048:0.0):5.5E-
4,(UltPor16:0.01926,UltB7436:0.00744)0.816.10:5.5E-4)0.257.2:5.5E-
4)0.945.12:5.3E-
4)0.875.15:0.00748,UltPor18:0.00377)0.876.10:0.01093)0.773.14:0.00563)0.9
56.10:0.01277)0.892.9:0.01622,UltPorp7:0.0261)0.883.10:0.01726,((UltB7435
:0.00784,Otu00251:0.01114)0.999.11:0.03098,(((((UltB7432:5.5E-
4,Otu00292:0.0036)0.909.7:0.01741,((UltB7431:0.01044,(((UltB7425:0.00369,
Otu00645:5.5E-
4)0.964.15:0.01938,(UltB7430:0.0038,(UltB7426:0.01148,(UltB7428:0.00369,(
UltB7429:0.00369,(UltB7427:0.0,Otu00209:0.0):5.5E-4)0.459.2:5.5E-
4)0.868.10:0.00756)0.770.14:0.0036)0.844.17:0.00758)0.747.10:0.00506,Z011
4440:0.01839)0.652.2:0.01112)0.908.9:0.01615,Otu00194:0.01229)0.059.1:0.0
0828)0.988.12:0.05102,Hmyy0016:0.02734)0.869.11:0.01555,((PahYyyy7:0.0111
9,PahggY20:0.00387)0.986.8:0.03534,(Bfhggg12:0.01382,(PahggY16:0.01106,Ps
hggg14:0.02108)0.971.5:0.03152)0.950.11:0.02525)0.504.2:5.4E-
4)0.864.13:0.01175,(UltPor11:0.01204,(((UltB7405:0.0,Otu00452:0.0):0.0461
5,(UltB7407:0.08499,UltB7406:0.01438)0.962.15:0.04409)0.914.12:0.02533,(U
ltPor10:0.06417,(UltB7453:0.05407,(UltPorp8:5.5E-
4,UltPorp9:0.00369)0.867.14:0.01496)0.834.10:0.01665)0.593.1:0.0031)0.871
.10:0.00914)0.919.14:0.01467)0.948.13:0.02158)0.219.2:5.4E-
4)0.770.15:5.4E-4,(P0114492:5.4E-
4,(Pshggg18:0.01415,(PahggY18:0.00743,PahggY19:0.00385)0.970.9:0.02319)0.
885.10:0.01384)0.818.3:0.00375)0.877.15:0.00869)0.942.5:0.01643)0.956.11:
0.02409)0.809.11:0.00985,((S0114370:0.03489,((PahYyyy8:0.0,PahggY12:0.0,P
ahggY13:0.0):0.01942,PahggY14:0.01615)0.725.7:0.01101)0.689.2:0.01223,((U
ltB7380:0.05856,Otu01215:0.08402)0.783.9:0.01418,((Otu00579:0.05559,(UltB
7392:0.01882,UltB7393:5.5E-
4)0.966.11:0.04173)0.981.3:0.04847,(Otu00447:0.05962,(UltB7379:0.01609,(U
ltB7378:0.04305,Otu01062:0.01002)0.993.6:0.06774)0.868.11:0.02672)0.521.1
:0.00823)0.788.8:0.01118)0.809.12:0.01055)0.449.4:0.00211)0.936.12:0.0312
4,S0114350:0.06619)0.883.11:0.02648,UltB7324:0.07946)0.777.9:0.01985)0.85
1.11:0.02844,(((UltB6444:0.0999,(UltB5948:0.07854,(UltB5893:0.14215,(((((
UltB5952:0.06343,((UltB5949:0.04053,((UltB5951:5.5E-
4,UltB5950:0.02749)0.970.10:0.03741,(PprXylan:0.05852,((UltB5933:0.0,UltB
5934:0.0,UltB7351:0.0):0.01453,(UltB5937:0.00407,((UltB5935:0.0,UltB5936:
0.0,UltB5938:0.0):0.00418,UltB5939:0.00332)0.457.2:0.00774)0.848.11:0.009
66)0.699.5:0.00817)0.970.11:0.04309)0.958.12:0.03379)0.086.3:5.5E-
4,((UltB5930:0.02822,UltB5929:0.08145)0.975.7:0.05919,((UltB5931:0.0,UltB
5932:0.0):0.05446,(((UltB5914:5.5E-4,UltB5916:5.5E-
4)0.836.8:0.01032,((UltB5912:5.5E-4,UltB5915:0.00373)0.999.12:5.5E-
4,((UltB5904:0.0,UltB5905:0.0,UltB6484:0.0):0.00218,((UltB5906:0.00374,Ul
tB5913:0.0153)0.969.12:0.01942,(UltB5908:5.5E-
4,((UltB5903:0.0,UltB5907:0.0,UltB5909:0.0):5.5E-
4,UltB5910:0.00373)0.875.16:5.5E-4)0.995.12:5.5E-
4)0.650.1:0.00183)0.953.10:0.0109)0.818.4:0.01743)0.992.9:0.05828,(UltB59
17:0.04902,UltB5919:0.04168)0.849.8:0.01433)0.918.18:0.02509)0.733.5:0.01
571)0.910.19:0.02938)0.942.6:0.0272)0.000.161:5.4E-
4,(((UltB5858:0.0314,UltB5857:0.02873)0.000.162:5.5E-

4,(UltB5921:0.0322,UltB5920:0.02713)0.998.9:0.08506)0.662.4:0.01994,(((Ul
tB5856:0.01199,((UltB5854:5.4E-
4,(UltB5852:0.00383,UltB5853:0.03581)0.296.1:0.00364)0.899.10:0.02082,Ult
B5855:0.06056)0.657.1:0.01358)0.546.3:0.00983,UltPre13:0.01374)0.964.16:0
.02325,(UltB5877:0.05005,((((((UltB5703:0.01531,(UltB5860:0.0,UltB5861:0.
0,UltB5862:0.0,UltB5863:0.0,UltB5864:0.0):0.00359)0.981.4:0.04845,(UltB58
65:0.00347,UltB5851:0.08451)0.340.1:0.00697)0.965.14:0.04287,GutMeta4:0.0
4169)0.579.1:0.01858,((UltB5872:0.07081,((UltB5876:0.0,UltB5875:0.0,UltB5
873:0.0,UltB5874:0.0):5.5E-4,Ult19274:0.00369)0.888.15:5.5E-
4)0.945.13:0.01962,((UltB5849:0.0307,(((UltB5842:0.00368,(UltB5847:0.0036
9,UltB5843:0.02397)0.014.1:5.5E-
4)0.810.8:0.00664,(UltB5845:0.00369,UltB5846:5.5E-
4)0.878.11:0.0089)0.780.14:0.00386,(UltB5844:0.01938,((UltB5831:0.01049,(
UltB5836:0.0073,((UltB5835:0.0,UltB5827:0.0):0.00368,(UltB5830:5.5E-
4,((UltB5828:0.0,UltB5840:0.0):5.5E-4,UltB5829:5.5E-4)0.350.1:5.5E-
4)0.278.1:5.5E-4)0.938.10:5.4E-
4)0.201.2:0.00486)0.908.10:0.01582,((UltB5848:0.03525,(GutMeta3:0.00708,(
UltB5841:5.5E-
4,Ult19276:0.00369)0.759.11:0.0041)0.973.5:0.02738)0.840.12:0.0088,(UltB6
375:0.01115,(UltB5837:0.00369,UltB5838:0.00369)0.828.6:0.00371)0.935.14:5
.5E-
4)0.329.2:0.01031)0.777.10:0.00625)0.660.1:0.00376)0.805.8:0.00808)0.410.
2:0.00654,((UltB5879:0.00363,UltB5880:0.00393)0.966.12:0.0429,UltB5878:0.
06735)0.795.12:0.02668)0.958.13:0.03166)0.781.12:0.00895)0.838.10:0.01168
,((UltB5868:5.4E-
4,UltB5869:0.00734)1.000.72:0.06104,(UltB6374:0.04303,(UltB5866:0.0498,Pr
eSpec9:0.02179)0.907.14:0.01955)0.388.4:5.5E-
4)0.796.5:0.00562)0.000.163:5.4E-
4,(UltB5867:0.03272,(UltB5870:0.05285,UltB5871:0.00964)0.973.6:0.02931)0.
879.12:0.01312)0.899.11:0.03897)0.892.10:0.02709)0.551.2:5.4E-
4)1.000.73:0.06323)0.952.17:0.02884,(UdnRum16:0.05153,(UltB5925:0.04488,(
(UltB5926:0.01708,UltB5927:0.05072)0.964.17:0.04221,(UltB5922:0.01173,(Ul
tB5924:0.04786,UltB5923:0.01472)0.859.11:0.00999)0.851.12:0.01611)0.868.1
2:0.02215)0.921.20:0.04149)0.894.12:0.03072)0.818.5:0.01274,(((UltB5891:0
.09598,(UltRu135:0.03383,UltRu136:0.03438)0.867.15:0.03365)0.570.2:0.0190
8,UltB5947:0.11379)0.486.2:0.00593,((((UltB5886:0.02637,(UltB5888:0.07954
,UltB5887:0.00533)0.965.15:0.03572)0.341.3:0.00638,((UltB5900:0.02766,Ult
B8388:0.08357)0.998.10:0.08213,UltB5901:5.3E-
4)0.975.8:0.03416)0.772.13:0.00683,(UltB5885:0.00372,((UltB5884:0.0,UltB5
882:0.0,UltB5892:0.0):5.5E-4,UltB5883:0.00388)0.240.1:5.5E-
4)0.995.13:0.02859)1.000.74:5.4E-
4,((UltB5898:0.00586,(((UltB5895:0.02634,UltB8038:0.08832)0.582.3:5.5E-
4,(UltB5896:0.0,UltB5897:0.0):0.00373)0.824.12:0.00878,UltB5899:0.02414)0
.794.10:0.00729)0.980.5:0.02458,(((UltB5902:0.01388,(UltRu133:0.03711,Ult
B5890:0.06388)0.465.2:0.01908)0.774.19:0.00446,(UltB5889:0.01916,UltB5894
:0.02767)0.699.6:0.00164)0.892.11:0.01132,UltRu134:0.01577)0.895.15:0.014
66)0.753.8:0.0038)0.907.15:0.02322)0.952.18:0.0237)0.832.8:0.00915,((((((
UltB7963:0.00405,Ult23738:0.05716)0.969.13:0.02698,(UltB5963:0.01535,UltB
5964:0.01504)0.111.1:5.4E-
4)0.802.8:0.01915,((UltB5962:0.0,UltB5965:0.0):5.5E-
4,UltB5961:0.05101)0.900.10:0.01095)0.899.12:0.0114,UltB5966:5.4E-
4)0.884.10:5.5E-
4,UltB5967:0.06983)0.999.13:0.07316,(((UltB5944:0.11456,((((UltB5535:0.04
368,(((((UltB5519:5.5E-

4,UltB5517:0.03051)0.844.18:0.00366,((UltB5522:0.01551,UltB5518:0.00379)0
.997.14:5.5E-4,UltB5521:5.5E-4)0.493.3:5.5E-
4)0.959.11:0.03363,(UltB5520:0.03454,((UltB5514:5.5E-
4,(UltB5506:0.01534,(((((UltB5508:0.00177,UltB5510:0.00378)0.482.3:6.9E-
4,UltB5516:0.00372)0.000.164:7.2E-4,UltB5509:0.00373)1.000.75:5.7E-
4,(UltB5515:0.00367,(UltB5505:0.01197,UltB5507:0.0268)0.903.8:0.01139)0.5
28.1:5.4E-
4)0.849.9:0.0043,(UltB5504:0.06464,UltB5512:0.00335)0.876.11:0.01068)0.91
2.14:0.00458)0.891.13:0.00791)0.862.13:0.00988,UltB5513:0.01728)0.939.8:0
.02826)0.510.2:0.00749)0.985.10:0.07907,UltB5722:0.05003)0.371.4:0.02081,
(XlnOryza:0.06765,(UltB5632:0.08951,UncUnc48:0.06181)0.948.14:0.05619)0.5
28.2:0.02227)0.888.16:0.03302)0.713.5:0.00747,((((((UltRum77:0.0269,UltRu
115:0.06007)0.740.6:0.00753,((((UltRu130:0.03436,(((UltB5280:0.00785,UltR
u103:0.06194)0.792.14:0.01449,((UltB5285:5.5E-
4,(UltB5274:0.00368,((UltB5279:0.0,UltB5281:0.0,UltB5272:0.0,UltB5275:0.0
,UltB5276:0.0,UltB5282:0.0,UltB5283:0.0,UltB5284:0.0):5.5E-
4,(UltB5273:0.00369,UltB5278:0.00372)0.606.1:5.5E-4)0.000.165:5.5E-
4)0.985.11:5.5E-4)0.891.14:5.4E-
4,(UltB5378:0.03861,((UltB5286:0.0,UltRum47:0.0):0.00737,(((UltRum42:0.01
721,(UltRu108:0.00745,(UltB5373:0.0,UltRu109:0.0):5.5E-
4)0.971.6:0.03208)0.932.11:0.02373,UltRum90:0.02115)0.720.6:0.00376,UltRu
m48:0.07469)0.511.2:0.01382)0.858.11:0.00732)0.910.20:5.4E-
4)0.837.8:0.00975)0.727.6:0.00591,UltB5312:0.03046)0.483.3:0.01071)0.913.
12:0.02305,((((((UltRum82:0.01507,(UdnRume6:5.5E-
4,(UltRum54:0.01131,UdnRume5:0.00369)0.718.5:5.5E-4)0.912.15:5.4E-
4)0.941.10:0.01097,((UltRum60:0.00719,UltRum72:0.02004)0.925.22:0.01234,(
UltRum67:0.00374,((UltRum66:0.0234,UltB5301:0.01937)0.734.4:0.00352,((Ult
Ru104:0.02137,(UltRu199:0.01731,(UltB5473:0.02387,((UltB5302:0.0,UltB5303
:0.0):5.5E-
4,UltB5304:0.00369)0.944.7:0.02261)0.960.10:0.02754)0.943.14:0.03327)0.96
6.13:0.03298,(UltRum68:0.01115,(UltRum70:5.5E-4,UltRum69:5.5E-
4)0.540.3:5.4E-4)1.000.76:5.5E-
4)0.775.8:0.00366)0.764.11:0.00375)0.825.6:0.00807)0.841.10:0.00702)0.819
.13:5.5E-
4,(UltRu101:0.01116,((UltRum61:0.00759,(UltB5306:0.00741,((UltRum63:0.0,U
ltRum62:0.0,UltRum64:0.0,UltRum65:0.0):5.5E-4,UltB5305:5.5E-
4)0.819.14:5.5E-
4)0.947.10:0.01535)0.858.12:0.00735,UltRum75:0.00782)0.587.3:0.00753)0.29
7.1:5.4E-4)0.290.3:5.4E-4,(UdnRume7:5.5E-
4,((UltRum73:0.0,UltRum74:0.0):0.01527,UltRum56:0.00373)0.058.1:0.00375)0
.950.12:0.01119)0.969.14:0.01515,(((UltRum59:0.01202,UltRum37:0.02794)0.9
12.16:0.0039,UltRum58:0.03131)0.916.18:0.00279,(UltRum55:0.01597,(UltB528
7:0.0,UltB5316:0.0):0.02335)0.855.9:0.0078)0.880.14:0.00817)0.754.11:0.00
585,(((UltB5307:0.01914,UltB5322:5.5E-
4)0.821.8:0.00987,(UltB5747:0.05097,UltB5377:0.00591)0.534.1:0.01301)0.81
3.6:0.01003,((((((UltB5277:0.0,UltB5310:0.0,UltB5311:0.0):5.5E-
4,UltB5309:5.5E-4)0.955.11:0.00744,(UltB5346:5.5E-
4,(UltRum91:0.0,UltRum92:0.0,UltRum93:0.0):5.5E-4)0.816.11:5.5E-
4)0.911.17:5.4E-
4,(((((UltRum76:0.0,UltB5332:0.0,UltB5334:0.0,UltPrevo:0.0,UltB5342:0.0,
UltRum84:0.0):5.5E-4,UltB5336:5.5E-4)0.000.166:5.5E-
4,UltB5340:0.00369)0.851.13:5.5E-
4,(UltB5337:0.03853,((((UltRum81:0.0,UdnRume8:0.0,UltB5333:0.0,UltRum83:0
.0,UltB5341:0.0):5.5E-4,PreRumin:0.0074)0.757.7:5.5E-4,UltB5335:5.5E-

4)1.000.77:5.5E-4,UltB5339:0.00737)0.832.9:0.00366)0.935.15:5.5E-
4)0.987.8:5.4E-4,((((UltRum99:0.0,UltRu100:0.0):5.5E-
4,UdnRum12:0.00747)0.993.7:0.02324,(UdnRum11:0.02707,(UdnRume9:0.03111,((
UdnRum10:0.0,UltB5344:0.0):5.5E-
4,(UltRum85:0.00745,UltRum87:0.00369)0.891.15:5.5E-4)0.976.7:5.5E-
4)0.342.1:0.00485)0.918.19:0.01473)0.755.9:0.00382,(((UltRum86:0.00744,Ul
tRum88:0.0037)0.651.5:5.5E-4,(UltRum89:5.5E-4,UltB5343:5.5E-
4)0.109.3:5.5E-4)0.603.5:5.5E-4,((UltB5313:0.0,UltRum97:0.0):5.4E-
4,UltRum98:0.01936)0.909.8:0.00744)0.995.14:5.4E-
4)0.451.5:0.01144)0.921.21:0.01121,(UltRum96:0.0,UltRum95:0.0):5.5E-
4)0.936.13:0.01101)0.874.14:0.00715,(UltRum94:5.4E-
4,UltB5338:0.01116)0.532.1:5.5E-
4)0.887.11:0.00719,((UltB5314:0.0,UltB5318:0.0,UltB5315:0.0,UltB5317:0.0,
UltB5319:0.0,UltB5320:0.0,UltB5321:0.0,UltB5323:0.0,UltB5324:0.0,UltB5345
:0.0,UltB5347:0.0,UltB5940:0.0):5.5E-4,UltB5385:0.00749)0.914.13:5.4E-
4)0.119.2:0.00371)0.937.11:0.01658)0.771.16:0.01824)0.599.2:0.01485,(((Ul
tB5356:0.0,UltB5359:0.0):5.4E-4,(UltB5360:0.01109,(UltB5357:5.5E-
4,UltB5358:0.00364)0.924.15:5.5E-
4)0.330.1:0.00364)0.759.12:0.00528,((UltRu105:0.02138,(((UltB5363:5.5E-
4,UltB5364:5.4E-
4)0.749.15:0.00374,((UltRu107:0.00631,(UltRu106:0.01231,UltB5367:0.0215)0
.843.7:0.01532)0.954.11:0.037,((UltB5350:0.0025,((UltB5349:0.0,UltB5353:0
.0):0.01135,((UltB5348:0.0,UltB5351:0.0):5.5E-
4,UltB5352:0.00369)0.992.10:5.5E-
4)0.801.10:0.01259)0.885.11:0.03289,UltRu102:0.05949)0.837.9:0.03161)0.87
3.19:0.0201)0.810.9:0.00738,(UltB5329:0.00368,(UltB5326:0.0037,(UltB5325:
0.0,UltB5327:0.0,UltB5328:0.0):5.5E-4)0.824.13:5.5E-4)1.000.78:5.5E-
4)0.991.11:0.02923)0.545.5:0.00334,(UltRum45:0.0192,(UltB5361:0.01415,Ult
B5362:0.0139)0.633.3:0.01448)0.826.8:0.01068)0.965.16:0.03175)0.838.11:0.
01666)0.804.11:0.00968,((UltB5308:0.05189,UltRu111:0.03085)0.344.3:0.0204
3,((UltRu110:0.05392,((UltRu112:0.01754,((UltRum71:0.00745,UltRu131:0.007
48)0.942.7:0.01724,((UltB5372:5.5E-4,UdnRum13:0.01518)0.376.1:5.4E-
4,(((UltB5370:0.0,UltB5371:0.0):5.5E-
4,(UltB5644:0.01495,UltB5368:0.00369)0.671.4:5.5E-4)0.687.3:5.5E-
4,UltB5369:0.00369)0.825.7:0.00368)0.793.11:0.00572)0.929.10:0.01955)0.74
7.11:0.00519,(UltRu113:0.0,UltRu114:0.0):0.00779)0.796.6:0.0082)0.686.5:0
.01467,((((UltRum36:0.03748,UltRum46:0.06069)0.828.7:0.02866,((UltB5268:0
.0,UltB5269:0.0):5.5E-4,UltB5270:5.5E-
4)0.957.8:0.03092)0.822.7:0.0115,((UltRum34:0.00373,(UltRum38:0.00447,(Ul
tRum52:0.00765,UltRum53:0.00732)0.994.15:0.04014)0.977.7:0.02663)0.000.16
7:0.0035,((((UltRum40:0.0,UltRum41:0.0):0.04007,(UltRum33:0.00368,UltB527
1:0.02678)0.657.2:5.5E-4)0.951.12:0.01896,UdnRume4:5.4E-4)1.000.79:5.4E-
4,(UltRum39:0.01483,UltB5288:0.01829)0.000.168:0.01371)0.746.9:0.00347)0.
787.11:0.00852)0.946.12:0.02636,UdnRume3:0.03608)0.936.14:0.02227)0.762.1
1:0.00644)0.787.12:0.0098)0.842.16:0.01663)0.958.14:0.02563,(UltRum43:0.0
8467,(UltB6478:0.04708,(UltB5366:5.5E-4,UltB5365:0.00369)0.871.11:5.4E-
4)0.930.8:0.03506)0.797.12:0.02087)0.222.1:0.01439,((UltB5384:0.03962,(Ul
tB5381:0.00706,(UltB5382:0.0768,(UltB5383:0.00369,(UltB5380:5.5E-
4,(UltB5379:0.00369,UltB5376:0.00369)0.668.7:5.5E-4)0.806.3:5.5E-
4)0.881.15:5.5E-4)0.831.14:5.5E-
4)0.397.2:0.01178)0.964.18:0.03042,((UltRum35:0.0,UltB5330:0.0):0.0168,Ul
tRum78:0.0104)0.968.5:0.02764)0.761.11:0.00902)0.755.10:0.00965,UltRum44:
0.05677)0.827.12:0.02272,(UltRu125:0.02482,(((((UltB5295:0.0,UltB5294:0.0
,UltB5296:0.0):0.00773,(UltB5293:5.4E-

4,((UltB5289:0.0,UltB5291:0.0):5.4E-
4,UltB5292:0.01537)1.000.80:0.07251)0.992.11:0.06624)0.929.11:0.03551,(Ul
tB5297:0.04969,(UltRum57:0.02537,(UltRum49:0.00511,(UltRum50:0.0,UltRum51
:0.0):0.02198)0.998.11:0.07253)0.732.1:0.03141)0.588.1:0.01424)0.360.2:0.
02165,(UltRum80:0.0038,(UltB5331:5.5E-
4,UltRum79:0.00369)0.870.9:0.00738)0.977.8:0.04707)0.737.12:0.02194,UltRu
126:0.11052)0.261.1:0.02422)0.824.14:0.02695)0.903.9:0.02564)0.830.12:0.0
2191,(((((PreNance:5.4E-
4,UltB5631:0.00728)1.000.81:0.09483,((PreTimon:0.01523,((UltB5628:5.5E-
4,UltB5629:0.03098)0.999.14:0.06987,UltB5630:0.00624)0.816.12:0.02846)0.9
75.9:0.05213,(UltPrev9:0.05093,(PreOrali:0.05347,(UltB5623:0.02906,PreMar
sh:0.03146)0.899.13:0.02688)0.134.3:0.01236)0.701.3:0.00793)0.807.6:0.016
2)0.502.1:0.00863,(PreSpec2:0.01633,(UltB5586:0.02848,((UltB5583:0.0,UltB
5584:0.0):5.5E-
4,UltB5585:0.01486)0.848.12:0.02107)0.990.11:0.04753)0.969.15:0.04097)0.6
04.2:0.01282,(((UltB5817:0.0,UltB5818:0.0):0.02314,(UltB6456:0.05208,(Ult
B6457:0.00369,UltB6458:5.5E-
4)0.863.13:0.01067)0.884.11:0.01513)0.864.14:0.03994,((UltB5355:0.0,UltB5
354:0.0):0.04541,UltB5460:0.07363)0.689.3:0.0161)0.935.16:0.03535)0.749.1
6:0.02036,((((UltB5386:0.04834,(UltB5443:0.05644,(UltB5745:0.0,UltB5746:0
.0):0.05617)0.825.8:0.03892)0.748.8:0.01221,(((((((UltB5411:0.0,UltRu117
:0.0,UltRu118:0.0):0.01851,UltB5410:0.01791)0.887.12:0.01381,(UltB5943:0.
0153,(UltB8037:0.01503,(UltB5409:5.5E-4,UltB5408:0.01112)0.148.1:5.5E-
4)0.896.10:0.00752)0.890.15:5.4E-
4)0.884.12:0.01429,(UltB5412:0.05611,(UltB5416:0.00369,(Ult19269:0.00369,
(UltB5413:0.0,UltB5415:0.0):5.5E-4)0.550.1:5.5E-
4)0.872.20:0.02663)0.957.9:0.03914)0.818.6:0.01362,(UltB5290:0.18893,UltR
u132:0.00637)0.997.15:0.07694)0.778.12:0.01189,(UltB5733:0.00476,((UltRu1
19:0.01239,((UltB5695:0.0,UltB5699:0.0,UltB5700:0.0):0.00245,(UltB5697:5.
5E-
4,UltB5698:0.0037)1.000.82:0.00132)0.997.16:0.04674)0.436.3:0.01207,(((Ul
tB5418:5.5E-4,UltB5741:0.00742)0.978.5:0.04197,(UltB5417:5.5E-
4,UltB5743:0.00369)0.947.11:0.03674)0.899.14:0.02147,((UltB5391:0.00367,U
ltB5959:0.05432)0.965.17:0.0405,UltB5682:0.0263)0.857.12:0.01706)0.495.2:
0.00306)0.771.17:0.0173)0.902.13:0.0149)0.860.8:0.00972,(UltB5554:0.06759
,((((UltB5604:5.5E-4,(((UltB5602:0.0,UltB5603:0.0):5.5E-
4,((((UltB5609:0.0,PreSterc:0.0):5.5E-4,UltB5607:0.00369)0.990.12:5.5E-
4,UltB5612:0.03094)0.954.12:0.01127,UltB5426:0.01122)0.487.3:5.4E-
4)0.935.17:5.4E-
4,(UltB5615:0.00756,((UltB5599:0.0191,(UltB5594:0.00369,((UltB5595:0.0,Ul
tB5597:0.0,UltB5726:0.0):5.5E-
4,((UltB5598:0.01116,UltB5593:0.00369)0.836.9:5.5E-
4,(Ult19277:0.01119,UltB5614:0.00369)0.729.4:5.5E-4)0.000.169:5.5E-
4)0.940.8:5.4E-4)0.187.2:5.4E-
4)0.799.10:0.00369,((((UltB5601:0.0,UltB5617:0.0):0.00349,UltB5613:0.0271
8)0.892.12:0.0077,UltB5600:0.0037)0.890.16:5.3E-4,UltB5596:5.5E-
4)0.827.13:5.4E-
4)0.783.10:0.00366)0.854.10:0.00733)0.868.13:0.00724)0.910.21:0.00739,(((
UltB5610:0.0,UltB5611:0.0,UltB5616:0.0,UltB5618:0.0):5.5E-
4,(UltB5605:0.00368,UltB5608:0.02663)1.000.83:5.5E-4)0.902.14:5.5E-
4,UltB5606:0.00742)0.928.11:5.5E-4)0.630.4:0.00515,(UltB5620:5.3E-
4,UltB5724:0.00741)1.000.84:0.07068)0.118.1:5.5E-
4,UltB5619:0.05072)0.960.11:0.03216)0.775.9:0.02103)0.796.7:0.01005,(((((
UltB5420:5.4E-4,(UltB5419:0.01922,(UltB5421:0.0,UltB5422:0.0):5.4E-

4)0.658.2:0.00363)0.999.15:0.05269,(UltB5398:0.00926,((UltB5396:5.5E-
4,UltB5395:0.00369)0.999.16:0.04848,UltB5399:0.01619)0.767.8:0.01327)0.81
2.3:0.01166)0.899.15:0.02147,((((((HumanGu2:5.5E-
4,UltB5424:0.00369)0.990.13:5.4E-
4,(UltB5425:0.00754,HumanGu3:0.01529)0.901.15:0.01135)1.000.85:0.08735,(U
ltB5458:0.01666,UltB5459:0.01846)0.845.10:0.0133)0.774.20:0.00966,(UltB54
56:0.0,UltB5457:0.0):0.01912)0.884.13:0.01886,(UltB5405:0.02285,(UltB5401
:0.02227,(UltB5400:0.00366,(UltB5402:0.00371,UltB5403:5.5E-
4)0.871.12:0.00765)0.983.9:0.03586)0.931.14:0.02166)0.839.7:0.0183)0.787.
13:0.01205,UltB5375:0.11266)0.857.13:0.02479,(PreSpec7:0.03639,(PreShahi:
0.00395,(UltB5622:0.00379,UltB5621:0.01136)0.789.7:0.00343)0.907.16:0.020
73)0.967.10:0.03463)0.833.14:0.01257)0.794.11:0.00808,(((((HllSereg:0.04
658,UltB5624:0.05449)0.772.14:0.01088,((UdnRum14:0.02196,UltRu127:0.00947
)0.965.18:0.03428,((UltB5580:0.00366,(UltB5581:0.03023,UltB5582:5.5E-
4)0.793.12:5.5E-4)0.953.11:0.03184,((UltB5563:0.00369,UltB5562:5.5E-
4)0.993.8:0.04291,((((UltB5572:0.03161,(UltB5577:5.4E-
4,((UltB5574:0.0848,(UltB5575:0.00369,(UltB5573:0.00369,(UltB5571:0.0,Ult
B5576:0.0,UltB5734:0.0):5.5E-4)0.116.2:5.5E-4)0.988.13:5.4E-
4)0.656.3:0.01004,UltB5737:0.01039)0.802.9:0.00573)0.741.8:0.00318)0.986.
9:0.03655,(PreMulti:0.06639,UdnRum15:0.00786)0.753.9:0.00412)0.869.12:0.0
166,PvtBacte:0.00289)0.455.5:0.01134,(UltB5564:0.00813,UltB5565:0.04845)0
.964.19:5.3E-
4)0.783.11:0.01703)0.913.13:0.02209)0.895.16:0.02244)0.764.12:0.00781)0.7
73.15:0.01203,(GutMeta2:0.01167,UltB5589:0.01183)0.996.9:0.05825)0.812.4:
0.02265,((((UltB5820:5.4E-
4,UltB5819:0.00743)0.992.12:0.03602,UltB5561:0.03172)0.762.12:0.00645,(Ul
tB5590:0.01347,(UltB5626:0.01918,(((((PreSpec3:0.0342,PreBerge:0.06523)0.
293.3:0.00735,(UltPrev4:5.5E-
4,((PreDenti:0.0038,PreMult2:0.0074)0.995.15:0.02729,(PreSpec5:0.00741,((
UltB5567:0.0,UltB5569:0.0):5.5E-4,UltB5568:0.00368)0.955.12:5.5E-
4)0.864.15:0.00742)0.277.3:0.00374)0.945.14:0.01506)0.198.4:5.3E-
4,((UltB5570:0.04665,(PreSpec6:0.02329,UltB5592:0.02414)0.828.8:0.01482)0
.085.1:0.01194,(((UltRu128:0.03531,(((UltPrev2:0.01135,(UltB5556:5.4E-
4,(PreSpeci:5.4E-4,(UltB5555:0.01884,PreMelan:5.5E-
4)0.926.13:0.00742)0.840.13:0.00369)0.776.8:0.00369)0.793.13:0.0037,(UltP
rev3:5.3E-4,PreVeror:0.0074)0.205.3:0.00369)1.000.86:5.4E-
4,PreHisti:0.00369)0.778.13:0.00396)0.761.12:0.00124,(UltPrev5:0.00919,Pr
eAlben:0.03514)0.246.1:0.01754)0.880.15:0.01826,(UltB5591:0.0,UltB5744:0.
0):0.0161)0.809.13:0.01314)0.754.12:0.01754)0.970.12:0.02284,(PreBaron:0.
03886,(UltB5627:0.00363,UltPrev8:5.5E-4)0.944.8:5.3E-
4)0.724.5:0.00717)0.811.10:0.00329,(PreSaliv:0.00767,(PreOulor:0.00351,Ul
tPrev7:0.00771)0.957.10:0.0195)0.943.15:0.01956)0.000.170:5.4E-
4)0.959.12:0.02551)0.650.2:0.00787)0.747.12:0.0088,(((PreSpec8:5.5E-
4,((UltB5813:0.02715,UltB5811:0.01926)0.763.16:0.00337,UltB5812:0.01497)0
.749.17:5.4E-4)0.686.6:5.4E-
4,(PreMican:0.05981,((UltB5816:0.02488,PreAmnii:0.01451)0.934.10:0.02041,
((PreDisie:0.01036,((PreCorpo:0.0,UltB5625:0.0):0.0427,(PreFalse:0.02066,
((UltB5814:0.00589,(UltPre10:0.0037,(PreInter:5.5E-
4,UltPre11:0.00368)0.606.2:5.5E-
4)0.794.12:0.00529)0.053.1:0.00653,((PrePall2:0.0039,PrePalle:5.4E-
4)0.911.18:0.01711,PreNigre:0.02398)0.683.6:0.01388)0.678.3:0.01136)0.885
.12:0.01921)0.605.2:0.01215)0.710.3:0.00896,UltB5815:0.01156)0.956.12:0.0
2307)0.940.9:0.02289)0.740.7:0.00671)0.795.13:0.0118,UltPre12:0.05358)0.5
62.1:0.0185)0.939.9:0.02961)0.360.3:0.01129,(UltB5559:0.05576,(((UltB5797

:5.5E-4,(((PvtBact2:0.00369,UltB5799:0.00369)0.653.2:5.5E-
4,(UltB5414:0.0,UltB5796:0.0,UltB5798:0.0):5.5E-4)0.000.171:5.5E-
4,((UltB5646:0.00369,UltB5795:0.00743)0.712.2:5.5E-4,UltB5725:5.5E-
4)0.000.172:5.5E-4)0.723.5:5.5E-4)0.747.13:5.5E-
4,(UltB5701:0.00744,UltB5732:5.4E-
4)0.939.10:0.00741)0.743.11:0.00601,(((((UltB5645:0.0,UltB5739:0.0,UltB57
65:0.0,UltB5783:0.0,UltB5786:0.0,UltB5791:0.0,UltB5792:0.0,UltB5800:0.0,U
ltB5804:0.0):5.5E-
4,(UltB5787:0.00369,(UltB5793:0.00369,UltB5731:0.01499)0.916.19:5.5E-
4)0.786.6:5.5E-4)0.459.3:5.5E-
4,((((UltB5785:0.01121,(UltB5789:0.07957,UltB5756:0.00359)0.761.13:0.0038
2)0.678.4:5.4E-
4,(UltB5640:0.0,UltB5685:0.0,UltB5728:0.0,UltB5735:0.0,UltB5738:0.0,UltB5
784:0.0,UltB5790:0.0,UltB5803:0.0):5.5E-4)1.000.87:5.4E-
4,UltB5781:0.02649)0.841.11:0.00368,UltB5782:0.0074)0.215.2:5.5E-
4)0.963.13:5.4E-4,((UltB5772:5.4E-
4,(UltB5788:0.00739,(((((((((((UltB5736:0.0074,((UltB5806:0.03458,((Ult
B5805:0.0,UltB5808:0.0,UltB5809:0.0):5.5E-
4,(UltB5810:0.0037,UltB5807:0.01116)0.922.15:5.5E-4)0.928.12:5.4E-
4)0.951.13:0.01118,(UltB5670:0.00741,((((((UltB5666:0.00368,UltB5673:0.00
369)0.820.3:0.00369,UltB5674:0.05609)0.000.173:5.5E-
4,UltB5675:0.00369)0.491.2:5.5E-4,UltB5665:5.5E-4)0.534.2:5.5E-
4,((UltB5660:0.0,UltB5661:0.0,UltB5662:0.0,UltB5667:0.0,UltB5669:0.0,UltB
5671:0.0,UltB5686:0.0,UltB5758:0.0):5.5E-
4,(UltB5672:0.00739,((UltB5668:5.5E-4,UltB5750:0.00713)0.906.14:5.5E-
4,UltB5659:0.00369)0.000.174:5.5E-4)0.000.175:5.5E-4)0.000.176:5.5E-
4)0.570.3:5.5E-4,UltB5707:5.5E-4)0.570.4:5.4E-
4)0.840.14:0.00373)0.361.1:5.3E-4)0.464.2:5.4E-
4,(UltB5647:0.0,UltB5633:0.0,UltB5637:0.0,UltB5638:0.0,UltB5648:0.0,UltB5
650:0.0,UltB5651:0.0,UltB5676:0.0,UltB5681:0.0,UltB5690:0.0,UltB5704:0.0,
UltB5705:0.0,UltB5708:0.0,UltB5709:0.0,UltB5716:0.0,UltB5727:0.0,UltB5729
:0.0,UltB5755:0.0,UltB5757:0.0):5.5E-4)0.506.3:5.5E-
4,UltB5702:0.00369)0.270.1:5.3E-4,UltB5717:0.01115)0.000.177:5.5E-
4,UltB5779:0.00369)0.485.2:5.5E-4,UltB5713:0.00369)0.000.178:5.5E-
4,(UltB5718:0.01496,UltB5714:0.00369)0.805.9:5.3E-4)0.000.179:5.5E-
4,UltB5754:0.00369)0.591.4:5.5E-
4,(UltB5642:0.07805,UltB5634:0.00737)0.758.12:0.0037)0.081.1:5.5E-
4,UltB5719:0.00367)0.386.1:5.5E-4,UltB5636:5.5E-4)0.621.4:5.5E-
4,(UltB5635:0.02236,((((((((UltB5763:0.00369,UltB5683:0.00369)0.865.12:5
.5E-
4,(((UltB5770:0.05308,UltB5696:0.00823)0.745.7:0.00297,UltB5693:0.01116)0
.895.17:5.5E-4,UltB5776:0.00369)0.849.10:5.5E-4)0.000.180:5.3E-
4,(UltB5639:0.01116,((UltB5767:0.02714,(UltB5751:0.0313,Ult19279:0.02696)
0.846.9:0.00743)0.917.13:0.01499,UltB5761:5.4E-
4)0.361.2:0.00368)0.900.11:5.4E-4)0.000.181:5.5E-
4,(UltB5654:0.0074,UltB5760:0.00369)0.902.15:5.5E-4)0.000.182:5.5E-
4,(UltB5769:0.00369,(UltB5657:0.0,UltB5689:0.0):0.00369)0.920.13:5.5E-
4)0.000.183:5.5E-
4,((UltB5641:0.0,UltB5649:0.0,UltB5652:0.0,UltB5653:0.0,UltB5655:0.0,UltB
5656:0.0,UltB5658:0.0,UltB5663:0.0,UltB5664:0.0,UltB5684:0.0,UltB5687:0.0
,UltB5680:0.0,UltB5679:0.0,UltB5688:0.0,UltB5691:0.0,UltB5694:0.0,UltB571
2:0.0,UltB5720:0.0,UltB5721:0.0,UltB5740:0.0,UltB5749:0.0,UltB5752:0.0,Ul
tB5753:0.0,UltB5759:0.0,UltB5762:0.0,UltB5764:0.0,UltB5766:0.0,UltB5773:0
.0,UltB5774:0.0,UltB5775:0.0,UltB5777:0.0,UltB5778:0.0):5.5E-

4,UltB5678:5.5E-4)0.000.184:5.5E-4)0.865.13:5.5E-
4,((UltB5710:0.01119,UltB5692:0.01878)0.622.3:0.00374,UltB5578:0.02301)0.
878.12:5.4E-4)0.321.2:5.5E-
4,UltB5768:0.00369)0.857.14:0.00367,((UltB5771:0.0,UltB5802:0.0):5.5E-
4,(UltB5643:0.00369,UltB5711:0.00369)0.691.2:5.5E-4)0.877.16:5.5E-
4)0.987.9:0.00201)0.987.10:0.00179)0.435.1:5.4E-
4,((UltB5706:0.02214,(UltB5677:0.00368,UltB5715:0.00741)0.785.17:0.00369)
0.937.12:0.00237,UltB5579:0.00249)0.937.13:0.00237)0.256.1:5.4E-
4)0.104.1:0.00718)0.919.15:0.01078,UltB5801:0.02688)0.766.8:0.00367)0.886
.16:0.01087,UltB5794:0.02085)0.190.1:0.00894)0.943.16:0.03381)0.703.6:0.0
0601)0.890.17:0.01284,(UltB5552:0.03213,UltB5553:0.00264)0.943.17:0.01571
)0.869.13:0.00847)0.853.11:0.01033,((UltB5560:0.07096,((UltB5389:0.0063,(
UltB5387:0.0356,UltB5388:0.02677)0.551.3:0.00517)0.959.13:0.02759,(UltB53
94:0.0518,(UltB5393:5.5E-
4,UltB5392:0.00368)0.985.12:0.05153)0.931.15:0.02566)0.813.7:0.00991)0.76
7.9:0.00449,(((UltB5298:0.0,UltB5299:0.0):0.03496,(UltB5537:0.02101,(UltB
5536:0.00377,UltB5539:0.00364)0.982.3:0.04981)0.973.7:0.0415)0.968.6:0.03
979,(UltRu116:0.06864,(PreBryan:0.05703,((UltB5525:0.01486,(UltB5523:5.5E
-
4,UltB5524:0.043)0.897.8:0.02087)0.968.7:0.04663,((UltB5390:0.09063,UltB5
588:0.03461)0.857.15:0.02093,(UltB5300:0.06913,PreBucca:0.03838)0.820.4:0
.02361)0.809.14:0.03342)0.870.10:0.02681)0.748.9:0.00626)0.755.11:0.00712
)0.864.16:0.01274)0.821.9:0.00882)0.843.8:0.00806)0.909.9:0.01169)0.809.1
5:0.00682,((UltB5374:0.04997,(((UltB5549:0.0,UltB5551:0.0):5.5E-
4,UltB5550:0.00369)0.842.17:0.01015,(((UltB5548:0.03502,UltB5547:0.00596)
0.810.10:0.00828,((UltB5538:0.0,UltB5541:0.0,UltB5543:0.0,UltB5544:0.0,Ul
tB5545:0.0,UltB5748:0.0):5.5E-
4,UltB5542:0.01486)0.947.12:0.01817)0.754.13:0.00716,UltB5546:0.05398)0.7
70.16:0.02825)0.724.6:0.025)0.857.16:0.016,(((((UltB5448:5.5E-
4,UltB5449:0.13623)0.966.14:0.01814,(UltB5446:0.00742,UltB5447:5.5E-
4)0.991.12:0.03379)0.761.14:0.0039,((UltB5445:0.03384,(UltB5444:0.0553,((
UltB5825:0.0,UltB5958:0.0):0.02299,UltB5826:0.04253)0.942.8:0.03298)0.678
.5:0.02022)0.913.14:0.01953,((UltB5822:0.0,UltB5823:0.0,UltB5821:0.0):0.0
1393,(UltB5557:0.00739,UltB5558:5.5E-
4)0.981.5:0.02864)0.755.12:0.00436)0.776.9:0.00545)0.888.17:0.01901,UltB5
450:0.02553)0.960.12:0.02187,UltB5423:0.02624)0.906.15:0.02445)0.731.3:0.
00295)0.900.12:0.00729,(((UltB5452:5.3E-4,(UltB5404:0.0037,UltB5451:5.5E-
4)0.910.22:0.00748)0.830.13:0.00744,(UltB5453:0.00376,UltB5455:0.0076)0.7
77.11:0.00368)1.000.88:5.4E-4,(UltB5454:0.00744,((((UltB5434:5.5E-
4,(UltB5433:0.0074,(UltB5430:5.5E-
4,UltB5431:0.00369)0.880.16:0.00369)0.687.4:5.5E-4)0.981.6:5.5E-
4,UltB5432:0.0187)0.962.16:0.02691,UltB5429:0.05243)0.369.1:0.00873,((Ult
B5440:5.5E-
4,(UltB5437:0.01495,(UltB5436:0.00368,(UltB5438:0.0,UltB5439:0.0,UltB5441
:0.0):5.5E-
4)0.952.19:0.0019)0.948.15:0.00201)0.883.12:0.01631,(UltB5442:0.0,UltB554
0:0.0):0.04025)0.739.14:0.01018)0.900.13:0.01519)0.982.4:5.5E-
4)0.773.16:0.00373)0.818.7:0.00766)0.937.14:0.01604)0.631.1:0.00121,((Ult
B5406:0.0,UltB5407:0.0):0.02491,((((UltB5428:0.03317,(UltB5532:0.036,(Ult
B5530:0.03441,((UltB5528:0.0,UltB5531:0.0,UltB5533:0.0,UltB5534:0.0):5.5E
-4,UltB5529:0.00741)1.000.89:5.3E-
4)0.847.16:0.01182)0.874.15:0.02017)0.712.3:0.02593,(((UltB5527:0.03332,(
UltB5587:0.00185,UltPrev6:0.00187)0.998.12:0.112)0.958.15:0.04239,(((UltB
5494:5.5E-4,(UltB5495:0.0,UltB5498:0.0):5.5E-

4)0.987.11:0.02688,((UltB5497:5.4E-
4,UltB5946:0.02713)0.819.15:0.00369,UltB5496:5.4E-
4)0.784.11:0.00541)0.764.13:0.00534,UltB5499:0.00834)0.749.18:0.00647)0.9
34.11:0.02457,(UltB5492:0.05584,((((UltB5489:5.4E-
4,UltB5490:0.00358)0.975.10:0.06511,UltB5491:0.06957)0.878.13:0.02647,(Ul
tB5493:0.06959,(UltB5526:0.08788,PreGenom:0.06314)0.884.14:0.02748)0.938.
11:0.03261)0.867.16:0.01471,(UltB5397:0.0567,UltB5500:0.07367)0.817.4:0.0
1516)0.036.1:0.01853)0.378.4:5.4E-
4)0.799.11:0.01532)0.920.14:0.03324,(UltB5501:0.00348,UltB5945:0.01918)0.
911.19:0.01533)0.599.3:0.01003,(UltB5502:0.05106,(UltB5435:0.08234,(UltB5
427:0.02989,((PreSpec4:0.05346,PreEnoec:0.03164)0.991.13:0.05995,UltRu129
:0.0333)0.896.11:0.02924)0.271.2:0.01271)0.446.1:0.01721)0.896.12:0.02243
)0.750.5:0.00436)0.942.9:0.02905)0.937.15:0.04108)0.992.13:0.06789,((((((
(((UltB6197:0.01528,((((((UltB6072:0.01127,((UltB6042:0.01491,(UltB6066:
0.01129,((((UltB6009:0.0,UltB6015:0.0,UltB6029:0.0,UltB6041:0.0,UltB6051:
0.0,UltB6054:0.0,HumanGu4:0.0,UltB6043:0.0,UltB6046:0.0,BcrDorei:0.0,UltB
6058:0.0,UltB6064:0.0,HumanGu5:0.0,UltB6065:0.0,UltB6067:0.0,UltB6068:0.0
,UltB6071:0.0,UltB6393:0.0,UltB6396:0.0,UltB7340:0.0,UltB7365:0.0,Ult1925
9:0.0):5.5E-
4,(((UltB6070:0.01116,(((UltB5978:0.01117,UltB6036:0.01881)0.786.7:0.0036
9,(((UltB6008:0.01465,(UltB5989:5.5E-4,UltB6028:5.5E-
4)0.944.9:0.00202)0.934.12:0.0019,((((((((((UltB5975:5.5E-
4,((UltB6035:0.00742,(((BacNLA79:5.5E-4,UltB6394:0.00742)0.000.185:5.5E-
4,(UltB5988:0.047,UltB6022:0.0037)0.889.15:0.00741)0.312.1:5.5E-
4,((UltB5982:5.5E-
4,UltB6094:0.00741)0.940.10:0.01104,UltB6395:0.00202)0.939.11:0.00199)0.2
75.4:5.5E-4)0.466.5:5.4E-
4,(UltB6006:0.0,UltB6007:0.0,UltB6016:0.0,UltB6019:0.0,UltB6020:0.0,UltB6
021:0.0,UltB5980:0.0,UltB5979:0.0,UltB5974:0.0,BcrVulga:0.0,UltB6025:0.0,
UltB6026:0.0,UltB6027:0.0,UltB6031:0.0,BcrVulg2:0.0,UltB6032:0.0,UltB6040
:0.0,BacNLA75:0.0,BacNLA78:0.0,UltB6050:0.0,UltB6053:0.0,UltB6062:0.0,Ult
B6061:0.0,UltB7337:0.0,UltB7338:0.0,Ult19254:0.0):5.5E-4)0.498.2:5.5E-
4)0.000.186:5.5E-
4,(UltB6023:0.05514,(UltB5977:0.03461,UltB6044:0.00724)0.877.17:0.00758)0
.783.12:0.00397)0.654.2:5.5E-4,(UltB6039:0.02696,UltB6038:5.4E-
4)0.838.12:0.00369)0.000.187:5.5E-4,(UltB7335:0.01534,UltB7334:5.4E-
4)0.845.11:0.00378)0.581.4:5.5E-
4,(UltB7364:0.0,UltB7631:0.0):0.00369)0.304.1:5.4E-4,UltB6017:5.5E-
4)0.450.2:5.5E-4,UltB6417:0.00369)0.000.188:5.5E-
4,UltB6037:0.05086)0.000.189:5.5E-4,UltB5976:0.00369)0.000.190:5.5E-
4,BacNLA74:0.00369)0.000.191:5.5E-4,UltB5981:0.00741)0.107.5:5.5E-
4)0.000.192:5.5E-4,BacNLA77:5.5E-4)0.082.1:5.5E-4)0.057.2:5.5E-
4,BacNLA76:5.5E-4)0.831.15:0.00415)0.059.2:5.5E-4,UltB6059:5.5E-
4)0.015.1:5.5E-
4,(UltB6069:0.00164,UltB6063:0.0398)0.989.12:0.00216)0.380.1:5.5E-
4)0.000.193:5.5E-4,UltB6057:0.00369)0.000.194:5.5E-
4,UltB6045:0.01124)0.000.195:5.5E-4)0.478.2:5.5E-4)0.000.196:5.5E-
4,UltB6060:0.00372)0.178.1:5.5E-4)0.211.1:5.5E-4,UltB7336:5.5E-
4)0.697.1:5.5E-4,(UltB6056:0.04437,(BcrBarne:5.4E-
4,UltB6209:0.00719)0.988.14:0.04414)0.914.14:0.02142)0.820.5:0.00434,UltB
5839:0.03201)0.779.11:5.5E-
4,((UltB6052:0.02305,(UltB5985:0.00368,(UltB6030:0.0227,UltB5987:5.4E-
4)0.832.10:0.00724)0.907.17:0.01157)0.904.12:0.01152,(UltB6080:0.02555,(U
ltB6078:5.5E-

4,UltB6079:0.00369)0.794.13:0.00661)0.941.11:0.01706)0.691.3:0.00789)0.86
9.14:0.00927,((UltB6004:0.0037,((UltB5834:0.0037,UltB5993:5.5E-
4)0.961.11:0.01117,(((UltB5992:0.00367,UltB6002:5.4E-
4)0.730.5:0.00368,(UltB5994:0.00729,(UltB5996:0.00384,UltB5990:0.00729)0.
452.3:0.00371)0.902.16:5.1E-4)0.811.11:5.3E-
4,((UltB5832:0.0,UltB5833:0.0,UltB5991:0.0,UltB5997:0.0,UltB6005:0.0,Ult1
9255:0.0):5.5E-4,UltB5995:0.00369)0.761.15:5.5E-4)0.774.21:5.5E-
4)0.795.14:0.00369)0.807.7:0.0037,((UltB5998:0.0,UltB6003:0.0):5.5E-
4,((UltB6000:0.00743,(UltB6001:0.00369,Ult19257:5.5E-4)0.699.7:5.5E-
4)0.859.12:0.0037,UltB5999:0.00743)0.850.11:5.4E-4)0.883.13:5.4E-
4)0.733.6:0.00284)0.995.16:0.0351,(UltB6212:0.00741,UltB6213:0.00373)0.75
9.13:0.00399)0.739.15:0.00398)0.872.21:0.01381,(UltB6196:0.04974,(UltB619
8:0.00369,((BcrCopro:0.0,UltB6201:0.0,UltB6202:0.0):5.5E-
4,(UltB6195:0.02431,UltB6194:0.00369)0.629.3:5.5E-4)0.398.3:5.5E-
4)0.615.4:5.4E-
4)0.850.12:0.01127)0.835.12:0.00951,((((Ult19265:0.01142,(UltB6014:0.0112
3,UltB6223:0.00376)0.962.17:0.01932)0.855.10:0.00763,((UltB6210:0.00743,U
ltB6211:5.5E-
4)0.957.11:0.00173,(BacteNe4:0.00771,((UltB6208:0.0,BcrCopr2:0.0):5.3E-
4,UltB6459:0.01502)0.988.15:0.02364)0.940.11:0.00199)0.939.12:0.01503)0.8
42.18:0.00784,((UltB6193:0.00412,(UltB6191:5.5E-4,UltB6192:5.5E-
4)0.789.8:0.00332)0.998.13:0.04022,(((((((UltB6186:0.0,UltB6188:0.0,UltB6
190:0.0,UltB7214:0.0):5.5E-4,UltB6185:0.00369)0.680.1:5.3E-
4,UltB6187:0.02653)0.386.2:0.00368,UltB6179:0.02266)1.000.90:5.5E-
4,(UltB6112:0.0,UltB6168:0.0,UltB6169:0.0,UltB6170:0.0,UltB6171:0.0,UltB6
172:0.0,UltB6173:0.0,UltB6174:0.0,UltB6175:0.0,UltB6176:0.0,UltB6177:0.0,
UltB6178:0.0,UltB6180:0.0,UltB6181:0.0,UltB6182:0.0,UltB6184:0.0,UltB6183
:0.0,UltB6189:0.0,BcrPlebe:0.0):5.5E-4)0.995.17:5.5E-
4,((UltB6200:0.01846,((BcrTectu:0.01783,(BacNL106:5.5E-
4,(BacNL107:0.00372,BcrPyoge:0.00741)0.299.2:5.5E-
4)0.860.9:0.00954)0.944.10:0.03113,(UltB6465:0.01434,UltB6464:0.0052)0.92
3.14:0.02511)0.934.13:0.02859)0.925.23:0.01998,((UltB6115:0.01135,UltB616
7:0.00755)0.744.9:0.00371,(UltB6166:0.01594,(UltB6165:5.5E-
4,(UltB6086:0.00751,(UltB6134:5.5E-
4,UltB6164:0.00369)0.892.13:0.00747)0.947.13:0.01902)0.242.3:0.0072)0.867
.17:0.01174)0.890.18:0.01445)0.777.12:0.00835)0.972.11:0.01525,(UltB6203:
0.00588,UltB6214:0.02278)0.781.13:0.0057)0.880.17:0.00794)0.388.5:0.00371
)0.877.18:0.01224,((((UltB6462:0.05706,(UltB6461:0.02625,UltB6460:0.01517
)0.329.3:0.0045)0.750.6:0.005,(UltB6448:0.04391,UltB6215:0.00418)0.960.13
:0.02179)0.786.8:0.00916,(((UltB6470:0.00368,UltB6471:5.5E-
4)0.980.6:5.3E-4,((((((UltB6435:0.00368,UltB6436:5.5E-4)0.806.4:5.5E-
4,((UltB6262:0.0,UltB6437:0.0):5.5E-
4,UltB6216:0.00369)0.951.14:0.00744)0.758.13:0.00577,((UltB6433:0.0,UltB6
434:0.0):0.00832,GutMeta6:0.01516)0.777.13:0.00563)0.955.13:0.02324,(UltB
6438:0.01971,(UltB6430:0.01879,UltB6431:5.5E-
4)0.874.16:0.0077)0.303.2:5.4E-
4)0.931.16:0.01249,UltB6439:0.01748)0.786.9:0.00751,UltB6473:0.00517)0.93
9.13:0.01444)0.757.8:0.00525,(BcrHelco:0.03918,(UltB7328:0.00742,(UltB640
7:0.00369,UltB6472:5.5E-4)0.960.14:5.5E-
4)0.908.11:0.01393)0.746.10:0.00475)0.888.18:0.01598)0.906.16:0.01563,(((
UltB6466:0.0,UltB6467:0.0,UltB6468:0.0):0.03073,((BcrSalan:0.00696,(UltB6
447:0.03055,((UltB6445:0.0,UltB6449:0.0):5.5E-
4,UltB6450:0.00741)0.882.8:5.4E-
4)0.969.16:0.02392)0.942.10:0.02808,(UltB6455:0.00843,(UltB6453:0.00746,(

(UltB6452:0.0,UltB6454:0.0):5.5E-
4,(UltB6446:0.00369,UltB6451:0.00375)0.660.2:5.5E-4)0.970.13:5.4E-
4)0.410.3:0.01516)0.992.14:0.05429)0.730.6:0.0086)0.231.3:0.00977,(UltB64
76:0.01811,UltB6477:0.03927)0.937.16:0.02859)0.805.10:0.00945)0.881.16:0.
01244)0.938.12:0.02335)0.961.12:0.02791,((UltB6154:0.03926,UltB6161:0.034
27)0.451.6:0.00513,(((((UltB6133:0.00744,(((UltB6146:5.5E-
4,(UltB6143:0.0037,(UltB6145:5.5E-4,(UltB6129:5.5E-
4,((UltB6113:0.0,UltB6125:0.0,UltB6147:0.0):5.5E-
4,UltB6148:0.00741)0.789.9:5.5E-4)0.417.2:5.5E-
4)0.857.17:0.00369)0.375.2:5.3E-4)0.952.20:5.5E-
4,(UltB6120:0.0434,UltB6126:0.01722)0.928.13:0.01785)0.839.8:0.00367,UltB
6144:5.4E-4)0.711.5:5.4E-
4)0.949.17:0.01547,(((UltB6117:0.0,UltB6132:0.0,UltB6150:0.0):5.5E-
4,UltB8147:0.00744)0.924.16:5.5E-4,(UltB6111:5.5E-
4,UltB6118:0.03097)0.905.12:0.0072)0.829.10:0.00684)0.934.14:0.01394,((Ul
tB6156:0.00408,((UltB6163:0.01493,UltB6162:5.5E-
4)0.944.11:0.01523,(UltB6149:0.09813,((UltB6130:0.01496,(UltB6138:0.00369
,((UltB5911:0.0,UltB6122:0.0,UltB6127:0.0,UltB6142:0.0):5.5E-
4,(UltB6137:0.00369,UltB6141:0.00369)0.706.4:5.5E-4)0.881.17:5.4E-
4)0.340.2:5.3E-4)0.912.17:0.00742,((UltB6139:5.5E-
4,UltB6136:0.00741)0.857.18:0.00369,(((UltB6119:0.0,UltB6121:0.0,UltB6128
:0.0,UltB6131:0.0,UltB6140:0.0,UltB6151:0.0):5.5E-
4,(UltB6110:0.00369,(UltB6114:0.0,UltB6123:0.0,UltB6135:0.0):0.00742)0.92
1.22:5.5E-4)0.737.13:5.5E-4,UltB6159:5.5E-4)0.830.14:5.4E-4)0.604.3:5.5E-
4)0.387.2:5.5E-
4)0.749.19:0.00741)0.934.15:0.01899)0.135.2:0.00365,(UltB6158:0.01016,(((
UltB6116:0.0,UltB6155:0.0):5.5E-4,UltB6153:0.00369)0.729.5:5.5E-
4,(UltB6152:0.01096,UltB6157:0.01105)0.915.15:5.3E-
4)0.787.14:0.00521)0.978.6:0.02326)0.960.15:0.01918)0.519.2:5.4E-
4,UltB6160:0.02041)0.883.14:0.01304,((UltB6474:0.00753,UltB6475:5.4E-
4)0.845.12:0.02696,BcdBacte:0.17381)0.933.15:0.04538)0.765.9:0.00366)0.95
5.14:0.02455)0.935.18:0.02447,UltB6469:0.01221)0.948.16:0.02497,UltB6463:
0.02224)0.849.11:0.01601,(UltRu139:0.03235,UltB6443:0.03215)0.998.14:0.07
311)0.776.10:0.01501,(((UltB6206:0.0,UltB6205:0.0,UltB6207:0.0):5.5E-
4,UltB6204:5.5E-
4)0.988.16:0.06337,(UltB6442:0.00186,(UltB6440:0.01203,UltB6441:0.0031)0.
971.7:0.05384)0.625.1:0.0329)0.994.16:0.0898)0.914.15:0.03003,(((PreTanne
:5.5E-
4,UltB5850:0.01489)1.000.91:0.11158,((((UltB5969:0.0,UltB5970:0.0):0.0047
8,UltB5968:0.08485)0.936.15:0.03617,(UltB5881:0.08367,UltB5928:0.03242)0.
884.15:0.03569)0.845.13:0.02604,UltB5503:0.12555)0.000.197:0.0028)0.939.1
4:0.0428,((((UltRu123:0.0385,(UltB5480:0.05606,(UltB5478:0.03355,(UltB547
9:0.03582,(UltB5476:5.5E-
4,(UltB5475:0.03077,UltB5477:0.1418)0.824.15:5.5E-4)0.998.15:5.4E-
4)0.874.17:0.02797)0.790.4:0.02156)0.329.4:0.01323)0.833.15:0.02072,(((Ul
tB5485:0.03212,UltB5486:0.0078)0.791.10:0.0236,(UltB5487:0.0148,UltB5488:
0.06358)0.679.2:0.04428)0.949.18:0.05012,(UltB5481:0.09186,(UltB5471:0.02
485,(UltB5470:0.01645,((UltB5469:0.04386,(UltB5467:5.5E-
4,UltB5468:0.00724)0.944.12:0.03466)0.396.4:0.01377,((UltB5472:0.03458,(U
ltRu121:0.04317,(UltB5465:0.00751,(UltB5824:5.5E-
4,(UltB5464:0.00375,UltB5466:0.00376)0.655.3:5.4E-4)0.938.13:5.3E-
4)0.865.14:0.01348)0.770.17:0.01424)0.589.4:0.00219,(((UltB5462:0.0,UltB5
463:0.0):5.4E-
4,UltB5461:0.00373)0.947.14:0.01958,(UltRu122:0.01699,((UltRu120:0.0114,U

ltB5474:5.4E-
4)0.245.1:0.00755,UltRu260:0.05862)0.677.4:0.00786)0.864.17:0.01235)0.968
.8:0.05582)0.967.11:0.03825)0.975.11:0.05787)0.989.13:0.06537)0.554.1:0.0
1612)0.955.15:0.05288)0.613.4:0.03194)0.853.12:0.02334,(UltRu124:0.05104,
(UltB5483:0.04451,UltB5482:0.09724)0.883.15:0.02088)0.786.10:0.01278)0.90
0.14:0.03761,UltB5484:0.09242)0.910.23:0.04432)0.862.14:0.01888)0.206.2:0
.00449)0.892.14:0.01939,(UltB5971:0.12897,(UltB5972:0.09578,(UltB5955:0.1
0128,(((UltB5956:0.07094,UltB5957:5.5E-
4)0.158.1:0.0033,(UltB5953:0.01118,UltB5960:0.00394)0.888.19:0.01208)0.91
1.20:0.01523,(UltRu138:0.00771,UltB5954:0.0272)0.866.12:0.00931)0.778.14:
5.3E-
4)0.947.15:0.04219)0.849.12:0.02924)0.753.10:0.01405)0.948.17:0.02957)0.9
10.24:0.01792)0.799.12:0.01105)0.887.13:0.01826)0.856.7:0.01842)0.943.18:
0.0367,((((((UltB6512:0.0,UltB6510:0.0):5.5E-
4,UltB6511:0.00745)0.901.16:0.0231,(((UltB6519:0.0,UltB6520:0.0):0.05722,
(UltB6522:0.04847,(UltB6730:0.00373,UltB6521:0.00373)0.888.20:5.5E-
4)0.828.9:0.0184)0.433.4:0.02855,(((UltRu143:0.01674,UltB6515:0.03055)0.8
53.13:0.01153,(UltRu144:5.5E-4,(UltB6516:5.4E-
4,(UltB6517:0.0,UltB6518:0.0):0.01923)0.919.16:0.01122)0.895.18:0.00798)0
.866.13:0.00759,((UltB5511:0.04917,UltB6514:5.4E-
4)0.769.13:0.00353,UltB6513:0.00766)0.931.17:0.01195)0.716.4:0.00331)0.87
6.12:0.02738)0.462.3:0.02182,((UltB6523:0.02593,UltB6509:0.02835)0.901.17
:0.01765,(((UltB6526:0.0,UltB6527:0.0):0.02887,((UltB6497:0.01896,(UltB65
04:0.00372,((UltB6727:0.0,UltB6729:0.0,UltB6498:0.0,UltB6499:0.0,UltB6500
:0.0,UltB6502:0.0,UltB6501:0.0,UltB6503:0.0,UltB6505:0.0,UltB6506:0.0):5.
5E-
4,((UltB6728:0.00183,UltB7718:0.05964)1.000.92:0.00191,UltB6507:0.03952)0
.970.14:5.5E-4)0.170.1:5.5E-4)0.916.20:5.5E-
4)0.866.14:0.01893,UltB6508:0.01809)0.723.6:0.00636)0.900.15:0.01353,(Ult
Ru145:0.01334,(UltB6525:0.03118,UltRu146:5.5E-
4)0.783.13:0.00608)0.990.14:0.03702)0.774.22:0.00762)0.896.13:0.02401)0.8
43.9:0.01892,(UltB6524:0.05012,UltB6535:0.0538)0.929.12:0.03072)0.906.17:
0.02959,((((UltB6531:5.5E-
4,(UltB6613:0.00372,(UltB6615:0.00371,UltB6532:5.5E-4)0.073.1:5.5E-
4)0.841.12:0.00371)0.987.12:0.03348,(UltB6530:0.03783,(UltB6528:0.02072,U
ltB6529:0.01143)0.927.10:0.02736)0.723.7:0.01876)0.613.5:0.0088,(UltB6533
:0.09363,UltB6496:0.09734)0.314.1:0.01352)0.936.16:0.03492,(((UltB6540:0.
0,UltB6541:0.0,UltB6542:0.0,UltB6543:0.0):5.3E-
4,Ult20675:0.08615)0.998.16:0.07926,(((((((UltB6623:0.0,UltB6624:0.0,Ult
B6625:0.0):0.03987,(UltB6622:0.03615,(UltB6621:0.06566,(UltB6627:0.0,UltB
6620:0.0):5.4E-
4)0.890.19:0.02696)0.980.7:0.04932)0.976.8:0.04209,((UltB6577:0.02656,(((
(UltB6579:0.07437,(UltB6578:0.01114,((UltB6582:0.0,UltB6580:0.0):5.5E-
4,UltB6572:0.00373)0.766.9:5.5E-4)0.405.4:5.4E-
4)0.824.16:0.00999,UltB6581:0.03106)0.955.16:0.02828,(UltB6596:0.02754,(U
ltB6575:0.00362,UltB6576:0.0154)0.980.8:0.02955)0.590.1:0.00244)0.941.12:
0.02176,((UltB6583:0.01581,(UltB6584:0.00763,UltB6585:0.00769)0.795.15:0.
00749)0.893.20:0.03257,((UltB6588:0.00366,((UltB6637:0.0,UltB6659:0.0,Ult
B6591:0.0,UltB6589:0.0):5.5E-
4,(UltB7001:0.00372,UltB6592:0.00372)0.649.5:5.5E-4)0.612.2:5.5E-
4)0.987.13:0.05039,((UltB6593:0.0,UltB6594:0.0):5.4E-
4,UltB6991:0.01893)0.761.16:0.01713)0.655.4:0.03513)0.047.2:0.00286)0.614
.1:0.00373)0.901.18:0.01612,(((UltB7038:0.0534,((UltB7034:0.0,UltB7037:0.
0,UltB7035:0.0,UltB7036:0.0,UltB6975:0.0,UltB6976:0.0,UltB6690:0.0):5.4E-

4,UltB7516:0.01535)0.791.11:0.00758)0.993.9:0.04973,((UltB7027:0.00756,((
UltB7030:0.0,UltB7029:0.0,UltB7026:0.0,UltB7031:0.0,UltB7028:0.0):5.5E-
4,UltB7032:0.01126)0.468.3:5.4E-
4)0.875.17:0.02287,(UltB7022:0.06017,(UltB7023:5.5E-
4,UltB7024:0.00373)0.878.14:5.5E-
4)0.859.13:0.02422)0.000.198:0.01679)0.946.13:0.03578,((UltB7183:0.02581,
(UltB7175:0.02007,UltB7176:0.05009)0.625.2:0.01955)0.940.12:0.02871,UltB6
974:0.07856)0.644.1:0.0071)0.317.2:0.01078)0.802.10:0.00709)0.806.5:0.004
02,(((UltB7123:0.01987,UltB7124:0.00368)0.786.11:0.00727,(UltB6720:0.0151
2,((UltB7130:0.0,UltB7131:0.0,UltB7132:0.0):5.5E-
4,(UltB7133:0.01545,((UltB6813:5.5E-
4,UltB6822:0.0152)0.838.13:0.00372,((UltB7129:0.0,UltB7128:0.0,UltB7060:0
.0,UltB7135:0.0,UltB6999:0.0,UltB7137:0.0,UltB6739:0.0,UltB7134:0.0):5.5E
-4,((UltB7126:5.5E-
4,UltB7127:0.04317)0.948.18:0.01124,UltB7071:0.00372)0.767.10:5.5E-
4)0.825.9:5.5E-4)0.709.2:5.5E-4)0.839.9:0.00372)0.795.16:5.4E-
4)0.959.14:0.02052)0.788.9:0.00802,((UltB7101:0.04162,(((UltB7109:0.0,Ult
B7110:0.0,UltB7108:0.0,UltB7107:0.0):0.03623,UltB7122:0.01512)0.876.13:5.
3E-
4,((UltB7102:0.02376,((UltB6986:0.02339,(UltB7085:0.01888,((UltB7084:0.00
371,((UltB7086:0.0,UltB7088:0.0):5.5E-
4,((UltB7081:0.03597,UltB7083:0.06244)0.824.17:0.00692,(UltB7082:0.03049,
(UltB7080:0.06878,UltB7079:0.03298)0.713.6:0.01579)0.445.2:5.4E-
4)0.525.1:5.5E-4)0.000.199:5.5E-4)0.164.1:5.5E-
4,UltB7087:0.00373)0.375.3:5.5E-
4)0.745.8:0.0038)0.872.22:0.01158,UltB7125:0.04533)0.766.10:0.00775)0.801
.11:5.3E-
4,(UltB7103:0.00393,((((UltB7106:0.00206,UltB7104:0.00706)0.909.10:0.0020
4,(UltB7098:0.00379,(UltB7095:0.0075,(UltB7097:0.00372,(UltB7099:0.0,UltB
7096:0.0):5.5E-4)0.908.12:5.4E-
4)0.761.17:0.00372)0.977.9:0.01565)0.793.14:0.0039,UltB7105:0.00373)1.000
.93:5.4E-
4,UltB7111:0.0111)0.412.2:0.01147)0.888.21:0.00741)0.918.20:0.00765)0.797
.13:0.00874)0.457.3:0.01023,(((UltB6619:0.01273,(UltB6616:0.02108,(UltB66
17:0.01136,UltB6618:5.5E-
4)0.778.15:0.00732)0.975.12:0.0407)0.989.14:0.04674,(((UltB7090:0.0,UltB7
089:0.0):5.5E-4,(UltB7091:0.0241,(UltB7092:0.00748,UltB6732:5.5E-
4)0.903.10:0.01184)0.799.13:0.01143)0.772.15:0.00536,((UltB7078:0.02708,U
ltB7174:5.4E-
4)0.441.4:0.00373,((UltB7148:0.01915,UltB6721:0.01137)0.767.11:0.00364,((
UltB7073:0.0,UltB7074:0.0,UltB7076:0.0,UltB7075:0.0,UltB7049:0.0):5.5E-
4,(UltB7077:0.03647,UltB7152:0.00372)0.368.1:5.5E-4)0.632.2:5.5E-
4)0.767.12:0.00385)0.881.18:0.01139)0.508.2:0.00633)0.773.17:0.00802,(Ult
B6587:0.07608,UltB6586:0.01593)0.890.20:0.01674)0.943.19:0.02139)0.794.14
:0.00672)0.369.2:0.00918)0.999.17:5.4E-
4,(((((UltB6684:0.00409,((UltB7093:0.07425,UltB7094:0.00662)0.954.13:0.02
983,(((UltB6611:0.02661,(UltB6661:0.0,UltB6660:0.0):0.02667)0.959.15:0.03
397,(((UltB6700:0.02684,UltB6695:0.06021)0.743.12:0.00373,(((UltB6702:0.0
,UltB6691:0.0,UltB6694:0.0,UltB6696:0.0,UltB7253:0.0):5.5E-
4,(UltB6697:0.00746,(((UltB6685:5.5E-
4,UltB6701:0.02292)0.790.5:0.00373,UltB7355:0.0075)0.821.10:0.00362,UltB6
699:0.3521)0.999.18:5.4E-4)0.253.2:5.5E-4)0.463.3:5.5E-
4,UltB6692:0.00749)0.116.3:5.4E-
4)0.772.16:0.00367,UltB6698:0.00756)0.823.7:0.01239)0.762.13:0.0097,((Ult

B6682:0.00747,UltB6683:5.5E-
4)0.890.21:0.01746,(UltB6673:0.05248,(UltB6671:0.03906,(UltB6804:0.00749,
((UltB6731:0.0,UltB6680:0.0,UltB6677:0.0,UltB6675:0.0,UltB6678:0.0,UltB66
76:0.0,UltB6672:0.0,UltB6681:0.0):5.5E-4,UltB6674:0.00373)0.402.3:5.5E-
4)0.745.9:0.00309)0.625.3:5.4E-
4)0.964.20:0.02646)0.937.17:0.02789)0.776.11:0.01071)0.843.10:0.01146)0.9
27.11:0.01771,(UltB6733:0.05293,(UltB6603:0.01474,(UltB6604:5.5E-
4,(UltB6605:0.0,UltB6609:0.0):0.01129)0.877.19:0.00569)0.723.8:0.00496)0.
860.10:0.01268)0.880.18:0.01324,((UltB6803:5.5E-
4,((UltB7046:0.02396,UltB7045:0.01083)0.970.15:0.02419,(((UltB6788:5.5E-
4,((UltB6784:0.00372,(((UltB6787:5.5E-4,((UltB6796:5.5E-
4,UltB6791:0.0115)0.846.10:0.00373,UltB6800:0.01502)0.183.2:5.5E-
4)0.000.200:5.5E-4,(UltB6793:0.00787,UltB6789:0.04338)0.945.15:5.4E-
4)0.043.2:5.5E-4,(UltB6795:0.00372,UltB6799:0.00372)0.000.201:5.5E-
4)0.210.2:5.5E-4)0.000.202:5.5E-
4,(UltB6722:0.0,UltB6801:0.0,UltB6802:0.0,UltB6782:0.0,UltB6797:0.0,UltB6
792:0.0,UltB6794:0.0):5.5E-4)0.498.3:5.5E-4)0.076.2:5.5E-
4,((UltB6806:5.4E-
4,(UltB6783:0.06982,UltB6790:0.04281)0.838.14:0.00783)1.000.94:0.00151,Ul
tB6798:0.04304)1.000.95:0.00222)0.386.3:5.4E-
4,(UltB6786:0.00217,UltB6785:0.03102)0.983.10:0.00166)0.878.15:0.00791)0.
778.16:0.00371)0.758.14:0.00402,(UltB6996:0.01978,(UltB7015:0.00372,(UltB
7016:0.00372,(UltB6934:0.0,UltB7014:0.0,UltB7017:0.0):5.5E-
4)0.061.2:5.5E-
4)0.851.14:0.00751)0.877.20:0.0116)0.771.18:0.00552)0.936.17:0.01703,(((U
ltB6600:0.01342,UltB6599:0.04538)0.865.15:0.01404,(((UltB7004:0.03006,(Ul
tB6656:0.00326,UltB6657:0.04007)0.837.10:0.01241)0.903.11:0.01628,((UltB6
610:0.01598,(UltB7257:0.09122,(UltB7072:0.0,UltB6717:0.0):0.00771)0.912.1
8:0.01621)0.763.17:0.00578,UltB6601:0.01474)0.794.15:0.00568)0.805.11:5.4
E-4,(UltB6597:0.0,UltB6598:0.0):5.4E-
4)0.750.7:0.00336)0.690.7:0.0047,((UltB6602:0.02328,(UltB6641:0.01469,(Ul
tB6644:0.02721,((UltB6994:0.01848,(UltB6632:0.02658,UltB6633:0.04807)0.37
8.5:0.00746)0.922.16:5.5E-
4,((UltB6707:0.0,UltB6708:0.0,UltB6706:0.0,UltB6639:0.0,UltB6631:0.0,UltB
6640:0.0,UltB6638:0.0,UltB6642:0.0,UltB6645:0.0,UltB6634:0.0,UltB6647:0.0
,UltB6646:0.0):5.5E-4,(UltB6643:0.00372,UltB6595:0.00372)0.694.5:5.5E-
4)0.537.2:5.5E-4)0.946.14:5.5E-
4)0.641.5:0.01373)0.960.16:0.02512)0.756.17:0.00784,((UltB7047:0.00759,((
Ult20674:0.04734,(UltB6607:0.01034,UltB6693:0.05854)0.319.2:0.01385)0.485
.3:0.01054,UltB6606:0.03059)0.911.21:0.01768)0.943.20:0.02436,UltB7048:0.
0036)0.981.7:0.03184)0.709.3:0.00154)0.767.13:0.00345)0.788.10:0.0041,(((
UltB7136:5.4E-4,(UltB6562:5.4E-
4,(UltB6568:0.01526,(UltB6563:0.0113,((UltB6558:0.01131,UltB6567:0.04384)
0.880.19:5.5E-4,(((((UltB6569:5.5E-
4,UltB6570:0.00372)0.833.16:0.00371,UltB6566:0.02344)0.686.7:5.5E-
4,UltB7252:0.01901)0.394.3:5.5E-4,UltB6561:0.0151)0.493.4:5.5E-
4,(UltB6571:0.0,UltB6574:0.0,UltB6573:0.0,UltB6564:0.0,UltB6565:0.0,UltB6
560:0.0,UltB6871:0.0):5.5E-4)0.589.5:5.5E-4)0.014.2:5.5E-
4)0.756.18:0.00383)0.919.17:0.01158)0.923.15:0.0074)0.967.12:0.0195,UltB6
723:5.4E-4)0.953.12:0.01544,((((((UltB7064:5.5E-
4,UltB7063:0.00372)0.861.13:0.00759,((UltB6612:0.02284,(UltB7044:0.00371,
(UltB6711:5.5E-4,(UltB7043:0.00372,UltB7251:0.00747)0.930.9:5.5E-
4)0.796.8:5.5E-4)0.954.14:0.02039)0.895.19:0.01581,(UltB7115:5.4E-
4,((UltB6778:0.0,UltB7114:0.0):5.4E-

4,UltB6779:0.02343)0.798.15:0.00371)0.761.18:0.00458)0.921.23:0.01469)0.7
58.15:0.00378,(UltB7051:0.01796,(UltB7120:0.01021,((((UltB7039:0.00639,Ul
tB7041:0.02276)0.854.11:0.01217,((UltB6777:0.01197,UltB7160:0.00322)0.948
.19:0.02456,(UltB7042:0.01909,UltB7112:5.4E-
4)0.700.6:0.01819)0.603.6:0.01297)0.776.12:0.00606,(UltB7050:0.00622,UltB
6807:0.02206)0.820.6:0.00893)0.777.14:0.00682,((((UltB6823:5.4E-
4,(UltB6559:0.01142,UltB8100:0.06132)0.843.11:0.00746)0.912.19:0.00721,((
(UltB6774:0.01559,(((((UltB6724:0.00372,UltB6771:0.01505)0.685.4:5.5E-
4,(UltB6669:0.00372,UltB6750:5.5E-4)0.420.4:5.5E-4)0.295.5:5.5E-
4,(((((UltB6780:0.01036,(UltB6768:0.01827,(UltB6987:0.02362,UltB6980:0.01
12)0.872.23:0.01455)0.846.11:5.4E-
4)0.870.11:0.00579,(UltB6776:0.00749,UltB6935:5.5E-4)0.067.1:5.4E-
4)0.910.25:0.00749,(UltB6557:0.0,UltB6740:0.0,UltB6741:0.0,UltB6742:0.0,U
ltB6758:0.0,UltB6746:0.0,UltB6745:0.0,UltB6749:0.0,UltB6756:0.0,UltB6760:
0.0):5.5E-4)0.959.16:5.5E-
4,(UltB6773:0.00372,(UltB6754:0.00373,(UltB6775:0.00372,(((UltB6738:0.011
29,UltB6772:0.02288)0.919.18:0.00245,UltB6781:0.00247)0.919.19:0.00247,Ul
tB6759:0.00372)0.000.203:5.5E-4)0.000.204:5.5E-4)0.000.205:5.5E-
4)0.220.1:5.5E-4)0.735.7:5.5E-4,((UltB6763:0.05964,UltB6757:5.5E-
4)0.856.8:0.0037,(((UltB6769:0.02244,UltB6770:0.06433)0.656.4:5.5E-
4,UltB6761:0.00749)0.813.8:0.00372,UltB6762:0.00372)0.787.15:0.00372)0.73
7.14:5.5E-4)0.408.6:5.5E-4)0.826.9:5.3E-4,UltB6764:0.0037)0.909.11:5.4E-
4,UltB6765:0.0191)0.959.17:0.01545)0.749.20:0.00332,UltB6767:0.01158)0.95
3.13:0.01546,(((((UltB6839:0.00373,(((UltB6833:0.01508,UltB6825:0.00721)0
.906.18:5.5E-
4,((UltB6812:0.0,UltB6818:0.0,UltB7057:0.0,UltB7056:0.0,UltB6816:0.0,UltB
6821:0.0,UltB6817:0.0,UltB6824:0.0,UltB6830:0.0,UltB6834:0.0,UltB6835:0.0
,UltB6831:0.0,UltB6827:0.0,UltB6832:0.0,UltB6737:0.0):5.5E-
4,((UltB6810:0.00754,(UltB6814:0.0,UltB6815:0.0,UltB6736:0.0):5.5E-
4)0.841.13:0.00375,(UltB6837:0.1248,(UltB6829:0.00741,UltB6828:0.03798)0.
486.3:0.00409)0.514.1:5.4E-4)0.431.7:5.5E-4)0.017.1:5.4E-4)0.006.2:5.5E-
4,UltB6819:0.00373)0.457.4:5.5E-4)0.345.2:5.5E-
4,UltB6836:0.00746)0.461.1:5.5E-
4,(UltB6811:0.01084,UltB6826:0.0037)0.986.10:5.5E-4)0.594.1:5.5E-
4,(UltB6636:0.00372,(UltB6838:0.00387,UltB7055:0.00748)0.415.5:5.5E-
4)0.000.206:5.5E-4)0.847.17:0.00366,(UltB6809:0.00372,UltB7052:5.5E-
4)0.663.3:5.5E-4)0.790.6:0.0036)0.828.10:0.00715)0.907.18:5.5E-
4,(UltB6755:0.01129,UltB6635:0.00371)0.794.16:0.00378)0.960.17:0.02128,Ul
tB7040:0.02304)0.084.1:0.00751)0.845.14:0.01054)0.317.3:0.00361)0.901.19:
0.00864)0.296.2:5.4E-
4,(UltB7062:0.08363,(UltB7053:0.0,UltB7054:0.0,UltB7065:0.0):5.5E-
4)0.985.13:0.01536)0.909.12:0.00736,(((((UltB7100:0.01264,UltB6556:0.0346
7)0.781.14:0.00765,(UltB7119:0.02392,(UltB7021:0.01454,((UltB6872:0.00746
,(UltB7019:5.5E-4,UltB7020:0.00372)0.667.5:5.5E-
4)0.818.8:0.00378,((UltB7178:5.5E-4,(UltB7177:5.5E-
4,UltB7179:0.01517)0.913.15:0.00751)0.781.15:0.00384,(((UltB7069:0.07782,
(UltB7068:0.00372,(UltB7067:0.0,UltB7066:0.0,UltB7070:0.0,UltB6710:0.0):5
.5E-
4)0.740.8:0.00243)0.968.9:0.02889,(((((UltB6667:0.00371,UltB6648:0.04836)
0.309.1:5.5E-
4,(UltB6670:0.0,UltB7058:0.0,UltB6668:0.0,UltB6743:0.0,UltB6666:0.0,UltB6
679:0.0):5.5E-4)0.361.3:5.5E-
4,UltB6630:0.00372)0.748.10:0.00497,UltB6665:0.05697)0.538.2:0.01006,(Ult
B6748:0.01802,(UltB6686:0.00382,((UltB6687:0.0,UltB6688:0.0):5.4E-

4,UltB6689:0.01536)0.756.19:0.00372)0.773.18:0.00591)0.905.13:0.02408)0.9
25.24:0.0209)0.746.11:0.00469,UltB7018:0.03187)0.919.20:0.01224)0.765.10:
0.00371)0.745.10:0.00522)0.960.18:0.02392)0.821.11:0.0078)0.000.207:5.3E-
4,(UltB6664:0.04406,((UltB7118:0.0,UltB7117:0.0):5.5E-
4,UltB7116:0.00372)0.761.19:0.00816)0.885.13:0.01643)0.955.17:0.02121,(((
((UltB7353:0.09033,UltB6993:0.01857)0.767.14:0.00793,(UltB7182:0.00663,((
((UltB7167:0.01874,(UltB7166:0.02824,UltB7168:0.01512)0.441.5:5.4E-
4)0.000.208:5.4E-
4,(UltB7173:0.0,UltB7172:0.0,UltB7169:0.0,UltB7170:0.0,UltB6820:0.0):5.5E
-4)0.317.4:5.5E-4,(UltB7171:0.01491,UltB7181:0.03916)0.876.14:5.4E-
4)0.251.2:5.5E-
4,UltB7165:0.00372)0.945.16:0.01307)0.755.13:0.00425)0.995.18:0.03447,Ult
B7113:0.01511)0.743.13:0.004,(UltB7164:0.03472,(UltB6554:0.01992,UltB6555
:0.00373)0.975.13:0.02731)0.547.1:0.00941)0.810.11:0.00776,((((((((UltB66
51:0.00753,UltB6649:0.00385)0.359.1:0.00371,((UltB6652:0.0,UltB7059:0.0):
5.5E-4,UltB6650:0.00372)0.819.16:5.5E-4)0.861.14:5.5E-
4,UltB6719:0.0189)0.999.19:0.04086,UltB6663:0.01092)1.000.96:5.3E-
4,(UltB6662:5.5E-
4,UltB6747:0.10079)0.793.15:0.00373)0.861.15:0.01589,UltB7149:0.02803)0.8
64.18:0.01487,((UltB7142:0.05546,(((UltB7143:0.00746,UltB7151:0.00372)0.8
04.12:0.00372,((UltB6766:0.0,UltB7140:0.0,UltB7146:0.0,UltB7145:0.0,UltB7
139:0.0,UltB7159:0.0,UltB7161:0.0,UltB7061:0.0,UltB7162:0.0):5.5E-
4,(UltB7144:0.00372,UltB7141:0.01525)0.322.2:5.5E-4)0.000.209:5.5E-
4)0.103.1:5.5E-4,UltB7138:0.01125)0.933.16:5.5E-
4)0.852.15:0.01322,UltB6992:0.03549)0.706.5:0.0121)0.194.1:0.0114,UltB714
7:5.4E-
4)0.958.16:0.01984)0.857.19:0.0106)0.823.8:0.00745,(UltB7163:0.02303,UltB
7121:0.02724)0.759.14:0.00473)0.860.11:5.4E-
4)0.825.10:0.00898,(UltB7009:0.02842,(UltB7003:0.05209,(UltB6658:0.01539,
((((UltB7156:0.0,UltB7155:0.0,UltB6933:0.0,UltB7158:0.0,UltB7010:0.0,UltB
6714:0.0,UltB7006:0.0,UltB7012:0.0,UltB7002:0.0,UltB7000:0.0,UltB7013:0.0
,UltB6712:0.0,UltB6713:0.0,UltB6705:0.0):5.5E-
4,(UltB7007:0.00372,UltB7005:0.00372)0.701.4:5.5E-4)0.000.210:5.5E-
4,(UltB7011:0.00372,UltB7154:0.00372)0.876.15:5.5E-4)0.562.2:5.5E-
4,(UltB6544:0.03514,(UltB7157:0.00515,UltB7008:0.03882)0.869.15:0.01002)0
.543.1:5.4E-4)0.803.9:5.5E-
4)0.835.13:0.00748)0.637.4:0.00462)0.895.20:0.01678)0.698.4:0.00887)0.030
.2:5.4E-
4)0.814.6:0.00463)0.829.11:0.00842,((UltPorp5:0.06873,(UltB6539:0.01348,(
UltB6716:0.03198,((UltB6715:0.0,UltB6537:0.0,UltB6536:0.0):5.5E-
4,UltB6538:5.5E-
4)0.843.12:0.0132)0.978.7:0.06213)0.945.17:0.03135)0.921.24:0.03482,(UltB
6342:0.06641,(UltB7352:0.12018,UltB7203:0.07497)0.612.3:0.00808)0.927.12:
0.02757)0.779.12:0.01121)0.858.13:0.00846,(((UltB6614:0.11184,UltB6534:0.
09691)0.924.17:0.05662,(UltB6590:0.02922,(UltB6963:0.01536,UltB6964:5.5E-
4)0.997.17:0.06666)0.831.16:0.03295)0.693.1:0.02089,((UltB6654:0.00749,((
UltB7275:0.17968,(UltB6629:0.00438,(UltB6628:5.5E-
4,UltB6626:0.00371)0.839.10:0.01089)0.926.14:0.03715)0.901.20:0.03142,Ult
B6653:0.05725)0.253.3:0.00825)0.928.14:0.02471,(UltB6655:0.05409,(UltB675
3:5.5E-
4,(UltB6751:0.0153,UltB6752:0.04773)0.927.13:0.01614)0.940.13:0.02894)0.9
59.18:0.04311)0.662.5:0.01546)0.933.17:0.026)0.285.2:5.5E-
4,((((UltB6971:0.01568,(UltB6970:0.00746,Ult18709:5.5E-
4)0.865.16:0.01162)0.944.13:0.02709,(UltB6840:0.04371,UltB6972:0.01423)0.

334.2:5.4E-4)0.443.1:0.01417,((UltB6973:0.04238,(UltB6968:5.5E-
4,UltB6967:0.00372)0.790.7:0.00902)0.764.14:0.012,UltB6969:0.01183)0.862.
15:0.01136)0.903.12:0.01489,((((((UltB6867:0.04281,((((((UltB6900:5.5E-
4,UltB6896:0.00372)0.876.16:0.00798,(UltB6899:0.00373,UltB6897:0.00375)0.
831.17:0.00725)0.720.7:0.00441,(UltB6898:0.01572,UltB6854:0.03802)0.920.1
5:0.02589)0.963.14:0.02696,((UltB6887:0.01823,((UltB6891:0.0,UltB6892:0.0
):0.03032,(UltB6808:0.00389,(UltB6881:0.01144,((UltB6884:0.0,UltB6885:0.0
,UltB6882:0.0):5.5E-4,(UltB6883:0.01128,UltB6927:0.00372)0.908.13:5.5E-
4)0.929.13:5.4E-
4)0.784.12:0.00357)0.859.14:0.00958)0.648.1:0.01117)0.858.14:0.00888,(Ult
B6894:0.02716,(UltB6895:0.00745,UltB6893:0.02343)0.753.11:0.00386)0.852.1
6:0.00819)0.448.2:0.00503)0.890.22:0.01669,(((UltB6868:5.5E-
4,UltB6869:0.00372)0.954.15:0.03226,(UltB6744:0.00463,UltB6870:0.00673)0.
990.15:0.0535)0.882.9:0.02128,((((UltB6863:0.03355,UltB6861:0.14751)0.723
.9:0.00556,((UltB6859:0.00372,UltB6862:0.02272)0.308.3:5.5E-
4,(MouseGut:0.01133,((UltB6866:0.0,UltB6864:0.0,UltB6860:0.0,UltB6858:0.0
):5.5E-4,UltB6875:0.00372)0.000.211:5.5E-4)0.235.2:5.5E-4)0.964.21:5.5E-
4)0.862.16:0.00759,UltB6879:0.01974)0.852.17:0.0078,(UltB6865:0.00779,(Ul
tB6873:5.5E-4,((((UltB6878:0.03916,UltB6874:0.02295)0.382.1:5.5E-
4,UltB6880:0.00372)0.360.4:5.5E-4,UltB6877:5.5E-4)0.391.4:5.5E-
4,UltB6876:0.00372)0.828.11:0.00376)0.939.15:0.01515)0.559.2:0.0039)0.916
.21:0.01263)0.823.9:0.00871)0.824.18:0.01315,((UltB6903:0.03261,(UltB6901
:0.00754,UltB6902:5.5E-
4)0.830.15:0.01176)0.974.8:0.03589,(UltB6889:0.04283,(UltB6890:0.04675,((
UltB6961:0.0,UltB6962:0.0):5.5E-4,(UltB6959:5.5E-4,UltB6960:5.5E-
4)0.858.15:0.00728)0.992.15:0.06398)0.327.2:0.0201)0.830.16:0.01802)0.857
.20:0.01066)0.711.6:0.00482)0.922.17:0.02097,(UltB6989:0.04536,UltB6943:0
.01365)0.906.19:0.01776)0.753.12:0.0044,((UltB6944:0.04024,(UltB6949:0.00
372,(UltB6952:5.5E-4,(UltB6950:5.5E-
4,(UltB6953:0.00371,UltB6951:0.07659)0.647.1:5.5E-4)0.375.4:5.5E-
4)0.059.3:5.3E-
4)0.919.21:0.01263)0.746.12:0.00311,((UltB7153:0.01501,(UltB6957:5.5E-
4,UltB6956:5.5E-4)0.882.10:5.4E-
4)0.994.17:0.03468,UltB6958:0.01835)0.911.22:0.0174)0.760.13:0.00396)0.94
7.16:5.4E-
4,UltB6888:0.0583)0.760.14:0.00359,(((UltB6907:0.02329,((((UltB6918:0.018
77,UltB6940:5.5E-
4)0.836.10:0.00368,((UltB6937:0.01499,UltB6905:0.03467)0.883.16:5.3E-
4,(((((UltB6936:0.01229,UltB6932:0.00696)0.421.2:5.3E-
4,UltB8099:0.07073)0.911.23:0.01035,(UltB6954:0.00748,((UltB6941:0.00749,
UltB6955:5.5E-
4)0.856.9:0.01016,UltB6947:0.03241)0.262.1:0.00993)0.950.13:5.5E-
4)0.849.13:0.00733,((UltB6938:0.00372,((UltB6917:0.00372,((((((UltB6924:0
.00372,UltB6912:0.00372)0.833.17:0.00372,UltB6906:0.01527)0.496.3:5.5E-
4,(UltB6925:0.0,UltB6928:0.0,UltB6981:0.0,UltB6983:0.0,UltB6982:0.0,UltB6
984:0.0,UltB6985:0.0,UltB6916:0.0,UltB6908:0.0,UltB6939:0.0,UltB6931:0.0,
UltB6997:0.0,UltB6998:0.0):5.5E-4)0.506.4:5.5E-
4,UltB6911:0.00372)0.480.1:5.5E-4,UltB6910:0.0075)0.000.212:5.5E-
4,UltB6930:0.00746)0.479.4:5.5E-4)0.000.213:5.5E-
4,UltB6929:0.00372)0.489.2:5.5E-4)0.000.214:5.5E-4,((UltB6942:5.4E-
4,(UltB6915:5.5E-
4,UltB6926:0.00372)0.906.20:0.00364)0.006.3:0.00372,UltB6988:5.0E-
4)0.906.21:0.00364)0.000.215:5.5E-4)0.883.17:5.5E-4,UltB6914:5.5E-
4)0.349.1:5.5E-4)0.056.1:5.5E-4)0.767.15:5.4E-

4,UltB6913:0.01094)0.575.1:5.5E-4,((UltB8105:5.3E-
4,UltB6909:0.06413)0.800.9:0.00341,UltB6904:0.09274)1.000.97:5.4E-
4)0.789.10:0.00404)0.763.18:0.00369,UltB6995:0.02326)0.774.23:0.01389,((U
ltB6946:0.0,UltB6945:0.0):0.01194,(UltB6805:0.00372,UltB6948:5.5E-
4)0.975.14:0.0274)0.932.12:0.02276)0.774.24:0.00687)0.970.16:0.02572,((Ul
tB6853:5.4E-
4,UltB6852:0.00371)0.997.18:0.05084,((UltB6966:0.04211,UltB6965:0.04408)0
.932.13:0.02551,(UltB6990:0.11333,(UltB6855:0.0206,(UltB6709:0.01823,((((
(UltB6856:0.00372,(((((UltB6847:0.07237,(UltB7025:0.01498,(UltB6850:0.021
77,UltB6704:0.01005)0.861.16:0.01418)0.748.11:0.00391)0.749.21:0.00383,Ul
tB6845:0.00363)0.966.15:5.5E-
4,(UltB6843:0.0038,UltB6849:0.00756)0.808.5:0.00369)0.000.216:5.4E-
4,UltB6844:0.02701)0.408.7:5.5E-4,UltB6735:0.00372)0.397.3:5.5E-
4)0.000.217:5.5E-4,(UltB6851:0.0,UltB6703:0.0):5.5E-4)0.474.5:5.5E-
4,(UltB6848:0.01118,(UltB6842:0.00859,UltB6841:0.10095)0.799.14:0.01057)0
.779.13:0.00379)0.000.218:5.5E-
4,UltB6846:0.03402)0.632.3:0.00165,(UltB6734:5.4E-
4,UltB6857:0.0578)0.932.14:0.00213)0.770.18:0.01167)0.816.13:0.00752)0.24
7.1:5.4E-
4)0.333.2:0.0154)0.861.17:0.01486)0.689.4:0.00506)0.375.5:0.00819)0.946.1
5:0.01699)0.857.21:0.01499)0.873.20:0.02378)0.693.2:0.01362)0.795.17:0.00
92,((((((UltB7266:0.00766,UltB7267:0.00363)0.988.17:0.03577,((UltB7273:0.
10535,UltB7270:5.4E-
4)0.971.8:0.01188,((UltB7260:0.01545,((UltB7263:0.00359,UltB7264:0.01159)
0.063.2:0.00474,((UltB7912:0.04191,UltB7265:5.4E-
4)0.282.1:0.00387,UltB7262:5.3E-
4)0.928.15:0.01564)0.976.9:0.02336)0.761.20:0.00411,((UltB7261:0.01038,(U
lt19268:5.4E-4,(UltB7258:5.5E-4,UltB7259:5.5E-
4)0.801.12:0.00727)0.957.12:0.02069)0.869.16:0.01634,UltB7274:0.02648)0.7
69.14:0.0044)0.966.16:0.01576)0.588.2:5.4E-
4)0.957.13:0.02841,(UltB7272:0.0,UltB7271:0.0):0.02037)0.704.2:0.01566,Ul
tB7277:0.02758)0.755.14:0.00958,((UltRu148:0.01547,UltB7269:0.00584)0.283
.1:0.01523,UltB7268:0.06982)0.097.2:0.03892)0.998.17:0.07908,(UltB6553:0.
04662,((UltB6549:0.0,UltB6550:0.0,UltB6545:0.0,UltB6547:0.0,UltB6546:0.0,
UltB6552:0.0):5.5E-4,(UltB6548:0.03078,UltB6551:0.01936)0.861.18:5.3E-
4)0.845.15:0.02211)0.947.17:0.03493)0.701.5:0.01164)0.850.13:0.04161)0.33
5.4:0.01549,(UltB5941:0.08779,((UltB8148:0.07224,Ult20676:0.03702)0.773.1
9:0.04925,UltB7922:0.08896)0.358.4:0.03739)0.923.16:0.0422)0.689.5:0.0117
4)0.894.13:0.03409)0.957.14:0.02407)0.966.17:0.01866,((UltB7665:0.06764,(
(((UltB7303:0.03753,UltB7305:0.01087)0.930.10:0.02002,(UltB7304:0.04121,U
ltB7602:0.05084)0.973.8:0.03347)0.929.14:0.01818,((UltB6485:0.04214,((((U
dnRum19:0.03427,(UltB6489:0.03033,(UltB7226:0.00881,(UltB8036:0.00745,(Ul
tB6490:0.00763,UltB6493:0.00373)0.330.2:5.5E-
4)0.962.18:0.02332)0.848.13:0.01067)0.695.4:0.00922)0.954.16:0.02614,((Ul
tB6495:0.0157,UltB6481:0.0446)0.697.2:0.00735,(UltPorp4:5.5E-
4,(UltB6491:5.5E-4,(UltB6482:0.0,UltB6494:0.0):5.5E-4)0.397.4:5.5E-
4)0.951.15:5.3E-4)0.908.14:0.00763)0.002.1:5.4E-
4,(UltB6483:0.00784,(UltPorp3:0.01095,(UltB6492:0.0171,(UdnRum18:0.01516,
((UltB6479:0.0,UltRu140:0.0,UltRu141:0.0):5.5E-
4,UltB6480:0.00374)0.902.17:5.4E-
4)0.920.16:0.02195)0.648.2:0.01556)0.905.14:0.01897)0.872.24:0.01271)0.96
6.18:0.0221,UltB6486:0.0259)0.239.2:5.3E-
4)0.976.10:0.02302,UltRu142:0.05268)0.967.13:5.4E-
4)0.036.2:0.00743,UltB6488:0.0175)0.950.14:0.02493)0.925.25:0.02325,(((Ul

tPor45:0.07413,UltB7306:0.03791)0.568.2:0.01276,(PahggY32:0.08348,(((((Ul
tB8543:0.0,UltB8544:0.0):0.03111,UltB8545:0.04358)0.950.15:0.04299,(UltB8
790:5.5E-
4,(UltB8617:0.09488,ArmMaris:0.00399)0.958.17:0.02697)0.998.18:0.07422)0.
838.15:0.01672,(UltB8541:0.1263,(UltB8410:0.02435,UltB8411:0.03721)0.802.
11:0.02132)0.863.14:0.01923)0.772.17:0.00811,UltB8412:0.08657)0.548.3:0.0
3106)0.113.2:0.01303)0.775.10:0.00677,((((((UltB8702:0.00369,(UltB7634:0.
00369,UltB7635:5.5E-4)0.424.5:5.5E-
4)0.763.19:0.00725,(UltB7633:0.01896,UltB7632:0.05333)0.945.18:0.02727)0.
999.20:0.06872,(UltB7630:0.00371,(UltB7625:0.0,UltB7626:0.0,Ult27511:0.0)
:0.01529)0.852.18:5.5E-
4)0.991.14:0.02984,((((UltB7638:0.02732,(PahggY29:0.05238,(PahggY08:5.5E-
4,PahggY31:5.5E-
4)0.764.15:0.00349)0.927.14:0.01968)0.729.6:0.00376,((Bfhggg25:0.02898,Ul
tPor44:0.01481)0.572.3:0.01098,(Otu00721:0.02757,(PahggY97:0.0,Pshggg25:0
.0):0.01954)0.877.21:5.4E-4)0.778.17:0.00653)0.877.22:5.5E-
4,(UltB7629:0.01134,UltB7627:0.01145)0.847.18:0.00736)0.953.14:0.02095,((
UltB7622:0.00415,(UltB7618:0.0445,(UltB7628:0.01856,(UltB7624:0.09126,(Ul
tB7621:0.00368,UltB7623:5.4E-
4)0.822.8:0.01091)0.754.14:0.00688)0.844.19:0.0096)0.747.14:0.00513)0.880
.20:0.0119,((UltB7664:0.0467,(Cuiiii17:0.04832,(UltB7544:0.02421,(UltB754
5:0.0,UltB7670:0.0):0.00306)0.959.19:0.02868)0.901.21:0.0231)0.913.16:0.0
2397,(UltDysgo:0.0428,(UltB7636:0.05635,UltB7637:0.00971)0.749.22:0.01126
)0.972.12:0.03275)0.493.5:0.00425)0.906.22:0.01457)0.053.2:5.5E-
4)0.224.3:0.00379,((UltB7603:0.0846,(UltB7619:0.03554,(((UltB7645:0.07743
,(UltB7620:0.0716,(((Cuiiii16:0.02853,Otu01217:0.0846)0.775.11:0.02195,((
UncUn448:0.0,UncUn449:0.0,UncUn447:0.0):5.3E-
4,UncUnc74:0.00366)0.982.5:0.0407)0.722.2:0.00473,Otu02055:0.0414)0.900.1
6:0.0256)0.592.3:0.01086)0.868.14:0.03265,Otu01188:0.07009)0.904.13:0.031
34,P0114484:0.03631)0.750.8:0.00936)0.908.15:0.0213)0.608.2:0.00663,(UltB
7649:0.03032,Otu00733:0.07835)0.500.2:0.01099)0.921.25:0.03065)0.471.6:0.
01026,(((UltB7644:5.4E-
4,(UltB7642:0.03201,(UltB7641:0.02751,(UltB7639:5.4E-
4,UltB7640:0.16439)0.968.10:0.03793)0.763.20:0.01842)0.621.5:0.01758)0.91
9.22:0.02269,((PahggY30:0.01526,UltB7650:0.01971)0.956.13:0.01991,P011447
4:0.01146)0.832.11:5.3E-
4)0.897.9:0.0129,(((PshYyy18:0.0,Pshggg26:0.0):0.02496,(UltB7648:0.04653,
Otu06349:0.04086)0.923.17:0.03612)0.259.2:0.02614,(Otu00890:0.01623,(Sgff
ff10:0.003,(Ult40947:0.0,UltB7646:0.0,UltB7647:0.0):0.04028)0.964.22:0.02
556)0.745.11:0.02368)0.690.8:0.0061)0.634.1:0.00411)0.920.17:0.02538)0.84
8.14:0.01465)0.839.11:0.01013)0.824.19:0.0067,(((UltB5973:0.09707,(((Bfhg
gg32:0.01626,UltB8391:0.01071)0.946.16:0.04566,(AtPYy118:0.17458,UltB8392
:5.5E-
4)0.935.19:0.04059)0.984.6:0.06729,((UltPor46:0.05301,(UltPor47:0.01837,(
((((OdoDenti:0.09921,UltB8432:0.06807)0.882.11:0.03442,UltB8434:0.0376)0.
828.12:0.01617,(((((UltB8429:0.00652,(UltB8428:0.0,Otu00435:0.0):0.00487)
0.989.15:0.06994,(UltB8430:0.00373,UltPor48:0.01553)0.864.19:0.02318)0.97
7.10:0.04935,(UltB8431:0.04381,(UltB8426:5.3E-
4,UltB8427:0.00748)0.963.15:0.0373)0.424.6:0.02667)0.878.16:0.02823,((Ult
B8423:5.4E-
4,UltB7350:0.03897)0.966.19:0.02493,UltB8422:0.02702)0.646.2:0.00474)0.90
6.23:0.02376,((UltB8419:0.01624,(UltB8420:0.00773,UltB8421:0.01547)0.718.
6:0.00299)0.991.15:0.04711,UltB8424:0.00955)0.946.17:0.0277)0.737.15:0.00
611)0.757.9:0.00949,(UltB8425:0.04514,(UltB8436:5.3E-

4,UltB8435:0.01926)0.935.20:0.01933)0.450.3:0.00604)0.805.12:0.0148,UltB8
433:0.08151)0.979.12:0.06207)0.853.14:0.02604)0.762.14:0.02276,(Bfhggg33:
0.035,((Otu00382:0.03564,Otu00438:0.00917)0.995.19:0.11527,(UltB8402:0.06
488,(BtcSyner:0.00914,(((((UltB8393:0.01137,(UltB8397:0.0,UltB8398:0.0):5
.4E-
4)0.780.15:0.00589,((UltB8394:0.00759,UltB8395:0.01159)0.873.21:0.01049,U
ltB8396:0.01889)0.765.11:0.00511)0.766.11:0.00633,UltB8400:0.00751)0.899.
16:5.4E-
4,UltB8399:0.00732)0.880.21:0.01803,UltB8401:0.02308)0.825.11:0.01895)0.9
56.14:0.05888)0.089.2:0.02698)0.982.6:0.08129)0.744.10:0.01781)0.987.14:0
.06943)0.790.8:0.01557)0.782.19:0.01484,UltB8418:0.09774)0.926.15:0.03971
,(Bfhggg24:0.07842,(UltB8035:0.0355,((UltB7616:5.5E-
4,UltB7617:0.00369)0.951.16:0.04444,((UltB7615:5.4E-
4,(UltB7613:0.00363,UltB7614:0.00376)0.465.3:0.00742)0.938.14:0.03134,((U
ltB7610:0.01512,UltB7609:5.4E-
4)0.923.18:0.01391,(UltB7608:0.04589,((UltB7361:0.01869,UltB7612:5.5E-
4)0.927.15:0.02698,(UltB7611:0.00738,(UltB7607:5.5E-
4,UltB7606:0.0113)1.000.98:0.07007)0.058.2:0.00703)0.774.25:0.01429)0.760
.15:0.00445)0.790.9:0.01701)0.329.5:0.02545)0.897.10:0.04519)0.823.10:0.0
2787)0.845.16:0.0215)0.467.2:5.4E-
4)0.855.11:0.01591,((((((Tryy0093:0.12763,UltRu259:0.10025)0.993.10:0.104
04,(((UltB8318:5.5E-4,UltB8316:5.5E-
4)0.972.13:0.0265,((UltB8307:0.0251,((UltB8602:0.0424,(UltB8317:0.00734,U
ltB8325:0.07524)0.886.17:0.01665)0.933.18:0.02393,(UltB8300:0.05788,((Ult
B8313:0.02765,(UltB8301:0.0,UltB8302:0.0):0.04068)0.949.19:0.02937,((((Ul
tB8312:0.03344,(UltB8309:0.05413,(UltB8311:0.0,UltB8310:0.0):5.5E-
4)0.941.13:0.02433)0.439.2:0.01148,UltB8308:0.0393)0.865.17:0.01657,(UltB
8304:0.01185,(UltB8305:0.00369,(UltB8303:0.00747,(UltB8306:0.0,UltB8601:0
.0):5.5E-4)0.014.3:5.5E-
4)0.758.16:0.00369)0.956.15:0.03089)0.808.6:0.01259,(UltRu238:0.0,UltRu26
2:0.0):0.01887)0.839.12:0.01061)0.872.25:0.0146)0.829.12:0.01281)0.858.16
:0.01645)0.936.18:0.02417,(UltB8314:0.00478,(UltB8315:0.00881,UltRu239:0.
06741)0.486.4:0.01439)0.942.11:0.02413)0.867.18:0.01191)0.880.22:0.01322,
(UltRu241:0.0155,(((UltB8320:0.00372,UltB8321:0.0037)0.766.12:0.00371,((U
ltRu245:0.0116,((UltRu243:0.01036,UltRu244:0.04425)0.851.15:0.00962,UltB8
322:0.011)0.874.18:0.00785)0.455.6:5.3E-
4,UltRu242:0.01514)0.968.11:0.02303)0.817.5:0.007,(UltB8319:5.5E-
4,UltRu240:0.0037)0.965.19:0.0149)0.938.15:5.3E-
4)0.385.5:0.0072)0.640.2:0.00495)0.952.21:0.03948,((PtlJodog:0.09904,((((
HmZZZZ04:0.04328,Otu00626:0.04347)0.861.19:0.0332,(((UltB8221:0.04428,(Sg
ffffffF:0.01687,(UltB8224:0.01802,(UltB8225:0.0,Otu00045:0.0):0.00903)0.87
8.17:0.02048)0.924.18:0.02628)0.487.4:0.02046,(UncUn111:0.00755,((UltB821
8:5.1E-
4,P0114463:0.00749)0.874.19:0.00758,(UltB8219:0.01679,UltB8220:0.02124)0.
857.22:0.01198)0.948.20:0.01529)0.624.4:0.0027)0.776.13:0.00672,(UltB8222
:0.03143,UltB8223:5.3E-
4)0.963.16:0.02943)0.669.3:0.01113)0.921.26:0.02736,Otu00497:0.03515)0.78
7.16:0.01209,UltB8226:0.01323)0.909.13:0.04135)0.942.12:0.04305,((((UltB8
386:0.03259,UltB8383:0.03258)0.960.19:0.0325,(UltB8385:0.0,UltB8384:0.0):
5.3E-
4)0.250.1:0.00882,UltB8377:0.04477)0.864.20:0.01756,((((UltB8365:0.0192,(
UltB8366:0.01499,(UltB8368:5.5E-4,UltB8371:0.00369)0.924.19:5.5E-
4)0.917.14:0.01778)0.854.12:0.01367,(UltB8367:0.01634,(UltB8369:0.02782,U
ltB8370:0.00481)0.877.23:0.01057)0.852.19:0.01393)0.890.23:0.03073,UltAna

e6:0.14543)0.678.6:0.0187,((UltB8389:0.00372,(((UltB8374:0.0,UltB8375:0.0
,UltB8376:0.0,UltB8378:0.0,UltB8381:0.0):5.5E-4,(UltB8373:5.5E-
4,UltRu257:0.00742)0.844.20:0.00369)0.567.1:5.5E-4,(UltB8379:5.5E-
4,UltB8380:0.02253)0.839.13:0.00367)0.774.26:0.00368)0.907.19:0.01993,((U
ltB8364:0.0,UltB8387:0.0):5.3E-4,(UltB8362:5.3E-
4,(((UltB7229:0.0037,UltB8360:0.00372)0.786.12:0.00371,UltB8363:0.00367)0
.957.15:5.4E-4,(UltB8361:0.02976,((UltB8354:0.00398,((UltB8355:5.4E-
4,UltB8356:0.01506)0.994.18:0.02762,(UltRu256:0.02299,UltB8353:0.01898)0.
764.16:0.00449)0.588.3:5.5E-
4)0.872.26:0.01002,((UltB8359:0.03063,UltB8358:0.19251)0.703.7:0.00507,((
BcsYyyy0:5.5E-
4,UltB8357:0.02659)0.990.16:0.02774,(UltB8352:0.01086,(UltRu249:0.00915,(
((UltRu254:5.5E-4,UltRu251:5.5E-
4)0.872.27:0.00669,((UltB8338:0.00342,(UltB8339:0.0,UltRu253:0.0):0.00796
)0.714.5:0.00563,(UltRu255:0.01391,(UltB8351:5.5E-4,(UltB8350:5.5E-
4,UltB8341:0.00369)0.879.13:0.01476)0.716.5:0.02539)0.621.6:0.00835)0.850
.14:0.00932)0.709.4:5.5E-4,((((UltB8349:5.5E-
4,((UltB8347:0.0,UltB8345:0.0,UltB8348:0.0):5.5E-
4,UltB8346:0.00369)0.740.9:5.5E-4)0.360.5:5.4E-4,(UltRu252:5.5E-
4,UltB8342:0.00369)0.921.27:0.00752)0.933.19:0.00224,(UltB8344:5.5E-
4,(UltRu250:0.00748,(UltB8343:5.5E-
4,UltB8340:0.00747)0.817.6:0.00372)0.300.2:5.5E-
4)0.933.20:0.00229)0.874.20:0.00369)0.878.18:0.0107)0.883.18:0.00998)0.75
5.15:0.00615)0.718.7:0.00233)0.080.1:0.00529)0.786.13:0.00996)0.956.16:0.
02421)0.434.1:0.00729)1.000.99:0.05337)0.876.17:0.01807)0.827.14:0.01199)
0.758.17:0.00822)0.966.20:0.03773)0.887.14:0.02707)0.738.6:0.01329,((((Ul
tB8217:0.03794,(UltRik14:0.00387,((UltRik11:5.5E-
4,((UltRik12:0.0,UltRik13:0.0):0.00383,Otu00145:0.00742)0.395.2:5.3E-
4)0.957.16:5.4E-4,((UncUn110:0.02762,UltB8216:5.5E-
4)0.439.3:0.00369,UltRik10:5.3E-
4)0.960.20:0.01143)0.932.15:0.01121)0.805.13:0.01563)0.966.21:0.05626,(Ul
tB8230:0.05965,((UltB8228:0.01106,(UltB8229:0.0,Otu00343:0.0):5.5E-
4)0.849.14:0.00753,(UltB8227:0.00679,((UltEndo3:0.021,(UltB8231:0.0036,Ot
u00197:5.4E-
4)0.728.5:0.00637)0.944.14:0.04538,(Otu00146:0.03555,UltEndos:0.07172)0.0
00.219:0.02646)0.788.11:0.01901)0.876.18:5.4E-
4)0.826.10:0.02162)0.973.9:0.05528)0.868.15:0.04358,(((UltB7287:0.05838,(
(UltB7284:0.06494,UltB7285:0.02529)0.971.9:0.05378,(UltB7286:0.06509,(Ult
B7281:5.5E-4,(UltB7280:0.05648,(UltB7278:0.00742,(UltB7279:5.5E-
4,((UltB7282:0.0,UltB8028:0.0):5.5E-4,UltB7283:0.0151)0.756.20:5.5E-
4)0.563.1:5.5E-4)0.997.19:5.4E-
4)0.833.18:0.00354)0.833.19:0.01225)0.915.16:0.03233)0.759.15:0.02124)0.9
62.19:0.05422,(((Otu00277:0.08345,(Otu00023:0.00165,Otu00268:0.02313)0.99
1.16:0.08105)0.895.21:0.03993,((UltB8215:0.07897,((Otu00103:0.00391,Otu00
310:0.00353)0.992.16:0.05226,(Otu00107:0.01117,Otu00192:5.5E-
4)0.785.18:0.01102)0.977.11:0.06343)0.888.22:0.02861,(((UltB8213:0.01108,
(UltB8211:0.01129,UltB8212:0.0075)0.737.16:0.00393)0.977.12:0.01944,(Kty0
0020:0.00369,Kty00021:5.5E-4)0.884.16:0.00788)0.739.16:5.5E-
4,(UltB8214:0.00585,(Otu00022:0.00738,Otu00038:0.0115)0.987.15:0.02678)0.
795.18:0.00537)0.990.17:0.07212)0.850.15:0.03113)0.801.13:0.01153,UltB797
7:0.10513)0.950.16:0.03418)0.870.12:0.02373,((UltB7298:0.00368,((UltB7296
:0.0,UltB7294:0.0,UltB7297:0.0):5.5E-
4,(UltB7295:0.00369,UltB7302:0.00369)0.879.14:5.5E-4)1.000.100:5.5E-
4)0.922.18:0.02668,(UltB7293:0.02244,((UltB7300:0.0,UltB7289:0.0):5.4E-

4,((UltB7290:0.0,UltB7291:0.0,UltB7299:0.0):5.5E-4,UltB7301:5.5E-4)0.839.14:0.00366)0.778.18:0.00419)0.937.18:5.3E-4)0.700.7:0.03051)0.743.14:0.01648)0.935.21:0.03725,UltB7276:0.04922)0.893.21:0.02119)0.835.14:0.0194,((((Tryy0092:0.0,Tryyy094:0.0):0.04113,(UltRik15:0.03845,UltRik16:0.03794)0.923.19:0.0405)0.942.13:0.04914,(Cuiiii20:0.13066,(UltMar42:0.01101,(UltMar41:0.0076,Bfhggg10:0.09225)0.667.6:0.00853)0.551.4:5.4E-4)0.748.12:0.03875)0.892.15:0.03869,((UltB8031:0.00331,UltB8032:5.4E-4)0.998.19:0.10668,(UltB8033:0.03566,UltB8034:0.14575)0.835.15:0.03298)0.557.2:0.02951)0.938.16:0.04094)0.931.18:0.03588,UltB7288:0.04478)0.979.13:0.05117)0.805.14:0.00869,(((((TrZZZZ04:5.4E-4,Tryy0025:0.01903)1.000.101:0.07865,((((UltRu258:0.0051,UltB8405:0.02289)0.999.21:0.0885,(UltB8407:0.03269,(UltB8406:0.01304,UltB8408:0.05459)0.875.18:0.01402)0.866.15:0.01853)0.869.17:0.01981,UltMar43:0.00997)0.815.8:0.02039,P0114452:0.01883)0.510.3:0.01929)0.923.20:0.0417,((UltB8404:0.01476,(AnhTherm:0.00525,AnhSpeci:0.07989)0.894.14:0.02379)0.984.7:0.05614,(UltB8403:0.00445,(AlfImshe:0.01187,RmfXylan:0.06657)0.466.6:0.01651)0.889.16:0.01714)0.000.220:0.01364)0.906.24:0.02301,(((((PebSpeci:0.04373,UltB8450:0.17101)0.196.3:0.02124,((UltB8613:0.05398,UltB8614:0.17382)0.831.18:0.01719,(((((AntBac28:5.4E-4,UltB8473:0.00745)0.785.19:0.00388,((PebDaech:5.3E-4,FlaSpec3:0.0232)0.682.4:0.00791,PebTerri:0.02393)0.753.13:0.00324)0.973.10:0.01535,(PebSalta:0.05807,UdnBac22:0.01138)0.308.4:5.4E-4)0.665.4:0.00758,((UltrSo63:0.0037,(((UltB8489:0.01142,(SplSpeci:0.0035,UltB8494:0.01523)0.871.13:0.00767)0.744.11:0.0036,(((UltB8496:5.5E-4,(UltB8498:0.0,PebSpec6:0.0):0.00369)0.815.9:0.00369,(UltB8500:0.01515,UltB8497:0.0037)0.590.2:5.5E-4)0.887.15:0.00983,(((UltB8499:0.0,PebSpec7:0.0):5.5E-4,((((PebAfric:0.0,PebSpec4:0.0):5.5E-4,(UltB8476:0.0071,UltB8479:0.06393)0.865.18:5.4E-4)0.921.28:0.00243,(UltB8444:0.00741,((PebSpec3:0.0,PebCaeni:0.0,PebDuraq:0.0):5.5E-4,ShgSpec6:0.0037)0.353.1:5.4E-4)0.921.29:0.00247)0.921.30:0.00243,((((PebCryoc:0.00372,(PebHarto:0.00373,(PebPisci:5.5E-4,(((((UltB8486:0.00768,(NubZeaxa:0.01112,UltB8485:5.5E-4)0.776.14:0.0075)0.848.15:0.00731,((PebRoseu:0.0,AntBac29:0.0,FlaSpec4:0.0,PebaAgri:0.0,UltB8482:0.0,PebSanda:0.0):5.5E-4,UltB8488:0.02296)0.898.22:0.00738)0.363.3:5.5E-4,(PebSpec5:0.0,PebKoree:0.0):5.4E-4)0.941.14:5.0E-4,((UltB8478:5.5E-4,PebHepar:0.01133)0.887.16:0.01128,(PebKwang:0.00746,UltB8487:0.00745)1.000.102:5.5E-4)0.777.15:0.00365)0.962.20:0.01118,(((ShbBact3:0.00374,(ShbBact2:5.5E-4,(UdnBac23:0.00378,(UltB8484:0.00718,(UltB8481:0.00379,PebAquat:0.02375)0.923.21:5.3E-4)0.884.17:0.00727)0.790.10:0.00371)0.775.12:0.00366)0.847.19:0.00744,UltSph27:0.04824)0.352.1:5.4E-4,((McgKamei:0.02725,(UltrSo62:0.02303,McgGraci:0.01413)0.857.23:0.00981)0.571.2:5.5E-4,(McgXimon:0.00745,UltrSo64:5.5E-4)0.942.14:0.00743)0.860.12:0.0037)0.592.4:5.4E-4)0.938.17:0.01517)0.767.16:0.00362)0.898.23:0.00753)0.889.17:0.00748,(PebPanac:0.0037,(PebGinse:0.0,ShbBacte:0.0,UltB8477:0.0):5.4E-4)0.676.1:5.4E-4)0.818.9:0.00237,UltB8480:0.00999)0.898.24:0.00989,((PebSpec2:0.00761,UltSph29:0.01141)0.862.17:0.00736,UltB8475:0.01884)0.893.22:5.4E-4)0.390.1:5.3E-

4)0.873.22:0.00731)0.906.25:0.0095,(UltB8495:0.01905,PebSpec8:5.5E-
4)0.746.13:0.00522)0.136.1:0.00716)0.932.16:0.01299)0.901.22:0.00733,((Mc
gOryza:0.01508,UltB8492:5.5E-
4)0.797.14:0.00392,((EnmbnO28:0.01931,(UltB8501:0.05324,UltB8490:5.4E-
4)0.960.21:0.0154)0.848.16:0.01087,((CtphgStr:0.01174,(UltB8493:0.00755,M
cgSpeci:0.01542)0.873.23:0.01148)0.768.9:0.00801,McgPalud:0.00376)0.952.2
2:0.0188)0.985.14:5.4E-4)0.888.23:0.01541)0.985.15:5.4E-
4)0.857.24:0.00758,(((((UltB8443:0.00391,McgDaeje:0.04347)0.237.4:0.00374
,UltrSo65:0.01568)0.942.15:0.01518,(UltB8474:0.02352,UltSph28:0.0196)0.74
3.15:0.00346)0.870.13:5.5E-
4,UltB8491:0.00723)0.847.20:0.01304,(UltB8462:0.0173,((((KarCaver:0.00352
,(ShgSpeci:0.00513,(UltB8448:0.00737,((((OlbSitie:0.04199,UltB8447:0.0576
2)0.755.16:0.0071,UltB8449:0.0045)0.839.15:0.00937,PpdbSoli:0.01026)0.932
.17:0.01386,PpdKoree:5.5E-4)0.698.5:5.3E-
4)0.985.16:0.03101)0.914.16:0.01468)0.903.13:0.00806,(UltB8446:0.01132,Ol
bcSoli:0.03245)0.183.3:0.0115)0.557.3:5.5E-
4,(OlbTerra:0.02457,OlbGinse:0.00694)0.950.17:0.01614)0.844.21:0.00857,Ul
tB8458:0.0808)0.565.2:0.00885)0.978.8:0.03139)0.771.19:0.00674)0.972.14:0
.02281)0.441.6:5.3E-
4,(UltB8232:0.0172,((((ShgSpec2:0.0,UltB8463:0.0,ShgSpec4:0.0):0.00336,Sh
gSpec3:5.3E-4)0.967.14:0.04881,((UltB8440:5.5E-
4,((UltB8445:0.0,UltB8439:0.0,Bact1B1b:0.0,UltB8441:0.0,UltB8442:0.0):5.5
E-4,UltSph26:5.5E-4)0.769.15:5.5E-4)1.000.103:5.5E-4,(ShgCanad:5.5E-
4,ShgThalp:0.01138)0.817.7:0.0036)0.938.18:0.02648)0.964.23:0.04287,((((U
ltB8457:0.01518,ShgCompo:0.02145)0.938.19:0.02,((UltB8456:0.00374,ShgSpir
i:0.04834)0.615.5:0.01109,(UltB8451:0.0037,((ShgDaeje:0.0,FlaMizut:0.0,Ba
cEnr21:0.0,UltB8452:0.0,UltB8455:0.0,UltB8460:0.0):5.5E-
4,(UltB8453:0.00368,UltB8454:0.02258)0.915.17:5.5E-4)0.693.3:5.5E-
4)0.902.18:5.4E-4)0.910.26:0.01976)0.927.16:0.01963,((UltB8469:5.3E-
4,ShgComp2:0.00741)0.967.15:0.0114,(UltB8468:0.01117,((UltB8467:0.00787,(
(UltB8471:0.01892,UltB8470:5.3E-
4)0.421.3:0.00374,(UltB8472:0.00256,UltB8461:0.09661)0.412.3:0.00126)0.99
5.20:0.03184)0.836.11:0.00738,((UltB8464:0.00369,(UltB8465:5.5E-
4,UltB8466:5.5E-4)0.702.1:5.5E-
4)0.748.13:0.00346,(UltB8459:0.00734,UltComp2:0.01554)1.000.104:0.06369)0
.851.16:0.00788)0.907.20:0.01543)0.168.2:5.5E-
4)0.769.16:0.00374)0.907.21:0.01511,ShgSpec5:5.4E-
4)0.913.17:0.02611)0.548.4:0.01286)0.972.15:0.02533)0.938.20:0.02888)0.82
2.9:0.01604)0.845.17:0.02125,ShbBact4:0.03801)0.986.11:0.06154,((((UltOrg
77:0.15698,UltB8282:0.09918)0.883.19:0.03605,((((UltRike9:0.13113,(UltB82
03:0.07821,UltB8204:0.03231)0.868.16:0.03337)0.704.3:0.01144,(UltB8205:0.
06893,(UltB8198:0.04743,((UltB8202:0.06823,(UltB8199:0.03291,(UltB8200:0.
01844,UltB8201:0.10998)0.965.20:0.06239)0.796.9:0.0208)0.887.17:0.04048,(
UltB8196:0.01734,UltB8197:0.04015)0.956.17:0.05679)0.780.16:0.0373)0.922.
19:0.04685)0.958.18:0.04843)0.886.18:0.03484,(((UltB8192:0.04808,UncUn108
:0.01814)0.891.16:0.03671,((UncUn103:0.01365,UltB8184:0.01351)0.991.17:0.
06017,((Pshggg30:0.05861,(UltB8177:0.03172,((((UltB8173:0.03107,((Ult4153
8:0.0,UltB8170:0.0,UltB8171:0.0):0.01039,UltB8172:0.01325)0.949.20:0.0339
1)0.901.23:0.04145,((UncUn102:0.0,UncUn529:0.0):0.03034,UltB8175:0.00552)
0.948.21:0.04031)0.398.4:0.00384,UltB8176:0.01121)0.910.27:0.0116,(PPaYyy
y2:0.03663,(EchYyyy5:0.01193,(PshgYyy9:0.01133,(UltB8174:0.0037,Pshggg29:
5.5E-4)0.787.17:5.5E-
4)0.752.10:0.00323)0.877.24:0.01119)0.950.18:0.02017)0.000.221:5.5E-
4)0.748.14:0.02194)0.345.3:0.02949,(UltRike7:0.00343,(UltB6034:0.05391,Ul

tB8183:0.01529)0.494.1:0.0044)0.874.21:0.02702)0.819.17:0.01759)0.928.16:
0.03777)0.503.1:0.01175,(((((UltRike4:0.01672,(((UncUn354:0.0,UncUnc96:0.
0):0.02072,(Ult42017:0.0,UltB8153:0.0):0.01048)0.982.7:0.03151,(UncUn518:
0.00753,UncUnc95:0.00364)0.939.16:0.01933)0.843.13:0.0121)0.728.6:0.00234
,(Pshggg31:0.00745,(UltB8167:0.00746,(UltB8165:5.5E-
4,UltB8166:0.00743)0.788.12:0.00371)0.877.25:0.00747)1.000.105:5.5E-
4)0.921.31:0.01153,(UltB8154:0.00823,UltB8155:0.01068)0.998.20:0.04421)0.
899.17:5.4E-
4,(EchggYy8:0.0506,(UncUn100:0.02321,(UltB8169:0.00723,((UltB8163:5.4E-
4,(UncUn615:0.00371,UncUnc99:5.5E-
4)0.846.12:0.00368)1.000.106:0.03569,(UltB8164:0.01528,UltB8168:0.01139)0
.777.16:0.00371)0.075.1:5.4E-
4)0.883.20:0.01545)0.874.22:0.01643)0.782.20:0.00841)0.909.14:0.00787,(((
UltRike6:0.00771,Bfhggg31:0.01106)0.983.11:0.03202,(((((UltB8150:5.5E-
4,(BcdBact4:0.0,UltB8139:0.0):0.00366)0.988.18:0.04347,(UltB8072:0.02552,
(UncUnc93:0.00475,UltB8073:0.01478)0.882.12:0.01357)0.879.15:0.01722)0.92
6.16:0.02071,(((UltB8143:0.0071,(UltB8124:0.01903,UltB8142:0.00744)0.420.
5:5.3E-
4)0.795.19:0.0143,(((Ult19281:0.05148,UltB8606:0.02598)0.885.14:0.01265,(
(UltB8149:0.0,UltB8083:0.0,UltB8098:0.0,AtsPutre:0.0,UltB8152:0.0):5.5E-
4,(UltB8595:0.0037,Ult41501:0.00739)0.798.16:5.5E-4)0.745.12:5.5E-
4)0.815.10:5.4E-
4,(UltB8076:0.0163,UltB8077:0.0163)0.928.17:0.01629)0.891.17:0.01007)0.85
5.12:0.0095,(((((((UltB8140:0.00763,((UltAlis2:5.5E-
4,(UltAlist:0.00746,((UltB8133:0.00369,(UltB8128:5.5E-4,UltB8129:5.5E-
4)0.739.17:5.5E-
4)0.808.7:0.00374,UltB8135:0.00758)0.783.14:0.0037)0.872.28:5.5E-
4)0.965.21:5.5E-
4,UltB8134:0.01103)0.779.14:0.00363)0.946.18:0.01144,UltB8605:0.00377)0.7
50.9:0.00358,UltB8138:0.0193)0.873.24:0.00748,(UltB8132:0.01127,UltB8599:
0.01901)0.736.8:0.00376)0.788.13:0.00372,((((AtsOnder:0.0,UltB8110:0.0,Ul
tB8102:0.0,UltB8109:0.0):0.00367,((UltRike2:0.0102,UltB8111:0.01861)0.768
.10:0.00652,(((UltB8089:0.02211,(UltB8097:0.02253,(((UltB8106:0.0681,(((U
ltB8091:0.0,UltB8096:0.0):0.0037,(UltB8090:0.00372,Ult41455:5.5E-
4)0.285.3:5.5E-
4)0.768.11:0.0037,(((UltB8093:0.00368,UltB8596:0.03462)0.907.22:5.5E-
4,(UltB8092:0.0,UltB8095:0.0):5.5E-4)0.721.2:5.5E-
4,UltB8094:0.00369)0.674.2:5.5E-4)0.403.2:5.5E-
4)0.902.19:0.00738,UltB8088:5.5E-4)0.789.11:5.4E-
4,UltB8087:0.01508)0.794.17:0.00565)0.384.1:0.00784)0.110.3:0.00561,(UltB
8078:0.0063,(UltB8597:0.03449,(UltB8114:0.00379,(UltB8112:0.03044,UltB811
3:5.5E-
4)0.771.20:0.0036)0.811.12:0.00853)0.910.28:0.01592)0.927.17:0.01933)0.80
7.8:0.0063,UltB8125:0.00861)0.814.7:0.00772)0.755.17:0.00313)0.939.17:0.0
1146,(UltB8119:0.00743,((UltB8107:5.5E-
4,UltB8108:0.00422)0.991.18:0.02679,((UltB8115:0.0,UltB8117:0.0,UltB8080:
0.0,UltB8082:0.0,UltB8118:0.0):5.5E-
4,(UltB8103:0.00744,(AtsSeneg:0.0037,UltB8116:5.5E-
4)0.840.15:0.00369)0.104.2:5.4E-4)0.926.17:5.3E-
4)0.912.20:0.00744)0.785.20:5.5E-
4)0.342.2:0.0037,((UltB8122:0.00369,((UltB8121:0.00369,UltB8123:0.01492)0
.461.2:5.5E-4,(UltB8600:0.00369,UltB8127:5.5E-4)0.766.13:5.5E-
4)0.748.15:5.2E-4)0.971.10:0.01499,(((UltB8603:5.5E-
4,UltB8604:0.00369)0.925.26:0.00741,UltB8126:0.00369)0.692.3:5.5E-

4,AtsFineg:5.5E-4)0.569.1:5.5E-4)0.836.12:5.4E-
4)0.899.18:0.00746)0.779.15:0.00423,(UltB8141:0.00371,((UltB8120:0.0,UltB
8594:0.0):0.03557,((UltB8079:0.00368,UltB8081:5.5E-
4)0.998.21:0.0364,((UltB8130:0.01297,UltB8131:0.00364)0.933.21:0.01744,(U
ltB8084:0.00369,(UltB8085:0.01492,UltB8086:0.00369)0.134.4:5.3E-
4)0.929.15:0.01619)0.942.16:0.0164)0.792.15:7.0E-
4)0.579.2:0.00193)0.917.15:0.01101)0.337.1:0.00372,((UltB8144:5.5E-
4,UltB8145:0.00369)0.859.15:0.00759,(UltB8075:0.0038,(UltB8074:0.01538,Un
cUnc94:0.01148)0.763.21:0.0035)0.958.19:0.01552)0.871.14:0.011)0.770.19:0
.00434)0.774.27:0.00398)0.947.18:0.02077,((UltB8151:5.5E-
4,UltB8101:0.015)0.777.17:0.00393,(UltB8104:0.02688,((UltB8136:5.5E-
4,UltB8612:0.051)0.833.20:0.00368,UltB8137:5.5E-4)0.993.11:5.5E-
4)0.781.16:0.00346)0.973.11:0.03013)0.816.14:0.01641,((UltRike3:0.03129,U
ncUn101:0.01919)0.797.15:0.00911,((UltB8162:0.06013,UltRike5:0.04242)0.91
2.21:0.02247,(UltB8161:0.03488,(UltB8574:0.0074,UltB8598:5.5E-
4)0.983.12:0.03269)0.745.13:0.00424)0.866.16:0.01251)0.287.1:0.00426)0.76
4.17:0.00693)0.932.18:0.01851,(((UltB8195:0.03782,(UltB8194:0.01957,Otu01
154:0.00738)0.933.22:0.02916)0.967.16:0.03863,((UltB8191:0.03457,((UltRik
e8:0.04493,((UncUn107:0.0,UncUn580:0.0):0.01898,(UltB8188:5.5E-
4,(UltB8187:0.00138,UltB8186:0.01045)0.933.23:0.0146)0.848.17:0.01215)0.9
51.17:0.03462)0.810.12:0.02469,(UltB8046:0.02662,((UltB8049:0.01304,((Ult
B8051:0.01549,UltB8041:0.00345)0.971.11:0.02415,UltB8048:0.00375)0.893.23
:0.01344)0.896.14:0.01208,((UltB8042:5.4E-
4,(UltB8045:0.01165,(((UltB8047:0.01178,UncUnc84:0.02738)0.740.10:0.00537
,(UltB8043:0.01822,UltB8044:0.02329)0.605.3:0.00991)0.926.18:0.01767,(((U
ncUnc78:0.0,UncUnc82:0.0,UncUnc79:0.0,UncUnc83:0.0):5.5E-
4,UncUnc80:0.00369)0.892.16:5.5E-4,((UltB8039:0.0037,UltB8040:5.5E-
4)0.911.24:0.0021,(UncUn461:0.0,UncUnc77:0.0,UncUnc81:0.0):0.007)0.776.15
:0.00208)0.792.16:0.00391)0.760.16:0.00347)0.959.20:0.01546)0.884.18:0.00
95,(((((UltB8054:5.4E-
4,((Ult41420:0.0,UltB8065:0.0):0.01443,((((UltB8055:0.01512,UltB8056:0.00
381)0.954.17:0.01933,UltB8057:5.4E-4)0.765.12:5.4E-
4,(UltB8062:0.00763,((((UncUnc87:0.01543,(UncUn585:0.0,UncUnc91:0.0):0.01
148)0.896.15:0.01108,((UltB8070:0.00723,UltB8069:0.00722)0.929.16:5.4E-
4,((UltB8066:0.00369,UncUnc92:0.02301)0.851.17:5.4E-
4,UltB8052:0.01511)0.439.4:0.0037)0.770.20:0.00197)0.928.18:0.00198,(((Ul
tB8064:0.02326,(((UncUnc90:0.04034,UltB8067:5.4E-
4)0.341.4:0.0077,UltB8059:0.03199)0.362.3:5.5E-
4,UltB8058:0.01092)0.887.18:0.01094)0.881.19:5.4E-4,UltB8053:5.5E-
4)0.936.19:0.00369,((UncUnc88:5.4E-
4,(UltB8068:0.00372,((UncUn501:0.0,UncUnc86:0.0):5.5E-
4,UncUnc85:0.01122)0.782.21:0.00368)0.902.20:0.00746)0.848.18:0.00368,Ult
B8063:0.02305)0.740.11:5.4E-4)0.961.13:5.5E-
4)0.821.12:0.01086,UltB8061:0.01894)0.895.22:5.5E-
4)0.860.13:0.00743)0.784.13:5.5E-
4,HmZZZZ03:0.05977)0.883.21:0.00984)0.775.13:0.00545)0.864.21:0.00788,Ult
B8060:0.01099)0.946.19:0.02121,UltB8071:0.04026)0.723.10:0.00453,UncUnc89
:0.03017)0.966.22:0.02601,UltB8050:0.01524)0.850.16:0.00972)0.543.2:5.4E-
4)0.813.9:0.01021)0.925.27:0.02364)0.883.22:0.01588)0.518.2:0.00815,(UncU
n106:0.02776,((UncUn105:0.0,UncUn560:0.0,UncUn104:0.0):0.01709,UltB8185:0
.00645)0.878.19:0.01882)0.845.18:0.0155)0.412.4:0.00905)0.934.16:0.02589,
((UltB8158:0.01555,(UncUnc97:5.5E-4,(UltB8159:0.00391,(UncUnc98:5.3E-
4,Bfhggg30:0.05296)0.775.14:0.00345)0.851.18:0.00747)0.897.11:0.01133)0.7
21.3:0.00394,(UltB8156:0.019,UltB8157:0.02013)0.945.19:0.02478)0.411.3:0.

01463)0.831.19:5.7E-
4)0.740.12:0.00322)0.856.10:0.01727)0.862.18:0.04092)0.812.5:0.02243,((((
UltB8024:0.04838,((((Tryy0090:0.0,Tryyy093:0.0):0.02275,((UltB8206:5.3E-
4,UncUn109:0.00742)0.846.13:0.01238,(Otu00391:0.03025,((Otu00150:5.4E-
4,Otu00696:0.02726)0.970.17:0.03759,(UltB8207:0.03527,UltB8208:0.05204)0.
692.4:0.0167)0.817.8:0.01389)0.808.8:0.01265)0.736.9:0.01257)0.410.4:0.00
556,Otu00360:0.01143)0.999.22:0.12964,Otu00320:0.06129)0.944.15:0.05915)0
.883.23:0.05241,(((Bfhggg29:0.01333,UltB7903:0.03951)0.979.14:0.04658,(((
(((((UltB7826:0.01625,UltB7827:0.00695)0.979.15:0.04408,(((UltB8021:0.003
68,UltB8022:5.3E-
4)0.930.11:0.0261,(UltB7902:0.00531,(UltB7901:0.00369,(UltB7899:5.5E-
4,(UltB7900:0.0,UltProk2:0.0):5.5E-4)0.813.10:5.5E-
4)0.908.16:0.01839)0.957.17:0.03433)0.841.14:0.01894,(UltB7891:0.02581,((
(((UltRu222:0.01529,(UltB5942:0.00742,((((UltB7859:0.0,UltB7861:0.0,UltB7
862:0.0,UltB7864:0.0,UltB7866:0.0,UltB8027:0.0):5.5E-
4,(UltB7867:0.03132,UltB7860:0.00368)0.604.4:5.5E-4)0.125.2:5.5E-
4,UltB7865:0.06806)0.052.2:5.5E-4,UltB7863:5.5E-4)0.565.3:5.5E-
4)0.780.17:0.00399)0.775.15:0.0091,(((UltB7360:0.03552,UltRu226:0.04605)0
.809.16:0.01969,((UltRu220:0.01111,(UltRu216:0.01525,(UltRu218:5.4E-
4,((UltB7890:0.0,UltB7868:0.0,UltRu219:0.0,UltB8030:0.0):5.5E-
4,UltB7869:0.00369)0.869.18:0.0037)0.928.19:0.00778)0.046.1:5.4E-
4)0.830.17:0.00709,((UltRu217:5.4E-
4,UltB7870:0.00362)0.112.1:0.0037,(UltB7871:5.4E-
4,UltRu221:0.0153)0.697.3:5.4E-
4)0.815.11:0.00368)0.578.2:0.0027)0.845.19:0.01335,UltRu215:0.00792)0.814
.8:0.00687)0.842.19:0.01068,(UltB7895:0.0273,(UltB7894:0.04052,(UltRu223:
0.01518,((UltRu224:0.0,UltB7893:0.0):5.5E-
4,(UltB7892:0.00369,UltRu225:0.01143)0.717.8:5.5E-4)0.995.21:5.5E-
4)0.860.14:0.02389)0.988.19:0.06427)0.945.20:0.04171)0.473.2:0.00307,(Ult
B7898:0.00935,(UltB7896:0.00333,UltB7897:0.0405)0.947.19:0.0236)0.952.23:
0.02513)0.757.10:0.00828,(UltB7872:5.4E-
4,UltB7880:0.09941)0.600.4:0.01363)0.373.2:0.00963)0.758.18:0.00892)0.983
.13:0.04985)0.888.24:0.01598,((UltB7978:0.04112,(((UltB7947:0.01435,((Ult
B7944:0.0,UltB7945:0.0):0.0015,UltB7946:0.01039)0.862.19:0.01319)0.916.22
:0.01811,(((UltB7948:0.00443,(UltB7949:0.01759,UltB7976:0.01773)0.912.22:
0.01038)0.881.20:0.00446,((((UltB7927:0.00371,UltB7943:0.0037)0.562.3:5.5
E-4,(UltB7925:0.0318,(UltB7926:0.01844,UltB7968:5.4E-
4)0.755.18:0.00788)0.812.6:0.00654)0.792.17:0.00369,((((UltB7930:0.01782,
(UltB7934:0.00373,(UltB7928:5.4E-
4,(UltB7970:0.01145,((((UltB7972:0.00695,((UltB7931:0.0074,UltB7971:0.003
69)0.919.23:5.4E-
4,UltB7933:0.03105)0.903.14:0.00206)0.908.17:0.00208,(UltB7924:0.0,UltB79
73:0.0):5.4E-4)0.760.17:0.00749,UltB7932:5.4E-
4)0.853.15:0.00713,(UltB7917:0.00319,UltB7974:0.03947)0.898.25:0.01152)0.
900.17:5.5E-
4)0.952.24:0.0114)0.759.16:0.00365)0.808.9:0.00829)0.365.1:0.00812,(UltB7
736:0.03627,(((UltB7935:0.00369,((UltB7919:0.04549,(UltB7921:0.01132,UltB
7957:0.00749)0.759.17:0.0036)0.763.22:0.00388,UltB7967:5.4E-
4)0.824.20:0.00381)0.544.1:5.5E-
4,UltB7936:0.01904)0.864.22:0.00751,UltB7938:0.00804)0.758.19:0.00364)0.8
88.25:0.01189)0.907.23:5.5E-
4,UltRiken:0.0037)0.921.32:0.00242,(UltB7942:0.00373,(UltB7952:0.0,UltB79
53:0.0):0.00747)0.921.33:0.00245)0.921.34:0.00244)0.783.15:0.0036,UltB797
5:0.0076)0.840.16:0.00752)0.888.26:0.00787,((((UltB7940:5.5E-

4,((UltB7954:0.00696,(UltB7955:0.0,UltB7956:0.0):0.0021)0.912.23:0.00207,
UltB7939:0.01502)0.660.3:5.4E-4)0.902.21:5.4E-
4,((UltB7958:0.0,UltB7965:0.0):5.4E-
4,(UltB7923:0.01146,UltB7879:0.03999)0.874.23:0.0112)0.772.18:0.01117)0.3
27.3:0.00402,(UltB7962:5.5E-
4,((UltB7959:0.00788,((UltB7920:0.0,UltB7937:0.0):0.00251,(UltB7966:0.026
71,(UltB7941:0.0,UltB7964:0.0):5.5E-
4)0.960.22:0.00141)0.884.19:0.01111)0.333.3:0.00752,(UltB7961:0.02382,Ult
B7969:0.02767)0.742.11:0.00258)1.000.107:5.3E-
4)0.861.20:0.00739)0.197.3:0.00726,(UltB7951:5.4E-
4,(UltB7950:0.00377,UltB7960:0.00756)0.804.13:0.00369)1.000.108:5.4E-
4)0.211.2:0.0032)0.920.18:0.01605)0.843.14:0.00945,((UltB7979:0.02071,Ult
Ru227:0.01165)0.915.18:0.02185,UltB7980:0.03845)0.747.15:0.01222)0.937.19
:0.01956)0.751.5:0.00379,(((UltB7913:0.03161,(UltB7911:0.03551,((UltB7910
:0.01955,(UltB7908:0.02639,UltB7909:5.4E-
4)0.947.20:0.01942)0.875.19:0.01588,UltB7914:0.03573)0.176.6:0.00946)0.45
3.2:0.00741)0.965.22:0.03601,(UltOrg74:0.036,(UltB7831:0.00368,UltB7832:5
.5E-
4)0.994.19:0.04659)0.787.18:0.00966)0.943.21:0.02536,(UltB7836:0.03556,((
UltB7838:0.04501,UltB7839:0.00421)0.970.18:0.03892,UltB7837:0.01984)0.554
.2:0.01403)0.941.15:0.03396)0.788.14:0.00758)0.862.20:0.01248)0.317.5:5.2
E-
4,(UltB7810:0.08308,((BtdBacte:0.06315,(BcdlOral:0.02475,(UltB7828:0.0036
9,(UltB7830:0.00369,UltB7829:5.5E-4)0.560.3:5.5E-
4)0.782.22:0.01121)0.991.19:0.07056)0.699.8:0.03025,(UltB7833:0.03188,Ult
B7834:0.05991)0.841.15:0.05207)0.831.20:0.02963)0.977.13:0.06564)0.488.4:
0.01133,(((((((UltB7857:0.01073,(((UltB7854:0.01162,(((((UltB7813:5.5E-
4,(UltB7815:0.0037,UltB7812:0.00377)0.629.4:5.5E-4)0.878.20:5.5E-
4,UltB7816:0.02695)0.894.15:5.5E-
4,UltB7811:0.02287)0.838.16:0.00967,UltB7814:0.00569)0.972.16:0.04905,Ult
B7817:0.03665)0.893.24:0.02875)0.931.19:0.03207,(UltRu172:0.01918,(UltRu1
71:0.01927,UltB7770:0.01752)0.799.15:0.01391)0.708.3:0.01671)0.740.13:0.0
1749,UltB7858:0.02333)0.290.4:5.5E-
4)0.943.22:0.01586,((UltB7758:0.0,UltRu167:0.0,UltRu168:0.0):5.3E-
4,(UltB7757:0.01113,(UltB7753:0.01419,(UltB7771:0.02461,(UltB7752:0.03871
,UltB7754:0.01873)0.717.9:0.02188)0.000.222:0.00773)0.043.3:5.4E-
4)0.929.17:0.00862)0.970.19:0.01858)0.724.7:5.4E-
4,UltB7751:0.02348)0.840.17:5.5E-
4,(UltB7756:0.01581,(((Ult22584:0.01134,(UltB7842:0.02753,UltB7769:0.0076
7)0.894.16:0.01076)0.050.2:5.4E-4,((UltB7761:0.01109,UltB7762:5.5E-
4)0.886.19:0.01125,((((UltB7747:0.01484,UltB7749:0.01217)0.588.4:0.00825,
(UltB7750:0.01,(UltB7743:0.00369,(UltB7748:5.5E-
4,UltB7760:0.00725)0.888.27:5.5E-
4)0.802.12:0.00558)0.899.19:0.01539)0.800.10:0.00896,(UltB7851:0.0,UltB78
52:0.0):0.02291)0.723.11:0.00596,(((((((UltRu157:0.0,UltRu158:0.0,UltRu1
69:0.0):5.5E-
4,UltRu162:0.01512)0.839.16:0.00368,UltB7745:0.08534)0.113.3:5.5E-
4,(UltB7759:0.00369,UltB7744:5.5E-4)0.918.21:5.5E-4)0.880.23:5.3E-
4,UltB7746:0.00732)0.776.16:0.00354,UltB7739:0.0076)0.769.17:0.00364,(((U
ltRu163:0.00642,(UltRu160:0.00202,(UltRu164:0.03049,(UltRu166:0.00367,Ult
Ru165:5.5E-4)0.970.20:5.5E-
4)0.951.18:0.00188)0.969.17:0.01466)0.777.18:0.00356,UltRu161:0.00654)0.8
20.7:0.00635,UltRu159:0.00383)0.878.21:0.00753)0.880.24:0.00729,((UltB776
6:0.01109,UltB7765:5.5E-

4)0.879.16:0.00718,(((UltB7742:0.0,UltB7767:0.0):5.5E-
4,UltB7768:0.05506)0.996.10:0.04967,((UltB7763:0.00735,(UltB7764:5.5E-
4,UltB7737:0.00369)0.748.16:5.4E-4)0.699.9:5.5E-
4,UltB7740:0.01884)0.364.3:0.00725)0.721.4:0.00355)0.757.11:0.00445)0.846
.14:0.00735)0.787.19:0.00431)0.880.25:0.00741)0.792.18:0.00539,UltB7755:0
.03984)0.745.14:0.00581)0.859.16:0.01121)0.871.15:0.01058,(((UltB7783:0.0
1559,UltB7846:0.03196)0.779.16:0.00341,(UltB7843:0.00369,((((UltB7777:0.0
2288,UltB7844:5.4E-
4)0.824.21:0.01926,(((UltB7845:0.01217,(UltB7849:0.04163,(UltB7848:5.4E-
4,UltB7847:0.00366)0.986.12:0.02958)0.688.2:0.00205)0.933.24:0.01908,(Ult
B7779:5.4E-
4,((UltB7780:0.00384,UltB7781:0.02279)0.859.17:0.0072,(((UltB7773:0.0,Ult
B7774:0.0,UltB7775:0.0):5.5E-4,UltB7776:0.00369)0.901.24:5.4E-
4,UltB7778:0.0393)0.859.18:0.00755)0.986.13:0.01968)0.981.8:0.02353)0.597
.4:0.00896,UltB7785:5.3E-
4)0.917.16:0.01847)0.792.19:0.00771,((UltB7741:0.00958,UltB7789:0.03485)0
.782.23:0.01289,(((BtdBact2:0.0,PrkSpeci:0.0):0.07097,(UltB7784:5.5E-
4,(UltB7883:0.01117,UltB7782:5.5E-4)0.791.12:0.00369)0.904.14:5.5E-
4)0.229.3:0.00818,UltB7786:0.02497)0.826.11:0.01061)0.754.15:0.00483)0.95
3.15:0.02179,UltB7855:0.01108)0.882.13:5.5E-
4)0.786.14:0.00375)0.786.15:0.00382,((UltRu170:0.01247,UltB7850:0.03134)0
.733.7:0.00376,(UltB7787:0.0,UltRu173:0.0):0.05021)0.796.10:0.00709)0.861
.21:0.00814)0.871.16:0.02094,UltB7788:0.03882)0.930.12:0.03094,(Ult22585:
0.03345,(UltB7822:0.04603,(((UltB7819:0.01326,(UltRu214:0.08148,UltRu212:
0.03437)0.312.2:0.00395)0.931.20:0.03036,(UltB7818:5.5E-4,UltRu211:5.5E-
4)0.985.17:0.04917)0.279.2:0.014,(UltRu213:0.00418,UltB7856:0.10438)0.801
.14:0.01576)0.966.23:0.04284)0.879.17:0.02904)0.850.17:0.02456)0.845.20:0
.02534,((UltB7721:0.08747,((UltRu150:0.01433,(UdnRum20:0.0161,(UltRu149:5
.5E-
4,(UltB7722:0.039,(UltB7724:0.00522,((UltRu151:0.00618,(UltB7725:0.02062,
UltB7726:0.03584)0.802.13:0.01364)0.229.4:0.01075,(((((((UltB7715:5.5E-
4,((UltB7696:0.0,UltB7698:0.0,UltB7700:0.0,UltB7701:0.0,UltB7702:0.0,UltB
7703:0.0,UltB7704:0.0,UltB7689:0.0,UltB7690:0.0,UltB7691:0.0,UltB7705:0.0
,UltB7706:0.0,UltB7707:0.0,UltB7708:0.0,UltB7709:0.0,UltB7714:0.0):5.5E-
4,UltB7713:0.0037)0.765.13:5.5E-4)0.687.5:5.5E-
4,UltB7711:0.00368)0.209.2:5.5E-4,(UltB7692:0.01111,((UltB7710:5.5E-
4,(UltB7720:0.01487,UltB7695:0.02252)0.902.22:5.4E-
4)0.859.19:0.00369,(UltB7699:0.00745,UltB8029:0.02286)0.872.29:0.00744)0.
074.2:5.5E-4)0.276.4:5.5E-4)0.999.23:5.5E-
4,UltB7693:0.02214)0.931.21:0.00719,(UltB7694:5.5E-
4,UltB7716:0.00369)0.991.20:5.3E-4)0.680.2:0.0019,(UltB7683:5.5E-
4,(UltB7682:5.5E-4,(((UltB7719:0.01112,UltB7688:0.01126)0.073.2:5.5E-
4,UltB7681:0.00369)0.536.3:5.5E-
4,(UltB7684:0.0,UltB7685:0.0,UltB7686:0.0,UltB7687:0.0):5.5E-
4)0.665.5:5.5E-4)0.574.1:5.5E-
4)0.920.19:0.00689)0.898.26:0.00205,(UltB7717:0.00741,(UltB7697:5.5E-
4,UltB7712:5.5E-4)0.648.3:5.3E-
4)0.932.19:0.01135)0.929.18:0.01744)0.730.7:0.00746)0.960.23:0.0217)0.867
.19:0.013)0.773.20:0.00689)0.930.13:0.02161)0.428.3:0.02001,(UltB7723:5.5
E-
4,UltB8608:0.00369)0.837.11:0.01503)0.765.14:0.01218)0.978.9:0.04722,((((
(UltB7823:0.01357,((UltB7820:0.01875,UltB7821:5.5E-
4)0.981.9:0.04211,(UltB7824:0.0063,UltB7825:0.00513)0.862.21:0.01616)0.95
4.18:0.03315)0.962.21:0.03841,(UltRu154:0.04542,((UltRu152:0.03127,UltRu1

53:0.02651)0.865.19:0.01931,(UltB7729:0.01116,((UltB7727:0.0,UltB7884:0.0
):5.5E-4,UltB7728:0.00369)0.984.8:5.4E-
4)0.966.24:0.03261)0.335.5:0.00485)0.883.24:0.02489)0.808.10:0.01428,(Ult
B7734:0.07365,(((UltRu207:0.04394,((UltRu204:0.00397,(UltRu209:0.10073,((
((UltRu203:0.02398,((UltRu202:5.4E-4,(UltB7805:5.4E-
4,(((UltB7807:0.01424,UltRu137:0.06468)0.959.21:0.02495,((UltRu194:0.0,Ul
tRu200:0.0,UltB7806:0.0,UltRu201:0.0):5.5E-
4,(UltRu193:0.00743,(UltLachn:0.0,UltRu360:0.0):0.01117)0.743.16:5.5E-
4)0.987.16:5.4E-
4)0.922.20:0.00244,UltRu195:0.00246)0.922.21:0.00246)0.912.24:0.00748)0.8
50.18:5.3E-4,(UltB7803:0.02339,((((UltRu198:5.5E-4,UltB7799:5.5E-
4)0.985.18:5.5E-
4,UltRu197:0.01126)0.826.12:0.00369,UltB7804:0.00369)0.401.1:5.5E-
4,(BcdBact2:0.0,BcdBact3:0.0,UdnRum21:0.0):5.5E-4)0.985.19:5.5E-
4)0.891.18:0.0109)0.869.19:0.01542)0.857.25:0.01277,(UltB7808:5.4E-
4,(UltRu196:0.01118,(UltB7801:0.0,UltB7800:0.0,UltB7802:0.0):0.02325)0.93
2.20:0.01934)0.892.17:0.01388)0.987.17:0.04186,(UltRu180:0.01735,(UltRu18
3:0.00996,(UltRu179:0.00897,(UltRu189:0.00327,(UltRu185:0.01117,(UltRu184
:5.5E-4,UltB7793:0.00743)0.046.2:5.5E-
4)0.901.25:0.00804)0.801.15:0.00913)0.827.15:0.01124)0.927.18:0.02232)0.8
67.20:0.01707)0.774.28:0.01064,(UltRu181:0.0309,((((UltB7797:0.01533,(Ult
Ru186:5.5E-
4,UltB7794:0.00369)0.764.18:0.00394)0.265.1:0.01153,(UltRu187:5.5E-
4,(UltB7735:0.01529,UltB7795:5.4E-
4)0.945.21:0.00745)0.901.26:0.00731)1.000.109:5.5E-
4,((UltRu188:0.00749,UltRu210:0.00374)0.933.25:5.5E-
4,(UltRu190:0.00388,(UltRu182:0.00473,UltRu191:0.019)0.975.15:0.02347)0.9
36.20:0.01512)0.840.18:0.00724)0.975.16:0.01506,((UltRu175:5.4E-
4,UltRu178:0.01463)0.863.15:0.00709,((((UltB7791:0.0,UltB7792:0.0):5.5E-
4,UltRu205:0.22768)0.763.23:0.01504,(UltRu192:0.01572,UltRu206:0.01958)0.
741.9:0.00346)0.865.20:0.00767,((UltB7798:0.00699,(UltB7790:5.4E-
4,UltRu176:0.00746)0.911.25:0.00193)0.884.20:0.00217,(UltB7796:0.00585,(U
ltRu177:5.4E-
4,UltRu174:0.01106)0.769.18:0.01063)0.814.9:0.00646)0.959.22:0.01538)0.97
3.12:5.5E-4)0.910.29:5.4E-4)0.000.223:5.4E-
4)0.687.6:0.02044)0.648.4:0.00235)0.920.20:0.03551)0.909.15:0.04991,UltRu
208:0.04423)0.978.10:0.05557)0.906.26:0.04254,UltB7809:0.10366)0.946.20:0
.04025,(UltB7730:0.09556,UltRu155:0.0685)0.841.16:0.02824)0.790.11:0.0211
9)0.849.15:0.02564)0.774.29:0.01251,((UltB7841:0.01794,UltB7840:0.009)0.9
91.21:0.05882,(UltRu156:0.01115,UltB7731:5.5E-
4)0.999.24:0.07819)0.749.23:0.01174)0.900.18:0.01902,(UltB7835:0.04473,((
UltB7886:0.0,UltB7888:0.0,UltB7889:0.0):5.5E-
4,UltB7887:0.00368)0.996.11:0.07155)0.850.19:0.02236)0.572.4:0.01058)0.91
4.17:0.02364)0.674.3:0.01255)0.895.23:0.02967,(UltB7929:0.19072,((UltB790
4:0.0,UltB7905:0.0,UltB7907:0.0):5.5E-
4,UltB7906:0.00335)0.888.28:0.04388)0.296.3:0.03741)0.743.17:0.02128,(AtP
Yy111:0.10025,(UltB7732:0.05936,UltB7733:0.13663)0.934.17:0.05296)0.785.2
1:0.01749)0.877.26:5.4E-
4,(UltB7877:0.08185,(UltB7876:0.06045,(UltB7878:0.0488,(((UltB8382:5.4E-
4,UltB7881:0.07685)0.912.25:0.01971,UltB7885:0.04053)0.718.8:0.00306,UltB
7882:5.4E-
4)0.995.22:0.06902)0.000.224:0.00378)0.843.15:0.01669)0.941.16:0.04364)0.
847.21:0.01889)0.147.2:5.5E-4,((Bfhggg26:5.3E-
4,(BfhYyy21:0.0,Bfhggg28:0.0):0.02292)0.778.19:0.0069,(Pshggg28:0.06392,(

Bfhggg27:5.4E-
4,BfhgYyy2:0.03965)0.000.225:0.00341)0.349.2:0.01274)0.979.16:0.0452)0.70
1.6:0.01886)0.594.2:0.01179,((UltB7875:0.08742,((UltRu229:5.5E-
4,UltRu228:0.00367)0.976.11:0.08877,(UltB8019:0.20601,(UltB8018:0.05033,(
UltB8016:0.01277,UltB8017:0.01043)0.987.18:0.06857)0.852.20:0.04067)0.591
.5:0.06024)0.861.22:0.04457)0.781.17:0.03248,(UltB8023:0.07615,(((UltB801
2:0.00359,UltB8013:0.00382)0.927.19:0.0242,(UltB8014:5.5E-
4,UltB8025:0.00369)0.907.24:0.02474)0.962.22:0.03653,(((UltB8209:0.00764,
(UltB8210:0.00733,Ult19278:5.5E-
4)0.860.15:0.00718)0.751.6:0.00835,(UltB7874:0.01895,UltB7873:0.01537)0.9
77.14:0.03716)0.114.1:0.02299,(UltB8020:0.01206,UltB8015:0.09307)0.981.10
:0.05942)0.651.6:0.00771)0.942.17:0.04247)0.494.2:0.02614)0.883.25:0.0438
)0.884.21:0.03538,(((UltB7995:0.02544,UltB7998:0.00983)0.975.17:0.04107,(
UltB7997:0.0551,((UltB7989:5.5E-
4,UltB7990:0.00726)0.876.19:0.01629,(UltB7991:5.5E-
4,(Ult12369:0.03849,((UltB7993:5.5E-
4,UltB8026:0.00369)0.845.21:0.0037,UltB7992:0.0037)0.992.17:5.5E-
4)0.953.16:0.0152)0.947.21:0.0232)0.262.2:0.01391)0.854.13:0.01697)0.787.
20:0.01093,((((UltB8003:0.03028,Otu00040:0.049)0.912.26:0.0243,(UltB8004:
0.01167,(Otu00129:0.02539,Otu01042:0.02597)0.881.21:0.01628)0.320.1:0.004
4)0.786.16:0.00906,(UncUnc76:0.03642,Z0114470:0.00851)0.902.23:0.01755)0.
829.13:0.00996,((((UltB8011:0.0,Otu00181:0.0):0.03533,((UltB8010:0.02892,
((Otu00025:0.01065,Otu00067:0.02917)0.812.7:0.01417,(Otu00080:0.00997,Otu
00163:0.0432)0.346.3:0.01751)0.939.18:0.03171)0.876.20:0.01736,Otu00104:5
.3E-
4)0.963.17:0.03525)0.993.12:0.0512,(((UltB8006:0.0,UltB8005:0.0):5.4E-
4,UltB8007:0.01915)0.959.23:0.01989,((AtPYy114:0.01876,UltB7994:0.03401)0
.948.22:0.03107,(UltB8008:0.02119,AtPYy116:0.04609)0.735.8:0.01095)0.327.
4:0.01161)0.714.6:0.00426)0.749.24:0.00383,((UltB7999:0.01148,(UltB8002:5
.4E-
4,(UltB8000:0.00575,UltB8001:0.01025)0.844.22:0.01029)0.524.2:0.00744)0.7
16.6:0.00768,(PahggY36:0.00719,UltB8009:0.04433)0.842.20:5.4E-
4)0.905.15:0.01479)0.866.17:0.0113)0.966.25:0.03935)0.932.21:0.04149)0.94
1.17:0.03213)0.821.13:0.02508)0.220.2:0.02089,(UltOrg75:0.09848,UltB8609:
0.03441)0.911.26:0.03378)0.680.3:0.01499,(((((UltB8619:0.0473,(UltB8390:0
.06036,((UltB8278:0.0738,((Pshggg34:0.01789,P0114514:0.11164)0.518.3:0.03
968,((PahggY38:0.00368,S0114495:5.5E-
4)0.968.12:0.05182,Otu02211:0.08024)0.000.226:0.00795)0.673.1:0.03202)0.0
00.227:0.02858,UltB2260:0.14471)0.836.13:0.02534)0.690.9:0.0196)0.878.22:
0.01263,(UltSph31:0.06128,((UltB8323:0.01812,UltB8324:0.0213)1.000.110:0.
11631,(((UltB8291:0.02746,(Otu01128:0.00363,(UltB8290:5.5E-
4,ZnvYyy02:0.00745)0.948.23:0.01136)0.917.17:0.01174)0.905.16:0.02023,(Ot
u01111:0.04943,((UltB8285:0.00369,UltB8284:5.5E-
4)0.909.16:0.02716,(UltB8287:5.4E-4,(UltB8286:5.5E-
4,UltB8288:0.02704)0.959.24:0.01888)0.903.15:0.0325)0.954.19:0.03261)0.69
1.4:0.00321)0.669.4:0.01032,(UltB8289:0.02935,((UltB8292:0.05511,(Otu0083
7:5.4E-
4,(Otu00079:0.03053,(UltB8293:0.02694,(UltB8294:0.0,Otu00614:0.0):5.5E-
4)0.748.17:0.01878)0.985.20:0.04447)0.812.8:0.02011)0.990.18:0.09572,UltB
8295:0.1054)0.718.9:0.0512)0.927.20:0.05389)0.925.28:0.0308)0.871.17:0.01
729)0.863.16:0.00982)0.713.7:0.00518,((((UltB8534:0.07253,(UltB8532:0.091
83,(UltB8530:0.00345,(UltB7680:5.5E-4,UltB8531:5.5E-4)0.912.27:5.4E-
4)0.942.18:0.05187)0.436.4:0.0244)0.892.18:0.02968,((UltB8270:5.4E-
4,((Tryy0091:0.03064,UltB8269:5.5E-

4)0.988.20:0.03889,(Cuiiii19:0.01138,AtPYy117:0.06158)0.620.4:0.01332)0.2
19.3:0.00998)0.861.23:0.02191,((UltB8279:5.3E-4,((((UltB8271:5.4E-
4,((UltB8276:0.00756,(Otu00272:0.02706,Otu00413:5.4E-
4)0.413.4:0.00737)0.996.12:0.03144,UltB8277:0.00383)0.928.20:0.0151)0.835
.16:0.00412,(AtPYy112:0.23327,UltB8280:0.01451)0.999.25:5.5E-
4)0.893.25:0.00766,(Otu00612:0.02313,UltB8275:0.01366)0.851.19:0.00969)0.
725.8:0.00245,((UltB8274:0.01877,UltB8273:0.02624)0.000.228:5.4E-
4,((UltB8272:0.00337,Pshggg35:0.03105)0.962.23:0.01534,EchggYy9:5.4E-
4)0.925.29:0.00794)0.444.2:0.00856)0.983.14:0.03573)1.000.111:0.10144,(((
(((((UltRu235:0.0,UltB8250:0.0,UltRu236:0.0):5.5E-
4,(UltB8251:0.00368,((UltB8249:0.0,Ult22892:0.0):5.5E-4,UltB8252:5.5E-
4)0.929.19:5.5E-4)0.874.24:0.00368)0.183.4:5.5E-
4,(UltB8248:0.00245,UltB8253:0.00245)0.916.23:0.00245)0.930.14:0.02404,((
(UltB8240:0.00371,(((UltB8238:0.0,UltB8241:0.0):5.5E-4,UltB8239:5.5E-
4)0.212.3:5.5E-
4,UltB8237:0.01879)0.886.20:0.00735)0.754.16:0.0036,((UltB8246:5.5E-
4,Ult24643:0.01877)0.994.20:0.02672,(UltB8247:0.03436,(UltB8242:0.04034,(
(UltB8243:0.00372,UltRu232:5.3E-4)0.958.20:0.01577,(UltRu233:5.4E-
4,(UltB8244:0.01131,(UltB5742:0.01888,UltB8245:5.5E-
4)0.866.18:0.00749)0.758.20:0.00371)0.772.19:0.00319)0.777.19:0.00739)0.2
26.2:0.01981)0.835.17:5.4E-
4)0.962.24:0.01537)0.886.21:0.0109,UltRu234:5.5E-
4)0.983.15:0.03514)0.659.4:0.0204,((UltRu230:0.02251,(UltRu237:0.08742,(U
ltB8235:0.10712,(UltB8236:0.0274,UltRu231:0.12358)0.081.2:0.0261)0.726.5:
0.01746)0.876.21:0.03002)0.820.8:0.01578,UltB8234:5.5E-
4)0.969.18:0.04628)0.807.9:0.02632,(UltB8267:0.00606,UltB8268:0.01689)0.9
15.19:0.02843)0.886.22:0.02269,(((Otu00694:0.01975,(Otu00361:0.00815,(Ult
B8259:0.01332,(UltB8258:0.00748,Otu00248:0.00369)0.940.14:0.01832)0.838.1
7:0.01112)0.948.24:0.02699)0.890.24:0.01644,(UltB8266:0.02388,(UltB8265:5
.3E-4,(UltB8263:5.5E-4,UltB8264:5.5E-
4)0.897.12:0.00719)0.968.13:0.03814)0.903.16:0.01962)0.778.20:0.00985,Ult
B8262:0.06273)0.804.14:0.01059)0.734.5:0.00807,(UltB8260:0.02831,(UltB826
1:0.02468,((UltB8256:0.00366,(UltB8255:0.01288,Pshggg32:0.02336)0.851.20:
0.00841)0.886.23:0.01124,((UltB8257:0.0,Otu00191:0.0):0.03979,(Pshggg33:0
.0059,(BfhggY34:0.0196,(UltB8254:0.01034,(P0114464:0.01112,PahggY37:0.011
36)0.779.17:0.00851)0.784.14:0.01539)0.970.21:0.03114)0.759.18:0.0057)0.7
56.21:0.00511)0.961.14:0.0304)0.959.25:0.02753)0.864.23:0.01125)0.743.18:
0.0057)0.342.3:0.02017)1.000.112:0.12356)0.911.27:0.03389,UltB8283:0.0795
4)0.825.12:0.02575,(UltB8281:0.02674,UltB8906:0.08722)0.933.26:0.02886)0.
731.4:0.00536)0.850.20:0.0207,(((UltB8335:0.04342,(((((UltB8337:0.0,Otu00
092:0.0):0.03417,(UltB8336:0.00381,Z0114493:0.00366)0.832.12:0.00985)0.94
4.16:0.0325,(UltB8332:0.03692,(UltB8330:0.02082,UltB8331:0.0533)0.964.24:
0.04262)0.959.26:0.034)0.962.25:0.04744,Hmyy0018:0.04791)0.159.1:0.01532,
((UltB8329:0.03296,(((UltRu248:0.0,UltRu247:0.0):5.5E-
4,UltRu246:0.00371)0.796.11:0.02148,((UltB8326:0.0,UltB8327:0.0):5.4E-
4,(BcdGenom:0.08149,UltB8328:0.07349)0.733.8:0.00304)0.976.12:0.04864)0.9
58.21:0.06105)0.998.22:0.11056,(UltB8333:5.3E-
4,UltB8334:0.03546)0.953.17:0.05175)0.838.18:0.02664)0.338.2:0.02268)0.99
9.26:0.09154,UltB8299:0.04581)0.722.3:0.03118,UltB8298:0.06215)0.791.13:0
.04003)0.897.13:0.0233,((((UltB8903:0.07968,CryIgnav:0.09312)0.948.25:0.0
4288,(((UltSapr4:0.06711,((((UltB9075:0.09547,((UltB9048:0.04019,((LewCoh
a2:0.00146,LewCohae:0.0258)0.999.27:0.06326,((LewAntar:0.0152,LewLutea:5.
4E-4)0.416.6:0.00576,(LewAgari:0.00344,(LewPersi:5.5E-
4,LewPers2:0.00741)0.985.21:0.01978)0.814.10:0.00998)0.609.6:0.02172)0.80

5.15:0.01438)0.485.4:0.0212,LewMarin:0.03804)0.019.2:0.00294)0.934.18:0.0
2738,((Ult28158:0.07454,(UltB9074:0.0342,BacEnr30:0.03413)0.596.2:0.01377
)0.687.7:0.00698,((((UltB8822:0.07578,UltB9059:0.0035)0.988.21:0.04743,((
(SrsGrand:0.10368,(SrsSpec2:0.11216,(ArpMarin:0.05834,ArpMarit:5.3E-
4)1.000.113:0.1264)0.638.2:0.02392)0.155.3:0.01906,SrsSpeci:0.13322)0.881
.22:0.04593,UltB9071:0.0549)0.877.27:5.4E-
4)0.132.5:0.00968,UltOrg84:0.08664)0.885.15:0.01813,((UltB9072:0.04624,((
UltB9055:0.0,UltB9056:0.0,UltB9057:0.0):0.11048,UltB9083:0.03461)0.667.7:
0.02683)0.781.18:0.01917,(((UltB9050:0.0832,(UltB9085:0.05067,UltB9084:0.
06185)0.583.2:0.01218)0.809.17:0.0118,(LewNigri:0.01915,LewNigr2:0.0039)0
.889.18:0.01506)0.576.4:0.00885,(((UltB9073:0.05431,(SapBacte:0.03896,Ult
Sph35:0.0423)0.933.27:0.02538)0.752.11:0.00874,((UltB9036:0.05239,UltB906
7:0.05008)0.947.22:0.03901,UltB9054:0.04456)0.758.21:0.01589)0.905.17:0.0
1945,(((UltB9060:0.10777,UltB9082:0.01855)0.246.2:0.01608,(UltB9061:0.026
47,(UltB9058:0.04068,UltFla17:0.02841)0.900.19:0.02492)0.300.3:0.01003)0.
830.18:0.01662,(((UltB9081:0.10357,(UltB9077:0.02601,(UltB9080:0.02541,(U
ltB9078:5.3E-
4,(UltB9079:0.01581,(UltSapr5:0.02361,UltB9076:0.00752)0.898.27:0.01176)0
.792.20:0.01499)0.755.19:0.00606)0.968.14:0.03225)0.732.2:0.01254)0.750.1
0:0.03999,(UltB9053:0.05944,(UltB9051:0.02199,UltB9052:0.01402)0.973.13:0
.0492)0.899.20:0.03073)0.626.3:0.00948,UltB9049:0.08241)0.896.16:0.02584)
0.767.17:0.00651)0.753.14:0.0053)0.860.16:0.01418)0.745.15:0.0087)0.882.1
4:0.01429)0.501.3:5.3E-
4)0.897.14:0.01536,(UltB9068:0.06615,((UltSapro:5.4E-
4,(UltSapr2:0.0,UltB9070:0.0):0.03549)0.794.18:0.00919,((UltB1542:0.00486
,UltSapr3:0.02675)0.891.19:0.01999,UltB9069:0.04095)0.121.2:0.01555)0.951
.19:0.0416)0.808.11:0.01865)0.909.17:0.02599,((UdnCytop:0.28831,(UltB9037
:0.06034,UltB9038:0.0319)0.912.28:0.0571)0.904.15:0.04507,((UltB9063:0.01
283,UltB9065:0.05522)0.899.21:0.02941,(UltB9062:0.08738,(UltB9064:0.03278
,UltB9066:0.05196)0.818.10:0.01495)0.646.3:0.01358)0.943.23:0.04439)0.545
.6:0.00775)0.806.6:0.01039)0.982.8:0.04387,((UltB8805:0.04339,(UltB9039:0
.13099,(UltB9045:0.01024,UltB9044:0.00485)0.971.12:0.07997)0.358.5:0.0378
7)0.910.30:0.05007,((((UltB9030:0.01088,UltB9031:0.03685)0.893.26:0.02013
,(((((UltB8985:0.04563,((ChpNiabe:0.01463,((((ChpPine2:5.4E-
4,((ChpGinse:0.00753,(ChpJapon:0.01419,ChpSpeci:0.00989)0.874.25:0.01471)
0.961.15:0.02823,ChpSkerm:0.00728)0.783.16:0.01571)0.961.16:0.01526,ChpPi
nen:5.5E-
4)0.917.18:0.01099,((FlxSpeci:0.01733,ChpSanct:0.00541)0.444.3:0.00808,(C
hpArven:0.00418,(UltB9024:0.02308,ChpTerra:0.02235)0.329.6:0.00932)0.891.
20:0.01276)0.785.22:0.0061)0.777.20:0.00374,((((((UltB8934:0.01137,((UltB
8935:5.5E-4,(UltB8930:0.02293,UltB8936:5.4E-
4)0.827.16:0.00369)0.851.21:5.5E-4,((UltB8932:5.5E-
4,(UltB8931:0.0037,Ult15702:0.01118)0.380.2:5.4E-
4)0.891.21:0.00761,UltB8937:0.01541)0.852.21:0.0075)0.925.30:0.01151)0.74
9.25:0.00447,(UltB8973:0.02737,BacEnr25:0.00758)0.926.19:0.0151)0.346.4:0
.01102,(UltB8933:0.01151,UltOrg83:0.00744)0.906.27:0.01279)0.926.20:0.018
51,(((UltB8962:0.04943,(NstYeong:0.00789,UltB8963:0.00712)0.800.11:0.0064
2)0.707.3:0.00444,UltB8970:0.04482)0.620.5:0.00754,((((UltB9014:0.01219,(
(UltB8994:0.01015,UltB8993:0.01355)0.939.19:0.01958,(UltB8992:8.3E-
4,(LcbCauen:0.01219,ShcBacte:0.03379)0.097.3:0.0205)0.897.15:0.01908)0.68
4.4:0.00258)0.919.24:0.01512,(((UltB8969:0.05882,UltB8960:0.01174)0.895.2
4:0.01449,UltB8986:0.01655)0.943.24:0.01945,(((((UltB9007:0.02307,(UltB90
12:0.00357,UltB9013:0.00385)0.775.16:0.00414)0.865.21:0.0077,(UltB9000:0.
01223,ShcBact2:0.01933)0.884.22:0.01106)0.868.17:0.00804,UltB9001:0.00778

)0.792.21:0.00635,((UltB9009:5.4E-
4,UltB8998:0.02307)0.790.12:0.00784,(UltB8999:0.00748,UltB9015:0.00746)0.
764.19:0.00341)0.750.11:0.00463)0.848.19:0.00958,(UltB8951:0.04183,(UltB8
952:0.01395,(SegKoree:5.4E-
4,(UltrSo72:0.02346,UltrSo73:0.02359)0.842.21:0.00737)0.945.22:0.02313)0.
445.3:0.00984)0.933.28:0.01871)0.530.1:7.1E-
4)0.961.17:0.02138)0.756.22:0.00493,(UltB9011:0.01104,(UltB9003:0.01879,(
(UltB9008:0.01124,(UltB9016:0.01554,UltB9002:0.01945)0.744.12:0.00338)0.4
75.1:5.5E-
4,((UltB8997:0.00369,(UltB8996:0.00533,UltB8995:0.01423)0.937.20:0.0143)0
.887.19:0.00758,(UltB9010:0.01513,(UltB9006:0.00746,UltB9005:0.00746)0.79
2.22:0.00366)0.866.19:0.00765)0.936.21:0.01136)0.843.16:5.4E-
4)0.777.21:0.00746)0.878.23:0.00759)0.910.31:0.01181,(((UltB8976:0.03599,
((UltB8979:0.01038,(((UltB8977:0.00374,(UltB8981:0.02665,UltB8984:0.06653
)0.980.9:5.4E-
4)0.879.18:0.00746,UltB8982:0.00379)0.924.20:0.00972,((SdtSalmo:5.5E-
4,(UltB8978:0.01486,(UltB8974:0.01143,UltB8975:0.02006)0.391.5:0.00732)0.
901.27:5.4E-4)0.771.21:5.5E-
4,BacEnr26:0.01121)0.905.18:0.00471)0.546.4:0.00196)0.951.20:0.01897,UltB
8988:0.032)0.829.14:0.00788)0.407.1:0.00389,(UltrRap8:0.02046,((UltB9029:
5.3E-4,(UltSph33:5.4E-4,(UltB9028:0.00369,UltFla16:5.5E-
4)0.966.26:0.01132)0.892.19:0.0074)0.770.21:0.0049,(UltrSo78:0.0191,(UltB
9021:5.4E-4,UltB9022:0.01124)0.887.20:5.5E-
4)0.936.22:0.01814)0.752.12:0.00788)0.909.18:0.01408)0.750.12:0.00413,(((
(UltrSo76:0.01517,UltB9019:0.0037)0.846.15:5.4E-
4,UltrSo75:0.00369)0.894.17:0.00369,((UltB9020:0.01126,UltFlexi:0.06107)0
.997.20:5.5E-4,(UltrSo77:0.00314,UltB9023:0.01127)1.000.114:5.5E-
4)0.627.2:5.5E-
4)0.799.16:0.0037,(((((NstSpeci:0.0173,((UltB8987:0.03863,UltB8989:0.0255
4)0.865.22:0.01646,(UltB8971:0.02988,(UltB8991:0.02545,(UltB8968:0.00747,
(UltrSo74:0.02739,UltB8967:5.5E-
4)0.939.20:0.01531)0.943.25:0.01851)0.746.14:0.00377)0.730.8:0.0072)0.930
.15:0.01987)0.162.3:5.5E-
4,(BcsBact8:0.05075,UltB8983:0.04014)0.867.21:0.01632)0.907.25:0.01336,Ul
tFlavi:0.03165)0.762.15:0.00425,((UltB8953:0.03026,BifSpart:0.05908)0.730
.9:0.01655,FlmLacun:0.04996)0.871.18:0.01982)0.509.2:0.01518,((((UltB8966
:0.02281,UltB9025:0.00358)0.865.23:0.00778,(UltB8950:0.00724,UltB8965:0.0
2345)0.863.17:0.00769)0.952.25:0.01893,(UltB8954:0.01539,((UltB8964:0.012
29,BacEnr27:0.02621)0.708.4:0.00756,UltB8990:0.01411)0.867.22:0.00847)0.8
64.24:0.00823)0.120.2:0.00286,(UltB8972:0.02311,(UltB8957:0.00372,((UltB9
017:0.00749,UltB8955:0.01125)0.872.30:0.0075,(((UltB8956:0.00744,(FvsGins
e:0.00747,UltB8959:0.02264)1.000.115:5.5E-
4)0.808.12:0.00373,UltB8961:0.01916)0.762.16:0.0037,UltB8958:0.00746)0.74
1.10:0.00371)0.896.17:0.00754)0.759.19:0.00347)0.870.14:0.00854)0.835.18:
0.01181)0.734.6:0.00316)0.779.18:0.00365)0.857.26:0.00765)0.902.24:0.0140
3)0.908.18:0.0163)0.827.17:5.4E-
4,((UltB8940:0.004,(UltB8946:0.00372,((TrrLutea:5.5E-4,((UltB8941:5.5E-
4,UltB8938:0.015)0.836.14:0.00369,UltB8945:0.00371)0.062.2:5.5E-
4)0.865.24:0.00369,UltB8939:5.5E-
4)0.895.25:0.00744)0.849.16:0.00716)0.629.5:0.0075,(UltB8943:0.01132,((Ni
aAuran:0.01573,UltB8929:0.0161)0.842.22:0.00713,(TrrFerru:0.00745,((NiaSp
eci:5.3E-4,(NiaGinse:5.4E-
4,UltB8942:0.04803)0.958.22:0.02724)0.908.19:0.00743,NiabSoli:0.00742)0.1
68.3:5.4E-4)0.774.30:0.00374)0.900.20:0.00759)1.000.116:5.4E-

4)0.878.24:0.00401)0.900.21:0.01582,UltB8944:0.01446)0.909.19:0.01242)0.935.22:0.01644)0.107.6:5.5E-4,UltB9027:0.07814)0.790.13:0.01632)0.700.8:0.0011,(UltB8949:0.05124,UltB8980:0.01025)0.900.22:0.02224)0.917.19:0.01332,(UltB9026:0.07914,(UltrSo79:0.0352,(UltB9035:0.03979,UltB9034:0.02109)0.889.19:0.02238)0.731.5:0.00675)0.810.13:0.00774)0.893.27:0.02344,(UltB9040:0.01954,UltB9046:0.05474)0.984.9:0.04657)0.933.29:0.02886,UltB9033:0.02026)0.744.13:0.01008)0.666.3:0.01024,(UltB9032:0.03455,UltChiti:0.04164)0.837.12:0.01673)0.883.26:0.01591,((UltB9042:0.07558,BacteIT4:0.17205)0.901.28:0.05553,(BacEnr29:5.5E-4,(UltB8948:5.5E-4,BacEnr28:5.5E-4)0.882.15:5.3E-4)0.956.18:0.04573)0.798.17:0.02078)0.886.24:0.03119)0.882.16:0.02559)0.589.6:0.00473,((UltB8715:0.02605,UltB8894:0.08034)0.863.18:0.01973,(((UltB8414:0.02678,(UltB8415:0.02961,UltB8416:0.05765)0.105.1:0.00973)0.921.35:0.02102,(LctBacte:0.0664,(UltB8413:0.0316,BcsBacte:5.5E-4)0.932.22:0.01946)0.802.14:0.0097)0.993.13:0.04113,((((((MaiMet12:0.03559,UltB8905:0.03771)0.906.28:0.01893,((UltB8904:0.03613,UltMar46:0.06303)0.949.21:0.02746,(UltB8891:0.05948,((UltFla15:0.02296,(UltB8889:5.5E-4,UltMar45:0.00741)0.738.7:0.00411)0.965.23:0.03437,(UltB8890:0.02753,CcnVermi:0.03792)0.889.20:0.02001)0.906.29:0.02799)0.922.22:0.02522)0.083.2:5.5E-4)0.855.13:0.01345,(UltB8893:0.06623,(OweHongk:0.02474,UltCyto7:0.0401)0.312.3:0.00294)0.903.17:5.4E-4)0.989.16:0.03028,(UltB8896:0.04414,(UltB8895:0.01172,(UltB8722:0.00347,UltB8897:0.0402)0.950.19:0.01925)0.746.15:0.00713)0.863.19:0.00822)0.880.26:0.00929,((UltB8417:0.088,UltB8593:0.04096)0.850.21:0.01195,(UltB8744:0.03127,UltB8926:0.08302)0.903.18:0.02006)0.895.26:0.01612)0.965.24:0.02649,(((UltB8886:0.29982,UltB8503:0.08658)0.462.4:5.5E-4,((UltB8923:0.08836,(UltB8438:0.08107,UltB9086:0.06556)0.968.15:0.06828)0.789.12:0.05279,(((CrbAtlan:0.09962,UltB8836:0.1065)0.858.17:0.02908,UltOrg89:0.20966)0.738.8:0.00496,((CoeAnati:0.04217,((((CapHaem2:0.00369,CapHaemo:0.00371)0.991.22:0.04098,((UltB8712:0.00849,(CapOchra:0.01199,((CapOchr2:5.5E-4,(CapOchr3:0.0037,(UltCapno:5.5E-4,UltCapn2:0.00371)0.050.3:5.4E-4)0.957.18:0.00746)0.587.4:5.4E-4,UltB8710:0.05136)0.933.30:0.01132)0.491.3:0.00278)0.734.7:0.00598,(UltB8709:0.00369,CapSputi:5.5E-4)0.907.26:0.01822)0.947.23:0.02423)0.874.26:0.01947,UltB8711:0.02676)0.712.4:0.00533,(CapCanim:5.5E-4,CapCynod:0.00369)0.908.20:5.4E-4)0.903.19:0.02968)0.979.17:0.05584,(((((AeqCapso:5.4E-4,AeqSubli:0.04413)0.104.3:0.01418,(AeqAntar:0.00381,((UltB8787:0.04754,((BacEnr22:5.5E-4,BacEnr24:0.00368)0.987.19:5.4E-4,(VtlVladi:5.5E-4,CfChryse:0.10015)0.977.15:0.01491)0.921.36:0.02922)0.907.27:0.01955,UltAequo:0.0037)0.870.15:0.00751)0.915.20:0.01771)0.858.18:0.01811,(UltB8788:0.03769,AmnPelag:0.06263)0.878.25:0.02567)0.861.24:0.02402,((((((((SthSpong:0.04322,(((NnlTeget:0.0,SnkSedim:0.0):0.0115,UltB8804:0.0116)0.862.22:0.0086,(((FlbBac13:0.00369,FlbBac11:0.01531)0.806.7:0.00368,(PcvDokdo:0.00362,PcvXylan:5.4E-4)0.866.20:0.00746)0.596.3:5.3E-4,FlbBac12:5.4E-4)0.825.13:0.00682)0.675.4:0.00474)0.982.9:0.03188,FlbBact9:0.00386)0.213.1:0.01528,(KroGenik:0.0,KroDiaph:0.0):0.00389)0.993.14:0.0435,(((UltB8773:0.01871,(SufMarit:5.5E-4,UltOrg82:0.03056)0.982.10:0.03297)0.210.3:0.01676,(((((GrmPorti:0.0,MaiBac15:0.0):5.3E-4,(GrmMarin:0.00379,GrmEchin:0.00366)0.407.2:0.00371)0.997.21:0.03101,(SgnFlavu:0.01216,((((PsfSedim:0.0,PsfSpeci:0.0):5.5E-4,PsfTorqu:0.01894)0.855.14:0.00369,UltB8799:5.5E-4)0.916.24:5.4E-

4,PsfTropi:0.00722)0.943.26:0.0188)0.805.16:0.00755)0.864.25:0.00663,(Msn
Algae:0.02288,(UltB8796:0.03014,(MsnMobil:0.00763,(SgnSaleg:0.00372,(SgnH
olot:0.00369,(SgnSalin:0.0,SgnMishu:0.0):5.5E-4)0.325.3:5.5E-
4)0.776.17:0.00364)0.948.26:0.02405)0.892.20:0.01574)0.371.5:5.4E-
4)0.953.18:0.02025,(SncSpeci:0.00774,((SncTerra:5.4E-
4,SncXinji:0.00725)0.976.13:0.01978,((SncCaten:0.01509,((UltrSo71:0.01544
,((UltB8802:0.01057,(UltB8801:0.05801,((SbgOutfl:5.4E-4,(GlsMyxil:5.4E-
4,GlsSanda:0.0351)0.811.13:0.00369)1.000.117:5.5E-
4,(GlsLimna:0.0,GlsHiemi:0.0,GlsMitsk:0.0):0.00734)0.961.18:0.02732)0.435
.2:0.01136)0.211.3:0.02026,FlaSpe28:5.4E-
4)0.962.26:0.01525)0.847.22:0.00742,UltB8800:5.5E-
4)0.710.4:0.00751)0.668.8:5.4E-
4,FlbBac10:0.00744)0.873.25:0.00751)0.757.12:0.00366)0.875.20:0.01168)0.8
59.20:0.01042)0.825.14:0.01073,(LpbFlave:0.01323,(GlrMesop:0.11332,((LwnA
equo:0.01657,(LwnMarin:0.00769,(CytSpe10:0.00726,(LwnSpeci:5.5E-
4,LwnBland:5.5E-4)0.939.21:5.5E-
4)0.345.4:0.00741)0.795.20:0.00609)0.875.21:0.01711,((UltB8708:0.00767,(U
ltB8771:5.4E-
4,UltB8772:0.00742)0.826.13:0.00736)0.988.22:0.05365,(((Bfhggg34:0.04201,
(UltCapn3:0.03522,(UltB8713:0.02429,(CapGranu:0.00693,(CapGing2:0.0037,Ca
pGingi:5.5E-4)0.977.16:5.3E-
4)0.438.3:0.02028)0.941.18:0.03972)0.993.15:0.05902)0.752.13:0.01636,(Ltn
Therm:0.02847,FlbBact3:0.05103)0.901.29:0.03665)0.926.21:0.02962,(((((Ult
Flav5:0.03812,(UltB8675:0.03243,((FlaSpe24:5.5E-
4,FlaSpe23:0.00368)0.783.17:0.00388,(UltB8676:0.01602,UltB8688:0.02792)0.
699.10:0.00272)0.708.5:0.00322)0.876.22:0.01878)0.847.23:0.00877,(((UltB8
655:5.5E-
4,UltB8679:0.03092)0.828.13:0.0098,(((FlcBacte:0.01505,UltOrg81:5.4E-
4)0.752.14:0.00352,(BacteN61:0.01777,(UltB8698:0.04554,(UltB8695:5.4E-
4,((UltB8642:0.00745,((UltB8693:0.0,UltB8696:0.0):5.5E-
4,UltB8694:0.00369)0.935.23:5.5E-
4)0.958.23:0.01556,UltB8697:0.00374)0.848.20:0.00741)0.087.3:0.00862)0.88
7.21:0.02522)0.904.16:0.0184)0.872.31:0.00775,(UltB8678:0.00369,(FlaKamog
:5.4E-4,((FlaGelid:0.01938,((((FlaWeave:5.4E-
4,FlaSeget:0.00723)0.927.21:0.01113,(FlaFleve:0.01508,(UltB8635:0.0,FlaSp
e22:0.0):5.5E-4)0.817.9:0.0075)0.761.21:0.00373,((FlaAntar:5.4E-
4,(UltB8621:0.01121,(FlaSpec5:5.5E-
4,(AntBac30:0.00748,FlaTeget:0.00745)0.848.21:5.4E-4)0.932.23:5.3E-
4)0.834.11:0.00367)0.981.11:0.02381,(UltB8660:0.03223,((UltB8687:0.01019,
(UltB8657:0.03098,UltB8677:0.00432)0.616.2:0.0099)0.509.3:0.00644,(FlbBac
t2:5.4E-
4,((UltB8661:0.01403,FlaLinda:0.02477)0.954.20:0.02021,((UltB8659:5.4E-
4,UltB8662:0.03543)0.866.21:0.00747,UltB8658:0.00752)0.762.17:0.00528)0.9
42.19:0.01698)0.787.21:0.0038)0.890.25:0.01181)0.812.9:0.00663)0.805.17:0
.00762)0.763.24:0.00246,(UltB8663:0.00376,UltFlavo:0.01127)0.742.12:0.010
13)0.856.11:0.0104)0.867.23:0.00754,UltFlav2:0.00387)0.772.20:0.00356)0.8
93.28:0.00744)0.873.26:0.00768)0.808.13:0.00919)0.774.31:0.01786,((UltB86
91:0.03529,((MrdOdora:0.0,UltB8690:0.0):0.03654,(UltB8689:0.03175,MrdPela
g:0.0184)0.893.29:0.0225)0.886.25:0.02872)0.549.2:0.02703,UltB8692:0.0304
1)0.880.27:0.0174)0.926.22:0.02075)0.187.3:0.00235,((UltB8686:0.02832,Fla
Indic:5.5E-
4)0.942.20:0.01147,(UltB8685:0.03507,(((AntBac36:0.00372,((((UltB8643:5.3
E-
4,((UltB8667:0.01184,(UltB8666:0.00436,UltB8668:0.10208)0.937.21:0.0227)0

.963.18:0.01967,UltFlav3:0.00387)0.867.24:0.00738)0.733.9:0.00663,UltFlav
4:0.13401)0.857.27:0.02154,(((UltB8620:5.4E-
4,((FlaDenit:0.00438,(UltB8622:0.00741,FlavSoli:5.5E-
4)0.933.31:0.01867)0.971.13:0.0276,(((FlaAnhu2:0.0,FlaAnhui:0.0):0.01575,
((UltB8641:5.4E-
4,MldSoil0:0.01474)0.222.2:0.01131,(Ult29494:0.00759,((((AntBac33:0.01934
,UltB8640:0.05209)0.823.11:0.01297,((((RaiTrou2:0.0,FlaSacch:0.0,UltB8651
:0.0,UltB8653:0.0,UltB8656:0.0):5.4E-4,(((UltB8664:5.5E-4,UltB8665:5.5E-
4)0.984.10:0.01878,(FlaHydat:5.5E-
4,UltB8654:0.01125)0.963.19:0.00213)0.961.19:0.00169,(FlaPsyc3:0.03519,Ul
tB8767:0.03912)0.815.12:0.00849)1.000.118:5.5E-
4)0.842.23:0.00371,((FlaSpe14:0.01128,UltB8639:0.00374)0.882.17:0.00758,U
ltB8638:0.02303)0.764.20:0.0036)0.792.23:5.5E-4,(FlaSpe13:5.5E-
4,FlaHiber:5.5E-4)0.337.2:5.4E-4)0.971.14:5.4E-
4)0.951.21:0.00747,(((UltB8632:0.0,UltB8652:0.0,FlaSucc2:0.0):5.5E-
4,(FlbBacte:0.00745,FlaSucci:0.00369)0.712.5:5.5E-4)0.906.30:5.4E-
4,(((((ArctiSe4:0.0077,((FlaPsyc2:0.00804,AntBac34:0.01882)0.871.19:0.011
75,(((FlaSpe21:0.00375,(FlaFryxe:0.0037,((AntBac31:0.0,FlaDege2:0.0,FlaDe
ger:0.0,FlaFrigo:0.0,FlaDege3:0.0):5.5E-
4,(FlaFrigi:0.00369,FlaXanth:0.01497)0.729.7:5.5E-4)0.677.5:5.3E-
4)0.940.15:5.5E-
4)0.916.25:0.00667,(UltB8644:0.01499,(UltB8624:0.00744,(FlaSpe12:0.0,FlaP
sych:0.0,UltB8645:0.0,FlaSpe18:0.0,UltB8649:0.0):5.5E-4)0.577.1:5.4E-
4)0.905.19:0.00208)0.903.20:0.00211,(FlaLimic:5.5E-
4,(FlaSpe19:0.00372,UltB8650:0.00369)0.745.16:5.5E-4)0.914.18:5.5E-
4)0.787.22:0.00398)0.907.28:0.01139)0.922.23:0.0115,FlaArcti:0.00369)0.98
8.23:5.4E-
4,(((UltB8631:0.00369,(((UltB8629:0.00369,(((FlaDaeje:0.01131,FlaSpe10:0.
00741)0.084.2:5.5E-4,(UltB8630:0.0,FlaSpe11:0.0):5.5E-
4)0.820.9:0.00369,FlaHercy:0.00742)0.312.4:5.5E-4)1.000.119:5.5E-
4,((UltB8623:0.0,SrtSpeci:0.0,FlaJohn2:0.0,FlaGlaci:0.0,FlaGranu:0.0):0.0
0363,(FlaResis:0.0,FlaSpec9:0.0,FlaSpe17:0.0):5.4E-
4)0.121.3:0.00371)0.845.22:0.00363,UltB8637:5.4E-
4)0.856.12:0.00371)0.752.15:5.5E-
4,(UltB8625:0.0,UltB8627:0.0,FlaSpe16:0.0):5.5E-4)0.000.229:5.5E-
4,(UltB8628:0.00369,UltB8626:0.00369)0.711.7:5.5E-4)0.762.18:5.5E-
4)0.788.15:5.4E-
4,UltB8646:0.00368)0.910.32:0.00209,(AntBac32:0.00212,((FlaMicro:0.0,UltB
8636:0.0):5.4E-
4,FlaSpe20:0.00371)0.917.20:0.0021)0.932.24:0.0064)0.911.28:0.00211)0.380
.3:5.5E-
4)0.948.27:0.00492,((FlaSpec8:0.0,UltB8647:0.0,UltB8648:0.0):5.4E-
4,(FlaPecti:0.0,FlaSpe15:0.0):0.0037)0.948.28:0.00499)0.948.29:0.00486)0.
850.22:0.0078)0.851.22:0.0079)0.758.22:0.00326,(FlaSpec7:0.0037,FlaSpec6:
0.00368)1.000.120:5.4E-
4)0.464.3:0.00823)0.812.10:0.00676)0.747.16:0.00359,((FlaJohns:0.0,OrzSat
i3:0.0):0.0075,FlaDeflu:5.4E-
4)0.873.27:0.0077)0.923.22:0.01219,FlaSunch:0.02414)0.700.9:0.00286)0.965
.25:0.01805,((UltB8684:0.02219,(FlaCheni:5.5E-
4,(FlaSasan:0.0074,(UltB8682:0.0,UltB8683:0.0):5.5E-
4)0.990.19:0.02269)0.514.2:0.01157)0.828.14:0.00196,FlaFilum:0.02937)0.66
9.5:0.00407)0.906.31:0.01302)0.941.19:5.5E-
4,((FlaSalip:0.0,FlaSpe25:0.0):5.5E-
4,UltB8680:0.0074)0.999.28:0.02659)0.964.25:0.02222,(FlaCroce:0.02695,(((

((UltB8669:0.01251,UltB8670:0.01118)0.877.28:0.00827,(UltB8671:5.5E-
4,(Bact3A13:0.03041,FlaAquat:0.00405)0.903.21:0.00382)0.875.22:0.00761)0.
881.23:0.01081,((UltB8673:0.0,UltB8674:0.0):0.01186,(((FlaColum:5.5E-
4,((FlaColu2:0.00202,FlxAuran:0.01506)0.950.20:0.00192,(FlaColu3:0.0,FlaS
pe26:0.0):0.00369)0.892.21:5.5E-4)0.850.23:5.3E-4,UltB8681:5.5E-
4)0.959.27:0.01856,FlaTerra:0.00789)0.230.5:0.00394)0.885.16:0.00983)0.80
8.14:0.00668,AntBac35:0.00369)0.487.5:5.4E-4,UltB8672:5.5E-
4)0.609.7:5.4E-4)0.870.16:5.3E-4)0.888.29:0.00679)0.260.3:5.4E-
4)0.902.25:0.01161)0.920.21:0.01939,(UltB8701:0.01793,(UltB8699:0.00369,U
ltB8700:5.5E-
4)0.991.23:0.04983)0.974.9:0.03964)0.906.32:0.01747,(UltB8706:0.01139,(Ul
tB8703:0.01172,((UltB8705:0.0,UltB8704:0.0):0.0153,(UltB8707:5.3E-
4,(UltB8633:5.4E-4,UltB8634:0.00743)0.848.22:0.00369)0.951.22:5.4E-
4)0.908.21:0.01124)0.733.10:0.00353)0.906.33:0.02132)0.243.2:0.01659)0.78
6.17:0.00853)0.945.23:0.02584)0.612.4:0.00945)0.851.23:0.01355)0.693.4:0.
00388)0.354.3:0.0112)0.855.15:0.01472,((((((((((((((WinSpec4:5.5E-
4,UltSubsa:0.01901)0.872.32:0.0083,(SdbFurfu:0.01398,UltB8729:0.0088)0.96
5.26:0.01912)0.785.23:0.00718,UdnBac24:0.03083)0.987.20:5.5E-
4,((GlsIllus:5.5E-4,(GelMesop:5.5E-
4,(UltB8732:0.00745,(((YeoAroma:0.0,SbsBroad:0.0):5.5E-
4,UdnBac25:0.00369)1.000.121:5.5E-
4,BacEnr23:0.01912)0.821.14:0.00369)0.841.17:5.5E-4)0.737.17:5.4E-
4)0.865.25:5.3E-
4,(GelSalic:0.01924,(GelGilvu:0.01134,GelAlgen:0.01127)0.947.24:0.01535)0
.748.18:0.00365)0.913.18:0.00741)0.811.14:5.4E-
4,(TamCroci:0.01536,(UltB8721:0.0,UltB8726:0.0):0.01131)0.858.19:0.00752)
0.783.18:0.00564,((WinPorif:0.00885,CccMarin:0.01405)0.433.5:0.01398,(((G
aeSpeci:0.00366,UltB8717:5.5E-
4)0.996.13:0.04546,UltB8728:0.0101)0.432.3:0.01514,FlsCheju:0.00601)0.770
.22:0.00953)0.963.20:0.02925)0.777.22:0.00625,MrfGromo:0.01986)0.762.19:0
.00299,(((CfCytoph:0.0,WinEchin:0.0):5.4E-
4,(GaeSaema:0.00743,UltB8727:5.3E-4)0.910.33:0.00744)0.799.17:5.4E-
4,(((BcsBact2:5.5E-4,((WinThala:0.0,WinAreno:0.0):5.5E-
4,(WinSpec3:0.0037,WinSpeci:0.0037)0.734.8:5.5E-4)0.728.7:5.5E-
4)0.788.16:5.5E-4,(PcpSpec3:0.00739,(AgtLectu:5.5E-4,(AgtMikha:5.4E-
4,(TamAgari:5.3E-4,(CrrBacte:5.5E-
4,AgtSpeci:0.0151)0.877.29:0.00739)0.964.26:0.01122)0.927.22:0.0111)0.984
.11:0.0189)0.017.2:5.3E-
4)0.910.34:0.00735,((LcnCopep:0.01478,WinSpec2:0.02262)0.874.27:5.3E-
4,(WinEximi:5.4E-4,WinEpiph:0.01123)0.828.15:0.00367)0.211.4:5.4E-
4)0.971.15:0.01113)0.901.30:0.00753)0.877.30:0.00755,FlbBact4:0.00745)0.8
90.26:0.00742,MaiBac12:5.5E-
4)0.879.19:0.00743,(((((BizSalef:0.00745,((FlaSpe27:0.0,UltB8725:0.0):5.5
E-4,OllMari2:0.00372)0.861.25:0.00371)0.051.1:5.5E-4,(PcpMeso2:5.5E-
4,(UltFlav7:0.01932,((((GaeMarin:5.4E-
4,(FlrBasal:0.00371,(MaiBac10:0.00747,PcpSpeci:5.5E-
4)0.802.15:0.00369)0.928.21:0.01122)0.888.30:0.00741,FlrBasa2:0.01121)0.8
47.24:5.4E-4,BizSpeci:5.5E-4)0.894.18:5.5E-4,((BizMyxar:5.4E-
4,BizArgen:0.01519)0.814.11:0.00369,BizAlgor:5.5E-
4)0.899.22:0.00742)0.423.1:5.5E-
4)0.845.23:0.00369)0.920.22:0.00745)0.902.26:5.5E-
4,(OllMaril:0.0,PcpMesop:0.0):5.5E-4)0.895.27:5.5E-4,(UltB8718:5.3E-
4,(((FrmAgari:0.0075,(UltFlav9:0.00369,(UltB8730:0.00369,FrmAlgae:0.00372
)0.103.2:5.5E-4)0.821.15:5.4E-4)0.924.21:0.00747,MaiMet10:5.5E-

4)0.908.22:0.00743,UltFlav8:0.01521)0.480.2:5.4E-
4)0.904.17:0.00369)0.940.16:5.4E-
4,((((UltB8731:0.00368,((PcpSpec2:0.0,LcnCope2:0.0):5.5E-
4,LcnMarin:0.00369)0.396.5:5.5E-4)0.801.16:5.3E-
4,(MaiPsych:0.11259,(PcpBurto:0.03061,SubWynnw:0.05478)0.382.2:0.00996)0.
871.20:0.01735)0.852.22:5.5E-4,GelSpeci:0.01103)0.010.2:5.4E-
4,BizParag:0.01096)0.845.24:0.00689)0.883.27:0.00755)0.755.20:0.00362,(Ms
fZeaxa:0.00739,(UltFlav6:0.0189,MaiBac11:5.5E-4)0.894.19:5.5E-
4)0.288.2:0.00383)0.890.27:0.01099,((UltB8734:0.00366,UltB8769:5.5E-
4)0.961.20:0.02247,(UltCyto4:0.00389,(UltB8724:0.01503,UltB8723:5.3E-
4)0.401.2:0.00739)0.862.23:0.0097)0.863.20:0.01014)0.735.9:0.00362,BizGel
id:0.0532)0.947.25:0.02194,((((MaiMet11:0.01459,UltB8761:0.00156)0.888.31
:0.01014,(UltB8760:0.01861,UltB8763:0.01034)0.786.18:0.00624)0.754.17:0.0
0479,(UltB8762:0.02086,((UltB8765:0.02024,UltB8764:0.01473)0.275.5:0.0086
9,UltB8766:0.05145)0.909.20:0.01642)0.462.5:0.00296)0.863.21:0.01334,(Ult
Fla10:0.02585,(UltB8768:0.01748,UltB8770:0.02311)0.806.8:0.00788)0.941.20
:0.02016)0.929.20:0.01979)0.754.18:0.00466,(UltB8776:0.0199,((((((XncMari
s:0.00612,UltB8756:0.01263)0.852.23:0.01511,FlbBact6:0.04842)0.888.32:0.0
1285,(UltB8755:0.03968,(CrcLitor:0.00858,(LobstGu2:0.0231,LtcSpeci:0.0076
9)0.657.3:0.00277)0.843.17:0.01195)0.733.11:0.0037)0.902.27:0.00793,UltB8
759:0.02702)1.000.122:5.5E-
4,(((UltB8754:0.01007,UltCyto6:0.0101)0.898.28:0.01014,(((UltB8749:0.0040
2,((((UltB8757:0.03111,(AbcSedim:0.00375,StmClon2:0.01521)0.913.19:0.0113
5)0.980.10:5.4E-4,(AtlSaema:0.0,LtsVermi:0.0):5.5E-
4)0.919.25:0.00369,FlbBact7:5.5E-4)0.500.3:5.5E-
4,UltB8751:0.01136)0.871.21:0.00709)0.609.8:0.00743,(UltCyto5:0.01129,Ult
B8752:5.4E-4)0.916.26:0.00337)0.964.27:5.5E-
4,(UltB8750:0.00752,UltB8753:0.00746)0.883.28:0.0075)0.822.10:5.4E-
4)0.910.35:0.0151,(TenMarin:0.03205,(UltB8741:0.02661,(((((UltB8735:0.0,U
ltB8736:0.0):0.01169,UltB8737:0.0399)0.877.31:0.011,UltB8740:0.00746)0.74
2.13:0.00373,UltB8738:5.4E-
4)0.962.27:0.01878,((UltB8742:0.0,TenCrass:0.0):0.00749,((TenSkage:5.3E-
4,((TenAmylo:0.0,CytSpec4:0.0):0.00342,(BacCWISO:0.00365,TenLitop:0.00375
)0.960.24:0.0154)0.962.28:0.01542)0.897.16:0.00742,((((TenSolea:5.5E-
4,(TenSpec3:0.0114,(TenOvoly:0.0,TenOvol2:0.0):0.01139)0.803.10:0.00366)0
.361.4:5.4E-4,((((PoaSpec3:0.0152,(((UltB8746:5.4E-
4,((PoaIrgen:0.00369,(PoaButke:0.00369,UltB8745:5.5E-4)0.157.1:5.5E-
4)0.802.16:0.00338,(PoaFranz:0.00717,(PoaIrge2:5.5E-
4,FlbBact5:0.00369)0.904.18:5.5E-
4)0.323.3:0.00374)0.821.16:0.00404)0.903.22:0.00744,PoaDokdo:0.00746)0.88
7.22:5.5E-4,(PoaDokd2:0.0,UltB8747:0.0):5.5E-4)0.875.23:5.5E-
4)0.816.15:0.00369,((((PoaFilam:0.0075,PoaSpec2:0.00747)0.955.18:0.0149,(
UltB8739:0.02273,UltPolar:0.004)0.593.2:0.00375)0.888.33:0.00776,MaiBac13
:0.00369)0.930.16:5.4E-4,PoaSpeci:5.5E-4)0.842.24:5.4E-
4)0.931.22:0.01126,(FlxAura2:5.4E-
4,UltB8743:0.00744)0.755.21:0.00376)0.870.17:0.00759,TenMarit:0.01518)0.8
63.22:0.00756)0.814.12:0.00368,TenSpec2:5.5E-4)0.482.4:5.4E-
4,(TenAdria:0.0,TenMesop:0.0,TenSpeci:0.0,TenDisco:0.0,TenGalla:0.0,TenAi
pta:0.0,TenLutim:0.0):5.5E-4)0.739.18:5.3E-
4)0.869.20:0.00371)0.912.29:5.4E-
4)0.854.14:0.00778)0.991.24:0.03219)0.809.18:0.0077)0.846.16:0.01914)0.75
2.16:0.00168,UltB8748:0.02878)1.000.123:0.05022)0.939.22:0.02475)0.925.31
:0.02143)0.734.9:0.00608,((ClpLytic:0.00383,(ClpSpec4:5.5E-
4,ClpTyros:0.04331)0.945.24:0.01537)0.761.22:0.00351,ClpSpec5:0.00769)0.8

87.23:0.01106)0.865.26:0.00915,(((ZhoAmylo:0.01667,(JooMarin:5.5E-
4,UltB8714:0.00369)0.991.25:0.0264)0.844.23:0.01128,((GilSedim:0.01632,((
UlvLitor:0.01232,((BcsBact6:0.0144,(UltB8733:0.04884,UlvAntar:0.01824)0.4
87.6:0.00946)0.878.26:0.01266,UltB8791:0.02036)0.747.17:0.00771)0.849.17:
0.0103,(UltB8775:0.01923,MxnOphiu:0.03166)0.757.13:0.00327)0.971.16:0.020
38)0.788.17:0.00658,(UltFla13:0.00369,UltB8792:5.5E-
4)0.920.23:0.01177)0.850.24:0.00858)0.849.18:0.00939,(UltMar44:0.02074,(F
ltTotto:0.01786,(KrdAlgic:5.4E-4,MaiBac14:5.5E-
4)0.975.18:0.03584)0.961.21:0.03524)0.418.4:0.0071)0.349.3:5.3E-
4)0.792.24:0.01838,((((AmrBrevi:0.0147,((FlbBact8:0.00572,(AmrLater:0.026
9,FlxTract:0.01141)0.174.2:0.00769)0.711.8:0.00822,((AmrMuell:5.5E-
4,AmrSpeci:0.0113)0.895.28:5.4E-
4,(GelSpec2:0.06423,UltB9018:0.11608)0.859.21:0.02506)0.850.25:0.01376)0.
772.21:0.00892)0.989.17:0.04481,AmrSpec2:0.00444)0.877.32:0.01485,(UltB87
93:0.02336,(UltFla11:0.03529,(UltB8798:0.08336,(UltB8795:5.5E-
4,UltB8794:0.00369)0.965.27:0.03135)0.569.2:0.01059)0.962.29:0.02327)0.72
8.8:0.00381)0.906.34:0.01135,UltB8716:0.0115)0.795.21:5.5E-
4)0.887.24:0.02051)0.917.21:0.02186,(((((FlcBact2:0.00369,(((MbcAntar:5.4
E-4,(BcsBact3:0.00382,(UltB8779:0.00756,BcsBact4:5.3E-
4)0.774.32:0.00362)0.959.28:0.01142)0.902.28:0.01121,(((CytSpec5:0.0,MbcS
edim:0.0):5.4E-
4,UltB8778:0.00369)0.944.17:0.01141,(PibPonti:0.00369,(ZblGalac:5.5E-
4,(ClpSpeci:0.0281,(MbcUlvic:0.0,MbcAquiv:0.0,MbcOrien:0.0,MbcForse:0.0):
0.00301)1.000.124:7.0E-
4)0.786.19:0.00372)0.752.17:0.00375)0.921.37:0.01579)0.961.22:0.01989,(Mb
cPolys:5.5E-
4,(((ArtSpec2:0.022,(((UltB8786:0.0,ArtPalla:0.0,ArtTroit:0.0):5.3E-
4,ArtLater:0.01502)0.830.19:0.01129,ArtSpeci:0.01136)0.391.6:0.01242)0.43
4.2:0.00763,SdcLuteu:0.0143)0.879.20:0.00868,UltB8789:0.00744)0.929.21:0.
01126)0.227.4:5.3E-4)0.923.23:0.0074)0.130.1:5.4E-
4,UltB8777:0.00747)0.927.23:0.01466,((KriAquim:0.01549,UltB8780:0.01105)0
.847.25:0.00781,((ClpSpec2:0.0073,ClpPacif:5.5E-
4)0.966.27:0.02606,((UltB8784:0.01119,(EudAdria:5.4E-
4,BactAMSU:0.02284)0.754.19:0.00383)0.740.14:0.0043,(ZxnEnosh:0.00339,Ult
B8785:0.02751)0.916.27:0.01524)0.994.21:0.04363)0.849.19:0.01085)0.904.19
:0.01211)0.738.9:0.00366,(((((MucBact5:0.04665,((((MurFlave:0.01529,((Bac
tA16s:0.00369,MurRuest:5.5E-4)0.934.19:0.0074,(CytSpec7:5.5E-
4,BcsBact5:5.5E-4)0.595.1:5.5E-4)0.426.5:5.5E-
4)0.923.24:0.01155,(MurOlear:0.01917,(CrtDokdo:0.02793,CrtEcklo:0.01528)0
.986.14:0.03625)0.032.1:5.5E-4)0.991.26:0.03222,(MurSpeci:5.4E-
4,UltB8782:0.01899)0.348.4:0.00364)0.743.19:0.00379,UltB8781:5.4E-
4)0.947.26:0.02889)0.936.23:0.02514,PfrNumaz:0.04816)0.915.21:0.02293,Cyt
Spec6:0.0331)0.745.17:0.00772,((FlgEcklo:0.06896,UltB8803:0.09592)0.426.6
:0.01015,(RbnBifor:0.01359,(UltB8783:0.01084,RbnMyxol:0.02382)0.841.18:0.
00969)0.964.28:0.02486)0.882.18:0.01875)0.672.3:0.01401,UltFla12:0.03964)
0.852.24:0.00999)0.947.27:5.3E-4,(CosAggre:0.0539,(PszTherm:5.4E-
4,(CytSpec8:0.01113,CytSpec9:5.4E-
4)0.992.18:0.02275)0.885.17:0.01513)0.894.20:0.03243)0.972.17:0.02756)0.9
65.28:0.0483)0.875.24:0.02343)0.770.23:0.02557)0.836.15:0.02797)0.882.19:
0.01803,((CrnCatal:0.0358,UltB8922:0.0651)0.985.22:0.06351,(((UltB8924:0.
02188,((BrbGlaci:0.00369,BrbSpeci:5.5E-
4)0.965.29:0.03574,(UltB8916:0.01182,(UltB8915:0.01491,CrpBacte:0.03084)0
.728.9:0.00487)0.893.30:0.01946)0.584.4:0.0201)0.724.8:0.00491,((UltB8918
:5.4E-

4,((UltB8914:0.02245,((UltB8920:0.00364,UltB8921:0.00752)0.877.33:0.00854
,(UltB8919:0.03572,((UltB8912:5.4E-
4,UltB8913:0.00724)0.422.2:0.00676,((UltB8908:0.00744,UltB8911:0.0075)0.9
20.24:5.4E-4,(UltB8909:5.3E-
4,UltB8910:0.03117)0.512.1:0.00714)0.932.25:0.0157)0.868.18:0.00824)0.926
.23:0.01895)0.942.21:0.01971)0.905.20:0.01235,(UltB8917:0.01127,FllTaffe:
5.4E-
4)0.936.24:0.01537)0.540.4:0.01529)0.964.29:0.03176,((LshCasei:0.01522,Ma
iBac16:5.4E-4)0.999.29:0.03492,UltB8925:5.5E-
4)0.734.10:0.00322)0.844.24:0.00902)0.918.22:0.02529,(UltCyto8:0.05368,(U
ltB8618:0.0394,(UltB8615:0.00573,UltB8616:0.0132)0.824.22:0.02617)0.969.1
9:0.04984)0.826.14:0.03377)0.767.18:0.0171)0.824.23:0.01916)0.949.22:0.03
31)0.705.6:0.00383)0.841.19:0.01353)0.837.13:0.01207)0.898.29:0.01787)0.9
54.21:0.02281,(((UltB8508:0.05383,((UltB8505:0.03626,UltB8506:0.05998)0.9
87.21:0.06701,((UltB8507:0.07265,(UltCytop:0.08373,UltB8504:0.05956)0.876
.23:0.03028)0.665.6:0.01329,(UltB8590:0.08768,(UltB8296:0.01986,UltB8297:
0.01897)0.942.22:0.04193)0.641.6:0.0201)0.689.6:0.02631)0.767.19:0.01502)
0.898.30:0.03487,(UltB9041:0.09454,UltB9043:0.02946)0.790.14:0.01121)0.94
9.23:0.02515,((((((((UltB8556:0.02171,(UltB8560:0.08663,UltB8559:0.03623
)0.737.18:0.01734)0.843.18:0.00874,((UltB1967:0.00368,(UltB8557:0.0,UltB8
576:0.0):5.5E-
4)0.997.22:0.04385,(UltB8558:0.00369,UltB8570:0.00371)0.878.27:0.00774)0.
929.22:0.01572)0.743.20:0.00506,((UltOrg79:0.02188,UltB8575:0.03027)0.820
.10:0.00851,(UltB8561:0.03386,(UltB8555:5.4E-
4,UncUn112:0.0074)0.994.22:0.05103)0.836.16:0.01013)0.887.25:0.02286)0.77
8.21:0.01144,((((UltB8586:0.0,UltB8587:0.0):0.12615,((UltB8563:0.00605,((
UltB8564:5.5E-
4,UltB8565:0.0037)0.883.29:0.00748,UltB1844:0.01909)0.761.23:0.00621)0.92
5.32:0.01006,UltB8562:5.3E-
4)0.806.9:0.01729)0.474.6:0.01274,(UltB8233:0.08601,PahggY39:0.07805)0.95
6.19:0.04639)0.845.25:0.02079,UltB7604:0.05834)0.902.29:0.02796)0.890.28:
0.01693,(UltrSo68:0.18106,((UltB8899:5.5E-
4,(UltB8900:0.03168,UltB8901:0.06)0.982.11:0.04162)0.355.3:0.01042,UltB88
98:0.01665)0.984.12:0.08357)0.803.11:0.01965)0.875.25:0.01424,((UltB8546:
0.03521,((UltB8548:0.03724,(PrxBella:0.03217,((((UltChry2:0.06589,(UltB89
07:0.11518,((((((CrsDaeje:5.4E-
4,UltB8854:0.01124)0.513.1:0.00752,(UltB8846:5.5E-
4,(CrsTaean:0.00753,UltB8852:5.4E-
4)0.942.23:0.00752)0.850.26:0.00367)1.000.125:5.5E-4,(UltB8849:5.4E-
4,(((CrsSpe16:0.0188,((CrsSolda:0.0,CrsAquat:0.0,CrsSpe15:0.0):5.5E-
4,(CrsGrega:0.00365,UltB8847:0.01525)0.890.29:0.00754)0.931.23:5.5E-
4)0.895.29:0.00757,CrsSpe17:0.00376)0.891.22:0.00758,((CrsSpe13:0.0,CrsPi
sci:0.0,UltB8850:0.0,CrsSpe18:0.0):5.5E-
4,(UltB8858:0.04731,UltB8851:0.00741)0.902.30:5.5E-4)0.999.30:5.5E-
4)0.946.21:0.00743)0.928.22:0.00741)0.265.2:5.5E-
4,(((CrsForm2:0.0,CrsFormo:0.0):5.4E-
4,((((UltB8720:0.01499,(CanChrys:0.0,CrsSpe21:0.0,CrsStagn:0.0):5.5E-
4)0.339.3:5.5E-4,(UltB8853:5.5E-
4,((CrsSpe10:0.0,CrsSpe19:0.0,CrsbSoli:0.0):5.5E-4,(CrsProte:5.5E-
4,CrsSpe20:0.01133)0.742.14:5.5E-4)0.786.20:5.5E-
4)0.981.12:0.01515)0.948.30:0.00498,(CrsDaech:5.5E-4,(CrsWanju:5.5E-
4,CrsDeflu:0.00373)0.882.20:0.00368)0.948.31:0.00495)0.948.32:0.0049,UltB
8857:0.02664)0.936.25:5.5E-4)0.945.25:0.0074,UltB8848:5.4E-
4)0.903.23:0.00739)0.991.27:0.02281,CrsSpe14:5.4E-

4)0.594.3:0.00222,CrsDaegu:0.00517)0.987.22:0.0597)0.920.25:0.03431)0.808
.15:0.01774,((UltB8885:5.4E-
4,UltB8884:0.01138)0.994.23:0.08554,(UltB8927:0.09117,UltB8928:0.11688)0.
923.25:0.05114)0.859.22:0.03322)0.925.33:0.03219,((UltB8888:0.0435,AgpRat
ko:0.12448)0.887.26:0.03393,UltB8437:0.08356)0.654.3:0.00717)0.934.20:0.0
2754,((UltB8547:0.01625,UltProli:0.01625)0.922.24:0.02213,(UltB8552:0.017
84,UltB8553:0.05817)0.839.17:0.02728)0.226.3:5.4E-
4)0.933.32:0.02166)0.846.17:0.01496)0.935.24:0.02333,(UltB8551:0.06425,(B
actXB45:0.02033,UltB8554:0.04613)0.681.2:0.00438)0.657.4:0.01085)0.845.26
:0.01515)0.852.25:0.02222,UltB8758:0.04134)0.728.10:0.00405)0.793.16:0.00
447,(((UltGa288:0.02824,UltB8591:0.02654)0.624.5:0.00473,(UltAl196:0.0493
6,(UltB8589:0.02754,UltB8892:0.03888)0.834.12:0.00979)0.777.23:0.00598)0.
916.28:0.01494,((UltB8592:0.02723,(UltB8583:0.0669,UltB8585:0.02613)0.838
.19:0.01495)0.444.4:0.015,(UltCyto2:0.04887,UltCyto3:0.03929)0.904.20:0.0
2696)0.935.25:0.00119)0.908.23:0.01591)0.737.19:0.0027,(((UltB8581:5.4E-
4,((UltB7996:0.0,UltB8568:0.0,UltB8578:0.0,UltB8577:0.0,UltB8580:0.0):0.0
0752,UltB8579:0.01137)0.462.6:0.02311)0.861.26:0.03444,((((UltB8569:0.027
63,Ult27023:0.03584)0.925.34:5.4E-
4,(UltB8571:0.01221,UltB8567:0.01085)0.602.1:0.01145)0.780.18:0.00681,(Ul
tB8572:5.4E-
4,(UncUn113:0.01072,UltB8566:0.01274)1.000.126:0.06174)0.482.5:0.0152)0.7
92.25:0.00836,UltB8573:0.03549)0.898.31:0.01741)0.925.35:0.02531,(UltB858
8:0.07,(UltB8409:0.04044,UltOrg76:0.04955)0.745.18:0.03006)0.724.9:0.0060
1)0.884.23:0.01268)0.759.20:0.00387,((UltB8584:0.01113,(UltOrg80:0.02993,
UltB9047:0.06028)0.785.24:0.02906)0.880.28:0.01786,(UltOrg78:0.11062,UltB
8582:0.05859)0.842.25:0.02392)0.718.10:0.00207)0.926.24:0.01851)0.934.21:
0.02439)0.756.23:0.00528,((UltB8511:0.04842,UltB8509:0.03064)0.866.22:0.0
1876,(UltB8902:0.09056,((((((UltSph30:0.06218,(UltB8528:0.05721,(((UltB85
38:0.00444,(UltB8526:0.09828,UltAnae7:0.02575)0.855.16:0.01908)0.945.26:0
.02163,((UltB8513:0.04492,(UltB8537:5.5E-
4,UltB8536:0.00369)0.930.17:0.02396)0.759.21:0.01456,(UltB8529:0.02572,Ul
tB8535:0.0531)0.918.23:0.02405)0.870.18:0.0182)0.766.14:0.0058,UltB8512:0
.07304)0.665.7:0.00541)0.846.18:0.00892)0.784.15:0.00835,(((((UltB8515:0.
0,UltB8517:0.0):0.05251,UltB8522:5.5E-
4)0.781.19:0.00906,(UltB8518:0.04957,(UltB8521:0.00378,(UltB8520:0.01917,
UltB8519:0.00397)0.934.22:0.0153)0.912.30:0.0192)0.942.24:0.02448)0.951.2
3:0.04118,(UltB8516:0.07847,(UltB8523:0.02476,UltB8524:0.02906)0.393.3:0.
02552)0.396.6:0.02271)0.997.23:0.07721,(UltB8514:0.03271,(UltB8525:0.0910
6,UltrSo67:0.05927)0.661.4:5.4E-
4)0.773.21:0.01047)0.881.24:0.01125)0.761.24:0.01363,((SoiCanad:0.03473,(
UltB8502:0.00368,SoiKoree:5.5E-
4)0.910.36:0.0186)0.989.18:0.03714,UltrSo66:0.0234)0.849.20:0.01663)0.678
.7:0.00513,UltB8527:0.04771)0.958.24:0.02768,UltB8510:0.01736)0.871.22:0.
02016,(UltB8539:0.04267,UltB8540:0.049)0.949.24:0.03807)0.760.18:0.01201)
0.751.7:0.0092)0.753.15:0.00547)0.829.15:0.00782)0.864.26:0.01495)0.748.1
9:0.00704)0.903.24:0.01978,((UltB7605:0.05538,(((((((UltB7658:0.00517,Ult
B7659:0.01464)0.869.21:0.01278,(AtPYy110:0.03437,Cuiiii18:0.0257)0.065.3:
0.00851)0.869.22:0.00962,(UltB7654:0.01975,((UltB7653:0.0,UltB7655:0.0):0
.0037,Ult40960:5.5E-4)0.814.13:0.00739)0.883.30:0.01958)0.581.5:5.4E-
4,((Try00027:0.0,Try00044:0.0,TrZZZZ09:0.0):0.00759,(UltB7657:0.0114,(Ult
40962:0.00369,UltB7656:5.5E-
4)0.920.26:0.01147)0.938.21:0.01534)0.964.30:0.01924)0.863.23:0.01568,Otu
00498:0.03194)0.948.33:5.2E-4,(((UltB7663:0.0,Otu00182:0.0):5.4E-
4,(UltB7662:5.4E-4,(Ult42211:0.00369,Ult40985:5.5E-

4)0.838.20:0.00369)0.350.2:0.00718)0.963.21:0.02697,((PahggY35:0.0,S01144
94:0.0):0.01366,((UncUn450:0.0,UltB7661:0.0,UncUn451:0.0):5.5E-
4,(UncUnc75:0.0,UncUn452:0.0):0.00368)0.986.15:0.02634)0.881.25:0.01479)0
.620.6:0.01555)0.962.30:0.02646,((EchggYy6:0.01149,S0114542:0.04123)0.962
.31:0.02937,UltB7660:0.02004)0.777.24:0.00761)0.987.23:0.04903)0.395.3:0.
01031,((UltB7651:0.04373,UltB7652:0.00861)0.837.14:0.01299,((P0114444:0.0
109,S0114432:0.04527)0.992.19:0.06151,(((HmZZZZ09:0.0,Hmyyy058:0.0,Hmyy00
50:0.0,Hmyy0042:0.0,Hmyy0047:0.0):5.5E-
4,(Hmyy0048:0.0,Hmyyy018:0.0):0.00368)0.985.23:0.04872,((PahggY33:0.0,Psh
ggg74:0.0,Pshggg27:0.0):0.00374,PahggY34:0.01935)0.956.20:0.0403)0.546.5:
0.0147)0.944.18:0.03265)0.951.24:0.02726)0.916.29:0.02871)0.427.3:0.01266
)0.532.2:5.4E-
4,(UltB6487:0.06064,UltB7643:0.0608)0.895.30:0.02785)0.740.15:0.00607)0.9
93.16:0.06033,((((((FlxTrac3:0.0,FlxTrac4:0.0):0.00822,PxlAuran:0.01062)0
.670.2:0.07625,(FmmKamog:5.4E-4,((FmmYaeya:5.5E-4,FmmSpeci:5.4E-
4)0.933.33:0.02168,(FmmArena:0.01126,(FmmApric:5.5E-4,FmmSpec2:5.5E-
4)0.886.26:5.5E-
4)0.955.19:0.02266)0.857.28:0.02629)1.000.127:0.15646)0.861.27:0.03488,(P
cbDiffl:0.06379,(LmbArmen:0.07135,(RpdThail:0.07271,(FlhDoro2:0.00745,Flh
Doro3:5.5E-
4)0.898.32:0.0174)0.763.25:0.02372)0.646.4:0.01796)0.192.3:0.00804)0.318.
5:0.01399,SdxFlava:0.04831)0.959.29:0.04125,((((UltFlamm:0.048,((((((UltB
9168:0.01917,FvvKasya:0.01524)0.856.13:0.01146,(UltFlex7:5.4E-
4,FlxTrac2:0.01117)0.789.13:5.3E-
4)0.361.5:0.01182,UltB9169:0.10543)0.630.5:0.01553,(UltB9170:0.03497,(Ult
Cyt10:0.03533,UltB9184:0.05601)0.822.11:0.01049)0.855.17:0.01071)0.912.31
:0.01573,(((((UltB9166:0.27091,FlxSpec2:0.02938)0.503.2:0.03532,((((FctLa
cus:5.3E-
4,(FctSpeci:0.04825,(ArlRosea:0.00314,UltB9111:0.01962)0.845.27:0.00767)0
.882.21:0.00779)0.966.28:0.03342,(((UltB9110:0.09759,RhgAerol:0.0652)0.16
4.2:0.02992,(UltB9091:0.05597,((((UltB9108:0.09398,(RudLutea:0.02972,(Ult
B9102:0.02194,(SrmLingu:5.5E-
4,((((UltB9100:0.0,UltB9101:0.0):0.04882,((UltB9103:0.01664,((UltFlex2:0.
02982,UltB9106:0.0414)0.950.21:0.03475,(SrmPanac:0.00897,(UltFlex3:0.0469
2,(UltB9105:0.06068,UltSph36:0.04728)0.879.21:0.02156)0.962.32:0.03757)0.
905.21:0.02766)0.799.18:0.01249)0.756.24:0.00421,UltB9104:0.06263)0.918.2
4:0.01711)0.699.11:0.00516,((SrmLuteu:5.4E-
4,SrmSpits:0.03358)0.993.17:0.03463,SrmRigui:0.00738)0.919.26:0.01398)0.8
46.19:0.01088,(SrmNavaj:0.0,SrmLing2:0.0):0.01932)0.374.3:0.00395)0.964.3
1:0.04408)0.972.18:0.05021)0.888.34:0.03062)0.810.14:0.03936,(UltrSo80:0.
04293,(UltFlex4:0.0518,UltB9107:0.02585)0.859.23:0.02931)0.464.4:0.04148)
0.819.18:0.0432,(LrkInspe:0.04332,UltB9109:0.04277)0.100.2:0.0475)0.988.2
4:0.10449,((DyaGinse:0.00788,((DyaAlkal:0.00755,((DyaKore2:0.00758,Ult153
45:0.02181)0.900.23:0.01257,(UltB9090:0.00371,(DyaCrust:5.4E-
4,((((DyaHamte:0.00369,(UltDyado:0.0,UltB9089:0.0):5.5E-4)0.964.32:5.5E-
4,DyaKoree:0.01542)0.912.32:0.0075,UltB9088:0.0075)0.772.22:0.00379,UltB9
087:0.00369)0.890.30:0.00748)0.826.15:0.00371)0.913.20:5.4E-
4)0.904.21:0.01491)0.756.25:5.5E-
4,DyaSpeci:0.02305)0.711.9:0.0041)0.810.15:0.00949,(DyaBeiji:5.5E-
4,(DyaFerme:5.5E-4,DyaFerm2:5.5E-
4)0.964.33:0.02338)0.291.2:0.0177)0.911.29:0.03822)0.759.22:0.02094)0.992
.20:0.10023)0.624.6:0.02303,(((RunDeflu:0.01161,(UltB9092:5.4E-
4,(RuneZeae:0.01118,UltB9093:5.4E-
4)0.999.31:0.03978)0.853.16:0.00737)0.097.4:0.00688,RunLimos:0.00903)0.99

2.21:0.10369,FlxFlex2:0.11603)0.291.3:0.02762)0.835.19:0.02574)0.826.16:0
.01381,(UltB9141:0.04768,((UltB8483:0.01485,(UltB9112:0.00369,UltB9113:5.
5E-4)0.851.24:5.3E-
4)0.968.16:0.03849,(UltB9130:0.09814,SipAquae:0.03123)0.864.27:0.04245)0.
381.7:0.01096)0.940.17:0.02692)0.970.22:0.04224,(((FlxFlexi:0.08857,UltFl
ex6:0.06576)0.270.2:0.0149,FlxRuber:0.18727)0.740.16:0.00985,((CytHutch:5
.5E-
4,UltB9142:0.02722)0.986.16:0.0499,((((UltB9116:0.02178,(UltB9119:0.01819
,UltB9115:0.00824)0.729.8:0.0043)0.880.29:0.01255,(UltB9114:0.02836,(UltF
lex5:0.0266,UltB9118:0.02215)0.756.26:0.00482)0.902.31:0.01194)0.925.36:0
.02133,(((UltB9126:0.02856,((UltB9122:0.00722,UltB9128:0.01162)0.874.28:0
.01333,(UltB9127:0.03883,(UltB9123:0.07088,(UltB9121:0.03215,(UltB9120:0.
03918,BcsBact9:0.01497)0.558.2:5.5E-
4)0.814.14:0.00873)0.591.6:0.01063)0.927.24:0.02103)0.254.2:0.01066)0.906
.35:0.01831,UltB9125:0.00704)0.830.20:0.01316,UltB9124:0.00375)0.921.38:0
.01455)0.665.8:0.00581,UltB9117:0.02343)0.961.23:0.04216)0.910.37:0.02612
)0.777.25:0.00858)0.906.36:0.01855)0.942.25:0.0284,(((BcsBac10:0.00582,Rc
hAgari:0.00155)0.400.3:0.01093,(UltB9172:0.02829,UltB9171:0.04094)0.868.1
9:0.0146)0.953.19:0.03888,UltB9186:0.05594)0.858.20:0.0151)0.579.3:0.0039
4,((FlxTrac5:0.03695,(RsgEhren:0.02292,(RsgSpong:0.01127,(FabHalot:0.0036
9,UltFlex8:5.4E-4)0.888.35:0.00752)0.895.31:5.5E-
4)0.989.19:0.03322)0.082.2:0.00788,UltB9167:0.10211)0.857.29:0.01485)0.88
1.26:0.01055,((((UltOrg85:0.01442,(UltB9159:0.023,(UltB9158:5.3E-
4,HymeSoli:0.01103)0.396.7:0.00371)0.935.26:0.01516)0.780.19:0.0079,((Ult
Hyme4:0.00369,UltHyme3:5.4E-
4)0.765.15:0.00878,(UltB9156:0.0035,(UltB9157:0.00352,Ult30683:0.00391)0.
967.17:0.01955)0.959.30:0.01857)0.901.31:0.01256)0.955.20:0.02284,(((HymC
hiti:0.01124,((HymXinji:0.00368,HymRigui:5.4E-
4)0.935.27:0.00741,(((((HymNorwi:0.00354,UltHymen:0.01145)0.891.23:0.0083
1,(HymGelip:0.02933,(HymRoseo:5.4E-
4,UltHyme2:0.01512)0.981.13:0.02975)0.959.31:0.02957)0.834.13:0.00697,Ant
Bac38:0.00372)0.887.27:0.00752,(HymSpeci:0.0,HymAerop:0.0):5.3E-
4)0.875.26:0.00747,UltB9145:0.0151)0.883.31:0.00741)0.508.3:5.5E-
4)0.891.24:0.01237,(HymPsych:0.00817,(HymDeser:0.01141,(HymDaech:0.01894,
(HymOcel2:5.5E-4,(HymOcell:5.5E-
4,UltB9162:0.00741)0.835.20:0.00369)0.862.24:5.5E-
4)0.963.22:0.02958)0.968.17:0.03061)0.871.23:0.01234)0.917.22:0.01694,(Ul
tB9161:0.04886,(UltB9150:0.0103,((UltB9146:0.0234,(UltB9148:5.4E-
4,((UltB9151:0.03509,UltB9149:5.4E-
4)0.915.22:0.01207,UltB9147:0.02851)0.805.18:0.00666)0.937.22:0.01159)0.9
06.37:5.5E-
4,(UltB9152:0.00733,(UltB9153:0.03584,(UltB9155:0.03992,UltB9154:0.01512)
0.736.10:0.00379)0.332.1:5.5E-
4)0.799.19:0.00667)0.930.18:0.01141)0.766.15:0.00446)0.844.25:0.01244)0.2
55.1:0.00289)0.989.20:0.06789,(UltB9163:0.02285,((((PnbKorle:0.0,PnbSpec2
:0.0):5.4E-4,(UltPonti:0.00367,(PnbAkesu:0.01885,(PnbSpeci:5.5E-
4,PnbActin:5.5E-4)0.900.24:5.4E-4)0.745.19:5.4E-
4)0.449.5:0.00742)0.948.34:0.01517,EffRoseu:0.01139)0.885.18:0.00732,UltC
yto9:5.4E-
4)0.995.23:0.07093)0.663.4:0.01896)0.979.18:0.06379)0.837.15:5.4E-
4)0.883.32:0.00908,(UltB9177:0.06503,(UltB9144:0.04613,((UltB9185:0.04875
,(((SrtMyxoc:0.04588,UltB9183:0.05511)0.903.25:0.02045,(AdhTerre:0.04829,
(UltB9165:0.01782,AdhSpeci:0.00503)0.923.26:0.01645)0.859.24:0.01284)0.39
9.3:0.00451,UltB9164:5.4E-

4)0.961.24:0.02291)0.754.20:0.0071,((UltB9176:0.04258,(UltB9175:0.00599,UltOrg88:0.07637)0.949.25:0.0371)0.823.12:0.01448,(UltOrg86:0.03169,(FlhDorot:5.4E-4,(UltB9143:0.03095,UltB9174:0.05791)0.756.27:0.00456)0.926.25:0.01432)0.462.7:0.00689)0.910.38:5.5E-4)0.608.3:0.0038)0.744.14:0.00385)0.787.23:0.00537)0.772.23:0.0041)0.714.7:0.00845,((UltB9181:0.10471,(((CdnEndo3:5.4E-4,(CdnEndos:0.0112,(CdnEndo2:0.00747,CanCardi:0.00742)0.715.2:5.4E-4)0.912.33:0.01508)0.998.23:0.05744,(CanAmoeb:0.03371,UltB9180:0.07193)0.306.2:0.00695)0.879.22:0.01937,UltCyt11:0.04243)0.913.21:0.03372)0.951.25:0.05008,((((UltSph34:0.15,(UltB8887:0.06594,((UltB8867:0.01198,(UltB8866:0.02475,(UltB8865:0.02058,UltB8868:0.0529)0.174.3:0.00824)0.986.17:0.04748)0.963.23:0.04135,((ClgMacul:0.03057,(((UltB8883:0.02138,((FlcEnd35:0.0,UltB8882:0.0):0.0054,FlcEnd36:0.00613)0.947.28:0.02363)0.788.18:0.01408,((((UltB8879:0.01893,((FlcEnd26:0.00739,UltB8880:0.0037)0.781.20:0.00369,(FlcEnd27:0.01116,((FlcEnd28:0.01525,(FlcEnd30:0.01525,FlcEnd29:0.00367)0.751.8:0.00381)0.880.30:0.00742,FlcEnd31:0.015)0.850.27:5.5E-4)0.822.12:0.00365)0.745.20:0.00359)0.782.24:0.00372,(FlcEnd32:0.01512,FlcEnd34:0.00749)0.777.26:0.00372)0.842.26:0.00708,(((FlcEnd20:0.01799,FlcEnd25:0.04794)0.753.16:0.01325,(((((FlcEndo9:0.01114,((FlcEnd10:5.4E-4,((FlcEnd16:0.00743,UltB8874:0.00371)0.807.10:5.3E-4,(FlcEnd15:5.4E-4,(FlcEnd13:0.01125,FlcEnd14:0.00369)0.939.23:0.01128)0.963.24:0.01124)0.985.24:0.01876)0.743.21:0.00365,(FlcEnd12:5.4E-4,FlcEnd11:0.0113)0.846.20:0.00372)0.874.29:5.4E-4)0.645.4:0.00366,((((FlcEndo8:0.01075,(FlcEndo4:0.00647,FlcEndos:0.01478)0.736.11:0.00289)0.815.13:0.0062,(FlcEndo3:0.00714,FlcEndo5:0.01561)0.595.2:0.00746)0.865.27:5.5E-4,FlcEndo6:5.3E-4)0.841.20:0.00366,(FlcEndo7:0.01898,FlcEndo2:0.00746)0.934.23:5.4E-4)0.790.15:0.00378)0.828.16:0.00691,(FlcEnd19:0.00742,(FlcEnd21:0.02264,(UltB8877:0.00343,FlcEnd22:0.02374)0.786.21:0.00589)0.872.33:0.01027)0.144.2:5.3E-4)0.886.27:0.0073,FlcEnd23:0.00372)0.788.19:0.00368,((FlcEnd17:0.01959,UltB8878:0.01493)0.484.1:0.01123,(UltB8875:5.4E-4,(FlcEnd18:0.00758,UltB8876:0.00365)0.935.28:0.01143)0.871.24:5.4E-4)0.859.25:0.00703)1.000.128:5.4E-4)0.910.39:0.00348,(FlcEnd33:0.03514,FlcEnd24:0.01124)0.740.17:0.00382)0.771.22:0.00404)0.906.38:0.02467,UltB8881:0.12469)0.859.26:0.01962)0.797.16:0.01258,((CanSul11:0.00404,((((CanSul14:0.02624,CanSul15:0.0212)0.994.24:0.0601,(CanSul12:0.01143,CanSul13:0.01125)0.931.24:0.02152)0.941.21:0.02398,(CanSulc9:0.01122,CanSulc8:0.00374)0.968.18:5.4E-4)0.958.25:0.02268,(CanSulc5:0.00751,((CanSulc4:0.00393,((CanSulc2:0.0,CanSulci:0.0):5.5E-4,CanSulc3:0.00369)0.880.31:0.00727)0.396.8:0.00742,(CanSulc6:0.00563,(CanSul10:0.01313,CanSulc7:0.01321)0.066.2:0.00372)0.824.24:0.00534)0.225.3:5.4E-4)0.856.14:0.0078)0.822.13:0.01112)0.993.18:0.03841,(UltB8873:0.03557,(BlbSpec3:0.01479,((((BlbSpec8:0.00371,BlbSpec2:0.00742)0.793.17:0.00363,((BlbSpec5:5.5E-4,((BlbSpec6:0.00371,(BlbPunc2:0.02644,BlbPunct:5.5E-4)0.972.19:0.01495)0.865.28:0.00737,BlbSpec4:0.00367)0.751.9:5.4E-4)0.909.21:0.00366,((PahggY40:5.5E-4,PahggY41:0.00366)0.000.230:5.5E-4,BlbSpeci:5.5E-4)0.831.21:5.3E-4)0.169.1:5.4E-4)0.842.27:0.00363,(UltB8872:0.01094,(BlbSpe10:0.01504,BlbSpe11:5.4E-4)0.914.19:5.4E-4)0.864.28:0.01488)0.063.3:5.5E-4,((BlbSpec9:0.00711,BlbSpec7:0.03065)0.913.22:5.4E-

4,Pshggg36:0.00364)0.831.22:0.00365)0.830.21:5.4E-
4)0.758.23:0.00375)0.762.20:0.00459)0.836.17:0.01271)0.708.6:0.00392)0.99
9.32:0.07924,(Otu04652:0.045,((((((UltB8820:5.5E-
4,UltB8823:0.00369)0.949.26:5.5E-4,(UltB8821:5.5E-
4,UltB8719:0.00369)0.808.16:0.00366)0.984.13:0.03787,UltB8871:0.07341)0.7
03.8:0.00239,(((((UltB8818:0.05803,((((((FlbBac14:0.01522,((UltB8833:5.5E
-4,(UltB8832:0.00369,((CrsSpe12:5.3E-
4,CrsHaife:0.00369)0.871.25:0.00371,UltB8834:0.00369)0.036.3:5.5E-
4)0.751.10:5.5E-4)0.723.12:5.5E-4,UltB8835:5.5E-4)0.882.22:5.4E-
4)0.834.14:0.00369,(CrsBovis:5.5E-
4,(CrsHispa:0.0153,((CrsHomin:0.0,CrsHomi2:0.0,UltB8844:0.0):5.5E-
4,(CrsPalli:0.0,CrsMolle:0.0,UltB8859:0.0):0.00369)0.931.25:5.4E-
4)0.956.21:0.00746)0.851.25:0.0037)0.689.7:5.5E-
4,(CrsHunga:0.0113,(CrsKoree:0.00376,(CrsCaeni:0.00345,UltB8837:0.02371)0
.961.25:0.01564)0.778.22:0.00374)0.890.31:0.00754)0.954.22:0.01904,(UltB8
840:0.01122,UltB8845:0.00778)0.824.25:0.01143)0.823.13:5.4E-
4,SejJeoni:5.4E-4)0.869.23:0.00775,((SejMarin:0.01078,AntBac37:5.5E-
4)0.939.24:0.03582,(UltB8831:0.00376,(SejAntar:0.00355,BcsBact7:0.01146)0
.770.24:0.00383)0.840.19:0.0073)0.876.24:0.00804)0.744.15:0.00802)0.901.3
2:0.02606,((((UltB8825:0.01846,UltB8855:0.01416)0.900.25:0.01422,(CrsGleu
2:5.5E-
4,((((UltB8830:0.00193,CrsIndol:0.02819)1.000.129:0.00175,(CrsSpec8:0.0,C
rsSpec4:0.0):0.00369)0.000.231:5.5E-
4,((CrsSpec3:0.0,CrsSpec2:0.0,UltChrys:0.0,UltB8827:0.0,CrsSpec7:0.0,CrsG
leum:0.0,UltB8829:0.0,CrsJoost:0.0,CrsSpe22:0.0):5.5E-
4,((UltB8828:0.00369,CrsSpeci:0.0037)0.000.232:5.5E-
4,((((CrsSpe11:0.0,CrsUreil:0.0):5.5E-
4,(BacSV70A:0.0,CrsOrani:0.0):0.00369)0.909.22:0.00702,(((CrsFlavu:0.0,Cr
sVryst:0.0,CrsSpec5:0.0):5.4E-4,(CrsSpec9:0.12373,((CrsSpec6:5.5E-
4,CrsShige:5.5E-4)0.507.2:5.5E-
4,(UltB8826:0.01879,CrsLuteu:0.00369)0.935.29:5.5E-4)1.000.130:5.5E-
4)0.845.28:0.00365)0.858.21:0.00372,(CrsIndo3:0.0,CrsAquif:0.0,CrsKwang:0
.0):5.4E-4)0.875.27:0.00211)0.041.2:0.00212,CrsJejue:5.4E-
4)0.837.16:0.00375)0.382.3:5.5E-4)0.000.233:5.5E-4)0.240.2:5.5E-
4,CrsIndo2:5.5E-4)0.486.5:5.5E-
4)0.895.32:0.00752)0.772.24:0.00373,CrsTaich:0.00373)0.963.25:0.01519,Crs
Moech:5.5E-
4)0.881.27:0.01483)0.887.28:0.01607,UltB8839:0.01226)0.819.19:0.00885,(Ul
tB8819:0.00335,(((((UltB8838:0.00759,BglSpeci:0.03622)0.801.17:0.00763,((
(ElzMenin:0.0,ElzMeni3:0.0):5.5E-
4,ElzMeni2:0.01115)0.986.18:0.0297,((UltB8842:5.5E-
4,UltB8843:0.00369)0.923.27:0.01395,(PlmTakli:0.00366,UltB8841:5.4E-
4)0.972.20:0.02571)0.182.2:0.00354)0.864.29:0.014)0.961.26:0.01946,UltFla
14:5.4E-4)0.877.34:0.01219,(RieColum:5.4E-
4,(RieAnati:0.01889,RieAnat2:5.5E-
4)0.944.19:0.01508)0.988.25:0.0353)0.796.12:0.01898,(UltB8815:0.03556,((U
ltB8812:0.01509,(UltB8817:0.01922,UltB8814:0.03064)0.756.28:0.00323)0.185
.3:5.5E-4,((((UltB8813:0.01114,(UltB8824:5.3E-
4,UltB8809:0.06878)0.350.3:0.00369)0.932.26:5.4E-4,UltB8816:5.5E-
4)0.616.3:5.4E-4,(Udntfde7:0.0,UltB8806:0.0,UltB8808:0.0):5.5E-
4)0.651.7:5.5E-4,(UltB8807:0.00368,(UltB8810:0.01493,UltB8811:5.5E-
4)0.799.20:0.00369)0.767.20:0.00368)0.356.1:5.5E-4)0.881.28:5.5E-
4)0.533.2:0.00454)0.514.3:0.01549)0.955.21:0.03054)0.626.4:0.01756,UltB88
56:0.047)0.881.29:0.01315)0.895.33:0.0162,(OrtRhino:0.03924,(UltB8860:0.0

0587,UltB8861:0.00153)0.968.19:0.03578)0.226.4:0.01435)0.736.12:0.00748,(
(UncUn114:0.05633,(Otu01084:0.01417,(Kty00022:0.04383,(UltB8870:5.4E-
4,UltB8869:0.00747)0.860.17:0.00803)0.527.2:0.00911)0.740.18:0.02188)0.94
7.29:0.03013,(((UltB8862:0.00369,WauFalse:5.4E-
4)0.980.11:0.02391,((EmpSpec2:0.00367,EmpSpeci:5.5E-
4)0.838.21:0.00689,(UltB8863:0.00381,UltB8864:0.01137)0.948.35:0.01609)0.
911.30:0.01181)0.922.25:0.01467,EmpBrevi:5.5E-
4)0.968.20:0.02998)0.884.24:0.01817)0.493.6:0.01926)0.896.18:0.01977)0.74
5.21:0.00881)0.904.22:0.02509)0.895.34:0.03149)0.910.40:0.03408,((LeaByss
o:0.06271,(UltB9097:0.01225,(UltB9096:0.01848,(UltB9099:0.04021,(UltB9098
:0.05909,(EmtSpeci:5.4E-4,(UltB9095:0.0531,(EmtGinse:5.5E-
4,(EmtOligo:5.5E-
4,UltB9094:0.01862)0.823.14:0.00366)0.741.11:0.00377)0.864.30:0.00732)0.9
90.20:0.06113)0.902.32:0.03853)0.859.27:0.01815)0.483.4:0.00331)0.936.26:
0.04054)0.998.24:0.10703,TmmRossi:0.09753)0.797.17:0.02232)0.849.21:0.033
18,((FlxRose3:0.10371,(FlxLitor:0.13694,FlxRose2:0.07927)0.442.2:0.02419)
0.941.22:0.06383,FlxPolym:0.15621)0.153.3:0.03375)0.847.26:0.02343,((MnsP
acif:0.01534,(UltB9182:0.09498,MnsFurve:0.00743)0.660.4:0.0047)0.971.17:0
.04067,(UltB9173:0.13019,(FlxRoseo:0.08921,McsMarin:0.11247)0.850.28:0.03
369)0.730.10:0.01619)0.867.25:0.02361)0.745.22:0.00461)0.950.22:5.5E-
4)0.846.21:0.01304,((UltComp3:0.05155,McsSeric:0.10278)0.702.2:0.01202,((
(UltB9132:0.03894,(UltB9131:0.01634,AqfBalti:0.04272)0.824.26:0.01336)0.7
74.33:0.01283,(((BacteSL3:0.09034,((UltB9138:5.4E-
4,(UltB9139:0.00765,(AgpHiton:5.4E-
4,(((((AgpChord:0.0,AgpSpeci:0.0,AgpAntar:0.0,ArctiSe5:0.0):5.4E-
4,(AgpSpec2:0.0037,CclSpeci:0.0037)0.797.18:0.0037)0.950.23:0.00752,AgpLo
cis:5.4E-4)0.933.34:0.01134,(AgpAlkal:5.4E-
4,(AgpAquae:0.00271,(AgphOlei:0.00378,UltSph37:0.00745)0.975.19:0.02057)0
.971.18:0.02018)0.898.33:0.00744)0.940.18:0.00201,(AgpTerri:0.00926,AgpBo
rit:0.01875)0.955.22:0.01857)0.771.23:0.0018)0.886.28:0.00739)0.266.1:0.0
0375)0.902.33:0.01987,((FxbBacte:0.00382,(AgpMarin:0.0111,(AgpManni:0.007
49,UltB9140:5.3E-4)0.975.20:0.01928)0.864.31:0.00765)0.908.24:5.5E-
4,AgpVanfo:0.03584)0.794.19:0.01473)0.846.22:0.01964)0.895.35:0.02754,(Ul
tB9135:0.01495,UltB9136:0.01612)0.771.24:0.00906)0.645.5:0.03084,UltB9137
:5.5E-
4)0.981.14:0.05238)0.183.5:0.01442,(((BacIFAM4:0.04462,(BacIFAM5:0.0,UltB
9133:0.0):5.4E-
4)0.988.26:0.05059,(((CclMarin:0.00374,CclAmurs:0.00752)0.970.23:0.01981,
(BacIFAM3:5.5E-4,BacIFAM2:0.05692)0.986.19:0.0227)0.515.1:5.4E-
4,CclLianu:0.02437)0.920.27:0.02382)0.861.28:0.02266,((EcnVietn:0.00657,(
EcnPacif:0.01426,(((BeeBalti:0.00838,BeeSpeci:0.03695)0.900.26:0.01986,(R
dnPsych:0.02367,(UdnHaila:0.00985,RdnPsyc2:0.00991)0.956.22:0.02401)0.831
.23:0.00832)0.945.27:0.02591,BeeSpec2:0.03488)0.560.4:0.01584)0.932.27:0.
03037)0.458.4:5.3E-
4,UltB9134:0.03036)0.885.19:0.01664)0.905.22:0.03131)0.980.12:0.05482)0.9
48.36:0.0392)0.685.5:0.01628,UltB9178:0.1351)0.316.1:0.00741)0.355.4:0.02
325)0.895.36:0.02888,((UltB5918:0.14846,UltB6124:0.07454)0.799.21:0.03516
,(UltB8542:0.0855,LgrCyan2:0.08456)0.957.19:0.06038)0.669.6:0.00516)0.725
.9:0.01735)0.936.27:0.02518,(Ult19267:0.07554,(UltB7915:0.07402,((UltB773
8:0.04262,(UltB8610:0.12475,UltB7853:0.0042)0.825.15:0.02066)0.874.30:0.0
2646,UltB8611:0.10511)0.893.31:0.03028)0.898.34:0.02506)0.925.37:0.02063)
0.726.6:0.00596)0.679.3:0.0183)0.728.11:0.01784)0.235.3:5.4E-
4,UltAna14:0.1656)0.985.25:0.04182,((((((Ult18327:0.0,Ult18326:0.0,Ult23
756:0.0):0.0121,((Ult23750:0.01967,Ult23752:0.04016)0.656.5:0.00393,((Ult

Cl347:0.00257,Ult23749:0.09827)0.592.5:0.01248,(Ult23751:0.01117,((Ult23754:0.0,Ult23755:0.0):0.01142,(UltRu508:0.00369,Ult23753:5.5E-4)0.979.19:0.02731)0.885.20:0.01147)0.901.33:5.4E-4)0.926.26:0.01306)0.997.24:0.04799)0.683.7:0.01182,Ult23757:0.01576)0.999.33:0.07011,(Ult23760:0.02403,(Ult23759:0.00551,Ult23758:0.05662)0.996.14:0.06171)0.913.23:5.4E-4)0.826.17:0.0198,((Ult24432:0.0507,Ult24591:0.14986)0.970.24:0.07137,((((((((Ult23630:0.01116,(UltRu492:0.01988,((Ult23638:0.01164,UltRu493:0.02332)0.954.23:0.01897,(Ult23639:0.01124,(((((Ult23741:5.4E-4,(((Ult23676:0.0,Ult23677:0.0):5.5E-4,Ult23679:0.0037)0.886.29:5.4E-4,Ult23680:0.01521)0.919.27:0.00723)0.844.26:0.00718,Ult23678:0.00383)0.915.23:0.01427,((Ult23641:5.5E-4,Ult23642:5.5E-4)0.788.20:0.00395,((Ult23646:0.0,Ult23643:0.0,Ult23645:0.0):5.5E-4,Ult23644:5.5E-4)0.914.20:0.00839)0.913.24:0.0172)0.758.24:0.00662,(Ult23659:0.01201,Ult23675:0.02296)0.586.4:0.00647)0.734.11:0.00805,((Ult23694:0.0028,(Ult23731:0.07758,Ult23730:0.0163)0.972.21:0.03984)0.529.2:0.00645,(((Ult23650:0.03796,UdnRum35:0.0385)0.892.22:0.01893,(UltRu495:5.4E-4,Ult23647:0.03911)0.866.23:0.01164)0.807.11:0.01249,(Ult23648:0.00368,Ult23649:5.5E-4)0.775.17:0.00883)0.986.20:0.03387)0.911.31:0.01691)0.700.10:0.00459)0.891.25:5.4E-4)0.362.4:0.00719)0.839.18:0.01162)0.962.33:5.4E-4,(((Ult23626:0.00741,(Ult23624:5.5E-4,Ult23625:5.5E-4)0.885.21:5.5E-4)0.813.11:0.00366,(Ult23629:0.01136,(Ult23623:5.5E-4,(Ult23622:5.5E-4,(Ult23627:0.00369,Ult23628:0.00371)0.757.14:5.5E-4)0.739.19:5.5E-4)0.926.27:5.4E-4)0.786.22:0.00372)0.790.16:0.00371,(Ult23707:0.02689,(((((Ult23661:0.00373,((UltRu494:0.03994,((Ult23683:5.3E-4,Ult23682:0.01131)0.966.29:0.01562,Ult23743:0.00361)0.798.18:0.00962)0.737.20:0.00343,((UltLac51:0.03413,(UltFir59:0.03932,Ult23713:0.00966)0.871.26:0.01745)0.784.16:0.00755,(Ult23665:0.00378,Ult23664:0.00366)0.932.28:0.01508)0.759.23:0.00413)0.737.21:0.00311)0.832.13:0.00369,(Ult23653:0.01529,Ult23663:0.01534)0.770.25:0.00382)0.995.24:5.4E-4,Ult23658:0.00369)0.840.20:5.3E-4,(((((Ult23660:0.0,UltCl335:0.0):5.3E-4,Ult23652:0.01128)0.240.3:0.00744,Ult23657:0.01129)1.000.131:5.3E-4,Ult23654:5.5E-4)0.931.26:5.3E-4,((UdnRum36:0.02748,Ult23712:0.0037)0.920.28:0.01145,(Ult23708:5.5E-4,UltRu500:0.00369)0.779.19:0.00362)0.886.30:0.00752)0.891.26:0.00748)0.836.18:0.00367,UltRu496:5.4E-4)0.916.30:0.01476)0.958.26:5.4E-4)0.881.30:0.00368)0.774.34:0.00355,UltCl336:0.04832)0.753.17:0.00375,((((Ult23686:0.00967,Ult23637:0.01721)0.586.5:0.01143,((Ult23671:0.0,Ult23672:0.0):0.00369,(((Ult23725:5.5E-4,(Ult23724:0.00372,(UltRu506:0.03989,Ult23721:0.01528)0.893.32:0.01144)0.799.22:0.00382)0.910.41:0.00749,((Ult23662:5.3E-4,((Ult23674:0.01169,Ult23670:0.01988)0.762.21:0.00337,(UltRu497:0.02001,(Ult23666:0.00775,((Ult23667:0.00369,Ult23668:5.5E-4)0.897.17:0.00757,(Ult23669:5.4E-4,Ult23740:0.00726)0.841.21:0.00728)0.869.24:0.00736)0.745.23:0.00371)0.872.34:0.00775)0.802.17:0.00398)0.856.15:0.00369,(Ult23693:0.01511,((Ult23692:0.01247,(Ult23688:5.3E-4,((UltRu499:5.5E-4,Ult23687:0.00743)0.956.23:0.01158,Ult23689:0.00364)0.773.22:0.00725)0.790.17:0.01119)0.851.26:0.00746,OphioYy7:0.01585)0.750.13:0.00324)0.669.7:0.00759)1.000.132:5.5E-4)0.292.1:5.4E-4,Ult23651:0.0074)0.384.2:5.4E-4)0.902.34:0.01724)0.555.3:5.4E-4,(((UltFir58:5.4E-4,(Ult23718:5.4E-

4,Ult23719:0.00366)0.995.25:0.03506)0.916.31:0.01747,(PahggY82:0.02238,(U
lt23695:5.4E-4,(Ult23737:0.04845,(Ult23696:0.01927,UltCl341:5.5E-
4)0.701.7:5.3E-
4)0.876.25:0.00741)0.791.14:0.00685)0.787.24:0.00647)0.856.16:0.00793,(Ul
t23726:0.04768,Ult24391:0.03013)0.941.23:0.02259)0.834.15:0.00736)0.913.2
5:0.00997,(Ult23655:0.00747,Ult23742:5.4E-
4)0.948.37:0.01118)0.607.1:5.4E-
4)0.919.28:0.00741,((((((Ult23748:0.00369,UltCl346:0.0037)0.880.32:5.5E-
4,(Ult23706:0.00939,(UltCl344:0.00376,UltCl345:0.00747)0.923.28:0.01464)0
.947.30:0.01866)0.966.30:0.01888,Ult23702:5.4E-
4)0.573.2:0.00757,((Ult23701:5.5E-4,(Ult23697:0.01114,(((Ult23698:5.5E-
4,Ult23699:5.5E-4)0.849.22:0.00368,Ult23739:0.00371)0.389.4:5.5E-
4,(Ult23954:5.5E-4,Ult23700:0.00742)0.767.21:5.5E-4)0.891.27:5.5E-
4)0.812.11:0.00367)0.971.19:0.0112,(Ult23703:0.00741,(UncUn194:0.00369,Ul
t23640:5.5E-4)0.906.39:0.00745)0.587.5:5.5E-
4)0.883.33:0.00755)0.877.35:0.00748,((Ult23704:0.0,Ult23705:0.0):0.0036,U
ltCl334:0.06226)0.945.28:5.4E-4)0.908.25:0.00798,((((UltRu498:5.5E-
4,(Ult23709:0.01502,Ult23711:0.00742)0.779.20:5.5E-4)0.835.21:5.5E-
4,(Ult23691:0.00746,(((Ult23747:5.5E-4,Ult23685:0.00745)0.458.5:5.5E-
4,Ult23684:0.0113)0.855.18:0.00367,Ult23681:0.02315)0.953.20:5.4E-
4)0.853.17:0.0037)0.564.3:5.4E-4,((Tryyy096:0.00369,Tryy0101:5.5E-
4)0.976.14:0.01943,Ult23710:0.00365)0.885.22:0.00758)0.923.29:0.01075,(Ul
t23727:0.0193,Ult23728:0.00755)0.454.3:5.4E-
4)0.340.3:0.00374)0.758.25:0.00314)0.808.17:5.3E-
4,(Bfhggg68:0.0,Bfhggg69:0.0):0.01131)0.901.34:0.00755,((((UltRu507:0.032
04,Ult23714:0.0327)0.741.12:0.00274,((UltRu504:0.0461,((Ult23656:0.03875,
(((Ult23716:5.4E-4,Ult23717:0.03129)0.785.25:5.4E-
4,Ult23720:0.01512)0.561.1:0.00952,(UltRu505:0.01699,(UltRu501:0.0,UltRu5
02:0.0):0.01333)0.347.2:0.00379)0.744.16:0.00629)0.835.22:0.0078,UltRu503
:0.01035)0.377.2:0.01841)0.727.7:0.00174,Ult23715:0.00399)0.964.34:0.0194
6)0.569.3:0.00234,((((Ult23690:0.04684,UltCl338:0.01147)0.901.35:0.01886,
((Ult23723:0.01207,UltCl343:0.00306)0.997.25:0.03229,(AtPYy145:0.04132,Ul
t23722:0.02149)0.789.14:0.00698)0.387.3:5.5E-
4)0.709.5:0.0184,(Ult23673:0.00782,Ult24718:0.01519)0.923.30:0.01712)0.85
3.18:0.01634,(UltCl340:0.01249,PahgYy83:0.0362)0.959.32:0.03105)0.877.36:
0.00771)0.981.15:0.01859,(UltCl337:0.06647,UltCl339:0.07686)0.081.3:0.018
55)0.987.24:5.3E-
4)0.769.19:0.00666,Ult11334:0.16726)0.549.3:0.01244,(Ult23732:0.02812,Ult
23733:0.039)0.964.35:0.03219)0.804.15:0.02061)0.967.18:0.05583)0.873.28:0
.03118,((Ult23573:0.04309,UltCl348:0.07582)0.764.21:0.02664,((((((Ult2359
9:0.0088,(Ult23575:0.03759,UltCl319:0.01534)0.038.1:0.00367)0.821.17:0.01
014,(OphioYy6:0.03805,(Otu00818:0.02423,(Otu00331:5.5E-
4,(Otu00111:0.00746,Otu00372:0.00784)0.785.26:0.00381)0.999.34:0.05175)0.
876.26:0.01439)0.448.3:0.00802)0.895.37:0.01616,UltRu491:0.01135)0.769.20
:5.4E-
4,(((Ult24918:0.05229,(UltCl318:0.0138,UltCl320:0.03327)0.852.26:0.01913)
0.522.1:0.02196,((UltCl325:0.01454,UltCl326:0.04598)0.840.21:0.02011,(Ult
Cl322:0.01147,(UltCl323:0.01564,UltCl324:0.02845)0.906.40:0.0169)0.886.31
:0.01943)0.312.5:0.01389)0.940.19:0.02858,(Ult23543:0.0326,((Ult23598:0.0
1499,UltCl321:0.02925)0.904.23:0.01329,(((Ult23546:0.00745,(Ult23540:0.00
369,UltrdLo9:0.00372)0.899.23:0.00749)0.963.26:5.3E-
4,(OphioYy4:0.01971,(Ult23544:5.4E-
4,Ult23545:0.04025)0.914.21:0.02358)0.880.33:0.01131)0.895.38:0.01062,(((
((Ult23542:0.02412,(OphioYy5:0.03867,Ult23541:0.00476)0.479.5:0.00487)0.7

83.19:0.00761,((UncUn193:0.01964,((UltCl310:0.01565,UltCl311:0.02357)0.79
7.19:0.0079,(Otu00274:5.4E-
4,((FasSangu:0.00489,Ult23562:0.01025)0.999.35:0.06293,(UltCl309:0.01329,
(Try00029:0.08466,AtPYy143:0.02289)0.487.7:0.00819)0.927.25:0.02826)0.743
.22:0.01313)0.772.25:0.00368)0.918.25:0.01933)0.858.22:0.01186,CsrTherm:0
.01547)0.752.18:0.00378)0.869.25:0.01289,((((CsrSuffl:0.00366,(CsrCell2:5
.4E-4,CsrPapyr:0.0074)0.000.234:0.0037)1.000.133:5.3E-
4,(UltFir55:0.01506,(Ult23530:0.01141,CsrTermi:5.4E-
4)0.951.26:0.01126)0.131.1:5.4E-4)0.890.32:0.00728,Otu00468:5.4E-
4)0.924.22:0.01131,Ult23551:0.00773)0.978.11:5.4E-
4)0.763.26:0.01098,(UltCl308:0.01877,(((UltCl312:0.01554,CsrClari:0.01568
)0.780.20:0.00374,(CsrTher2:0.00376,(CsrStram:5.4E-
4,((AcvCellu:0.00374,CsrAldri:0.01514)0.777.27:0.00374,CsrAlkal:0.01525)0
.802.18:0.00368)0.748.20:0.0038)0.750.14:0.00371)0.889.21:0.01115,Ult2357
0:0.03645)0.775.18:0.00598)0.756.29:0.0062)0.919.29:0.01148,(BcrCell3:0.0
0786,(Ult23550:0.007,Ult23549:0.01976)1.000.134:0.04553)0.846.23:0.00767)
0.741.13:0.00436)0.799.23:0.00787)0.726.7:0.00571)0.837.17:0.01247)0.744.
17:0.00378)0.406.3:0.00754,((((((Ult23574:0.02002,(UltRu489:0.04176,Ult23
572:0.03676)0.506.5:0.01766)0.870.19:0.02004,(((Ult23597:0.00353,Ult23596
:0.01153)0.910.42:0.01138,(((Ult23590:0.0074,Ult23593:5.5E-
4)0.584.5:5.4E-
4,((Ult23589:0.0181,Ult23592:0.0222)0.944.20:0.01817,(UltB7916:5.4E-
4,Ult23588:0.03114)0.757.15:0.00369)0.880.34:0.00747)0.942.26:5.4E-
4,(UltB7224:0.08787,(Ult23584:0.0,Ult23582:0.0):5.5E-
4)0.956.24:0.01883)0.336.2:0.00794)0.838.22:0.01342,((Ult23586:0.04306,((
Ult19249:0.01587,Ult20623:0.0319)0.885.23:0.01368,(Ult23580:0.02371,(Ult2
3576:0.00374,((Ult18293:0.0,Ult23578:0.0,Ult23583:0.0,Ult23579:0.0):5.5E-
4,(Ult23581:0.00374,Ult23577:0.01128)0.926.28:5.5E-4)0.913.26:5.5E-
4)0.769.21:5.4E-
4)0.761.25:0.00141)0.714.8:0.00634)0.803.12:0.00765,Ult23591:0.01802)0.83
8.23:0.01251)0.950.24:0.02427)0.874.31:0.01179,((Ult23594:5.4E-
4,Ult23595:0.03975)0.905.23:0.01096,(Ult23618:0.0169,(UltRu490:0.01844,Ul
tCl313:0.02239)0.214.2:0.00341)0.833.21:0.0093)0.853.19:0.00869)0.853.20:
0.01113,((Ult23619:0.05121,((Ult25582:0.02305,Ult25581:0.00843)0.994.25:0
.07722,(Ult21911:0.04444,LtpTherm:0.0272)0.409.1:0.02175)0.901.36:0.0295)
0.783.20:0.01508,(((UltFir57:0.02118,UltCl316:0.05439)0.967.19:0.03626,((
((Ult23104:0.0351,((Ult23498:0.00414,(UltCl293:0.06307,(Ult12224:0.03099,
((((UltB7985:0.09691,((Cuiiii26:0.01487,(((((UltCl296:0.07207,Ult23484:0.
01557)0.863.24:0.02014,UltCl294:0.04643)0.792.26:0.01407,UltCl297:0.04933
)0.762.22:0.01306,((UltLac49:0.06463,((UltCl298:0.04154,(Cuiiii27:0.00879
,(Ult23485:0.00827,OphiYyy5:0.07724)0.936.28:0.02624)0.367.3:0.01387)0.42
9.2:0.02469,UltFir54:0.08381)0.185.4:0.01623)0.874.32:0.02346,(UltCl295:0
.0521,UltLac50:0.03504)0.285.4:0.00998)0.867.26:0.02875)0.907.29:0.0222,(
(AtPYy132:0.0,AtPYy141:0.0):0.02457,Ult23483:0.04838)0.436.5:0.01679)0.44
5.4:0.00599)0.999.36:0.07301,(Ult20738:0.01807,Ult23453:0.02187)0.899.24:
0.02412)0.849.23:0.02318)0.309.2:0.00323,Ult23504:0.10262)0.825.16:0.0195
6,((((((Ult23488:0.01494,Ult22791:0.03715)0.901.37:0.01489,((Ult23499:0.0
2417,Ult22600:0.03233)0.867.27:0.01629,(((Ult23492:0.07798,((Ult23496:0.0
395,Ult23330:0.0528)0.483.5:0.01788,UltRu476:0.04664)0.425.3:0.00468)0.18
2.3:0.00398,(Ult23495:0.03107,((UltB7236:0.00464,UltRu477:0.0104)0.877.37
:0.00775,(Ult23494:0.02811,Ult23493:0.01391)0.573.3:5.5E-
4)0.927.26:0.01574)0.828.17:0.01116)0.948.38:0.02484,(Ult23478:0.01373,((
((UltB7234:0.03169,(Ult21711:0.01806,Ult23502:0.01275)0.440.3:0.01115)0.8
57.30:0.01108,(Ult23490:0.02629,Ult23523:0.02061)0.829.16:0.00761)0.885.2

4:0.01596,((Ult23481:0.00366,(Ult23480:0.00765,(Ult23479:5.4E-
4,Ult23456:0.11337)0.907.30:0.01916)0.947.31:0.01546)0.962.34:0.01565,((U
lt23462:0.04602,(Ult23461:0.02853,(Ult23463:0.02658,Ult23464:5.1E-
4)0.949.27:0.016)0.872.35:5.4E-
4)0.970.25:0.02104,(Ult23491:0.02211,Ult23465:0.11394)0.906.41:0.01661)0.
589.7:5.5E-4)0.924.23:0.01435)0.466.7:5.4E-
4,(Ult23466:0.02717,Ult26398:0.04086)0.330.3:0.00521)0.781.21:0.00786)0.7
95.22:0.00717)0.729.9:0.00757)0.366.1:0.00784)0.819.20:0.00921,Ult23500:0
.07397)0.803.13:0.00677,((((Ult23452:0.01827,(Ult12225:0.00912,((Ult23449
:0.0,Ult23450:0.0):0.00868,Ult23451:0.03515)0.957.20:0.02642)0.941.24:0.0
4166)0.887.29:0.02817,Ult23501:0.04786)0.954.24:0.0337,((Ult23302:0.03408
,(Ult23486:0.07434,(Ult23455:0.07599,Ult23454:0.00169)0.212.4:0.01826)0.7
86.23:0.01226)0.975.21:0.03155,((Ult23460:0.03987,(((UltB7235:0.03566,Ult
23459:0.00786)0.506.6:0.00365,Ult23814:0.04915)0.940.20:0.01565,UltRu475:
5.5E-4)0.881.31:0.0074)0.786.24:0.00405,Ult23474:0.04383)0.690.10:5.4E-
4)0.643.2:0.0105)0.863.25:5.3E-
4,(Ult23476:0.00762,Ult23475:0.00735)0.992.22:0.02767)0.795.23:0.00393)0.
773.23:0.00375,(Ult23470:0.04471,((((Ult23468:0.04674,Ult23472:0.00557)0.
929.23:0.02884,(Ult23471:0.01835,Ult23469:0.03151)0.910.43:0.02162)0.881.
32:0.01902,Ult23477:0.02952)0.865.29:0.01308,Ult23473:0.00707)0.875.28:0.
0115)0.942.27:0.01806)0.750.15:0.00369,Ult23458:0.01523)0.883.34:0.00972)
0.753.18:0.00535,(((Ult23467:0.02925,Ult23482:0.0715)0.748.21:0.02109,(P0
114570:0.02503,(Ult23487:0.0,Otu00329:0.0):0.05375)0.718.11:0.00815)0.754
.21:0.00817,Ult23489:0.06125)0.397.5:0.01249)0.786.25:0.00467)0.866.24:0.
01624)0.877.38:0.02201)0.930.19:0.01672,Ult23497:0.00949)0.955.23:0.02301
)0.878.28:0.01375,(Ult23107:0.00369,UltRu451:5.5E-
4)0.928.23:0.01667)0.947.32:0.01972,(UltB7239:0.07965,((((((Ult21783:0.01
808,Ult23352:0.04752)0.859.28:0.01734,Ult23345:0.00405)0.818.11:0.00692,(
((Ult23333:0.01519,(((Ult20606:0.0,Ult23371:0.0):5.5E-
4,(Ult23343:0.00369,((((Ult23364:0.00756,(UltRu464:0.00369,Ult23365:5.5E-
4)0.944.21:5.5E-4)0.984.14:0.0113,(UltRu465:0.00752,Ult23332:5.5E-
4)0.886.32:5.5E-
4)0.804.16:0.00372,((Ult20604:0.01537,(Ult23369:0.03671,UltRu467:0.02838)
0.462.8:0.00771)0.879.23:0.01462,((Ult23358:0.00522,(Ult20605:0.01902,Ult
23359:5.5E-
4)0.887.30:0.01389)0.771.25:0.01187,(Ult20603:0.05199,Ult23336:5.4E-
4)0.774.35:0.00885)0.800.12:0.0088)0.789.15:0.0022)0.786.26:0.00372,((Ult
23334:5.5E-4,((((((Ult23335:5.4E-
4,Ult25422:0.00744)0.897.18:0.00744,(Ult23360:0.0037,Ult23361:0.00742)0.4
76.6:5.4E-4)0.844.27:0.00367,(Ult23368:0.0,Ult23354:0.0):5.3E-
4)0.116.4:0.00349,(Ult21440:0.0039,Ult23366:0.01126)0.802.19:0.01146)1.00
0.135:5.3E-4,Ult23337:5.5E-
4)0.936.29:0.00738,(((Ult23084:0.0,Ult23355:0.0):0.0077,UltB7218:0.04018)
0.801.18:0.00355,Ult23367:0.0075)0.602.2:5.5E-4)0.461.3:5.5E-
4,(UltRu466:0.0,UltRu461:0.0):5.5E-4)0.459.4:5.4E-4)0.363.4:5.4E-
4,Ult23370:0.01123)0.278.2:5.4E-4)0.866.25:0.00375)0.445.5:5.5E-
4)0.129.3:5.5E-4,(Ult23362:0.0,Ult23363:0.0):0.00369)0.903.26:5.4E-
4)0.996.15:0.02721,((Ult23348:0.0,Ult23347:0.0):5.4E-
4,(Ult22433:0.03618,(Ult23350:5.4E-
4,Ult23349:0.01125)0.475.2:0.00367)0.995.26:5.4E-4)0.341.5:5.4E-
4)0.863.26:0.00394,(Ult23346:0.00362,((Ult23374:0.02489,(Ult23373:0.02348
,(Ult23338:0.04281,(Ult23339:0.01121,(Ult23340:0.0,Ult23341:0.0):5.5E-
4)0.892.23:5.4E-
4)0.900.27:0.01562)0.816.16:0.00698)0.706.6:0.00395,(UltRu462:0.01444,(Ul

t23356:0.00786,((UltB7243:0.04374,(Ult23372:0.00389,(UltRu463:0.00778,Ult
23344:0.01967)0.861.29:0.00724)0.746.16:0.00311)0.934.24:0.01654,Ult23357
:0.01954)0.759.24:0.00326)0.861.30:0.00971)0.874.33:0.01003)0.999.37:5.3E
-4)0.882.23:0.00779)0.759.25:5.4E-
4)0.000.235:0.00577,((UltCl269:0.00775,((((UltFir49:0.00831,UltCl268:0.01
885)0.960.25:0.01661,(UltFir48:0.01104,Otu00421:0.01378)0.632.4:5.4E-
4)0.768.12:0.00397,Otu00330:0.01288)0.998.25:0.04059,UltCl270:0.00246)0.8
82.24:0.01777)0.983.16:0.0516,(UltB7918:0.08052,Ult20641:0.0519)0.854.15:
0.0232)0.956.25:0.04465)0.855.19:0.01305,((((((Ult23316:0.03392,(Ult2295
8:5.4E-
4,Ult23291:0.02729)0.972.22:0.0365)0.680.4:0.01974,UltCl305:0.04891)0.887
.31:0.01771,Ult23512:0.01443)0.951.27:0.02902,PahggY74:0.02816)0.920.29:0
.02297,((((AtPYy133:0.0072,(AtPYy135:5.4E-
4,((AtPYy131:0.25314,AtPYy136:0.50563)0.994.26:0.27504,(AtPYy134:0.16865,
AtPYy137:0.03155)0.786.27:0.01977)0.878.29:0.02919)0.956.26:0.01496)0.683
.8:5.4E-
4,AtPYy130:0.00372)0.939.25:0.01228,(Ult23319:0.02684,AtPYyyy5:0.02343)0.
841.22:0.00925)0.459.5:0.00713,(((((Ult20609:0.02695,Ult23389:0.00376)0.8
60.18:5.3E-4,((Ult23391:0.15084,Ult23392:5.4E-
4)0.588.5:0.00384,(Ult20607:0.01908,Ult23390:0.00382)0.774.36:0.00333)0.6
90.11:0.00755)0.929.24:0.01523,(Ult22676:0.01126,(UltB7219:0.00369,Ult233
94:0.01124)0.770.26:0.00392)0.753.19:0.00364)0.887.32:0.01099,(((UltCl274
:5.3E-
4,((Ult23315:0.02642,(Bfhggg62:0.0179,UltLac48:0.02169)0.769.22:0.00446)0
.861.31:0.01211,UltCl276:0.0187)0.843.19:0.01222)0.905.24:0.00952,UltCl27
5:0.00174)0.935.30:0.01193,(AtPyyyy8:0.04329,(UltLac47:0.01026,Ult23318:0
.02145)0.923.31:0.01251)0.845.29:5.3E-4)0.913.27:0.009)0.813.12:5.4E-
4,(((UltRu469:0.0149,(Bfhggg61:0.00756,EchggY15:0.01537)0.878.30:5.4E-
4)0.937.23:0.01481,(Bfhggg65:0.02224,(S0114422:0.00369,(Pshggg57:5.5E-
4,P0114542:0.00743)0.922.26:5.5E-
4)0.788.21:0.00821)0.107.7:0.0081)0.952.26:5.4E-
4,Ult23385:0.01931)0.945.29:0.01136)0.606.3:5.3E-
4)0.736.13:0.00163,((((((AtPYy138:0.29035,AtPYy139:5.5E-
4)0.995.27:0.03613,((AtPYy140:0.01837,(Ult23311:0.01796,((Ult23312:0.0339
1,(Ult23310:5.3E-
4,Ult23313:0.02725)0.745.24:0.01487)0.950.25:0.02714,Ult23314:0.01614)0.7
00.11:0.0064)0.889.22:0.01949)0.915.24:0.02224,(Ult23507:0.01158,(Tryy009
9:0.00773,Tryy0100:5.5E-4)0.867.28:0.0111)0.997.26:0.04021)0.693.5:5.3E-
4)0.954.25:0.01694,((((Bfhggg64:5.5E-
4,UltCl277:0.00725)0.746.17:0.00778,((Ult23393:0.02669,(Ult23387:0.02289,
Ult23386:0.02131)0.814.15:0.01301)0.839.19:0.01701,PahggY76:0.00931)0.779
.21:0.00633)0.878.31:0.01444,((PahggY75:5.4E-4,((Ult23401:5.5E-
4,((Ult23403:0.0,Ult23404:0.0):0.0037,Ult23402:0.01117)0.922.27:5.5E-
4)0.780.21:5.4E-4,(((Ult23426:0.0037,Ult23432:5.5E-
4)0.944.22:0.01915,(BuyPulli:0.00322,(((((Ult23408:0.00375,(Ult23407:5.5E
-4,(((Ult23409:0.0,Ult23413:0.0,Ult23414:0.0):5.5E-
4,(Ult23412:0.04364,(Ult23410:5.5E-
4,Ult23411:0.01126)0.917.23:0.00742)0.927.27:5.3E-4)0.959.33:5.5E-
4,((Ult23418:5.5E-4,(Ult23417:0.00368,HumanG14:0.00371)0.331.2:5.5E-
4)0.919.30:5.5E-4,((Ult23416:5.5E-4,(UltRu472:5.5E-
4,((Ult18273:0.0,Ult23415:0.0,Ult23420:0.0,Ult23424:0.0,Ult23325:0.0):5.5
E-4,(Ult23421:0.00369,(Ult23425:0.00369,Ult23419:0.00369)0.263.2:5.5E-
4)0.000.236:5.5E-4)0.562.4:5.5E-4)0.220.3:5.5E-4)0.612.5:5.4E-
4,(Ult20201:0.00743,(Ult22153:0.03468,Ult23422:5.3E-

4)0.786.28:0.00368)0.770.27:0.00368)0.844.28:0.00366)0.998.26:0.02269)0.8
07.12:0.00368)0.880.35:0.00742)0.952.27:0.01503,(BacNL311:0.0,Ult23406:0.
0):5.5E-
4)0.827.18:0.02046,(Ult23429:0.01796,((Ult23427:0.01129,Ult23428:5.4E-
4)0.994.27:0.03055,((Ult23431:0.0075,(Ult23430:0.00369,((Ult23423:0.0,Ult
23433:0.0):5.5E-
4,((Ult18990:0.00359,Ult19048:0.0113)0.935.31:0.0113,UltB7221:0.01883)0.7
18.12:5.5E-4)0.916.32:5.4E-4)0.891.28:5.4E-
4)0.741.14:0.00312,Ult26966:0.00807)0.811.15:0.00952)0.829.17:0.01149)0.8
12.12:0.00805)0.770.28:0.01841,(UltRu473:0.03615,BacNL312:5.5E-
4)0.908.26:0.01946)0.786.29:0.00852,UltCl281:5.5E-4)0.939.26:5.5E-
4)0.769.23:0.01519)0.937.24:0.01634,Ult23405:0.00745)0.314.2:5.4E-
4)1.000.136:0.03114)0.792.27:0.00406,Cuiiii25:0.02741)0.631.2:0.00109)0.9
69.20:0.0199,(Ult23317:0.00366,((Bfhggg59:0.01126,Bfhggg58:5.4E-
4)0.893.33:0.00746,(((((UltCl249:0.01505,Otu00558:5.4E-
4)0.996.16:0.03995,((Ult23122:0.01923,Ult23029:0.00377)0.898.35:0.00385,(
(((Ult23027:0.0,Ult23028:0.0,Ult23033:0.0,Ult23034:0.0):5.5E-
4,(Ult24429:0.00369,Ult23032:0.00369)0.857.31:5.5E-4)0.912.34:5.5E-
4,(Ult23031:0.07314,Ult17909:0.02271)0.549.4:5.4E-4)0.838.24:5.3E-
4,UltB7222:0.07026)0.914.22:0.01388)0.854.16:0.00416)0.244.2:5.4E-
4,(Ult23038:0.01145,(Ult23039:0.01408,Ult23041:0.02749)0.861.32:0.00965)0
.869.26:0.00764)0.863.27:0.00729,(((CsrVirid:0.01119,(Ult23037:0.01516,(U
lt22822:0.0,Ult23026:0.0):5.4E-4)0.871.27:5.5E-
4)0.256.2:0.00369,(Ult23025:0.00383,UltRu446:0.03568)0.787.25:0.00356)0.9
04.24:5.4E-4,Ult23040:0.02313)0.929.25:5.4E-
4)0.940.21:0.01099,(((PahggY71:0.08336,(UltCl250:0.00585,UncUn189:0.01409
)0.842.28:0.01368)0.895.39:0.01699,UltRu447:0.00381)0.758.26:0.00352,Ult2
3030:0.01545)0.487.8:0.01534)0.957.21:5.3E-
4)0.981.16:0.01889)0.929.26:0.01714)0.773.24:5.3E-
4)0.834.16:0.00791,(PapCinna:0.015,((((Ult23305:0.04491,(UltCl301:0.05345
,UltCl302:0.01028)0.454.4:0.0047)0.961.27:0.02401,((Ult23508:0.02072,(Ult
23563:0.05731,(UltRu480:0.03584,Ult23509:0.01645)0.904.25:0.01772)0.766.1
6:0.00786)0.983.17:0.02715,(((Ult23304:0.01364,(Ult23303:0.05633,UltCl259
:0.01435)0.896.19:0.0204)0.912.35:0.01597,(UltCl300:0.02768,(UltCl299:0.0
4881,(Ult23510:0.04709,Pshggg58:0.03654)0.723.13:0.01532)0.817.10:0.01187
)0.795.24:0.02191)0.440.4:9.1E-
4,(((((BacNL315:0.00365,(BacNL313:0.00364,(BacNL318:5.5E-
4,BacNL320:0.00367)0.887.33:5.5E-4)0.914.23:0.0073)0.718.13:5.5E-
4,(BacNL319:5.5E-4,BacNL314:0.0266)0.237.5:5.4E-
4)0.972.23:0.01863,BacNL317:0.00384)0.763.27:0.00701,BacNL316:0.01288)0.9
41.25:0.02229,(Ult23442:0.04333,(Ult23443:0.02512,(((PahggY78:5.4E-
4,(Bfhggg66:0.02696,UltRu474:0.01407)0.910.44:0.01751)0.981.17:0.02864,((
Ult23434:0.00378,Ult23435:0.01523)0.869.27:0.01589,(Bfhggg67:0.02656,(Ult
23436:5.5E-
4,Ult23441:0.00369)0.777.28:0.006)0.897.19:0.02038)0.910.45:0.01979)0.849
.24:0.01218,(Ult23440:0.02423,(Ult23439:0.01463,(Ult23437:0.00368,(Ult206
10:5.5E-4,Ult23438:0.00371)0.519.3:5.4E-
4)0.913.28:0.01252)0.850.29:0.01309)0.736.14:0.00596)0.769.24:0.00696)0.9
18.26:0.01917)0.722.4:0.00498)0.891.29:0.00812)0.775.19:0.00417)0.783.21:
0.00674)0.886.33:0.01377,(UltCl306:0.04387,((UltCl264:0.02779,PahggY80:0.
02332)0.954.26:0.02034,(PahggY72:5.4E-
4,(Ult23249:0.00773,Ult23306:0.02288)0.948.39:0.0267)0.723.14:0.01518)0.7
61.26:0.00339)0.878.32:0.00753)0.624.7:5.4E-
4,((Bfhggg63:0.01915,Ult23388:0.02012)0.401.3:0.00358,(PahggY77:0.0216,Ul

tCl279:0.01203)0.871.28:0.0121)0.782.25:0.00426)0.862.25:5.4E-
4)0.946.22:0.01717)0.902.35:0.01452,(P0114562:0.02352,UltCl278:0.01146)0.
338.3:5.5E-
4)0.895.40:0.01694,((EchggY16:0.00734,UltRu470:0.02749)0.696.2:0.00393,Ul
tCl280:5.5E-4)0.970.26:0.01118)0.000.237:5.4E-
4)0.847.27:0.00498)0.000.238:5.4E-
4,(Tryy0107:0.04164,(UltCl288:0.00879,(((UltCl286:0.04287,(((UltCl291:5.5
E-
4,((UltFir52:0.01537,UltFir53:0.01105)0.889.23:0.01123,(UltCl289:0.00756,
UltCl290:0.02697)0.859.29:5.4E-
4)0.998.27:0.03474)0.954.27:0.00737,UltCl292:5.4E-
4)0.947.33:0.02524,((UltCl285:5.5E-4,(UltFir50:5.4E-
4,UltFir51:0.01124)0.957.22:0.0074)0.976.15:0.04571,(UltCl283:0.01503,(Ul
tCl282:0.02626,UltCl284:0.01314)0.804.17:0.01198)0.740.19:0.01289)0.997.2
7:0.08464)0.626.5:0.00425)0.946.23:0.03231,Ult23447:0.04028)0.058.3:0.010
07,UltCl287:0.05455)0.985.26:0.04384)0.949.28:0.03098)0.959.34:0.02904)0.
378.6:0.00375,(((((Ult22993:5.5E-
4,Ult22992:0.0037)0.842.29:0.00357,(UncUn190:0.0503,Ult23511:0.04642)0.92
2.28:0.02372)0.295.6:5.4E-4,Ult22994:5.5E-
4)0.998.28:0.03359,(UltRu452:0.01284,(((Ult23398:0.02695,((((Ult23115:0.0
3481,Ult23124:0.00577)0.631.3:0.02204,(Ult23035:5.3E-
4,(Ult23135:0.02305,Ult23036:5.5E-4)0.906.42:0.00743)1.000.137:5.4E-
4)0.914.24:0.00733,(Ult23098:0.02243,((Ult23096:0.0,Ult23097:0.0,Ult23123
:0.0):5.5E-4,Ult31859:0.03489)0.087.4:5.4E-
4)0.909.23:0.00731)0.400.4:5.3E-
4,((((UltCl253:0.0,Ult23105:0.0):0.01759,((Ult23126:0.0,Ult23137:0.0):5.5
E-
4,Ult23136:0.00742)0.896.20:0.01306)0.813.13:0.00386,Ult23106:0.00374)0.9
42.28:0.00495,(((((((Ult23077:0.01117,Ult22680:0.00371)0.772.26:0.00377,(
Ult23091:0.04338,((Ult23087:0.0,EubPlaut:0.0,Ult23088:0.0,Ult23089:0.0,Ul
t23090:0.0,Ult23092:0.0):5.5E-4,BacNL310:0.00368)0.999.38:5.4E-
4)0.977.17:0.01511)0.916.33:0.01137,Ult23457:0.00753)0.752.19:0.00367,Ult
23125:5.5E-
4)0.962.35:0.01533,(Ult23093:0.00371,UltCl254:0.02721)0.771.26:0.00358)0.
953.21:0.01516,(((((Ult23078:0.00755,Ult23103:0.01142)0.904.26:0.00457,((
Ult23086:5.5E-4,(BcrCapil:5.5E-
4,Ult23095:0.00369)0.866.26:0.0037)0.821.18:5.4E-
4,((Ult23109:0.00367,(Ult23118:5.5E-4,Ult22973:5.5E-4)0.332.2:5.5E-
4)0.978.12:0.00186,((UltCl255:0.0,Ult23119:0.0):5.5E-
4,Ult23108:0.03031)0.966.31:0.00195)0.988.27:0.01904)0.913.29:0.00417)0.9
16.34:0.00974,Ult23943:0.00372)0.889.24:0.00737,((Ult20620:5.4E-
4,Ult23094:0.01109)0.960.26:0.01524,((Ult23813:0.01127,(Ult23075:5.4E-
4,(Ult23074:0.01066,((Ult23053:5.4E-4,(Ult23114:0.00369,Ult23110:5.5E-
4)0.930.20:0.00746)0.854.17:0.00366,((Ult23058:0.00369,((UltB7255:0.0,Ult
23052:0.0,Ult23051:0.0,Ult23056:0.0,Ult23060:0.0,CstBact7:0.0,Ult23061:0.
0,Ult23072:0.0,Ult23073:0.0,UltCl251:0.0,UltCl252:0.0):5.5E-
4,(Ult23057:0.00369,Ult23059:0.00741)0.706.7:5.5E-4)0.000.239:5.5E-
4)0.739.20:5.5E-
4,(Ult23007:0.02671,(Ult23064:0.00776,(Ult23066:0.02689,(Ult23065:5.4E-
4,((Ult23067:0.00369,(Ult23069:0.0,Ult23070:0.0,Ult23071:0.0):5.5E-
4)0.929.27:0.00701,((Ult23062:0.0,Ult23063:0.0):0.00372,Ult23068:5.5E-
4)0.920.30:0.00209)0.898.36:0.0021)0.736.15:5.4E-
4)0.772.27:0.0034)0.976.16:0.01545)0.917.24:5.5E-4)0.974.10:5.4E-
4)0.810.16:0.0061)0.783.22:0.00598)0.782.26:0.0037)0.816.17:0.00363,((Ult

23080:0.0,Ult23081:0.0):0.00362,(Ult23113:0.00362,(Ult23076:0.00369,Ult23
112:5.5E-4)1.000.138:5.4E-4)0.573.4:0.00753)0.979.20:5.3E-4)0.678.8:5.4E-
4)0.868.20:0.00365)0.422.3:5.4E-
4,(CsrSpe14:0.00369,(Ult23079:0.00373,Ult23042:0.00753)0.805.19:0.00372)0
.960.27:5.4E-4)0.978.13:5.4E-4)0.908.27:0.01482,(Ult23043:5.5E-
4,((Ult23099:0.0,Ult23100:0.0):0.0076,(Ult23101:0.0,Ult23102:0.0,Ult23111
:0.0,Ult23127:0.0):0.00757)0.882.25:0.00744)0.876.27:0.00764)0.940.22:0.0
0471)0.942.29:0.00485)0.863.28:5.5E-4)0.832.14:5.5E-
4,((Ult18257:0.04659,(Ult22599:0.02173,(Ult23444:5.3E-
4,(Ult23445:0.02663,(Ult18271:0.01111,Ult23446:0.00369)0.317.6:5.5E-
4)0.947.34:0.00735)0.899.25:0.00927)0.758.27:0.00341)0.929.28:0.01656,Ult
Pse49:0.00336)0.901.38:0.01207)0.888.36:0.00864,(Ult22967:0.02286,(Ult213
97:0.01232,((Ult23129:5.5E-4,Ult23130:0.00369)0.648.5:5.5E-
4,((Ult23128:0.0,Ult23131:0.0,Ult23132:0.0,Ult23134:0.0):5.5E-
4,Ult23133:0.04274)0.906.43:0.00742)0.751.11:0.00273)0.978.14:0.02604)0.8
56.17:0.0092)0.866.27:0.00763)0.848.23:0.00738)0.854.18:5.3E-
4,(((Ult23047:0.01131,(Ult23045:0.00368,Ult23046:0.01137)0.847.28:0.00762
)0.821.19:0.00826,((Ult23044:5.5E-4,(UltRu449:5.4E-
4,UltRu448:0.01126)0.802.20:0.00708)0.885.25:0.02001,UltRu450:0.0309)0.98
8.28:0.04224)0.966.32:0.02808,(((Ult23018:5.5E-4,(Ult23120:5.4E-
4,(Ult21288:0.00742,((Ult23009:0.0,Ult23011:0.0,Ult23014:0.0,Ult23015:0.0
):5.5E-4,Ult23016:0.0037)0.843.20:5.5E-
4)0.995.28:0.0233)0.896.21:0.01092)0.958.27:0.02324,(Ult23121:0.0343,UltC
l256:0.00661)0.867.29:0.00977)0.934.25:5.4E-
4,(Ult23017:0.01536,(Ult22971:0.00748,((((Ult22960:0.00369,Ult22976:0.003
69)0.689.8:5.5E-4,((Ult22974:0.01119,Ult22959:0.00369)0.806.10:5.5E-
4,(Ult22963:0.0074,Ult22996:0.00742)0.674.4:5.5E-4)1.000.139:5.5E-
4)0.000.240:5.5E-
4,(Ult22981:0.0,Ult22989:0.0,Ult22990:0.0,Ult22995:0.0):5.5E-
4)0.990.21:5.4E-
4,(UltCl248:0.00374,(((((Ult23012:0.0074,Ult22968:0.00369)0.905.25:5.5E-
4,(Ult23117:0.0,Ult23116:0.0,Ult22969:0.0,Ult22985:0.0,Ult22986:0.0):5.5E
-4)0.896.22:5.5E-4,(Ult23054:0.06067,Ult22970:0.01472)0.884.25:5.5E-
4)0.853.21:5.5E-4,((Ult22962:0.00217,(Ult22977:5.4E-
4,((Ult22982:0.02273,(Ult22983:0.0,Ult22984:0.0):5.5E-4)0.927.28:5.5E-
4,(((Ult23048:5.4E-
4,Ult23049:0.00366)0.454.5:0.00371,(((((Ult23023:0.08309,Ult23022:0.0304)
0.954.28:5.4E-
4,((Ult23019:0.02344,Ult23020:0.02344)0.817.11:0.00689,UltB7191:0.00742)0
.928.24:5.4E-4)0.939.27:5.5E-4,Ult23024:5.5E-4)0.000.241:5.5E-
4,(Ult23021:0.00369,Ult23050:0.00743)0.863.29:5.5E-4)0.838.25:5.5E-
4,Ult23085:0.01481)0.998.29:5.3E-
4)0.886.34:0.00198,(((Ult22975:0.0,Ult22978:0.0,Ult22987:0.0,Ult22988:0.0
,Ult22991:0.0,Ult22998:0.0,Ult22999:0.0,Ult23010:0.0):5.5E-
4,(Ult22972:0.03501,UltCl247:0.00718)0.915.25:5.4E-4)0.801.19:5.5E-
4,(Ult23008:5.5E-4,(Ult22979:5.5E-
4,Ult22980:0.00369)0.839.20:0.00369)0.850.30:0.0037)0.924.24:0.00211)0.93
9.28:0.00662)0.897.20:0.01107)0.920.31:0.0069)0.911.32:0.00195,Ult22961:0
.03443)0.459.6:5.5E-4)0.949.29:0.00733,((Ult22965:0.0,Ult22997:0.0):5.5E-
4,(Ult23013:0.0037,Ult22964:0.00369)0.710.5:5.5E-4)0.988.29:5.5E-
4)0.811.16:0.00361)0.784.17:0.00362)0.879.24:0.00733)0.881.33:0.00761)0.8
47.29:0.00692)0.000.242:0.00685)0.949.30:0.01707)0.927.29:0.01376)0.968.2
1:0.02485)0.495.3:0.01395,((Ult22224:0.08793,(Ult20608:0.05389,(UltRu483:
0.0922,((UltB9855:0.06992,Ult22770:0.00653)0.958.28:0.03492,EchggY17:0.02

24)0.567.2:0.00989)0.912.36:0.02941)0.401.4:0.0033)0.015.2:0.01357,((((((
Ult23263:0.02347,Ult18314:0.03288)0.823.15:0.00762,(Ult22548:0.04678,((Ul
t22571:0.01117,((((Ult22591:0.00741,(Ult22590:0.0228,Ult22588:0.03091)0.8
95.41:5.5E-4)0.964.36:5.5E-4,((Ult22609:5.5E-
4,((Ult22611:0.0,Ult22576:0.0):5.5E-
4,Ult22577:0.00369)0.928.25:0.00746)0.857.32:0.00369,Ult22578:0.00369)0.6
36.2:5.5E-4)0.000.243:5.5E-
4,((Ult22607:0.0,Ult22574:0.0,Ult22575:0.0,Ult22572:0.0,Ult22573:0.0,Ult2
2579:0.0):5.5E-
4,(Ult23323:0.00369,(Ult22582:0.00369,(Ult22580:0.0,Ult22581:0.0):0.01122
)0.835.23:5.5E-4)0.765.16:5.5E-4)0.000.244:5.5E-4)0.882.26:5.5E-
4,Ult22592:0.00368)0.554.3:5.4E-
4)0.764.22:0.00368,Ult18315:0.0037)0.843.21:0.00703)0.920.32:5.4E-
4)0.762.23:0.00542,Ult22583:0.02207)0.982.12:0.03745,(Ult22233:0.03756,((
((((((Ult22229:0.0,Ult22251:0.0):5.5E-
4,Ult22605:0.03091)0.832.15:0.00538,(Ult18259:0.00368,((EubSirae:0.0,Ult2
2253:0.0):5.5E-4,Ult22230:0.00369)0.475.3:5.5E-
4)0.804.18:0.00579)0.429.3:0.00381,Ult22243:0.00922)0.887.34:0.00952,Ult2
2242:5.5E-4)0.917.25:0.00729,(Ult22240:0.0,Ult22241:0.0):5.5E-
4)0.883.35:0.00997,(((Ult22246:0.0,Ult22254:0.0):5.5E-
4,Ult22247:0.00749)0.278.3:5.4E-
4,(Ult22244:0.00364,(Ult18258:0.02686,Ult22245:5.4E-
4)0.351.2:0.00372)0.804.19:0.00377)0.799.24:0.00543)0.778.23:0.00469,(Ult
22248:0.01272,(((Ult22235:0.0,Ult22236:0.0):5.3E-
4,(Ult25385:0.0147,((Ult18274:0.02261,Ult22237:0.00369)0.958.29:5.5E-
4,(Ult22238:0.01503,Ult22239:0.00749)0.959.35:5.5E-4)0.957.23:5.3E-
4)0.966.33:0.01102)0.918.27:5.5E-
4,(Ult22234:0.00611,(Ult22232:0.00375,Ult22231:0.01127)0.802.21:0.00592)0
.784.18:0.01048)0.735.10:0.01055)0.862.26:0.00886)0.756.30:0.00477)0.883.
36:0.01369)0.802.22:5.4E-
4,((((Ult22460:0.02982,((((Ult22466:0.0,Ult22462:0.0):5.5E-
4,(Ult22464:0.00385,UltB7329:0.00369)0.132.6:5.5E-4)0.960.28:5.4E-
4,(Ult22461:0.00376,(Ult22467:5.4E-
4,Ult22463:0.00718)0.960.29:0.01529)0.885.26:0.00727)0.936.30:0.01829,((U
lt18308:0.0037,Ult22465:0.01119)0.756.31:0.00388,(((Ult22475:0.01876,Ult2
2476:5.3E-
4)0.943.27:0.01476,(Ult22472:0.01507,UltCl206:0.01956)0.734.12:0.0112)0.7
59.26:5.5E-
4,Ult22473:0.01916)0.973.14:0.0189)0.708.7:0.00909)0.773.25:0.0091)0.762.
24:0.00711,((UltCl203:0.0164,(UltCl202:0.01474,(Cuiiiii7:0.02801,UltCl205
:0.02743)0.749.26:0.00406)0.734.13:0.00795)0.757.16:0.00466,UltCl204:0.02
111)0.897.21:0.01589)0.833.22:0.0159,((((Ult22637:0.01285,(Ult22636:0.038
43,UltRu430:0.03056)0.958.30:0.03159)0.776.18:0.01005,(Ult22589:0.04969,(
Pshggg56:0.03177,((((((((((Ult22477:0.0,Ult22478:0.0,Ult22499:0.0):5.5E-
4,((Ult22483:0.0,Ult22484:0.0):5.5E-
4,Ult18276:0.01118)0.830.22:0.00369)0.318.6:5.4E-
4,(Ult22479:0.0074,(Ult22482:5.5E-4,Ult22480:5.5E-4)0.742.15:5.5E-
4)0.855.20:0.00368)0.940.23:0.01121,((Ult22494:0.00369,(Ult22485:5.5E-
4,(Ult22491:0.00372,((Ult22510:0.0112,(Ult22489:0.01125,(Ult22490:0.00369
,Ult22500:5.5E-4)0.814.16:5.5E-4)0.842.30:0.00369)0.818.12:5.5E-
4,(Ult22481:5.5E-4,(Ult22493:0.00369,Ult22492:0.01878)0.768.13:5.5E-
4)0.112.2:5.5E-4)0.731.6:5.5E-4)0.850.31:0.00369)1.000.140:5.4E-
4)0.608.4:0.0037,(Ult22486:0.0037,Ult22487:0.00372)0.890.33:5.4E-
4)0.876.28:5.4E-4)0.941.26:0.01113,Ult22488:5.4E-

4)0.847.30:0.00756,(Ult22504:0.01128,((Ult22502:0.0,Ult22501:0.0,Ult22503
:0.0):5.5E-4,(UltCl207:0.01898,(((Ult22508:0.01923,(Ult22507:5.4E-
4,Ult22509:0.01907)0.360.6:5.5E-4)0.923.32:0.01153,(Ult22505:5.4E-
4,Ult22506:0.01124)0.743.23:0.00347)0.846.24:0.00762,Ult17776:0.01175)0.9
68.22:0.02265)0.573.5:0.00881)0.319.3:5.4E-
4)0.924.25:0.01577)0.916.35:0.01241,Ult22633:0.00796)0.752.20:0.00344,((U
lt22631:0.0074,(Ult22606:0.00369,Ult22632:5.5E-4)0.287.2:5.5E-
4)0.961.28:0.01946,Ult22612:0.01557)0.833.23:0.00686)0.406.4:5.5E-
4,UltRu428:0.02335)0.971.20:0.01936,(((((Ult22621:0.00734,((Ult18891:0.0,
Ult22602:0.0):5.5E-4,((Ult22601:0.00716,Ult22619:0.02679)0.922.29:5.5E-
4,Ult22620:0.00369)0.643.3:5.5E-4)0.823.16:5.5E-
4)0.792.28:0.00368,Ult22603:0.00369)0.988.30:0.03479,((Ult22634:0.01146,(
(Ult22617:5.4E-4,Ult22618:0.0111)0.995.29:0.03829,(Ult18339:5.5E-
4,Ult22615:0.00369)0.885.27:0.01427)0.872.36:0.01474)0.733.12:0.00313,(Ul
t22614:0.00761,(((Ult22616:0.00757,UltRu429:0.01916)0.784.19:0.0036,((Ult
22604:0.01547,UltCl215:0.01921)0.246.3:0.00372,(((((((UltCl210:0.01587,P0
114424:0.03599)0.253.4:0.0072,(UltFir41:0.0037,Otu00235:5.4E-
4)1.000.141:5.4E-4)0.924.26:0.00348,UltCl211:0.0037)0.950.26:5.4E-
4,(Ult22613:0.01854,(UltCl213:0.01969,Otu00411:0.00354)0.854.19:0.01005)0
.838.26:0.00994)0.475.4:0.00367,(UltFir40:0.00744,UltCl212:5.5E-
4)0.884.26:0.00744)0.618.2:0.00366,CuiiiiY2:0.00806)0.865.30:0.00772,UltF
ir42:0.00712)0.952.28:0.0157)0.856.18:5.4E-
4)0.863.30:0.00747,(((Ult22628:0.00369,((Ult22627:5.5E-
4,Ult22625:0.01511)0.909.24:0.0075,Ult22626:0.01525)0.624.8:5.5E-
4)0.938.22:0.01131,Ult22624:0.0037)0.798.19:0.00373,Ult22608:0.00755)0.92
9.29:0.01153)0.761.27:0.00375)0.931.27:0.01195)0.860.19:0.01165)0.910.46:
0.01653,UltB3909:0.01521)0.886.35:0.0079,((Pshggg55:0.07186,UltCl214:0.01
108)0.844.29:5.5E-
4,(Ult22630:0.03776,Ult22629:0.00198)0.970.27:0.0171)0.901.39:0.00787)0.6
10.2:5.5E-
4)0.850.32:0.01275,((Ult22635:0.0354,(((((((UncUn187:0.03656,(UltCl194:0.
00879,((UncUn186:0.0047,Ult22303:0.01081)0.954.29:0.02218,(UncUn184:0.007
41,UncUn185:5.5E-
4)0.884.27:0.01198)0.545.7:0.00781)0.790.18:0.01118)0.936.31:0.03304,(Pah
gYy68:0.02596,((Ult22307:0.05532,(Ult22306:0.02668,UltRu420:0.00487)0.828
.18:0.02048)0.979.21:0.03957,(UltCl195:0.03253,((UltCl196:0.02322,(Pshggg
53:0.02786,UltCl197:0.01206)0.923.33:0.01544)0.736.16:0.00383,Ult22305:0.
00371)0.883.37:0.0076)0.122.1:5.4E-
4)0.748.22:0.00571)0.861.33:0.01838)0.710.6:0.00989,(((((((UncUn163:0.0,U
ltRu414:0.0):5.5E-4,(Ult22211:0.01512,Ult22210:0.03097)0.396.9:5.4E-
4)0.944.23:0.01142,Ult22212:0.00364)0.768.14:0.00404,((UncUn160:0.01159,(
((EchggY14:5.4E-
4,(UltRu413:0.02901,UltRumi9:0.04109)0.783.23:0.00605)0.904.27:0.00741,Ul
tRu412:0.0076)0.748.23:5.5E-
4,Ult22208:0.01116)0.879.25:0.01081)0.366.2:0.00394,(UncUn166:0.03999,((U
ncUn164:5.3E-4,(UncUn165:5.5E-
4,(UltCl153:0.04802,UncUn168:0.00367)0.959.36:5.3E-
4)0.929.30:0.00743)0.946.24:0.01131,UncUn167:5.3E-
4)0.747.18:0.00399)0.468.4:0.00445)0.949.31:0.02266)0.759.27:0.00348,UncU
n161:0.01531)0.882.27:0.00757,((Ult22209:0.03791,UncUn162:0.06679)0.000.2
45:0.01108,(UltCl154:0.01564,((UncUn169:0.0399,P0114520:0.01347)0.931.28:
0.02217,Hmyy0019:5.4E-4)0.843.22:0.01968)0.853.22:0.01104)0.956.27:5.4E-
4)0.803.14:5.3E-
4,Bfhggg55:0.01513)0.967.20:0.02009)0.749.27:0.0065,(Ult22304:0.06651,(Ul

tCl189:0.04796,(UltCl188:0.01929,(UltCl190:0.01862,UltFir38:0.01009)0.945
.30:0.03344)0.277.4:0.00539)0.989.21:0.05849)0.919.31:0.03555)0.649.6:0.0
1829,((((((((((((Ult22101:0.01635,(((((Ult22160:5.3E-
4,(Ult18239:0.0681,Ult22167:0.02329)0.173.2:0.00696)0.992.23:0.03487,(((U
lt22142:0.06765,Ult22143:0.05862)0.733.13:0.0052,((Ult22146:5.5E-
4,Ult22152:0.01864)0.861.34:5.4E-
4,(Ult22145:0.00368,((((((Ult22132:0.00369,(Ult22182:0.00371,Ult22141:0.0
037)0.444.5:5.5E-4)0.211.5:5.5E-
4,(Ult22144:0.0188,Ult22147:0.00196)0.974.11:0.0019)0.537.3:5.5E-
4,(Ult22598:0.00201,Ult22451:0.01089)0.930.21:0.00198)0.536.4:5.5E-
4,Ult22165:0.00369)0.449.6:5.5E-
4,(Ult21937:0.00369,Ult22156:0.01116)0.585.2:5.5E-4)0.670.3:5.5E-
4,(Ult22014:0.0,Ult22123:0.0,Ult22137:0.0,Ult22136:0.0,Ult22135:0.0,Ult22
138:0.0,Ult22134:0.0,Ult22131:0.0,Ult22139:0.0,Ult22140:0.0,Ult22151:0.0,
Ult22161:0.0,Ult22168:0.0,Ult22170:0.0,Ult22179:0.0,Ult22610:0.0):5.5E-
4)0.670.4:5.5E-4)0.414.1:5.5E-4)0.717.10:5.3E-4)0.421.4:5.4E-
4,((Ult22164:0.02269,((SbdVaria:0.0,Ult22157:0.0):5.5E-
4,(Ult22178:0.00369,Ult22150:0.00369)0.711.10:5.5E-4)0.868.21:5.3E-
4)1.000.142:5.4E-
4,((Ult22129:0.00375,Ult22130:0.01496)0.952.29:0.015,(Ult22127:0.0037,((U
lt22069:0.0,Ult22126:0.0,Ult22128:0.0,Ult22181:0.0):5.5E-4,Ult22108:5.5E-
4)0.413.5:5.5E-
4)0.776.19:0.00376)0.567.3:0.00747)0.902.36:0.00352)0.864.32:0.00734)0.75
9.28:0.00367,Ult22176:5.4E-
4)0.778.24:0.00375,(Ult22155:0.00363,((Ult22154:0.00765,Ult22185:0.02791)
0.766.17:0.00359,(Ult22021:0.01534,(Ult22148:5.4E-
4,Ult22149:0.01941)0.779.22:0.00375)0.762.25:0.00375)0.771.27:0.0038)0.87
0.20:0.00759)0.907.31:0.01187,(Ult22124:5.3E-
4,((UltB7331:0.03924,Ult22053:0.00329)0.757.17:0.00389,((Ult22103:0.0231,
(Ult22102:0.00648,Ult22104:0.02207)0.415.6:0.01052)0.943.28:0.01673,((Ult
22060:5.5E-
4,(((Ult21968:0.0037,((((Ult21919:0.0,Ult22058:0.0):0.00369,(Ult21967:0.0
0369,(Ult21970:0.00369,(((((Ult18240:0.0,Ult21959:0.0,Ult21964:0.0,Ult219
66:0.0,Ult21954:0.0,Ult21955:0.0,Ult21956:0.0,Ult21971:0.0,Ult21973:0.0,U
lt21974:0.0,Ult21975:0.0,Ult21923:0.0,Ult21916:0.0,Ult21917:0.0,Ult21918:
0.0,Ult21921:0.0,Ult21926:0.0,Ult21927:0.0,Ult21928:0.0,Ult21929:0.0,Ult2
1933:0.0,Ult21934:0.0,Ult21939:0.0,Ult21944:0.0,Ult21943:0.0,Ult22001:0.0
,Ult22003:0.0,Ult21999:0.0,Ult21995:0.0,Ult22007:0.0,Ult22010:0.0,Ult2201
2:0.0,Ult22018:0.0,Ult22020:0.0,Ult22023:0.0,Ult22054:0.0,Ult22059:0.0,Ul
t22062:0.0,Ult22078:0.0,Ult22087:0.0,Ult22174:0.0):5.5E-
4,((Ult21924:0.00369,((((Ult21925:0.015,(Ult21994:0.00369,Ult22016:5.4E-
4)1.000.143:5.1E-
4)1.000.144:0.00318,(Ult22082:0.08404,Ult21969:0.00368)0.473.3:5.5E-
4)0.248.3:5.5E-4,(Ult21963:0.0,Ult22813:0.0):0.00369)0.426.7:5.5E-
4,Ult21972:0.00369)0.416.7:5.3E-4)0.000.246:5.5E-
4,Ult22079:0.00369)0.426.8:5.5E-4)0.000.247:5.5E-
4,Ult22081:0.00369)0.000.248:5.5E-4,Ult22061:0.00369)0.000.249:5.5E-
4,(Ult22015:0.0037,Ult22005:0.0037)0.787.26:0.00369)0.000.250:5.5E-
4)0.420.6:5.5E-4)0.015.3:5.5E-4)0.419.3:5.5E-
4,(Ult21920:0.00369,(Ult21951:0.00369,Ult21952:5.5E-4)0.329.7:5.5E-
4)0.849.25:0.00369)0.000.251:5.5E-4,Ult22019:0.00369)0.000.252:5.5E-
4)0.385.6:5.5E-4,Ult22063:0.00371)0.442.3:5.5E-
4,(Ult21930:0.0,Ult21941:0.0):0.00369)0.483.6:5.5E-4)0.963.27:5.4E-
4,(((Ult22043:0.0155,(((Ult22044:0.0,Ult22064:0.0,Ult22065:0.0,Ult22066:0

.0,Ult22067:0.0,Ult22068:0.0):5.4E-
4,(Ult22042:0.0037,Ult22080:0.00747)0.807.13:0.0037)0.935.32:0.00234,(Ult
22093:0.02355,(Ult22006:0.00742,((Ult21962:0.0,Ult21931:0.0,Ult22038:0.0,
Ult22039:0.0,Ult22040:0.0,Ult22041:0.0,Ult22047:0.0,Ult22088:0.0):5.5E-
4,Ult21949:5.5E-4)0.852.27:5.5E-
4)0.786.30:0.00374)0.933.35:0.00259)0.868.22:0.00219)0.938.23:0.01164,(((
(Ult21942:0.00369,Ult21981:0.00741)0.663.5:5.5E-
4,((Ult21946:0.00369,Ult21960:0.00369)0.898.37:5.5E-
4,((Ult21961:0.0,Ult21945:0.0,Ult21950:0.0,Ult21977:0.0,Ult21998:0.0,Ult2
2048:0.0,Ult22050:0.0):5.5E-
4,(Ult21978:0.01507,Ult22017:0.00369)0.558.3:5.5E-4)0.000.253:5.5E-
4)0.000.254:5.5E-4)0.810.17:5.5E-4,Ult22045:5.5E-4)0.690.12:5.5E-
4,(Ult21976:5.5E-4,UltRumi5:0.00366)0.856.19:0.00366)0.917.26:5.4E-
4)0.946.25:0.00739,((Ult22030:0.00738,(((Ult22072:0.01495,(Ult23234:0.063
44,((Ult22095:0.0,Ult22096:0.0):5.4E-4,(Ult22346:0.03871,(Ult22092:5.5E-
4,((Ult22089:0.0,Ult22090:0.0,Ult22091:0.0,Ult22097:0.0,Ult22099:0.0):5.4
E-4,(Ult22094:0.00741,Ult23729:5.5E-
4)0.157.2:0.00369)0.837.18:0.00366)0.452.4:5.3E-
4)0.918.28:0.00741)0.823.17:0.01597)0.731.7:0.00335)0.937.25:5.4E-
4,(Ult22029:5.5E-4,(Ult21932:0.0,Ult22057:0.0,Ult22071:0.0):5.5E-
4)0.352.2:5.5E-4)1.000.145:5.5E-
4,Ult21957:0.00369)0.875.29:0.00369)0.614.2:5.3E-
4,(((((((Ult22056:0.0037,Ult22086:5.5E-
4)0.842.31:0.00369,(Ult22077:0.0037,Ult21940:0.01904)0.781.22:0.00369)0.0
00.255:5.5E-
4,((Ult22033:0.0,Ult22034:0.0):0.00368,(Ult21965:0.0074,(((((Ult22008:0.0
0369,Ult22083:0.00369)0.000.256:5.5E-
4,((Ult21935:0.00369,((Ult21936:0.04284,Ult21988:0.01109)0.915.26:5.4E-
4,(Ult22032:0.01126,(Ult21958:0.01846,Ult21983:0.0052)0.878.33:0.01038)0.
757.18:0.00367)0.399.4:5.3E-4)0.000.257:5.5E-
4,(Ult22046:0.00369,(Ult22051:0.00741,Ult22052:5.5E-
4)0.882.28:0.00369)0.000.258:5.5E-4)0.475.5:5.5E-4)0.000.259:5.5E-
4,(Ult21938:0.0,Ult21947:0.0,Ult21948:0.0,Ult21979:0.0,Ult21980:0.0,Ult21
985:0.0,Ult21986:0.0,Ult21987:0.0,Ult21990:0.0,Ult21997:0.0,Ult22009:0.0,
Ult22013:0.0,Ult22022:0.0,Ult22024:0.0,Ult22025:0.0,Ult22026:0.0,Ult22035
:0.0,Ult22037:0.0,Ult22049:0.0,Ult22055:0.0,Ult22073:0.0,Ult22075:0.0,Ult
22076:0.0):5.5E-4)0.475.6:5.5E-
4,(Ult21984:0.00692,Ult22175:0.00209)0.904.28:0.00207)0.396.10:5.5E-
4,((Ult22028:0.0,Ult22070:0.0):0.00369,Ult21989:0.00744)0.688.3:5.5E-
4)0.000.260:5.5E-4)0.463.4:5.5E-4)0.000.261:5.5E-4)0.000.262:5.5E-
4,Ult21953:0.00369)0.006.4:5.5E-
4,(Ult22027:0.00743,Ult22074:0.00369)0.000.263:5.5E-4)0.065.4:5.5E-
4,(UltB6401:0.06913,(((Ult22173:0.01751,((Ult22180:0.02607,(Ult22162:5.5E
-
4,(Ult22159:0.01509,(Ult22125:0.0037,((Ult22106:0.00369,(((UltB7213:0.0,U
lt22105:0.0,Ult22109:0.0,Ult22111:0.0,Ult22112:0.0,Ult22114:0.0,Ult22115:
0.0,Ult22119:0.0,Ult22120:0.0,Ult22121:0.0,UltRumi6:0.0,Ult22133:0.0,Huma
nG12:0.0,Ult22163:0.0,Ult22171:0.0,Ult22172:0.0,Ult22177:0.0):5.5E-
4,(Ult22116:0.00372,Ult22117:0.00369)0.924.27:5.5E-4)0.000.264:5.5E-
4,(Ult22118:5.5E-4,(Ult22110:0.00371,(Ult22113:5.5E-
4,Ult22122:0.03478)0.826.18:0.00369)0.731.8:5.5E-4)0.000.265:5.5E-
4)0.799.25:5.4E-4)0.800.13:5.5E-
4,Ult22166:0.096)0.815.14:0.00367)0.779.23:0.00371)0.785.27:0.00368)0.912
.37:0.0165)0.979.22:0.02789,(((UltClos9:0.00513,Ult18242:0.02621)0.911.33

:0.01391,Ult21996:0.004)0.528.3:0.00745,Ult21993:0.004)0.847.31:0.00666)0
.740.20:0.004)0.909.25:0.01269,((Ult22084:0.00749,(Ult18241:0.00974,(Ult2
2000:0.01421,Ult22011:0.08875)0.939.29:0.02478)0.866.28:0.00946)0.883.38:
0.00744,Ult22685:5.4E-4)0.966.34:0.01492)0.880.36:5.5E-
4,Ult22036:0.02284)0.758.28:0.00346)0.801.20:0.00408)0.193.1:5.5E-
4,(UltFaeca:0.00739,Ult21982:0.0037)0.603.7:5.5E-4)0.124.1:5.5E-
4)0.974.12:5.5E-4)0.921.39:0.00734)0.619.1:5.4E-
4)0.911.34:0.01092)0.969.21:0.01926)0.523.2:0.0074)0.796.13:0.00635)0.927
.30:0.0195,(Ult22169:0.00364,Ult22183:0.00379)0.881.34:0.01326)0.884.28:0
.01771,((Ult22215:0.01835,(((AnfPento:0.0,BacNL305:0.0):5.5E-
4,Ult22213:5.5E-
4)0.962.36:0.02307,Ult22214:0.03461)0.762.26:0.00683)0.955.24:0.03182,(Ul
t22186:0.02928,(UltRumi7:5.5E-
4,Ult21991:0.06249)0.926.29:0.01391)0.832.16:0.01067)0.942.30:0.02213)0.7
71.28:0.00587,Ult22187:0.00857)0.839.21:0.0108,(Ult22204:0.03221,((Ult221
58:0.0,Ult22203:0.0):0.01577,Ult22205:0.02011)0.737.22:0.00333)0.922.30:0
.01555)0.390.2:0.01168,Ult22184:0.0309)0.250.2:5.4E-
4,((Ult22100:0.01554,(Ult22188:0.00369,Ult22189:5.5E-
4)0.852.28:0.00723)0.944.24:0.01919,Ult22197:0.01595)0.797.20:0.00758)0.7
51.12:0.00715,(((Ult22218:0.02255,(Ult22200:0.00335,Ult22195:0.01577)0.63
4.2:0.00755)0.857.33:5.4E-4,(Ult22193:0.0,Ult22194:0.0):5.3E-
4)0.852.29:5.4E-4,(Ult22217:5.4E-
4,(Ult22201:0.06093,Ult22216:0.02805)0.769.25:0.00888)0.921.40:0.01492)0.
956.28:0.0188)0.785.28:0.0058,(Ult22202:5.5E-
4,UltRumi8:0.00369)0.956.29:0.01191)0.710.7:0.00236,(Ult22593:0.09285,((U
lt22107:0.0,Ult22190:0.0,Ult22192:0.0):5.5E-4,(Ult21992:5.5E-
4,Ult22191:0.03953)0.842.32:0.00375)0.978.15:5.4E-
4)0.995.30:0.02518)0.935.33:0.0164,((Ult22196:0.01141,Ult22199:0.00759)0.
773.26:0.00595,(Ult22198:0.00984,Ult22206:0.00917)0.782.27:0.00766)0.822.
14:0.00448)0.975.22:5.4E-
4,Ult22207:0.02395)0.963.28:0.02285,((Ult22219:0.02886,Ult22220:0.06722)0
.948.40:0.04889,((Ult22002:0.02948,UltRu415:0.01684)0.290.5:0.01195,(Ult2
2221:0.02364,(Ult22222:5.4E-
4,Ult22223:0.03198)0.965.30:0.04415)0.759.29:0.02617)0.851.27:0.0276)0.95
4.30:0.04645)0.011.3:5.5E-
4)0.889.25:0.02959,(Ult22227:0.06395,((Ult22276:0.05606,(UltCl192:0.03725
,(CsrCellu:0.04684,Ult22275:0.04548)0.442.4:0.01353)0.959.37:0.04294)0.83
2.17:0.01514,EthHarbi:0.00506)0.413.6:0.01345)0.921.41:0.0386)0.755.22:0.
00468,((UltCl219:0.01345,UltFir44:0.01347)0.908.28:0.02252,(UltCl216:0.04
425,UltCl217:0.00945)0.892.24:0.03276)0.950.27:0.03006)0.881.35:0.01268)0
.816.18:0.01816,(((UltRu436:0.11729,(((Ult21289:0.01729,Ult22744:0.01717)
0.395.4:0.0091,((Ult18307:0.05325,Ult21331:0.08661)0.713.8:0.00314,(Ult23
816:0.01687,(Ult21718:0.00693,UltB7233:0.06237)0.889.26:0.01341)0.798.20:
0.0086)0.914.25:0.01631)0.912.38:0.01688,((((Ult22643:5.5E-
4,Ult22653:0.01117)0.805.20:0.00363,(((((((Ult22691:0.00369,((Ult18313:0
.0,Ult22734:0.0):5.5E-
4,Ult22690:0.064)0.942.31:0.01131)0.993.19:0.03972,(Ult22732:0.00767,Ult2
2731:0.00783)0.999.39:0.06177)0.979.23:5.3E-
4,Ult22733:0.02379)0.866.29:0.00821,((Ult22688:0.01793,Ult22664:0.04325)0
.884.29:0.01314,(((Ult22689:0.0,Ult22692:0.0):5.5E-
4,((Ult21302:0.00752,Ult22687:0.03948)0.772.28:0.00363,Ult22686:0.00369)0
.680.5:5.5E-4)0.738.10:5.5E-
4,BacMpnIs:0.00745)0.772.29:0.00386)0.757.19:0.00367)0.901.40:0.01249,(Ul
t22661:0.01655,(Ult22662:0.00767,(Ult22660:0.0037,Ult22659:5.5E-

4)0.770.29:0.00356)0.911.35:0.01467)0.685.6:0.00963)0.811.17:0.00466,Ult2
2657:0.01905)0.460.1:5.4E-
4,(((Ult22646:0.0,Ult22650:0.0):0.0154,((Ult22658:0.01182,Ult22644:0.0279
6)0.919.32:0.01477,((Ult22647:0.00767,UltCl220:0.01146)0.770.30:0.00346,(
(Ult22666:0.00369,Ult22667:5.5E-4)0.871.29:5.5E-
4,Ult22663:0.04408)0.776.20:0.00384)0.232.3:0.00191)0.622.4:0.00197)0.786
.31:0.00398,(Ult22648:0.00751,(Ult22656:0.00369,(Ult22655:5.5E-
4,(Ult22642:0.00368,Ult22649:0.01141)0.763.28:5.5E-4)0.861.35:5.5E-
4)0.899.26:0.00749)0.752.21:0.00375)0.793.18:0.00368)0.920.33:0.00742,((U
lt22652:0.00369,(Ult22645:5.5E-4,Ult22651:0.00369)1.000.146:5.5E-
4)0.477.2:0.01512,((((PahggY68:5.5E-4,P0114872:0.00746)0.952.30:5.4E-
4,(UltRu434:0.00381,PahggY69:0.02322)0.620.7:0.00744)0.969.22:0.01537,(Ul
tRu433:0.01503,UltRu432:0.0037)0.882.29:5.4E-
4)0.734.14:0.00341,UltRu431:0.01572)0.917.27:0.01556)0.923.34:5.5E-
4)1.000.147:5.5E-
4)0.269.1:0.00441,Ult22654:0.02677)0.630.6:0.00278,(((UltCl238:0.00372,Ul
tCl237:0.00376)0.995.31:0.03397,Ult22730:0.02278)0.734.15:0.00417,((((Sgf
ffff3:0.00713,UltCl235:0.0079)0.918.29:0.01339,(UltCl221:0.02386,Ult22699
:0.03901)0.729.10:0.00967)0.899.27:0.01372,((((Ult22693:0.02723,((Ult2269
5:0.01533,((Ult22696:0.0,Ult22697:0.0):0.00759,Ult22698:0.02778)0.940.24:
0.01959)0.680.6:5.5E-
4,Ult22694:0.00721)0.071.4:0.00821)0.177.5:0.00854,(Ult22728:0.03854,(Bac
IrtJ2:0.01121,BacIrtJ3:0.00375)0.963.29:0.03378)0.680.7:0.01273)0.752.22:
0.01211,((Ult22723:0.02594,((Ult22724:0.0,Ult22720:0.0,Ult22721:0.0):5.5E
-4,Ult22722:0.00369)0.936.32:5.5E-
4)0.996.17:0.04476,Ult22700:0.02517)0.601.2:0.00979)0.350.4:5.5E-
4,UltCl236:0.01881)0.879.26:0.008)0.584.6:0.00943,CsrLeptu:0.03155)0.753.
20:0.00544)0.905.26:0.01045)0.858.23:0.00932)0.240.4:5.4E-
4)0.970.28:0.03261,Ult22729:0.04206)0.943.29:0.02879,Ult22545:0.01867)0.6
46.5:0.00863)0.963.30:0.02706)0.951.28:0.02623)0.299.3:0.01084)0.839.22:0
.01225)0.877.39:0.01248,(((((Ult22354:0.00842,((Ult22355:0.01476,(UltRu42
2:0.0411,UltRu421:0.0672)0.851.28:0.01181)0.866.30:0.01236,Ult22331:0.019
68)0.465.4:0.00395)0.873.29:5.4E-
4,((((((((Ult22326:0.01092,((Ult22322:0.0,Ult22318:0.0):5.5E-
4,Ult18539:0.027)0.877.40:5.4E-
4)0.954.31:0.03229,(Ult22316:0.00372,((Ult18191:0.0,Ult22320:0.0,Ult22321
:0.0,Ult22315:0.0,Ult22313:0.0,Ult22314:0.0):5.5E-4,Ult22319:5.5E-
4)0.377.3:5.5E-
4)0.989.22:0.03421)0.825.17:0.01259,(HdbSacch:0.03091,((((((Ult22296:0.00
372,Ult18458:0.0435)0.517.2:5.5E-4,(Ult22328:0.0,Ult22290:0.0):5.5E-
4)0.918.30:5.4E-4,(Ult22295:0.03569,((Ult22289:0.0,Ult22291:0.0):5.5E-
4,(Ult22292:0.01129,Ult22293:0.00376)0.106.1:0.00751)0.914.26:0.01066)0.9
71.21:5.3E-
4)0.812.13:0.01483,Ult22294:0.00967)0.764.23:0.00579,Ult22288:0.00819)0.8
94.21:0.01243,UltCl193:0.02245)0.554.4:0.00839)0.896.23:0.01248)0.741.15:
0.00381,(AnnColi2:0.0,AnnColih:0.0,Ult22323:0.0):0.02355)0.803.15:0.00472
,(((S0114462:0.02894,(UncUn183:0.04621,UltCl199:0.03224)0.774.37:0.02393)
0.722.5:0.00241,((Ult20097:0.01529,((((Ult22352:0.0037,(Ult22349:0.02369,
Ult22350:0.02776)0.882.30:0.01108)0.766.18:0.00381,((Ult22344:0.01158,Ult
22345:0.02522)0.856.20:0.00816,(Ult22342:0.02374,Ult22343:0.01988)0.749.2
8:0.00302)0.957.24:0.01626)0.874.34:5.3E-
4,((Ult22330:0.02693,(Ult22333:5.5E-4,Ult22337:0.00372)0.942.32:5.4E-
4)0.779.24:0.00342,((Ult22336:0.00373,(Ult22335:0.00262,Ult22334:0.06492)
0.999.40:0.00121)0.846.25:0.00373,Ult22332:5.5E-

4)0.855.21:0.00792)0.997.28:0.02832)0.756.32:0.00468,(Ult22338:0.00786,(U
lt22341:0.00727,((Ult22339:0.00381,Ult22340:0.01527)0.945.31:0.01154,(Ult
22310:0.0073,Ult22311:0.01176)0.987.25:0.02879)0.934.26:5.4E-
4)0.501.4:0.00775)0.946.26:0.01881)0.210.4:0.00511)0.976.17:0.03104,(Ult2
2357:0.01144,(Ult22356:0.03203,Ult22358:0.01574)0.741.16:0.00341)0.889.27
:0.00823)0.857.34:5.5E-
4)0.961.29:0.01803,(Ult22279:0.0336,Ult22317:0.04534)0.784.20:0.01108)0.9
10.47:5.5E-
4)0.830.23:0.00822,((UltCl186:0.04418,UltCl187:0.01839)0.992.24:0.06159,(
((AtnElong:0.03712,Ult22278:0.03173)0.502.2:0.009,(Ult22309:0.00434,UltCl
198:0.04486)0.958.31:0.02401)0.229.5:5.3E-
4,(Ult22274:0.00637,(Ult22273:0.03967,(UltRu419:0.07364,UncUn188:0.052)0.
133.2:0.01141)0.854.20:0.01965)0.936.33:0.01982)0.874.35:0.01951)0.774.38
:0.00971)0.885.28:0.01473,(Otu00469:0.05281,(PahggY66:0.00389,PahggY67:0.
00747)0.939.30:0.0305)0.903.27:0.02107)0.525.2:5.4E-
4,(Ult22351:0.03421,(UltCl191:0.00577,Otu00541:0.00571)1.000.148:0.1186)0
.562.5:0.0249)0.896.24:0.02134)0.859.30:0.011,(((Ult22325:0.01515,(Ult223
24:5.5E-4,Ult22327:0.00373)0.142.1:5.3E-
4)0.980.13:0.02758,(Ult22308:0.04936,(Ult22225:0.00793,(Ult22347:0.0,Ult2
2348:0.0):0.03182)0.435.3:0.02293)0.771.29:0.0054)0.000.266:5.4E-
4,Ult22353:0.02032)0.488.5:0.00615)0.849.26:0.01626,(((Ult22256:0.02821,(
(Bfhggg56:0.04303,(((((UltCl176:0.0,Otu00294:0.0):0.01084,UltCl177:0.0208
1)0.854.21:0.00826,(UltCl183:0.03134,UltCl184:5.4E-
4)0.979.24:0.02056)0.731.9:0.00483,((UncUn172:0.00745,UncUn173:0.00776)0.
762.27:0.00322,(Otu00225:0.00756,(Ult22263:0.02324,(PahggY65:0.01509,(Pah
ggY64:5.5E-4,Ult22262:0.0037)0.780.22:5.4E-4)0.185.5:5.5E-
4)0.821.20:0.00709)0.921.42:0.01253)0.971.22:0.02404)0.786.32:0.00741,((U
ltRu418:0.03579,UltCl178:5.4E-
4)0.683.9:0.01561,(UltRu417:0.01376,(UltCl180:0.0561,UltCl175:0.01821)0.6
25.4:0.02381)0.811.18:0.01791)0.861.36:0.01219)0.844.30:0.01113)0.909.26:
0.01729,((Ult22258:0.01088,(Otu00098:0.00422,UncUn178:0.02288)0.013.1:0.0
0777)0.776.21:0.00473,((Ult22260:0.00792,Ult22259:0.00338)0.986.21:0.0240
1,((UncUn175:0.00737,(UltCl179:0.00411,(UncUn174:0.04109,Ult22257:0.01648
)0.714.9:0.00217)0.862.27:0.01178)0.946.27:0.01683,(UncUn179:0.02279,(Unc
Un171:0.03561,UltCl174:0.02863)0.355.5:0.00852)0.828.19:0.0079)0.753.21:0
.00384)0.888.37:0.00781)0.766.19:5.5E-
4)0.855.22:0.00796)0.752.23:0.00644,(((UncUn182:0.02733,(UncUn180:5.4E-
4,UncUn181:0.00741)0.803.16:5.4E-
4)0.840.22:0.01189,((UncUn170:0.00976,Ult22255:0.01737)0.962.37:0.03246,(
Ult22470:5.4E-
4,(Ult22469:0.00368,(Ult22471:0.00369,Ult22468:0.00369)1.000.149:5.3E-
4)0.936.34:0.01897)1.000.150:0.05846)0.800.14:0.01385)0.829.18:0.02051,((
(UltCl182:5.4E-
4,(UncUn177:0.0054,UltCl185:0.01849)0.884.30:0.01008)0.597.5:0.01145,(Unc
Un176:0.06751,UltCl181:5.4E-
4)0.808.18:0.00754)0.744.18:0.00437,(MbByy000:0.02057,(MmDYyyyy:0.01199,U
lt22261:0.01929)0.907.32:0.01311)0.752.24:0.00489)0.964.37:0.02822)0.743.
24:0.0078)0.889.28:0.02469,(((Ult22266:0.00373,Ult22268:5.5E-
4)0.979.25:5.3E-4,(Ult22265:0.00484,(Ult22271:0.01546,(Ult22270:5.5E-
4,Ult22267:0.00369)0.747.19:0.00352)0.927.31:0.01493)0.814.17:0.01856)0.8
90.34:0.01976,(Ult22269:0.08183,(Ult22312:0.01744,Ult22272:0.1054)0.762.2
8:0.00819)0.855.23:0.02085)0.988.31:0.05244)0.840.23:0.02442)0.743.25:0.0
0665,(Ult22302:0.03023,(Ult22297:0.0201,((Ult22298:0.02684,Ult22299:5.5E-
4)0.983.18:0.03615,(Ult22300:5.5E-

4,Ult22301:0.00369)0.829.19:0.00951)0.848.24:0.01609)0.216.3:0.01235)0.84
1.23:0.01047)0.961.30:0.02001)0.712.6:0.00359,(CsrSpe13:0.02419,((((Ult22
285:0.0,Ult22286:0.0):0.00174,Ult22287:0.01097)0.915.27:0.00222,((Ult2228
1:0.00372,(Ult22282:5.5E-4,(Ult22280:0.0,Ult22283:0.0):5.5E-
4)0.561.2:5.5E-4)0.998.30:5.3E-
4,Ult22284:0.113)0.791.15:0.00375)0.998.31:0.06545,(Ult22474:0.00369,UltR
u426:5.5E-
4)0.906.44:0.02104)0.458.6:0.00737)0.966.35:0.02835)0.746.18:0.00621)0.77
1.30:0.00525,((((((RmcAlbu2:0.00221,RmcAlbus:0.02487)0.681.3:0.00371,(Ult
Ru424:0.02309,Ult22453:0.02916)0.719.2:0.00503)0.853.23:0.00825,((UltRu42
5:0.01596,((Ult22329:0.0037,Ult22455:0.00371)0.761.28:0.00357,Ult18280:0.
02325)0.877.41:0.00775)0.626.6:5.4E-
4,Ult22456:0.02709)0.922.31:0.01442)0.913.30:0.01302,((Ult22412:0.01458,(
UltRu423:0.02392,(UltB7238:5.5E-
4,Ult22413:0.00743)0.929.31:0.02171)0.400.5:0.00835)0.959.38:0.02644,(((U
dnRum33:5.4E-4,(Ult22420:0.03126,Ult22402:0.03128)0.879.27:5.4E-
4)0.918.31:0.01467,((((Ult22404:0.01563,(Ult22410:0.01132,Ult22411:5.4E-
4)0.855.24:0.00762)0.924.28:0.01831,((Ult22424:0.00369,(RmcFlav3:0.00745,
((Ult22427:0.00741,Ult22430:5.5E-
4)0.848.25:0.00369,((Ult22429:0.0,Ult22432:0.0,Ult22677:0.0):5.5E-
4,(Ult22428:0.0037,Ult22431:0.00373)0.811.19:0.00371)0.871.30:5.5E-
4)0.960.30:0.01123)0.762.29:5.4E-4)0.912.39:5.5E-
4,((Ult22440:0.0,Ult22441:0.0):0.00777,((Ult22443:0.01381,(Ult22439:0.007
41,Ult22438:0.00375)0.969.23:0.02526)0.930.22:0.01975,((Ult22398:0.0,Ult2
2442:0.0):5.3E-4,(Ult22437:0.0112,(Ult22435:0.00369,Ult22436:5.5E-
4)0.965.31:5.5E-
4)0.955.25:0.02265)0.919.33:0.01721)0.760.19:0.0061)0.940.25:0.01536)0.78
1.23:0.00509)0.607.2:0.00774,((Ult22406:0.00365,(Ult22399:0.03092,Ult2241
4:0.01797)0.937.26:0.01795)0.571.3:0.01146,((Ult22407:0.0,Ult22409:0.0):5
.5E-4,Ult22408:0.00369)0.976.18:5.3E-
4)0.931.29:0.01241)0.950.28:0.01833,(((Ult18291:0.01082,Ult22422:0.06172)
0.734.16:0.00436,(((RmcFlav2:0.0,RmcFlave:0.0):0.00759,(Ult22416:5.5E-
4,(Ult22405:0.01512,Ult22423:5.5E-
4)0.857.35:0.00369)0.807.14:0.00379)0.895.42:0.00757,Ult22418:0.01886)0.7
58.29:5.5E-4)0.455.7:5.4E-4,(Ult22419:0.0,Ult22417:0.0):5.5E-
4)0.905.27:5.4E-4)0.453.3:5.5E-
4)0.655.5:0.01145,Ult22415:0.01949)0.892.25:0.01429)0.756.33:0.00444)0.79
4.20:0.00397,((Ult19025:0.04408,(((Ult22446:5.5E-
4,Ult31661:0.0074)0.976.19:5.4E-
4,(Ult22448:0.00387,(Ult22447:0.00383,Ult18290:0.015)0.826.19:0.00742)0.9
94.28:0.03559)0.961.31:0.02419,(Ult22459:0.0,Ult22458:0.0):0.02379)0.446.
2:0.01093)0.863.31:0.01176,(Ult22454:0.01428,(Ult22445:0.00466,(((((RmcFl
av4:5.5E-
4,Ult22425:0.00368)0.921.43:0.00741,(Ult22401:0.00369,(Ult22403:0.0,Ult22
426:0.0,RmcSpec3:0.0):5.5E-4)0.681.4:5.5E-
4)1.000.151:0.00315,(Ult18283:0.0118,(Ult18278:0.01429,Ult18289:0.01961)0
.795.25:0.01128)0.453.4:0.0117)1.000.152:5.4E-
4,(Ult18282:0.00372,(Ult22457:0.00206,Ult22444:0.01111)0.086.4:0.00198)0.
813.14:0.00379)0.770.31:0.00374,((((Ult22449:0.0,Ult22452:0.0):5.5E-
4,(Ult22450:0.0074,Ult18281:0.00369)0.912.40:5.5E-4)0.909.27:5.5E-
4,(Ult18279:0.01117,Ult22665:0.01114)0.603.8:5.5E-4)0.924.29:5.5E-
4,Ult22400:0.01121)0.949.32:0.01141)0.873.30:0.00733)0.000.267:5.5E-
4)0.000.268:0.00371)0.925.38:5.3E-
4)0.780.23:0.01396,(((((Ult22719:0.00979,(((Ult22718:0.03969,(UltCl230:0.

00369,Otu00559:5.5E-4)0.986.22:5.4E-
4)0.950.29:0.02537,(UltRu435:0.01913,Ult22717:0.0045)0.929.32:0.02607)0.9
59.39:0.02539,(PahggY70:0.00718,UltCl227:5.3E-
4)0.999.41:0.07316)0.882.31:0.01478)0.780.24:0.0184,(OphioYy3:0.03817,(Bf
hggg57:0.01334,UltCl226:0.06824)0.919.34:0.0236)0.900.28:0.02166)0.935.34
:0.02439,(Ult18152:0.05558,(Try00024:0.19944,(((Otu00998:0.02754,(UltCl23
4:0.02789,(UltCl233:5.3E-
4,(UltCl231:0.01992,UltCl232:0.04239)0.969.24:0.02331)0.774.39:0.00737)0.
725.10:0.01569)0.967.21:0.03943,((Ult22741:0.03747,UltCl239:0.04765)0.755
.23:0.00818,((((((UltRu443:0.01894,(UltCl242:0.004,(UltCl241:0.004,UltFir
47:0.0072)0.146.3:0.0037)0.992.25:0.02692)0.193.2:5.5E-
4,(Ult22761:0.00369,((Ult18266:0.0,Ult18265:0.0,Ult18268:0.0,Ult22745:0.0
,Ult22760:0.0):5.5E-4,Ult18267:5.5E-4)0.712.7:5.5E-
4)0.914.27:0.00758)0.877.42:0.00853,(Ult18270:0.00855,(Ult22757:0.01813,U
ltCl240:0.01072)0.747.20:0.00525)0.783.24:0.00731)0.895.43:0.01087,(UltRu
441:0.0112,((((UltRu440:0.01138,Ult22765:0.01141)0.869.28:0.00767,(((Ult2
1303:0.0101,Ult22769:0.02668)0.770.32:0.00529,((Ult18264:0.0,Ult22752:0.0
,Ult22751:0.0,Ult22750:0.0,UltRu438:0.0,Ult22758:0.0,Ult22748:0.0,Ult2274
7:0.0,Ult22749:0.0):5.5E-
4,(Ult22759:0.00369,Ult21922:0.00369)0.923.35:5.5E-4)0.920.34:5.5E-
4)1.000.153:5.4E-4,(((Ult22753:0.0,Ult22746:0.0):5.4E-
4,Ult22768:0.00743)0.902.37:0.0076,(Ult22755:0.01163,Ult22756:0.00371)0.8
94.22:0.00773)0.798.21:0.00364)0.937.27:0.00756)0.326.1:5.4E-
4,(Ult22763:0.01201,(Ult22766:0.01142,(Ult22638:5.5E-
4,Ult22764:0.01512)0.764.24:0.00375)0.818.13:0.00709)0.650.3:0.00766)0.81
7.12:0.00695,(((UltRu437:0.02731,Ult22743:0.01577)0.854.22:0.00811,(Ult22
742:0.02288,(Ult22740:0.01227,((Ult22738:0.0,Ult22739:0.0):0.00318,BacNL3
06:0.04593)0.779.25:0.02089)0.952.31:0.02595)0.782.28:0.00634)0.936.35:0.
01949,UltRu439:0.03126)0.461.4:5.4E-4)0.911.36:0.00822)0.915.28:5.4E-
4)0.780.25:0.00376,(UltCl243:0.02042,((Ult22727:0.02613,(Ult22726:0.04381
,Ult20701:0.02987)0.859.31:0.01179)0.759.30:0.00635,Ult22754:0.0117)0.917
.28:0.0183)0.495.4:0.00726)0.949.33:5.5E-
4,((UltRu442:0.01147,UltRu444:0.01524)0.860.20:0.00806,(RmnBact5:0.00794,
Ult22762:0.01115)0.958.32:0.01944)0.936.36:0.01979)0.940.26:0.02491)0.875
.30:0.02359)0.930.23:0.02562,((((Ult22705:5.4E-
4,((Ult22704:0.00751,((Ult22701:0.0,Ult22703:0.0):5.5E-
4,Ult22702:0.02359)0.764.25:0.00364)0.932.29:0.01912,(UltCl222:0.03911,(U
ltFir45:0.06862,Try00030:0.03612)0.911.37:0.02576)0.867.30:0.02909)0.860.
21:0.01239)0.869.29:0.00783,Ult22706:0.01102)0.949.34:0.02324,(UltCl223:0
.03866,UltCl229:0.08086)0.405.5:0.00749)0.855.25:0.01559,(UltCl225:0.0472
3,(UltCl224:0.08285,UltFir46:0.06253)0.987.26:0.07153)0.957.25:0.04663)0.
743.26:0.02353)0.505.1:0.02361)0.843.23:0.02011)0.137.3:0.01168)0.794.21:
0.00993,Ult22421:0.08353)0.629.6:0.02401,(((UltRu478:0.02704,Ult23505:0.0
4125)0.942.33:0.02893,((UncUn191:0.01898,UltCl201:0.08877)0.929.33:0.0326
8,(UltRu479:0.03681,(Ult23506:0.0208,UltCl260:0.08454)0.869.30:0.02494)0.
874.36:0.02096)0.816.19:5.4E-
4)0.976.20:0.04122,((((UltCl172:0.02295,Otu01426:5.4E-
4)1.000.154:0.0673,((((((UltCl159:0.00773,UltCl160:0.00348)0.984.15:0.041
28,((UltCl166:0.0,UltCl167:0.0):0.01112,UltCl168:0.013)0.920.35:0.02573)0
.889.29:0.01884,(UltCl170:0.03121,Otu00295:0.03495)0.767.22:0.00618)0.768
.15:0.00756,(UltCl171:5.5E-
4,Otu00270:0.01104)0.931.30:0.0199)0.846.26:0.0092,Otu00293:0.04513)0.313
.2:0.01811,(Ult22250:0.0,Otu00503:0.0):0.02206)0.949.35:5.4E-
4)0.833.24:0.02712,((Otu00536:0.01883,(((UltCl155:0.0,UltCl156:0.0):0.043

29,(UltCl165:0.02157,(UltCl164:0.04041,(UltCl163:0.00984,Otu00994:0.009)0
.967.22:0.05257)0.966.36:0.04296)0.940.27:0.02995)0.867.31:0.01974,(UltCl
157:0.05386,(UltCl158:0.0,Otu00076:0.0):0.00467)0.799.26:0.01177)0.883.39
:0.02052)0.864.33:0.01932,(Otu01549:0.04458,(UltCl162:0.0812,(UltCl161:0.
01498,Otu00819:5.5E-
4)0.995.32:0.08919)0.876.29:0.03129)0.930.24:0.04116)0.479.6:0.01199)0.86
2.28:0.01454,(UltCl173:0.05365,((Cuiii24:0.00374,Ult22249:0.0151)0.943.3
0:0.01952,(UltLac45:0.01187,UltCl169:0.01498)0.884.31:0.01164)0.877.43:0.
01539)0.681.5:0.00655)0.875.31:0.02027)0.916.36:0.02566)0.807.15:0.00899)
0.797.21:0.00888)0.924.30:0.01724)0.891.30:0.01962,Ult23351:0.04278)0.916
.37:0.02785)0.898.38:0.01981)0.771.31:0.00666)0.743.27:0.00422)0.749.29:0
.00451,(Ult23378:0.03789,((Ult23005:0.01659,(((Ult22928:0.03976,Ult23002:
0.03211)0.339.4:0.01614,(Ult23000:0.00491,((UltCl245:0.01501,(Ult22870:5.
5E-4,((Ult22872:0.0,Ult22873:0.0,Ult22896:0.0,Ult22897:0.0):5.5E-
4,(Ult22874:0.00369,Ult22871:0.00369)0.924.31:5.5E-4)0.735.11:5.5E-
4)0.346.5:5.5E-
4)0.739.21:0.0038,((Ult20638:0.01487,((Ult22867:0.01119,Ult22868:0.00374)
0.963.31:0.01509,(Ult22866:5.5E-
4,Ult22895:0.00741)0.954.32:0.00746)0.888.38:5.4E-
4)0.065.5:0.00734,Ult22886:0.01153)0.894.23:0.01113)0.958.33:0.02198)0.95
7.26:0.03967)0.749.30:0.00957,Ult20639:0.02683)0.347.3:0.01718)0.167.1:0.
01089,Ult20615:0.06829)0.879.28:0.02596)0.919.35:0.02961)0.932.30:0.01905
)0.803.17:0.00208,(((((PahggY73:0.00758,(Bfhggg60:0.01954,(P0114720:5.5E-
4,UltCl267:0.00744)0.904.29:0.01136)0.860.22:0.00758)0.787.27:0.0041,Ult2
3376:0.02743)0.700.12:0.00157,(Ult23395:0.0366,UltCl261:0.02972)0.882.32:
0.01635)0.903.28:0.01341,((((Ult23396:0.00793,((UltCl266:0.01587,(UltCl26
5:0.01745,(UltCl262:0.00755,UltCl263:0.01127)0.859.32:0.00938)0.935.35:0.
01866)0.861.37:0.01184,((UltRu471:0.02402,(Ult23245:5.5E-
4,(Ult23153:0.01494,((Ult23243:0.0,Ult23248:0.0):5.5E-
4,(Ult23244:0.00369,((Ult23246:0.01901,Ult23247:0.00365)0.959.40:0.01919,
UltB7223:0.03065)0.613.6:5.5E-4)0.396.11:5.5E-4)0.603.9:5.4E-
4)0.895.44:0.01376)0.942.34:0.02565)0.840.24:0.01382,(Ult23322:0.02253,(U
lt23308:5.4E-
4,(Ult23309:0.01993,(SbcTermi:0.02333,(Z0114383:0.01041,(Otu00414:5.5E-
4,(UltCl271:0.00372,UltCl272:0.00374)0.948.41:0.01138)0.937.28:0.0208)0.9
33.36:0.0201)0.610.3:0.00563)0.945.32:0.02057)0.892.26:0.0147)0.769.26:0.
00527)0.836.19:0.00936)0.972.24:5.3E-
4)0.802.23:0.00468,(Ult23320:0.00372,Ult23321:5.5E-
4)0.964.38:0.0191)0.875.32:0.00959,(((Ult23138:0.02309,(Ult22930:0.01875,
((Ult22951:0.00181,(Ult22923:5.5E-
4,(((Ult22920:0.00749,(Ult24430:0.01156,((Ult22921:0.0,Ult22924:0.0):0.00
367,Ult22929:0.03666)0.000.269:5.5E-4)0.304.2:5.5E-4)0.363.5:5.5E-
4,(Ult22919:0.0,Ult22922:0.0,Ult22925:0.0,Ult23055:0.0):5.5E-
4)0.456.2:5.5E-4,Ult22926:5.5E-4)0.213.2:5.4E-
4)0.900.29:0.0025)0.923.36:0.00748,((Ult22953:0.00926,(Ult22931:0.00407,U
lt22952:0.01486)0.768.16:0.00972)0.816.20:0.0035,(Ult22956:0.00717,(Ult22
954:0.00745,((Ult20612:0.06121,Ult22869:0.02669)0.949.36:5.5E-
4,Ult22955:5.5E-4)0.092.3:5.4E-4)0.886.36:0.00709)0.905.28:5.5E-
4)0.750.16:5.4E-
4)0.761.29:0.00602)0.829.20:0.01113)0.754.22:0.00601,Ult22932:0.03117)0.7
90.19:0.00699,((((((Ult22938:5.4E-4,(Ult22935:0.01879,Ult22934:5.5E-
4)0.947.35:0.00742)0.770.33:0.00585,(Ult22936:0.00747,(Ult22937:0.0,Ult22
948:0.0):5.5E-
4)0.891.31:0.01029)0.856.21:0.01234,((Ult22945:0.01481,((Ult22909:0.0,Ult

22927:0.0,Ult22941:0.0,Ult22942:0.0,Ult22943:0.0,Ult22944:0.0,Ult22947:0.
0):5.5E-4,Ult22946:0.00369)0.979.26:5.4E-4)0.659.5:5.5E-
4,(Ult22966:0.01321,Ult18546:0.02675)0.887.35:0.01383)0.977.18:0.02492)0.
620.8:0.00802,Ult22933:0.00629)0.981.18:0.03215,(UltB7240:0.11878,(Ult229
50:0.00369,Ult22949:0.00743)0.105.2:5.4E-
4)0.859.33:0.00765)0.841.24:0.01481,Ult22957:5.5E-
4)0.823.18:0.00345)0.772.30:0.00371)0.894.24:0.00866,(((Ult23377:5.4E-
4,((Ult23379:0.0151,(Ult23381:0.01877,Ult23521:5.4E-
4)0.776.22:0.00369)0.167.2:5.4E-
4,(Ult23397:0.07767,(Ult23382:0.01158,Ult23383:0.02245)0.795.26:0.00106)0
.856.22:0.01095)0.999.42:0.03153)0.883.40:5.4E-
4,((UltRu261:0.05182,UltRu459:5.5E-4)0.979.27:0.01561,(UltRu458:5.5E-
4,(Ult23254:0.01957,Ult23307:0.01576)0.927.32:0.0151)0.766.20:0.00364)0.7
98.22:0.0073)0.816.21:0.01298,((Ult22843:0.05243,(((((Ult23141:0.01826,(U
lt22805:0.00766,(((Ult22793:0.0,Ult22835:0.0,Ult22837:0.0,Ult22841:0.0,Ul
t22844:0.0,Ult23003:0.0,Ult23264:0.0):5.5E-
4,(Ult22838:0.00369,(Ult22836:0.00741,((Ult22840:0.0,Ult23327:0.0):5.5E-
4,Ult20625:0.02347)0.832.18:0.00372)0.487.9:5.5E-4)0.236.1:5.5E-
4)0.245.2:5.3E-4,Ult22839:5.5E-
4)0.908.29:0.01151)0.907.33:0.01481)0.830.24:0.01006,((Ult22830:0.0,Ult22
831:0.0,Ult22827:0.0,Ult22828:0.0,Ult22829:0.0,Ult22842:0.0):0.01122,Ult2
2832:5.3E-
4)0.874.37:0.00801)0.859.34:0.00766,Ult22806:0.01159)0.754.23:0.00324,(((
(Ult22894:5.5E-
4,(((UltB8146:0.0,Ult22889:0.0,Ult22890:0.0,Ult22887:0.0,Ult22888:0.0):0.
00369,Ult22893:5.5E-4)0.999.43:5.4E-4,(Ult22891:5.5E-
4,Ult23001:0.0233)0.826.20:0.00367)0.978.16:0.02279)0.988.32:0.04393,((Ul
t22833:0.02659,Ult22834:5.5E-
4)0.965.32:0.03716,(Ult22940:0.03363,Ult22939:0.04778)0.793.19:0.02055)0.
902.38:0.01549)0.058.4:0.00574,(((Ult22845:5.4E-
4,Ult22847:0.01509)0.987.27:0.0277,(((Ult22880:0.0,Ult22881:0.0,Ult22882:
0.0):0.0097,(Ult22879:0.01914,Ult22883:0.00401)0.893.34:0.01426)0.804.20:
0.00241,((Ult22884:0.01184,(((UltCl246:0.03176,Ult22877:0.0079)0.686.8:0.
00754,Ult22876:0.00718)0.987.28:5.4E-
4,Ult20635:0.01895)0.664.3:0.01586)0.829.21:0.00745,((Ult22875:5.5E-
4,(BacNL308:0.00368,(LctBact2:0.00372,OscValer:0.00747)0.822.15:0.00369)0
.671.5:5.5E-4)0.882.33:5.4E-4,(BacNL307:5.5E-4,BacNL309:5.5E-
4)0.862.29:0.00367)0.785.29:0.00365)0.857.36:0.00783)0.960.31:0.0202)0.86
0.23:7.9E-
4,(UltBaci2:0.03199,((((Ult22846:0.00746,Ult22915:0.00759)0.893.35:0.0111
,(Ult22918:0.00619,(Ult22826:0.01829,Ult22917:0.02314)0.855.26:0.0118)0.9
20.36:0.01485)0.841.25:0.00788,(Ult22898:0.00744,Ult22916:5.4E-
4)0.893.36:5.5E-
4)0.875.33:0.00738,(((((Ult22862:0.00413,(Ult22905:0.03136,(Ult20599:5.4E
-4,Ult22885:0.00743)0.912.41:0.01175)0.812.14:0.00759)0.978.17:5.3E-
4,(Ult22907:0.02969,(Ult22904:0.01505,(Ult22903:5.5E-
4,(Ult20636:0.0037,((Ult22899:0.0,Ult22901:0.0,Ult22902:0.0):5.5E-
4,Ult22900:5.5E-4)0.835.24:5.3E-4)0.855.27:0.00372)1.000.155:5.3E-
4)0.660.5:0.02085)0.877.44:0.01531)0.824.27:0.00764,(Ult22861:0.0332,Ult2
2906:0.01921)0.902.39:0.01531)0.197.4:0.0072,((Ult20613:0.00369,Ult22878:
5.5E-
4)0.799.27:0.00835,(((CstBact6:0.0,Ult22864:0.0):0.0036,Ult22865:0.02692)
0.978.18:0.02447,(((Ult22849:0.00383,((Ult22850:0.03045,(Ult22851:5.5E-
4,(Ult22852:0.0,Ult22853:0.0,Ult22854:0.0):5.5E-4)0.998.32:5.5E-

4)0.919.36:0.00189,(((Ult22859:0.0,Ult22908:0.0):5.5E-
4,Ult22848:0.01923)0.978.19:5.5E-
4,Ult22860:0.08232)0.872.37:0.00208)0.858.24:0.00695)0.720.8:0.00752,(Ult
Cl244:5.4E-4,((Ult22855:0.0,Ult22856:0.0):5.5E-4,(Ult22857:5.5E-
4,Ult22858:0.00393)0.864.34:0.00395)0.864.35:0.00397)0.948.42:5.3E-
4)0.858.25:0.00771,Ult22863:0.00402)0.830.25:0.00792)0.863.32:0.01393)0.7
73.27:0.01493)0.950.30:0.01912,(Ult22912:0.00744,(Ult22913:0.00368,Ult229
14:5.5E-4)0.105.3:5.5E-4)0.034.3:5.5E-4)0.931.31:0.01135)0.063.4:5.3E-
4)0.989.23:0.03737)0.679.4:0.01844)0.778.25:0.00656,((((((Ult22809:0.0,Ul
t22814:0.0):5.5E-4,(Ult22811:0.00368,Ult22820:0.00368)0.877.45:5.5E-
4)0.920.37:5.5E-4,(Ult22810:0.04251,((Ult22781:5.5E-
4,((Ult22792:0.0,Ult22771:0.0,Ult22772:0.0,Ult22774:0.0,Ult22775:0.0,Ult2
2776:0.0,Ult22777:0.0,Ult22778:0.0,Ult22779:0.0,Ult22782:0.0,Ult22783:0.0
,Ult22784:0.0,Ult22825:0.0,Ult23004:0.0):5.5E-
4,(Ult22773:0.00368,(Ult22780:0.00368,Ult23006:0.01884)0.988.33:5.5E-
4)0.864.36:5.5E-4)0.759.31:5.5E-4)0.923.37:5.4E-
4,Ult22252:0.04345)0.911.38:0.01099)0.874.38:5.4E-
4)0.933.37:0.00737,((Ult22807:0.0,Ult22808:0.0):5.5E-
4,((Ult22803:0.03054,((Ult22365:0.0,Ult22802:0.0):5.5E-
4,Ult22804:0.01872)0.655.6:5.4E-
4)0.874.39:0.00366,Ult19100:0.01494)0.868.23:5.3E-4)0.915.29:5.5E-
4)0.727.8:5.5E-
4,(Ult22798:0.01125,(((Ult23281:0.00368,Ult23944:0.0074)0.732.3:5.5E-
4,((Ult22800:0.01116,Ult22797:0.00368)0.696.3:5.5E-
4,(Ult22801:0.0,Ult23301:0.0):5.5E-4)0.000.270:5.5E-4)0.941.27:5.5E-
4,Ult22799:0.00368)0.960.32:5.5E-
4)0.926.30:0.00749)0.874.40:0.00776,(((UltB7241:0.00369,(Ult22786:0.00368
,(Ult22785:5.5E-4,(Ult22795:0.0,Ult22789:0.0,Ult22812:0.0):5.5E-
4)0.940.28:5.5E-4)0.691.5:5.5E-
4)0.928.26:0.00744,(Ult22824:0.0074,((Ult22796:0.00747,UltRu445:5.4E-
4)0.813.15:0.00365,(Ult22819:5.4E-
4,(Ult22790:0.00368,(Ult22794:0.00368,(Ult22787:0.0,Ult22788:0.0):5.5E-
4)0.874.41:5.5E-
4)0.990.22:0.01515)0.819.21:0.00374)0.899.28:0.00748)0.004.2:5.4E-
4)0.532.3:5.4E-
4,Ult22910:0.01478)0.923.38:0.01541)0.775.20:0.00352)0.877.46:5.4E-
4)0.979.28:0.02614,UltRu468:0.01045)0.778.26:0.00853)0.523.3:5.0E-
4,(Ult22594:0.00369,Ult23375:5.5E-4)0.934.27:5.4E-
4)0.879.29:0.01288)0.867.32:0.01166)0.992.26:5.4E-
4)1.000.156:0.00303,(Ult22383:0.02735,((Ult23517:0.00363,((((Ult23204:0.0
0704,Ult20630:0.00716)0.931.32:5.5E-
4,(((((Ult23175:0.00385,(Ult22362:0.00744,(Ult23176:0.0,Ult23196:0.0,Ult2
3197:0.0):0.00369)0.809.19:0.00371)0.203.1:5.5E-
4,(Ult22363:0.0,Ult23202:0.0,Ult23205:0.0):5.5E-4)0.553.1:5.5E-
4,Ult23173:0.01111)0.211.6:5.5E-
4,(Ult23226:0.00375,Ult23198:0.01127)0.888.39:0.00743)0.107.8:5.5E-
4,((Ult23201:0.00775,(Ult23203:5.5E-
4,Ult23235:0.00368)0.931.33:0.01166)0.869.31:0.00749,Ult20594:0.01481)0.1
64.3:5.4E-4)0.203.2:5.4E-
4)0.861.38:0.0037,(((((((Ult23188:0.02706,(((UltRu455:0.0037,Ult23237:0.0
0369)0.724.10:5.5E-4,(Ult23199:0.00369,Ult23167:0.00369)0.902.40:5.5E-
4)0.000.271:5.5E-4,(Ult20586:0.0,Ult23238:0.0):5.5E-4)0.975.23:5.4E-
4)0.867.33:0.00743,Ult23200:0.00745)0.762.30:5.4E-
4,(Ult23225:0.03162,(((Ult23172:5.5E-

4,(Ult23179:0.0037,(((Ult23160:0.00359,Ult23177:0.01496)0.787.28:0.00374,
(Ult23181:0.00369,((Ult23155:0.00369,((Ult23157:0.00373,((UltB7358:0.0265
4,(Ult20611:0.0,Ult23159:0.0):5.5E-4)0.910.48:0.00749,(((((Ult18409:5.5E-
4,Ult18410:0.01511)0.364.4:0.00391,(Ult20624:0.01531,Ult23184:0.02331)0.8
51.29:0.00728)0.749.31:0.0042,(Ult11343:0.08521,(Ult23233:5.4E-
4,Ult25756:0.09659)0.978.20:0.03452)0.926.31:0.02725)0.108.2:5.4E-
4,Ult23182:0.0188)0.845.30:0.0071,Ult23236:0.04144)0.000.272:5.5E-
4)0.117.1:5.5E-4)0.292.2:5.5E-4,Ult25302:0.00743)0.433.6:5.5E-
4)0.391.7:5.5E-
4,(Ult20629:0.0,UltRu453:0.0,Ult23142:0.0,Ult23143:0.0,Ult23150:0.0,Ult23
151:0.0,Ult23152:0.0,Ult23158:0.0):5.5E-4)0.000.273:5.5E-4)0.551.5:5.5E-
4)0.278.4:5.4E-4,UltRu454:0.0152)0.913.31:0.00792)0.405.6:5.5E-
4)0.031.1:5.5E-4,(((((Ult20588:5.4E-
4,Ult23195:0.00744)0.823.19:0.00369,Ult23165:0.00369)0.291.4:5.4E-
4,(Ult23149:0.0,Ult23169:0.0):5.5E-4)1.000.157:5.5E-
4,Ult20589:0.02295)0.832.19:0.00367,(Ult20631:0.01123,(Ult23146:0.02224,(
(Ult23503:0.01495,(Ult20585:0.01863,Ult23189:0.00378)0.847.32:0.007)0.904
.30:5.4E-
4,(Ult23168:0.00365,(Ult23166:0.0112,(Ult23178:0.00738,(((Ult22359:0.0,Ul
t22360:0.0):0.00742,Ult18075:0.00369)0.813.16:0.00369,((((Ult23171:0.0036
9,(Ult23164:5.5E-4,Ult23170:0.01906)0.305.1:5.5E-4)0.324.2:5.5E-
4,Ult20584:0.00369)0.457.5:5.5E-4,Ult23147:0.00369)0.458.7:5.5E-
4,(UltBa701:0.0,Ult20590:0.0,Ult23145:0.0,Ult23144:0.0,Ult23148:0.0,Ult23
154:0.0,Ult23156:0.0,Ult23180:0.0,Ult23185:0.0,Ult23187:0.0,Ult23241:0.0)
:5.5E-4)0.458.8:5.5E-4)0.374.4:5.5E-4)0.000.274:5.5E-4)0.496.4:5.5E-
4)0.171.2:5.5E-4)0.633.4:5.4E-4)0.860.24:0.00387)0.198.5:5.5E-
4)0.090.1:5.5E-4)0.914.28:5.4E-
4,((Ult23230:0.0,Ult23231:0.0):0.00747,((((((Ult20591:0.00746,(Ult23192:0
.00742,(Ult23191:0.00369,(Ult20587:0.0,Ult23227:0.0):0.00369)0.596.4:5.4E
-
4)0.783.25:0.00368)0.796.14:0.00371,Ult23190:0.00367)0.900.30:0.00737,(((
(Ult23161:5.4E-4,(Ult23162:0.00371,(Ult23221:5.5E-
4,Ult23220:0.01119)0.924.32:0.01131)0.882.34:0.00758)0.946.28:0.00746,Ult
23216:0.03864)0.999.44:5.5E-4,Ult23218:0.00369)0.304.3:5.4E-
4,(Ult23186:0.0,Ult23219:0.0,Ult23222:0.0,Ult23224:0.0):5.5E-
4)1.000.158:5.3E-
4)0.890.35:0.00347,(Ult23812:0.02334,(Ult22815:0.0115,Ult23183:0.00737)0.
828.20:0.00754)0.847.33:0.01537)1.000.159:5.5E-
4,((Ult23163:0.0,Ult23209:0.0,Ult23217:0.0):5.4E-4,((((Ult22595:5.4E-
4,((((Ult23213:0.00369,Ult22818:0.01886)0.912.42:5.5E-
4,(Ult23206:0.0,Ult23207:0.0,Ult23211:0.0,Ult23210:0.0):5.5E-
4)0.781.24:5.5E-4,Ult20593:5.5E-4)0.304.4:5.5E-
4,(Ult23212:0.01901,Ult23208:5.5E-
4)0.936.37:0.00741)0.901.41:0.00709)0.164.4:0.00368,Ult23215:0.00339)0.78
1.25:0.00401,((Ult25301:0.02707,Ult23242:0.0076)1.000.160:5.5E-
4,(Ult22004:0.04588,Ult23223:0.03833)0.390.3:0.01166)0.908.30:0.00307)0.7
80.26:0.00369,(Ult23228:5.5E-4,((Ult20592:0.0,Ult23193:0.0):5.5E-
4,Ult23194:5.5E-4)0.864.37:0.00369)0.858.26:0.0037)0.624.9:5.5E-
4)0.861.39:5.4E-4)0.824.28:0.00738,Ult23229:0.00741)0.741.17:5.5E-
4)0.887.36:0.00729)0.643.4:0.00773)0.755.24:0.00776)0.943.31:0.01146,(Ult
21782:0.00751,Ult23240:0.01129)0.763.29:0.00366)0.810.18:0.00387,(Ult2325
5:0.00371,((Ult20596:0.0,Ult23256:0.0):0.01555,(((UltCl258:0.02724,((Ult2
3295:0.0191,Ult23296:5.5E-
4)0.125.3:0.00997,(((UltRu456:0.0,Ult23286:0.0):5.4E-

4,(((Ult23251:0.01535,(Ult23250:5.5E-
4,UltCl257:0.00367)0.786.33:0.00363)0.904.31:0.01134,Ult23292:0.01138)0.8
75.34:0.00754,(Ult23290:0.0037,(Ult20619:5.5E-
4,Ult23284:0.00375)0.951.29:0.01161)0.908.31:0.00765)0.735.12:5.3E-
4)0.934.28:5.5E-
4,Ult23285:0.0112)0.908.32:0.01444)0.880.37:0.01054)0.991.28:5.4E-
4,((((Ult23261:5.5E-
4,(Ult23269:0.00367,(((Ult22823:0.00368,Ult20618:0.01117)0.666.4:5.5E-
4,Ult23270:5.5E-4)0.688.4:5.5E-
4,(Ult20600:0.0,Ult20602:0.0,Ult20614:0.0,Ult23267:0.0,Ult23268:0.0,Ult23
271:0.0,Ult23273:0.0):5.5E-4)0.735.13:5.5E-4)0.771.32:5.5E-
4)0.436.6:5.4E-
4,(Ult23083:0.00369,(Ult23399:0.02291,Ult22817:0.02679)0.520.3:5.4E-
4)0.806.11:0.00369)0.933.38:0.00738,(((Ult23283:0.02308,((((((Ult22911:0.
00369,Ult23139:5.5E-
4)0.834.17:0.00373,(Ult20634:0.00745,((Ult23272:0.0,Ult23278:0.0):5.5E-
4,(UltB7242:0.00369,(Ult23258:0.0,Ult23259:0.0):0.00743)0.639.1:5.5E-
4)0.938.24:5.5E-4)0.923.39:0.00747)0.875.35:5.4E-4,((UltRu460:5.5E-
4,UltCl273:5.5E-4)0.996.18:5.5E-
4,Ult23300:0.02301)0.934.29:0.01111)0.971.23:0.01916,Ult20601:5.5E-
4)0.695.5:0.00199,Ult23253:0.01831)0.926.32:0.0181,(Ult23276:5.4E-
4,(Ult23277:0.00397,(Ult23279:0.0075,Ult23297:0.00376)0.785.30:0.00343)0.
777.29:0.00755)0.916.38:0.01102)0.885.29:5.4E-4)0.676.2:5.5E-
4,((((Ult23293:0.00369,Ult23294:5.5E-
4)0.937.29:0.01149,(Ult23265:0.03088,(Ult23266:5.5E-
4,(Ult23282:0.0,UltRu457:0.0,Ult23299:0.0,Ult23298:0.0):5.5E-
4)1.000.161:5.5E-
4)0.786.34:0.00373)0.902.41:0.00756,Ult23140:0.00368)0.705.7:5.5E-
4,Ult23275:5.5E-4)0.723.15:5.5E-4)0.945.33:0.00739,(Ult23274:5.5E-
4,(Ult23260:0.00747,Ult23214:0.01125)0.874.42:5.5E-4)0.925.39:5.4E-
4)0.876.30:5.3E-
4)0.930.25:0.00731,(Ult23288:0.00744,(Ult23289:0.01142,Ult23953:0.00753)0
.790.20:0.0037)0.929.34:5.4E-
4)0.922.32:0.00729)0.919.37:0.00737,(Ult23287:5.5E-
4,(Ult20702:0.0074,Ult23082:5.5E-4)0.851.30:0.00368)0.663.6:5.4E-
4)0.892.27:0.0075)0.792.29:0.00371)0.890.36:0.00749)0.998.33:5.4E-
4,(Ult20632:0.01194,(Ult23252:0.03405,Ult23257:0.01089)0.789.16:0.00882)0
.235.4:0.00761)0.794.22:0.00704,Ult23239:0.02926)0.360.7:5.5E-
4)0.872.38:0.00366,Ult20598:5.3E-
4)0.792.30:0.00377)0.219.4:0.01102,Ult24037:0.03963)0.327.5:5.5E-
4)0.960.33:0.01929)0.923.40:0.01624)0.882.35:0.00868)0.829.22:0.01519)0.7
56.34:0.00465,(((Ult23606:8.7E-
4,Ult23587:0.03083)0.764.26:0.00744,(Ult23547:0.04795,(((UltCl317:0.03092
,(UncUn192:0.02017,Ult23531:0.00722)0.970.29:0.03662)0.359.2:0.01167,(Ult
23534:0.02734,((UltRu482:0.01948,(Ult23538:0.0042,(Ult23539:0.01089,UltCl
307:0.00419)0.073.3:0.00368)0.889.30:0.011)0.746.19:0.00548,((Ult23554:0.
04182,(Ult23553:5.4E-4,(Ult22277:0.01109,CsrCaeni:5.5E-
4)0.608.5:0.00364)0.945.34:0.02843)0.369.3:0.00808,(IroBac20:0.02067,(Ult
22587:0.03106,OphiYyy6:5.5E-
4)0.994.29:0.05675)0.789.17:0.02318)0.168.4:0.01232)0.928.27:0.02486)0.79
7.22:0.00747)0.537.4:0.00862,((((PahggY81:0.03925,UltFir56:0.03039)0.969.
25:0.04146,(UltOr290:0.04327,Ult23552:0.02812)0.057.3:0.01718)0.863.33:0.
02292,(Ult23536:0.01969,(Ult23535:0.02223,(Ult23568:0.0609,((((((UltRu488
:0.00382,(Ult23559:5.4E-

4,Ult23400:0.05698)0.892.28:0.00734)0.761.30:0.00371,Ult23353:0.00757)0.7
62.31:0.00339,(((Ult21788:5.5E-
4,Ult23557:0.00369)0.923.41:0.01828,((Ult23556:5.5E-
4,(UltB7231:0.0037,Ult23555:5.5E-
4)0.965.33:0.01136)0.932.31:0.00336,(UltRu484:0.00369,Ult23561:5.5E-
4)1.000.162:5.4E-4)0.534.3:0.01284)0.953.22:0.03164,(UltRu486:5.4E-
4,(Ult20739:0.01912,(Ult23558:0.01199,(UltRu485:0.00384,(UdnRum34:5.5E-
4,UltRu487:0.01132)0.782.29:0.00359)0.919.38:0.01475)0.885.30:0.01192)0.7
73.28:0.00335)0.404.1:0.00951)0.938.25:0.02226)0.711.11:0.00363,Ult23560:
0.12556)0.790.21:0.00789,CsrSpe15:0.00403)0.866.31:0.01582,(((Ult23569:0.
0239,Ult25880:0.07335)0.926.33:0.02764,(Ult23566:0.01426,(Ult23565:0.0532
1,Ult23564:0.07497)0.955.26:0.05215)0.996.19:0.06786)0.701.8:0.00315,(Ult
23567:0.10215,UltAct24:0.00717)0.911.39:0.02867)0.872.39:0.02442)0.778.27
:0.00978)0.957.27:0.02831)0.847.34:5.4E-
4)0.902.42:0.01714)0.779.26:0.01437,(Ult23548:0.0799,Ult22816:0.03639)0.8
91.32:0.03024)0.939.31:5.3E-
4)0.744.19:0.01047)0.435.4:0.00531)0.919.39:0.01826,((((UltCl314:0.00933,
((Ult23448:0.04037,(Ult18317:0.06917,(((Ult22708:0.01536,(Ult22226:0.0866
1,(UltCl208:0.02208,((UltFir43:0.02271,(((UltB7230:0.0,Ult22555:0.0,Ult22
565:0.0,Ult22566:0.0):5.5E-
4,Ult22567:0.00369)0.887.37:0.01463,(Ult22554:0.00358,Ult22553:0.01541)0.
888.40:0.01589)0.901.42:0.01568)0.788.22:0.00934,((Ult22542:0.00742,((Ult
22537:5.4E-4,(((Ult22533:0.00738,Ult22534:5.5E-
4)0.811.20:0.00373,((Ult22540:0.00369,Ult22541:0.00368)0.940.29:5.3E-
4,(((Ult22543:0.02321,(UltCl209:0.01485,UltFir39:0.01838)0.789.18:0.01445
)0.755.25:0.00406,(((Ult22544:0.02747,UltCl218:0.03049)0.840.25:0.01357,U
lt22536:0.00463)0.445.6:0.00992,(((Ult12228:0.0,Ult22520:0.0):0.0037,Ult2
2563:0.00746)0.968.23:5.4E-
4,((((((UltRu427:0.00378,((Ult22394:0.0,Ult22564:0.0):5.4E-
4,(((Ult22521:5.5E-
4,Ult22556:0.00369)0.975.24:0.01143,(Ult22561:0.00369,Ult22562:5.5E-
4)0.816.22:5.5E-4)0.827.19:5.4E-
4,(Ult26395:0.03696,Ult24424:0.04632)0.840.26:0.02125)0.834.18:0.01086)0.
900.31:0.0075)0.738.11:0.00362,(Ult22570:5.5E-
4,(Ult22560:0.00637,(Ult22547:0.01948,(((Ult22557:5.5E-4,((HumanG13:5.5E-
4,Ult22546:5.5E-
4)0.956.30:0.01911,(((Ult18418:0.00366,Ult23945:0.03101)0.885.31:0.00747,
Ult22549:0.00368)0.918.32:5.4E-
4,Ult22550:0.00368)0.979.29:0.02325)0.919.40:0.01131)0.442.5:0.00719,Ult2
2569:5.4E-
4)0.899.29:0.01407,Ult22552:0.02989)0.044.3:0.00867)0.598.1:0.01243)0.904
.32:0.01643)0.568.3:0.0076)0.972.25:0.0198,Ult22522:0.01956)0.104.4:0.003
53,((Ult22523:0.0,Ult22524:0.0):5.5E-
4,Ult18309:0.04337)0.760.20:0.00395)0.771.33:0.00338,((Ult22516:0.02639,U
lt22511:0.00363)0.903.29:5.4E-
4,(Ult22517:0.00742,(Ult22551:0.01885,((Ult22512:0.0,Ult22513:0.0,Ult2251
5:0.0):5.5E-4,Ult22514:0.00741)0.840.27:0.00368)0.434.3:5.5E-
4)0.912.43:0.0074)0.997.29:0.03109)1.000.163:5.3E-
4,(Ult22518:0.01121,Ult22519:0.00369)0.045.2:5.4E-
4)0.831.24:0.00362)0.972.26:0.01446)0.372.2:5.4E-
4)0.865.31:0.0075,(Ult22538:0.00743,Ult22539:5.5E-
4)0.801.21:0.00367)0.941.28:0.01107)0.907.34:0.00739)0.940.30:0.00207,Ult
22568:0.01101)0.940.31:0.0018)0.937.30:0.01069,(Ult22532:0.00746,(((((Ult
22529:0.00759,(Ult22531:5.5E-

4,Ult23636:0.02677)0.910.49:0.00758)0.783.26:0.00355,Ult22530:0.00376)0.919.41:0.0075,Ult22497:0.01496)0.631.4:5.4E-4,((Ult21378:0.02315,Ult22527:0.01899)0.879.30:0.01857,(Ult22525:0.0,Ult22528:0.0):5.5E-4)0.929.35:0.01094)0.937.31:0.00732,(Ult22526:0.0,Ult22535:0.0):5.5E-4)0.843.24:0.00736)0.897.22:5.4E-4)0.770.34:0.00364)0.700.13:5.5E-4,(Ult22558:0.01479,Ult22559:5.5E-4)0.971.24:0.03976)0.906.45:0.02661)0.735.14:0.00461)0.944.25:0.03893)0.876.31:0.03772)0.954.33:0.03028,Ult22715:0.02038)0.549.5:5.5E-4,(Ult23529:0.03624,(Ult22712:0.01133,(Ult22714:0.0075,((Ult22716:5.4E-4,(Ult22434:0.02811,(((Ult18254:0.01954,Ult22713:5.3E-4)0.854.23:0.00379,Ult18319:0.0037)0.933.39:5.5E-4,Ult26397:0.05811)0.876.32:0.01193)0.875.36:0.01182)0.783.27:0.00369,((S0114572:0.02694,Ult22707:0.01831)0.951.30:0.02186,(UltB7237:0.02328,(UltCl228:0.01919,(Ult22711:0.01325,Ult22710:0.01809)0.896.25:0.01674)0.823.20:0.00863)0.320.2:5.4E-4)0.937.32:0.01909)0.867.34:0.00792)0.866.32:0.00787)0.897.23:0.01161)0.724.11:0.00282)0.780.27:0.01307)0.647.2:0.02468)0.879.31:0.02196,UltCl315:0.01707)0.897.24:0.017)0.090.2:0.01519,(UltCl303:0.07535,(UltCl304:0.04409,PahggY79:0.01562)0.426.9:0.02402)0.982.13:0.06315)0.735.15:0.00519,((UltCl329:0.01178,(UltCl328:0.00333,Ult23620:0.01583)0.877.47:0.01115)0.887.38:0.01186,(UltCl331:0.01158,(UltCl333:0.0065,(UltCl332:0.01283,(UltCl330:0.01826,Ult23608:0.01416)0.865.32:0.01315)0.865.33:0.01572)0.879.32:0.01265)0.905.29:0.01243)0.931.34:0.01284)0.749.32:0.00108,Ult23571:0.03736)0.895.45:0.0126)0.820.11:5.5E-4)0.872.40:0.00741,((Ult23600:0.00376,((Ult23604:0.00714,(Ult23603:0.00718,Ult23607:0.00775)0.386.4:0.00744)0.910.50:5.4E-4,(Ult23602:0.0037,Ult23601:0.00746)0.708.8:5.5E-4)0.968.24:5.4E-4)0.783.28:0.00469,(((Ult23617:0.01617,(Ult23614:0.03106,(Ult23615:0.04342,Ult23616:0.02613)0.494.3:0.01854)0.143.3:0.01162)0.938.26:0.02549,(Z0114460:0.06138,(CsrSter2:0.0,CsrSterc:0.0):0.0204)0.906.46:0.02797)0.455.8:0.01148,(Ult23605:0.02714,Cuiiii28:0.02424)0.103.3:5.4E-4)0.823.21:0.01249)0.889.31:0.01132)0.826.21:0.00901)0.855.28:0.01228,(Ult17775:0.05398,(Ult23611:0.02662,(Ult23610:0.02045,Ult23609:0.03301)0.730.11:0.0037)0.664.4:0.00464)0.968.25:0.02497)0.505.2:0.00794)0.753.22:0.01214)0.061.3:0.01106,((((Ult21873:0.07391,(((UltCl145:0.02767,(((Ult21869:0.0437,Ult21870:0.01662)0.877.48:0.02118,Ult21875:0.05585)0.495.5:0.01281,(Ult21874:0.02324,(Otu00787:0.03989,UltCl147:5.5E-4)0.732.4:0.01207)0.802.24:0.00948)0.853.24:0.01328)0.850.33:0.01218,(Ult21872:0.0201,Ult21871:0.03303)0.901.43:0.01643)0.852.30:0.00937,((UltCl142:0.03781,UltCl143:0.01918)0.987.29:0.03295,(Ult21868:0.03424,UltCl144:0.02381)0.929.36:0.01892)0.814.18:0.01293)0.728.12:0.00929)0.926.34:0.02463,((((UltCl140:0.02205,UltCl141:0.02789)0.990.23:0.05559,(UltCl139:0.02032,(Ult21867:5.4E-4,Otu00340:0.01126)0.906.47:0.02399)0.882.36:0.02236)0.839.23:0.01715,UltCl146:0.06356)0.868.24:0.02104,((EchggY13:0.02419,(UltFir34:0.04963,Otu00367:0.01751)0.947.36:0.02832)0.215.3:0.01625,(Ult25103:0.065,((Ult21866:0.04398,(Otu00385:5.4E-4,(UltCl137:5.3E-4,UltCl138:0.02755)0.908.33:0.00741)0.715.3:0.00429)0.980.14:0.04817,((Ult21865:0.0,Otu00757:0.0):0.06225,(((((UltCl129:0.04831,(UltCl127:0.01509,(((UltCl119:0.01505,(((UltCl117:0.00369,((UltCl116:0.02109,(UltCl125:0.00443,UltCl126:0.04766)0.908.34:0.02046)0.927.33:0.01948,(UltCl122:0.01086,(UltCl118:0.0205,(UltCl115:0.01206,UltCl120:0.0268)0.061.4:0.00722)0.980.15:0.02249)0.802.25:5.4E-4)0.802.26:0.00371)0.726.8:5.4E-

4,UltCl123:0.00742)0.843.25:0.00824,UltCl121:0.01871)0.890.37:0.02658)0.994.30:0.05991,(UltCl124:0.02448,Tryy0105:0.08235)0.270.3:0.01729)0.435.5:0.01244,(Otu00419:0.04429,UltCl130:0.04441)0.765.17:0.02519)0.623.1:5.4E-4)0.810.19:0.01077)0.894.25:0.02498,UltCl128:0.02882)0.766.21:0.0204,Ult21863:0.0792)0.743.28:0.01774,UncUn148:0.02995)0.960.34:0.05597,((UltCl135:0.0349,(((UltCl132:0.00349,((Otu00179:0.01115,(Hmyyy059:0.0267,UltCl131:0.01484)0.940.32:6.3E-4)0.594.4:0.00757,Otu00240:0.01118)0.874.43:0.00796)0.606.4:0.00623,UltCl133:0.00527)0.683.10:0.0067,((Otu00598:0.01134,(UltCl134:0.0114,Otu00149:0.02333)0.745.25:0.00375)0.755.26:0.00434,Ult21864:0.05681)0.790.22:0.00908)0.561.3:0.02432)0.954.34:0.07262,((Tryy0097:0.0,Tryy0098:0.0):0.08577,UltCl136:0.05917)0.779.27:0.0487)0.777.30:0.02422)0.954.35:0.03545)0.810.20:0.01224)0.790.23:0.02102)0.892.29:0.02999)0.781.26:0.01123)0.566.3:0.00929)0.899.30:0.02047,((((Ult21847:0.03417,((Ult21838:0.08147,Ult22098:0.00547)0.395.5:0.00859,((Ult21830:5.3E-4,(Ult21829:0.01544,Ult24051:0.00744)0.927.34:0.0153)1.000.164:0.07667,(Ult21836:0.02351,(Ult21835:0.0035,((UltCl109:0.01926,((Ult21831:0.00359,(Ult21832:0.00366,Ult21833:0.00749)0.914.29:0.01158)0.914.30:0.01152,(Ult20930:5.5E-4,Ult21834:0.00369)0.977.19:0.02331)0.840.28:0.00761)0.902.43:0.01146,((Ult21824:0.0074,(((Ult21823:5.5E-4,(Ult21837:0.02774,((Ult21827:0.00421,Ult21826:0.01466)0.712.8:0.01584,(Ult18133:0.02286,Ult21828:0.01137)0.926.35:0.01884)0.798.23:0.00752)0.941.29:0.01996)0.899.31:0.01202,Ult23522:0.05339)0.844.31:0.01169,Ult18076:0.00666)0.855.29:0.00854)0.887.39:0.01937,Ult21825:5.4E-4)0.916.39:0.01895)0.764.27:0.0038)0.882.37:0.02263)0.744.20:0.01992)0.684.5:0.01229)0.987.30:0.05747)0.770.35:0.01538,(((Ult21846:0.01534,(Ult21839:0.0039,((Ult21841:0.00792,Ult21842:0.02762)0.863.34:0.01241,Ult21840:0.00407)0.631.5:0.00754)0.931.35:0.01164)0.979.30:5.4E-4,Ult21843:0.0148)0.974.13:0.02868,(Ult21844:0.03359,Ult21845:0.03171)0.913.32:0.02323)0.377.4:0.00351)0.957.28:0.03691,(((Ult21859:0.03532,(UltCl110:0.04923,(Bfhggg51:0.03343,(Ult22586:0.07017,((UltCl114:0.01873,((((UltRu411:0.01968,UltRu410:0.00354)0.969.26:0.03919,UltCata3:0.04953)0.889.32:0.0247,(Bfhggg52:0.03434,(Otu00793:0.06093,(UltCl111:0.01851,Otu01443:5.3E-4)0.984.16:0.05234)0.502.3:0.02892)0.648.6:0.00816)0.875.37:0.01425,(UltCata2:0.01573,UltCatab:0.00722)0.986.23:0.03983)0.784.21:0.01012)0.952.32:0.02392,(Ult21862:0.04329,Ult21861:0.00847)0.864.38:0.01294)0.800.15:0.01398)0.529.3:0.01979)0.907.35:0.02815)0.776.23:0.01643)0.891.33:0.02152,(Ult21858:0.00403,(UltRu409:0.06732,(Ult21856:0.00722,Ult21857:5.2E-4)0.812.15:0.00494)0.773.29:0.00707)0.919.42:0.01969)0.461.5:0.00709,(((OphiYyy4:0.05837,UltCl112:0.07125)0.848.26:0.02252,UltCl113:0.10485)0.243.3:0.02528,(UltFir33:0.03891,(Ult21860:0.05423,Otu01542:0.05293)0.815.15:0.01299)0.834.19:0.00983)0.740.21:0.00988)0.959.41:0.04341)0.967.23:0.05054,((Ult21850:5.4E-4,(((Ult21852:0.01585,Ult21854:0.02825)0.832.20:0.00717,Ult21851:0.00372)0.769.27:0.00367,Ult21853:0.01133)0.902.44:0.00751)0.988.34:5.5E-4,(Ult21848:5.5E-4,Ult21849:5.5E-4)0.916.40:0.0109)0.989.24:0.05306)0.706.8:0.00879)0.986.24:0.0366,(((((((Ult21898:0.03386,(Ult16707:5.5E-4,Ult21897:0.00719)0.845.31:0.01013)0.866.33:0.01425,((Ult21904:0.02018,Ult23815:0.18823)0.951.31:0.04639,(((Ult21896:0.00866,(Bfhggg54:0.03938,(UltCl390:0.05798,(Ult24287:0.03585,((SdrSpeci:5.5E-4,(SdrSaale:5.5E-4,Ult24283:5.5E-

4)0.982.14:0.01815)0.767.23:0.00442,((Ult24288:0.00782,Ult24289:0.01159)0
.775.21:0.00342,(Ult24286:0.01554,((Ult24285:0.0,Ult24284:0.0,Ult24290:0.
0):0.00869,Ult23807:0.08794)0.892.30:0.01492)0.855.30:0.00783)0.834.20:0.
00796)0.898.39:0.02447)0.876.33:0.02333)0.976.21:0.04988)0.867.35:0.02399
)0.906.48:0.01895,(Ult18295:5.4E-
4,Ult21895:0.03087)0.585.3:0.00857)0.828.21:0.01449,(((UncUn150:0.01997,B
fhggg50:0.03816)0.834.21:0.01584,(((Ult21892:0.04038,Bfhggg53:0.01315)0.9
63.32:0.02674,UncUn158:0.02729)0.840.29:5.4E-
4,UncUn151:0.00773)0.819.22:0.01632)0.196.4:0.01357,((UncUn159:0.01511,(R
mcSpec2:5.5E-
4,Ult21891:0.0269)0.912.44:0.03429)0.937.33:0.02749,((PahggY63:0.00405,(P
shggg51:0.00368,Pshggg52:5.4E-
4)0.854.24:0.01102)0.967.24:0.03981,(Ult21878:0.01731,UncUn149:0.01432)0.
771.34:0.02119)0.867.36:0.02771)0.706.9:0.01114)0.689.9:0.0139)0.854.25:0
.01905)0.325.4:0.00623)0.875.38:0.01472,((Ult21900:0.02629,UltFir37:0.014
71)0.917.29:0.02174,(((Ult21894:0.01716,((Ult21888:0.01827,(Ult21396:5.5E
-
4,Ult21889:0.00369)0.781.27:0.00516)0.929.37:0.01563,Ult21887:0.03574)0.8
45.32:0.01067)0.584.7:0.02161,((Ult21741:0.04069,(Ult23767:0.02597,(Ult18
244:0.02116,Ult19869:0.00591)0.840.30:0.01258)0.954.36:0.03192)0.924.33:0
.02953,((UncUn147:0.03972,(UltCl149:0.0277,(UltCl150:0.06591,UncUn152:5.4
E-
4)0.882.38:0.01153)0.861.40:0.0103)0.740.22:0.00559,((Ult21740:0.01035,(U
ltFir35:0.06288,Ult21886:0.06805)0.154.1:0.01564)0.700.14:0.00565,((UncUn
145:0.0,UncUn146:0.0):0.03457,(Ult28417:0.03032,(UncUn144:0.01326,(OphioY
y2:0.01937,(P0114402:0.02267,Bfhggg49:5.5E-
4)0.017.3:0.00532)0.815.16:0.00903)0.900.32:0.01566)0.533.3:0.00812)0.765
.18:0.00626)0.870.21:0.00855)0.733.14:0.00332)0.905.30:0.01721)0.631.6:0.
00236,Ult21879:0.03462)0.921.44:0.01992)0.796.15:0.00955)0.946.29:0.02111
,((((((Ult25762:0.15378,Ult25761:0.0313)0.986.25:0.07374,(((Ult25835:0.0
3697,(Ult23994:0.08474,(Ult16782:0.12293,(UltVeil5:0.04682,((UncUn203:0.0
0343,Ult25694:0.00399)1.000.165:0.06265,(UncUn205:5.4E-
4,(UncUn204:0.00377,Ult25710:0.00375)0.797.23:0.00376)0.839.24:0.00125)0.
577.2:0.01936)0.959.42:0.06305)0.914.31:0.03883)0.874.44:0.03586)0.682.5:
0.02434,(Ult25836:0.07545,Ult25837:0.0338)0.830.26:0.01456)0.913.33:0.020
48,((Ult25833:0.02141,((((Ult25827:0.0,Ult25832:0.0):5.4E-
4,(Ult25825:5.4E-4,((Ult25828:0.0,Ult25824:0.0):5.5E-
4,Ult25826:0.00751)0.844.32:0.0037)0.917.30:0.00743)1.000.166:5.5E-
4,((Ult25829:0.00745,Ult25830:0.00757)0.877.49:0.00787,Ult25831:0.01897)0
.569.4:0.0075)0.761.31:0.00322,(Ult26393:0.00379,Ult25834:0.04421)0.890.3
8:0.01174)0.856.23:0.01002)0.960.35:0.04206,((Ult25842:0.0073,Ult25843:0.
02373)0.962.38:0.02085,(Ult25841:0.03269,(Ult25840:0.00788,(Ult25838:0.0,
Ult25839:0.0):0.01961)0.774.40:0.00386)0.744.21:0.00727)0.935.36:0.0327)0
.926.36:0.04135)0.231.4:0.00356)0.727.9:0.004,((UltFir99:0.03553,Ult25917
:0.06883)0.686.9:0.03671,((((((DtmHydro:5.4E-
4,((DtmCarbo:0.00762,DtmSpec5:0.01155)0.858.27:0.00729,(DtmAeron:0.01952,
(DtmRumin:0.01155,(DtmSpec6:0.01914,DtmReduc:5.4E-
4)0.934.30:0.01147)0.855.31:0.00717)0.878.34:0.00781)0.783.29:0.00395)0.9
87.31:0.04692,(((UltSulf7:0.02897,(((PmlSchin:0.00675,PmlSchi2:0.02094)0.
943.32:0.02004,(PmlTherm:0.02272,(CptPheno:0.02362,PmlPropi:0.00768)0.741
.18:0.00437)0.191.1:0.00776)0.763.30:0.00687,(Ult25821:0.01833,(Ult25817:
0.01485,Ult25820:0.03022)0.727.10:0.01396)0.900.33:0.01936)0.542.1:0.0187
9)0.918.33:0.02494,((Ult25819:0.0,Ult32004:0.0):0.01548,(PmlTerep:0.02321
,PmlIsoph:5.4E-

4)0.903.30:0.01149)0.844.33:0.01227)0.711.12:0.00664,Ult25818:0.02669)0.9
46.30:0.03054)0.922.33:0.03227,((Ult25815:0.05483,((Ult25814:0.04231,(((U
lt25812:0.00987,(DtmArcti:0.02607,DtmAlcoh:5.4E-
4)0.825.18:0.0109)0.968.26:0.02748,(((((((DtmGibs2:0.0,DtmGibso:0.0):5.4E
-
4,(DtmGibs4:0.01151,DtmGibs3:0.01156)0.210.5:0.00349)0.999.45:0.04431,Sml
Syntr:0.00784)0.880.38:0.01183,SmlHydro:0.0238)0.961.32:0.02951,DtmSapom:
0.00904)0.867.37:0.0228,DtmTher4:0.0147)0.894.26:0.02428,UltMa109:0.03374
)0.312.6:0.01358)0.830.27:0.01305,(Ult25816:0.05455,(DtmGeot2:0.0,DtmGeot
h:0.0):0.02163)0.767.24:0.00757)0.871.31:0.01604)0.772.31:0.0094,(Ult2581
3:0.00423,UltrdL16:0.00424)0.892.31:0.01251)0.903.31:0.03516)0.793.20:0.0
4024,(UltrdL15:0.0223,(Ult25811:0.08889,DeiTherm:0.06819)0.893.37:0.03033
)0.857.37:0.01946)0.510.4:0.01385)0.410.5:0.01353,(((DvgTherm:0.01822,(Dt
mSpeci:0.01985,((DtmTher2:0.01882,((DtmSpec2:0.0,DtmTher3:0.0,DtmSpec4:0.
0):5.5E-4,DtmSpec3:5.5E-4)0.751.13:5.5E-
4)0.817.13:0.00372,((UltCl394:0.01202,(((((Ult25875:0.03658,(Ult16755:5.3
E-4,Ult16756:0.07442)0.755.27:0.00328)0.528.4:0.00771,CanDesul:5.3E-
4)0.920.38:0.0283,Ult25874:0.07696)0.951.32:0.03973,(AmfThiop:0.05717,Ult
25873:0.04264)0.721.5:0.00993)0.980.16:0.04325,Ult25810:0.01254)0.295.7:0
.01112)0.886.37:0.01072,((DtmTherm:0.0,DtmKuzn2:0.0,DtmSolfa:0.0):5.5E-
4,(DtmKuzne:0.00369,DtmLucia:0.00369)0.684.6:5.4E-4)0.937.34:5.4E-
4)0.799.28:0.00369)0.762.32:0.00313)0.897.25:0.0177)0.960.36:0.03171,((Tb
tCompo:5.4E-4,(TbtLitor:0.0074,TbtMaria:5.5E-
4)0.901.44:0.00744)1.000.167:0.0639,((((UltThe43:0.0031,LowGC004:0.02868)
0.859.35:0.02016,(TnmToyoh:0.0211,Ult32127:0.01773)0.378.7:0.01463)0.977.
20:0.04734,(((TnrSulfu:5.5E-
4,((TnrSpe10:0.00368,(Ult32124:0.0037,(((Ult32126:0.01808,UltGeot7:0.0659
8)0.882.39:0.01689,CabSubt4:0.02184)0.460.2:0.00886,CbbPaci2:5.4E-
4)0.971.25:0.01498)0.941.30:5.4E-4)0.437.2:0.00367,(((TnrSpec9:5.5E-
4,(((CabHydro:5.5E-4,CabUzone:5.5E-4)0.992.27:0.01906,((CabSubte:5.3E-
4,(BacteCP0:0.00376,Ult32125:0.02281)0.796.16:0.00367)0.817.14:0.00369,Cb
bPacif:0.00374)0.845.33:5.3E-4)0.926.37:0.00745,CabSubt3:5.5E-
4)0.970.30:0.01124)0.783.30:0.00369,CabProte:0.01136)0.800.16:0.00366,((U
lt32118:0.0,Ult32120:0.0,Ult32121:0.0,Ult32123:0.0):5.5E-
4,((Ult32119:0.00372,Ult32122:0.01886)0.900.34:5.5E-
4,CabSubt2:0.00742)0.559.3:5.5E-4)0.607.3:5.5E-4)0.870.22:5.4E-
4)0.989.25:0.02291)0.939.32:0.01475,(TnrUzone:5.5E-4,CsrUzoni:5.5E-
4)0.920.39:5.4E-4)0.713.9:0.01404,(UltThe42:0.00421,((TnrSpec5:5.5E-
4,(((TnrSpeci:5.5E-
4,(CsrSpe35:0.0,TnrSpec2:0.0,UltThe40:0.0,TnrSpec4:0.0):5.5E-
4)0.749.33:5.5E-4,TnrSpec3:0.00369)0.836.20:5.5E-
4,UltThe41:0.01116)0.830.28:0.00368)0.753.23:0.00364,((TnrAceto:0.00521,(
Ult32117:0.0151,(TnrSpec7:0.0,UltCl398:0.0,TnrSpec8:0.0):5.4E-
4)0.826.22:0.01397)0.817.15:0.01135,(TnrKivui:0.006,(TnrItali:5.4E-
4,(TnrTherm:0.00372,(TnrMathr:0.00369,TnrSpec6:5.5E-
4)0.811.21:0.0037)0.817.16:0.0037)0.992.28:0.0272)0.661.5:5.3E-
4)0.892.32:0.00901)0.990.24:0.03655)0.833.25:0.01331)0.966.37:0.0369)0.61
3.7:0.01644,(Ult25913:0.0124,((MooTherm:5.4E-
4,MooPerch:0.00753)0.950.31:0.02449,(MooGlyce:5.5E-
4,MooMulde:0.02672)0.899.32:0.01234)0.915.30:0.01679)0.819.23:0.01065)0.5
11.3:0.00545)0.890.39:0.02048)0.656.6:0.00116,UltrdL19:0.11023)0.976.22:0
.05126)0.939.33:0.02707,((((((UltOr381:0.08478,((UltCh349:0.04897,(Ult285
88:0.00933,Ult28587:0.05374)0.990.25:0.07611)0.909.28:0.04351,(((((Ult285
73:0.0,Ult28575:0.0):5.5E-4,Ult28574:5.5E-

4)0.992.29:0.05772,((Ult28568:0.0,Ult28569:0.0,Ult28570:0.0):0.00964,(Ult
28567:0.03928,UltOr380:0.10581)0.042.1:0.01509)0.967.25:0.04137)0.919.43:
0.02935,(((Ult28589:0.02986,(Ult28580:0.01428,((Ult28586:0.00743,UltCh348
:0.00371)0.974.14:5.4E-
4,((Ult28584:0.0,Ult28585:0.0):0.0037,Ult28590:0.0075)0.977.21:0.02313)0.
999.46:0.06468)0.837.19:0.02223)0.753.24:0.01261,((UltCh346:0.02645,UltCh
347:5.5E-
4)0.993.20:0.04471,(Ult28579:0.00338,Ult28578:0.00796)0.946.31:0.02494)0.
751.14:0.01374)0.808.19:0.01344,(UltCh350:0.00758,(UltrS362:0.02776,(Ult2
8576:5.5E-
4,Ult28583:0.00371)0.964.39:0.02776)0.980.17:0.02904)0.767.25:0.00688)0.4
88.6:0.01476)0.770.36:0.01259,(Ult28577:0.04505,(((Ult28571:0.02568,Ult28
572:0.02372)0.941.31:0.03179,((Ult28552:0.00374,((Ult28553:0.0,Ult28554:0
.0):5.5E-4,Ult28555:0.00369)0.998.34:5.5E-
4)0.940.33:0.01661,((Ult28564:5.4E-
4,Ult27578:0.07008)0.471.7:0.00367,(Ult28558:5.5E-
4,((Ult28556:0.0,Ult28557:0.0,Ult28559:0.0,Ult28560:0.0,Ult28561:0.0,Ult2
8562:0.0,Ult28563:0.0,Ult28565:0.0):5.5E-
4,Ult28566:0.00369)0.746.20:5.5E-4)0.800.17:5.4E-
4)0.785.31:0.00609)0.966.38:0.03114)0.886.38:0.01602,(Ult28581:0.05107,Ul
t28582:0.03441)0.993.21:0.06176)0.744.22:0.007)0.802.27:0.00892)0.879.33:
0.02431)0.915.31:0.03872)0.998.35:0.12121,((Ult25892:0.08866,((Ult25890:0
.06481,(Ult27558:0.03551,(Ult25889:5.4E-
4,(Ult25885:0.0076,((((Ult25877:0.00368,UltSymb2:0.02404)0.706.10:5.5E-
4,Ult12403:0.00369)0.355.6:5.5E-
4,(Ult25887:0.0,Ult25886:0.0,Ult31803:0.0,Ult31804:0.0,Ult32145:0.0):5.5E
-4)0.962.39:5.4E-4,(UltSymb3:0.0171,(Ult27480:0.01902,(Ult27481:5.4E-
4,Ult31805:0.03864)0.213.3:0.0074)0.923.42:0.01712)0.778.28:0.00689)0.753
.25:0.00358)0.996.20:0.03152)0.844.34:0.00686)0.838.27:0.01236)0.908.35:0
.02064,Ult25891:0.06877)0.652.3:0.00911)0.871.32:0.01673,(UltrdL17:0.0800
9,(UltCh486:0.01817,(Ult29185:0.05203,Ult29186:0.03046)0.664.5:0.00972)1.
000.168:0.1225)0.835.25:0.02965)0.463.5:5.5E-
4)0.757.20:0.00876,((BclSchle:0.03302,(UltCa398:0.02279,Ult31519:0.03882)
0.999.47:0.09544)0.962.40:0.05928,((((Ult17107:0.02387,((((Ult17075:0.011
92,(Ult17073:5.5E-
4,(Ult17072:0.01519,Ult17074:0.00376)0.763.31:0.00365)0.804.21:0.00766)0.
790.24:0.01509,(((((((Ult17078:0.0,Ult17110:0.0,Ult17096:0.0):5.5E-
4,(Ult17066:0.01497,(Ult17108:0.00369,Ult17109:5.5E-
4)0.855.32:0.00369)0.906.49:5.5E-
4)0.832.21:0.00369,(Ult17098:0.00744,Ult17086:0.0037)0.806.12:0.0037)1.00
0.169:5.3E-
4,(Ult17067:0.0021,Ult17102:0.0268)0.981.19:0.00166)0.819.24:0.00744,((((
(Ult17082:0.0,Ult17106:0.0):0.0037,Ult17101:0.00372)0.792.31:5.5E-
4,(Ult17097:0.00369,Ult17080:0.00742)0.738.12:5.5E-4)0.000.275:5.5E-
4,Ult17104:5.5E-4)0.704.4:5.4E-
4,(Ult17115:0.00742,((Ult17085:0.00737,Ult17105:0.01122)0.173.3:5.5E-
4,(((Ult17068:0.00369,(Ult17070:0.00371,Ult17071:0.0037)0.324.3:5.5E-
4)0.256.3:5.5E-
4,((Ult17081:0.0,Ult17113:0.0):0.00369,Ult17079:0.00369)0.700.15:5.5E-
4)0.559.4:5.5E-
4,(Ult17069:0.0,Ult17077:0.0,Ult17084:0.0,Ult17111:0.0,Ult17112:0.0,Ult17
114:0.0,Ult17100:0.0):5.5E-4)0.792.32:5.5E-4)0.523.4:5.1E-
4)0.830.29:0.00369)1.000.170:5.5E-
4)0.810.21:0.00359,Ult17083:0.01506)0.619.2:5.5E-

4,Ult17076:0.00716)0.423.2:0.01622)0.885.32:0.01623,UltDe213:0.05625)0.20
5.4:0.00385,((Ult17088:0.03651,(Ult17087:0.01789,((UltCa128:5.5E-
4,(UltrS301:0.09846,UltrS300:0.00746)0.914.32:5.4E-4)1.000.171:5.1E-
4,(UltCa129:5.4E-
4,(((UltCa125:0.01141,UltCa124:0.00753)0.874.45:0.00751,(UltCa126:0.0075,
UltCa127:0.00373)0.904.33:5.5E-
4)0.874.46:0.00752,CanEntot:0.00762)0.970.31:0.01946)0.913.34:0.00331)0.9
57.29:0.04068)0.922.34:0.02208)0.833.26:0.01504,(((Ult17093:0.0,Ult17103:
0.0):5.5E-
4,(Ult17094:0.00747,Ult17095:0.00369)0.779.28:0.00372)0.928.28:0.02829,(U
lt17092:0.0409,((Ult17089:0.0037,Ult17116:0.00746)0.977.22:5.5E-
4,(Ult17091:0.02333,Ult17090:0.0084)0.999.48:0.0457)0.984.17:0.03533)0.22
8.2:0.00887)0.851.31:0.01793)0.908.36:0.0172)0.992.30:0.05898)0.782.30:0.
01358,Ult17099:0.0563)1.000.172:0.18144,(Ult25758:0.0639,Ult31451:0.05681
)0.784.22:0.02109)0.894.27:0.04321,((((((Ult31484:0.04723,((Ult31482:0.03
427,(Ult31481:0.03812,Ult31485:0.04271)0.846.27:0.01414)0.756.35:0.00384,
Ult31483:0.01377)0.795.27:0.02078)0.994.31:0.09181,(Ult31498:0.06924,(((U
lt31473:0.01044,(Ult31471:5.5E-
4,Ult31472:0.01122)0.976.23:0.03093)0.881.36:0.01886,((Ult31480:0.03405,(
Ult31476:0.01591,(Ult31477:0.01169,Ult31478:0.01981)0.748.24:0.00778)0.54
6.6:0.00847)0.730.12:0.00626,((Ult31474:0.00867,Ult31475:0.03879)0.988.35
:0.04055,Ult31479:0.03574)0.137.4:5.4E-
4)0.914.33:0.01945)0.999.49:0.09137,(Ult31493:0.0298,(((Ult31496:0.02727,
Ult31495:5.4E-
4)0.874.47:0.01167,((Ult31487:0.0044,((Ult31488:0.01931,((Ult29181:5.5E-
4,Ult31489:0.00742)0.954.37:0.01136,Ult31486:0.00764)0.769.28:0.00358)0.3
56.2:5.5E-4,Ult31490:5.4E-
4)0.962.41:0.01511)0.706.11:0.00327,(Ult31492:0.01275,Ult31497:0.04703)0.
671.6:0.01279)0.940.34:0.01949)0.745.26:0.00437,(Ult31491:0.01629,Ult3149
4:0.02015)0.674.5:0.00239)0.822.16:0.01509)0.339.5:0.01505)0.603.10:0.017
08)0.543.3:0.03039)0.923.43:0.03452,(UltrS452:0.0332,(Ult31511:0.09448,((
Ult31509:0.02745,Ult31510:0.03087)0.903.32:0.02121,((((((UltAc946:0.0396,
(Ult31499:0.01575,((UltCa395:0.11795,UltCa396:0.10249)0.946.32:0.02639,Ul
tCa394:0.00383)0.858.28:0.01096)0.945.35:0.0168)0.523.5:0.00753,(Ult31500
:0.02057,((Ult31502:0.01126,Ult31503:5.5E-
4)1.000.173:0.07639,UltAc945:0.04809)0.612.6:0.00815)0.889.33:0.02097)0.9
42.35:0.02071,Ult31508:0.03106)0.818.14:0.00198,(Ult31506:0.0039,(UltCa39
7:0.05525,(Ult31504:5.5E-
4,Ult31505:0.00742)0.949.37:0.02079)0.836.21:0.01003)0.791.16:0.01007)0.8
86.39:0.01402,Ult31501:0.02121)0.896.26:0.02105,Ult31507:0.06359)0.681.6:
0.00507)0.793.21:0.01658)0.776.24:0.01148)0.971.26:0.04025)0.731.10:0.009
79,(Ult31517:0.03898,(Ult31518:0.02958,((Ult31515:0.00731,Ult31516:0.0039
9)0.912.45:0.02899,(Ult31513:0.04952,(UltrS453:0.0149,Ult31514:0.00413)0.
934.31:0.02341)0.843.26:0.01609)0.770.37:0.02757)0.902.45:0.03615)0.870.2
3:0.03165)0.942.36:0.04629,(((Ult31243:0.17885,((Ult29168:0.09592,(Ult291
65:0.0,Ult29166:0.0,Ult29167:0.0):0.26239)0.688.5:0.05272,((((((((KteRac
em:0.0114,(BacSOSP2:0.0,BacSOSP3:0.0):0.00372)0.953.23:0.02785,(UltCh477:
0.03245,(BacSOSP6:0.017,BacSOSP7:0.01021)0.734.17:0.02079)0.984.18:0.0556
7)0.833.27:0.01029,(BacSOSP4:5.4E-
4,BacSOSP5:0.01513)0.999.50:0.06013)0.679.5:9.6E-
4,((Ult29086:0.06938,((((Ult29083:0.00751,(Ult29081:0.01041,Ult29082:0.00
938)0.906.50:0.01896)1.000.174:0.10717,(Ult29113:0.00367,Ult29114:5.5E-
4)0.995.33:0.05803)0.785.32:0.01162,(Ult29087:0.04476,Ult29088:0.01358)0.
987.32:0.04559)0.785.33:0.02033,Ult29140:0.11193)0.714.10:0.00389)0.554.5

:0.00412,((((((UltCh483:0.07268,Ult29141:0.00589)0.845.34:0.01249,UltOr38
6:0.04758)0.269.2:0.01027,((UltCh480:0.01105,Ult29143:0.01153)0.886.40:0.
00745,((UltCh482:0.02266,(UltCh481:0.02663,Ult29145:0.04309)0.561.4:0.011
38)0.814.19:0.00835,Ult29144:0.01471)0.808.20:5.4E-
4)0.884.32:0.00943)0.740.23:0.00615,((((((Ult29163:0.06271,(Ult29161:0.03
258,Ult29162:0.08706)0.854.26:0.02489)0.992.31:0.08237,((Ult29158:0.1,Ult
29164:0.06606)0.893.38:0.04031,UltOr385:0.12304)0.804.22:0.01477)0.754.24
:0.01064,(((Ult29139:0.01453,(Ult29137:0.02764,Ult29138:0.01944)0.873.31:
0.00891)0.829.23:0.01411,(Ult29142:0.09054,Ult29160:0.0764)0.831.25:0.021
43)0.938.27:0.02881,(((Ult29100:0.0,Ult29101:0.0,Ult29102:0.0):0.09652,Ul
t29132:0.07022)0.946.33:0.04258,(Ult29159:0.0269,(UltCh485:0.01256,ChfBac
t2:0.03619)1.000.175:0.08431)0.963.33:0.03828)0.509.4:5.4E-
4)0.862.30:0.0092)0.739.22:0.00575,(Ult29150:0.03029,(Ult29147:5.5E-
4,(Ult29149:0.00744,Ult29148:0.00749)0.775.22:0.00362)0.973.15:0.03421)0.
958.34:0.03713)0.579.4:0.01156,(((Ult29157:0.00327,(Ult29156:0.00749,Ultr
S373:5.5E-4)1.000.176:5.4E-
4)0.880.39:0.05163,(Ult29152:0.08163,(Ult29153:0.03724,((UltrS371:0.01173
,(Ult29154:0.00817,(UltCh484:5.3E-
4,(Ult29155:0.02254,UltrS372:0.0184)0.803.18:0.0134)0.974.15:0.02791)0.80
4.23:0.00697)0.802.28:0.00809,Ult29151:0.00742)0.605.4:0.01353)0.492.6:0.
01612)0.856.24:0.04538)0.890.40:0.05213,(Ult29090:0.02555,(Ult29089:0.032
9,Ult29091:0.07821)0.955.27:0.06046)0.999.51:0.1402)0.844.35:0.02298)0.79
5.28:0.01351,Ult29146:0.02793)0.766.22:0.01631)0.914.34:0.02596,((UltrS37
0:0.05384,Ult29108:0.02611)0.992.32:0.06052,((((((Ult29122:0.07251,((Ult2
9119:0.08179,Ult29121:0.06346)0.687.8:0.01464,Ult29085:0.11295)0.879.34:0
.04666)0.134.5:5.5E-4,(Ult29123:0.04977,(Ult29134:0.04517,(Ult29124:5.5E-
4,(Ult29125:0.0,Ult29126:0.0):5.5E-
4)0.997.30:0.07828)0.938.28:0.03466)0.162.4:0.01201)0.790.25:0.01302,Ult2
9127:0.05715)0.445.7:0.03185,(Ult29128:0.13895,Ult29136:0.0572)0.857.38:0
.04483)0.734.18:0.01564,(UltCh479:0.11021,Ult29135:0.06395)0.655.7:0.0227
1)0.475.7:0.02444,Ult29133:0.1352)0.879.35:0.02287)0.134.6:0.01611)0.683.
11:0.02794,(((((Ult29095:0.04891,Ult29096:0.00897)0.946.34:0.03588,((Ult2
9092:0.02185,Ult29093:0.00922)0.844.36:0.0158,(Ult29094:0.04327,(Ult29098
:5.5E-
4,Ult29099:0.11017)0.989.26:0.06208)0.855.33:0.01845)0.138.5:0.0071)0.866
.34:0.02723,((Ult29105:0.11503,(Ult29106:0.00754,(Ult29103:0.00369,Ult291
04:5.5E-4)0.986.26:5.4E-4)0.993.22:5.3E-
4)0.986.27:0.08224,((Ult29131:0.13004,(Ult29129:5.5E-
4,Ult29130:0.01119)0.985.27:0.14799)0.979.31:0.13332,Ult29107:0.02862)0.8
79.36:0.07296)0.941.32:0.05405)0.901.45:0.04034,((Ult29084:0.10864,(Ult29
118:0.15752,(Ult29112:0.08294,Ult29120:0.23287)0.776.25:0.02366)0.433.7:0
.02158)0.862.31:0.02602,(Ult29097:0.13045,UltCh478:0.07482)0.888.41:0.022
88)0.328.3:0.01395)0.432.4:0.01388,KtdBacte:0.10535)0.958.35:0.03829)0.80
8.21:0.01343)0.935.37:0.01453)0.937.35:0.02604,(((Ult29116:0.0,Ult29117:0
.0):0.04345,BacSOSP8:0.10858)0.672.4:0.00553,(Ult29109:0.01174,Ult29110:0
.05351)0.998.36:0.09149)0.936.38:0.04042)0.849.27:0.01905,(BacSOSP1:0.074
43,(((Ult29051:0.07772,((Ult29041:5.5E-
4,(Ult29043:0.01754,Ult29050:0.03497)0.923.44:0.0178)0.813.17:0.01096,Ult
29042:5.4E-
4)0.900.35:0.01412)0.452.5:0.00936,(((((Ult29022:0.0037,(Ult29025:0.02265
,(((Ult29037:0.0,Ult29023:0.0,Ult29027:0.0,Ult29179:0.0):5.5E-
4,(Ult29026:0.00371,Ult29038:0.00745)0.631.7:5.5E-4)0.000.276:5.5E-
4,(Ult29024:0.00371,Ult29028:0.00371)0.870.24:5.5E-4)0.867.38:5.5E-
4)0.000.277:5.4E-

4)0.771.35:0.00489,((Ult29029:0.0,Ult29180:0.0):0.01869,(Ult29039:0.00873
,Ult29040:0.01016)0.969.27:0.0308)0.277.5:0.01258)0.908.37:0.02277,(Ult29
063:0.09303,((Ult29077:0.00376,Ult29078:0.00375)0.768.17:0.00403,((Ult290
62:0.0142,(Ult29060:0.00739,(Ult29059:0.00368,(Ult29061:0.0,Ult29178:0.0)
:5.5E-4)0.958.36:5.5E-
4)0.981.20:0.03927)0.999.52:0.0645,(Ult29074:0.01985,(Ult29073:0.01131,((
Ult29034:0.0,Ult29076:0.0):5.5E-4,Ult29075:0.00373)0.626.7:5.4E-
4)0.754.25:0.00384)0.877.50:0.01784)0.766.23:0.01077)0.835.26:0.01216)0.9
53.24:0.03347)0.963.34:0.03149,(((Ult29053:0.00206,((Ult29047:0.0,Ult2905
4:0.0):5.5E-
4,Ult26812:0.00372)0.988.36:0.0018)0.945.36:0.02784,((Ult29036:0.0,Ult290
45:0.0,Ult29046:0.0):5.5E-
4,Ult29044:0.00377)0.895.46:0.01675)0.995.34:0.05813,(Ult29035:0.0,Ult290
48:0.0,Ult29049:0.0):0.00851)0.457.6:0.00837)0.728.13:0.00361,((Ult29057:
0.00372,(Ult29058:0.00372,(Ult29055:0.0,Ult29056:0.0):5.5E-
4)0.493.7:5.5E-
4)0.871.33:0.00791,(Ult29052:0.05095,(((Ult29030:0.0,Ult29033:0.0):5.5E-
4,Ult29031:0.00372)0.822.17:5.4E-4,(((Ult29064:5.5E-
4,Ult29065:0.00366)0.970.32:0.00134,(Ult29072:0.00347,((Ult29066:0.0,Ult2
9067:0.0,Ult29068:0.0):5.4E-
4,(Ult29069:0.0,Ult29070:0.0):0.00718)0.177.6:0.00368)0.916.41:0.00275)0.
958.37:0.01476,(Ult29032:0.00372,Ult29071:0.01518)0.912.46:5.4E-
4)0.825.19:0.0037)0.971.27:0.02838)0.815.17:0.01006)0.987.33:0.02828)0.74
9.34:0.00418)0.000.278:5.4E-
4,Ult29079:0.06728)0.992.33:0.07416)0.760.21:0.035)0.000.279:0.00299,Ult2
9080:0.07347)0.956.31:0.04522,Ult29115:0.15038)0.201.3:0.01575,Ult29111:0
.07659)0.795.29:0.03506)0.959.43:0.10046)0.394.4:0.04623,(Ult14547:5.5E-
4,Ult14548:0.00356)0.999.53:0.1615)0.734.19:0.02595,(Ult31470:0.0879,((Ul
t31522:0.02715,Ult31521:0.01271)0.934.32:0.04772,(Ult31523:0.03895,Ult315
24:0.04733)0.670.5:0.01745)1.000.177:0.13876)0.056.2:5.4E-
4)0.871.34:0.04014)0.246.4:0.00293,(Ult29183:0.20234,((Ult31421:0.05333,(
Ult31410:5.5E-
4,((Ult31409:0.01591,((Ult31403:0.02239,(Ult31411:0.03989,(Ult31407:0.003
68,(Ult11745:5.5E-4,Ult31406:0.00363)0.806.13:0.00367)0.999.54:5.5E-
4)0.809.20:0.01467)0.920.40:5.4E-4,((Ult31408:0.03631,(Ult31401:5.3E-
4,Ult31402:0.01097)0.973.16:0.03338)0.890.41:0.0161,(Ult31404:0.00731,Ult
31405:5.5E-
4)0.901.46:0.01511)0.909.29:0.0157)0.953.25:0.01982)0.847.35:0.01084,Ult3
1422:0.01995)0.927.35:0.01495)0.957.30:0.04364)0.959.44:0.03773,(Ult31416
:5.4E-
4,((((Ult31417:0.01788,(Ult31414:0.04861,((UltCh511:0.024,UltCa386:0.0657
1)0.091.1:0.00301,Ult31415:0.04847)0.351.3:0.01746)0.812.16:0.00807)0.897
.26:5.5E-
4,(Ult31419:0.0,Ult31420:0.0):0.01914)0.796.17:0.00857,Ult31413:0.04156)0
.695.6:0.00645,(CddDivi9:0.12904,Ult31418:0.03507)0.266.2:0.01928)0.974.1
6:0.02788)0.998.37:0.09227)0.906.51:0.03734)0.764.28:0.01992)0.914.35:0.0
5598)0.902.46:0.03822)0.819.25:0.01526)0.851.32:0.00841,(((UltFir97:0.079
4,UltFir98:0.12194)0.925.40:0.05518,(((GeiGluta:0.05206,((Ult25897:0.0372
6,(TmgPhaeu:0.03595,(Ult25896:0.02096,(Ult25895:0.00369,Ult32003:5.5E-
4)0.912.47:0.02545)0.935.38:0.03071)0.863.35:0.02697)0.987.34:0.07001,((C
bxHydro:0.0,CbxSider:0.0):0.00444,CbxFerri:0.00672)0.951.33:0.03819)0.899
.33:0.03101)0.665.9:0.00659,Ult25884:0.06919)0.891.34:0.0155,(Ult25872:0.
09902,((Ult32144:0.01126,(TvnSpeci:0.00222,(TsdLitor:0.03245,(((UltThe46:0
.00378,(TrxSubte:5.5E-

4,(FrvFerri:0.01563,UltThe47:0.0073)0.915.32:0.01162)0.328.4:0.0037)0.871
.35:0.00736,CdgAceti:5.5E-
4)0.928.29:0.01549)0.747.21:0.00953)0.977.23:0.03686)0.884.33:0.03564,Ult
Ca415:0.19102)0.873.32:0.03739)0.479.7:0.00215)0.719.3:0.00881)0.792.33:0
.01826,((UltCl395:0.07751,(((((((Ult32183:0.0,UltDes33:0.0,UltThio3:0.0,
UltThe60:0.0):5.3E-4,(Ult32184:0.00745,UltPr100:5.3E-
4)0.837.20:0.00369)0.817.17:0.0036,UltDes34:5.5E-
4)0.982.15:0.07021,(Ult32185:0.06372,UltThe61:0.05676)0.157.3:0.02796)0.9
90.26:0.07569,((((Ult32163:0.04324,(Ult32168:0.18812,(Ult32166:0.0,Ult321
67:0.0):0.05836)0.991.29:0.13062)0.293.4:0.03451,((UltOr491:5.5E-
4,(UltThe58:0.01519,GtgSubte:0.00753)0.177.7:0.00359)0.964.40:0.03479,((G
tgAestu:5.5E-
4,ThgBacte:0.00368)0.759.32:0.01108,((PtgSpeci:0.02608,((PtgOlear:0.00376
,PtgMexic:5.5E-4)0.934.33:5.4E-4,((Ult32160:0.01911,Ult32161:5.5E-
4)0.772.32:0.00388,Ult32162:0.02343)0.634.3:0.00714)0.982.16:0.05934)0.96
5.34:0.05348,((((((Ult32149:0.00369,(Ult32146:0.00741,(UltThe50:0.07086,U
ltThe57:5.5E-4)0.842.33:0.00409)0.065.6:5.5E-4)0.274.2:5.4E-
4,Ult32151:5.5E-4)1.000.178:5.3E-
4,UltThe52:0.06888)0.829.24:0.00358,(((Ult32147:0.0,Ult32148:0.0,Ult32152
:0.0,Ult32153:0.0,Ult32155:0.0,Ult32156:0.0,Ult32157:0.0,Ult32158:0.0):5.
5E-
4,((UltThe49:0.00216,Ult32154:0.05978)0.999.55:0.00161,Ult32150:0.00369)0
.312.7:5.5E-4)0.807.16:5.5E-4,UltThe51:0.00736)1.000.179:5.3E-
4)0.897.27:0.00732,(UltThe56:5.5E-4,(UltThe54:0.11369,((UltThe55:5.5E-
4,UltThe53:0.04662)0.687.9:5.4E-4,UltThe48:0.03898)0.637.5:5.4E-
4)0.737.23:0.00356)0.989.27:0.0227)0.020.2:5.5E-
4,Ult32159:0.01483)0.973.17:0.04322)0.998.38:0.08471)0.853.25:0.02258)0.9
48.43:0.04351)0.160.1:0.01445,((MngPiezo:0.0025,ThglsSt2:0.02441)0.604.5:
0.00804,(MngCamin:0.0109,(MngOkina:5.5E-
4,(MngSpeci:0.01153,MngHydro:0.01154)0.789.19:0.00709)0.982.17:0.03362)0.
749.35:0.00416)0.994.32:0.09393)0.486.6:0.01869,((Ult32164:0.0,Ult32165:0
.0):0.0034,UltThe59:5.2E-
4)1.000.180:0.16524)0.890.42:0.04458)0.878.35:0.03642,(((Ult32182:5.4E-
4,(KosSpeci:0.03237,(Ult32181:0.04376,(((Ult32171:0.00369,((Ult32169:0.0,
Ult32170:0.0,Ult32172:0.0,Ult32175:0.0,Ult32177:0.0,Ult32176:0.0):5.5E-
4,Ult32174:0.00369)0.579.5:5.3E-4)0.555.4:5.5E-
4,(Ult32173:0.00372,(Ult32178:0.00369,Ult32179:0.00372)0.795.30:0.00373)0
.942.37:0.01122)0.998.39:0.06825,Ult32180:0.06216)0.654.4:0.01968)0.912.4
8:0.02854)0.994.33:0.0517)1.000.181:0.10781,(UltSynt7:0.06467,Ult32186:0.
01264)0.952.33:0.04463)0.465.5:0.02474,(((Ult32202:0.01161,((((TeiMelan:0
.0,TeiSpeci:0.0):0.0037,((TeiSpec2:0.00369,(UltThe63:0.0,Ult32201:0.0):5.
5E-4)0.991.30:0.02273,TeiFerri:0.00718)0.897.28:5.3E-
4)0.793.22:0.0037,(Ult32200:0.01125,TeiAtlan:5.4E-
4)0.801.22:0.00373)0.731.11:0.0024,UltThe62:0.0412)0.758.30:0.00486)0.299
.4:0.0054,((FvdNodos:0.02458,((FvdGondw:0.02904,FvdSpeci:0.0055)0.994.34:
0.04146,(FvdSpec2:0.01897,(ThglsSt3:5.4E-
4,(Ult32198:0.0074,Ult32199:0.01508)0.780.28:0.00373)0.755.28:5.4E-
4)0.918.34:0.01531)0.762.33:0.00764)0.794.23:0.01752,((UltFerv2:0.00389,U
ltFervi:0.0036)0.861.41:0.01461,((Ult32187:0.0,Ult32188:0.0,Ult32196:0.0,
Ult32197:0.0):5.5E-4,(Ult32194:5.5E-
4,((Ult32190:0.00368,(Ult32189:0.00368,Ult32195:5.5E-4)0.730.13:5.5E-
4)0.931.36:0.01139,(Ult32192:5.4E-
4,Ult32193:0.14519)1.000.182:0.00702)0.974.17:7.9E-
4)1.000.183:0.00281)0.829.25:0.01287)0.958.38:0.05372)0.998.40:0.08768)0.

829.26:0.03879,(((TtgSpeci:5.4E-4,(TtgHypog:0.00369,Ult32203:5.5E-
4)0.965.35:0.01505)0.745.27:0.00529,(Ult32206:0.02066,(Ult32205:5.4E-
4,TtgLetti:0.03108)0.191.2:0.01428)0.992.34:0.0411)0.923.45:0.02169,(TtgT
herm:5.4E-4,((Ult32207:0.01856,(TtgNeapo:5.5E-
4,UltThe65:0.00368)0.945.37:5.4E-4)0.994.35:0.04967,(Ult32204:5.5E-
4,UltThe64:5.5E-
4)0.966.39:0.02764)0.209.3:0.01594)0.906.52:0.01291)0.840.31:0.01571)0.95
2.34:0.03151)0.782.31:0.01054)0.997.31:0.08734,((UltSymb5:0.03201,(Ult258
45:0.10079,(UltSymbi:0.04615,(((((Ult29169:0.0,Ult29170:0.0,Ult29171:0.0)
:0.00961,Ult29172:0.01012)0.991.31:0.04568,(ChfBact3:0.03827,(Ult29176:0.
01657,Ult29177:0.01973)0.993.23:0.05339)0.844.37:0.018)0.623.2:5.5E-
4,((Ult29175:0.02919,(Ult29174:0.0433,UltrS374:0.03896)0.143.4:0.01489)0.
735.16:0.00726,Ult29173:0.09433)0.403.3:0.01381)1.000.184:0.12932,(Ult142
63:0.04219,((UltAc140:0.0,Ult14858:0.0):0.03519,((UltAc138:0.00375,UltAc1
39:0.00743)0.759.33:0.00804,(Ult14262:0.05263,(Ult14260:0.00368,Ult14261:
0.0073)0.904.34:0.01922)0.949.38:0.03455)0.872.41:0.05222)0.910.51:0.0474
6)0.874.48:0.03941)0.971.28:0.0697)0.239.3:0.02334)0.851.33:0.02497)0.993
.24:0.09202,(((Ult32272:0.0583,(Ult32229:0.04953,(UdnCyto4:0.00349,(Ult32
228:0.0281,Ult32227:0.00694)0.155.4:0.00856)0.886.41:0.06373)1.000.185:0.
22017)0.946.35:0.07442,(Ult31643:0.01725,((UltCopr6:5.4E-
4,UltCop13:0.02281)0.781.28:0.00452,((UltCop12:0.10988,(Ult31624:5.5E-
4,Ult31638:0.01509)0.985.28:5.4E-4)0.970.33:0.02333,(((Ult31639:5.5E-
4,UltCopr3:0.01095)0.868.25:5.4E-4,((Ult27708:0.00368,(Ult31648:5.5E-
4,(((Ult31644:5.3E-4,((Ult31655:5.5E-
4,((Ult31627:0.0,Ult31633:0.0,Ult31634:0.0,Ult31646:0.0):5.5E-
4,Ult31632:5.5E-4)0.000.280:5.5E-4)0.553.2:5.5E-
4,Ult31656:0.0037)0.893.39:0.00366)0.407.3:0.00374,(((Ult27485:0.0,Ult316
23:0.0,Ult31625:0.0,UltCopr5:0.0,Ult31626:0.0,Ult31635:0.0,Ult31636:0.0,U
lt31637:0.0,Ult31641:0.0,Ult31642:0.0,Ult31645:0.0,Ult31647:0.0,Ult31650:
0.0,Ult31651:0.0):5.5E-4,Ult31652:0.0037)0.210.6:5.5E-4,(Ult31640:5.5E-
4,(UltCopr4:0.01078,Ult31653:5.5E-4)0.886.42:5.3E-4)0.780.29:5.4E-
4)0.879.37:0.0036)1.000.186:5.5E-4,Ult31654:0.0037)0.414.2:5.5E-
4)0.000.281:5.5E-4)0.336.3:5.4E-4,UltCopr2:5.5E-4)0.917.31:5.5E-
4)0.805.21:0.00371,(((UltCop10:0.01541,(UltCopr7:0.04471,CpoPlate:0.03662
)0.802.29:0.00876)0.914.36:0.01159,(Ult27483:0.05253,Ult31657:0.04799)0.7
47.22:0.00385)0.079.1:5.4E-
4,(UltCop14:0.01932,((UltCop11:0.03166,UltCopr9:0.02328)0.734.20:0.00395,
(UltCop15:0.03263,UltCopr8:0.04676)0.009.3:5.4E-
4)0.747.23:0.00408)0.886.43:0.00797)0.783.31:0.00365)0.954.38:0.00234)0.9
57.31:5.4E-
4)0.973.18:0.06432)0.998.41:0.11006)0.386.5:0.01548,Ult28955:0.19147)0.85
6.25:0.03242)0.549.6:0.01363)0.106.2:0.00184,(UltCh455:0.14549,UltCh454:0
.03697)0.995.35:0.11282)0.884.34:0.02012)0.477.3:0.00803,(((UltB4473:0.13
127,(((((Ult13176:0.03635,Ult13175:0.09291)0.981.21:0.06714,(Ult32083:0.0
5237,Ult32085:0.12479)0.884.35:0.04736)0.927.36:0.04149,(Ult13167:0.12824
,(((Ult13171:0.01974,((Ult13173:0.00732,Ult13174:0.00762)0.893.40:0.01893
,(Ult13178:0.02421,(Ult13177:0.01006,Ult28545:0.07654)0.995.36:0.06737)0.
939.34:0.05714)0.894.28:0.02978)0.638.3:0.031,Ult13172:0.03985)0.803.19:0
.01917,(Ult13170:0.01792,(Ult13169:0.0202,Ult13168:0.05825)0.104.5:0.0073
2)0.809.21:0.01595)0.239.4:0.02264)0.000.282:0.00839)0.709.6:0.00984,Ult3
2084:0.07845)0.966.40:0.04475,(Ult28857:0.1481,(((Ult13054:0.00688,Ult130
55:0.00811)0.713.10:0.00825,((Ult13062:0.02178,(((Ult13059:5.2E-
4,(((UltCan88:0.0,UltCan89:0.0,Ult13060:0.0):5.4E-
4,Ult13057:0.04254)0.393.4:0.00741,Ult13061:0.00368)0.999.56:5.3E-

4)0.829.27:0.00369,Ult13058:5.5E-
4)0.882.40:0.01439,(UltOr210:0.00591,UltOr211:0.01723)0.809.22:0.01794)0.
526.1:0.00705)0.808.22:0.01099,Ult13056:0.02124)0.799.29:0.00847)0.781.29
:0.01662,(UltCan87:0.02931,(Ult13047:0.01982,((((Ult13031:0.00369,((Ult13
050:0.01906,(Ult13051:0.01519,(Ult13052:0.00374,Ult13053:0.01502)0.870.25
:0.00743)0.756.36:0.00379)0.930.26:0.01127,((Ult13034:0.00387,(((((Ult130
48:0.0,Ult13049:0.0):0.0189,Ult13025:0.0074)0.478.3:5.5E-
4,(UltCan86:0.0112,(((((UltGram3:0.0,UltHydr7:0.0):5.5E-
4,Ult13045:0.0074)0.874.49:0.00369,((((Ult13043:0.01127,UltCan80:0.00742)
0.158.2:5.5E-4,UltCan85:0.00741)0.917.32:5.5E-4,BenMine2:5.5E-
4)0.000.283:5.5E-4,Ult13046:5.5E-4)0.907.36:5.5E-4)0.901.47:5.4E-
4,(UltGram2:5.4E-
4,Ult13044:0.02689)0.438.4:0.00369)0.812.17:0.00365,(Ult13037:0.00739,((B
enMiner:0.0,Ult13065:0.0):5.5E-
4,(Ult13032:0.00392,(((Ult13036:0.00382,Ult13038:0.00371)1.000.187:0.0018
9,Ult13035:0.00392)0.000.284:5.3E-4,Ult13033:0.00402)0.328.5:5.4E-
4)1.000.188:7.1E-4)0.915.33:5.4E-4)0.786.35:0.00744)1.000.189:5.4E-
4)0.371.6:5.5E-4)0.437.3:5.5E-4,UltCan84:5.5E-4)0.437.4:5.5E-
4,Ult13026:5.5E-4)0.209.4:5.5E-4)0.423.3:5.4E-4,Ult13027:5.5E-
4)0.937.36:5.5E-4)0.840.32:0.00369)0.446.3:5.5E-4,Ult13028:5.5E-
4)0.210.7:5.5E-4,((Ult13040:0.00371,(Ult13041:0.00369,Ult13066:5.5E-
4)0.814.20:0.0037)0.809.23:0.0037,Ult13042:0.0038)0.237.6:5.5E-
4)0.996.21:5.4E-4,((UltCan81:5.5E-4,(UltCan82:0.0,UltCan83:0.0):5.5E-
4)0.833.28:0.00369,(Ult13039:0.00368,(Ult13064:0.00372,Ult13029:0.00381)0
.382.4:5.5E-4)0.411.4:5.5E-
4)0.853.26:0.00367)0.890.43:0.01081)0.838.28:0.01066)0.737.24:0.02914)0.9
75.25:0.07354)0.832.22:0.03293)0.910.52:0.04893)0.828.22:0.02107,(UltB526
7:0.06442,((UltB5265:0.02748,UltB5266:0.00591)0.217.2:0.00384,(UltB5264:5
.4E-
4,(UltB5261:0.01919,(UltB5263:0.01157,UltB5262:0.03188)0.844.38:0.00764)0
.951.34:0.01917)0.930.27:0.02094)0.862.32:0.0198)0.958.39:0.04683)0.806.1
4:0.01738,(((((UltCh470:0.14343,Ult32134:0.16742)0.862.33:0.03647,Ult1310
6:0.09757)0.779.29:0.01228,(((UltCal24:0.00372,(UltSp260:0.0,UltSp261:0.0
):0.00378)0.713.11:0.01801,(Ult32092:0.00933,(Ult32093:0.04263,(Ult27476:
0.02319,Ult32091:5.4E-
4)0.941.33:0.02054)0.850.34:0.02745)0.992.35:0.07562)0.999.57:0.11271,((U
lt32088:0.03899,(Ult32087:0.01477,((Ult32086:0.0,Ult32090:0.0):5.5E-
4,Ult32089:5.5E-4)0.989.28:5.4E-4)0.981.22:5.4E-
4)0.981.23:0.04794,(UltEu136:0.06376,(UltrdL22:0.02704,UltFi106:5.5E-
4)0.953.26:0.05357)0.545.8:0.02768)0.942.38:0.05664)0.981.24:0.06158)0.29
4.3:0.01813,(Ult32082:0.0748,UdnBac87:0.07417)1.000.190:0.20383)0.868.26:
0.03645,((((Ult32110:0.11792,(Ult32112:0.00451,(Ult32109:0.01187,((Ult321
07:0.01539,((Ult32106:5.5E-
4,Ult32108:0.01976)0.764.29:0.00351,Ult32105:0.01537)0.912.49:0.01168)0.9
05.31:0.0112,(Ult32103:0.01497,Ult32104:0.00369)0.082.3:5.4E-
4)0.757.21:0.00321)0.998.42:0.07158)0.512.2:0.04635)0.954.39:0.05927,((((
(UltThe92:0.02925,Ult32293:0.02539)0.978.21:0.03791,((((VenStagn:0.03108,
(Ult32279:0.02659,(PpoGuaym:5.4E-
4,((Ult32280:0.00718,((((UltHyd25:0.00369,((Ult32265:0.0,Ult32266:0.0,Ult
Hyd26:0.0,UltHyd27:0.0,SfhSpeci:0.0,Ult32267:0.0,UltSulf9:0.0,UltSul11:0.
0,Ult32271:0.0):5.5E-4,(((UltAqui8:0.01523,Ult32269:5.5E-
4)0.851.34:0.00369,(UltHyd28:0.00202,(UltHyd23:0.01113,UltHyd22:0.06169)0
.851.35:0.00717)0.903.33:0.00214)0.311.1:5.5E-
4,Ult32268:0.00369)0.427.4:5.5E-4)0.350.5:5.5E-4)0.574.2:5.1E-

4,UltHyd24:0.01127)0.175.1:5.4E-
4,(UltSul10:0.01128,Ult32270:0.03941)0.902.47:0.01124)0.929.38:0.0343,(((
UltHyd14:5.5E-4,((UltHyd18:0.00369,(UltHyd15:5.5E-4,(((HrgAcido:5.5E-
4,(HrgAcid2:5.5E-4,HrgSpeci:5.5E-4)0.919.44:0.00369)0.612.7:5.5E-
4,UltHyd17:0.00745)0.833.29:0.0037,((Ult32245:0.00331,Ult32244:0.05979)0.
847.36:0.00737,UltHyd19:0.00208)0.901.48:0.00205)0.890.44:5.5E-
4)0.598.2:5.5E-
4)1.000.191:0.00205,UltHyd20:0.01122)1.000.192:0.00162)0.304.5:5.4E-
4,UltHyd16:0.01515)0.997.32:0.09525,((UltAqui5:0.00375,HdtSpec2:0.01117)1
.000.193:5.4E-
4,(((HdtSpeci:0.00368,((((UltAqui7:0.0074,((((Ult32256:0.0073,(((AqxAeoli
:0.00754,(AfclsSt2:5.4E-
4,AqxPyrop:0.00369)0.702.3:0.00366)0.804.24:0.00662,(AqxSpeci:5.4E-
4,(HrnSpec2:0.01126,(HrnCaldi:0.01107,HrnSpeci:0.03193)0.874.50:5.4E-
4)0.357.5:0.00372)0.970.34:0.024)1.000.194:0.05728,Ult32262:5.5E-
4)0.736.17:0.01913)0.511.4:0.00736,TecSpeci:0.0186)0.813.18:5.4E-
4,((((BactEM17:0.00744,(UltEu137:0.00748,(((UltThe68:0.01502,(Ult32248:0.
00749,(UltThe73:0.01124,((UltThe69:0.0,Ult32247:0.0,UltThe74:0.0,Ult32249
:0.0):5.5E-
4,(((UltThe72:0.00371,Ult32253:0.00196)1.000.195:0.00171,((UltThe79:0.003
7,UltThe70:0.00741)0.995.37:5.5E-4,Ult32246:0.00369)0.282.2:5.5E-
4)0.235.5:5.5E-4,UltThe76:0.00369)0.611.2:5.5E-4)0.921.45:5.5E-
4)0.756.37:5.3E-4)0.405.7:0.00369)0.918.35:5.3E-4,UltThe71:5.4E-
4)0.912.50:0.00369,UltAqui4:5.5E-4)0.678.9:5.5E-
4)0.836.22:0.0037)0.641.7:5.5E-
4,(Ult32254:0.07849,(Ult32250:0.0074,(UltThe77:0.0,UltThe78:0.0):5.5E-
4)0.952.35:5.5E-4)0.819.26:0.00366)0.451.7:5.4E-
4,Ult32255:0.01121)0.811.22:0.00367,Ult32252:0.01129)0.773.30:0.00374)0.8
42.34:0.0037,(Ult32261:0.00369,UltHyd21:5.5E-4)0.825.20:5.4E-
4)0.932.32:0.00749)0.383.3:5.4E-
4,((TmxThiop:0.00745,(Ult32259:0.0,Ult32260:0.0):0.00747)0.367.4:5.5E-
4,(Ult32258:0.0,HdtSubte:0.0):5.5E-4)0.904.35:0.00369)0.863.36:5.3E-
4,Ult32251:0.0112)0.762.34:0.00364,Ult32257:0.01136)0.964.41:0.01527)0.75
0.17:0.00368,(UltAqui6:0.02321,UltThe81:0.01942)0.951.35:0.01959)0.884.36
:0.00743,(AfclsStr:0.00369,UltThe80:0.01902)0.648.7:5.4E-
4)0.909.30:0.00322)0.656.7:0.03915)1.000.196:0.12148)0.409.2:0.01105)0.93
3.40:0.01202,(((Ult32277:0.00371,(Ult32276:0.0113,SfhSubte:5.5E-
4)0.578.3:5.3E-
4)0.774.41:0.00374,((Ult32278:0.00738,(SfhRodma:0.0,UltSul12:0.0,SfhKrist
:0.0,AfclsSt3:0.0):5.5E-4)0.996.22:5.5E-
4,UdnEuba8:0.0308)0.813.19:0.00362)0.747.24:0.00377,((PpoSpeci:5.5E-
4,UltProk9:0.00369)0.971.29:0.01926,((PpoSpec5:0.0037,((PpoSpec4:0.00367,
(((AfclsSt4:0.0,PpoSpec2:0.0):5.4E-4,PpoSpec3:0.00369)0.906.53:5.4E-
4,PpoHydro:0.01122)0.807.17:0.00744)1.000.197:5.4E-
4,PpoSpec6:0.00753)0.919.45:0.00747)0.792.34:5.5E-
4,Ult32281:0.03061)0.300.4:0.00368)0.967.26:0.01923)0.873.33:0.00753)0.76
1.32:0.00363)0.915.34:5.4E-
4)0.434.4:0.01393)0.825.21:0.01484,PpoSpec7:0.01094)0.995.38:0.06821,((((
(Ult32283:0.00369,Ult32282:0.01947)0.035.3:5.5E-
4,(((TvbAmmon:0.00742,TvbGuaym:5.4E-
4)0.788.23:0.00359,(BlmLitho:0.01507,(AfclsSt5:0.01524,Ult32285:0.00746)0
.253.5:5.5E-4)0.514.4:0.00752)0.774.42:0.00382,(Ult32284:5.5E-
4,TvbRuber:0.00745)0.783.32:0.00372)0.929.39:0.0075)0.983.19:0.0199,UltAq
u10:5.4E-4)0.795.31:0.00393,(UltAqui9:5.5E-

4,UltPr101:0.00741)0.942.39:0.01119)0.704.5:0.00283,(UltEu139:5.5E-
4,UltEu138:0.01513)0.786.36:0.00473)0.997.33:0.09402)0.544.2:0.01689,TslT
akai:0.0186)0.854.27:0.0129)0.580.2:0.00766,((UltThe89:0.03091,(TdtIndic:
0.00696,(UltThe91:0.0074,(TdtSpeci:5.5E-4,UltThe90:5.5E-4)0.686.10:5.5E-
4)0.886.44:0.00803)0.401.5:9.5E-
4)0.867.39:0.0224,((UltThe75:0.06445,(((Ult32264:0.0,Ult32275:0.0,UltGeot
3:0.0,UltThe85:0.0,UltGeot5:0.0,UltThe86:0.0):5.5E-4,(((UltThe84:5.4E-
4,Ult32288:0.01505)0.719.4:0.00747,((Ult32287:0.0037,(UltGeot4:0.00369,(U
ltGeot6:0.00369,UltGeot2:0.00741)0.563.2:5.5E-4)0.576.5:5.5E-
4)0.772.33:0.00372,UltThe83:0.01531)0.764.30:0.0037)0.998.43:5.5E-
4,TdlSpeci:0.00369)0.888.42:5.5E-4)0.751.15:5.4E-4,Ult32286:5.5E-
4)0.949.39:5.5E-
4)0.691.6:0.0165,((((Ult32291:0.00258,Ult32292:0.08549)0.954.40:0.00118,U
ltThe88:0.00753)0.778.29:0.00553,(Ult32290:0.01511,(UltThe87:0.00751,Ult3
2289:0.01137)0.747.25:0.00389)0.771.36:0.00608)0.979.32:0.02344,(TdbHydro
:0.00372,(TdbHvera:0.01139,TdbCommu:0.00371)0.877.51:0.00751)0.886.45:0.0
0775)0.682.6:0.0021)0.975.26:0.05059)0.988.37:0.073)0.925.41:0.04499,((Ul
t15411:0.05526,Ult15412:0.04509)0.988.38:0.08891,Ult26724:0.16835)0.803.2
0:0.02252)0.640.3:0.03108,((Ult28897:0.10305,(Ult32133:5.5E-
4,(Ult32132:0.00751,Ult32131:0.00757)0.904.36:0.011)1.000.198:0.15208)0.1
76.7:0.02535,UltB4326:0.07112)0.647.3:0.00363)0.844.39:0.02428)0.954.41:0
.04025,(((((AthAraba:0.03975,ShlShrif:0.14053)0.969.28:0.0571,((UltOr226:
0.13855,(HrsSilve:0.00679,((UltrdLo6:0.0,UltrdLo7:0.0):5.4E-
4,Ult13131:0.00748)0.907.37:0.02027)0.953.27:0.06972)0.969.29:0.07332,(Hl
xOreni:0.04386,((UltHala2:0.03155,(((Ult13113:0.09052,(UltrdLo4:0.0384,((
UltOr221:0.00368,((UltOr219:0.0,UltOr220:0.0,Ult13109:0.0,Ult13110:0.0):5
.5E-4,(Ult13111:0.00369,Ult13112:0.00369)0.685.7:5.5E-4)1.000.199:5.5E-
4)0.998.44:0.0631,((UltOr218:8.3E-
4,UltOr217:0.022)0.987.35:0.03976,(HnbSalsu:0.01096,((((HnbSacc2:0.0,HnbL
acus:0.0):5.5E-4,HnbSacch:0.0037)0.428.4:5.5E-
4,(((UltOr212:0.02231,(UltOr214:0.03519,UltOr216:0.03483)0.914.37:0.01141
)0.847.37:5.4E-
4,(Ult13108:0.01904,UltOr213:0.05249)0.755.29:0.00376)0.723.16:0.0037,((U
ltOr215:0.0,HnbSpeci:0.0,HnbAcete:0.0,UltrdLo3:0.0):5.5E-
4,Ult13107:0.00744)1.000.200:5.5E-
4)0.898.40:0.00357)0.805.22:0.00717,HrbBacte:5.4E-
4)0.928.30:0.0289)0.990.27:0.04392)0.777.31:0.01189)0.820.12:0.01241)0.86
2.34:0.01304)0.911.40:0.0211,(UltHalan:0.00694,(Ult13114:0.01546,Ult13115
:0.02289)0.167.3:0.00881)0.957.32:0.02703)0.945.38:0.02616,(((Ult13120:0.
01117,(Ult13121:0.00369,(Ult13119:5.5E-
4,(Ult13118:0.00741,Ult13122:0.00369)0.946.36:5.5E-4)0.903.34:5.5E-
4)0.768.18:5.4E-4)0.872.42:0.00749,(Ult13123:0.00742,UltComp4:5.5E-
4)0.956.32:0.01519)0.815.18:0.00941,(Ult13124:0.02399,(HaoCellu:0.03871,(
Ult13125:0.00369,UltOr222:0.0037)0.770.38:0.00435)0.855.34:0.01598)0.699.
12:0.01442)0.538.3:0.01205)0.724.12:0.00689)0.829.28:0.01911,(HnlBacte:0.
0277,(Ult13117:0.0151,Ult13116:0.00759)0.999.58:0.09613)0.527.3:0.01585)0
.955.28:0.04485)0.943.33:0.04282)0.838.29:0.0193)0.844.40:0.02231,(((Ultr
dLo5:0.0462,((HbtBacte:0.03541,HnmTunis:0.00748)0.993.25:0.04726,(HbcHalo
2:5.4E-
4,HbcHalob:0.06188)0.624.10:0.0218)0.893.41:0.04423)0.708.9:0.00734,(((Hl
nLacu2:0.00687,(UltHala3:5.5E-
4,UltHala4:0.00694)0.958.40:0.02328)0.883.41:0.01243,(Ult13127:5.3E-
4,(Ult13126:0.01912,(HlnLacun:0.0075,HlnSalin:5.4E-
4)0.899.34:0.01113)0.966.41:0.01912)0.760.22:0.00352)0.978.22:0.03595,(Nr

nAceti:0.05029,((UltOr224:0.03199,(UltOr223:0.00259,(((Ult13128:0.06129,U
ltHalob:0.00384)0.553.3:5.3E-
4,UltOreni:0.01497)0.956.33:0.02947,OreSalin:0.05322)0.813.20:0.00817)0.9
96.23:0.037)0.614.3:0.00331,(OreMaris:5.4E-
4,OreSivas:0.03512)0.767.26:0.00405)0.820.13:0.01003)0.988.39:0.04411)0.8
53.27:0.02098)0.861.42:0.02551,(UltEps37:0.0079,(ThglsStr:0.00768,UltB952
3:0.01934)0.765.19:0.00734)1.000.201:0.16432)0.925.42:0.03097)0.858.29:0.
02133,((UltB9517:0.04689,(UltB9516:0.06271,(UltB9515:0.06156,((UltB9513:5
.4E-
4,(UltB9512:0.01166,UltB9514:0.01933)0.748.25:0.0033)0.826.23:0.00863,(((
UltB9519:0.02037,UltB9520:0.03389)0.889.34:0.01537,(UltB9506:5.5E-
4,(((UltB9507:0.0,UltB9509:0.0):5.5E-
4,UltB9508:0.00369)0.953.28:0.01514,(UltB9510:0.00399,((UltB9511:0.0,UltB
9522:0.0):0.00711,UltB9521:5.5E-
4)0.239.5:0.00369)0.794.24:0.00348)0.820.14:0.00739)0.698.6:0.00425)0.733
.15:0.01066,UltB9518:0.01274)0.901.49:0.0192)0.815.19:0.02004)0.531.1:0.0
3373)0.946.37:0.0653)0.996.24:0.09962,((Ult12740:0.03088,(Ult12738:0.0091
8,Ult12739:0.01731)0.920.41:0.02325)0.994.36:0.05971,((Ult12741:0.03146,U
ltCan56:0.03234)0.837.21:0.01465,(Ult12744:0.02362,((Ult12742:0.00366,Ult
12743:5.4E-
4)0.852.31:0.01198,(UltMar63:0.04009,(Ult12745:0.01206,Ult12746:0.01502)0
.958.41:0.02785)0.869.32:0.01221)0.957.33:0.02342)0.958.42:0.02644)0.440.
5:5.4E-
4)0.950.32:0.0512)0.909.31:0.03819)0.975.27:0.05138,(((((Ult17031:0.09172
,(Ult16981:0.02387,(((Ult16993:0.01215,(Ult16992:0.02214,((Ult16990:0.0,U
lt16991:0.0):0.0157,((Ult16987:5.4E-
4,Ult16986:0.01122)0.889.35:0.00747,((((Ult16984:5.4E-
4,Ult16985:0.00744)0.562.6:5.5E-
4,(Ult16982:0.01913,Ult16983:0.00748)0.879.38:0.00745)0.844.41:0.00368,Ul
t16989:5.5E-4)0.770.39:5.5E-
4,Ult16988:0.00744)0.811.23:0.00364)0.874.51:0.01125)0.118.2:0.00781)0.96
5.36:0.03277)0.614.4:0.01079,(Ult16980:0.00371,UltrS299:0.00391)0.871.36:
0.01301)0.786.37:0.00851,((((((Ult17002:0.00739,Ult17010:5.5E-
4)0.836.23:0.0037,((Ult17003:0.0,Ult17006:0.0):0.0037,(Ult17009:0.0843,Cd
dDivi4:5.5E-4)0.838.30:0.00367)0.807.18:0.00374)1.000.202:5.5E-
4,((Ult17001:0.00369,(Ult17008:0.00741,Ult17004:0.00369)0.142.2:5.5E-
4)1.000.203:5.4E-
4,((CddDivi5:0.06523,Ult17005:0.02204)0.864.39:0.01858,(((Ult16996:5.4E-
4,((Ult16999:0.0,Ult16998:0.0):5.5E-
4,Ult16995:0.0037)0.945.39:0.00747)0.740.24:0.00365,Ult31629:5.5E-
4)0.238.3:0.00367,(Ult17000:5.5E-4,Ult16997:0.00368)0.923.46:5.4E-
4)0.955.29:0.01576)0.710.8:0.00705)0.826.24:0.00334)0.750.18:0.01137,(((U
lt16977:0.0,Ult16978:0.0):5.5E-
4,Ult16979:0.00368)0.999.59:0.05384,(UltCa115:0.01572,Ult17007:0.04766)0.
931.37:0.02494)0.917.33:0.0165)0.976.24:0.03053,(UltCa116:0.05525,(UltCa1
17:0.05512,(((UltCa111:0.02984,Ult16976:0.01689)0.300.5:0.01063,((UltCa11
3:0.02198,UltCa114:0.02891)0.759.34:0.01509,UltCa112:0.00534)0.810.22:0.0
1492)0.763.32:0.00815,Ult16994:0.03309)0.853.28:0.01369)0.833.30:0.0127)0
.822.18:0.00808)0.929.40:0.01789,(((UltCa106:0.01539,(Ult16943:0.02365,Ul
t16942:0.0234)0.886.46:0.01163)0.784.23:0.0038,(((Ult16923:0.0073,Ult1692
4:0.00772)0.979.33:0.02168,(Ult16922:0.01046,(Ult16921:5.5E-
4,Ult16920:0.01509)0.792.35:0.00565)0.814.21:0.00835)0.874.52:0.01599,(((
(UltCa103:0.01453,(Ult16936:5.4E-
4,(((UltCa104:0.05497,(Ult16938:0.00369,(((Ult16940:5.5E-4,Ult16941:5.5E-

4)0.911.41:5.5E-4,Ult16937:0.00367)0.427.5:5.5E-
4,Ult16939:0.0112)0.803.21:0.00368)0.612.8:5.4E-
4)0.989.29:0.02274,Ult16935:5.5E-4)0.841.26:5.5E-
4,Ult16933:0.00739)0.439.5:0.00368)0.849.28:5.3E-
4)0.894.29:0.00776,Ult16934:0.00344)0.743.29:0.00399,((Ult16930:0.01904,(
(UltOr266:0.0765,Ult16960:0.01579)0.348.5:0.00771,Ult16953:0.02064)0.938.
29:0.0227)0.716.7:0.00381,(Ult16931:0.0074,(Ult16932:0.0113,(UltOr267:0.0
0741,UltOr268:5.5E-
4)0.990.28:0.02731)0.859.36:0.00768)0.912.51:0.01151)0.907.38:0.01061)0.7
39.23:0.00473,((Ult16929:0.00764,(UltCa102:0.01447,Ult16928:0.00521)0.920
.42:0.01448)0.964.42:0.02952,(Ult16927:0.05503,Ult16975:0.03315)0.861.43:
0.01679)0.948.44:0.02807)0.881.37:0.02233)0.547.2:0.00916)0.868.27:0.0073
,(((((UltCa109:0.03542,Ult16974:0.01911)0.863.37:0.00768,(Ult16970:0.0267
4,(Ult16973:0.0112,(Ult16971:0.00369,Ult16972:5.5E-
4)0.926.38:0.00744)0.839.25:5.4E-4)0.990.29:0.02299)0.766.24:5.4E-
4,(UltCa110:0.012,(Ult16958:0.0075,Ult16959:0.00743)0.982.18:0.02689)0.77
1.37:0.01119)0.865.34:0.00341,(((Ult16954:0.01155,Ult16956:0.01547)0.952.
36:0.01994,(Ult16955:0.02225,(Ult16952:0.00755,Ult16951:0.01913)0.865.35:
5.4E-
4)0.200.2:0.00749)0.732.5:0.00332,((Ult16949:0.01502,((Ult16944:5.5E-
4,((Ult16946:5.5E-
4,Ult16948:0.01143)0.844.42:0.00369,Ult16945:0.00369)0.861.44:5.4E-
4)0.999.60:5.4E-
4,(Ult16947:0.00762,UltCa105:0.02287)0.770.40:0.00359)0.932.33:0.01134)0.
879.39:5.4E-
4,Ult16950:0.00369)0.960.37:0.01142)0.879.40:0.00799)0.778.30:0.00393,((U
lt16966:0.03136,(Ult16969:0.00394,((Ult16925:0.01324,Ult16926:0.01784)0.9
55.30:0.0258,(UltCa108:0.01214,Ult16961:0.04136)0.485.5:0.00685)0.798.24:
0.00824)0.886.47:0.01138)0.775.23:0.00371,(Ult16967:0.00744,(Ult16957:5.5
E-4,(((Ult16965:5.4E-
4,UltCa107:0.01112)0.247.2:0.00585,(Ult16963:0.00401,(Ult16962:0.00394,Ul
t16964:0.01388)0.453.5:0.0047)0.766.25:0.00512)0.838.31:0.01152,Ult16968:
0.01338)0.652.4:0.00902)0.797.24:0.01129)0.761.33:0.00381)0.778.31:0.0037
)0.856.26:5.4E-
4)0.851.36:0.01137)0.884.37:0.01729)0.863.38:0.01695)0.990.30:0.06655)0.9
07.39:0.03795,((Ult17025:0.02591,UltCa122:0.00779)0.884.38:0.01685,((Ult1
7022:0.04691,((((Ult17012:0.01531,UltCa119:0.01133)0.749.36:0.00377,((Ult
17014:0.00802,UltCa120:0.06283)0.760.23:0.00746,(Ult17013:0.01927,(Ult170
15:0.0155,(Ult17016:0.01116,Ult17017:5.4E-
4)0.902.48:0.01147)0.838.32:0.00754)0.774.43:0.00378)0.780.30:0.0038)0.97
1.30:0.02065,UltCa118:0.0361)0.056.3:5.4E-
4,(Ult17019:0.04009,(UltCa121:0.01118,((Ult17021:0.01033,(Ult17020:5.5E-
4,Ult17023:0.01121)0.873.34:0.01126)0.962.42:0.03087,UltDe168:0.08543)0.6
36.3:0.01432)0.557.4:5.3E-
4)0.918.36:0.01372)0.746.21:0.00407)0.479.8:0.00546,(Ult17018:0.00797,Ult
17024:0.00323)0.996.25:0.03442)0.972.27:5.5E-
4)0.989.30:0.04839)0.916.42:0.02391,((Ult17030:5.5E-
4,UltCa123:0.04345)0.968.27:0.06635,((Ult17026:0.0,Ult17027:0.0,Ult17028:
0.0):0.11233,Ult17029:0.09175)0.921.46:0.05507)0.844.43:0.02684)0.698.7:0
.00562,(UltB4601:0.03262,(Ult13149:0.00382,((Ult13148:0.03083,(Ult13146:0
.0148,Ult13147:0.05047)0.875.39:0.01397)0.906.54:0.01883,(Ult13152:0.0131
8,(Ult13150:5.5E-
4,Ult13151:0.0037)0.788.24:0.00779)0.890.45:0.01425)0.811.24:0.02389)0.96
6.42:0.04159)1.000.204:0.13628)0.874.53:0.01125,(((UltAc301:0.15879,((Ult

31270:0.00366,UltFi103:5.5E-
4)0.972.28:0.08027,(Ult31251:0.07617,((UltCy250:0.01409,Ult31264:0.01278)
0.990.31:0.0433,((((Ult31263:0.0,Ult31268:0.0):5.5E-
4,Ult31267:0.00368)1.000.205:0.06914,((((UltCy249:0.02684,(Ult31255:0.003
69,(Ult31248:0.00743,(Ult31269:5.5E-
4,Ult31247:0.03157)0.923.47:0.00743)0.858.30:5.4E-4)1.000.206:5.3E-
4)0.951.36:0.00746,Ult31250:5.4E-
4)0.790.26:0.00341,((Ult31249:0.01853,(Ult31253:0.025,(Ult31252:0.01977,U
lt31254:0.00745)0.857.39:0.01087)0.901.50:0.01236)0.963.35:0.02104,UltEu1
32:0.01137)0.990.32:5.5E-
4)0.732.6:0.00359,Ult31256:0.02752)0.946.38:0.02664)0.529.4:0.01197,(Ult3
1266:0.04016,(((Ult31261:0.03365,((Ult31259:0.0077,UltrS446:0.0154)0.903.
35:0.01455,(UltFor75:0.00381,(UltFor74:0.0196,(Ult31257:5.5E-
4,Ult31258:0.00369)0.923.48:0.01175)0.751.16:0.00331)0.845.35:0.00881)0.9
77.24:0.03755)0.872.43:0.02027,(Ult31260:0.01552,Ult31262:0.01578)0.693.6
:0.00233)0.916.43:0.0183,Ult31265:0.01709)0.665.10:0.01649)0.942.40:0.024
3)0.650.4:5.4E-
4)0.859.37:0.02324)0.962.43:0.07109)0.859.38:0.03952)0.794.25:0.03431,(((
(Ult31235:0.02881,(((UltOr482:0.01671,((UltFi102:0.01975,(Ult31239:0.0172
1,(UltrS444:5.3E-
4,UltrS445:0.03084)0.965.37:0.02621)0.977.25:0.03156)0.985.29:0.04321,Ult
rS443:0.02284)0.913.35:0.02027)0.892.33:0.01801,Ult31236:0.0286)0.944.26:
0.01923,Ult31238:0.05167)0.087.5:0.00485)0.764.31:0.01052,Ult31237:0.0423
5)1.000.207:0.10404,(Ult31241:0.03912,Ult31240:0.02936)0.943.34:5.4E-
4)0.919.46:0.03573,Ult31242:0.14109)0.857.40:0.02741)0.989.31:0.0871,(Tds
Narug:0.01435,Ult32129:0.00451)0.999.61:0.0934)0.086.5:0.00368)0.827.20:0
.01578)0.854.28:0.01984)0.791.17:0.01199,(Ult32005:0.06005,(((((Ult31233:
0.04878,(Ult31231:0.1233,(Pshggy85:0.07054,((Ult31232:0.04623,Otu00499:0.
06123)0.879.41:0.03847,Ult31246:0.13974)0.880.40:0.05798)0.227.5:0.01318)
0.852.32:0.03034)0.925.43:0.04738,(Ult31230:0.05199,Ult31229:0.15739)0.98
7.36:0.09482)0.698.8:0.00514,((Ult31227:0.0285,Ult31228:0.03688)0.995.39:
0.09226,(((Ult31217:0.00326,Ult31218:5.4E-
4)0.128.1:0.02288,((Ult31224:0.00337,(Ult31225:0.0075,Ult31226:0.00372)1.
000.208:5.4E-4)0.926.39:0.04756,(Ult31223:0.07383,(Ult31220:5.4E-
4,(Ult31222:0.00372,(Ult31221:0.0,Ult31219:0.0):5.5E-
4)0.920.43:0.00345)0.980.18:0.07346)0.149.2:0.03309)0.991.32:0.10413)0.48
6.7:0.0207,((Ult31212:0.01483,(Ult31211:0.00385,(Ult31213:0.0037,(Ult3120
9:0.01117,Ult31210:0.0037)0.802.30:0.00369)0.783.33:0.00355)0.838.33:0.01
202)0.934.34:0.03708,(Ult31214:0.02866,(Ult31216:0.07384,Ult31215:0.00459
)0.124.2:0.00726)0.944.27:0.03479)0.947.37:0.03415)0.860.25:0.02687)0.925
.44:0.03565)0.819.27:0.01756,Ult31234:0.11543)0.904.37:0.04544,((Ult31830
:0.03341,((Ult13101:5.5E-
4,(((Ult13099:0.00612,Ult13098:0.15313)0.650.5:5.4E-
4,(Ult13095:0.00369,(Ult13097:5.5E-4,Ult13103:5.5E-4)0.801.23:5.5E-
4)0.922.35:0.00705)0.757.22:0.00132,Ult13102:5.5E-4)0.919.47:5.4E-
4)0.995.40:5.4E-
4,((Ult13096:0.0037,Ult13105:0.00371)0.898.41:0.00213,(Ult13100:0.01926,U
lt13104:0.00761)0.845.36:0.0069)0.894.30:0.00203)0.866.35:0.01191)0.920.4
4:0.05089,(((CddDivi3:0.00368,Ult13091:0.00758)0.873.35:0.01354,(Ult13090
:0.02093,(Ult13093:0.0162,(Ult13092:0.00369,Ult13094:5.5E-
4)0.815.20:0.00731)0.917.34:0.02009)0.760.24:0.00875)0.426.10:0.01294,((U
lt13089:0.00682,(Ult13079:0.05094,((Ult13070:5.4E-
4,(Ult13082:0.00742,((Ult13068:0.0,Ult13069:0.0,Ult13072:0.0,Ult13074:0.0
,Ult13075:0.0,Ult13078:0.0,Ult13084:0.0):5.5E-4,((Ult13071:5.5E-

4,Ult13083:5.5E-4)0.730.14:5.5E-
4,(Ult13076:0.0037,Ult13077:0.0037)0.712.9:5.5E-4)0.000.285:5.5E-
4)0.867.40:5.5E-
4)0.805.23:0.0037)0.750.19:0.00365,(((Ult13088:0.01118,(Ult13073:0.0,Ult1
3080:0.0,Ult13081:0.0,Ult13087:0.0,Ult31631:0.0):5.5E-4)0.347.4:5.5E-
4,Ult13086:0.03057)0.777.32:0.00366,Ult27488:0.02286)0.878.36:0.00764)0.7
02.4:0.00377)0.871.37:0.01207)0.934.35:0.02192,UltCan90:0.06853)0.818.15:
0.01425)0.166.3:0.04221)0.975.28:0.0662)0.857.41:0.03251)0.870.26:0.02523
)0.732.7:0.00512)0.835.27:0.01238)0.857.42:0.01614)0.850.35:0.01314)0.918
.37:0.01968)0.855.35:0.01329,((((((UncUn225:0.08709,((((Ult31781:0.00739,
((Ult31780:0.0,Ult31777:0.0,Ult31779:0.0,Ult31754:0.0,Ult31782:0.0,Ult316
65:0.0):5.5E-4,Ult31778:5.5E-4)0.959.45:5.5E-
4)0.997.34:0.06114,((((((Bfhggg87:0.00448,((Ult31820:0.03503,UncUn224:5.4
E-
4)0.972.29:0.02645,Ult31819:0.012)0.728.14:0.01176)0.903.36:0.01707,Ult31
817:0.00663)0.777.33:0.00516,((Otu00201:0.05776,(Ult31818:5.5E-
4,Otu00167:0.00739)0.898.42:0.02086)0.868.28:0.0162,(PahggY92:0.01157,(Ul
t31816:0.03309,(Ult31814:5.4E-
4,Ult31815:0.01098)0.958.43:0.03154)0.834.22:0.01197)0.751.17:0.00777)0.7
12.10:0.00838)0.885.33:0.01341,(((Z0114710:0.02677,(Ult31811:0.01799,(Ult
31810:0.01175,(AtPYy157:0.0196,AtPYy156:0.01914)0.728.15:0.00372)0.849.29
:0.00966)0.918.38:0.02092)0.935.39:0.02087,(((Otu00036:0.0044,(Ult31813:0
.00239,CanTamme:0.02968)0.790.27:0.0097)0.937.37:0.02215,(UltFi104:0.0139
7,Otu00444:0.02527)0.097.5:0.01186)0.868.29:0.01146,(UltSyn42:0.00377,Otu
00222:0.0075)0.932.34:0.02178)0.844.44:0.01459)0.865.36:0.01268,(Otu00214
:0.00811,(UltSyn41:0.00591,(Ult31812:0.00372,UltSyner:0.00373)0.925.45:0.
01408)0.858.31:0.01011)0.875.40:0.01448)0.767.27:0.00754)0.274.3:0.0081,S
yrgiyyy:0.04129)0.933.41:0.0286,(Pshggg66:0.04786,(((Ult31793:0.04686,(Ul
t31794:0.05881,(((Ult31791:0.0,CbsEvrye:0.0):0.01844,(BacNL449:0.01101,(S
yrSpeci:0.00368,(BacNL447:5.5E-4,(BacNL446:5.5E-4,BacNL448:5.5E-
4)0.426.11:5.5E-4)0.846.28:5.5E-4)0.893.42:5.4E-
4)0.883.42:0.01261)0.783.34:0.01452,(Ult31792:0.04817,(Ult31808:0.0,Ult31
809:0.0):0.08183)0.363.6:0.00992)0.758.31:0.02354)0.857.43:0.01901)0.897.
29:0.02019,((Ult31798:0.06398,(Ult31796:0.00533,(Ult31795:0.02449,Ult3179
7:5.5E-4)0.976.25:0.02088)0.827.21:5.3E-
4)1.000.209:0.07122,(Ult31801:0.02331,(Ult31799:5.5E-
4,Ult31800:0.00366)0.855.36:0.01285)0.928.31:0.03034)0.743.30:0.01721)0.5
37.5:0.00502,(Ult31784:0.02117,((Ult31783:0.00742,(Ult31786:0.00357,Ult31
732:0.04786)0.967.27:5.5E-
4)0.780.31:0.0071,((Ult31677:0.00193,(Ult31787:5.5E-
4,Ult31785:0.00366)0.991.33:0.00186)0.946.39:0.0255,Ult31802:0.06065)0.23
3.2:0.00836)0.946.40:0.0245)0.812.18:0.00966)0.736.18:0.00536)0.861.45:0.
01264)0.810.23:0.01652)0.374.5:0.00226,(SgsBacte:0.05023,(Ult31789:0.0036
4,(Ult31788:5.5E-4,Ult31790:5.5E-4)1.000.210:5.4E-
4)0.679.6:0.00309)0.998.45:0.06125)0.066.3:0.00515,(((((((Ult31774:0.0036
8,(Ult31682:5.5E-4,Ult31773:0.01487)0.857.44:0.00365)0.975.29:5.5E-
4,(CstBac10:0.01518,((Ult31771:0.00368,Ult31772:5.5E-4)0.838.34:5.4E-
4,(Ult31768:5.5E-
4,((BacEnr55:0.0,Ult31704:0.0,Ult31722:0.0,Ult31769:0.0,Ult31721:0.0):5.5
E-4,(Ult31770:0.00366,(((Ult31666:0.0,Ult31759:0.0):5.5E-
4,(Ult31715:0.00366,Ult31703:0.00366)0.687.10:5.5E-4)0.934.36:5.5E-
4,(Ult31766:5.5E-4,Ult31767:0.00317)0.790.28:5.1E-
4)0.961.33:0.01112)0.928.32:5.5E-4)0.972.30:5.5E-
4)0.898.43:0.00727)0.908.38:0.01104)0.868.30:0.00741)0.404.2:0.0036,Ult31

723:5.4E-
4)0.953.29:0.01092,((Ult31712:0.00737,((Ult31761:0.01133,(Ult31775:0.0036
7,Ult31668:5.5E-4)0.000.286:5.5E-
4)0.827.22:0.00366,((((Ult31699:0.00366,(Ult31739:5.5E-
4,Ult31760:0.11652)0.855.37:5.4E-4)0.850.36:5.5E-4,(Ult31684:5.5E-
4,(Ult31734:0.0,Ult31731:0.0,Ult31748:0.0,Ult31749:0.0,Ult31746:0.0,Ult31
765:0.0,Ult31700:0.0,Ult31702:0.0,Ult31706:0.0,Ult31736:0.0,Ult31751:0.0,
Ult31752:0.0,Ult31757:0.0,Ult31711:0.0,Ult31680:0.0,Ult31681:0.0,Ult31683
:0.0,Ult31689:0.0,Ult31686:0.0,Ult31724:0.0,Ult31763:0.0,Ult31758:0.0):5.
5E-4)0.000.287:5.5E-4)0.000.288:5.5E-
4,(Ult31764:0.00366,Ult31687:0.00366)0.921.47:5.5E-4)0.000.289:5.5E-
4,(((Ult31747:0.01114,Ult31750:0.00367)0.712.11:5.5E-
4,Ult31685:0.00366)0.921.48:5.5E-4,Ult31762:5.5E-4)0.000.290:5.5E-
4)0.965.38:5.5E-4)0.365.2:5.3E-
4)0.933.42:0.00737,Ult31720:0.00739)0.957.34:5.4E-4)0.658.3:5.5E-
4,(Ult31707:0.01754,(Ult31725:0.01578,(Ult31729:0.0172,(Ult31701:0.03481,
Ult31728:0.02193)0.832.23:0.0108)0.835.28:0.01302)0.841.27:0.01428)0.969.
30:0.02753)0.311.2:0.00395,((Ult31755:5.5E-
4,Ult31821:0.00366)0.959.46:0.01118,(Ult31716:5.5E-4,(Ult31674:5.5E-
4,((Ult31679:0.00366,Ult31756:0.00367)0.705.8:5.5E-4,((Ult31697:5.5E-
4,((Ult31710:0.00366,Ult31691:5.5E-4)0.000.291:5.5E-
4,((Ult31727:0.0,Ult31733:0.0,Ult31730:0.0,Ult31737:0.0,Ult31690:0.0,Ult3
1743:0.0,Ult31744:0.0,Ult31745:0.0,Ult31742:0.0,Ult31708:0.0,Ult31709:0.0
,Ult31735:0.0,Ult31738:0.0,Ult31740:0.0,Ult31713:0.0,Ult31717:0.0,Ult3175
3:0.0,Ult31714:0.0,Ult31719:0.0,Ult31667:0.0,Ult31670:0.0,Ult31669:0.0,Ul
t31671:0.0,Ult31672:0.0,Ult31678:0.0,Ult31673:0.0,Ult31688:0.0,Ult31692:0
.0,Ult31693:0.0,Ult31694:0.0,Ult31696:0.0):5.5E-4,Ult31698:5.5E-
4)0.000.292:5.5E-4)0.000.293:5.5E-4)0.000.294:5.5E-
4,(((Ult31675:0.00372,Ult31741:0.00366)0.898.44:5.5E-
4,(Ult31676:0.00366,Ult31718:0.00366)0.898.45:5.5E-4)0.000.295:5.5E-
4,Ult31705:5.5E-4)0.000.296:5.5E-4)0.724.13:5.5E-4)0.699.13:5.5E-
4)0.414.3:5.5E-4)0.880.41:5.4E-
4)0.835.29:0.00675)0.990.33:0.04366,Ult31776:0.02254)0.800.18:0.01089)0.9
69.31:0.06052)0.878.37:0.03313,((Ult31829:0.00692,((Ult31827:5.4E-
4,(Ult31826:0.02025,(((Ult31837:0.0,Ult31845:0.0,Ult31858:0.0):0.01135,((
Ult31838:0.0,Ult31840:0.0,Ult31843:0.0,Ult31844:0.0,Ult31861:0.0,Ult31841
:0.0):5.5E-4,Ult31832:5.5E-
4)0.882.41:0.00751)0.781.30:0.00366,(Ult31695:0.03877,(Ult31834:0.00368,(
(Ult31835:0.0,Ult31862:0.0):0.00369,(Ult31833:0.0,Ult31836:0.0,Ult31839:0
.0):5.5E-4)0.847.38:5.5E-4)1.000.211:5.4E-
4)0.934.37:0.01122)0.922.36:0.01134)0.758.32:0.0037)0.822.19:0.00366,Ult3
1828:5.3E-4)0.906.55:0.00825)0.651.8:5.5E-
4,Ult31831:0.01846)0.924.34:0.02437)0.879.42:0.02583,(UltCl396:0.0,Ult318
63:0.0):0.05919)0.936.39:0.04494,(TmvAcida:0.10033,UltAmin2:0.0411)0.742.
16:0.01106)0.032.2:5.4E-
4,((((Ult31999:0.04155,((((Ult31998:0.0037,((Ult31993:0.01117,((Ult31994:
0.0,Ult31988:0.0,Ult31996:0.0,Ult31997:0.0):5.5E-
4,UltAna41:0.00371)0.448.4:5.5E-
4)0.885.34:0.00763,(Ult32000:0.00374,BacEnr59:0.00743)0.930.28:0.01142)0.
868.31:0.00744)0.951.37:5.4E-
4,(Ult31989:0.0,Ult31991:0.0,Ult31990:0.0,Ult31992:0.0,Ult32001:0.0):5.5E
-4)0.932.35:5.5E-
4,UltSyn54:0.03116)0.763.33:0.00549,Ult31995:0.00195)0.781.31:0.01193)0.9
89.32:0.07359,(UltSyn53:0.06284,(((Ult31849:0.01115,(Ult31853:5.5E-

4,Ult31848:5.5E-4)0.744.23:5.5E-4)0.836.24:0.0036,(Ult31851:5.5E-
4,(((Ult31846:0.01498,Ult31852:0.00745)0.895.47:5.5E-4,Ult31850:5.5E-
4)0.971.31:5.5E-
4,(Ult31854:0.0,Ult31857:0.0,Ult31855:0.0,Ult31856:0.0,Ult31847:0.0):5.5E
-4)0.746.22:5.5E-4)1.000.212:5.5E-
4)0.997.35:0.08933,((((UltSyn52:0.0,UltSyn51:0.0):0.02218,UltThe38:0.01654
)0.936.40:0.03448,((UltSynt6:0.00369,((TvrLieni:0.0,UltThe37:0.0):5.5E-
4,(Ult31980:0.01489,UltThe36:0.00724)0.911.42:5.4E-
4)0.786.38:0.00375)0.937.38:0.01881,(((Ult31979:5.5E-
4,Ult31975:0.00369)0.951.38:5.4E-
4,((Ult31976:0.0,Ult31977:0.0,UltSyn49:0.0,UltSyn50:0.0):5.4E-
4,((Ult31978:0.0,Ult31974:0.0):0.00369,UltThe35:5.5E-
4)0.911.43:0.01108)0.715.4:0.00702)0.528.5:0.00908,((Ult31822:0.00747,(Ul
t31971:0.00369,Ult31972:5.5E-4)0.936.41:5.5E-
4)0.814.22:0.00723,(Ult31973:0.00713,Ult31664:0.02717)0.926.40:0.0121)0.9
57.35:0.02705)0.988.40:0.04187)0.856.27:0.02141)0.934.38:0.04163)0.597.6:
0.01604)0.199.1:0.03284)0.829.29:0.02265,((Ult31981:0.0,Ult31982:0.0,Ult3
1983:0.0,Ult31985:0.0):5.4E-
4,(Ult31986:0.03149,Ult31984:0.01179)0.760.25:0.00335)0.847.39:0.01688)0.
580.3:0.01726,((Ult31865:0.0115,(Ult31987:0.00752,(AmuCircu:5.5E-
4,Ult31866:5.5E-4)0.109.4:5.5E-4)0.334.3:5.4E-
4)0.720.9:0.00592,(UltEu135:0.02244,Ult31726:0.06955)0.779.30:0.0123)0.98
4.19:0.07088)0.932.36:0.04543)0.911.44:0.03785,((UltDethi:0.02271,(DtlSpe
ci:0.02744,(DtlPepti:0.00384,DtlRusse:5.3E-4)0.768.19:5.5E-
4)0.915.35:0.02641)0.989.33:0.08089,(((Ult31965:0.00374,(Ult31962:0.00375
,Ult31963:5.5E-4)0.995.41:5.5E-
4)0.695.7:0.0105,((Ult31954:0.0,Ult31964:0.0,Ult31956:0.0,Ult31957:0.0,Ul
t31958:0.0,Ult31959:0.0,Ult31960:0.0,Ult31961:0.0):5.4E-
4,(Ult31953:0.00376,(Ult31955:5.5E-4,AiiMobil:0.00375)0.599.4:5.5E-
4)0.919.48:0.0076)0.749.37:0.00481)0.921.49:0.07162,((Ult31948:0.07813,((
((((Ult31949:0.0,UltAmin3:0.0,Ult31950:0.0,Ult31951:0.0):0.00788,((Ult270
24:0.0,UltDef18:0.0):5.5E-
4,Ult31952:0.06156)0.315.4:0.00739)0.906.56:0.03174,(((Ult31881:0.06861,(
((FirmOra3:0.0,FlpSpeci:0.0,DbsSpeci:0.0,UltSyn45:0.0,DbsSpec2:0.0):0.009
49,Syrgiyy0:0.0096)0.806.15:0.01051,(((((((Otu00004:0.01914,((Ult31879:0
.00757,Otu00008:5.5E-
4)0.934.39:0.01546,Otu00142:0.00444)0.938.30:0.01506)0.835.30:5.4E-
4,Ult31875:0.01533)0.799.30:0.0038,((AtPYy153:0.00374,(Ult31868:0.02389,O
tu00014:0.01977)0.757.23:0.0036)0.996.26:5.4E-4,((Ult31876:5.5E-
4,Ult31873:0.00375)0.799.31:0.00374,(Ult31869:0.00753,(((Ult31874:0.01147
,UncUn244:5.5E-
4)0.778.32:0.00369,(Ult31867:0.0076,(((UncUn226:0.00375,UncUn230:5.5E-
4)0.896.27:0.00758,(((UncUn229:0.0,UncUn239:0.0):5.5E-4,(((UncUn234:5.5E-
4,(UncUn236:0.0,UncUn237:0.0):0.00375)0.914.38:0.00249,((UncUn228:0.0,Unc
Un235:0.0):5.5E-
4,UncUn232:0.01521)0.914.39:0.0025)0.914.40:0.00251,UncUn227:0.00376)0.79
5.32:5.5E-4)0.000.297:5.5E-4,((UncUn238:5.5E-
4,UncUn231:0.00752)0.416.8:0.00374,UncUn233:0.00371)1.000.213:5.4E-
4)1.000.214:5.4E-4)0.878.38:0.00762,(UncUn240:5.5E-
4,((UncUn242:0.0,UncUn241:0.0):5.5E-
4,UncUn243:0.00375)0.929.41:0.00758)0.890.46:0.00758)0.878.39:0.00765)0.7
86.39:0.00384)0.405.8:0.00378,AtPYy151:0.03678)0.260.4:5.4E-
4)0.797.25:0.00378)0.796.18:0.00379)0.801.24:0.0038)0.884.39:0.00765,((Ul
t31870:0.00372,Ult31871:0.0076)0.892.34:0.00775,(Otu00174:0.01147,(UltSyn

43:0.00374,S0114202:5.5E-
4)0.931.38:0.0116)0.853.29:0.00768)0.775.24:0.00372)0.782.32:0.00367,Otu0
0039:0.01164)0.825.22:0.00668,((Ult31877:0.00755,UncUn245:5.5E-
4)0.835.31:0.00711,(Ult31878:0.00728,Otu00273:0.00799)0.784.24:0.00882)0.
099.1:5.5E-
4)0.624.11:0.00772,Otu00089:0.03524)0.868.32:0.00927,(((Ult31872:0.0,Otu0
0370:0.0):0.04252,(UltSyn44:5.4E-
4,Otu00094:0.01113)0.985.30:0.04214)0.242.4:0.01682,UltFi105:0.01844)0.86
4.40:5.4E-
4)0.941.34:0.01973)0.843.27:0.01895)0.770.41:0.00682,(FirmOra4:0.01585,((
(Ult31880:0.08248,(JnqAnthr:0.06361,((((Ult31970:0.05095,(Ult31967:0.0696
2,(PymPisco:0.03934,Ult31966:0.01549)0.963.36:0.05458)0.885.35:0.04017)0.
906.57:0.02923,Ult31968:0.04374)0.000.298:0.00652,(SgsBact2:5.4E-
4,SyrSpec2:0.02719)0.999.62:0.11158)0.104.6:0.02091,Ult31969:0.08635)0.94
4.28:0.05157)0.988.41:0.08805)0.554.6:0.04175,UltRu558:0.09499)0.822.20:0
.02608,UltRu559:0.03771)0.844.45:0.01366)0.626.8:0.00503)0.891.35:0.02415
,(((Tryy0108:0.0,UltSyn46:0.0):5.3E-
4,((AtPYy154:0.0485,AtPYy155:0.00776)0.874.54:0.02029,Try00010:0.03638)0.
874.55:0.01509)0.895.48:0.01497,(UncUn247:0.00374,UncUn246:5.5E-
4)0.994.37:5.4E-
4)0.998.46:0.08531)0.936.42:0.03856)0.655.8:0.03332,(Ult31900:0.00371,Ult
31932:5.5E-
4)0.981.25:0.06806)0.679.7:0.01026,(Ult31921:0.08601,(Ult31910:0.0,Ult318
95:0.0,Ult31925:0.0,Ult31924:0.0,Ult31926:0.0):0.0115)0.282.3:6.6E-
4)0.769.29:0.0092,(((Ult31928:0.0,Ult31930:0.0,Ult31931:0.0):5.5E-
4,Ult31929:5.5E-
4)0.998.47:0.03967,(((Ult31903:0.00374,Ult31908:0.00754)0.258.3:5.5E-
4,((((UltSyn48:0.04418,(UltSyn47:0.01125,Ult31920:0.00394)0.640.4:0.01034
)0.760.26:0.00531,Ult31911:0.00375)0.923.49:5.5E-
4,((Ult25879:0.0,Ult31882:0.0,Ult31904:0.0,Ult31896:0.0,Ult31906:0.0,Ult3
1898:0.0,Ult31899:0.0,Ult31901:0.0,Ult31902:0.0,Ult31909:0.0,Ult31912:0.0
,Ult31914:0.0,Ult31913:0.0,Ult31907:0.0):5.5E-
4,(Ult31894:0.00374,(Ult31919:0.0526,Ult31893:0.00242)0.996.27:0.00143)0.
679.8:5.5E-4)0.000.299:5.5E-4)0.000.300:5.5E-4,(((Ult31922:5.5E-
4,Ult31916:5.5E-4)0.000.301:5.5E-
4,(UltDef17:0.00374,Ult31927:0.00374)0.526.2:5.5E-4)0.000.302:5.5E-
4,(Ult31915:0.00375,Ult31905:0.00374)0.578.4:5.5E-4)0.000.303:5.5E-
4)0.937.39:5.5E-4)0.877.52:5.3E-4,((Ult31897:5.5E-
4,UltrdL21:0.00375)0.782.33:0.0035,Ult31923:0.02413)0.783.35:0.00402)0.87
0.27:5.5E-
4)0.832.24:0.01131)0.844.46:0.02251)0.698.9:0.03925,((Ult31890:0.02655,(U
lt31891:5.4E-
4,(BacEnr58:0.01536,(Ult31889:0.00373,((Ult31892:0.00753,Ult31944:0.00375
)0.655.9:5.5E-
4,((Ult31887:0.0,Ult31888:0.0,UltDef16:0.0,Ult31885:0.0,Ult31886:0.0,Ult3
1945:0.0,Ult31946:0.0):5.5E-
4,(Ult31947:0.00375,(Ult31884:0.00375,Ult31883:0.00376)0.396.12:5.5E-
4)0.634.4:5.5E-4)0.000.304:5.5E-4)0.827.23:5.5E-
4)0.800.19:0.00376)0.770.42:0.00375)0.748.26:0.00474)0.809.24:0.0226,(Ult
31842:0.05288,(Ult31938:5.5E-4,((Ult31933:5.4E-
4,(Ult31943:0.00937,UltB7987:0.08627)0.954.42:0.02606)0.941.35:0.01126,(U
lt31934:0.00381,(((Ult31935:0.0,Ult31942:0.0,Ult31936:0.0):5.5E-
4,(Ult31937:0.00375,Ult31940:0.00377)0.773.31:5.5E-4)0.000.305:5.5E-
4,(Ult31941:0.00374,Ult31939:0.00374)0.537.6:5.5E-4)0.888.43:5.0E-

4)0.855.38:5.4E-
4)0.847.40:0.00347)0.844.47:0.01364)0.928.33:0.04413)0.892.35:0.03449)0.6
60.6:0.04016)0.126.1:0.01943)0.916.44:0.04078)0.952.37:0.04316)0.936.43:0
.02568,(((((Ult25861:5.5E-
4,(Ult25862:0.00743,(Ult25863:0.00369,Ult25860:0.0074)0.012.1:5.5E-
4)0.858.32:0.00368)0.908.39:0.01742,((Ult25859:0.01324,((Ult25855:0.06394
,(Ult25850:0.00737,((Ult25848:0.0,Ult25849:0.0,Ult25851:0.0,Ult25852:0.0,
Ult25853:0.0,Ult25864:0.0):5.5E-4,Ult25854:0.00366)0.155.5:5.5E-
4)0.992.36:5.3E-
4)0.278.5:0.01549,Ult25865:0.0536)0.843.28:0.03302)0.814.23:0.01004,(Ult2
5858:5.4E-4,(Ult25856:0.01113,Ult25857:5.5E-
4)0.982.19:0.019)0.973.19:0.01931)0.918.39:0.01894)0.987.37:0.03703,SmbSp
eci:0.01401)0.532.4:0.00572,SmbTherm:0.01188)0.999.63:0.06327,(UltThe15:0
.05303,((Ult25883:0.05181,(Ult25894:0.03944,Ult25893:0.04774)0.968.28:0.0
5053)0.851.37:0.02339,(Ult25882:0.0055,((((Ult27475:0.0368,Ult31630:0.056
8)0.662.6:0.0161,UltSymb4:0.00632)0.756.38:0.00472,((Ult27470:0.0,Ult2747
2:0.0,Ult27474:0.0):5.4E-
4,((Ult25876:0.0074,(Ult25878:0.01119,Ult27473:5.4E-
4)0.767.28:0.00396)0.153.4:0.0074,(Ult25881:0.00372,Ult27471:0.00758)0.92
7.37:0.00346)1.000.215:5.3E-
4)0.883.43:0.00733)0.876.34:0.00931,Ult31649:0.02885)0.930.29:0.01877)0.8
99.35:0.01735)0.811.25:0.00937)0.926.41:0.02758)0.569.5:0.00885)0.906.58:
5.4E-
4)0.518.4:0.00699,((Ult25846:0.01865,Ult25916:0.07862)0.883.44:0.02544,((
Ult32136:0.03279,(Ult32143:0.03765,(Ult32142:0.09379,(((Ult32140:5.5E-
4,Ult32141:5.5E-4)0.982.20:5.5E-
4,(UltThe45:0.01198,AnbBact3:0.00321)0.989.34:0.0199)0.970.35:0.02087,(Ul
t32139:0.02192,((Ult32138:0.01175,TpnSyntr:0.00711)0.700.16:0.00601,((Ult
32135:0.22555,TpnSpeci:5.4E-4)0.905.32:0.00742,Ult32137:5.5E-
4)0.798.25:0.00629)0.917.35:0.01634)0.837.22:0.01177)0.670.6:5.5E-
4)0.076.3:0.00918)0.996.28:0.08162)0.940.35:0.03533,(UltrdL18:0.05031,(Nr
rTherm:0.01886,NtvWadin:0.06742)0.891.36:0.02307)0.957.36:0.03659)0.873.3
6:0.02427)0.773.32:0.01258)0.944.29:0.0297)0.745.28:0.0058)0.655.10:5.4E-
4,((Ult25915:0.02094,DttAlkal:0.03418)0.835.32:0.01507,Ult24412:0.04577)0
.419.4:0.02)0.898.46:0.01637,IroBac16:0.1087)0.810.24:0.01404,(GrbTherm:0
.01626,(((Ult21908:0.0,Ult21915:0.0):5.5E-
4,Ult21914:0.00367)0.990.34:0.02269,(Ult21907:0.00945,(Ult21909:0.01518,(
(IroBac17:0.0,IroBac18:0.0):5.5E-4,(Ult21910:0.00736,IroBac19:5.4E-
4)0.113.4:0.00367)0.781.32:0.00364)0.778.33:0.00573)0.786.40:0.0075)0.848
.27:5.5E-
4)0.996.29:0.03653)0.740.25:0.00357,(((UltFir36:0.04391,(UltB4320:0.03042
,UltB4321:5.5E-
4)0.999.64:0.11079)0.939.35:0.04641,(((((Ult25790:0.03988,((((UltDes32:0.
07328,(((DtbSpec2:5.5E-4,DtbSpec3:5.5E-
4)0.911.45:0.01209,(DtbMetal:0.00702,Ult25764:0.00183)0.845.37:0.00172)0.
964.43:0.02379,(Ult25763:5.5E-
4,((DtbHafn2:0.0,DtbHafni:0.0,DtbHafn3:0.0,DtbHafn4:0.0):5.4E-
4,(DtbSpeci:0.00746,DtbDichl:5.5E-
4)0.892.36:0.00725)0.927.38:0.00705)0.705.9:0.00785)0.777.34:0.01307)0.80
5.24:0.00999,(((((DprAurip:0.0,Ult25767:0.0):0.00381,DprMerid:0.00757)0.7
71.38:0.00369,(DprYoung:5.4E-
4,DprHippe:0.02667)0.170.2:0.00739)0.907.40:0.00711,(DprSpeci:0.00762,Ult
25766:5.5E-
4)0.902.49:0.00218)0.648.8:0.00217,DprOrien:0.01151)0.058.5:5.4E-

4)0.943.35:0.02092,Ult25765:0.00605)0.989.35:0.0342,(Ult25772:5.4E-
4,((Ult25771:0.00366,Ult26396:0.0113)0.925.46:0.01144,Ult25773:0.0075)0.9
19.49:0.01113)0.863.39:0.01534)0.735.17:0.00373)0.930.30:0.01567,(((DnsTh
ios:0.07315,(Ult25806:0.0643,(Ult25808:0.06189,Ult25809:0.03166)0.917.36:
0.02614)0.925.47:0.03703)0.710.9:0.00754,Ult25807:0.0424)0.900.36:0.01293
,Ult25914:0.08107)0.766.26:0.00404)0.641.8:0.00434,(((UltMoore:0.04344,(U
lt25777:0.05557,TthFerri:0.11719)0.703.9:0.01009)0.968.29:0.03188,((Ult25
911:0.01491,(((((PesGluta:0.01734,((UltGram8:0.05415,(UltSynt5:0.06116,Ts
hLipol:0.02991)0.943.36:0.03571)0.993.26:5.5E-
4,((((SytWolf2:0.0,SytWolf3:0.0):0.01837,((SytCelli:0.01131,(SytSpeci:0.0
0741,Ult25905:0.00368)0.445.8:5.4E-
4)0.749.38:0.0049,(Ult25906:0.02475,SytErect:0.03021)0.604.6:0.01278)0.38
7.4:0.0033)0.874.56:0.00328,SytWolfe:5.4E-
4)0.990.35:0.03031,(SytCurva:0.0218,(SytSapov:0.01131,(Ult25900:0.00742,U
lt25901:5.5E-4)0.987.38:5.3E-
4)0.850.37:0.01248)0.325.5:0.00409)0.401.6:0.00427)0.815.21:0.0103)0.975.
30:0.02957,Ult25909:5.4E-
4)0.409.3:0.00742,Ult12487:0.00373)0.968.30:0.02071,((Ult25899:0.00883,((
SytPalm2:0.0,SytPalmi:0.0):0.01353,Ult25907:0.01342)0.716.8:0.00786)0.895
.49:0.01748,((Ult25898:0.01135,(SytZehnd:0.0,Ult25910:0.0):5.3E-
4)0.833.31:0.01025,(Ult25904:0.02337,Ult25903:0.02061)0.753.26:0.00492)0.
522.2:0.00819)0.724.14:0.00312)0.874.57:0.0114,Ult25902:0.01613)0.978.23:
0.03621)0.969.32:0.03256,((ShhLipoc:0.04912,(CrxSporo:0.00741,CrxSpeci:5.
5E-
4)0.982.21:0.05918)0.853.30:0.04171,(Ult25912:0.05409,Ult25789:0.04482)0.
767.29:0.0304)0.150.1:0.02136)0.695.8:5.5E-
4)0.939.36:0.01351,((Ult24413:0.04332,Ult24414:0.04548)0.985.31:0.05896,(
DtcAlkal:0.08466,(AbnCalif:0.02062,AbnGotts:0.00908)0.942.41:0.04545)0.06
2.3:0.02292)0.711.13:0.00726)0.813.21:0.0074)0.831.26:0.01103,(Bfhggg83:0
.026,(Ult25776:0.09831,(UltPept8:0.04916,(UltFir96:0.03499,(TncFerri:0.00
374,UltFir95:0.00722)0.796.19:5.4E-
4)0.988.42:0.05669)0.477.4:0.01737)0.875.41:0.0317)0.884.40:0.02392)0.840
.33:0.014,((((Ult25788:0.06419,UncUn214:0.03524)0.853.31:0.01966,(Ult2578
3:0.02335,(Ult25786:0.0037,(Ult25785:0.00362,Ult25784:0.03583)0.760.27:0.
00378)0.887.40:0.00817)0.763.34:0.00687)0.758.33:5.4E-
4,AtPYy150:0.00784)0.897.30:0.01015,((Ult25782:0.02219,(UltOr225:0.14889,
Ult25781:0.00813)0.930.31:0.03187)0.896.28:0.01662,((Ult25778:0.0158,(Ult
25779:5.5E-4,(UltB6886:0.16668,Ult25780:0.00297)1.000.216:7.4E-
4)0.840.34:0.00701)0.902.50:0.01305,Ult25787:0.04111)0.069.2:5.4E-
4)0.805.25:0.01225)0.803.22:0.009)0.947.38:0.03022)0.883.45:0.01819,(((Ul
t25775:0.01961,(Ult31806:0.09998,(Otu00430:5.5E-
4,(UltFir94:0.00514,Ult25774:0.08077)0.866.36:0.00988)0.997.36:0.07145)0.
328.6:0.01794)0.860.26:0.02213,(((AzlSpec7:0.04352,Ult31208:0.05057)0.985
.32:0.07647,((Ult31199:0.07685,((Ult31206:0.15511,Ult31207:0.00512)0.975.
31:0.06121,(Ult31205:0.01376,(UltCy248:0.02687,(UltCy247:0.00711,(Ult3120
4:0.01931,Ult31203:0.01137)0.292.3:5.5E-
4)0.937.40:0.02623)0.923.50:0.03311)0.941.36:0.04851)0.972.31:0.05924)0.8
07.19:0.03446,((((Ult31198:0.06576,Ult31196:0.01607)0.898.47:0.02452,Ult3
1197:0.03052)0.907.41:0.02139,(Ult31194:0.01919,Ult31195:0.03285)0.840.35
:0.01197)0.930.32:0.02574,(UltrS442:0.12163,(Ult31200:0.04204,(Ult31201:0
.05561,Ult31202:0.0241)0.535.3:0.01938)0.959.47:0.04101)0.788.25:0.01419)
0.889.36:0.02646)0.547.3:0.01793)0.992.37:0.08021,((Ult13132:0.05787,(Ult
31449:0.01003,Ult31450:0.03361)1.000.217:0.17862)0.883.46:0.03814,(Ult258
47:0.02626,Ult32002:0.09545)0.793.23:0.03525)0.793.24:0.02966)0.127.1:0.0

2564)0.965.39:0.04263,((UltCl327:0.09562,(Ult25067:5.5E-
4,(UltRu546:0.03576,Ult25794:5.3E-
4)0.970.36:0.01506)0.861.46:0.01707)0.927.39:0.036,((S0114444:0.03541,(Ul
t21876:0.06355,S0114496:0.01602)0.962.44:0.0385)0.972.32:0.03949,(Ult2352
7:0.03681,(Ult21877:0.00368,UltCl148:0.05901)0.657.5:0.00435)0.900.37:0.0
2334)0.825.23:0.01781)0.888.44:0.02151)0.755.30:0.00803)0.745.29:0.00563)
0.886.48:0.00828)0.745.30:0.00356,(Ult21744:0.03562,(((Ult21742:0.05905,M
ahAustr:0.07512)0.963.37:0.05594,((Ult21906:0.05837,(AnbBacte:5.5E-
4,Ult21903:0.01478)0.959.48:0.03192)0.932.37:0.03043,((Ult13850:0.05198,(
DtmAceto:5.5E-
4,DtmAcet2:0.00361)0.991.34:0.06385)0.866.37:0.02003,(DtmHalop:0.04192,(U
lt25822:0.01464,Ult25823:0.01203)0.992.38:0.05579)0.924.35:0.02747)0.782.
34:0.00941)0.799.32:0.01299)0.701.9:0.00764,(Ult21902:0.02592,(UltThe14:0
.01433,Ult21901:0.00104)0.862.35:0.01507)0.929.42:0.02748)0.652.5:0.00576
)0.867.41:0.0142)0.000.306:5.3E-
4,(((((UltCl362:0.05188,((((CsrSpe29:0.01951,CsrCylin:0.02787)0.952.38:0.
01971,Ult23991:5.4E-4)0.422.4:0.01105,UltCl363:5.3E-
4)0.943.37:0.02118,(Ult23988:0.01647,(Ult23989:0.01455,Ult23990:0.02052)0
.966.43:0.02311)0.904.38:0.0148)0.857.45:0.01312)0.757.24:0.01124,((CmtCo
olh:0.01093,(TrbCeler:5.5E-
4,Ult23992:0.00744)0.946.41:0.00778)0.000.307:5.4E-4,(((CmtAustr:5.5E-
4,CmtFervi:0.01133)0.842.35:0.01119,Ult26394:0.09446)0.923.51:0.01979,(Cm
tProte:5.4E-4,CmtViter:0.02291)0.000.308:5.5E-
4)0.801.25:0.00383)0.880.42:0.03132)0.935.40:0.02818,(((CsrSpe24:0.01521,
(((((CsrTepid:0.02721,(BacNL335:5.5E-4,((BacNL334:5.5E-
4,(BacNL331:0.00368,(BacNL329:0.0,BacNL330:0.0,BacNL332:0.0,BacNL333:0.0,
Ult23984:0.0):5.5E-4)0.404.3:5.5E-4)0.608.6:5.5E-4,BacNL336:5.5E-
4)0.258.4:5.5E-
4)0.756.39:0.00649)0.838.35:0.01306,((BacNL337:0.00368,CsrSporo:5.5E-
4)0.962.45:0.02081,((((UltCl357:0.01459,(CsrDrake:0.01965,((Ult23970:0.01
175,(CsrSpe20:5.5E-4,CsrNitro:5.5E-
4)0.977.26:0.02324)0.776.26:0.00371,CsrMagnu:0.00794)0.756.40:0.00284)0.8
65.37:0.00872)0.875.42:0.01171,(CsrKluyv:0.02345,(CsrAutoe:0.0,Ult23966:0
.0):0.01133)0.955.31:0.02009)0.782.35:0.00417,CsrTyrob:5.5E-
4)0.940.36:0.01743,(CsrTeta2:0.01116,(BacNL327:5.5E-4,BacNL328:5.5E-
4)0.895.50:5.5E-
4)0.998.48:0.04136)0.770.43:0.00774)0.880.43:0.00947)0.794.26:5.4E-
4,((CsrSpe16:0.03182,CsrFrigi:0.00749)0.920.45:0.01437,((((((BacNL324:0.0
0737,(Ult23961:0.01117,(CsrAmylo:0.0,Ult23968:0.0,Ult23986:0.0):5.5E-
4)0.119.3:5.5E-4)0.998.49:5.5E-
4,(((CsrGrant:0.02333,CsrAestu:0.01162)0.762.35:0.00496,((((CsrHaemo:0.0,
CsrNovyi:0.0):0.02335,(Ult23965:5.5E-4,UltCl356:0.00367)0.963.38:5.4E-
4)0.930.33:0.01881,(CsrSpe19:5.5E-4,CsrGangh:0.00744)0.041.3:5.4E-
4)0.789.20:0.00804,((UltB4789:0.05675,CsrSpe27:0.01609)0.977.27:0.03901,(
CsrSpe18:0.0069,CsrTher3:0.04124)0.838.36:0.01393)0.132.7:0.00755)0.914.4
1:0.01728)0.977.28:0.02373,(Ult23960:0.01112,(Ult23892:0.04039,(Ult23967:
0.00341,Ult29018:0.01949)0.998.50:0.05699)0.752.25:0.00836)0.903.37:0.013
84)0.855.39:0.00957)0.912.52:0.01094,(Hmyyy060:0.03342,Otu01105:0.01283)0
.981.26:0.03822)0.981.27:5.5E-
4,(CsrPepti:0.0,CsrSpe25:0.0):0.01133)0.920.46:0.00735,(((((((Ult23923:5.
5E-4,((Ult23949:0.0109,SaiMaxim:0.0037)0.890.47:5.4E-
4,Ult23946:0.0037)0.064.2:5.5E-4)0.995.42:5.4E-
4,(Ult23882:0.00368,(UltSarci:0.00369,((((((Ult23952:0.00369,Ult23331:0.0
0368)0.623.3:5.5E-

4,((Ult18331:0.0,Ult18332:0.0,Ult18333:0.0,Ult19660:0.0,Ult23779:0.0,Ult2
3862:0.0,Ult23883:0.0,Ult23924:0.0,Ult23925:0.0,Ult23926:0.0,Ult23927:0.0
,Ult23930:0.0,Ult23932:0.0,Ult23934:0.0,Ult23937:0.0,Ult23936:0.0,Ult2393
5:0.0,Ult23941:0.0,Ult23947:0.0,Ult23948:0.0,SwnFeca6:0.0,Ult23950:0.0,Ul
t23951:0.0):5.5E-4,UltSarc2:5.5E-4)0.000.309:5.5E-4)0.000.310:5.5E-
4,Ult23931:0.00369)0.000.311:5.5E-
4,(Ult23942:0.01885,(UltBa767:0.01085,Ult23939:0.00201)0.914.42:0.00198)0
.999.65:5.5E-4)0.000.312:5.5E-
4,(Ult23940:0.00369,Ult23938:0.00369)0.888.45:5.5E-4)0.000.313:5.5E-
4,((Ult23933:0.00369,Ult18334:0.0074)0.906.59:5.5E-
4,(Ult23929:0.00369,(Ult23957:0.002,Ult18335:0.03059)0.995.43:0.00181)0.8
65.38:5.5E-4)0.000.314:5.5E-4)0.863.40:5.5E-4)0.089.3:5.4E-
4)0.920.47:0.00748)0.855.40:0.00749,Ult23959:0.00378)0.981.28:0.02309,((U
ltCl353:0.00371,(((Ult23910:0.00743,(Ult23909:0.0037,(Ult23913:0.00741,((
Ult19655:0.00368,(((Ult23823:0.01117,(Ult18329:0.01894,(UltClo34:0.00197,
Ult23888:0.02298)0.929.43:0.00191)0.808.23:5.4E-4)0.455.9:5.5E-
4,(Ult18330:0.0,Ult19653:0.0,Ult19654:0.0,Ult19659:0.0,Ult19663:0.0,UltCl
o35:0.0,Ult23886:0.0,Ult23907:0.0,Ult23911:0.0,Ult23903:0.0,Ult23904:0.0,
CsrPerfr:0.0,Ult23912:0.0,Ult24031:0.0):5.5E-4)0.027.2:5.5E-
4,(Ult23824:5.5E-4,Ult23880:0.01118)0.841.28:0.00369)0.369.4:5.5E-
4)0.481.2:5.5E-4,Ult23785:0.00369)0.000.315:5.5E-4)0.077.2:5.5E-
4)0.126.2:5.5E-4)0.907.42:0.0036,((Ult19661:0.0068,(Ult24030:5.5E-
4,UltCl373:0.00369)0.907.43:0.00237)0.873.37:0.00176,(Ult19658:0.00369,Ul
t19656:0.01492)0.772.34:0.00385)0.447.2:0.00369)1.000.218:5.4E-
4,UltClo33:0.00369)0.926.42:0.00763)0.941.37:5.4E-
4,((Ult23921:0.00388,((Ult23861:0.0,Ult23881:0.0,Ult23916:0.0,Ult23915:0.
0,Ult24035:0.0):5.5E-
4,Ult23918:0.0723)0.773.33:0.00343)0.892.37:0.01146,(UltBac45:0.0117,((Ul
t23860:0.0,Ult23920:0.0,Ult23928:0.0):5.5E-
4,Ult23919:0.01845)0.754.26:0.00321)0.981.29:0.02731)0.889.37:0.0111)0.98
5.33:5.5E-
4)0.865.39:0.011,((((UltEub87:0.0,UltEub88:0.0):0.00375,((Ult23825:0.0,Cs
rColic:0.0):5.5E-4,(Ult23872:0.01483,(Ult23873:5.5E-
4,Ult23874:0.00371)0.893.43:5.5E-
4)0.825.24:0.00364)0.944.30:0.0113)0.865.40:0.00725,((Ult23894:5.4E-
4,((((Ult23895:0.0,Ult23897:0.0):5.5E-
4,(((Ult23768:0.00371,(Ult12398:0.0074,Ult23896:5.4E-
4)0.784.25:0.00374)0.789.21:0.00375,((CsrChart:5.5E-4,CsrLongi:5.5E-
4)0.932.38:5.4E-
4,Ult23834:0.0266)0.394.5:0.00371)0.893.44:0.00746,Ult23765:0.01116)0.046
.3:5.5E-
4)0.889.38:0.00751,(Ult23893:0.02647,(Ult23770:0.00736,CsrVince:0.0112)0.
749.39:5.4E-4)0.910.53:0.00741)0.823.22:7.9E-
4,(Ult23899:0.03304,(CsrAuran:5.4E-4,(Ult23898:0.03137,(Ult18328:5.5E-
4,(Ult23833:5.5E-4,(Ult23900:0.00376,Ult23901:0.00376)0.898.48:5.5E-
4)0.709.7:5.5E-4)1.000.219:5.5E-
4)0.930.34:0.00355)0.574.3:0.01448)0.856.28:0.01195)0.774.44:0.0064)0.926
.43:0.00729,(((((Ult19657:5.5E-
4,Ult23869:0.00368)0.964.44:0.01122,(Ult19662:0.0037,(Ult23784:0.0,Ult238
70:0.0,Ult23871:0.0,Ult23908:0.0,Ult23914:0.0,Ult24029:0.0):5.5E-
4)0.635.1:5.5E-
4)0.942.42:0.00192,Ult19652:0.01484)0.938.31:0.00195,((EubNitri:0.0,CsrBa
rat:0.0):5.5E-
4,BacNL322:0.00396)0.827.24:0.00399)0.783.36:0.00398,CsrSardi:0.0038)0.81

3.22:0.00404)0.897.31:5.3E-
4)0.753.27:0.0065,((Ult23847:0.01456,Ult23875:0.00575)0.812.19:0.00779,(C
srQuini:0.00332,((((UltCl354:0.00745,(Ult23774:5.5E-
4,Ult23775:0.00369)0.753.28:5.4E-4)0.791.18:0.00369,(Ult23848:5.5E-
4,(UltCl352:0.00747,(Ult23868:0.0037,(Ult23864:0.0,Ult23865:0.0,Ult23866:
0.0):5.5E-4)0.616.4:5.4E-4)0.848.28:0.0037)0.295.8:5.5E-
4)0.809.25:0.0037,(Ult23867:0.00362,CsrIsati:0.00761)0.882.42:0.00766)0.7
80.32:0.00373,(((Ult23849:0.0,CsrChauv:0.0):5.5E-
4,(((Ult23793:0.0182,((Ult23843:0.03045,(Ult23788:5.5E-
4,(Ult23801:0.01902,(((Ult23852:0.00369,(UltCl350:0.00369,((Ult23821:0.0,
Ult23827:0.0,Ult23828:0.0,Ult23829:0.0,Ult23830:0.0,Ult23832:0.0,Ult23835
:0.0,Ult23841:0.0,Ult23842:0.0,Ult23769:0.0,Ult23773:0.0,Ult23776:0.0,Ult
23780:0.0,Ult23782:0.0,Ult23783:0.0,Ult23786:0.0,CsrDispo:0.0,Ult23789:0.
0,Ult23790:0.0,Ult23795:0.0,Ult23799:0.0,Ult23802:0.0,Ult23845:0.0,Ult238
84:0.0,Ult23879:0.0):5.5E-
4,(Ult23787:0.0037,((Ult23771:0.00369,(Ult23791:0.00369,(CsrCelat:5.5E-
4,((Ult23798:0.02305,Ult23796:0.08266)0.616.5:5.5E-
4,(Ult23800:0.02338,Ult23844:0.03936)0.831.27:0.0076)0.830.30:0.00395)0.5
67.4:5.4E-4)0.272.3:5.5E-4)0.452.6:5.5E-
4,UltCl351:0.00369)0.000.316:5.5E-4)0.093.3:5.5E-4)0.000.317:5.5E-
4)0.363.7:5.5E-4)0.376.2:5.5E-4,(Ult23839:5.5E-
4,Ult23840:0.0037)0.910.54:0.00744)0.000.318:5.5E-
4,((Ult23846:0.00369,(Ult23836:5.5E-
4,(((UltB7981:0.01497,(Ult23810:0.00369,(((((Ult23817:0.0,Ult23819:0.0,Ul
t23820:0.0,Ult23831:0.0):5.5E-
4,((Ult23809:0.0356,(Ult24032:0.01516,Ult23818:0.01505)0.178.2:0.0036)0.8
24.29:5.3E-4,Ult23811:0.0037)0.437.5:5.5E-4)0.000.319:5.5E-
4,Ult23837:0.00369)0.470.1:5.5E-4,UltB7982:0.00368)0.000.320:5.5E-
4,Ult23838:0.00369)0.182.4:5.5E-4)0.493.8:5.5E-
4)0.779.31:0.00375,Ult23808:0.00373)0.958.44:0.0115,(Ult23850:0.0,Ult2385
1:0.0,Ult23853:0.0,Ult24034:0.0):5.3E-
4)0.932.39:0.00758)0.930.35:0.00762)0.692.5:5.3E-
4,(UltCl349:0.01923,Ult23958:5.4E-
4)0.402.4:0.00369)0.788.26:0.00376)0.456.3:5.5E-4)0.207.2:5.5E-
4)0.070.1:5.5E-4)0.336.4:5.5E-4,(Ult23794:5.3E-
4,(Ult23797:0.01869,Ult24033:0.01879)0.732.8:0.00367)0.832.25:0.00366)0.4
79.9:5.4E-4)0.077.3:5.4E-4,(Ult23826:0.00369,(Ult23772:5.5E-
4,(Ult23822:0.00364,Ult23792:0.04357)0.537.7:5.3E-4)0.407.4:5.4E-
4)0.841.29:0.00371)0.782.36:0.00475,(Ult18336:0.01513,Ult18337:0.01126)0.
918.40:0.01129)0.802.31:0.00475)0.869.33:5.5E-
4,(Ult23858:0.05896,((Ult23856:0.0,Ult23857:0.0,Ult23859:0.0,BacNL321:0.0
,Ult23889:0.0):5.5E-4,(Ult23854:0.01119,Ult23855:5.5E-
4)0.837.23:0.0037)1.000.220:5.5E-
4)0.841.30:0.00364)0.871.38:0.00381)1.000.221:5.4E-
4)0.375.6:0.01062)0.849.30:0.01169)0.780.33:0.00461)0.808.24:0.00618,((Cs
rSpe17:5.5E-
4,Ult23964:0.02648)0.949.40:0.01752,((Ult23876:0.00731,((Ult23902:0.01507
,((Ult23878:0.00369,HumanG15:0.01917)0.776.27:0.00373,(Ult23877:5.5E-
4,((Ult23890:0.0,CsrButy2:0.0,CsrButyr:0.0,Ult23891:0.0):0.00369,Ult23956
:0.00369)0.710.10:5.5E-4)0.906.60:5.3E-
4)0.792.36:0.0037)0.784.26:0.0037,((Ult23887:0.0,CsrBeij3:0.0,CsrFavos:0.
0):5.5E-4,(CsrBeij2:0.01493,CsrBeije:0.01879)0.655.11:5.5E-
4)0.632.5:5.4E-4)0.914.43:5.5E-
4)0.796.20:0.00748,((CsrBotu2:0.0,CsrBotul:0.0):5.5E-

4,CsrUligi:0.02338)0.932.40:0.01101)0.776.28:0.00528)0.511.5:0.01131)0.79
1.19:0.00529,((BacNL325:5.5E-
4,BacNL326:0.00751)0.953.30:0.01123,(AbrPolye:0.00649,((CsrIntes:0.0,Ult2
3962:0.0):0.0181,CsrAlgi2:0.01136)0.792.37:0.01263)0.877.53:0.01232)0.886
.49:5.4E-4)0.918.41:0.00752)0.939.37:5.3E-
4,Ult23963:0.03175)0.883.47:0.00996)0.875.43:0.01006)0.873.38:0.00768,(((
UltCl358:0.0038,CsrColla:0.02329)0.781.33:0.00364,Ult23972:0.01123)0.881.
38:5.5E-4,(CsrSubte:0.0,SwnFeca7:0.0):0.00369)0.920.48:5.4E-
4)0.825.25:0.00373,(((((BacNL323:0.03477,UltCl355:0.01524)0.272.4:0.01528
,((Ult23978:0.01519,((UltB7984:0.08944,CsrSpe26:0.00357)0.998.51:5.4E-
4,CloBact2:5.4E-
4)0.887.41:0.00752)0.768.20:0.00586,Ult23977:0.02672)0.829.30:0.01109)0.8
23.23:0.02069,(CsrSpe28:5.4E-
4,((BacNL338:0.00193,BacNL339:0.00185)0.999.66:0.03565,Ult23985:0.01492)0
.099.2:0.00382)0.982.22:0.03418)0.746.23:0.00512,Ult23971:5.5E-
4)0.892.38:0.00812,(((Ult23982:0.01135,Ult23983:0.02326)0.852.33:0.00796,
((CsrSpe23:0.01058,CsrSpe22:0.01614)0.981.30:0.03159,(Ult23981:5.5E-
4,UltB5128:0.12615)0.775.25:0.00669)0.785.34:0.01306)0.941.38:0.01507,((C
srTetan:0.03972,(CsrLunde:0.01919,(Ult23979:0.01932,Ult23980:5.4E-
4)0.869.34:0.0077)0.011.4:0.00745)0.467.3:5.4E-4,((CsrTunis:5.5E-
4,Ult23973:0.0037)0.893.45:0.00751,((Ult23969:0.00779,UltB9129:0.08334)0.
851.38:0.00704,(Ult23975:5.4E-
4,((Ult23974:0.0,CsrFrigo:0.0,CsrEster:0.0,UltFir60:0.0):5.5E-
4,(CsrPsych:0.0,CsrBowma:0.0):0.00369)0.916.45:0.00733)0.569.6:0.0075)0.7
46.24:0.00386)0.899.36:0.00762)0.872.44:0.00742)0.871.39:0.00772)0.770.44
:5.4E-4)0.822.21:0.00384)0.604.7:5.5E-
4,(CsrCell3:0.02352,((CsrAceto:0.0,CsrFelsi:0.0,CsrRoseu:0.0,CsrAcet2:0.0
):0.02663,(CsrPaste:0.01513,(CsrSpe21:0.00389,(CsrAcidi:0.01147,CsrAkagi:
0.01531)0.860.27:0.0072)0.376.3:0.00365)0.895.51:5.4E-
4)0.346.6:0.00746)0.834.23:0.00734)0.828.23:0.01251,((UltCl360:0.0,UltCl3
59:0.0):0.02371,((CanArth2:5.4E-4,(CanArthr:5.5E-
4,Ult23976:0.01107)0.975.32:0.02668)1.000.222:0.12717,UltCl361:0.01231)0.
346.7:0.03019)0.875.44:0.01972)0.897.32:0.02555)0.912.53:0.02208,(Ult2398
7:0.01736,Ult23993:0.05913)0.730.15:0.0044)0.868.33:0.01148,((Ult25791:0.
01613,(Ult25792:5.5E-
4,Ult25793:0.00369)0.744.24:0.00271)0.998.52:0.04712,(UltRu481:0.02416,(A
nbBact2:0.04435,(Otu01118:0.0557,(Tryyy097:0.03703,(BactMB71:0.00883,(Ult
Pept9:0.04062,UncUn215:0.032)0.740.26:0.00347)0.875.45:0.01864)0.956.34:0
.03293)0.944.31:0.03794)0.867.42:0.02784)0.500.4:0.00188)0.782.37:0.00831
)0.925.48:0.01939)0.762.36:0.0042,(((((Ult21743:0.05364,(Ult21723:0.0915,
(Ult21747:0.00156,((Ult21745:0.0,Ult21748:0.0):0.01012,Ult21746:0.00954)0
.971.32:0.03508)0.981.31:0.06294)0.742.17:0.02803)0.739.24:0.02132,((Pshg
gg48:0.00753,(P0114420:0.006,P0114300:0.03913)0.909.32:0.01776)0.740.27:0
.00312,((PahYyy11:0.0,PahggY58:0.0):5.5E-
4,PahggY59:0.00369)0.872.45:0.00811)0.912.54:0.02397)0.814.24:0.0172,(Ult
Cl105:0.0255,Pshggg50:0.0234)0.965.40:0.03796)0.202.4:0.00981,((((UltClo9
2:0.01765,Pshggg46:0.02671)0.866.38:0.01038,((Ult21739:0.01931,((Otu00350
:0.0037,PahggY54:0.01933)0.069.3:5.4E-
4,(((Pshggg47:0.01386,UltClo91:0.00517)0.877.54:0.0078,(Ult21736:0.02343,
PshggY82:5.4E-
4)0.818.16:0.00739)0.199.2:0.00302,(Ult21737:0.01165,Ult21738:0.01157)0.9
48.45:0.007)0.961.34:0.01273)0.700.17:0.00377)0.813.23:0.00385,((((((UltF
ir31:0.0421,UltCl104:0.01957)0.899.37:0.01576,UltFir32:0.01171)0.126.3:0.
00821,((UltCl101:0.01301,(UltCl100:0.01018,Otu00249:0.00882)0.792.38:0.00

783)0.913.36:0.01423,((UltFir28:0.00718,UltClo95:0.03153)0.341.6:5.4E-
4,(Otu00154:0.00738,(UltClo93:0.00369,UltFir27:5.4E-
4)0.970.37:0.0194)0.950.33:0.0156)0.944.32:0.01155)0.287.3:0.00288)0.752.
26:5.4E-
4,((UltClo94:0.01874,(((UltFir29:0.00745,UltFir30:0.00745)0.918.42:0.0031
9,(UltClo98:0.02416,(UltClo96:0.00684,UltClo97:0.01605)0.965.41:0.03267)0
.936.44:0.02444)0.772.35:5.5E-
4,UltClo99:0.01926)0.894.31:0.00812)0.290.6:0.00761,(UltCl102:0.01881,Ult
Cl103:0.04031)0.770.45:0.0048)0.948.46:0.01491)0.881.39:0.0121,Pshggg49:0
.07758)0.917.37:0.01516,(((Ult21731:0.04383,Ult21732:0.05099)0.963.39:0.0
3339,UncUn142:0.00383)0.858.33:0.0076,((((PahggY61:0.0,PahggY60:0.0):0.03
079,(((PshggY83:0.03641,PshggY84:0.03198)0.743.31:0.00757,UltClo86:0.0056
9)0.872.46:0.00856,(EchggY11:0.00855,((Bfhggg48:5.4E-
4,(UltClo89:0.02379,UltClo88:0.01513)0.840.36:0.00772)0.894.32:0.01565,Ul
tClo87:0.01246)0.796.21:0.01122)0.846.29:0.00931)0.850.38:0.01099)0.778.3
4:0.01253,UltClo90:0.0108)0.278.6:5.5E-
4,(UncUn143:0.01596,((EchggY12:0.00371,Ult21733:5.4E-4)0.839.26:5.5E-
4,(Ult21734:0.00391,Ult21735:0.00354)0.016.1:0.00372)0.884.41:0.01116)0.8
98.49:0.0156)0.732.9:5.5E-
4)0.883.48:0.00788)0.923.52:0.01526)0.962.46:5.5E-
4)0.811.26:0.01558,Ult21749:0.03828)0.754.27:0.01003,(((Ult12226:0.10432,
(Ult21821:0.01477,Ult21820:0.05854)0.092.4:0.01544)0.983.20:0.07551,(Ult1
7748:0.01862,Ult18299:0.04415)0.942.43:0.03025)0.725.11:0.00563,(((Ult218
04:0.04764,(Ult21805:0.01525,(Ult21803:0.04026,(Ult21809:0.00369,Ult21806
:5.5E-4)0.961.35:5.4E-
4)0.344.4:0.00377)0.862.36:0.0195)0.944.33:0.02843,((Ult21574:0.08939,((U
lt21722:0.03911,Ult21819:5.5E-
4)0.960.38:0.01831,((Ult21759:0.0065,((Ult21797:0.01477,Ult21801:0.00822)
0.923.53:0.01302,UltRu408:0.01905)0.779.32:0.0058)0.965.42:0.02307,(Pahgg
Y57:5.4E-4,((((((Ult21817:5.5E-4,PahgYy60:0.0114)0.844.48:5.5E-
4,(Ult21814:0.0262,(((Ult21758:0.0,Ult21808:0.0):0.00751,((((((Ult21811:5
.5E-4,Ult21822:5.5E-4)0.798.26:5.4E-4,((Ult21779:5.5E-
4,((Ult21785:0.00375,Ult21778:0.0113)0.780.34:0.00368,Ult21707:0.00754)0.
786.41:0.00373)0.232.4:5.3E-
4,Ult21802:0.02637)0.804.25:0.00364)0.895.52:0.01293,Ult23885:0.00844)0.8
38.37:0.00827,(((Ult21761:0.01179,((Ult21769:0.00369,((((((Ult24027:0.014
94,Ult21813:0.00369)0.909.33:5.5E-4,Ult21753:5.5E-4)0.980.19:5.5E-
4,(Ult21786:5.5E-
4,Ult21766:0.01487)0.839.27:0.00367)0.805.26:0.00363,((PahggY55:5.5E-
4,PahggY56:0.00748)0.916.46:0.00742,(Ult21784:0.00369,Ult21772:5.5E-
4)0.926.44:5.5E-4)0.167.4:0.00369)1.000.223:5.5E-
4,((((((UltRu407:0.03543,(Ult21790:0.00762,Ult21750:0.0155)0.775.26:0.003
66)0.928.34:5.4E-
4,Ult21791:0.00707)0.857.46:0.00741,(UltRu403:0.00741,(UltRu404:0.01166,U
ltRu402:0.01144)0.779.33:0.00365)0.763.35:0.00412)0.772.36:0.00335,UltB73
30:0.0227)0.465.6:5.5E-4,Ult21751:0.00741)0.399.5:5.5E-
4,((Ult21789:0.0,Ult21760:0.0,Ult21771:0.0,Ult21770:0.0):0.00437,Ult21768
:0.00983)0.927.40:0.00437)0.559.5:5.5E-4)0.614.5:5.4E-
4,Ult21812:0.00741)0.828.24:0.00374)0.666.5:5.4E-
4,Ult21763:0.04325)0.793.25:0.00748)0.898.50:0.01128,((Ult21787:0.00725,U
ltB7227:0.10862)0.792.39:5.4E-
4,((Ult21781:0.0037,(Ult21752:0.01504,Ult21767:5.5E-
4)0.855.41:0.0037)0.846.30:5.4E-
4,UltCl106:0.02323)0.825.26:0.00752)0.938.32:0.0018)0.854.29:0.00166,((Ul

t21792:5.5E-4,Ult21762:0.01885)0.918.43:0.01434,(Ult21773:5.4E-
4,(Ult21794:0.01776,Ult21774:0.0484)0.132.8:0.01003)0.971.33:0.02571)0.84
8.29:0.01339)0.996.30:5.4E-4)0.948.47:0.00748,UltRu405:5.3E-
4)0.795.33:0.00623,Ult21765:0.04019)0.708.10:0.00231)0.808.25:0.00731,(Ul
t21815:5.3E-
4,Ult21816:0.00368)0.998.53:0.03158)0.746.25:0.00524)0.737.25:0.00658)0.7
10.11:0.00403,Ult21818:0.0495)0.847.41:0.01103,(UltCl107:0.0,UltCl108:0.0
):0.00744)0.335.6:5.5E-4,(((Ult21712:0.01116,Ult21775:5.5E-
4)0.630.7:5.5E-4,((Ult21717:0.0,Ult21777:0.0):5.5E-
4,Ult21776:0.01133)0.822.22:0.00372)0.937.41:0.01147,(Ult21780:0.00755,Ul
tRu406:0.01516)0.780.35:0.00361)0.879.43:0.00753)0.930.36:0.01502,(Ult218
07:0.01924,Ult21764:0.0153)0.760.28:0.00372)0.993.27:5.4E-
4)0.884.42:5.4E-4)0.872.47:5.4E-
4)0.911.46:0.01232)0.701.10:0.00285,((Ult21795:5.5E-
4,Ult21796:0.00369)0.937.42:0.02345,(Ult21799:0.03451,(Ult21800:0.02622,U
lt21798:0.0236)0.816.23:0.01258)0.151.3:0.01068)0.860.28:0.01353)0.922.37
:0.01711)0.869.35:0.0113,((((((((PahggY49:0.0129,UltClo83:0.01415)0.809.
26:0.00801,((Ult21671:0.03805,((UltLac44:5.4E-
4,(Ult21659:0.02318,((Ult21658:0.01117,(Ult21664:0.01131,Ult21709:0.02669
)0.642.4:5.4E-4)0.916.47:0.0035,((Ult21660:5.5E-
4,Ult21654:0.01109)0.960.39:0.015,(Ult21655:0.0,Ult21656:0.0):5.5E-
4)1.000.224:5.4E-
4)0.525.3:0.00755)0.862.37:0.00767)0.822.23:0.00974,(Ult21673:0.03423,((P
ahggY51:0.0,PahggY53:0.0):5.5E-4,PahggY52:5.5E-
4)0.783.37:0.01042)0.995.44:0.04371)0.729.11:0.00206)0.779.34:0.01541,(Ps
hggg42:0.00385,(Ult21657:0.0123,((Ult21670:0.02789,(Ult21668:0.0,Ult21669
:0.0):0.00339)0.871.40:0.01582,(Ult21565:0.00434,Ult21719:0.01844)0.770.4
6:0.00423)0.922.38:0.01513)0.913.37:0.01487)0.845.38:0.0097)0.756.41:0.00
425)0.872.48:0.00898,(PahggY45:0.0,PahYyy10:0.0,PahggY47:0.0,PahggY48:0.0
):5.5E-
4)0.891.37:0.01047,((Pshggg45:0.00371,(Bfhggg47:0.00366,(Otu01438:0.01528
,Ult21672:0.01136)0.746.26:0.0038)0.885.36:0.00754)0.907.44:5.5E-
4,((PahgYyy4:0.00207,(PshYyy14:0.0,Pshggg44:0.0):0.00209)0.855.42:0.00728
,(Pshggg43:0.00744,PahggY50:5.5E-
4)0.881.40:0.00753)0.450.4:0.00758)0.784.27:0.00559)0.882.43:0.01031,Ult2
1667:5.4E-4)0.887.42:0.00743,((Ult21653:5.5E-
4,Ult21652:0.00742)0.998.54:0.02765,(Ult21666:0.02704,(Ult21663:5.5E-
4,((((Ult21661:0.0,Ult21662:0.0):5.5E-
4,Ult23520:0.015)0.923.54:0.00744,(Ult21577:5.4E-
4,Ult26391:0.01122)0.112.3:0.00369)0.769.30:5.4E-
4,Ult21665:0.01486)0.828.25:0.00366,Ult21681:5.4E-
4)0.849.31:0.00367)0.991.35:5.4E-
4)0.921.50:0.01152)0.758.34:0.00348)0.749.40:0.0039,((((((UltRu395:0.0036
8,Ult21623:5.5E-4)0.969.33:5.5E-4,(((Ult21628:6.4E-
4,Ult21640:0.01911)0.893.46:0.01013,Ult21626:0.00146)0.940.37:0.01462,(((
UltRu399:0.0,UltRu398:0.0,Ult21625:0.0,UltRu396:0.0,Ult21633:0.0,Ult21629
:0.0):5.5E-
4,(Ult21632:0.0037,(Ult21631:0.00746,Ult18297:0.00744)0.426.12:5.5E-
4)0.840.37:5.4E-4)0.918.44:5.5E-4,UltRu397:0.00739)0.923.55:5.4E-
4)0.778.35:0.00691)0.832.26:0.00722,((Ult21635:5.5E-4,(Ult21637:5.4E-
4,(Ult21636:0.01047,(Ult21643:0.01131,(Ult21634:0.04376,Ult21644:0.00488)
0.615.6:0.01081)0.899.38:0.01544)0.884.43:0.00126)0.815.22:0.00365)0.933.
43:0.01137,(Ult21568:0.00208,(Ult18298:5.5E-
4,Ult21627:0.00369)0.932.41:0.00208)0.864.41:0.00722)0.757.25:0.00372)0.7

08.11:0.00372,(Ult21645:0.02401,UltRu400:0.064)0.890.48:0.02005)0.666.6:0
.00415,(Ult21639:0.00411,(Ult21650:0.02116,(Ult21649:0.01102,((Ult21648:0
.0,Ult21647:0.0):0.01512,Ult21646:0.02521)0.983.21:0.0351)0.737.26:0.0056
6)0.956.35:0.02496)0.439.6:0.00744)0.929.44:0.0036,((Ult21638:0.01584,(Ul
t21641:0.00741,Ult21642:5.5E-
4)0.988.43:0.02778)0.826.25:0.00699,((Ult21715:0.01174,UltRu401:0.03207)0
.753.29:0.00358,Ult21624:0.00358)0.815.23:0.00387)1.000.225:5.5E-
4)0.941.39:0.01871)0.848.30:0.00707,((((((Ult21710:0.06788,(Ult21706:5.4E
-4,Ult21684:0.00369)0.417.3:5.4E-4)0.974.18:0.02323,Ult21702:5.4E-
4)0.458.9:0.00373,(((Ult21698:0.00363,Ult21755:0.03111)0.794.27:0.00378,(
((Ult21676:0.00369,Ult21682:0.00369)0.687.11:5.5E-
4,(Ult21700:0.0,Ult21703:0.0,Ult21691:0.0,Ult21683:0.0,Ult23519:0.0):5.5E
-4)0.000.321:5.5E-4,((Ult21674:0.00369,Ult21692:0.00372)0.081.4:5.5E-
4,Ult21675:0.00369)0.731.12:5.5E-4)0.889.39:5.5E-4)0.708.12:5.5E-
4,(Ult21679:0.00747,(Ult21677:0.0074,(Ult21678:0.00369,(Ult21701:0.0,Ult2
1716:0.0):5.5E-4)0.605.5:5.5E-
4)0.813.24:0.00371)0.790.29:0.00369)0.952.39:0.00757)1.000.226:5.5E-
4,((UltClo85:5.5E-
4,(Ult21708:0.00757,Ult21689:0.01149)0.887.43:0.00755)0.837.24:0.0037,(Ul
t21810:0.04037,((Ult21704:0.0,Ult21694:0.0):5.4E-
4,(((((Ult21699:0.01557,(Ult24753:0.02321,(Ult21690:0.01539,(Ult21697:0.0
0747,Ult21695:5.4E-
4)0.869.36:0.00755)0.745.31:0.00368)0.872.49:0.00775)0.952.40:5.3E-
4,Ult21693:0.00717)0.886.50:0.00627,(Ult21686:0.02375,((Ult21685:0.0,Ult2
1687:0.0):5.5E-
4,Ult21688:0.00746)0.952.41:0.01592)0.772.37:0.00615)0.806.16:0.00609,(Ul
t21680:0.00738,Ult21696:5.5E-4)0.740.28:5.3E-
4)0.780.36:0.00764,Ult21705:0.01133)0.953.31:5.5E-
4)0.826.26:0.00374)0.823.24:5.3E-
4)0.872.50:0.00361)0.519.4:0.00811,(Ult23532:0.01254,Ult14086:0.12292)0.2
45.3:0.01521)0.964.45:0.02556,UltB7292:5.3E-
4)0.799.33:0.00386)0.911.47:0.01221,(Ult27003:0.05713,((Ult12227:0.02546,
Ult21584:0.00751)0.985.34:0.03373,(((((Ult21726:5.4E-
4,((Ult21576:0.01162,(Ult21757:0.03234,Ult21573:0.16184)0.817.18:0.0279)0
.603.11:0.01609,(Ult21571:0.0,Ult21728:0.0):5.4E-
4)0.918.45:0.00732)0.835.33:0.0037,Ult21729:5.3E-
4)0.878.40:0.00371,((Ult21619:5.4E-4,(Ult21605:0.00371,Ult21621:5.4E-
4)0.848.31:0.00368)0.180.3:5.5E-
4,((Ult21586:0.0131,Ult21583:0.00847)0.827.25:0.00967,(Ult21592:0.0183,(U
lt21578:0.02342,Ult24973:0.00389)0.767.30:0.0041)0.665.11:0.00761)0.814.2
5:0.00664)0.845.39:0.00364)0.895.53:5.4E-4,((((Ult21597:5.4E-
4,Ult21566:0.02301)0.819.28:0.00365,UltClo84:0.02724)0.090.3:5.4E-
4,((Ult21580:0.0919,(Ult21581:0.01487,(((Ult21579:0.02267,Ult21570:0.0036
8)0.673.2:5.5E-4,(Ult21582:0.00369,Ult21754:0.00742)0.629.7:5.5E-
4)0.000.322:5.5E-
4,(Ult21756:0.0,Ult21575:0.0,UltChris:0.0,Ult21572:0.0):5.5E-
4)0.908.40:5.4E-4)0.999.67:5.4E-4)0.469.5:0.0036,Ult21651:5.5E-
4)0.856.29:0.00362)0.903.38:0.00726,((((((Ult21599:0.0,UltRu390:0.0,UltRu
389:0.0,Ult21616:0.0,Ult21618:0.0,Ult21595:0.0,Ult21596:0.0,Ult21606:0.0,
UltRu381:0.0):5.5E-4,(Ult21615:0.0,Ult21607:0.0):5.5E-4)0.013.2:5.5E-
4,(UltRu392:0.00368,Ult21620:0.0037)0.515.2:5.5E-4)1.000.227:5.4E-
4,((((UltRu394:0.00372,(UltRu382:0.00367,UltRu384:5.5E-
4)0.951.39:0.01133)0.899.39:0.00213,(Ult21604:5.5E-
4,((Ult21611:0.0,Ult21612:0.0):5.1E-

4,UltRu391:0.0037)0.846.31:0.00369)0.903.39:0.00207)0.956.36:0.00633,(UltRu383:5.5E-4,((Ult21601:5.5E-4,(Ult21613:5.5E-4,Ult21614:5.5E-4)0.444.6:5.5E-4)0.870.28:0.00368,(Ult21600:0.0075,UltRu386:0.01876)0.660.7:5.5E-4)0.849.32:0.00365)0.126.4:0.00207)0.907.45:0.00208,(Ult21598:0.00368,(Ult21617:5.5E-4,(Ult21593:0.0,Ult21594:0.0):0.00738)0.856.30:0.00367)0.836.25:5.5E-4)0.884.44:0.00357)0.086.6:0.00364,(UltRu385:5.5E-4,UltRu393:0.00368)0.907.46:0.0036)1.000.228:5.1E-4,(Ult21588:5.5E-4,Ult21590:0.00367)0.977.29:5.4E-4)0.957.37:5.5E-4)0.856.31:0.00357)0.880.44:0.00731,((((Ult21725:0.01048,UltClo82:0.00613)0.780.37:0.00595,Ult21585:0.00764)0.725.12:0.00749,Ult21567:5.4E-4)0.944.34:0.00725,(UdnRum32:0.01519,(Ult21589:0.0021,((Ult21622:0.01124,(Ult21603:0.00328,(((((Ult21793:0.0,Ult21724:0.0):5.5E-4,Ult21730:0.00742)0.946.42:0.00745,(Ult21569:0.0074,(Ult21713:0.0,Ult21714:0.0):5.5E-4)0.574.4:5.5E-4)0.907.47:0.00994,(Ult21591:0.00376,(Ult21610:5.4E-4,((UltRu387:5.4E-4,UltRu380:0.04396)0.876.35:0.01107,(Ult21609:5.5E-4,UltRu388:0.00744)0.062.4:5.4E-4)0.932.42:0.00745)0.956.37:0.01151)0.838.38:0.00405)0.890.49:0.00529,(Ult21608:0.01155,Ult21602:0.01547)0.847.42:0.00732)0.029.1:0.00767)1.000.229:5.5E-4)0.811.27:0.00371,(Ult21727:0.01485,Ult21587:0.01497)0.874.58:5.5E-4)0.928.35:0.00694)0.907.48:0.00207)0.910.55:5.4E-4)0.757.26:0.00353)0.711.14:0.0039)0.602.3:0.00772)0.719.5:0.00294)0.767.31:0.0048)0.859.39:0.01312)0.862.38:0.01411)0.974.19:0.03568)0.951.40:0.03067,(Ult21882:0.05795,((Ult21880:0.02784,((PahggY62:0.01197,(UncUn155:0.00748,(UncUn153:0.00369,UncUn154:0.00369)0.698.10:5.5E-4)0.881.41:0.01113)0.846.32:0.01087,(Ult21881:0.03776,UncUn156:0.03315)0.932.43:0.02283)0.778.36:0.00946)0.861.47:0.01596,(UltCl152:0.01591,(UncUn157:0.01338,Ult21883:0.04566)0.108.3:0.02448)0.850.39:0.01687)0.782.38:5.4E-4)0.995.45:0.05766)0.874.59:0.01922)0.858.34:0.01191,(((((((((CsrUltun:0.03314,Ult24383:0.02028)0.762.37:0.02641,(CsdBact5:0.04203,(CstBact9:0.01097,(TpmFerri:0.0417,(CstBact8:5.4E-4,Udntfd12:0.00754)0.952.42:0.02309)0.050.4:0.00317)0.932.44:0.02133)0.862.39:0.01837)0.772.38:0.00614,(Ult24382:0.03394,(UltB4791:0.03148,(UltBaci3:0.00374,CsrSpe33:5.5E-4)0.985.35:5.4E-4)0.874.60:0.01229)0.449.7:0.01243)0.865.41:0.01562,(((Ult24361:0.01474,Ult24362:0.02273)0.995.46:0.05433,(Ult24376:0.02822,((Ult24375:5.4E-4,(Ult24162:0.06768,((Ult24381:0.02783,(CsrSpe32:5.4E-4,(((TsrSpec2:0.00758,TsrSpeci:0.00375)0.752.27:0.00383,Ult24380:0.01142)0.753.30:0.00369,((((BacNL352:0.00373,BacNL354:0.00206)1.000.230:0.00165,((BacNL357:0.0,BacNL358:0.0,TsrPraea:0.0,BacNL360:0.0):5.5E-4,BacNL359:5.5E-4)0.870.29:5.5E-4)0.105.4:5.5E-4,(BacNL356:0.00371,(BacNL355:5.5E-4,BacNL362:0.00749)0.862.40:0.00365)0.672.5:5.5E-4)0.857.47:5.5E-4,(BacNL353:0.00736,BacNL361:0.00302)1.000.231:6.3E-4)0.443.2:5.4E-4)0.769.31:0.0036)0.848.32:0.00727)0.862.41:0.01169,((((Ult24378:5.5E-4,Ult24379:0.00753)0.767.32:0.00414,Ult24377:0.03216)0.731.13:0.00396,UltB7983:0.07757)0.874.61:0.01063,TsrCrea2:0.01929)0.345.5:5.3E-4)0.645.6:0.01107)0.814.26:0.00758)0.874.62:0.01241,(Ult24374:0.02459,((Ult24373:5.4E-4,(Ult24372:0.04759,(UltSoehn:0.03273,(SoeSacch:5.5E-4,SoeSpeci:0.12724)0.794.28:0.00625)0.971.34:0.02755)0.823.25:0.01329)0.7

93.26:0.00392,TsrCreat:0.02367)0.752.28:0.00311)0.882.44:0.01365)0.861.48
:0.01369)0.657.6:0.00234)0.874.63:0.02296,(((((Ult24335:0.01114,(AnuHydro
:0.00764,AnuSpec2:0.0038)0.943.38:0.00181)0.905.33:0.00194,((Ult24336:0.0
0375,Ult24337:0.00375)0.865.42:5.5E-
4,Ult24341:0.03992)0.800.20:0.00408)0.962.47:0.04777,(Ult24340:0.03395,((
Ult24334:0.02677,(Ult24329:5.4E-
4,(Ult24331:0.00373,(((((Ult24323:0.0,Ult24325:0.0,Ult24326:0.0,Ult24328:
0.0,Ult24330:0.0,Ult24332:0.0,Ult24339:0.0):5.5E-4,AnuSpeci:5.5E-
4)0.883.49:5.5E-4,(Ult24324:0.01908,Ult24327:0.01524)0.307.1:5.3E-
4)0.911.48:5.3E-
4,(Ult24333:0.00375,((AnuPrev2:0.0,Ult24318:0.0,Ult24319:0.0,AnuTetra:0.0
):5.5E-
4,(((AnuTetr2:0.01052,AnuPrevo:0.00609)0.844.49:0.00602,Ult24321:5.5E-
4)0.420.7:0.00375,Ult24320:5.4E-4)0.899.40:0.00751)0.198.6:5.5E-
4)0.880.45:0.00732)0.897.33:0.00729,Ult24322:0.00759)0.999.68:5.4E-
4)0.213.4:0.00375)0.762.38:0.00668)0.927.41:0.01855,(Ult24338:0.0505,(((U
lt24305:0.0,Ult24306:0.0,Ult24307:0.0):5.3E-
4,((Ult24312:0.00374,(Ult24308:0.00746,Ult24314:0.01886)0.850.40:5.4E-
4)0.882.45:5.5E-4,((((Ult24316:5.5E-
4,Ult24315:0.00756)0.844.50:0.00372,(Ult24317:5.5E-
4,((((Ult24295:0.0,Ult24296:0.0,Ult24298:0.0):5.4E-
4,Ult24299:0.00757)0.867.43:5.4E-4,(((AnuOctav:0.0,Ult24291:0.0):5.5E-
4,Ult24297:0.00375)0.727.11:0.00293,Ult24292:0.05458)0.846.33:0.00846)0.8
34.24:0.00369,(Ult24293:5.5E-4,Ult24294:0.00374)0.988.44:5.4E-
4)0.986.28:0.01515)0.527.4:5.4E-4)0.997.37:0.03187,(Ult24304:5.4E-
4,(UltAna15:0.01176,(Ult24309:0.01143,(Ult24310:5.5E-
4,Ult24311:0.00374)0.857.48:5.4E-
4)0.756.42:0.00374)0.942.44:0.0156)0.955.32:0.01952)0.911.49:5.4E-
4,Ult24313:0.00378)0.920.49:0.00751)0.997.38:0.02768)0.919.50:0.01902,((U
lt24303:0.0,Ult24301:0.0,Ult24300:0.0):0.00376,Ult24302:5.4E-
4)0.794.29:0.00679)0.899.41:5.5E-
4)0.884.45:0.01205)0.838.39:0.00788)0.666.7:0.00297)1.000.232:0.10843,((U
lt24367:0.00751,(Ult24366:0.0,Ult24368:0.0):5.5E-
4)0.948.48:0.05383,(Ult24371:0.02397,(Ult24370:5.5E-
4,Ult24369:0.01511)0.921.51:0.03903)0.997.39:0.09731)0.679.9:0.03051)0.54
0.5:0.00873,(UltCl391:0.02941,((UltPept4:0.069,(FinMagna:0.03495,(UltFir7
0:0.00504,Ult24347:0.02256)0.969.34:0.04852)0.396.13:0.02008)0.940.38:0.0
3719,(UltPept5:0.02864,((PvmMicra:5.5E-4,PvmMicr2:0.00372)0.880.46:5.4E-
4,(Ult24352:0.0076,(PepSpeci:0.00376,Ult24351:0.00375)0.273.1:5.2E-
4)0.954.43:0.01149)0.835.34:0.01195)0.985.36:0.05111)0.636.4:0.00485)0.82
0.15:0.01116)0.941.40:0.02632)0.900.38:0.02496)0.762.39:0.01113,((((((Ult
24360:0.00705,(SwnManu3:0.03261,PpsBact2:0.04967)0.169.2:0.01651)0.822.24
:0.01499,(((PptLacri:0.00185,(PptLacr2:5.5E-
4,Ult24358:0.00752)1.000.233:0.00195)0.952.43:0.03254,(((Ult24353:0.0,Ppt
Speci:0.0,Ult24355:0.0):5.4E-
4,(((PptAsacc:0.0,Ult24354:0.0,Ult24357:0.0):5.5E-
4,Ult24356:0.00374)0.996.31:5.4E-
4,PptIndol:0.01539)0.850.41:0.00375)0.833.32:0.00373,PptTimon:5.3E-
4)0.973.20:0.03224)0.992.39:0.04259,((Ult24359:0.01171,(((UltStrep:0.0202
6,UltPept6:0.04053)0.923.56:0.02502,PptIndo2:0.00579)0.249.2:0.00933,PptA
sac2:0.00234)0.992.40:0.0575)0.228.3:0.01177,PpsBacte:0.05866)0.891.38:0.
02597)0.734.21:0.00566)0.897.34:0.02103,PepSpec2:0.01719)0.965.43:0.03887
,(PptIvori:0.01543,(Ult24365:0.00376,(Ult24363:5.5E-
4,(PptSpec2:0.00756,Ult24364:0.00755)0.765.20:5.5E-4)0.789.22:5.3E-

4)0.932.45:5.4E-
4)0.989.36:0.06083)0.891.39:0.02346,(Ult24350:0.03976,(Ult24349:0.032,Ult
24348:0.05325)0.852.34:0.03705)0.761.34:0.00678)0.922.39:0.03166,(DtsAmin
o:0.06064,EubSpec3:0.06529)0.794.30:0.01891)0.842.36:0.01303)0.944.35:0.0
259,(((((Ult24275:0.04186,(((((Ult24271:0.01574,Ult24274:0.0706)0.515.3:0
.01242,((FlfAloci:0.0,Ult24158:0.0):0.02703,FlfVillo:0.00984)0.990.36:0.0
473)0.797.26:0.02561,Ult24276:0.09101)0.777.35:5.5E-4,(Ult24277:5.5E-
4,Ult24278:0.00362)0.918.46:0.01748)0.917.38:0.0227,FsbPauci:5.3E-
4)0.902.51:0.0159)0.763.36:0.01534,TinTexco:0.00429)0.850.42:0.0144,TinCa
lif:0.01323)0.958.45:0.01825,(((GugBovis:0.01415,(Ult24280:0.04311,UltCl3
88:0.01498)0.999.69:0.07858)0.992.41:0.04969,(AnoSibir:0.03271,(((Ult2428
2:0.01576,CsrCamin:0.01576)0.957.38:0.02069,(((CloBacte:0.00786,CsrLitor:
0.00357)0.946.43:0.01956,(((TmlMetal:5.4E-
4,Ult24844:0.03556)0.938.33:0.01537,GspSubte:5.4E-
4)0.895.54:0.00762,((UltCl389:0.0,CaiSporo:0.0):0.03633,(((AnvMulti:0.003
75,NrlPepti:5.5E-4)0.942.45:0.01156,((CsrFels2:5.5E-4,CsrFormi:5.5E-
4)0.315.5:5.3E-4,(CsrAceti:5.5E-
4,NrlFerri:0.00377)0.878.41:0.00375)0.766.27:0.00383)0.804.26:0.00366,Cln
Colom:5.4E-
4)0.795.34:0.00357)0.871.41:0.00779)0.770.47:0.00473)0.968.31:0.0273,UltF
ir69:0.00836)0.758.35:0.00359)0.730.16:0.00407,(((((((((Ult24130:5.5E-
4,(Ult24044:5.5E-
4,(((Ult23766:0.0,UltCl366:0.0,Ult24000:0.0,Ult24003:0.0,Ult24007:0.0,Ult
24013:0.0,Ult24016:0.0,Ult24041:0.0,Ult24042:0.0,UltCl367:0.0,Ult24054:0.
0,Ult24055:0.0,Ult24056:0.0,Ult24129:0.0,Ult24138:0.0,Ult24142:0.0,Ult241
49:0.0,Ult24153:0.0):5.5E-4,(((Ult24008:5.5E-
4,Ult24049:0.01129)0.210.8:0.00374,(Ult24010:5.5E-
4,Ult24015:0.00376)0.886.51:0.00372)0.993.28:5.3E-
4,(((Ult24043:0.01124,Ult24012:0.03565)0.105.5:5.5E-
4,(((Ult11298:0.05215,(Ult24002:5.5E-4,Ult24011:5.5E-4)1.000.234:5.4E-
4)0.803.23:0.00369,Ult24004:0.00375)0.663.7:5.4E-
4,((((Ult24045:0.0231,Ult24001:0.0273)0.468.5:5.5E-
4,Ult24014:0.00754)0.469.6:5.5E-4,Ult24148:0.00374)0.444.7:5.5E-
4,Ult24039:0.00374)0.452.7:5.5E-4)0.000.323:5.5E-4)0.056.4:5.5E-
4,(Ult24126:0.00374,Ult24053:0.02311)0.626.9:5.5E-4)0.482.6:5.5E-
4)0.000.324:5.5E-4)0.583.3:5.5E-4,(UltCl364:0.01156,((Ult24133:5.5E-
4,Ult19746:0.01138)0.969.35:5.5E-4,((Ult23996:5.5E-
4,(((Ult23995:0.00376,(Ult24009:5.5E-
4,Ult24137:0.00375)0.836.26:0.00375)0.990.37:5.5E-
4,((Ult24038:0.00375,Ult23997:0.00752)1.000.235:5.5E-4,(Ult24141:5.5E-
4,Ult24006:0.03566)0.844.51:0.00374)0.396.14:5.5E-4)0.000.325:5.5E-
4,(Ult23998:0.0,Ult23999:0.0,Ult24145:0.0):5.5E-4)0.867.44:5.4E-
4)1.000.236:5.3E-4,(UltCl372:5.3E-4,(Ult24111:0.00374,(Ult24110:5.5E-
4,((UltCl365:0.0,BacNL343:0.0,Ult24109:0.0,SwnFeca9:0.0,CsrSpe31:0.0,UltC
l371:0.0,Ult24135:0.0,Ult24140:0.0):5.5E-
4,(CsrBifer:0.00374,(BacCCSD2:0.00755,Ult24108:0.00375)0.507.3:5.5E-
4)0.682.7:5.5E-4)0.891.40:5.5E-4)0.910.56:5.5E-
4)0.977.30:0.01951)0.917.39:0.01131)0.830.31:0.00368)0.886.52:0.00747)0.8
99.42:5.4E-4)0.133.3:5.4E-4)0.340.4:5.4E-
4)0.881.42:0.01034,(Ult23764:0.02606,(Ult21308:0.00874,(((Ult24125:0.0037
5,Ult21304:0.01534)0.906.61:5.5E-
4,Ult24127:0.02739)0.430.4:0.00379,Ult24128:0.01138)0.829.31:0.00878)0.85
6.32:0.01031)0.221.3:0.00376)0.872.51:0.00862,((Ult23761:0.0078,Ult23763:
0.01911)0.233.3:0.00727,(((((Ult24102:0.01135,(CsrDiffi:5.4E-

4,(CsrIrreg:0.00377,((Ult24144:0.01526,((Ult24060:0.00375,Ult24057:0.0037
6)0.688.6:5.5E-4,((Ult24058:0.00752,Ult23516:0.00374)0.918.47:5.5E-
4,(Ult24040:0.0,Ult24059:0.0,Ult24061:0.0,Ult24131:0.0,Ult24152:0.0):5.5E
-4)0.000.326:5.5E-4)0.928.36:5.4E-4)0.841.31:0.00375,CsrBartl:5.5E-
4)0.904.39:0.00761)0.764.32:0.00378)0.895.55:0.00735)0.908.41:5.3E-
4,(((Ult24046:0.0,Ult24094:0.0,UltCl369:0.0):5.5E-
4,(Ult24005:0.02348,((((UltCl368:0.02723,UltFir61:0.01464)0.767.33:0.0020
7,(CsrSpe30:5.5E-4,(Ult24101:0.01512,(SwnFeca8:5.4E-
4,Ult24100:0.0193)0.885.37:5.4E-
4)0.964.46:0.01929)0.655.12:0.01117)0.988.45:0.02771,(Ult24107:0.00377,(C
srSorde:5.5E-4,(((UltCl370:0.0,Ult24112:0.0,UltEub89:0.0):5.5E-
4,Ult24117:0.00376)0.979.34:5.4E-
4,(EubTenue:0.00376,((Ult24114:0.0,Ult24113:0.0,Ult24115:0.0,EubTenu2:0.0
,Ult24116:0.0):5.5E-4,Ult24105:0.00375)0.135.3:5.5E-
4)0.815.24:0.00375)0.484.2:0.00376)0.949.41:0.01531)0.693.7:0.00757)0.832
.27:5.5E-4,Ult24106:5.5E-4)0.913.38:0.00754)0.963.40:5.4E-
4)0.975.33:0.01131,(Ult24047:0.0,Ult24156:0.0):5.3E-4)0.848.33:5.4E-
4)0.779.35:0.00618,(Ult21376:0.08996,Ult24048:0.01817)0.801.26:0.00778)0.
809.27:0.00737,((Ult24123:0.0075,(((((((Ult24089:0.00751,(((Ult19488:0.0,
Ult23632:0.0,Ult23906:0.0,Ult23905:0.0,Ult24071:0.0,Ult24076:0.0,Ult24078
:0.0,Ult24081:0.0,CsrHiran:0.0,Ult24083:0.0,Ult24086:0.0,Ult24087:0.0,Ult
24092:0.0,Ult24093:0.0,Ult24095:0.0,Ult24143:0.0,Ult24150:0.0):5.5E-
4,((Ult24119:0.00375,((Ult24088:0.00764,Ult23631:0.00753)0.145.1:5.5E-
4,(Ult24073:0.0,Ult24091:0.0):0.00375)0.245.4:5.5E-4)0.000.327:5.5E-
4,Ult24085:0.00375)0.532.5:5.5E-4)0.000.328:5.5E-
4,Ult24096:0.03102)0.432.5:5.5E-4)0.484.3:5.5E-
4,(Ult24097:0.00752,(Ult24075:0.0,Ult24077:0.0):0.00375)0.000.329:5.5E-
4)0.532.6:5.5E-4,Ult24122:5.5E-4)0.000.330:5.5E-
4,(((Ult24079:0.01462,(UltB4726:5.5E-
4,Ult24124:0.00375)0.782.39:0.00559)0.881.43:0.01038,Ult24074:5.5E-
4)0.876.36:0.00375,(Ult23762:0.0,Ult24121:0.0):5.5E-
4)0.873.39:0.00375)0.442.6:5.5E-4,Ult24084:0.00751)0.497.1:5.5E-
4,(Ult24080:0.00375,Ult24072:0.00375)0.000.331:5.5E-4)0.574.5:5.5E-
4,Ult24082:0.00374)0.163.2:5.4E-
4)0.834.25:0.01277,((PepAnaer:0.00724,(PepStoma:0.00764,(Ult24099:0.00375
,(Ult24098:5.5E-4,(BacNL340:5.5E-4,BacNL341:5.5E-
4)0.932.46:0.00754)0.704.6:5.4E-4)0.967.28:0.01544)0.837.25:5.4E-
4)0.893.47:0.02433,(UltPepto:0.01124,BacNL342:0.02096)0.998.55:0.06667)0.
901.51:0.02345)0.918.48:0.01989)0.952.44:0.02728,((((Ult24050:0.0,Ult2405
2:0.0,Ult24062:0.0,Ult24064:0.0,Ult24065:0.0,Ult24134:0.0,Ult24136:0.0,Ul
t24146:0.0,Ult24147:0.0,Ult24151:0.0,Ult24154:0.0,Ult24155:0.0):5.5E-
4,(Ult24028:0.01137,(((Ult24139:0.03139,Ult24120:0.00203)0.984.20:0.00185
,Ult24069:0.00752)0.339.6:5.4E-
4,(Ult24068:0.0,Ult24067:0.0,Ult24070:0.0):5.5E-4)1.000.237:5.5E-
4)0.860.29:0.00377)0.194.2:5.5E-4,Ult24063:0.00373)0.880.47:5.5E-
4,Ult24066:0.02315)0.818.17:0.00399)0.875.46:5.5E-
4)0.931.39:0.0194)0.822.25:0.00933,(Ult24273:0.0315,NrlHisti:0.02286)0.83
4.26:0.01335)0.835.35:0.01161,BacNL344:0.0442)0.205.5:0.01239,(UltCl374:0
.02233,(Ult24160:0.0366,(FriPatag:0.00754,Ult24159:5.5E-
4)0.790.30:0.00729)0.978.24:0.03992)0.687.12:0.01451)0.844.52:0.01725,(Ak
pCroto:0.0,AkpTrans:0.0):0.00782)0.783.38:0.00472,((EubAcida:0.02837,(Ult
24161:0.0527,Ult24281:0.02708)0.850.43:0.0128)0.706.12:0.01376,AkpMetal:0
.02862)0.373.3:0.00854)0.848.34:0.00897,(AkpPepti:0.0123,(((AkpOreml:0.00
597,(Ult24165:5.4E-

4,Ult24164:0.03575)0.798.27:0.00594)0.911.50:0.01577,Ult24163:0.06792)0.8
42.37:0.00756,((TpbThala:0.01956,(SrgMesop:0.01152,(TpbFormi:5.5E-
4,(CsrPara3:0.00379,(CsrPara4:0.00374,(CsrPara2:0.0,CsrParad:0.0):5.5E-
4)0.235.6:5.5E-
4)0.969.36:0.01149)0.785.35:0.0039)0.751.18:0.00403)0.852.35:0.01591,Ult2
4118:0.06213)0.752.29:0.00541)0.753.31:0.00254)0.848.35:0.01091)0.469.7:0
.01365,((UltrdL10:0.04207,(Ult24272:0.149,BacteStr:0.02994)0.794.31:0.029
04)0.703.10:0.01638,Ult24166:0.02969)0.881.44:0.02056)0.809.28:0.01166)0.
844.53:0.01073)0.842.38:0.01155)0.905.34:5.5E-
4,((AdmHydro:0.03599,((((Ult24261:0.01115,(Ult24236:0.18153,(((((Ult24266
:5.4E-4,((Ult24255:5.5E-4,Ult24265:0.00375)0.948.49:5.5E-
4,((CsrAmin2:0.00374,Ult24254:0.01157)0.963.41:0.02009,(CsdBact4:5.5E-
4,UltCl377:0.0075)0.894.33:0.01531)0.763.37:0.00406)0.908.42:0.00756)0.82
9.32:0.00375,(BacteTC8:0.01557,((((EchggY18:0.03971,Ult24270:0.04402)0.91
9.51:0.02967,Ult24267:5.4E-
4)0.939.38:0.02987,Ult24262:0.02047)0.864.42:0.01371,Ult24256:5.4E-
4)0.821.21:0.00374)0.143.5:5.5E-
4)0.899.43:0.00766,((((UltEu100:0.01829,(Otu00186:0.02719,Z0114472:0.0127
6)0.195.2:0.00785)0.441.7:0.00468,(((Bfhggg71:0.0193,((UltEu101:5.4E-
4,(UncUn199:0.01462,Bfhggg72:0.01042)0.769.32:0.00551)0.932.47:0.00756,Pa
hggY84:5.5E-4)0.941.41:5.3E-4)0.932.48:0.00761,(Bfhggg70:5.5E-
4,PahggY83:0.00373)0.966.44:5.4E-
4)0.930.37:0.01561,UltPept2:0.02375)0.921.52:0.01515)0.135.4:0.00426,((((
((((Ult24210:0.0,Ult24211:0.0):0.05291,Ult24212:0.06251)0.665.12:0.00468,
(Ult24213:0.01027,(Ult24216:0.02114,(Ult24214:0.0037,Ult24215:0.00775)0.8
34.27:0.01056)0.776.29:0.01196)0.962.48:0.02448)0.994.38:0.0494,UltEu102:
0.0187)0.602.4:0.00923,Cuiiii29:0.03077)0.704.7:0.006,(Otu00138:0.02415,(
AtPYy147:0.03264,(Ult24263:0.04806,(UltFir68:0.01025,Try00031:0.04983)0.9
23.57:0.03498)0.556.2:0.01564)0.825.27:0.01331)0.973.21:0.02566)0.849.33:
0.01211,(((((((Ult24217:5.2E-
4,(Ult24190:0.00387,Ult24189:0.03641)0.968.32:0.01923)0.766.28:0.00358,Ul
t24218:0.00394)0.978.25:0.02352,(UltEub92:5.4E-
4,(UltEub91:0.00785,UltFir62:0.01966)0.770.48:0.00349)0.987.39:0.0196)0.0
00.332:5.5E-
4,(((EubBrach:0.04166,UltFir63:0.02465)0.683.12:0.01195,Ult27642:0.02858)
0.802.32:8.6E-
4,((Ult24242:0.00389,Ult24132:0.00367)0.938.34:0.01341,(Ult24240:0.00173,
(Ult24239:0.01552,(Ult24237:0.00375,Ult24238:0.00758)0.766.29:0.00392)0.9
54.44:0.01795)0.909.34:0.0122)0.827.26:0.00778)0.809.29:0.00541)0.911.51:
0.01136,((UltCl375:0.00375,((Ult24201:0.0,Ult24209:0.0):0.00375,Ult23280:
0.02319)0.849.34:5.5E-
4)0.904.40:0.01367,(Ult24200:0.01704,Ult24198:0.01041)0.900.39:0.01208)0.
775.27:0.0054)0.943.39:5.4E-
4,((((Ult24202:0.03519,((EubNodat:0.01069,(EubMinut:5.4E-
4,EubMinu2:0.04311)0.174.4:0.01387)0.995.47:0.0646,(Ult24204:0.00584,(Ult
24203:0.03657,EubPyruv:0.02261)0.956.38:0.03495)0.897.35:0.02416)0.844.54
:0.0182)0.935.41:0.02494,(UltEub90:0.05256,(Ult24171:0.05746,((Ult24167:0
.01016,Ult24168:0.02972)0.808.26:0.01738,((MogTimid:0.00792,MogNegle:0.00
343)0.736.19:0.00764,((Ult24173:0.02353,((Ult24174:5.5E-
4,(Ult24177:0.01133,UltRu509:0.01135)0.776.30:5.5E-4)0.967.29:5.4E-
4,(Ult24175:0.00374,Ult24172:0.01126)0.803.24:0.00366)0.775.28:0.00367)0.
924.36:0.01133,((((Ult24181:0.0,Ult24180:0.0,Ult24179:0.0):5.5E-
4,Ult24169:5.5E-4)1.000.238:0.0443,Ult24176:5.5E-
4)0.792.40:0.00361,UltRu510:0.00385)0.923.58:0.0116)0.900.40:0.01207)0.88

7.44:0.01519)0.721.6:0.00958)0.949.42:0.03743)0.698.11:0.00634)0.933.44:0
.01987,((Ult24253:0.00766,((Ult24207:0.02691,Ult24206:0.03648)0.736.20:0.
02112,Ult24170:0.03625)0.886.53:0.01654)0.431.8:0.00843,(UltCl386:0.0,Otu
00262:0.0):0.04108)0.365.3:7.2E-
4)0.753.32:0.00698,((Ult24205:0.04235,(BacNL345:0.00741,(BacNL349:0.00373
,((BacNL347:5.5E-4,(BacNL351:5.5E-4,(BacNL348:5.5E-4,BacNL346:5.5E-
4)0.979.35:0.01581)0.164.5:5.5E-4)0.257.3:5.5E-4,BacNL350:5.5E-
4)0.267.3:5.5E-4)0.518.5:5.5E-
4)0.854.30:0.01159)0.863.41:0.01146,((Ult24188:0.04671,UltAna13:0.02668)0
.869.37:0.01873,(EubInfir:0.02728,(Ult24199:0.00367,(Ult24195:0.03487,Ult
24197:5.5E-
4)0.961.36:0.01975)0.332.3:0.00373)0.912.55:0.01649)0.535.4:5.4E-
4)0.774.45:0.00531)0.846.34:0.00739)0.629.8:0.00707,((Ult24233:0.05814,((
Ult24231:0.00378,(Ult24230:5.5E-
4,Ult24225:0.00375)0.784.28:0.00377)0.822.26:0.00373,(Ult24224:5.5E-
4,((Ult24223:5.5E-
4,Ult24192:0.00375)0.888.46:0.00762,(((Ult24220:0.0038,Ult24221:0.00375)0
.918.49:0.01158,((Ult24228:0.01135,(Ult24227:5.4E-
4,Ult24191:0.0153)0.812.20:0.00374)0.294.4:5.4E-4,Ult24226:5.5E-
4)0.871.42:0.00769)0.859.40:0.00737,(Ult24229:0.03531,Ult24222:5.5E-
4)0.911.52:5.4E-4)0.857.49:0.00736)0.813.25:0.00375)0.815.25:5.5E-
4)0.994.39:0.03592)0.977.31:5.5E-4,(Ult24232:5.5E-
4,UltCl376:0.00374)0.725.13:0.00369)0.958.46:0.01981)0.888.47:5.5E-
4,(Ult24241:0.03521,((Ult24219:0.02318,UltRu513:0.00811)0.945.40:0.02027,
(Ult24196:0.00567,(Ult23514:0.07533,(Ult24235:0.00649,(Ult24234:5.5E-
4,UltRu512:0.00374)0.966.45:0.01647)0.246.5:0.00375)0.409.4:0.01131)0.986
.29:0.03189)0.139.4:0.00328)0.926.45:0.01342)0.685.8:0.00879)0.601.3:0.01
2,Ult24264:0.05936)0.896.29:0.01427)0.339.7:5.5E-
4,(((((((UncUn198:0.00757,(Ult24257:0.04028,AtPYy146:5.4E-
4)0.928.37:0.01153)0.943.40:0.01518,(UncUn197:0.01114,(UltEub95:0.00756,O
tu00404:5.3E-4)0.898.51:0.00784)0.356.3:0.0038)0.923.59:5.5E-
4,(UltEub93:0.02961,Otu00569:0.04463)0.956.39:0.02952)0.872.52:0.00932,((
(Hmyy0022:0.00147,Hmyy0038:0.10994)0.963.42:0.03315,((UltCl382:0.02403,(U
ltFir66:0.00336,UltFir67:5.3E-
4)0.911.53:0.02795)0.918.50:0.01712,(UltEub97:0.00331,UltEub96:0.03627)0.
199.3:8.2E-
4)0.829.33:0.01089)0.616.6:0.01848,(UltEub98:0.0153,(Ult24259:5.5E-
4,Otu00456:5.5E-4)1.000.239:5.4E-
4)0.878.42:0.03559)0.766.30:0.00586)0.711.15:0.00401,(EubSaphe:0.03968,((
UltCl384:0.00374,Otu00403:5.5E-4)0.988.46:0.02871,(Otu00531:5.4E-
4,((((UltCl381:0.00374,Otu00455:0.00383)0.834.28:0.01038,(Otu00241:0.0152
,((UltCl378:0.02033,UltFir65:0.03269)0.883.50:0.01673,(UltCl380:0.01448,U
ltCl379:0.02965)0.791.20:0.0123)0.846.35:0.01318)0.807.20:0.00937)0.917.4
0:0.01749,UltCl383:0.01766)0.764.33:0.00403,((((Ult24258:0.06012,(UltEub9
9:0.0,Otu00763:0.0):0.03641)0.770.49:0.01046,Z0y00000:0.01246)0.708.13:0.
00522,((Z0114442:0.02073,(UltFir64:0.04764,Hmyy0021:0.02667)0.672.6:0.002
78)0.905.35:0.01433,(Hmyy0020:0.01817,(Sgffff11:0.06959,Hmyy0039:0.03643)
0.331.3:0.02037)0.824.30:0.01448)0.851.39:0.0083)0.858.35:0.00946,(Otu000
65:0.01878,Otu00085:0.01719)0.916.48:0.01367)0.784.29:0.00701)0.783.39:0.
01827)0.928.38:0.01445)0.929.45:0.01717)0.778.37:0.00732)0.017.4:0.01099,
((UltLac52:0.0,Otu00767:0.0):0.02412,Ult24243:0.02393)0.759.35:0.00447)0.
485.6:0.01099,((Ult24251:0.00948,(Ult24269:0.05869,UltEub94:0.05333)0.865
.43:0.01498)0.865.44:0.01031,(UdnEuba5:0.01909,Ult24268:5.5E-
4)0.849.35:0.00781)0.792.41:0.00291)1.000.240:5.5E-

4)0.799.34:0.00355)0.762.40:0.00394,((Cuiiii30:0.02299,(AnxOdori:0.01691,
Ult24252:0.01075)0.774.46:0.00784)0.807.21:0.00879,((((((Ult24185:0.028,U
lt24186:0.01958)0.965.44:0.02798,(Ult24183:0.01522,Ult24182:5.4E-
4)0.794.32:0.00848)0.510.5:0.01129,Ult24187:0.0154)0.665.13:0.00262,((Ult
24184:0.04109,UltRu511:0.00458)0.891.41:0.02846,EubSpec2:0.06685)0.919.52
:0.02298)0.939.39:0.02586,(Ult24249:0.06135,(((UltB4752:0.01504,Ult24250:
5.4E-4)0.974.20:0.02697,((Ult24248:5.5E-
4,(Ult24247:0.01385,(Ult24246:0.00882,Ult24178:0.0148)0.992.42:0.03862)0.
959.49:0.02507)0.802.33:0.01065,UltRu514:0.03736)0.726.9:0.01471)0.257.4:
0.0066,(Ult24244:0.0297,Ult24245:0.02089)0.920.50:0.02382)0.821.22:0.0158
2)0.968.33:0.04219)0.875.47:0.01567,(UltCl385:0.05322,(UncUn195:0.00374,U
ncUn196:5.5E-4)0.949.43:0.02919)0.877.55:0.02021)0.888.48:5.5E-
4)0.621.7:0.00394)0.949.44:0.02245,Ult24194:0.00807)0.767.34:0.01146)0.41
6.9:0.01229)0.781.34:0.01579,Ult24260:0.04481)0.859.41:0.01511,Otu00166:0
.03792)0.331.4:0.00949,UltB5049:0.18312)0.905.36:0.02441)0.711.16:0.01216
,(UltCl387:0.08012,Ult24279:0.01243)0.930.38:0.02834)0.899.44:0.02031)0.8
03.25:0.00491)0.512.3:0.00476,((((HecSueci:0.02321,UltPept3:5.4E-
4)0.688.7:0.01941,(Ult24342:0.0152,Ult24343:0.0237)0.943.41:5.4E-
4)0.788.27:0.01257,(HecoOvis:0.01626,HecKunzi:0.04732)0.118.3:0.01448)1.0
00.241:0.12368,(Ult24157:0.09008,(EubYyyyy:0.01236,(EubYuri2:5.4E-
4,EubYurii:5.5E-
4)0.879.44:0.0196)0.995.48:0.07574)0.774.47:0.01222)0.865.45:0.02736)0.95
3.32:0.02895)0.802.34:0.01497,(((Ult24384:0.0274,SbtAceti:0.00937)0.954.4
5:0.02429,(IroBac21:0.01712,PtnEthan:0.03645)0.855.43:0.02355)0.667.8:0.0
1105,(GarNitra:0.04318,((CbcAzore:0.00376,(CssPauci:0.04162,UltOr291:0.04
167)0.865.46:0.01097)0.860.30:0.01121,(Ult24385:0.01933,(UltOr292:0.09199
,TmhBerre:0.03911)0.857.50:0.01374)0.749.41:0.00416)0.961.37:0.03152)0.82
9.34:0.01191)0.739.25:0.01548)0.866.39:0.02413,(((PatPenet:0.15513,(Ult25
844:0.07181,UltPept7:0.15441)0.157.4:0.02326)0.737.27:0.01947,(Ult21913:0
.07861,((AicMacro:0.01157,(((((SfbSpeci:0.02853,AicToler:0.00239)0.982.23
:0.03453,(AicSpec2:0.03621,(AicDisul:0.02826,AicSpeci:0.00685)0.844.55:0.
00932)0.726.10:0.01094)0.703.11:0.00214,((AicConta:0.01978,(AicFerro:0.02
355,AicPomor:0.00728)0.867.45:0.01809)0.750.20:0.01368,(AicCyclo:0.05168,
((Ult25585:0.01903,(AicHespe:5.4E-4,(AicAcido:5.5E-4,(AicAcid2:5.5E-
4,AicVulca:0.00373)0.816.24:0.00368)0.997.40:0.03132)0.770.50:0.00395)0.5
26.3:0.00639,(AicAcid3:5.4E-4,(AicAcidi:0.02702,AicFasti:5.4E-
4)0.190.2:0.00368)0.816.25:0.0068)0.714.11:0.00327)0.095.1:0.01408)0.855.
44:0.01276)0.403.4:0.00233,((GrpIrono:0.054,(((Ult25587:0.03113,(((Ult255
89:5.5E-
4,Ult25590:0.03499)0.952.45:0.02393,(UltFir91:0.16736,Ult25591:0.02076)0.
913.39:0.03248)0.343.1:0.01106,BacS1201:0.00324)1.000.242:5.5E-
4)0.399.6:0.01281,((Ult25592:0.01666,Ult25593:0.02641)0.801.27:0.00729,Ai
cPohli:0.04864)0.972.33:0.02574)0.881.45:0.01998,(TumPerma:0.0136,Ult2558
8:0.02655)0.581.6:0.00917)0.945.41:0.04067)0.914.44:0.04133,Ult25586:0.03
206)0.975.34:0.04581)0.397.6:0.02901,(AicShizu:0.01957,AicHerba:0.01589)0
.966.46:0.04178)0.513.2:0.03169)0.981.32:0.0739,(UltAlicy:0.0167,(UltAlic
2:0.0,UltAlic3:0.0):0.01031)0.985.37:0.07513)0.970.38:0.0619)0.037.1:5.4E
-
4)0.838.40:0.01551,((Ult13129:0.04393,UltHala5:0.0736)0.964.47:0.04511,((
CdsFijie:0.04075,(CdsPolys:5.5E-
4,CdsZeae0:0.00372)0.737.28:0.00341)0.998.56:0.08021,(TmrBryan:0.02216,(T
mrAcidi:5.4E-
4,(TmrSacch:0.00778,(Ult24410:0.00742,(TmbBrock:0.0037,(TmrTherm:0.0,Ult2
4409:0.0,Ult24411:0.0):5.5E-4)0.679.10:5.5E-

4)0.762.41:0.00356)0.212.5:0.00371)0.842.39:5.7E-
4)0.997.41:0.08592)0.536.5:0.01507)0.932.49:0.03722)0.889.40:0.02468)0.72
0.10:0.01573,(((((((UltB4749:0.04458,(UltB4748:0.02946,((((UltB4654:0.00
756,(UltB4655:0.0,UltB4661:0.0,UltB4662:0.0):5.5E-
4)0.967.30:0.00716,((UltB4747:5.5E-4,(((((UltB4656:5.4E-
4,UltB4665:0.0075)0.426.13:0.00374,(UltB4716:0.01104,(UltB4720:0.01912,((
(UltB4635:0.00375,((((((UltB4666:5.3E-4,(UltB4644:5.5E-
4,(UltB4642:0.00374,UltB4663:0.00374)0.113.5:5.3E-
4)0.922.40:0.00374)0.741.19:5.4E-
4,UltB4650:0.03925)0.843.29:0.00374,(((((UltB4647:0.13237,UltB4717:0.0365
5)0.908.43:0.04626,UltB4653:0.01177)0.802.35:0.00732,UltB4649:0.00753)0.7
26.11:0.0038,(UltB4646:5.4E-
4,UltB4648:0.54178)0.822.27:0.00352)0.793.27:0.00382,(UltB4636:0.03944,Ul
tB4718:0.02315)0.477.5:5.3E-4)0.515.4:5.5E-4)0.180.4:5.5E-
4,UltB4719:0.00375)0.474.7:5.5E-4,UltB4637:0.00374)0.407.5:5.4E-
4,UltB4641:0.00374)0.315.6:5.5E-4,UltB4671:0.00374)0.000.333:5.5E-
4)0.469.8:5.5E-
4,(UltB4634:0.0,UltB4638:0.0,UltB4639:0.0,UltB4643:0.0,UltB4645:0.0,UltB4
664:0.0,UltB4674:0.0):5.5E-4)0.511.6:5.5E-
4,UltB4672:0.00374)0.674.6:5.5E-
4)0.918.51:0.00202)0.913.40:0.00203)1.000.243:5.5E-4,UltB4659:5.5E-
4)0.499.2:5.4E-
4,(UltB4640:0.0,UltB4651:0.0,UltB4652:0.0,UltB4658:0.0,UltB4660:0.0,UltB4
673:0.0):5.5E-4)0.552.3:5.5E-
4,UltB4657:0.00755)0.844.56:0.00382)0.875.48:5.4E-
4,UltB4669:0.01909)1.000.244:5.4E-4)0.000.334:5.5E-
4,((((((((UltB4679:0.01947,(PrgModes:0.01934,(IlyPolyt:5.4E-
4,((IlyInsue:0.0,UltFuso7:0.0):5.3E-
4,IroEnric:0.00375)0.796.22:0.00373)0.241.3:0.00375)0.631.8:5.5E-
4)0.902.52:0.00758,(UltFuso5:0.00292,(UltFuso3:0.00434,UltFuso4:0.00799)0
.784.30:0.00771)0.920.51:8.1E-4)0.733.16:5.4E-
4,UltFuso6:0.01116)0.893.48:0.01767,(UltCetob:0.04543,(CtbcCeti:5.5E-
4,UltB4675:0.00755)0.839.28:0.0179)0.927.42:0.02541)0.933.45:0.02004,(Ult
B4624:0.01999,(Otu00264:0.0268,Otu00312:5.4E-
4)0.991.36:0.04285)0.810.25:0.01368)0.850.44:0.00909,(UltB4715:0.03545,((
UltProka:5.5E-4,(UltB4743:5.5E-
4,(UltB4742:0.00752,((UltFuso8:0.00757,(UltPsyc2:0.0,UltPsyc3:0.0):0.0037
6)0.638.4:5.5E-4,(PclAtlan:0.0,UltB4681:0.0):5.5E-
4)0.852.36:0.00375)0.969.37:5.5E-4)0.478.4:5.5E-
4)0.996.32:0.0018,UltB4680:0.00203)0.986.30:0.0343)0.666.8:0.01497)0.877.
56:0.01331,(UltB4798:0.02312,((UltB4722:0.0,UltFus13:0.0,UltB4721:0.0):5.
5E-
4,UltB4723:0.00375)0.752.30:0.00378)0.793.28:0.00612)0.828.26:0.00917,(((
UltB4670:5.5E-4,(UltB4678:0.01149,UltB4744:5.3E-
4)0.866.40:0.00368)0.670.7:0.01111,(UltB4677:0.00754,(UltB4728:5.5E-
4,UltB4676:0.00373)0.770.51:5.5E-
4)0.990.38:0.03199)0.476.7:0.00395,((((((UltB4733:0.00373,(((UltB4633:5.5
E-
4,(((((UltB4632:0.01129,((FusNecro:0.0,FusSpec2:0.0):0.00755,UltFuso2:5.5
E-4)0.917.41:5.3E-
4)0.891.42:0.0129,(UltB4626:0.01868,((UltB4620:0.0196,((UltB4616:0.01168,
(UltFusob:5.5E-
4,FusSpeci:0.00759)0.899.45:0.00786)0.920.52:0.00326,((FusNucl5:0.00376,(
((UltEuba6:0.00756,(((UltB4625:5.5E-

4,(UltB4615:0.0038,UltB4729:0.00753)0.597.7:5.5E-
4)0.908.44:0.00374,(FusNucle:0.0,FusNucl2:0.0,UltB4618:0.0):5.5E-
4)0.657.7:5.4E-4)0.849.36:0.00376,(FusNucl3:0.0,UltEuba7:0.0):5.5E-
4)0.838.41:0.00375,UltB4617:5.5E-4)0.092.5:5.5E-
4)0.832.28:0.00374,FusNucl4:5.3E-4)1.000.245:5.5E-
4)0.919.53:0.01256)0.744.25:0.00333,UltB4619:0.02511)0.600.5:0.00943)0.88
5.38:0.0152)0.311.3:0.01102,UltB4732:0.00331)0.998.57:5.4E-
4,UltB4738:0.0158)0.665.14:5.5E-4,UltB4668:0.00375)0.406.5:5.5E-
4)0.000.335:5.5E-
4,(UltB4627:0.0,UltB4628:0.0,UltB4735:0.0,UltB4734:0.0,UltB4737:0.0,UltB4
741:0.0,UltB4740:0.0):5.5E-4)0.163.3:5.5E-
4,((Ult20616:0.00743,(UltB4622:5.4E-4,(UltB4667:5.3E-
4,Ult21022:0.00374)0.881.46:0.00755)0.781.35:0.00393)0.776.31:0.00758,Ult
B4725:0.0074)0.871.43:5.4E-4)0.254.3:5.5E-4)1.000.246:5.4E-
4,(BacNLA69:0.00234,(BacNLA67:0.00477,((BacNLA73:0.00764,(BacNLA55:5.5E-
4,(((BacNLA60:0.00371,(BacNLA56:0.00746,(BacNLA54:0.0,BacNLA51:0.0,BacNLA
58:0.0,BacNLA59:0.0,BacNLA61:0.0):5.5E-4)0.471.8:5.5E-4)0.696.4:5.5E-
4,(((BacNLA52:0.00383,BacNLA49:0.00743)0.027.3:0.00377,BacNLA50:0.00735)0
.921.53:5.5E-4,(BacNLA48:5.5E-4,BacNLA53:5.5E-4)0.988.47:5.5E-
4)0.451.8:5.5E-4)0.377.5:5.5E-
4,BacNLA57:0.0037)1.000.247:0.00224)0.287.4:8.2E-4)0.926.46:5.2E-
4,(BacNLA68:5.5E-4,(BacNLA72:5.5E-4,(BacNLA65:5.5E-4,((BacNLA66:5.5E-
4,(BacNLA70:5.5E-4,((BacNLA62:5.5E-4,BacNLA63:5.5E-
4)0.912.56:0.00375,(BacNLA64:5.3E-4,P0114150:5.5E-4)0.603.12:5.5E-
4)0.552.4:5.5E-4)0.235.7:5.5E-4)0.000.336:5.5E-4,BacNLA71:5.5E-
4)0.177.8:5.5E-4)0.384.3:5.3E-4)0.000.337:5.5E-4)0.644.2:5.5E-
4)0.737.29:5.5E-
4)0.886.54:0.0016)0.950.34:0.01039)0.917.42:0.00733,(FusMorti:0.00374,((U
ltB4621:0.0,UltB4623:0.0,UltB4631:0.0,UltB4736:0.0):5.5E-
4,(UltB4630:0.00377,UltB4730:0.00375)0.761.35:5.5E-4)0.798.28:5.4E-
4)0.833.33:5.5E-
4)0.740.29:0.00366,(UltB4727:0.03059,UltB4731:0.04363)0.823.26:0.00971)0.
884.46:0.00741,(UltB4629:0.0,UltB4739:0.0):0.00756)0.865.47:0.00733,UltB4
724:5.5E-4)0.723.17:5.4E-4)0.888.49:0.01536)0.000.338:5.5E-
4,(UltFus12:0.03367,(UltFus11:0.00319,(((((UltB4698:0.05828,LptSpe12:0.02
176)0.895.56:0.02138,(((LptGoodf:0.02992,LptSpe11:0.01974)0.901.52:0.0190
8,(((UltLept9:0.00783,(UltB4696:0.02414,UltLept8:0.00731)0.777.36:0.00388
)1.000.248:5.3E-4,(UltB4691:0.00273,(UltLep10:0.00377,(UltB4697:5.4E-
4,UltLep11:0.00724)0.873.40:0.00732)0.937.43:0.00214)0.737.30:0.00195)0.9
72.34:0.03188,((((UltB4690:5.5E-4,((UltB4683:5.5E-4,UltB4692:5.5E-
4)0.311.4:5.5E-4,(UltB4695:0.0074,UltLept3:0.00744)0.903.40:5.5E-
4)0.549.7:5.5E-4)0.911.54:5.5E-4,((LptSpeci:0.01901,(LptSpec2:5.5E-
4,(UltLepto:0.0037,UltB4682:0.0037)0.713.12:5.5E-4)0.939.40:5.4E-
4)0.820.16:0.00368,(UltLept2:0.0,UltB4694:0.0):5.4E-
4)0.929.46:0.01119)0.339.8:0.0037,LptSpec3:0.00734)0.800.21:0.00706,(((Ul
tB4684:5.5E-
4,UltB4693:0.02666)0.925.49:0.02122,(LptSpec5:0.01641,LptSpec6:0.06448)0.
782.40:0.01537)0.989.37:0.04441,(LptSpec4:5.5E-
4,((LptSpec8:0.01307,((UltLept4:0.0,UltLept5:0.0):0.01574,((UltB4685:0.0,
LptShahi:0.0,UltLept7:0.0):0.0114,((LptSpec9:0.01144,UltB4687:5.5E-
4)0.966.47:0.01513,(LptSpe10:0.00369,(UltB4688:0.0,UltB4689:0.0):5.5E-
4)0.776.32:0.00373)0.732.10:0.00377)0.840.38:0.00719)0.943.42:0.02197)0.6
46.6:0.01214,(LptSpec7:0.0,UltB4686:0.0,UltLept6:0.0):5.3E-
4)0.902.53:0.00324)0.760.29:0.00432)0.149.3:0.00816)0.904.41:0.01591)0.95

1.41:0.02351)0.970.39:0.03579,UltLep12:0.01646)0.589.8:0.00744)0.695.9:0.
00724,((UltB4706:5.5E-
4,((UltB4699:0.0,UltB4700:0.0,UltB4701:0.0,UltB4703:0.0,UltB4705:0.0,UltB
4707:0.0):5.5E-4,(UltB4702:5.5E-4,UltB4704:5.5E-4)0.000.339:5.5E-
4)0.000.340:5.5E-4)0.939.41:0.02213,(((SebTermi:0.0,Otu00306:0.0):5.4E-
4,(Otu00538:0.0217,UltB4708:0.01743)0.873.41:0.01154)1.000.249:0.06591,(U
ltB4712:0.03387,(UltB4711:0.08416,(UdnBac21:0.10768,((UltB4709:0.00537,Lp
tAmnio:0.01809)0.645.7:0.00711,(((StbMonil:0.0,StbMoni3:0.0):5.5E-
4,StbMoni2:5.5E-
4)0.916.49:0.01106,((LptSpe13:0.00742,(UltEuba8:0.00369,(LpcSpeci:0.0,Ult
Euba9:0.0):5.5E-4)0.262.3:5.5E-
4)0.905.37:0.00792,UltB4710:0.02751)0.591.7:5.4E-
4)0.932.50:0.01817)0.903.41:0.01446)0.268.1:0.00146)0.877.57:0.01161)0.88
6.55:0.01301)0.758.36:0.00653)0.963.43:0.04058)0.945.42:0.05028,(UltB4714
:0.07078,UltFuso9:0.04801)0.980.20:0.07284)0.989.38:0.10954,UltFus10:0.01
473)0.211.7:0.00947)0.922.41:0.02518)0.992.43:0.04511)0.934.40:0.02799)0.
999.70:0.06068,(UltB4746:0.01704,UltB4751:0.01999)0.823.27:0.01659)0.676.
3:0.00507)0.992.44:0.07337)0.994.40:0.08235,((Ult25173:0.13173,((Ult24907
:0.14602,(UltFir88:0.21687,(XxxCandi:0.11272,(((Ult25169:0.0,Ult25170:0.0
):5.5E-
4,Ult25171:0.00371)0.922.42:0.031,(Ult25172:0.01649,CanLumbr:0.04308)0.86
4.43:0.03332)0.806.17:0.021)0.908.45:0.03868)0.809.30:0.04811)0.395.6:0.0
0376,(((McmHomin:0.00757,McmEquir:0.00389)0.465.7:0.00761,((McmGatea:0.00
37,((McmArgin:5.5E-
4,(McmIndie:0.00374,(((McmBucca:0.00768,(McmFauci:0.0,McmHyosy:0.0,McmFal
co:0.0):5.4E-4)0.940.39:5.4E-
4,McmSaliv:0.0075)0.029.2:0.00381,McmSubdo:0.00371)1.000.250:5.5E-
4)0.323.4:5.4E-4)0.275.6:5.5E-
4,(McmPhoc2:0.01545,McmAnser:0.01141)0.597.8:5.5E-4)1.000.251:5.4E-
4)0.784.31:0.00956,McmSpuma:0.00381)0.659.6:5.4E-
4)0.724.15:0.00803,(McmGypis:0.01631,(((((((((McmSynov:0.0207,McmGall2:0.0
1395)0.927.43:0.01546,(((McmGlyco:0.00371,(McmCorog:0.00332,(McmFelis:0.0
0788,McmMuste:0.01101)0.403.5:0.00754)1.000.252:5.4E-4)0.905.38:5.5E-
4,(McmLeoni:0.01959,((McmSpec2:5.3E-4,(McmGallo:5.4E-
4,McmButeo:0.01487)0.916.50:0.01455)0.773.34:0.00373,((McmCynos:0.00372,M
cmCanis:5.4E-
4)0.782.41:0.00374,McmBovir:0.00749)0.890.50:0.00752)0.773.35:0.00368)0.8
03.26:0.00687)0.840.39:0.01493,(McmSpec3:0.01896,McmVerec:0.00399)0.846.3
6:0.00801)0.768.21:0.00472)0.943.43:0.01414,McmMelea:0.01921)1.000.253:5.
4E-
4,(((McmColu2:0.00768,(McmIners:0.00731,McmColum:0.0155)0.880.48:0.00772)
0.852.37:0.00711,(((McmCalif:5.4E-
4,(((McmAdler:0.00371,McmFelif:0.01147)0.789.23:0.00373,(McmOpale:0.01129
,McmBovig:0.00372)0.867.46:5.3E-
4)0.803.27:0.0037,(McmSimba:0.00374,(McmHyoph:0.0392,McmPhoci:0.00719)0.9
11.55:5.4E-4)0.926.47:0.00756)0.984.21:5.4E-
4)0.802.36:0.0038,(McmCavia:0.00439,(((McmBovis:5.5E-
4,McmPrima:0.00751)0.742.18:0.0039,(McmLeoph:0.0085,McmSpeci:0.01409)0.84
7.43:0.01208)0.453.6:0.00665,McmSperm:0.02537)0.763.38:0.00506)0.834.29:0
.00687)0.750.21:0.00412,McmLipof:0.01881)0.428.5:0.00759)0.753.33:0.00418
,(McmAnati:0.01534,(McmCroco:0.00752,McmAllig:5.4E-
4)0.948.50:0.01544)0.914.45:0.01155)0.926.48:0.00326)0.760.30:0.00525,(Mc
mGalli:0.03361,(McmOxoni:0.00972,(McmColu3:5.5E-
4,(McmCitel:0.01138,McmSturn:0.03896)0.996.33:5.4E-

4)0.782.42:0.00526)0.369.5:0.01207)0.819.29:0.01567)0.996.34:0.04382,(Mcm
Eleph:0.0167,Ult25174:0.00273)0.971.35:0.03297)0.891.43:0.02175,(CanMyco5
:0.06577,((McmPulmo:0.06308,(((McmHyor2:0.01598,McmHyorh:0.00364)0.788.28
:0.00747,((McmOvipn:0.01529,(McmDispa:5.5E-
4,(McmFlocc:0.00754,McmHyopn:0.00372)0.882.46:0.00754)0.997.42:5.5E-
4)0.849.37:0.00798,(McmBovoc:0.00608,McmConju:0.00607)0.853.32:0.01019)0.
958.47:0.02485)0.970.40:0.03531,(McmIguan:0.01842,((McmMolar:0.00372,McmL
agog:5.3E-
4)0.907.49:0.01292,McmColli:0.04879)0.341.7:0.00343)0.883.51:0.01895)0.54
7.4:0.02534)0.752.31:0.03417,(McmTest2:0.07361,McmAgass:0.01921)0.959.50:
0.03869)0.597.9:0.00503)0.805.27:0.01257)0.927.44:0.02573,(UltMyc19:0.091
59,((Ult25175:0.01864,(McmMoats:0.00826,McmSualv:0.04603)0.729.12:0.01841
)0.994.41:0.0484,(McmMobil:0.01266,(UltMyc17:0.02954,UltMyc18:0.02067)0.9
29.47:0.02207)0.945.43:0.02093)0.319.4:0.00668)0.398.5:0.01101)0.660.8:0.
01692)0.836.27:0.01706)1.000.254:0.11363)0.492.7:0.01443)0.561.5:5.4E-
4,(UltOr294:0.08885,(UdnRum37:0.12395,((((Ult25549:0.0639,Ult25551:0.0289
4)0.871.44:0.02691,(Ult25550:0.03571,(Ult25548:0.03121,Ult25552:0.06211)0
.920.53:0.03304)0.393.5:0.01466)0.984.22:0.06291,(Ult25555:0.01181,(Ult25
553:0.06225,Ult25557:0.02061)0.722.6:0.00813)0.818.18:0.01263)0.565.4:0.0
3346,(Ult25556:0.03839,Ult25554:0.19779)0.748.27:0.01909)0.836.28:0.02297
)0.337.3:0.00855)0.926.49:0.03425)0.848.36:0.01956)0.319.5:0.01465,((((((
UltErys4:0.01899,(EubSpec4:5.3E-
4,UltErys3:0.01947)0.924.37:0.01612)0.593.3:0.00791,((Ult25407:0.04644,(U
lt25405:0.03491,Ult25406:0.01648)0.904.42:0.02595)0.822.28:0.02284,(Ult25
583:0.00307,Ult25584:0.00828)1.000.255:0.09264)0.733.17:0.00975)0.768.22:
0.00669,((Ult25409:0.00379,Ult25408:0.00374)0.928.39:0.02007,(Ult25412:0.
07848,(((Ult25547:0.09585,((((((Ult25337:0.04493,Ult25341:0.01961)0.955.
33:0.03029,((Ult25320:0.01885,(Ult25352:5.5E-4,Ult25351:5.5E-
4)0.905.39:0.00747)0.775.29:5.5E-4,((((Ult25338:0.0189,Ult25325:5.4E-
4)0.785.36:0.00371,(Ult25335:0.00371,Ult25336:0.00371)0.797.27:0.00371)0.
903.42:5.4E-
4,(Ult25324:0.00371,((Ult25347:0.00371,Ult25342:0.00372)0.709.8:5.5E-
4,(Ult25340:0.0,Ult25344:0.0,Ult25345:0.0,Ult25346:0.0,Ult25348:0.0,Ult25
349:0.0,Ult25328:0.0,Ult25327:0.0,Ult25329:0.0,Ult25330:0.0,Ult25323:0.0,
Ult25319:0.0,Ult25331:0.0,Ult25332:0.0,Ult25334:0.0,Ult25353:0.0):5.5E-
4)0.000.341:5.5E-4)0.608.7:5.5E-4)0.615.7:5.5E-
4,((Ult25339:0.00368,Ult25321:0.05253)0.149.4:0.00378,Ult25350:0.00744)0.
922.43:5.4E-4)0.586.6:5.5E-4)0.983.22:5.5E-
4)0.242.5:0.00363,(Ult25343:0.0,Ult25322:0.0,Ult25326:0.0,Ult25333:0.0):0
.00382)0.945.44:0.03527,((Ult25363:0.04983,Ult25364:0.10567)0.916.51:0.04
291,(Ult25355:0.05605,Ult25362:0.07414)0.934.41:0.03854)0.316.2:5.5E-
4)0.956.40:0.02729,((Ult25366:5.4E-
4,((Ult25360:0.01392,Ult25361:0.00523)0.923.60:0.01894,((Ult25357:5.5E-
4,(AluSterc:5.5E-4,GutMeta9:0.00754)0.996.35:0.03595)0.333.4:5.3E-
4,((Ult25356:0.00291,Ult25358:0.03703)0.666.9:0.01306,Ult25359:0.06579)0.
608.8:0.00734)0.884.47:0.01166)0.940.40:0.02044)0.924.38:0.01587,(Ult2536
7:0.00792,(Ult19754:5.4E-
4,Ult25365:0.00725)0.444.8:0.00773)0.780.38:0.00724)0.977.32:0.02752)0.65
8.4:5.4E-
4,Ult25354:0.01764)0.780.39:0.01379,((((Ult25370:0.09883,(Ult25389:0.0409
8,((Ult19748:0.0037,Ult25373:0.01882)0.044.4:5.4E-
4,(Ult25384:0.01115,(((Ult17749:0.06573,Ult19751:0.01168)0.938.35:0.01988
,(Ult25383:0.0,Ult25386:0.0):0.00361)0.993.29:5.3E-
4,(Ult24193:0.00371,((((Ult19739:0.09492,Ult25368:5.2E-

4)0.873.42:0.00727,Ult25369:0.00377)0.582.4:5.5E-
4,Ult25378:0.0037)0.449.8:5.5E-
4,(Ult19743:0.0,Ult19745:0.0,Ult25379:0.0):5.5E-4)0.337.4:5.5E-
4)0.693.8:5.4E-4)0.921.54:5.4E-4)0.867.47:0.00713)0.906.62:5.5E-
4)0.827.27:0.00689)0.743.32:0.00395,((Ult25380:0.0151,Ult25376:0.06026)0.
764.34:0.00374,(((Ult19737:0.00375,Ult25381:0.01134)0.890.51:5.5E-
4,(Ult19735:0.0,Ult19736:0.0,Ult19738:0.0,Ult19742:0.0,Ult19744:0.0,Ult19
749:0.0,Ult19750:0.0,Ult19753:0.0,Ult25371:0.0,Ult25372:0.0,Ult25374:0.0,
Ult25382:0.0,Ult25388:0.0):5.5E-4)0.000.342:5.5E-
4,((Ult25387:0.00375,Ult25377:0.00375)0.900.41:5.5E-4,Ult19747:5.5E-
4)0.000.343:5.5E-4)0.904.43:5.3E-4)0.892.39:5.4E-
4)0.693.9:0.0049,Ult25375:0.07129)0.853.33:0.02264,(Ult19733:0.10207,Ult1
9755:0.04515)0.874.64:0.03105)0.977.33:0.07489)0.933.46:0.05554)0.858.36:
0.027,((Ult25402:0.00415,(Ult25400:0.04407,((((((BacNL408:5.5E-
4,BacNL410:0.00762)0.847.44:0.00375,(((CsrInnoc:0.0,BacNL399:0.0,BacNL405
:0.0,Ult25403:0.0):5.5E-4,Ult25401:0.01133)0.717.11:5.5E-4,BacNL406:5.5E-
4)0.000.344:5.5E-4)0.504.3:5.5E-4,BacNL400:5.5E-4)0.611.3:5.5E-
4,(BacNL401:5.5E-4,BacNL409:5.5E-4)0.924.39:0.00371)0.084.3:5.5E-
4,(BacNL404:5.5E-4,((BacNL398:0.00366,(BacNL403:0.00369,(BacNL397:5.5E-
4,BacNL407:0.00371)0.659.7:5.5E-4)1.000.256:5.5E-
4)0.373.4:0.00366,(BacNL402:5.5E-4,BacNL412:5.5E-
4)0.904.44:0.00363)1.000.257:5.5E-4)0.072.2:5.3E-4)0.084.4:5.5E-
4,BacNL411:5.5E-4)1.000.258:5.4E-
4)0.763.39:0.00328)0.813.26:0.01346,((Ult25404:0.02975,(((Ult25399:0.0163
9,((Ult25396:0.0,Ult25397:0.0):0.00387,(UltFir89:0.09096,Ult25398:0.00448
)0.821.23:0.00696)0.940.41:0.02462)0.882.47:0.02173,(Ult25391:0.0308,((Ul
t25393:0.0,Ult25392:0.0):0.03442,((Ult25394:5.5E-
4,Ult25395:0.00375)0.789.24:0.00792,(Ult25390:0.03597,(Ult19752:5.5E-
4,EubCylin:0.0075)0.850.45:0.00901)0.787.29:0.01184)0.847.45:0.02443)0.88
2.48:0.02354)0.924.40:0.02177)0.805.28:0.01707,(Ult19740:5.5E-
4,EubCyli2:0.01519)0.775.30:0.00775)0.611.4:0.01212)0.788.29:0.01016,(Eub
Tortu:0.02474,(Ult19741:0.01398,EubDolic:0.00526)0.838.42:0.01348)0.839.2
9:0.01435)0.982.24:0.0418)0.915.36:0.02664)0.835.36:0.01992,(CsdBact6:0.0
,CsrSpe34:0.0):0.05261)0.797.28:0.02654)0.681.7:0.00526)0.896.30:0.01411)
0.880.49:0.01526,((GutMet11:0.04214,(((Ult25505:0.01245,(BacEnr44:0.01989
,Ult25502:0.03366)0.833.34:0.01205)0.809.31:0.01152,(UltRu524:0.06059,(((
(Ult25498:5.4E-
4,(((Ult25491:0.05906,Ult25496:0.00413)0.930.39:0.02201,((Ult25490:0.0362
6,Ult25495:0.01956)0.958.48:0.02445,(Ult25494:0.01642,(Ult25492:0.01942,U
lt25493:0.01653)0.698.12:0.00717)0.862.42:0.01181)0.858.37:0.01423)0.960.
40:0.02622,((UltRu525:0.02922,Ult25503:0.07854)0.861.49:0.02691,(Ult25500
:0.03638,((Ult25504:0.05353,Hmyy0025:0.09272)0.041.4:0.02551,Ult25501:0.0
4301)0.886.56:0.01833)0.839.30:0.01465)0.838.43:0.01782)0.842.40:0.01246)
0.753.34:0.00367,(Ult25499:0.00756,Ult21630:0.0233)0.775.31:0.00378)0.900
.42:0.03339,Ult25497:0.08558)0.899.46:0.03093,(Ult25484:0.2044,(Ult25486:
0.04823,(Ult25482:0.07963,(Ult25483:0.05282,Ult25485:0.02094)0.178.3:0.01
128)0.887.45:0.03067)0.608.9:0.03928)0.958.49:0.06021)0.691.7:0.02456)0.8
48.37:0.02175)0.972.35:0.05284,(((((((((Ult25456:5.5E-
4,(CpbCaten:0.01508,(Ult25472:0.02282,(Ult25469:0.03385,(Otu00429:0.02116
,(Ult25470:0.00762,Ult25471:0.00383)0.170.3:0.00281)0.780.40:0.00735)0.80
2.37:0.00696)0.973.22:0.02645)0.794.33:5.4E-
4)0.864.44:0.00757,Ult25455:0.00765)0.920.54:0.01788,((GutMet10:0.00899,(
UltB6402:0.11624,Ult25425:0.02244)0.964.48:0.03236)0.696.5:0.00205,(Ult25
473:5.5E-

4,Ult25474:0.01929)0.997.43:0.03208)0.805.29:0.00824)0.898.52:0.01354,(Ul
t25457:0.01142,(Ult25459:0.01154,(Ult25454:5.4E-
4,Ult25475:0.02333)0.910.57:0.00761)0.760.31:0.00375)0.784.32:0.00381)0.7
63.40:0.00374,(Ult25458:5.5E-4,Ult25460:0.00375)0.530.2:5.4E-
4)0.948.51:0.01851,(((((Ult25445:0.00863,Ult25442:0.08978)0.780.41:0.0116
,(Ult25450:0.01521,((Ult25443:0.0,Ult25446:0.0,Ult25448:0.0,Ult25452:0.0,
Ult25453:0.0):5.5E-4,(Ult25481:0.00753,((Ult25447:0.00252,(Ult25441:5.5E-
4,Ult25451:5.5E-
4)0.923.61:0.00248)0.000.345:0.00127,Ult25449:0.00248)0.923.62:0.00252)0.
315.7:5.4E-4)0.576.6:5.5E-4)0.879.45:5.3E-
4)0.967.31:0.01536,Ult25444:5.4E-
4)0.981.33:0.03415,Ult25476:0.03497)0.582.5:0.01238,((Ult25461:0.0,Ult254
62:0.0):0.03921,((((((CtnMitsu:0.01135,CtnMits2:0.02707)0.887.46:0.00755,
Ult25428:0.00752)0.704.8:5.5E-
4,((Ult25426:0.00375,Ult25418:0.00374)0.911.56:5.5E-
4,(Ult25427:0.0,Ult25429:0.0,Ult25431:0.0,Ult25419:0.0,Ult25413:0.0,Ult25
414:0.0,Ult25415:0.0,Ult25416:0.0,Ult25417:0.0,Ult25420:0.0):5.5E-
4)0.905.40:5.5E-4)0.990.39:5.5E-
4,Ult25430:0.05235)0.729.13:0.00619,((Ult25432:0.0499,Ult25439:0.06291)0.
763.41:0.01555,(LclCaten:0.0,UltLac91:0.0):0.07297)0.250.3:0.0157)0.688.8
:0.00287,(LclVitul:0.02224,(Ult25438:0.0145,((Ult25437:0.01119,(Ult25434:
5.5E-4,(Ult25435:0.03066,Ult25433:0.03062)0.875.49:5.3E-4)0.208.3:5.4E-
4)0.798.29:0.00348,Ult25436:0.00396)0.838.44:0.00843)0.930.40:0.0216)0.96
1.38:0.03184)0.758.37:0.00832)0.882.49:0.01593)0.871.45:0.01125)0.851.40:
0.01088,((Ult25480:0.06508,(Ult25479:0.12198,Ult25478:0.04438)0.884.48:0.
04274)0.987.40:0.07912,((((Ult25440:0.02055,Ult25465:0.01145)0.991.37:0.0
4311,(((BacNL418:5.5E-4,(BacNL414:5.5E-4,BacNL416:5.5E-4)0.420.8:5.5E-
4)0.309.3:5.5E-4,(((BacNL413:0.00749,BacNL419:5.5E-
4)0.854.31:0.00373,BacNL415:5.5E-4)0.322.3:5.5E-
4,(BacNL417:0.00373,(BacNL420:0.00374,Ult25463:0.00374)0.176.8:5.5E-
4)0.384.4:5.5E-4)0.004.3:5.5E-4)0.923.63:0.00744,CsrSacc2:5.5E-
4)0.765.21:0.00332)1.000.259:5.5E-
4,Ult25468:0.0076)0.776.33:0.00374,((CsrSpiro:0.00375,(Ult25464:5.5E-
4,(Ult25466:0.00752,CsrSpir2:0.00374)0.928.40:5.5E-4)0.917.43:5.5E-
4)0.955.34:0.02019,Ult25467:0.04936)0.717.12:0.00336)0.740.30:0.00537)0.7
95.35:0.00904)0.840.40:0.02204,Ult25477:0.05797)0.998.58:0.10181,Ult25506
:0.0573)0.768.23:0.0152)0.455.10:0.01818)0.936.45:0.04436,(Ult20617:0.126
98,Ult25489:0.02622)0.887.47:0.03385)0.927.45:0.02474)0.944.36:0.0242,(S0
114622:0.12078,((((((McmHaem2:0.09572,((Udntded4:0.03284,McmCocco:0.0067
6)0.369.6:0.00709,(CanMyco4:0.00898,McmHaemo:0.05235)0.973.23:0.0433)0.77
4.48:0.01312)0.947.39:0.04857,(McmSuis0:0.08904,((CanMyco2:0.02908,CanMyc
o3:0.0108)0.967.32:0.0424,((CanMycop:0.02687,McmOvis0:5.5E-
4)0.954.46:0.0191,(UltMyc16:5.4E-4,McmWenyo:0.01171)0.881.47:5.4E-
4)0.990.40:0.05897)0.732.11:0.00705)1.000.260:0.21447)0.938.36:0.05324,((
(((Ult25166:0.01993,(UltFir86:0.00753,Otu00062:5.5E-
4)0.737.31:0.0032)0.996.36:0.04787,((UltFir87:0.01292,(((((CbDBBBvB:5.5E-
4,Otu00136:5.5E-4)0.958.50:0.01139,(Sgffff14:5.4E-
4,UltMoll2:0.01117)0.873.43:0.00726)0.805.30:5.5E-
4,((UltMyc12:0.0,UltMyc13:0.0):0.01534,Otu00297:5.3E-
4)0.999.71:0.03942)0.749.42:0.0035,UltFir85:0.03177)0.881.48:0.00783,(MsH
tH005:0.02326,(UltMyc14:0.0,Otu00121:0.0):0.00749)0.746.27:0.00391)0.846.
37:0.01034)0.884.49:0.01222,Otu00638:0.01803)0.711.17:0.00614)0.877.58:0.
00858,(UltMoll3:0.03847,((UltMyc15:0.03705,ZnvYyy03:0.06757)0.901.53:0.01
991,(Ult25165:0.11042,(UltMyc11:0.19795,(((Otu00357:0.01205,(Ktyy0041:0.0

,Kty00024:0.0):0.06371)0.737.32:0.00332,(((Sgffff13:0.04074,((Ult25163:0.
0,Otu00433:0.0):0.0356,(((Ult25164:0.0344,Otu00405:0.11285)0.171.3:0.0097
4,(UltMyc10:0.01347,Sgfffff4:0.00964)0.925.50:0.02287)0.809.32:0.03196,((
Ult25162:0.0,Otu00286:0.0):0.01921,(UltMyco9:5.5E-
4,(Sgffff12:0.00374,UltMolli:0.01518)0.982.25:5.5E-4)0.913.41:5.5E-
4)0.925.51:0.02535)0.807.22:0.01748)0.876.37:0.02555)0.438.5:0.01565,ZnGr
G000:0.03891)0.939.42:0.03096,((ZneTTyYy:0.00598,UltMyco6:0.03061)0.896.3
1:0.01864,(UltMyco7:0.03139,(Ult25161:0.0,Otu00529:0.0):0.01601)0.564.4:0
.0049)0.712.12:0.00231)0.886.57:0.00857)0.791.21:0.01065,UltMyco8:0.07866
)0.829.35:0.01961)0.828.27:0.02045)0.929.48:0.03337)0.751.19:0.00877)0.85
7.51:0.01097)0.987.41:0.03755,((McmMicro:0.03278,(((UltMyco2:0.00706,UltM
yco3:0.01202)0.748.28:0.00487,((UltMyco4:0.0,UltMyco5:0.0):0.06073,(McmCa
vip:0.00293,(McmInson:0.03258,McmFasti:0.00312)0.782.43:0.01709)0.984.23:
0.04395)0.966.48:0.04067)0.606.5:0.01413,(((UrplCati:0.00313,(UrpCanig:0.
01643,(UrpFelin:5.5E-
4,(UrpUreal:0.00378,(UrpDiver:0.0074,UrpGallo:0.00781)0.967.33:0.02138)0.
889.41:0.00799)0.045.3:0.00794)0.667.9:0.00463)0.999.72:0.08369,((UltUrea
3:5.5E-4,(UltUrea4:0.00378,(UltUrea2:0.00752,UltUreap:5.5E-
4)0.761.36:0.00398)0.954.47:0.01593)0.992.45:0.04481,(Ult25160:0.00608,(U
ltUrea6:0.0247,UltUrea5:0.04219)0.841.32:0.01785)0.892.40:0.01977)0.775.3
2:0.01064)0.963.44:0.03566,UltMycop:0.08437)0.896.32:0.0211)0.699.14:0.01
795)0.615.8:0.01369,McmPenet:0.03506)0.910.58:0.01781)0.936.46:0.02317,((
McmAlvi0:5.5E-
4,McmPirum:0.00378)0.737.33:0.00168,(McmImita:0.04247,(McmAmpho:0.00767,M
cmTestu:0.00752)0.790.31:0.00634)0.502.4:0.00495)0.980.21:0.04178)0.831.2
8:0.01082)0.674.7:0.00308,McmPneum:0.0261)1.000.261:0.23185,(UltMyc21:0.0
5615,((CanHepa2:0.02098,UltMyc20:0.01082)0.897.36:0.03837,(UltEnto3:0.048
57,Ult25176:0.17305)0.421.5:0.02961)0.926.50:0.05243)0.987.42:0.07956)0.2
65.3:0.01591,((((SsmSyrph:0.01469,(SsmEndos:0.00779,SsmPenae:0.01128)0.98
6.31:0.03166)0.760.32:0.00526,((UltEart2:0.30764,(UltEnto4:0.11944,UltSp2
57:5.5E-
4)0.519.5:0.00297)0.746.28:0.00459,(UltSp255:0.00375,UltSp256:5.5E-
4)0.938.37:0.01148)0.762.42:0.00338)0.920.55:0.03888,(Ult25168:0.07519,Ul
t25167:0.0815)0.998.59:0.15471)0.868.34:0.02292,(SsmPlaty:0.04268,(UltSp2
59:0.02838,(UltEnto5:0.01157,(UltSp258:0.02318,Ult25177:0.00891)0.967.34:
0.02349)0.812.21:0.00724)0.747.26:0.00604)0.905.41:0.02673)0.362.5:0.0167
4)0.848.38:0.03225,(SsmLampy:0.06566,((((MsmLactu:0.0548,(UltMesop:0.0075
4,UltEnto2:5.5E-4)0.886.58:0.01207)0.966.49:0.0275,((EtmLuciv:5.4E-
4,EtmLumin:0.00755)0.863.42:0.00381,(AhlMulti:0.00508,(EtmFreun:0.00831,(
UltEntom:0.01258,EtmSomni:0.01869)0.299.5:0.00352)0.966.50:0.02264)0.928.
41:0.01468)0.902.54:5.4E-4)0.520.4:0.00715,(((MsmFloru:5.4E-
4,MsmCorru:0.01141)0.997.44:0.03779,(MsmPhotu:0.03134,MsmSeiff:0.00908)0.
469.9:0.00494)0.717.13:0.00245,(McmMycoi:0.00765,McmPutre:0.00765)0.774.4
9:0.00397)0.768.24:0.00405)0.984.24:0.03164,(SsmAlleg:0.03119,(SsmLepti:0
.03698,((SsmFlori:0.0155,(SsmCulic:0.0036,SsmLitor:0.04516)0.824.31:0.008
93)0.367.5:5.5E-
4,(SsmLineo:0.01392,SsmMonta:0.01217)0.262.4:0.01269)0.904.45:0.01569)0.8
63.43:5.3E-
4)0.965.45:0.02218)0.768.25:0.0086)0.946.44:0.02811)0.672.7:0.00782)0.954
.48:0.04136)0.648.9:0.00346,(((UltErys7:0.05738,(((((((Ult25529:0.05694,U
lt25528:0.01982)0.677.6:0.00765,(UltB7256:0.10438,Ult24727:0.04287)0.826.
27:0.01932)0.927.46:0.01998,(UltRu527:0.07187,((Ult25526:0.00922,Ult21362
:0.16587)0.813.27:0.01851,Ult25527:0.01592)0.679.11:5.3E-
4)0.171.4:0.00675)0.955.35:0.01958,Ult25523:0.00814)0.741.20:0.01171,(Ult

25530:0.00697,Ult25531:0.03267)0.955.36:0.02005)0.800.22:0.00892,((Bfhggg
79:0.0161,UltAna17:0.01609)0.794.34:0.00676,Ult25522:0.07144)0.941.42:5.5
E-4)0.905.42:0.0156,((((((Ult25518:5.5E-
4,Ult25517:0.00375)0.940.42:0.01146,(((UltB7220:0.01984,((Ult25519:0.0663
9,(Ult25514:0.02602,Ult25516:0.02397)0.731.14:0.0052)0.748.29:0.00658,Ult
25515:0.01717)0.922.44:0.01818)0.738.13:0.00413,(UltErys5:0.01176,GutMet1
2:0.01134)0.862.43:0.00753)0.892.41:0.0076,((((Ult25507:0.00374,(UltB4788
:0.09063,Ult25513:0.03042)0.763.42:0.00108)0.441.8:5.5E-
4,(Ult25509:0.0,Ult25511:0.0,Ult25510:0.0):5.5E-4)0.341.8:5.5E-
4,Ult25508:0.00374)0.420.9:5.5E-4,Ult25512:5.5E-4)0.903.43:5.5E-
4)0.908.46:0.01124)0.854.32:5.5E-
4,(SbrMoore:0.06672,BulExtru:0.02467)0.990.41:0.04689)0.918.52:0.02351,(U
lt25520:5.4E-
4,(UltRu526:0.01144,Ult25521:0.01147)0.856.33:0.00749)0.939.43:0.01989)0.
977.34:0.02976,Ult25541:0.03316)0.708.14:0.00231,((Ult25524:0.01449,Ult25
525:0.01341)0.996.37:0.05126,(HmnFilif:5.4E-
4,Ult25532:0.01516)0.808.27:0.02239)0.873.44:0.01936)0.836.29:0.01273)0.3
13.3:0.00748)0.958.51:0.02699,(((Ult25534:0.02628,(ErsInopi:0.00516,(ErsR
husi:5.5E-
4,Ult25533:0.06311)0.423.4:0.00616)0.613.8:0.0113)0.930.41:0.01764,((Ult2
5536:0.01955,(Ult25540:0.01572,(Ult25537:0.0116,Ult25538:0.02395)0.886.59
:0.01153)0.849.38:0.00769)0.908.47:0.01532,(Ult25539:0.11634,(UltErys6:0.
01575,Ult25542:0.10206)0.726.12:0.02051)0.818.19:0.01887)0.881.49:0.01217
)0.855.45:0.008,(Ult25535:0.07372,(Ult25543:0.06553,Ult25544:0.05419)0.97
8.26:0.05131)0.839.31:0.01789)0.758.38:0.00378)0.831.29:0.01034,(((Ult255
45:0.05983,(Ult21720:0.05521,Ult25546:0.10002)0.740.31:0.01401)0.806.18:0
.03159,(Ult25311:0.06681,((Hmyyy061:0.1163,Ult25313:0.09075)0.843.30:0.02
379,(((UltMoll9:0.01019,(((Ult21721:0.01988,Ult25310:0.02005)0.991.38:0.0
5581,(GutMeta8:0.06641,(((UltRu523:0.0257,(Ult25306:0.03712,AplAbact:0.03
394)0.913.42:0.02698)0.843.31:0.01163,Ult25305:0.07569)0.728.16:0.00431,(
(UltRu522:0.05936,(Ult19287:0.02312,(Ult25307:0.01133,Ult25308:5.5E-
4)0.868.35:5.3E-
4)0.835.37:0.01394)0.858.38:0.01534,(Ult25309:0.04703,UltAna16:0.05815)0.
890.52:0.02586)0.812.22:0.01302)0.882.50:0.01885)0.943.44:0.03292)0.956.4
1:0.04075,(Ult25315:0.0,Ult25421:0.0):0.03683)0.731.15:0.00121)0.401.7:0.
00836,(Ult25316:0.01546,(Bfhggg78:0.02252,(Ult25317:0.0272,Ult25318:0.037
33)0.727.12:0.00966)0.976.26:0.04188)0.818.20:0.00832)0.935.42:0.029,(Mcm
Felim:0.11603,((AhlBrass:0.00788,(((AhlParvu:0.01874,(UltMoll7:0.01743,(U
lt25314:5.4E-
4,(Ult25312:0.01624,BacEnr57:0.00381)0.993.30:0.03238)0.984.25:0.0346)0.8
29.36:0.01522)0.767.35:0.01879,(((AhlEquif:5.4E-
4,((UltB7988:0.04498,AhlHippi:0.01224)0.934.42:0.01664,(AhlSpeci:0.0156,(
(AhlGranu:5.5E-
4,(AhlLaidl:0.01866,AhlPleci:0.01001)0.926.51:0.01416)0.975.35:0.01579,(A
hlOcul2:0.00377,AhlOculi:0.0076)0.899.47:0.00745)0.130.2:5.5E-
4)0.598.3:0.01125)0.897.37:0.01469)0.939.44:0.02336,(AhlAxant:0.03357,Ahl
Cavig:0.04236)0.957.39:0.03356)0.860.31:0.0103,((((LetYello:0.02967,(((Ma
aPeriw:0.00412,(CanPhyt3:0.00363,(CanPhyt2:0.00372,CanPhyto:0.02333)1.000
.262:5.4E-
4)0.454.6:0.01112)0.984.26:0.02862,((CanPhyt5:0.06366,CanPhyt7:0.01507)0.
754.28:0.00586,LpdDymas:0.04339)0.746.29:0.00401)0.342.4:5.4E-
4,(CanPhyt4:0.00909,UdnMolli:0.01033)0.826.28:0.01171)0.905.43:0.02113)0.
866.41:0.02153,(CanPhyt6:0.03495,(XxxAtrip:0.02725,(PgnPea00:5.5E-
4,UdnMoll2:0.0113)0.958.52:5.4E-

4)0.728.17:0.00573)0.999.73:0.0775)0.747.27:0.01784,(CanPhyt8:0.0293,(Can
Phyt9:0.0536,((StwVires:5.5E-
4,(RusPotat:0.0116,(SugPhyto:0.01536,(AseYello:0.00374,(MexPotat:0.00757,
CayWhite:0.02341)0.865.48:5.5E-
4)0.749.43:0.00375)0.961.39:0.01565)0.785.37:0.00366)0.729.14:0.00466,Ptp
Speci:0.02261)0.527.5:0.00934)0.761.37:0.0069)0.851.41:0.0196)0.924.41:0.
02369,UltMoll8:0.03406)0.893.49:5.3E-
4)0.940.43:0.02343)0.904.46:0.01906,AhlPalma:0.02372)0.617.2:0.00759)0.65
1.9:5.5E-
4,(AhlMorum:0.01937,AhlVitul:0.03314)0.387.5:0.01162)0.582.6:0.00295)0.86
9.38:0.03132)0.542.2:0.00327)0.927.47:0.02471)0.967.35:0.03089)0.373.5:5.
4E-4,((UncUn202:5.5E-
4,Ult25410:0.00357)0.784.33:0.03463,(Ult25487:0.07512,Ult25488:0.07796)0.
998.60:0.19298)0.524.3:0.07652)0.932.51:0.03958)0.940.44:0.02164)0.879.46
:0.01416)0.951.42:0.0339,(UltFir90:0.10748,(HtrBacte:0.00126,((HbrChlor:5
.4E-
4,HbsMobil:0.00352)0.998.61:0.05073,(HrtDaure:0.05635,(HbrSulfi:0.00774,(
HbrModes:5.3E-
4,HbrGesti:0.01946)0.562.7:0.00364)0.833.35:0.01171)0.864.45:0.01595)0.92
2.45:0.03436)0.999.74:0.11266)0.723.18:0.02996)0.767.36:0.0179,((HplContr
:0.02424,(Ult25579:0.06336,Ult25580:0.07014)0.971.36:0.05238)0.897.38:0.0
1872,(((((CtlMarim:0.01154,((UltVibr6:0.00372,((Ult24801:0.0,Ult24802:0.0
):5.5E-4,EtcCecor:5.5E-4)0.803.28:5.5E-
4)0.944.37:0.00752,(EtcColu2:0.0,EtcColum:0.0):5.5E-
4)0.771.39:0.00374)0.809.33:0.0038,(((Ult24671:0.00415,Ult24676:0.01516)0
.902.55:0.01424,((((AkcPelag:0.0,MnlPsych:0.0):5.4E-
4,Ult24670:0.01528)0.813.28:0.00376,(Ult24663:5.5E-
4,((AkcIbur2:0.0,AkcIburi:0.0):0.00752,AkcPsych:5.3E-
4)0.973.24:0.01134)0.812.23:0.00375)0.812.24:0.0037,UltAlkal:5.4E-
4)0.756.43:0.00559)0.898.53:0.01117,(Ult24679:0.02348,(ApcTabac:5.5E-
4,Ult24677:5.5E-
4)0.789.25:0.00374)0.801.28:0.00627)0.865.49:0.0089)0.774.50:0.00366,(((E
tcSpec4:0.06834,(Ult24660:0.00375,AbiDefe2:0.00751)0.859.42:0.0102)0.765.
22:0.00512,((((((((UltVagoc:0.00384,VagFluvi:0.01537)0.789.26:0.00364,Swn
Fec12:5.4E-4)0.915.37:0.00752,TruFlocc:0.00749)0.404.4:5.3E-
4,(BclSpec2:0.0,TruPaste:0.0,TruPast2:0.0,TruColli:0.0):5.5E-
4)0.810.26:0.00376,(((Ult13130:0.0,AbiParaa:0.0,UltStr21:0.0):0.00746,Grl
Balae:5.4E-
4)0.775.33:0.00373,(AtoPhoca:0.00761,CbtBacte:0.00756)0.798.30:0.00375)0.
775.34:0.0037)0.894.34:0.00722,(Ult24904:0.00778,(((((VulModes:0.0193,((B
clAuran:0.02331,UltrdL12:0.00901)0.858.39:0.01495,((((BclHorti:5.5E-
4,BclHort2:5.5E-4)0.935.43:0.00747,BclSpe94:0.00371)0.172.6:5.4E-
4,(((BclClark:5.5E-4,BclSp138:0.00372)1.000.263:5.3E-
4,(BclVedde:0.00366,(((Bacte3E1:0.02298,BclSalip:5.5E-
4)0.949.45:0.0112,Ult25117:5.4E-
4)0.764.35:0.00372,(BclCellu:0.0,BclSp135:0.0,BclSp137:0.0):5.4E-
4)1.000.264:5.5E-
4)0.169.3:0.00374)0.776.34:0.0036,BclSp136:0.00755)0.806.19:0.00379)0.438
.6:0.00754,BclManna:5.4E-
4)0.877.59:0.01303)0.447.3:0.0119)0.729.15:0.00521,(((PloAntar:5.5E-
4,(((PnrPsych:0.00373,((Ult25002:5.5E-4,(PloPsych:5.5E-
4,(((((Ult24998:5.4E-4,((Ult24919:0.0,BclSpe51:0.0,Ult25057:0.0):5.5E-
4,UltPlano:0.00761)0.840.41:0.00364)0.136.2:0.00373,(((PnrOkean:0.0,PnrSp
ec2:0.0):5.5E-4,(PnrChine:5.5E-

4,(BclSeoha:0.00372,(BclSpe28:0.0,BclSpe29:0.0,BclSpe30:0.0):5.5E-
4)0.874.65:0.00374)0.000.346:5.5E-4)0.670.8:5.3E-
4,(PloPsyc2:0.0,PnrSpeci:0.0,PnrGlaci:0.0,Ult24979:0.0,PloSpeci:0.0):0.00
372)1.000.265:5.4E-
4)0.847.46:0.00365,((PloCitre:0.0,PloMarit:0.0,BclPoche:0.0):5.4E-
4,((Ult25036:5.5E-
4,(((((((((SsrAquim:0.0,SsrSpec2:0.0,SsrUreae:0.0,SsrSarom:0.0,Ult25080:
0.0):5.5E-4,(BvbSpeci:0.00372,(Ult24996:5.5E-
4,Ult24995:0.00372)0.985.38:0.01938)0.913.43:5.5E-4)0.752.32:5.5E-
4,Ult25000:0.00371)0.854.33:0.00365,(SsrLuteo:0.0,UltSporo:0.0):5.4E-
4)0.130.3:0.00375,(BclSpe43:5.5E-4,(Ult24993:0.00366,((SsrAntar:5.5E-
4,FbcLimic:0.00374)0.868.36:0.00372,((UltrS315:0.0,Ult25035:0.0,Ult25024:
0.0,UltrdL11:0.0,BclSpe46:0.0):5.5E-
4,(Ult24994:0.00378,Ult25025:0.00378)0.728.18:5.5E-4)0.000.347:5.5E-
4)0.700.18:5.5E-4)1.000.266:0.00314)1.000.267:5.3E-4)1.000.268:5.4E-
4,Ult25034:0.00372)0.144.3:5.5E-
4,(Ult25009:0.0,BclFucos:0.0,BclDrent:0.0,BclSpe41:0.0,BclSpe42:0.0,BclNo
val:0.0,BcllSoli:0.0,BclSpe44:0.0,BclPseud:0.0,BclFuma2:0.0,BclFumar:0.0,
BclSpe45:0.0,BclSene2:0.0,BclSeneg:0.0,BclSpe74:0.0):5.5E-4)0.449.9:5.5E-
4,(BclSpe18:0.0,Ult25001:0.0):0.00372)0.530.3:5.3E-
4,(BclArbut:0.03498,BclAestu:0.01117)0.889.42:0.00755)0.783.40:0.00372,(U
lt24976:0.0037,(((Ult24965:5.5E-
4,(BclSpe14:0.00373,Ult24964:0.00228)1.000.269:0.00143)0.781.36:0.00376,U
lt24949:0.00756)0.798.31:0.0037,(Ult24974:0.05602,(PnpQuisq:5.5E-
4,(BclInsol:0.00748,(BclSpe17:0.00371,(BclSpe16:0.01123,Ult24975:0.00749)
0.750.22:5.5E-4)0.742.19:5.4E-4)0.916.52:0.00749)0.880.50:5.3E-
4)0.842.41:0.00369)0.674.8:5.5E-
4)0.793.29:0.00368)0.798.32:0.00368)0.781.37:0.00368,((((((((LsnXylan:0.
02304,(LsnSpec2:0.0037,Ult24963:0.02304)0.889.43:0.01162)0.767.37:0.0037,
(((BclMass2:0.0,Ult24961:0.0):0.00752,(LsnSpha5:5.5E-4,(BclSpe10:5.5E-
4,(BclSpe13:5.5E-4,BclSpe11:5.5E-4)0.468.6:5.5E-4)0.273.2:5.5E-
4)0.380.4:5.5E-4)0.850.46:0.00411,RenSalmo:0.00748)0.159.2:5.5E-
4)0.000.348:5.3E-
4,((Ult25016:0.00368,(Ult24940:0.00374,Ult24956:0.00372)0.004.4:5.4E-
4)0.579.6:0.00749,((BclMassi:0.0,BclHorik:0.0,BclHori2:0.0):5.5E-
4,Ult24957:0.00373)0.919.54:5.4E-4)0.888.50:0.00743)0.398.6:5.5E-
4,Ult24950:0.00748)0.226.5:5.5E-4,Ult24948:0.00371)0.361.6:5.5E-
4,(BclSpec6:0.02283,BclSpec7:0.01126)0.783.41:0.00371)0.063.5:5.4E-
4,(BclOdyss:0.0,BclSpec8:0.0,BclSpe15:0.0,Ult24985:0.0,BclDecis:0.0):5.5E
-4)0.263.3:5.1E-4,LsnBoron:0.00746)0.582.7:5.5E-4,(LsnSpeci:5.5E-
4,PloColum:0.03867)0.768.26:0.00737)0.790.32:0.00404)0.780.42:0.004)0.293
.5:5.5E-4)0.866.42:0.00402,PnrAlkan:5.4E-
4)0.840.42:0.00399,PloStack:0.00746)0.149.5:5.5E-4)0.217.3:5.4E-
4)0.910.59:0.00385,(Ult24958:0.00766,Ult25088:0.02701)0.715.5:0.00751)1.0
00.270:5.4E-4)0.304.6:5.3E-
4,PloKocur:0.01526)0.840.43:0.00399,(Ult24978:0.02307,(((SsrSpeci:0.0,Ssr
Globi:0.0):5.5E-
4,PnpMacmu:0.00372)0.771.40:0.00367,Ult24999:0.02724)0.879.47:0.0078)0.76
6.31:0.00351)0.423.5:5.5E-4)0.451.9:5.2E-
4,AntBac39:0.00374)0.775.35:0.004,PloSpec2:5.5E-
4)0.853.34:0.00879)0.891.44:0.01531,(((((Ult25083:5.5E-4,(BclNiabe:5.5E-
4,Ult25056:0.00371)0.780.43:5.5E-4)0.888.51:5.4E-
4,(BclSpe70:0.01321,BclSpe71:0.01316)0.808.28:0.00782)0.845.40:0.00371,(B
acN159B:0.02283,((Ult25023:0.02292,(BclFasti:0.0038,Ult25059:0.01529)0.81

7.19:0.00726)0.735.18:5.5E-4,(((((BclCarbo:0.0,BclAcidi:0.0):5.5E-
4,(SblKushn:5.5E-4,SblAidin:0.00368)0.975.36:0.01507)0.895.57:5.5E-
4,SldEuzeb:0.00749)0.860.32:0.00742,((((((((OrhSpec2:5.4E-
4,((OrhBavar:0.0,OrhSpeci:0.0):0.00751,((((Ult25110:5.5E-
4,(((VrgDokdo:0.0,VrgSpec5:0.0):5.5E-4,(VrgProom:0.00374,(VrgPanto:5.4E-
4,(PacGlobu:0.01136,Ult25113:0.00753)0.790.33:0.00372)0.858.40:0.00374)0.
685.9:5.4E-4)0.834.30:0.00372,(VrgMaris:0.0,VrgSalar:0.0):5.5E-
4)0.868.37:0.00372)0.813.29:0.00372,(VrgHalod:0.0,VrgSpec6:0.0):0.00372)0
.726.13:5.5E-4,(VrgSedim:0.0,OrhCalif:0.0):5.5E-4)0.957.40:5.4E-
4,Ult25109:0.00746)0.332.4:0.00372)0.863.44:5.5E-
4)0.861.50:0.00374,(((BclNitri:5.5E-
4,BclPumi5:0.00752)0.820.17:0.00372,(((UltComp8:0.03592,((LntKapia:0.0112
6,LntSalic:5.5E-4)0.936.47:0.01458,(LntHalod:5.5E-
4,LntSalin:0.00368)0.784.34:0.00593)0.791.22:0.00609)0.841.33:0.00748,(Vr
gSpec2:0.01527,(VrgHalop:5.5E-4,(Ult25114:0.00371,CsbQuisq:5.5E-
4)0.982.26:0.01528)0.765.23:0.00358)0.883.52:0.00763)0.852.38:5.5E-
4,VrgSpec3:5.5E-4)0.858.41:5.3E-4)0.839.32:0.00371,VrgKeken:5.5E-
4)0.313.4:5.4E-4)0.808.29:0.0039,((Ult25115:5.5E-4,VrgSpec4:5.5E-
4)0.843.32:5.3E-4,(VrgArcti:5.4E-4,(VrgNecro:5.5E-
4,((OnbProf2:0.0,OnbProfu:0.0):0.00372,OnbSpec5:0.00372)0.807.23:0.00373)
0.878.43:0.00372)0.771.41:0.00372)0.873.45:0.0074)0.259.3:0.01145,(BclAlk
al:0.00748,UltrS316:0.00376)0.894.35:0.01113)0.995.49:5.3E-
4,(BclKribb:0.0,BclSpe40:0.0):0.00372)1.000.271:5.4E-
4,BclIsabe:0.00364)0.444.9:0.00371,((((((LstMonoc:5.5E-
4,(LstWelsh:0.0,LstSeeli:0.0,LstIvano:0.0):5.5E-
4)0.963.45:0.01637,(UltBroch:0.04787,BroCampe:5.4E-
4)0.975.37:0.02007)0.735.19:0.0027,(BclWakoe:0.0,BclSpe92:0.0,BclSpe93:0.
0):5.4E-
4)0.536.6:0.00372,(BclSpe68:0.0037,(Ult25076:0.0499,(BclSp125:0.01156,(((
ExiAntar:0.0,ExiOxido:0.0,ExiSibir:0.0,ExiArtem:0.0):5.5E-
4,ExiSpec4:5.5E-
4)0.816.26:0.00373,(ExiAcety:0.00319,(BacNL396:0.00361,(ExiSpec3:5.5E-
4,(ExiSpec2:5.5E-4,(Ult25085:0.00748,ExiSpeci:0.00369)0.591.8:5.5E-
4)0.545.9:5.5E-4)0.974.21:5.5E-4)0.997.45:0.02686)1.000.272:5.5E-
4)0.763.43:0.0035)0.832.29:0.00716)0.872.53:0.00805)0.864.46:0.00738)0.66
3.8:5.5E-4,(BclHemic:5.5E-4,BclHalod:0.00373)0.581.7:5.3E-
4)0.945.45:0.00733,(((BclPseu2:0.00373,Ult25071:0.01133)0.933.47:5.3E-
4,BclAkiba:5.5E-4)0.919.55:0.00372,BclSpe91:5.5E-4)0.911.57:5.4E-
4)0.844.57:0.00354)1.000.273:5.4E-
4,(BclAlka2:0.0,BclGinse:0.0):0.00374)0.921.55:0.00736,(BclCohni:5.5E-
4,Ult25058:5.5E-4)0.896.33:5.5E-4)0.989.39:5.4E-
4)0.791.23:0.00356,((BclRacem:0.01159,BclSp130:0.01917)0.908.48:0.01127,(
((((TsbSpeci:0.0075,(Ult24892:0.00372,(((((BclLich3:0.0,BclLich4:0.0,BclL
ich6:0.0,BclSp116:0.0,BclLic10:0.0,BclSp119:0.0):5.5E-4,((UltBaci9:5.5E-
4,((BclLich9:0.03234,(BclLich5:0.0039,BclSp115:0.00719)0.896.34:5.5E-
4)0.182.5:5.5E-4,BclLich8:0.01947)0.413.7:5.5E-4)0.000.349:5.5E-
4,BclLiche:5.5E-4)0.458.10:5.5E-4)0.000.350:5.5E-4,BclLich2:5.5E-
4)0.000.351:5.5E-4,BclSpe95:0.00748)0.000.352:5.5E-
4,(BclSp114:0.00754,(BclSub13:0.00362,((BclSp111:5.5E-
4,(BclSub10:0.00374,(((BclPumil:0.0,BclSafen:0.0,BclSp107:0.0,BclSp108:0.
0,BclSp109:0.0,BclPumi3:0.0,BclPumi4:0.0,Ult25077:0.0,BclSp110:0.0,BclSp1
17:0.0,BclSafe2:0.0):5.5E-4,((BclSp105:5.5E-
4,BclSp118:0.02344)0.088.3:5.4E-4,((BclPumi2:5.5E-
4,BclSp104:0.01571)0.820.18:0.00387,SrrMar10:0.00858)0.386.6:5.5E-

4)0.346.8:5.0E-4)0.918.53:0.00455,(((BclSubt8:5.5E-
4,((BclSub11:0.00371,((BclSp112:0.00372,BclMalac:5.5E-
4)0.848.39:0.00363,BclSub14:0.06742)1.000.274:5.5E-4)0.322.4:5.5E-
4,(BclSp103:0.0,BclSubti:0.0,BclSubt5:0.0,BclSubt7:0.0,BclAmylo:0.0,BclVe
lez:0.0,BclSp113:0.0,BclAtrop:0.0,BclSub15:0.0):5.5E-4)0.476.8:5.5E-
4)0.000.353:5.5E-4,BclSubt3:5.5E-4)0.000.354:5.5E-
4,(BclSubt2:0.01174,BclSp106:0.01573)0.880.51:5.3E-4)0.000.355:5.5E-
4)0.059.4:5.4E-4)0.134.7:5.5E-4)0.197.5:5.5E-
4,((BclSubt6:0.01891,BclSubt9:0.00744)0.846.38:0.002,BclSub12:0.01092)0.9
17.44:0.002)0.562.8:5.5E-
4)0.765.24:0.00443)0.896.35:0.00893)0.906.63:5.5E-4)0.308.5:5.5E-
4)0.553.4:5.5E-4,BclSub16:0.01479)0.898.54:5.5E-
4,(Ult25008:0.07329,BclLich7:0.01553)0.367.6:0.00801)0.834.31:0.00497,(Bc
lSpe65:0.0,BclSpe66:0.0,BclSpe67:0.0,BclSpe64:0.0,BclIdrie:0.0):5.4E-
4)0.586.7:5.4E-
4,Ult25101:0.02701)0.385.7:0.00526)0.868.38:0.01008)0.468.7:5.4E-
4)0.834.32:0.00494)0.531.2:5.4E-4)0.164.6:5.4E-
4,((((Ult25074:0.01144,(TubCalid:0.02373,((AbsTherm:5.5E-
4,BclSp140:0.00371)0.875.50:5.5E-4,(AbsTerra:0.0153,(BclAcido:5.4E-
4,(AbsAneur:5.5E-
4,AbsDanic:0.00372)0.925.52:0.00331)0.354.4:0.00751)0.855.46:0.01193)0.96
7.36:0.01945)0.762.43:0.00336)0.882.51:0.00761,PulNagan:0.00372)0.882.52:
0.00756,(Ult25118:0.0622,(((SdsAlbus:0.0,SdsHalop:0.0):0.00371,(FblMilos:
0.00716,(TnbMulti:0.01922,(PicSpeci:0.00754,AblElong:0.00748)0.902.56:0.0
077)0.931.40:5.5E-4)0.862.44:0.00721)0.882.53:0.0076,((TsbDevor:5.4E-
4,((VrgKoree:5.5E-
4,(BlcBact2:0.00765,((TblSacch:0.0,Ult25116:0.0):0.01136,TblSpeci:0.00372
)0.961.40:0.0155)0.878.44:0.00744)0.375.7:0.00372,TsbSpec2:0.00373)1.000.
275:5.4E-4)0.147.3:5.5E-4,((((HblDaban:5.5E-
4,HblSeoha:0.01522)0.403.6:5.5E-
4,((((SmcAlbum:0.00373,SsrUrea2:0.00747)0.798.33:0.00367,((Ult25105:0.007
48,HblSalin:0.00749)0.785.38:5.4E-4,(HblSpeci:0.0,HblFaeci:0.0):5.5E-
4)0.895.58:5.3E-4)1.000.276:5.4E-4,(HblSpec3:5.5E-4,HblSpec2:5.5E-
4)0.870.30:0.00365)0.164.7:0.00369,HblMangr:0.00366)1.000.277:5.4E-
4)0.941.43:0.00176,(HblTruep:0.03509,HblSpec4:0.00374)0.897.39:0.00215)0.
962.49:0.0148,(PotSpeci:0.00372,(PotChung:0.00353,(((AksFilif:0.0074,(Aks
Haloa:0.0075,AksSilvi:5.5E-4)0.914.46:5.4E-
4)0.250.4:0.00734,(AksHalop:0.01091,AksSpeci:5.5E-
4)0.117.2:0.00389)0.906.64:0.01146,PotMarin:5.5E-
4)0.884.50:0.00773)0.933.48:0.01163)0.799.35:0.00374)0.762.44:0.00369)0.7
90.34:0.00371)0.753.35:0.00336)0.841.34:0.00783)0.795.36:0.00374,((((((Bc
lFunic:0.0042,UltFir82:0.02665)0.410.6:0.00397,(BclBeiji:0.00756,Ult25084
:5.4E-4)0.771.42:0.00444)0.797.29:0.00294,(BclKore2:5.5E-4,BclKoree:5.5E-
4)1.000.278:5.3E-
4)0.864.47:0.00744,((((((UltFir83:0.00384,(UltFir81:0.01187,(BclInfer:0.0
1143,((Ult24992:0.00745,(((((StrMal14:0.00211,BclFirmu:0.007)0.896.36:0.0
0208,Ult24920:0.0114)0.937.44:5.5E-4,(BclSpe33:0.0,BclFirm2:0.0):5.5E-
4)0.527.6:5.5E-4,BactK283:5.5E-4)0.935.44:5.5E-4,Ult25012:5.5E-
4)0.914.47:5.3E-4)0.821.24:0.00747,(BclMass3:5.5E-
4,BclAzoto:0.00372)0.807.24:0.00366)0.995.50:5.5E-4)0.741.21:5.2E-
4)0.873.46:0.00791)0.793.30:0.00373,((((Ult25106:0.0,Ult25107:0.0):0.0112
6,BclSpe50:0.06844)0.869.39:5.5E-4,BclSubte:5.5E-4)0.385.8:5.2E-
4,(Ult25028:0.0,BclSpe47:0.0,BclSpe48:0.0,BclSpe49:0.0,BclSelen:0.0):5.5E
-4)0.960.41:5.4E-

4)0.713.13:0.00781,(BclSp126:0.00369,((BclSp127:0.0,BclEndo2:0.0):5.5E-
4,BclEndop:0.00781)0.804.27:5.5E-4)0.981.34:5.3E-
4)0.850.47:0.00376,(BclSiral:0.00373,((BclSpe39:0.00372,((BclPanac:5.5E-
4,Ult25065:0.00746)0.908.49:0.00746,(BclFlexu:5.4E-
4,(((Ult25022:0.0,BclSpe59:0.0,BclSpe57:0.0,Ult25053:0.0,BclFlex2:0.0,Bcl
Spe58:0.0,BclSpe60:0.0,BclMega2:0.0,BclSpe61:0.0,BclSpe62:0.0,UdnBac72:0.
0,BclSpe63:0.0,Ult25054:0.0):5.5E-
4,(BclMega3:0.00374,(BclMegat:0.00746,BclFlex3:0.02948)0.258.5:5.5E-
4)0.079.2:5.5E-4)0.251.3:5.5E-
4,BclSimp4:0.00746)0.907.50:0.00818)0.530.4:0.00826)0.745.32:5.4E-
4)0.078.4:5.5E-4,BclPalli:5.5E-4)0.474.8:5.5E-
4)0.935.45:0.00818)0.554.7:5.4E-4,(BclMetha:0.0,BclMeth2:0.0):5.4E-
4)0.203.3:5.5E-
4,(AnlSpeci:0.01908,(((AnlBogro:0.0,AnlFlavi:0.0,AnlRupie:0.0,GbcSpec6:0.
0,AnlSpec4:0.0):5.4E-
4,((((GbcSpec5:0.00373,(BclSpe88:0.03794,(GbcTher3:0.0,GbcTher4:0.0):5.5E
-4)0.863.45:0.00452)0.435.6:5.5E-4,(GbcSpec4:0.0,GbcCaldo:0.0):5.5E-
4)0.509.5:5.5E-4,(ShsTherm:5.4E-4,((GbcSpec2:5.5E-
4,((GbcTherm:0.0,BclCaldo:0.0):0.00204,GbcToebi:5.5E-
4)1.000.279:0.00169)0.872.54:5.5E-
4,((GbcSpeci:0.00735,GbcStear:0.00389)0.899.48:0.01121,(GbcSpec3:5.5E-
4,GbcTher2:5.5E-4)0.893.50:0.00735)0.926.52:5.3E-
4)0.880.52:0.00736)0.813.30:0.00363)0.553.5:5.4E-4,BclSpe89:5.5E-
4)0.963.46:0.01229)0.227.6:5.5E-4,(AnlSpec2:0.0515,AnlAyder:5.4E-
4)0.802.38:0.00362)0.202.5:5.5E-4)0.820.19:0.00393)0.939.45:5.4E-
4)0.816.27:0.00405,((Ult24997:0.02637,BclAmino:0.00709)0.941.44:0.01751,B
clSpe72:5.5E-
4)0.906.65:0.00761)0.800.23:0.00421,((BclAeoli:0.01508,(GbcCald2:5.5E-
4,(GbcSpec7:5.5E-4,(BclSmith:5.5E-
4,(((((((AnlSpec3:0.0,AnlAmylo:0.0,UltSacch:0.0):5.5E-
4,GbcTepid:0.00372)0.841.35:0.00372,BclForam:0.00371)0.000.356:5.5E-
4,UltComp5:0.00371)0.858.42:0.00368,(Ult25032:5.5E-
4,(BclSpe86:0.00746,(BclSpe85:0.0,Ult25066:0.0):5.5E-4)0.319.6:5.5E-
4)1.000.280:5.4E-4)0.334.4:0.00755,BcllHumi:5.3E-
4)0.899.49:0.00748,(BclCanav:0.00374,(((Ult25078:0.00749,((BclForti:0.0,B
clFarra:0.0):5.5E-4,(Ult25079:0.0075,BclSp121:5.5E-
4)0.854.34:0.00371)0.713.14:5.5E-4)0.903.44:0.00746,(BclSporo:5.5E-
4,(BclSp120:0.00748,BclLentu:0.00372)0.709.9:5.5E-4)1.000.281:5.5E-
4)0.655.13:0.00747,(BclSp124:0.01147,((BclBadiu:0.0,BclSp123:0.0):5.5E-
4,(BclFordi:0.0228,BclSp122:5.5E-4)0.818.21:0.00371)0.861.51:5.4E-
4)0.883.53:0.00746)0.890.53:5.5E-4)0.853.35:0.00372)0.748.30:5.4E-
4)1.000.282:0.00319)1.000.283:5.1E-4)0.840.44:5.4E-
4)0.894.36:0.00746,(BclTaean:0.00373,(BclSp100:0.0114,(BclSp101:0.0,BclSp
102:0.0):0.00751)0.781.38:0.00381)0.961.41:5.3E-
4)0.475.8:0.00753)0.698.13:5.4E-
4)0.839.33:0.00405)0.906.66:0.0096,((BclSp139:0.02715,Ult25120:0.02257)0.
846.39:0.00905,(((((BclGelat:5.5E-
4,(BclSolis:0.00747,BclSpe96:0.01127)0.940.45:5.5E-4)0.983.23:5.4E-
4,((BclMacau:5.5E-4,BclSpe98:5.5E-4)1.000.284:0.00283,(BclSpe97:5.4E-
4,(LowGC000:5.5E-4,(BclArse3:0.0,BclBarba:0.0):5.5E-
4)0.811.28:0.00722)0.898.55:0.01151)0.784.35:0.00412)0.947.40:0.01157,(((
BclLongi:0.0,Ult25018:0.0,Ult25021:0.0,BclSpe35:0.0):5.5E-
4,Ult25017:0.00371)0.890.54:5.4E-
4,(Ult25020:0.0075,((((BclSpe54:0.00746,((Ult25027:0.0,Ult25051:0.0):0.00

372,(Ult25050:0.0,BclSpe55:0.0):5.5E-
4)0.790.35:0.00372)0.806.20:0.00372,((Ult25003:0.0,Ult25004:0.0,Ult25007:
0.0,BclCircu:0.0,Ult25011:0.0,BacL1942:0.0,BclCirc2:0.0,BclSpe31:0.0,BclS
pe32:0.0):5.5E-
4,(Ult25010:0.00371,(Ult25005:0.02284,Ult24835:0.00371)0.537.8:5.5E-
4)0.155.6:5.5E-4)0.746.30:5.5E-4)0.774.51:5.5E-
4,((Ult25006:0.03989,Ult25031:0.00345)0.755.31:0.00397,(((BclBenzo:0.0036
5,(Ult25082:0.00365,BclSp128:5.3E-4)0.821.25:0.00753)1.000.285:5.4E-
4,Ult25095:5.5E-4)0.923.64:5.5E-
4,BclSpe34:0.00373)0.931.41:0.00759)0.779.36:5.5E-4)0.575.2:5.4E-
4,(((Ult25030:0.00371,Ult25014:0.02264)0.358.6:5.5E-
4,(Ult25013:0.0,UltrS314:0.0,Ult25093:0.0):5.5E-4)0.359.3:5.5E-
4,(Ult25015:0.0,OphioYy9:0.0):0.00374)0.866.43:0.0037)0.810.27:0.00366)0.
779.37:0.00366)0.894.37:0.00761)0.790.36:0.00348,Ult25086:0.0125)0.769.33
:0.00375,(Ult25019:5.5E-
4,(BclKore3:0.0,BclKorle:0.0,BclSp129:0.0):0.01943)0.893.51:0.00774)0.907
.51:0.0081)0.904.47:5.4E-
4)0.777.37:0.00431)0.736.21:0.00452,((BclSpe99:0.00371,BclDecol:0.00753)0
.952.46:0.01592,BlcBact4:0.01545)0.885.39:0.01163)0.416.10:0.01305,((BclC
ytot:5.3E-4,((((((((Ult24876:0.00372,(Ult24879:5.3E-
4,Ult24881:0.00746)0.839.34:0.00371)0.683.13:5.5E-
4,(Ult24884:0.00747,Ult24882:0.02279)0.769.34:0.00371)0.872.55:0.00371,((
((((Ult24868:0.00227,Ult24873:0.06474)1.000.286:0.00152,((Ult24867:0.0,U
lt24869:0.0,Ult24870:0.0,Ult24874:0.0,Ult24875:0.0,Ult24878:0.0,Ult24880:
0.0,Ult24887:0.0,Ult24888:0.0,StsFleur:0.0,Ult24889:0.0,Ult24905:0.0,Ult2
4906:0.0):5.5E-
4,((Ult24872:0.01516,(Ult24897:0.00365,(SasHalod:0.00749,(((Ult24911:0.01
551,MauBrune:0.00731)0.886.60:5.2E-4,(Ult24898:5.4E-4,((SasSpeci:5.5E-
4,(SasIrane:0.01497,((((((Ult24902:0.01493,(JeoPinni:0.00852,(NscAmpul:0.
02266,Ult24901:0.00621)0.803.29:0.01033)0.860.33:0.01222)0.898.56:0.01254
,(Ult24899:0.00376,JeoPsych:0.00373)0.800.24:0.00443)1.000.287:5.7E-
4,MirLuteu:0.00284)0.994.42:0.03186,((MauCaseo:0.0,MauLamae:0.0):5.4E-
4,Ult24896:0.00373)0.737.34:0.00381)0.827.28:0.00423,SasHispa:5.4E-
4)0.902.57:0.01291,SasLuteu:0.04251)0.734.22:0.00266)0.449.10:5.4E-
4)0.899.50:0.00785,SasJeotg:5.4E-
4)0.968.34:0.01199)0.838.45:0.0039)0.452.8:5.5E-
4,(SasAlkal:0.0,SasKunmi:0.0):5.5E-4)0.374.6:5.4E-4)0.485.7:5.5E-
4)0.924.42:0.00769)0.655.14:5.5E-4,((Ult24871:0.0,Ult24885:0.0):5.4E-
4,Ult24890:0.044)0.846.40:0.00371)0.456.4:5.5E-4)0.000.357:5.5E-
4)0.467.4:5.5E-4,Ult24877:0.00371)0.200.3:5.5E-
4,Ult24886:0.00372)0.445.9:5.5E-4,Ult24903:0.00372)0.466.8:5.5E-
4,Ult24866:0.00371)0.000.358:5.5E-4,Ult24883:0.00371)0.332.5:5.5E-
4)0.925.53:0.00769,Ult24859:0.01138)0.893.52:5.4E-
4,(((((((StsSpec4:5.5E-
4,(((((StsSpec3:0.0,StsSpec5:0.0,StsSpec6:0.0,StsSpec7:0.0):0.00372,BacNL
388:0.01149)0.000.359:5.5E-4,(StsEquo3:5.5E-
4,(UltStap9:0.0049,StsEquo2:5.5E-4)0.000.360:5.4E-
4)0.923.65:0.00493)0.154.2:5.5E-4,StsPette:0.0075)0.000.361:5.5E-
4,Ult24852:0.00745)0.365.4:5.5E-4)0.000.362:5.5E-4,StsNepal:5.5E-
4)0.363.8:5.5E-4,StsSapro:5.5E-4)0.000.363:5.5E-
4,(Ult24853:0.01881,Ult24854:0.01497)0.687.13:5.5E-4)0.000.364:5.4E-
4,UltStap5:0.00746)0.336.5:5.5E-4,StsCohni:0.00738)0.909.35:5.5E-
4,((UltStap3:0.00372,(((Ult24849:0.01088,Ult24865:0.03005)0.986.32:5.4E-
4,((UltStap8:0.00701,(Ult24855:0.00368,StsAuric:5.5E-

4)0.921.56:0.00206)0.923.66:0.00209,(Ult24850:5.5E-
4,((((((Ult24848:0.00371,Ult24861:0.00371)0.573.6:5.5E-
4,(Ult24863:0.00371,Ult24851:5.5E-4)0.820.20:5.5E-4)0.000.365:5.5E-
4,(BacTe13A:0.0,UltStap4:0.0,StsCapra:0.0,BacNL386:0.0,StsAure2:0.0,StsAu
reu:0.0,StsEquor:0.0,SwnFec13:0.0,StsWarne:0.0,StsWarn2:0.0,StsHomin:0.0,
Ult24858:0.0,BacNL389:0.0,BacNL392:0.0,BacNL393:0.0,Ult24893:0.0,Ult24910
:0.0):5.5E-4)0.000.366:5.5E-
4,(Ult24857:0.00372,(BacNL390:0.00371,Ult24846:0.01507)0.000.367:5.5E-
4)0.513.3:5.5E-4)0.716.9:5.5E-4,StsSpec2:0.00371)0.000.368:5.5E-
4,(((Ult24891:0.02664,UltSta11:5.5E-4)0.826.29:5.5E-
4,(Ult24864:0.01536,SpcSpe13:0.01142)0.773.36:0.00357)0.741.22:5.5E-
4,(Ult24862:0.03477,Ult24909:5.5E-4)0.861.52:5.4E-4)0.716.10:5.4E-
4)0.713.15:5.5E-4)0.738.14:5.5E-4)0.146.4:5.5E-4)0.861.53:5.5E-
4,((((BacNL385:5.4E-
4,BacNL391:0.00375)0.862.45:0.0037,(BacteN47:0.00371,((UltStap7:0.00386,(
(StsCarno:0.0,StsPisci:0.0,StsSimul:0.0):5.5E-
4,UltStap6:0.00372)1.000.288:0.00172)1.000.289:0.00193,((StsLutra:0.0,Sts
Schle:0.0,StsMicro:0.0):0.00365,StsChrom:5.3E-
4)0.387.6:0.00372)1.000.290:5.3E-4)0.312.8:5.5E-4)0.060.3:5.5E-
4,BacNL387:0.00368)0.757.27:5.5E-
4,(Ult24845:0.00732,(Ult24847:0.00369,Ult24860:5.5E-4)0.974.22:5.5E-
4)0.835.38:0.00365)0.543.4:5.5E-4)0.149.6:5.5E-
4)0.801.29:0.00356,UltStap2:0.01125)0.798.34:0.00352)0.776.35:0.00339,Ult
24856:0.00397)0.355.7:0.00362)0.925.54:0.01443,(Ult24908:0.04711,(StsSpec
i:0.05179,BclThur4:0.20789)0.922.46:0.04241)0.759.36:5.4E-
4)0.783.42:0.00531,((StsInter:0.09192,BclSubt4:0.25964)0.930.42:0.05696,U
lt24917:0.01553)0.897.40:0.02382)0.873.47:0.00768,(((UltSta10:0.00751,(Ul
t25060:0.01512,((BclSpe87:0.00752,(BacPFCr1:0.01138,(RmmPycn2:0.0,RmmPycn
u:0.0):0.00372)0.927.48:0.01138)0.759.37:0.00374,(((BclSpe12:0.00376,((Ul
t25033:0.0,BclLucif:0.0):5.5E-
4,BclSpe84:0.00746)0.773.37:0.00368)0.888.52:0.00745,((((((KurSpec2:0.003
72,KurSibir:0.00376)0.827.29:0.00376,(((Ult24927:0.0,Ult24990:0.0):5.5E-
4,(Ult24929:0.00367,(Ult24960:5.4E-
4,(((Ult24928:0.0,CrhTenue:0.0,Ult24972:0.0):5.5E-
4,(Ult24926:0.007,(CrhLatum:0.0,CrhLatu2:0.0):0.00211)0.886.61:0.00209)0.
877.60:0.00374,(Ult24925:0.00372,(Ult24932:5.5E-
4,((Ult24939:0.00372,Ult24987:0.00371)0.698.14:5.5E-
4,(BclSpec9:0.0,Ult24924:0.0):5.5E-4)0.000.369:5.5E-4)0.902.58:5.5E-
4)0.334.5:5.5E-4)0.846.41:0.00367)0.081.5:0.00375)1.000.291:5.3E-
4)0.746.31:5.5E-4,(Ult24943:0.00371,Ult24942:0.01121)0.268.2:5.5E-
4)0.855.47:5.4E-
4)0.763.44:0.00378,Ult25037:0.01152)0.772.39:0.00368,(Ult24941:5.5E-
4,Ult24944:0.01126)0.590.3:5.4E-
4)0.920.56:0.00747,BclPichi:0.00371)0.901.54:5.3E-4,BclSpe27:5.5E-
4)0.960.42:5.4E-
4)0.405.9:0.00743,((((((LsnSphae:0.0,LsnFusif:0.0,LsnSpha4:0.0):5.5E-
4,((Ult25092:0.00372,BclSpec5:0.00746)0.793.31:5.5E-
4,(Ult24921:0.00371,BclSpec4:0.02672)0.917.45:5.5E-4)0.000.370:5.5E-
4)0.736.22:5.5E-4,(LsnSpha3:0.00384,LsnSpha2:5.5E-4)0.725.14:5.4E-
4)0.960.43:5.5E-4,(BclCecem:5.5E-4,((LsnParvi:5.5E-
4,(Ult25042:0.01568,(Ult24922:0.00739,(((Ult24935:0.0,Ult24930:0.0,BclMac
ro:0.0):5.5E-4,Ult24923:0.00371)0.000.371:5.5E-
4,(Ult24945:0.00371,Ult20234:0.0151)0.737.35:5.5E-4)0.903.45:5.5E-
4)0.586.8:5.4E-4)0.852.39:0.00369)0.228.4:5.4E-

4,AceEstu2:0.00747)0.835.39:0.00367)0.845.41:0.00368)0.888.53:0.002,((Ult
24933:5.5E-4,Ult24931:0.00372)0.483.7:5.5E-
4,(((RmmStabe:0.0272,((KurSpeci:0.0,KurGibso:0.0):5.5E-4,Ult24966:5.5E-
4)0.999.75:5.5E-4)0.839.35:0.00369,Ult24970:5.5E-
4)0.852.40:0.00372,(Ult25061:0.00371,(((Ult24969:0.00372,Ult24967:0.00372
)0.701.11:5.5E-4,(Ult24977:0.00746,Ult24951:0.00747)0.673.3:5.5E-
4)0.000.372:5.5E-4,(Ult24968:0.0,Ult24971:0.0):5.5E-4)0.740.32:5.5E-
4)0.552.5:5.5E-
4)0.890.55:0.0037)0.886.62:0.00215)0.920.57:0.00682,((((BclLitor:5.5E-
4,Ult25055:0.00369)0.879.48:0.00368,((BclSpe56:0.0,BclHalma:0.0):5.5E-
4,((((BclChand:5.5E-
4,Ult25048:0.00373)0.779.38:0.00377,(Ult25049:0.00372,(((Ult25045:0.0,Ult
25047:0.0,Ult25094:0.0):0.00371,(Ult25046:0.00372,(Ult25044:0.0,Ult25068:
0.0):5.5E-4)0.998.62:5.4E-
4)0.935.46:0.0115,((MnbMari2:0.0,MnbMarin:0.0):5.5E-
4,(JtgAlime:0.0,JtgSpeci:0.0):0.0075)0.939.46:0.0115)0.759.38:0.00362)0.7
85.39:0.00378)0.808.30:0.00369,(UltrS313:0.0,BclPsych:0.0):5.4E-
4)0.881.50:0.00744,(((BclSpe36:5.0E-
4,BclSpe37:0.00374)0.924.43:0.00748,((BclSpe52:0.00372,((Ult25052:0.00372
,(Ult24938:0.00372,Ult25043:0.01223)0.113.6:5.5E-4)0.367.7:5.5E-
4,(BclCoahu:0.0,BclCoah2:0.0,Ult25038:0.0,BclSimp2:0.0,BvcFrigo:0.0,BvcSp
eci:0.0,Ult25091:0.0):5.5E-4)0.449.11:5.5E-4)0.544.3:5.4E-
4,((BclSpe69:0.00366,(BclSpe38:0.00749,BclSpe53:5.5E-4)1.000.292:5.1E-
4)0.416.11:0.00372,((Ult24934:0.0,BclSimp3:0.0,Ult25039:0.0,BclSpe73:0.0)
:5.5E-4,BclSimpl:5.5E-4)0.858.43:0.00361)0.874.66:5.4E-4)0.103.4:5.5E-
4)0.837.26:0.00389,((BclSpe19:5.5E-
4,((Ult25040:0.0,Ult25041:0.0):0.00372,BclSpe20:5.5E-
4)1.000.293:0.00251)1.000.294:0.00113,((((BclAquim:0.0,BclSpe25:0.0):5.5E
-4,(BclSpe22:0.00372,(BclSpe24:5.5E-
4,(BclSpe21:0.01041,BacWMB23:0.03344)0.760.33:0.00559)0.833.36:0.00429)0.
420.10:5.5E-4)0.000.373:5.4E-4,BclSpe23:0.00747)0.295.9:5.5E-
4,BclSpe26:5.5E-4)1.000.295:5.4E-4)0.594.5:0.00425)0.968.35:5.4E-
4)0.509.6:5.5E-4)0.554.8:5.5E-
4)0.918.54:0.00927,(VrdNeide:0.01905,(Ult21404:0.00371,((Ult24953:0.00745
,((Ult24936:0.0,Ult24946:0.0,Ult24959:0.0,Ult24952:0.0,Ult24954:0.0):5.5E
-4,VrdAreno:5.5E-4)0.000.374:5.5E-4)0.442.7:5.5E-
4,(Ult25090:0.00752,Ult24955:0.00373)0.476.9:5.5E-4)0.555.5:5.5E-
4)0.864.48:5.5E-4)0.675.5:0.00376)0.533.4:5.3E-
4,Ult24947:0.00371)0.573.7:5.5E-4)0.911.58:5.4E-
4)0.815.26:0.00408)0.871.46:0.00834)0.770.52:0.00413)0.156.3:5.5E-
4,((Ult25572:0.02774,(((Ult25571:5.5E-
4,(Ult25568:0.0071,Ult25569:0.02809)0.991.39:0.03205)0.928.42:0.01499,(Ul
tB4745:0.01882,(((Ult25560:0.05967,((Ult25558:0.04628,(((Ult21377:0.0,Ult
25563:0.0,Ult25564:0.0,Ult25561:0.0):5.5E-4,Ult25577:5.5E-
4)0.889.44:5.5E-
4,(Ult25562:0.01123,(Ult24728:0.0,TcbSangu:0.0):0.00373)0.267.4:5.4E-
4)0.818.22:0.00369)0.976.27:5.4E-4,(Ult25559:5.5E-
4,Ult25576:0.00371)0.963.47:5.3E-4)0.961.42:5.5E-
4)0.897.41:0.00725,(Ult24208:5.5E-4,Ult25570:0.00741)0.960.44:5.4E-
4)0.587.6:0.00728,((Ult25565:0.00366,Ult25566:0.06391)0.765.25:0.0037,(Ul
t25575:5.5E-4,(Ult25567:5.5E-
4,(Ult25573:0.01949,Ult25574:0.03556)0.977.35:5.4E-4)0.424.7:5.5E-
4)0.508.4:5.4E-4)1.000.296:5.3E-4)0.829.37:0.00376)0.804.28:5.5E-
4)0.717.14:0.00184,(Ult24683:0.00629,Ult24980:0.04757)0.998.63:0.06013)0.

980.22:0.00318)0.989.40:0.0379,((((BclCere3:0.02296,Ult25062:0.00761)0.75
8.39:0.00365,((((((BclSpec3:0.0,BclSpe77:0.0,BclSpe76:0.0,BacNL394:0.0,Bc
lCereu:0.0,BclSpe75:0.0,BclCere2:0.0,BclThuri:0.0,BclCere5:0.0,BclCere6:0
.0,BclCere7:0.0,BclSpe79:0.0,BclAnthr:0.0,BclThur3:0.0):5.5E-
4,(BacNL395:0.00556,(BacWMB25:0.03741,(BclThur2:5.5E-
4,BacTLCL1:0.00373)1.000.297:0.00179)0.983.24:0.00202)0.468.8:5.5E-
4)0.151.4:5.5E-4,BclSpe82:0.00371)0.000.375:5.5E-
4,(BclSpe81:0.02254,BclCere4:0.00219)0.912.57:0.0018)0.368.2:5.5E-
4,Ult25063:0.00371)0.046.4:5.5E-4,BclSpe83:0.00746)0.387.7:5.5E-
4)0.059.5:5.5E-4,BclSpe78:0.00744)0.166.4:5.4E-4,BclSpe80:5.5E-
4)0.806.21:5.4E-
4)0.788.30:0.00414)0.655.15:0.00823,Ult25064:0.02295)0.805.31:5.4E-
4)0.483.8:0.00352)0.894.38:0.00711,Ult25087:0.00375)0.468.9:5.3E-
4)0.586.9:0.01088)0.828.28:0.01106)0.902.59:5.4E-
4,(((Ult24680:0.01525,(UltEnt24:5.4E-4,((((Ult24798:5.5E-
4,((Ult24795:5.5E-
4,UltFir79:0.00749)0.821.26:0.00373,Ult24797:0.00749)0.008.1:5.4E-
4)0.852.41:0.00372,(Ult24794:0.01125,Ult24796:0.00747)0.078.5:5.5E-
4)0.895.59:0.01117,(Otu01751:0.01939,((Ult24662:0.00734,(GrlSpeci:0.00746
,GrlElega:5.5E-4)0.833.37:5.5E-
4)0.853.36:0.00978,(TtrSoli2:0.00378,((TtrHalo2:0.0,TtrHalop:0.0):0.00749
,(TtrSolit:5.5E-4,TtrKoree:5.5E-4)0.591.9:5.5E-4)0.025.1:5.5E-
4)0.911.59:0.01434)0.020.3:0.00545)0.425.4:5.5E-
4)0.889.45:0.00794,((Ult24804:0.00378,(UltEnt23:5.4E-
4,((EtcCamel:0.00373,(EtcItali:0.00377,EtcItal2:5.5E-4)0.286.3:5.5E-
4)0.810.28:0.00371,(((((EtcSulfu:0.00375,Ult24838:0.00371)0.627.3:5.5E-
4,(LcgNapht:0.00372,(((CnmPleis:5.5E-4,((CnmMobil:0.0,CnmSpeci:0.0):5.4E-
4,CnmFundi:0.01521)0.941.45:0.0113)0.914.48:0.00751,((UltCarno:0.01126,Ul
t24984:0.00371)0.460.3:5.5E-
4,(CnmVirid:0.0,Ult24672:0.0,UltCarn2:0.0,CnmMalta:0.0,CnmGalli:0.0):5.5E
-4)0.999.76:5.3E-
4)0.909.36:0.00747,(Ult24673:0.01436,((DszIncer:0.0,DszInce2:0.0):5.5E-
4,Ult24674:0.01907)0.740.33:0.00518)0.962.50:0.01848)0.630.8:5.5E-
4)0.000.376:5.4E-4)0.607.4:5.5E-
4,(Ult24675:0.0,Ult24774:0.0,VagFessu:0.0):5.5E-4)0.535.5:5.5E-
4,CnmDiver:0.00371)0.929.49:5.4E-
4,(Ult24841:0.0,VagSalmo:0.0):0.01133)0.404.5:0.00375)0.989.41:5.4E-
4)0.786.42:0.00378)0.791.24:0.00384,(MccPlut2:0.0,MccPluto:0.0):0.00381)0
.774.52:0.00401)0.787.30:0.00376)0.925.55:0.01052)0.788.31:5.5E-
4,(((EtcDevri:5.5E-
4,((EtcPalle:0.0,Ult24827:0.0,EtcMalod:0.0,EtcRaffi:0.0,UltLac90:0.0,EtcV
iikk:0.0):5.5E-4,((BacNL383:0.00738,((((BacNL381:5.5E-
4,((Ult24833:0.00696,BacNL384:0.00208)0.852.42:0.00205,((EtcMund3:5.5E-
4,(Ult24831:0.01897,(((Ult24799:0.01898,UltEnt29:5.5E-
4)0.834.33:0.00371,(BacNL382:0.00371,((((EtcFaeci:0.00509,((EtcMund4:0.00
371,(((((((Ult24634:0.0,EtcCanin:0.0,EtcDispa:0.0,Ult24820:0.0,EtcVillo:
0.0,EtcHirae:0.0,EtcDura6:0.0,EtcDura7:0.0,EtcDura8:0.0,EtcDura9:0.0,EtcF
aec5:0.0,EtcFaec2:0.0,EtcFaec4:0.0,EtcFaec6:0.0,EtcMundt:0.0,EtcFaec8:0.0
,EtcDur10:0.0,EtcDur11:0.0,EtcDur12:0.0,EtcDur13:0.0,EtcMund5:0.0,EtcFae1
0:0.0,EtcCanis:0.0,EtcFae11:0.0,UltEnt25:0.0,EtcFaeca:0.0,EtcFae12:0.0,Ul
tEnt28:0.0,Ult24839:0.0,EtcRivo2:0.0,Ult24895:0.0,Ult24989:0.0):5.5E-
4,(((UltEnt22:0.00763,(UltEnt27:5.4E-
4,(EtcGalli:0.04382,Ult24834:0.02706)0.741.23:0.00363)0.880.53:0.00374)0.
449.12:5.4E-4,(Ult24828:5.5E-

4,(Ult24819:0.00373,EtcHira2:0.01882)0.846.42:5.4E-
4)0.934.43:0.00372)0.481.3:5.4E-
4,(Ult24830:0.00371,UltBa741:0.00372)0.000.377:5.5E-4)0.485.8:5.5E-
4)0.000.378:5.5E-4,(UltBa742:0.00371,(EtcFaec7:0.00718,EtcFaec9:5.5E-
4)0.915.38:5.5E-4)0.000.379:5.5E-4)0.485.9:5.5E-4,EtcSpec3:5.5E-
4)0.485.10:5.5E-4,Ult24808:5.5E-4)0.356.4:5.5E-
4,Ult24832:0.00746)0.442.8:5.5E-4,EtcRivor:0.00372)0.464.5:5.5E-
4,Ult24809:0.00371)0.481.4:5.5E-4)0.000.380:5.5E-
4,EtcMund2:0.00371)0.000.381:5.5E-4)0.415.7:5.5E-
4,UltIsopo:0.00371)0.363.9:5.5E-4,((UltEnt17:5.4E-
4,UltLac89:0.02292)0.852.43:0.01111,EtcAsini:5.4E-
4)0.834.34:0.00371)0.000.382:5.5E-
4,(EtcSpec5:0.00372,((Ult24681:0.0,EtcSangu:0.0,EtcThail:0.0,EtcPhoen:0.0
,EtcSiles:0.0,EtcSpec6:0.0):5.5E-4,(((IsbMelis:5.5E-
4,Ult24842:0.00373)0.936.48:0.00342,(Ult24986:0.02919,Ult24991:0.05176)0.
042.2:0.01152)1.000.298:5.5E-
4,(EtcDisp2:0.00716,((BclArse2:0.01754,BclArsen:5.5E-
4)0.869.40:0.00391,(BclMacya:5.5E-
4,(BclAlka3:0.00374,(LstGray2:0.0,LstGrayi:0.0):0.00388)0.319.7:5.5E-
4)0.999.77:5.5E-4)0.770.53:0.00912)0.806.22:0.00493)0.158.3:5.4E-
4)0.138.6:5.4E-4)0.847.47:0.00449)0.676.4:5.5E-4)0.000.383:5.4E-
4)0.383.4:5.5E-4,Ult24837:5.5E-4)0.048.1:5.5E-4)0.150.2:5.5E-
4)0.000.384:5.4E-4,EtcFaec3:0.01125)0.456.5:5.5E-4)0.000.385:5.4E-
4)0.248.4:5.5E-
4,(Ult24836:0.00372,UltEnt26:0.00738)0.796.23:0.00367)0.253.6:5.5E-
4,(Ult24814:0.01103,(Ult24811:0.00372,(Ult24813:0.00372,UltBaci6:0.00372)
0.075.2:5.5E-4)0.999.78:5.5E-4)0.847.48:0.00364)0.156.4:5.5E-
4,((((((Tryy0102:0.0,AtPYy149:0.0,UltEnt18:0.0,Tryy0104:0.0,AtPYyy16:0.0)
:5.5E-4,Ult24793:0.00374)0.851.42:0.00373,Ult24825:0.0037)0.467.5:5.5E-
4,((((EtcSacc2:0.0,EtcSacch:0.0,EtcSacc3:0.0,Ult24810:0.0,UltLac88:0.0,Ul
t24821:0.0,Ult24816:0.0,Ult24815:0.0,EtcCasse:0.0,Ult24818:0.0,SwnFec11:0
.0,EtcCass3:0.0,BacNL379:0.0,Ult24829:0.0):5.5E-4,EtcCass2:5.5E-
4)0.000.386:5.5E-4,(EtcGall2:0.00746,Ult24894:0.00371)0.715.6:5.5E-
4)0.000.387:5.5E-4,(Ult24823:0.00371,Ult24826:0.00371)0.707.4:5.5E-
4)0.730.17:5.5E-4)0.727.13:5.5E-
4,(Ult24824:0.00747,Ult24817:0.00369)0.329.8:5.5E-4)0.664.6:5.4E-
4,((EtcSpec2:0.07383,Ult24822:0.00719)0.951.43:5.5E-
4,BacNL378:0.00369)0.168.5:5.5E-4)0.918.55:0.00362)0.813.31:5.5E-
4)0.825.28:0.00411,BacNL380:5.5E-4)0.366.3:5.5E-4)0.000.388:5.5E-
4)0.131.2:5.4E-4,Ult23806:0.00373)0.976.28:5.5E-4,(Ult24807:5.4E-
4,(Ult24805:0.00742,(Ult24806:5.5E-4,(EchggY19:0.00371,(EchggY20:5.5E-
4,Pshggg61:0.00373)0.156.5:5.5E-4)0.830.32:0.0037)0.887.48:5.2E-
4)0.849.39:0.00359)0.351.4:0.00366)0.801.30:0.00406)0.895.60:0.00435,(((Z
neTTTTY:5.4E-4,((UltBaci8:0.00372,Otu00574:5.5E-
4)0.935.47:0.00756,(UltEnt21:0.00372,(UltBaci7:0.0075,Ult24803:0.00374)0.
619.3:5.5E-4)0.917.46:5.3E-
4)0.574.6:0.00726)0.898.57:0.01117,(Otu00631:0.00482,(Otu00339:0.03289,Ot
u00373:0.04315)0.885.40:0.01929)0.934.44:0.01963)0.846.43:0.01062,TtrMuri
a:0.03478)0.892.42:0.01436)0.864.49:0.00486)1.000.299:5.4E-
4,(UltEnt19:0.02786,UltEnt20:0.01926)0.869.41:0.01594)0.801.31:0.01058)0.
094.1:5.4E-4,((((((Tryy0103:0.03652,((Ult24792:5.5E-4,UltStr19:5.5E-
4)0.972.36:5.5E-
4,(UltStr20:0.00757,Otu01346:0.0915)0.769.35:0.00377)0.983.25:0.02765)0.1
42.3:5.4E-

4,(((((Ult24790:0.0028,(Otu00271:0.01714,(Ult24789:0.00375,Otu00058:5.5E-
4)0.717.15:0.00271)0.794.35:0.00447)0.966.51:0.01688,((GutBacte:0.0,BacFC
C52:0.0):0.01146,((LatRaffi:0.0,LatRaff2:0.0,UltLac87:0.0,Ult24791:0.0):0
.00367,((LatPisci:0.0,Ult24787:0.0):5.5E-
4,Ult24788:0.00751)1.000.300:5.5E-
4)0.776.36:0.00378)0.735.20:0.00388)0.869.42:0.00811,((LatSpeci:0.01593,(
(Ult23917:0.01134,Ult24778:5.5E-
4)0.863.46:0.00753,(Ult24776:0.00375,((Ult24682:0.0,Ult24773:0.0,LatGarv2
:0.0,LatGarv3:0.0):5.5E-
4,(Ult24777:0.00375,LatGarvi:0.00387)0.445.10:5.5E-4)0.091.2:5.5E-
4)0.699.15:5.5E-
4)0.839.36:0.00759)0.558.4:0.01174,((UncUn200:0.03963,(UltStr17:0.01528,S
0114662:0.00424)0.913.44:0.02212)0.884.51:0.01633,(Ult24754:5.4E-
4,((Ult24772:0.01902,UltLac86:0.00369)0.780.44:0.00363,(Ult24684:5.5E-
4,(((((((Ult24786:0.00758,Ult24771:0.01144)0.764.36:0.00376,((LatLact2:0.
0,LatLact3:0.0,Ult24768:0.0,Ult24769:0.0,LatLact5:0.0,LatLact6:0.0,UltStr
16:0.0,AtPYy148:0.0,Ult24784:0.0,LatLact8:0.0,LatLact9:0.0,LatLac11:0.0,O
tu00662:0.0):5.5E-4,((((LatSpec2:0.00375,LatLact7:0.00763)0.198.7:5.5E-
4,LatLac10:0.00374)0.451.10:5.5E-4,Ult24767:0.00374)0.043.4:5.5E-
4,(LclDelb3:0.03514,(LatLact4:5.5E-
4,Ult24988:0.00374)0.852.44:0.00753)0.404.6:5.4E-4)0.000.389:5.5E-
4)0.000.390:5.5E-4)0.000.391:5.5E-4,Ult24785:0.00375)0.483.9:5.5E-
4,Ult24766:5.5E-4)0.465.8:5.5E-4,Ult24783:5.5E-4)0.259.4:5.5E-
4,Ult24782:0.00746)0.024.2:5.5E-4,Ult24770:0.00374)0.095.2:5.5E-
4)0.870.31:5.4E-4)0.842.42:0.00365)0.880.54:5.5E-4)0.652.6:5.5E-
4)0.805.32:0.01558)0.847.49:0.01004,(UltStr18:0.00748,((LcvMisce:0.02381,
((UltFir77:0.0,UltLac85:0.0):0.0633,((Ult24779:0.0114,(UltFir76:0.03372,(
UltFir75:0.0,Ult24780:0.0):5.5E-4)0.855.48:0.00395)0.639.2:5.4E-
4,UncUn201:5.3E-4)0.966.52:5.4E-4)0.942.46:0.01142)0.767.38:5.5E-
4,Ult24781:0.00721)0.988.48:0.02346)0.758.40:0.00576)0.843.33:0.01109,Ult
Fir78:5.4E-
4)0.903.46:0.0122)0.949.46:0.02195,((((((((StcSpec9:0.01133,((Ult24723:0
.0,StccSui3:0.0,Ult24762:0.0,StccSui2:0.0,StccSuis:0.0,Ult24759:0.0):5.5E
-4,Ult24761:0.00375)0.759.39:5.3E-4)0.789.27:0.00366,(UltStr15:5.4E-
4,Ult24760:0.00375)0.957.41:0.01151)0.780.45:0.00382,((((StcTherm:0.0,Stc
Ther2:0.0,Ult24713:0.0,Ult24714:0.0,Ult24715:0.0,Ult24716:0.0,Ult24719:0.
0,Ult24720:0.0,Ult24721:0.0):5.5E-4,Ult24722:5.5E-4)0.455.11:5.5E-
4,(UltStre8:0.00752,Ult20283:0.01913)0.701.12:5.5E-4)0.991.40:5.5E-
4,Ult24712:0.01903)0.780.46:0.00375)0.767.39:0.00373,((StcAgal2:0.00374,(
StcPorci:0.0,StcUberi:0.0):5.5E-4)0.787.31:0.00373,(StcPseud:5.3E-
4,StcEqui2:0.00375)0.800.25:0.00379)0.795.37:0.0037)0.941.46:0.00507,(Stc
Phoca:0.00757,StcDidel:0.00759)0.941.47:0.00502)0.941.48:0.00505,Ult24765
:0.00761)0.925.56:5.3E-
4,((((StcEnter:0.01136,((StcMarim:0.01531,((Ult24717:0.00362,((StcGordo:0
.0,Ult24698:0.0,StcGord2:0.0,UltStre5:0.0,StcSpec5:0.0):5.4E-
4,((((Ult24704:5.4E-
4,Ult26505:0.01136)0.827.30:0.00374,(((Ult24702:0.0,BacNL375:0.0,BacNL376
:0.0,StcPara2:0.0):5.5E-
4,((StcParas:0.00374,StcAustr:0.01535)0.474.9:5.5E-4,Ult24705:5.5E-
4)0.473.4:5.5E-4)0.000.392:5.5E-4,Ult24701:0.00374)0.042.3:5.5E-
4)0.912.58:0.00335,((Ult24696:0.00322,(((UltLac84:0.0,Ult24692:0.0,Ult247
07:0.0,StcPero2:0.0,StcPeror:0.0,UltStr14:0.0):5.5E-
4,Ult24706:0.00375)0.996.38:0.00322,((BacNL374:0.00195,UltStre4:0.01552)1
.000.301:0.0017,(StcSpec2:0.00372,((StcSpec3:0.00374,(Ult24695:0.00375,Ul

t24691:0.01136)0.898.58:5.5E-4)0.000.393:5.5E-
4,((StcSpeci:0.0114,(Ult24689:0.023,(StcPneum:0.0,StcPneu2:0.0):0.00374)0
.314.3:5.5E-4)0.417.4:5.5E-
4,(Ult24690:0.0,UltStre2:0.0,UltStre3:0.0,StcSpec4:0.0,Ult24694:0.0,StcOr
ali:0.0,StcOral2:0.0,Ult24697:0.0,StcOral3:0.0,StcSangu:0.0,StcMiti2:0.0,
UltStr13:0.0):5.5E-4)0.452.9:5.5E-4)0.485.11:5.5E-4)0.854.35:5.5E-
4)1.000.302:5.4E-4)0.691.8:0.00737)0.401.8:5.5E-
4,StcMitis:0.01573)0.455.12:0.00737)1.000.303:5.4E-
4,(((((UltStre6:0.0,StcSang2:0.0,StcSang3:0.0):5.5E-
4,BacNL377:0.00381)0.649.7:5.4E-
4,UltB4750:0.01915)0.832.30:0.0038,(Ult24724:0.01535,UltStre9:0.01144)0.8
94.39:0.00754)0.556.3:5.4E-
4,(Ult24703:0.0,StcOligo:0.0,StcSinen:0.0,StcCrist:0.0):5.4E-
4)0.571.4:5.3E-
4)0.454.7:0.00376)0.789.28:0.0039)0.438.7:0.00759,(Ult24699:5.5E-
4,Ult24700:0.07951)0.933.49:0.01519)0.879.49:5.3E-4)0.399.7:5.4E-
4,((StcMassi:0.0079,(StcMuta2:0.00374,((StcMutan:0.0,StcMuta3:0.0):5.5E-
4,(Ult24726:5.5E-4,UltStr12:5.5E-4)0.725.15:5.5E-4)0.763.45:5.5E-
4)0.988.49:0.03215)0.919.56:0.01522,StcFerus:0.03687)0.749.44:0.00471)0.7
67.40:0.00759)0.767.41:0.0038,(((StcAngin:0.0,StcInter:0.0,Ult24708:0.0):
5.5E-4,((Ult24709:0.00374,(Ult24710:0.0,StcAngi2:0.0):5.5E-
4)0.706.13:5.5E-
4,UltStre7:0.0076)0.844.58:0.00375)0.866.44:0.00374,StcInte2:5.3E-
4)0.806.23:0.00378)0.774.53:0.00388,StcSpe10:0.01157)0.883.54:0.0077,((St
ccEqu2:0.0,StccEqu3:0.0):5.4E-
4,((((StcHalic:0.01561,(((StcUrina:0.01143,(StcParau:0.01953,(StcIniae:0.
01145,StcInia2:5.4E-4)0.793.32:0.00377)0.439.7:0.0038)0.850.48:5.4E-
4,((StcDysga:0.00374,((UltStr10:0.01163,((StcPyoge:0.0,StcPyog2:0.0,StcPy
og3:0.0):0.00377,(StcCanis:0.00375,StcIctal:5.5E-4)0.131.3:5.5E-
4)0.764.37:0.00378)0.768.27:0.00376,(StcDysg2:0.0,StcDysg3:0.0):0.00374)0
.934.45:5.5E-4)0.895.61:5.3E-4,UltStr11:5.5E-4)0.954.49:5.4E-
4)0.816.28:0.00381,((StcOrisr:0.04003,(StcHenry:0.02344,(BacMpnI2:0.0,Ult
24725:0.0):5.5E-4)0.561.6:5.4E-4)0.926.53:0.00756,StcAgala:5.5E-
4)0.770.54:0.00376)0.887.49:0.00776)0.757.28:0.0039,((StcCasto:0.01562,((
(StccOvis:5.5E-
4,StcDevri:0.01946)0.913.45:0.01141,StcGalli:0.00755)0.767.42:5.5E-
4,StcMinor:0.00753)0.776.37:0.00387)0.884.52:0.01057,((((StcPlure:0.00965
,((Ult24764:0.00371,Ult24763:5.5E-
4)0.848.40:0.01039,((Ult24758:0.01115,((StcDenta:0.02196,StcMacac:0.05307
)0.867.48:0.01626,(StcSobri:0.0,StcDowne:0.0,StcDown2:0.0):5.4E-
4)0.854.36:0.00798)0.000.394:0.00358,(StcCrice:0.0,StcOrisu:0.0):0.00436)
0.768.28:0.00459)0.826.30:0.01278)0.317.7:0.00844,StcSpec8:0.00527)0.955.
37:0.0195,StcRatti:0.00533)0.805.33:0.00735,(Ult24757:0.01948,StcMerio:0.
01266)0.580.4:0.00696)0.912.59:0.01499)0.760.34:0.00544)0.404.7:0.00383,S
tccEqui:0.00411)0.913.46:0.01146,(((StcSpec6:0.00751,(Ult24735:0.06185,(U
lt23805:0.02356,Ult24741:0.10047)0.915.39:0.0185)0.732.12:0.00377)0.923.6
7:5.4E-4,((Ult24756:5.5E-
4,((Ult19758:0.0,Ult23804:0.0,Ult24729:0.0,StcGallo:0.0,Ult24731:0.0,Ult2
4732:0.0,Ult24746:0.0,Ult24749:0.0,Ult24755:0.0,StcAlact:0.0):5.5E-
4,(((Ult24685:0.03266,(Ult23922:0.09095,(Ult24687:0.02058,Ult24688:0.0410
9)0.975.38:0.04446)0.525.4:0.01402)0.913.47:0.02326,Ult24751:0.01558)0.71
8.14:0.00348,(Ult24739:0.00374,((Ult19757:0.0,Ult23803:0.0,Ult24686:0.0,U
lt24693:0.0,Ult24730:0.0,StcEquin:0.0,Ult24733:0.0,Ult24743:0.0,Ult24750:
0.0,Ult24747:0.0,Ult24748:0.0):5.5E-

4,(((Ult24740:0.00375,((Ult24734:5.5E-
4,Ult24738:0.0039)0.849.40:0.0039,(Ult24745:0.00115,Ult24742:0.03289)0.96
6.53:0.00285)0.530.5:5.5E-4)0.454.8:5.5E-4,Ult24737:0.00374)0.314.4:5.5E-
4,Ult24736:0.00375)0.483.10:5.5E-4)0.246.6:5.5E-4)0.334.6:5.1E-
4)0.850.49:0.00415)0.000.395:5.4E-4)0.553.6:5.4E-
4,(Ult24744:0.01112,StcSpec7:0.01529)0.746.32:0.00366)0.766.32:5.4E-
4)0.948.52:0.00741,StccEqu4:5.3E-4)0.845.42:0.00366)0.258.6:5.5E-
4)0.816.29:0.00374)0.855.49:0.00745)0.926.54:0.01103,((StcHyoi2:0.0,StcHy
oin:0.0):0.00382,StcCabal:0.01154)0.949.47:5.3E-
4)0.940.46:0.02066,(PilTermi:0.04533,Tryyy049:0.07463)0.801.32:0.01327)0.
191.3:0.01266)0.293.6:0.00402,UltFir80:0.00989)0.834.35:0.01107,Ult24775:
0.01042)0.995.51:0.054,((Ult24840:0.00424,LstSpeci:0.0438)0.000.396:5.4E-
4,((Ult24600:0.09498,Ult24602:5.4E-
4)1.000.304:0.05798,((((((LcsSpeci:0.0038,(LcsFalla:0.0077,((FruDurio:5.
5E-4,(FruFicul:5.4E-
4,FruPseud:0.00752)0.930.43:0.00746)0.932.52:0.00198,UltFir74:0.00201)0.9
17.47:0.01123)0.875.51:0.00752)0.923.68:0.0028,((Ult24646:0.02679,((OnsKi
tah:5.4E-
4,(OnsOeni0:0.01687,UltLac82:0.0782)0.305.2:0.01471)1.000.305:0.06997,((L
csCarno:5.5E-
4,Ult24636:0.0037)0.799.36:0.00373,(LcsKimch:0.00752,(((Ult24640:0.00369,
LcsGelid:0.0076)0.557.5:5.5E-
4,(Ult24638:0.0,Ult24639:0.0,Ult24644:0.0):5.5E-4)0.537.9:5.5E-
4,Ult24637:0.00369)0.885.41:5.5E-
4)0.781.39:0.00378)0.867.49:0.00744)0.847.50:5.4E-4)0.232.5:5.4E-
4,(((Ult24635:0.00369,((LatLacti:0.0,LcsCitre:0.0):5.5E-
4,EnmbnO30:0.00369)0.004.5:5.5E-
4)0.966.54:0.01559,(Ult24645:0.00369,LcsMese2:5.5E-
4)0.778.38:0.00338)0.331.5:0.00378,Ult24641:0.03469)0.850.50:5.5E-
4)0.995.52:0.0319)0.793.33:0.00311,(UltLeuco:0.00413,FruFruct:0.00735)0.7
78.39:0.00602)0.984.27:0.02912,(((WeiKoree:5.4E-
4,WeiKandl:0.00369)0.889.46:0.00762,((((UltLac83:0.0,WeiConfu:0.0,Ult2464
8:0.0,WeiHelle:0.0,WeiParam:0.0):5.5E-4,(WeiCibar:5.5E-
4,((WeiCiba2:0.00385,Ult24647:0.03078)0.762.45:0.00371,WeiSpeci:5.5E-
4)0.652.7:5.5E-4)0.000.397:5.5E-4)0.357.6:5.5E-
4,(WeiPara2:0.0,WeiPara3:0.0):0.00369)0.549.8:5.4E-
4,((Hmyy0024:0.00367,(Hmyy0023:5.5E-4,WeisSoli:5.5E-4)0.644.3:5.5E-
4)0.994.43:0.00205,UltB7232:0.03019)0.994.44:0.00169)0.743.33:0.00372)0.8
76.38:0.00783,(WeiHalot:0.00374,(WeiSpec2:5.4E-
4,Ult24649:0.01515)0.944.38:0.01128)0.896.37:0.00759)0.747.28:0.0035)0.99
0.42:0.04397,(WeiGhane:0.0,WeiSpec3:0.0):0.01169)0.993.31:0.05591,((((Ult
24900:0.0675,(LclNoden:0.00369,((Ult24577:0.00746,(((LclParal:0.0074,LclF
arci:0.03874)0.964.49:5.5E-4,LclCrust:5.5E-4)0.924.44:5.5E-
4,LclKimch:0.00369)0.994.45:5.5E-4)0.999.79:0.03476,LclTucce:5.5E-
4)0.898.59:0.00742)0.776.38:5.3E-
4)0.699.16:0.00743,(LclVersm:0.01082,(LclSpe11:0.01485,Ult24578:0.00401)0
.164.8:0.00377)0.775.36:0.00408)0.902.60:0.01083,((LclKunke:0.0381,(Ult24
582:0.03425,(UltLac80:0.01838,(UltLac78:0.00373,(UltBaci4:5.5E-
4,UltLac79:0.00364)0.783.43:0.00361)0.951.44:0.02159)0.771.43:0.00389)0.7
53.36:0.00397)0.869.43:0.00904,(((LclPsitt:0.00369,(UltLac62:0.0,LclForni
:0.0,LclJense:0.0):5.4E-
4)0.884.53:0.01492,(LclPlant:0.03655,((((Ult24415:5.5E-4,(LclAcido:5.5E-
4,Ult24422:5.5E-4)0.900.43:5.4E-
4)0.842.43:0.00369,((LclAmylo:0.01459,LclKalix:0.0104)0.781.40:0.00534,((

(Ult24532:0.0426,(Ult24418:0.01105,(Ult24417:0.00368,((Ult24416:0.0,Ult24
438:0.0,Ult24509:0.0):5.5E-4,Ult24433:0.00741)0.000.398:5.5E-
4)0.823.28:5.5E-4)0.968.36:5.5E-
4)0.823.29:0.00615,(LclAceto:0.00762,(UltLac61:0.00462,(((UltLact8:0.0037
8,((((UltLact6:0.0,UltLac60:0.0):5.5E-4,UltLact4:0.00368)0.782.44:5.4E-
4,(UltLac53:0.01144,(UltLac54:0.01483,(UltLact9:0.01111,(UltLac59:0.01123
,UltFir71:0.00369)0.569.7:5.5E-4)0.542.3:5.5E-
4)0.790.37:0.00342)0.917.48:0.01138)0.920.58:0.00166,(UltLact5:0.01107,(L
clInsec:0.00374,LcllApis:0.00739)0.934.46:0.01124)0.839.37:0.00218)0.955.
38:0.01433)0.449.13:0.00474,((UltLac55:0.00369,UltLac57:5.5E-
4)0.877.61:5.5E-4,(UltLac56:5.5E-
4,UltLac58:0.04132)0.840.45:0.00361)0.887.50:0.01019)0.120.3:0.01053,UltL
act7:0.01483)0.909.37:0.01668)0.971.37:0.02552)0.759.40:0.00469)0.815.27:
0.00728,((((Ult24443:0.0,LclSpeci:0.0,LclSpec2:0.0,LclSpec3:0.0):5.5E-
4,(LclDelbr:5.5E-4,Ult24442:5.5E-4)0.911.60:0.00369)0.829.38:5.5E-
4,Ult24446:0.00369)0.985.39:0.01906,(Ult24445:5.4E-
4,((LbcBacte:0.0,LclDelb2:0.0,Ult24447:0.0):5.5E-
4,Ult24444:0.0037)0.827.31:0.0037)0.946.45:0.01365)0.954.50:5.4E-
4)0.776.39:0.00828)0.809.34:5.4E-
4)0.922.47:0.00319,((Ult24441:0.00367,(((LclAcid2:0.0,LclGalli:0.0,UltLac
to:0.0,UltLact2:0.0,Ult24439:0.0,Ult24440:0.0,LclCrisp:0.0,LclCris3:0.0,U
lt24711:0.0):5.5E-
4,(Ult24419:0.00369,(Ult24425:0.04261,(((Ult24420:0.0,Ult24448:0.0):0.003
69,LclHelve:5.5E-4)1.000.306:5.5E-
4,Ult24423:0.03874)0.808.31:0.00367)0.721.7:5.5E-4)0.874.67:5.5E-
4)0.000.399:5.5E-4,((Ult24502:0.00369,(Ult24426:0.00369,Ult24437:5.5E-
4)0.772.40:0.00369)0.818.23:0.00369,Ult24450:0.01115)0.966.55:5.5E-
4)0.878.45:5.5E-4)0.771.44:5.5E-
4,((Ult24449:0.04638,LclHamst:0.00386)0.865.50:0.00233,((LclKefi2:0.0,Lcl
Kefir:0.0):0.00741,(LclHelv2:5.5E-4,Ult24421:5.5E-4)0.862.46:5.5E-
4)0.931.42:0.00245)0.931.43:0.00236)0.996.39:5.4E-
4)0.810.29:0.00738,Ult24589:0.0162)0.719.6:0.00971)0.733.18:0.01412)0.466
.9:0.0099,(Ult24504:0.00381,(LclAmyl2:0.01798,((((Ult24529:5.5E-
4,(Ult24472:5.5E-
4,((LclCris2:0.01122,((((Ult24488:0.00369,(((Ult24468:0.00369,(Ult24475:0
.0,Ult24476:0.0):5.5E-4)0.840.46:0.00369,((((((UltLac66:5.5E-
4,(((Ult24436:0.0,Ult24434:0.0,Ult24435:0.0,UltLact3:0.0,MouseGu4:0.0,Ult
24454:0.0,LclSpec4:0.0,UltLac64:0.0,LclGasse:0.0,Ult24457:0.0,Ult24458:0.
0,LclGass2:0.0,Ult24461:0.0,LclHomin:0.0,Ult24479:0.0,Ult24481:0.0,Ult244
84:0.0,Ult24499:0.0,Ult24498:0.0,Ult24500:0.0,Ult24501:0.0,Ult24487:0.0,U
lt24489:0.0,Ult24491:0.0,Ult24493:0.0,Ult24495:0.0,Ult24496:0.0,Ult24503:
0.0,Ult24515:0.0,Ult24517:0.0,Ult24518:0.0,Ult24519:0.0,Ult24521:0.0,Ult2
4522:0.0,Ult24523:0.0,Ult24525:0.0,Ult24526:0.0,Ult24527:0.0):5.5E-
4,((UltLac70:5.5E-
4,(((Ult24467:0.00369,(Ult24483:0.02265,Ult24512:0.01693)0.741.24:0.00392
)0.417.5:5.5E-4,Ult24490:0.01114)0.421.6:5.5E-
4,Ult24465:0.00369)0.480.3:5.5E-4)0.000.400:5.5E-4,(((((Ult24528:5.4E-
4,Ult24462:0.08559)0.826.31:0.00374,((LclJohns:0.00212,Ult24480:0.02285)0
.930.44:0.00179,Ult24477:0.01926)0.118.4:5.5E-4)0.798.35:5.3E-
4,Ult24492:0.00369)0.490.1:5.5E-4,Ult24514:0.00369)0.088.4:5.5E-
4,(Ult24453:0.00694,(Ult24474:0.01476,Ult24516:0.00184)0.000.401:5.4E-
4)0.866.45:0.00202)0.000.402:5.5E-4)0.634.5:5.5E-4)0.000.403:5.5E-
4,Ult24466:0.00369)0.475.9:5.5E-4)0.000.404:5.5E-
4,Ult24464:0.00369)0.299.6:5.5E-4,Ult24486:0.00369)0.000.405:5.5E-

4,Ult24470:0.00369)0.000.406:5.5E-4,Ult24510:0.00369)0.000.407:5.5E-
4,Ult24463:0.00369)0.000.408:5.5E-4)0.126.5:5.5E-
4,(Ult24451:0.00245,((Ult24460:0.0,Ult24469:0.0):5.5E-
4,(Ult24459:0.00369,(Ult24471:5.5E-4,Ult24478:0.00369)0.000.409:5.5E-
4)0.848.41:0.00369)0.920.59:0.00245)0.920.60:0.00245)0.288.3:5.5E-
4)0.066.4:5.5E-4,Ult24530:0.00741)0.000.410:5.5E-
4,Ult24482:0.00743)0.311.5:5.5E-4,(Ult24541:5.4E-
4,UltLac63:0.01119)0.839.38:0.00369)0.371.7:5.5E-4)0.446.4:5.5E-
4,(Ult24524:0.00741,(Ult24507:0.03934,((Ult24497:0.04764,(Ult24452:5.5E-
4,LclIners:0.00369)0.989.42:5.5E-
4)0.994.46:0.0318,(Ult24513:0.01555,(Ult24494:0.02604,Ult24531:0.01815)0.
888.54:0.01377)0.809.35:0.00742)0.767.43:0.00344)1.000.307:5.5E-
4)0.822.29:0.00368)0.411.5:5.5E-4)0.188.1:5.5E-4)0.297.2:5.4E-
4,Ult24485:0.00735)0.078.6:5.5E-4,Ult24520:0.00366)0.923.69:5.4E-
4,Ult24473:0.04221)0.988.50:0.03159)0.762.46:0.00621)0.764.38:0.00541)0.8
79.50:0.01177)0.780.47:0.01456)0.000.411:5.5E-
4,((PblSelan:0.00981,Ult24590:0.01707)0.918.56:0.01188,((LclHarbi:0.01485
,(LclConca:0.00732,(LclAlgid:0.02356,(((LclHayak:5.4E-
4,(Ult24611:0.02156,LclSpe16:0.02746)0.209.5:0.01098)0.886.63:0.01117,(((
((LcllCeti:0.01899,(((LclNagel:0.0,Ult24627:0.0):5.5E-
4,LcllVini:0.00742)0.804.29:0.00372,((((Ult24606:0.00365,Ult24609:0.02281
)0.914.49:5.4E-4,(Ult24605:5.5E-
4,(((Ult24594:0.00369,((Ult24603:0.0,Ult24607:0.0,Ult24593:0.0,Ult24596:0
.0,Ult24612:0.0,Ult24613:0.0,LclMurin:0.0,Ult24614:0.0,Ult24615:0.0,Ult24
752:0.0):5.5E-4,(Ult24608:0.0037,Ult24592:0.00369)0.715.7:5.5E-
4)0.000.412:5.5E-4)0.000.413:5.5E-4,Ult24604:5.5E-4)0.742.20:5.5E-
4,(Ult24597:0.00368,(Ult24595:0.03333,Ult24598:0.01174)0.882.54:0.01175)0
.790.38:0.0037)0.771.45:5.5E-4)0.716.11:5.5E-
4)0.915.40:0.0024,Ult24610:0.00248)0.915.41:0.00238,Ult24599:0.01124)0.79
1.25:0.00372)0.818.24:0.00361)0.600.6:5.5E-4,(LclRumin:5.5E-
4,Ult24616:0.00369)0.819.30:0.0037)0.826.32:0.00358,((Ult24625:0.0,Ult249
83:0.0):5.4E-4,((((LclSaliv:0.0,Ult24624:0.0,LclSali2:0.0):5.5E-
4,LclSpe14:0.00369)0.998.64:5.5E-
4,LclSali3:0.04672)0.932.53:0.00327,((LclAviar:0.00373,LclInter:0.0037)0.
791.26:0.00412,((LclAcid3:0.0,LclAcidi:0.0):5.5E-
4,LclSpe15:0.00743)0.837.27:0.00708)0.735.21:0.00749)1.000.308:5.5E-
4)0.438.8:0.0037)0.977.36:5.4E-4,((Ult24619:0.01118,LclSucic:5.5E-
4)0.784.36:0.0037,((LclHorde:0.0,LcllMali:0.0):0.0075,LclCacao:0.0037)0.7
69.36:0.00375)0.804.30:0.00371)0.898.60:0.00728,(Ult24618:0.00365,((Ult24
456:0.0,SwnFec10:0.0,Ult24623:0.0):5.5E-
4,(Ult24622:0.00369,Ult24617:0.00369)0.875.52:5.5E-4)1.000.309:5.4E-
4)0.949.48:0.01528)0.747.29:5.4E-4)0.580.5:5.4E-
4,LcllEqui:0.00724)0.701.13:0.01148)0.948.53:0.01694)0.290.7:0.00773)0.80
1.33:5.5E-
4,(((((PshYyy15:0.0,Pshggg59:0.0,Bfhggg73:0.0,Bfhggg76:0.0):5.5E-
4,(LclSuebi:0.00371,((LclMalef:0.01586,UltLac74:0.04732)0.899.51:0.01586,
(((PeiArgen:0.01314,PeiSiame:0.02996)0.041.5:0.00676,((PeiLolii:0.0074,((
PeiAcid3:0.0,PeiClaus:0.0):5.5E-4,((PeiAcidi:0.01774,PeiAcid2:5.5E-
4)0.852.45:0.00431,PeiPento:0.0037)0.423.6:5.5E-4)0.303.3:5.5E-
4)0.810.30:0.00443,(Ult24570:0.0,Ult24571:0.0):0.00367)0.879.51:0.00879)0
.911.61:5.4E-4,PeiDamno:5.4E-
4)0.785.40:0.00439)0.775.37:0.00437)0.460.4:5.4E-4)0.140.2:5.4E-
4,(((((LclCoryn:5.4E-
4,LclBacki:0.00763)0.749.45:0.00362,(((Bfhggg74:0.00372,Bfhggg75:0.00371)

0.170.4:5.5E-
4,LclRenni:0.02709)0.561.7:0.00367,(Ult24588:0.00734,(Ult25643:0.05348,Ul
t24587:5.5E-4)0.842.44:0.00374)0.274.4:5.4E-
4)0.926.55:0.01153)0.930.45:0.0117,((LclSpe10:0.0037,(LclFruct:0.01906,((
LclLindn:0.01453,UltLac73:0.00876)0.296.4:0.00751,(LclSanfr:0.01124,LclSp
ec9:5.4E-
4)0.960.45:0.01798)0.917.49:0.01277)0.771.46:0.00374)0.799.37:0.00372,(((
LclPara2:0.00747,(LclBrev3:0.0037,LclBrevi:5.5E-4)0.494.4:5.4E-
4)0.771.47:0.00355,(LclBrev2:0.02274,(((((Ult24551:0.00766,LclPonti:0.011
43)0.809.36:0.00331,((UltLac65:0.00906,Ult24511:0.01292)0.804.31:0.009,((
((Ult24540:0.0,Ult24569:0.0):0.00367,(((((Ult24544:0.05644,Ult24557:0.109
34)0.876.39:0.01502,LclPanis:5.5E-
4)0.833.38:0.00368,(((Ult24554:0.0074,(Ult24543:0.00369,((((Ult24547:0.00
369,((((LclReute:5.5E-
4,(Ult24555:0.00219,Ult24537:0.07348)0.995.53:0.0016)0.420.11:5.5E-
4,Ult24542:0.00369)0.367.8:5.3E-4,Ult24559:0.00369)0.000.414:5.5E-
4,(Ult24505:0.0,Ult24506:0.0,Ult24535:0.0,Ult24545:0.0,Ult24546:0.0,LclRe
ut2:0.0,Ult24548:0.0,LclAntri:0.0,LclIOris:0.0,Ult24552:0.0,LclFrume:0.0,
Ult24558:0.0):5.5E-4)0.000.415:5.5E-4)0.000.416:5.4E-
4,(UltLac67:0.00245,Ult24556:0.00245)0.912.60:0.00245)0.000.417:5.5E-
4,(Ult24539:0.00369,Ult24549:5.4E-4)0.840.47:0.00369)0.000.418:5.5E-
4,Ult24560:0.00369)0.157.5:5.5E-4)0.124.3:5.5E-4)0.427.6:5.5E-
4,LclSpec5:0.01125)0.111.2:5.5E-4,Ult24553:0.00368)0.233.4:5.5E-
4)0.407.6:5.5E-
4,(Ult24538:0.03017,Ult24550:0.05117)0.746.33:0.00379)0.080.2:5.5E-
4,(UltB7254:0.05682,(Ult24508:0.00311,Ult24534:0.02415)0.851.43:0.01459)0
.766.33:0.00507)0.625.5:5.5E-
4)0.417.6:0.00366,LclSpec6:0.01113)0.826.33:5.4E-
4,Ult24536:0.01501)0.772.41:0.00303)0.852.46:0.00812)0.986.33:5.3E-
4,(LclInglu:0.01763,((LclSpec7:0.0,UltRu515:0.0):0.0212,(LclSecal:0.00528
,(LclColeo:0.00368,UltLac69:5.5E-
4)0.980.23:0.02591)0.730.18:0.00786)0.351.5:5.3E-
4)0.506.7:0.01068)0.929.50:0.01551,LclNamur:0.00358)0.746.34:0.00374,Ult2
4579:0.00401)0.878.46:0.00736)0.962.51:5.4E-
4)0.887.51:0.00724,(UltLac75:0.00743,(Ult24572:0.00371,(((LclKefi3:0.0,Lc
lParab:0.0):0.00741,(LclSpec8:0.00369,(LclParak:5.5E-
4,((LclDioli:0.0,LclBuchn:0.0,LclFarra:0.0):0.0037,LclParaf:0.0037)0.778.
40:5.5E-4)0.876.40:5.5E-4)0.940.47:5.3E-
4)0.863.47:0.0074,(Ult24575:0.0037,(LclSimil:0.0,Ult24576:0.0):5.5E-
4)0.828.29:0.00366)1.000.310:5.4E-4)0.467.6:5.3E-
4)0.792.42:0.00354)1.000.311:5.4E-4)0.835.40:0.00364)0.770.55:5.4E-
4,(((LclFuch2:5.5E-
4,(LclFuchu:0.00379,((LclPlan2:0.0,LclPlan3:0.0,LclPento:0.0,EtcDura2:0.0
,EtcDuran:0.0,EtcDura3:0.0,EtcDura4:0.0):5.5E-
4,(LclCompo:0.00369,LclPlan4:0.00741)0.318.7:5.5E-
4)0.848.42:0.00369)0.961.43:5.5E-4)0.996.40:5.5E-
4,(((Ult24642:0.01503,Ult24800:0.00369)0.800.26:0.0037,(Ult24533:5.5E-
4,((Ult24632:0.00369,Ult24629:0.00369)0.646.7:5.5E-
4,((Ult24628:0.0,LclCurva:0.0,LclSakei:0.0,LclSake2:0.0,Ult24630:0.0,Ult2
4631:0.0,Ult24633:0.0):5.5E-4,LclCase4:5.5E-4)0.000.419:5.5E-
4)0.000.420:5.5E-4)0.864.50:5.5E-4)0.766.34:5.5E-
4,UltLac76:0.00743)0.854.37:0.0037)0.851.44:0.00369,Ult24573:5.4E-
4)0.869.44:0.00369)0.874.68:0.00414,(((((LclRhamn:0.00741,(LclOligo:0.011
3,((LclSaniv:0.00375,(Ult24584:5.3E-

4,(Ult24574:0.00743,Ult24585:0.00744)0.939.47:0.0112)0.915.42:0.0075)0.78
8.32:0.00368,LclBrant:5.5E-4)0.671.7:5.4E-
4)0.392.4:0.00369)0.780.48:5.5E-
4,(LclThail:0.0112,(Pshggg60:0.0,Ult24583:0.0):5.5E-
4)0.989.43:0.01892)0.428.6:5.5E-4,UltLac77:5.5E-4)0.759.41:5.5E-
4,LclSpe12:5.5E-4)0.671.8:5.4E-
4,(((LclSpe13:0.0037,(LclCase3:0.0062,(LclCase2:0.0,EtcDura5:0.0):5.5E-
4)0.197.6:5.5E-4)0.826.34:5.5E-
4,(LclPara3:0.00754,LclLarva:0.04842)0.755.32:0.00332)0.913.48:0.00308,Lc
lCasei:0.00384)0.948.54:5.1E-4)0.936.49:0.00721)0.045.4:5.4E-
4)0.883.55:5.4E-4,(((((Ult24568:0.0,LclEquig:0.0,Ult24566:0.0):5.5E-
4,LclGastr:0.00371)0.758.41:0.00414,Ult24567:0.04317)0.498.4:0.0072,((((L
clSilig:0.00366,LclRossi:0.00757)0.875.53:0.00992,Ult24626:0.06852)0.220.
4:0.00413,(((((UltLac71:5.5E-4,((UltLac72:0.0,LclFerm4:0.0):5.5E-
4,(LclFerm2:0.14454,LclFerme:0.01906)0.942.47:5.5E-4)0.000.421:5.5E-
4)0.000.422:5.5E-4,Ult24565:5.5E-4)0.382.5:5.5E-4,LcsMesen:5.5E-
4)0.224.4:5.5E-
4,LclFerm3:0.00367)0.870.32:0.00964,((Ult24562:0.0,Ult24563:0.0):5.5E-
4,Ult24564:0.0037)0.785.41:0.00556)0.857.52:0.00724)0.708.15:0.00433,Ult2
4561:0.07979)0.931.44:0.02486)0.958.53:0.01986,(((LclManih:5.5E-
4,Ult24580:0.05873)0.917.50:0.00187,LclParac:0.01106)0.923.70:0.00198,((L
clCamel:0.0,Ult24581:0.0):5.5E-
4,(Ult24621:0.00369,(Ult24586:0.00369,Ult24455:0.00744)0.167.5:5.5E-
4)0.000.423:5.5E-4)0.798.36:5.5E-4)1.000.312:5.4E-
4)0.808.32:0.00343)0.841.36:0.01308,UltLac68:0.03948)0.864.51:0.00881)0.8
92.43:0.01026)0.798.37:0.0048)0.000.424:0.00331)0.470.2:0.00742,((UltBaci
5:0.0,UltFir72:0.0):0.01031,(UltFir73:5.4E-
4,UltLac81:0.00363)0.607.5:0.00506)1.000.313:0.12867)0.825.29:0.01333)0.8
27.32:0.00834)0.800.27:0.01499)0.000.425:5.5E-
4)0.913.49:0.00773)0.789.29:5.5E-4,((((AeoUrina:0.00548,((AeoSangu:5.4E-
4,(AeoUrin2:0.00222,AeoChris:0.01673)0.993.32:0.0329)0.125.4:0.00371,((Ae
ocSuis:0.00756,UltAero2:0.02736)0.763.46:0.00354,(Ult24651:0.03071,((Ult2
4650:0.0,AeoViri2:0.0,AeoVirid:0.0):5.5E-
4,UltAeroc:0.00371)1.000.314:5.5E-
4)0.893.53:0.00764)0.833.39:0.0075)0.852.47:0.00938)0.718.15:0.00375,Ult2
4659:0.02508)0.934.47:0.01343,((ArhTaetr:5.4E-
4,(((Ult24658:0.04811,UltAero3:0.01152)0.875.54:5.5E-
4,Ult24666:0.01888)0.876.41:0.00759,(IgvRuoff:0.00733,Ult24657:0.02469)0.
868.39:0.00856)0.842.45:0.00748)0.848.43:0.00383,((FacHomin:5.5E-
4,(Ult24655:0.00389,(DolPauci:0.00758,(FacIgnav:0.04059,((FacLangu:5.5E-
4,Ult24653:0.00371)0.893.54:0.00372,(Ult24654:0.0,EreColeo:0.0):0.00751)0
.449.14:5.4E-
4)0.789.30:0.00372)0.763.47:0.00362)0.844.59:0.00727)0.786.43:5.4E-
4,FacMirou:0.01893)0.903.47:5.5E-4)0.933.50:0.01617)0.555.6:5.4E-
4,(((Ult24652:0.05738,FacSoure:5.5E-
4)0.951.45:0.01448,(Ult24661:0.10737,(AfsSemin:0.06876,(FirmOra2:0.00484,
(Ult24664:0.01041,(AptSuicl:0.0,Ult24665:0.0):0.00851)0.618.3:0.0076)0.75
9.42:0.00868)0.481.5:0.03816)0.129.4:0.00356)0.743.34:0.00491,((FacTabac:
0.00465,((AloOtiti:5.5E-
4,Ult24667:0.01497)0.958.54:0.03632,(Ult24668:0.0419,(DgrPigru:5.4E-
4,Ult24669:0.00742)0.984.28:0.04232)0.047.3:0.00836)0.932.54:0.02638)0.79
1.27:0.01251,Ult24656:0.03296)0.865.51:0.01085)0.928.43:0.01601)0.862.47:
0.01533)0.985.40:0.03185,((Ult21188:0.10041,(GmlBerge:0.00922,((GmlCunic:
5.5E-

4,(Ult24916:0.00746,(((Ult24915:0.00371,Ult24913:0.01894)0.916.53:5.5E-
4,(Ult24914:0.0,GmlHaemo:0.0):5.5E-4)0.490.2:5.5E-
4,Ult24912:0.00748)0.110.4:5.5E-
4)0.842.46:0.00372)0.842.47:0.00372,GmlPalat:5.3E-
4)0.715.8:0.00238)0.964.50:0.03677)0.838.46:0.01221,(((((BvbSpe12:0.01163
,BvbTherm:0.0037)0.889.47:0.01388,((BvbSpec6:0.0241,(BvbSpec4:0.01961,(((
((BvbBorst:5.5E-4,((BvbBrev3:0.0,BvbcAgri:0.0,BvbInvoc:0.0):5.5E-
4,(((BvbCentr:0.0,BvbChosh:0.0,BvbSpec3:0.0,BvbBrevi:0.0):5.5E-
4,BvbSpec2:0.00372)0.951.46:0.00751,BvbBrev2:0.02019)0.472.2:5.5E-
4)0.926.56:0.00396)0.178.4:5.5E-
4,(PbsSpe50:0.0,BvbSpec9:0.0,BclSp145:0.0):5.5E-4)0.356.5:5.4E-
4,(BvbLimno:5.5E-4,(BvbSpec7:0.01572,BvbSpec5:0.00754)0.391.8:5.4E-
4)0.847.51:0.00375)0.904.48:0.00812,(BvbGinse:0.00504,(BvbSpec8:0.01415,(
BvbLater:5.4E-4,(PbsSpe51:0.06127,PbsLarv3:5.5E-
4)0.814.27:0.00368)0.863.48:0.00884)0.928.44:0.01553)0.791.28:0.00635)0.9
08.50:0.00346,(UltBrevi:0.00765,(BvbSpe10:0.0,BvbSpe11:0.0):0.00766)1.000
.315:5.5E-
4)0.464.6:0.01204)0.895.62:0.01312)0.887.52:0.01212,BvbLevic:5.4E-
4)0.877.62:0.0113)0.882.55:0.01796,((((CoaLaevi:5.4E-
4,PbsSpe49:0.01182)0.838.47:0.00813,(CoaTherm:0.00798,((CoaYongn:5.4E-
4,((CoaHongk:0.0115,(CoaPhase:0.00374,UltPiet3:5.5E-4)1.000.316:5.5E-
4)0.933.51:0.0143,(CoaSpeci:0.01157,Ult25156:0.00761)0.909.38:0.00456)0.9
06.67:0.00406)0.322.5:0.01546,PahggY85:5.4E-
4)0.949.49:0.01503)0.743.35:0.00836)0.976.29:0.02429,(((((BclEdap2:5.4E-
4,BclEdaph:0.0231)0.983.26:0.0238,((PbsSpec5:0.00385,PbsSpec6:0.0076)0.93
3.52:0.01338,(((PbsLarva:0.0,PbsLarv2:0.0):0.00614,(PbsSpec2:0.00368,(Pbs
Pecti:5.5E-4,PbsSpeci:5.5E-
4)0.958.55:0.01555)0.926.57:0.0174)0.887.53:0.00834,(PbsKoleo:0.01554,(((
PbsSpec4:0.01204,Ult25129:0.02032)0.847.52:0.00743,(PbsSpec3:5.5E-
4,PbsChond:5.5E-4)0.841.37:5.4E-4)0.833.40:0.00372,PbsAlgin:5.4E-
4)0.941.49:0.01731)0.824.32:0.00784)0.899.52:0.0121)0.904.49:0.01376)0.19
0.3:5.4E-
4,(PbsSoli0:0.00793,(PbsNapht:0.01271,(PbsEhime:0.0028,((PbsElgii:0.0,Pbs
Spec8:0.0):5.5E-
4,PbsSpec7:0.00749)0.903.48:0.00864)0.926.58:0.01252)0.807.25:0.0065)0.89
7.42:0.0117)0.899.53:5.4E-4,(PbsVali2:5.5E-4,PbsValid:5.5E-
4)0.939.48:5.5E-4)0.942.48:0.00746,(((BclMuci2:5.5E-
4,(BclMucil:0.0,PbsMucil:0.0):5.5E-4)0.848.44:5.4E-4,(PbsSpec9:5.4E-
4,(PbsGinse:0.0,PbsPueri:0.0):0.01548)0.804.32:0.00376)0.788.33:0.00374,(
BclSp141:0.00381,PbsChinj:0.00764)0.894.40:0.00772)0.783.44:0.00385)0.702
.5:5.5E-
4)0.797.30:0.0094,(((((Ult25152:0.05374,(((Ult25154:0.01071,Ult25151:0.05
173)0.402.5:0.00762,Ult25133:0.03725)0.914.50:0.01553,(PbsSpe12:0.02748,P
bsSpe11:0.00566)0.876.42:0.01012)0.576.7:5.3E-
4)0.807.26:0.00764,((((Ult25134:0.00842,PbsSangu:0.03647)0.973.25:0.02979
,Ult25132:0.02215)0.331.6:0.00408,(PbsRumin:0.02688,((Ult25145:0.01106,(U
lt25069:0.00757,Ult25146:5.5E-4)0.993.33:5.3E-
4)0.951.47:0.01938,((PbsThail:5.5E-4,((PbsAgari:5.5E-
4,PbsSpe37:0.00376)0.855.50:0.00376,((PbsSpe38:0.03677,(((PbsCook2:0.0,Pb
sCooki:0.0):0.0116,Ult25148:0.01568)0.856.34:0.00766,((((PbsSepte:0.00788
,(PbsGrani:0.01152,(PbsMonta:5.5E-4,PbsSiame:0.01135)0.164.9:5.4E-
4)0.881.51:0.00765)0.891.45:0.00774,PbsSpe39:5.5E-
4)0.926.59:0.00426,Ult25147:0.01848)0.929.51:0.00423,BclSp144:5.4E-
4)0.341.9:5.4E-

4)0.800.28:0.00547)0.745.33:0.00579,PbsSpe40:0.01076)0.778.41:0.00573)0.325.6:5.5E-4)0.830.33:0.00381,PbsNanen:5.4E-4)0.828.30:0.00804)0.933.53:0.02288)0.779.39:0.01047)0.840.48:0.01135,((PbsChiti:0.00374,PbsPolym:5.5E-4)0.891.46:0.01131,(Ult25135:0.03865,(PbsConta:0.0152,PbsGansu:0.0332)0.403.7:0.00768)0.851.45:0.00982)0.795.38:0.00281)0.862.48:0.00981)0.865.52:0.00854,((((PbsAgare:0.00522,(PbsSpe41:0.0141,Ult25149:0.00522)0.739.26:0.01586)0.786.44:0.0091,((PbsAlkal:0.00376,PbsHaren:5.5E-4)0.881.52:0.01012,Ult25139:0.08035)0.493.9:0.0035)0.832.31:0.00717,(((Ult25143:0.03892,PbsTuric:0.01823)0.545.10:0.01366,((((PbsStell:0.01178,((PbsDurus:0.0,PbsDuru2:0.0):5.4E-4,(PbsSabin:5.5E-4,PbsZanth:5.5E-4)0.847.53:0.00384)0.911.62:0.01185)0.921.57:0.0121,PbsSpe21:5.3E-4)0.997.46:5.4E-4,(((((UdnBac73:0.00375,(PbsGrami:0.0,Ult25141:0.0):0.00375)0.915.43:5.5E-4,PbsOdori:5.5E-4)0.000.426:5.5E-4,(PbsWynni:0.0,BclSp143:0.0):5.5E-4)0.973.26:5.3E-4,(PbsCaesp:5.5E-4,(BacteH25:0.00372,PbsBorea:5.4E-4)0.864.52:0.00373)0.931.45:0.00751)0.909.39:0.00749,PbsSpe24:5.5E-4)0.473.5:0.00372)0.891.47:0.00354,((PbsGram2:5.5E-4,PbsSpe23:5.5E-4)0.891.48:5.5E-4,(PbsSpe25:0.00762,((PbsMacer:0.00729,Ult25140:0.01122)0.913.50:5.3E-4,(PbsRiogr:0.00375,PbsSpe22:5.5E-4)0.968.37:0.01163)0.810.31:0.00378)0.836.30:5.3E-4)1.000.317:5.5E-4)0.850.51:0.01319)0.732.13:0.00787,((PbsGleba:0.0,PbsSpe10:0.0):0.0193,(((PbsSpe45:0.0,PbsTellu:0.0):0.04914,PbsDaeje:5.5E-4)0.979.36:0.01535,((((PbsCellu:0.0,PbsSpe46:0.0):0.00739,PbsCurdl:5.5E-4)0.945.46:0.01985,((PbsSpe42:0.01141,PbsXinji:5.4E-4)0.964.51:0.01143,(PbsCasta:5.4E-4,(PbsSpe43:0.00375,PbsSpe44:5.4E-4)0.909.40:0.00756)0.495.6:5.4E-4)0.842.48:0.00758)0.898.61:0.0117,((PbsTarim:0.0076,(UltEarth:0.0039,PbsSpe47:0.02673)0.925.57:0.00422)0.861.54:0.00701,(((PbsPhyll:0.00769,Ult25150:0.01952)0.915.44:5.4E-4,(((PbsSepul:0.00855,(ScbKuerl:0.0,ScbSacch:0.0):0.04502)0.752.33:0.01577,PbsMend2:0.00466)0.804.33:0.00296,PbsMende:0.00382)0.857.53:0.00728)0.868.40:0.00733,(((PbsPasad:0.0,PbsHumic:0.0):0.01184,PbsSpe48:0.01565)0.715.9:0.00804,Ult25073:0.05574)0.736.23:0.00329)0.877.63:0.00209)0.868.41:0.00224)0.884.54:0.0077)0.820.21:5.3E-4)0.981.35:0.02602)0.407.7:0.0037)0.931.46:0.0139,((((Ult25144:0.01524,(PbsXylan:5.3E-4,Ult25138:0.00375)0.973.27:5.4E-4)0.875.55:0.01354,((PbsSpe19:5.5E-4,Ult25137:0.00751)0.864.53:0.0123,((PbsAssam:0.0233,PbsTaiwa:5.5E-4)0.758.42:0.00797,(PbsAlvei:0.01599,PbsApiar:0.00348)0.837.28:0.00797)0.946.46:0.02142)0.776.40:0.01437)0.943.45:0.01815,(UltPaen2:0.03225,(Ult25153:0.0076,((PbsLenti:0.0,PbsSpe35:0.0,PbsThiam:0.0):5.5E-4,PbsSpe36:0.00374)0.924.45:0.01161)0.779.40:0.00763)0.913.51:5.4E-4)0.837.29:0.00377,((((PbsMotob:0.02902,((PbsMace2:0.00738,(PbsPoly4:0.01558,((PbsSpe33:0.01141,PbsKribb:5.5E-4)0.961.44:5.4E-4,((PbsPoly2:0.0,PbsPeori:0.0,PbsPoly3:0.0):5.5E-4,PbsSpe32:0.00377)0.816.30:0.00373)0.348.6:0.00381)0.940.48:0.01628)0.770.56:0.00391,(PbsTimo2:5.5E-4,(PbsTimon:5.5E-4,(PbsBaren:0.0038,PbsBare2:0.00376)0.798.38:0.00374)0.848.45:0.00376)0.924.46:5.5E-4)0.557.6:0.00705)0.889.48:0.01652,PbsKonsi:0.01137)0.774.54:0.00411,((PbsWooso:0.01153,(PbsFonti:0.00753,PbsSpe26:0.01949)0.769.37:0.00371)0.770.57:0.00381,PbsSpe34:0.00748)0.762.47:0.00385)0.777.38:0.00353,(((((PbsSpe

16:5.4E-4,BclPumi6:0.03799)0.838.48:0.00364,(PbsAmylo:5.5E-
4,((PbsSpe13:0.0,BclSp142:0.0,PbsTaich:0.0):5.5E-4,((PbsIllin:5.5E-
4,((PbsPanac:0.00756,(PbsSpe18:0.0,Udntfd13:0.0):5.5E-
4)0.862.49:0.00377,(PbsBarci:5.5E-
4,(PbsSpe15:0.0025,PbsSpe17:0.0025)0.935.48:0.0025)0.883.56:0.00377)0.422
.5:5.4E-4)0.852.48:0.00374,PbsSpe14:0.00762)0.651.10:5.5E-
4)0.000.427:5.3E-4)0.991.41:5.4E-
4)0.920.61:0.00202,(UltComp9:0.03158,(Ult25136:0.03191,(PbsAzore:0.01149,
(PbsCiner:5.5E-4,PbsSpe20:0.00375)0.921.58:5.5E-
4)0.923.71:0.01213)0.587.7:0.00738)0.854.38:0.00758)0.916.54:0.00206,((Pb
sAnaer:5.5E-4,PbsGins3:5.5E-4)0.895.63:0.00785,(PbsMacq2:5.5E-
4,PbsMacqu:5.5E-4)0.800.29:0.00384)0.791.29:0.004)0.858.44:5.4E-
4,((PbsSpe29:0.00755,Ult25142:5.5E-
4)0.954.51:0.01573,(PbsCampi:0.01142,((PbsSpe28:0.0,PbsSpe31:0.0):0.01142
,((PbsLautu:0.0,PbsLaut2:0.0):5.4E-4,(PbsSpe27:0.0191,(PbsSpe30:5.5E-
4,PbsLacti:0.00754)0.833.41:5.5E-
4)0.793.34:0.00372)0.337.5:0.00375)0.741.25:5.4E-
4)0.749.46:0.00362)0.962.52:0.0197)0.447.4:0.00385)0.885.42:0.00788)0.381
.8:5.4E-
4)0.869.45:0.00805)0.925.58:0.01268,((Ult25155:0.02965,Ult25157:0.10365)0
.817.20:0.01315,Ult25158:0.03489)0.777.39:0.0054)0.744.26:0.00409,((Ult25
130:0.03269,(Ult25131:0.02642,(TmsCompo:0.0,TmsXylan:0.0):0.05821)0.848.4
6:0.01547)0.737.36:0.00352,(UltPaeni:0.01954,(PbsGins2:0.00373,PbsHodog:5
.5E-4)0.950.35:0.01909)0.798.39:5.3E-
4)0.981.36:0.01987)0.745.34:0.00223)0.853.37:0.01568)0.999.80:0.05195,(((
((UltGeob3:0.01187,UltGeob4:0.04791)0.249.3:0.00309,(UltGeob5:0.00369,Ult
25594:0.02716)0.005.1:0.00398)1.000.318:0.08197,(Ult25121:0.01709,(Ult251
27:5.5E-
4,Ult25128:0.16802)0.949.50:0.01729)0.762.48:0.00758)0.879.52:0.01695,(Bl
cBacte:0.06809,(((((MecMesop:0.01339,(SeiPepto:0.06609,(UltFir84:0.07444,
(Ult27569:0.14171,(Ult25123:0.00407,(Ult25122:0.00376,ShzKribb:0.00377)0.
902.61:0.01122)0.649.8:0.01954)0.800.30:0.02011)0.904.50:0.04065)0.953.33
:0.04655)0.689.10:0.00173,(TmcSangu:0.01224,(DmpActiv:5.4E-
4,(TgnSpeci:0.02733,LowGC002:0.0155)0.642.5:0.00742)0.932.55:0.01471)0.85
9.43:0.00788)0.830.34:0.01147,((TmfDicho:0.02258,(Ult25125:5.4E-
4,(TmcInter:5.3E-4,(LllSacch:0.03618,(BaiBacte:0.01935,TmcVulga:5.4E-
4)0.629.9:0.01104)0.646.8:5.4E-
4)0.977.37:0.01908)0.487.10:0.01724)0.871.47:0.0123,(PlfYunna:0.01126,Plf
Fimet:5.5E-
4)0.992.46:0.03423)0.579.7:0.00646)0.392.5:0.00758,Ult25124:0.02694)0.947
.41:0.02705,((AmmOxali:0.01314,(OxpOxali:0.01138,Ult25126:5.4E-
4)0.882.56:0.01007)0.883.57:0.01677,(ClkTherm:0.02774,(ClkUzone:0.00704,(
BclTher2:0.01135,(BclSp134:5.5E-
4,(UreTherm:0.00755,(UreCompo:0.0,UreTher2:0.0,UreSuwon:0.0):0.00375)0.78
0.49:0.00373)0.769.38:0.00374)0.962.53:0.02181)0.894.41:0.01659)0.894.42:
0.02411)0.793.35:0.0112)0.563.3:0.01435)0.834.36:0.01536)0.880.55:0.01443
,(((Bac95LM4:0.04344,SsgKocur:0.00564)0.982.27:0.04864,((((MroHalop:0.0,M
roSpeci:0.0):0.04046,(PotHalop:0.02682,((UltBa745:0.09253,((Ult25111:0.01
285,Ult25112:0.01033)0.999.81:0.06407,((GclHalop:0.0155,((Ult24962:0.0516
5,((((BlcBact3:0.03094,LntSalis:0.00438)0.850.52:0.00752,(LntJurip:0.0122
4,LntHalop:0.01921)0.838.49:0.0074)0.738.15:0.00383,LntLacis:0.01196)0.79
3.36:0.00707,((((BclMurim:0.01109,(HctHalop:0.00387,HctMiure:0.00383)0.92
8.45:0.01185)0.767.44:0.00746,(OnbChiro:0.01138,((Ult25108:0.01501,(OnbKa
pia:0.00374,OnbPictu:5.5E-4)0.949.51:5.5E-

4)0.760.35:0.00766,(((OnbSpec2:0.0,OnbSpec3:0.0,OnbcSoja:0.0):5.4E-
4,((OnbSpeci:0.0,OnbIhey2:0.0):5.3E-
4,OnbIheye:0.01126)0.956.42:0.01137)0.829.39:0.00369,(OnbSpec4:5.5E-
4,OnbOncor:0.00755)0.706.14:5.4E-
4)0.917.51:0.01178)0.846.44:0.00765)0.772.42:0.0114)0.971.38:5.4E-
4,((Ult25102:0.01551,(Ult25099:0.00607,Ult25100:0.00639)0.782.45:0.00633)
0.924.47:0.01153,(((((BclBadi2:0.0,BclCoagu:0.0,BclCoag3:0.0):5.4E-
4,(BclCoag2:5.4E-4,((SrlTerra:0.00372,(SrlLaevo:5.5E-
4,SrlNakay:0.00372)0.980.24:5.5E-
4)0.776.41:0.00437,(SrlInuli:0.01212,SrlVinea:0.01886)0.695.10:0.00757)0.
940.49:0.01463)0.789.31:0.00371)0.777.40:0.00741,BclSp131:5.5E-
4)0.933.54:0.01126,(BclOler2:0.00371,(BclOlero:5.5E-4,BclOler3:5.5E-
4)0.271.3:5.3E-
4)0.789.32:0.0037)0.871.48:0.00757,((((GclHalot:0.00748,(GclDipso:5.4E-
4,(GclSpec2:5.4E-4,(GclLacis:0.00372,GclSalip:5.4E-
4)0.935.49:0.00752)0.825.30:0.00372)0.878.47:0.00754)0.764.39:0.00488,((A
mhSpeci:0.0114,AmhSedim:0.0104)0.768.29:0.005,(AmhXylan:0.01041,(AmhTropi
:0.00388,(NrbAzoti:0.00408,(PaaQuing:0.01113,(GclSpeci:0.01138,(PaaRyuky:
0.0038,GclBorac:0.01929)0.880.56:0.00758)0.864.54:5.5E-
4)0.860.34:0.00761)0.772.43:0.00328)0.786.45:0.00497)0.781.41:0.00362)0.8
71.49:0.00755)0.818.25:0.00651,HlkHalop:5.3E-4)1.000.319:5.4E-
4,(((BclRuris:0.0,BclGalac:0.0):0.01956,Ult25081:0.00765)0.915.45:0.01206
,FblSpeci:0.02291)0.842.49:0.00797)0.747.30:0.0037)0.790.39:0.00387)0.798
.40:0.0035)0.798.41:0.00764,((SrlDextr:0.0269,(OphioYy8:5.3E-
4,((UltComp7:0.01516,(UltComp6:0.00691,(Ult25159:0.03478,(Ult25096:5.5E-
4,((BclSp132:0.00118,BclSp133:5.5E-
4)1.000.320:0.0025,(AnlToebi:0.0,Ult25097:0.0):0.02729)0.777.41:0.0038)0.
651.11:5.4E-4)0.668.9:5.0E-
4)0.672.8:0.00824)0.443.3:0.00702,(BclTherm:5.4E-
4,Ult25098:0.01131)0.791.30:0.00435)0.889.49:0.01033)0.760.36:0.01271)0.7
75.38:0.00681,(BclAlvea:5.5E-4,(GbcSpec8:0.01149,Ult25070:5.5E-
4)0.808.33:0.00769)0.850.53:0.01535)0.917.52:0.01396)0.783.45:0.00367)0.7
57.29:0.0036)0.765.26:0.00426,(((BclTrypo:0.02459,(BclAlca2:5.5E-
4,(BclAlca3:0.0,BclAlca4:0.0):5.5E-4)0.798.42:5.4E-
4)0.779.41:0.00497,(BclBogor:0.0212,((BclLehen:0.0,BclSpe90:0.0):5.5E-
4,((((BclClau2:0.0,BclClaus:0.0,BclClau3:0.0,BclClau4:0.0):5.5E-
4,BclAlcal:0.00369)0.843.34:0.00369,UdndedeY:5.5E-
4)0.833.42:0.00365,BacEnr43:0.03872)0.756.44:5.4E-
4)0.971.39:0.02316)0.168.6:0.00677)0.929.52:0.0151,Ult25072:0.00388)0.762
.49:0.00355)0.984.29:0.02431)0.778.42:0.0026,VrgSpeci:0.00475)0.544.4:0.0
1362)0.308.6:0.00837)0.729.16:0.00645,(SmcHalop:0.01507,SmcLuteu:5.5E-
4)0.987.43:0.03449)0.653.3:0.01284)0.838.50:0.00985)0.878.48:0.01451,(Hkl
Bacte:5.5E-
4,Ult25119:0.0442)0.990.43:0.0561)0.279.3:0.00443,SasAlbus:0.07476)0.813.
32:0.01075)0.613.9:0.01,(BclAidin:0.007,(BclSalar:0.00379,HlpBacte:0.0113
7)0.876.43:5.3E-
4)0.100.3:0.00622)0.916.55:0.02332)0.873.48:0.00991)0.198.8:5.4E-
4,BclChaga:0.05957)0.944.39:0.02474)0.747.31:0.00412)0.853.38:0.0095)0.65
3.4:0.00761)0.700.19:0.01716,((((Ult25769:5.3E-
4,(ShtGlyco:0.01549,(DehRestr:0.00368,Ult25768:0.01521)0.760.37:0.0039)0.
941.50:0.01525)0.997.47:0.04996,(OphiYyy7:0.00421,(UltFir93:0.01822,UltVe
i11:0.0316)0.764.40:0.00563)0.908.51:0.02106)0.906.68:0.02117,(UltGram7:0
.0822,(OphioY10:0.03174,Ult25578:5.3E-
4)0.357.7:0.00578)1.000.321:0.11391)0.738.16:0.00291,((Ult25729:0.04421,U

lt25739:0.04428)0.980.25:0.04786,((((UltVeil1:0.05334,(((((Ult25618:0.015
39,((Ult25615:0.0,Ult25616:0.0,Ult25617:0.0,Ult25609:0.0,Ult25612:0.0):5.
5E-4,Ult25610:5.5E-4)0.987.44:5.4E-4)0.851.46:0.00375,Ult25611:5.4E-
4)0.000.428:5.4E-4,(((Ult25608:0.02345,(Ult25613:5.5E-
4,(Ult25614:0.00375,Ult25715:0.01524)0.000.429:5.4E-4)0.923.72:5.3E-
4)0.330.4:0.00378,(Ult25619:0.02398,DtrMicra:0.02387)0.757.30:0.00416)0.8
71.50:0.00771,(DtrPropi:5.3E-
4,(((((((Ult25598:0.0134,(Ult25601:0.014,Ult30312:0.08836)0.548.5:0.0158
8)0.759.43:0.00183,Ult25599:0.01537)0.931.47:5.4E-4,Ult25603:5.5E-
4)0.472.3:5.5E-
4,(Ult25602:0.0,Ult25604:0.0,Ult25605:0.0,Ult25597:0.0,UltDiali:0.0,Ult25
607:0.0):5.5E-4)0.474.10:5.5E-4,Ult25595:5.5E-4)0.422.6:5.5E-
4,Ult25596:0.00374)0.137.5:5.5E-
4,Ult25600:0.00375)0.923.73:0.00203,Ult25606:0.00203)0.945.47:0.0111)0.77
2.44:0.00382)0.251.4:0.00378)0.985.41:0.02223,(Ult25623:0.00993,(((AsnHis
ta:0.0,Ult25621:0.0):5.5E-
4,Ult25620:0.00373)0.997.48:0.0459,(DtrPneum:0.0,DtrSpeci:0.0):0.0117)0.2
18.3:0.00539)0.000.430:5.2E-4)0.560.5:0.02068,Ult25622:5.5E-
4)0.967.37:0.03061)0.380.5:0.00957,Ult24620:0.06646)0.638.5:0.01441,(UltM
egas:0.01705,(((((MgsMicro:5.5E-4,Ult25632:5.5E-
4)0.890.56:0.01541,((VeiMagna:0.01214,(UltVeil3:0.05296,(((Ult25633:5.5E-
4,((Ult25638:0.02308,(((Ult25635:0.0,Ult25646:0.0):5.5E-
4,(Ult25637:0.02308,Ult25641:0.00752)0.887.54:5.5E-4)0.793.37:5.5E-
4,(VeiRoden:0.00731,Ult25677:0.04821)0.920.62:5.3E-4)0.377.6:5.4E-
4)0.856.35:5.4E-4,VeiAtypi:5.5E-4)0.936.50:0.00736)0.921.59:5.5E-
4,((BacRA211:0.01261,Ult25647:0.01101)0.494.5:0.00741,VeiMontp:0.02317)0.
737.37:0.0047)0.709.10:0.00334,((Ult25634:0.0,UltVeil2:0.0,Ult25640:0.0,U
lt25644:0.0):5.4E-
4,(Ult25636:0.02324,Ult25639:0.00232)0.985.42:0.00166)0.790.40:0.00432)0.
841.38:0.01262)0.922.48:0.02487)0.797.31:0.00783,(Ult25645:0.01531,(VeiRa
tti:5.5E-
4,Ult25642:0.00375)0.860.35:0.00813)0.859.44:0.01173)0.945.48:0.01721)0.8
64.55:0.0129,(MgsSpeci:0.01623,Ult25629:0.00695)0.882.57:0.00819)0.758.43
:0.00398,Ult25630:0.00838)0.771.48:0.00441,((MgsElsde:0.01592,(Ult25628:0
.00374,((Ult25624:0.0,Ult25625:0.0):5.5E-
4,(Ult25627:0.00756,Ult25626:0.00752)0.707.5:5.5E-4)1.000.322:5.4E-
4)0.809.37:0.01046)0.899.54:0.02319,(MgsSueci:0.02441,(Ult25631:0.00844,A
rgGemin:0.03317)0.919.57:0.02741)0.827.33:0.01449)0.851.47:0.01375)0.930.
46:0.01822)0.907.52:0.01354)0.952.47:0.03617,(((((SpuAcido:5.5E-
4,(SpuAeriv:5.5E-4,((SpuSphae:5.5E-
4,Ult25701:0.0037)0.924.48:0.0116,(SpuMalon:5.4E-
4,(((SpuSilva:0.01533,UltSpor2:5.4E-
4)0.924.49:0.00753,(SpuOvata:0.0072,Ult25702:0.01122)0.907.53:5.5E-
4)0.807.27:0.00377,SpuRhiza:0.00382)0.931.48:0.01134)0.801.34:0.00763)0.8
55.51:0.0109)0.846.45:0.02299)0.999.82:0.07797,Ult25709:0.03662)0.926.60:
0.03142,(((Ult25704:0.02829,Ult25708:0.06095)0.881.53:0.01853,((Ult25707:
0.02626,Ult25705:0.01346)0.717.16:0.00876,((Ult25703:0.00366,UltEu106:5.4
E-
4)0.715.10:0.01518,Ult25706:0.04389)0.080.3:0.00826)0.759.44:0.00448)0.83
5.41:0.0128,((((Ult25713:0.02351,Ult25759:0.02266)0.943.46:0.02467,(((Ult
25695:0.0078,Ult25699:0.03171)0.887.55:0.01137,(Ult25712:0.04539,((VllBac
t2:5.5E-
4,(UltPelos:0.00754,(Ult25697:0.0,Ult25698:0.0):0.01613)0.144.4:5.3E-
4)0.996.41:0.00224,((Ult25696:0.0,SpeColon:0.0):0.01146,UltVeil4:0.00378)

1.000.323:0.00165)0.997.49:0.03712)0.773.38:5.3E-
4)0.740.34:0.00407,(Ult25700:0.00766,((Ult25757:0.04021,((((((UltVei10:0.
00374,UltVeil9:5.5E-4)0.872.56:5.4E-
4,(((Otu00567:0.04693,(UncUn212:0.04027,UltFir92:0.08917)0.866.46:0.02521
)0.751.20:0.01691,UncUn211:0.03149)0.950.36:0.03068,(UncUn213:0.03253,(Ul
t25754:0.03986,Bfhggg82:5.5E-
4)0.492.8:0.01433)0.883.58:0.01357)0.958.56:5.5E-
4)0.798.43:0.00454,Ult25755:0.03478)0.484.4:0.00381,PahggY88:0.04018)0.89
6.38:0.01172,(UncUn207:5.5E-4,((UncUn209:0.00375,UncUn210:5.5E-
4)0.975.39:0.01158,UncUn208:5.5E-
4)0.828.31:0.00375)0.921.60:0.01214)0.740.35:0.00444,((ScpMobil:0.02658,U
lt25751:0.02265)0.910.60:0.01828,(((((((Ult25746:0.0,UltRu545:0.0):5.5E-
4,Ult25747:0.00375)0.966.56:5.4E-
4,(UltRu543:0.01911,(UltRu542:0.01146,UltRu541:0.01136)0.754.29:0.00375)0
.762.50:0.00366)0.945.49:5.4E-
4,(UltRu544:0.02351,(UltRu540:0.00346,Ult25745:0.02293)0.844.60:0.00235)0
.864.56:0.01025)0.433.8:0.00418,(Ult25744:0.00526,(Ult25749:5.5E-
4,(Ult25748:5.4E-
4,Ult25750:0.01954)0.842.50:0.00368)0.987.45:0.02724)0.943.47:0.01844)0.9
82.28:0.0317,((AdnIntes:5.5E-4,(Ult25753:0.05299,(AdnFerme:5.5E-
4,AdnSpeci:0.00379)0.987.46:5.4E-
4)0.581.8:0.0037)0.887.56:0.01358,(((Ult25742:0.00374,Ult25743:0.00385)0.
902.62:0.01496,(Ult25741:0.03949,((Bfhggg80:0.00374,Bfhggg81:5.5E-
4)0.927.49:5.5E-4,((Ult25740:0.0,PbmFaeci:0.0):5.5E-
4,(Ult25736:0.00377,Ult25735:5.5E-
4)0.825.31:0.00375)0.970.41:0.01149)0.678.10:0.00506)0.064.3:0.00485)0.84
7.54:0.01755,(((((Ult25737:5.4E-
4,Ult25730:0.13765)0.836.31:0.00732,((Ult25718:0.0,Ult25726:0.0):5.5E-
4,(Ult25724:0.00753,Ult25719:0.00376)0.609.9:5.4E-
4)0.848.47:0.00375)0.702.6:5.3E-
4,(Ult21405:0.0,Ult23232:0.0,Ult25723:0.0,Ult25725:0.0,Ult25727:0.0,Ult25
728:0.0,Ult25731:0.0):5.5E-4)0.963.48:5.5E-
4,(((Ult25720:0.0,Ult25738:0.0):5.4E-
4,Ult25734:0.03156)0.911.63:0.0072,Ult25722:0.26684)0.521.2:5.4E-
4)0.788.34:0.00378,(Ult25721:0.00629,(Ult25733:0.02646,(UltVeil8:0.00376,
Ult25732:0.00375)0.995.54:5.5E-
4)0.893.55:0.01048)0.784.37:0.006)0.903.49:0.0137)0.816.31:0.01126)0.927.
50:0.02622)0.785.42:0.00847,Ult25760:5.4E-
4)0.873.49:0.01085,Ult25752:5.4E-4)0.693.10:6.6E-
4)0.989.44:0.03938)0.328.7:0.00767)0.932.56:0.02799,AetLongu:0.04302)0.88
4.55:0.01943)0.760.38:0.00378)0.873.50:0.01252)0.604.8:0.00457,AprHongk:0
.03608)0.742.21:0.00373,((Ult25714:0.04021,LowGC003:0.02599)0.739.27:0.00
99,(Ult25711:0.01417,(Ult25716:0.00752,(UltVeil7:5.5E-4,(Ult25717:5.5E-
4,AmsAcida:0.00375)0.715.11:5.5E-4)0.973.28:5.5E-
4)0.988.51:0.04239)0.959.51:0.03399)0.923.74:0.02329)0.884.56:0.01632)0.8
60.36:0.01653)0.812.25:0.01044,(PppHippe:0.00733,PppVibri:5.4E-
4)0.993.34:0.05494)0.787.32:0.00819,((Ult23777:0.05011,Ult25648:0.01128)0
.992.47:0.05653,(Ult25675:0.12875,((((UltEu105:0.00386,SmsSputi:0.00372)0
.921.61:0.02226,SmsSput2:0.02056)0.873.51:0.01493,((((((UltRu530:5.5E-
4,(UltRu529:0.0038,Ult25659:0.01144)0.783.46:0.00372)0.952.48:0.01139,(((
SmsRumi4:0.00416,(SmsRumi3:0.03978,UltRu532:0.01064)0.235.8:0.00545)0.958
.57:0.01913,Ult25656:5.3E-4)1.000.324:5.5E-
4,Ult25655:0.00367)0.934.48:0.01141)0.995.55:5.5E-
4,Ult25650:0.01945)0.854.39:0.01518,UltSele3:0.02792)0.814.28:0.0088,(Vll

Bacte:0.0,Ult25658:0.0):0.00391)0.897.43:0.00751,(UltRu531:0.01132,((Ult2
5653:0.0,Ult25654:0.0,Ult25652:0.0):0.01135,((((SmsSpec3:5.5E-
4,((Ult25649:5.4E-
4,((PpnArbor:0.0,ZmpRaffi:0.0):0.0179,(Ult25693:0.01931,AniGlyce:0.01206)
0.865.53:0.01296)0.857.54:0.01215)0.933.55:0.00754,Ult25691:0.04376)0.286
.4:5.4E-
4)0.936.51:0.01158,((UltSele2:0.03114,(((PcnHaika:0.01485,PcnPorta:0.0100
7)0.873.52:0.01046,((Ult25680:0.00375,Ult25681:5.4E-
4)0.889.50:0.00761,(((Ult25688:0.0,Ult25689:0.0,Ult25682:0.0,MgmFunif:0.0
,Ult25685:0.0):5.5E-
4,(Ult25684:0.00755,Ult25687:0.044)0.756.45:0.00377)0.153.5:5.5E-
4,MgmRupel:0.01137)0.916.56:0.00759)0.742.22:0.00392)0.898.62:0.01172,((U
lt25679:5.5E-4,(Ult31807:0.00764,MgmHyper:0.00752)0.879.53:5.5E-
4)0.997.50:5.5E-4,(MgmHype2:5.4E-
4,Ult25683:0.00375)0.812.26:0.01135)0.743.36:0.01171)0.988.52:0.03625)0.8
89.51:0.01362,(Ult25670:0.00771,(((((Ult25668:0.01506,(Ult25666:0.00378,U
lt25664:5.5E-4)0.923.75:5.5E-
4)0.865.54:0.00376,Ult25663:0.00376)0.561.8:5.5E-
4,(Ult25667:0.0,Ult25669:0.0):5.5E-4)0.548.6:5.4E-
4,Ult25665:0.02305)0.886.64:0.00755,(Ult25662:0.01915,(Ult25660:0.00748,U
lt25661:5.3E-
4)0.001.2:0.00389)0.772.45:0.00405)0.703.12:0.00389)0.815.28:0.00809)0.86
1.55:0.00841)0.834.37:0.01007,((((ArvLipol:0.0039,UdnRum38:0.00365)0.886.
65:0.0182,(Ult23778:0.11384,(UncUn206:0.02765,(UltVeil6:0.0,Otu00323:0.0)
:0.02068)0.995.56:0.07089)0.399.8:0.01532)0.283.2:0.01055,(Ult25651:5.5E-
4,((SmsRumi2:0.0,UltRu528:0.0,SmsRumi6:0.0):5.5E-
4,(SmsRumin:0.00407,SmsRumi5:0.00752)0.500.5:5.5E-4)0.171.5:5.5E-
4)0.868.42:0.0099)0.863.49:0.01604,(Ult25672:5.5E-
4,Ult25692:0.04825)0.922.49:0.01936)0.780.50:0.00376)0.845.43:0.00999,(Ul
t25657:0.01534,SmsBovis:0.00374)0.784.38:0.00201)0.915.46:0.00217)0.217.4
:5.4E-4)0.912.61:5.4E-4)0.929.53:0.01583)0.773.39:5.5E-
4,((SlkSpeci:0.06316,((TsnCarbo:0.00518,VllBact3:0.01027)0.965.46:0.04557
,(UltRu534:0.01584,(UltRu533:0.02667,UltRu535:0.04541)0.964.52:0.04455)0.
993.35:0.05475)0.817.21:0.02508)0.842.51:0.01532,(UltRu539:0.03516,((Ult2
5690:0.0264,((ScwSucci:5.4E-
4,(Ult25671:0.03682,Ult25676:0.01153)0.635.2:0.01179)0.889.52:0.01799,(Ul
tSele7:0.041,((UltRu537:0.02404,(UltRu536:0.00375,UltRu538:5.5E-
4)0.792.43:0.01169)0.985.43:0.06207,Ult25678:0.03548)0.913.52:0.03164)0.8
42.52:0.03149)0.851.48:0.01223)0.901.55:0.01552,(SmsSpeci:0.01787,(((SmsA
rtem:0.0198,(UltSele4:0.00747,(SmsFlueg:0.00757,(UltSele5:5.5E-
4,(UltSele6:5.5E-4,(Ult25673:0.00753,Ult25674:0.00753)0.666.10:5.5E-
4)0.000.431:5.5E-4)0.914.51:5.5E-4)0.380.6:5.4E-
4)0.797.32:0.00381)0.794.36:0.00369,SmsGenom:5.4E-
4)0.800.31:0.00372,(SmsNoxia:0.0,SmsSpec2:0.0):5.3E-
4)0.873.53:0.01418)0.782.46:0.01717)0.738.17:0.00464)0.920.63:0.01695)0.7
65.27:0.00668)0.876.44:0.02871)0.241.4:5.4E-
4)0.805.34:0.01298)0.995.57:0.05738)0.925.59:0.02442)0.803.30:0.01006)0.9
16.57:0.02221)0.788.35:0.01488,UltrdL13:0.12492)0.828.32:0.02014,(((AkbSa
cch:0.02383,PeaBacte:0.08347)0.739.28:0.00772,((((UltCl393:0.01829,UltEu1
04:0.08767)0.871.51:0.02191,(UltCl392:0.01597,(Ult24407:0.02026,Ult24406:
0.04672)0.932.57:0.03097)0.783.47:0.02584)0.954.52:0.0557,((Ult24404:0.01
08,(ArfSterc:5.4E-4,UltEu103:0.05242)0.752.34:0.00423)0.000.432:5.5E-
4,(Ult21893:0.03046,Ult24405:0.06743)0.184.1:0.00986)0.897.44:0.02554)0.9
42.49:0.03784,Ult24408:0.044)0.969.38:0.04566)0.779.42:0.00907,((((Ult243

90:0.04112,(EubAggre:5.5E-4,(PmbAlact:5.5E-
4,Ult24394:0.00741)0.986.34:0.01892)0.902.63:0.01193)0.105.6:0.00346,Ult2
4393:0.00523)0.840.49:0.00583,((Ult24392:0.03293,(((Ult18246:0.0,Ult24397
:0.0):5.4E-4,(BacNL371:5.5E-
4,((BacNL366:0.00216,BacNL370:0.00733)1.000.325:0.00143,((Ult24387:0.0036
7,((((BacNL363:0.0,Ult24386:0.0,BacNL367:0.0,BacNL373:0.0,Ult24389:0.0,Ul
t24395:0.0):5.5E-
4,(BacNL364:0.00753,(BacNL369:0.00753,Ult24388:0.02265)0.785.43:0.00368)0
.423.7:5.5E-4)0.196.5:5.5E-4,BacNL365:0.00369)0.093.4:5.5E-
4,BacNL372:5.5E-4)0.048.2:5.5E-4)0.095.3:5.5E-4,BacNL368:5.5E-
4)0.704.9:5.5E-4)0.916.58:5.5E-
4)0.865.55:0.00697)0.212.6:0.00361,Ult18247:0.00378)0.912.62:0.01478)0.59
4.6:0.01626,((Ult24401:0.05332,Ult24402:0.05192)0.997.51:0.07477,(UltAce1
2:0.01126,(((AtmWoodi:0.00741,UltBani4:0.0074)0.608.10:5.5E-
4,(Ult24396:5.5E-4,UltBani2:0.0558)0.858.45:5.5E-4)0.000.433:5.5E-
4,((((AtmPalud:5.4E-
4,AtmBakii:0.02687)0.914.52:0.01473,(UltAce11:0.04779,AtmFimet:5.4E-
4)0.913.53:0.00738)0.917.53:5.4E-4,UltAce13:5.5E-4)0.605.6:5.5E-
4,(AtmCarbi:0.0,Ult24398:0.0,Ult24403:0.0):5.5E-4)0.454.9:5.4E-
4)0.943.48:5.4E-
4)0.905.44:0.01711)0.198.9:0.0083)0.690.13:0.00912)0.745.35:0.02533,(UltO
r293:0.1338,(((PpcNiger:5.4E-
4,(UltEu107:0.01066,Ult25798:0.01712)1.000.326:0.09414)0.873.54:0.01253,(
Ult25795:0.01134,(Ult25796:0.0,Ult25797:0.0):0.0076)0.930.47:0.02269)0.90
1.56:0.02012,((Ult25799:0.04416,(UltCl342:0.15235,Ult25800:0.00889)0.972.
37:0.06333)0.568.4:0.01118,(Ult25805:0.04455,(Ult18256:0.01435,(Ult25801:
0.02753,(Ult25802:0.03126,(Ult25803:0.0,Ult25804:0.0):5.5E-
4)0.731.16:0.00341)0.944.40:0.0191)0.720.11:0.00527)0.973.29:0.04295)0.91
3.54:0.03049)0.821.27:0.02521)0.949.52:0.05164)0.717.17:0.02071)0.953.34:
0.0342)0.855.52:0.01767)0.815.29:0.01227,((((UltB5109:0.0235,(UltB5104:0.
07155,((UltB5105:5.5E-4,UltB5106:5.5E-
4)0.987.47:0.04432,(UltB5107:0.02144,UltB5108:0.00983)0.926.61:0.02523)0.
841.39:0.01955)0.854.40:0.01171)0.970.42:0.02475,(((UltB5111:0.03853,(Ult
20640:0.06766,UltSelen:0.02261)0.925.60:0.02701)0.911.64:0.03388,UltRum32
:0.03955)0.842.53:0.02734,UltFirm2:0.07919)0.886.66:0.02525)0.608.11:0.00
504,(UltB5110:0.07136,((UltB5097:0.00369,(UltB5098:0.0,UltB5099:0.0):5.5E
-4)0.639.3:5.4E-4,(((((UltB5114:0.0,UltB5115:0.0,UltB5116:0.0):5.5E-
4,((UltB5096:0.00751,(UltB5094:0.01119,(UltB5095:5.5E-
4,(UltB5100:0.00369,UltB5103:0.01149)1.000.327:5.5E-4)0.498.5:5.5E-
4)0.432.6:5.4E-
4)0.912.63:0.00754,(UltB5113:0.0021,UltB5101:0.01087)0.936.52:0.00192)0.5
65.5:5.5E-4)0.629.10:5.4E-
4,UltB5117:0.00739)0.827.34:0.00373,(UltB5092:0.0,UltB5112:0.0):5.5E-
4)0.858.46:0.00739,((UltSp250:0.01145,UltB5102:0.02763)0.893.56:0.01124,U
ltB5093:0.00774)0.842.54:0.01133)0.305.3:5.3E-
4)0.908.52:0.01767)0.566.4:0.01058)1.000.328:0.13187,(((((((((((Ult14811
:5.5E-
4,Ult14812:0.00367)0.962.54:0.01937,(UltAc256:0.01473,(Ult14807:0.00368,(
(((Ult14809:0.0,Ult14810:0.0):5.5E-4,UltAc255:5.5E-4)0.000.434:5.5E-
4,Ult14806:0.00367)0.516.2:5.5E-4,Ult14808:0.00367)0.380.7:5.5E-
4)0.904.51:5.5E-4)0.764.41:0.00737)0.908.53:0.01674,((Ult13550:5.5E-
4,Ult14820:0.00369)0.998.65:0.04881,((UltAc254:0.0041,Ult14805:0.00707)0.
997.52:0.03605,(UltAc252:0.00388,(UltAc251:0.00762,UltAc253:0.00748)0.775
.39:0.00373)0.784.39:0.0039)0.909.41:0.01507)0.741.26:0.00596)0.797.33:0.

01423,(Ult14813:0.023,((UltAc258:0.05075,(UltAc257:0.05096,(Ult14816:0.00
577,(UltB4246:0.0268,(Ult14817:0.00365,(Ult14818:0.0,Ult14819:0.0):5.5E-
4)0.941.51:5.4E-
4)0.787.33:0.00544)0.993.36:0.08105)0.716.12:0.04165)0.832.32:0.02355,(Ul
t14814:0.05493,Ult14815:0.04657)0.773.40:0.02166)0.344.5:0.01541)0.917.54
:0.03013)0.916.59:0.03535,(((Ult14747:0.0516,(((Ult14466:0.0,Ult14468:0.0
):5.4E-4,(((Ult14469:0.0,Ult16028:0.0):0.00745,Ult14470:5.4E-
4)0.773.41:0.00392,(((((Ult14584:0.02996,(BacEll23:0.01148,(Ult14510:0.00
751,(Ult14499:0.01522,Ult14511:0.01516)0.507.4:0.01517)0.934.49:5.5E-
4)0.881.54:0.01303)0.881.55:0.02779,((((((BacEll25:0.0,Ult14514:0.0):5.5E
-4,Ult14543:0.00369)0.929.54:5.3E-4,((Ult14537:5.4E-
4,(UltEub34:0.00371,UltAc179:0.00368)0.896.39:0.00745)0.904.52:0.00747,((
((Ult14516:0.0,Ult14519:0.0,Ult14520:0.0):5.5E-
4,(Ult14517:0.01124,((Ult14515:0.00745,Ult14518:0.00372)0.813.33:0.00364,
(Ult14529:0.00369,Ult14531:5.5E-4)1.000.329:5.5E-
4)0.130.4:0.0037)0.917.55:5.5E-
4)0.843.35:0.00716,(Ult14538:0.01124,((((Ult14542:5.4E-
4,Ult14541:0.01501)0.942.50:0.01174,UltAc238:0.00327)0.765.28:0.00397,Bac
tK5b2:0.0273)0.929.55:0.01555,(Ult14528:0.00743,(Ult14530:0.00751,(Ult145
27:0.00369,(UltrS199:5.5E-4,((Ult14526:0.0,Ult14534:0.0):5.5E-
4,(Ult14525:0.00369,Ult14475:0.0074)0.916.60:5.5E-4)0.000.435:5.5E-
4)0.870.33:5.5E-
4)0.772.46:0.00371)0.803.31:0.00378)0.813.34:0.00761)0.841.40:0.00754)0.9
17.56:5.4E-4)0.959.52:0.01109,((Ult14521:5.5E-
4,(Ult14523:0.00744,Ult14522:0.00743)0.800.32:5.5E-4)0.897.45:5.5E-
4,(UltAc237:5.5E-4,Ult14524:0.00745)0.841.41:0.00369)0.241.5:5.4E-
4)0.940.50:0.01128)0.785.44:0.00366)0.870.34:0.00747,(((((((UltAc176:0.00
369,Ult14720:0.0151)0.904.53:0.00747,Ult14536:5.4E-
4)0.927.51:0.00198,(Ult14502:0.0,Ult14503:0.0):0.00202)0.919.58:0.01101,U
ltAc166:0.02716)0.741.27:0.00377,(Ult14504:5.4E-
4,((UltAc175:0.00743,((BacEll24:0.0,Ult14501:0.0):5.4E-4,(Ult14500:5.4E-
4,UltAc174:0.0037)0.921.62:0.00748)0.845.44:0.00369)0.010.3:5.5E-
4,UltHol15:0.00369)0.920.64:0.00741)0.723.19:5.4E-
4)0.748.31:0.00366,Ult14505:0.01134)0.908.54:0.01133,(UltB4198:0.0037,Ult
Ac177:5.5E-
4)0.856.36:0.00768)0.899.55:0.01163)0.889.53:0.00755,(Ult14540:5.3E-
4,(Ult14513:0.01519,(Ult14539:5.5E-
4,UltAc178:0.0074)0.861.56:0.00756)0.367.9:0.00719)0.944.41:0.01492)1.000
.330:5.4E-4,(Ult14533:5.4E-
4,(Ult14507:0.00377,(UltPro69:0.02752,((Ult14476:0.0,Ult14676:0.0):0.0077
,(Ult14508:0.01918,Ult14512:0.02312)0.881.56:0.01119)0.762.51:0.00324)0.9
07.54:0.01153)0.790.41:0.01142)0.835.42:0.00307)0.768.30:0.00495)0.779.43
:0.00625,Ult14532:0.00697)0.891.49:0.00821,((Ult14495:0.0,Ult14496:0.0,Ul
t14498:0.0,UltAc172:0.0):5.5E-
4,Ult14497:0.00368)1.000.331:0.05161)0.770.58:0.00395,(((UltAc206:0.01001
,(UltAci45:0.01187,Ult14583:0.0189)0.730.19:0.00935)0.984.30:0.03946,((((
((UltEub30:0.01161,Ult14370:0.01136)0.847.55:0.00739,((((BacEll19:0.0,Ult
Eub31:0.0,Ult14368:0.0):5.5E-4,(UltFor57:5.5E-
4,Ult14374:0.00742)0.849.41:0.0037)0.789.33:0.00369,(Ult14371:0.01132,Ult
14372:0.00744)0.208.4:5.5E-4)0.700.20:0.00359,(Ult14369:5.5E-
4,Ult14373:0.00741)0.903.50:0.00772)0.767.45:0.0039)0.305.4:0.0038,(((Ult
14359:5.5E-
4,(Ult14355:0.0037,(Ult14360:0.00774,((((Ult14356:0.00512,Ult14358:0.0136
8)0.213.5:0.00372,(Ult14357:0.00383,((UltOr240:0.11161,(UltOr241:0.0,AdcC

aps2:0.0):5.5E-4)0.336.6:5.4E-
4,AdcCapsu:0.01111)0.982.29:0.01891)0.809.38:0.00624)0.879.54:0.00873,Ult
For55:0.00388)0.841.42:0.00727,(Ult14348:0.00372,(Ult14347:5.5E-
4,Ult14382:0.00375)0.794.37:0.00375)0.943.49:0.01549)0.924.50:0.01143)0.1
06.3:5.5E-
4)0.847.56:0.0039)0.741.28:0.00361,(Ult14366:0.07317,(Ult14367:0.00369,((
((((((Ult14459:0.01126,(TrgRoseu:5.5E-4,UltAc163:0.00369)0.920.65:5.5E-
4)0.883.59:0.00743,(Ult14324:0.00743,(TrgSpec2:0.0,TrgSpeci:0.0):5.5E-
4)0.807.28:5.3E-4)0.957.42:0.01514,(AdbBac10:0.00369,AdbBact9:5.5E-
4)0.881.57:0.00748)0.759.45:0.00368,Ult14458:5.5E-
4)0.968.38:0.01514,(((UltrS192:0.00747,Ult14437:0.00751)0.861.57:0.00749,
((((Ult14395:0.00384,(Ult14396:0.0,Ult31245:0.0):0.01502)0.533.5:0.00748,
(Ult14403:5.4E-4,Ult14323:0.0037)1.000.332:5.5E-
4)0.930.48:0.00355,Ult14406:0.00369)0.526.4:5.4E-
4,(UltrS193:0.00372,(Ult14407:0.00372,(AdrBact2:5.4E-
4,(UltrS194:0.01122,(UltEdaph:5.5E-4,Ult14408:0.0074)0.957.43:5.3E-
4)0.938.38:0.00746)0.783.48:0.0037)0.787.34:0.00372)0.779.44:0.00372)0.78
8.36:0.00373)0.878.49:0.00756,((Ult14414:0.00741,((Ult14444:0.0075,((Ult1
4420:0.00371,(Ult14401:5.5E-
4,((UltAc161:0.00372,((Ult14427:0.00371,Ult14428:0.00371)0.774.55:0.00372
,(Ult14429:5.3E-
4,(Ult14399:0.00745,Ult14400:0.01126)0.796.24:0.00371)0.911.65:0.00749)0.
792.44:0.00372)0.791.31:0.00369,Ult14404:0.01124)0.709.11:5.3E-
4)0.792.45:0.0037)0.909.42:0.00201,((((Ult14446:0.02251,(Ult14445:5.5E-
4,Ult14447:5.5E-4)0.997.53:5.5E-
4)0.904.54:0.00736,(AdrBact4:0.00369,Ult14448:5.5E-4)0.892.44:5.5E-
4)0.753.37:5.5E-
4,(((((Ult14393:0.0,Ult14394:0.0,Ult14431:0.0,Ult14432:0.0,Ult14433:0.0):
5.4E-4,(Ult14425:0.00369,Ult14430:5.5E-
4)0.905.45:0.00735)0.391.9:0.01118,(((Ult14421:0.00369,(Ult14423:0.0,Ult1
4424:0.0):5.5E-4)0.992.48:5.4E-
4,((Ult14422:0.00741,Ult14443:0.01133)0.373.6:5.4E-
4,UltrS196:0.02318)0.827.35:0.00747)0.824.33:0.0037,((BacEll21:0.0,EdaMod
es:0.0,EdaAggre:0.0,AdrBact3:0.0):5.5E-4,Ult14426:0.00369)0.349.4:5.5E-
4)0.898.63:0.00742)0.996.42:5.5E-4,(Ult14398:5.5E-
4,Ult14441:0.0074)0.231.5:0.00369)0.915.47:0.01092,((UltrS197:0.00774,(Ad
bBact7:0.00718,(Ult14456:5.5E-4,Ult14457:0.00369)0.901.57:5.5E-
4)0.571.5:0.00749)0.846.46:0.00698,(Ult14435:0.0113,(Ult14434:0.0037,Ult1
4436:0.00369)0.808.34:0.00373)0.815.30:5.4E-
4)0.817.22:0.00696)0.875.56:5.5E-
4)0.794.38:0.00741,(((BacEll20:0.00371,(Ult14413:5.5E-
4,((UltrS195:0.0,Ult14412:0.0,UltEdap2:0.0,Ult14418:0.0):0.0037,Ult14416:
0.00741)0.633.5:5.5E-4)0.832.33:0.00369)0.762.52:5.5E-4,Ult14415:5.5E-
4)0.923.76:5.5E-
4,(((Ult14451:0.01957,Ult14454:0.00743)0.966.57:0.01948,Ult14453:5.5E-
4)0.915.48:0.01117,(UltrS198:0.01108,(AdbBact8:0.00724,((Ult14439:0.0,Ult
14442:0.0):5.5E-4,(UltAct20:0.00369,Ult14440:0.00369)0.059.6:5.5E-
4)0.922.50:5.4E-
4)0.673.4:0.00744)0.898.64:0.00778)0.783.49:0.00366)0.796.25:0.00385)0.88
8.55:0.00196)0.851.49:0.00667)0.875.57:5.5E-
4,((((((Ult14450:0.0037,UltEub33:0.00744)0.483.11:5.4E-
4,(Ult14452:0.0077,Ult14455:0.01308)0.897.46:0.01307)0.954.53:0.01117,Ult
14419:5.4E-
4)0.769.39:0.00354,AdbBact6:0.00762)0.960.46:0.01893,(Ult14397:0.01132,Ul

t26343:0.00371)0.880.57:0.01098)0.679.12:5.4E-4,((((Ult14411:5.4E-
4,Ult14417:0.01882)0.911.66:0.00743,(Ult14402:5.4E-
4,AdbBact5:0.035)0.832.34:0.00745)0.846.47:0.00328,(Ult14409:0.01123,Ult1
4410:0.01123)1.000.333:5.4E-
4)0.113.7:0.00748,Ult14405:0.01097)0.884.57:0.00809)0.171.6:5.5E-
4)0.989.45:0.01488)0.002.2:5.4E-4,(((Ult14438:0.0,UltAc162:0.0):5.5E-
4,Ult14449:0.00373)0.987.48:0.01511,UltEub32:0.00372)0.771.49:0.00366)0.9
56.43:0.01122)0.780.51:0.00364)0.782.47:0.00367,(Ult14321:0.01106,UltAc16
0:0.03079)0.894.43:5.4E-4)0.958.58:0.01492,Ult14365:0.00369)0.535.6:5.5E-
4,Ult14364:5.5E-4)0.928.46:5.5E-4,(((Ult14354:5.5E-
4,((Ult14351:0.0,Ult14352:0.0):5.5E-4,Ult14353:5.5E-
4)0.956.44:0.00741)0.945.50:5.3E-4,((Ult14377:5.5E-
4,(UltAc159:0.00745,((UltAc158:0.0,UltAc157:0.0,UltFor58:0.0):5.5E-
4,(Ult14378:0.00369,Ult14379:0.00369)0.695.11:5.5E-4)0.814.29:5.5E-
4)0.839.39:0.0037)0.835.43:5.3E-
4,(Ult14380:0.00363,AdrBacte:0.00377)0.900.44:0.01127)0.989.46:0.02284)0.
809.39:0.00363,((Ult14296:0.0,Ult14297:0.0):0.01156,Ult14460:0.01934)0.74
9.47:0.00353)0.901.58:0.00752)0.790.42:0.00363)0.982.30:5.5E-
4)0.898.65:0.00758)0.872.57:0.00785,((Ult14376:0.0037,(Ult14362:0.0153,(U
lt14363:0.00369,Ult14361:5.5E-4)0.975.40:5.5E-
4)0.836.32:0.00369)0.855.53:5.4E-
4,(AdbBact4:0.01896,(UltFor56:0.00708,UltFor59:5.5E-
4)0.235.9:0.01515)0.914.53:0.01194)0.979.37:0.01901)0.860.37:5.4E-
4)0.778.43:5.4E-
4,((Ult14375:0.00753,UltAc155:0.00371)0.839.40:0.00349,(((Ult14309:0.0129
6,(Ult14308:5.4E-4,((UltFor62:0.05162,(Ult14311:0.0,Ult14312:0.0):5.4E-
4)0.806.24:0.00358,(UltFor50:0.00362,(UltFor51:0.0037,(Ult14307:0.0,UltAc
164:0.0):0.0037)1.000.334:5.3E-4)0.806.25:0.00746)1.000.335:5.4E-
4)0.844.61:0.0098)0.344.6:0.0067,(Ult14313:5.5E-
4,Ult14314:0.01124)0.771.50:0.00562)0.778.44:0.01296,(Ult14349:0.00698,Ul
t14350:0.03675)0.974.23:0.03128)0.272.5:0.01086)0.998.66:5.3E-
4)0.800.33:0.00815,(((Ult14327:0.02717,Ult14328:0.00377)0.952.49:0.01501,
((Ult14300:0.00375,(Ult14344:0.01375,(((Ult14320:0.0075,UltFor61:0.01927)
0.907.55:0.01139,Ult14345:5.5E-4)0.808.35:0.00759,(BacEnr39:5.5E-
4,(Ult14317:0.00594,BacEll18:0.00589)0.950.37:0.01455)0.821.28:0.00884)0.
489.3:0.006)0.874.69:0.00901)0.857.55:0.00759,((Ult14294:0.0,Ult14295:0.0
):0.00727,(Ult14292:0.00744,Ult14293:5.5E-4)0.901.59:5.5E-
4)0.879.55:0.01148)0.948.55:0.015)0.847.57:5.4E-4,(Ult14346:5.5E-
4,((((Ult14298:5.5E-4,((((Ult14306:5.4E-
4,((((Ult14333:0.0037,(UltAc151:5.5E-
4,(Ult14336:0.00369,Ult14334:0.00369)0.644.4:5.5E-4)0.799.38:5.5E-
4)0.923.77:0.00744,Ult14332:0.00369)0.713.16:5.4E-
4,(ZnBrBBrB:0.0,Ult14329:0.0,Ult14330:0.0,Ult14331:0.0):5.5E-
4)0.954.54:5.5E-4,(((Ult14290:0.00749,Ult14338:5.5E-4)0.750.23:5.5E-
4,(((UltrS191:5.5E-4,Ult14340:0.00742)0.912.64:5.5E-
4,((((UltAc152:0.0,Ult14335:0.0):5.5E-
4,Ult14318:0.00741)0.796.26:0.0037,Ult14337:0.00372)0.818.26:0.00367,(Ult
14289:0.0,Ult14339:0.0):5.4E-
4)0.859.45:0.00738)0.953.35:0.01114,(Ult14461:0.01123,UltFor60:5.3E-
4)0.928.47:0.0112)0.911.67:5.4E-4)0.400.6:5.4E-
4,UltRho45:0.00368)0.853.39:0.00367)0.904.55:0.00711)0.821.29:0.01096,((B
acK5b10:0.00743,((UltFor48:0.0112,Ult14304:0.01136)0.251.5:5.5E-
4,((Ult14315:0.00711,(UltrS190:0.00333,(Ult14305:5.5E-
4,UltAc150:0.0037)0.801.35:0.0041)0.751.21:0.0075)0.901.60:5.4E-

4,(Ult14316:5.4E-4,(((UltFor53:0.0074,(Ult14303:0.00369,((Ult14301:5.5E-
4,UltFor49:0.0074)0.815.31:0.00372,(UltFor52:0.00374,Ult14299:0.0113)0.77
6.42:0.0037)0.767.46:0.00372)0.360.8:5.4E-4)0.272.6:5.5E-
4,Ult14302:0.00369)0.888.56:0.0075,Ult14322:0.00749)0.791.32:0.00359)0.87
8.50:0.00743)0.750.24:5.5E-4)0.849.42:0.0037)0.755.33:5.3E-
4,(Ult14291:0.0,UltAc149:0.0,UltEub29:0.0):5.4E-4)0.977.38:5.4E-
4)0.899.56:0.01133,AdbBact3:0.0155)0.940.51:0.01558,BacEnr38:0.00376)0.94
4.42:0.01513)0.830.35:0.00368,UltAc156:0.00371)0.914.54:5.0E-
4,((UltPro68:0.00741,(BacEll17:5.5E-4,Ult14319:5.5E-4)0.866.47:5.5E-
4)0.909.43:0.01552,(Ult14386:0.00717,(Ult14387:0.0074,((Ult14383:0.0,Ult1
4384:0.0):5.5E-4,Ult14385:0.00378)0.303.4:5.5E-4)0.942.51:5.5E-
4)0.989.47:0.0328)0.843.36:0.01125)0.937.45:0.01521,UltAc154:0.01126)0.24
9.4:5.5E-
4)0.924.51:0.00767)0.839.41:0.00744)0.969.39:0.03132,(Ult14494:0.0032,(Ba
cEll22:0.00365,Ult14493:5.4E-
4)0.981.37:0.03196)0.952.50:0.02954)0.787.35:0.01132)0.743.37:0.00485,(((
((Ult14743:0.0146,((UltAc199:0.0,Ult14568:0.0):0.0112,UltAc204:5.4E-
4)0.238.4:0.00427)0.655.16:0.00764,Ult14567:0.00427)0.826.35:0.00685,(((U
lt14575:0.02646,(((UltrS201:0.00378,Ult14577:0.01525)0.790.43:0.00367,Ult
14574:0.01517)0.068.4:5.4E-4,(Ult14573:5.5E-
4,UltAc205:0.00741)0.968.39:0.01124)0.977.39:0.00283)1.000.336:9.0E-
4,(Ult14582:0.02314,(UltAc168:5.4E-
4,((Ult14479:0.0,Ult14480:0.0):0.01141,(Ult14478:0.0,UltAc167:0.0):0.0152
3)0.944.43:0.01526)0.911.68:0.01164)0.858.47:0.01138)0.811.29:0.00394,(Ul
t14549:0.00371,((Ult14572:0.01124,((((((UltFor64:0.0,Ult14570:0.0):5.4E-
4,UltAc198:0.01125)0.246.7:5.4E-
4,((UltAc234:0.01499,(UltAc180:0.0,UltAc201:0.0,UltAc203:0.0):5.5E-
4)0.946.47:0.00745,Ult14562:5.4E-
4)0.941.52:0.01127)0.834.38:0.01124,(Ult14571:0.01478,(((UltAc211:0.0,Ult
14592:0.0):5.5E-4,(Ult14594:0.00369,((UltrS200:0.00388,((((Ult14596:5.5E-
4,((Ult14593:0.01496,Ult14595:5.4E-
4)0.846.48:0.00722,(Ult14552:0.00369,((Ult14564:0.00369,(((UltAc196:5.5E-
4,((UltAc183:0.0,UltAc184:0.0,UltAc185:0.0,Ult14553:0.0,UltAc197:0.0):5.5
E-4,(UltAc188:0.0037,(UltAc209:0.00369,UltAc181:0.00382)0.000.436:5.5E-
4)0.572.5:5.5E-4)0.366.4:5.5E-4)0.156.6:5.5E-
4,UltAc193:0.00369)0.878.51:0.00381,Ult14550:0.00741)0.202.6:5.5E-
4)0.281.1:5.4E-
4,(UltAc182:0.0,UltAc186:0.0,UltAc187:0.0,Ult14563:0.0,UltAc194:0.0,Ult14
565:0.0,UltAc210:0.0,Ult14668:0.0,Ult16425:0.0):5.5E-4)0.525.5:5.5E-
4)0.773.42:0.00383)0.882.58:0.00775)0.819.31:0.00379,Ult14740:0.00741)0.1
14.2:5.5E-4,Ult14590:5.5E-
4)0.929.56:0.01127,Ult14667:0.01493)0.882.59:5.4E-
4)0.352.3:0.00382,(((UltAc212:0.0,Ult14591:0.0):5.5E-
4,(UltAc214:0.00745,Ult14556:0.00743)0.776.43:5.4E-4)0.889.54:5.4E-
4,((UltAc202:0.00372,((Ult14597:5.4E-
4,Ult14566:0.0037)0.821.30:0.0037,UltAc213:5.5E-
4)0.808.36:0.00371)0.786.46:0.00372,(UltAc200:0.00369,Ult14569:5.5E-
4)0.246.8:5.4E-4)0.870.35:0.0037)0.847.58:0.00352)1.000.337:5.4E-
4)0.193.3:5.4E-4)0.558.5:5.5E-4,UltrS202:0.01917)0.203.4:5.5E-
4)0.945.51:0.01141)0.741.29:5.3E-
4,((Ult14578:0.03481,UltrS204:0.02278)0.381.9:0.00841,(((((UltAc189:0.0,U
ltAc217:0.0):5.5E-4,Ult14598:5.5E-
4)0.838.51:0.00367,(UltAc236:0.00361,((Ult14636:0.02682,Ult14558:5.5E-
4)1.000.338:5.4E-4,(((Ult15347:0.01118,UltAc191:0.01537)0.917.57:5.5E-

4,Ult14557:5.5E-4)0.913.55:5.5E-
4,UltAc190:0.0037)0.886.67:0.00361)0.148.2:0.00369)0.881.58:5.5E-
4)0.926.62:0.00741,(UltFor63:0.00753,((Ult14555:0.0,UltAc192:0.0,Ult14561
:0.0):5.5E-4,Ult14560:0.0074)0.521.3:5.5E-
4)0.827.36:0.00369)0.001.3:5.3E-4,Ult14559:5.4E-4)0.922.51:5.3E-
4)0.886.68:0.00721)0.856.37:0.00374,(Ult14576:5.4E-4,(((Ult14641:5.4E-
4,((((Ult14714:0.0,UltAc231:0.0):5.4E-
4,((((Ult14669:0.0,UltAc228:0.0):5.4E-
4,((Ult14726:0.00369,((Ult14722:0.00369,Ult14712:0.01904)1.000.339:5.5E-
4,(Ult14723:0.0,Ult14724:0.0):5.5E-4)0.609.10:5.5E-4)0.729.17:5.5E-
4,Ult14715:0.00742)0.847.59:0.0037)0.787.36:0.00746,Ult14725:0.05519)0.19
1.4:0.00756,((Ult14716:0.01884,(UltHol16:0.00382,((Ult14551:5.5E-
4,UltAc195:0.01501)0.838.52:0.00714,(((Ult14719:5.4E-
4,(Ult14708:0.00539,(Ult14710:5.5E-
4,Ult14727:0.01723)0.478.5:0.00865)0.895.64:0.01002)0.922.52:0.00746,Ult1
4718:0.00369)0.970.43:5.5E-
4,Ult14717:0.00369)0.722.7:0.00375)0.790.44:0.00354)0.411.6:0.00365)0.877
.64:5.3E-4,((Ult14670:0.0074,Ult11743:0.01883)0.674.9:5.5E-
4,(UltAc229:0.0,UltAc230:0.0,Ult14713:0.0,UltAc232:0.0):5.5E-
4)0.929.57:5.4E-4)0.957.44:5.5E-
4)0.889.55:0.0071)0.800.34:0.0073,(Ult14554:0.01108,Ult14721:0.00367)1.00
0.340:5.5E-
4)0.779.45:0.00348,UltAc233:0.00756)0.941.53:0.01101)0.770.59:0.00361,((U
lt14600:0.00433,(Ult14599:0.00657,(Ult14601:0.00469,((((((((Ult14732:0.0
0369,((((Ult14733:0.01499,((((UltAc226:0.01534,((((UltFor66:0.0227,(UltFo
r67:5.5E-4,Ult14683:0.0037)0.685.10:5.4E-
4)0.833.43:0.00369,((UltAc221:0.0,UltAc223:0.0,Ult14737:0.0):5.3E-
4,((((UltPro46:0.02671,Ult14699:0.012)0.583.4:5.5E-
4,(UltAc219:0.0,Ult14689:0.0,UltFor69:0.0):5.5E-4)0.459.7:5.4E-
4,((Ult14544:0.00742,(Ult14666:0.0,UltAc224:0.0):0.0037)1.000.341:5.4E-
4,((((Ult14656:0.00369,((Ult14742:0.01496,(Ult14657:0.0,Ult14658:0.0):0.0
0369)0.640.5:5.5E-4,UltAc218:5.5E-4)0.877.65:5.5E-4)0.842.55:5.5E-
4,(Ult14650:0.0,Ult14651:0.0,Ult14652:0.0,Ult14659:0.0,Ult14660:0.0):0.00
369)0.920.66:0.00746,(Ult14681:0.00983,(UltAc215:0.02274,(Ult14621:0.0074
4,((Ult14618:0.0,Ult14623:0.0):5.5E-4,Ult14622:0.00369)0.865.56:5.5E-
4)0.921.63:0.01029)0.890.57:5.2E-4)0.903.51:0.00988)1.000.342:5.4E-
4,(UltFor70:5.0E-4,(((Ult14690:0.0,Ult14694:0.0):5.5E-
4,(UltAc227:0.00738,((Ult14691:0.0,Ult14692:0.0):0.00378,Ult14629:0.01517
)0.164.10:0.00371)0.917.58:5.5E-4)0.889.56:5.4E-
4,UltAc225:0.00369)0.928.48:0.00746)0.804.34:0.00359)0.152.2:0.0037)0.835
.44:0.00359)0.912.65:0.00792,((Ult14663:0.0,UltFor65:0.0):0.00738,(Ult146
96:0.01126,Ult14685:0.0112)1.000.343:5.4E-
4)0.778.45:0.01119)0.999.83:5.5E-4)0.432.7:5.5E-
4)0.866.48:0.00396,Ult14638:0.00744)0.359.4:5.4E-
4,(Ult14686:0.0,Ult14688:0.0,Ult14695:0.0):5.5E-4)0.659.8:5.5E-
4)0.471.9:0.00395,Ult14736:0.01119)0.136.3:5.4E-4,Ult14687:5.4E-
4)0.866.49:0.0016,(Ult14684:0.0,Ult14698:0.0):0.01464)0.875.58:0.01126)0.
473.6:5.4E-4,UltFor71:0.00369)0.419.5:5.5E-
4,(Ult14728:0.0,Ult14729:0.0):5.5E-4)0.437.6:5.3E-
4,Ult14734:0.00369)0.188.2:5.5E-
4)0.908.55:0.0079,(Ult14735:0.01125,((UltFor68:0.0,Ult14701:0.0):5.5E-
4,(Ult14680:0.0037,(Ult14664:0.00392,Ult14700:0.00713)0.923.78:5.5E-
4)0.385.9:5.5E-4)0.473.7:5.5E-4)0.136.4:5.4E-
4)0.982.31:0.0153,Ult14697:5.4E-

4)0.810.32:0.00371,Ult14665:0.00372)0.961.45:0.00503,(((Ult14661:0.00333,
(UltHol17:0.00369,(Ult14730:0.00369,Ult14731:5.5E-
4)0.778.46:0.00373)0.967.38:0.01561)0.156.7:0.00371,Ult14662:0.02264)0.85
9.46:5.3E-
4,((Ult14702:0.0,Ult14703:0.0):0.0036,(((Ult14706:0.00742,Ult14711:5.3E-
4)0.930.49:0.00747,Ult14709:0.00372)0.844.62:5.4E-
4,((Ult14671:0.01107,Ult14707:5.5E-
4)0.979.38:0.01975,(Ult14704:0.0,Ult14705:0.0):0.00713)0.233.5:0.00377)0.
958.59:0.01495)0.896.40:0.00761)0.961.46:0.00495)0.961.47:0.00497,((Ult14
653:0.0,Ult14655:0.0):5.5E-
4,((((Ult14631:0.0,UltAc216:0.0):0.00368,Ult14632:0.00374)0.900.45:0.0074
8,UltAc220:5.5E-4)0.859.47:0.00369,Ult14654:0.00741)0.811.30:5.5E-
4)0.600.7:5.4E-4)0.851.50:0.00372,Ult14693:0.01121)0.903.52:5.4E-
4,(Ult14682:0.01134,((Ult14674:0.00746,(Ult14741:5.5E-
4,((Ult14672:0.00372,Ult14679:0.0037)0.786.47:0.0037,(Ult14477:5.5E-
4,(Ult14673:0.00369,UltAc222:0.00369)0.690.14:5.5E-4)0.929.58:5.5E-
4)0.840.50:0.0037)0.867.50:0.00369)0.854.41:5.4E-
4,(Ult14677:0.0,Ult14678:0.0):5.4E-4)0.936.53:5.4E-
4)0.465.9:0.00369)0.912.66:0.00718,((((((Ult14647:0.0,Ult14648:0.0):0.003
57,Ult14649:0.00763)0.770.60:0.0037,(Ult14603:0.00781,Ult14675:0.01143)0.
853.40:0.0074)0.789.34:0.00395,(Ult14604:5.5E-
4,Ult14606:0.0037)0.959.53:0.01125)0.850.54:5.3E-
4,(((((Ult14642:0.00369,((Ult14607:5.5E-
4,Ult14646:0.00742)0.844.63:0.0037,(((Ult14535:0.02225,Ult14619:0.04345)0
.833.44:0.00888,Ult14611:5.4E-4)0.781.42:0.00373,(Ult14608:5.3E-
4,((((Ult14612:0.0,Ult14614:0.0,Ult14615:0.0):5.5E-
4,Ult14613:0.00369)0.959.54:0.01109,(Ult14634:0.0112,(Ult14635:5.5E-
4,Ult14639:0.01886)0.921.64:0.00748)0.202.7:5.4E-
4)0.420.12:0.00746,(Ult14624:0.0,Ult14625:0.0,Ult14626:0.0,Ult14627:0.0,U
lt14628:0.0,Ult14630:0.0):0.00357)0.799.39:0.00383)0.798.44:0.00371)0.796
.27:0.00371)0.266.3:5.5E-4)0.383.5:5.5E-4,UltrS203:5.5E-4)0.454.10:5.5E-
4,(Ult14610:0.0,Ult14616:0.0,Ult14637:0.0,Ult14643:0.0,Ult14644:0.0,Ult14
645:0.0):5.5E-4)0.445.11:5.5E-
4,((Ult16027:0.02301,Ult14602:0.00371)0.793.38:0.0037,((Ult14617:5.5E-
4,(Ult14506:5.5E-
4,Ult14620:0.00744)0.946.48:0.00747)0.916.61:0.0111,(Ult14605:0.0153,Ult1
4633:0.00385)0.043.5:5.4E-4)0.964.53:0.01534)0.155.7:5.5E-
4)0.857.56:0.0037,Ult14609:0.0037)0.914.55:5.5E-
4)0.814.30:0.00371,Ult14640:0.01125)0.929.59:0.00153)0.811.31:0.00142)0.8
33.45:0.0075)0.877.66:5.4E-
4)0.796.28:0.00383,(Ult14509:0.03927,(((Ult14481:0.0,Ult14483:0.0):5.5E-
4,(UltAc169:0.01131,Ult14484:0.00369)0.958.60:0.01525)0.925.61:0.00195,Ul
t14482:0.00212)0.829.40:0.00747)0.907.56:0.01165)0.757.31:0.0035)0.945.52
:0.01541,Ult15078:0.01491)0.668.10:5.5E-4)0.762.53:0.00378)0.736.24:5.5E-
4)0.300.6:5.4E-4,Ult14579:0.01897)0.895.65:0.00782)0.557.7:5.4E-
4)0.432.8:5.5E-
4)0.909.44:0.00777,((((Ult14587:0.01611,(Ult14462:0.00717,Ult14588:5.4E-
4)0.081.6:0.00631)0.906.69:0.01594,Ult14581:0.05576)0.778.47:0.00787,(((U
lt14463:0.03089,UltFor10:0.08917)0.921.65:0.02061,(((Ult16426:0.0154,(Ult
Ac235:0.00616,Ult14738:0.05102)0.986.35:0.04917)0.779.46:0.01681,UltPro79
:0.05211)0.974.24:0.03824,Ult15390:0.04222)0.751.22:7.9E-
4)0.762.54:0.00536,(Ult14464:0.06725,Ult14465:0.06581)0.914.56:0.03112)0.
953.36:0.03071)0.870.36:5.4E-4,(Ult14580:0.03519,(UltAc165:5.4E-
4,((Ult14471:0.0179,((Ult14472:5.5E-

4,Ult14473:0.00753)0.942.52:0.01625,(UltAc171:0.00359,(((((Ult14488:0.0,Ult14492:0.0):5.5E-4,Ult14491:0.00368)0.987.49:5.5E-
4,Ult14487:0.04347)0.889.57:0.00204,Ult14486:0.00697)0.908.56:0.00208,((UltAc170:0.0033,(Ult14485:0.00786,Ult14490:0.01091)0.377.7:0.0075)1.000.344:5.4E-4,Ult14489:5.5E-4)0.825.32:5.5E-
4)0.923.79:0.01132)0.956.45:0.019)0.858.48:0.01029)0.365.5:0.00557,Ult14589:0.03817)0.884.58:0.00851)0.722.8:0.00376)0.287.5:0.00753)0.893.57:0.0109)0.589.9:5.4E-
4,((UltAc173:0.0,UltAc207:0.0):0.0036,(UltAc208:0.01507,(Ult14585:0.00369,Ult14586:5.5E-
4)0.769.40:0.00369)0.762.55:0.00384)0.976.30:0.01524)0.905.46:0.01325)0.879.56:0.01036)0.855.54:0.00772)0.878.52:0.0074)0.815.32:0.00681,Ult14467:0.00439)0.926.63:0.01792)0.762.56:0.00566,(UltAc239:0.01923,(UltHol18:0.04931,(Ult14748:0.02424,Ult14749:0.01778)0.871.52:0.0124)0.890.58:0.01829)0.777.42:0.0087)0.873.55:0.00853,(((((Ult14266:0.07261,Ult14267:0.01337)0.989.48:0.06658,(Ult14264:0.01971,(Ult14265:0.04426,UltAc148:0.07107)0.736.25:0.0064)0.791.33:0.01346)0.975.41:0.03946,(Ult14745:0.00789,((Ult14285:5.5E-4,(Ult14286:5.5E-
4,Ult14287:0.02283)0.828.33:0.00368)0.919.59:0.01593,((Ult14273:0.0232,(UltAc143:5.4E-
4,UltAc144:0.01108)0.998.67:0.04819)0.855.55:0.01068,(UltAc145:0.02719,((Ult14283:5.5E-
4,(Ult14282:0.0037,((UltHolo9:0.00408,(Ult14268:0.01504,(UltHolo8:0.0,Ult14269:0.0,Ult14270:0.0):5.5E-
4)0.964.54:0.01821)0.926.64:0.00408,UltHol14:5.5E-4)0.621.8:5.4E-
4)0.957.45:0.00744)0.756.46:0.00362,(((Ult14281:0.03893,(UltAc146:0.01146,Ult14280:0.02701)0.653.5:0.00439)0.008.2:0.0037,(((UltAc142:0.01129,(UltFor46:0.00369,UltFor47:5.5E-
4)0.948.56:0.01524)0.921.66:0.00428,((Ult14271:0.02226,Ult14272:0.00954)0.706.15:0.01397,((Ult14275:0.02294,(Ult13814:5.4E-
4,Ult14279:0.01506)0.906.70:5.4E-
4)0.832.35:0.00368,(((UdnBac60:0.00369,(Ult14274:0.02762,Ult14278:0.00727)0.958.61:0.01977)0.722.9:0.00757,Ult14277:0.00737)0.909.45:5.5E-
4,(Ult14276:5.3E-
4,UltHol12:0.02301)0.911.69:0.00745)0.915.49:0.00757)0.917.59:5.5E-
4)0.972.38:0.02213)0.887.57:0.00378,((UltHol11:0.01499,(UltAc147:0.01512,UltHol13:0.00752)0.762.57:0.0036)0.866.50:5.5E-4,UltHol10:5.4E-
4)0.922.53:5.5E-
4)0.845.45:0.00694)0.888.57:0.00797,Ult14284:0.00379)0.301.3:0.00368)0.991.42:5.4E-
4)0.881.59:0.00803)0.577.3:0.00742)1.000.345:0.0569)0.598.4:0.00367)0.824.34:0.00663,Ult14746:0.01204)0.941.54:0.01751,(((UltAci77:0.05519,UltAci76:0.03427)0.846.49:0.02577,(UltAci78:0.04783,((((((((((((Ult13866:0.01109,(UltAci87:0.00389,Ult13955:0.01137)0.893.58:0.01116)0.268.3:5.5E-
4,(((UltAci90:0.00369,((Ult13898:5.5E-4,UltrS182:5.5E-
4)0.837.30:0.0037,(Ult13899:5.5E-4,UltAci93:0.0037)0.371.8:5.5E-
4)0.920.67:0.00751)0.116.5:5.5E-
4,(((Ult13887:0.00246,(Ult13889:0.00368,Ult13888:5.5E-
4)0.922.54:0.00245)0.922.55:0.00242,(Ult13903:0.00369,((Ult13895:0.0037,((Ult13892:0.0,Ult13893:0.0):5.5E-
4,UltAci92:0.00744)0.912.67:0.00752)0.843.37:5.5E-
4,(Ult13897:0.00369,(Ult13894:0.00369,UltPro65:5.5E-
4)0.863.50:0.0037)0.748.32:5.5E-4)0.759.46:5.3E-4)1.000.346:5.5E-
4)0.552.6:0.0037,(((((Ult13972:5.5E-

4,Ult13873:0.00754)0.830.36:0.00371,(Ult13900:0.0,UltrS183:0.0):0.0037)0.817.23:5.4E-4,Ult13874:5.5E-4)0.868.43:5.4E-
4,Ult13890:0.00746)0.922.56:0.00245,(Ult13875:0.00751,Ult13954:0.00374)0.922.57:0.00246)0.922.58:0.00242)1.000.347:5.5E-4)0.877.67:5.3E-
4,(((((Ult13870:0.00368,Ult13885:5.5E-
4)0.927.52:0.00233,((Ult13876:0.00369,Ult13877:5.5E-
4)0.790.45:0.00371,Ult13886:0.00371)0.926.65:0.00233)0.922.59:0.00318,(UltAci91:0.0034,((UltAci89:0.01102,UltAci99:0.00718)0.915.50:5.4E-
4,UltPro67:0.01127)0.951.48:5.5E-
4)0.656.8:0.00789)0.772.47:0.00338,(Ult13884:0.01121,UltOr238:5.4E-
4)0.825.33:0.00742)0.868.44:5.4E-4,((Ult13969:0.01148,((Ult13891:5.5E-
4,Ult13971:0.0037)0.816.32:5.4E-4,((Ult13872:0.00743,Ult13953:5.4E-
4)0.924.52:0.0115,Ult13908:0.01543)0.850.55:0.00751)0.786.48:0.00371)0.787.37:0.00372,(Ult13952:0.00415,(Ult13936:0.0159,(Ult13913:0.00373,(Ult13906:5.4E-
4,Ult13914:0.0153)0.795.39:0.00374)0.746.35:0.00317)0.864.57:0.00793)0.922.60:0.01123)0.231.6:5.4E-
4)0.861.58:0.0077)0.749.48:0.01183)0.269.3:0.00433,((Ult13871:0.00369,(((Ult13865:0.0025,(Ult13863:5.5E-
4,Ult13957:0.00369)0.922.61:0.00241)0.922.62:0.00242,(Ult13864:0.00366,Ult13933:5.3E-4)0.449.15:0.00369)1.000.348:5.5E-
4,(((Ult13862:0.00371,(((Ult13861:0.00744,Ult13968:0.00743)0.771.51:5.5E-
4,(Ult13860:0.00372,((((Ult13848:0.01126,Ult13867:5.5E-
4)0.858.49:0.0072,UltAci86:5.3E-4)0.896.41:0.01091,((Ult13854:5.4E-
4,(UltAci97:0.01502,((CanSolib:0.0,Ult13858:0.0):0.0037,Ult13935:0.00369)0.882.60:5.5E-
4)0.307.2:0.00369)0.788.37:0.00384,Ult13855:0.00734)0.085.2:0.00752)0.996.43:5.5E-4,Ult13934:5.5E-4)0.876.45:0.0037)0.846.50:5.5E-4)0.783.50:5.3E-
4,(Ult13868:0.00747,Ult13962:0.0037)0.807.29:0.0037)0.851.51:0.0037)0.312.9:5.5E-4,Ult13859:0.00369)0.896.42:5.5E-4,BacEll16:5.5E-4)0.889.58:5.4E-
4)0.848.48:0.00369)0.252.2:5.4E-
4,((Ult13911:0.00746,Ult13912:0.01137)0.912.68:0.01144,Ult13910:0.00368)0.793.39:0.00373)0.846.51:0.00716)0.444.10:0.00404,(Ult13909:5.5E-
4,Ult13999:0.02709)0.943.50:0.01116)0.883.60:5.4E-
4,(Ult13856:0.00371,Ult13857:0.01521)0.790.46:0.00381)0.924.53:0.00794,(Ult14001:0.0231,Ult14000:0.03147)0.913.56:5.3E-
4)0.874.70:0.00805,(UltAc113:0.02316,(Ult13917:5.4E-
4,Ult13915:0.03925)0.692.6:0.01086)0.882.61:0.01154)0.877.68:0.00732,((((Ult13951:0.03515,((Ult13924:0.01131,(UltHolo3:0.00542,(Ult14019:5.5E-
4,((Ult14020:0.0242,((((Ult13905:0.00369,(UltAci88:0.00369,Ult13966:0.00747)0.093.5:5.5E-4)1.000.349:5.4E-
4,Ult13975:0.00357)0.035.4:0.0038,Ult13883:0.01508)0.149.7:0.00376,(((Ult14017:0.0037,((Ult13880:0.0037,Ult13974:5.5E-
4)0.987.50:0.01528,(Ult14015:0.00369,Ult14016:5.5E-4)0.672.9:5.3E-
4)0.787.38:0.00371)0.896.43:0.00751,(Ult13879:5.5E-
4,Ult13881:0.0037)0.815.33:0.00372)0.783.51:0.0037,(Ult13878:0.0,Ult13882:0.0):0.00369)0.945.53:5.4E-
4)0.980.26:0.02415)0.833.46:0.00657,Ult13996:0.03993)0.655.17:5.4E-
4)0.947.42:0.01467)0.879.57:0.01054)0.743.38:5.5E-
4,(Ult13921:0.01118,(((Ult13922:0.00372,UltAci96:0.0115)0.815.34:0.00366,Ult13925:0.0037)1.000.350:5.5E-
4,(Ult13920:0.0,Ult13923:0.0,Ult13965:0.0):0.00344)0.323.5:0.0075)0.787.39:0.00389)0.430.5:0.00736)0.745.36:0.0039,(Ult13927:0.0037,(Ult13929:0.0394,Ult13926:0.06589)0.895.66:5.4E-4)0.811.32:0.00375)0.568.5:5.4E-

4,((Ult13916:0.01487,Ult26810:5.4E-
4)0.895.67:0.0112,Ult13928:0.01137)0.777.43:0.00369)0.854.42:0.00751,((Ul
t14004:0.00748,((Ult13904:0.0,Ult13961:0.0):5.5E-4,Ult14003:5.5E-
4)0.920.68:5.5E-
4)0.935.50:0.01146,(((Ult14014:0.00303,(Ult14010:0.02817,(Ult14009:0.0241
7,(Ult13997:0.01881,Ult13998:5.4E-
4)0.980.27:0.02929)0.823.30:0.01047)0.843.38:0.0079)0.928.49:0.01174,(Ult
14005:0.00368,Ult14012:5.5E-4)0.889.59:5.4E-
4)0.455.13:0.00372,(((Ult14008:0.0072,Ult14018:0.01535)0.878.53:5.4E-
4,Ult14013:5.4E-4)0.972.39:5.4E-4,(((Ult14006:0.0,Ult14007:0.0):5.5E-
4,Ult14011:0.00369)0.810.33:0.00372,((Ult13964:0.00724,Ult14002:0.01556)0
.974.25:0.0234,UltAci94:0.01471)0.885.43:5.4E-
4)0.923.80:0.00752)0.801.36:0.00375)0.869.46:0.00723)0.773.43:0.00382)0.7
99.40:0.00753)0.435.7:5.5E-
4,(UltAc109:0.02586,((Ult14050:0.0217,(((((Ult14085:0.02277,Ult14087:0.00
904)0.950.38:0.0227,(Ult14084:0.01764,UltAc123:0.00913)0.870.37:0.01188)0
.949.53:0.01791,(((((((((Ult14043:0.01257,(UltAci83:0.02643,(((Ult14033
:0.01928,(Ult14037:0.02339,(((Ult14034:0.00371,Ult14036:0.00371)0.847.60:
0.00371,(Ult14035:0.01904,(Ult14028:0.01419,((Ult14026:0.0,Ult14027:0.0):
5.5E-4,Ult14025:0.00369)0.838.53:0.0097)0.961.48:0.0232)0.558.6:5.3E-
4)0.909.46:0.01587,Ult14032:0.02293)0.750.25:0.00366)0.878.54:0.01202)0.7
50.26:0.00357,((UltAc104:0.02851,Ult14088:0.02863)0.871.53:0.00621,(Ult14
030:0.01898,(Ult14029:0.01129,((Ult14022:0.0,Ult14023:0.0):5.5E-
4,((Ult14031:0.15792,UltAc102:0.00385)0.579.8:0.00772,Ult14024:0.0116)0.7
54.30:0.00347)0.150.3:5.3E-
4)0.852.49:0.00774)0.854.43:0.01533)1.000.351:5.4E-
4)0.842.56:0.0072,UltAc103:0.004)0.722.10:0.00501)0.801.37:0.0194)0.895.6
8:0.01557,UltAci84:0.0402)0.887.58:0.01145,Ult13846:0.00418)0.943.51:0.01
685,(Ult13844:5.5E-4,Ult13845:5.5E-
4)0.962.55:0.02167)0.759.47:0.00736,(Ult13847:0.01381,((Ult13836:0.00752,
Ult13835:0.00736)0.990.44:0.03163,(Ult13833:5.5E-
4,Ult13834:0.00371)0.726.14:0.00327)0.996.44:0.03975)0.903.53:0.01582)0.7
40.36:0.00366,(AdbBacte:0.02445,UltAci53:0.01974)0.997.54:0.05163)0.939.4
9:0.02171,(((UltAci63:0.00382,Ult13841:0.03162)0.903.54:0.01152,(((Ult137
91:0.0,Ult13797:0.0,Ult13947:0.0):5.4E-
4,UltAci82:0.04394)0.854.44:0.00775,((UltAci67:5.5E-
4,Ult13800:0.00735)0.926.66:0.00977,((UltAci68:5.4E-
4,Ult13959:0.01131)0.874.71:5.4E-
4,(Ult13792:0.00257,((Ult13801:0.00368,(Ult13794:0.0151,UltAci66:0.00369)
0.841.43:5.4E-4)0.983.27:5.4E-4,(((Ult13795:0.0,Ult13796:0.0):5.5E-
4,UltAci65:0.0037)0.859.48:5.4E-4,(Ult13798:0.04738,(Ult13793:5.5E-
4,(Ult13788:5.5E-4,Ult13799:0.00369)0.846.52:0.0037)0.370.3:5.5E-
4)0.944.44:0.00747)0.436.7:0.00372)0.922.63:0.00232)0.844.64:0.00234)0.75
4.31:0.00378)0.756.47:0.00487)0.920.69:0.01533)0.758.44:0.00779,((((UltAc
100:0.0105,(Ult13843:0.01382,(Ult13777:0.0083,Ult13837:0.00777)0.947.43:0
.02454)0.437.7:0.01387)0.303.5:0.00573,(((Ult13820:5.5E-
4,Ult13821:0.01876)0.941.55:5.4E-
4,(Ult13762:0.01525,(UltAci79:0.01117,(Ult13761:0.00741,(UltAci62:0.00743
,((Ult13772:0.00369,Ult13773:5.5E-
4)0.951.49:0.01119,((Ult13756:0.00373,Ult13774:0.00366)0.960.47:0.01121,(
(((Ult13771:0.00381,((Ult13757:0.0,UltHolo2:0.0):0.00362,Ult13763:0.00381
)0.916.62:0.00755)0.789.35:0.00361,((Ult13950:0.00742,Ult13764:0.00369)0.
716.13:5.5E-4,(Ult13758:0.0,Ult13760:0.0):5.5E-4)0.841.44:5.4E-
4)0.919.60:0.00744,Ult13963:0.00745)0.814.31:5.5E-4,Ult13759:5.5E-

4)0.936.54:5.4E-4)0.450.5:0.00369)0.327.6:5.5E-
4)0.854.45:0.00763)0.355.8:0.00746)0.593.4:5.5E-
4)0.883.61:0.00749)0.902.64:0.00921,(Ult13822:0.02385,(UltAc122:0.02373,U
lt14083:0.01527)0.960.48:0.02049)0.760.39:0.00599)0.849.43:0.01015)0.000.
437:5.4E-
4,(Ult13830:0.02009,Ult13838:0.03214)0.798.45:0.01843)0.819.32:0.0038,((U
ltAci85:0.01719,((((Ult13823:0.03731,UltrS180:0.00727)0.978.27:0.03174,((
Ult13802:0.00745,(UltAci75:0.01938,(Ult13810:0.00794,UltAci73:0.00326)0.8
43.39:0.0081)0.904.56:0.01191)0.937.46:0.01445,(((UltAci74:0.00728,(Ult13
812:0.09554,Ult13811:0.00298)0.988.53:0.03317)0.933.56:0.01669,(UltrS177:
0.02637,(Ult13789:5.5E-
4,Ult13803:0.00376)0.946.49:0.0162)0.827.37:0.00761)0.865.57:0.00786,(((U
lt13832:0.02242,((Ult13828:0.03379,Ult13829:0.01065)0.924.54:0.02218,Ult1
3831:0.01327)0.303.6:5.4E-4)0.748.33:0.00382,(((((Ult13808:5.4E-
4,(((Ult13806:0.00372,(Ult13804:0.0037,Ult13805:5.5E-
4)0.794.39:0.00373)0.873.56:0.00755,Ult13807:0.00375)0.875.59:0.00755,Ult
rS178:0.00369)0.788.38:0.00373)0.778.48:0.00579,UltAci72:0.00545)0.925.62
:0.01561,UltrS179:0.00941)0.837.31:0.00962,(UltAci71:0.00378,(Ult14545:0.
08829,Ult13815:0.01293)0.983.28:0.0451)0.856.38:0.01172)0.982.32:0.03131,
((Ult13809:0.02315,Ult13813:0.03048)0.822.30:0.01,(Ult13819:0.00766,(Ult1
3818:5.4E-4,(Ult13816:5.5E-
4,Ult13817:0.00368)0.994.47:0.0191)0.993.37:0.03594)0.925.63:0.01953)0.61
7.3:0.00905)0.708.16:0.0051)0.943.52:0.01782,((UltAci70:0.01489,(UcvtSoi2
:0.02255,UltAci69:0.08369)0.704.10:0.00314)0.875.60:0.00924,(Ult13824:0.0
1105,(Ult13825:0.01162,(UltAci81:0.01528,(Ult13826:0.00376,Ult13827:0.007
55)0.884.59:0.00792)0.863.51:0.00756)0.727.14:0.00386)0.938.39:0.01144)0.
448.5:5.4E-4)0.719.7:0.00414)0.665.15:5.5E-
4)0.411.7:0.00623)0.902.65:0.01892,(Ult13721:5.3E-
4,((Ult13839:0.01562,Ult13840:0.01538)0.959.55:0.02445,((Ult13695:0.0,Ult
13696:0.0):0.01159,(Ult13694:0.01057,(((Ult13700:0.01118,(UltAci49:0.0,Ul
t13938:0.0):5.5E-
4)0.767.47:0.00414,(Ult13781:0.0154,((Ult13698:0.00742,Ult13699:5.5E-
4)0.796.29:0.00373,(Ult13701:0.00742,Ult13939:5.5E-
4)0.811.33:0.00373)0.768.31:0.00366)0.482.7:0.00799)0.812.27:0.01027,(Ult
13697:0.01532,UltHolop:0.00382)0.785.45:0.0043)0.820.22:0.00803)0.823.31:
0.00814)0.782.48:0.0067)0.682.8:0.01206)0.845.46:0.01267)0.499.3:0.00804,
(Ult13776:0.00452,(Ult13941:0.02796,((Ult13775:0.0,Ult13948:0.0):5.5E-
4,Ult13949:0.00369)0.956.46:0.02056)0.142.4:0.01084)0.967.39:0.02445)0.85
5.56:0.00989)0.730.20:0.00333,(((Ult13723:0.0285,UltSolib:0.0149)0.900.46
:0.0145,(Ult13724:0.00741,(Ult13725:5.5E-
4,Ult13726:0.00369)0.845.47:5.4E-
4)0.960.49:0.01975)0.727.15:0.00396,(UltAci51:0.02046,UltAci52:5.4E-
4)0.934.50:0.01394)0.897.47:0.01203)0.942.53:0.01681)0.804.35:0.00488)0.7
35.22:0.00415)0.863.52:0.01181,(((UltEub24:0.01138,(Ult13765:0.00378,UltA
ci61:0.02309)0.415.8:0.00376)0.288.4:5.5E-
4,UltFor25:0.14091)0.249.5:0.00361,Ult13785:0.01533)0.317.8:0.00348)1.000
.352:5.4E-
4,((((((((Ult13683:0.08096,((UltAci48:0.00369,((((((Ult13693:0.00369,((U
lt13702:0.00369,Ult13703:5.5E-
4)0.905.47:0.00747,(Ult13782:0.0037,(Ult13714:0.00368,Ult13940:0.02292)0.
761.38:5.4E-4)0.957.46:0.01123)0.840.51:5.3E-4)0.801.38:5.4E-
4,((Ult13692:0.01525,Ult13712:0.00758)0.914.57:0.002,((Ult13685:0.0037,(U
lt13942:0.00369,Ult13945:5.5E-4)0.358.7:5.5E-
4)0.873.57:0.0037,Ult13713:5.4E-

4)0.912.69:0.00204)0.924.55:0.00696)0.814.32:0.00707,((Ult13715:0.00372,(
Ult11604:0.02272,(Ult13716:0.0,Ult13779:0.0,Ult13787:0.0,UltPro66:0.0):5.
5E-
4)0.791.34:0.00367)0.936.55:0.01186,Ult13710:0.007)0.738.18:0.00755)0.789
.36:5.4E-4,Ult13711:0.02308)0.661.6:5.4E-
4,Ult13718:0.00747)0.896.44:0.00748,((Ult13682:5.5E-
4,(Ult13709:0.01134,Ult13943:0.00369)0.565.6:5.4E-4)1.000.353:5.5E-
4,Ult13684:0.11562)0.907.57:0.0075)0.787.40:0.00374)0.972.40:5.3E-
4,((UltAci46:0.01509,Ult13944:5.5E-
4)0.854.46:0.00744,(Ult13708:0.01508,((Ult13678:0.01481,((Ult13704:0.0,Ul
t13783:0.0):0.00746,Ult13707:0.00375)0.762.58:0.00407)0.173.4:0.0038,(Ult
13688:0.09458,((Ult13690:5.5E-
4,Ult13780:0.02275)0.979.39:0.02261,(Ult13689:0.01502,(Ult13686:0.00369,U
lt13687:5.5E-
4)0.784.40:0.00405)0.710.12:0.00393)0.733.19:0.00336)0.926.67:0.01976)0.8
54.47:0.00778)0.763.48:5.4E-
4)0.963.49:0.0112)0.846.53:0.00944)0.694.6:0.00179,((Ult13680:5.4E-
4,(Ult13677:5.5E-
4,Ult13679:0.00369)0.962.56:0.0112)0.948.57:0.01096,(Ult13681:0.01144,Ult
13717:0.01952)0.880.58:5.4E-4)0.973.30:0.01488)1.000.354:5.3E-
4,UltAci47:0.0075)0.876.46:0.00755,((Ult13705:0.0,Ult13786:0.0):5.5E-
4,Ult13706:0.00742)0.751.23:0.00365)0.945.54:0.01745,Ult13720:0.01479)0.8
89.60:0.01131,Ult13719:0.01066)0.680.8:0.00497,Ult13691:0.02014)0.886.69:
0.01202,(((((Ult13737:0.00352,(Ult13752:0.02817,((((Ult13747:0.01436,Ult1
3748:0.01426)0.918.57:0.01419,(Ult13746:5.5E-4,(Ult13744:5.5E-
4,Ult13745:0.00368)0.997.55:0.02348)0.926.68:0.01136)0.759.48:0.00379,Ult
13749:5.5E-
4)0.850.56:0.01063,(Ult13750:0.00581,Ult13751:0.027)0.120.4:0.00782)0.569
.8:0.01415)0.875.61:0.0134)0.939.50:0.01555,Ult14021:0.00376)0.797.34:5.5
E-4,((Ult13738:0.02696,Ult13755:5.5E-
4)0.639.4:0.00402,((((Ult13739:0.00369,((Ult13754:5.5E-
4,(UltAci59:0.00371,UltrS175:0.00743)0.458.11:5.5E-
4)0.857.57:0.0037,Ult13740:0.00369)0.000.438:5.4E-4)0.002.3:5.5E-
4,Ult13741:0.01124)0.821.31:0.00369,((((Ult13735:0.01133,((Ult13731:5.3E-
4,(Ult13733:5.5E-
4,Ult13736:0.01497)0.823.32:0.00369)0.837.32:0.0037,Ult13753:0.01126)0.10
9.5:5.5E-4)0.822.31:0.00369,Ult13732:5.5E-
4)0.444.11:0.00369,(AdbBact2:0.01397,UltAci60:0.01395)0.900.47:0.01393)0.
707.6:5.4E-4,Ult13734:5.4E-4)0.852.50:0.0037)0.617.4:5.5E-
4,(UltAci57:0.0,UltAci58:0.0):0.01124)0.779.47:0.00342)0.844.65:0.00748)0
.907.58:0.0109,(Ult13743:0.01913,Ult13742:0.0111)0.878.55:0.00785)0.868.4
5:0.00747,((Ult13730:0.06039,(UltAci56:0.04033,((Ult14041:5.4E-
4,(Ult14046:0.03073,(Ult14040:5.5E-
4,(Ult14038:0.00743,Ult14039:0.00369)0.727.16:5.5E-4)0.975.42:5.4E-
4)0.816.33:0.00366)0.923.81:0.01256,Ult14042:0.03494)0.743.39:0.00373)0.9
45.55:5.4E-
4)0.889.61:0.01095,(UltrS176:0.03689,Ult14045:0.02251)0.658.5:0.0088)0.90
5.48:0.01463)0.872.58:5.4E-
4)0.781.43:0.00383,((Ult13766:0.01851,Ult13767:0.07149)0.718.16:0.00418,U
lt13768:5.5E-
4)0.783.52:0.00732)0.888.58:0.00708)0.898.66:0.0145,((Ult13784:0.02354,Ul
tAci64:0.04065)0.870.38:0.01379,(Ult13722:0.03553,(UltAci50:0.02749,((Ult
14049:0.0038,((Ult14047:0.00743,Ult14048:0.01161)0.185.6:0.0073,UltAc106:
0.02705)0.273.3:5.4E-

4)0.758.45:0.00517,UltAc105:0.01806)0.931.49:0.02028)0.676.5:0.00617)0.32
8.8:0.01685)0.714.12:0.00573)0.725.16:0.01173,UltAc107:0.01982)0.847.61:0
.00878)0.740.37:0.00436,(Ult14044:0.04015,(Ult14055:0.01151,(((Ult14056:0
.03471,Ult14057:5.5E-
4)0.902.66:0.00979,((Ult14053:0.02253,Ult14054:0.00911)0.640.6:0.01027,((
UltrS185:0.01725,Ult14059:0.01131)0.069.4:0.00617,(Ult14058:5.4E-
4,((UltAci55:0.0107,((Ult13778:0.05259,((Ult13727:0.0,Ult13937:0.0):5.4E-
4,UltAci98:0.01092)0.845.48:0.01042)0.845.49:0.00926,(Ult13729:0.02581,(U
lt13946:5.3E-
4,(Ult13728:0.00747,UltAci54:0.0037)0.759.49:0.00716)0.890.59:0.0125)0.84
9.44:0.01105)0.856.39:0.0085)0.906.71:0.01197,Ult14060:0.00791)0.835.45:0
.00727)0.800.35:0.00884)0.668.11:0.01024)0.657.8:5.4E-
4)0.926.69:0.0167,(UltAci80:5.5E-4,Ult13901:5.5E-
4)0.999.84:0.05439)0.735.23:0.00344)0.937.47:0.01588)0.763.49:0.00447)0.8
44.66:0.00819,(UltAc108:0.03802,(UltAc112:0.02471,(UltAc110:0.02815,(UltA
c111:0.0149,(Ult14052:0.00369,Ult14051:5.5E-4)0.903.55:5.4E-
4)0.712.13:0.00659)0.326.2:0.00795)0.765.29:0.00675)0.710.13:0.00543)0.91
8.58:0.01615)0.674.10:5.3E-
4,((UltrS181:0.00622,Ult13842:0.00887)0.746.36:0.01267,((Ult13770:0.00845
,(Ult13769:0.00741,Ult13958:5.4E-
4)0.741.30:0.00651)0.959.56:0.02016,((Ult14061:0.01088,((Ult14063:0.00321
,Ult14065:0.01173)0.907.59:0.01135,(Ult14062:0.04801,UltHolo4:0.01095)0.7
33.20:0.0037)0.810.34:0.00758)0.667.10:5.5E-
4,Ult14064:0.03138)0.184.2:0.00437)0.906.72:0.01955)0.595.3:0.01377)0.820
.23:0.00725)0.924.56:0.01276)0.887.59:5.5E-
4,((Ult14739:0.05051,(UltAc141:0.0737,Ult13970:0.0887)0.152.3:0.00895)0.9
09.47:0.02089,(((((Ult14069:5.3E-
4,(Ult14070:0.02718,(Ult14072:0.02659,Ult14071:5.4E-
4)1.000.355:0.09659)0.683.14:0.01262)0.997.56:0.04162,((UltAc115:0.04809,
(UltAl198:0.0194,Ult14066:0.02305)0.353.2:5.4E-
4)0.899.57:0.01893,((UltAc114:5.5E-
4,UltrS186:0.00717)0.969.40:0.01905,(Ult14067:0.01116,Ult14068:0.00762)0.
951.50:0.01573)0.949.54:5.4E-
4)0.586.10:0.02321)0.617.5:0.00899,(((((Ult14076:0.01919,(Ult14075:0.0531
5,(UltSoli2:0.01529,UltAc119:0.01526)0.839.42:0.00715)0.898.67:5.4E-
4)0.673.5:0.00353,(Ult14077:0.0418,UltAc117:0.01595)0.745.37:0.01368)0.69
3.11:0.00387,(UltAc118:0.01908,(Ult14078:0.0,Ult14079:0.0):0.00356)0.865.
58:0.00753)0.770.61:0.0037,(UltAc116:0.00371,Ult14074:0.01498)0.747.32:0.
00376)0.813.35:0.00784,Ult14073:0.03737)0.402.6:0.00517)0.839.43:7.0E-
4,UltAc120:0.01112)1.000.356:0.05559,(UltAci95:5.4E-
4,((Ult13918:0.00743,(((Ult13869:0.0,Ult13956:0.0):5.5E-
4,Ult13896:0.01113)0.916.63:0.01514,Ult13919:0.00366)1.000.357:5.5E-
4)0.938.40:0.01094,(((Ult14081:5.4E-
4,(UltAc121:0.00711,Ult14082:0.01173)0.881.60:0.01137)0.815.35:0.00718,(U
lt14080:0.03175,UltHolo5:0.01198)0.727.17:0.00325)0.993.38:0.03224,Ult139
30:0.0156)0.873.58:0.00231)0.956.47:0.00129)0.755.34:0.0039)0.806.26:0.00
732)0.931.50:0.01929)0.920.70:0.01399,(((UdnAcido:0.07989,Ult13932:0.0128
5)0.351.6:0.01349,Ult13790:0.02753)0.901.61:0.01494,(Ult13907:0.05164,(Ul
t13931:0.04011,(((Ult13989:0.00756,((Ult13988:0.01164,((Ult13995:0.01206,
UltAc101:0.02267)0.996.45:0.04878,UltEub25:0.00501)0.878.56:0.01103)0.858
.50:0.01045,Ult13990:0.01904)0.765.30:0.00514)0.999.85:5.4E-
4,((Ult13986:0.0075,Ult13987:0.00374)0.799.41:0.00369,(Ult13991:0.00758,(
Ult13994:0.00368,(Ult13992:0.00369,(Ult13967:0.0,UltFor23:0.0,Ult13993:0.
0):5.5E-4)0.262.5:5.5E-

4)0.793.40:0.00371)0.764.42:0.00376)0.791.35:0.00327)0.988.54:0.02865,((U
lt13980:0.01156,UltFor24:0.01542)0.758.46:0.00328,(((Ult13976:0.0,Ult1398
5:0.0):5.5E-
4,((Ult13984:0.00334,(Ult13983:0.00717,(UltFor22:0.01499,(Ult13979:0.0074
4,Ult13982:0.00753)0.817.24:5.3E-
4)0.782.49:0.00399)0.467.7:0.00748)1.000.358:5.3E-
4,(Ult13977:0.00368,(Ult13981:0.01508,UltrS184:5.3E-
4)0.748.34:0.00745)1.000.359:5.4E-4)0.803.32:5.4E-4)0.956.48:5.5E-
4,Ult13978:0.00742)0.785.46:0.00389)0.801.39:0.00646)0.644.5:0.00819)0.68
0.9:0.00388)0.896.45:0.01408)0.748.35:0.00389)0.969.41:0.02658,(Ult13973:
0.09831,Ult13853:0.06415)0.843.40:0.01888)0.696.6:0.00288,Ult13960:0.0308
3)0.967.40:0.03723)0.942.54:0.04676)0.961.49:0.05028,(Ult14161:0.04191,((
(((((Ult14125:0.00744,(((((Ult14218:0.00445,Ult14219:0.02269)0.953.37:0.0
2266,((((((UltFor36:0.02791,(UltFor26:0.00368,(UltFor35:0.0,UltFor37:0.0)
:5.5E-
4)0.981.38:0.02395)0.762.59:0.00301,((Ult14221:0.0,Ult14222:0.0):5.5E-
4,Ult14223:0.00743)0.772.48:0.00346)0.876.47:0.00751,(Ult14224:0.00369,Ul
t14235:5.5E-4)0.968.40:0.01488)0.871.54:5.5E-
4,Ult14220:0.00739)0.433.9:5.5E-
4,Ult14233:0.02272)0.826.36:0.00681,(UltHolo7:0.00374,(((Ult14244:5.3E-
4,(Ult14238:0.00365,(UltFor42:5.3E-
4,(Ult14239:0.00376,(Ult14240:0.01511,UltFor43:0.00377)0.784.41:0.0036)0.
851.52:0.01099)0.883.62:0.01138)0.788.39:0.00376)0.499.4:0.00746,(((((Ult
14227:0.00756,(Ult14241:0.00748,(Ult14226:0.00362,Ult14216:0.01133)0.789.
37:0.00384)0.866.51:0.00745)0.960.50:0.01531,(Ult14217:0.00747,(Ult14237:
0.01118,Ult14234:5.5E-4)0.277.6:5.4E-4)0.900.48:0.00748)0.190.4:5.3E-
4,((((Ult14231:5.4E-
4,(((Ult14213:0.01129,UltEub28:0.00745)0.799.42:0.00609,((Ult14214:0.0,Ul
t14215:0.0):0.01164,(Ult14123:5.4E-4,(((Ult14212:5.5E-4,Ult14253:5.5E-
4)0.942.55:0.01508,(Ult14206:0.02367,(Ult14207:0.00392,((Ult14208:0.0,Ult
14209:0.0):0.0113,(UltFor33:0.00369,UltFor41:0.00369)0.808.37:5.4E-
4)0.923.82:0.01119)0.815.36:0.00749)0.688.9:0.01184)0.770.62:0.0038,(Ult1
4210:5.5E-4,Ult14211:0.00369)0.880.59:5.4E-
4)0.943.53:0.00739)0.786.49:0.00343)0.887.60:0.01075)0.794.40:0.0059,(Ult
For32:0.00371,Ult14229:5.5E-4)0.924.57:0.00748)0.335.7:5.4E-
4)0.942.56:0.00737,(Ult14228:0.00369,Ult14230:5.5E-4)0.906.73:5.4E-
4)0.854.48:0.00367,(Ult14232:0.0,UltFor39:0.0,UltFor40:0.0,UltAc136:0.0):
5.5E-
4)0.868.46:0.00714,(Ult14245:0.01156,(UltFor34:0.01189,(Ult14242:5.4E-
4,UltrS189:0.00332)0.362.6:0.0075)0.928.50:0.01493)0.949.55:0.01529)0.913
.57:5.5E-4)0.920.71:5.4E-
4,Ult14236:0.01133)0.837.33:0.00737,(Ult14225:0.01958,(Ult14243:0.02303,U
lt14246:5.4E-4)0.754.32:0.00779)0.776.44:0.00334)0.984.31:5.5E-
4)0.946.50:0.01123,UltFor38:0.00376)0.779.48:0.00361)0.879.58:0.00811)0.8
57.58:0.00749)0.950.39:0.01545,(((Ult14259:0.01596,((Ult14256:0.04225,(Ul
t14196:0.00366,(Ult14197:0.0037,Ult14198:0.00371)0.999.86:5.5E-
4)0.876.48:0.01502)0.939.51:0.02936,UltOr239:0.02404)0.475.10:0.01672)0.7
29.18:5.4E-
4,(Ult14255:0.01285,UltAc137:0.01782)0.988.55:0.02794)0.962.57:0.01782,(U
ltHolo6:0.01041,(Ult14257:0.04096,Ult14258:0.02046)0.826.37:0.02231)0.799
.43:0.00901)0.775.40:0.0037)0.727.18:0.00364,Ult14195:0.04396)0.461.6:0.0
0755,UltAc135:0.01092)0.994.48:5.3E-4)0.907.60:5.4E-
4,(Ult14181:0.00373,(Ult14180:0.01136,((((UltAc133:0.00742,((Ult14178:5.5
E-4,(((UltAc132:5.5E-

4,(UltFor30:0.00369,Ult14170:0.00369)0.792.46:0.00369)0.417.7:5.5E-
4,Ult14252:0.00743)0.923.83:0.00743,(Ult14171:5.5E-
4,(Ult14172:0.0,Ult14204:0.0):5.5E-4)0.845.50:0.0037)0.689.11:5.4E-
4)0.932.58:0.00369,Ult14176:5.5E-4)0.058.6:5.4E-
4)0.903.56:0.00744,(Ult14096:0.00204,Ult14177:0.00694)0.914.58:0.00212)0.
804.36:0.0037,Ult14179:5.5E-
4)0.916.64:0.01124,(Ult14122:0.01918,(Ult14175:0.0077,(Ult14173:0.0037,Ul
t14174:0.00371)0.769.41:0.00351)0.419.6:0.00379)0.384.5:0.00764)0.758.47:
0.00372)0.939.52:0.0113)0.951.51:0.0149)0.927.53:0.00681,((Ult14185:0.015
72,((UltAc134:5.3E-
4,(Ult14186:0.0195,Ult14187:0.01556)0.909.48:0.01099)0.940.52:0.01061,Ult
rS188:0.03934)0.848.49:5.4E-
4)0.970.44:0.01523,(((Ult14124:0.03582,Ult14189:0.01536)0.848.50:0.00738,
Ult14190:5.5E-4)0.916.65:0.00741,Ult14188:5.4E-4)0.877.69:5.5E-
4)0.823.33:0.00683)0.920.72:5.4E-4,((Ult14205:5.5E-
4,(UltFor27:0.00741,(Ult14203:0.02276,Ult14183:0.00747)0.933.57:5.5E-
4)0.878.57:5.5E-4)0.890.60:5.4E-
4,Ult14184:0.00742)0.904.57:0.00743)0.823.34:0.00369,((UltFor31:0.01123,(
Ult14191:5.5E-4,Ult14193:0.00369)0.829.41:5.5E-
4)0.853.41:0.0037,(Ult14182:0.00371,(Ult14194:0.00369,UltrS187:5.5E-
4)0.394.6:5.5E-4)0.874.72:0.00371)0.450.6:5.4E-
4)0.824.35:0.00363,(Ult14192:0.00369,Ult14199:0.00742)0.978.28:5.4E-
4)0.961.50:0.0285,(Ult14102:5.5E-
4,((Ult14104:0.00374,(Ult14169:0.01151,UltAc131:0.01923)0.755.35:0.00341)
0.976.31:0.01918,(Ult14200:5.3E-4,(((Ult14148:0.01118,(Ult14166:5.5E-
4,Ult14167:0.00369)0.720.12:5.5E-
4)0.815.37:0.00371,(((((Ult14131:0.00367,Ult14146:0.00379)0.899.58:0.0075
,((Ult14098:0.00365,((Ult14154:0.00742,Ult14157:5.5E-4)0.923.84:5.4E-
4,(Ult14153:0.00742,(Ult14127:0.00369,(Ult14151:0.0,Ult14152:0.0,Ult14156
:0.0):5.5E-4)0.945.56:5.4E-
4)0.083.3:0.0037)0.917.60:0.00755)0.899.59:0.00756,((Ult14145:0.0037,((Ul
tFor28:5.5E-4,UltFor29:0.04084)0.922.64:0.00747,(UltAc129:5.5E-
4,Ult14144:5.5E-
4)0.801.40:0.00367)0.791.36:0.00376)0.911.70:0.00245,((UltEub27:0.0,Ult14
147:0.0,Ult14150:0.0):5.5E-
4,Ult14149:0.00741)0.911.71:0.00245)0.911.72:0.00246)0.928.51:5.5E-
4)0.634.6:0.0037,(Ult14137:5.4E-
4,Ult14202:0.0037)0.831.30:0.00367)1.000.360:5.5E-
4,(((UltAc124:0.00745,((Ult14141:0.01153,(Ult14121:0.00372,((UltEub26:0.0
,Ult14132:0.0,Ult14134:0.0):5.3E-
4,Ult14133:0.00744)0.904.58:0.00746)0.899.60:0.00768)0.781.44:0.00348,(((
((((Ult14108:0.0,Ult14129:0.0):5.5E-4,Ult14113:0.00742)1.000.361:5.4E-
4,Ult14110:0.14272)0.842.57:0.00348,(Ult14109:0.00371,(Ult14111:0.00369,U
lt14112:5.5E-4)0.388.6:5.5E-
4)0.780.52:0.00373)0.958.62:0.01538,(Ult14128:5.4E-
4,(Ult14100:0.0037,Ult14095:5.5E-
4)0.878.58:0.00371)0.825.34:0.00369)0.117.3:5.5E-
4,((Ult14093:0.00368,((Ult14091:0.0037,((Ult14089:0.0,Ult14094:0.0):5.5E-
4,Ult14090:0.00741)0.796.30:0.0037)0.911.73:0.00747,Ult14092:0.0037)0.221
.4:5.5E-4)0.000.439:5.5E-4,(Ult14097:0.0,Ult14119:0.0):5.5E-
4)0.000.440:5.4E-
4)0.847.62:0.00753,Ult16349:0.05256)0.753.38:0.00753)1.000.362:5.1E-
4)0.581.9:5.5E-4,((Ult14099:5.4E-
4,(UltAc130:0.0037,Ult14168:0.00371)0.812.28:0.0036)0.139.5:0.00368,((((U

lt14101:0.0037,((Ult14139:0.0,Ult14143:0.0):5.5E-
4,(UltAc128:0.00369,Ult14106:0.00369)0.691.9:5.5E-4)0.843.41:5.5E-
4)0.863.53:0.00371,Ult14142:0.00745)0.639.5:5.4E-4,Ult14105:5.5E-
4)0.873.59:0.0037,Ult14140:0.00369)1.000.363:5.4E-
4)0.851.53:0.00359)0.920.73:0.00739,((Ult14117:0.01552,Ult14118:0.0117)0.
776.45:0.00324,Ult14116:0.00373)0.775.41:0.00374)0.216.4:5.4E-
4)0.838.54:5.3E-4,((Ult14160:0.00748,(UltAc127:5.4E-
4,((Ult14159:0.00369,Ult14164:0.00373)0.886.70:0.00721,(Ult14162:5.4E-
4,(Ult14126:0.00369,Ult14163:5.5E-
4)0.846.54:0.00369)0.973.31:0.01503)0.920.74:5.5E-
4)0.776.46:0.00367)0.787.41:0.00368,((Ult14165:0.00372,(Ult14114:0.00744,
Ult14115:5.4E-
4)0.813.36:0.00372)0.797.35:0.00371,(((Ult14135:0.00372,(UltAc126:0.00369
,(Ult14130:5.5E-4,Ult14155:0.00369)0.369.7:5.5E-
4)0.811.34:0.0037)0.775.42:0.00375,(Ult14158:0.00743,(UltAc125:5.5E-
4,(Ult14136:0.0,Ult14138:0.0):5.5E-4)0.857.59:5.5E-
4)0.801.41:0.00369)0.904.59:0.00755,(Ult14107:0.00757,Ult14120:0.00755)0.
876.49:0.00749)0.616.7:5.5E-4)0.524.4:5.5E-
4)0.866.52:0.00366)0.800.36:0.00367)0.773.44:0.00367,(Ult14103:0.0,Ult142
01:0.0):5.4E-
4)0.858.51:0.00365)0.805.35:0.00368)0.786.50:0.00363)0.712.14:0.00591)0.9
35.51:0.03813)0.959.57:0.04288)0.934.51:0.03423)0.487.11:5.4E-
4)0.920.75:0.03478)0.187.4:0.01902,Ult14744:0.10914)0.790.47:0.03627,(((U
ltDe158:5.5E-
4,(((Ult14803:0.0056,Ult14802:0.0118)0.809.40:0.00558,UltAc250:5.4E-
4)0.775.43:0.00368,Ult14804:5.5E-
4)0.815.38:0.01518)0.990.45:0.04576,((Ult14801:0.01136,(Ult14798:0.00717,
Ult14800:5.4E-
4)0.829.42:0.0073)0.749.49:0.00377,(Ult14799:0.01188,((((UltrS205:0.02669
,((Ult14756:5.2E-4,(UltAc243:5.5E-
4,Ult14786:0.00369)0.920.76:0.00741)0.000.441:5.3E-
4,((((Ult14785:0.00375,Ult14775:5.5E-4)0.507.5:5.4E-
4,(Ult14751:0.05597,(((((((Ult14765:0.00367,Ult14764:0.01127)1.000.364:5
.5E-
4,(Ult14776:0.00399,Ult14288:0.04791)0.344.7:0.00725)0.754.33:0.00359,(Ul
t14750:0.01495,Ult14781:5.4E-
4)0.893.59:0.00752)0.848.51:0.00761,(((((UltAc246:5.3E-
4,((Ult14759:0.0,Ult14792:0.0):5.5E-
4,Ult14791:0.00371)0.842.58:0.00369)0.988.56:0.01905,UltAc247:5.3E-
4)0.830.37:0.00368,(Ult14795:0.00358,(UltAc241:0.01137,(UltAc248:0.0,UltH
ol20:0.0,Ult14794:0.0):0.00373)0.878.59:0.00772)0.940.53:0.01157)0.221.5:
5.4E-4,UltrS206:0.00369)0.912.70:5.4E-
4,Ult14758:0.00718)0.993.39:0.03205)0.830.38:0.00736,((Ult14752:0.0,Ult14
779:0.0,Ult14780:0.0):5.4E-4,((Ult14783:5.5E-
4,(Ult14789:0.01109,Ult14787:0.02262)0.870.39:5.5E-
4)0.954.55:0.0074,(Ult14778:0.0,Ult14782:0.0):5.5E-
4)0.839.44:0.00367)0.943.54:0.00743)0.904.60:5.4E-
4,Ult14763:0.00361)0.868.47:0.00735,(Ult14767:0.00373,Ult14768:0.01525)0.
894.44:0.00735)0.907.61:5.4E-
4,(Ult14766:0.01535,Ult14788:0.01151)0.761.39:0.00361)0.764.43:0.00368)0.
796.31:0.00363)0.880.60:0.00371,UltAc244:5.5E-4)0.456.6:5.5E-
4,Ult14784:0.01489)0.957.47:0.01136)0.867.51:5.5E-
4)0.846.55:0.01014,(((Ult14760:5.5E-4,Ult14754:0.0037)0.700.21:5.3E-
4,(Ult14762:0.00372,Ult14757:0.00742)0.792.47:0.00366)0.847.63:0.01086,Ul

t14753:0.03772)0.445.12:0.01146)0.789.38:0.00133,(UltHol19:0.0037,((((Ult
Ac240:0.0,Ult14770:0.0,Ult14769:0.0):5.5E-
4,Ult14761:0.00367)0.815.39:0.00371,(((Ult14755:0.0,UltAc245:0.0):5.5E-
4,(UltAc242:0.00753,(Ult14777:5.5E-
4,Ult14772:0.01885)0.813.37:0.00367)0.809.41:5.5E-4)0.992.49:5.4E-
4,Ult14771:0.0037)0.971.40:0.01125)0.580.6:5.5E-
4,(Ult14774:0.03438,Ult14773:0.02702)0.326.3:0.01283)0.912.71:0.00326)0.8
95.69:0.01086)0.881.61:0.01149,((Ult14790:0.03121,(Ult14797:0.01177,(Ult1
4796:5.5E-
4,UltAc249:0.0191)0.830.39:0.00726)0.749.50:0.00467)0.945.57:0.01874,Ultr
S207:0.00428)0.779.49:0.01066)0.755.36:0.00441)0.932.59:0.01574)0.850.57:
0.01361)0.950.40:0.0347,((((Ult13141:0.02777,(UltGemm8:0.00368,Ult13140:5
.3E-
4)0.915.51:0.02545)0.966.58:0.06506,(Ult13142:0.06656,(UltDe151:0.13248,U
lt12720:0.01806)0.992.50:0.12014)0.007.3:0.07236)0.824.36:0.02485,((((Ult
13222:0.04242,(Ult13223:0.03195,Ult13224:0.01931)0.887.61:0.02466)0.892.4
5:0.02763,(Ult13225:0.0479,(UltOr233:0.06546,((UltOr232:0.0,UltCan94:0.0)
:0.01184,UltCan95:0.0666)0.933.58:0.03556)0.980.28:0.05566)0.748.36:0.010
53)0.983.29:0.04364,(Ult13221:0.0947,((UltOr234:0.03129,(Ult13227:0.02336
,Ult13226:0.02234)0.918.59:0.03123)0.920.77:0.03529,(Ult16039:0.07745,((U
lt13216:0.0456,(Ult13217:0.03149,((UltOr230:0.03176,Ult13215:0.08659)0.81
1.35:0.01568,UltDe149:0.07107)0.845.51:0.01393)0.843.42:0.01565)0.703.13:
0.00665,((Ult13206:0.05113,Ult13207:0.00823)0.455.14:0.00377,(((Ult13202:
0.00367,(Ult13203:0.00371,Ult13204:5.5E-4)0.954.56:5.4E-
4)0.942.57:0.01829,Ult13205:0.02242)0.854.49:0.00928,(Ult13208:0.0399,((U
lt13214:0.05389,UltOr231:0.00882)0.739.29:0.00245,(Ult13209:0.03542,((Ult
13219:0.03037,(Ult13220:0.02983,(UltOr229:0.02694,(((Ult13210:0.00933,Ult
13211:0.02912)0.907.62:0.01144,Ult13213:0.01725)0.784.42:0.00557,Ult13212
:0.0037)0.906.74:0.01236)0.500.6:0.00873)0.429.4:0.00731)0.854.50:0.01013
,Ult13218:0.02715)0.828.34:0.00787)0.787.42:0.00374)0.936.56:0.01167)0.75
9.50:0.00349)0.905.49:0.01073)0.886.71:5.4E-
4)0.897.48:0.02062)0.719.8:0.0255)0.846.56:0.0229)0.399.9:0.01987)0.760.4
0:0.0094,((Ult17194:0.0,Ult17195:0.0,Ult17196:0.0,Ult17197:0.0,Ult17198:0
.0):5.6E-
4,(Ult17199:0.01311,BacEnr41:0.01975)0.971.41:0.03769)1.000.365:0.10107)0
.940.54:0.02943)0.705.10:0.00593,(((Ult17193:0.02315,Ult17192:0.0035)0.92
6.70:0.02883,(Ult13019:0.12677,(UltPl554:0.03492,(Ult13018:0.0583,(UltAci
44:5.5E-
4,UltSpon3:0.00369)0.866.53:0.01892)0.587.8:0.01953)0.965.47:0.06438)0.95
2.51:0.05565)0.864.58:0.03448,((Ult17190:0.02573,(UltCal14:0.03739,(Ult17
191:0.02174,((UltCal13:0.0214,UltCal15:0.04846)0.952.52:0.02869,(Ult17187
:0.00704,(Ult17186:0.0054,(Ult17188:0.00371,Ult17189:0.0037)0.799.44:0.00
585)0.853.42:0.01421)0.011.5:0.00526)0.972.41:0.03266)0.366.5:0.012)0.842
.59:0.01707)0.835.46:0.01353,(Ult17200:0.04037,Ult17201:0.02804)1.000.366
:0.09908)0.430.6:0.01842)0.895.70:0.02539)0.823.35:0.01226)0.906.75:0.028
61)0.790.48:0.01415,(((Ult14879:5.3E-
4,((((Ult14887:0.00744,(((((UltAc289:0.02116,UltAc292:0.03422)0.809.42:0.
00372,UltAc291:0.00755)0.779.50:0.00364,UltAc290:5.4E-
4)0.980.29:0.02431,UltAc294:0.00329)0.737.38:0.00881,(UltAc293:0.01099,Ul
tAc295:0.02455)0.176.9:0.00873)0.999.87:0.06375)0.269.4:5.1E-
4,UltAc286:0.01499)0.804.37:0.00987,UltAc287:0.02037)0.925.64:0.01776,((U
lt14881:0.00748,(UltAc282:0.00371,UltAc285:0.02286)0.790.49:0.00367)0.770
.63:0.00319,((UdnBac61:0.00745,((UltAc283:0.00757,Ult14882:0.00377)0.825.
35:0.00361,(((Ult14883:0.0,Ult14884:0.0,Ult14885:0.0):5.5E-

4,((((Ult14877:0.00355,UltAc280:0.01155)0.866.54:0.0078,UltAc279:0.01127)
0.783.53:0.00382,((Ult14880:0.00754,Ult14895:0.00366)0.791.37:0.00397,Ult
14866:0.02309)0.774.56:0.00365)0.777.44:0.00364,((((Ult14890:0.0,UltAc288
:0.0):0.0037,(UltAc278:0.02301,((Ult14888:0.02361,Ult14878:0.00779)0.950.
41:0.00212,Ult14894:0.0307)1.000.367:0.00137)0.874.73:5.5E-
4)0.887.62:5.4E-
4,((((Ult14893:0.00758,UltAc281:0.00757)0.780.53:0.00367,Ult14892:5.4E-
4)0.825.36:0.00369,Ult14891:0.00743)0.302.4:5.5E-
4,Ult14889:0.0271)0.908.57:0.00738)0.183.6:0.00369,(((Ult14874:0.01816,Ul
t14875:0.01822)0.867.52:0.00952,(UltAc276:0.01143,Ult14870:0.0036)0.968.4
1:0.01546)0.602.5:0.00361,((Ult14873:0.01112,(Ult14871:0.01143,Ult14872:0
.00362)0.765.31:0.00408)0.190.5:0.00368,(UltAc277:0.01135,(Ult14876:0.003
69,Ult14865:0.01119)0.589.10:5.3E-
4)0.966.59:0.01897)0.766.35:0.00409)0.989.49:5.4E-
4)0.845.52:0.00364)0.789.39:5.4E-4)1.000.368:5.3E-
4,((Ult14886:0.01547,((Ult14860:0.0037,Ult14864:0.00369)0.937.48:5.3E-
4,(Ult14863:0.00373,((Ult14859:0.0,Ult14867:0.0):5.5E-
4,(Ult14861:0.00716,Ult14862:0.0186)0.908.58:5.3E-
4)0.799.45:0.00369)0.726.15:0.00367)0.946.51:0.01548)0.774.57:0.00375,Ult
14869:0.01547)0.802.39:0.00351)0.842.60:0.0074)1.000.369:5.4E-
4)0.750.27:5.1E-4,UltAc284:0.00369)1.000.370:5.5E-
4)0.439.8:0.01479)0.913.58:0.01547)0.987.51:0.07148,(Ult14897:0.04632,(((
((Ult14922:0.01777,Ult14923:0.00484)0.742.23:0.00586,(Ult14920:0.02049,Ul
t14921:0.03141)0.747.33:0.0061)0.984.32:0.04896,(Ult14899:0.01424,(((Ult1
4900:5.5E-
4,Ult14911:0.00743)0.955.39:0.02152,(UltrS208:0.02378,((UltAc297:0.0111,(
Ult14909:0.02356,Ult14910:0.01136)0.758.48:0.00443)0.725.17:0.0072,(UltAc
298:5.5E-
4,UltAc299:0.02281)0.468.10:0.01664)0.981.39:0.04415)0.881.62:0.01442)0.8
88.59:0.01375,((Ult14901:0.00375,(Ult14902:0.0,Ult14903:0.0,Ult14904:0.0,
Ult14906:0.0,Ult14908:0.0):5.5E-
4)0.991.43:0.02378,(Ult14905:0.0037,Ult14907:0.00744)0.869.47:5.3E-
4)0.781.45:0.004)0.845.53:0.00865)0.820.24:0.01641)0.837.34:0.01979,((Ult
14918:5.5E-
4,Ult14919:0.00368)0.947.44:0.02577,((UltrS210:0.00634,Ult14926:0.0562)0.
786.51:0.01743,((Ult14916:0.02366,(Ult14917:0.00749,Ult15396:0.00374)0.55
7.8:0.00329)0.854.51:0.02172,(((Ult14913:0.00747,Ult14925:5.5E-
4)0.940.55:0.01828,((Ult14912:0.0,Ult14924:0.0):5.5E-
4,UltrS209:0.00754)0.839.45:0.00916)0.967.41:0.02784,(Ult14914:0.0315,Ult
14915:0.02243)0.107.9:0.00957)0.764.44:0.00946)0.943.55:0.0298)0.827.38:0
.01353)0.904.61:0.02223)1.000.371:0.08519,(Ult14898:0.08712,(Ult16889:0.0
4717,(Ult16890:0.02256,((Ult16031:0.01924,((Ult16887:0.0,Ult16891:0.0):5.
4E-4,(Ult16030:5.5E-4,Ult16892:0.01116)0.902.67:0.00742)0.980.30:5.3E-
4)0.964.55:0.02832,Ult16888:0.01196)0.892.46:0.02326)0.658.6:0.03601)0.97
8.29:0.06468)0.050.5:0.00958)0.696.7:0.01208)0.157.6:0.01897)0.954.57:0.0
5166,(((UltAce14:0.21048,(Ult15157:0.0862,((Ult15746:0.09191,Ult16429:0.0
977)0.870.40:0.03041,((Ult15923:0.0152,(Ult16026:0.0037,UltAc705:5.5E-
4)0.921.67:0.01378)0.769.42:0.00465,(((((((Ult15977:0.02958,Ult16003:0.01
024)0.916.66:0.01658,Ult15976:0.01315)0.717.18:0.00196,(UltAc690:0.04127,
((UltAc706:0.01155,(Ult16001:0.02278,(UltAc695:5.5E-
4,((((((((UltAc400:0.00369,(UltHol25:0.00375,Ult16006:0.01515)0.800.37:0.
00366)0.929.60:5.5E-
4,(Ult15706:0.0,UltAc399:0.0,UltrS260:0.0,Ult16047:0.0):5.5E-
4)0.000.442:5.5E-4,UltHol22:5.5E-4)0.890.61:5.4E-

4,(Ult16000:0.00742,(UltAc555:5.4E-
4,UltAc557:0.00745)0.919.61:0.01135)0.938.41:5.3E-
4)0.840.52:0.00369,Ult16038:0.00744)0.864.59:5.4E-
4,Ult15463:0.02257)0.820.25:0.00367,(UltOr257:5.4E-
4,(Ult15462:0.00371,Ult16046:5.5E-
4)0.936.57:0.01096)0.826.38:0.00367)0.013.3:5.5E-
4,(Ult15450:0.00369,((UltrS276:0.01869,(Ult16005:0.01151,Ult16004:0.01552
)0.351.7:0.0073)0.948.58:5.3E-4,(Ult15466:0.0,UltAc402:0.0):5.5E-
4)0.166.5:5.4E-4)0.851.54:0.00369)0.876.50:0.00365)0.864.60:5.5E-
4)0.226.6:0.00365)0.752.35:0.00338,(((UltAc461:5.4E-4,(Ult15572:5.3E-
4,UltAc460:0.01507)0.933.59:0.00741)0.997.57:0.03189,(Ult15447:0.03902,(U
lt15442:0.02675,(Ult15573:0.04602,Ult15574:0.01101)0.917.61:5.5E-
4)0.170.5:0.0041)0.770.64:0.00335)0.948.59:0.0161,(((Ult16002:0.04754,((U
lt15577:0.0167,(Ult16009:0.02466,((UltAc694:5.5E-
4,(UltAc692:0.00369,UltAc693:0.00742)0.654.5:5.5E-4)0.862.50:5.4E-
4,(Ult16008:0.00202,Ult16007:0.011)0.639.6:0.00196)0.937.49:0.0185)0.740.
38:0.00388)0.710.14:0.00343,(Ult15576:0.04941,UltAc462:0.04251)0.524.5:0.
01776)0.606.6:0.01703)0.339.9:5.5E-
4,((Ult15999:0.02761,(Ult15998:0.00357,(Ult15997:0.00742,((UltOr261:0.0,U
ltOr262:0.0):5.5E-
4,Ult15996:0.01124)0.876.51:0.00762)0.984.33:0.02795)0.836.33:0.00778)0.8
72.59:0.01089,((((Ult15994:5.3E-
4,Ult15995:0.01129)0.831.31:0.00369,(UltAc684:0.01138,(Ult15991:0.00753,(
UltGa298:0.01507,(UltOr260:0.00369,Ult15992:0.00369)0.780.54:5.5E-
4)0.869.48:0.00746)0.887.63:0.00758)0.761.40:0.00363)0.769.43:0.00165,(((
UltAc685:5.4E-
4,Ult15993:0.02934)0.085.3:0.00354,UltAc686:0.00366)0.807.30:0.00363,UltA
c687:5.5E-4)0.789.40:0.00552)0.799.46:0.00397,(((((Ult15982:5.4E-
4,(((Ult15981:0.02514,((Ult15978:0.01931,Ult15980:0.00376)0.849.45:5.5E-
4,(UltAc681:0.0408,Ult15979:0.0152)0.141.1:0.00709)0.742.24:0.01543)0.987
.52:0.03944,Ult15984:0.00809)0.769.44:0.00321,UltAc682:0.01952)0.783.54:0
.00358)0.347.5:0.00368,((Ult15983:0.00741,(Ult15989:0.01497,Ult15990:0.00
37)0.239.6:5.5E-4)0.855.57:0.00368,(UltAc683:0.00744,Ult15985:5.5E-
4)0.856.40:0.0037)0.908.59:5.5E-
4)0.863.54:0.00742,(Ult15986:0.0154,Ult15988:0.01128)0.767.48:0.00392)0.7
63.50:0.00356,Ult15987:0.01534)0.992.51:0.02493,(UltAc688:0.00366,UltAc68
9:0.00376)0.960.51:0.02067)0.000.443:5.4E-
4)0.909.49:0.00724)0.734.23:0.00112)0.885.44:0.00688,(Ult15924:0.0074,(((
UltAc530:5.4E-
4,(Udntdde4:0.0315,(Ult15661:0.00843,(Ult15420:0.01872,UltAc547:5.5E-
4)0.858.52:0.01806)0.868.48:0.00798)0.910.61:5.4E-
4)0.935.52:0.01158,((Ult16049:0.02774,Ult15605:0.05822)0.949.56:0.0229,((
(UltAc504:5.3E-4,((Ult15753:0.03079,UltrS273:5.3E-
4)0.891.50:0.01117,((((UltAc500:0.0037,(Ult15637:0.00361,(UltAc502:0.0036
1,(Ult15663:0.0,UltAc553:0.0):5.4E-4)0.536.7:0.00746)1.000.372:5.3E-
4)0.713.17:5.5E-
4,(Ult15634:0.00369,(Ult15674:0.00741,(Ult15633:0.00369,(Ult15691:0.0,Ult
Ac499:0.0,Ult15666:0.0):5.5E-4)0.682.9:5.5E-
4)0.877.70:0.0037)0.728.19:5.5E-4)0.000.444:5.5E-
4,(Ult15644:0.0,Ult15635:0.0,UltAc506:0.0,Ult15675:0.0):5.5E-
4)0.932.60:5.3E-4,Ult15638:0.0037)0.835.47:0.00368)0.924.58:5.4E-
4)0.909.50:0.00369,((Ult15631:0.00737,((((Ult15601:0.0,UltAc548:0.0,UltAc
529:0.0,UltrS258:0.0):5.5E-
4,Ult15652:0.01504)0.923.85:0.00738,(((Ult15613:5.5E-

4,((UltAc494:0.00368,(UltAc524:5.4E-
4,(Ult15649:0.00745,((((UltAc526:0.0,UltAc527:0.0,UltAc528:0.0):5.4E-
4,Ult15651:0.00369)0.932.61:0.00745,((Ult15664:0.00745,((Ult15604:0.0,Ult
15662:0.0,Ult15669:0.0,Ult15670:0.0):0.02326,(UltrS259:0.00365,Ult15671:0
.00762)0.924.59:0.0113)0.898.68:5.5E-4)0.816.34:0.00372,(Ult15619:5.5E-
4,(UltAc490:0.0,Ult15618:0.0,UltAc491:0.0):5.5E-4)0.907.63:5.5E-
4)0.360.9:5.4E-4)0.852.51:0.0037,(UltAc484:5.5E-
4,(UltAc485:0.0,UltAc549:0.0):5.5E-4)0.414.4:5.5E-4)1.000.373:5.0E-
4)1.000.374:0.00322)0.856.41:0.00373)0.368.3:5.4E-4,UltAc480:5.5E-
4)0.538.4:5.5E-4)0.503.3:5.5E-
4,UltAc486:0.0037)0.849.46:0.00372,Ult15608:5.3E-4)0.705.11:5.5E-
4)0.879.59:0.0037,(UltAc507:0.0,UltAc508:0.0,UltAc550:0.0,UltAc551:0.0,Ul
tAc552:0.0):5.4E-4)0.927.54:0.00745)0.135.5:5.4E-
4,((Ult15754:0.0,UltAc495:0.0,Ult15632:0.0,UltAc496:0.0,UltAc503:0.0,UltA
c505:0.0,Ult15673:0.0):5.5E-
4,(UltAc498:0.00369,(UltAc497:0.02635,Ult15672:0.00194)0.986.36:0.00187)0
.870.41:5.5E-4)0.543.5:5.5E-4)0.241.6:5.5E-4)0.882.62:5.4E-
4,(((((Ult15459:0.0,UltAc493:0.0):5.4E-
4,(UltrS253:0.01154,Ult15578:0.01223)0.853.43:0.0074)0.557.9:5.4E-
4,(((UltAc542:0.0112,(UltAc514:0.00742,UltAc541:0.01118)0.771.52:0.0037)0
.034.4:5.5E-
4,(((Ult15653:0.00366,(((UltAc472:0.0,UltAc473:0.0,UltAc475:0.0,Ult15620:
0.0):5.5E-4,Ult15704:0.00741)0.930.50:5.4E-
4,Ult15621:0.00739)0.306.3:0.00369)1.000.375:5.3E-
4,(UltAc478:0.00369,(UltAc561:5.5E-
4,((UltAc535:0.00724,UltAc543:0.00396)0.621.9:0.00751,UltAc544:0.03066)0.
747.34:0.00391)0.829.43:0.00369)0.613.10:5.5E-4)0.000.445:5.3E-
4,((UltAc536:0.0,UltAc537:0.0,UltAc539:0.0,UltAc540:0.0,Ult16427:0.0):5.5
E-4,(UltAc476:0.00369,UltAc477:0.00369)0.940.56:5.5E-4)0.000.446:5.5E-
4)0.904.62:5.5E-4)0.996.46:5.2E-
4,(Ult15654:0.0074,(UltAc538:0.00367,((Ult15656:0.00766,Ult15657:5.4E-
4)0.972.42:0.01885,(Ult15655:0.01477,(Ult15658:0.00738,Ult15659:5.5E-
4)0.499.5:5.4E-4)1.000.376:5.4E-
4)0.924.60:0.00734)0.753.39:0.00371)0.896.46:0.00724)0.852.52:0.00359)0.9
44.45:0.00478,((UltrS255:5.4E-4,(UltAc513:5.3E-
4,Ult15639:0.01132)0.863.55:0.00741)0.825.37:0.00367,(((Ult15643:0.00371,
Ult15645:0.01127)0.796.32:0.00369,((UltAc531:0.0112,(UltAc533:5.4E-
4,((Ult15660:0.01917,UltAc545:0.00747)0.142.5:0.00366,(UltAc560:5.5E-
4,UltAc532:0.00369)0.900.49:0.0076)0.742.25:0.0036)0.661.7:5.5E-
4)0.949.57:0.01119,(Ult15630:0.00743,(UltAc554:0.00369,((Ult15647:0.0,Ult
15641:0.0,UltAc518:0.0,Ult15642:0.0,UltAc520:0.0,UltAc522:0.0):5.5E-
4,UltAc519:5.4E-4)0.020.4:5.5E-4)0.000.447:5.5E-4)0.397.7:5.5E-
4)0.200.4:5.5E-4)0.374.7:5.4E-4,(Ult15617:5.4E-
4,((Ult15600:0.0,Ult15616:0.0):5.3E-
4,(((Ult15623:0.01502,(Ult15622:0.01482,((Ult15609:0.0,Ult15667:0.0):5.5E
-
4,(Ult15627:0.01123,(Ult15665:0.00208,(Ult15625:0.00749,Ult15624:0.00373)
0.872.60:0.007)0.808.38:0.00209)0.859.49:5.4E-4)0.665.16:5.5E-
4)0.590.4:5.4E-4)0.483.12:0.00366,(Ult15628:5.5E-
4,Ult15629:0.00744)0.914.59:5.3E-
4)0.879.60:0.00725,UltAc492:0.00384)0.798.46:0.0074)0.862.51:0.00725)0.79
9.47:0.00366)0.997.58:5.4E-
4)0.944.46:0.00496)0.944.47:0.00469,(((UltAc459:0.01507,((UltAc510:0.0,Ul
tAc488:0.0,UltAc489:0.0):5.5E-

4,(UltAc509:0.00369,UltAc487:0.00369)0.707.7:5.5E-4)0.983.30:5.5E-
4)0.310.2:0.00369,((((UltAc525:0.0075,(UltAc483:0.00368,Ult15614:5.5E-
4)0.487.12:5.4E-4)0.831.32:0.00365,(UltrS257:0.00738,(Ult15611:5.5E-
4,(Ult15636:0.00369,(UltAc501:0.0,UltAc512:0.0):0.0037)0.872.61:5.4E-
4)0.940.57:5.4E-4)0.346.9:0.00369)1.000.377:5.5E-
4,((UltAc515:0.0,Ult15640:0.0,UltAc516:0.0,UltAc482:0.0):5.5E-
4,(UltAc523:0.00741,UltAc481:0.00369)0.950.42:5.5E-4)0.763.51:5.5E-
4)0.769.45:5.4E-4,((Ult15648:5.5E-
4,UltrS256:0.00368)0.908.60:0.00743,(((UltrS254:5.3E-
4,((Ult15452:0.00741,Ult15678:0.00757)0.931.51:0.01135,(UltAc511:5.4E-
4,((UltAc521:5.5E-4,UltAc546:0.00744)0.683.15:5.5E-
4,(UltAc534:0.01118,UltAc474:0.0111)0.906.76:5.5E-
4)0.980.31:0.01499)0.413.8:5.4E-
4)0.866.55:0.00739)0.922.65:0.01098,UltAc517:5.5E-
4)0.914.60:0.0066,((Ult15610:0.00369,Ult15615:5.5E-
4)0.955.40:0.00746,Ult15612:5.4E-
4)0.881.63:0.00212)0.888.60:0.00206)0.570.5:5.4E-4)0.890.62:5.4E-
4)0.915.52:0.00737,(Ult15607:5.4E-4,(Ult15646:5.4E-
4,(Ult15626:0.06397,(Ult15668:5.5E-4,Ult15650:0.015)0.586.11:5.5E-
4)0.815.40:0.00367)0.845.54:0.00369)0.469.10:5.5E-4)0.960.52:5.4E-
4)0.951.52:0.01078)0.656.9:0.00357)0.734.24:0.00366)0.943.56:0.01117,(((U
ltAc666:0.09341,(Ult15570:5.5E-4,(UltAc401:0.01117,(UltAc709:5.4E-
4,((((((UltrS274:0.0,Ult15586:0.0,Ult15588:0.0,Ult15589:0.0):5.5E-
4,(Ult15597:5.5E-4,(Ult15596:0.00369,Ult16033:0.00369)0.276.5:5.5E-
4)0.909.51:0.00369)0.992.52:5.4E-4,((Ult15583:5.5E-
4,Ult15584:0.00742)0.107.10:5.5E-
4,(Ult15599:0.015,(UltAc470:0.00369,(UltAc471:0.0,Ult15598:0.0):5.5E-
4)0.661.8:5.5E-
4)0.938.42:0.00741)0.924.61:0.00744)0.840.53:0.0036,Ult15587:5.5E-
4)0.845.55:0.00742,((Ult15581:5.5E-4,((UltAc465:5.5E-
4,(UcvtSoi5:0.00739,(Ult15580:5.5E-4,UltAc464:0.00368)0.713.18:5.5E-
4)0.873.60:0.00368)0.887.64:0.00365,((UltAc468:0.00743,((Ult15579:0.00365
,UltrS251:5.5E-
4)0.843.43:0.00365,((Ult15929:0.0,UltAc466:0.0,UltAc467:0.0):5.5E-
4,UltAc469:0.00747)0.602.6:5.5E-4)0.884.60:5.5E-
4)0.892.47:0.00735,((Ult15592:0.00359,(Ult15593:0.00368,Ult15595:5.5E-
4)1.000.378:5.5E-4)0.530.6:0.00366,Ult15594:0.00361)1.000.379:5.4E-
4)0.612.9:5.4E-
4)0.925.65:0.00726)0.912.72:0.00727,((Ult15582:0.0,Ult15585:0.0,Ult15590:
0.0):5.5E-4,Ult15591:0.00367)0.994.49:5.4E-
4)0.835.48:0.00353)1.000.380:5.5E-4,(UltAc463:5.5E-
4,UltrS252:0.0074)0.976.32:5.4E-
4)0.926.71:0.01091)0.961.51:0.01847)0.673.6:5.4E-
4)0.789.41:0.00336)0.818.27:0.00744,(UltAc479:0.01123,(Ult15676:5.5E-
4,Ult15677:5.5E-4)0.991.44:5.5E-4)0.850.58:0.01113)0.994.50:5.3E-
4,((UltAc667:0.00742,UltrS240:0.00367)0.934.52:5.3E-4,UltAc665:5.5E-
4)0.995.58:5.4E-4)0.750.28:5.4E-4)0.390.4:0.00353)0.719.9:5.4E-
4)0.968.42:5.5E-
4)0.805.36:0.00347)0.818.28:0.00677)0.854.52:0.00798)0.928.52:0.01117,(((
(((Ult15922:5.5E-4,(Ult15921:0.01913,Ult15920:0.04609)0.966.60:5.4E-
4)0.940.58:5.3E-
4,(Ult15919:0.00784,UltAc664:0.01962)0.894.45:0.01114)0.608.12:0.00732,((
UltAc657:0.00374,UltAc658:0.00369)0.923.86:0.01686,(((Ult15910:0.0072,((U
lt16050:0.01204,(((UltAc700:0.0,UltAc701:0.0,UltAc703:0.0):5.4E-

4,((UcvtSoi6:0.01532,UltAc702:0.0076)0.790.50:0.00349,((UltrS277:0.0074,(
UltrS278:5.5E-4,(Ult16023:0.00743,(Ult16022:0.00368,Ult16045:5.5E-
4)0.917.62:0.00745)0.782.50:5.5E-4)0.831.33:0.00368)0.500.7:5.4E-
4,Ult16024:5.5E-4)1.000.381:5.5E-
4)0.385.10:0.00369)0.854.53:0.00361,(Ult16021:0.0,UltAc699:0.0):5.5E-
4)0.862.52:5.5E-
4)0.940.59:0.01522,(UltAc697:0.04319,(UltAc698:0.00753,((((UltAc696:5.5E-
4,(Ult15686:0.00752,Ult15457:0.00372)0.810.35:0.00372)0.315.8:5.3E-
4,UltAc704:0.00371)0.887.65:0.00717,(Ult16025:5.3E-
4,((Ult15458:0.0,Ult16020:0.0):5.5E-
4,Ult16014:0.0037)0.925.66:0.00743)0.956.49:0.01125)0.856.42:5.4E-
4,((Ult16012:5.4E-
4,(Ult15709:0.00756,Ult16011:0.0037)0.898.69:0.00752)0.854.54:0.00368,(((
Ult15569:0.00783,Ult15568:0.00387)0.757.32:0.00389,((Ult16017:5.5E-
4,(Ult16013:0.0149,((Ult16015:5.5E-
4,(Ult16018:0.00369,Ult16019:0.00369)0.922.66:5.5E-4)0.917.63:5.5E-
4,Ult16016:0.0037)0.766.36:5.5E-4)0.845.56:0.00368)0.934.53:5.5E-
4,Ult16041:0.01909)0.774.58:0.0071)0.764.45:0.00421,Ult16010:0.01134)0.00
0.448:5.4E-4)0.824.37:0.00393)0.789.42:0.00388)0.902.68:5.4E-
4)0.912.73:0.01552)0.742.26:0.00799)0.373.7:5.4E-
4,(UltAc662:0.0115,Ult15913:0.02067)0.895.71:0.01192)0.792.48:8.4E-
4,UltSlu33:0.02212)0.925.67:0.01751)0.869.49:0.01291)0.667.11:5.4E-
4,(Ult15915:0.0,Ult15916:0.0,Ult15918:0.0):5.3E-4)0.930.51:5.4E-
4,(Ult15914:5.5E-
4,Ult15917:0.00369)0.962.58:0.01116)0.772.49:0.00668,((Ult15907:0.01677,(
Ult15908:0.02556,(UltAc659:0.00368,Ult15606:5.5E-
4)0.842.61:0.01308)0.914.61:0.02246)0.914.62:0.01708,UltAc663:0.01353)0.4
57.7:0.0058)0.152.4:5.4E-
4)0.896.47:0.01313,((Ult16034:0.0739,(((((Ult16434:0.05729,(Ult15936:0.02
315,(Ult15937:5.4E-4,((UltAc669:5.5E-4,(Ult15925:0.00369,UltAc668:5.5E-
4)0.871.55:0.0037)0.949.58:0.01122,((Ult15926:0.00369,(Ult15928:0.00369,U
lt15927:5.5E-4)0.933.60:0.00743)0.539.1:5.3E-
4,((Ult15940:0.00369,((((Ult15966:0.00738,UltAc677:0.00373)0.825.38:5.5E-
4,(Ult15968:0.00369,UltrS275:0.01509)0.833.47:5.5E-4)0.772.50:5.4E-
4,((Ult15948:5.5E-
4,Ult15949:0.01116)0.372.3:0.00369,((Ult15965:0.01125,Ult15967:0.00369)0.
266.4:5.5E-4,Ult15964:5.5E-4)0.852.53:0.00364)1.000.382:5.5E-
4)0.953.38:5.5E-4,(((Ult15947:0.0112,((Ult15953:5.4E-
4,(((((UltAc675:0.0037,Ult15956:0.00744)0.694.7:5.5E-
4,(UltAc678:0.01509,(UltAc670:0.01912,((UltAc676:0.01118,(((((UltAc673:0.
01494,Ult15955:5.5E-4)0.738.19:0.00371,(UltHol24:5.5E-
4,Ult15934:0.02757)0.823.36:0.00723)0.884.61:0.00771,UltAc674:0.0115)0.69
9.17:0.00346,Ult15946:0.00757)0.722.11:0.00383,(UltAc671:0.01945,Ult15939
:0.01939)0.890.63:0.01141)0.869.50:0.00746)0.884.62:5.5E-
4,Udntddd4:0.01129)0.605.7:5.4E-4)0.851.55:0.00367)0.949.59:5.5E-
4)0.976.33:5.4E-4,(Ult15950:0.01494,Ult15951:5.4E-
4)0.911.74:0.00743)0.898.70:0.0075,((Ult15943:5.4E-4,(Ult15941:5.5E-
4,(Ult15690:5.4E-
4,(Ult15942:0.0,Ult15957:0.0):0.00362)0.142.6:0.0037)0.810.36:0.00361)0.9
87.53:0.01923,((Ult15958:0.00743,(Ult15945:0.01371,(Ult15961:0.00947,(Ult
15932:0.00742,Ult15933:5.5E-
4)0.921.68:0.0133)0.839.46:0.01165)0.871.56:0.00939)0.817.25:5.3E-
4,((Ult15944:0.00334,Ult15425:0.01166)0.872.62:0.01264,(Ult15959:0.0227,U
lt15960:0.00843)0.922.67:0.0198)0.921.69:0.01469)0.501.5:0.00373)0.742.27

:0.00381)0.885.45:0.00776,((((Ult15970:5.4E-
4,UltAc679:0.00368)0.980.32:0.03146,(Ult15971:5.4E-
4,Ult15972:0.01123)0.986.37:0.03148)0.823.37:0.00864,UltAc672:5.4E-
4)0.904.63:0.0081,(Ult15952:0.00336,Ult15930:0.00813)0.762.60:0.00401)0.7
54.34:0.00324)0.070.2:0.01134)0.799.48:0.01535,(Ult15962:5.5E-
4,Ult15963:0.00369)0.870.42:0.0078)0.029.3:0.00356)0.866.56:5.5E-
4,Ult15969:0.01517)0.894.46:5.4E-
4,Ult15954:0.00741)0.254.4:0.00368)0.889.62:0.00735)0.126.6:5.4E-
4,Ult15938:0.03106)0.892.48:0.00738)0.935.53:5.4E-
4)0.941.56:0.00689)0.835.49:0.01656)0.857.60:0.0216)0.703.14:0.00436,Ult1
6032:0.03596)0.732.14:0.02441,((UltPro74:0.03687,(Ult15701:0.05008,(UltB1
955:0.07514,Ult15703:0.04722)0.820.26:0.02945)0.428.7:5.4E-
4)0.931.52:0.0195,UltAc707:0.01103)0.818.29:0.01392)0.865.59:0.01607,(((U
lt15567:0.01509,(Ult15536:0.01119,(((((Ult15424:0.01113,UltAc448:5.5E-
4)0.759.51:0.00741,(((UltAc438:0.0,Ult15546:0.0,Ult15560:0.0,Ult15561:0.0
):5.5E-
4,(Ult15446:0.00362,((((((((UltAc443:0.01116,((Ult16037:0.00738,(Ult15575
:0.01496,(UltAc435:0.01095,((((UltAc432:0.00367,Ult15519:0.00369)0.927.55
:5.5E-4,(Ult15448:0.0,UltAc430:0.0,Ult15520:0.0,Ult15525:0.0):5.5E-
4)0.000.449:5.5E-4,(Ult15496:0.00368,UltAc431:0.0074)1.000.383:5.5E-
4)0.000.450:5.5E-4,UltAc433:0.00369)0.890.64:5.4E-4)0.467.8:5.3E-
4)0.451.11:0.00362)0.563.4:5.5E-4,(Ult15497:5.5E-
4,(Ult15495:0.00367,UltAc434:0.00367)0.904.64:5.5E-4)0.890.65:5.5E-
4)0.877.71:5.3E-
4)0.318.8:0.00362,(((Ult15451:0.0,Ult15464:0.0):0.00739,(Ult15429:5.5E-
4,Ult15468:0.00369)0.928.53:5.5E-4)0.195.3:0.00369,(UltrS241:5.5E-
4,(Ult15467:0.00742,Ult15465:0.00369)0.564.5:5.5E-4)1.000.384:5.4E-
4)0.754.35:0.00363)0.992.53:5.5E-
4,(Ult15540:0.0037,((UltAc691:0.0037,(Ult15514:5.5E-4,((Ult15510:5.5E-
4,(Ult15509:0.00743,Ult15511:0.00742)0.188.3:5.5E-
4)0.911.75:0.00737,Ult15515:5.5E-
4)0.429.5:0.00744)0.804.38:0.00366)1.000.385:0.00204,Ult15526:5.5E-
4)1.000.386:0.00164)0.953.39:5.5E-4)0.163.4:5.5E-
4,((UltAc422:0.01115,(UltrS248:0.00368,((UltAc419:0.0,UltrS249:0.0,UltAc4
21:0.0,Ult15488:0.0,Ult15489:0.0):5.5E-4,UltAc420:0.00369)0.472.4:5.5E-
4)0.792.49:5.4E-4)0.894.47:0.00706,Ult15513:5.5E-4)0.909.52:5.4E-
4)0.819.33:0.00345,((Ult15461:0.00748,UltAc453:0.01132)0.922.68:0.01135,(
UltAc556:0.00371,(Ult15552:0.01114,(((Ult15419:5.5E-
4,(Ult15432:0.00367,(Ult15427:0.01118,(UltrS244:5.5E-4,Ult15562:5.5E-
4)0.709.12:5.5E-4)0.838.55:0.00367)0.568.6:5.4E-4)0.984.34:5.4E-
4,(Ult15453:0.00738,(Ult15418:5.5E-
4,(UltAc449:0.00369,(Ult15413:0.00738,(UltrS239:5.5E-
4,UltrS238:0.00365)0.316.3:5.5E-
4)0.811.36:0.00365)0.806.27:0.00368)0.864.61:5.4E-
4)0.206.3:0.00364)0.907.64:0.00732,((Ult15415:5.5E-4,Ult15416:5.5E-
4)0.922.69:0.00368,(Ult15417:0.00367,Ult15571:0.00368)0.441.9:5.5E-
4)0.377.8:5.4E-
4)0.801.42:0.00359)0.778.49:0.00363)0.811.37:0.00362)1.000.387:5.5E-
4)0.828.35:0.00707,(((Ult15975:0.0,UltAc680:0.0):5.4E-
4,(Ult15973:0.0037,Ult15974:0.01925)0.760.41:0.00368)0.994.51:0.01881,((U
lt15454:0.0,Ult16035:0.0):5.5E-
4,(Ult15551:0.01876,(Ult16040:0.01508,(Ult15445:0.0,Ult15460:0.0,Ult15549
:0.0,Ult15550:0.0):5.4E-4)0.821.32:0.00368)0.933.61:5.4E-4)0.914.63:5.3E-
4)1.000.388:5.3E-4)0.881.64:0.00339,((((Ult15556:5.5E-

4,(UltAc445:0.00369,((UltAc414:0.0,Ult15501:0.0,Ult15523:0.0,Ult15533:0.0
,UltAc444:0.0,UltAc446:0.0,Ult15547:0.0,Ult15554:0.0,Ult15555:0.0,UltAc45
2:0.0,UltAc458:0.0):5.5E-
4,((((Ult15542:0.01104,(Ult15516:0.00611,Ult15565:0.01025)0.196.6:0.00596
)0.708.17:5.4E-
4,(Ult15543:0.00368,((UltAc395:0.0,Ult15422:0.0,Ult15473:0.0,Ult15527:0.0
):5.5E-4,((Ult15544:0.00373,((Ult15421:0.00368,(UltFor72:5.5E-
4,UltHol21:0.00761)0.434.5:5.5E-4)0.000.451:5.5E-
4,Ult15469:0.00369)0.295.10:5.5E-4)0.370.4:5.5E-
4,Ult15423:0.00369)0.450.7:5.5E-4)0.264.2:5.5E-4)0.065.7:5.5E-
4)0.862.53:0.00408,(((Ult15481:0.00738,Ult15482:5.5E-
4)0.782.51:0.00368,((Ult15480:5.5E-4,(UltrS245:0.00369,Ult15479:5.5E-
4)0.766.37:5.5E-4)0.226.7:5.4E-
4,Ult16036:0.01122)0.816.35:0.00368)0.887.66:0.00741,Ult15475:5.4E-
4)0.816.36:0.00367)0.119.4:5.4E-4,Ult15548:0.00368)0.434.6:5.5E-
4)0.000.452:5.5E-4)0.343.2:5.5E-4)0.249.6:5.5E-
4,(((UltAc450:0.0,UltAc451:0.0):5.5E-
4,(Ult15470:0.0,UltAc403:0.0,Ult15471:0.0,Ult15472:0.0,UltrS242:0.0):5.5E
-4)0.951.53:5.5E-4,(UltHol23:0.00737,UltAc457:0.00743)0.898.71:5.4E-
4)0.900.50:0.00366)0.237.7:5.5E-4,UltAc454:0.00368)0.488.7:5.5E-
4,(Ult15566:0.00743,(Ult15531:0.0419,Ult15553:0.04241)0.848.52:0.00849)0.
791.38:0.00367)0.534.4:5.4E-4)0.972.43:5.4E-
4,(Ult15750:0.00746,Ult15563:5.5E-
4)0.844.67:0.00362)0.003.3:0.00387)1.000.389:5.5E-4)0.273.4:5.4E-
4,(((UltAc398:0.00742,UltAc407:0.01113)0.883.63:5.5E-
4,(((UltAc396:0.0,Ult15433:0.0,Ult15435:0.0,UltAc397:0.0,Ult15437:0.0,Ult
15440:0.0):5.5E-4,(Ult15438:0.00369,Ult15490:0.00369)0.702.7:5.5E-
4)0.000.453:5.5E-4,((((UltAc408:0.0,UltAc409:0.0,Ult16044:0.0):5.5E-
4,Ult15491:0.00369)0.851.56:0.0037,Ult15436:0.00375)0.432.9:5.5E-
4,Ult15439:0.00369)0.747.35:5.5E-4)0.736.26:5.5E-4)0.394.7:5.4E-
4,Ult15434:5.5E-4)0.948.60:0.00734)0.822.32:0.00383)0.677.7:5.5E-
4,(Ult15559:5.5E-4,((Ult15500:0.00491,(UltAc425:0.00368,Ult15557:5.5E-
4)0.945.58:0.00492)0.945.59:0.00491,(Ult15443:0.00748,((Ult15505:0.00369,
((UltAc447:0.00372,(UltAc416:0.00734,(((UltAc413:5.5E-
4,(Ult15517:0.00371,Ult15478:0.00743)0.541.2:5.5E-
4)0.934.54:0.00367,(Ult15524:0.00367,(((Ult15558:0.01114,((Ult15935:0.011
23,(((Ult15455:0.0,Ult15492:0.0,UltAc427:0.0,UltAc428:0.0,UltAc429:0.0):5
.5E-4,((((Ult15518:0.00361,(Ult15506:0.0,UltAc426:0.0):5.3E-
4)0.668.12:0.00744,(Ult15494:5.5E-4,(Ult15507:5.5E-
4,Ult15512:0.0038)0.753.40:5.5E-4)1.000.390:5.4E-
4)1.000.391:0.00215,Ult15931:0.0037)1.000.392:0.00145,Ult15508:0.0074)0.6
05.8:5.5E-4)0.947.45:5.5E-4,Ult15528:0.00369)0.693.12:5.4E-
4)0.919.62:0.00743,(Ult15521:0.00256,(((((UltAc455:0.002,Ult15537:0.01474
)0.949.60:0.00191,((Ult15486:0.00738,(UltrS250:0.00744,(UltAc423:0.0,UltA
c424:0.0):5.5E-4)0.916.67:0.00744)0.801.43:5.5E-4,Ult15564:5.5E-
4)0.765.32:5.1E-
4)0.846.57:0.00365,((Ult15426:0.00376,UltAc417:0.01129)0.898.72:0.00739,(
((UltrS246:0.00367,(UltrS247:0.00369,UltAc418:0.01115)0.869.51:5.5E-
4)0.744.27:5.5E-
4,(Ult15483:0.0,Ult15484:0.0,Ult15487:0.0,Ult15493:0.0,Ult15522:0.0,UltAc
456:0.0):5.5E-4)0.643.5:5.5E-4,Ult15499:0.00744)0.898.73:5.4E-
4)0.321.3:0.00367)1.000.393:5.4E-4,(Ult15502:0.0,UltAc439:0.0):5.5E-
4)0.690.15:5.5E-
4,Ult15485:0.00741)0.885.46:0.00175)0.914.64:0.00747)0.090.4:5.5E-

4)0.458.12:5.5E-
4,(Ult15477:0.0,UltAc436:0.0,UltAc437:0.0,Ult15529:0.0,Ult15530:0.0):5.5E
-4)0.234.5:5.5E-4,(Ult15414:5.4E-
4,(Ult15534:0.00369,(Ult15430:0.00367,Ult15535:5.5E-
4)0.795.40:0.00367)0.896.48:0.0074)0.832.36:0.00368)0.275.7:5.5E-
4)0.295.11:5.4E-4)0.791.39:5.5E-
4,(Ult15532:0.00366,(Ult15504:0.00369,Ult15539:0.00368)0.405.10:5.4E-
4)0.807.31:0.00362)0.209.6:0.0039)0.748.37:5.5E-4)0.386.7:5.5E-
4,(UltAc415:0.0,Ult15503:0.0,Ult15545:0.0):5.5E-4)0.301.4:5.5E-
4)0.165.2:5.3E-4,(UltAc404:0.00246,(UltAc405:5.5E-
4,UltrS243:0.00369)0.922.70:0.00246)0.922.71:0.00246)0.852.54:0.00392)0.3
56.6:5.5E-4)0.382.6:5.4E-4)0.273.5:5.5E-4)0.518.6:5.3E-4,(UltAc442:5.4E-
4,(UltAc440:0.0,UltAc441:0.0):0.00368)0.846.58:0.00363)0.010.4:0.0071,(Ul
tOr258:0.0037,Ult15538:5.5E-4)0.914.65:0.00716)0.941.57:5.4E-
4)0.774.59:0.00375)0.764.46:0.00361,(UltAc411:0.00369,UltAc412:0.01132)0.
897.49:0.0075)0.886.72:0.00337,((Ult15474:5.5E-
4,(Ult15476:0.00369,UltAc708:0.00742)0.825.39:5.4E-4)0.895.72:5.4E-
4,(Ult15541:0.01107,(Ult15444:0.01122,(Ult15431:5.5E-
4,UltAc406:0.00742)0.762.61:0.00368)0.691.10:0.00748)0.867.53:5.5E-
4)1.000.394:5.4E-
4)0.727.19:0.00379)0.768.32:0.0038,UltAc410:0.01953)0.832.37:0.01069)0.92
1.70:0.02575,(((Ult15895:0.00373,(((Ult15868:0.01125,Ult15876:5.4E-
4)0.956.50:0.01548,(Ult15867:0.0132,(((Ult15851:0.03864,(((Ult15857:0.0,U
lt15859:0.0):5.5E-4,(Ult15849:0.02289,Ult15850:0.00369)0.948.61:5.4E-
4)0.894.48:5.5E-4,Ult15848:0.04688)0.038.2:5.5E-
4)0.950.43:0.01486,(Ult15865:5.4E-
4,(((((UltAc630:0.0037,(Ult15864:0.0,Ult15866:0.0):5.5E-4)0.937.50:5.5E-
4,((UltAc633:0.00368,((Ult15862:0.0,Ult15863:0.0,UltAc635:0.0):5.5E-
4,(Ult15873:0.00741,Ult15861:0.00369)0.987.54:5.5E-4)0.000.454:5.5E-
4)0.893.60:5.5E-4,UltAc634:5.5E-
4)0.840.54:0.00362)0.796.33:0.00364,(UltAc628:0.00741,((Ult15853:5.5E-
4,(UltAc629:5.4E-
4,Ult15858:0.01995)0.957.48:0.01501)0.869.52:0.00369,UltAc631:5.5E-
4)0.686.11:5.4E-
4)0.792.50:0.00376)0.906.77:0.00745,(Ult15860:0.00369,((UltAc627:0.01416,
(Ult15854:0.0,Ult15855:0.0):0.00936)0.960.53:0.01835,(Ult15852:0.00365,Ul
t15856:5.5E-4)0.860.38:0.00383)0.994.52:5.5E-4)0.900.51:5.4E-
4)0.925.68:0.00764,(UltAc632:0.00371,UltOr259:0.00748)0.762.62:0.00372)0.
142.7:5.5E-4)0.968.43:5.4E-
4)0.757.33:0.00351,UltrS269:0.00781)0.923.87:0.01561)0.937.51:5.5E-
4)0.950.44:0.01132,(Ult15869:0.04937,((Ult15870:0.01165,(UltAc637:0.00369
,Ult15871:5.5E-4)0.276.6:5.5E-
4)0.830.40:0.00378,(UltAc636:0.00745,(UltAc639:0.01011,(UltAc640:0.00512,
(UltAc638:0.00663,Ult15872:0.00127)0.552.7:0.01098)0.863.56:0.01233)0.890
.66:0.00956)0.871.57:5.3E-
4)0.771.53:0.00599)0.782.52:0.00534)0.769.46:0.00364)0.918.60:5.3E-
4,UltAc654:0.01929)0.736.27:0.00292,((((Ult15879:5.3E-
4,(Ult15892:0.00369,Ult15894:0.0037)0.858.53:0.00714)0.827.39:0.00713,(Ul
tAc649:0.00378,UltAc650:0.01906)0.464.7:0.00375)0.899.61:0.0076,(((UltAc6
44:0.00751,(UltPro75:0.00369,Ult15880:5.5E-
4)0.971.42:0.01525)0.911.76:0.00405,((Ult15902:0.04711,((Ult15875:5.5E-
4,UltAc642:0.00369)0.816.37:0.0037,UltAc641:0.01512)0.897.50:5.4E-
4)0.951.54:0.01103,((Ult15898:0.0,Ult15900:0.0,Ult15901:0.0):5.5E-
4,(UltAc643:0.00369,(Ult15878:5.5E-

4,Ult15899:0.00369)0.914.66:0.00746)0.737.39:5.5E-4)0.991.45:5.4E-
4)0.950.45:0.01826)0.906.78:0.00418,((UltAc645:0.0,UltAc646:0.0):5.4E-
4,(Ult15882:5.4E-
4,((UltAc648:0.00748,((UltrS271:0.0,Ult15881:0.0,UltAc647:0.0):5.5E-
4,(UltrS270:0.00742,Ult15885:5.5E-4)0.840.55:0.00368)0.911.77:5.5E-
4)0.982.33:0.01509,(Ult15883:0.01115,Ult15884:0.00369)0.548.7:5.5E-
4)0.829.44:0.00366)0.948.62:0.0074)0.970.45:0.01538)0.884.63:0.00757)0.89
7.51:5.3E-
4,(((Ult15874:0.0037,((Ult15886:0.0074,(Ult15897:0.01466,(Ult15896:0.0036
9,(UltAc655:5.5E-4,(UltAc656:0.00369,UltSlu32:0.01124)0.829.45:5.5E-
4)0.996.47:5.4E-
4)0.739.30:0.00479)0.974.26:0.02326)0.865.60:0.0076,UltAc651:5.3E-
4)0.931.53:0.0074)0.656.10:5.5E-
4,(Ult15893:0.0075,UltAc653:0.01128)0.981.40:5.4E-
4)0.832.38:0.00368,(UltAc652:5.5E-
4,(Ult15890:0.00741,(Ult15441:0.01907,((Ult15877:5.5E-
4,Ult15889:0.00369)0.852.55:5.4E-4,(Ult15888:0.00374,(Ult15887:5.5E-
4,Ult15891:0.01127)0.791.40:0.00367)0.882.63:0.00748)0.758.49:0.00378)0.8
79.61:0.00742)0.847.64:5.4E-4)0.782.53:5.5E-
4)0.916.68:0.01107)0.906.79:0.01177)0.911.78:0.01706)0.895.73:0.01604)0.7
28.20:0.00446,(((((((UltAc584:0.00696,((((UltAc574:0.01103,Ult15905:7.2E
-4)0.492.9:5.3E-
4,UltAc568:0.00696)0.878.60:0.00696,((Ult15692:0.0,Ult15772:0.0,Ult15784:
0.0,UltAc595:0.0):5.5E-4,Ult15785:0.00369)0.933.62:5.4E-
4)0.752.36:0.0037,((Ult15824:5.5E-
4,Ult15844:0.00367)0.864.62:0.00364,Ult15724:0.02702)0.919.63:5.2E-
4)0.895.74:0.00186)0.901.62:0.00238,((UltrS263:0.00745,Ult15788:5.4E-
4)0.893.61:0.00986,(Ult15815:0.00591,(((Ult15732:0.0,Ult15768:0.0):5.5E-
4,Ult15769:5.5E-
4)0.851.57:0.00367,(Ult15770:0.00369,(UltAc612:0.00369,((Ult15683:0.0,Ult
15767:0.0,Ult15775:0.0):5.5E-4,Ult15771:0.00369)0.808.39:5.4E-
4)0.916.69:0.00746)0.086.7:5.4E-
4)0.860.39:0.0091)0.129.5:0.00375)0.775.44:0.00511)0.796.34:0.00733,((((U
lt15778:0.0,UltAc600:0.0,Ult15805:0.0):5.5E-
4,(UltrS265:0.00741,((Ult15830:5.5E-4,Ult15831:5.5E-4)0.929.61:5.4E-
4,(Ult15829:0.00371,Ult15813:0.00746)0.012.2:5.3E-
4)0.854.55:0.00371)0.881.65:5.5E-4)0.535.7:5.4E-
4,(((((UltAc583:0.0,Ult15837:0.0):5.5E-
4,(UltAc598:0.00369,(Ult15780:0.00369,UltAc596:5.4E-
4)0.837.35:0.00369)0.788.40:5.5E-4)0.809.43:5.4E-
4,Ult15839:0.00749)0.839.47:0.00368,((UltAc610:0.0,UltAc611:0.0):0.00369,
((((((((Ult15796:0.01116,((UltAc558:5.5E-
4,Ult15720:0.00369)0.832.39:0.00369,(Ult15721:5.5E-
4,UltAc601:0.00369)1.000.395:5.5E-
4)0.757.34:0.00369)0.782.54:0.0037,Ult15760:0.00372)0.817.26:0.00359,(Ult
15808:5.4E-4,((Ult15802:5.5E-
4,(Ult15774:0.00369,UltAc606:0.00742)0.696.8:5.5E-4)0.921.71:5.5E-
4,(Ult15738:0.00371,Ult15759:0.00747)0.656.11:5.5E-
4)0.848.53:0.00362)0.406.6:0.00369)1.000.396:5.5E-4,UltAc607:5.4E-
4)0.828.36:0.00368,UltAc569:0.01138)0.740.39:5.4E-
4,((Ult15700:0.0,Ult15843:0.0):5.4E-
4,Ult15799:0.0152)0.541.3:0.0037)0.884.64:0.00129,((Ult15689:0.00369,((Ul
t15761:0.01117,(Ult15807:0.00374,UltAc608:0.01535)0.899.62:5.4E-
4)0.930.52:0.01103,(UltAc609:5.5E-4,((UltAc591:0.00369,UltAc593:5.3E-

4)0.815.41:5.4E-4,((UltAc592:0.0,Ult15804:0.0):5.5E-
4,(UltB3272:0.01101,UltAc566:0.01889)0.867.54:5.5E-
4)0.444.12:0.00373)0.912.74:0.00749)0.190.6:5.5E-
4)0.915.53:0.00889)0.132.9:5.3E-4,((Ult15832:5.4E-
4,(UltAc619:0.00743,(Ult15835:0.00369,UltAc618:0.00742)0.004.6:5.5E-
4)0.838.56:0.0037)0.922.72:0.00749,Ult15698:5.5E-
4)0.866.57:0.00369)0.911.79:0.00328)0.922.73:0.00848,((Ult15838:5.5E-
4,((Ult15723:5.5E-4,(UltAc615:0.00373,(UltAc603:5.5E-
4,UltAc604:0.00749)0.814.33:0.00369)0.782.55:0.00373)0.613.11:5.5E-
4,UltAc620:0.00741)0.861.59:0.00369)0.795.41:0.00372,Ult15836:0.00762)0.7
86.52:0.0037)0.493.10:5.4E-4,(((UltAc616:0.0,Ult15834:0.0):5.5E-
4,(UltAc617:0.01513,Ult15833:5.5E-
4)0.809.44:0.00369)0.946.52:0.01148,UltAc621:0.00766)0.790.51:0.00352)0.3
35.8:5.5E-4)0.819.34:5.4E-4)0.362.7:5.4E-
4,((Ult15794:0.0074,Ult15795:5.5E-
4)0.910.62:0.01119,((((Ult15809:0.00372,((Ult15756:0.00364,(UltAc613:0.00
369,UltAc614:5.5E-
4)0.808.40:0.00384)0.754.36:0.00379,Ult15826:0.00767)0.791.41:0.00365)0.4
13.9:5.5E-4,Ult15820:5.5E-4)0.808.41:0.0037,((Ult15708:5.5E-
4,UltrS261:0.00372)0.519.6:5.4E-4,((((UltAc576:0.00371,Ult15741:5.5E-
4)0.696.9:5.5E-
4,((Ult15710:0.01114,((Ult15757:0.0,Ult15743:0.0,UltAc575:0.0):5.5E-
4,Ult15739:0.00369)0.316.4:5.5E-
4)0.782.56:0.00378,((UltAc570:0.0037,UltAc573:0.00372)0.835.50:0.00675,(U
lt15827:0.02247,Ult15841:0.00457)0.878.61:0.01136)0.873.61:0.00822)0.884.
65:0.00744)0.932.62:0.00196,UltAc577:0.01484)0.934.55:0.00193,(((Ult15740
:0.00246,Ult15742:0.00246)0.922.74:0.00246,Ult15735:0.00369)0.760.42:5.5E
-4,(Ult15758:0.0,Ult15736:0.0,Ult15737:0.0):5.5E-4)0.988.57:5.5E-
4)0.649.9:0.00367)0.905.50:0.00738)0.785.47:0.00366,(Ult15776:0.0037,Ult1
5777:0.00369)0.601.4:5.5E-4)1.000.397:5.5E-
4)0.829.46:0.00362)0.939.53:0.0074,Ult15747:0.02293)0.848.54:5.3E-
4)0.384.6:5.5E-4,((Ult15717:0.00369,Ult15718:5.3E-
4)0.702.8:0.00369,((((UltAc586:0.00369,Ult15688:0.00369)0.453.7:5.5E-
4,(Ult15696:0.0,Ult15697:0.0,UltAc559:0.0,UltAc582:0.0,UltAc585:0.0,UltAc
597:0.0,UltAc599:0.0):5.5E-4)1.000.398:5.5E-
4,UltAc580:0.05604)0.785.48:0.00349,((((((Ult15685:0.03568,UltrS267:5.5E-
4)0.845.57:5.4E-4,(Ult15755:0.0,Ult15797:0.0):5.5E-4)0.934.56:5.4E-
4,((Ult15810:0.02719,(Ult15793:5.3E-4,((Ult15715:5.5E-
4,(((Ult15790:0.00366,Ult15789:0.11349)0.787.43:0.00366,(((Ult15716:0.011
09,(Ult15707:5.5E-4,Ult15748:0.00365)0.755.37:0.01137)0.940.60:5.3E-
4,Ult15714:0.00368)0.959.58:5.5E-
4,(UltAc562:0.0,Ult15713:0.0,UltAc563:0.0,UltAc564:0.0,Ult15792:0.0):5.5E
-4)0.903.57:5.3E-4)0.899.63:5.4E-
4,Ult15791:0.01497)0.827.40:0.00366)0.788.41:0.00471,UltAc565:0.00267)0.8
90.67:0.00738)0.782.57:0.00371)0.754.37:0.00367,Ult15798:0.00376)0.799.49
:0.00371)0.864.63:0.00373,(Ult15764:0.0037,Ult15811:5.5E-
4)0.917.64:0.00747)0.593.5:5.5E-4,UltAc602:5.5E-4)1.000.399:5.4E-
4,Ult15906:0.00361)0.180.5:0.00373)0.780.55:5.1E-4)0.611.5:5.5E-
4)1.000.400:5.3E-4)0.890.68:0.00189,(Ult15825:5.5E-
4,((((((Ult15779:0.0,Ult15786:0.0):5.5E-4,(((UltAc588:5.5E-
4,((UltrS262:0.00735,(UltAc567:0.00747,UltAc578:0.00371)0.924.62:5.4E-
4)0.632.6:0.00745,((Ult15749:0.00369,(Ult15763:0.0,UltAc579:0.0):5.5E-
4)0.835.51:0.00704,Ult15773:0.02669)0.919.64:5.3E-
4)0.833.48:0.00385)0.642.6:5.4E-

4,(Ult15787:0.0114,Ult15803:0.00381)0.801.44:0.00364)0.910.63:0.0075,Ultr
S266:0.00369)0.642.7:5.5E-4)0.765.33:5.4E-
4,((((UltAc581:0.00359,((Ult15722:0.0,UltAc572:0.0,Ult15725:0.0,Ult15726:
0.0,Ult15727:0.0,Ult15728:0.0,Ult15730:0.0,Ult15781:0.0):5.5E-
4,((Ult15711:0.0037,Ult15719:5.5E-
4)0.963.50:0.01141,Ult15762:0.00369)0.810.37:5.5E-4)1.000.401:5.5E-
4)0.795.42:0.00369,UltAc594:0.00371)0.785.49:0.00368,(Ult15731:0.00745,((
Ult15752:5.4E-4,(UltAc571:0.00369,((Ult15699:0.0,Ult15782:0.0):5.5E-
4,Ult15712:5.5E-4)0.693.13:5.5E-
4)0.892.49:0.00738)0.102.1:0.00369,((Ult15729:0.0,UltAc589:0.0,UltAc590:0
.0):5.5E-4,Ult15783:0.01114)0.831.34:0.00364)1.000.402:5.4E-
4)0.918.61:5.5E-
4)0.758.50:0.00357,(Ult15682:0.01649,((Ult15684:0.01392,Ult15903:0.02868)
0.869.53:0.01498,((Ult15679:0.02671,Ult15681:0.03881)0.742.28:0.01271,((U
lt16363:0.07282,Ult15766:0.01325)0.863.57:0.01198,(UltGa299:0.04755,UltB2
361:0.03419)0.856.43:0.0114)0.720.13:0.003)0.922.75:0.02623)0.796.35:0.01
611)0.920.78:0.01406)0.948.63:5.5E-4)1.000.403:5.4E-
4,Ult15818:0.0389)0.915.54:0.00733,(((UltAc587:0.0,UltAc605:0.0):5.5E-
4,((UltrS264:0.0,Ult15812:0.0,Ult15814:0.0):5.5E-
4,(Ult15751:0.00754,Ult15765:0.00378)0.811.38:0.00366)0.830.41:0.0037)0.8
86.73:5.5E-
4,((Ult15816:0.0074,Ult15817:0.0037)0.780.56:0.00368,(Ult15456:0.03889,Ul
t15819:0.00714)0.919.65:5.4E-4)0.834.39:0.00366)0.650.6:5.4E-
4)0.927.56:0.00195,Ult15823:0.01091)0.930.53:0.00194)0.922.76:0.00205)0.9
20.79:0.00661,(((Ult15733:0.0,Ult15734:0.0,Ult15821:0.0):5.5E-
4,Ult15822:0.00734)0.925.69:0.00735,((UltAc623:0.0,UltAc622:0.0):5.5E-
4,(UltAc624:0.00369,((Ult15840:0.0,Ult15845:0.0):0.0037,Ult15842:0.00369)
0.172.7:5.5E-4)0.902.69:5.5E-4)0.792.51:5.5E-4)0.977.40:5.4E-
4)0.772.51:0.00347,Ult15828:0.00749)0.793.41:0.00532,Ult15806:0.01032)0.9
08.61:0.00956,(((UltAc626:0.0,Ult15904:0.0,Ult15603:0.0):5.5E-
4,(Ult15801:0.00369,UltrS268:0.00741)0.654.6:5.5E-4)0.916.70:5.5E-
4,(Ult15846:0.01128,((UltAc625:5.5E-4,Ult15847:5.5E-
4)0.918.62:0.00211,(Ult15800:0.00397,(Ult15909:0.00774,UltAc660:0.01133)0
.919.66:0.01171)0.866.58:0.00685)0.731.17:0.00199)0.694.8:5.5E-
4)0.987.55:5.4E-4)0.858.54:0.01034)0.752.37:5.4E-
4,(((Ult15911:0.00369,(UltrS272:0.0,Ult15428:0.0):0.00752)0.769.47:0.0037
5,Ult15912:5.5E-4)0.701.14:5.5E-4,UltAc661:5.5E-
4)0.822.33:0.00365)0.544.5:0.00342)0.725.18:0.00545)0.904.65:0.02022)0.84
9.47:0.02052)0.348.7:0.00679,((Ult16511:0.0451,(Ult16510:5.4E-
4,(Ult16508:0.02253,(Ult16507:0.02158,Ult16509:0.01346)0.566.5:0.01468)0.
976.34:0.03008)0.914.67:0.02027)0.987.56:0.04525,(((((Ult29490:0.02656,((
Ult16501:0.0087,(Ult16502:0.01521,(Ult16503:0.00475,Ult16504:0.0104)0.945
.60:0.02075)0.912.75:0.01833)0.959.59:0.02823,UltAc869:0.00216)0.832.40:0
.02378)0.979.40:0.05742,(Ult15695:0.02238,((Ult16256:0.01553,(UltAc799:0.
00372,Ult16279:0.00745)0.756.48:0.00357)0.966.61:0.04853,((((((Ult16266:0
.00746,(UltPro81:5.5E-4,((Ult16282:0.0,Ult16283:0.0):5.5E-
4,UltAc800:0.02277)0.845.58:0.00368)0.417.8:5.4E-
4)0.891.51:0.00743,Ult16271:0.00377)0.787.44:0.00388,((Ult16261:0.00738,U
lt16262:5.5E-
4)0.897.52:0.00763,Ult16364:0.00757)0.779.51:0.00355)0.768.33:0.00381,(Ul
t16259:0.02074,Ult16265:0.02841)0.701.15:0.00227)0.776.47:0.00347,((Ult16
272:0.00743,((Ult16258:0.0,Ult16270:0.0,Ult16281:0.0):5.5E-
4,((Ult16360:0.00739,((Ult16260:0.0,Ult16267:0.0):5.5E-
4,UltAc796:0.00374)0.885.47:5.4E-

4)0.174.5:0.0037,(((UltAc797:0.00745,Ult16269:0.00757)0.071.5:5.5E-
4,(Ult16257:0.0,Ult16263:0.0,Ult16264:0.0,Ult16268:0.0):5.5E-
4)0.985.44:0.00207,Ult16280:0.03044)0.984.35:0.00171)1.000.404:5.4E-
4)0.693.14:5.5E-4)0.805.37:0.00369,Ult16278:5.4E-
4)0.797.36:0.00388)0.885.48:0.01502,(Ult28884:0.04766,((Ult16274:5.3E-
4,(Ult16276:0.04707,Ult16277:0.00352)0.885.49:0.0076)0.784.43:0.00368,(Ul
tAc798:0.02304,(Ult16273:0.00369,Ult16275:5.5E-4)0.810.38:5.4E-
4)0.926.72:0.00745)0.799.50:0.00372)0.958.63:5.4E-
4)0.846.59:0.03037)0.958.64:0.04465)0.752.38:0.02812)0.381.10:0.01972,Ult
17464:0.07595)0.736.28:0.00993,((TlrDenit:0.09597,(UltrdL14:0.13805,((Ult
rSo54:0.14556,UltDel34:0.06371)0.859.50:0.0366,(((((UltB4297:5.5E-
4,UltB4295:0.0037)0.353.3:5.4E-
4,Ult27571:0.06951)0.938.43:0.01328,(UltB4301:0.01132,(UltB4298:5.5E-
4,(UltAci40:5.5E-4,(UltAci39:5.5E-4,(UltDel31:5.3E-
4,((UltB4299:0.01914,UltB4300:5.4E-
4)0.550.2:0.00373,Ult16423:0.01518)0.783.55:0.00375)0.843.44:0.00371)0.00
0.455:5.3E-
4)0.834.40:0.00372)0.762.63:0.00374)0.865.61:0.00967)0.848.55:0.01338,Ult
Del32:0.04086)0.957.49:0.04014,(UltDel33:0.08276,UltB4302:0.02402)0.857.6
1:0.0371)0.145.2:0.00906)0.736.29:0.04216)0.888.61:0.04075)0.601.5:0.033,
(UltB4614:0.17193,((Ult12564:0.19313,Ult16222:0.14907)0.854.56:5.4E-
4,((UltAc807:0.04606,((UltAc802:0.00749,(((Ult16290:5.5E-
4,FreSedim:0.00369)0.878.62:0.00736,(Ult16291:0.01905,(UltAc803:0.00369,U
ltAc804:5.5E-4)0.996.48:0.02736)0.367.10:5.4E-
4)0.813.38:0.01152,(Ult16285:0.01777,((Ult16286:0.00372,Ult16287:0.00369)
0.856.44:0.00764,(((Ult16284:0.0,UltAc805:0.0):5.5E-
4,UltAc806:0.00369)0.853.44:5.5E-4,(UltAc801:0.00369,Ult16292:5.5E-
4)0.858.55:0.0037)0.915.55:0.01139)0.781.46:0.00756)0.889.63:0.01307)0.75
2.39:0.00387)0.763.52:0.00268,(Ult16288:0.00725,Ult16289:0.00772)0.958.65
:0.02046)0.994.53:0.05445)0.409.5:0.01386,((Ult16244:0.14444,((Ult16239:0
.01729,(((UltDe162:0.08732,Ult16245:0.07143)0.773.45:0.00851,((UltAc793:0
.01141,Ult16247:0.01168)0.842.62:0.00746,(UltOr263:0.00371,((Ult16238:0.0
1959,((UltAc790:0.00369,((Ult16233:0.0158,(Ult16234:0.0038,UltAc788:0.019
01)0.854.57:0.00736)0.885.50:0.0079,Ult16235:0.00758)0.753.41:0.0036)0.57
6.8:5.5E-
4,(Ult16237:0.00785,(UltAc789:0.00399,Ult16236:0.0036)0.785.50:0.00374)0.
873.62:0.00759)0.752.40:0.00377)0.908.62:0.01152,UltAc791:5.4E-
4)0.971.43:0.01522)0.736.30:5.5E-4)0.883.64:5.4E-
4)0.847.65:0.00727,(Ult16248:5.4E-4,(Ult16242:5.4E-
4,((UltAc794:0.01638,(UltAc792:5.4E-4,(Ult16240:5.5E-
4,Ult16241:0.00369)0.942.58:0.00749)0.880.61:0.01101)0.757.35:0.00416,(((
Ult16246:0.0443,Ult16227:0.00424)0.782.58:0.0031,((Ult16225:5.5E-
4,UltAc786:0.0037)0.845.59:0.00372,((Ult16232:0.00747,((Ult16223:0.00742,
Ult16230:5.5E-4)0.833.49:0.00368,((Ult16224:0.0,UltAc784:0.0):5.5E-
4,(Ult16229:0.0037,UltHol31:0.01866)0.626.10:5.5E-4)0.501.6:5.3E-
4)0.870.43:0.00452)0.189.1:5.4E-4,(UltAc785:0.00499,(((Ult16226:5.5E-
4,UltHol30:5.5E-4)0.903.58:5.4E-
4,(UdnBac64:0.00375,UltHol32:0.00744)0.912.76:0.01109)0.855.58:0.01108,Ul
t16231:5.4E-4)0.959.60:0.00491)0.959.61:0.00491)0.839.48:5.4E-
4)0.064.4:5.5E-
4)0.874.74:0.00766,(UltAc787:0.0037,(Ult16243:0.0115,Ult16228:0.00767)0.7
90.52:0.00353)0.996.49:5.5E-
4)0.767.49:0.00293)0.951.55:0.01267)0.775.45:0.00766)0.780.57:0.00391)0.8
75.62:0.01442)0.556.4:0.00784,(((UltCan96:0.02664,UltCan97:0.00985)0.804.

39:0.00892,((Ult16253:0.00764,(Ult16252:0.01531,Ult16254:0.00351)0.753.42
:0.00376)0.764.47:0.00371,(Ult16251:0.00373,(Ult16249:5.4E-
4,Ult16250:0.01128)0.941.58:0.01133)0.791.42:0.00387)0.801.45:5.4E-
4)0.841.45:0.02009,(UltAc795:0.02272,Ult16255:0.00829)0.115.1:0.00344)1.0
00.405:0.09232)0.913.59:0.02304)0.753.43:0.01238,((((((UltAc851:0.0186,((
Ult16464:0.04385,(Ult16448:0.027,((Ult16450:0.03957,UltAc848:0.00222)0.98
2.34:0.0016,((Ult16457:0.01045,Ult16456:0.02601)0.442.9:0.01,(Ult16443:5.
5E-
4,(UltAc847:0.00753,Ult16444:0.01534)0.604.9:0.01149)0.881.66:0.00649)0.7
80.58:0.00455)0.798.47:0.00746)0.935.54:5.3E-
4)0.924.63:0.00746,(Ult16446:0.01884,(((UltAc846:0.04912,Ult16455:0.00288
)0.839.49:0.01623,Ult16479:0.09037)0.082.4:0.00396,(Ult16452:0.0149,((((U
ltAc854:0.03884,Ult16454:0.01001)0.781.47:0.0072,Ult16447:0.03864)0.654.7
:0.01515,((UltAc849:0.01403,(Ult16453:0.01743,UltAc853:0.02194)0.627.4:0.
00757)0.876.52:0.0093,((Ult16451:0.08801,UltAc859:0.01353)0.365.6:0.00645
,UltB2340:0.07641)0.889.64:0.0194)0.384.7:0.00288)0.773.46:0.00516,(Ult16
216:0.02643,UltAc855:0.0141)0.666.11:0.00927)0.545.11:5.4E-
4)0.899.64:5.4E-4)0.984.36:0.02314)0.828.37:5.3E-
4)0.793.42:0.00531)0.695.12:0.0024,((((Ult16491:0.00744,Ult16492:0.00769)
0.976.35:0.03276,(Ult16493:0.01319,(UltOr264:0.01498,UltPro83:0.00413)0.8
09.45:0.01051)0.551.6:0.01087)0.609.11:0.01073,Ult16494:0.04776)0.893.62:
0.02539,Ult16449:0.04484)0.850.59:0.02047)0.911.80:0.0218,(((UltAc862:0.0
0842,(Ult16487:0.00476,UltAc863:0.02714)0.955.41:0.02679)0.869.54:0.02169
,(Ult16481:0.02237,(Ult16480:0.03586,(Ult16470:0.00389,(Ult16471:5.5E-
4,(Ult16469:0.02717,Ult16468:0.00367)0.912.77:0.02319)0.994.54:0.03152)0.
740.40:0.00456)0.880.62:0.01691)0.837.36:0.01737)0.883.65:0.01772,(Ult164
82:0.01777,Ult16483:0.03007)0.758.51:0.00459)0.906.80:0.01596)0.478.6:0.0
1084,((((Ult16437:0.00705,(UltAc844:0.01528,(Ult16441:5.5E-
4,UltAc845:0.00745)0.763.53:0.00361)0.995.59:0.04564)0.831.35:0.00849,Ult
16442:0.03803)0.771.54:0.00561,UltAc842:0.01583)0.732.15:0.00836,(Ult1644
0:0.01528,((Ult16439:0.00645,(UltHol36:0.01378,(Ult16438:0.02838,Ult16436
:0.02633)0.747.36:0.00905)0.882.64:0.01561)0.886.74:0.01452,((Ult16445:5.
4E-4,(UltDe164:0.03092,UltAc850:5.5E-
4)0.860.40:0.00367)0.794.41:0.00376,(Ult16458:0.0235,((UltAc843:0.01072,U
lt16462:0.0308)0.770.65:0.0053,((((Ult16461:0.01148,UltAc856:0.00348)0.99
7.59:0.04939,UltAc860:0.03226)0.383.6:0.0034,(Ult16467:0.01301,((Ult16466
:5.5E-4,((Ult16463:0.0,Ult16465:0.0):5.5E-4,UltAc857:5.5E-
4)0.714.13:5.5E-4)1.000.406:5.1E-4,(UltAc858:5.4E-
4,UltHol37:0.00369)0.957.50:0.0188)0.554.9:0.03079)0.996.50:0.06299)0.775
.46:0.00429,(Ult16460:0.0044,Ult16459:0.01504)0.955.42:0.02892)0.873.63:0
.01091)0.768.34:0.00311)0.781.48:0.00378)0.785.51:0.00434)0.775.47:0.0030
2)0.759.52:0.00459)0.746.37:0.00444)0.972.44:0.0261,(((UltAc781:0.04305,(
Ult16198:0.00453,UltGeob2:0.00669)0.848.56:0.02483)0.896.49:0.02032,Ult16
199:0.04318)0.828.38:0.01086,((Ult16203:0.00373,((((UltAc782:0.01121,Ult1
6205:0.03487)0.006.5:5.4E-
4,Ult16200:0.03068)0.784.44:0.00366,Ult16204:0.019)0.849.48:5.4E-
4,((((UltAc780:0.00369,Ult16195:0.01121)0.639.7:5.5E-
4,(Ult16196:0.00369,((UltAc778:0.01129,UltAc779:0.00369)0.300.7:5.4E-
4,UltAc777:5.5E-4)0.843.45:0.00369)0.766.38:5.5E-4)0.836.34:5.3E-
4,Ult16192:5.5E-
4)0.856.45:0.00699,(Ult16193:0.00988,Ult16194:0.03406)0.761.41:0.00524)0.
996.51:0.03142)0.364.5:0.00363)0.855.59:0.00761,(((((((((Ult16179:0.0073
4,UltAc746:0.0311)0.780.59:0.00388,((Ult16180:0.02801,Ult16181:0.0184)0.9
96.52:0.06673,((((Ult16169:0.01528,(Ult16177:0.00738,(Ult16176:5.4E-

4,Ult16178:0.01933)0.799.51:0.00407)0.730.21:0.00494)0.895.75:0.01476,((U
lt16168:0.01505,(UltAc765:0.01533,Ult16167:0.02753)0.876.53:5.4E-
4)0.925.70:0.01141,(((Ult16174:0.01113,(UltAc766:0.01532,UltHol28:0.01135
)0.768.35:0.00408)0.784.45:0.01127,((Ult16173:0.0,UltAc767:0.0,UltrS281:0
.0,Ult16172:0.0):0.00376,Ult16171:0.00369)0.930.54:0.01127)0.997.60:5.3E-
4,(Ult16175:0.0077,UltrS282:0.01554)0.752.41:0.00357)0.781.49:0.00403)0.8
54.58:0.00691)0.958.66:5.4E-4,UltAl200:5.4E-
4)0.959.62:0.00746,Ult16170:5.4E-
4)0.766.39:0.00332)0.882.65:0.00783)0.763.54:0.00376,(UltAc776:0.03834,((
Ult16190:0.03913,(HolSpeci:0.01208,(UltAc774:0.04492,Ult16189:0.01586)0.7
64.48:0.00681)0.551.7:0.01246)0.885.51:0.01491,(((UltAc771:0.00466,(Ult16
184:0.02768,UltPro80:0.05774)0.356.7:0.00812)0.925.71:0.02198,(Ult16182:0
.01428,Ult16361:8.7E-
4)0.826.39:0.00823)0.765.34:0.00721,UltAc772:0.02799)0.886.75:0.01469)0.7
94.42:0.01728)0.819.35:0.01088)0.759.53:0.00357,((((Ult16157:0.0037,(Ult1
6161:0.01103,Ult16160:0.00371)0.236.2:5.5E-
4)0.913.60:0.00735,Ult16159:5.4E-
4)0.802.40:0.00359,((Ult16165:0.01125,((Ult16162:5.4E-4,(UltDe160:5.5E-
4,(UltAc763:0.00346,Ult16166:0.00396)0.995.60:0.02694)0.913.61:0.01096)0.
843.46:5.5E-
4,Ult16163:0.01893)0.688.10:0.00374)0.921.72:0.01118,(Ult16164:0.01507,Ul
t16158:0.0076)0.340.5:0.00366)0.953.40:5.4E-
4)0.993.40:0.03066,((Ult16197:0.057,Ult16213:0.04215)0.842.63:0.0179,Ult1
6212:0.02302)0.710.15:0.00354)0.749.51:0.00365)0.802.41:0.00356,(UltAc768
:0.03057,(Ult16210:0.01021,Ult16211:0.00542)0.874.75:0.01207)0.868.49:0.0
1177)0.000.456:0.00106,((Ult16123:0.03087,(UltAc747:0.00368,((Ult16124:0.
0,Ult16125:0.0):5.5E-4,Ult16135:0.00369)0.048.3:5.5E-
4)0.754.38:0.00382)0.940.61:0.01715,((Ult16141:0.02674,(((UltAc764:0.0174
,(Ult16152:0.02743,UltHol27:0.01316)0.884.66:0.02027)0.990.46:0.04674,(((
(Ult16132:5.4E-4,((Ult16131:0.0,UltAc748:0.0):5.5E-
4,(UltAc754:0.01885,UltAc753:0.00369)0.457.8:5.4E-
4)0.845.60:0.00368)0.991.46:0.02297,(UltAc758:0.02291,Ult16148:5.3E-
4)0.985.45:0.02287)0.851.58:5.5E-
4,(UltAc759:0.00764,(Ult16149:0.00432,Ult16150:0.01471)0.984.37:0.02818)0
.896.50:0.01113)0.885.52:0.00749,(UltAc755:0.01937,(Ult16151:0.00369,(Ult
Ac752:0.00774,Ult16140:0.04991)0.816.38:0.0075)0.757.36:0.00399)0.775.48:
0.00353)0.776.48:0.00411)0.787.45:0.00326,UltAc760:0.0113)0.728.21:5.4E-
4)0.926.73:0.01224,((Ult16139:0.0228,(Ult16156:0.02317,((((((Ult16137:5.4
E-4,UltAc751:0.0149)0.946.53:0.01507,(UltB4797:0.0038,Ult16138:5.5E-
4)0.776.49:0.00366)0.758.52:0.00374,Ult16136:0.0075)0.871.58:0.00731,(Ult
Ac750:5.4E-4,UltAc749:0.01891)0.899.65:5.4E-
4)0.436.8:0.00369,(Ult16128:0.0039,((Ult16126:0.0075,(Ult16134:5.4E-
4,Ult16129:0.00745)0.868.50:0.0037)1.000.407:5.4E-
4,Ult16127:0.00348)0.476.10:0.00743)0.927.57:0.01118)0.895.76:5.5E-
4,Ult16130:0.01483)0.915.56:0.01119)0.656.12:5.5E-
4)0.823.38:0.0123,(((((UltAc761:0.02393,Ult16153:0.01486)0.725.19:0.01469
,(Ult16154:0.04403,Ult16155:0.0308)0.699.18:0.00923)0.817.27:0.01274,UltA
c762:0.00608)0.762.64:0.00599,(((Ult16144:5.5E-
4,Ult16147:0.02689)0.216.5:0.00648,(UltAc756:0.00868,Ult16143:0.03043)0.8
23.39:0.00752)0.898.74:0.00848,(Ult16145:0.023,(Ult16146:0.01503,UltAc757
:0.0114)0.936.58:0.01138)0.936.59:5.3E-
4)0.965.48:0.01795)0.766.40:0.00491,Ult16142:0.0117)0.810.39:0.00647)0.29
5.12:0.00705)0.743.40:5.5E-
4)0.978.30:0.01901)0.716.14:0.00584,(((((Ult16185:0.0,UltAc773:0.0):5.5E-

4,Ult16186:5.5E-4)0.967.42:5.5E-
4,(Ult16183:0.0038,(UltAc769:0.00369,UltAc770:5.5E-
4)0.963.51:0.01519)0.771.55:0.0152)0.884.67:0.00888,(Ult16187:0.01742,Ult
16188:0.02206)0.313.5:0.00693)0.871.59:0.00989,(Ult16209:0.04378,((Ult162
08:0.01176,(Ult16206:0.02628,Ult16207:5.5E-
4)0.916.71:0.01088)0.960.54:0.01532,UltAc783:0.0192)0.741.31:0.00367)0.86
3.58:0.00747)0.066.5:5.3E-4)0.926.74:5.5E-
4,(Ult16201:0.03115,Ult16202:0.01972)0.828.39:0.01536)0.950.46:0.00638,((
UltAc775:0.00753,Ult16435:0.03141)0.735.24:0.0047,(Ult16191:0.01678,UltDe
161:0.02698)0.636.5:0.01082)0.901.63:0.01081)0.747.37:0.00295,Ult16133:0.
01585)0.833.50:0.00723)0.792.52:0.01372)0.937.52:5.5E-
4)0.887.67:0.01169,(((((UltAc819:0.01578,UltAc820:0.01568)0.751.24:0.0033
6,((Ult16350:5.5E-
4,(Ult16355:0.01576,UltAc825:0.00722)0.853.45:0.01154)0.854.59:0.01109,Ul
tHol33:0.02406)0.769.48:0.00372)0.869.55:5.5E-
4,((Ult16352:0.01525,(Ult16328:5.5E-4,Ult16330:0.00369)0.994.55:5.3E-
4)0.774.60:0.00368,(((Ult16348:0.0115,(Ult16334:0.00369,Ult16335:5.5E-
4)0.796.36:0.0037)0.788.42:0.00375,(((Ult16358:0.00744,Ult16314:0.0153)0.
935.55:5.4E-4,((((Ult16326:0.00319,((((Ult16320:5.5E-
4,Ult16321:0.00369)0.927.58:0.00772,((((Ult16331:0.00369,Ult16343:0.02265
)0.363.10:5.5E-4,Ult16333:0.0074)0.908.63:5.3E-4,((Ult16325:5.3E-
4,Ult16327:0.00744)0.797.37:0.00369,(UltPro82:5.5E-
4,Ult16324:0.00369)0.526.5:5.5E-
4)0.900.52:0.00722)0.886.76:0.00694,(Ult16317:0.0,Ult16332:0.0):5.4E-
4)0.745.38:0.00745)0.886.77:5.4E-4,((Ult16336:5.5E-
4,Ult16337:0.00371)0.940.62:0.00752,(Ult16357:0.00369,((Ult16338:0.0,Ult1
6341:0.0,Ult16342:0.0):5.5E-
4,(Ult16359:0.00369,Ult16339:0.01493)0.913.62:5.5E-4)0.932.63:5.5E-
4)0.680.10:5.4E-
4)0.830.42:0.00696)0.888.62:0.00715,(Ult16322:0.00369,(Ult16319:0.00369,U
lt16316:0.01499)0.871.60:5.1E-4)0.965.49:5.5E-
4)0.942.59:0.00988)0.563.5:5.3E-
4,(Ult16323:0.01005,(Ult16329:0.00457,Ult16345:0.00712)0.752.42:0.00459)0
.790.53:0.00768)0.778.50:0.00505,Ult16318:0.00759)0.888.63:0.00754,(Ult16
315:0.00743,UltAc817:0.0155)0.883.66:0.00768)0.895.77:5.5E-
4)0.838.57:0.00367,(Ult16340:0.0037,Ult16344:0.0037)0.794.43:0.00369)1.00
0.408:5.4E-
4)0.763.55:0.00364,(Ult16362:0.05302,(((((Ult16301:0.018,(UltAc815:5.5E-
4,Ult16309:0.00369)0.930.55:0.00475)0.730.22:0.00396,(UltAc810:0.00346,Ul
t16302:5.5E-
4)0.880.63:0.00767)0.771.56:0.00337,((Ult16293:0.0075,(UltrS284:0.00756,(
UltrS283:0.00369,UltAc808:5.5E-
4)0.792.53:0.00369)0.766.41:0.00379)0.758.53:0.00396,((((UltAc809:0.0,Ult
16299:0.0,Ult16304:0.0):5.5E-
4,(Ult16298:0.01132,(Ult16295:0.00382,((UltrS285:0.00363,((UltrS288:5.5E-
4,Ult16310:0.00745)0.845.61:0.00371,Ult16296:5.3E-4)1.000.409:5.3E-
4)0.139.6:0.00376,(UltAc812:0.0,Ult16300:0.0):5.4E-
4)0.832.41:0.00369)0.787.46:0.00387)0.752.43:5.4E-4)0.402.7:5.4E-
4,UltrS286:0.00369)0.898.75:5.4E-4,(((UltAc816:5.5E-
4,(Ult16307:0.00372,((Ult16311:5.3E-
4,(Ult16297:0.00368,UltAc813:0.00376)0.974.27:0.01909)0.921.73:0.01554,Ul
trS287:0.00785)0.739.31:0.00343)0.795.43:0.00371)0.445.13:5.4E-
4,(UltAc811:0.0037,(Ult16303:0.0,UltAc814:0.0):5.5E-
4)0.930.56:0.00744)0.811.39:0.00614,(Ult16308:5.4E-

4,((Ult16306:0.01139,Ult16312:0.00748)1.000.410:5.5E-
4,(Ult16294:0.00386,UltrS289:0.01116)0.448.6:0.00755)0.900.53:0.00349)0.8
89.65:0.01052)0.455.15:0.00611)0.929.62:0.01165)0.863.59:0.00804)0.765.35
:0.00655,Ult16313:0.00582)0.812.29:0.00939,Ult16305:0.02268)0.903.59:0.01
875)0.821.33:0.00965)0.808.42:0.00391)0.816.39:0.00385)0.997.61:5.4E-
4,(((Ult16354:0.02354,(Ult16353:0.01133,((UltAc822:5.4E-
4,(UltAc824:0.00742,UltAc823:0.00382)0.299.7:0.0037)0.937.53:0.01121,Ult1
6356:0.0037)0.939.54:5.4E-
4)0.790.54:0.00399)0.750.29:0.00279,(Ult16347:0.02696,(Ult16346:0.01626,U
ltrS290:0.04017)0.843.47:0.01062)0.931.54:0.01952)0.872.63:0.00871,(UltAc
821:0.00769,(Ult16351:0.00734,UltrS291:5.5E-
4)0.082.5:0.00375)0.908.64:0.01166)0.839.50:0.00754)0.741.32:0.004,UltAc8
18:0.01925)0.981.41:0.03131)0.711.18:0.01036)0.743.41:0.00658)0.925.72:0.
0201)0.638.6:0.01486)0.381.11:0.00858)0.741.33:0.01237)0.882.66:0.01283,(
(((UltAc865:0.00486,(Ult16498:0.00378,Ult16499:0.01956)0.918.63:0.01176)0
.890.69:0.01393,((Ult16496:0.0,Ult16497:0.0):0.03256,(UltAc864:0.00561,Ul
t16495:0.02612)0.841.46:0.01383)0.735.25:0.00422)0.984.38:0.04014,(UltAc8
68:0.04668,(UltAc866:0.00758,(Ult16500:0.0075,UltAc867:0.00367)0.852.56:0
.00748)0.980.33:0.05207)0.972.45:0.03926)0.865.62:0.01225,(((Ult14896:0.0
5734,Ult16512:0.09605)0.915.57:0.03037,(((Ult16505:0.03534,Ult16506:0.016
07)1.000.411:0.06492,(((Ult16120:0.06771,Ult16119:0.01829)0.947.46:0.0385
6,(Udntddd5:0.02607,((Ult16118:0.05815,(Ult16116:0.03642,Ult16117:0.03632
)0.888.64:0.02685)0.934.57:0.03067,((Ult16113:5.4E-
4,Ult16115:0.03963)0.896.51:0.00996,(Ult16114:0.02015,(UltAc744:0.01457,(
UltAct21:0.0137,(Ult16112:0.00397,UltAc743:0.03948)0.070.3:0.00581)0.968.
44:0.03003)0.709.13:0.0183)0.780.60:0.0084)0.606.7:0.00178)0.738.20:0.004
92)0.911.81:0.02567)0.872.64:0.02947,(Ult14931:0.07652,(UltAc296:0.09773,
(UltAc300:0.09116,(Ult14930:5.3E-
4,(Ult14927:0.01236,(Ult14928:0.02199,Ult14929:0.00465)0.147.4:0.00741)0.
905.51:0.01786)0.849.49:0.03186)0.921.74:0.04974)0.035.5:0.03138)0.827.41
:0.02568)0.669.8:0.01853)0.674.11:0.00937,((((((Ult17215:0.00984,(UltDe21
9:0.02412,UltDe220:0.0027)0.982.35:0.04409)0.999.88:0.07611,((UltOr265:0.
13873,Ult17216:0.01351)0.936.60:0.03969,(Ult17219:0.03743,Ult17220:0.0063
6)0.980.34:0.05379)0.639.8:0.01263)0.886.78:0.028,((Ult17117:0.00838,Ult1
7118:0.01517)0.930.57:0.02802,(UltDe229:0.01269,(Ult17493:0.01451,Ult1749
2:5.4E-4)0.846.60:0.01338)0.981.42:0.04491)0.927.59:0.03532)0.123.3:5.5E-
4,((((Ult16911:0.04657,Ult16910:0.0231)0.981.43:0.05256,Ult17214:0.07686)
0.283.3:0.01425,(Ult16783:0.09681,Ult17211:0.05569)0.817.28:0.01586)0.870
.44:0.02242,(((Ult16919:0.0861,Ult17358:0.03442)0.007.4:0.01641,((((UltDe
210:0.0187,Ult17060:0.03586)0.991.47:0.0741,((Ult17061:0.00382,Ult17062:0
.01925)0.998.68:0.05768,(UltDe211:0.03892,UltDe209:0.02333)0.759.54:0.008
61)0.778.51:0.01636)0.925.73:0.04283,(Ult17064:0.0423,(UltDe212:0.02913,U
lt17063:0.02168)0.940.63:0.02487)0.334.7:0.01492)0.922.77:0.0266,Ult17221
:0.01369)0.820.27:0.01628)0.873.64:0.02671,(Ult16121:0.04916,((((Ult17065
:0.0673,((UltOr179:0.08254,((Ult12560:0.0,Ult12561:0.0):0.04378,Ult12562:
0.02282)0.986.38:0.07649)0.950.47:0.04238,(((UltOr183:0.06731,(UltOr182:0
.0445,(UltOr181:0.03159,UltOr180:0.01186)0.872.65:0.01378)0.850.60:0.0279
7)0.352.4:0.01738,UltOr184:0.0252)0.951.56:0.04727,((UltOr188:0.03071,(Ul
tOr186:0.02318,UltOr187:0.02054)0.956.51:0.04178)0.962.59:0.05327,((Ult12
568:0.02542,(Ult12569:0.0731,(Ult12566:0.02434,Ult12567:0.00711)0.990.47:
0.05479)0.142.8:0.00526)0.998.69:0.11739,(Ult12563:0.02228,(UltOr185:0.02
172,UltCan42:0.0735)0.857.62:0.0198)0.858.56:0.04064)0.798.48:0.03445)0.5
99.5:0.0222)0.659.9:0.01446)0.901.64:0.03513)0.908.65:0.03989,UltOr189:0.
0992)0.793.43:0.02074,(Ult17058:0.06134,Ult17059:0.02034)0.968.45:0.04034

)0.823.40:0.01716,((Ult17217:0.05225,UltDe221:0.03693)0.993.41:0.07422,((
(UltDe204:5.4E-4,((UltDe203:5.5E-
4,((Ult17050:0.00377,Ult17051:0.00733)0.638.7:0.00369,(UltB4804:0.01122,(
Ult17048:0.00369,Ult17049:5.5E-4)0.954.58:0.01128)0.868.51:5.4E-
4)0.969.42:0.01506)0.901.65:0.0076,Ult17052:0.00365)0.853.46:0.01107)0.85
0.61:0.01409,(((((Ult17054:0.02587,UltDe207:0.00895)0.956.52:0.02289,(((U
lt17046:0.01494,((UltDe205:0.02761,(Ult17053:0.01535,(NspSpeci:0.02393,Ul
t17040:0.01246)0.679.13:0.01409)0.794.44:0.01346)0.911.82:0.01755,((Ult17
038:0.01146,Ult17039:0.01534)0.734.25:0.00555,(Ult17047:0.03206,UltDe208:
0.01564)0.897.53:0.01489)0.707.8:0.01049)0.726.16:0.00371)0.860.41:0.0114
6,(Ult17056:0.03107,((UltDe169:0.00778,((UltDe173:0.00369,UltDe175:5.5E-
4)0.863.60:0.002,(Ult17032:0.00744,((UltDe170:0.0,UltDe171:0.0,Ult17033:0
.0,UltDe172:0.0):5.5E-4,UltDe174:5.5E-4)0.734.26:5.5E-
4)0.918.64:0.00693)0.894.49:0.00186)0.991.48:0.0316,(((UltDe179:0.0,UltDe
180:0.0):5.4E-
4,UltDe181:0.0111)0.982.36:0.01913,((((UltDe182:0.00369,((UltDe185:0.0,Ul
tDe189:0.0):5.5E-
4,(((UltDe188:0.01136,UltDe190:0.00368)0.862.54:0.00755,(((Ult17057:0.003
72,(UltDe178:5.5E-
4,UltDe177:0.0037)0.915.58:0.00749)0.810.40:0.00371,(Ult17037:0.0037,((Ma
iMet25:0.0,UltOr270:0.0):5.5E-
4,UltDe186:0.00369)0.856.46:0.00371)0.015.4:5.5E-4)1.000.412:5.4E-
4,(Ult17034:0.00362,(Ult17035:0.0,UltDe176:0.0):5.4E-
4)0.322.6:0.0037)0.771.57:0.00368)0.354.5:0.00757,(UltOr269:0.01105,UltDe
187:0.03674)0.841.47:0.00764)0.958.67:5.5E-
4)0.870.45:0.00373)0.398.7:5.5E-4,UltDe183:5.5E-4)0.464.8:5.5E-
4,Ult17036:0.00368)0.204.3:5.4E-4,UltDe184:5.5E-4)0.982.37:5.4E-
4)0.810.41:0.0081)0.777.45:0.00741)0.442.10:5.4E-
4)0.040.1:0.01139,(Ult17041:0.03552,Ult17055:0.01581)0.833.51:0.00885)0.8
84.68:0.0154)0.657.9:0.00429,(UltDe201:0.00814,(Ult17042:0.0037,((UltDe19
3:0.0,UltDe192:0.0):5.5E-
4,(Ult17043:0.01451,(((UltDe195:0.00742,Ult17044:0.00746)0.313.6:5.4E-
4,(((UltDe200:0.00753,UltDe202:0.01141)0.758.54:0.00367,((UltDe194:0.0,Ul
tOr273:0.0):0.01548,UltOr272:0.00767)0.766.42:0.00359)0.882.67:0.00746,((
UltMa107:0.00763,(UltOr274:0.00753,(UltDe196:0.01151,(UltMa108:0.0152,Ult
17045:0.0075)0.742.29:0.00384)0.760.43:0.0037)0.788.43:0.00368)0.895.78:0
.00762,((UltDe198:0.00375,UltOr271:0.01527)0.927.60:0.00243,UltDe199:0.00
245)0.927.61:0.00245)0.233.6:5.5E-
4)0.849.50:0.00367)0.873.65:0.00368,UltDe197:5.4E-
4)0.793.44:0.0054)0.889.66:0.01028)0.604.10:5.5E-
4)0.936.61:0.01459)0.973.32:0.02873)0.788.44:0.00947,UltDe206:0.06487)0.0
85.4:0.0061,UltDe191:0.05723)0.883.67:0.02886)0.368.4:0.02885,((UltNitr7:
0.0,UltNitr8:0.0,UltGa285:0.0):0.02865,(UltAlte8:0.00715,UltNitr6:0.00401
)1.000.413:0.17948)0.940.64:0.04895)0.852.57:0.06572)0.301.5:0.01854)0.82
6.40:5.5E-
4)0.763.56:0.00396)0.771.58:0.00417)0.807.32:0.00827)0.881.67:0.01295,(((
Ult16122:0.08201,((Ult16486:0.01369,(Ult16485:0.0536,(Ult16484:0.01135,Ul
tAc861:0.00758)0.775.49:0.00553)0.780.61:0.00947)0.956.53:0.03073,Ult1648
8:0.03034)0.674.12:0.01208)0.905.52:0.02446,Ult17235:0.09996)0.501.7:0.00
945,(Ult16489:0.00721,Ult16490:0.00793)0.968.46:0.03973)0.799.52:0.01339)
0.763.57:0.00721,(((Ult16100:0.00373,Ult16099:0.00367)0.879.62:0.00834,(U
lt16098:0.01526,(((((Ult16095:0.00746,Ult16094:0.00744)0.770.66:0.00368,(
UltAc732:0.00369,(UdnBac63:0.00367,UltAc731:0.02714)0.757.37:0.00374)0.32
5.7:5.5E-4)0.862.55:5.3E-4,((Ult16091:0.01142,((UltHydr9:5.4E-

4,Ult16092:0.01908)0.892.50:0.0075,(UltAc733:0.00763,UltAc734:0.00762)0.7
75.50:0.00371)0.780.62:0.00371)0.783.56:0.00379,Ult16090:0.0037)0.969.43:
0.019)0.319.8:0.00368,Ult16093:0.00737)0.871.61:0.00772,(Ult16096:5.4E-
4,UltAc735:0.03954)0.412.5:0.00747)0.733.21:0.00364)0.951.57:0.01912)0.85
0.62:0.01231,((UltAc742:0.03293,(UltAc741:0.04397,(UltAc740:0.00747,(Ult1
6111:0.00759,Ult16110:0.01519)0.768.36:0.00383)0.732.16:0.00415)0.818.30:
0.01278)0.987.57:0.03645,((((Ult16101:0.00408,(Ult16102:0.01196,(Ult16105
:0.00306,(Ult16103:0.01539,UltAc736:0.00769)1.000.414:5.4E-
4)0.065.8:0.0038)0.931.55:0.01427)0.913.63:0.01181,((Ult16104:0.03229,Ult
Ac737:0.02037)0.877.72:0.01129,(UltAc738:0.00925,(UltHyd10:0.00544,UltAc7
39:0.00569)0.370.5:0.00372)0.883.68:0.0101)0.815.42:0.00705)0.232.6:0.009
04,Ult16109:0.03453)0.744.28:0.00441,(Ult16097:0.04218,(Ult16107:0.01417,
(UltB2754:0.01476,(Ult16106:0.00797,Ult16108:0.02727)0.788.45:0.00566)0.8
75.63:5.5E-
4)0.853.47:0.01278)0.811.40:0.00761)0.413.10:0.00993)0.715.12:0.00524)0.9
87.58:0.03371)0.897.54:0.0159)0.847.66:0.01156)0.950.48:0.02285,((((Ult16
085:0.02445,UltAc726:0.00288)0.966.62:0.03709,(((UltAc715:0.01914,Ult1605
8:0.03121)0.191.5:5.5E-
4,(((Udntfde9:0.00755,UltAc728:0.00754)0.779.52:0.00367,((UltrS280:0.0154
6,(((Ult16069:5.5E-4,(Ult16066:5.3E-4,(Ult16065:5.4E-4,(Ult16064:5.4E-
4,UltAc718:0.01135)0.210.9:0.00369)0.806.28:0.00367)0.873.66:0.00369)0.91
7.65:5.5E-4,((Ult16067:0.00374,(Ult16062:5.5E-
4,Ult16088:0.00366)0.790.55:0.00364)0.860.42:0.00712,(Ult16068:0.00751,(U
lt16055:0.0,UltHol26:0.0):0.02305)0.897.55:5.3E-
4)0.940.65:0.01479)0.805.38:0.01153,UltAc725:0.02383)0.888.65:0.01119)0.8
36.35:0.00742,(Ult16057:5.4E-4,(((UltrS279:5.5E-
4,Ult16052:0.0037)0.612.10:5.2E-
4,(Ult16051:0.01896,Ult16087:0.01537)0.758.55:0.00368)0.789.43:0.00368,Ul
tAc717:0.0114)0.796.37:0.0037)0.827.42:5.3E-
4)0.916.72:0.00745)0.788.46:0.00353,(Ult16053:0.05109,((Ult16894:0.00715,
(UltAc713:0.0037,Ult16076:5.5E-4)0.934.58:0.01113)0.907.65:5.4E-
4,(((Ult16056:0.00374,(((((Ult16082:0.01132,(UltAc723:0.0037,UltAc724:0.0
1139)0.749.52:0.00372)0.886.79:0.00764,(Ult16059:0.0233,Ult16079:5.3E-
4)0.960.55:0.01528)0.884.69:0.01009,Ult16077:0.01033)0.842.64:0.00959,Ult
Ac711:0.00424)0.877.73:0.01165,Ult16061:0.01487)0.768.37:0.00415)0.902.70
:0.00774,((Ult16078:0.0228,(UltAc712:5.5E-
4,Ult16054:0.00369)0.919.67:5.5E-
4)0.978.31:0.01902,(Ult16083:0.0053,((Ult16075:0.01514,((Ult16060:5.5E-
4,((((Ult16070:5.5E-4,UltAc719:0.00744)0.914.68:0.00741,UltAc721:5.5E-
4)0.941.59:5.3E-
4,(UltAc720:0.00381,Ult16071:0.0114)0.269.5:0.0037)0.829.47:0.00745,(Ult1
6074:5.5E-
4,((UltAc722:0.01152,Ult16072:0.00361)0.872.66:0.00794,(Ult16073:0.02302,
Ult16080:5.4E-
4)0.663.9:0.00727)0.950.49:0.01523)0.940.66:0.01526)0.943.57:0.01951)0.83
2.42:0.00341,Ult16084:0.01121)0.759.55:0.00372)0.793.45:5.5E-
4,Ult16081:0.01874)0.927.62:0.0142)0.876.54:0.01018)0.822.34:5.4E-
4)0.920.80:0.00747,(Ult16063:0.02782,(UltAc729:0.00339,UltAc730:0.00784)0
.314.5:0.00375)0.910.64:5.3E-
4)0.820.28:0.00386)0.921.75:0.01117)0.960.56:5.4E-
4)0.786.53:0.00734)0.894.50:0.01047,((UltAc716:0.00182,Ult31244:0.02137)0
.909.53:0.01311,(UltAc714:0.03526,(Ult16086:0.00639,UltAc727:0.01228)0.97
0.46:0.0244)0.776.50:0.00715)0.902.71:0.00934)0.546.7:0.00467)0.912.78:0.
02437,((((Ult14839:0.00919,Ult14840:0.03105)0.955.43:0.02204,(UltAc274:0.

04847,((Ult14856:0.0,UltAc271:0.0):0.01982,(Ult14855:0.01164,((UltAc270:5
.5E-4,Ult14854:5.5E-4)0.862.56:5.5E-4,(Ult14853:5.5E-
4,Ult14857:0.0037)0.860.43:0.00371)0.763.58:0.00334)0.890.70:0.01175)0.90
8.66:0.01973)0.892.51:5.4E-
4)0.771.59:0.00373,(((((UltAc275:0.06499,(Ult14841:5.5E-
4,(Ult14842:0.0,Ult14843:0.0):5.5E-
4)0.942.60:0.02577)0.797.38:0.01734,((UltAc272:5.4E-
4,UltAc273:0.03446)0.979.41:0.04954,(((Ult14844:0.00344,(UltAl199:0.01904
,Ult14845:5.5E-4)1.000.415:5.5E-
4)0.342.5:0.00856,(Ult14847:0.00741,Ult14846:5.4E-
4)0.988.58:0.03504)0.513.4:0.01614,(UltHydr8:0.01858,(UltAc266:5.4E-
4,(Ult14848:0.02697,(Ult14849:0.00369,UltAc265:0.01125)0.962.60:5.3E-
4)0.804.40:0.00402)0.999.89:0.03997)0.448.7:5.3E-
4)0.982.38:0.03694)0.790.56:0.01246)0.680.11:0.00661,(((((UltOr243:0.0142
4,(Ult14824:0.02731,(Ult14823:0.00707,(Ult14822:0.00745,(Ult14821:5.3E-
4,(UltAc259:0.00373,UltOr242:0.0037)0.909.54:0.00748)0.926.75:0.00749)0.8
99.66:5.4E-
4)0.963.52:0.02402)0.839.51:0.0088)0.699.19:0.01162,Ult14825:0.01639)0.93
3.63:0.01792,(Ult14829:0.00369,(Ult14827:0.00751,Ult14828:0.0231)0.872.67
:0.0075)0.752.44:0.00437)0.887.68:0.01489,((Ult14837:0.04041,(((UltAc262:
0.04688,UltAc263:5.5E-
4)0.870.46:0.00714,((Ult12963:0.03879,Ult14831:0.00437)0.990.48:0.0317,(U
lt14834:0.01125,(Ult14830:0.01907,Ult14835:0.01541)0.837.37:5.5E-
4)0.908.67:0.01214)0.828.40:0.00704)0.307.3:0.00369,Ult14836:0.03126)0.74
9.53:5.4E-4)1.000.416:5.5E-4,(Ult14833:5.5E-
4,(((UltAc260:0.00767,Ult14838:0.00758)0.915.59:0.00238,UltAc261:0.00244)
0.088.5:8.5E-
4,Ult14832:0.00177)0.996.53:0.02366)0.915.60:0.00341)0.900.54:0.02444)0.8
47.67:0.00865,Ult14826:0.00367)0.996.54:0.03519)0.666.12:0.00147,(UltAc26
8:0.05106,(Ult14852:0.01811,UltAc269:0.04737)0.815.43:0.02484)0.970.47:0.
03991)0.792.54:0.00861,(UltAc264:0.04463,(Ult14851:0.03133,(UltAc267:0.00
371,Ult14850:0.00743)0.857.63:0.00714)0.905.53:0.01146)0.845.62:0.0075)0.
741.34:0.00465)0.984.39:0.06993,(Ult17202:0.11866,(Ult15344:0.03692,((Ult
15693:0.00373,(Ult17173:0.04521,((((((((Ult17179:5.4E-
4,(Ult17144:0.00371,Ult17139:0.00373)0.803.33:0.00372)0.706.16:0.00375,(U
lt17126:0.0119,(Ult17146:0.0042,(Ult17145:5.5E-
4,Ult17181:0.00369)0.986.39:0.02678)0.450.8:0.00389)0.834.41:0.00692)0.77
5.51:0.00371,((Ult17128:0.0,Ult17180:0.0):5.5E-
4,(Ult17127:0.00374,Ult17154:0.01509)0.790.57:0.00366)0.785.52:0.00371)0.
794.45:0.00379,(Ult17147:0.0,Ult17148:0.0):0.01517)0.966.63:0.00208,((Ult
17150:0.0,Ult17167:0.0):0.00352,Ult17166:0.00763)0.975.43:0.01904)0.973.3
3:0.00174,(Ult17149:0.01907,((Ult17137:5.5E-
4,Ult17170:0.0037)0.845.63:0.0037,(Ult17162:0.00369,(Ult17141:0.00368,((U
lt17130:0.0,Ult17131:0.0,Ult17134:0.0,Ult17138:0.0,Ult17152:0.0,Ult17155:
0.0,Ult17159:0.0,Ult17163:0.0,Ult17169:0.0,Ult17182:0.0,Ult17183:0.0):5.5
E-4,(((Ult17143:0.01116,Ult17160:5.3E-
4)0.543.6:0.00369,Ult17132:0.00367)0.999.90:5.4E-
4,(Ult17177:0.01118,Ult17164:0.00751)0.595.4:5.5E-4)0.382.7:5.5E-
4)0.000.457:5.5E-4)0.000.458:5.5E-4)0.179.1:5.5E-4)0.985.46:5.4E-
4)0.223.1:0.00374)0.997.62:5.5E-4,Ult17153:5.4E-4)0.661.9:5.5E-
4,((Ult17158:0.00375,Ult17123:0.0306)0.919.68:0.00235,(Ult17135:0.00371,(
Ult17157:0.00369,(Ult17129:0.00369,(Ult17136:0.0,Ult17140:0.0,Ult17156:0.
0,Ult17168:0.0,Ult17172:0.0,Ult17174:0.0):5.5E-4)0.000.459:5.5E-
4)0.796.38:0.00371)0.918.65:0.00249)0.918.66:0.00243)0.897.56:0.00214,(Ul

t17125:5.4E-
4,(Ult17151:0.01115,((Ult17142:0.0,Ult17161:0.0):0.00371,(Ult17175:0.0074
5,((Ult17124:0.0,Ult17171:0.0,Ult17178:0.0):5.5E-
4,Ult17176:0.00369)0.810.42:5.5E-4)0.852.58:0.0037)0.210.10:5.4E-
4)0.869.56:0.00368)0.898.76:0.00205)0.821.34:0.00618)0.918.67:0.01227)0.7
56.49:0.00422,Ult17133:0.02683)0.939.55:0.03298)0.972.46:0.06176)0.852.59
:0.03012)0.558.7:0.02066)0.629.11:0.02084,Ult16089:0.05285)0.819.36:0.016
27)0.795.44:5.4E-4)0.898.77:0.01135)0.428.8:5.3E-
4)0.800.38:0.01312)0.802.42:0.01565,((CanChlor:0.03062,(Ult15282:0.0,Ult1
5283:0.0):0.01285)0.998.70:0.09473,(AdbBac11:0.0581,((((((UltB3873:0.0243
1,(UltB3870:0.00743,UltB3871:5.5E-
4)0.851.59:0.01177)0.891.52:0.01722,(UltB3872:0.02242,UltB3895:0.04025)0.
856.47:0.0155)0.813.39:0.01222,(Ult14392:0.05066,(UltrSo48:0.01892,(UltrS
o47:0.00358,UltEuba5:0.0115)0.948.64:0.02883)0.893.63:0.02134)0.775.52:0.
00855)0.903.60:0.01587,UltB3874:0.0028)0.933.64:0.02166,((((UltEuba3:0.01
645,(((UltB3841:0.01923,UltB3842:0.05182)0.804.41:0.00566,AccFacil:0.0074
2)0.344.8:0.00363,AccAmino:0.00417)0.951.58:0.02362)0.925.74:0.02348,UltB
3845:0.01148)0.545.12:0.01128,((((UltEuba4:0.01145,(UltrSo44:0.01137,(Ult
rSo43:0.00367,UltB3844:5.5E-
4)0.764.49:0.0037)0.773.47:0.00366)0.750.30:0.00533,(UltB3843:0.03513,Ult
B3840:0.04431)0.716.15:0.00528)0.983.31:0.02685,UltrSo45:5.4E-
4)0.827.43:0.00372,(RdpGlobi:0.01871,(UltB3848:0.02013,((((UltB3861:0.024
12,((UltB3855:0.01023,UltB3857:0.01006)0.775.53:0.00972,(UltB3849:0.02338
,(UltB3859:0.00732,UltB3860:0.00779)0.827.44:0.00777)0.880.64:0.01237)0.7
33.22:0.00379)0.789.44:0.00782,(UltB3858:0.01914,UltB3850:0.01541)0.773.4
8:0.00389)0.756.50:0.00353,UltFore8:0.0115)0.894.51:0.0117,(UltB3851:0.01
449,(UltB3846:0.02453,UltB3847:0.0291)0.892.52:0.0177)0.548.8:0.00924)0.7
53.44:0.0032)0.946.54:0.01548)0.853.48:5.2E-4)0.788.47:5.4E-
4)0.933.65:0.01911,(UltB3875:0.02799,UltB3876:0.0264)0.384.8:0.0046)0.941
.60:0.0191)0.639.9:0.00319,((UltB3853:0.02288,((UltB3008:0.0,UltB3852:0.0
):0.00369,AcsOrgan:5.5E-
4)0.773.49:0.00431)0.976.36:0.02068,((UltB3887:0.00926,Ult31168:0.11088)0
.998.71:0.08134,(((((UltrSo46:0.0079,(AdsSibir:0.01118,AdsTundr:5.4E-
4)0.886.80:0.01116)0.802.43:0.009,(UltrSo49:0.01668,(UltB3856:0.00381,Ult
11741:0.02299)0.930.58:0.02006)0.877.74:0.01513)0.827.45:0.00941,((((((((
(((BacEnr16:0.01518,(UltRose5:0.00386,((RsnLacus:0.0037,(UltB3881:0.00744
,UltB3896:0.00369)0.684.7:5.5E-
4)0.911.83:0.01149,RsnTerra:0.01548)0.764.50:0.00365)1.000.417:0.04073)0.
841.48:0.01061,(UltAnta8:0.0216,((((CraRoseu:0.03148,PcrRuber:0.00869)0.9
80.35:0.03929,UltB3883:0.00568)0.979.42:0.0329,RccSudun:0.01087)0.776.51:
0.01636,(UltrSo50:5.3E-
4,UltB3884:0.05757)0.856.48:0.01238)0.831.36:0.0169)0.965.50:0.03186)0.28
8.5:0.00829,((((DriWate2:5.4E-
4,(RsnStagn:0.0041,(RsnFrigi:0.01744,((RsnLudip:0.01212,(RsnGenom:0.01537
,((UltB3885:0.00772,(AtcBacte:5.4E-
4,(RsnGeno2:0.01607,RsnCervi:0.00326)0.901.66:0.01165)0.905.54:0.01136)0.
786.54:0.00345,UltB3464:5.4E-
4)0.801.46:0.00732)0.803.34:0.00916)0.774.61:0.01562,(RsnSpeci:0.00369,(R
snGilar:5.5E-4,RsnMucos:5.5E-4)0.700.22:5.5E-
4)0.941.61:0.02628)0.892.53:0.01736)0.864.64:0.01654)0.791.43:0.00334)0.9
81.44:0.01484,(PcrSpeci:0.00745,((((UltB3889:0.00764,UltB3890:0.01943)0.8
64.65:0.00753,UltB3891:0.01131)0.661.10:5.4E-
4,(BelMoabe:0.00385,UltB3892:0.01537)0.955.44:0.01543)0.917.66:0.00749,(R
snAquat:0.01521,UltB3888:0.04852)0.936.62:5.5E-

4)0.838.58:0.00375)0.891.53:5.4E-4)0.880.65:5.4E-
4,(UltB3882:0.01826,(UltB3886:0.00838,(AphPro50:0.02358,UltB3894:0.00716)
0.898.78:0.01084)0.753.45:0.00763)0.228.5:0.00423)0.869.57:0.01052,(RsnVi
nac:0.00817,(RsnAeril:0.00657,UltB3893:0.03184)0.965.51:5.5E-
4)0.817.29:0.01166)0.847.68:0.00108)0.898.79:0.01367,RsnRosea:0.01449)0.9
13.64:0.01782,(BacteG60:0.01204,RdvLipoc:0.07017)0.481.6:0.00563)0.916.73
:0.01713,UltB3880:0.03103)0.832.43:0.01158,UltB3879:0.01303)0.837.38:0.01
054,UltB3877:0.03494)0.777.46:0.01058,UltB3878:0.01226)0.714.14:0.00409,R
btFlocc:0.02607)0.262.6:0.01186,((AdpRubru:5.4E-
4,AdpAcido:0.01133)0.978.32:0.02982,((UltB3868:0.00742,(AdpCrypt:0.0,UltB
3869:0.0):5.5E-
4)0.958.68:0.01993,(((UltB3866:0.01118,UltB3867:0.01492)0.685.11:5.5E-
4,(UltB3864:0.00369,(UltB3863:0.01482,UltB3865:0.01872)0.872.68:5.4E-
4)0.670.9:5.5E-4)0.813.40:5.5E-
4,UltB3862:0.00368)0.879.63:0.01117)0.861.60:0.01385)0.887.69:0.01638)0.7
72.52:0.0113)0.579.9:0.00871,AcpRubri:0.02015)0.756.51:0.00539,UltB3854:0
.00594)0.734.27:0.01591)0.745.39:0.00657)0.569.9:0.00491)0.987.59:0.07217
)0.985.47:0.07989)0.865.63:0.03441)0.937.54:0.03227)0.732.17:0.00624)0.61
0.4:0.00222,((((((Ult27013:0.02499,((((((UltAc929:5.5E-
4,(UltAc927:0.00378,Ult27005:0.00378)0.651.12:5.5E-4)0.911.84:5.5E-
4,UltAc928:0.0194)1.000.418:5.4E-
4,((UltAc923:0.00756,((((UltAc925:0.01164,(UltAc930:0.0,Otu00185:0.0):0.0
1165)0.755.38:0.00358,PahggY91:0.00779)0.261.2:0.00767,UltAc922:5.4E-
4)0.946.55:0.01915,(UltAc924:5.5E-
4,UltAc921:0.0076)0.446.5:0.00378)0.916.74:5.4E-4)0.565.7:5.5E-
4,((UltAc926:0.00397,Otu00300:0.01515)0.733.23:0.00758,Tryyy098:5.3E-
4)0.918.68:0.00359)0.910.65:0.00364)0.944.48:0.01508,(UncUn216:0.01948,Ul
t27006:0.04443)0.769.49:0.00363)1.000.419:5.3E-4,(Otu00059:5.5E-
4,(UltAc931:0.00378,Z0114250:0.01928)0.642.8:5.5E-4)0.877.75:5.3E-
4)0.928.54:0.01613,((((UltAc934:0.0,Otu00620:0.0):0.17266,(((Ult27011:0.0
0859,(Ult27008:0.1756,((UncUn218:0.0016,((((UltAc933:0.03001,UncUn219:0.0
3026)0.992.54:0.06026,(((((Ult26970:0.04558,(Ult26973:0.00358,((Ult26971:
0.0,EggLenta:0.0):5.5E-
4,(Ult26972:0.00374,EggSinen:0.00759)0.626.11:5.3E-4)0.839.52:5.3E-
4)0.942.61:0.03325)0.808.43:0.01145,(((Ult26967:0.03481,Ult27009:0.10248)
0.280.2:0.01217,(Ult23955:0.01132,(Ult26965:5.5E-
4,(Ult26964:0.06021,Ult26963:0.03909)0.867.55:5.4E-
4)0.773.50:0.00372)0.863.61:0.01285)0.746.38:0.0133,Ult26935:0.02565)0.99
3.42:0.04279)0.839.53:0.00949,((((((Ult26942:0.02585,(Ult19734:0.01171,Ul
t26998:0.00729)0.979.43:0.02639)0.648.10:0.01213,((EggSpeci:0.03975,(UltB
6923:0.01334,Ult26974:0.00584)0.977.41:0.03328)0.773.51:0.00865,((Ult2693
8:0.0,Ult26961:0.0):0.01574,Ult26968:0.04005)0.893.64:0.01661)0.555.7:0.0
1235)0.489.4:0.01335,(Ult26936:0.00703,((Ult21364:0.00374,((Ult20103:0.0,
AhcCelat:0.0):5.5E-4,(Ult26962:0.00754,Ult26952:0.00374)0.579.10:5.5E-
4)0.960.57:5.5E-4)0.897.57:0.01122,(CooBact2:0.00737,Ult26960:5.5E-
4)0.192.4:0.00383)0.871.62:0.00847)0.602.7:0.01033)0.795.45:0.00552,((((U
lt26954:5.4E-
4,(Ult26959:0.01536,(((Ult26939:0.00202,Ult17560:0.01497)0.945.61:0.00196
,Ult26956:0.00375)0.885.53:5.5E-
4,(Ult26940:0.0,EtbMucos:0.0,Ult26957:0.0):5.5E-4)0.988.59:5.3E-
4)0.939.56:0.0114)0.831.37:5.5E-
4,((Ult26946:0.0,Ult26947:0.0,Ult26948:0.0,Ult26949:0.0,Ult26950:0.0):5.4
E-
4,Ult26951:0.00374)0.999.91:0.05247)0.249.7:0.0035,Ult26958:0.01934)0.942

.62:0.019,(((Ult26941:0.01096,(Ult26943:0.01899,(Ult26937:0.09561,(Ult269
45:0.09551,Ult26944:0.00883)0.875.64:0.01961)0.803.35:0.01254)0.972.47:0.
03072)0.705.12:0.00444,(BacNL444:0.0,BacNL445:0.0,PggHongk:0.0):0.0271)0.
974.28:0.02613,((Ult26985:0.03924,Ult26986:0.02703)0.931.56:0.02203,((Ult
20102:0.0191,(Ult20101:0.00745,((Ult26979:0.00371,Ult26975:0.01909)0.801.
47:5.5E-4,((Ult26977:0.0,Ult26978:0.0):5.5E-
4,Ult26976:0.02278)0.194.3:5.5E-4)0.893.65:5.3E-
4)0.734.28:0.00373)0.993.43:0.03297,(((Ult26981:5.5E-4,(Ult26984:5.5E-
4,Ult26982:5.5E-4)0.416.12:5.5E-4)1.000.420:5.5E-
4,(Ult26980:0.00375,Ult26983:0.02314)0.780.63:0.00374)0.968.47:0.01974,(U
lt26969:0.00372,UltCori2:5.5E-
4)0.968.48:0.01959)0.742.30:0.00251)0.900.55:0.01627)0.788.48:0.00828)0.3
72.4:0.01967)0.934.59:5.3E-4)0.813.41:5.4E-
4,Ult26955:0.03088)0.873.67:0.00864,(((((Ult26991:5.4E-
4,(Ult26988:0.01137,(Ult26993:5.5E-
4,((Ult26987:0.0,Ult26989:0.0,Ult26990:0.0,Ult26992:0.0):5.5E-
4,Ult26994:0.00375)0.743.42:5.5E-4)0.806.29:5.4E-
4)0.442.11:0.00373)0.973.34:0.03006,(Ult26997:0.02034,(Ult20104:0.05261,U
lt26995:0.03073)0.567.5:0.01465)0.888.66:0.02409)0.880.66:0.01795,Ult2699
6:0.07515)0.824.38:0.01223,CpcCurtu:0.05345)0.418.5:0.01319,((SlaHelio:0.
04537,SlaExigu:5.4E-
4)0.984.40:0.05425,((Ult26927:0.0036,Ult26928:0.00384)1.000.421:0.06752,(
Ult26933:0.04767,((Ult26934:0.03616,(Ult26932:0.0229,(SlaFaeci:0.0,Ult269
29:0.0):5.4E-
4)0.759.56:0.00425)0.862.57:0.00963,((Ult26930:0.0,Ult26931:0.0,Ult26953:
0.0):5.4E-
4,Ult26365:0.04338)0.765.36:0.00525)0.914.69:0.01597)0.737.40:0.0051)0.36
8.5:0.00149)0.990.49:0.05242)0.920.81:0.02392)0.679.14:0.005)0.973.35:0.0
3311,(GrtPamel:0.03436,DbrSpeci:0.02107)0.102.2:0.01385)0.822.35:0.01774,
UltGord2:0.04983)0.985.48:0.05029)0.449.16:0.00871,EtcCass4:0.01667)0.929
.63:0.02418,(Ult27007:0.02985,UncUn217:0.03778)0.605.9:0.00747)0.730.23:0
.0029)0.730.24:0.00529,UltAc932:0.02598)0.940.67:0.04344)0.187.5:0.02044)
0.940.68:0.02193,Ult27010:0.0168)0.900.56:0.02461,(UltAc939:5.4E-
4,((UltAc937:0.0681,UltAc938:0.02595)0.998.72:0.10666,Ult27021:5.5E-
4)0.685.12:0.02289)1.000.422:0.12037)0.893.66:0.02695)0.550.3:0.01867,(((
UltAc935:0.0152,Otu00912:5.4E-
4)0.999.92:0.08843,((((Ult27016:0.03097,Ult27019:0.01831)0.845.64:0.00932
,(Ult27018:0.02417,UltAc936:0.02769)0.821.35:0.00696)0.832.44:0.0076,(Iro
Bac22:0.02318,(Ult27020:0.01961,Ult27015:0.02748)0.740.41:0.00362)0.755.3
9:0.00415)0.784.46:0.00773,(Ult27014:0.01807,Ult27017:0.01504)0.389.5:0.0
0991)0.239.7:0.02058)0.035.6:0.01053,(((Ult26999:0.07708,Ult27001:0.00976
)0.933.66:0.03314,(Ult27000:0.01714,(((((((ApbVagi2:0.0,ApbVagin:0.0):0.0
3981,ApbSpeci:5.4E-
4)0.933.67:0.01144,Ult26906:0.00756)0.773.52:0.00374,(((Ult26902:0.0076,(
UltRu549:0.00375,OlsenUli:0.00756)0.001.4:5.5E-
4)0.828.41:0.00377,((Ult26903:0.00374,Ult26904:0.00384)0.937.55:0.01179,U
lt26905:0.01967)0.765.37:0.00352)0.856.49:5.3E-4,Ult26914:5.4E-
4)0.849.51:5.3E-
4)0.886.81:0.00765,(Ult26907:0.0038,(OlsProfu:0.00744,((UltCorio:0.02406,
(((ApbMinut:5.5E-
4,ApbFosso:0.00743)0.966.64:0.01553,(ApbRimae:0.01136,Ult26898:0.02737)0.
856.50:0.00746)0.858.57:0.00811,((Ult26901:0.0,Ult26900:0.0):0.02015,UltR
u550:0.01584)0.834.42:0.01063)0.909.55:0.01202)0.927.63:0.01562,Ult26911:
0.01115)0.850.63:0.00802)0.745.40:0.00407)0.748.38:0.00375)0.756.52:0.002

84,((Ult26908:0.00525,(UltRu548:0.08216,Ult26915:0.03505)0.890.71:0.02585
)0.916.75:5.4E-4,((((Ult26899:0.0804,(Ult26909:0.0,Ult26910:0.0):5.5E-
4)0.950.50:0.02111,(((Ult26922:0.02049,(((CcrGlome:0.01019,CcrGlom2:0.009
49)0.978.33:0.04063,((Ult26878:0.01233,((Ult26879:0.00736,Ult26880:0.0040
6)0.933.68:0.01639,(((((Ult26861:0.01124,(((((((Ult26856:0.01696,Ult2686
0:0.07285)0.849.52:0.01134,Ult26870:0.00845)0.416.13:5.4E-
4,Ult26865:0.01133)0.285.5:5.5E-4,Ult26877:0.00371)0.000.460:5.5E-
4,(Ult21293:0.04111,Ult26854:0.0222)0.965.52:0.0272)0.000.461:5.5E-
4,(Ult26847:0.0,Ult26848:0.0,Ult26849:0.0,Ult26852:0.0,Ult26853:0.0,Ult26
855:0.0,Ult26857:0.0,Ult26862:0.0,Ult26868:0.0,Ult26869:0.0,Ult26866:0.0,
Ult26863:0.0,Ult26875:0.0):5.5E-4)0.000.462:5.5E-
4,Ult26867:0.00372)0.243.4:5.5E-4,Ult26864:0.00371)0.000.463:5.5E-
4)0.223.2:5.5E-4,Ult26876:0.0037)0.144.5:5.5E-
4,(Ult26858:0.02706,Ult26859:0.03529)0.747.38:0.00415)0.444.13:5.4E-
4,CoiAerof:5.5E-4)0.299.8:5.3E-4,CoiAero2:5.5E-
4)0.851.60:0.00941)0.813.42:0.01096)0.962.61:0.02623,((Ult26884:0.00384,(
(Ult26851:0.0,Ult26874:0.0,Ult26882:0.0):0.00503,((Ult26883:0.0,Ult26886:
0.0):5.5E-4,(Ult26881:0.00382,Ult26885:0.00382)0.566.6:5.5E-
4)0.680.12:5.5E-
4)0.872.69:0.00987)0.867.56:0.00795,((((CoiSterc:0.0,CoiIntes:0.0):0.0075
6,((Ult26872:0.0,Ult26890:0.0,Ult26891:0.0):5.5E-4,Ult26889:5.5E-
4)0.793.46:5.4E-4)0.927.64:0.01923,(((Ult26887:5.5E-
4,(Ult26873:0.00375,(Ult26850:0.0,Ult26888:0.0,Ult26894:0.0):5.5E-
4)0.415.9:5.5E-4)0.857.64:5.5E-
4,(Ult26926:0.00375,(Ult26895:0.00375,(Ult26871:0.0,CcrSpeci:0.0):5.5E-
4)0.525.6:5.5E-
4)0.946.56:0.01138)0.997.63:0.04049,(Ult26896:0.034,CooBacte:0.03523)0.88
8.67:0.01933)0.840.56:8.5E-
4)0.921.76:0.01927,(Ult26892:0.02782,Ult26893:0.02762)0.912.79:5.4E-
4)0.857.65:0.00687)0.850.64:0.00939)0.861.61:0.01314)0.929.64:0.02133,Ult
Olsen:0.02047)0.755.40:0.00977)0.869.58:0.0091,(Ult26921:0.0317,(Ult26919
:5.5E-4,Ult26920:0.00374)0.937.56:0.01098)0.846.61:5.5E-
4)0.946.57:0.02806,(Ult26897:0.08834,(Ult26925:5.5E-4,(Ult26923:5.5E-
4,Ult26924:0.00375)0.903.61:0.00752)0.972.48:0.04526)0.893.67:0.03344)0.8
04.42:0.01215)0.845.65:0.00778,(Ult26912:0.04455,Ult26913:0.02181)0.850.6
5:0.00938)0.931.57:0.01562)0.763.59:0.00682)0.873.68:0.00813,(Ult26916:0.
01571,(Ult26917:0.00374,Ult26918:5.5E-4)0.995.61:0.04496)0.935.56:5.5E-
4)0.985.49:0.02789)0.892.54:0.01917)0.939.57:0.02832,Ult27002:0.01952)0.9
53.41:0.03379)0.752.45:0.00935)0.782.59:0.01141,Ult27012:0.04127)0.878.63
:0.01248)0.777.47:0.00805)0.958.69:0.0367,Ult27004:0.04424)0.986.40:0.056
95,((Ult27048:0.11974,(Ult27047:0.05128,(Ult27046:0.0215,(Ult27045:0.0203
8,Ult27044:0.01471)0.960.58:0.04216)0.851.61:0.04693)0.872.70:0.04929)0.8
97.58:0.04193,((Ult31354:0.01181,(Ult31353:0.03392,(Ult31351:0.0078,Ult31
352:0.01111)0.965.53:0.03224)0.179.2:0.02171)0.969.44:0.08455,(((UltPori3
:0.00788,(UltPorib:0.00426,UltPori2:0.03531)0.152.5:0.00742)0.808.44:0.01
057,UltPl555:0.00489)0.998.73:0.14709,((UltPl556:0.00821,UltPori4:0.01554
)0.957.51:0.03535,(UltPl557:0.00813,UltPl558:0.07096)0.348.8:0.02226)0.82
9.48:0.04462)0.977.42:0.09323)0.958.70:0.06444)0.561.9:0.01808)0.705.13:0
.01299,(((Ult26787:0.03801,Ult26788:0.02897)0.992.55:0.06664,(((Ult26786:
0.04877,UltDe230:0.01005)0.954.59:0.03136,(UltB4495:0.06588,Ult26789:0.07
619)0.714.15:0.00703)0.779.53:0.00729,((Ult26790:0.07433,(Ult26785:0.0338
,(Ult26783:0.0364,Ult26784:0.01044)0.829.49:0.00945)0.891.54:0.02129)0.95
1.59:0.03478,(UltAc916:0.0878,(Ult14325:0.07712,(((Ult26754:0.02686,(((Ul
t26745:0.01099,(Ult26772:5.5E-4,((Ult26773:5.4E-

4,((Ult26771:0.00369,Ult26770:5.5E-
4)0.892.55:0.00746,((Ult26738:0.03064,Ult26753:5.3E-
4)0.956.54:0.01407,(Ult26761:0.01492,Ult27034:5.5E-
4)0.903.62:0.00411)0.902.72:0.00421)0.853.49:0.00361)0.719.10:0.00738,Ult
26774:0.00361)1.000.423:5.4E-
4)0.878.64:0.00985)0.754.39:0.00613,(Ult26737:0.01945,UltRubr3:0.03328)0.
151.5:0.00793)0.707.9:0.00324,(Ult26777:0.02317,Ult26776:0.02714)0.756.53
:0.00381)0.769.50:0.0039)0.841.49:0.0075,(((((Ult26767:0.00721,Ult26768:0
.01163)0.980.36:0.02439,Ult26769:0.01212)0.753.46:0.00274,((Ult26759:0.08
815,(Ult26765:0.00543,(Ult26743:0.0201,(Ult15363:0.01201,(Ult15366:0.0,Ul
t16886:0.0,Ult26758:0.0):0.00324)0.981.45:0.03374)0.872.71:0.01771)0.521.
4:0.01515)0.961.52:5.5E-
4,((UltRubr5:0.01913,(Ult26760:0.00376,Ult26756:0.03086)0.745.41:0.00359)
0.895.79:0.01054,Ult26775:0.00841)0.475.11:0.00674)0.854.60:0.01021)0.926
.76:0.01152,(Ult26744:0.01479,(Ult26751:0.00747,(Ult26752:0.00369,Ult2676
4:5.5E-
4)0.819.37:0.00371)0.933.69:0.00201)0.915.61:0.00193)0.982.39:5.3E-
4,((((Ult26739:0.02365,Ult15367:0.08536)0.842.65:0.01166,(Ult26782:5.5E-
4,Ult26836:0.00369)0.966.65:5.4E-
4)0.865.64:0.00736,UltRubr6:0.00753)0.989.50:0.02772,(Ult26766:0.0,Ult267
81:0.0):0.00701)0.547.5:0.01191)0.934.60:0.01087)0.943.58:5.5E-
4,(((((Ult26735:0.01916,(Ult26780:5.5E-
4,(UltrS332:0.0037,(Ult26728:0.01895,UltrS333:0.00369)0.158.4:5.4E-
4)0.759.57:0.00369)0.743.43:0.00377)0.944.49:0.01942,(((Ult26731:0.02219,
((Ult26730:0.0037,(Ult26729:5.5E-4,Ult26778:0.00369)0.820.29:5.5E-
4)0.887.70:0.00774,(Ult26763:5.4E-
4,Ult15365:0.03068)0.734.29:0.00698)0.766.43:0.00462)0.844.68:0.01155,(Ul
t26741:0.01136,(Ult26736:0.01525,((Ult26732:5.4E-
4,UltAc915:0.03583)0.974.29:0.01917,((Ult26734:0.00752,(Ult26727:0.00762,
(Ult26726:0.00625,UltRubro:0.00622)0.810.43:0.0061)0.930.59:0.01145)0.772
.53:0.00372,Ult26733:0.01135)0.934.61:0.01139)0.746.39:0.00356)0.874.76:0
.00767)0.863.62:0.00838)0.915.62:0.01481,Ult26746:0.02355)0.758.56:0.0031
1)1.000.424:5.4E-
4,Ult26757:0.03124)0.454.11:0.00748,(Ult26749:0.0077,(Ult26742:0.02804,(U
ltRubr2:0.01222,Ult26762:0.01916)0.788.49:0.01596)0.757.38:0.00775)0.761.
42:0.00356)0.809.46:5.5E-4,(Ult26755:0.03145,(((UltrS334:5.5E-
4,Ult26740:0.00369)0.964.56:0.02075,Ult26750:0.03732)0.744.29:0.00181,(Ul
t26779:0.01891,(UltRubr4:0.0076,(UltrS335:0.01513,(Ult26748:0.0037,(Ult14
546:0.01509,Ult26747:0.00725)0.931.58:5.4E-4)0.879.64:5.3E-
4)0.877.76:0.0075)0.864.66:5.3E-
4)0.911.85:0.0114)0.887.71:0.00818)0.757.39:0.00357)0.807.33:0.00713)0.00
6.6:0.01228)0.861.62:0.02592)0.906.81:0.04004)0.909.56:5.4E-
4)0.944.50:0.03339)0.970.48:0.03587,((((Ult26602:0.06437,((Ult26621:0.051
19,(Ult26622:5.4E-4,(((UltAc885:0.0,UltAc886:0.0):5.5E-
4,UltAc887:0.00368)0.878.65:0.00747,(Ult26623:0.02732,Ult26620:0.01941)0.
741.35:0.00338)0.905.55:0.0074)0.808.45:0.00968)0.939.58:0.02223,((((Ult2
6696:0.02421,(FrhTherm:0.04235,((FmcAcidi:0.01183,AidFerro:0.02721)0.762.
65:0.00387,(Ult26694:0.01083,Ult26695:0.05917)0.646.9:0.00432)0.849.53:0.
01333)0.947.47:0.02652)0.892.56:0.01808,UltAc906:0.02978)0.664.7:0.01775,
(Ult26693:0.05418,((((((Ult26678:0.00371,Ult26677:5.5E-
4)0.987.60:0.02984,((Ult26608:0.02162,(UltAc880:0.03055,((UltAc879:0.0227
8,((Ult26611:0.02301,(Ult26604:0.02312,UltAct97:0.03543)0.734.30:0.00373)
0.772.54:0.00447,UltAct99:0.03024)0.139.7:0.00786)0.775.54:0.00918,Ult266
15:0.04875)0.374.8:0.01257)0.877.77:0.01951)0.890.72:0.01247,(Ult26612:0.

02541,(Ult26627:0.02852,((Ult26659:0.04041,((Ult26656:0.02041,(((((UltAc8
95:0.01508,((UltAc893:0.01115,UltAc892:5.3E-
4)0.433.10:0.00379,((BacEnr49:0.01169,UltAc896:0.02359)0.866.59:0.01106,(
Ult26644:0.01881,Ult26643:0.00394)0.518.7:0.00382)0.774.62:0.00371)0.774.
63:0.00423)0.876.55:0.00779,(Ult26652:0.01107,Ult26651:0.01591)0.869.59:0
.0076)0.801.48:5.5E-
4,((IluFlumi:0.02082,(((Ult26646:0.01893,Ult26645:5.5E-
4)0.757.40:0.00395,Ult26647:0.04825)0.097.6:0.00378,Ult26650:0.00348)0.74
8.39:0.00294)0.862.58:0.00785,((Ult26649:0.0034,(Ult26648:0.03237,((((Ult
Ac890:0.02618,(Ult26640:0.0129,((Ult26639:0.01231,UltB3954:0.05921)0.766.
44:0.00518,(Ult26606:5.5E-
4,Ult26638:0.00372)0.760.44:0.00533)0.860.44:0.01158)0.896.52:0.01534)0.6
59.10:0.01185,((BacEnr47:0.00728,Ult26633:5.4E-
4)0.979.44:0.02905,((Ult26453:0.0,Ult26630:0.0,Ult26635:0.0,Ult26642:0.0)
:0.0289,(Ult26632:0.05645,(MaiMet26:0.0,Ult26631:0.0):0.03625)0.795.46:0.
00995)0.846.62:0.01372)0.858.58:0.01367)0.869.60:0.01282,((UltAc891:5.5E-
4,(Ult26641:0.00504,Ult26634:0.03539)0.816.40:0.01044)0.742.31:0.00749,Ba
cEnr48:5.5E-
4)0.779.54:0.00398)0.721.8:0.00213,(Ult26636:0.00497,Ult26637:0.01074)0.9
62.62:0.02073)0.935.57:0.01184)0.707.10:5.5E-
4)0.934.62:0.01199,UltAc894:0.01837)0.769.51:0.0036)0.854.61:5.5E-
4)0.000.464:0.00357,Ult26674:0.03082)0.887.72:0.01358,((Ult15400:0.05677,
Ult26655:0.00404)0.966.66:0.02911,(Ult26654:0.03057,Ult26653:0.01323)0.88
3.69:0.016)0.759.58:0.00613)0.917.67:0.0144)0.740.42:0.00407,Ult26664:0.0
3323)0.705.14:0.0073)0.125.5:0.00601,Ult26660:0.01781)0.868.52:0.0121)0.8
76.56:0.01314)0.818.31:0.00884)0.870.47:0.01524)0.304.7:0.00776,((Ult2661
9:0.05254,((UltAc888:0.0052,(UltAc889:0.0,UltMic12:0.0):0.02204)0.933.70:
0.02782,(Ult26629:0.05027,IamMajan:0.01219)0.968.49:0.03092)0.626.12:5.4E
-
4)0.662.7:0.00784,(((Ult26618:0.01911,((Ult26661:0.01172,(Ult26614:0.0037
3,(UltAc881:5.4E-4,((Ult26624:0.04,(Ult26625:5.5E-4,Ult26626:5.5E-
4)0.782.60:5.4E-
4)0.802.44:0.00543,(UltFi100:0.02151,Ult26628:0.00582)0.875.65:0.01201)0.
983.32:0.02633)0.765.38:0.00375)0.776.52:0.0033)0.875.66:0.01538,(UltAc88
2:0.03559,UltAc884:0.02355)0.367.11:0.01305)0.971.44:5.4E-
4)0.883.70:5.3E-
4,(Ult26617:0.04352,(Ult26616:0.01525,(Ult26607:0.07035,UltSlu34:0.01508)
0.776.53:0.01171)0.823.41:0.00976)0.858.59:0.0117)0.899.67:0.01137,(UltAc
883:0.02627,(Ult26605:0.04517,Ult26663:0.01054)0.855.60:0.01164)0.792.55:
5.5E-4)0.836.36:0.00954)0.915.63:0.01979)0.662.8:5.5E-
4,(UltAct98:0.07416,(Ult26680:0.02337,Ult26679:0.00336)0.978.34:0.03808)0
.431.9:0.01617)0.862.59:0.01377,((UltAct96:0.01826,(Ult16630:0.03599,Ult2
6603:0.028)0.966.67:0.03561)0.955.45:0.03447,((Ult14326:0.00751,Ult26613:
5.4E-
4)0.831.38:0.01284,(Ult26609:0.04924,Ult26610:0.04979)0.974.30:0.04646)0.
774.64:0.00833)0.734.31:0.00948)0.853.50:0.01222,((Ult15705:0.00344,UltAc
898:0.00393)0.989.51:0.04759,(((Ult26673:0.02712,(Ult26671:0.00773,(UltB2
360:0.01408,Ult26670:0.01143)0.866.60:0.01137)0.314.6:0.00899)0.528.6:5.4
E-
4,Ult26672:0.0625)0.774.65:0.00411,(((Ult26666:0.00503,(Ult26665:0.00455,
(Ult26668:5.5E-
4,UltAc897:0.03918)0.991.49:0.03018)0.571.6:0.01039)0.790.58:0.00778,Ult2
6667:0.01083)0.938.44:0.01566,Ult26669:0.06355)0.788.50:0.00696)0.866.61:
0.01413)0.943.59:0.03021)0.755.41:0.00721)0.738.21:0.01167)0.880.67:0.017

85,((UltAc904:0.03738,Ult28335:0.11732)0.686.12:0.00767,(((UltAc899:0.022
85,Ult26691:0.0111)0.872.72:5.4E-
4,(((Ult26686:0.02583,UltPro38:0.04154)0.703.15:0.00246,((Ult26675:0.0077
6,UltAc903:0.02719)0.100.4:0.00346,((((Ult26687:0.02079,(Ult26688:0.02039
,UltBani5:0.02205)0.284.2:0.0037)0.962.63:0.02815,UltAc900:0.03867)0.922.
78:0.02272,(UltAc901:0.0274,Ult26689:0.00769)0.823.42:0.01194)0.090.5:0.0
0961,UltAc902:0.00623)0.983.33:0.0355)0.754.40:0.00438)0.873.69:0.008,Ult
26676:0.0115)0.777.48:0.00388)0.000.465:0.00739,((Ult26692:0.03573,(Ult26
681:0.01946,Ult26682:0.01971)0.756.54:0.00338)0.849.54:0.00802,((UltAc874
:5.4E-
4,((Ult26683:0.01414,Ult26684:0.00956)0.989.52:0.03973,Ult26685:0.00769)0
.822.36:0.02994)0.989.53:0.0365,((Ult26657:0.04117,Ult26658:0.02821)0.913
.65:0.0209,(UltAc905:0.05135,(Ult26690:0.00893,UltFor73:0.01361)0.289.1:0
.00694)0.937.57:0.02106)0.827.46:0.01135)0.703.16:0.01142)0.071.6:5.4E-
4)0.934.63:0.02809)0.947.48:0.0353)0.765.39:0.00607)0.976.37:0.03824)0.87
6.57:0.01886,(Ult11744:0.04032,((((Ult26711:0.01576,(UltAc907:0.04505,(Ul
tB4800:5.4E-
4,UltActi3:0.03477)0.652.8:0.00728)0.872.73:0.01191)0.926.77:0.01531,(Ult
26707:0.02332,(((UltAc910:0.03945,(Ult26709:0.03313,Ult26710:0.02461)0.13
6.5:0.01061)0.095.4:0.00479,((Ult26708:0.01924,UltAc909:0.00749)0.905.56:
0.015,UltActi4:0.05835)0.938.45:0.02729)0.936.63:0.02236,(((Ult26697:0.0,
Ult26698:0.0,Ult26699:0.0):5.5E-
4,(UltMa110:0.01134,Ult26700:0.04471)0.763.60:0.00377)1.000.425:5.4E-
4,Ult26701:0.0036)1.000.426:0.11059)0.827.47:0.01468)0.832.45:0.00775)0.7
69.52:0.00742,((UltB4799:0.03578,(((UltAc908:0.01223,Ult26706:0.01854)0.5
31.3:0.00767,Ult26705:0.01032)0.749.54:0.00437,((Ult26702:0.02016,Ult2670
4:0.03115)0.934.64:0.02705,Ult26703:0.02009)0.745.42:0.00743)0.963.53:0.0
3)0.969.45:0.03113,UltActi2:0.05848)0.756.55:0.00739)0.029.4:0.00916,Ult2
6712:0.02198)0.866.62:0.01799)0.958.71:0.03001)0.843.48:0.01189,((((Ult27
037:0.00944,((Ult27030:0.0,Ult27041:0.0):5.5E-
4,(((Ult27039:0.01437,(UltrS339:0.01818,UltOr297:0.00853)0.727.20:0.01155
)0.770.67:0.00463,(Ult27038:0.02702,(Ult27033:5.5E-
4,(Ult27036:0.00369,(Ult27035:0.0,Ult28397:0.0):5.5E-
4)0.867.57:0.00369)0.897.59:5.5E-
4)0.914.70:0.01132)0.838.59:0.00759,((Ult27028:0.00488,(Ult27040:0.01407,
(Ult31343:0.03993,(Ult27031:0.00285,Ult27032:0.02794)0.962.64:0.02421)0.9
27.65:0.01664)0.146.5:0.00358)0.939.59:0.01415,((RrbRadio:0.03586,Ult2702
7:0.01634)0.700.23:0.01126,Ult27029:0.01079)0.744.30:0.0042)0.928.55:0.01
131)0.877.78:5.5E-
4)0.880.68:0.00941)0.822.37:0.01503,RrbXylan:0.01369)0.172.8:0.01414,(Rrb
Taiwa:0.02591,(UltAc940:0.0228,(Ult27025:0.01489,Ult27026:5.4E-
4)0.826.41:0.01269)0.917.68:0.02504)0.339.10:0.01775)0.963.54:0.04088,(Th
uAlbum:0.02828,(((((Ult26840:5.3E-
4,Ult15370:0.07521)0.783.57:0.00367,(Ult26839:0.00374,Ult26842:0.00373)0.
783.58:0.00379)0.957.52:0.01629,(Ult26843:0.02194,(Ult26838:0.01593,(Ult2
6837:0.01226,Ult26833:0.03372)0.286.5:0.00327)0.913.66:0.01172)0.944.51:5
.4E-
4)0.839.54:0.01158,((((((UltAc917:0.01033,(((UltEnd11:0.01582,CxbWoese:0.
01155)0.735.26:0.00344,(Ult26808:0.00753,(Ult26805:0.03116,Ult26807:0.004
17)0.933.71:0.01604)0.847.69:0.008)0.859.51:0.00769,((Ult26804:0.02251,(P
aiAmeri:0.02169,UltPatul:0.01906)0.992.56:0.04464)0.340.6:0.01052,((Ult26
803:0.01972,(Ult26802:0.00376,Ult26801:0.00752)0.764.51:0.00356)0.884.70:
0.00795,(((Ult26794:0.01557,((((((SbbcSoli:0.0,Ult26795:0.0,SbbSpeci:0.0)
:5.5E-4,SbbPauli:0.00758)0.837.39:0.00369,Ult26799:5.2E-

4)0.833.52:0.00369,Ult26800:0.00744)0.784.47:5.3E-
4,Ult26831:0.03488)0.898.80:0.00758,((Ult26797:0.0112,(Ult26793:0.01143,(
Ult26792:0.00374,Ult26791:0.01132)0.785.53:0.00378)0.923.88:5.4E-
4)0.847.70:0.0074,Ult26798:0.01943)0.770.68:0.00365)0.873.70:0.00744)0.85
1.62:0.00757,Ult26811:0.0585)0.196.7:5.4E-
4,Ult26796:0.00755)0.964.57:0.01517)0.769.53:0.00462)0.792.56:0.0065)0.87
5.67:0.01031)0.788.51:0.00582,Ult26841:0.01141)0.769.54:0.00371,(UltThe16
:0.01959,UltrS336:0.01168)0.770.69:0.00356)0.750.31:0.00437,(Ult26826:0.0
3593,(Ult26827:0.00787,Ult26828:0.04918)0.737.41:0.00424)0.831.39:0.01197
)0.789.45:0.00303,((Ult26806:0.00765,UltAc918:0.01532)0.789.46:0.00362,(U
lt26825:0.01116,UltrS337:0.03148)0.814.34:5.4E-4)0.986.41:5.3E-
4)0.860.45:0.00724,((UltRubr8:0.01,Ult26829:0.02687)0.841.50:0.0015,((Ult
26835:0.09358,Ult26834:5.4E-
4)0.912.80:0.01491,((((((Ult26815:0.00612,Ult26814:0.00519)0.993.44:0.05
11,((Ult26820:0.01133,Ult26821:5.5E-
4)0.708.18:0.00536,(Ult26819:0.02413,UltRubr7:0.00776)0.836.37:0.01431)0.
524.6:0.01835)0.795.47:0.01345,UltAc920:5.4E-
4)0.532.7:0.00373,Ult26822:5.3E-
4)0.966.68:0.02193,UltAc919:0.01353)0.758.57:0.00685,Ult26813:0.01345)0.9
11.86:0.01369,(((((Ult15368:0.00689,Ult15369:0.07425)0.697.4:0.04611,((Ul
t26725:0.03496,Ult17165:0.04804)0.052.3:5.4E-
4,(Ult26832:0.01536,(Ult15364:0.0238,Ult26830:0.01153)0.953.42:0.01972)0.
742.32:0.00357)0.459.8:5.4E-
4)0.994.56:0.03127,Ult26817:0.01939)1.000.427:5.3E-
4,UltrS338:0.00718)0.836.38:0.00759,(Ult26818:0.00804,Ult26816:0.03941)0.
848.57:0.00736)0.763.61:0.00378)0.852.60:0.00797)0.936.64:0.02005)0.936.6
5:0.01964)0.858.60:0.00902)0.936.66:0.01917,(Ult26824:0.01955,(Ult26809:0
.01959,Ult26823:0.01567)0.935.58:0.01579)0.857.66:0.00872)0.828.42:0.0129
9)0.974.31:0.041)0.893.68:0.02264)0.887.73:0.02255,(((UdnActi3:0.0668,(Ul
t26598:0.09974,(((UltAct36:0.00812,((Ult17122:0.00767,(UltrS318:0.00762,U
lt26146:0.00357)0.744.31:0.00366)0.950.51:0.01606,Ult26145:0.01132)0.848.
58:0.01138)0.986.42:0.04245,(Ult27042:0.02753,Ult27043:0.01536)0.783.59:0
.0154)0.886.82:0.02943,(Ult26601:0.0896,Ult26600:0.05465)0.019.3:0.02692)
0.000.466:0.0298)0.593.6:0.04117)0.819.38:0.0276,((TbsBisp2:0.03526,TnpCh
ro3:0.03426)0.905.57:0.02851,(Ult26595:0.06125,(((Ult17121:0.02759,(StpSp
e69:5.5E-
4,((StpGlob2:0.0,StpLurid:0.0,StpRose5:0.0,StpMauve:0.0,StpSpe62:0.0,StpE
niss:0.0,StpAlbi2:0.0,StpPeuce:0.0,StpKurss:0.0):5.5E-
4,((StpHachi:0.0,StpNetro:0.0,StpNetr2:0.0,StpEuroc:0.0,StpOliv4:0.0):0.0
037,(StpSpe78:0.11398,(((((StpBeiji:0.00377,((StpAlbo2:0.0,StpAlbol:0.0):
0.00368,KitGansu:0.00376)0.791.44:0.00372)0.805.39:0.00369,SrdAnmyo:0.003
7)0.943.60:5.5E-4,(StpCandi:0.0,KitMedio:0.0,SrdMelan:0.0):5.4E-
4)1.000.428:5.5E-
4,((((StpSpe77:0.00368,((KitPhosa:0.00368,((KitCochl:0.00369,KitGrise:0.0
0369)0.948.65:5.5E-4,(KitVirid:0.00369,KitCysta:0.00369)0.915.64:5.5E-
4)0.000.467:5.5E-4)0.000.468:5.5E-
4,(StpPsamm:0.0,KitSetae:0.0,KitPhos2:0.0,StpSpe76:0.0,KitSampl:0.0,StpAv
ell:0.0,KitAzati:0.0,KitKifun:0.0,KitKifu2:0.0):5.5E-4)0.705.15:5.5E-
4)1.000.429:5.1E-4,(((StpCitri:0.0,SrdOryza:0.0):5.5E-
4,(KitNiiga:0.0,KitArbor:0.0):0.0037)1.000.430:5.4E-
4,SrdNeutr:0.00363)0.129.6:0.00371)0.868.53:0.00362,(So0Actin:0.0,SrdCarb
o:0.0,StptAlni:0.0):0.0037)0.102.3:5.4E-4,SrdJiang:5.5E-
4)0.910.66:0.0036)0.708.19:0.00743,(((StpHypol:0.0,StpSpe56:0.0,StpCreme:
0.0,StpRose4:0.0,StpKatsu:0.0,StpGobit:0.0,StpSpiro:0.0,StpSpir2:0.0):5.5

E-4,((((((((StpFlave:5.5E-
4,StpVastu:0.00742)0.847.71:0.0037,((StpLong2:0.0,StpHygro:0.0,StpFlavi:0
.0):5.5E-
4,((StpSpe35:0.00381,StpSpe53:0.01525)0.703.17:0.00757,((StpClavi:0.00369
,(((StpOliv2:0.00387,StpCorc2:0.02273)0.793.47:0.00368,((StpSpe61:0.00369
,(((StpCorch:0.0,StpLongw:0.0,StpLucen:0.0,StpNiveo:0.0,StpRegen:0.0,StpT
umes:0.0,StpPruni:0.0,StpSpe43:0.0,StpLinco:0.0,StpSpe44:0.0,StpGri11:0.0
,Ult25921:0.0,StpCinn2:0.0,StpCyane:0.0,StpGlome:0.0,StpChart:0.0,StpAver
m:0.0,StpPanay:0.0,StpCurac:0.0,StpSpe59:0.0,StpOliv3:0.0,StpMirab:0.0,St
pPlumb:0.0):5.5E-4,StpArgil:5.5E-4)0.000.469:5.5E-
4,Ult25918:0.00369)0.000.470:5.5E-4)0.600.8:5.5E-4,TcmCaesi:5.5E-
4)0.599.6:5.5E-4)0.352.5:5.5E-4,StpAure2:5.5E-4)0.579.11:5.5E-
4)0.216.6:5.4E-
4,((StpMutom:0.0,StpSpe70:0.0):0.01505,(((StpSpe72:0.0037,(StpSpe74:0.003
69,StpCalif:0.00744)0.277.7:5.5E-4)0.912.81:5.5E-
4,(StpLave3:0.0,StpFulvi:0.0,StpGris8:0.0,StpGlobi:0.0,StpPluri:0.0,StpMi
chi:0.0,StpFlori:0.0,StpCyan2:0.0,StpSpe73:0.0,KitParac:0.0,StpPolya:0.0,
StpLong4:0.0,StpSpe75:0.0,Udntfd14:0.0):5.5E-4)0.786.55:5.5E-
4,(StpBacil:0.01113,StpSpe71:0.02295)0.894.52:5.4E-4)0.860.46:5.5E-
4)0.857.67:0.00369)1.000.431:5.4E-4)0.877.79:0.00359)0.234.6:5.4E-
4)0.863.63:0.00374,(StpSpe48:5.5E-
4,StpSpe49:0.00742)0.837.40:0.00369)0.761.43:5.5E-4,StpSpe45:5.5E-
4)0.535.8:5.3E-4,StpShiod:5.5E-4)0.535.9:5.5E-
4,(StpPurpu:0.0,StpBungo:0.0,StpLivid:0.0,StpClavu:0.0,StpAlbos:0.0,StpSp
e80:0.0):5.5E-4)0.568.7:5.4E-
4,((StpSpe15:0.0,StpSpe47:0.0,StpGlob3:0.0,StpBikin:0.0,StpSpe68:0.0,StpP
hae8:0.0,StpRose7:0.0,StpLater:0.0,StpExfol:0.0,StpNarbo:0.0,StpCasta:0.0
):5.5E-4,StpRose6:5.5E-
4)0.851.63:0.00369)0.917.69:0.00204,(((((StpNivei:0.0,StpSpe41:0.0):5.4E-
4,(((StpFulvo:0.0,StpDias2:0.0,StpFilip:0.0,StpSpe40:0.0,StpRoseo:0.0,Stp
Spe42:0.0,StpEchin:0.0,StpEchi2:0.0,StpGri12:0.0):5.5E-
4,StpPunic:0.00744)0.844.69:5.4E-4,(StpGri10:5.5E-
4,(StpCosta:0.0,StpGris9:0.0,StpSpe39:0.0,StpLanat:0.0,StpSpe51:0.0,StpSp
e50:0.0):5.5E-
4)0.985.50:0.01509)0.899.68:0.0075)0.828.43:0.00369,StpSpe46:5.3E-
4)0.861.63:5.4E-4,(StpLave6:5.5E-4,(StpGlobo:0.00752,(StpSpe36:5.5E-
4,(StpSpe37:5.5E-
4,((StpColom:0.0,StpLave4:0.0,StpVirgi:0.0,StpVinac:0.0,StpKatra:0.0,StpL
ave7:0.0,StpSakai:0.0,StpSubru:0.0,StpVirg2:0.0,StpAlbi3:0.0):5.5E-
4,(StpLave5:5.5E-4,StpSpe38:5.5E-4)0.326.4:5.5E-4)0.188.4:5.5E-
4)0.925.75:0.00382)0.215.4:5.5E-
4)0.830.43:0.00382)0.915.65:0.00765)0.241.7:0.00768,((StpKunmi:0.0,StpAur
e3:0.0,StpAureu:0.0,StpLong3:0.0,StpRecti:0.0,StpPhae3:0.0,StpSpe55:0.0,S
tpAmaku:0.0,StpDrozd:0.0):5.5E-
4,((StpSpe52:0.00369,((StpCyano:0.0,StpHirsu:0.0,StpPrasi:0.0,StpBambe:0.
0,StpBamb2:0.0):0.00743,((StpEmeie:0.0,StpPras3:0.0):5.5E-
4,StpPras2:0.00747)0.106.4:5.3E-
4)0.798.49:0.00371)0.824.39:0.00366,(StpSpe12:5.5E-
4,(StpSpe23:0.00744,StpSangl:0.00743)0.773.53:5.3E-4)0.881.68:5.5E-
4)0.857.68:0.00366)0.793.48:5.3E-
4)0.895.80:0.00708)0.887.74:0.0022,((StpWelli:0.01521,(((StpGris4:0.0,Stp
Albov:0.0,StpCaten:0.0,StpRamul:0.0,StpFasic:0.0,StpLydic:0.0,StpPlat3:0.
0,AmyLurid:0.0,StpAure4:0.0,StpRose8:0.0,StpHygr6:0.0,StpAscom:0.0):5.5E-
4,((StpRame2:5.5E-4,(((StpTher2:5.5E-4,(((StpCangk:5.4E-

4,StpYogya:0.00369)0.815.44:0.00369,(StpRhizo:0.00369,StpJaven:0.00369)0.
780.64:0.00369)0.696.10:5.4E-
4,(StpIndon:0.0,StpRosei:0.0,StpRose2:0.0,StpRose3:0.0,StpGlau2:0.0,StpAc
idi:0.0,StpCuspi:0.0,StpPropu:0.0,StpHygr3:0.0,StpPlat4:0.0,StpMoba2:0.0,
StpMobar:0.0,StpOliv5:0.0,StpPurp3:0.0,StpRimos:0.0,StpAlbo3:0.0,StpAlbo4
:0.0,StpEndus:0.0,StpCaste:0.0,StpIndo2:0.0,StpHygr5:0.0,StpHimas:0.0,Stp
Indo3:0.0,StpHygr4:0.0,StpOrino:0.0,StpParvi:0.0,StpCaer2:0.0,StpLuteo:0.
0,StpKashi:0.0,StpLute2:0.0,StpLute3:0.0,StpThiol:0.0,StpKishi:0.0,StpMas
hu:0.0):5.5E-4)0.000.471:5.5E-4)0.000.472:5.5E-
4,((StpMalay:0.0,StpSpe84:0.0,StpSporo:0.0):0.0037,StpLutei:0.0037)0.720.
14:5.5E-4)0.641.9:5.5E-4,(StpHygr7:5.5E-
4,((StpAcul2:0.0,StpAcule:0.0):5.5E-
4,StpSynne:0.00736)0.759.59:0.00742)0.841.51:0.00364)1.000.432:0.0027)1.0
00.433:9.8E-
4,((StpFradi:0.0,StpFrad2:0.0):0.00371,((((StpDalie:0.00739,((StpArmen:0.
00752,StpHalot:5.5E-4)0.856.51:0.00371,(((StpSpe82:5.3E-
4,StpAlbia:0.00371)0.919.69:0.00361,(((((StpCattl:0.0037,Ult25923:0.00369
)0.700.24:5.5E-
4,(StpPauci:0.0,StpBalie:0.0,StpFerra:0.0,StpMucof:0.0,StpSpe86:0.0,StpSp
e87:0.0):5.5E-4)0.000.473:5.4E-
4,((((StpPoone:0.0,StpRibos:0.0,StpAlbul:0.0):5.5E-4,((Ult25924:5.3E-
4,UltrS317:0.01128)0.839.55:0.00369,StpEndo2:0.00369)0.667.12:5.3E-
4)1.000.434:5.4E-4,StpSpe79:0.00362)0.433.11:0.00369,((StpRubid:5.5E-
4,(StpYeoc2:0.0,StpYeoch:0.0):0.0037)0.850.66:0.00369,((StpYangl:0.0,Ult2
5925:0.0):5.5E-4,(Ult25926:0.00369,StpGuand:0.00742)0.714.16:5.5E-
4)0.724.16:5.5E-4)0.912.82:0.00362)1.000.435:5.0E-4)0.000.474:5.5E-
4,((SmtBacte:5.4E-4,(((StpMegas:0.0,StpMacr2:0.0):5.5E-
4,StpGlau4:0.00369)0.966.69:0.01127,((StpCaca2:5.5E-
4,StpViol3:0.01128)0.829.50:0.00367,(StpGibso:5.5E-4,StpRango:5.5E-
4)1.000.436:5.4E-4)0.424.8:0.00369)0.266.5:5.4E-
4)0.833.53:0.0037,(StpSulph:0.01889,(StpGri13:0.0,AmcBacte:0.0,StpRadio:0
.0):0.00209)0.966.70:0.00181)0.871.63:5.4E-4)1.000.437:5.4E-
4,(StpAxine:0.0037,(StpNansh:0.00372,UltStr22:0.00372)0.784.48:0.00373)0.
887.75:0.00363)0.028.2:0.00369)1.000.438:5.1E-
4,(StpSpe83:0.0,StpFlav3:0.0,StpPlat5:0.0):5.5E-4)0.482.8:5.1E-
4)0.901.67:5.4E-
4)0.390.5:0.00369,(((StpDias3:0.0,StpPact2:0.0,CanStrep:0.0,StpSpe85:0.0)
:5.5E-4,(((StpAlthi:5.5E-
4,((StpPlemo:0.0,StpSpe11:0.0,StpSpect:0.0,StpPhae2:0.0,StpLabed:0.0,StpV
iola:0.0,StpSpe21:0.0,StpJanth:0.0,StpHawai:0.0,StpInaeq:0.0,StpAlth2:0.0
,StpGris5:0.0,StpAfgha:0.0,StpCoer3:0.0,StpCoer4:0.0,StpGris6:0.0,StpMate
n:0.0,StpSpe31:0.0,StpSpe32:0.0,StpNanni:0.0):5.5E-
4,((StpSpe17:0.00397,((((StpSpe66:0.0037,((StpOliva:0.0037,((StpSpe33:0.0
0744,(StpGrise:5.5E-4,(StpSpe26:5.4E-4,StpTend2:0.00792)0.387.8:5.4E-
4)0.289.2:5.3E-4)0.194.4:5.4E-
4,(StpCoeru:0.0,StpHeter:0.0,StpSpec3:0.0,StpSpec4:0.0,StpSpe10:0.0,StpLa
ven:0.0,StpOlivo:0.0,StpSpe16:0.0,StpAmbof:0.0,StpTenda:0.0,StpNobil:0.0,
StpScle2:0.0,StpAureo:0.0,StpAlbid:0.0,StpSpe18:0.0,StpSpe19:0.0,Acu22051
:0.0,StpPlica:0.0,StpSpe27:0.0,StpSpe29:0.0,StpSpe30:0.0,StpViol2:0.0,Stp
Coeli:0.0,Ult25919:0.0,StpAlbof:0.0,Ult25920:0.0,StpFrad3:0.0,StpGris7:0.
0,StpSpe34:0.0,UltAct26:0.0,StpFlav2:0.0,StpSpe67:0.0):5.5E-
4)0.443.4:5.5E-4)0.417.9:5.5E-
4,(StpParvu:0.01126,StpSpe65:0.00744)0.766.45:0.00371)0.000.475:5.4E-
4)0.405.11:5.5E-4,StpJieta:0.0074)0.382.8:5.4E-

4,(StpSpe25:0.03107,StpSulfo:0.00384)0.778.52:0.00355)0.886.83:5.5E-
4,(StpSpe64:0.0074,StpRoche:0.00368)0.784.49:0.00368)0.805.40:0.00405)0.3
67.12:5.4E-4,StpMisio:0.0037)0.403.8:5.5E-4)0.206.4:5.5E-4)0.546.8:5.4E-
4,(((StpScopi:0.0037,(StpGhana:0.0,StpEspin:0.0):0.00747)1.000.439:5.5E-
4,(BactIM14:5.4E-4,((StpLave2:5.5E-
4,(((StpVarie:0.0,StpCoer2:0.0,StpNogal:0.0,StpCiner:0.0,StpGlauc:0.0,Stp
Spe14:0.0,StpTosae:0.0,StpHebei:0.0,StpHebe2:0.0,StpSpe28:0.0,StpPactu:0.
0,StpDiast:0.0,StpAlano:0.0,StpSpe63:0.0,StpFrad4:0.0,StpCast2:0.0):5.5E-
4,StpSpira:0.00369)0.731.18:5.5E-4,StpSpec9:5.5E-4)0.787.47:5.5E-
4)0.846.63:5.5E-
4,StpGris2:0.00751)0.853.51:0.00356)0.185.7:0.00365)0.842.66:0.00357,((((
StpTher8:0.00369,(StpFimbr:0.0,StpSpe13:0.0,StpGris3:0.0,StpPurp2:0.0,Stp
Cinna:0.0,StpTher7:0.0,StpVello:0.0,StpAcrim:0.0,StpPalli:0.0,StpHelio:0.
0,StpHumid:0.0,StpTher9:0.0,StpMacro:0.0,StpCellu:0.0):5.5E-
4)0.863.64:5.5E-4,((StpSpe20:5.5E-
4,(StpViren:0.0037,StpAlbus:0.0037)0.742.33:5.5E-4)0.000.476:5.5E-
4,(StpCaele:0.00369,(StpSpeib:5.5E-
4,StpFuman:0.0037)0.802.45:0.00369)0.908.68:5.4E-4)0.000.477:5.5E-
4)0.762.66:5.5E-
4,(StpDjaka:0.01946,(StpThe10:0.01126,((StpSannu:0.0,StpSpec8:0.0):0.0036
9,(StpSodii:5.5E-4,StpGlau3:5.5E-4)0.794.46:5.5E-4)0.120.5:5.4E-
4)0.835.52:0.00368)0.998.74:5.4E-4)0.463.6:5.4E-
4,(((StpSpec2:0.0,StpTher5:0.0,StpTher6:0.0):0.0037,(StpTher3:0.0,StpTher
4:0.0):5.5E-4)0.858.61:0.00369,(StpSpe24:0.01878,StpFlavo:5.5E-
4)0.840.57:0.00367)0.526.6:5.5E-4)0.936.67:5.4E-
4)0.859.52:0.00363)0.925.76:0.00804,StpEndos:0.00369)0.478.7:5.5E-
4)0.183.7:5.3E-4,StpLunal:5.5E-4)0.846.64:5.3E-
4)0.791.45:0.00392,(StpBluen:0.01508,(StpSpe22:0.00369,StpSpe81:0.00746)0
.912.83:5.5E-4)0.824.40:0.01132)0.677.8:5.3E-
4,StpPlat2:0.00746)0.010.5:5.5E-4)0.082.6:5.5E-4)0.253.7:5.5E-
4)0.417.10:5.5E-
4,(StpScler:0.02238,StpRameu:0.00368)0.776.54:0.00428)0.416.14:5.4E-
4)0.937.58:0.008,McnMegal:5.3E-4)0.929.65:0.00801)0.683.16:5.4E-
4)0.888.68:5.3E-4,((Ult25922:5.4E-
4,(((StpSpe88:0.0,StpPuni2:0.0,StpPhae4:0.0,StpPhae5:0.0,StpPhae6:0.0,Stp
Phae7:0.0):5.5E-
4,((StpLacey:0.0,StpCaeru:0.0,StpLace2:0.0):0.0037,StpCine3:0.00369)0.708
.20:5.5E-4)0.897.60:5.5E-4,(StpSpe54:0.01118,(((StpHygr2:5.3E-
4,UltAct25:0.02261)0.868.54:0.00716,(((StpDecca:0.00717,StpStell:0.00718)
0.941.62:5.4E-4,StpIpomo:0.0037)0.677.9:5.5E-
4,(StpSpe57:0.0,StpScabi:0.0):5.5E-4)0.983.34:5.5E-
4)0.805.41:0.00365,(StpTauri:0.0,StpTaur2:0.0,StpRishi:0.0,StpSpe58:0.0,S
tpTurgi:0.0,StpTurg2:0.0,StpTurg3:0.0,StpSpe60:0.0,StpPlate:0.0,StpCacao:
0.0):5.4E-4)0.388.7:0.00367)0.815.45:5.4E-
4)0.852.61:0.00368)0.886.84:0.00736,(StpCine2:0.07614,(StpLave8:0.04407,S
tpAlbu2:0.10816)0.431.10:0.00792)0.949.61:0.02268)0.276.7:5.1E-
4)0.710.16:5.4E-4)1.000.440:0.0026)1.000.441:0.00113)0.394.8:5.5E-
4)0.298.5:5.5E-
4)0.876.58:0.01131)0.968.50:0.02377,(Ult26475:0.03175,((((((((Ult26042:0.
00492,(AiteSera:0.00808,AitSilic:0.01474)0.984.41:0.03953)0.987.61:0.0548
3,(StkAlbif:0.00867,(StkNassa:0.02244,(HglAlbus:0.03945,((GlyScopa:0.0233
,(GlyIllin:0.03602,(Ult26262:0.0,Ult26263:0.0,Ult26264:0.0):5.5E-
4)0.645.8:0.00763)0.926.78:0.01753,(GlyArizo:5.5E-
4,GlyTenui:0.01487)0.937.59:0.01815)0.363.11:0.01174)0.931.59:0.0246)0.89

6.53:0.01698)0.984.42:0.04774)0.933.72:0.02978,(AtdrAlba:0.00487,((AtdUmb
ri:5.5E-4,(AtdEchi2:0.00369,AtdEchin:5.5E-4)0.986.43:0.0151)0.051.2:5.4E-
4,(((AirLong2:0.0,AirLongi:0.0):0.01546,(AirCaver:0.03158,((((AirLiba2:0.
0,AirLiban:0.0):5.5E-4,AirAuran:0.00368)0.998.75:0.03546,AirAurea:5.4E-
4)0.357.8:0.00362,((AtdSpec3:5.4E-
4,(AtdAtram:0.00755,PvpPalli:0.01507)0.877.80:0.01129)0.565.8:5.4E-
4,(((AtdOligo:5.4E-4,(AtdNamib:5.5E-4,AtdKijan:5.5E-
4)0.836.39:0.00717)0.890.73:0.00756,(AtdRubro:0.00523,AtdMiaol:0.01448)0.
917.70:0.01461)0.745.43:0.00343,(AtdHibi2:0.00369,AtdHibis:0.00373)0.864.
67:0.00754)0.594.7:0.00781)0.970.49:0.01882)0.490.3:0.0107)0.144.6:5.5E-
4)0.958.72:0.01543,((AtdForm2:0.00369,AtdFormo:5.5E-
4)0.852.62:0.0037,((AtdSpec2:5.3E-4,((((AtdMadur:0.00369,(AtdCitre:5.5E-
4,((AtdPelle:0.0075,AtdPell2:0.0075)0.777.49:0.00365,(AtdCoeru:5.5E-
4,(((((AtdGlauc:5.5E-4,AtdCrem2:5.5E-4)0.217.5:5.5E-
4,AtdMadu2:0.00371)0.000.478:5.5E-4,AtdLatin:0.0074)0.189.2:5.5E-
4,((AtdCreme:0.00375,AtdSpec4:0.00371)0.821.36:0.00367,(((AtdVirid:0.0,At
dVinac:0.0,AtdRugat:0.0):0.0037,AtdVina2:5.5E-
4)0.778.53:0.00411,((AtdHalle:0.00369,Ult26470:5.5E-
4)0.800.39:0.00401,(TnpCurva:0.00369,TnpCurv2:5.5E-
4)0.898.81:0.01105)0.868.55:0.01161)0.875.68:0.0109)0.584.8:5.5E-
4)0.511.7:5.5E-
4,(AtdCrem3:0.0,AtdMacra:0.0,AtdMadu3:0.0,AtdChoko:0.0,UltAct85:0.0,AtdCh
ibe:0.0):5.5E-4)0.511.8:5.5E-4)0.000.479:5.5E-4)0.000.480:5.5E-
4)0.000.481:5.5E-4)0.489.5:5.5E-4,(AtdLivid:5.5E-
4,AtdCatel:0.00369)0.837.41:0.00368)0.904.66:5.4E-
4,(AtdSpeci:0.00741,((AtdNapie:0.0,AtdNitri:0.0):5.5E-
4,(AtdFibro:0.0,AtdYumae:0.0):0.00369)0.228.6:5.4E-
4)0.846.65:0.00367)0.379.2:0.0038,Ult26471:0.01506)0.451.12:5.4E-
4)0.707.11:0.00379,((SirRubra:0.01107,AtdSpec5:0.0189)0.903.63:5.4E-
4,(SirAlbid:5.5E-4,(AtdRuden:5.4E-
4,AtdFulve:0.00372)0.785.54:0.00369)0.382.9:5.3E-
4)0.888.69:0.00744)0.827.48:5.5E-
4)0.872.74:0.0079)0.786.56:0.00357)0.922.79:0.0186)0.544.6:0.00853)0.905.
58:0.01374,(UltAct73:0.01868,(Ult26419:0.01094,Ult26420:0.01712)0.747.39:
0.01365)0.840.58:0.01133)0.458.13:0.01014,(((AiaFulvu:5.5E-
4,(AiaSpadi:5.5E-4,AiaLurid:0.01125)0.927.66:0.00752)0.892.57:5.4E-
4,AiaIriom:0.00737)0.925.77:0.01549,((Ult26418:0.02296,UltrS326:0.00371)0
.310.3:0.01516,(Ult26417:5.5E-4,UltrS327:0.01502)0.999.93:5.5E-
4)0.972.49:0.01932)0.908.69:0.02205)0.460.5:0.00963,Ult26469:0.01885)0.80
1.49:0.01037,((TnpChrom:0.01189,TnpChro2:0.00332)0.950.52:0.02338,((NnmAf
ri2:0.00709,(NnmAfric:0.00742,(NnmDietz:5.5E-4,NnmRose3:5.5E-
4)0.632.7:5.5E-4)0.924.64:5.3E-
4)0.324.4:0.00767,((SsnViri2:0.03085,((PbpRosea:5.5E-4,(PmnVenez:5.5E-
4,PbpLongi:5.5E-
4)0.923.89:0.00354)0.486.8:0.01155,((PmnParo2:0.0,PmnParon:0.0):5.4E-
4,(PmnsAlba:0.00755,(StgPurpu:0.03509,((StgYunna:0.04768,(StgSubro:0.0074
,(StgAlbum:5.5E-4,(StgRoseu:0.0,StgSpeci:0.0,StgVulga:0.0):5.5E-
4)0.954.60:5.5E-4)0.953.43:5.5E-
4)0.486.9:0.00369,((StgViola:0.0,StgViol2:0.0):0.01917,(StgPseud:0.00376,
(StgFrag2:0.00369,StgFragi:5.5E-
4)0.873.71:0.00742)0.360.10:0.00371)0.873.72:5.3E-
4)0.770.70:0.00325)0.971.45:0.01954)0.800.40:0.00346)0.944.52:0.01923)0.7
53.47:0.00504)0.697.5:0.01886,(((NnmSpec2:0.00369,((NnmSpira:0.0075,NnmFa
sti:5.5E-4)0.855.61:5.5E-4,((((NnmSpec3:5.5E-

4,(NnmLongi:0.00744,NnmFast2:0.0037)0.846.66:5.5E-4)0.847.72:5.4E-
4,(NnmFerru:0.00374,NnmPolyc:0.01493)0.856.52:5.5E-
4)0.920.82:0.00369,CthAlbof:5.5E-4)0.000.482:5.5E-4,(((NnmSalmo:5.5E-
4,NnmAngio:0.00373)0.751.25:5.5E-4,(NnmRose2:5.5E-
4,NnmRoseo:0.0037)0.924.65:0.00743)0.740.43:5.5E-
4,(NnmRubra:0.0,NnmSpeci:0.0,NnmTerri:0.0,NnmRubr2:0.0,NnmBangl:0.0,NnmCo
xe2:0.0,NnmCoxen:0.0,NnmSpec4:0.0,NnmAsiat:0.0,NnmSpec5:0.0,NnmKuest:0.0)
:5.5E-4)0.000.483:5.5E-4)0.753.48:5.4E-4)1.000.442:5.4E-
4)0.761.44:0.00404,(TplFlexu:0.02715,(NnmAegyp:0.00429,NnmPusil:0.00308)0
.974.32:0.02762)0.738.22:0.00349)0.805.42:0.01128,(((SsnFlavi:0.0,SsnViri
d:0.0,SsnMelle:0.0):5.4E-
4,(SsnAlbum:0.0,SsnSpeci:0.0,SsnRubeu:0.0):0.00369)0.950.53:5.5E-
4,((((MprNiveo:0.0,MprMalay:0.0):5.5E-
4,MprGlauc:0.00369)0.926.79:0.01543,(TbsBispo:0.03685,Ult26468:0.05378)0.
833.54:0.00921)0.842.67:0.00742,(((MbpRose7:5.4E-
4,((((MbpRose3:0.01091,(MbpRose2:5.5E-
4,MbpMesop:0.0037)0.933.73:0.00199)0.933.74:0.00201,AimycPtL:5.5E-
4)0.117.4:5.5E-4,MbpSpeci:0.00368)0.880.69:0.00361,((MbpCoral:5.4E-
4,(PaoThail:0.0,PaoSilva:0.0,PaotMira:0.0):0.0037)1.000.443:5.4E-
4,(MbpRose6:5.5E-
4,(MbpSpec2:0.0,MbpRose4:0.0,MbpRose5:0.0,MbpRosea:0.0):5.5E-
4)0.839.56:0.00362)0.000.484:0.0037)1.000.444:5.1E-4)0.932.64:5.4E-
4,(AocCorr2:0.0037,((AocMacro:0.0,AocSpeci:0.0):0.00747,AocCorru:5.5E-
4)0.778.54:0.0037)0.905.59:0.00739)0.725.20:0.00744,HrdSpeci:5.3E-
4)0.734.32:0.00311)0.995.62:0.02808)0.908.70:0.01097)0.281.2:5.5E-
4)0.979.45:0.02287)0.930.60:0.01775)0.876.59:0.01204)0.777.50:0.00614,(Ul
t26473:0.02353,((HtnsAlba:0.01513,(NcdArabi:0.00743,((SmrSpec2:0.0,SmrSpe
ci:0.0):0.01125,(((NcdRhodo:0.0,NcdRosea:0.0):0.01512,(NcdSpec5:0.0,NcdCo
mpo:0.0):5.3E-4)0.919.70:0.00229,((NcdSpec6:0.0,NcdTreha:0.0):5.5E-
4,((NcdChrom:0.0,NcdHalop:0.0):0.0037,(((NcdKunsa:0.00341,NcdXinji:0.0154
)0.927.67:0.01157,NcdSalin:5.5E-
4)0.873.73:0.00369,((((NcdSpec3:0.0,NcdpAlb2:0.0,NcdpAlba:0.0,StpSpe89:0.
0,NcdDasso:0.0,NcdDass2:0.0,NcdSpec4:0.0):5.5E-
4,(NcdExhal:0.01126,(((NcdSpec2:0.00369,Ult26472:0.02403)0.548.9:5.5E-
4,NcdQuing:5.5E-4)0.839.57:0.00389,NcdBenis:0.01553)0.807.34:5.5E-
4)0.134.8:5.5E-4)0.450.9:5.5E-
4,NcdSpeci:0.0074)0.846.67:0.00402,NcdHalot:0.0037)0.000.485:5.5E-
4)0.833.55:0.00402)0.019.4:5.3E-
4)0.919.71:0.00275)0.919.72:0.0028)0.784.50:0.00402)0.221.6:5.4E-
4)0.913.67:0.01353,(TmdAlba0:0.00367,((TmdFusca:0.0,TmdCellu:0.0):5.3E-
4,MntTherm:0.00745)0.771.60:0.00379)0.645.9:0.00288)0.802.46:0.00966)0.96
0.59:0.03063)0.844.70:0.01201)0.916.76:0.0174)0.728.22:0.00375,(((JiaGans
u:5.3E-4,(JiaSpeci:0.03842,JiaSpec2:5.5E-
4)0.857.69:0.00742)0.999.94:0.02687,(((AppMorti:0.00926,((AppHalop:0.0036
6,AppXinji:5.4E-
4)0.856.53:0.01108,AppSalin:0.01165)0.416.15:0.00598)1.000.445:0.1195,(((
((Ult26045:0.04523,(CbrJeik4:0.03508,(Ult26044:0.02592,Ult26053:0.05079)0
.954.61:0.03491)0.867.58:0.01793)0.542.4:0.01416,(CbrXero3:5.5E-
4,(((CbrFrene:0.0,CbrXeros:0.0,CbrXero2:0.0,CbrXero4:0.0):5.5E-
4,((Ult26007:0.02642,(CbrAmyco:5.5E-
4,(Ult26008:0.0,CbrAmyc2:0.0,Ult26009:0.0):5.5E-4)0.779.55:5.5E-
4)0.835.53:0.00365,(CbrHanse:0.00366,((((Ult26000:0.00366,((((((Ult26021:
0.00366,(Ult26050:0.00366,(((((CbrSimul:0.0,CbrStria:0.0):0.00282,CbrTerp
e:5.5E-4)1.000.446:5.8E-

4,((((((((CbrConfu:0.00773,(((((CbrMacgi:0.0,CbrFasti:0.0,CbrSegme:0.0,Ul
t25989:0.0):5.4E-
4,(Ult25988:0.02251,Ult26015:0.03055)0.735.27:0.00431)0.783.60:0.00353,Ul
t25987:0.00382)0.426.14:0.00368,((Ult25978:0.02646,Ult25985:0.00371)0.764
.52:0.0036,(Ult25981:0.00366,((Ult25979:0.0,CbrTuber:0.0,Ult25982:0.0,Ult
25983:0.0,Ult25984:0.0,Ult25991:0.0,Ult25990:0.0):5.5E-
4,(Ult25986:0.01106,(Ult25980:0.01113,CbrPseu2:0.00372)0.202.8:5.5E-
4)0.577.4:5.5E-4)0.000.486:5.3E-4)0.364.6:5.5E-4)0.833.56:5.5E-
4)0.449.17:5.4E-4,(CbrMassi:5.5E-
4,Ult25992:0.02297)0.990.50:0.03098)0.276.8:0.00377)0.752.46:0.00348,(Cbr
Durum:0.02296,(((Ult26018:0.07226,CbrCaspi:0.01856)0.955.46:5.4E-
4,CbrMasti:5.5E-4)0.605.10:5.4E-
4,CbrAtypi:0.01108)0.436.9:0.0037)0.806.30:0.0037)0.773.54:0.00373,((UltC
ory5:0.01678,(CbrPropi:0.0,CbrPseu3:0.0):0.03097)0.761.45:0.01144,CbrCico
n:0.00377)0.758.58:0.00363)0.881.69:0.011,CbrCapit:0.03072)0.979.46:5.4E-
4,(CbrCysti:0.00366,(UltCory2:5.5E-
4,UltCoryn:0.00366)0.907.66:0.0074)0.816.41:0.00377)0.963.55:0.0157,((Cbr
Spec3:0.00713,((CbrPseud:5.5E-4,(CbrSpeci:5.4E-
4,((((Ult25951:0.00596,(Ult25966:0.00366,((Ult25970:0.0,CbrGeni4:0.0,CbrG
eni5:0.0):5.5E-4,(CbrGeni2:5.5E-4,(CbrTusca:0.00367,((((Ult25968:5.4E-
4,(((Ult25961:0.0,Ult25956:0.0,Ult25973:0.0):5.5E-4,(((Ult25977:5.4E-
4,Ult25976:0.00358)0.424.9:0.00366,(Ult25959:0.02255,(Ult25975:5.5E-
4,(Ult25960:0.00369,Ult25958:0.00367)0.598.5:5.5E-4)0.733.24:5.4E-
4)0.862.60:0.00359)1.000.447:5.5E-4,Ult25971:0.00735)0.330.5:5.5E-
4)0.268.4:5.5E-
4,Ult25972:0.00366)0.855.62:0.00361)0.036.4:0.00367,(Ult25954:5.5E-
4,Ult25974:0.01108)1.000.448:5.2E-
4)0.871.64:0.00359,Ult25955:0.00366)0.263.4:5.4E-
4,((Ult25962:0.0,CbrAppen:0.0,Ult25967:0.0,Ult25969:0.0):5.5E-
4,(((Ult25963:0.0,Ult25964:0.0):5.5E-
4,Ult25965:0.00366)0.852.63:0.00366,CbrGeni3:0.00741)0.696.11:5.5E-
4)0.000.487:5.5E-4)0.849.55:0.00366)0.310.4:5.5E-4)0.000.488:5.5E-
4)0.321.4:5.5E-
4)0.812.30:0.00597)0.908.71:0.01036,UltOr295:0.01129)0.644.6:5.4E-
4,(((Ult25948:0.01494,Ult25937:5.4E-4)0.840.59:0.00366,((((CbrUreic:5.5E-
4,Ult25940:0.00114)0.214.3:7.6E-
4,Ult25938:0.02629)0.981.46:0.00188,CbrGenit:0.00369)0.766.46:5.5E-
4,(Ult25929:0.0,Ult25928:0.0,CbrMucif:0.0,Ult25931:0.0,CbrAferm:0.0,Ult25
941:0.0):5.5E-4)0.000.489:5.5E-4)0.000.490:5.5E-4,(Ult25950:5.5E-
4,(Ult25939:0.00734,Ult25932:0.00734)0.968.51:5.5E-4)0.000.491:5.5E-
4)0.533.6:5.3E-4)0.466.10:5.4E-
4,((((Ult25930:0.0,Ult25927:0.0,CbrCoyle:0.0,Ult25933:0.0,Ult25949:0.0):5
.5E-
4,(Ult25957:0.00735,((UltCory4:0.00736,Ult25952:0.00366)0.724.17:5.5E-
4,(Ult25936:0.00366,Ult25953:0.00367)0.702.9:5.5E-4)0.701.16:5.3E-
4)0.848.59:0.00366)0.488.8:5.4E-4,Ult25935:0.00363)0.171.7:5.4E-
4,Ult25934:0.01478)0.805.43:0.00363)0.953.44:0.01106)0.854.62:0.00362)0.8
51.64:0.00363,(CbrMycet:5.5E-4,((Ult25945:0.00207,(CbrRiege:5.5E-
4,Ult25946:0.00366)0.904.67:0.00206)0.885.54:0.00685,(Ult25947:0.01114,(C
brAuris:0.00745,CbrTimon:5.5E-4)0.916.77:5.3E-
4)0.157.7:0.00372)0.971.46:5.5E-4)0.474.11:5.4E-4)0.916.78:5.4E-
4)0.990.51:0.02706,CbrPilos:0.00382)0.727.21:0.00331)0.737.42:0.00386,(Cb
rLubri:5.5E-4,CbrSpec2:5.5E-
4)0.881.70:0.00763)0.778.55:0.00438,(CbrSpec6:0.04039,(((Ult26020:0.02649

,(Ult26019:0.00366,CbrGlucu:5.5E-4)0.663.10:5.4E-
4)0.990.52:0.04802,(Ult26016:0.00386,Ult26017:0.01926)0.816.42:0.00956)0.
712.15:0.01525,(CbrKropp:0.01099,Ult26005:5.5E-
4)0.993.45:0.03616)0.775.55:0.00644)0.211.8:5.0E-
4)0.758.59:0.0108)1.000.449:5.5E-
4,((CbrAquil:0.00362,Ult25995:0.0719)0.767.50:0.00376,((CbrUlcer:5.5E-
4,CbrTestu:0.00373)1.000.450:5.5E-
4,UltRho46:0.05699)0.990.53:0.00344)0.351.8:5.5E-4)0.295.13:5.5E-
4,(CbrBovis:0.00365,(CbrBovi2:5.5E-
4,(((((((CbrFelin:0.01468,CbrSpec8:5.5E-
4)0.124.4:0.00597,((Ult26014:0.01099,(CbrMatru:5.5E-
4,Ult26013:0.00366)0.900.57:5.4E-4)0.977.43:0.01886,(Ult25944:5.4E-
4,((Ult25942:0.0,Ult25943:0.0,CbrGlauc:0.0):5.4E-
4,CbrSunds:0.0111)0.352.6:0.00738)0.883.71:0.00748)0.768.38:0.00568)0.901
.68:0.01059,((((Ult26011:0.0,CbrAmmon:0.0):5.5E-
4,CbrCasei:0.03452)1.000.451:5.5E-4,(BvcStat2:5.5E-
4,BvcStati:0.00366)0.850.67:0.00358)0.167.6:0.00367,((((CbrAurim:0.0,CbrA
uri2:0.0):5.5E-
4,(Ult26010:0.0074,Ult25998:0.0037)0.789.47:0.00364)0.796.39:0.00367,Ult2
5994:0.00367)0.797.39:0.0037,(CbrGeni7:0.00377,((CbrJeik2:5.5E-
4,(((Ult26001:0.00611,(Ult26004:0.01931,Ult26003:0.03457)0.749.55:0.00364
)0.249.8:5.5E-4,CbrGeni8:5.5E-4)0.240.5:5.5E-4,CbrJeik3:5.5E-
4)0.000.492:5.5E-4)0.288.6:5.2E-4,CbrJeike:5.5E-4)0.909.57:5.4E-
4)0.812.31:0.00368)0.819.39:0.0036)1.000.452:5.3E-4)0.146.6:5.4E-
4,CbrCampo:5.5E-4)1.000.453:5.4E-
4,((CbrGluta:0.0,CbrAceto:0.0,CbrGlut2:0.0,Ult26012:0.0,CbrEffic:0.0):5.4
E-
4,CbrCallu:0.00359)0.138.7:0.00367)0.806.31:0.00358,CbrPhoca:0.01974)0.83
8.60:5.4E-4,((Ult26046:0.00752,(CbrSuico:0.01136,(CbrAuri3:5.5E-
4,CbrResis:0.00368)0.973.36:0.01915)0.852.64:0.00722)0.250.5:0.00366,CbrS
pec4:0.0188)0.881.71:5.4E-4)0.440.6:5.5E-4)0.513.5:5.4E-
4)0.856.54:0.00367)0.077.4:5.5E-4)0.000.493:5.5E-4)0.000.494:5.5E-
4,(CbrFlav2:0.0,CbrFlave:0.0):0.00367)0.480.4:5.5E-
4,((CbrSpec7:0.01495,CbrHalot:5.4E-
4)0.223.3:0.00366,(Ult25997:0.00366,(Ult25996:0.00366,CbrSphe2:5.5E-
4)0.160.2:5.5E-4)0.801.50:0.00365)0.685.13:5.4E-4)0.000.495:5.4E-
4,CbrVaria:5.5E-4)0.518.8:5.1E-4,Ult25999:0.00366)0.158.5:5.5E-
4,(CbrSputi:0.00366,(CbrArgen:5.5E-
4,((CbrPseu4:0.0,CbrUlce2:0.0,CbrVitae:0.0):5.3E-
4,((CbrDiph2:0.0,CbrDipht:0.0,CbrDiph3:0.0):5.5E-
4,CbrKutsc:0.00366)0.833.57:0.00366)0.858.62:0.00366)0.219.5:5.5E-
4)0.832.46:0.00366)0.061.5:5.4E-
4)0.861.64:0.00378,(Ult25993:0.01112,(Ult26006:0.01488,(((CbrRena2:0.0,Cb
rRenal:0.0):0.00368,(Ult26002:0.02287,((CbrGeni6:0.00374,CbrUreal:5.5E-
4)0.842.68:0.00367,(CbrUrea2:5.5E-4,Ult26049:0.00376)0.803.36:5.5E-
4)0.905.60:5.5E-4)0.925.78:0.01101)0.775.56:0.00368,Otu00673:5.5E-
4)0.825.40:0.00366)0.430.7:5.5E-4)0.847.73:0.00366)0.341.10:5.5E-
4,CbrSphen:5.4E-4)0.637.6:5.5E-
4,UltCory3:0.02283)0.899.69:0.00757)0.514.5:5.5E-4)0.435.8:5.5E-
4)0.214.4:5.5E-4,CbrSpec5:5.5E-4)0.005.2:5.4E-
4)0.761.46:0.00646)0.907.67:0.01642,((((((RdcRhod2:0.0,RdcRhodn:0.0,RdcRh
od3:0.0):5.4E-4,(((Ult26036:0.063,((DieMaris:5.5E-
4,DieKunja:0.01558)0.813.43:0.00738,(((Ult26033:0.0,Ult26034:0.0):5.5E-
4,((Ult26031:0.01872,Ult26030:0.05947)0.854.63:5.4E-

4,Ult26032:0.00369)0.919.73:5.5E-4)0.775.57:5.4E-4,Ult26029:5.5E-
4)1.000.454:0.04418)0.870.48:0.01251)0.830.44:0.0075,((DiePsych:0.0,DieSp
eci:0.0,DieNatro:0.0):0.02204,(((GorParaf:0.00369,(((Ult26072:0.01503,((G
orAmara:0.0,GorRubri:0.0,GorAmica:0.0,GorSpec5:0.0):5.5E-
4,GorSpeci:0.00369)0.015.5:5.5E-4)1.000.455:5.4E-
4,((AmtctYyy:0.0,GorTerra:0.0,UltGordo:0.0,GorTerr2:0.0,GorSpec3:0.0,GorS
pec4:0.0):5.5E-
4,GorLacun:0.0037)0.857.70:0.00362)0.177.9:0.00369,((GorMalaq:0.0,GorSpec
2:0.0):5.5E-4,GorSihwe:0.00369)0.876.60:0.00361)1.000.456:5.4E-
4)1.000.457:5.5E-4,(GorHirsu:5.3E-
4,Ult26073:0.00742)0.919.74:0.00348)0.799.53:0.00751,((GorShand:0.00359,(
(GorSines:5.5E-
4,(((GorSput2:0.0,GorSput3:0.0,GorOtiti:0.0,GorOtit2:0.0):5.5E-
4,GorSputi:5.5E-
4)0.908.72:0.00375,(GorSpec6:0.0,Ult26074:0.0,GordSoli:0.0,GorPolyi:0.0):
5.5E-4)0.000.496:5.5E-
4)0.893.69:0.00365,((GorEffus:0.0,GorBronc:0.0,GorRhizo:0.0):5.5E-
4,GorRhiz2:5.5E-4)1.000.458:5.4E-4)0.178.5:0.00375)1.000.459:5.4E-
4,(GorAraii:5.3E-
4,GorDeflu:0.01498)0.663.11:0.00369)0.803.37:0.00394)0.855.63:0.01005)0.8
78.66:0.01)0.768.39:0.00813,(RdcSpec4:0.01184,Ult26080:0.02129)0.902.73:0
.01644)0.928.56:5.4E-4)0.886.85:5.5E-
4,((((RdcCopr2:0.0,RdcCopro:0.0):5.5E-4,RdcSpec3:5.5E-
4)0.871.65:0.0075,RdcSpeci:0.01138)0.791.46:0.0052,((RdcMaans:0.01124,Rdc
Tukis:5.5E-
4)0.892.58:0.00804,(((UltRho47:0.0,AntSeaw3:0.0,RdcSpec5:0.0,RdcCerci:0.0
,ArcSeaw3:0.0,PolarSe3:0.0,RdcSpec6:0.0,RdcFasci:0.0,UltDietz:0.0):0.0130
9,Ult26083:0.01427)0.991.50:0.03603,(((Ult26089:0.0037,Ult26023:0.01122)0
.803.38:0.00369,RdcJosti:5.5E-4)1.000.460:5.4E-
4,(RdcMari2:0.0,RdcMarin:0.0):0.00346)0.913.68:0.01172)0.760.45:0.00311)0
.884.71:0.01048)0.878.67:0.01074)0.906.82:0.0079,(((RdcSpe12:0.0037,(((No
cSpe17:0.08251,(RdcCoryn:0.0,UdnBac74:0.0,RdcCory2:0.0,RdcSpe13:0.0):5.3E
-4)0.974.33:0.019,((Ult26022:5.5E-
4,Ult26024:0.01117)0.946.58:0.01125,((RdcSpec7:5.5E-
4,((Ult26025:0.0,Ult26026:0.0,Ult26027:0.0,Ult26028:0.0,Ult26051:0.0,Ult2
6084:0.0,Ult26085:0.0,Ult26086:0.0,Ult26087:0.0,RdcEryt2:0.0,RaiTrou3:0.0
,Ult26088:0.0,NcrSimpl:0.0,RdcSpec9:0.0,RdcEryt3:0.0,RdcEryt4:0.0):5.5E-
4,RdcSpec8:5.5E-4)0.000.497:5.5E-4)0.910.67:5.5E-
4,(Ult26048:0.00369,RdcEryth:0.01115)0.499.6:5.5E-
4)0.783.61:0.0037)0.872.75:0.00757)0.760.46:0.00362,RdcTriat:5.5E-
4)0.785.55:0.0037)0.819.40:0.0037,((RdcZopfi:0.0,RdcZopf2:0.0,RdcPheno:0.
0,RdcRuber:0.0,RdcRube2:0.0):5.5E-4,((RdcGord2:5.5E-
4,((Ult26052:0.01917,(RdcSpec2:0.0,GorRubr2:0.0,Ult26078:0.0):5.5E-
4)1.000.461:5.2E-4,Ult26038:0.06918)0.842.69:0.00371)0.152.6:5.5E-
4,RdcGordo:0.00369)0.847.74:0.00376)0.189.3:5.5E-
4)0.777.51:0.00371,(((SkrPinif:0.00763,(((((NocAmami:0.0,NocBeiji:0.0,Noc
Araoe:0.0,NocSpe11:0.0,NocBeij2:0.0,NocBeij3:0.0,NocBeij5:0.0,NocSpe14:0.
0):5.5E-4,((NocGamke:5.5E-4,((NocTake2:5.5E-
4,NocTaked:0.00369)0.960.60:5.4E-
4,(NocXisha:0.00742,(NocPolyr:0.0112,(NocMexic:0.0,NocSpec2:0.0):0.01126)
0.727.22:5.5E-
4)0.094.2:0.00369)0.962.65:0.01124)0.908.73:0.00742,NocSpe12:0.00373)0.79
0.59:5.5E-4)0.921.77:5.4E-
4,NocBeij4:0.00734)0.768.40:0.0038,((NocPauci:0.00378,(NocPigri:5.4E-

4,((NocPuris:0.0,NocSpe15:0.0):0.0074,((NocCarne:0.0,NocFlavo:0.0,NocSpe1
0:0.0,Ult26081:0.0,NocSiena:0.0):5.4E-4,(NocAster:5.5E-
4,(NocCyria:0.00372,(NocCyri2:0.0,NocCyri3:0.0,NocCyri4:0.0,NocAste2:0.0,
NocCyri5:0.0,NocCyri6:0.0):5.5E-4)0.183.8:5.5E-
4)0.906.83:0.00745)0.228.7:0.00748)1.000.462:5.0E-
4)0.987.62:0.0233)0.932.65:0.01514,((((NocOtiti:0.0,StpLaete:0.0):5.4E-
4,((NocConca:0.0,NocSpeci:0.0):0.00746,((NocSeri2:0.0,NocSerio:0.0):0.015
26,NocCrass:0.02321)0.877.81:0.00749)0.962.66:0.01519)0.799.54:0.00538,(N
ocOtit3:0.0,NocTrans:0.0,NocTran2:0.0):0.01468)0.896.54:0.01069,((((NocJi
ang:0.0,NocaNova:0.0,NocaNov2:0.0,NocaNov3:0.0):5.4E-
4,(NocAcidi:0.01138,NocAnaem:0.01522)0.757.41:0.00367)0.874.77:0.00761,((
(NocKrucz:0.0,NocVeter:0.0):5.4E-
4,(NocUnifo:0.01571,NocOtit2:0.01116)0.936.68:0.0154)0.763.62:0.00747,(No
cAfric:5.4E-4,(((NocVacc2:0.0,NocVacc3:0.0):5.5E-
4,NocVacci:0.0074)0.943.61:0.01124,NocVermi:5.3E-
4)0.847.75:0.00369)0.752.47:0.00339)0.903.64:0.00791)0.845.66:0.00742,((N
ocMiyun:0.0037,(NocInoha:5.5E-4,(NocNiiga:0.00376,(NocPseud:5.4E-
4,(NocPseu2:0.0,NocPseu3:0.0,NocPseu4:0.0):0.0037)0.888.70:0.00747)0.881.
72:0.00755)0.846.68:0.00374)0.563.6:5.4E-4,NocYaman:5.3E-4)0.970.50:5.5E-
4)0.963.56:0.01555)0.760.47:0.00382)0.045.5:0.00796)0.964.58:0.02308,((((
McbAroma:0.00369,((McbGadi2:0.0,McbGadiu:0.0):5.5E-
4,((((((McbDiern:0.00743,McbSpec2:0.00369)0.931.60:5.5E-
4,(McbRhod2:0.0,McbFluor:0.0,McbSpec7:0.0):5.5E-4)0.000.498:5.5E-
4,(Ult26056:0.00369,McbGilvu:0.00369)0.310.5:5.5E-4)1.000.463:5.3E-
4,((((McbTherm:0.00261,McbFlave:0.0023)0.897.61:0.00229,McbcAgri:0.00389)
0.394.9:0.00371,(McbTokai:0.0,McbCosme:0.0,McbSpec3:0.0,McbSpec5:0.0,McbS
pec6:0.0,McbSpe13:0.0,McbSpe14:0.0):5.4E-
4)0.881.73:0.0023,(((Ult26071:0.00368,McbAlvei:0.00371)0.892.59:5.5E-
4,(McbSmeg2:0.0,McbSmegm:0.0,McbSpe17:0.0,McbFarci:0.0):5.5E-
4)0.765.40:5.5E-
4,McbAbsce:0.00369)0.922.80:0.00261)0.922.81:0.00226)0.454.12:0.0037,((((
McbAurum:0.0,McbVanba:0.0,McbRhode:0.0):5.5E-4,McbKomos:5.5E-
4)1.000.464:5.4E-4,(McbVacca:0.00366,McbRutil:5.5E-
4)0.470.3:0.0037)0.922.82:0.00241,((McbSpeci:0.01122,(McbHodle:0.0,McbIso
ni:0.0):5.3E-4)1.000.465:5.5E-
4,(((((Ult26058:0.01137,Ult26068:0.00755)0.808.46:0.00355,(McbChita:5.4E-
4,McbHolsa:0.00362)0.189.4:0.0037)1.000.466:5.4E-
4,(McbAubag:0.0,Bact2bG6:0.0):5.4E-4)0.916.79:0.00369,UltAct29:5.4E-
4)0.830.45:5.5E-4,(Ult26069:0.01111,Ult26061:0.00716)0.923.90:5.5E-
4)0.891.55:0.00741)0.922.83:0.00239)0.922.84:0.00247)1.000.467:5.5E-
4,McbTusci:5.5E-4)0.000.499:5.5E-4)0.884.72:5.4E-
4)0.818.32:0.00369,((((((McbHiber:0.0,McbFalla:0.0):0.00366,(McbElep2:0.0
,McbBruma:0.0):5.4E-4)0.463.7:0.0037,(((McbSpe10:5.4E-
4,((McbSpe15:0.00371,McbKumam:0.0037)0.811.41:0.0037,((McbTerra:5.5E-
4,McbEngba:0.00369)0.960.61:0.00168,(UltMyc23:0.00373,UltMyc22:0.00757)0.
951.60:0.0156)0.890.74:0.0023)0.105.7:5.5E-
4)0.863.65:0.00383,((Ult26059:5.4E-4,((McbMalmo:5.5E-
4,((McbHaemo:0.0,McbAsiat:0.0,McbSpec9:0.0):5.5E-
4,Ult26065:0.00369)0.000.500:5.5E-4)0.227.7:5.5E-
4,(((((Ult26063:0.0,McbAvium:0.0,McbColom:0.0,McbParaf:0.0,McbScrof:0.0,M
cbTube2:0.0,McbTuber:0.0,McbSaska:0.0,McbSpec8:0.0):5.5E-4,McbKansa:5.5E-
4)0.851.65:5.5E-
4,McbLepra:0.00744)0.922.85:0.00245,(Ult26062:0.01121,Ult26064:0.00375)0.
922.86:0.00243)0.922.87:0.00246,McbConsp:0.0037)0.056.5:5.4E-

4)0.858.63:0.00369)0.796.40:0.00371,(((UltMyc25:0.00327,(((McbParas:0.001
99,McbSpec4:0.01109)0.930.61:0.00201,(McbParme:0.0,McbHeide:0.0,McbFlore:
0.0,McbLenti:0.0):5.4E-4)1.000.468:5.5E-
4,UltMycob:0.00361)0.416.16:0.01312)0.931.61:0.02172,((UltAct30:0.00374,(
(McbBrand:0.0,McbKyori:0.0,McbCela2:0.0,McbCelat:0.0):5.4E-
4,Ult26066:0.0037)0.912.84:0.00747)0.806.32:0.0041,(McbHassi:0.01882,((Mc
bTriv2:0.0,McbTrivi:0.0):5.5E-
4,McbTriv3:0.0037)0.787.48:0.00417)0.235.10:0.00368)0.897.62:5.4E-
4)0.863.66:0.00685,((McbShimo:5.5E-
4,(McbNovio:0.01124,(McbBotni:0.00373,(McbXenop:0.0,McbHecke:0.0):0.01131
)0.886.86:0.00747)0.787.49:5.4E-4)0.839.58:0.00369,Ult26067:5.4E-
4)0.799.55:0.0037)0.765.41:0.00374)0.781.50:0.00371)0.598.6:5.5E-
4,(Ult26070:0.0,McbEleph:0.0,McbSpe16:0.0,Ult26055:0.0,McbBrisb:0.0,Ult26
060:0.0,McbRufum:0.0,McbSpe12:0.0,McbPalle:0.0):5.5E-4)1.000.469:5.5E-
4)0.922.88:0.00256,((Ult26054:0.0,McbMucog:0.0,McbLlatz:0.0,McbPhlei:0.0,
McbInsub:0.0):5.5E-
4,Ult26057:0.0074)0.922.89:0.00235)0.922.90:0.00264,McbPyren:5.5E-
4)0.863.67:0.00385,McbSpe11:5.4E-4)0.336.7:5.5E-
4)0.753.49:0.0039,(UltB3560:0.07059,UltAct31:0.01426)0.829.51:0.00866)0.7
52.48:0.00406,(SmaNiiga:0.00751,Ult26090:0.00749)0.994.57:0.03148)0.577.5
:0.00818)0.743.44:0.00381)0.860.47:0.00792,(NocSpe13:0.00754,((((DieCinna
:0.0,DieCinn2:0.0):5.5E-
4,Ult26035:0.00368)0.880.70:0.01576,((UltWilli:5.4E-4,((Ult26077:5.5E-
4,(WilDelig:0.00369,WilMaris:0.0037)0.706.17:5.5E-4)0.864.68:5.4E-
4,WilSerin:0.01505)0.963.57:0.01125)0.819.41:0.00369,((WilMural:0.0,Ult26
076:0.0):0.00741,(Ult26075:0.0,WilMura2:0.0):5.5E-4)0.125.6:5.4E-
4)0.793.49:0.0069)0.572.6:0.01141,((((NocAbsc2:0.0,NocAbsce:0.0,NocAbsc3:
0.0,NocFarci:0.0,NocFarc2:0.0,NocSpe16:0.0,NocFarc3:0.0):5.4E-
4,((NocBras2:0.0,NocBrasi:0.0,NocAltam:0.0):5.5E-
4,NocSpec7:0.00746)0.919.75:0.00742)0.817.30:5.4E-
4,((NocTran3:0.0,NocTran4:0.0,NocSpec8:0.0,NocBlack:0.0,NocTran5:0.0,NocB
lac2:0.0):5.5E-4,(NocThail:5.5E-4,(((NocNeoca:0.0,NocaNov4:0.0):5.5E-
4,NocAste3:0.00744)0.614.6:5.4E-
4,(NocCaish:0.00722,((NocSpec3:0.00368,((NocIgnor:0.0,NocaSoli:0.0,NocSpe
c4:0.0,NocaSol2:0.0):5.5E-
4,(NocSalmo:0.00746,NocFlumi:0.00369)0.734.33:5.5E-4)0.501.8:5.5E-
4)0.778.56:0.00786,(((NocaAlba:5.5E-4,(NocJejue:5.5E-4,NocRoseo:5.5E-
4)0.920.83:0.00366)0.894.53:5.4E-4,(((NocSpec6:0.0,NocIowen:0.0):5.5E-
4,(NocJinan:0.00365,NocSpec9:5.4E-
4)0.703.18:0.00743)0.903.65:0.00737,NocSpelu:5.5E-
4)0.387.9:0.00369)0.912.85:0.00737,NocSpec5:5.5E-
4)0.869.61:0.01096)0.855.64:0.01527)0.894.54:0.01145)0.914.71:0.00735)0.9
19.76:0.00744)0.922.91:0.00738)0.955.47:0.00734,(RdccEqu5:5.5E-
4,(GorKropp:0.00743,(((RdccEqu2:0.0,RdccEqui:0.0,RdccEqu3:0.0):5.5E-
4,(Ult26082:0.00369,RdccEqu4:0.00369)0.721.9:5.5E-4)0.000.501:5.5E-
4,((RdcKunmi:0.00368,Ult26162:0.02677)0.758.60:5.5E-
4,Ult26047:0.00369)0.894.55:5.5E-4)0.698.15:5.5E-4)0.396.15:5.5E-
4)0.968.52:5.4E-
4)0.764.53:0.00761)0.837.42:0.01087)0.924.66:0.01112)0.891.56:0.0078,(Rdc
Spe10:0.00808,(RdcImtec:0.01496,(RdcSpe11:0.0,RdcWrati:0.0):5.5E-
4)0.968.53:0.0219)0.779.56:0.00816)0.755.42:0.0039)0.786.57:0.00391)0.166
.6:5.3E-4,Ult26079:0.00383)0.995.63:5.4E-
4,MilBrevi:0.01107)0.563.7:0.01372)0.720.15:0.00312,(SglRotun:0.04686,(Ts
uIncho:5.5E-

4,TsuTyros:0.00369)0.793.50:0.0086)0.836.40:0.01675)0.242.6:0.01627,((Ult
Act27:0.05684,(Otu00467:0.00769,Otu00488:5.5E-
4)0.974.34:0.04639)0.980.37:0.04544,(UltAct32:0.03554,UltAct33:0.00382)0.
972.50:0.03324)0.546.9:5.3E-
4)0.000.502:0.02112)0.955.48:0.05064,(((((((FrkSpec2:0.00371,FrkSpec3:0.0
1526)0.782.61:0.00371,Ult26484:0.01906)0.816.43:0.00369,FrkSpeci:5.4E-
4)0.964.59:0.01888,(StpSeria:0.0079,(Ult26461:0.02533,((Ult26460:0.00943,
Ult26463:0.02139)0.846.69:0.00743,(Ult26459:0.03781,(StpScab2:5.5E-
4,StpScabr:5.5E-
4)0.772.55:0.00558)0.793.51:0.00373)0.902.74:0.008)0.709.14:0.00129)0.894
.56:0.01144)0.857.71:5.4E-
4,((((((((CspCoxen:0.00321,(((((PpgFlavi:0.01134,ApnBrasi:0.0037)0.772.56
:0.00372,((((AmcBact4:5.5E-4,McnMirob:5.5E-
4)0.841.52:0.00369,McnRifam:0.00741)0.286.6:5.5E-
4,(McnFlori:0.0,StpPeuc2:0.0,McnLupin:0.0,McnSpec6:0.0):5.5E-
4)0.919.77:0.00369,(((Ult26485:0.00369,McnCoxen:0.00369)0.707.12:5.5E-
4,(McnPurpu:0.0,McnCarbo:0.0,Ult26476:0.0,McnSpeci:0.0):5.5E-
4)0.737.43:5.5E-4,McnCiner:5.5E-4)0.433.12:5.4E-4)0.682.10:5.5E-
4)0.923.91:0.00741,((McnSpec2:0.0,AmcBact5:0.0,McnSpec3:0.0,McnSpec7:0.0)
:5.5E-4,(McnChal2:0.00372,McnChalc:0.00369)0.542.5:5.5E-4)0.971.47:5.5E-
4)0.897.63:0.00745,(ApnSpec2:0.00371,ApnFerru:0.00371)0.779.57:0.00375)0.
820.30:0.00377,Ult26491:0.03156)1.000.470:5.5E-
4)0.808.47:0.01147,(((LngAlbid:0.03044,(Ult26482:0.00369,VrsOchra:0.00373
)0.748.40:0.00568)0.761.47:0.01028,(CspYuxie:5.4E-
4,(CtgKoree:0.02098,(HamTsuno:0.0294,(Ult26487:0.04425,(Ult26477:5.4E-
4,(UltCatel:5.5E-
4,Ult26478:0.00369)0.972.51:0.01124)0.751.26:0.00383)0.663.12:0.00748)0.1
41.2:0.00559)0.851.66:0.00984)0.883.72:0.00804)0.775.58:0.01083,(MmnBact2
:0.0,MmnBacte:0.0):5.4E-
4)0.883.73:0.01048)0.888.71:0.008,(((ApnSarve:5.5E-
4,((ApnTeic2:0.0,ApnTeich:0.0):0.01125,(ApnPhili:0.00369,ApnPhil2:5.5E-
4)0.812.32:5.5E-
4)0.832.47:0.00369)0.937.60:0.00744,(((ApnTufto:0.0037,(ApnDurh2:5.5E-
4,ApnDurha:5.5E-
4)0.801.51:0.00368)0.883.74:0.00743,((((ApnArizo:0.0,ApnLobat:0.0,ApnDerw
e:0.0,ApnGarba:0.0,ApnSpec8:0.0,ApnRecti:0.0,ApnSpec9:0.0):5.5E-
4,(ApnPenic:0.00744,ApnCapil:0.01498)0.880.71:5.4E-4)0.724.18:5.3E-
4,(ApnCouch:5.4E-
4,(ApnItali:0.00361,((ApnCyan2:0.0,ApnCyane:0.0):0.00361,ApnLigur:5.4E-
4)0.738.23:0.00745)1.000.471:5.3E-
4)0.903.66:0.00741)0.976.38:0.01511,((ApnMisso:0.02698,ApnDigi2:0.0074)0.
808.48:5.5E-4,ApnKinsh:5.5E-4)0.998.76:5.4E-
4)0.840.60:0.00369)0.389.6:5.4E-4,ApnPhil3:5.4E-4)0.644.7:5.5E-
4)0.876.61:0.00759,((ApnIant2:0.0,ApnIanth:0.0,ApnSpec5:0.0,ApnSpec6:0.0,
ApnPurpe:0.0,ApnSpec7:0.0):5.4E-
4,ApnUtahe:0.0037)0.878.68:0.00737)0.880.72:0.00745)0.646.10:5.4E-
4,(((((((Ult26493:0.01127,Ult26479:0.01107)0.430.8:5.4E-
4,(McnPatta:0.0,McnSpec5:0.0,McnEburn:0.0):5.5E-4)1.000.472:5.4E-
4,(((((BacEll27:0.01129,(ApnSpeci:0.00757,((DacRose2:0.0,DacRoseu:0.0,Dac
Fulvu:0.0,DacThail:0.0,DacSalmo:0.0,DacMatsu:0.0,DacAuran:0.0):5.5E-
4,DacAura2:0.00369)0.956.55:0.01136)0.741.36:0.0037)0.881.74:0.00749,((Pl
hSpeci:5.4E-4,((((ApnGlob2:0.01504,ApnGlobi:5.4E-
4)0.956.56:0.01122,VrpAuran:0.0037)0.603.13:5.5E-4,SlwSpeci:5.5E-
4)0.791.47:0.00351,((LdmFlava:0.01504,(PiiTerev:0.01129,PiiAnula:5.5E-

4)0.889.67:0.00754)0.890.75:0.01096,(McnSpe13:0.0074,McnSpe14:5.5E-
4)0.888.72:5.4E-
4)0.886.87:0.00734)0.784.51:0.00383)0.722.12:0.00364,Ult26097:0.01128)0.8
01.52:5.5E-4)0.815.46:0.00364,((SiiYaman:0.00369,(PlhRubra:5.3E-
4,Ult26481:0.01126)0.837.43:0.00369)0.652.9:5.5E-4,Ult26490:5.5E-
4)0.886.88:5.4E-
4)0.853.52:0.00365,((ApnNippo:0.0,ApnAuran:0.0,Ult26492:0.0):5.4E-
4,(Ult26486:0.01102,(McnSpe12:0.0037,(ApnDigit:0.0037,ApnHumid:5.5E-
4)0.926.80:0.00745)0.907.68:5.4E-4)0.868.56:0.00715)0.744.32:5.4E-
4)0.887.76:0.00356,(CouCaeru:5.5E-4,(ApnSpec3:5.5E-
4,((ApnSpec4:0.0,PdpFerru:0.0,CouCaer3:0.0,KraCinna:0.0):5.5E-
4,CouCaer2:0.00376)0.000.503:5.5E-4)0.714.17:5.4E-4)1.000.473:5.4E-
4)0.153.6:0.00363)0.888.73:0.00356,(McnSpe11:0.00369,((Ult26480:0.00741,U
ltAct86:5.4E-
4)0.888.74:0.01125,(((McnSpec4:0.0,McnEchin:0.0,McnChers:0.0,McnSpec8:0.0
):5.5E-
4,(((McnPeuce:0.00744,(ApnDecca:0.0,ApnDecc2:0.0):0.01503)0.984.43:5.5E-
4,McnSpec9:0.00369)0.915.66:5.5E-4,McnAurat:5.5E-4)0.000.504:5.5E-
4)0.933.75:5.4E-4,McnSpe10:0.00733)1.000.474:5.5E-
4)0.829.52:0.00362)0.479.10:5.5E-
4)0.922.92:0.00338,((VrpGifh2:0.0,VrpGifho:0.0):5.5E-4,((Ult26489:5.2E-
4,(CplIndic:5.5E-4,(((CplCasta:0.0,CplJapon:0.0):5.5E-
4,CplNepal:0.00369)0.014.4:5.5E-4,CplAtrov:0.00369)0.899.70:5.4E-
4)0.932.66:0.00718)0.901.69:5.5E-
4,(Ult26483:0.01173,((AsoFerr2:0.0,AsoFerru:0.0):0.01108,AsoIriom:5.4E-
4)0.839.59:0.00681)0.578.5:0.0075)0.803.39:0.00703)1.000.475:5.5E-
4)0.084.5:0.00362,(SliSpeci:0.0,SliTropi:0.0):0.00396)0.922.93:5.4E-
4,(MmnYyyyy:0.00718,(MmnBact3:0.00754,Ult26488:0.01533)0.931.62:5.4E-
4)0.764.54:0.0067)0.894.57:0.007)0.992.57:0.02232,Ult26432:0.01131)0.767.
51:0.00357,(Ult26431:0.00371,(UltNakam:0.01903,(((UltPro26:0.05261,NakMul
ti:0.00351)0.888.75:0.01161,(AntBac43:0.01123,(Ult26428:5.5E-
4,(SaxLacte:0.00369,Ult26429:0.01118)0.651.13:5.5E-4)1.000.476:5.3E-
4)0.896.55:0.00773)0.192.5:0.00358,(HmsFlavi:0.0,Ult26430:0.0):0.00384)0.
871.66:0.00746)0.748.41:0.00367)0.891.57:0.00747)0.866.63:0.00703,((((Ult
26426:0.01163,(((GdrBacte:0.00751,Ult26442:0.0075)0.789.48:0.00379,Ult264
41:0.00367)0.765.42:0.00372,(((Ult26443:0.01542,GdmObsc2:0.00372)0.777.52
:0.00371,BstSpec3:0.01513)0.825.41:5.3E-4,BstSpec2:5.5E-
4)0.890.76:0.00755)0.870.49:0.00757)0.479.11:0.00746,((Ult26427:0.00927,(
(Ult26435:5.4E-4,((MdsMulti:5.4E-
4,Ult26440:0.0389)0.778.57:0.00383,(((Ult26378:0.0,Ult26438:0.0,BstSpeci:
0.0):5.5E-4,(Ult26439:0.0037,Ult26437:0.00369)0.846.70:5.3E-
4)0.973.37:5.5E-4,(Ult26375:0.03486,(BstAggr2:0.0,BstAggre:0.0):5.5E-
4)0.975.44:0.01893)0.527.7:0.01131)0.861.65:0.00736)0.952.53:0.01141,(Mds
Speci:0.01508,Ult26434:0.00376)0.766.47:0.00363)0.776.55:0.00581)0.783.62
:0.01142,(Ult26446:0.01531,(UltAct76:0.0167,UltrS328:0.01901)0.908.74:0.0
2056)0.879.65:0.02025)0.848.60:0.00917)0.882.68:5.4E-
4,(UltAct75:0.0149,Ult26425:0.02766)0.873.74:5.5E-
4)0.762.67:0.00385,Ult26433:0.00736)0.556.5:0.00758)0.556.6:5.5E-
4,(((((UltAct72:0.01935,((Ult26422:0.0,Ult26423:0.0):0.00744,(Ult26424:0.
00369,UltAct74:0.00372)0.904.68:0.00747)0.882.69:5.5E-
4)0.904.69:0.00745,UltSpori:0.00742)0.543.7:5.5E-
4,UltAct34:0.01513)0.902.75:0.00824,((((UltAct82:0.05276,(Ult26467:0.0444
8,(Ult26458:0.066,((Ult26457:0.05872,(((Ult26449:0.03952,(((Ult26454:5.4E
-4,(Ult26455:5.4E-

4,BacEnr46:0.00369)0.843.49:0.00368)0.995.64:0.02707,(Ult26452:0.00332,(U
lt26451:0.01473,Ult26450:5.5E-
4)0.964.60:0.01903)0.919.78:0.01168)0.757.42:0.00384,(Ult26448:0.0077,(Ul
t26447:0.02739,Ult26456:0.01912)0.859.53:0.01282)0.766.48:0.00355)0.869.6
2:0.00746)0.741.37:0.00383,((UltAct79:0.00787,UltAct80:0.01933)0.886.89:0
.01112,(UltAct81:0.01519,(UltAct77:0.02701,UltAct78:5.4E-
4)0.553.7:0.01194)0.983.35:0.02892)0.759.60:0.0042)0.959.63:0.03021,(((((
Otu00276:0.04685,UltAct56:0.07495)0.772.57:0.01375,((ZneTYyyy:0.01194,(Ul
tAct50:0.00369,UltTr378:5.5E-
4)0.908.75:0.01474)0.964.61:0.01962,(Otu00170:0.023,((CanAncil:0.01322,Ul
tAct49:0.00315)0.993.46:0.03125,UltAct48:0.00793)0.345.6:0.00342)0.777.53
:0.00412)0.863.68:0.00875)0.204.4:0.00935,((UltAct57:0.0221,UltAct53:0.00
842)0.084.6:0.00955,(UltAct55:0.01873,(UltAct51:0.02022,(UltAct52:0.02897
,UltAct54:0.01649)0.812.33:0.01059)0.940.69:0.02719)0.092.6:0.01469)0.661
.11:0.01512)0.808.49:0.01497,(ZnvYyy04:0.02169,UltAct58:0.08032)0.171.8:0
.00612)0.935.59:0.04048,(Ult26284:0.05356,Otu00333:0.01598)0.933.76:0.023
96)0.885.55:0.02091)0.922.94:0.02226)0.744.33:0.00708,(Ult26466:0.01138,(
UltAct84:0.02762,(AcumGP60:0.00369,AmcBact3:5.5E-
4)0.934.65:0.02089)0.965.54:0.02893)0.893.70:0.0176)0.566.7:0.01393)0.427
.7:0.01468)0.875.69:0.01985)0.810.44:0.01235,(UltOr296:5.3E-
4,UltAct83:0.02677)0.918.69:0.01863)0.733.25:0.00521,AhmCellu:0.02226)0.4
72.5:0.00805,((Ult17119:0.00861,(Ult26416:0.00664,Ult26421:0.0045)0.110.5
:0.00454)0.849.56:0.01555,(((CtsSubtr:0.00723,CtsAcidi:5.4E-
4)0.985.51:0.06143,(AcuAac15:5.5E-4,(AcuAac38:0.01133,AtiRobin:5.5E-
4)0.764.55:0.00347)0.919.79:0.03519)0.467.9:0.02444,(Ult26445:0.03391,(((
(((Ult26545:0.01552,Ult26546:0.00751)0.863.69:0.00767,(((((UltAct93:0.003
82,(((PahgYy91:0.0,PahggY90:0.0):5.5E-
4,PahggY89:0.00369)0.888.76:0.00763,(UltAct91:0.01166,UltAct92:0.00374)0.
775.59:0.00364)0.966.71:0.01601)0.626.13:0.00367,((((Ult26515:0.01358,(Po
iBact2:0.01421,PoiBacte:0.06813)0.894.58:0.01877)0.864.69:0.01173,((PopAc
id2:0.01893,PopAustr:5.3E-
4)0.977.44:0.0195,(PopCyclo:0.00334,PopFreud:0.02724)0.763.63:0.00384)0.9
40.70:0.01855)0.877.82:0.01241,PmmLymph:0.01087)0.755.43:0.00514,(Ult2652
2:0.01138,BrwCercl:0.00377)0.923.92:0.0111)0.865.65:5.1E-
4)0.863.70:0.00778,Ult26523:0.03093)0.460.6:0.00727,((Ult26520:0.00487,((
Ult26516:0.0037,TesSpeci:5.5E-4)0.783.63:0.00511,((TesFlave:5.5E-
4,(TesBendi:0.01125,(Ult26519:0.03064,Ult26517:5.4E-
4)0.923.93:0.00743)0.549.9:5.4E-
4)0.909.58:0.00741,((TesLubri:0.0075,(TesSpec2:0.00746,Bfhggg85:0.00369)0
.778.58:0.0037)0.711.19:5.4E-4,Ult26518:5.5E-4)0.500.8:5.4E-
4)0.894.59:0.01376)0.755.44:0.01135)0.841.53:0.00985,PopPropi:0.01575)0.8
94.60:0.01525)0.950.54:5.4E-
4,((UltAct88:0.01569,UltProp2:0.06189)0.727.23:0.00327,(((Ult26524:5.4E-
4,PpfInnoc:0.00742)0.900.58:0.0074,(((PoiBact3:0.01171,(LueJapo2:0.01499,
(LueSangu:0.0,LuePerit:0.0):5.5E-
4)0.961.53:0.01549)0.760.48:0.00327,Ult26528:5.4E-4)0.893.71:5.5E-
4,GnlPheno:0.00739)0.901.70:0.0112)0.303.7:5.5E-
4,Ult26547:0.04445)0.886.90:0.00746)0.805.44:0.00372)0.795.48:5.5E-
4)0.939.60:0.01128,(((UltProp3:0.01119,(Ult26525:0.00548,(Ult26526:5.5E-
4,Ult26527:0.02687)0.935.60:0.01335)0.797.40:0.01878)0.896.56:0.02661,((P
opJense:0.0037,PopThoen:0.00742)0.859.54:7.9E-
4,(((Ult26504:0.02671,Ult26512:0.00785)0.959.64:0.02666,(((Ult26499:0.060
61,(BacNL440:0.0036,(((((Ult26503:0.0268,Ult26508:0.03875)0.767.52:0.0036
5,((Ult26498:5.5E-

4,((((BacNL428:0.01475,(BacNL429:0.00742,(Ult26494:0.02651,Ult26500:0.01504)0.087.6:5.5E-4)0.269.6:5.4E-4)0.828.44:0.00361,(((BacNL441:0.00371,BacNL430:0.0037)0.322.7:5.5E-4,(BacNL438:5.5E-4,(BacNL435:0.00222,Ult26502:0.03424)0.306.4:8.4E-4)1.000.477:7.2E-4)0.000.505:5.5E-4,BacNL439:0.00369)0.000.506:5.5E-4)0.300.8:5.5E-4,BacNL442:0.00369)0.244.3:5.5E-4,(Ult26495:0.0149,Ult26496:0.01489)0.882.70:5.3E-4)0.482.9:5.5E-4)0.000.507:5.5E-4,(Ult26501:0.0,Ult26497:0.0,Ult26506:0.0,BacNL433:0.0,Ult26509:0.0,Ult26510:0.0,BacNL434:0.0,BacNL436:0.0,BacNL437:0.0,BacNL443:0.0,Ult26514:0.0):5.5E-4)0.000.508:5.5E-4)0.442.12:5.5E-4,UltPropi:0.01119)0.146.7:5.5E-4,BacNL432:0.00735)0.309.4:5.5E-4,(Ult26507:0.0267,BacNL431:0.00734)0.152.7:5.5E-4)0.372.5:5.5E-4)0.881.75:5.4E-4)0.866.64:0.00703,Ult26511:5.4E-4)0.774.66:0.00363,(PopAcidi:0.00369,(Ult26513:0.02642,UltAct87:5.5E-4)0.986.44:0.01894)0.789.49:0.00371)0.825.42:0.00346)1.000.478:5.5E-4,PopAvidu:0.0037)0.752.49:0.00654)0.735.28:0.02162)0.612.11:0.02423,((((MpnGlyco:0.01519,Ult26544:0.00365)0.988.60:0.02317,Ult26542:5.3E-4)0.364.7:0.00358,(((Ult26548:0.00906,Ult26549:0.00988)0.924.67:0.01586,((Ult26540:0.01439,(((Ult26534:0.0071,(FrdSpec3:5.5E-4,(Ult26535:0.00737,((((Ult26529:5.5E-4,(FrdSpec2:0.00371,(FrdSpeci:5.5E-4,(Ult26531:0.0037,Ult26530:0.00369)0.726.17:5.5E-4)0.773.55:5.5E-4)0.854.64:0.0037)0.770.71:0.00372,Ult26533:0.01528)0.760.49:0.00368,Ult26532:5.5E-4)0.866.65:0.00368,FrdLacus:5.5E-4)0.576.9:5.4E-4)0.837.44:0.00366)0.883.75:5.1E-4)0.476.11:0.00793,(UltEndo9:0.01141,Ult26541:0.01941)0.925.79:0.01577)0.542.6:0.01067,(UltrS330:0.00395,(MiuGinse:0.00395,((MiuPanac:0.0071,(MiuAuran:5.4E-4,(MiuPhosp:0.00377,UltrS329:0.00738)0.228.8:0.00372)0.874.78:0.00773)0.035.7:0.00365,Ult26539:0.01475)0.872.76:5.4E-4)0.894.61:0.00751)0.912.86:5.5E-4)0.147.5:0.00799)0.833.58:0.01134,AmbKwang:0.01421)0.868.57:0.01096)0.888.77:0.00877,((Ult26543:0.0,UltAct90:0.0):5.4E-4,((PooSpeci:5.5E-4,UltAct89:0.02713)0.900.59:0.0075,(PooPalu2:0.00362,PooPalud:0.01504)0.748.42:0.00387)0.895.81:0.00745)0.911.87:5.5E-4)0.796.41:0.00755)0.982.40:0.0189,PplSuper:5.4E-4)0.696.12:0.01124)0.562.9:5.3E-4)0.846.71:0.01234,(Ult26521:0.0435,(Ult26536:0.02058,(Ult26538:5.3E-4,(PbnBacte:0.00724,Ult26537:0.0319)0.807.35:0.00786)0.954.62:0.02286)0.872.77:0.01444)0.517.3:0.01509)0.904.70:0.01512,(Ult26596:0.06708,(ApmSinga:0.00795,ApmSpeci:0.02664)0.871.67:0.01952)0.953.45:0.02885)0.725.21:0.0022,((UltEnd10:0.01778,(((KrlJejue:0.0,KrlSolan:0.0,KrlSwart:0.0):5.4E-4,(KrlFlavi:5.5E-4,(KrlCatac:0.00369,KrlYunna:0.01496)0.079.3:5.4E-4)0.923.94:0.00742)0.981.47:0.02527,(Ult26592:0.00786,((Ult26587:0.01754,(Ult26585:0.00523,Ult26586:0.01349)0.342.6:0.00367)0.977.45:0.02485,UltrS331:0.00775)0.941.63:0.01569)0.821.37:0.00905)0.904.71:0.01201)0.767.53:0.00473,((((Ult26594:0.03509,(((AemAlkal:0.00372,AemSpeci:0.01536)0.553.8:0.00755,UltAero6:0.01919)0.939.61:5.5E-4,AemPonti:5.3E-4)0.993.47:5.5E-4)0.852.65:0.00366,((((UltAero4:0.0,AemEryth:0.0,AemMarin:0.0):5.5E-4,(AemTamle:0.00746,(AemFasti:0.0,NcrSpe12:0.0):0.00743)0.820.31:5.5E-4)0.346.10:5.5E-4,Ult26593:0.00369)0.749.56:5.4E-4)0.883.76:0.00733,UltAero5:5.3E-4)0.812.34:0.01125,((((UltNocar:0.0037,(NcrDaphn:0.0,UltNoca2:0.0):0.0037)0.107.11:5.4E-

4,((NcrSpec8:0.00751,Ult26572:0.00365)0.953.46:0.01142,(NcrLentu:0.01106,
((((Ult26567:0.00741,(((((NcrAquit:5.5E-
4,((Ult26556:0.00744,Ult26575:0.01498)0.714.18:5.5E-
4,Ult26562:0.00369)0.967.43:5.4E-4)0.000.509:5.5E-
4,(((NcrFonti:0.0037,(NcrMarin:0.00369,(((Ult26566:0.03107,(Ult26554:0.00
773,((Ult26551:0.0,UltAct94:0.0,Ult26552:0.0,Ult26555:0.0):5.5E-
4,NcrFurvi:0.0037)0.739.32:0.0035)0.850.68:0.01152)0.680.13:0.00384,(NcrM
ari2:5.4E-4,(NcrDokdo:5.5E-
4,NcrAquat:0.00742)0.909.59:0.00745)0.778.59:0.00334)0.923.95:0.00372,(Ul
t26568:0.01104,(NcrPlant:0.00744,Ult26569:5.5E-
4)0.852.66:0.00788)0.906.84:0.01536)1.000.479:5.4E-4)0.119.5:5.4E-
4)0.914.72:0.00743,NcrSpec6:0.00746)0.441.10:5.4E-
4,(Ult26570:0.00746,((NcrSpec3:0.01504,NcrInsul:5.5E-
4)0.932.67:0.01103,(NcrSpec5:0.00756,Ult26560:0.019)0.900.60:5.4E-
4)0.851.67:0.00719)0.811.42:0.00374)0.355.9:5.5E-4)0.859.55:5.4E-
4,Ult26574:0.00742)0.787.50:0.0037,NcrBigeu:0.0113)0.776.56:0.0037,(((((U
lt26553:0.00369,(((BactSPE2:0.0,NcrHwasu:0.0):5.5E-4,(((Ult26550:5.5E-
4,(NcrAlbus:5.5E-
4,NcrSpeci:0.01121)0.821.38:0.00368)0.987.63:0.02251,Pshggg64:0.00857)0.0
01.5:0.00346,Ult26559:0.00843)0.898.82:0.01052)0.926.81:0.00743,(UltBac26
:0.079,(Ult26576:0.00369,NcrJense:5.5E-4)0.921.78:5.4E-
4)0.946.59:0.01494)0.707.13:5.5E-4)0.885.56:5.4E-
4,(Ult26557:0.00596,Ult26558:0.01462)0.806.33:0.0059)0.956.57:0.01487,((N
crKoree:0.00751,((NcrAlkal:0.00359,Ult26565:0.02682)0.881.76:0.00755,NcrS
pe11:5.3E-
4)0.792.57:0.0037)0.780.65:0.00376,NcrAgari:0.00748)0.975.45:5.5E-
4)0.900.61:0.01456,((NcrSpec4:5.4E-
4,(NcrPanac:0.00703,(Ult26573:0.00951,NcrHalot:0.01823)0.925.80:0.01476)0
.726.18:0.01518)0.904.72:0.01842,(NcrHanko:5.5E-
4,Ult26571:0.0074)0.876.62:5.4E-4)0.935.61:0.01489)0.390.6:5.4E-
4,NcrIslan:0.00745)0.894.62:0.00754)0.495.7:5.4E-
4)0.842.70:0.00369,((Ult26563:0.0,NcrKribb:0.0,NcrSpec7:0.0):5.5E-
4,((NcrTrito:0.01488,NcrAestu:0.00201)0.846.72:0.00189,((Ult26561:0.02284
,(NcrGinse:0.0037,NcrSpec2:5.5E-4)0.902.76:5.4E-
4)0.951.61:0.01106,PimSimpl:5.4E-
4)0.945.62:0.01128)0.790.60:0.00375)0.409.6:5.5E-
4)0.830.46:0.00369,NcrSpe10:5.5E-
4)0.946.60:0.00494,(NcrBasal:0.00742,(NcrSedim:0.0,NcrTerri:0.0):5.5E-
4)0.946.61:0.00495)0.913.69:0.00463)0.909.60:5.4E-
4)0.931.63:0.0075)0.929.66:0.00744,(((NcrDubiu:0.00744,Ult26589:0.00759)0
.919.80:0.01126,(MmrAequo:0.00745,(Ult26581:0.0037,MmrBigeu:5.5E-
4)0.800.41:0.00373)0.667.13:0.00755)0.861.66:5.4E-
4,(Ult26564:0.00369,Ult26577:5.5E-
4)0.953.47:0.01121)0.915.67:0.00743)0.000.510:5.3E-
4,(((((Ult26588:0.0,Ult26590:0.0):5.5E-
4,Ult26584:0.01125)0.446.6:0.00747,(Ult26580:0.00369,Ult26583:0.01501)0.9
23.96:5.5E-4)0.807.36:0.00365,NcrSpec9:0.00743)0.248.5:5.5E-
4,((Ult26591:0.00369,(Ult26582:5.5E-
4,Udntfd16:0.00371)0.846.73:0.0037)0.706.18:5.5E-
4,(Ult26578:0.0,Ult26579:0.0):5.5E-4)0.916.80:5.5E-4)0.250.6:5.4E-
4)0.782.62:0.00378)0.922.95:0.01149)0.875.70:0.00835)0.968.54:0.03107)0.3
81.12:0.016)0.896.57:0.02192)0.828.45:0.01819)0.874.79:0.0151)0.749.57:0.
00306,((((GdmObscu:0.00718,(AtuBrasi:5.5E-
4,Ult26436:0.00369)0.867.59:0.00797)0.815.47:0.00942,Ult15395:0.06086)0.4

60.7:0.00872,(Ult26464:5.5E-
4,Ult26465:0.00368)0.994.58:0.0479)0.914.73:0.01869,Ult26444:5.5E-
4)0.942.63:0.01614)0.788.52:0.004)0.850.69:0.00824)0.723.20:5.4E-
4,(((SpyPolym:0.00369,SpyPoly2:5.5E-
4)0.968.55:0.01944,(FodFeeng:0.01143,((CpsAuran:0.0,CpsSpeci:0.0):5.5E-
4,CpsArvum:0.00369)0.881.77:0.00748)0.896.58:0.01176)0.882.71:0.01133,(Ul
t26412:0.01533,((UltrS325:5.5E-
4,(Ult26414:0.01117,Ult26411:0.0037)0.888.78:5.5E-4)0.871.68:5.5E-
4,(Ult26413:0.00744,(UltAct71:0.0037,Ult26415:5.5E-
4)0.928.57:0.00753)0.552.8:5.4E-
4)0.777.54:0.0036)0.956.58:0.01527)0.639.10:5.4E-
4)0.918.70:0.0084,((((AmyTaiwa:0.0,AmyIriom:0.0):0.03331,((((((AoacAlba:5
.4E-4,((GooCoeru:5.5E-
4,((((StpTeneb:0.01862,(CslCryop:0.0,CsllEqui:0.0):5.5E-
4)0.305.5:0.00748,(AlzAlbat:0.00686,((AmyAustr:0.00524,((SctViola:5.5E-
4,((SctSpeci:0.0,SctSpec2:0.0,SctLongi:0.0):0.00743,((SctEspan:0.0,SctAlg
er:0.0,SctXinj2:0.0,SctXinji:0.0):5.5E-
4,(SctTexas:0.00369,SctAustr:0.00369)0.543.8:5.5E-4)0.806.34:5.5E-
4)0.841.54:0.00369)0.962.67:0.01107,((AmyColor:0.00369,((AmyColo2:0.0,Amy
Kerat:0.0,AmyJapon:0.0):5.5E-4,AmyLuri2:5.5E-4)0.917.71:5.5E-
4)0.963.58:0.01127,((AmyUltim:5.5E-
4,(AmyPlume:0.0112,((AmyMedit:0.00744,(AmyToly2:0.0,AmySpeci:0.0,AmyBalhi
:0.0):5.5E-4)0.749.58:5.5E-4,AmyTolyp:5.5E-4)0.675.6:5.4E-
4)0.858.64:0.00368)0.964.62:5.5E-
4,((AmyRubid:0.0,AmyAlbid:0.0,AmyBenzo:0.0,AmyHalot:0.0):5.5E-
4,AmyNiiga:0.00368)0.825.43:0.00367)0.807.37:0.0037)0.891.58:5.5E-
4)0.879.66:0.01015)0.925.81:0.01385,(Ult26095:0.00396,(UltAmyco:0.01508,(
(AmySacc2:0.0,AmySacch:0.0):5.5E-
4,((((AmyLacta:0.0,AmySpec3:0.0):0.00366,(AmyMinne:5.4E-
4,(AmyFlava:0.0,AmyMarin:0.0,AmyPalat:0.0):0.00738)0.432.10:0.00369)1.000
.480:5.4E-4,((AmyJejue:0.00369,AmySulph:5.5E-
4)0.905.61:0.00743,((AmyNigr2:0.0,AmyNigre:0.0):5.4E-4,(AmySaalf:5.5E-
4,AmySpec2:0.00369)0.942.64:0.01124)0.282.4:0.00743)1.000.481:5.4E-
4)0.835.54:5.5E-
4,(SspEryth:0.00198,SspTaber:0.00201)0.948.66:0.0109)0.927.68:0.00348)0.3
70.6:0.00747)0.767.54:0.00375)0.819.42:0.00815)0.771.61:0.00762)0.766.49:
0.00429)0.850.70:0.00713,LcrAeroc:0.01164)0.893.72:5.3E-
4,((AtePreti:5.4E-
4,(((AteMiru2:0.0,LcrFlava:0.0,LnzSpec2:0.0,LcrFradi:0.0):5.5E-
4,LcrAero2:5.5E-4)1.000.482:5.5E-
4,AteMirum:0.01553)0.842.71:0.00368)0.854.65:0.00369,(Ult26112:5.5E-
4,(((LnzFlavi:0.0,LnzSpeci:0.0,LnzViola:0.0,LnzWaywa:0.0):5.5E-
4,LnzCalif:0.04295)0.824.41:0.00367,LnzAlbid:5.5E-
4)0.850.71:0.0037)0.826.42:5.5E-
4)0.859.56:0.00378)0.876.63:0.00737)0.931.64:0.01114,KibAridu:0.00373)0.7
76.57:0.00366)0.777.55:0.00368,AhsHymen:0.01519)0.793.52:0.00367,((AckEnz
an:0.0,AckRipar:0.0):0.00369,(AckDiosp:5.5E-4,(((AckInage:5.5E-
4,((KutKofue:0.0,KutAlbid:0.0,KutVirid:0.0):5.4E-
4,(AhsAlkal:0.00827,AhsSpiti:5.5E-
4)0.913.70:0.0083)0.886.91:0.00831)0.947.49:0.01208,((((SspCebue:0.00369,
((SspSpec3:5.5E-
4,SspFlava:0.00369)0.845.67:0.00369,(SspSalin:0.01132,((SspSpeci:5.5E-
4,SspShand:0.00718)0.850.72:5.5E-4,((SspPogon:0.0,SspRosea:0.0):5.5E-
4,(SspSpino:0.00369,SspTherm:0.01659)0.438.9:5.5E-4)0.249.9:5.5E-

4)0.780.66:0.00408)0.800.42:0.00413)0.000.511:5.5E-
4)0.845.68:0.00409,SspHalop:5.5E-4)1.000.483:5.5E-
4,(SspSpec2:0.0021,UmeTange:0.01485)0.913.71:0.00152)0.451.13:0.00412,((((
(Ult26107:0.01108,((PddHydro:5.5E-
4,PddBenze:0.00374)0.982.41:0.0154,(UltPse50:0.01427,((AcyChian:5.5E-
4,(Ult26105:0.00367,Ult26106:0.00368)0.793.53:0.00369)0.894.63:0.01031,((
PddHalo2:5.5E-
4,PddHalop:0.01109)0.792.58:0.00448,((((PddCalli:0.0,PddSpec7:0.0):5.5E-
4,Ult26109:5.5E-
4)0.920.84:0.00366,((PddSpec5:0.0,PddAmmon:0.0,PddAlni0:0.0,PddSpec6:0.0)
:5.5E-4,(PddEndop:0.00366,PddSpec4:0.0148)0.667.14:5.5E-4)0.798.50:5.3E-
4)0.999.95:5.3E-4,((PddAuran:5.4E-
4,(Ult26111:0.01487,((PddChlor:0.01118,(Ult26102:5.5E-
4,PddSpeci:0.00366)0.916.81:5.5E-4)0.423.8:0.00366,(PddYunna:5.5E-
4,(Ult26103:5.5E-4,(((PddOroxy:0.00737,((Ult26104:0.0,PddSpec8:0.0):5.5E-
4,(Ult26101:0.00366,Ult26100:0.00366)0.881.78:5.5E-4)0.623.4:5.1E-
4)1.000.484:5.5E-4,(PddSpec2:0.00739,(PddPetro:5.5E-
4,PddSpec3:0.00737)0.138.8:5.5E-
4)0.862.61:0.00358)0.648.11:0.00744,(PddZijin:0.00366,PddSatur:5.5E-
4)0.868.58:0.00358)1.000.485:5.4E-4)0.925.82:0.00366)0.982.42:5.5E-
4)0.825.44:0.00362)0.575.3:5.4E-4)0.888.79:0.00739,(PddAutot:5.4E-
4,Ult26108:0.01492)0.782.63:0.00369)0.792.59:0.00365)0.910.68:0.01031)0.4
62.9:0.00373)0.797.41:0.00691)0.423.9:0.00572)0.885.57:5.4E-
4)0.891.59:5.4E-
4,(PddAcaci:0.00363,(PddAsacc:0.0111,Ult26110:0.00366)1.000.486:5.4E-
4)0.405.12:0.00366)0.861.67:0.00755,(PddSpino:0.00746,PddTherm:0.03092)0.
865.66:0.00743)0.908.76:5.3E-4,(Ult26098:0.00714,(PruRugos:5.5E-
4,(PruSpeci:0.00739,(PruSpec2:0.01515,((((ShpAzure:0.0,ShpSpeci:0.0,ShpGl
auc:0.0):5.4E-4,ShpVirid:0.01124)0.806.35:0.00368,(ShpHalop:5.4E-
4,ShpSpec2:0.00366)0.808.50:0.00368)0.782.64:0.00367,ShpSpec3:5.5E-
4)0.773.56:0.00362)0.886.92:0.00751)0.788.53:0.00367)0.880.73:0.00771)0.4
85.12:0.00371)0.880.74:0.00763)0.836.41:5.4E-
4)0.878.69:0.00808,AckGlobi:0.00369)0.189.5:5.5E-
4)0.802.47:0.00419)0.951.62:0.01276)0.088.6:5.4E-4,AmyFasti:5.5E-
4)0.792.60:0.00568,((Ult17120:0.0,Ult26096:0.0):0.01774,Ult26099:0.00885)
0.525.7:0.00739)0.749.59:0.00476,(TcrMunic:0.00372,(((SspAntim:0.00369,(S
spJiang:0.0,SspHirsu:0.0,SspHirs2:0.0,SspHorde:0.0):5.5E-
4)0.945.63:0.01633,SciMarin:0.0443)0.493.11:0.00742,TcrAgres:0.01145)0.75
4.41:0.00385)0.788.54:0.00611)0.879.67:0.01474)0.404.8:0.01281,((AmySpec4
:0.0,AmyMetha:0.0):5.5E-4,AmyEuryt:5.5E-
4)0.811.43:0.00884)0.816.44:0.00971,(((((((AruSpec2:0.01125,Ult26407:5.5E
-4)1.000.487:0.04401,(UltCell3:5.5E-
4,(BacEll26:0.01125,Ult26408:0.00369)0.717.19:5.5E-4)0.942.65:5.5E-
4)0.532.8:0.00746,Ult26409:0.0151)0.921.79:0.01159,(((KncXinji:5.3E-
4,((((KncAura2:0.00747,KncAuran:0.02296)0.903.67:0.01132,((KncRadio:0.0,K
ncSpeci:0.0):5.5E-4,KncGynur:0.00747)0.852.67:5.4E-
4)0.964.63:0.01119,KnkBacte:5.4E-4)0.984.44:0.01899,(KnpRhamn:5.5E-
4,UltKineo:0.02694)0.972.52:0.00193)0.973.38:0.00191)0.782.65:0.00379,(Ul
t26410:5.5E-
4,(QdrGranu:0.0236,(GrgSpeci:0.01163,(BeuCaver:0.00718,(GrgMural:5.5E-
4,(GrgRuani:0.0,GrgTherm:0.0):5.5E-4)0.920.85:5.5E-
4)0.393.6:0.00745)0.877.83:0.01073)0.885.58:0.01519)0.520.5:0.01089)0.811
.44:0.007,(((KnpBaben:0.00368,(KnpAuran:5.5E-
4,KnpSucci:0.01514)0.908.77:0.00752)0.884.73:0.00752,KnpRhizo:5.5E-

4)0.815.48:0.00671,((((BvcMarin:5.5E-
4,((Ult26225:0.00367,(((BvcOcean:5.5E-
4,BvcAvium:0.0037)0.855.65:0.00369,Ult26228:0.01114)0.336.8:5.5E-
4,((BvcIodin:0.0,Ult26224:0.0,Ult26226:0.0,BvcCasei:0.0,BvcPictu:0.0):5.5
E-4,Ult26227:0.00369)0.846.74:5.5E-4)0.370.7:5.5E-
4)0.846.75:0.00365,((BvcSpec2:0.0,BvcAuran:0.0):5.5E-
4,BvcAntiq:0.00369)0.815.49:5.5E-
4)0.857.72:0.00365)0.877.84:0.00365,OerSpeci:5.5E-
4)0.879.68:0.00737,(((BvcAlbum:0.01261,BvcSpec4:5.5E-
4)0.913.72:0.013,BvcSpec5:0.00431)0.817.31:0.00768,BvcSamya:0.00427)0.863
.71:0.00743)0.294.5:5.4E-
4,(BvcSpec3:0.01122,(Ult26229:0.00369,Ult26230:5.5E-
4)0.751.27:0.00395)0.883.77:0.0195)0.997.64:0.04888)0.899.71:0.01208)0.83
7.45:5.4E-
4)0.938.46:0.01582,KncMarin:0.02388)0.830.47:0.00734,((TprWhipp:0.06563,(
(NsnAethi:0.0,NsnSpeci:0.0):5.5E-
4,(Ult26197:0.303,(((BacteWE2:0.0153,((AhbEchig:5.5E-
4,(AhbAlbid:0.00739,(SioAtroc:0.0,SioFlava:0.0,CbrCyclo:0.0):5.5E-
4)0.841.55:0.00368)0.853.53:0.00709,(Ult26178:5.5E-
4,(YnlFlava:0.01122,YnlSpeci:5.5E-
4)0.821.39:0.00369)0.975.46:0.01497)0.899.72:5.5E-
4)0.925.83:0.00752,(NsnJeotg:0.0,NsnLutea:0.0):5.4E-
4)0.858.65:0.00739,((((AhbCaste:0.00772,((Ult26174:0.00371,BactK266:0.003
74)0.921.80:5.5E-4,((Ult26179:0.0,AhbCummi:0.0):5.5E-
4,AhbAlbus:0.00369)0.944.53:0.01471)0.194.5:0.00371)0.777.56:0.00347,((Ns
nHalob:0.00369,NsnHalop:5.5E-4)0.984.45:0.01511,((NsnkAlba:5.5E-
4,Ult26176:0.02282)0.830.48:0.00368,Ult26175:0.00369)0.431.11:5.3E-
4)0.850.73:0.00369)0.171.9:5.4E-4,AhbCasei:5.4E-
4)0.925.84:0.00369,(NsnLacus:5.4E-
4,(((NsnFlava:0.00377,((RotNasim:0.01132,((RotSpec3:0.0,Ult26215:0.0):5.5
E-4,UltAct37:0.0037)0.898.83:5.5E-
4)0.814.35:0.00369,(RotAmara:0.01509,(RotTerra:5.5E-4,((Ult26214:5.5E-
4,(RotMucil:5.5E-
4,Ult26213:0.00359)0.859.57:0.00724)0.958.73:0.01511,(RotDento:0.01136,(R
otSpec2:5.5E-4,RotSpeci:5.5E-
4)0.770.72:0.00399)0.779.58:0.00364)0.534.5:0.00793)0.905.62:0.01551)0.02
3.1:5.4E-
4)0.927.69:0.01148)0.581.10:0.00762,(((KocRhizo:0.0,KocRhiz2:0.0,Ult26219
:0.0):5.3E-4,(((Ult26218:0.03886,(Ult26221:0.0,KocMarin:0.0):5.4E-
4)0.829.53:0.00366,KocVaria:0.00742)0.528.7:5.5E-
4,(Ult26220:0.04274,KocSpec4:0.04212)0.750.32:0.00393)0.717.20:0.00361)0.
819.43:0.0036,((((KocSpeci:0.01889,(((KocFlava:0.0,KocTurfa:0.0,KocSpec3:
0.0,Ult26216:0.0):5.5E-4,((KocSpec2:5.5E-
4,(KocHimac:0.05736,KocPolar:0.00242)1.000.488:0.00146)0.380.8:5.5E-
4,((Ult26212:0.01535,KocSpec5:0.01132)0.752.50:0.00369,(Ult26217:5.3E-
4,((AhbPigme:0.0037,((((((Ult26040:0.01298,(Ult26037:0.01869,(UltBac25:0.
01832,Ult26156:0.02609)0.641.10:0.00556)0.775.60:0.0102)0.962.68:0.02618,
AhbNasip:5.4E-
4)0.845.69:0.00368,(((Ult26207:0.0074,(Ult26180:0.02274,AhbSpe39:0.0154)0
.663.13:5.5E-4)0.237.8:5.5E-
4,(AhbSpe41:0.0,AhbCryst:0.0,AhbSpe42:0.0):0.00369)0.588.6:5.4E-
4,(Ult25089:0.0,Ult26152:0.0,Ult26153:0.0,Ult26194:0.0,Ult26193:0.0,Ult26
196:0.0,Ult26199:0.0,Ult26200:0.0,AhbCitre:0.0,Ult26202:0.0,Ult26204:0.0,
Ult26203:0.0,Ult26206:0.0,Ult26205:0.0,Ult26208:0.0,AhbLuteo:0.0,Ult26210

:0.0):5.5E-4)0.553.9:5.4E-4)0.690.16:5.5E-
4,(Ult26198:0.01111,Ult26201:0.01494)0.771.62:0.00359)0.873.75:0.00372,Ul
t26211:0.00741)0.125.7:5.4E-
4,Ult26177:0.00744)0.824.42:0.00372)0.249.10:5.4E-4,AhbMonum:5.5E-
4)0.912.87:0.00371)0.291.5:5.5E-4)0.863.72:0.00372)0.209.7:5.5E-
4)0.202.9:5.5E-4,KocAegyp:5.5E-4)0.590.5:5.5E-
4)0.928.58:0.00813,(KocSpec6:0.02696,(KocPalus:0.0,Ult26223:0.0):5.4E-
4)0.658.7:0.00369)0.606.8:5.5E-4,KocCarni:0.00363)0.248.6:5.5E-
4,(KocCarn2:0.0,Ult26222:0.0):5.5E-4)0.597.10:5.4E-
4)0.876.64:0.00782)0.943.62:5.5E-4,((((CtcAlkal:0.0,MirSpec4:0.0):5.4E-
4,BacteWB3:0.04376)0.823.43:0.00368,((((AhbAgili:0.0,AntBac40:0.0,AhbSpec
i:0.0,Ult26113:0.0,AhbSpe22:0.0):5.5E-4,((Ult26141:5.5E-
4,((Ult26129:0.00368,(AhbSpe34:5.5E-
4,((Ult26188:0.0,AhbUreaf:0.0,AhbSpe35:0.0,Ult26189:0.0,AhbSpe36:0.0,AhbN
itro:0.0,AhbSpe37:0.0,AhbSpe38:0.0,Ult26192:0.0):5.5E-
4,AhbHisti:0.0112)0.000.512:5.5E-4)0.932.68:5.5E-4)0.745.44:5.5E-
4,Ult26187:0.01114)0.951.63:0.0112)0.924.68:0.00739,(ZhiHalot:5.4E-
4,((Ult26181:0.01509,((((BactWMA5:0.01955,AhbPsych:0.01562)0.748.43:0.003
1,((Ult26143:5.5E-
4,(((((((AhbSpec2:0.0,AhbParie:0.0,AhbSpec3:0.0,AhbSpec4:0.0):0.0037,(Ahb
Flav2:0.0,AhbFlavu:0.0):5.5E-
4)0.851.68:0.00367,(AarPhyto:0.01123,(RenSalm3:5.5E-
4,(RenSalm2:0.00369,AhbRussi:0.0037)0.715.13:5.5E-4)0.925.85:5.5E-
4)0.846.76:0.00742)0.867.60:5.4E-
4,(((((((Ult26131:0.0,Ult26151:0.0):0.0037,((((Ult26155:0.00368,(Ult26160
:0.00369,((Ult26135:0.00752,Ult26128:0.04669)0.764.56:0.00372,Ult26117:0.
0037)0.424.10:5.5E-4)0.428.9:5.5E-4)0.000.513:5.5E-
4,Ult30748:0.00369)0.000.514:5.5E-4,Ult26136:0.00369)0.000.515:5.5E-
4,Ult26149:0.00369)0.375.8:5.5E-4)0.113.8:5.5E-
4,Ult26115:0.0074)0.426.15:5.5E-4,Ult26126:0.00368)0.432.11:5.5E-
4,AhbSpec6:5.5E-4)0.437.8:5.5E-
4,(AhbChlor:0.0,AhbPolyc:0.0,AhbScler:0.0,AhbSpe10:0.0,AhbSpe11:0.0,AhbSp
e12:0.0,Ult26123:0.0,Ult26124:0.0,Ult26120:0.0,Ult26122:0.0,Ult26118:0.0,
Ult26119:0.0,Ult26125:0.0,Ult26127:0.0,Ult26130:0.0,AhbSpe13:0.0,Ult26132
:0.0,AhbSpe16:0.0,AhbSpe15:0.0,Ult26137:0.0,Ult26138:0.0,AcumEC50:0.0,Ahb
Pasce:0.0,AhbHumic:0.0,AhbGlobi:0.0,Ult26140:0.0,Ult26142:0.0,AhbSpe19:0.
0,StpLiban:0.0,AhbSpe20:0.0,AhbSpe21:0.0,Ult26144:0.0,Ult26157:0.0,Ult261
58:0.0,Ult26159:0.0,Ult26182:0.0,Ult26183:0.0,Ult26290:0.0):5.5E-
4)0.000.516:5.5E-
4,((AhbSpe29:0.02334,(((Ult26121:0.0,AhbSpe32:0.0,Ult26184:0.0,AhbMethy:0
.0,Ult26186:0.0,AhbAlkal:0.0):5.5E-
4,Ult26185:0.00369)0.839.60:0.00383,(AhbWoluw:0.01499,(((Ult26039:0.0,Ult
26195:0.0,Ult26209:0.0,RatRatha:0.0,AhbSpe40:0.0,AhbGanda:0.0):5.5E-
4,(Ult19764:0.02277,AhbRoseu:0.00369)0.910.69:5.5E-4)0.948.67:5.5E-
4,Ult26154:0.00743)0.879.69:0.00712)0.864.70:5.5E-
4)0.890.77:0.00757)0.853.54:0.0074,((AhbSpec7:0.0,AhbSpec5:0.0,AhbStack:0
.0,AhbRhomb:0.0,AhbSpe31:0.0):5.4E-4,(((AhbSulfu:0.0,AhbSpe17:0.0):5.5E-
4,(AhbGango:0.0037,Ult26133:0.00748)0.072.3:5.3E-
4)0.923.97:0.00747,((AhbKergu:0.0,AhbSpe18:0.0):5.3E-4,(AhbSpe24:5.5E-
4,(AhbCreat:5.5E-4,(AhbSpe14:0.02672,((AhbBerge:5.5E-
4,(AhbSpe23:0.01122,AhbArdle:0.0037)0.472.6:5.5E-
4)0.815.50:0.00369,((UltArth2:5.5E-
4,((((((AhbProto:0.0,AhbSpe25:0.0,AhbSpe26:0.0,Ult26163:0.0,AhbNicot:0.0,
AhbMysor:0.0,AhbSpe30:0.0,Ult26168:0.0):5.5E-4,(AhbSpe27:5.5E-

4,(Ult26167:0.00742,(AhbSpe28:0.0256,Ult26166:0.00717)0.925.86:5.5E-
4)0.208.5:5.5E-4)0.000.517:5.5E-4)0.000.518:5.5E-
4,AhbUrato:0.00369)0.329.9:5.5E-4,UltArthr:0.00746)0.190.7:5.5E-
4,Ult26161:0.00372)0.342.7:5.5E-4,Ult26164:0.00369)0.438.10:5.5E-
4)0.190.8:5.5E-4,Ult26165:0.00369)0.631.9:5.5E-
4)0.426.16:0.00411)0.551.8:5.4E-4)0.105.8:5.4E-
4)0.921.81:0.00412)0.836.42:5.5E-4)0.923.98:0.00757)0.133.4:5.4E-
4)0.860.48:0.00375)0.629.12:5.5E-4)0.215.5:5.5E-4,UltAct35:5.5E-
4)0.000.519:5.5E-4,AhbSpec8:0.00375)0.472.7:5.5E-
4,Ult26114:0.00368)0.175.2:5.5E-4)0.176.10:5.5E-
4,(Ult26094:0.02241,(Ult26116:0.01122,Ult26139:0.00743)0.980.38:0.00204)0
.982.43:0.00178)0.052.4:5.5E-4)0.200.5:5.1E-4,AhbSpec9:5.5E-
4)0.871.69:5.4E-
4,Ult26134:0.02285)0.355.10:0.00371)0.750.33:0.00379,ZhihAlba:0.00371)0.8
37.46:0.0037)0.849.57:0.00749)0.970.51:5.4E-4)0.546.10:5.4E-
4,AhbYyyyy:0.01125)0.812.35:0.00372,((MirLyla3:0.00743,((AhbSpe33:0.01108
,((MirSpeci:5.5E-
4,((Ult26172:0.00369,(((MirSpec2:0.0,MirLute4:0.0,MirLute5:0.0,MirLute6:0
.0,MirLute2:0.0,MirLute3:0.0,Ult26170:0.0,MirAntar:0.0,UltMicr2:0.0):5.5E
-4,(((UltMicro:0.00757,Ult26169:0.01205)0.888.80:5.4E-
4,(MirSpec3:0.05763,Ult26171:0.00168)0.987.64:0.00216)0.291.6:5.3E-
4,AcumJS14:0.01134)0.330.6:5.5E-4)0.000.520:5.5E-
4,BacEnr45:0.04644)0.000.521:5.5E-4)0.273.6:5.5E-
4,MirFlavu:0.00369)0.267.5:5.5E-4)0.025.2:5.5E-
4,MirIndic:0.00753)0.932.69:5.5E-
4)0.893.73:0.00829,(MirLyla2:0.0,MirLylae:0.0):5.3E-
4)0.933.77:0.00851)0.401.9:5.4E-4,Ult26173:5.5E-4)0.900.62:5.4E-
4)0.935.62:0.00777)0.066.6:5.4E-
4,MirXinji:0.00744)0.986.45:0.01981)0.366.6:5.4E-4)0.437.9:5.5E-
4)0.486.10:5.4E-4)0.994.59:5.4E-
4)0.885.59:0.00814)0.776.58:0.00421,KocHalot:0.0073)0.991.51:0.02182)0.03
9.2:5.4E-
4,(((((LbtSpec2:0.00747,((Ult26318:0.00741,(((((LbtKoma4:0.00773,(Ult2636
0:0.02274,((PlvHelvo:0.00745,(((Ult26363:0.00743,(((GlialIc2:0.0,Ult2635
1:0.0,KlgXanth:0.0,UltAct68:0.0,CbmMesop:0.0):5.5E-
4,((((((Ult26353:0.0,UltMic10:0.0,UltAct64:0.0,UltMic11:0.0):5.5E-
4,UltAct65:0.00369)0.884.74:0.00739,(UltAct66:0.02291,(((Ult26356:0.01129
,Ult26354:0.0037)0.944.54:0.01524,(Ult26355:0.01113,CanLimno:5.4E-
4)0.266.6:0.00739)0.883.78:0.00877,(CanFlavi:0.01519,UltAct67:0.01519)0.8
55.66:0.00755)0.842.72:5.3E-
4)0.881.79:0.00849)0.761.48:0.0043,AgsCasei:0.00745)0.506.8:5.5E-
4,Ult26322:0.04004)0.906.85:0.00843,(((((((MctSpe21:0.00795,MctHalop:0.03
522)0.771.63:0.0074,(((MctOleiv:0.01634,MctArbo2:0.13954)0.921.82:0.0284
7,MctArbo3:0.06708)0.903.68:0.01523,((((Ult26314:0.0,OkiFriti:0.0):0.0036
1,(PliFlavu:0.00361,(MytRebl2:0.0,MytReblo:0.0):5.4E-
4)0.736.31:0.00747)0.990.54:5.4E-
4,(((((((MctPalud:0.0,MctSpe23:0.0,MctArbor:0.0,MctArbo5:0.0,Udntfd15:0.0
):5.5E-4,(MctArbo6:0.00369,MctSchle:5.5E-4)0.950.55:5.5E-4)0.943.63:5.5E-
4,(MctSpe16:0.02243,(MctSpe24:0.0074,MctArbo4:0.00747)0.797.42:0.00361)0.
529.5:5.3E-4)0.862.62:0.00363,MctKoree:5.5E-
4)0.854.66:0.00364,((((Ult26324:0.00744,Ult26306:0.00372)0.801.53:0.00359
,(MctBarke:0.00362,((GroBiof5:0.05546,MctTerra:0.00374)0.865.67:5.4E-
4,(((MctThala:5.5E-
4,Ult26298:0.00369)0.860.49:0.00369,((Ult26302:0.00375,Ult26148:0.04725)0

.876.65:0.00739,Ult26294:0.01118)0.371.9:5.5E-4)0.853.55:5.5E-
4,(MctFlave:0.0,MctTerr2:0.0,MctMarin:0.0,MctSpe20:0.0,UltMicr7:0.0):5.5E
-4)0.720.16:5.5E-4)1.000.489:5.4E-4)0.353.4:0.00369)1.000.490:5.5E-
4,Ult26309:0.00369)0.743.45:5.5E-
4,(MctPumil:0.0,Ult26310:0.0,Ult26312:0.0):5.5E-4)0.944.55:5.5E-
4)0.914.74:0.00241,((UltMicr6:0.0,MctPygma:0.0):5.5E-
4,MctSpe19:0.00369)0.914.75:0.00248)0.914.76:0.0024,((MctLutic:0.0,MctXyl
an:0.0):5.5E-4,((Ult26299:0.00722,((((((MctInsul:0.0,MctAwaji:0.0):5.5E-
4,(MctAquim:0.00737,(MctSpe13:0.01114,MctSpe14:0.00379)0.318.9:0.00369)0.
942.66:5.4E-4)0.778.60:5.5E-
4,MctThal2:0.0037)0.863.73:0.00371,(Ult26301:0.00374,((((MctNator:5.5E-
4,(MctSpeci:0.0,Ult26296:0.0,Ult26303:0.0,MctSpe11:0.0,MctTrich:0.0,Ult26
304:0.0,MctResis:0.0,MctSpe17:0.0,Ult26308:0.0,MctFolio:0.0,Ult26307:0.0,
MctParao:0.0,MctSpe18:0.0,MctLacti:0.0):5.5E-4)0.623.5:5.5E-
4,MctDextr:5.5E-4)0.647.4:5.5E-4,MctSpec4:5.5E-4)0.488.9:5.5E-
4,MctLact2:0.00369)0.480.5:5.5E-4)0.000.522:5.5E-4)0.288.7:5.5E-
4,(((((MctSpe15:0.0037,((MbtBacte:5.5E-4,MctLaeva:5.5E-4)1.000.491:5.3E-
4,(MctrUlmi:5.4E-
4,MctSpe22:0.00362)0.433.13:0.00369)0.887.77:0.00361)0.489.6:5.5E-
4,MctHomi2:0.00369)0.867.61:5.4E-
4,(MctHomin:0.0,MctSpe12:0.0,Ult26313:0.0,MctFlavu:0.0):5.5E-
4)0.892.60:5.5E-4,Ult26311:0.0149)0.999.96:5.4E-
4,(MctDext2:0.0149,MctLaev2:5.4E-
4)0.321.5:0.00717)0.834.43:0.00366)0.303.8:5.5E-
4,(MctFlav2:0.00386,(((MctSpec2:0.0074,(((((MctXinji:0.00329,(MctGubbe:0.
01079,(Ult26305:5.5E-
4,UltAct61:0.01105)0.748.44:0.00788)0.364.8:0.00757)1.000.492:5.5E-
4,MctIndic:5.5E-
4)0.855.67:0.00367,((((MctSpec8:0.00369,MctAuru2:0.00369)0.862.63:5.5E-
4,MctAurum:5.5E-4)0.901.71:5.4E-4,((Ult26316:5.5E-
4,((AgoLuteo:0.0,LfsSpeci:0.0,LfsGinse:0.0,UdnActin:0.0):5.4E-
4,(((((Ult26191:0.0,Ult26325:0.0,Ult26327:0.0,Ult26328:0.0,Ult26329:0.0,U
lt26330:0.0,Ult26331:0.0,Ult26332:0.0):0.00361,(((UltAct63:0.0,LfsSpec3:0
.0):5.5E-4,((UltMicr9:0.00741,LfsSpec2:0.0037)0.841.56:5.5E-
4,(((Ult26345:0.0,Ult26347:0.0,UltLeifs:0.0):5.5E-
4,Ult26346:0.00743)0.833.59:0.00369,CrkBacte:0.00369)0.871.70:5.5E-
4)0.000.523:5.5E-4)1.000.493:5.4E-
4,Ult26357:0.00361)0.405.13:0.00369)1.000.494:5.5E-
4,(SueFrigo:0.0,CttSpeci:0.0,AgeSpeci:0.0,LfsBigeu:0.0):5.5E-
4)0.000.524:5.3E-
4,(((RdgVesta:0.01116,(AcumP600:0.0037,Ult26352:0.00371)0.846.77:5.4E-
4)0.797.43:0.00743,(SueBoreu:0.0,ClaSpec2:0.0):5.5E-
4)0.826.43:0.00367,(AgeBicol:0.00745,LfsAntar:0.00747)0.379.3:5.5E-
4)0.932.70:5.5E-4)0.768.41:5.4E-4,Ult26341:5.5E-
4)0.827.49:0.00368)0.858.66:0.00364)0.774.67:0.0074,UdnActi2:0.00738)0.91
5.68:5.4E-
4)0.919.81:0.00368,(MctHatan:0.0,MctSpec7:0.0,MctBino2:0.0,MctBinot:0.0,U
ltMicr4:0.0):5.4E-4)0.186.4:5.4E-
4)0.834.44:0.00367,(((MctSpec5:0.0,GlialIce:0.0,MctInvic:0.0):0.00369,(Mc
tKitam:0.0,MctSpec6:0.0,Ult26297:0.0,MctSpec9:0.0,MctKerat:0.0):5.4E-
4)0.856.55:0.0037,MctKera2:5.5E-4)0.862.64:0.0037)1.000.495:5.4E-
4,MctHalot:0.00366)0.873.76:0.00738)0.894.64:5.4E-
4,((MctOxyda:0.0,MctSpe10:0.0,MctMarit:0.0):5.5E-
4,(Ult26300:0.00369,(MctSpec3:0.00195,Ult26295:0.02244)0.972.53:0.00188)0

.874.80:5.5E-4)0.933.78:5.4E-4)0.893.74:5.5E-
4,MctLuteo:0.00795)0.877.85:0.00688)0.883.79:5.5E-
4)0.895.82:0.00197)0.898.84:0.00237,(MctKribb:0.0075,(UltMicr5:0.00369,Mc
tArabi:5.5E-4)0.804.43:0.00372)0.793.54:0.0037)0.421.7:5.5E-
4)0.582.8:5.4E-4)0.871.71:0.00385)0.379.4:5.5E-4,MytSapro:5.5E-
4)0.038.3:5.4E-4)0.908.78:5.5E-
4,UltAct28:0.01106)0.840.61:0.00718)0.763.64:0.00445,(Ult26315:0.01486,(A
gsJejue:0.0042,AgsVersi:0.00698)0.893.75:0.01163)0.785.56:0.00398)0.776.5
9:0.00394,(AgsLahau:0.0037,((AgsSpeci:5.5E-
4,PccSpe11:0.06703)0.813.44:0.004,(AgsJene2:0.0,AgsCitre:0.0):5.5E-
4)0.056.6:5.5E-4)0.850.74:0.00402)0.540.6:5.5E-4,AgsJenen:5.4E-
4)0.801.54:0.0043,(((CttAmmon:0.0,BacCAGY8:0.0,CttSpec5:0.0):5.5E-
4,CttLuteu:5.5E-4)0.853.56:0.00369,(CttSpec2:5.5E-
4,(CttFlacc:0.0,CttSpec4:0.0,CttFlac2:0.0):5.5E-4)0.698.16:5.5E-
4)0.794.47:0.0037)0.791.48:0.00429,((((((LfsLiche:5.4E-
4,(((((AgoSalen:0.0,AgoTerre:0.0):0.00821,(AgoLapid:5.3E-
4,(AgoItali:5.3E-4,(AgoSpeci:5.5E-
4,(AgoMedio:0.0959,(AgoLute2:0.00361,(AgoAuran:5.5E-
4,AgoBracc:0.00378)0.791.49:0.00384)1.000.496:0.03571)0.240.6:5.4E-
4)0.999.97:0.02715)0.854.67:0.00366)0.785.57:0.00859)0.842.73:0.01345,(Ul
trS321:5.5E-4,((UltAgrom:0.0,AgoHumat:0.0,AgoNeoli:0.0):5.4E-
4,AgoSubbe:0.00742)0.481.7:0.00369)0.991.52:5.5E-
4)0.869.63:0.01095,(((Ult26317:0.01894,(((MbtBact2:5.5E-
4,MbtBact3:0.0037)0.975.47:0.01139,(Pshggg63:0.0037,CttSpec6:5.5E-
4)0.957.53:5.5E-
4)0.995.65:0.03129,(HmbAlbus:0.00391,CttGinse:0.01891)0.431.12:0.00358)0.
856.56:0.00778)1.000.497:5.4E-4,UltrS324:5.5E-
4)0.926.82:0.00741,(((LfsAquat:0.00741,LfsnXyli:0.0037)0.843.50:5.4E-
4,LfsnXyl2:5.5E-4)0.901.72:5.5E-4,LfsAqua2:5.5E-4)0.845.70:5.4E-
4)0.805.45:0.00376)0.088.7:0.00377,((UltMicr8:0.00406,UltrS323:0.00333)0.
333.5:0.0037,(RatToxic:0.01132,Ult26364:0.0075)0.908.79:5.5E-
4)0.885.60:0.00701)0.928.59:5.4E-4)0.404.9:5.4E-
4,((ScuLuteo:0.02736,((RatFestu:0.0,RatCaric:0.0,RatTriti:0.0,RatTrit2:0.
0):5.5E-4,UltRu547:0.01497)0.887.78:5.5E-
4)0.260.5:0.00368,MbtrcStr:0.0037)0.846.78:5.4E-
4)0.937.61:0.00497,((((((CsmSpec4:0.01507,(((Ult26370:0.0,CeuSpec8:0.0,Ul
t26373:0.0,CeuHomin:0.0,BacWS011:0.0):5.5E-
4,CeuDenv2:0.00362)0.381.13:0.0037,(((AioFerme:0.0,CeuBogo2:0.0):5.5E-
4,CeuBogor:0.00742)0.939.62:5.5E-
4,((((SaaMulti:0.01147,SenSalmo:0.00747)0.929.67:0.01454,((((BacNL426:0.0
1122,((((((CsmTerre:0.0,CsmSpeci:0.0,CsmCell3:0.0,CsmCell4:0.0,CsmCell5:0
.0):5.5E-4,(CsmSpec2:0.00744,CsmCell2:0.02667)0.924.69:5.5E-
4)0.717.21:5.5E-
4,CsmCellu:0.00369)0.941.64:0.00745,(((((Ult26242:0.0,SgbAntar:0.0,SgbMar
in:0.0,OerJenen:0.0,OerTurba:0.0,Ult26371:0.0,Ult26372:0.0):5.5E-
4,OerEnter:5.5E-4)0.000.525:5.5E-4,(Ult26150:0.00369,(SgbcSoli:5.5E-
4,Ult26241:0.03514)0.826.44:0.00368)0.698.17:5.5E-4)0.960.62:5.4E-
4,(PskMarin:0.00369,AmcBact2:0.00747)0.803.40:0.00372)1.000.498:5.5E-
4,(SgbKeddi:5.3E-
4,RuaAlbid:0.01504)0.468.11:0.01126)0.904.73:0.00742)0.831.40:5.5E-
4,LmcSpeci:5.5E-4)0.981.48:5.5E-4,((RarFaec2:0.0,RarFaeci:0.0):5.5E-
4,RarIncan:0.01126)0.978.35:0.01122)0.845.71:0.00743)0.340.7:5.5E-
4,CeuSpec5:0.01508)0.798.51:0.00216,(((MlgXilig:0.01193,((XlbcUlmi:0.0037
1,(XlsCellu:5.5E-4,(Ult26288:5.5E-4,XlmPachn:5.5E-

4)0.925.87:0.00369)0.052.5:5.5E-
4)0.760.50:0.0033,((PmrSukum:0.00372,(PmrSuku2:0.0,PmrSuku3:0.0):5.5E-
4)0.919.82:5.3E-4,(UltrS320:5.5E-
4,(PmrVindo:0.00369,Ult26289:0.01502)0.464.9:5.5E-
4)0.870.50:0.00715)0.958.74:0.01547)0.912.88:0.01143)0.379.5:0.0074,((((I
spVaria:0.0,Ult26287:0.0,IspVari3:0.0):5.5E-
4,PmrFlava:0.00369)0.914.77:0.00181,CeuSpec3:0.00706)0.028.3:8.9E-
4,CeuSpec2:0.00373)0.987.65:9.0E-4)1.000.499:5.4E-4,IspVari2:5.5E-
4)0.906.86:0.01005)0.895.83:0.01114,(CeuSpec4:0.00369,IspDokdo:5.5E-
4)0.749.60:0.00279)0.914.78:0.01137)0.843.51:5.4E-
4,(UltAct69:0.01496,((((CeuCompo:5.5E-
4,((CdcStr00:0.00369,Ult26092:0.01504)0.885.61:5.5E-4,CeuCella:5.5E-
4)0.000.526:5.5E-4)0.960.63:5.4E-
4,(CdcStr02:0.03851,((CeuSpec7:0.0,CeuDenve:0.0):5.5E-
4,(CeuGelid:0.00369,CeuGeli2:0.00377)0.898.85:0.00755)0.753.50:0.00373)0.
794.48:0.00364)0.948.68:0.00504,((Ult26291:0.00773,Ult26366:0.00738)0.400
.7:0.0075,(CeuXylan:5.5E-4,(CeuXyla2:0.0,CeuTerra:0.0,CeuAeril:0.0):5.5E-
4)0.948.69:5.5E-
4)0.926.83:0.00482)0.946.62:0.00477,(CeuSpec6:0.0,CeuFlavi:0.0,CeuBiazo:0
.0):5.4E-4)0.864.71:5.4E-
4)0.859.58:0.00749)0.953.48:0.01152,CsmSpec3:0.00368)0.689.12:5.4E-
4)1.000.500:5.4E-4)0.845.72:0.00356)0.713.19:5.5E-4,Ult26367:5.5E-
4)0.000.527:5.5E-4,((DemAestu:5.5E-
4,(CeuSpeci:0.00768,((Ult26246:0.00375,UltMicr3:0.00369)0.919.83:0.00342,
(DemLutea:0.00749,Ult26245:0.01505)0.990.55:5.3E-
4)0.330.7:0.00772)0.920.86:0.01505)0.268.5:0.00365,(UltSalin:0.07445,Ult2
6369:0.00391)0.857.73:0.00751)0.922.96:0.01141)0.750.34:0.00368,Ult26368:
0.00372)0.747.40:0.00366,(UltAct62:0.01916,PlvbSoli:0.00749)0.895.84:0.00
75)0.792.61:0.00374,(AgoRhiz2:0.0,AgoRhizo:0.0):0.01126)0.937.62:0.00494)
0.937.63:0.00495,Ult26348:0.0074)0.227.8:5.3E-4,LfsKribb:5.5E-
4)0.198.10:5.5E-4,(((CyoAntiq:0.0037,(AgoCerin:0.0074,AgoCeri2:5.5E-
4)0.948.70:0.01126)0.785.58:5.5E-
4,(Ult26147:0.0,LfsnPoae:0.0,Ult26350:0.0):5.5E-
4)0.914.79:0.00342,(FrgrbAff:0.03516,CttSpec3:0.0702)0.775.61:0.01195)1.0
00.501:5.5E-4)0.840.62:0.00371)0.000.528:5.4E-4)0.529.6:5.5E-
4)0.000.529:5.4E-4,UltLeif2:0.00368)0.444.14:5.4E-4)0.621.10:5.4E-
4,((((CbmPsych:0.0037,(GcbSuper:0.00373,(BacteIFO:5.5E-
4,Ult26362:0.00741)0.928.60:0.00744)0.040.2:5.5E-4)0.608.13:5.4E-
4,(HymSpec2:0.0,CbmSpec2:0.0,AntBac42:0.0,CbmSpeci:0.0,Ult26358:0.0):5.5E
-4)0.634.7:5.5E-4,CbmAff00:0.00369)0.347.6:5.4E-
4,((Ult26334:0.00772,(AcuMWHTa:0.02682,(Ult26336:0.0037,(((FrgMesop:5.5E-
4,(Ult26340:0.00742,(EnhBact3:0.0037,((CctIleco:0.0,MioVirid:0.0,FroAustr
:0.0,FrgFaeni:0.0):5.5E-4,FrgSpeci:0.00369)0.532.9:5.3E-
4)0.847.76:0.00369)0.536.8:5.5E-
4)0.921.83:0.00361,(((Ult26337:0.0,AntBac41:0.0):5.5E-
4,(Ult26344:0.00746,LfsAurea:0.0037)0.805.46:5.4E-4)1.000.502:5.4E-
4,(Ult26339:0.00369,(Ult26342:5.5E-
4,(Ult26093:0.0,Ult26240:0.0,Ult26326:0.0,Ult26338:0.0,SbmAmurs:0.0,ClaSp
eci:0.0):5.5E-4)0.305.6:5.5E-
4)0.907.69:0.0036)0.405.14:0.00368)1.000.503:5.4E-
4,(ArctiSe6:0.00756,(Ult26190:0.0,Ult26333:0.0,Ult26335:0.0):5.5E-
4)0.222.3:5.4E-4)0.465.10:5.4E-
4)0.807.38:0.00399)0.322.8:0.00372)0.957.54:0.01479,(Ult26349:5.4E-
4,((LbdGwakj:5.5E-

4,((UltrS322:0.00752,Ult26323:0.00373)0.780.67:0.00371,(YonAlkal:5.5E-
4,MctAoyam:0.01117)0.847.77:0.00369)0.106.5:5.5E-
4)0.774.68:0.00372,(MieAlkal:0.0,MiePutea:0.0):0.0037)0.812.36:0.00371)0.
848.61:0.00716)0.880.75:5.4E-
4)0.897.64:0.00368)0.914.80:0.00833,((BacTSBY9:0.0,ClaMich2:0.0,ClaMichi:
0.0,ClaMich4:0.0):5.5E-4,(ClaMich3:0.00369,Ult26359:0.01954)0.389.7:5.5E-
4)0.816.45:5.5E-4)0.108.4:0.00417,((ZimFaec2:5.5E-4,ZimFaeca:5.5E-
4)0.980.39:5.5E-4,(ZimBifid:5.5E-
4,BvcEquis:0.00744)0.915.69:0.00745)0.922.97:0.00754)0.752.51:5.5E-
4)0.815.51:0.00807,(GsbMolin:0.01092,Ult26361:0.01563)0.474.12:0.00741)0.
917.72:5.5E-4)0.859.59:0.00842)0.753.51:0.00858,(LbtChrom:5.5E-
4,(Ult26321:0.01119,((((LbtAlluv:5.5E-
4,LbtSpeci:0.00369)0.908.80:0.00407,LbtSpec3:0.01819)0.909.61:0.00408,Lbt
Tardu:5.5E-4)0.428.10:5.3E-
4,BacteK32:0.00366)0.844.71:0.00366)0.729.19:5.4E-4)0.839.61:5.4E-
4)0.883.80:0.0082,LbtSpec4:0.00369)0.518.9:5.5E-4,LbtChiro:5.5E-
4)0.829.54:5.5E-4,Ult26320:0.05071)0.920.87:0.00833)0.538.5:5.4E-
4,LbtKoma2:5.5E-4)0.218.4:5.4E-4)0.317.9:5.4E-4,(Ult26319:5.5E-
4,LbtKoma3:0.01132)0.882.72:0.00745)0.971.48:5.5E-
4,LbtKomag:0.0559)0.920.88:0.01673,BogCasei:0.00372)0.893.76:0.00841,((((
(((BfdIndic:0.00721,(BfdSimia:0.00369,((BfdSubti:0.01505,((BfdPsych:0.018
86,((MsrCrice:0.00355,AsdOmnic:0.00762)0.989.54:0.01923,(Ult26255:5.3E-
4,GrdVagin:0.00742)0.998.77:0.02712)0.646.11:5.4E-
4)0.919.84:0.00745,BfdMongo:0.0151)0.754.42:5.5E-
4)0.787.51:0.01136,((BfdBombi:0.01904,(BfdAngul:0.0,BfdMeryc:0.0):5.5E-
4)1.000.504:5.5E-4,(((BfdbBoum:0.00747,(BfdSpec2:5.5E-
4,(((Ult26258:0.00741,(BfdAnima:0.0,SwnFec14:0.0):0.0037)0.843.52:5.5E-
4,BacMpnI3:5.5E-4)0.935.63:5.5E-
4,BfdTherm:0.01899)0.826.45:0.00369)0.825.45:0.00372)0.782.66:0.00369,(Bf
dCaten:5.5E-4,(Ult26250:0.0,Ult26251:0.0,BfdCate2:0.0,BfdGalli:0.0):5.5E-
4)0.896.59:5.5E-4)1.000.505:5.4E-4,(BfdScard:5.4E-
4,((((((Ult26260:0.02678,(BfdDenti:5.5E-4,Ult26252:5.5E-4)0.974.35:5.4E-
4)0.728.23:0.00744,BfdTsuru:0.00368)0.472.8:5.4E-
4,(((BfdAdole:0.0,Ult26249:0.0,Ult26247:0.0,BfdSpeci:0.0,BfdLong2:0.0):5.
3E-4,(((Ult26259:0.0,BfdLongu:0.0,BfdBreve:0.0,Ult26254:0.0):5.3E-
4,(((BfdSpec5:0.00743,(BfdReute:0.00742,(BacNL422:0.0037,BfdPseud:0.07412
)0.482.10:5.4E-4)0.238.5:5.5E-4)0.455.16:5.5E-
4,(BacNL421:0.0,BfdChoer:0.0):5.5E-4)0.614.7:5.5E-4,BacNL423:5.5E-
4)0.863.74:0.00373)0.811.45:0.00372,(Ult26248:5.4E-
4,Ult26253:0.0187)0.481.8:0.00715)0.993.48:5.4E-4)1.000.506:5.3E-
4,Ult26257:0.05632)0.439.9:0.0075)0.777.57:0.00371,(Ult26256:5.3E-
4,BfdBifid:0.00369)0.911.88:0.0075)0.765.43:0.00378,BfdAster:0.01136)0.73
2.18:0.00373,(BfdSubt2:5.4E-
4,(Ult26261:0.01423,(BfdSpec3:0.01274,BomSpeci:0.01834)0.915.70:0.01536)0
.878.70:0.00865)0.796.42:0.00374)0.750.35:0.00372)0.860.50:0.00365)0.225.
4:0.00374)0.760.51:0.00363)0.995.66:5.4E-4)0.888.81:5.4E-
4)0.588.7:0.00918,Bfhggg84:0.02534)0.879.70:0.02287,(BfdSpec4:0.00885,(Bf
dMery2:0.03264,(PadDenti:5.5E-4,(ScaInopi:5.5E-
4,ScaInop2:0.00369)0.968.56:0.01124)0.973.39:0.02963)0.483.13:0.0119)0.49
2.10:0.01081)0.763.65:0.0106,ArdAerip:0.03959)0.766.50:0.01674,(LueJapon:
0.63435,(UltB2549:0.76996,BfdAnim2:0.37237)0.212.7:0.05641)0.926.84:0.115
42)0.947.50:0.06046,((((Ult26292:0.00363,Ult26293:0.00758)0.893.77:0.0145
7,(SgbSpeci:0.02107,(JonDenit:0.01172,JonQuing:0.0033)0.858.67:0.01186)0.
950.56:0.02774)0.734.34:0.00478,(((MobCurti:0.0,UltMobil:0.0):0.00369,Mob

Mulie:5.5E-
4)0.972.54:0.02864,(((((((Ult26265:0.02897,(BacNL424:0.00366,BacNL425:5.4
E-
4)0.992.58:0.05534)0.695.13:0.0167,AinHongk:0.0405)0.865.68:0.01381,AinMa
rim:0.01419)0.727.24:0.00519,(((AinColeo:0.01378,AinEurop:0.01379)0.929.6
8:0.01898,(Ult26268:0.03648,(Ult26266:0.01292,Ult26267:0.00586)0.543.9:0.
01714)0.901.73:0.02578)0.818.33:0.00857,(UltrS319:0.01977,(Ult26270:0.022
66,((((AinCardi:5.5E-
4,((Ult26275:0.01526,((AinSpeci:0.0431,AinRumin:5.5E-
4)0.987.66:0.01895,((AinDenti:0.01095,(AinDenta:0.00783,(AinGenom:0.01164
,Ult26276:0.00343)0.932.71:0.01151)0.924.70:0.01192)0.871.72:0.01556,(((U
lt26272:5.5E-4,AinGeorg:5.5E-4)0.884.75:5.4E-
4,(AinOrico:0.00974,(Ult26277:0.01293,((AinHowel:0.00371,(Ult26282:5.4E-
4,(AinRadic:0.0037,(((AinSpec2:5.5E-4,((Ult26278:0.0,Ult26281:0.0):5.5E-
4,AinNaes2:5.5E-4)0.745.45:5.5E-
4)0.853.57:0.00369,((AinNaesl:0.0,AinVisco:0.0,Ult26279:0.0):5.5E-
4,(AinVisc2:0.00368,Ult26280:0.02666)0.957.55:5.5E-4)0.712.16:5.5E-
4)0.898.86:0.00362,(AinUroge:5.4E-4,(AinBowde:5.5E-
4,((AinSlac2:0.0,AinSlack:0.0):0.0037,AinBovis:0.0037)0.090.6:5.5E-
4)0.896.60:0.00361)0.812.37:0.00741)1.000.507:5.2E-
4)0.874.81:0.00371)0.801.55:0.00372)0.783.64:0.00372,AinCatul:5.3E-
4)0.885.62:0.00968)0.130.5:0.0037)0.788.55:0.00526)0.611.6:0.00371,(AinMa
ssi:0.00369,(AinGeren:0.0037,AinIsrae:0.00371)0.615.9:5.5E-
4)0.805.47:0.00378)0.901.74:5.4E-
4)0.704.11:0.00706)0.754.43:0.00387)0.793.55:0.00365,(AinHyova:0.00746,Ul
tAct39:5.5E-4)0.863.75:0.00369)0.782.67:5.4E-4)0.967.44:5.5E-
4,((AinFunke:0.00752,(AinTuric:5.5E-4,Ult26271:5.5E-
4)0.847.78:0.0037)0.326.5:5.4E-
4,AinVacci:0.03117)0.897.65:0.00746)0.467.10:0.00727,AinSuima:0.01124)0.9
02.77:5.3E-4,(((AinLingn:5.5E-
4,UltAct41:0.00369)0.798.52:0.00376,(AinOdont:5.5E-
4,(AinMeyer:0.00746,((AinOdon2:0.0,Ult26274:0.0):5.5E-
4,(UltAct40:0.00741,Ult26273:0.00369)0.732.19:5.5E-4)0.779.59:5.4E-
4)0.881.80:5.4E-
4)0.863.76:0.00721)0.877.86:0.00763,Ult26285:0.01517)0.781.51:0.00362)0.8
72.78:5.5E-
4)0.237.9:0.00357)0.935.64:0.01655)0.744.34:0.00274)0.745.46:0.00529,(Ain
Canis:0.00402,(AinHorde:0.01078,AinNasic:0.01586)0.946.63:0.01568)0.449.1
8:0.00985)0.904.74:0.01812,(UltAct43:0.03303,((UltAct42:5.4E-
4,(UltAct44:0.03151,UltAct45:0.01929)0.854.68:0.01925)0.829.55:0.00736,(U
ltAct46:0.00352,UltAct47:0.0154)0.962.69:0.01555)0.867.62:0.01072)0.801.5
6:0.00998)0.948.71:0.02384,(Ult26283:0.05333,(Ult26269:0.06139,(Try00032:
0.04447,((Tryy0106:0.01161,(Ult26243:0.00498,Ult26244:0.04382)0.908.81:0.
01458)0.827.50:0.01358,UltAct38:0.01815)0.944.56:0.02571)0.916.82:0.02268
)0.064.5:0.00811)0.325.8:0.00934)0.810.45:0.01235)0.960.64:0.02737)0.850.
75:0.00742,((((AroSpeci:0.00369,AroPhoca:5.5E-4)0.512.4:5.5E-
4,(((AroPyoge:5.5E-4,AroAbort:0.00368)1.000.508:5.5E-
4,(AroBialo:0.00638,AroBonas:0.01722)0.994.60:0.03439)0.997.65:0.03122,Ar
oPlura:0.00764)0.773.57:0.00352)0.853.58:0.00369,(UltArcan:5.5E-
4,(AroHippo:0.00369,AroHaemo:0.00372)0.689.13:5.4E-4)0.895.85:5.4E-
4)0.908.82:0.00753,(AhbSangu:0.03482,(KocKris2:0.0,KocKrist:0.0,KocKris3:
0.0):0.02269)0.681.8:5.5E-
4)0.869.64:0.00842)0.296.5:0.00838)0.857.74:0.01645,((((((Ult26390:5.5E-
4,(OntHorte:0.00743,Ult26389:5.4E-

4)0.853.59:0.00366)0.984.46:0.01907,((OrrHumi2:0.0,OrrHumip:0.0,OrrPekin:
0.0):0.0037,Ult26405:0.00745)0.731.19:5.4E-4)0.919.85:5.5E-
4,((KrbSpeci:0.0037,(((Ult26404:0.00713,(DmtTerra:0.00743,((DccAbyss:5.4E
-4,DccNishi:0.00748)0.781.52:0.00365,(UltDerma:5.4E-
4,DccSpeci:0.00745)1.000.509:5.3E-4)0.707.14:0.00748)0.947.51:5.3E-
4)0.441.11:0.00378,Ult26403:0.00775)0.854.69:0.00721,SeoMarin:0.00366)0.8
03.41:0.00377)0.852.68:5.5E-
4,(Ult26402:0.0037,SeoSpeci:0.00371)0.812.38:0.00368)0.939.63:0.01105)0.8
14.36:0.00781,(((Ult26401:0.01515,(((((KytSeden:0.00366,KytSpeci:0.00747)
0.847.79:0.00665,((DrtSpeci:0.00371,DrtChelo:0.00749)0.752.52:0.00456,(Jn
bCoral:5.5E-4,(((Ult26382:5.4E-
4,(((PhuBigeu:0.0,PhuAerop:0.0):0.00364,Ult26379:0.01165)0.897.66:0.00781
,Ult26381:0.01526)0.885.63:0.00765)0.011.6:5.4E-
4,OrmLepto:0.01128)0.898.87:0.00767,(((InpBacte:0.0037,TtsElong:5.5E-
4)0.833.60:0.00369,(((PhuSpeci:0.0,TtsSpeci:0.0,Ult26386:0.0,JnbLimos:0.0
,JnbAnoph:0.0,JnbSangu:0.0,Udntddd6:0.0,JnbMelon:0.0,JnbMelo2:0.0,JnbMelo
3:0.0,KnoSubte:0.0,KnoSinen:0.0):5.5E-4,((JnbTerra:0.0037,UltKnoel:5.5E-
4)0.246.9:5.5E-4,(Ult26380:0.00368,(TtsJapon:0.00743,TtsRemsi:5.3E-
4)0.395.7:0.00369)0.980.40:5.5E-4)0.424.11:5.5E-4)0.000.530:5.5E-
4,(JnbSang2:0.0037,Ult26041:0.0112)0.482.11:5.5E-4)0.232.7:5.5E-
4)0.480.6:5.4E-4,((KnoAerol:5.4E-
4,(((TtsAustr:0.00743,(TtsJenki:0.0113,TtsVeron:0.0037)0.904.75:5.5E-
4)0.463.8:0.00714,(TtsVanve:0.00719,Ult26388:0.0078)0.471.10:0.00747)0.29
1.7:5.5E-
4,(((Ult26387:0.0037,(LapJejue:0.0037,Ult26406:0.01123)0.885.64:5.1E-
4)0.919.86:5.4E-4,(((PhuJeju2:0.0,PhuJejue:0.0):5.4E-
4,Ult26383:0.00747)0.856.57:0.00408,(Ult26384:0.00429,Ult26385:0.01065)0.
437.10:0.00953)0.905.63:0.00875)0.921.84:0.00337,(TtsDuode:0.0071,(TeaLut
eu:0.00369,((Ult26376:0.00368,(TbrAerol:5.5E-
4,(TbrTumes:0.0,TbrSpeci:0.0):5.5E-4)0.864.72:5.5E-4)0.648.12:5.5E-
4,Ult26377:0.00369)0.917.73:0.00747)0.777.58:0.00407)0.757.43:0.00744)0.9
19.87:5.5E-4)0.880.76:5.3E-4)1.000.510:5.4E-
4,((UltIntra:0.0037,((Ult26091:0.0,InrCalvu:0.0,HmhOryz2:0.0,HmhOryza:0.0
,HmcXanth:0.0,Ult26374:0.0):5.5E-4,UltAct70:5.5E-4)0.912.89:5.5E-
4)0.843.53:0.00362,((Ult26043:5.4E-
4,KrbDiese:0.00742)0.918.71:0.00741,InrSpeci:5.5E-4)0.887.79:5.5E-
4)0.390.7:0.00367)0.841.57:0.0036)0.779.60:0.00381)0.792.62:0.00386)0.899
.73:0.01106)0.665.17:0.00793)0.756.56:0.0048,(DrtCongo:5.4E-
4,TonsSuis:0.01128)0.792.63:0.00371)0.779.61:0.00384,MbnAsiat:0.01135)0.8
93.78:0.00786,((BcbPheno:0.00747,(DrbSpeci:0.00689,((AruSpeci:0.00288,(Ar
uBolid:5.5E-4,BacNL427:5.5E-
4)0.947.52:0.02002)0.980.41:0.02437,((((BcbRhamn:0.0,BcbNeste:0.0,BcbZhon
g:0.0,Ult26236:0.0,Ult26237:0.0):5.5E-
4,(Ult26235:0.01948,((Ult26231:0.00741,Ult26232:0.01893)0.252.3:5.5E-
4,(BcbSpec2:0.0,BcbSpeci:0.0,BcbSpec3:0.0,BcbAlime:0.0,Ult26233:0.0,BcbSa
ce2:0.0,BcbSacel:0.0):5.5E-4)0.824.43:0.0037)0.682.11:5.5E-
4)0.253.8:5.5E-
4,Ult26234:0.00369)0.970.52:0.01581,(Ult26239:0.00372,(DrbHomin:0.00369,U
lt26238:5.5E-
4)0.890.78:0.00744)0.875.71:0.00757)0.759.61:0.00407)0.920.89:5.5E-
4)0.978.36:0.01959)0.771.64:0.00379,DvsAgama:5.4E-
4)0.978.37:0.01558)0.644.8:5.4E-
4)0.878.71:0.00822,KnsLimos:0.00746)0.835.55:0.00797,Ult26400:0.0154)0.91
3.73:0.01618)0.638.8:0.01265,Ult26286:0.02286)0.751.28:0.00411,(UltAct60:

0.02319,((AtaUrina:0.0112,(AtaMassi:5.4E-
4,((AtaScha2:0.0,AtaSchaa:0.0):5.5E-4,(AtaScha3:0.0,AtaScha4:0.0):5.5E-
4)0.837.47:0.01076)0.240.7:0.00724)0.983.36:0.0238,(AtaSpeci:0.01498,UltA
ct59:5.4E-
4)0.881.81:0.01525)0.846.79:0.00757)0.892.61:0.01186)0.700.25:0.01225)0.4
30.9:5.4E-
4)0.863.77:0.00419)0.927.70:0.0125)0.911.89:0.01376,Ult26462:0.03019)0.33
8.4:0.00402)0.883.81:0.00994)0.356.8:5.5E-4)0.299.9:0.00796)0.566.8:5.5E-
4,(StpVitam:0.01494,((((Udntded5:0.00742,(StpSpec5:0.00744,StpSpec7:0.015
07)0.043.6:5.5E-
4)0.769.55:0.00367,StpAvice:0.00375)0.764.57:0.0037,(StpTherm:0.01947,(St
pCarpa:0.0,StpSpec6:0.0):0.00367)0.789.50:0.00381)0.764.58:0.00374,(StpPh
aeo:5.5E-4,StpHaina:5.5E-
4)0.762.68:0.00364)0.870.51:0.00719)0.885.65:5.4E-
4)0.939.64:0.01705)0.892.62:0.0133)0.806.36:0.01808)0.949.62:0.0406)0.897
.67:0.03281,((Ult26597:0.00416,(NlrAlkal:0.01735,UltAct95:0.06782)0.780.6
8:0.02243)0.999.98:0.07551,((Ult13237:0.07043,(Ult26721:0.00369,Ult26722:
5.5E-4)0.979.47:5.4E-
4)0.991.53:0.04375,((((Ult26717:0.0557,(UltAc913:0.00532,(Ult26720:0.0118
8,(Ult26719:0.00369,UltAc912:5.5E-
4)0.775.62:0.00319)0.970.53:0.02636)0.947.53:0.02636)0.751.29:0.00795,((U
ltAc911:0.00473,(Ult26715:0.02448,UltAc914:0.01512)0.024.3:0.00297)0.979.
48:0.02646,((Ult26599:0.00851,(Ult26716:0.02639,Ult26714:0.03678)0.561.10
:0.01202)0.881.82:0.01896,Ult26713:0.01092)0.340.8:5.3E-
4)0.803.42:0.00851)0.941.65:0.02239,Ult26723:0.041)0.882.73:0.01577,(Ult2
6718:0.0236,Ult29498:0.02738)0.910.70:0.02006)0.884.76:0.01947)0.829.56:0
.01569)0.718.17:0.03021)0.329.10:0.01271)0.812.39:5.4E-
4)0.931.65:0.03183)0.919.88:0.02887,((Ult32314:0.0223,(Ult32312:0.00429,U
lt32313:0.10773)0.927.71:0.01997)0.998.78:0.07431,((((Ult15318:0.10632,(U
lt15319:0.03003,(UltAc382:0.01789,(Ult15317:0.02314,(Ult15316:0.01115,Ult
Ac381:5.5E-
4)0.744.35:0.00374)0.903.69:0.01238)0.034.5:0.0075)0.813.45:0.02654)0.966
.72:0.0838,(((((((Ult15373:0.02389,(Ult15387:0.03458,(Ult15374:0.03732,U
lt15388:0.03531)0.691.11:0.02531)0.875.72:0.01901)0.887.80:0.01458,Ult153
99:0.00542)0.896.61:0.00999,((Ult15111:0.00761,Ult15112:0.08045)0.754.44:
0.00355,((Ult15115:0.00734,((Ult15110:0.00365,(Ult15124:0.0,Ult15133:0.0,
Ult15135:0.0,Ult15372:0.0):5.5E-
4)0.915.71:0.00205,(Ult15114:0.01853,Ult15125:0.00368)0.916.83:0.00203)0.
912.90:0.00683)0.805.48:5.2E-4,(UltAc332:5.5E-
4,((Ult15113:0.00365,Ult15116:0.00364)0.678.11:5.5E-
4,(Ult14951:0.0,UltAc331:0.0,Ult15134:0.0):5.5E-4)0.000.531:5.5E-
4)0.864.73:5.5E-4)0.926.85:5.4E-4)0.852.69:0.00361)0.651.14:5.3E-
4,(Ult15010:0.0,Ult15121:0.0):5.5E-4)0.969.46:5.5E-
4,(((Ult15151:0.01106,((UltAc337:5.5E-
4,(Ult15079:0.00365,((((Ult15128:0.0,Ult15129:0.0,Ult15138:0.0):5.4E-
4,((((((((Ult15026:5.5E-4,UltAc394:0.0148)0.444.15:5.5E-
4,(Ult15014:0.0,Ult15030:0.0,Ult15389:0.0):5.5E-4)0.624.12:5.5E-
4,Ult15016:0.00366)0.000.532:5.5E-
4,Ult15032:0.00365)0.832.48:0.00398,UltrS215:5.3E-4)0.192.6:5.4E-
4,(((Ult15140:0.01182,(Ult15141:0.00365,(UltAc335:5.5E-4,UltAc336:5.5E-
4)0.616.8:5.5E-
4)0.785.59:0.00366)0.775.63:0.00368,((Ult15005:0.0,Ult15009:0.0,Ult15021:
0.0,Ult15077:0.0):5.5E-
4,(((Ult15391:0.00366,UltAc317:0.01127)0.783.65:0.00366,(Ult15012:0.0,Ult

15017:0.0,Ult15020:0.0):0.00365)0.904.76:5.5E-
4,(Ult15023:0.00364,Ult15025:0.00364)0.883.82:5.5E-4)0.000.533:5.5E-
4)0.915.72:5.5E-4)0.728.24:5.4E-4,(Ult14937:0.00735,Ult15142:5.3E-
4)0.941.66:0.00734)0.926.86:0.00735)0.771.65:0.00395,Ult15393:0.01112)0.8
82.74:0.00816,Ult15146:0.01118)0.870.52:0.00802)0.203.5:5.4E-
4,Ult15139:5.5E-4)0.828.46:0.00396,Ult15147:0.00734)0.388.8:5.4E-
4)0.067.2:5.4E-4)0.526.7:5.5E-4,Ult15143:0.01108)0.432.12:5.5E-
4)0.916.84:0.00805,((UcvtSoi3:5.5E-
4,(Ult15117:0.00366,Ult15118:0.00742)0.774.69:0.00362)0.802.48:5.5E-
4,(UltAc334:5.4E-
4,Ult15119:0.0111)0.841.58:0.00365)0.944.57:0.00739)0.877.87:5.3E-
4,(Ult15145:5.4E-
4,((((Ult15008:0.0,Ult15028:0.0,Ult15029:0.0):0.00357,((Ult15013:0.0,Ult1
5019:0.0):0.00361,UltAc315:5.4E-4)0.713.20:0.00728)1.000.511:5.4E-
4,Ult15015:5.3E-4)0.936.69:0.00732,((UltAc316:5.5E-
4,Ult15027:0.00365)0.878.72:5.5E-
4,(Ult15031:0.0074,(Ult15006:0.00361,((Ult15007:0.0,Ult15018:0.0,Ult15024
:0.0):5.5E-4,Ult15022:0.00368)1.000.512:5.3E-
4)0.755.45:0.00732)0.952.54:5.5E-4)0.844.72:5.4E-
4)0.940.71:0.00728)0.922.98:0.00725)0.586.12:0.00727)0.890.79:0.00721,(((
(((Ult14935:0.03273,UltAc330:5.5E-
4)0.909.62:0.00207,(Ult14949:0.01904,Ult14952:0.01891)0.903.70:0.01091)0.
924.71:0.002,(((UltrS212:0.06198,Ult14948:0.00269)0.998.79:0.00107,((((((
((Ult14988:0.0,Ult15001:0.0):5.5E-4,Ult15386:0.00369)0.990.56:5.5E-
4,(Ult14976:0.01093,((UltAc303:0.0,UltAc313:0.0):5.5E-
4,(Ult14981:0.03446,Ult15002:0.00738)0.821.40:5.5E-4)0.929.69:5.4E-
4)0.540.7:0.00741)0.817.32:0.00362,((((Ult14984:0.00742,Ult14978:0.00369)
0.928.61:5.5E-4,(Ult15137:0.00369,((Ult14979:5.5E-
4,UltAc333:0.01983)0.850.76:0.00364,Ult14983:0.05128)0.998.80:5.5E-
4)0.763.66:5.5E-4)0.000.534:5.5E-
4,(Ult14941:0.0,Ult14953:0.0,Ult14954:0.0,UltAc302:0.0,UltrS211:0.0,UltrS
213:0.0,Ult14995:0.0,UltAc305:0.0,Ult14996:0.0,Ult14997:0.0,UltAc306:0.0,
Ult14998:0.0,Ult15040:0.0,Ult15385:0.0):5.5E-4)0.759.62:5.4E-
4,(Ult16422:0.01917,(UltrS214:5.5E-4,UltAc312:5.5E-4)0.317.10:5.5E-
4)0.836.43:0.00366)0.261.3:5.4E-4)0.288.8:5.4E-4,((((((UltAc304:5.5E-
4,Ult14994:5.5E-4)0.922.99:5.5E-
4,((Ult15054:0.00376,Ult15687:0.00749)0.950.57:0.01134,(Ult15052:5.4E-
4,(Ult14957:0.00747,Ult14977:5.5E-
4)0.914.81:0.00751)0.884.77:0.00743)0.920.90:5.5E-
4)0.832.49:0.0037,Ult14946:5.3E-4)0.890.80:0.00356,((Ult15000:5.5E-
4,(Ult15041:0.00735,(Ult15045:0.00365,Ult15120:0.01899)0.825.46:5.5E-
4)0.917.74:5.5E-4)0.902.78:5.5E-
4,((Ult14944:0.0,Ult14964:0.0,Ult15131:0.0):5.5E-
4,(UltrS217:0.00759,Ult15053:0.03451)0.779.62:0.00371)0.863.78:5.4E-
4)1.000.513:5.4E-
4)0.320.3:0.00356,((((Ult15063:0.00371,(Ult15059:0.0112,(Ult15062:0.0,Ult
15123:0.0):5.5E-4)0.865.69:0.00371)0.797.44:5.4E-
4,((((Ult15060:0.00367,Ult15004:0.00368)0.908.83:5.5E-
4,(Ult15003:0.00367,Ult14968:0.00367)0.884.78:5.5E-4)0.000.535:5.5E-
4,(Ult15398:0.00368,Ult14934:0.00367)0.713.21:5.5E-4)0.000.536:5.5E-
4,(Ult14939:0.0,Ult14947:0.0,Ult14966:0.0,Ult14972:0.0,Ult15039:0.0,Ult15
046:0.0,Ult15047:0.0,Ult15122:0.0,Ult15127:0.0):5.5E-4)0.908.84:5.5E-
4)0.892.63:0.00206,Ult14999:0.00701)0.888.82:0.00204,(Ult14975:0.00374,Ul
t15048:5.5E-4)0.772.58:5.5E-4)0.991.54:5.4E-

4)0.866.66:0.00347,((Ult14989:0.0,Ult15049:0.0):5.5E-
4,(Ult14982:0.00369,Ult15150:0.00369)0.883.83:5.5E-4)0.961.54:5.4E-
4)0.832.50:0.00355)0.868.59:5.5E-
4,(Ult14971:0.00897,Ult14980:0.09785)0.942.67:0.02153)0.861.68:0.00684,((
(((Ult14961:0.0,Ult15050:0.0):5.5E-4,((UltAc307:0.0,UltAc321:0.0):5.5E-
4,(Ult15011:5.5E-4,((UltAc311:0.00739,((Ult15068:5.5E-4,(Ult14986:5.5E-
4,(UltAc308:5.5E-4,Ult14985:0.01508)0.000.537:5.5E-4)0.000.538:5.5E-
4)0.961.55:0.0112,((Ult15132:0.0,Ult15149:0.0):5.5E-
4,Ult14987:0.01668)0.393.7:5.4E-4)0.917.75:0.00827)0.356.9:5.4E-
4,(UltAc309:0.0,UltAc310:0.0):0.00369)0.508.5:5.5E-4)0.081.7:5.5E-
4)0.847.80:0.00407)0.867.63:0.00407,UltAc322:0.00777)0.514.6:5.5E-
4,(Ult14940:0.0,Ult15055:0.0):5.5E-4)0.206.5:5.4E-
4,((((Ult14992:0.00733,(Ult14993:0.01114,(UltAc318:5.4E-
4,((Ult14936:0.0,Ult14938:0.0,Ult14956:0.0,Ult14962:0.0,Ult15407:0.0):5.5
E-4,Ult15037:0.01851)0.953.49:0.01105)0.704.12:0.0073)0.821.41:5.5E-
4)0.753.52:0.0036,(Ult15095:0.01105,((Ult14963:0.00367,Ult15065:5.5E-
4)0.919.89:5.5E-4,((Ult15105:0.01867,Ult15107:0.03727)0.852.70:5.3E-
4,((((Ult14969:0.00738,Ult14960:0.00368)0.615.10:5.5E-
4,Ult15044:0.00367)0.726.19:5.5E-
4,(Ult14942:0.0,Ult15103:0.0,Ult15104:0.0,Ult15106:0.0,UltAc329:0.0,Ult15
108:0.0,Ult15392:0.0):5.5E-4)0.000.539:5.5E-4,Ult15102:5.5E-
4)0.919.90:5.5E-4)0.898.88:0.00359)0.909.63:5.3E-
4)0.892.64:0.00694)0.858.68:5.4E-4,(((Ult15056:0.00366,(UltAc320:5.1E-
4,Ult15057:0.00368)0.121.4:5.4E-
4)0.914.82:0.00738,(UltrS218:0.0,Ult15069:0.0):0.00736)0.918.72:5.3E-
4,(Ult14990:0.00368,(UltAc314:0.00367,((Ult14991:0.0,UltAc319:0.0):5.5E-
4,UltAc338:5.5E-4)0.724.19:5.5E-4)0.295.14:5.3E-
4)0.816.46:0.00363)0.760.52:0.00732)0.896.62:0.00701,Ult14950:0.00372)0.7
78.61:0.00349)0.881.83:0.00404)0.051.3:5.4E-
4,((((Ult15064:0.00367,(((((((Ult15042:0.00867,Ult15067:0.01505)0.753.53:
0.00419,Ult14943:0.00369)0.435.9:5.3E-
4,(Ult14945:0.0,Ult14955:0.0,Ult15038:0.0,Ult15043:0.0,Ult15051:0.0,Ult15
130:0.0,Ult15136:0.0):5.5E-4)0.484.5:5.5E-4,(Ult14959:5.5E-
4,Ult15070:0.03415)0.914.83:0.00738)0.993.49:5.3E-
4,(Ult15383:0.00381,Ult15371:0.01871)0.488.10:0.00741)0.907.70:0.00408,Ul
t14967:0.00369)0.429.6:5.5E-4,Ult15066:5.5E-4)0.484.6:5.5E-
4)0.818.34:0.00416,Ult14973:0.00367)0.134.9:5.5E-4,Ult14965:5.5E-
4)0.519.7:5.4E-4,(Ult15058:0.02237,((Ult14958:0.0,Ult15061:0.0):5.5E-
4,Ult14974:0.00737)0.991.55:5.5E-4)0.840.63:0.00361)0.373.8:5.5E-
4)0.960.65:0.00807)0.059.7:5.5E-4,(UltrS216:0.00366,(Ult15148:5.5E-
4,(Ult15126:0.00366,UltPro73:5.5E-
4)0.840.64:0.00365)0.797.45:0.00366)0.809.47:0.00365)0.959.65:0.0122)0.34
1.11:5.4E-4,(((Ult15152:0.0,Ult15154:0.0):5.5E-
4,Ult15156:0.0074)0.962.70:5.5E-
4,Ult15153:0.02248)0.831.41:0.00358)0.833.61:5.5E-4,((Ult14970:5.5E-
4,Ult15155:0.01849)0.810.46:0.00358,(Ult15035:5.4E-
4,((Ult15033:0.00733,Ult15109:5.5E-
4)0.848.62:0.00215,(Ult15034:0.01476,(Ult15384:0.02643,Ult15036:0.00566)0
.613.12:5.4E-
4)0.832.51:0.00441)0.834.45:0.00209)0.586.13:0.0073)0.983.37:5.4E-
4)0.922.100:5.4E-4,(((Ult15161:0.0,Ult15162:0.0):5.5E-
4,AdrBact5:0.0073)1.000.514:0.06647,(((((Ult15090:0.02316,((UltAc323:0.02
377,UltOr249:0.1116)0.556.7:0.03522,((UltrS221:0.00491,((((UltAc339:0.050
91,(Ult15164:0.01124,(Ult15163:0.01505,UltAc340:0.00742)0.028.4:5.4E-

4)0.762.69:0.00489)0.865.70:0.00944,(UltMa104:0.00809,Ult15165:0.02758)0.935.65:0.01874)0.780.69:5.3E-4,(Ult15166:0.00651,UltAc341:0.05408)0.855.68:0.00869)0.879.71:0.00764,(((Ult15254:0.00369,(Ult15255:0.0,Ult15256:0.0):5.5E-4)0.949.63:0.01129,((((((Ult15205:0.0,Ult15208:0.0,Ult15212:0.0,Ult15244:0.0):5.4E-4,(((((Ult15241:0.0,Ult15251:0.0):5.5E-4,Ult15242:0.00369)0.919.91:5.5E-4,Ult15377:0.01886)0.791.50:0.00373,Ult15211:0.00374)0.798.53:0.00374,((UltAc353:0.00748,Ult15216:5.4E-4)0.927.72:0.01134,(Ult15243:5.5E-4,Ult15246:0.00369)0.758.61:0.00378)0.902.79:0.00747)0.264.3:5.4E-4)0.925.88:0.00189,(((Ult15198:0.01508,UltAc352:5.4E-4)0.427.8:0.01458,(Ult15196:0.01923,(Ult15197:0.00558,UltSlu31:0.01714)0.813.46:0.01445)0.300.9:0.01072)0.689.14:0.00368,((UltrS226:0.00745,Ult15247:5.4E-4)0.968.57:0.02411,Ult15245:0.00894)0.904.77:0.01209)0.846.80:0.00715)0.924.72:0.00234,(((Ult15210:0.01218,Ult15209:0.00425)0.930.62:0.01485,((Ult15200:0.00723,((Ult15226:0.02782,Ult15227:5.5E-4)0.931.66:0.01514,(((((UltAc349:0.02644,(((UltAc366:0.0,Ult15252:0.0,Ult15266:0.0):0.00373,Ult15249:0.0075)0.775.64:0.00401,(((Ult15220:0.01503,((Ult15221:0.0,Ult15222:0.0):5.3E-4,UltAc356:0.00342)0.927.73:0.01533)0.849.58:0.0076,(((Ult15376:0.00367,(Ult15268:0.00368,(UltPiet2:0.00369,(Ult15269:0.00369,Ult15267:5.5E-4)0.283.4:5.5E-4)0.779.63:0.00372)0.986.46:0.02297)0.596.5:5.5E-4,Ult15382:5.5E-4)0.819.44:0.00367,(Ult15234:5.5E-4,((((((Ult15238:0.0,UltOr255:0.0,UltAc369:0.0):5.5E-4,UltAc368:0.00369)0.960.66:0.01115,(UltAc364:5.5E-4,UltAc365:0.00369)0.880.77:0.00369)0.449.19:5.3E-4,Ult15240:5.5E-4)0.907.71:0.00369,(UltAc372:0.00369,Ult15265:0.01501)0.741.38:5.5E-4)0.873.77:5.5E-4,(Ult15235:0.0,UltAc363:0.0,Ult15237:0.0,UltAc367:0.0,Ult15236:0.0,Ult15239:0.0):5.5E-4)0.997.66:5.5E-4)0.998.81:5.5E-4)0.694.9:0.00367)0.093.6:5.3E-4,((Ult15199:0.01102,UltrS225:0.01499)0.863.79:5.4E-4,(Ult15207:0.0111,(UltAc348:5.4E-4,(UltAc354:0.00742,(UltrS231:0.00369,(UltAc355:0.0,Ult15230:0.0,UltMa105:0.0):5.5E-4)0.373.9:5.5E-4)0.862.65:0.00369)0.900.63:0.00727)0.888.83:5.5E-4)0.859.60:0.00369)0.859.61:0.00712)0.152.8:0.00367)0.796.43:0.00395,(((Ult15231:0.00369,((Ult15206:0.0,UltAc362:0.0):5.5E-4,Ult15232:0.00741)0.815.52:0.00369)0.861.69:5.5E-4,(UltAc359:0.00369,Ult15233:0.00369)0.662.9:5.5E-4)0.000.540:5.5E-4,(UltrS232:0.0,UltAc360:0.0,UltAc361:0.0):5.5E-4)0.953.50:5.4E-4)0.795.49:0.0037,(Ult15250:0.00265,(Ult15248:5.5E-4,UltDe159:5.5E-4)0.997.67:0.04696)0.968.58:0.02508)0.846.81:5.5E-4,Ult15264:0.01902)0.974.36:0.01879,(Ult15228:0.00749,Ult15229:0.0114)0.744.36:0.00416)0.271.4:0.00378)0.106.6:0.00524)0.759.63:0.01094,(Ult15223:5.5E-4,UltAc357:5.5E-4)0.934.66:0.0145)0.832.52:0.00844)0.882.75:0.00779,(Ult15217:0.00756,Ult15218:0.00752)0.872.79:0.00761)0.785.60:0.00368)0.728.25:0.00382,(UltAc373:0.00431,((UltrS223:0.01936,(((((Ult15192:0.00745,Ult15193:5.5E-4)0.967.45:0.01521,(Ult15172:0.01127,((Ult15170:5.5E-4,UltAc343:0.00369)0.952.55:0.01124,((UltOr250:0.0,UltOr253:0.0):5.5E-4,(Ult15171:5.5E-4,Ult15179:0.01116)0.800.43:5.5E-4)0.950.58:5.5E-4)0.980.42:5.5E-4)0.448.8:0.00753)1.000.515:5.5E-

4,((Ult15174:0.0079,(Ult15168:5.4E-
4,(UltrS222:0.00743,(Ult15167:0.00744,(((Ult15177:0.00395,Ult15178:0.0269
)0.901.75:0.01154,UltOr252:0.0073)0.767.55:0.00384,UltOr251:0.00381)0.966
.73:0.01528)0.124.5:5.4E-
4)0.798.54:0.00369)0.955.49:0.01886)0.896.63:0.01148,(Ult15180:0.00196,(U
lt15181:0.02269,Ult15182:5.3E-
4)0.910.71:0.0131)0.999.99:0.05048)0.910.72:0.01146)0.800.44:0.00342,(Ult
Ac342:5.4E-4,((UltOr247:5.5E-
4,(UltOr248:0.00743,((UltAc344:0.0,Ult15176:0.0):5.5E-
4,Ult15175:0.00369)0.261.4:5.5E-4)0.917.76:0.00743)0.959.66:5.4E-
4,Ult15184:0.01516)0.456.7:0.00746)0.899.74:0.00751)0.777.59:0.00363,(Ult
15169:0.04016,((Ult15185:0.00703,(Ult15187:0.0,UltAc346:0.0):0.01207)0.74
3.46:0.01558,((Ult15188:0.01504,(Ult15183:5.4E-
4,UltAc345:0.00715)0.788.56:0.01509)0.953.51:0.01579,(((((Ult15195:0.0041
1,((Ult15259:5.4E-4,(UltrS233:0.015,(Ult15258:5.5E-
4,(Ult15262:0.00367,Ult15261:0.00368)0.683.17:5.5E-4)0.935.66:5.4E-
4)0.916.85:0.00749)0.875.73:5.3E-
4,Ult15260:0.00708)0.964.64:0.01902)0.784.52:0.00327,(UltAc347:0.0,UltrS2
30:0.0):5.5E-4)0.615.11:5.4E-
4,(((Ult15253:0.00812,(UltAc371:0.00382,UltAc370:0.00363)0.923.99:0.01471
)0.972.55:0.01933,AdrBact6:0.01461)0.740.44:0.00409,((Ult15213:0.01917,(U
dnBac62:0.0037,Ult15219:5.5E-
4)0.880.78:0.00748)0.916.86:0.01128,((Ult15194:5.5E-
4,(Ult15191:0.01907,((((Ult15186:0.00369,UltOr254:5.5E-
4)0.831.42:0.00366,(UltrS224:0.00369,Ult15189:0.00742)1.000.516:5.4E-
4)0.794.49:0.00746,Ult15201:0.00745)0.940.72:5.4E-4,Ult15173:5.5E-
4)0.754.45:0.00371)0.874.82:0.00748)0.916.87:0.00743,((((UltrS227:0.00372
,UltrS228:0.00749)0.762.70:0.00371,((Ult15214:5.4E-
4,((Ult15375:0.03395,Ult15204:5.5E-4)0.888.84:0.00784,Ult15224:5.5E-
4)0.866.67:5.4E-4)0.886.93:0.00327,(UltrS229:5.3E-
4,Ult15263:0.01888)0.911.90:0.00107)0.956.59:0.01276)0.444.16:5.2E-
4,UltAc351:5.5E-4)0.197.7:5.4E-
4,Ult15215:0.0074)0.905.64:0.00861)0.799.56:5.5E-4)0.764.59:5.4E-
4)0.999.100:0.0233)0.784.53:0.00342,UltAc350:0.01928)0.874.83:0.00766,Ult
15190:0.00369)0.784.54:5.5E-4)0.930.63:0.01838)0.934.67:5.4E-
4)0.897.68:0.00765)0.879.72:5.4E-
4)0.950.59:0.01885,(Ult15257:0.01486,(UltAc374:0.0,UltAc375:0.0):5.5E-
4)0.982.44:0.02373)0.417.11:0.0068)0.258.7:0.00383)0.956.60:0.01825,(Ult1
5202:0.01493,Ult15225:5.4E-4)0.951.64:0.01463)0.870.53:5.4E-
4)0.915.73:0.01118,UltAc358:0.00381)0.935.67:0.01475)0.762.71:0.00999)0.9
46.64:0.02623,((Ult15272:0.02712,((UltAc376:0.03748,((Ult15271:0.00474,(U
ltOr256:0.01136,Ult15270:0.00374)0.050.6:0.01068)0.995.67:0.05183,((Ult15
274:5.4E-4,(Ult15278:0.00973,(Ult15276:5.5E-
4,Ult15277:0.03184)0.598.7:0.00988)0.970.54:0.02874)0.947.54:0.02012,Ult1
5275:0.00743)0.743.47:0.00635)0.655.18:0.02047)0.684.8:0.00625,(Ult15281:
0.00772,(Ult15280:5.4E-
4,Ult15279:0.02681)0.525.8:0.02401)0.862.66:0.01526)0.915.74:5.4E-
4)0.854.70:0.0198,Ult15273:0.0209)0.964.65:0.03415)0.837.48:0.01336)0.672
.10:0.00961)0.723.21:0.00347,(((Ult15097:0.00375,(UltrS220:0.00717,(Ult15
098:5.4E-
4,Ult15092:0.01074)0.275.8:0.0037)0.906.87:0.01146)0.821.42:0.00667,(UltA
c328:5.4E-4,(Ult15091:0.01112,Ult15099:5.5E-
4)0.907.72:0.00738)0.905.65:0.01072)0.786.58:0.00417,(Ult15093:0.01142,((
((Ult15082:0.00369,(((Ult15083:0.00741,(UltAc326:0.00745,((UltAc327:0.011

34,Ult15087:0.00371)0.629.13:5.5E-
4,(Ult15085:0.00372,(Ult15088:0.0075,(Ult15084:0.00371,Ult15086:5.5E-
4)0.829.57:5.0E-4)0.935.68:0.00757)0.788.57:5.4E-4)0.953.52:5.4E-
4)0.795.50:0.00749)0.784.55:5.3E-
4,Ult15089:0.00369)0.754.46:0.00367,UltrS219:0.0112)0.580.7:5.4E-
4)0.889.68:5.4E-4,(Ult15081:5.4E-
4,(Ult15076:0.0151,(Ult15075:0.00375,Ult15080:0.02316)0.753.54:0.00369)0.
772.59:0.00368)0.905.66:0.00744)0.684.9:0.00261,(UltAc324:0.06793,Ult1507
4:0.01861)0.864.74:0.01832)0.353.5:0.00496,Ult15100:0.01903)0.938.47:0.01
232)0.802.49:0.00632)0.795.51:0.00705)0.869.65:5.4E-
4,((Ult15096:0.03042,(Ult15094:5.4E-
4,Ult15144:0.03137)0.791.51:0.015)0.903.71:5.4E-
4,(UltOr246:0.0037,(Ult15073:0.03183,Ult15101:0.00366)0.764.60:0.00374)0.
899.75:0.00745)0.914.84:0.00742)0.915.75:5.4E-
4,(UltOr245:0.02381,(Ult15071:5.5E-4,Ult15072:5.5E-
4)0.996.55:0.04128)0.955.50:0.02669)0.948.72:0.01754,((Ult15158:5.5E-
4,Ult15159:5.5E-4)0.995.68:5.3E-4,((Ult15160:0.0,Ult15381:0.0):5.5E-
4,Ult15397:0.00371)0.999.101:0.03102)0.970.55:0.01826)0.637.7:5.5E-
4)0.922.101:0.01644)0.810.47:0.00644)0.715.14:0.00327,(((Ult16431:0.0262,
UltAc325:0.06908)0.343.3:0.00926,(Ult14932:0.01963,Ult16424:0.04876)0.796
.44:0.01232)0.668.13:0.0146,(Ult14933:0.03608,(Ult15203:0.03814,Ult15378:
0.09844)0.955.51:0.04704)0.861.70:0.02188)0.924.73:0.02888)0.998.82:0.081
07,Ult15320:0.03742)0.744.37:0.00929)0.943.64:0.03752,((Ult15334:0.01503,
(Ult15351:0.0,Ult15352:0.0):0.01107)0.847.81:5.4E-
4,(((Ult15405:0.05242,(((Ult15335:0.0,UltPro72:0.0):5.5E-
4,(UltPro71:0.00369,Ult15336:0.01505)1.000.517:5.5E-4)0.992.59:5.4E-
4,((((Ult15340:0.01194,Ult15410:0.01852)0.835.56:0.00795,(UltAc383:0.0130
7,((Ult15323:5.4E-
4,(Ult15325:0.02562,(((((Ult15327:0.0,Ult15328:0.0,Ult15349:0.0):5.5E-
4,UcvtSoi4:0.00732)0.917.77:0.00734,(Ult15326:0.00374,Ult15350:5.5E-
4)0.891.60:5.5E-4)0.889.69:5.4E-
4,Ult15409:0.02716)0.886.94:0.01399,Ult15324:0.00785)0.890.81:0.01373)0.2
73.7:5.5E-
4)0.912.91:0.01279,Ult15322:0.01787)0.714.19:0.00775)0.906.88:0.01284)0.8
67.64:0.0084,(Ult15321:0.00732,(UltAc387:0.0125,(Ult15337:0.01395,(Ult153
38:0.01127,Ult15339:0.00372)0.771.66:0.00481)0.009.4:0.00372)0.895.86:0.0
1049)0.749.61:0.00375)0.780.70:0.00355,Ult15332:0.03557)0.878.73:0.00735)
0.832.53:0.00361)0.742.34:5.4E-4,UltAc386:5.3E-
4)0.839.62:0.00367,((UltAc385:5.4E-
4,((Ult15331:0.01907,(Ult15329:0.0037,UltAc384:0.01919)0.345.7:0.00406)0.
784.56:0.00368,Ult15333:0.0113)0.968.59:5.4E-4)0.964.66:5.4E-
4,(Ult15330:5.4E-
4,Ult15348:0.01129)0.870.54:0.00744)0.910.73:0.00745)0.111.3:5.4E-
4)0.995.69:0.06305)0.322.9:0.02115,(((Ult15361:0.01945,((Ult15360:0.0,Ult
15406:0.0):0.01726,((UltAc389:0.02341,Ult15359:0.01523)0.730.25:0.00368,(
UltAc390:0.01924,((UltrS237:0.00351,(UltAc391:5.5E-
4,UltAc392:0.00744)0.990.57:0.02338)0.967.46:0.01536,(Udntfde8:0.03428,(U
lt15358:0.00371,UltAc388:0.0037)0.945.64:5.5E-
4)0.961.56:0.01611)0.857.75:5.5E-
4)0.892.65:0.00755)0.735.29:0.00564)0.516.3:0.0073)0.904.78:0.01886,Ult15
362:5.5E-
4)0.967.47:0.03453,((Ult15356:0.05493,(Ult15355:0.03232,(Ult15353:0.01081
,Ult15354:0.0122)0.696.13:0.00352)0.359.5:0.00375)0.912.92:0.03718,(AdbBa
c12:0.0514,Ult15357:0.02295)0.824.44:0.0231)0.780.71:0.01611)0.694.10:0.0

1461)0.792.64:0.03751)0.350.6:0.0222)0.811.46:0.01068,(((Ult14341:0.05117
,(Ult14342:0.04695,UltFor54:0.03168)0.814.37:0.01071)0.957.56:0.03621,((U
lt16218:0.07908,((Ult16219:0.02755,Ult16220:0.01668)0.969.47:0.03643,(Ult
16217:0.02437,Ult16221:0.10619)0.104.7:0.01369)0.876.66:0.0262)0.229.6:0.
00759,(Ult11065:0.1565,(Ult15284:0.03411,(((Ult15294:0.01514,Ult15298:0.0
0765)0.883.84:0.00738,((UltrS234:0.02366,Ult15290:0.01129)0.874.84:0.0077
7,(Ult15291:0.01507,(Ult15296:5.4E-
4,(UltrS236:0.01923,((Ult15295:0.00369,Ult15297:0.0037)0.872.80:5.5E-
4,(Ult15289:5.3E-4,(((Ult15307:5.5E-
4,Ult15408:0.00369)0.995.70:0.0464,((Ult15310:0.0,Ult15315:0.0):0.00617,(
((Ult15303:0.02389,Ult15292:0.03257)0.846.82:0.0106,Ult15311:0.01546)0.72
1.10:0.00376,((((Ult15313:5.4E-
4,Ult15314:0.01856)0.994.61:0.03048,((Ult15304:0.02997,(Ult15306:0.02686,
Ult15305:5.5E-
4)0.904.79:0.01649)0.782.68:0.01605,Ult15302:0.01511)0.903.72:5.5E-
4)0.797.46:0.00357,(UltAc378:5.4E-
4,Ult15312:0.01538)0.822.38:0.00367)0.875.74:5.4E-
4,(UltAc379:0.00366,Ult15308:0.01915)0.893.79:0.01092)0.383.7:0.00502)0.8
12.40:0.00776)0.560.6:0.00999)0.944.58:0.01879,UltrS235:0.0227)0.534.6:5.
4E-
4)0.995.71:0.02215)0.385.11:0.00963)0.772.60:0.00918)0.417.12:0.00716)0.8
74.85:0.00734)0.753.55:0.00358)0.766.51:0.00361,((Ult15309:0.01097,(Ult15
293:0.00761,UltAc380:0.0115)0.760.53:0.00385)0.829.58:0.00764,(((Ult15287
:0.04334,((UltAc377:5.5E-4,(Ult15285:5.4E-
4,Ult15286:0.01501)0.912.93:0.00744)0.827.51:0.00369,Ult15299:5.4E-
4)0.994.62:5.4E-
4)0.997.68:0.04733,Ult15300:0.00449)0.713.22:0.0035,(Ult15288:0.00758,Ult
15301:0.01951)0.888.85:0.01093)0.802.50:0.00686)0.780.72:0.00499)0.979.49
:0.05297)0.980.43:0.08591)0.191.6:0.0284)0.809.48:0.01545)0.947.55:0.0208
9,((UdnBac86:0.06661,(Ult31619:0.07332,(Ult31618:0.02644,((((Ult31611:0.0
16,(UltCa412:0.0238,Ult31610:0.01122)0.962.71:0.02367)0.866.68:0.01175,(U
lt31612:0.04575,UltOr486:0.04609)0.799.57:0.01056)0.895.87:0.01216,((((Ul
t31604:5.5E-
4,((Ult27479:0.0,Ult31603:0.0):0.00789,(Ult31605:0.0,Ult31606:0.0,Ult3160
7:0.0):0.02214)0.574.7:0.00846)0.789.51:0.01135,Ult31608:0.00751)0.745.47
:0.00335,(Ult31609:0.04934,UltCa411:0.01912)0.650.7:0.00357)0.867.65:0.01
063,Ult31593:0.0485)0.774.70:0.0052)0.861.71:0.01517,(((Ult31615:0.02834,
(UltAc947:0.03213,((UltOr488:0.01188,(UltOr489:0.00812,Ult31617:0.01469)0
.390.8:0.0036)0.991.56:0.04434,Ult31616:0.01241)0.699.20:0.01595)0.747.41
:0.00757)0.979.50:0.04417,(UltOr487:0.09786,(Ult31613:0.00757,Ult31614:0.
03683)0.987.67:0.07008)0.157.8:0.028)0.752.53:0.01449,((Ult31591:5.5E-
4,Ult31592:0.00368)0.987.68:0.03242,((Ult31596:5.4E-
4,((Ult31597:0.0,Ult31598:0.0,Ult31600:0.0,Ult31601:0.0,Ult31602:0.0):0.0
3133,Ult31599:0.05954)0.569.10:0.01721)0.921.85:0.01614,(Ult31594:0.0048,
Ult31595:0.01403)0.224.5:0.00946)0.115.2:0.0112)0.976.39:0.03489)0.728.26
:0.00298)0.985.52:0.05567)0.960.67:0.04329)0.877.88:0.02842)0.805.49:0.01
472,(CddDiv11:0.132,(UltCa414:0.07234,(UltAc948:0.04642,(UltCa413:0.01199
,(Ult31620:0.0,Ult31621:0.0,Ult31622:0.0):0.02482)0.879.73:0.03065)0.963.
59:0.05088)0.973.40:0.05063)0.386.8:5.4E-
4)0.950.60:0.0317)0.324.5:0.01188)0.761.49:0.00365)0.979.51:0.02158,((((U
ltVe263:0.05786,((((((Ult12502:0.04601,Ult12503:0.01656)0.831.43:0.02071
,(((Ult12508:0.03333,((UltRu290:0.0,UltRu291:0.0):0.01577,Ult12501:0.0334
)0.820.32:0.01086)0.331.7:0.00911,((Ult12506:0.00711,Ult12507:0.02424)0.9
67.48:0.02805,(Ult12505:0.03057,Ult12504:0.03841)0.919.92:0.02409)0.925.8

9:0.02612)0.861.72:0.02403,(UltRu292:0.00715,Ult12509:5.5E-
4)1.000.518:0.07021)0.829.59:0.01193)0.809.49:0.01582,Ult12523:0.07234)0.
915.76:0.02685,(Ult12522:0.09291,(UltLen22:0.09399,UltLen23:0.03395)0.372
.6:0.02053)0.937.64:0.02709)0.799.58:0.00997,(((UltVe260:0.02931,(UltVe26
2:0.03446,(CytSpe13:0.01828,UltVe261:0.02938)0.849.59:0.02861)0.669.9:0.0
1629)0.759.64:0.01109,((UdnBac49:0.01461,(Ult12500:0.00599,(Ult12499:0.01
133,UltCyt14:0.01144)0.345.8:0.01381)0.998.83:0.05704)0.731.20:0.00881,Ul
t12498:0.09996)0.743.48:0.01347)0.914.85:0.0179,((Ult12497:0.0442,(Ult124
96:0.01478,(UltLen15:0.00368,(UltLen14:0.00746,CytSpe12:0.00743)0.964.67:
5.4E-
4)0.865.71:0.01607)0.507.6:0.02444)0.950.61:0.04067,((UltLen16:0.0,UltLen
17:0.0):0.03508,(((Ult12517:0.05341,Ult12521:0.03741)0.590.6:0.01024,(Ult
25029:0.02467,(((Ult12514:0.0,Ult12513:0.0,VicBacte:0.0):5.3E-
4,(Ult12515:0.00725,(Ult12516:0.01505,UltEub22:5.4E-
4)0.960.68:0.01558)0.248.7:0.0037)0.891.61:0.01311,(VctVaden:0.00577,(Ult
12520:0.04026,(Ult12518:5.3E-
4,Ult12519:0.03922)0.874.86:0.02521)0.975.48:0.03944)0.670.10:0.02014)0.9
12.94:0.01651)0.886.95:0.01687)1.000.519:0.06814,(Ult12510:0.01815,(UltLe
n18:0.01646,(Ult12511:5.5E-4,Ult12512:5.5E-
4)0.753.56:0.00266)0.806.37:0.00901)0.894.65:0.01978)0.721.11:0.00366)0.9
89.55:0.03866)0.859.62:0.01674)0.983.38:0.03456)0.549.10:0.00644,(UltOr17
6:0.06838,(UltLen24:0.08136,(Ult12493:0.03074,(UltLen13:0.01191,Ult12492:
0.02981)0.518.10:0.01788)0.822.39:0.0124)0.930.64:0.0277)0.796.45:0.01357
)0.937.65:0.02669,((((UltOr175:0.14115,(((UltLen26:0.01254,(Ult12527:0.04
042,UltOr178:0.00505)0.880.79:0.02243)0.947.56:0.04966,((LenArane:5.4E-
4,UltOr177:0.01132)0.881.84:0.01459,(Ult12526:0.02348,(UltLen25:0.0046,Ul
t12525:0.06798)0.806.38:0.01133)0.791.52:0.00818)0.943.65:0.04428)1.000.5
20:0.14637,Ult12491:0.02889)0.562.10:0.02217)0.842.74:0.02648,((Ult12495:
0.01773,UltVe259:0.0514)0.993.50:0.06192,(UltLen21:0.01328,(UltLen19:0.00
365,UltLen20:0.00376)0.949.64:0.02138)0.967.49:0.03453)0.606.9:0.01036)0.
704.13:0.0224,(Ult12494:0.0192,UltVe258:0.02908)0.999.102:0.08088)0.800.4
5:0.01452,((Ult12524:0.05978,(Ult12488:0.03273,Ult12489:0.01938)0.997.69:
0.09573)0.937.66:0.04886,((((((UltLent6:0.00802,((Ult12464:0.05223,(((Ul
tLent8:5.5E-
4,UltLent9:0.00366)1.000.521:0.06693,(Ult12470:0.04262,((Ult12472:5.5E-
4,(Ult12399:0.09308,Ult12468:0.0024)1.000.522:0.00132)0.915.77:0.00352,(U
lt12471:5.5E-4,Ult12469:0.0037)1.000.523:5.4E-
4)0.901.76:0.01518)0.201.4:5.4E-4)0.905.67:0.00889,((UltLen10:5.5E-
4,UltLen11:0.01118)0.872.81:0.01632,(UltLent7:5.5E-
4,Ult12474:0.00367)0.996.56:0.05768)0.887.81:0.01475)0.705.16:0.00391)0.9
14.86:0.0236,Ult12473:0.01377)0.785.61:0.01392)0.000.541:5.5E-
4,((Ult12467:0.09423,(Ult12465:5.5E-
4,Ult12466:0.0112)0.759.65:0.00482)0.791.53:0.01134,(((((UltLent5:0.01355
,(Ult12457:0.0,Ult12458:0.0):0.00907)0.899.76:0.01975,(UltLenti:0.02382,(
(Ult12454:0.0,Ult12455:0.0,Ult12456:0.0):5.4E-
4,(((UltLent2:0.0,UltLent4:0.0):5.5E-
4,UltLent3:0.00367)0.793.56:0.00369,(Ult12453:5.5E-
4,Ult12452:0.0193)0.786.59:0.00367)0.901.77:0.0074)0.733.26:0.0029)0.635.
3:0.014)0.963.60:0.03847,Ult12463:0.03188)0.751.30:0.01176,(Ult12462:0.00
778,Ult12459:0.10095)0.760.54:0.00849)0.064.6:0.01159,(Ult12461:0.02417,U
lt12460:0.05194)0.846.83:0.03034)0.979.52:0.04341)0.361.7:0.03465)0.996.5
7:0.08075,Ult12476:0.0483)0.988.61:0.06959,Ult12475:0.04061)0.753.57:0.00
758,(UltVe256:0.07638,(UltOr171:0.063,(UltOr173:0.02714,UltOr172:0.01639)
0.894.66:0.02402)0.839.63:0.02042)0.744.38:0.00543)0.743.49:0.01688,((Ult

12480:0.03624,((Ult12481:0.04013,(Ult12484:0.03436,(CytSpe11:0.00729,Ult1
2483:0.02783)0.782.69:0.01318)0.813.47:0.01331)0.109.6:0.00718,(Ult12482:
0.02036,(UltLen12:0.05747,((Ult12477:0.00349,UltVe257:0.00391)0.171.10:0.
00372,(Ult12478:0.01517,Ult12479:0.01125)0.556.8:5.3E-
4)0.882.76:0.01188)0.840.65:0.01389)0.867.66:0.01514)0.908.85:0.02261)0.8
65.72:0.01304,(Ult12485:0.05812,(Ult12486:0.06864,UltOr174:0.02583)0.908.
86:0.02352)0.838.61:0.02851)0.875.75:5.4E-
4)0.960.69:0.03634,(UltSp254:0.08411,Ult12490:0.07959)0.956.61:0.05347)0.
771.67:0.01783)0.801.57:0.01868)0.748.45:0.00436)0.966.74:0.04461)0.975.4
9:0.05943,((((((Ult11386:0.01524,Ult11308:0.0153)0.773.58:0.00317,(((Ult1
1321:5.5E-
4,((Ult11296:0.00741,((Ult11331:0.00369,Ult11294:0.02662)0.702.10:5.5E-
4,((Ult11295:0.00749,Ult11288:0.00371)0.835.57:5.5E-
4,(Ult11304:0.0,Ult11257:0.0,Ult11287:0.0):5.5E-4)0.000.542:5.5E-
4)0.278.7:5.4E-
4)0.776.60:0.00381,((Ult11336:0.03467,Ult11338:0.06533)0.924.74:0.01871,(
Ult11273:0.01867,(((Ult11263:0.00369,(Ult11303:0.00369,(((((Ult11302:0.0,
Ult11307:0.0,Ult11305:0.0,Ult11309:0.0,Ult11310:0.0,Ult11312:0.0,Ult11315
:0.0,Ult11319:0.0,Ult11320:0.0,Ult11322:0.0,Ult11324:0.0,Ult11325:0.0,Ult
11326:0.0,Ult11330:0.0,Ult11328:0.0,Ult11329:0.0,Ult11333:0.0,Ult11256:0.
0,Ult11259:0.0,Ult11270:0.0,Ult11271:0.0,Ult11277:0.0,Ult11278:0.0,Ult112
80:0.0,Ult11283:0.0,Ult11286:0.0,Ult11289:0.0,Ult11290:0.0,Ult11292:0.0,U
lt11293:0.0,AkkMucin:0.0,Ult11339:0.0):5.5E-
4,(((((((Ult11276:0.0074,(Ult11327:0.00371,(((Ult11265:0.0,Ult11269:0.0)
:5.5E-
4,(Ult11262:0.00369,((Ult11268:0.1022,Ult11340:0.15315)0.721.12:0.00863,U
lt11266:5.5E-4)0.851.69:0.00379)0.405.15:5.5E-4)0.414.5:5.5E-
4,(Ult11261:0.00369,Ult11267:5.5E-
4)0.856.58:0.00369)0.831.44:0.0038)0.348.9:5.4E-4)0.267.6:5.5E-
4,Ult11264:0.00369)0.203.6:5.5E-4,Ult11323:0.00369)0.000.543:5.5E-
4,Ult11291:0.00369)0.000.544:5.5E-4,Ult11301:0.00741)0.000.545:5.5E-
4,Ult11313:0.00369)0.000.546:5.5E-4,Ult11281:0.00368)0.000.547:5.5E-
4,Ult11258:0.00741)0.444.17:5.5E-4)0.000.548:5.5E-
4,((Ult11335:0.00231,Ult11279:0.03045)0.974.37:0.0015,Ult11337:0.04241)0.
542.7:5.5E-4)0.504.4:5.5E-4,Ult11272:0.00369)0.000.549:5.5E-
4,Ult11260:0.01115)0.000.550:5.5E-4)0.339.11:5.5E-4)0.166.7:5.4E-
4,((Ult11317:0.00371,(Ult11316:0.02605,((Ult11300:0.0358,Ult22085:0.02747
)0.850.77:0.01167,Ult11332:0.06523)0.741.39:0.00517)0.974.38:0.02266)0.98
3.39:0.02257,Ult11314:5.5E-4)0.863.80:0.00713)0.583.5:5.5E-
4,(Ult11275:0.04632,Ult11274:0.04168)0.766.52:0.00492)0.706.19:5.5E-
4)0.299.10:5.5E-4)0.896.64:0.01073)0.840.66:0.00709)0.909.64:5.4E-
4,Ult11341:0.05465)0.812.41:0.0037,(Ult11306:0.0,Ult11285:0.0,Ult11342:0.
0):5.4E-4)0.989.56:5.4E-
4)0.753.58:0.0224,(((Ult11368:0.03463,(Ult11363:0.01282,(Ult21855:0.04765
,(Ult11366:0.00974,Ult11367:0.01455)0.801.58:0.01339)0.791.54:0.00782)0.9
63.61:0.02945)0.263.5:0.00996,((Ult11346:0.01503,(Ult11347:0.00369,(Ult11
348:0.0,Ult11349:0.0):5.5E-4)0.842.75:5.5E-
4)0.979.53:0.01971,((((Ult11380:5.5E-
4,((Ult11311:0.0,Ult11382:0.0,Ult11383:0.0):5.5E-
4,Ult11381:0.00373)0.936.70:0.00753)0.869.66:0.0082,(((Ult11375:0.0,Ult11
374:0.0):5.3E-4,((Ult11351:0.0,Ult11377:0.0):5.5E-
4,Ult11376:0.00371)0.926.87:0.00751)0.774.71:0.00402,(Ult11370:0.01864,(U
lt11369:0.00369,(Ult11371:0.0,Ult11372:0.0):5.5E-4)0.804.44:5.5E-
4)0.999.103:0.02678)0.719.11:0.00307)0.838.62:0.01101,(((Ult11359:0.0,Ult

11373:0.0):5.5E-4,Ult11361:0.01111)0.928.62:5.4E-
4,(Ult11358:0.07115,Ult11360:0.06059)0.732.20:5.4E-
4)0.773.59:0.00547)0.895.88:0.0106,((Ult11379:5.3E-
4,(Ult11378:0.00599,Ult11357:0.01003)0.361.8:0.01024)0.408.8:0.00706,(((U
lt11365:5.5E-4,(Ult11350:5.5E-
4,Ult11345:0.00372)0.843.54:0.00373)0.794.50:0.00411,(Ult11352:0.01023,Ul
t11354:0.03945)0.813.48:0.0089)0.697.6:0.00417,((Ult11353:0.00369,Ult1136
2:5.5E-
4)0.964.68:0.01581,(Ult11355:0.01126,(Ult11356:0.0,Ult11364:0.0):5.5E-
4)0.751.31:0.00316)0.705.17:0.0037)0.895.89:0.01169)0.898.89:0.01194)0.01
1.7:5.4E-
4)0.710.17:0.00295)0.687.14:0.00255,(Ult11385:0.01204,Ult11384:0.05926)0.
858.69:0.02642)0.974.39:0.07249)0.939.65:0.08902,Ult12211:0.13083)0.881.8
5:0.06812,(((Ult11782:0.03234,Ult11801:0.102)0.985.53:0.1017,((((Sgfffff0
:0.0,ZnrEEErE:0.0):0.00951,Otu00586:0.01323)0.982.45:0.06103,((UltVe118:0
.15255,((UltVe121:0.05671,(((Ult11790:0.01098,Ult11791:5.4E-
4)0.894.67:0.02369,(Ult11786:0.01508,(Ult11787:0.00372,(Ult11789:0.01912,
(Ult11788:0.01131,Ult11785:5.5E-4)0.592.6:5.4E-
4)0.896.65:0.00752)0.858.70:0.00776)0.964.69:0.03715)0.942.68:0.04163,(Ul
t11792:0.07691,((VrcBact3:5.3E-
4,((Ult11797:0.0,Ult11798:0.0):0.00753,Ult11799:0.00757)0.888.86:0.00728)
0.914.87:0.03281,(MlhInfer:0.01478,Ult11796:0.00421)0.936.71:0.04333)0.99
1.57:0.06294)0.422.7:0.01961)0.743.50:0.01266)0.623.6:0.02319,Ult11793:0.
08593)0.362.8:0.03026)0.924.75:0.03619,Ult11794:0.07795)0.210.11:0.00538)
0.934.68:0.04404,Ult11795:0.14793)0.806.39:0.02954)0.927.74:0.04056,(((Ul
t11783:0.02728,Ult11784:0.03052)0.934.69:0.02901,((Ult12074:0.07216,(Ult1
1800:0.08587,((Ult12076:0.04499,Ult12075:0.02226)0.395.8:0.0152,(UltVe159
:0.09191,((((Ult12070:0.02483,UltVe158:0.01433)0.731.21:0.00322,UltVe157:
0.03281)0.916.88:0.0122,Ult12071:0.01378)0.680.14:0.00609,(Ult12073:0.008
16,(Ult12072:0.00766,Ult15343:0.00352)0.998.84:0.0539)0.772.61:0.01083)0.
452.10:0.03729)0.969.48:0.05355)0.978.38:0.03563)0.751.32:0.00515)0.768.4
2:0.01074,((Ult11888:0.08969,UltCrat6:0.03434)0.816.47:0.01808,(((Ult1188
2:0.03536,(Ult11880:0.04215,Ult11881:0.00773)0.930.65:0.03418)0.997.70:0.
07341,((((((Ult11980:0.0184,((Ult11978:0.03257,Ult11979:0.04228)0.872.82
:0.01482,(Ult11845:0.02859,(((((Ult11921:0.03434,(UltVe141:0.03234,((Ult1
1963:0.01151,(Ult11964:0.0255,Ult11850:0.03025)0.849.60:0.01064)0.798.55:
0.00835,(Ult11849:5.4E-
4,((Ult11847:0.00741,(Ult11846:0.01881,(UltVe134:0.01121,(Ult11951:5.5E-
4,Ult11844:0.00746)0.782.70:0.00369)0.612.12:5.4E-
4)0.972.56:0.01552)0.748.46:0.00481,UltVe133:0.04037)0.867.67:0.01054)0.9
66.75:0.02577)0.737.44:0.00979)0.542.8:0.00564)0.429.7:0.01013,((((Ult118
92:0.0037,Ult11893:0.0037)0.799.59:0.00367,(Ult11911:0.00369,Ult11977:0.0
1918)0.999.104:5.4E-
4)0.875.76:0.00741,(Ult11894:0.00371,(((Ult11950:0.01213,((UltrS134:0.0,U
lt11947:0.0):5.4E-4,(Ult11945:5.5E-
4,Ult11946:0.00368)0.919.93:0.00729)0.801.59:0.00649)0.644.9:0.00748,(Ult
11948:0.05085,(Ult11942:5.5E-4,Ult11949:0.00747)0.792.65:5.5E-
4)0.854.71:0.00191)0.636.6:0.00551,(Ult11944:0.0,Ult11941:0.0):5.3E-
4)0.794.51:0.00371)0.462.10:5.5E-4)0.159.3:5.4E-4,((((Ult12022:5.4E-
4,(Ult12023:0.00758,Ult12024:0.00367)0.905.68:0.00752)1.000.524:0.07686,(
(((Ult11899:0.00473,(Ult11933:0.00332,(Ult11870:0.00904,Ult12013:0.00704)
0.877.89:0.0106)0.873.78:0.00791)0.894.68:0.01047,Ult11900:0.0037)0.793.5
7:0.00371,(Ult11934:0.00747,(((UltVe140:5.5E-
4,Ult11897:0.00745)0.854.72:0.0037,((BacElli9:0.0037,Ult14250:0.00742)0.8

32.54:5.5E-4,(Ult11922:0.0,Ult11938:0.0,Ult11939:0.0):5.5E-
4)0.714.20:5.3E-4)0.413.11:5.5E-
4,(((((((Ult12053:0.04011,Ult12062:0.02759)0.755.46:0.00364,Ult11988:5.4E
-4)0.774.72:0.00371,Ult11987:0.00747)0.810.48:0.00369,Ult11986:5.3E-
4)0.850.78:0.0037,Ult11908:5.5E-4)0.923.100:5.5E-4,((Ult11905:5.5E-
4,Ult11906:0.02656)0.978.39:0.01508,((Ult11909:0.0037,(Ult11936:0.01529,U
lt11937:0.01122)0.639.11:5.4E-
4)0.763.67:0.00377,Ult11910:0.02723)0.631.10:0.01126)0.996.58:5.5E-
4)0.908.87:5.3E-
4,((Ult11869:0.0,Ult11872:0.0,Ult11895:0.0,Ult11930:0.0,Ult11932:0.0):0.0
0712,(Ult11923:0.01926,((Ult11928:0.0,Ult11929:0.0):0.01141,Ult11931:0.00
366)0.904.80:0.0077)0.912.95:5.4E-
4)0.987.69:0.01911)0.972.57:0.01486)0.769.56:0.00365)0.776.61:0.00367)0.5
51.9:5.4E-4,(Ult11907:0.00368,Ult11896:0.03172)0.960.70:5.4E-
4)0.909.65:0.00734)0.525.9:5.3E-4,Ult11901:5.5E-4)1.000.525:5.3E-
4,((Ult15342:0.01881,(UltVe142:0.00367,((BacEll10:0.0,Ult11935:0.0):5.5E-
4,((Ult11902:0.00777,Ult11903:0.00348)0.635.4:0.00754,Ult11898:0.01486)0.
930.66:5.4E-4)0.758.62:5.4E-4)0.730.26:5.5E-
4)0.825.47:0.00366,(Ult11904:5.3E-4,UltrS133:0.00373)0.752.54:5.4E-
4)0.861.73:0.00359)0.735.30:0.00736)0.771.68:0.00437)0.799.60:0.0029,((((
(Ult11925:0.0,Ult11926:0.0):5.5E-
4,Ult11927:0.0037)0.960.71:0.01121,Ult11871:5.3E-
4)0.954.63:0.01129,((Ult11940:0.00745,Ult11955:0.01508)0.653.6:5.5E-
4,((Ult11914:0.0,Ult11915:0.0):5.5E-
4,(Ult11924:0.01519,Ult11917:0.00381)0.534.7:0.00748)0.921.86:0.01123)0.6
24.13:0.00368)0.910.74:5.4E-4,Ult11912:0.00369)0.282.5:5.4E-
4)0.996.59:5.4E-
4,(Ult11916:0.01759,((Ult11873:0.0,Ult11918:0.0):0.01229,Ult11913:0.01714
)0.872.83:0.01119)0.861.74:0.0069)0.813.49:0.01998,(Ult11957:0.00583,(Ult
11956:0.00532,Ult11958:0.0306)0.940.73:0.02174)0.925.90:0.01861)0.841.59:
0.008)0.902.80:0.0136)0.615.12:5.4E-
4)0.971.49:0.01708,(((UltVe146:0.08133,Ult14310:0.11195)0.889.70:0.03598,
(UdnVerru:0.01312,Ult12063:0.01883)0.996.60:0.05871)0.682.12:0.00327,((Ul
tVe132:0.01975,((Ult11868:0.0,Ult11969:0.0):0.02794,(Ult11827:0.02582,Ult
11852:0.046)0.093.7:0.00846)0.908.88:0.01987)0.736.32:0.00403,((((Ult1184
0:0.05367,((Ult12015:0.01121,Ult12016:5.5E-
4)0.981.49:0.02259,(Ult11822:0.01141,Ult16843:0.01139)0.946.65:0.00118)0.
874.87:0.00766)0.890.82:0.0147,((BacElli8:0.0,Ult11821:0.0):0.03529,(Ult1
1962:0.04885,((Ult11816:5.5E-
4,(Ult11817:0.01478,((Ult11820:0.0116,(Ult11879:5.4E-
4,(UltVe126:0.0034,Ult11818:0.00404)0.196.8:0.00373)0.821.43:0.00684)0.92
1.87:0.01178,Ult11819:0.00769)0.758.63:0.00529)0.913.74:0.01059)0.917.78:
0.01898,Ult11823:5.3E-
4)0.865.73:0.00761)0.351.9:0.0079)0.777.60:0.00357)0.671.9:0.00199,(Ult11
853:0.03023,(Ult11854:0.00801,Ult11855:0.03117)0.924.76:0.02553)0.837.49:
0.01744)0.860.51:0.00946,((Ult11864:0.03295,(Ult12055:0.00161,(Ult12058:0
.02403,(Ult12056:0.04278,(Ult12054:5.5E-4,Ult12057:0.00369)0.975.50:5.5E-
4)0.737.45:0.00254)0.973.41:0.03456)0.991.58:0.039)0.703.19:0.00264,(Ult1
5341:0.01468,(Ult11972:0.01025,Ult11971:0.03649)0.811.47:0.00798)0.253.9:
5.4E-
4)0.961.57:0.01552)0.754.47:0.00428)0.932.72:0.01631)0.810.49:0.00726)0.9
45.65:5.4E-4,(((Ult12026:5.5E-
4,Ult16029:0.00743)0.824.45:0.01333,(Ult12061:0.03449,(Ult12059:5.5E-
4,Ult12060:0.00741)0.896.66:0.01986)0.927.75:0.03384)0.902.81:0.0278,(Ult

11976:0.00443,((Ult11991:0.01948,((Ult11996:5.5E-
4,(((((Ult11865:0.00369,((Ult11994:0.0,Ult11995:0.0):0.00748,UltrS136:0.0
0367)0.882.77:0.00748)0.914.88:5.4E-
4,Ult11999:0.0227)0.958.75:0.01131,(((UltrS137:0.00371,UltrS138:0.01117)0
.885.66:0.0074,UltVe145:5.5E-
4)0.855.69:0.0037,(Ult12000:0.0037,((Ult12001:5.4E-
4,(Ult11997:0.00746,Ult11998:0.00384)0.243.5:0.00728)0.941.67:0.01539,Ult
12002:0.00359)0.882.78:0.0077)0.866.69:5.5E-4)0.865.74:5.5E-
4)0.872.84:0.00758,UltVe144:0.0195)0.867.68:0.00767,(Ult11989:0.00369,Ult
11990:5.5E-4)0.792.66:0.00363)0.889.71:0.00754)0.812.42:5.4E-
4,(Ult11993:0.02398,UltrS139:0.0195)0.740.45:0.00342)0.894.69:0.01138)0.8
70.55:0.01232,(Ult12005:0.03069,(((Ult12031:5.4E-
4,(Ult12030:0.01524,Ult12032:0.02313)0.911.91:0.01117)0.950.62:0.02028,((
Ult12029:0.00739,(Ult12028:0.02284,(UltVe148:5.5E-
4,(BacEll12:0.01545,(UltVe149:0.00369,UltrS141:5.5E-
4)0.764.61:0.00364)0.865.75:0.00747)0.407.8:0.00381)0.859.63:0.01136)0.95
3.53:5.5E-4,(Ult12027:5.5E-
4,(UltVe147:0.02765,UltrS140:0.0114)0.750.36:0.00369)0.932.73:0.00745)0.9
38.48:0.0184)0.890.83:0.01469,Ult12006:0.02847)0.752.55:0.00447)0.844.73:
0.00804)0.981.50:0.03981)0.868.60:0.02217)0.923.101:0.02322)0.807.39:0.00
777,(Ult11851:0.041,Ult11842:0.03953)0.356.10:0.00348)0.948.73:0.01882,(U
ltVe138:0.05077,((Ult11835:0.05559,(Ult11836:0.01656,(Ult11887:0.01138,(U
ltVe137:0.00736,(Ult11886:0.01143,Ult11885:0.01525)0.756.57:0.00389)0.856
.59:0.00755)0.953.54:0.01935)0.669.10:0.00939)0.888.87:0.01389,((Ult11841
:0.02784,(((((UltVe129:0.0167,UltVe130:0.03753)0.902.82:0.02048,((Ult118
33:0.0,UltGa290:0.0):5.3E-
4,(UltVe128:0.0136,Ult11832:0.02213)0.919.94:0.01378)0.968.60:0.02806)0.9
06.89:0.02005,((((Ult12035:0.05864,(Ult12042:0.03445,Ult12040:0.04774)0.0
70.4:0.01752)0.450.10:0.00768,Ult12041:0.04581)0.885.67:0.01534,((UltVe15
2:0.01158,((Ult11837:0.05007,Ult11863:0.16454)0.918.73:0.03794,Ult11867:0
.00687)0.964.70:0.04129)0.744.39:0.00365,UltVe151:0.01156)0.927.76:0.0155
6)0.789.52:0.00377,(((UltVe139:0.01119,Ult12037:0.00714)0.913.75:5.4E-
4,(Ult11970:0.01541,(Ult11889:0.01939,Ult11890:0.01152)0.761.50:0.00333)0
.901.78:0.0078)0.784.57:0.0038,(((Ult11866:0.0,Ult12036:0.0):0.02413,(Ult
12034:0.00749,UltVe150:0.00372)0.849.61:0.00735)0.725.22:0.01121,Ult12033
:0.10794)0.972.58:5.3E-
4)0.757.44:0.0037)0.780.73:0.00399)0.102.4:0.00321,((((Ult12007:0.00626,U
lt12038:0.02394)0.684.10:0.00649,(Ult12004:0.03552,Ult12025:0.0299)0.902.
83:0.02211)0.865.76:0.01161,((((Ult12039:0.03302,(Ult12047:5.4E-
4,((Ult11861:0.00741,(Ult11862:5.5E-4,(Ult12051:0.0,Ult12049:0.0):5.5E-
4)0.957.57:5.4E-4)0.676.6:0.00744,(Ult12048:0.00742,Ult12050:5.5E-
4)0.942.69:5.5E-
4)0.900.64:0.00723)0.901.79:0.01912)0.950.63:0.02061,(Ult11981:0.00391,(B
acEll11:0.01134,((UltVe143:0.00798,(Ult11984:0.01098,(UltrS132:0.01143,Ul
t12003:0.01143)0.886.96:5.5E-
4)0.791.55:0.00761)0.859.64:0.0072,((Ult11891:0.01526,Ult11982:5.4E-
4)0.963.62:0.01139,(Ult11983:0.00371,(Ult11953:0.00754,Ult11954:0.00372)0
.966.76:0.01528)0.912.96:5.3E-4)0.884.79:0.00749)0.281.3:5.5E-
4)0.875.77:0.00744)0.867.69:0.00849)0.766.53:0.00569,(Ult11985:0.02295,Ul
tVe155:0.03923)0.845.73:0.01258)0.759.66:0.0044,((Ult12009:0.0085,(Ult120
45:0.03532,Ult12010:0.00225)0.995.72:7.7E-
4)0.910.75:0.01237,(Ult12044:0.00976,Ult12046:0.01457)0.865.77:0.00959)0.
697.7:0.00408)0.926.88:0.01248)0.163.5:5.5E-
4,((((Ult11965:0.01868,Ult12008:0.01965)0.385.12:5.4E-

4,Ult12043:0.1183)0.783.66:0.0045,(((((Ult11960:0.01211,((Ult11966:0.0074
5,(Ult11876:0.00372,Ult11959:5.5E-4)0.250.7:5.5E-
4)0.786.60:0.00435,Ult11967:0.0267)0.340.9:0.00397)0.554.10:0.00666,Ult12
011:0.01139)0.000.551:5.3E-4,(Ult12019:5.5E-
4,(Ult12021:0.00753,(Ult12018:0.00372,Ult12020:0.00373)0.787.52:0.00371)0
.788.58:0.00369)0.987.70:0.02316)0.702.11:0.00753,Ult11943:0.00737)0.913.
76:5.3E-
4,((Ult12014:0.00848,(Ult11952:0.01807,Ult11973:0.00839)0.861.75:0.00771)
0.898.90:0.0102,(UltrS135:0.01121,(((Ult12017:0.02227,Ult11878:0.01623)0.
898.91:0.0116,(Ult11974:0.0,Ult11975:0.0):0.01044)0.786.61:0.00458,Ult119
68:0.00371)0.797.47:0.00369)0.477.6:5.4E-
4)0.725.23:0.00368)0.961.58:5.5E-
4)0.865.78:0.0108,Ult11839:0.01615)0.640.7:0.01158)0.907.73:0.01523)0.547
.6:0.01971,UltVe153:0.07009)0.625.6:0.00682,((Ult11884:0.04743,Ult12052:0
.02683)0.847.82:0.01534,(UltSlu30:0.07801,UltVe154:0.05063)0.728.27:0.017
41)0.719.12:0.01617)0.880.80:0.0176)0.879.74:0.01537,((((((Ult12065:0.003
74,UltChlam:0.01515)0.799.61:0.00368,Ult12066:5.4E-
4)0.837.50:0.00365,UltMar56:5.5E-
4)0.625.7:0.00293,(Ult12069:0.01444,(Ult12067:0.00898,Ult12068:0.01003)0.
937.67:0.0196)0.749.62:0.00976)0.983.40:0.05235,(((UltOr160:0.02274,(UltO
r162:0.00517,UltOr163:0.01023)0.972.59:0.03746)0.977.46:0.03688,(UltVe156
:0.01465,UltOr161:0.00628)0.870.56:0.01484)0.781.53:0.01315,Ult12064:0.02
494)0.994.63:0.06519)0.912.97:0.03461,Ult11992:0.02447)0.741.40:0.00681)0
.787.53:0.00735)0.136.6:0.00403)0.840.67:0.00893)0.884.80:0.01243,((((Ult
Pl544:0.02141,((((UltVe124:0.01165,Ult11810:0.01946)0.837.51:0.00743,Ult1
1811:0.00754)0.918.74:0.01165,Ult11809:5.5E-
4)0.794.52:0.00727,(Ult11830:0.01736,(UltVe127:0.01733,((Ult11824:0.00501
,Ult11825:0.01389)0.972.60:0.01946,(Ult11812:0.0073,Ult11813:5.4E-
4)0.978.40:0.02113)0.787.54:5.4E-
4)0.954.64:0.01955)0.765.44:0.00561)0.906.90:0.01569)0.853.60:0.00966,((U
ltVe123:0.01114,(((UltVe135:0.0114,(Ult11859:0.01136,UltVe136:0.00747)0.7
45.48:0.00367)0.960.72:0.01528,((((Ult11856:0.0,Ult11877:0.0):5.5E-
4,(Ult11848:0.02682,(Ult11858:0.00739,Ult11857:5.5E-4)1.000.526:5.5E-
4)0.858.71:0.00367)0.973.42:0.01119,Ult12012:0.02272)0.141.3:5.4E-
4,Ult11860:0.00369)0.792.67:0.00373)0.872.85:0.00744,((Ult11802:5.4E-
4,(Ult11803:0.01474,(Ult11808:0.01941,Ult11807:0.00717)0.194.6:0.00373)0.
898.92:5.3E-4)0.859.65:0.00737,(UltVe122:0.01603,(Ult11805:5.3E-
4,(Ult11804:0.02336,Ult11806:0.00353)0.923.102:0.0115)0.838.63:0.00742)0.
836.44:0.00762)0.899.77:0.00804)0.826.46:5.4E-
4)0.956.62:0.01811,(Ult11814:0.01136,UltVe125:0.00368)0.554.11:0.00757)0.
872.86:5.3E-
4)0.965.55:0.0156,((Ult11826:0.00827,Ult11843:0.0716)0.737.46:0.0036,(Ult
11828:0.0062,Ult11829:0.00616)0.831.45:0.00636)0.919.95:0.01103)0.861.76:
0.00794,(Ult11815:0.02995,Ult11961:0.02191)0.752.56:0.00663)0.755.47:0.00
609)0.745.49:0.01925,(Ult11831:0.01082,(Ult11919:0.00368,((Ult11875:0.0,U
lt11920:0.0):5.5E-4,Ult11874:0.00369)0.000.552:5.5E-
4)0.978.41:0.02395)0.862.67:0.0138)0.825.48:0.00908)0.735.31:0.00523,(Ult
11838:0.09483,(Ult11883:0.19539,(Ult11834:0.04749,UltVe131:0.03193)0.739.
33:0.02284)0.921.88:0.0453)0.935.69:0.0408)0.741.41:0.01168)0.949.65:0.03
463)0.200.6:0.01537)0.863.81:0.01151,(((UltVe119:0.05324,UltVe120:0.04256
)0.766.54:0.02072,(Ult15602:0.03216,Ult29201:0.06323)0.985.54:0.05396)0.6
85.14:0.00386,(((((((Ult11446:0.05995,(((UltVer56:0.04425,(Ult11467:0.015
84,(Ult11466:0.02764,UltVer55:0.0071)0.841.60:0.00752)0.936.72:0.01972)0.
934.70:0.02106,((Ult11465:0.04007,(Ult11460:0.00738,(((UltVer43:0.00552,(

Ult11457:0.01088,((UltVer44:5.5E-
4,(UltVer46:0.03707,Ult11459:0.05026)0.664.8:0.01087)0.831.46:0.0086,(Ult
Ver45:0.00343,UltVer68:0.01152)0.977.47:0.02704)0.931.67:0.01927)0.812.43
:0.00816)0.956.63:0.01844,((UltVer48:0.0141,((((UltVer39:5.5E-
4,UltVer40:5.5E-
4)0.804.45:0.00405,(UltVer38:0.02276,(UltVer42:0.00309,Ult11456:0.01662)0
.615.13:0.00772)0.721.13:5.4E-4)0.977.48:0.02039,Ult11453:5.3E-
4)0.639.12:0.00807,((Ult11455:0.00594,Ult11454:0.01036)0.817.33:0.00692,(
UltVer41:0.01515,UltVer47:5.4E-
4)0.777.61:0.009)0.844.74:0.00929)0.906.91:0.00418)0.868.61:0.00403,(UltO
r152:0.0037,UltVer50:0.02318)0.882.79:0.01249)0.978.42:0.02497)0.721.14:5
.4E-
4,Ult11458:0.00377)0.781.54:0.00396)0.978.43:0.03424)0.897.69:0.01361,(((
Ult11472:0.01225,(Ult11462:0.00439,(UltVer53:0.00744,UltVer54:5.4E-
4)0.489.7:0.00691)1.000.527:0.08467)0.769.57:0.00815,Ult11468:0.05166)0.8
00.46:0.01547,((UltVer57:0.02377,(Ult11469:0.0,Ult11470:0.0):0.00327)0.97
5.51:0.02382,(((Ult11444:0.01939,Ult11443:0.06732)0.911.92:0.01943,((((((
Ult11435:0.00389,UltVer23:0.027)0.983.41:0.02982,UltVer10:0.03388)0.774.7
3:0.0076,(((UltVerr6:0.02807,(HlfSarga:0.01325,VrcBacte:0.00602)0.930.67:
0.02182)0.078.7:0.00917,((HlfPhyci:0.01919,HlfHelve:0.00372)0.941.68:0.01
579,(Ult11397:0.01925,(UltVerr4:5.4E-
4,(HlfHaren:0.0119,(UltVerr5:0.00797,HlfRosea:0.0072)0.738.24:0.00374)0.9
44.59:0.01499)0.357.9:0.00759)0.859.66:0.01103)0.893.80:0.01702)0.790.61:
0.00709,(((((((((Ult11415:0.0,Ult11416:0.0):0.00371,((Ult11421:0.00369,(
((UltVer17:0.0,UltVer19:0.0,Ult11417:0.0,Ult11422:0.0):5.5E-
4,((Ult11419:0.08283,UltVer18:0.00262)0.999.105:0.00116,((Ult11420:0.0036
9,UltCrat5:5.5E-
4)0.931.68:0.00745,(UltVer21:0.00372,Ult11393:0.01497)0.290.8:5.5E-
4)0.000.553:5.5E-4)0.458.14:5.5E-4)0.000.554:5.5E-
4,UltVer20:0.00369)0.000.555:5.5E-4)0.251.6:5.4E-
4,Ult11418:0.00369)0.905.69:0.00762)0.716.16:5.4E-
4,(Ult11408:0.00353,Ult11426:0.00387)0.392.6:0.00372)0.743.51:5.5E-
4,(Ult11423:0.00348,UltVer22:0.01182)0.807.40:0.01147)0.891.62:0.01099,Ul
t11425:5.4E-4)0.839.64:0.00374,VrrBact2:5.5E-4)0.892.66:5.4E-
4,((((Ult11404:0.00367,(Ult11403:5.5E-
4,(Ult11402:0.00369,(UltVer11:0.0,Ult11432:0.0):0.0037)0.775.65:5.5E-
4)0.891.63:5.5E-4)0.021.1:5.4E-
4,(((Ult11431:0.00908,Ult11400:0.01906)0.900.65:0.00997,(((Ult11410:0.007
41,Ult11405:0.00369)0.628.2:5.5E-4,Ult11409:5.5E-4)0.000.556:5.5E-
4,(UltVer12:0.0,Ult11406:0.0,Ult11407:0.0):5.5E-4)0.947.57:5.3E-
4)0.829.60:5.3E-4,((UltVer13:0.0,Ult11411:0.0,UltVer14:0.0):5.3E-
4,(Ult11430:0.00733,Ult11414:5.4E-
4)0.820.33:0.00363)0.983.42:0.01477)0.851.70:0.00363)0.778.62:0.0037,Ult1
1429:0.00371)0.932.74:0.01128,((VrrBact3:5.5E-
4,(Ult11428:0.01914,Ult11427:0.00367)0.931.69:5.5E-4)0.180.6:5.5E-
4,Ult11401:0.01122)0.362.9:5.4E-
4)0.845.74:0.00368)0.949.66:0.00767,((Ult11390:5.4E-4,(((VrrBacte:5.5E-
4,(((Ult11388:0.03525,Ult11389:0.00778)0.869.67:0.00719,(Ult11387:0.00718
,UdnBac43:5.5E-4)0.885.68:5.4E-
4)0.821.44:0.00372,(Ult11391:0.00717,(UltVerr2:0.0348,Ult11424:0.00372)0.
925.91:5.4E-4)0.862.68:0.00716)0.132.10:5.5E-4)0.999.106:5.3E-
4,Ult11392:0.05958)0.831.47:0.00361,UltVerr3:5.4E-
4)0.899.78:0.00742)0.992.60:0.02345,Ult11434:0.00354)0.836.45:0.00763)0.8
74.88:5.4E-

4,(LtlPohnp:0.0106,(Ult11433:0.01447,(Ult11452:0.01513,UltVer37:0.01608)0
.968.61:0.02542)0.978.44:0.03065)0.922.102:0.02007)0.972.61:0.02324,(((Ul
t11413:5.5E-4,Ult11398:0.00741)0.214.5:5.4E-
4,(Ult11412:0.00259,Ult11399:0.0631)0.999.107:0.00119)0.883.85:0.0075,(Ul
tVer15:0.00374,UltVer16:0.01128)0.882.80:0.00752)0.776.62:0.00355)0.749.6
3:0.00445)0.741.42:0.00484)0.407.9:0.00566,((((((UltOr147:0.01383,UltOrg1
6:0.03005)0.863.82:0.00929,(UltOr145:0.0,UltOr149:0.0):5.5E-
4)0.201.5:5.4E-4,UltOr148:0.00369)0.971.50:0.01132,((Ult11395:5.5E-
4,((Ult11394:0.01068,UltOr146:0.06761)0.749.64:0.00474,Ult11396:0.0037)0.
650.8:5.4E-4)0.912.98:0.00368,UltVerr8:5.3E-4)0.684.11:5.4E-
4)0.902.84:0.00745,LtlAlgae:5.5E-4)0.795.52:5.4E-
4,UltVerr9:0.03056)0.761.51:0.00406)0.757.45:0.00426,((((((UltVer24:0.0,U
ltVer26:0.0):0.01912,((UltRubri:0.00734,(Ult11437:0.01135,((RrtSpong:0.00
75,(UltVer27:0.00369,UltVer28:5.5E-
4)0.937.68:0.01129)0.768.43:0.00365,UltOpitu:5.4E-
4)0.764.62:0.00373)0.788.59:0.0115)1.000.528:5.4E-4,RrtTange:5.4E-
4)0.905.70:0.00743)0.039.3:5.4E-
4,Ult11436:0.04811)0.292.4:0.00375,UltVer25:0.00747)0.950.64:0.01985,(Rrt
Sabul:0.00721,VrcBact2:5.5E-
4)0.946.66:0.01866)0.835.58:0.00794,(((Ult11451:0.07649,(((Ult11450:0.011
54,UltVer36:0.03133)0.861.77:0.01503,((UltVer35:0.00363,UltOr151:0.01141)
0.880.81:0.01148,((Ult11447:0.00369,Ult11448:0.0037)0.940.74:0.01096,(Ult
11449:0.01889,UltVer34:5.5E-4)0.879.75:5.5E-
4)0.893.81:0.01156)0.886.97:0.01177)0.990.58:0.03771,(PcdSedim:0.0378,(Ul
t11440:9.2E-4,(Ult11441:0.01518,((UltVer29:0.03469,UltVer30:5.4E-
4)0.827.52:0.00364,((Ult11442:0.00363,(UltMar52:5.4E-
4,(UltVer31:0.00785,UltVer49:0.01956)0.766.55:0.0033)0.432.13:0.0037)1.00
0.529:5.4E-4,Ult11439:0.00743)0.498.6:5.4E-
4)0.747.42:0.0038)0.856.60:0.01812)0.991.59:0.06342)0.942.70:0.03343)0.86
5.79:0.01169)0.715.15:0.00494,Ult11438:0.04504)0.425.5:0.00927,UltVerr7:0
.05403)0.734.35:0.005)0.985.55:0.02671)0.937.69:0.01833,((RbsPersi:0.0145
8,RbsIshig:0.00428)0.946.67:0.02046,(UltOr150:0.02727,(UltVer32:0.00469,(
UltVer33:0.02258,Ult11445:0.00824)0.184.3:0.01171)0.826.47:0.01223)0.951.
65:0.0276)0.971.51:0.03115)0.652.10:5.5E-
4)0.997.71:0.04043,(Ult11471:0.0281,(Ult11463:0.01885,Ult11464:0.00375)0.
923.103:0.01184)0.716.17:0.00433)0.504.5:0.00721)0.951.66:5.4E-
4)0.961.59:0.01649)0.744.40:0.00614)0.795.53:0.00972,((Ult11461:0.0147,(U
ltMar54:0.00753,(UltMar53:5.5E-
4,UltVer51:0.0037)0.783.67:0.00371)0.783.68:0.0087)0.375.9:0.00677,UltVer
52:0.09431)0.693.15:0.00435)0.749.65:0.01052)0.697.8:0.02131,Ult11499:0.0
3676)0.883.86:0.0161,(UltVer63:0.06792,((((UltOr158:0.00382,UltOr159:0.03
517)0.764.63:0.00358,UltVe117:5.5E-4)0.957.58:5.3E-
4,UltOr157:0.06088)1.000.530:0.08562,(UltMar55:0.05036,Ult11473:0.02033)0
.949.67:0.04981)0.369.8:0.02693)0.559.6:0.02184)0.723.22:0.00919,(UltVer6
4:0.05282,((((Ult11484:0.00725,Ult11485:0.02953)0.911.93:0.0224,(Ult11482
:0.0235,(Ult11488:0.02277,Ult11483:0.0038)0.740.46:0.00318)0.858.72:0.008
81)0.926.89:0.01919,((((Ult11478:0.00576,(PtbDebon:0.01466,(PtbFusif:0.00
702,PtbDejon:0.00424)0.679.15:0.01151)0.252.4:0.00439)0.988.62:0.00127,(U
lt11479:5.3E-
4,(Ult11477:0.01553,Ult11480:0.01551)0.849.62:0.0073)0.953.55:0.01174)0.7
55.48:0.00392,(((UltVer58:0.01513,((Ult11481:0.01503,Ult11486:5.4E-
4)0.989.57:0.02302,(Ult11474:5.5E-
4,(PtbFluvi:0.00743,Ult11475:0.0037)0.682.13:5.3E-
4)0.799.62:0.00385)0.889.72:5.4E-

4)0.851.71:0.00717,PtbVanne:0.00373)0.786.62:0.00332,Ult11476:0.0041)0.816.48:0.00743)0.995.73:0.04493,UdnBac44:0.00309)0.877.90:0.01606)0.685.15:0.00255,(UltVer59:5.4E-
4,UltVer60:0.00741)1.000.531:0.06401)0.982.46:0.0362)0.667.15:0.01872)0.807.41:0.01034,((Ult11493:0.07845,((Ult11497:5.5E-
4,(Ult11495:0.02757,(UltAci41:0.02211,UltSlu29:0.05148)0.921.89:0.02441)0.722.13:0.00335)0.761.52:0.01107,Ult11496:0.01219)1.000.532:0.15986)0.321.6:0.00992,(Ult11492:0.25421,Ult11491:0.15052)0.800.47:0.03798)0.881.86:0.02393)0.552.9:0.00546,(((((Ult11489:5.4E-
4,(UltVer61:0.0043,UltVer62:0.00379)0.993.51:0.02243)0.999.108:0.0501,(UltVer66:0.01039,((VrmSpino:0.00751,UltVer65:0.01513)0.979.54:0.02361,(Ult11487:0.00369,Ult11494:5.5E-
4)0.943.66:0.01561)0.862.69:0.01222)0.840.68:0.00959)0.217.6:0.01025,(Ult15401:0.04767,Ult15404:0.0854)0.962.72:0.04196)0.309.5:5.4E-
4,Ult11498:0.06287)0.970.56:0.03375,((UltVer67:0.00366,Ult11500:5.5E-
4)1.000.533:0.08674,(Ult11501:0.04424,((((Ult11506:0.00358,Ult11507:0.02739)0.881.87:0.01084,Ult11505:0.04906)0.905.71:0.01956,(Ult11503:0.00745,Ult11504:0.00371)0.866.70:5.3E-
4)0.977.49:0.02529,Ult11502:0.00144)0.930.68:0.0212)0.805.50:5.7E-
4)0.956.64:0.03006)0.128.2:0.00445)0.987.71:0.04048,(UltXiph4:0.03175,((((((Ult11747:0.11134,(Ult11684:0.03524,((UltrS131:0.01921,Ult11686:0.0329)0.850.79:0.01504,((Ult11681:0.04141,BacElli4:0.00343)0.988.63:0.02395,UltVer92:0.01125)0.174.6:5.4E-4)0.913.77:0.01118)0.910.76:5.4E-
4)0.414.6:0.01482,Ult11685:0.00387)0.761.53:0.00354,(BacElli5:5.4E-
4,(((((Ult11703:0.12774,(Ult11701:0.04986,((Ult11699:5.3E-
4,Ult11700:0.02668)0.998.85:0.07216,Ult11702:0.06908)0.407.10:0.01667)0.926.90:0.03443)0.911.94:0.0343,Ult11691:0.00982)0.822.40:5.2E-
4,(Ult11690:0.016,Ult11689:0.04299)0.750.37:0.01269)0.895.90:0.00959,((UltVer95:0.03569,UltVer96:0.01524)0.970.57:0.02858,(Ult11687:5.5E-
4,Ult11688:0.00368)1.000.534:0.06783)0.753.59:0.00252)0.846.84:0.00778,Ult11708:0.03578)0.905.72:0.01893)0.759.67:0.00354)0.903.73:0.00748,(((Ult11698:0.00762,((Ult11611:0.0,Ult11697:0.0):0.00759,UltVer97:0.01546)0.901.80:0.01133)0.846.85:0.00986,Ult11704:0.05262)0.887.82:0.01346,(UltVer94:5.5E-4,Ult11730:0.03643)0.759.68:0.00382)0.937.70:0.01559)0.683.18:5.4E-
4,(((UltVer93:0.01531,(((Ult11705:5.5E-4,UltXiph3:0.00369)0.692.7:5.3E-
4,(UltEndo8:0.01516,(((Ult11714:5.5E-4,Ult11715:0.00736)0.758.64:5.4E-
4,(Ult11713:0.00719,Ult11716:0.01932)0.906.92:5.4E-
4)0.911.95:0.00741,(((UltVer99:0.04285,Ult11607:0.00365)0.991.60:5.4E-
4,Ult11707:5.5E-
4)0.913.78:0.00698,((Ult11706:0.01497,(((Ult11694:0.0,Ult11696:0.0):5.5E-
4,(((BacElli6:0.0,Ult11709:0.0):5.5E-4,UltVe100:0.0037)0.355.11:5.4E-
4,(BacElli7:5.5E-
4,Ult11710:0.0037)0.861.78:0.0037)0.935.70:0.00749)0.390.9:5.4E-
4,Ult11695:0.01881)0.972.62:0.01539)0.738.25:5.5E-
4,(Ult11711:0.0,Ult11712:0.0):5.5E-
4)0.921.90:0.00205)0.912.99:0.00209)0.783.69:5.5E-
4)0.918.75:0.0074)0.651.15:5.5E-
4,UltVer98:0.02998)0.860.52:0.00749)0.974.40:0.01882,((Ult11693:0.00667,(Ult11683:5.3E-
4,(Ult11599:0.01542,Ult11682:0.01128)0.874.89:0.01159)0.913.79:0.01755)0.971.52:0.02204,((UltVe102:5.5E-
4,(UltVe104:0.00276,Ult11692:0.03224)0.776.63:0.01235)0.808.51:0.00365,UltVe103:0.00744)0.954.65:5.5E-4)0.822.41:0.01099)0.876.67:5.4E-
4,(((Ult11729:0.01912,(((UltVe107:0.0,Ult11726:0.0):5.5E-

4,(Ult11727:0.00372,Ult11725:0.00369)0.666.13:5.3E-4)0.924.77:5.3E-
4,Ult11728:0.0274)0.377.9:0.01083)0.992.61:5.2E-
4,((Ult11721:0.00742,(UltVe101:0.0037,Ult11720:0.00749)0.780.74:0.00372)0
.516.4:5.4E-4,((Ult11719:0.0231,Ult11717:0.01906)0.432.14:5.4E-
4,Ult11718:0.01505)0.914.89:0.00744)0.905.73:0.00744)0.812.44:5.4E-
4,Ult11724:0.03109)0.870.57:0.00746)0.898.93:0.00736)0.762.72:0.0079,(Ult
Xiph6:0.02077,(Ult11552:0.00434,(Ult11551:0.00267,(Ult11550:0.00369,(Ult1
1548:0.0,Ult11549:0.0):5.5E-
4)0.966.77:0.01638)0.036.5:0.01207)0.997.72:0.06803)0.500.9:0.02487)0.857
.76:0.01531,(((Ult11608:0.02786,UltVe106:0.06062)0.824.46:0.01017,(Ult117
22:0.01144,(((((Ult11760:0.00543,(SpaBacte:0.00617,((Ult11758:0.01897,(Ul
tVe113:5.4E-
4,(Ult11762:0.01076,(Ult11763:0.02544,(Ult11759:0.01291,Ult11750:0.01378)
0.384.9:0.00369)0.770.73:0.00879)0.780.75:0.00458)0.559.7:5.4E-
4)0.891.64:0.00743,Ult11761:5.5E-
4)0.823.44:0.00922)0.959.67:0.03009)0.399.10:0.01568,(Ult11774:0.00402,((
Ult11775:5.5E-4,(Ult11777:0.02263,UltVe116:5.5E-
4)0.852.71:0.00367)0.175.3:5.4E-4,((Ult11773:5.5E-
4,(Ult11776:0.00371,Ult11778:0.0037)0.785.62:5.5E-4)1.000.535:5.4E-
4,Ult11772:0.06058)0.838.64:0.00362)0.780.76:0.00341)0.966.78:0.0254)0.94
9.68:0.03331,(UltXiph5:0.02722,(Ult11610:0.01881,Ult11733:5.5E-
4)0.765.45:0.004)0.992.62:0.03843)0.608.14:0.00342,((((Ult11755:5.4E-
4,((Ult11751:0.00369,Ult11752:5.5E-4)0.999.109:5.4E-
4,Ult11753:0.02694)0.895.91:0.01504)0.877.91:0.00732,((UltEub19:5.4E-
4,(Ult11756:0.00366,Ult11757:0.01514)0.934.71:0.01131)0.978.45:0.019,Ult1
1754:0.00366)0.143.6:5.4E-
4)0.990.59:0.05815,(CanXiph3:0.02831,(CanXiph2:0.01643,CanXiphi:0.01451)0
.124.6:0.01521)0.961.60:0.04777)0.897.70:0.03203,((Ult11780:0.00429,Ult11
781:0.00701)0.996.61:0.03188,(UltXiph7:0.02099,Ult11779:0.04458)0.776.64:
5.4E-
4)0.877.92:0.01801)0.800.48:0.01138)0.935.71:0.02168,((((Ult11668:0.00747
,((((UltVer77:5.5E-
4,Ult11653:0.00369)0.921.91:0.00209,(((Ult11640:0.00743,((UdnBac45:0.0,Ul
tXiph2:0.0,Ult11639:0.0):5.5E-4,Ult11637:0.00369)0.945.66:5.5E-
4)0.879.76:0.00791,Ult11746:0.03951)0.840.69:0.00716,(Ult11646:0.00747,((
Ult11625:0.0,UltOr156:0.0):5.5E-4,Ult11649:0.01134)0.220.5:5.4E-
4)0.881.88:0.00763)0.850.80:0.00705)0.914.90:0.00206,((Ult11619:0.0037,Ul
tVer86:0.0037)0.863.83:5.5E-
4,(Ult11650:0.0139,UltVer82:0.02251)0.897.71:0.0141)0.948.74:0.01505)0.97
6.40:5.4E-
4,((Ult11601:0.0,Ult11647:0.0,Ult11648:0.0,UltrS126:0.0,UltVer89:0.0,UltV
er90:0.0,Ult11654:0.0,Ult11655:0.0):5.5E-
4,(Ult11605:0.00366,(((UltVer78:5.5E-4,(Ult11651:0.0,UltVer88:0.0):5.5E-
4)0.000.557:5.5E-4,(((UltrS124:0.01504,((Ult11679:5.4E-
4,(Ult11626:0.00752,(Ult11636:0.00369,Ult11678:5.5E-
4)0.857.77:0.00752)0.965.56:0.01537)0.963.63:0.0074,(UltrS128:5.4E-
4,(Ult11616:5.5E-4,Ult11572:0.00369)0.836.46:0.00369)1.000.536:5.4E-
4)0.883.87:0.015)0.969.49:5.5E-
4,Ult11674:0.00744)0.923.104:0.00742,(Ult11630:5.4E-
4,((((Ult11602:0.0,Ult11603:0.0,UltVer81:0.0):5.5E-
4,Ult11634:0.00742)1.000.537:5.4E-4,((UltVer85:0.0,Ult11641:0.0):5.5E-
4,Ult11642:0.01125)0.915.78:0.00361)0.504.6:0.00743,(((Ult11614:0.00369,U
lt11635:0.01506)0.891.65:5.5E-
4,(Ult11633:0.00371,UltVer83:0.00369)0.737.47:5.5E-4)0.000.558:5.5E-

4,(UltOr155:0.0,Ult11631:0.0,Ult11632:0.0,UltVer80:0.0,Ult11638:0.0):5.5E
-4)1.000.538:5.3E-4)0.916.89:0.00361)0.952.56:0.00741)0.954.66:5.5E-
4)0.919.96:5.3E-4,Ult11652:0.00739)0.412.6:0.00369)1.000.539:5.0E-
4)0.446.7:5.5E-4)0.887.83:0.00747)0.785.63:0.00373,(((Ult11558:5.5E-
4,(((Ult11615:0.0,Ult11618:0.0,Ult11560:0.0,Ult11571:0.0):5.5E-
4,Ult11567:5.5E-4)0.000.559:5.5E-
4,((Ult11617:0.0,Ult11561:0.0):0.00369,(Ult11562:0.00369,(Ult11563:0.0036
9,(Ult11564:0.0,Ult11573:0.0):5.5E-4)0.717.22:5.5E-
4)0.968.62:0.01127)0.135.6:5.5E-
4)0.861.79:0.00369)0.887.84:0.00372,(((Ult11628:5.5E-
4,(Ult11627:0.01511,(((Ult11613:0.0,Ult11657:0.0,UltVer91:0.0):5.5E-
4,((Ult11664:0.0,Ult11665:0.0):0.00369,Ult11660:0.00369)0.691.12:5.5E-
4)0.351.10:5.5E-4,Ult11659:0.00369)0.839.65:5.5E-
4)0.856.61:0.00369)0.773.60:0.00377,UltVer76:0.02317)0.453.8:0.00371,((Ul
t11663:0.0075,(Ult11669:0.01114,(Ult11670:5.5E-
4,Ult11671:0.0074)0.610.5:5.5E-
4)0.758.65:0.00374)0.906.93:0.00748,((Ult11662:0.01117,(UltOr154:5.5E-
4,(Ult11590:0.0037,((Ult11554:0.0,Ult11556:0.0,Ult11557:0.0,Ult11568:0.0,
Ult11569:0.0,Ult11570:0.0,UltVer75:0.0):5.5E-
4,(Ult11559:0.00741,Ult11555:0.00369)0.679.16:5.5E-4)0.885.69:5.5E-
4)0.854.73:0.00369)0.790.62:0.00377)0.383.8:0.00758,((Ult11587:0.00367,((
Ult11585:0.0,Ult11586:0.0):5.5E-4,(Ult11645:0.01115,((Ult11658:5.5E-
4,(((Ult11581:5.5E-4,(((Ult11533:0.00369,(Ult11522:5.5E-
4,(UltrS123:0.00369,Ult11594:0.00369)0.678.12:5.5E-4)0.674.13:5.5E-
4)0.844.75:0.00361,(((((Ult11595:0.0,Ult11596:0.0,UltOr153:0.0,UltVer71:0
.0):5.4E-4,((Ult11510:0.0,Ult11534:0.0):5.5E-
4,(Ult11514:0.01495,Ult11736:0.02265)0.390.10:5.5E-
4)0.848.63:0.00369)0.867.70:0.00369,(((UltrS122:0.00372,((Ult11588:0.0,Ud
nEuba4:0.0):5.5E-4,Ult11515:0.00369)0.038.4:5.4E-
4)0.844.76:0.00369,((Ult11565:0.00744,((Ult11543:0.00369,((((Ult11521:0.0
0745,(Ult11541:0.00369,((Ult11545:0.00747,Ult11544:0.00369)0.746.40:5.5E-
4,(Ult11542:0.0,Ult11767:0.0):5.5E-4)0.809.50:5.5E-4)0.760.55:5.3E-
4)0.849.63:0.00371,(Ult11511:0.015,Ult11535:0.00407)0.882.81:5.4E-
4)0.000.560:5.5E-4,Ult11566:0.00744)0.374.9:5.5E-4,((Ult11530:5.5E-
4,Ult11539:0.00369)0.873.79:0.00361,((Ult11509:0.00369,Ult11546:5.5E-
4)0.875.78:0.00361,((Ult11536:0.0,Ult11540:0.0,Ult11547:0.0):0.00372,UltV
er72:0.0037)1.000.540:5.3E-4)0.104.8:0.0037)0.998.86:5.5E-
4)0.000.561:5.5E-4)0.456.8:5.5E-4,Ult11538:0.00739)0.000.562:5.5E-
4)0.410.7:5.5E-
4,(Ult11591:0.0,Ult11592:0.0,Ult11512:0.0,Ult11516:0.0,Ult11520:0.0,Ult11
531:0.0,Ult11532:0.0,Ult11553:0.0,UltVer74:0.0):5.5E-4)0.000.563:5.5E-
4)0.371.10:5.5E-4,Ult11593:0.00369)0.000.564:5.4E-4)0.457.9:5.5E-
4,Ult11735:0.00369)0.384.10:5.5E-
4,(Ult11526:0.00362,((((Ult11518:0.0304,Ult11523:0.00209)0.990.60:0.00171
,Ult11537:0.01117)0.193.4:5.5E-
4,(((Ult11589:0.0,Ult11513:0.0,Ult11508:0.0,UltXiphi:0.0,Ult11519:0.0,Ult
11524:0.0,Ult11525:0.0,Ult11734:0.0):5.5E-
4,(Ult11600:0.00369,(Ult11527:5.3E-
4,Ult11582:0.00369)0.832.55:0.00369)0.910.77:5.5E-4)0.000.565:5.5E-
4,(Ult11517:0.00369,UltVer69:0.00369)0.692.8:5.5E-4)0.919.97:5.3E-
4)1.000.541:5.4E-4,(Ult11529:0.00371,((Ult11528:0.0,UltVer70:0.0):5.5E-
4,UltVer73:0.00369)0.406.7:5.5E-
4)0.884.81:0.00362)0.413.12:0.00368)0.994.64:5.5E-4)1.000.542:5.4E-
4)0.068.5:0.00393,((Ult11577:0.01113,(Ult11597:0.00369,(Ult11621:5.5E-

4,((Ult11598:0.0,Ult11612:0.0,Ult11576:0.0,Ult11578:0.0):5.5E-
4,(Ult11579:5.5E-
4,((Ult15402:0.0747,Ult15403:0.05606)0.833.62:0.02276,Ult11580:0.01399)0.
366.7:0.00504)0.922.103:0.00346)0.000.566:5.5E-4)0.291.8:5.4E-
4)0.862.70:5.5E-
4)0.913.80:0.00747,Ult11583:0.00376)0.816.49:0.00361)1.000.543:5.5E-
4)0.127.2:5.5E-
4,Ult11575:0.00743)0.818.35:0.00395,Ult11574:0.00369)0.798.56:0.00395)0.9
19.98:0.00791,UltVer87:0.00369)0.107.12:5.4E-4)0.372.7:5.5E-
4)0.412.7:5.5E-4)0.280.3:5.5E-
4,(Ult11622:0.00744,((Ult11673:0.00371,Ult11620:0.01897)0.771.69:0.00369,
(Ult11606:0.0,Ult11584:0.0,Ult11680:0.0):5.5E-4)0.742.35:5.3E-
4)0.851.72:0.0037)0.919.99:0.00374)1.000.544:5.4E-
4)0.919.100:0.00792)0.841.61:5.5E-4)0.898.94:0.00786,(Ult11656:5.4E-
4,((Ult11676:0.01124,(UltVer79:0.00369,(Ult11629:5.5E-
4,(Ult11623:0.00369,UltrS125:0.00369)0.667.16:5.5E-4)0.941.69:5.5E-
4)0.894.70:5.5E-
4)0.892.67:0.00759,((UcvtSoil:0.0151,((Ult11624:0.0,Ult11667:0.0,UltrS127
:0.0):5.3E-4,Ult11666:0.01119)0.650.9:0.0037)0.751.33:5.5E-
4,(Ult11677:5.3E-
4,Ult11672:0.01539)0.583.6:0.00744)0.902.85:0.00748)0.871.73:0.00765)0.93
4.72:0.00748)0.928.63:5.5E-4)0.549.11:5.4E-
4,UltVe114:0.01492)0.780.77:0.00398,(UltVe105:0.02311,(Ult11675:5.5E-
4,((UltVer84:0.0,Ult11644:0.0,Ult11661:0.0):0.00751,Ult11643:0.00749)0.87
8.74:0.00738)0.844.77:0.00756)0.736.33:0.00367)0.881.89:0.01449)0.605.11:
0.00994)0.958.76:0.02608)0.830.49:0.01199,(UltVe108:0.05269,(Ult11723:0.0
4149,((UltVe115:0.00499,(Ult11766:0.02388,(Ult11764:0.01895,Ult11765:0.01
897)0.280.4:5.4E-
4)0.618.4:0.01054)0.838.65:0.01117,(((Ult11768:0.0,Ult11769:0.0,Ult11770:
0.0):0.00372,(Ult11609:0.03093,(UltrS129:0.00395,UltrS130:0.00748)0.655.1
9:5.4E-
4)0.795.54:0.00392)0.820.34:0.01008,Ult11771:0.07737)0.878.75:0.01862)0.9
38.49:0.01756)0.789.53:0.007)0.889.73:0.01198)0.987.72:5.4E-
4)0.763.68:0.00291,((UltVe109:0.06002,(UltVe112:0.01173,(UltVe111:5.5E-
4,(UltVe110:5.5E-4,(Ult11748:5.5E-
4,Ult11749:0.00741)0.847.83:0.0037)0.861.80:0.00371)0.659.11:0.00342)0.66
3.14:0.02639)0.880.82:0.02088,(Ult11731:0.03056,Ult11732:0.03056)0.918.76
:0.0164)0.861.81:0.01442)0.894.71:0.01256)0.659.12:0.00439)0.849.64:0.011
44)0.874.90:0.01154)0.833.63:0.01314)0.707.15:0.02004)0.992.63:0.07312,((
Ult12327:0.04593,((((Ult12356:0.05505,(Ult12330:0.02523,(Ult12328:0.02537
,Ult12326:0.04905)0.110.6:0.02172)0.901.81:0.0199)0.910.78:0.01882,((((Ul
tVe221:0.03793,Ult12343:0.03399)0.795.55:0.00818,((((UltVe230:0.00815,Ult
Ve232:0.106)0.788.60:0.02373,(Ult12341:0.00737,(UltrS145:0.03304,UltEub21
:0.04046)0.779.64:0.00689)0.887.85:0.01105)0.594.8:0.00457,(Ult12342:0.03
286,((Ult12354:0.02318,Ult12353:0.05802)0.862.71:0.01672,Ult12345:0.02521
)0.829.61:0.01351)0.609.12:0.00718)0.882.82:0.01144,(((UltVe225:0.02193,(
UltPl546:0.02661,(UltPl545:0.03748,UltOr169:0.07474)0.534.8:0.02011)0.883
.88:0.01937)0.714.21:0.00877,UltMar60:0.0506)0.750.38:0.0054,(Ult12347:0.
01489,((UdnBac47:0.04784,UltVe226:0.01534)0.856.62:0.00735,(Ult12324:0.01
9,Ult12325:0.0117)0.443.5:0.00765)0.855.70:5.3E-
4)0.786.63:0.00393)0.789.54:0.00351)0.784.58:0.00415)0.761.54:0.0047,((((
UltVe213:0.04176,UltVe231:0.02216)0.640.8:0.00136,UltOr170:0.06592)0.923.
105:0.02819,((UltVe227:0.02655,Ult12355:0.03712)0.742.36:0.00666,Ult12352
:0.04738)0.790.63:0.01105)0.814.38:0.0095,((((Ult12333:0.09438,Ult12344:0

.02073)0.123.4:0.00302,((UltVe214:0.05283,Ult12332:0.02869)0.803.43:0.008
09,UltVe222:0.02369)0.747.43:0.00464)0.899.79:0.01023,(Ult12331:0.03554,(
(UltVe215:0.00587,UltVe220:0.0679)0.927.77:0.01786,((Ult12334:0.03352,((U
ltVe217:0.01729,Ult12337:0.04691)0.672.11:0.01017,(UltVe218:5.5E-
4,UltVe219:0.00369)0.978.46:0.03029)0.900.66:0.01382)0.789.55:0.00584,Ult
12357:0.03052)0.287.6:0.00373)0.892.68:0.01446)0.781.55:5.3E-
4)0.327.7:0.00735,(((Ult12335:0.02238,UltVe216:0.03155)0.961.61:0.02764,U
lt12336:0.02528)0.836.47:0.00993,(UltVe224:0.00367,Ult12340:0.07218)0.858
.73:0.01633)0.065.9:0.00759)0.781.56:0.00821)0.867.71:0.00892)0.803.44:0.
00865,(UltVe223:0.03635,(Ult12339:0.03595,(Ult12338:0.08535,((UltVe229:0.
02313,(Ult12349:0.03721,Ult12350:0.04015)0.953.56:0.02922)0.746.41:0.0051
4,((Ult12348:0.05093,Ult12351:0.07137)0.731.22:0.0064,UltVe228:0.06958)0.
659.13:0.0106)0.873.80:0.01804)0.217.7:0.01479)0.871.74:0.02299)0.807.42:
0.01097)0.869.68:0.01153)0.855.71:0.00874,((((((UltVe239:5.4E-
4,UltVe238:0.0268)0.669.11:0.01275,((((Ult12420:0.02074,Ult12421:0.0534)0
.733.27:0.0113,(UltVe241:0.00722,UltVe242:5.5E-
4)0.925.92:0.01948)0.755.49:0.00552,Ult12422:0.00672)0.768.44:0.0067,(Ult
12419:0.03002,UltVe240:0.02949)0.157.9:0.00724)0.920.91:0.02146)0.658.8:0
.01089,(Ult12417:0.11182,UltVe245:0.02835)0.673.7:0.01364)0.848.64:0.0119
8,((Ult12418:0.03494,((UltrS146:0.00751,(Ult12409:0.06668,Ult12410:0.0159
2)0.863.84:0.015)0.735.32:0.00403,(Ult12413:0.01981,Ult12411:0.06147)0.21
4.6:0.00347)0.876.68:0.01228)0.452.11:0.01132,(((UltVe246:0.05683,UdnBac4
8:0.01305)0.848.65:0.01284,(UltVe234:0.05718,(((UltVe235:0.02974,Ult12407
:0.02763)0.943.67:0.03161,(UltPl548:0.06827,Ult12406:0.03318)0.131.4:0.01
418)0.949.69:0.03661,Ult12404:0.011)0.820.35:0.01393)0.862.72:0.01741)0.8
92.69:0.02011,(Ult12405:0.02272,((Ult12416:0.00411,UltVe237:0.01113)0.986
.47:0.05831,(Ult12414:0.01565,UltVe236:0.00742)0.998.87:0.06957)0.723.23:
0.03465)0.781.57:0.01182)0.946.68:0.02593)0.789.56:0.00694)0.892.70:0.018
04,((Ult12423:0.02829,(((Ult12425:0.03138,Ult12451:0.05752)0.891.66:0.020
74,(UltVe244:0.01452,Ult12426:0.03416)0.734.36:0.00435)0.859.67:0.01038,U
ltVe243:0.01304)0.924.78:0.01767)0.817.34:0.01908,(UltVe233:0.03543,Ult12
424:0.053)0.877.93:5.3E-
4)0.745.50:0.00884)0.940.75:0.01555,((Ult12412:0.03766,(Ult12329:0.04299,
Ult12408:0.04038)0.789.57:0.01624)0.797.48:0.01318,((Ult12386:0.00368,Ult
12400:5.5E-4)0.999.110:0.09236,((Ult12384:0.02014,(((((Ult12390:5.5E-
4,(Ult12358:5.5E-4,(Ult12373:0.0,Ult12381:0.0):5.5E-4)0.000.567:5.5E-
4)0.778.63:5.5E-
4,Ult12391:0.00369)0.916.90:0.01142,((Ult12372:0.0,Ult12359:0.0,Ult12402:
0.0):5.5E-4,(Ult12371:5.5E-
4,Ult12370:0.04772)0.810.50:0.00371)0.966.79:0.01533)0.718.18:0.00394,(((
Ult12376:0.0,Ult12378:0.0,Ult12380:0.0,Ult12389:0.0,Ult12401:0.0):5.5E-
4,(Ult12379:0.00369,Ult12382:0.00372)0.816.50:5.2E-4)1.000.545:5.5E-
4,(Ult12396:0.03181,Ult12397:0.00321)0.906.94:0.01951)0.941.70:0.01123)0.
745.51:0.00381,((Ult12374:0.0,Ult12375:0.0):5.4E-4,(((Ult12392:5.5E-
4,((Ult12367:0.00369,Ult12393:0.00369)0.927.78:5.5E-
4,(Ult12363:0.0,Ult12365:0.0,Ult12366:0.0,Ult12387:0.0):5.5E-
4)0.000.568:5.5E-4)0.927.79:5.5E-
4,(Ult12364:0.01113,((Ult12360:0.0,Ult12361:0.0,Ult12362:0.0,UltPl547:0.0
):5.5E-4,Ult12395:0.00369)0.909.66:0.00716)0.912.100:5.3E-
4)0.890.84:0.00742,(Ult12383:0.0,Ult12394:0.0):0.0038)0.411.8:0.00373)0.9
37.71:0.01129)0.908.89:0.01493)0.758.66:0.00454,Ult12385:0.02018)0.000.56
9:0.00275)0.994.65:0.06658)0.536.9:5.4E-
4)0.753.60:0.00516)0.675.7:0.00416,((((Ult12377:0.01757,UltVe253:0.08389)
0.844.78:0.01767,(Ult12443:0.02729,(UltVe254:0.00369,((Ult12442:0.0,Ult12

444:0.0):5.5E-4,Ult12441:0.01129)0.835.59:0.00369)0.853.61:5.5E-4)0.955.52:0.0213)0.933.79:0.02373,((UltVe255:0.00485,((Ult12434:0.00367,(((Ult12437:0.00367,Ult12438:5.4E-4)0.929.70:0.01464,(Ult12439:5.4E-4,(((Ult12428:0.0205,((UltMar61:0.00748,(((UltVe247:5.3E-4,Ult12440:0.03115)0.515.5:0.00368,Ult12430:5.4E-4)0.990.61:0.00183,Ult12433:0.03109)0.664.9:0.00203)0.883.89:0.01092,((UltMar62:0.02297,UltVe252:5.5E-4)0.721.15:0.00743,((UltVe248:5.5E-4,(Ult12432:0.00715,Ult12431:0.08124)0.855.72:5.4E-4)0.871.75:0.00366,Ult12435:5.5E-4)0.745.52:0.00358)0.751.34:5.5E-4)0.800.49:0.0155)0.812.45:0.00676,UltVe249:5.4E-4)0.813.50:0.00359,Ult12429:0.00751)0.935.72:0.01113)0.924.79:0.01428)0.865.80:0.00934,(UltVe250:0.00745,UltVe251:5.5E-4)0.767.56:0.00378)0.886.98:0.00747)0.907.74:0.01139,Ult12436:0.00351)0.999.111:0.05926)0.952.57:0.02452,(Ult12445:0.01885,Ult12446:5.3E-4)0.238.6:0.00901)0.886.99:0.01878)0.997.73:0.05675,(Ult12427:0.12444,((Ult12447:0.04286,Ult12448:0.01514)0.973.43:0.03745,(Ult12449:0.06948,Ult12450:0.03423)0.728.28:0.02097)0.799.63:0.0129)0.190.9:0.01986)0.746.42:0.00902)0.950.65:0.03233)0.928.64:0.03617,((Ult12256:0.11293,((((Ult12078:0.0229,(UltVe162:0.0281,(UltVe163:0.00375,(Ult12077:0.00374,Otu00044:5.4E-4)0.770.74:0.00373)0.701.17:0.00323)0.810.51:0.01461)0.972.63:0.03164,(UltVe160:0.01953,((UltOpit3:0.01974,(UltVe161:0.00372,Otu00115:0.00371)0.747.44:0.00336)0.979.55:0.01988,(UltOpit2:0.04896,Otu00389:0.00745)0.853.62:5.5E-4)0.885.70:0.01135)0.879.77:0.01742)0.996.62:0.06544,(((((VrcBact5:0.00301,(CerArena:0.00797,VrcBact4:0.01096)0.912.101:0.0118)0.981.51:0.03491,((((Ult12089:0.00369,Ult12091:5.5E-4)0.999.112:5.3E-4,((Ult12090:0.00789,(Ult12085:0.00205,(Ult12084:0.01496,(Ult12086:0.00749,Ult12087:0.0037)0.595.5:5.5E-4)0.994.66:0.00175)0.994.67:0.03188)0.927.80:0.00405,(Ult12088:0.01107,UltCrat7:0.01988)0.961.62:0.02338)0.919.101:0.00359)0.929.71:0.01963,((Ult12081:5.5E-4,Ult21418:0.03854)0.998.88:0.04503,(((UltVe171:0.03759,((((Otu00155:0.00411,MstBtBt2:0.03439)0.880.83:0.00372,Z0114633:0.00747)0.473.8:5.4E-4,Sgfffff2:0.00369)0.755.50:0.00529,((UltVe168:0.01943,Otu00097:0.01441)0.515.6:5.3E-4,(UltVe169:0.01083,UltVe170:0.03091)0.508.6:5.4E-4)0.926.91:0.01496)0.898.95:0.02127)0.980.44:0.04234,Ult12083:5.4E-4)0.889.74:0.01117,Ult12082:0.00369)0.875.79:0.01267)0.942.71:0.02698)0.974.41:0.04699,(Ult12093:0.04243,Ult12092:0.07988)0.744.41:0.03597)0.681.9:0.00401)0.976.41:0.03108,((MaiMet16:0.07963,((UltVe185:0.05073,(Ult12126:5.5E-4,(Ult12127:0.0,Ult12128:0.0):5.5E-4)0.981.52:0.05021)0.975.52:0.03587,((UltVe186:0.00855,((UdnBac46:5.5E-4,PunVermi:0.02283)0.888.88:0.01497,Ult12129:0.02574)0.457.10:0.01139)0.999.113:0.08114,((((((Ult12106:5.5E-4,(Ult12105:5.5E-4,Ult12096:0.00371)0.454.13:5.5E-4)0.871.76:5.4E-4,UltVe174:0.01876)0.468.12:0.00377,UltVe175:0.00333)0.943.68:0.01163,Ult12104:5.4E-4)0.845.75:0.01142,(Ult12107:0.0,UltVe176:0.0):0.01932)0.752.57:0.00677,((((UltMar59:0.00369,(UltMar58:0.0037,((Ult12112:0.0037,(Ult12110:0.0037,(Ult12111:5.5E-4,(UltMar57:5.5E-4,Ult12108:5.5E-4)0.773.61:5.5E-4)0.952.58:0.0074)0.109.7:5.4E-4)0.961.63:0.0111,(Ult12109:5.5E-4,Ult12114:0.01122)0.245.5:5.4E-4)0.902.86:0.00707)0.892.71:5.4E-4)0.948.75:0.00483,Ult12113:0.00505)0.947.58:0.00481,UltVe179:5.3E-4)0.966.80:0.02032,((UltVe178:0.08836,(Ult12101:0.01129,(Ult12100:5.4E-4,(UltVe177:0.00739,(Ult12095:5.5E-4,(Ult12103:0.00369,((Ult12098:5.5E-

4,Ult12099:0.00744)0.841.62:0.0037,(Ult12097:5.5E-4,UltVe173:5.5E-
4)0.867.72:0.00372)0.619.4:5.5E-4)0.480.7:5.5E-4)0.963.64:5.5E-
4)0.859.68:0.00367)0.630.9:5.3E-4)0.000.570:5.4E-
4)0.133.5:0.00466,Ult12102:0.01815)0.819.45:0.00777)0.828.47:0.01051)0.98
5.56:0.04803)0.777.62:0.01276)0.034.6:0.00413)0.922.104:0.01819,(((EpxnsO
f0:0.0541,((Ult12133:0.03726,(UltVe195:0.00364,UltVe196:0.00754)0.918.77:
0.02143)0.950.66:0.02464,((UltVe183:0.0,UltVe184:0.0):0.00688,((Ult12122:
5.5E-4,(Ult12125:5.4E-4,(CrgAkaji:5.4E-
4,((Ult12119:0.0,Ult12123:0.0):5.5E-
4,Ult12124:0.00741)0.813.51:0.00368)0.951.67:0.01513)0.997.74:0.03059)0.2
99.11:0.0041,((Ult12117:0.01652,Ult12120:0.0101)0.889.75:0.01145,((UltOpi
t8:0.00367,UltVe182:0.00374)0.796.46:0.00363,((Ult12118:0.0037,Ult12121:5
.5E-4)0.894.72:0.00748,((UltVe180:0.0,Udntdde3:0.0,UltVe181:0.0):5.5E-
4,(Ult12116:0.00369,UltGa291:0.0112)0.766.56:0.00369)0.914.91:0.01124)0.8
74.91:0.00761)0.949.70:0.01848)0.758.67:0.00432)0.893.82:0.01778)0.843.55
:0.01811)0.809.51:0.0113)0.954.67:0.02433,(((Ult12115:0.01985,(Ult12132:0
.02213,UltVe194:0.02564)0.995.74:0.05954)0.741.43:0.01052,(UltVe172:0.032
94,(Ult12094:0.06997,(UltVe167:0.05117,(Ult12079:0.0,Otu00651:0.0):0.0583
1)0.952.59:0.03956)0.811.48:0.01668)0.725.24:0.02458)0.238.7:0.02129,UltO
r164:0.05135)0.836.48:0.01013)0.609.13:5.4E-
4,UltVe189:0.04442)0.951.68:0.01849)0.733.28:0.00372)0.977.50:0.04257,(((
UltVe188:0.02528,(Ult12130:0.00371,UltVe187:0.02334)0.792.68:0.00952)0.32
2.10:0.0181,(((UltOpit7:0.0,Otu00371:0.0):0.02958,UltVe166:0.01825)0.996.
63:0.08744,(UltGa295:0.07765,(((PgcLitor:0.0072,MaiBac17:0.02364)0.332.6:
0.01582,(VrbBact2:5.5E-4,(PgcAlbus:0.0037,PgcCroce:0.0037)0.760.56:5.5E-
4)1.000.546:5.4E-4)0.870.58:0.00725,PgcMobil:5.4E-
4)0.733.29:0.00701)0.983.43:0.05234)0.776.65:0.02161)0.762.73:0.01194,((O
piSpeci:0.03772,UltOpit9:0.03362)0.972.64:0.0473,(UltVe193:0.0282,(UltGa2
92:0.01671,(UltOr167:0.00795,(((UltVe190:0.0192,(UltVe191:0.0075,UltVe192
:0.02301)0.738.26:0.00393)0.801.60:0.00352,Ult12131:5.4E-
4)0.752.58:0.00317,(UltOr165:0.01376,(UltPro57:0.02073,UltOr166:0.01386)0
.065.10:0.00348)0.781.58:0.0058)0.903.74:0.01176)0.877.94:0.01142)0.834.4
6:0.01103)0.935.73:0.03972)0.930.69:0.035)0.378.8:0.01846)0.909.67:0.0196
2,(Otu00489:0.0361,((Otu00232:0.02663,(UltOpit5:0.00596,UltOpit6:0.07245)
0.728.29:0.0065)0.967.50:0.02923,((UltVe164:0.00122,Otu00423:0.00282)0.99
0.62:0.03799,((UltOpit4:0.01127,Otu00124:5.5E-
4)0.368.6:0.00475,(Otu00313:0.0103,UltVe165:0.02142)0.397.8:0.00658)0.759
.69:0.00658)0.948.76:0.0292)0.874.92:0.01445)0.834.47:0.00996)0.854.74:0.
01856)0.734.37:0.01024,((((Ult12178:5.4E-
4,(Ult12177:0.00835,(AleAgaro:0.02641,UltVe204:0.03276)0.689.15:0.01566)0
.808.52:0.00658)0.913.81:5.5E-4,((Ult12175:0.0,Ult12179:0.0):5.5E-
4,Ult12176:0.00369)0.864.75:0.00724)0.895.92:0.02859,(((((((OptBacte:0.01
435,(UltGramp:0.02966,Ult12136:0.02174)0.978.47:0.02871)0.760.57:0.00444,
(Ult12162:0.01126,Ult12163:5.5E-
4)0.943.69:0.01581)0.958.77:0.01985,(Ult12137:0.0176,(Ult12134:0.01376,(U
ltOpi11:0.00369,(UltOpi12:0.0,UltOpi10:0.0):5.5E-
4)0.960.73:0.02147)0.913.82:0.01558)0.882.83:0.00974)0.866.71:0.0078,(((U
lt12167:0.01303,(UltPro58:0.00756,(UltVe203:0.02306,(Ult12166:5.4E-
4,((UltVe202:0.0,UltrS144:0.0):5.5E-
4,Ult12165:0.05538)0.346.11:0.01122)0.858.74:0.00753)0.883.90:0.01132)0.8
90.85:0.01318)0.879.78:0.01209,Ult12168:5.4E-
4)0.947.59:0.01103,(Ult12173:0.01123,(Ult12172:0.0575,((Ult12139:0.002,(U
lt12138:0.00378,UltOpi13:0.0191)0.887.86:0.00181)0.911.96:0.01452,(Ult121
80:0.02249,(Ult12141:0.00826,Ult12140:0.0225)0.894.73:0.01199)0.886.100:0

.01249)0.925.93:0.01644)0.759.70:0.00384)0.807.43:0.00744)0.898.96:5.4E-
4)0.851.73:0.00722,((OptBact2:0.0,Otu00258:0.0):0.02287,(((UltOpi14:5.5E-
4,((((((Ult12152:0.01915,Ult12159:0.00747)0.927.81:5.4E-
4,(Ult12153:0.02271,((Ult12143:0.0191,(UltGa293:0.04815,(Ult12146:0.00754
,Ult12142:0.0151)0.956.65:0.00389)0.953.57:0.00375)0.874.93:0.00758,(UltV
e200:0.01116,((UltVe198:5.3E-4,((Ult12145:5.5E-4,(Ult12135:5.4E-
4,(Ult12155:0.00741,UltOpi15:0.00762)0.965.57:0.01532)0.443.6:0.00368)0.9
61.64:0.01127,((UltEnt16:0.01557,UltVe199:0.00352)0.872.87:0.00784,Ult121
44:0.0038)0.786.64:0.00361)0.968.63:5.4E-4)0.688.11:5.4E-
4,(Ult12157:0.00361,(Ult12156:5.4E-
4,Ult12158:0.00361)0.510.6:0.00754)1.000.547:5.5E-
4)0.884.82:0.00743)0.231.7:5.5E-4)0.840.70:0.00367)0.906.95:5.3E-
4)0.951.69:0.0074,(OpiSpec3:0.0037,(Ult12154:5.5E-4,(((UltrS142:5.5E-
4,Ult12150:0.00748)0.800.50:0.00368,(Ult12149:0.01525,UltVe197:0.0037)0.2
05.6:5.3E-
4)0.858.75:0.01509,(VrbBacte:0.00541,(OpiTerra:0.0037,OpiSpec2:5.5E-
4)0.885.71:0.01015)0.963.65:0.0225)0.988.64:5.5E-4)0.600.9:5.4E-
4)0.863.85:0.00376)0.354.6:5.4E-
4,Ult12151:0.00369)0.959.68:0.01137,Ult12148:5.4E-4)0.946.69:5.4E-
4,(Ult12147:0.00511,UltGa294:0.11736)0.753.61:0.00989)0.836.49:0.00372)0.
967.51:0.0115,Ult12160:5.4E-
4)0.926.92:0.00755,UltrS143:0.01128)0.855.73:5.5E-
4)0.827.53:0.00768)0.875.80:0.01216,(Ult12164:0.03904,Ult12174:0.01701)0.
676.7:0.00797)0.894.74:0.01612,((Ult12169:0.00345,Ult12170:5.5E-
4)0.728.30:0.01126,Ult12171:0.10514)0.682.14:0.01857)0.838.66:0.02567)0.8
55.74:0.02166,(Ult12161:0.02386,UltVe201:0.00383)0.765.46:0.00738)0.981.5
3:0.04859)0.984.47:0.06884)0.923.106:0.04251,((Ult12323:0.06846,Ult12346:
0.07811)0.857.78:0.03001,(((UltVe211:0.07275,(UltVe210:0.04565,UltVe212:0
.06298)0.296.6:0.04545)0.946.70:0.04295,(UltVe209:0.06707,((Ult12322:0.04
646,Ult12321:0.04288)0.995.75:0.07487,(UltB4505:0.01554,(UltEub20:0.00197
,(Ult12320:0.00694,Ult12319:0.00207)0.506.9:5.9E-
4)0.846.86:0.00662)0.814.39:0.00795)0.775.66:0.00907)0.984.48:0.05905)0.6
66.14:0.02785,(((((UltVe206:5.5E-
4,UltVe207:0.0075)0.982.47:0.03751,UltVe208:0.04203)0.365.7:0.0055,Ult123
13:0.08683)0.372.8:0.01177,Cuiiii21:0.03437)0.918.78:0.0191,((((((((((Ul
tRu277:0.02495,UltRu276:0.18986)0.769.58:0.00992,((UltRu279:0.0287,UltRu2
80:0.00622)0.807.44:0.01196,(UltRu281:0.01142,(Ult12217:0.0,Ult12218:0.0)
:0.00757)0.821.45:5.4E-
4)0.881.90:0.01343)0.908.90:0.01362,(((UltRu278:0.01637,(Ult12249:0.0,Ult
12250:0.0):0.01822)0.967.52:0.02428,UltRu282:0.0227)0.825.49:0.00796,((((
Ult12260:0.01874,Ult12261:5.4E-4)0.856.63:5.4E-
4,((Ult12253:0.06185,(Ult12251:0.01519,Ult12252:0.0073)0.949.71:0.0254)0.
920.92:0.02465,Ult12263:0.00747)0.435.10:0.01635)0.919.102:0.0112,(Ult122
58:0.05561,Ult12265:0.0076)0.722.14:0.0037)0.771.70:0.00336,(Ult12269:0.0
2244,(Ult12267:5.5E-4,(Ult12268:0.01877,Ult12266:0.01496)0.503.4:5.3E-
4)0.981.54:5.2E-
4)0.890.86:0.00734)0.847.84:0.00722)0.909.68:0.01059)0.195.4:5.5E-
4,UltRu283:0.09995)0.946.71:0.02026,(UltRu272:0.05715,((UltRu271:5.5E-
4,(Ult12216:0.01622,(Ult12193:0.01462,(Ult12219:0.04192,Ult12276:0.00939)
0.981.55:0.03146)0.820.36:0.00879)0.952.60:0.01908)0.939.66:0.01497,((Ult
Ru275:0.01875,(UltRu273:5.4E-
4,Ult12215:0.01124)0.989.58:0.01924)0.900.67:5.5E-4,UltRu274:5.5E-
4)0.024.4:5.5E-4)0.877.95:5.4E-
4)0.922.105:0.01754)0.722.15:0.00346,Ult12285:0.05597)0.668.14:0.00342,((

(Ult12257:0.00773,Ult12230:0.07057)0.736.34:0.00467,(Ult12262:0.02259,(((
Ult12242:0.01454,(UltProk3:0.00335,Ult12243:0.10284)0.949.72:0.0209)0.854
.75:0.0106,(Ult12247:0.02427,Ult12274:0.04265)0.858.76:0.01617)0.898.97:0
.01603,(((Ult12231:0.03485,((Ult12238:0.10173,(Ult12232:0.01516,Ult12239:
5.5E-4)0.162.5:5.3E-4)0.884.83:0.0067,Ult12236:0.0391)0.925.94:5.4E-
4)0.960.74:5.4E-
4,((Ult12264:0.00366,Ult12270:0.01519)0.971.53:0.02417,((Ult12194:0.02646
,(((Ult12272:0.05304,Ult12273:0.01623)0.893.83:0.01424,Ult12241:5.4E-
4)0.873.81:0.00854,(Ult12240:5.5E-4,(Ult12246:0.0471,(Ult12245:5.5E-
4,(((Ult12233:5.5E-
4,Ult12237:0.02899)0.903.75:0.00805,Ult12271:0.00739)0.046.5:5.5E-
4,Ult12234:0.00368)0.403.9:5.5E-4)0.918.79:5.4E-
4)0.785.64:0.00356)0.922.106:0.01817)0.740.47:0.01001)0.774.74:0.00551,((
Ult12254:0.01495,Ult12255:5.3E-4)0.894.75:5.5E-
4,(((Ult12204:0.0389,(Ult12205:0.00373,(((((((Ult12191:0.05751,Ult12202:0
.05076)0.752.59:0.02366,(((Ult12182:0.0112,Ult12195:5.5E-
4)0.990.63:0.01928,((((((Ult12184:0.0,Ult12189:0.0):5.5E-
4,Ult12190:0.0074)0.141.4:5.5E-
4,Ult12188:0.00742)0.843.56:0.00367,((Ult12186:0.0,Ult12207:0.0):5.5E-
4,Ult12192:0.00368)0.830.50:5.5E-4)0.836.50:0.00363,(Ult12183:5.5E-
4,(Ult12212:0.0113,Ult12221:0.03494)0.751.35:0.00362)1.000.548:5.5E-
4)0.868.62:0.00741,Ult12185:0.00744)0.562.11:5.5E-4)0.334.8:5.5E-
4,Ult12209:5.3E-4)0.919.103:5.4E-
4)0.870.59:0.00719,(Ult12198:0.01111,(Ult12200:0.01127,(Ult12197:5.5E-
4,((Ult12181:0.0,Ult12201:0.0,Ult12214:0.0):0.00369,Ult12208:0.00369)0.67
8.13:5.5E-4)0.884.84:5.5E-4)0.059.8:5.5E-
4)0.849.65:0.00367)0.299.12:5.0E-4,(Ult12210:0.0,Ult12213:0.0):5.5E-
4)0.165.3:5.4E-
4,Ult12196:0.00369)0.835.60:0.00366,Ult12220:0.0424)0.986.48:5.4E-
4,(Ult12203:0.01502,Ult12206:0.00741)0.780.78:0.00369)0.786.65:0.00365)0.
746.43:0.00317)0.877.96:0.00757,Ult12187:0.00392)0.738.27:0.00367,Ult1219
9:0.01105)0.962.73:0.0151)0.894.76:0.00818)0.719.13:0.00331)0.891.67:0.01
506)0.882.84:0.01293,(Ult12235:0.03502,(Ult12244:0.00369,Ult12248:5.5E-
4)0.761.55:0.00458)0.364.9:0.00615)0.562.12:0.00679)0.859.69:0.00874)0.95
9.69:0.02323)0.826.48:0.00803,(((((Ult12259:0.0,Ult12277:0.0):0.00754,(Ul
t12275:0.0233,Ult12278:0.00792)0.923.107:0.01208)0.908.91:0.01355,(Ult122
79:0.00889,Ult12223:0.04922)0.708.21:0.01022)0.862.73:0.01174,(Ult12284:5
.5E-
4,(Ult12280:0.01483,(Ult12282:0.09601,Ult12281:0.01989)0.784.59:0.01067)0
.947.60:0.03094)0.892.72:0.01627)0.297.3:5.5E-
4,UltRu284:0.01674)0.947.61:0.02407)0.768.45:0.01601)0.857.79:0.0154,(Ult
Ru288:0.05572,UltRu289:0.01952)0.923.108:0.02065)0.894.77:0.02214,((UltRu
285:0.0,UltRu286:0.0):0.01207,UltRu287:0.05022)0.963.66:0.04694)0.879.79:
0.0199,(((Ult12287:0.00793,((Ult12283:0.05754,Ult12316:0.0112)0.489.8:5.4
E-
4,Ult12286:0.11085)0.634.8:0.01901)0.777.63:0.00637,(Ult12314:0.02762,(Ul
t12293:5.4E-
4,(UltVe205:0.04039,(Ult12289:0.01851,(Ult12290:0.00744,((Ult12291:0.0,Ul
t12292:0.0):5.5E-4,Ult12288:0.00369)0.875.81:5.5E-
4)0.778.64:0.00571)0.772.62:0.00609)0.970.58:0.02775)0.944.60:0.01591)0.9
53.58:0.02324)0.676.8:0.00273,(Ult12315:0.00375,(Ult12296:5.5E-
4,(Ult12295:0.04386,(Otu01000:0.01591,(Ult12317:0.0,Ult12318:0.0):0.05775
)0.845.76:0.0116)0.956.66:0.01932)0.284.3:0.00746)0.956.67:0.02702)0.432.
15:0.00953)0.891.68:0.02778,(((Ult12309:0.00802,Ult12310:0.0364)0.983.44:

0.04722,(UltOr168:0.03862,(((((Ult12301:0.04993,(Ult12299:0.08456,Ult1230
0:0.10977)0.996.64:0.10943)0.819.46:0.00922,((Ult12297:0.0,UltProk4:0.0,U
lt12304:0.0):5.5E-4,(Ult12302:5.5E-
4,Ult12303:0.00369)0.830.51:0.00369)0.859.70:5.5E-4)0.863.86:5.4E-
4,Ult12298:0.01105)0.983.45:0.03926,(Ult12305:5.5E-4,(Ult12306:5.5E-
4,Ult12307:0.01499)0.900.68:0.00728)0.272.7:0.01545)0.946.72:0.03175,Ult1
2308:0.02423)0.921.92:0.02496)0.805.51:0.01297)0.916.91:0.03524,(Ult12312
:0.01402,Ult12311:0.05142)0.967.53:0.04821)0.957.59:0.03775)0.728.31:0.00
573)0.982.48:0.05129)0.823.45:0.02277)0.941.71:0.02693)0.690.17:0.00785)0
.826.49:0.01195)0.722.16:0.00778)0.920.93:0.02495,(((UltB2753:0.06557,(Ul
trZeta:0.02097,UltB2752:0.03068)0.984.49:0.05796)0.955.53:0.0456,(UltB355
5:0.10205,((Ult29230:0.04627,(Ult29281:5.5E-
4,((Ult29277:0.0,Ult29279:0.0):0.00369,((MaiMet29:5.4E-
4,Ult29280:0.00754)0.874.94:0.0075,((Ult29229:0.0,Ult29228:0.0,Ult29272:0
.0,Ult29273:0.0,Ult29274:0.0,Ult29278:0.0,Ult29282:0.0):0.00362,(Ult29275
:5.5E-
4,Ult29276:0.0072)0.953.59:0.01525)0.749.66:0.00365)0.975.53:0.01936)0.31
0.6:0.01489)0.954.68:0.03756)1.000.549:0.13826,((UltOrg71:0.0,UltOrg72:0.
0):0.03734,(UltOrg70:0.00746,(UltCand7:0.00745,UltOrg69:5.5E-
4)0.889.76:0.00744)0.887.87:0.019)0.973.44:0.05379)0.848.66:0.03565)0.082
.7:0.01975)0.833.64:0.01647,(Ult16519:0.08728,((((Ult17417:0.0195,(((UltO
r284:0.05488,(((UltOr283:0.01355,Ult17412:0.00912)0.958.78:0.02512,(Ult17
410:0.04107,((Ult17421:0.02626,(Ult17419:5.5E-
4,Ult17420:0.01492)0.872.88:0.0253)0.933.80:0.02523,Ult17418:0.04697)0.44
1.12:0.00378)0.394.10:0.00746)0.768.46:0.00964,Ult17413:0.0337)0.786.66:0
.02047)0.708.22:0.00466,(Ult17402:0.05031,((UltOr280:0.0,UltOr281:0.0):0.
00412,(UltAct23:0.00741,(Ult17400:0.0074,(Ult17398:0.0,Ult17399:0.0,Ult17
401:0.0,Ult17446:0.0,Ult17447:0.0):5.5E-4)0.106.7:5.5E-
4)0.925.95:0.0148)0.618.5:0.01137)0.776.66:0.01621)0.730.27:0.00423,(((Ul
tChl60:0.08664,Ult17450:0.05503)0.818.36:0.04544,(Ult17444:0.09666,(((Ult
17438:0.00836,(Ult17437:0.02707,(Ult17435:5.5E-
4,Ult17436:0.01865)0.422.8:5.5E-
4)0.744.42:0.00288)0.980.45:0.04173,(Ult17439:0.03784,Ult17434:0.04404)0.
378.9:0.01564)0.753.62:0.00639,(Ult17440:0.06418,(((Ult17426:0.00764,((Ul
t17424:0.0306,(Ult17427:0.00369,Ult17428:5.5E-4)0.931.70:5.5E-
4)0.863.87:0.00765,(Ult17429:0.00369,(Ult17423:5.5E-
4,Ult17425:0.00369)0.000.571:5.5E-
4)0.896.67:0.00744)0.954.69:0.01553)0.792.69:0.0062,(Ult17430:0.0,Ult1743
1:0.0):0.0126)0.838.67:0.01165,(Ult17432:0.00757,Ult17433:0.01515)0.853.6
3:0.00878)0.985.57:0.0418)0.605.12:0.01017)0.886.101:0.02303)0.907.75:0.0
2479)0.743.52:0.00615,(UltCa152:0.01113,Ult17414:5.5E-
4)0.866.72:0.00828)0.831.48:0.00779)0.862.74:0.01093)0.756.58:0.00789,(((
(Ult17441:0.02498,((Ult17411:0.00408,((((Ult17405:0.03888,Ult17404:0.0428
6)0.783.70:0.01554,((Ult17407:0.01509,Ult17406:5.5E-
4)0.816.51:0.00733,UltOr282:0.05436)0.825.50:0.00901)0.757.46:0.00756,(Ul
tDe228:0.00405,UltFus14:0.03894)0.907.76:0.01828)0.117.5:0.00821,(Ult1740
8:5.5E-
4,Ult17409:0.03062)0.997.75:0.04683)0.536.10:0.01412)0.864.76:0.01225,(Ud
nBac71:0.03461,Ult17445:0.0572)0.714.22:0.01375)0.838.68:0.01215)0.516.5:
0.01262,(Ult17422:0.06139,Ult17443:0.01809)0.315.9:0.01107)0.785.65:0.016
39,(Ult17403:0.07884,Ult17442:0.06272)0.746.44:0.00666)0.930.70:0.01595,(
(Ult17448:5.4E-
4,Ult17449:0.04302)0.990.64:0.05239,(Ult17416:0.02574,(UltPro87:0.04521,U
lt17415:0.05178)0.942.72:0.03008)0.635.5:0.01607)0.900.69:0.02627)0.396.1

6:0.00149)1.000.550:0.08487,((((UltPro17:0.03648,UltB4304:0.04442)0.981.5
6:0.05536,(UltDel36:0.07234,BacEnr18:0.01756)0.983.46:0.06396)0.891.69:0.
02499,(((((UltB4451:0.05095,UltB4450:0.12198)0.936.73:0.04718,((UltPro49:
0.08824,((UltB4281:0.06361,UltB4493:0.08303)0.988.65:0.11189,UltB4280:0.1
2428)0.240.8:0.01667)0.950.67:0.06329,(UltB4314:0.11391,((((UltDel42:0.0,
UltB4319:0.0):0.02684,(((BdlSpeci:5.4E-
4,(BcvStolp:0.01515,UltB4316:0.00751)0.546.11:0.00349)0.879.80:0.02576,Ul
tB4312:0.05551)0.847.85:0.01401,((UltB4313:0.10496,(UltDel39:0.04295,PbtS
tarr:0.03725)0.315.10:0.01041)0.793.58:0.01955,((UltDel43:0.06056,UltDel4
4:0.01173)0.899.80:0.02233,(UltB4311:0.00805,(UltB4317:0.01116,UltB4318:5
.5E-4)0.704.14:0.00336)0.995.76:0.04626)0.827.54:7.5E-
4)0.942.73:0.01707)0.763.69:0.00415)0.899.81:5.4E-
4,(UltDel40:0.01532,((UltB4310:0.00752,BcvSpec4:0.00741)0.956.68:0.01485,
BcvMarin:0.01491)0.903.76:5.5E-4)0.907.77:0.00772)0.809.52:5.5E-
4,(UltDel41:0.02632,((BcvSpeci:5.5E-
4,(UltBdel2:0.01125,(UltBdell:0.0037,BcvLitor:5.5E-
4)0.915.79:0.00749)0.898.98:5.4E-4)0.971.54:5.3E-
4,(BcvSpec2:0.02893,BcvSpec3:0.0192)0.995.77:0.0509)0.972.65:0.0261)0.881
.91:0.0129)0.653.7:0.00615)0.998.89:0.12658)0.817.35:0.02866)0.925.96:0.0
4777,(UltDe152:0.10762,(((ChgArsen:0.04927,(BacCCSDD:5.5E-
4,DrpAlkal:5.5E-
4)0.790.64:0.00659)0.993.52:0.05184,BacteAHT:0.019)0.862.75:0.01177,Bacte
AH2:5.5E-
4)1.000.551:0.20191)0.682.15:0.04722)0.454.14:0.02433,((UltSp238:0.18074,
(Ult24346:0.0297,(Ult24345:0.03548,Ult24344:0.07315)0.910.79:0.03437)0.88
4.85:0.04532)0.844.79:0.02477,(((UltB5038:0.07788,(UltSp223:0.01747,UltSp
224:0.03226)0.970.59:0.07082)0.983.47:0.07282,(UltB5037:0.12691,(UltB5036
:0.05465,UltGa286:0.06338)0.764.64:0.01208)0.055.1:0.014)1.000.552:0.1242
3,(((CytSpec2:5.4E-
4,CytSpec3:0.03051)0.975.54:0.05886,(((UltB4991:0.00718,((CytSpeci:0.0150
3,UltMar40:0.00773)0.746.45:0.00391,(UltB4990:0.00742,UltSp161:5.3E-
4)0.925.97:0.01123)0.376.4:0.00753)1.000.553:5.4E-
4,(UltB4989:0.04409,OlvAlga4:0.06158)0.340.10:0.0039)0.912.102:0.02594,Ol
vLois3:0.03164)0.674.14:0.00724)1.000.554:0.10316,(((UltSp121:0.01485,(((
UltSp122:0.01572,(UltB4839:0.03273,(UltSp120:0.0036,UltB4840:0.01166)1.00
0.555:0.07007)0.734.38:0.00244)0.788.61:0.00705,(SpcTaiwa:0.04456,(UltB49
04:0.08248,((((UltB4937:0.02324,(UltB4936:0.00947,(UltSp132:0.00375,UltEu
b12:0.01138)0.896.68:0.01388)0.769.59:0.00891)0.886.102:0.02263,((TreSpe1
9:0.06036,(TreSpe20:0.02686,(TreRefri:0.01361,(TreSpe18:0.02,TreCalli:0.0
2045)0.711.20:0.01361)0.880.84:0.01445)0.806.40:0.01243)0.899.82:0.02039,
(((TreSpe26:0.01918,(TreSpe25:0.00368,(UltTr374:0.00738,(TreSpe21:0.01518
,(TreVinc2:5.5E-4,TreVince:0.00369)0.861.82:0.0037)0.669.12:5.4E-
4)0.713.23:0.00767)0.799.64:0.00784)0.755.51:0.00569,(TreSpe22:0.01125,(T
reSpe23:0.00369,TreSpe24:5.5E-
4)0.986.49:0.03218)0.752.60:0.00577)0.992.64:0.04634,(((((TreDent6:0.0,Tr
eDenti:0.0):0.00373,TreSpe15:0.00368)0.955.54:0.01129,((TreSpe13:0.01117,
(TreSpe12:0.01473,(TreSpe14:0.01939,TreGenom:0.00771)0.998.90:5.5E-
4)0.984.50:0.03598)0.935.74:0.01561,(SrcIsola:0.01131,TreSpe16:5.3E-
4)0.976.42:0.01893)0.489.9:5.5E-4)0.794.53:0.00368,TreSpe17:5.4E-
4)0.966.81:0.02687,((TrePall2:0.0,TrePalli:0.0):0.04162,TrePhage:0.02301)
0.866.73:0.01935)0.845.77:0.01263)0.893.84:0.01344)0.905.74:0.03212)0.887
.88:0.02659,((((UltB4928:0.01771,UltB4927:0.01825)0.999.114:0.09827,(((Tr
eParv3:0.00367,TreParvu:5.4E-
4)0.993.53:0.04934,(((UltB4924:0.01276,UltB4912:0.10504)0.149.8:0.00735,(

(UltB4926:0.09859,UltRum22:5.5E-
4)0.975.55:0.03118,TrePorci:0.01485)0.812.46:0.00842)0.802.51:0.00742,(((
TrePecti:0.03233,(TreBerli:5.5E-4,UltB4914:5.5E-
4)0.969.50:0.03666)0.991.61:0.04472,((UltTr373:0.07781,((UltSp130:0.0,Ult
Sp130.1:0.0,UltTr372:0.0,Otu00267:0.0):0.01702,(((UltTr364:5.5E-
4,UltTr369:5.5E-4)0.768.47:5.5E-
4,((UltTr366:0.0,UltTr367:0.0,UltTr368:0.0,Otu00049:0.0):5.5E-
4,UltTr365:0.0037)0.843.57:0.00371)0.913.83:5.3E-
4,(UltTr370:0.00377,UltTr371:0.01129)0.795.56:0.00701)0.953.60:0.02216)0.
979.56:0.05096)0.812.47:0.01668,(((HsTcTTc7:0.0,UltSp131:0.0):0.01713,(Ul
tSp128:0.00728,(UltSp129:0.00369,(Otu00207:5.5E-
4,UltTr363:0.00368)0.000.572:5.5E-4)0.920.94:5.4E-
4)0.996.65:0.07496)0.879.81:0.02915,(Hmyy0011:0.05705,((Otu00217:0.08968,
((UltTr356:5.4E-4,(UltTr354:5.4E-
4,((UltTr355:0.0,UltSp126:0.0):0.00359,UltSp125:0.00381)0.281.4:0.0037)0.
973.45:0.01877)0.981.57:0.02562,(((Otu00375:0.01543,(UltSp127:5.5E-
4,(UltTr182:0.00369,(UltTr357:0.0,UltTr362:0.0,Otu00109:0.0):5.5E-
4)0.847.86:0.0037)0.874.95:0.00754)0.774.75:0.00656,(UltTr360:0.00766,(Ul
tTr359:5.3E-
4,UltTr358:0.01896)0.692.9:0.00761)0.307.4:0.00729)0.912.103:0.01257,UltT
r361:5.4E-
4)0.835.61:0.00914)0.823.46:0.01872)0.865.81:0.05332,((TreSocr6:5.4E-
4,TreSocra:0.01116)0.999.115:0.12642,(MsDtD000:5.4E-4,SpraaacY:5.5E-
4)0.998.91:0.12016)0.610.6:0.01243)0.471.11:0.00979)0.926.93:0.03324)0.82
8.48:0.01495)0.766.57:0.00784)0.852.72:0.01471,((((UltB4913:0.00823,UltRu
m20:0.01495)0.840.71:0.0161,(((UltB4909:0.0981,(TreSpeci:0.04018,TreZiole
:0.05388)0.913.84:0.03881)0.910.80:0.03163,(UltB4911:0.11123,UltB4910:0.0
2007)0.867.73:0.0318)0.865.82:0.04015,(UltB4905:0.06122,(UltB4906:5.5E-
4,UltB4907:0.01115)0.802.52:0.00709)0.965.58:0.03936)0.970.60:0.05095)0.8
89.77:0.04168,((UltRum21:0.02146,((TreSpec4:0.0,TreSpec5:0.0):0.07336,(Tr
eSpec3:0.01921,(TreSpec2:0.01538,TreAmylo:0.01545)0.759.71:0.0043)0.849.6
6:0.01606)0.969.51:0.04131)0.666.15:0.03115,(UltB4925:0.01896,UltRum29:0.
08637)0.935.75:0.03442)0.909.69:0.03407)0.658.9:5.5E-
4,(((UltRum28:0.02259,UltRum27:0.01669)0.954.70:0.0204,(UltRum23:5.4E-
4,(UltRum25:0.01096,UltRum24:0.00766)0.771.71:0.00745)0.840.72:0.00464)0.
808.53:0.00896,UltRum26:0.00502)0.899.83:0.01204)0.969.52:0.02667)0.775.6
7:0.0072)0.949.73:0.01849)0.062.5:0.00509,((UltB4917:5.5E-
4,(UltB4918:5.5E-4,(UltB4915:0.01108,(TreSpec7:5.5E-
4,TreSpec6:0.0074)0.997.76:0.03124)0.873.82:5.4E-
4)0.738.28:0.00369)0.771.72:0.00381,((UltB4932:0.02659,(UltB4920:0.01181,
((UltB4921:0.0,UltB4923:0.0):0.00372,UltB4929:0.00374)0.753.63:0.00319)0.
864.77:0.0077)0.862.76:5.5E-4,((((UltB4922:0.00369,UltB4934:5.5E-
4)0.923.109:0.02079,UltB4933:0.06419)0.935.76:0.02288,(UltB4919:0.05146,(
UltB4930:0.00385,(UltB4931:5.3E-
4,UltB4916:0.00746)0.786.67:0.00356)0.927.82:0.02373)0.970.61:5.5E-
4)0.763.70:0.00281)0.971.55:0.01883)0.785.66:0.00553)0.881.92:0.02952)0.8
39.66:0.0176,(TreSpe10:0.00586,(TreSpe11:0.01873,(TreLecit:0.01075,(TreSp
ec8:0.01157,(UltB4935:0.01103,TreSpec9:5.4E-
4)0.840.73:0.00697)0.876.69:0.0124)0.901.82:0.01629)0.832.56:0.01829)0.97
9.57:0.04916)0.988.66:0.05841,(UltTr375:0.01357,((Ult36140:0.0,UltB4938:0
.0):0.02332,UltTr376:0.02447)0.176.11:0.00584)0.986.50:0.06833)0.209.8:0.
00678)0.981.58:0.05713,((((UltEub11:0.0154,((UltB4886:0.01173,((UltB4883:
5.4E-4,((((UltB4879:5.5E-
4,(UltB4878:0.01101,(UltB4876:0.0074,UltB4877:5.5E-

4)0.874.96:0.00716)0.922.107:5.3E-
4)0.765.47:0.00362,(((UltB4841:0.00348,Ult27567:0.06953)0.849.67:0.01205,
(UltB4862:5.5E-4,((UltB4868:0.00369,((UltB4850:0.0,UltB4851:0.0):5.5E-
4,UltB4873:5.5E-4)0.000.573:5.5E-4)0.775.68:5.5E-
4,(UltB4843:0.0037,(UltB4849:0.0,UltB4852:0.0,UltB4853:0.0):0.00378)0.873
.83:5.5E-4)0.724.20:5.4E-4)0.957.60:5.5E-
4)0.878.76:0.00766,(UltB4857:0.00369,(((UltB4859:5.5E-4,UltB4845:5.5E-
4)0.000.574:5.5E-4,(UltB4863:0.01118,UltB4854:0.00369)0.734.39:5.5E-
4)0.000.575:5.5E-
4,(UltB4861:0.00369,(UltB4842:0.0,UltB4844:0.0,UltB4847:0.0,UltB4848:0.0,
UltB4855:0.0,UltB4856:0.0,UltB4858:0.0,UltB4860:0.0,UltB4864:0.0,UltB4866
:0.0,UltB4867:0.0,UltB4869:0.0,UltB4870:0.0,UltB4871:0.0,UltB4872:0.0,Ult
B4874:0.0,UltB4875:0.0):5.5E-4)0.000.576:5.5E-4)0.796.47:5.5E-
4)0.766.58:0.0035)0.993.54:0.02808)0.921.93:0.01151,UltB4882:5.5E-
4)0.834.48:0.00367,(UltB4880:0.0,UltB4881:0.0):5.5E-
4)0.993.55:0.01898)0.892.73:0.00793,(UltB4885:0.01522,(UltB4884:0.00773,U
ltSp123:5.5E-
4)0.956.69:0.01938)0.824.47:0.00746)0.911.97:0.01498)0.979.58:0.02314,((U
ltEub10:0.01773,(UltB4887:0.01939,UltB4888:0.02508)0.642.9:0.01035)0.950.
68:0.02225,(((UltB4891:5.3E-
4,UltB4890:0.03086)0.824.48:0.01139,(UltSp124:5.5E-
4,UltB4892:0.01608)0.748.47:0.00357)0.879.82:0.00758,(UltB4889:0.0037,Spt
Bacte:0.00371)0.884.86:5.5E-
4)0.784.60:0.00784)0.860.53:0.0077)0.319.9:5.5E-
4)0.966.82:0.03149,((UltB4900:0.00741,(UltB4899:0.00369,(UltB4895:0.00369
,UltB4901:5.5E-4)0.534.9:5.5E-
4)0.850.81:0.0077)0.976.43:0.03744,(UltB4897:0.01272,(UltB4898:0.01448,(U
ltB4865:5.5E-4,UltB4896:5.5E-
4)0.965.59:0.02065)0.355.12:0.00778)0.976.44:0.03152)0.818.37:0.00879)0.9
22.108:0.02689,(UltB4893:0.0,UltB4902:0.0,UltB4903:0.0):0.04708)0.838.69:
0.02367,UltB4894:0.03585)0.851.74:0.01813)0.893.85:0.0402)0.866.74:0.0218
8)0.919.104:0.02542)0.782.71:0.00462,UltB4838:0.01104)0.319.10:5.5E-
4)0.994.68:0.04998,(((((UltSpi10:0.01141,((Tryy0056:0.00713,(AtPYy108:0.1
985,(Tryy0057:5.4E-4,Try00011:5.5E-
4)0.690.18:0.01001)0.819.47:0.01919)0.823.47:0.01791,(UltTre62:0.00766,((
((TrtGut00:0.0,UltTre61:0.0):0.00369,(UltTre57:0.01516,UltTre60:5.4E-
4)0.760.58:0.00751)0.410.8:5.5E-
4,((UltTre52:0.0,UltTre51:0.0,UltTre58:0.0,UltTre53:0.0,UltTre56:0.0):5.5
E-
4,((UltTre63:0.00369,UltTre66:0.03627)0.767.57:0.00384,UltTre55:0.00369)0
.278.8:5.5E-4)0.077.5:5.5E-4)0.879.83:0.0037,UltTre64:5.5E-
4)0.733.30:0.00783)0.778.65:0.00748)0.961.65:0.02303)0.717.23:5.4E-
4,(((((UltTr320:0.01114,(UltTr319:0.0037,UltTr321:5.5E-4)0.897.72:5.4E-
4)0.904.81:0.01475,(((((((UltTr324:0.0,UltTr325:0.0,UltTr327:0.0):0.0037,
UltTr326:0.0075)0.766.59:0.00625,(UltTr303:5.4E-
4,(UltTr304:0.01136,UltTr305:5.3E-
4)0.960.75:0.01499)0.853.64:0.01015)0.759.72:0.0054,((UltTr338:0.01548,Ul
tTr339:0.0197)0.882.85:0.01504,Try00018:0.0474)0.843.58:0.01169)0.715.16:
0.00711,((Tryy0085:0.01649,(Tryy0084:5.4E-
4,(Try00022:0.00369,((Tryy0068:0.0,Tryy0079:0.0):0.00371,Try00021:0.00369
)0.023.2:5.5E-
4)0.922.109:0.00336)0.020.5:0.00667)0.973.46:0.02612,(UltTr217:0.01384,((
UltTr218:0.0,UltTr219:0.0):5.5E-4,(UltTr342:5.4E-
4,UltTr343:0.00369)0.918.80:0.00746)0.856.64:0.00961)0.923.110:0.0141)0.8

71.77:5.3E-4)0.903.77:0.01251,((((UltTr300:5.4E-
4,((UltTr293:0.00738,(UltTr289:5.3E-
4,UltTr291:0.01509)0.808.54:0.00745)0.760.59:0.00379,(UltTr469:0.01507,((
UltTr297:0.00742,UltTr298:0.00369)0.990.65:5.4E-4,(UltTr292:5.3E-
4,(UltTr288:0.01149,(UltTr299:0.00708,UltTr287:0.01107)0.900.70:5.3E-
4)0.958.79:0.01162)0.741.44:0.00366)0.480.8:0.0075)0.908.92:5.5E-
4)0.922.110:0.00743)0.932.75:5.4E-
4,UltTr302:0.0356)0.784.61:0.00381,((UltTr294:0.00369,(UltTr296:0.0,UltTr
295:0.0):5.5E-
4)0.934.73:0.01139,UltTr301:0.00387)0.774.76:0.0037)0.771.73:0.00344,((Ul
tTr315:0.0,UltTr317:0.0,UltTr316:0.0):0.01519,UltTr318:5.5E-
4)0.960.76:0.01567)0.756.59:0.00313)0.911.98:0.0128,(((AtPYy107:0.04418,(
UltTr310:0.03637,((UltTr307:0.00783,(UltTr309:0.0199,((UltTr313:5.4E-
4,(UltTr314:0.00773,(UltTr306:0.00372,UltTr311:0.0037)0.759.73:0.00352)0.
677.10:0.01138)0.949.74:0.02083,UltTr312:0.00972)0.289.3:0.004)0.920.95:0
.01507)0.759.74:0.0051,UltTr308:0.00619)0.899.84:0.01963)0.848.67:0.01702
)0.891.70:0.02146,(UltTr290:0.00757,UltTr328:0.01138)0.813.52:0.00746)0.7
80.79:0.00861,(UltTr329:0.0133,UltTr330:0.00637)0.997.77:0.04579)0.719.14
:0.003)0.974.42:0.02493)0.870.60:0.01337,(((((UltTr346:5.5E-
4,Otu00120:0.00369)0.871.78:5.4E-
4,((UltTre25:0.02367,((((UltSpi15:0.0,UltTre21:0.0):5.4E-
4,(Otu00253:0.01153,UltTre24:0.01541)0.760.60:0.00355)0.950.69:0.03001,(U
ltTre22:0.01474,(UltTre23:0.00754,Otu00921:0.00756)0.422.9:5.4E-
4)0.160.3:0.01449)0.982.49:0.0375,(Otu00706:0.0262,(NoName00:0.01367,(Ult
Tre26:5.5E-
4,Otu00881:0.00743)0.895.93:0.01384)0.648.13:0.00539)0.888.89:0.01394)0.8
50.82:0.01383)0.941.72:0.03485,(UltSpi95:0.05936,UltSpi16:0.08396)0.949.7
5:0.03946)0.870.61:0.02081)0.710.18:0.01241,(Otu00326:5.5E-
4,(UltB4812:5.5E-
4,Otu00709:0.0037)0.807.45:0.00363)1.000.556:0.10865)0.973.47:0.0326,((Sp
rcaaaa:0.00368,((Ma0sttt5:0.0,MaswgY17:0.0):0.0037,Ma0stttt:5.5E-
4)0.000.577:5.5E-
4)0.995.78:0.04045,(Ma0sttt2:0.01557,(SpraaYyy:0.00772,(SpraYyyy:5.4E-
4,RodSymbi:0.0188)0.771.74:0.00753)0.764.65:0.00301)0.885.72:0.01446)0.81
2.48:0.00942)0.703.20:0.00677,(((UltSp100:0.00678,UltSp101:0.01624)0.984.
51:0.04905,UltSpi99:0.02396)0.920.96:0.0264,((Hmyy0000:0.09373,Hmyy0003:0
.06423)0.991.62:0.0841,MaswgY12:5.5E-
4)0.196.9:0.03253)0.884.87:0.02184)0.878.77:0.01484)0.863.88:0.01597,(Otu
00545:0.0254,(UltSp104:0.01856,UltB4908:0.08339)0.950.70:0.02794)0.538.6:
0.0127)0.676.9:0.00746,(MaswgY13:0.08722,((MasgYyyy:0.0,MaswgY14:0.0,MsAt
AtA3:0.0):5.5E-4,((MaswgY16:0.0,MstBttt2:0.0):5.4E-
4,(MaswgY15:0.0,MstBttB2:0.0):0.0037)0.857.80:0.00369)0.805.52:0.01082)0.
988.67:0.04991)0.752.61:0.00535)0.244.4:0.01219,(UncUnc44:0.01697,(UncUnc
45:0.01518,(UltTr350:5.5E-4,UltTr351:0.0112)0.932.76:5.5E-
4)0.972.66:0.02652)0.874.97:0.01712)0.814.40:0.0117,UltSpi36:0.01147)0.76
8.48:0.00713,(((((Otu00245:0.02789,(((UltTr142:5.5E-
4,Otu00410:0.00366)1.000.557:0.08859,((((UltTr178:5.5E-
4,Otu00428:0.00366)1.000.558:0.06464,((UltTr183:0.0,UltTr184:0.0,Otu00425
:0.0):0.04266,(UltTr198:0.01335,(UltSpi85:0.00422,(UltTr197:0.01133,(UltB
4830:5.5E-4,Otu00159:0.00369)0.739.34:5.4E-
4)0.777.64:0.0032)0.959.70:0.02186)0.701.18:0.02035)0.846.87:0.01849)0.93
3.81:0.024,(((Sgffff05:0.01859,(SpcSpec4:0.03112,SprYyyyy:0.00866)0.956.7
0:0.02384)0.951.70:0.02247,(UltSpi89:0.04579,Otu00096:0.01479)0.947.62:0.
0174)0.957.61:0.02457,((((UltSpi87:0.02533,(Otu00204:0.00886,(UltTr201:5.

4E-4,(UltB4832:5.5E-4,Otu00084:5.5E-
4)0.942.74:0.01131)0.889.78:0.01412)0.896.69:0.01593)0.988.68:0.04481,Hmy
y0010:5.5E-
4)0.816.52:0.01472,Hmyy0009:0.02904)0.285.6:0.01231,(((Otu00064:0.03107,(
(UltB4837:0.0,UltSp119:0.0):5.5E-
4,UltTr352:0.00368)0.994.69:0.05188)0.960.77:0.03848,((UltSpi67:0.0,Otu00
229:0.0):0.02664,UltSpi74:5.5E-
4)0.710.19:0.0042)0.944.61:0.02321,(UltTr175:0.0,Otu00177:0.0):0.02311)0.
418.6:0.01609)0.960.78:0.03638)0.755.52:0.00847)0.474.13:0.02769,((((UltS
pi72:0.0119,((UltSpi70:0.0,Otu00019:0.0):0.0215,(Otu00381:0.01872,((UltTr
185:0.00724,((UltTr200:0.0,Otu00093:0.0):0.0037,Otu00183:5.4E-
4)0.771.75:0.00403)0.847.87:0.00813,UltSpi73:0.04451)0.935.77:0.01436)0.7
63.71:0.00635)0.857.81:0.01252)0.875.82:0.01396,(UltSpi71:0.03697,(UltTr1
86:0.0331,UltTr187:0.0646)0.782.72:0.01337)0.769.60:0.00916)0.863.89:0.01
149,((((UltTr189:5.5E-4,Otu00634:0.0037)0.998.92:5.4E-
4,(Otu00112:0.02809,((((UltB4826:5.4E-
4,((UltSpi56:0.0,UltSpi56.1:0.0):0.00754,UltSpi57:0.00764)0.885.73:0.0075
2)0.823.48:0.0037,UltTr145:5.4E-
4)0.776.67:0.00373,(UltSpi55:0.00374,(UltTr144:5.5E-
4,((UltSpi54:0.0,Otu00050:0.0):5.5E-
4,UltTr465:0.00369)0.926.94:0.00745)0.792.70:0.00371)0.901.83:0.00753)0.7
77.65:0.00378,UltTr143:0.0037)0.872.89:0.0108)0.893.86:0.02794)0.617.6:0.
02629,(Otu00122:0.01356,(UltB4829:0.0053,(UltSpi78:5.4E-
4,Otu00237:0.00723)0.857.82:0.00976)0.909.70:0.01896)0.267.7:0.01411)0.74
7.45:0.0039,((UltSpi45:5.4E-4,(UltSpi44:5.5E-
4,Otu00900:0.00369)0.842.76:0.00367)0.994.70:0.03647,(((((UltB4823:0.0197
2,(((Otu00116:0.01832,(UltTr137:0.00838,Otu00303:0.02525)0.869.69:0.01335
)0.890.87:0.01645,Otu00236:5.5E-
4)0.868.63:0.00536,UltTr138:0.00597)0.994.71:0.03226)0.751.36:0.00357,(Z0
114640:0.0038,(TrePrimi:0.00373,((UltSpi46:0.00743,UltSpi47:5.5E-
4)0.818.38:0.00745,((UltSpi49:0.0,UltTr136:0.0,Otu00060:0.0):5.5E-
4,UltSpi48:0.0037)1.000.559:5.5E-
4)0.827.55:0.00366)0.760.61:0.00369)0.781.59:0.00365)0.940.76:0.01143,(Zn
TrDrD0:0.01121,SpcSpeci:0.01122)0.147.6:5.4E-
4)0.891.71:0.01446,((UltSpi75:0.0,Otu00256:0.0):0.01762,Otu00521:0.00899)
0.871.79:0.01282)0.824.49:0.01117,UltTr190:0.00704)0.912.104:0.0115)0.857
.83:0.00768)0.867.74:0.008)0.866.75:0.01116,((((Z0114632:0.0311,(UltTre12
:0.01051,Otu00231:0.03874)0.354.7:0.00535)0.921.94:0.01272,((Kty00005:0.0
,Kty00006:0.0):0.02041,Otu00101:0.01903)0.827.56:0.01407)0.856.65:0.00771
,(UltTre46:0.00594,UltSpi30:0.01443)0.794.54:0.00538)0.900.71:0.01178,Otu
00715:0.01196)0.543.10:0.00807)0.395.9:0.0163)0.396.17:5.5E-
4)0.772.63:0.00446,Otu00903:0.01388)0.201.6:0.00379)0.871.80:0.0081,(((((
Ktyy0009:0.0,SpcSpe14:0.0):0.00377,(KtYyyyy7:0.0,Kty00008:0.0,SpcSpec3:0.
0,Kty00009:0.0):0.02695)0.734.40:0.00753,(((Ult35830:0.0,Otu00010:0.0):5.
5E-4,(UltB4831:5.5E-4,Ult36445:0.00372)0.753.64:5.5E-
4)0.808.55:0.00374,(UltSp290:5.4E-
4,(UltTr199:0.01567,UltSpi86:0.02515)0.786.68:0.00945)0.851.75:0.00722)0.
754.48:0.00393)0.958.80:0.02003,((UltTr174:0.0,Otu00301:0.0):0.01423,(Znv
Yyyy0:5.4E-4,((Sgffff04:5.5E-
4,(UltTr179:0.00744,(UltSpi65:0.00742,Otu00562:5.5E-
4)0.832.57:0.00369)0.736.35:5.5E-4)0.897.73:5.4E-4,((Sgffff06:5.5E-
4,(UltB4833:0.00367,(UltSpi66:0.00369,Otu00137:5.5E-4)1.000.560:5.5E-
4)0.833.65:0.00746)0.875.83:0.0076,(UltSpi88:5.4E-
4,((UltB4834:0.0,Otu00127:0.0):0.02323,(UltTr177:0.00371,UltTr176:0.01147

)0.763.72:0.00507)0.888.90:0.01061)0.912.105:0.00762)0.784.62:0.0036)0.93
8.50:0.00746)0.792.71:0.00494)0.895.94:0.01475)0.760.62:0.00686,((Hmyyy02
0:0.03683,(SpraaaYy:0.01979,(UltSpir2:0.03142,((UltSpi37:0.00499,Otu00477
:0.01041)0.987.73:0.02995,(UltTre44:5.3E-
4,Otu00364:0.00742)0.800.51:0.00808)0.437.11:0.00835)0.836.51:0.00759)0.4
62.11:0.00386)0.809.53:0.0087,((((SpKlSpec:0.07754,Otu00156:0.03396)0.784
.63:0.01574,(Otu00494:0.04539,(UltSpi68:0.03606,Otu00672:0.01249)0.449.20
:0.02916)0.883.91:0.02776)0.816.53:0.02143,((((UltTr141:0.0,Otu00141:0.0)
:0.01866,(((UltSpi24:0.0,Otu00526:0.0):0.03134,UltSpi25:0.00743)0.886.103
:0.01192,(Otu00550:0.00863,(UltSpi77:0.0,UltTr188:0.0):0.04921)0.540.8:0.
00263)0.949.76:0.02388)0.458.15:0.01585,UltSpi79:0.01712)0.944.62:0.02246
,(Ktyy0039:0.01821,Otu00173:0.00852)0.759.75:0.00774)0.847.88:0.01338)0.9
06.96:0.01633,((Ktyy0004:0.01531,((UltTre49:0.01605,UltTre50:0.00339)0.97
6.45:0.02786,(UltTre48:0.00702,((UltSpi33:0.0,UltSpi33.1:0.0):0.00757,Otu
00041:5.5E-
4)0.768.49:0.00426)0.931.71:0.01683)0.731.23:0.00468)0.123.5:0.00668,(Kty
00007:0.03427,UltTre13:0.02115)0.851.76:0.01175)0.730.28:0.0044)0.713.24:
0.00545)0.986.51:0.02743)0.843.59:5.3E-
4)0.871.81:0.00941,((((UltSpi31:0.00421,UltTre47:0.01868)0.836.52:0.01576
,(UltB4815:0.03033,Otu00259:0.01676)0.890.88:0.01463)0.886.104:0.02064,((
((((((Hmyy0004:0.06932,Hmyy0005:0.0159)0.997.78:0.07594,((Hmyy0007:0.0309
4,Hmyy0008:0.00411)0.915.80:0.03311,(Hmyy0002:0.06968,Hmyy0006:5.4E-
4)0.943.70:0.03578)0.898.99:0.03174)0.888.91:0.02959,UltSpi40:0.02892)0.8
66.76:0.02617,((UltSpi91:0.01661,UltTr265:0.00633)0.871.82:0.01981,((((At
PYy106:0.09327,((AtPYy104:5.5E-4,(AtPYy101:0.00373,(AtPYy100:5.5E-
4,AtPYy102:0.00741)0.804.46:0.00369)0.800.52:0.00369)0.772.64:0.00369,(Ul
tTr258:5.4E-4,(((((Tryy0080:0.00369,(UltTr224:0.0236,((UltTr195:5.4E-
4,(((((UltTr192:5.5E-4,UltTr194:0.00373)0.851.77:0.00369,UltTr191:5.5E-
4)0.712.17:5.4E-
4,UltTr193:0.00745)0.815.53:0.00373,((UltTr256:0.00369,(UltTr323:0.02688,
UltTr255:0.01112)0.912.106:5.4E-4)0.994.72:5.5E-
4,(UltSpi41:0.01894,(((UltTr261:0.0,UltTr262:0.0):5.4E-
4,(UltSpi42:0.01687,(UltTr216:0.0072,UltTr322:0.02124)0.680.15:0.00611)0.
875.84:0.00993)0.904.82:5.5E-
4,((((Try00013:0.00368,((Try00014:0.0,Tryy0076:0.0,Tryy0078:0.0):5.5E-
4,(Tryy0070:0.0037,Tryy0077:0.00742)0.859.71:5.5E-4)0.808.56:5.5E-
4)0.869.70:5.4E-4,((TrZZZZ03:0.01115,(Try00017:0.0,Tryy0075:0.0):5.4E-
4)0.325.9:0.00366,((UltTr331:0.0,UltTr332:0.0):5.5E-
4,(Tryy0069:0.01128,((UltTr333:0.0,TrtGut06:0.0):5.5E-4,((UltTr215:5.5E-
4,(((((UltTr337:5.5E-
4,(UltTr334:0.01503,UltTr340:0.00199)0.960.79:0.00194)0.931.72:0.00237,Ul
tTr336:0.01086)0.920.97:0.0016,TrtGut03:5.4E-
4)0.529.7:0.00417,UltTr335:0.01138)0.111.4:5.3E-
4,(SpcSpec6:0.00419,UltTr341:0.02338)0.842.77:0.00699)0.865.83:0.00725)0.
822.42:0.00371,TrtGut05:0.00371)0.164.11:5.3E-
4)0.831.49:0.00369)0.693.16:5.4E-4)0.959.71:5.4E-
4)0.950.71:0.01115)0.962.74:0.01094,((Tryy0066:0.00367,((Tryy0065:0.0,Try
y0071:0.0,Tryy0074:0.0,Try00019:0.0,Tryy0081:0.0,Tryy0082:0.0):5.5E-
4,Try00016:0.00369)0.536.11:5.5E-4)0.968.64:5.5E-
4,(Try00023:0.0337,(Tryy0072:5.5E-4,Try00020:5.5E-
4)0.832.58:0.00366)0.668.15:5.3E-4)1.000.561:5.3E-
4)0.660.9:0.01455,Try00015:0.00771)0.952.61:0.01805)0.342.8:0.00352)0.895
.95:0.00724)0.894.78:0.0072)0.927.83:5.5E-
4,UltTr196:0.00714)0.855.75:0.00683)0.876.70:0.00711,(UltTre85:5.5E-

4,UltTr286:0.01911)0.884.88:0.00749)0.914.92:0.01119)0.768.50:0.00325)0.8
13.53:0.00363,(UltTr222:0.0117,(Tryy0086:0.04899,((((UltTr249:0.00745,Ult
Tr250:5.5E-
4)0.926.95:0.0129,(AtPYy103:0.00912,AtPYy097:0.03)0.877.97:0.01152)0.810.
52:0.00597,AtPYy105:0.0153)0.771.76:5.4E-
4,(AtPYy098:0.0,AtPYy099:0.0):0.0231)0.916.92:0.01176)0.845.78:0.00782)0.
896.70:0.01134)1.000.562:5.5E-
4,((UltTr252:0.01128,(UltTr251:0.00372,(UltTr345:0.00375,UltTr344:0.01142
)0.890.89:0.00756)0.778.66:0.00374)0.769.61:0.00376,(UltTr263:5.5E-
4,(UltTr260:5.4E-4,((((UltTr257:5.5E-
4,((UltTr238:0.0,UltTr232:0.0,UltTr231:0.0,UltTr229:0.0,UltTr230:0.0):5.4
E-4,(UltTr221:5.5E-4,((UltTr226:5.5E-
4,(UltTr259:0.00763,UltTr234:0.01948)0.899.85:0.01122)0.429.8:0.00375,((U
ltTr227:0.01115,(UltTr228:5.5E-
4,(((UltTr206:0.0,UltTr207:0.0):0.00369,(UltTr524:5.5E-
4,(UltTr210:0.00369,UltTr208:0.00369)0.905.75:5.5E-4)0.963.67:5.3E-
4)0.900.72:5.4E-4,(UltTr205:5.4E-4,(UltTr220:0.01127,(UltTr202:5.5E-
4,(UltTr204:0.00369,UltTr203:0.00369)0.812.49:5.5E-4)0.940.77:5.5E-
4)0.947.63:0.00751)0.902.87:0.00743)0.913.85:0.00744)0.889.79:5.5E-
4)0.606.10:0.0038,UltTr211:0.01155)0.889.80:0.01101)0.886.105:0.01151)0.9
30.71:0.01114)0.842.78:0.00369)0.949.77:0.00749,UltTr254:5.4E-
4)0.868.64:0.00815,UltTr253:0.01443)0.414.7:0.0038,(UltTr225:0.01157,UltT
r223:0.01553)0.883.92:0.01074)0.790.65:0.00436)0.764.66:0.0037)0.900.73:0
.00748)0.793.59:0.00352)0.803.45:0.00714,(UltTr209:0.0228,(UltTr285:0.003
79,((UltTr282:5.3E-4,UltTr283:0.02993)0.943.71:0.01096,UltTr284:5.4E-
4)0.052.6:0.00743)0.910.81:0.01115)1.000.563:5.4E-
4)0.753.65:0.00361,((UltTr214:0.0152,(UltTr212:0.00376,UltTr213:0.01126)0
.780.80:0.00369)0.883.93:0.00816,(((UltTr248:0.00327,Tryy0083:0.02795)0.9
29.72:0.01527,(UltTr264:5.5E-4,((UltTr235:0.0,UltTr237:0.0):5.5E-
4,UltTr236:0.00369)0.848.68:0.00369)0.767.58:0.00589)0.783.71:0.00557,Ult
Tr233:5.5E-
4)0.651.16:0.0113)0.836.53:0.00741)0.780.81:0.00338)0.787.55:0.00361)0.87
0.62:0.00708)0.879.84:5.5E-
4,(UltTr279:0.01111,(((UltTr280:0.00369,((((UltTr275:5.4E-
4,UltTr276:0.02274)0.590.7:5.3E-
4,UltTr272:0.01471)0.426.17:0.00365,UltTr271:0.04445)0.880.85:5.4E-
4,(UltTr266:0.0,UltTr269:0.0,UltTr268:0.0,UltTr278:0.0,UltTr267:0.0,UltTr
273:0.0,UltTr281:0.0):5.5E-4)0.000.578:5.4E-4)0.180.7:5.5E-
4,UltTr277:0.00371)0.298.6:5.3E-4,((UltTr274:5.3E-
4,(Try00008:0.0,Try00009:0.0,Tryy0064:0.0):0.01121)0.831.50:0.00369,(UltT
r270:5.5E-4,UltTr433:0.00742)0.154.3:5.5E-
4)0.859.72:0.00371)0.024.5:5.4E-
4)0.775.69:0.00392)0.803.46:0.00375,(AtPYy090:0.02748,UltB4835:0.03177)0.
845.79:0.00778)0.549.12:5.5E-
4,(UltSpi90:0.04142,(AtPYy091:0.1505,(Tryy0073:0.14561,TrZZZZ02:0.08979)0
.847.89:0.02357)0.784.64:0.01206)0.926.96:0.01894)0.923.111:0.02234)0.871
.83:0.0168)0.811.49:0.01042,(UltTre14:0.00542,Otu00283:0.01022)0.924.80:0
.01966)0.874.98:0.01163,((((UltTr348:0.01841,((UltB4836:0.0,Otu00552:0.0)
:0.00631,(UltTr489:0.01082,(UltTr347:5.5E-
4,Otu00157:0.00369)0.950.72:0.01664)0.762.74:0.00432)0.991.63:0.02872)0.0
90.7:5.4E-
4,Otu00384:0.00721)0.980.46:0.02843,(UltSpi39:0.02443,Otu00205:0.01442)0.
239.8:0.0078)0.919.105:0.01931,Otu00033:0.02616)0.763.73:0.00388)0.799.65
:0.00901,((Otu00400:0.01888,(((((UltTr135:0.00369,(UltTr180:0.01118,(UltT

r134:0.00369,(UltTr128:0.0,UltB4822:0.0,Otu00003:0.0):5.5E-
4)0.823.49:5.5E-4)0.850.83:0.0037)0.936.74:5.3E-
4,(UltTr130:0.00366,(UltB4821:5.4E-4,UltTr133:0.01514)0.850.84:5.4E-
4)0.574.8:0.00751)0.935.78:0.00736,(UltTr129:0.01505,((UltSp267:0.0,UltSp
267.1:0.0):5.5E-4,(UltTr132:5.5E-
4,NoName02:0.0037)0.951.71:0.01135)0.856.66:0.00369)0.927.84:5.5E-
4)0.894.79:0.01123,UltTr127:0.00365)1.000.564:5.4E-
4,UltTr131:0.00369)0.953.61:0.02011)0.980.47:0.03242,(((UltTr126:0.01579,
Otu00693:0.00745)0.819.48:0.00674,(UltTr125:5.4E-
4,Otu00257:0.0192)0.880.86:0.00864)0.820.37:0.01228,(UltB4820:0.02019,(At
PYy088:0.00925,UltB4819:0.04776)0.786.69:0.01321)0.249.11:0.0076)0.854.76
:0.01309)0.895.96:0.01361)0.588.8:0.00676,(((Otu00525:0.02351,(UltSpi69:0
.0652,Otu00379:0.03579)0.811.50:0.01114)0.428.11:0.005,((UltTr181:0.05999
,(Otu00637:0.02675,(UltB4813:0.00978,UltSp102:0.03153)0.766.60:0.00724)0.
820.38:0.0112)0.943.72:0.02273,(UltSpi93:0.01405,UltSpir9:0.0356)0.937.72
:0.02113)0.764.67:5.5E-
4)0.847.90:0.00944,(Otu00335:0.02382,(UltSp106:0.01613,UltB4816:0.03212)0
.734.41:0.00247)0.869.71:0.00952)0.831.51:0.01236)0.763.74:0.00439)0.889.
81:0.01303,((((((Otu00397:0.03519,(UltSpi59:0.0071,(((UltTr147:5.5E-
4,Otu00398:0.0037)0.977.51:0.01528,(((((UltTr158:5.3E-
4,(UltTr157:0.00382,UltTr150:0.01123)0.925.98:0.01526)0.782.73:0.0036,(Ul
tB4827:5.5E-
4,Otu00102:0.0037)0.785.67:0.00364)0.969.53:0.01528,(((((UltB4828:5.5E-
4,UltTr155:0.0075)0.849.68:0.00369,UltTr149:5.5E-
4)0.953.62:0.00744,UltTr154:5.3E-
4)0.844.80:0.00215,(UltSpi61:0.0098,UltTr148:0.0183)0.878.78:0.00833)0.89
3.87:0.01419,UltTr146:0.01503)0.782.74:5.5E-
4)0.837.52:0.00366,(UltTre18:5.4E-
4,UltTre19:0.01117)0.737.48:0.00745)1.000.565:5.4E-
4,UltTr153:0.00748)0.902.88:0.00751)0.883.94:5.3E-
4,(UltSpi60:0.00369,Otu00152:5.5E-
4)0.912.107:0.01087)0.796.48:0.00759)0.888.92:0.01118)0.760.63:0.00455,Ot
u00336:0.00378)0.896.71:0.00764,((((UltSpi27:0.0088,(Otu00479:0.02047,(Ot
u00422:0.02395,UltTr353:0.03771)0.825.51:0.0111)0.943.73:0.01919)0.951.72
:5.4E-
4,Otu00208:0.01)0.336.9:0.01053,(UltSpi28:0.00699,(UltTre45:0.0165,Otu003
25:0.04889)0.773.62:0.0063)0.639.13:0.00274)0.874.99:0.01076,(UltSpi80:5.
3E-
4,(UltSpi76:0.01935,((UltSpi51:0.0,UltSpi52:0.0):0.02202,Otu00002:0.00599
)0.950.73:0.02235)0.908.93:0.01518)0.894.80:0.01509)0.907.78:0.01155)0.84
7.91:0.00777,(((UltTre16:0.01121,UltTre17:5.4E-
4)0.768.51:0.00387,((UltSpi13:0.0,Otu00118:0.0):0.01545,(Otu00063:0.0072,
(UltSpi12:0.00713,Otu00100:0.00397)0.979.59:0.01912)0.855.76:5.5E-
4)0.905.76:0.01136)0.705.18:0.0112,(Otu00476:0.03524,((UltB4825:0.01037,O
tu00009:0.00516)1.000.566:0.04388,Otu00160:5.4E-
4)0.240.9:0.00375)0.749.67:5.5E-
4)0.917.79:0.01476)0.954.71:0.01579,(((UltTre36:0.01129,(UltTre35:0.01509
,UltTre34:5.4E-4)0.903.78:5.4E-
4)0.964.71:0.02235,((Otu00304:0.0049,Otu00328:0.02685)0.989.59:0.04275,(U
ltB4811:0.00713,(UltSpi11:0.0101,Otu00072:0.00153)0.992.65:0.04002)0.875.
85:0.02352)0.751.37:0.00689)0.891.72:0.01481,((UltSpir6:0.02263,UltSpir7:
5.3E-
4)0.974.43:0.03997,((UltSpi82:0.00732,(UltSpi81:0.00106,UltSpi92:0.01838)
0.821.46:0.01914)0.866.77:0.01326,(UltSpi83:0.02497,UltSpi84:0.02244)0.34

4.9:0.0207)0.886.106:0.01463)0.951.73:0.02589)0.756.60:0.0039)0.768.52:0.
00495,(((UltSpi38:0.02241,Otu00342:0.02216)0.897.74:0.01337,(((UltSp283:0
.0,UltSpi29:0.0):0.00972,(((UltTre20:0.02284,((UltTre41:0.0153,((Otu00776
:0.01059,(UltTre40:0.0044,Otu00216:0.01454)0.833.66:0.00763)0.888.93:0.00
838,TreAzoto:0.02372)0.795.57:0.00361)0.835.62:5.5E-
4,(UltTre42:0.01482,(UltSpi26:0.00382,Otu00035:0.00736)0.467.11:0.00723)0
.917.80:5.3E-
4)0.865.84:0.00794)0.764.68:0.0085,(Otu00213:0.01255,Otu00401:0.02245)0.0
21.2:0.00389)0.924.81:0.01384,Otu00024:5.4E-
4)0.978.48:0.01838)0.747.46:0.00232,((((UltSpi18:0.00376,UltSpi19:0.0234)
0.856.67:0.00742,(Otu00081:0.00949,Otu00561:0.01897)0.908.94:0.01456)0.77
1.77:0.00406,(SpcSpec5:0.02356,UltSpi22:0.01135)0.750.39:0.00366)0.769.62
:0.00397,((UltSpi20:0.00751,UltSpi21:0.00369)0.752.62:0.00397,(Otu00337:5
.5E-
4,Otu00424:0.02738)0.973.48:0.01916)0.828.49:0.00767)0.920.98:0.01501)0.7
60.64:0.00762)0.753.66:0.00383,UltSp105:0.02366)0.894.81:0.01013)0.874.10
0:0.01071)0.772.65:0.00642)0.910.82:0.01089,(((((UltSp112:0.02004,(UltSp1
10:0.02261,UltSp111:5.4E-
4)0.421.8:0.00748)0.720.17:0.00439,UltSp107:0.03525)0.849.69:0.01185,Otu0
0091:0.01845)0.955.55:0.01661,Otu00006:5.5E-
4)0.817.36:0.01004,((UltTre31:0.02117,(UltSp108:0.00557,UltSp109:0.03055)
0.923.112:0.02074)0.867.75:0.01392,(UltSpi43:0.00232,UltSpi53:0.04755)0.8
88.94:0.0128)0.950.74:0.03127)0.948.77:0.01897)0.753.67:0.00274,(((((Otu0
0105:0.01563,(Otu00399:5.3E-4,(UltSp269:5.3E-
4,UltSpi14:0.0037)0.819.49:0.00352)0.759.76:0.01528)0.863.90:0.00825,(Otu
01026:0.0394,(UltSpi50:0.0031,(UltTrep3:0.00743,UltTrep2:5.5E-
4)0.996.66:0.03657)0.491.4:0.00723)0.558.8:5.4E-
4)0.888.95:0.00831,((((((UltTr140:0.01087,(UncUnc34:0.00745,UncUnc35:5.5
E-4)0.886.107:5.4E-4)0.261.5:0.00739,(UncUnc39:5.5E-
4,(UncUnc41:0.0111,(UncUnc36:5.5E-
4,UncUnc37:0.00369)0.918.81:0.01161)0.483.14:0.00748)0.835.63:0.00711)0.8
86.108:0.01579,(UltTr139:0.00745,UncUnc40:5.4E-4)0.300.10:5.4E-
4)0.416.17:0.01143,UltB4817:0.00744)0.902.89:0.01523,((UltTre94:0.01281,U
ltTr124:0.01282)0.927.85:0.0116,(((((UltTre99:0.01526,((UltTr102:0.0,UltT
r103:0.0):0.00374,(UltTre98:0.01181,UltTr100:0.01132)0.130.6:0.01215)0.90
6.97:0.01615)0.900.74:0.01165,(((UltTre92:5.4E-
4,((UltTre90:0.00365,UltTre91:5.4E-4)0.772.66:0.00745,((UncUnc33:5.5E-
4,(UncUnc38:0.00756,UncUnc32:0.00372)0.783.72:0.00372)0.950.75:0.00746,Ul
tTre93:0.00747)0.946.73:5.4E-
4)0.929.73:0.00742)0.789.58:0.00369,(UltTre96:0.00369,UltTr110:5.3E-
4)0.902.90:0.00753)0.915.81:0.01168,(UltTre95:0.00211,((UltTre89:5.5E-
4,(UltTre97:0.00751,UltTr109:0.00371)0.849.70:0.00726)0.915.82:0.01125,Ul
tTr111:0.00365)0.788.62:0.00215)0.859.73:0.00701)0.931.73:0.01443)0.891.7
3:5.4E-4,(UltTre86:0.02638,(UltSpi17:5.4E-
4,Otu00028:0.01508)0.932.77:0.01264)0.514.7:0.0076)0.865.85:0.00846,((((U
ltTre73:0.0,UltTre75:0.0):5.4E-
4,((((UltTre72:0.00372,(Tryy0058:0.00368,((UltTre77:0.0,UltTre76:0.0,UltT
re69:0.0):5.5E-
4,((Try00003:0.0,Try00004:0.0):0.00369,(Try00007:0.0037,TrtGut02:0.00372)
0.179.3:5.5E-4)0.925.99:5.5E-4)0.899.86:5.5E-4)0.256.4:5.5E-
4)0.874.101:0.00369,(UltTre74:0.0,UltTre71:0.0,UltTre70:0.0):5.4E-
4)1.000.567:5.4E-4,(UltTre80:0.00373,(UltTre68:5.4E-
4,((Tryy0060:0.00369,Tryy0061:5.5E-
4)0.916.93:0.00753,UltTre65:0.0037)0.777.66:0.00375)0.792.72:0.00376)0.80

1.61:0.00365)0.150.4:0.0037,(UltTre59:0.00762,(UltTre78:0.00375,(UltTre54
:0.00374,(UltTre79:5.5E-
4,UltTre67:0.00369)0.806.41:0.00373)0.773.63:0.00376)0.810.53:0.00373)0.7
88.63:0.00367)1.000.568:5.5E-
4)0.917.81:0.00749,UltTre84:0.01923)0.352.7:5.4E-
4,(UltTre81:0.0,UltTre82:0.0,UltTre83:0.0):5.5E-
4)0.871.84:0.00784)0.781.60:0.00376,UltB4818:0.02339)0.860.54:0.01036)0.7
64.69:0.00207)0.931.74:5.4E-
4,((((UncUnc29:0.0,UncUnc31:0.0):0.02305,UncUnc30:0.00971)0.825.52:0.0098
9,(UltSpir8:0.01523,Otu00555:0.01546)0.916.94:0.0157)0.042.4:5.5E-
4,((UncUnc42:0.01081,(UncUnc28:5.4E-
4,UltB4824:0.01488)0.810.54:0.00795)0.756.61:0.00412,((UncUn335:0.0,UncUn
c43:0.0):0.01972,(AtPYy089:0.01647,((UltTre27:0.00355,UltTre28:0.00771)0.
902.91:0.01435,(((UltTr108:0.01123,UltTre88:5.5E-4)0.736.36:5.4E-
4,(UltTr105:0.00367,((UltTr119:0.0,UltTre30:0.0,UltTr122:0.0):0.00748,(Ul
tTr104:5.5E-4,(UltTr106:5.5E-
4,UltTr107:0.0037)0.847.92:0.00369)0.871.85:5.5E-
4)0.899.87:0.00756)0.792.73:0.00374)0.948.78:5.5E-
4,((UltTre87:0.01548,UltTr120:0.0153)0.669.13:7.3E-
4,((UltTr121:0.00747,UltTr123:0.00376)0.481.9:5.4E-
4,UltTre29:0.01914)0.913.86:0.01075)0.916.95:0.01104)0.896.72:0.01292)0.8
20.39:0.01371)0.805.53:0.00882)0.837.53:0.00702)0.879.85:0.01169)0.893.88
:0.0113)0.771.78:0.00384,((UltSpi35:0.00372,(UltSpi34:0.0075,Otu00037:0.0
0749)0.769.63:0.00374)0.782.75:0.00372,(Otu00260:0.0231,(((UltTre33:0.011
25,Otu00057:0.00765)0.866.78:0.00782,(Otu00043:0.00377,(Otu00042:0.01873,
(UltSpir5:0.00551,Otu00158:0.0107)0.812.50:0.00907)0.865.86:0.01018)0.845
.80:0.00744)0.800.53:0.01508,(UltSpir3:0.00373,((UltTrep5:0.0,UltTrep9:0.
0):0.01542,(UltSpir4:0.00379,UltTre15:0.01903)0.777.67:0.00372)0.768.53:0
.00366)0.880.87:0.00733)0.858.77:5.4E-
4)0.914.93:0.00759)0.871.86:0.00751)0.891.74:0.00777)0.765.48:0.00393,(((
UltTre37:0.01927,Otu00108:0.00368)0.881.93:0.0076,(Otu00114:0.01831,(UltS
pi32:0.03072,((UltSpi23:0.0,UltTre38:0.0):5.4E-
4,(((UltSp115:0.00374,((UltSp114:0.01557,UltSpi94:0.00733)0.696.14:0.0195
6,Otu00066:0.01886)0.898.100:5.4E-
4)0.951.74:0.01137,(UltSp116:0.00744,(UltSp117:5.5E-
4,((UltSp113:0.01542,(UltSp103:0.00364,UltSpi98:0.02313)0.948.79:0.0114)0
.998.93:5.5E-
4,(UltSp118:0.00389,UltTr349:0.01879)0.761.56:0.00742)0.800.54:0.00354)0.
006.7:5.4E-
4)0.881.94:0.00748)0.764.70:0.00373,Otu00215:0.00371)0.897.75:0.00748)0.7
73.64:0.00546)0.373.10:0.00526)0.793.60:0.00558)0.899.88:0.00441,((Otu003
96:0.04845,(Otu00054:0.01123,Otu00134:0.01928)0.154.4:0.00747)0.979.60:5.
4E-4,((((((UltTr165:5.5E-
4,(UltTr164:0.00372,UltSpi64:0.00368)0.894.82:0.00744)0.921.95:0.00744,(U
ltTr167:0.0,SpcSpec2:0.0,Otu00030:0.0):5.4E-4)0.757.47:5.4E-
4,UltTr166:0.01504)0.778.67:0.00371,(UltTr160:0.01576,UltTr163:0.01163)0.
866.79:0.00763)0.879.86:0.00749,(UltTr156:5.4E-
4,(UltTre32:0.01595,(UltTr159:5.4E-
4,(((UltTr151:0.00374,UltSpi58:0.00372)0.805.54:0.0037,UltTr152:5.5E-
4)0.793.61:0.00363,UltSpi63:0.00377)0.971.56:0.01513)0.761.57:0.00311)0.9
73.49:0.01581)0.992.66:0.01895)0.877.98:5.3E-4,(((((UltTr172:5.4E-
4,(UltTr161:5.5E-
4,UltTr162:0.00749)0.918.82:0.00751)0.856.68:0.0037,UltTr168:5.4E-
4)0.843.60:0.00369,(((UltTr173:5.5E-

4,Otu00305:0.01895)0.964.72:0.01479,UltSpi62:0.00198)0.948.80:0.00193,(Ul
tTr170:0.00369,(UltTr169:5.4E-
4,UltTr171:0.01121)0.395.10:0.0037)1.000.569:5.4E-4)0.737.49:5.4E-
4)0.964.73:0.015,(Otu00282:0.01507,(UltTre43:5.3E-
4,Otu00017:0.01115)0.942.75:0.01893)0.120.6:5.4E-
4)0.855.77:0.00744,UltB4814:0.06286)0.858.78:0.00758)0.936.75:0.00745)0.9
37.73:0.01401)0.907.79:0.00403)0.784.65:5.4E-
4,((UltTre39:0.00374,Otu00110:0.01148)0.746.46:0.00346,(((UltB4809:0.0074
6,(Try00012:0.00745,((Tryy0067:0.00358,((Tryy0062:0.0,TrZZZZ00:0.0):0.035
5,Tryy0088:0.00372)1.000.570:5.5E-
4)0.970.62:0.01884,((Try00006:0.0,Tryy0063:0.0):0.00165,(Try00002:5.5E-
4,Tryy0055:5.5E-4)0.906.98:0.00775)0.875.86:0.00258)0.997.79:5.4E-
4)0.922.111:0.00826)0.514.8:5.3E-
4,(((((Try00005:0.02829,UltTr118:0.03723)0.810.55:0.01247,(UltTr116:0.007
42,(UltTr114:0.01715,(UltTr113:0.0037,(UltTr115:0.00744,UltTr112:0.00743)
0.587.9:5.5E-
4)0.895.97:0.01225)0.907.80:0.01718)0.879.87:0.00816)0.746.47:0.00479,Ult
Tr117:0.01846)0.997.80:0.03696,SpracYyy:0.06389)0.824.50:5.5E-
4,(UltSpi96:5.4E-
4,UltSpi97:0.00369)0.993.56:0.02283)0.809.54:0.00658)0.231.8:5.5E-
4,((UltTrepo:5.5E-
4,((UncUn308:0.0,UncUnc27:0.0):0.00373,UltB4808:0.01545)0.777.68:0.00373)
0.776.68:0.00373,((((UltB4810:0.02736,UltTrep8:0.00374)0.774.77:0.00368,(
(Otu02385:0.01943,(Otu00480:0.03106,Otu00708:5.5E-
4)0.943.74:0.01525)0.956.71:0.01942,(UltTrep7:5.3E-
4,((UltTr101:0.01137,(Tryy0059:0.02045,(Tryy0087:0.01817,((((TrtGut04:0.0
0369,((UltTr241:0.0,UltTr491:0.0,UltTr244:0.0,UltTr240:0.0,UltTr242:0.0):
5.5E-4,(UltTr243:0.0037,UltTr245:0.00371)0.769.64:5.5E-4)0.910.83:5.5E-
4)0.961.66:5.4E-4,UltTr239:0.01544)0.917.82:0.00751,UltTr247:5.5E-
4)0.899.89:5.5E-
4,UltTr246:0.01117)0.900.75:0.01214)0.538.7:0.00392)0.985.58:0.03068)0.34
0.11:0.01162,(UltSpiro:0.00744,(UltTrep4:5.4E-
4,UltTrep6:0.00372)0.236.3:5.4E-4)0.651.17:0.01113)0.987.74:5.4E-
4)0.867.76:0.00749)0.871.87:0.0075)0.077.6:5.5E-4,UltTre10:5.4E-
4)0.781.61:0.00374,UltTre11:0.00373)0.794.55:0.00371)0.903.79:0.00755)0.9
54.72:0.01272)0.727.25:0.00419)0.895.98:0.00959)0.749.68:0.00908)0.260.6:
0.01071)0.987.75:0.0594,((((((UltSp237:0.04832,(UltB5045:0.03498,UltCand5
:0.01006)1.000.571:0.16341)0.923.113:0.04868,(UltOrg67:0.1251,UltSp239:0.
06456)0.935.79:0.05013)0.640.9:0.00767,((UltChlor:0.05091,UltSp236:0.0566
9)0.997.81:0.12223,(((UltSp228:0.00741,UltSp229:5.5E-
4)0.768.54:0.00598,(UltSp231:0.01403,(UltSp230:5.0E-
4,UltSp232:0.0345)0.929.74:0.01768)0.532.10:0.01276)0.830.52:0.01799,((Ul
tOrg65:0.09778,UltSp233:0.05823)0.933.82:0.04697,UltOrg64:0.06973)0.852.7
3:0.02259)0.996.67:0.11512)0.607.6:0.01154)0.847.93:0.04061,(UltSp221:0.0
8552,UltSp222:0.02105)0.987.76:0.0713)0.912.108:0.04305,((UltOrg63:0.1402
8,UltSp219:0.07766)0.990.66:0.1367,((BrrSpec4:0.03004,((((BrrDutt2:0.0149
2,(BrrLones:0.01489,BrrMiyam:5.4E-
4)0.894.83:0.0074)0.787.56:0.00361,(BrrPersi:5.4E-
4,(BrrDutto:0.00365,BrrSpec3:5.5E-4)0.839.67:0.00365)0.031.2:5.4E-
4)0.896.73:0.00735,BrrTurci:5.4E-4)0.914.94:0.01487,(BrrAfzel:5.5E-
4,(BrrJapon:0.01375,(BrrSpeci:0.00978,BrrSpec2:0.01268)0.018.1:0.00376)0.
589.11:0.00586)0.846.88:0.01421)0.874.102:0.01411)0.947.64:0.08769,CriSpe
ci:0.30901)0.039.4:0.03652)0.844.81:0.0304)0.901.84:0.03678,(((((((UltSp1
76:0.02,((((UltOrg49:0.01346,UltOrg50:0.03914)0.991.64:0.05249,(((((UltSp

142:0.01154,(Kty00010:0.03473,UltSp141:0.01763)0.919.106:0.02318)0.303.9:
0.00706,((UltRum31:0.0397,UltB4970:0.01311)0.961.67:0.06669,(UltAl195:0.0
4648,(UltSp139:0.02916,(UltB4969:0.05323,(UltSp140:5.5E-
4,Otu00580:0.00357)0.816.54:0.01253)0.738.29:0.00523)0.948.81:0.02872)0.8
03.47:0.02094)0.955.56:0.04687)0.933.83:0.02076,SrcEndos:0.06006)0.836.54
:0.02109,(UltB4963:0.02824,UltB4972:0.01146)0.957.62:0.05156)0.991.65:0.0
6925,((UltOrg40:5.4E-
4,(UltOrg39:0.0097,UltB4967:0.014)0.961.68:0.02252)0.982.50:0.03465,((Ult
B4968:0.01118,UltSp137:5.5E-
4)0.884.89:0.01362,(UltSp138:0.01657,UltSp148:0.04468)0.720.18:0.0049)0.9
10.84:0.02329)0.809.55:0.01965)0.350.7:0.0046)0.681.10:0.00264,((UltOrg51
:0.05535,((((UltSp153:5.4E-
4,(((UltOrg43:0.00716,UltOrg44:0.01106)0.917.83:5.5E-
4,(((UltSp151:0.01066,((UltB4982:5.5E-
4,UltOrg42:0.00704)0.650.10:0.00753,UltSp152:0.0239)0.798.57:0.00595)0.79
7.49:0.00562,(UltSp150:0.00368,UltSp149:0.00369)0.647.5:5.5E-
4)0.782.76:0.00353,UltB4981:0.01531)0.884.90:0.00765)0.744.43:0.0031,UltO
rg45:0.06859)0.976.46:0.02766)0.873.84:0.00747,(UltB4983:0.01546,UltSp154
:0.01147)0.764.71:0.00378)0.913.87:0.01241,(UltSp155:0.0274,(((UltB4985:0
.00648,UltOrg47:0.01775)0.953.63:0.02603,(UltB4988:0.04599,(UltB4980:0.03
663,(UltSp160:0.01871,((UltB4997:0.00664,(UltB4995:0.0074,UltB4996:5.5E-
4)0.933.84:0.01226)0.816.55:0.0095,(SpcAlkal:0.00962,UltSp163:0.01398)0.9
55.57:0.02725)0.965.60:0.02779)0.940.78:0.0324)0.796.49:0.01994)0.738.30:
0.00634)0.722.17:0.01738,((UltB4984:0.00744,UltOrg46:5.5E-
4)0.851.78:0.01293,UltSp164:0.04111)0.381.14:0.01427)0.101.1:0.00502)0.81
6.56:0.00739)0.875.87:0.01557,(UltSp170:0.08512,(((UltSp157:0.00369,(UltS
p158:5.5E-4,(UltB4986:0.00743,UltOrg48:0.00747)0.836.55:5.5E-
4)0.968.65:5.5E-4)0.981.59:0.01511,(UltSp156:5.5E-4,UltSp159:5.5E-
4)0.846.89:5.5E-4)0.953.64:5.4E-
4,UltB4987:0.02235)0.340.12:0.02274)0.668.16:0.02915)0.863.91:0.0169)0.80
5.55:0.01123,(UltSp173:5.3E-4,(((((UltOrg41:0.0,UltOrg55:0.0):5.5E-
4,UltOrg56:0.08862)0.754.49:0.00349,(UltSp172:0.0,UltSp175:0.0):0.01924)0
.840.74:0.00765,UltSp171:0.1987)0.695.14:5.4E-
4,UltSp174:0.04753)0.746.48:0.00331)0.942.76:0.02837)0.919.107:0.02596)0.
860.55:0.01327,(SpcAfric:0.00719,SpcDissi:0.01992)1.000.572:0.0612)0.923.
114:0.021)0.937.74:0.02636,(UltB4971:0.05527,UltB4973:0.01856)0.822.43:0.
01202)0.806.42:0.00957,((UltB4979:0.0,UltSp146:0.0):0.0116,(UltB4978:0.01
14,UltSp145:0.00376)0.765.49:0.00335)0.965.61:0.02354)0.870.63:0.01502,(U
ltSp144:0.04316,(UltSp143:0.01804,(UltB4977:0.01897,SpcBajac:0.01692)0.95
7.63:0.02562)0.817.37:0.00876)0.934.74:0.02586)0.740.48:0.00477,((((UltSp
206:0.10355,((UltB5006:0.02664,(UltSp178:0.04006,(UltB5008:0.01143,(UltB5
007:0.01361,UltSp177:0.06155)0.853.65:0.02907)0.924.82:0.03191)0.894.84:0
.0287)0.952.62:0.03223,(((UltB5001:0.00744,UltB5000:0.02679)0.481.10:5.5E
-4,(UltB4999:0.00746,(UltB5005:0.00369,((UltB4998:0.0,UltB5002:0.0):5.5E-
4,UltB5003:0.00744)0.921.96:0.00746)0.882.86:0.00754)0.796.50:0.00372)0.7
84.66:0.00363,UltB5004:0.00374)0.984.52:0.03874)0.916.96:0.02165)0.834.49
:0.01413,(((UltSp205:0.04368,UltSp204:0.05929)0.841.63:0.02078,(((UltB502
4:5.3E-
4,SpcTherm:0.00369)0.998.94:0.09678,((UltB5015:0.02084,(UltB5013:5.5E-
4,UltB5014:5.5E-
4)0.712.18:0.002)0.991.66:0.07159,(((SpcSpec9:0.0037,UltB5009:0.0037)0.77
3.65:0.00516,(UltB5011:0.03964,(UltB5010:5.5E-
4,Ult14343:0.0037)0.794.56:0.00374)0.761.58:0.00606)0.821.47:0.01683,(Ult
Sp180:0.03066,(UltB5012:0.03664,UltSp179:0.01048)0.859.74:0.01945)0.989.6

0:0.05294)0.978.49:0.05563)0.947.65:0.04693)0.160.4:0.00901,(((UltSp199:0
.031,Kty00011:0.03095)0.890.90:0.01911,((((UltSp195:5.5E-
4,(UltTr377:0.01916,Otu00431:0.00372)0.776.69:0.0037)0.880.88:0.00792,(Sp
cSpe10:0.00778,((UncUnc46:0.02222,UncUnc47:0.0208)0.839.68:0.01185,Otu002
42:0.03491)0.868.65:0.00891)0.719.15:0.00358)0.976.47:0.02332,UltSp196:5.
4E-
4)0.856.69:0.01399,(UltSp197:0.0487,Otu00280:0.02827)0.769.65:0.01595)0.9
20.99:0.02405)0.754.50:0.01224,((UltSp198:0.01933,Otu00434:0.02091)0.902.
92:0.02152,(UltB5027:0.0112,(UltB5025:0.01115,UltB5026:5.5E-
4)0.962.75:0.03436)0.996.68:0.05301)0.678.14:0.00377)0.914.95:0.02082)0.9
58.81:0.03424)0.810.56:0.01276,((((UltSp216:5.5E-4,UltSp217:5.5E-
4)0.888.96:0.00754,(((UltOrg62:0.0,UltSp215:0.0):5.5E-4,UltSp214:5.5E-
4)0.848.69:5.2E-4,(UltSp213:5.4E-
4,(UltSp212:0.17937,UltSp211:0.00487)0.666.16:0.00255)0.807.46:0.00369)0.
864.78:0.00763)0.859.75:0.00712,((UltOrg58:0.01145,(UltSp200:0.00738,(Ult
Sp193:5.5E-4,UltB5028:0.00369)0.931.75:5.4E-
4)0.862.77:0.0075)0.967.54:0.01965,((UltB5029:0.10553,UltB5030:5.4E-
4)0.912.109:0.01482,(UltOrg59:0.04026,UltSp203:0.00384)0.931.76:0.01529)0
.863.92:0.00785)0.308.7:5.5E-
4)0.966.83:0.02223,UltSp202:0.02196)0.515.7:5.4E-
4)0.967.55:0.01866)0.288.9:5.5E-4,((UltSp182:0.03367,((UltOrg54:5.5E-
4,UltSp169:0.00369)0.933.85:0.03432,((UltOrg52:5.5E-4,(UltSp166:5.5E-
4,UltSp167:0.0074)0.832.59:0.00368)0.950.76:0.03766,(UltSp165:0.03391,(Ul
tOrg53:5.3E-
4,UltSp168:0.00701)0.904.83:0.01885)0.772.67:0.01247)0.913.88:0.0284)0.89
9.90:0.02619)0.862.78:0.01685,((((((UltB5019:0.02499,UltB5020:0.02494)0.9
81.60:0.03833,(((((UltSp190:0.01124,UltSp189:5.5E-
4)0.993.57:0.03319,(UltSp192:0.0153,UltSp191:0.00771)0.703.21:0.00197)0.9
79.61:0.03898,UltSp183:0.02674)0.783.73:0.00984,((UltB4940:0.01112,UltB49
39:5.4E-
4)1.000.573:0.0723,(UltSp181:0.06592,(UltB5022:0.01934,UltB5023:0.0244)0.
826.50:0.0184)0.668.17:0.01058)0.953.65:0.03139)0.749.69:0.00791,(UltSp18
6:0.0189,(UltSp187:5.4E-4,UltSp188:0.01529)0.779.65:5.4E-
4)0.970.63:0.01926)0.859.76:0.00835)0.745.53:0.00465,UltB5017:0.07707)0.3
61.9:0.004,(((UltSp208:0.02824,UltOrg61:0.02593)0.650.11:0.00578,(UltSp20
9:0.03726,UltSp210:0.02663)0.679.17:0.00585)0.861.83:0.01673,((UltOrg60:0
.00862,UltSp207:0.03109)0.909.71:0.02482,UltOrg57:0.05741)0.672.12:0.0157
4)0.991.67:0.05537)0.416.18:0.00805,((UltB5016:0.0436,UltSp194:0.05255)0.
911.99:0.03083,((UltB5018:0.0342,UltSp185:0.02322)0.880.89:5.4E-
4,(UltSp184:0.05054,UltB5021:0.04543)0.835.64:0.01439)0.799.66:0.00848)0.
884.91:0.01299)0.801.62:0.00673,UltSp201:0.01969)0.786.70:0.00703)0.968.6
6:0.02334)0.929.75:0.02884,(((ShrCocco:0.04916,((((UltB4954:0.02823,(UltB
4953:0.02256,UltRum30:0.06658)0.924.83:0.05038)0.994.73:0.10535,UltB4959:
0.11645)0.635.6:0.00429,(UltB4955:0.0372,(EchggYy4:0.00514,(BfhgggY8:0.00
4,BfhgggY9:0.00353)0.997.82:0.08147)0.996.69:0.09648)0.771.79:0.01216)0.8
79.88:0.02981,(UltB4956:0.01636,(UltB4957:0.01056,UltB4958:0.10231)0.698.
18:0.03085)0.976.48:0.06478)0.780.82:0.04207)0.000.579:0.0138,(UltB4952:0
.16599,((((UltB4947:0.03502,UltB4948:5.5E-
4)0.973.50:0.01903,(((UltB4949:5.5E-
4,(ShrSpec2:0.00369,ShrSpec3:0.0037)0.810.57:0.00368)0.993.58:0.01889,Shr
Speci:5.5E-4)0.931.77:0.01108,(UltB4946:0.00404,((UltB4942:5.5E-
4,(UltB4941:5.5E-4,UltB4943:0.00369)0.883.95:5.4E-
4)0.787.57:0.00376,(UltB4944:5.5E-4,UltB4945:5.5E-
4)0.804.47:0.00369)0.860.56:0.00726)0.404.10:0.0037)0.481.11:5.4E-

4)0.492.11:0.0112,UltSp133:0.00784)0.937.75:0.03019,((UltB4950:0.00368,Ul
tSp134:5.5E-
4)0.924.84:0.03271,UltB4951:0.11327)0.077.7:0.01505)0.785.68:0.01552)0.92
8.65:0.03702)0.934.75:0.03729,(UltB4960:0.05054,(UltB4961:0.08098,UltB496
2:0.03187)0.716.18:5.5E-
4)0.817.38:0.01931)0.987.77:0.06201)0.846.90:0.01798)0.764.72:0.00388,((U
ltSp147:0.1257,(((UltOrg37:0.00378,UltOrg38:0.02311)0.728.32:0.00349,(Ult
Sp136:0.01874,((UltSp135:0.00271,UltOrg36:0.01278)0.022.1:0.01597,(UltB49
65:0.03502,UltB4964:0.02726)0.561.11:0.01981)0.967.56:0.0484)0.608.15:0.0
044)0.931.78:0.01854,UltB4966:0.00888)0.385.13:0.02151)0.456.9:0.02336,Sp
cSymbi:0.09073)0.721.16:0.0025)0.924.85:0.02396,((UltB4976:0.10488,(SpcSp
ec7:0.08616,(UltB4974:0.02378,UltB4975:0.00363)0.999.116:0.14375)0.878.79
:0.03752)0.833.67:0.02581,((SpcCello:0.06586,((((UltB5032:0.01612,(SpcSpe
11:0.01154,UltSp218:0.01133)0.892.74:0.01125)0.856.70:0.02031,SrhBacte:0.
05003)0.950.77:0.02604,UltB5031:0.01204)0.766.61:0.00636,SpcSpe12:0.01612
)0.989.61:0.06491)0.870.64:0.0267,((UltB4994:0.04717,(SpcSpec8:0.02946,(U
ltB4993:0.12753,UltB4992:0.02892)0.741.45:0.01626)0.995.79:0.09003)0.492.
12:0.04943,(UltSp162:0.13424,(SrnCulic:0.0634,UltB5035:0.10419)0.970.64:0
.08011)0.375.10:0.03268)0.859.77:0.03815)0.925.100:0.02615)0.892.75:0.019
36)0.744.44:0.00487)0.865.87:0.02192)0.754.51:0.0132)0.793.62:0.01413)0.9
73.51:0.05551)0.892.76:0.02715)0.852.74:0.01678,((UltB4448:0.13003,(UltDe
l96:0.04786,(UltrB188:0.04859,UltB4449:0.06696)0.414.8:0.01139)0.849.71:0
.02645)0.946.74:0.04633,(UltSynt4:0.01977,((UltB4610:0.00782,UltB4612:0.0
4102)0.949.78:0.02377,UltB4611:0.02608)0.842.79:0.01573)0.997.83:0.06991)
0.773.66:0.01596)0.898.101:0.01881)0.824.51:5.5E-
4,((UltMyxo2:0.02006,(UltB4308:0.01444,UltB4309:0.02435)0.160.5:0.00748)0
.830.53:0.02248,((UltB4305:0.00385,(UltB4306:0.03643,UltB4307:0.03305)0.5
28.8:0.01106)0.995.80:0.06972,(UltDel37:0.05491,(UltB4303:0.01938,(UltDel
35:0.01339,UltSlu21:0.01352)0.754.52:0.01431)0.508.7:0.01758)0.220.6:0.01
377)0.914.96:0.03769)0.576.10:0.0242)0.874.103:0.01321)0.623.7:5.4E-
4,(((UltB4195:0.09856,UltB4279:0.18311)0.640.10:0.01004,(Ult25075:0.06494
,(Ult26662:0.06034,(UltB4276:0.09899,(Ult16400:0.03022,(UltDel14:0.01945,
(UltPro48:0.02707,UltB4277:0.04788)0.139.8:5.5E-
4)0.765.50:0.01345)0.833.68:0.05102)0.984.53:0.09726)0.956.72:0.06504)0.8
80.90:0.05788)0.873.85:0.03136,(((UltB4341:0.05403,UltB4342:0.02913)0.918
.83:0.02986,((UltB4339:0.06232,UltB4340:0.05736)0.912.110:0.07074,UltPro5
1:0.09316)0.343.4:0.01536)0.980.48:0.04937,((((((DrnPalmi:0.0048,(((((Ult
B4443:0.00358,((DrmFerri:0.01121,((DrmBakii:0.01119,(MnnRubra:0.00377,(Pb
rAcidi:0.00754,PbrSelen:0.00744)0.792.74:0.00374)0.902.93:5.5E-
4)0.849.72:0.00718,(DrmSucci:5.5E-4,UltDel92:5.5E-4)0.936.76:5.5E-
4)0.925.101:5.5E-
4)0.965.62:0.02233,(GpsElect:0.01122,UltDel93:0.00375)0.754.53:0.00539)0.
883.96:0.01026)0.986.52:0.01922,(UltDel94:0.01507,((UltB4446:0.03492,(Drn
Aceto:5.4E-4,((DrnThiop:0.01914,DrnSvalb:5.1E-
4)0.815.54:0.00372,UltDel95:0.00387)0.780.83:0.00372)0.948.82:5.4E-
4)0.027.4:0.0037,((UltB4444:0.02557,UltDesu9:0.0262)0.716.19:0.01482,UltB
4445:0.0278)0.825.53:0.01277)0.750.40:0.00759)0.824.52:5.4E-
4)0.913.89:0.01119,(((UltB4429:0.00749,UltB4435:0.00752)0.772.68:0.00412
,((((((GeoSpeci:5.4E-4,((PbrPropi:5.5E-
4,(GeoChape:0.00374,UltDel89:0.02691)0.775.70:0.00358)0.135.7:0.00369,Ult
B4432:0.00361)1.000.574:5.4E-4)0.908.95:0.00742,((GeoSpec2:5.4E-
4,UltDel90:0.02274)0.677.11:0.01127,GeoPsych:0.01507)0.949.79:5.4E-
4)0.884.92:0.00762,(UltB4438:0.00754,(GeoUrani:5.5E-4,(UltB4428:5.4E-
4,(GeoBemid:0.0,UltB4425:0.0,UltB4426:0.0,UltB4427:0.0):0.00751)0.833.69:

0.00369)0.792.75:0.00375)0.867.77:0.00746)0.863.93:0.00763,(((UltB4439:0.
01879,UltB4441:5.3E-
4)0.849.73:0.00771,UltB4430:0.05276)0.755.53:0.00339,GeoLovle:5.4E-
4)0.741.46:0.00366)0.748.48:0.00356,UltB4440:0.0113)0.810.58:0.00729,((Ul
tB4436:0.015,GeoPicke:5.3E-
4)0.458.16:0.01025,UltB4433:0.02275)0.833.70:0.0017)0.884.93:0.00744)0.37
9.6:5.4E-4,(((UltGeoba:0.11374,GeoMetal:5.5E-
4)0.900.76:0.00327,(GeoSulfu:0.0,IroBac13:0.0):0.02667)0.444.18:0.00127,(
((UltB4431:0.0,IroBac12:0.0):5.5E-
4,(UltAcid5:0.04776,(GeoPelop:0.0242,GeoArgil:0.01092)0.526.8:0.00352)0.8
22.44:0.00428)0.857.84:0.00722,(UltB4434:0.01144,UltB4437:0.01499)0.754.5
4:0.00397)0.769.66:0.00378)0.914.97:0.01153)0.986.53:0.02464,(((UltB4447:
0.01124,(PbrAcety:0.0074,(PbrVenet:0.00741,PbrCarbi:0.00743)0.081.8:5.4E-
4)0.094.3:5.4E-4)0.830.54:0.00369,(UltB4442:0.01912,UltPelob:5.5E-
4)0.959.72:0.01119)0.937.76:5.4E-
4,GthEhrli:0.03138)0.379.7:0.00366)0.776.70:0.00402)0.707.16:5.5E-
4,((GlkSubte:0.01114,GlkFerri:5.4E-
4)0.979.62:0.04845,DenBact7:0.06758)0.779.66:0.00921)0.822.45:0.01682,(Dr
nMichi:0.00833,(DrnAcete:5.4E-
4,(UltDes11:0.02489,UltDes10:0.06741)0.725.25:0.00245)0.909.72:0.01108)0.
720.19:0.0024)0.875.88:0.03049)0.994.74:0.05626,((((UltDe106:0.02464,(((U
ltDe105:0.01457,(UltB4502:0.00245,UltDe107:0.00881)0.535.10:0.00752)0.823
.50:5.4E-
4,(Ult14868:0.06913,Ult27575:0.03175)0.967.57:0.04676)0.777.69:0.00392,(U
ltB4501:0.03946,UltDe104:0.0157)0.773.67:0.00319)0.787.58:0.00637)0.918.8
4:0.0245,(Ult13063:0.05472,(UltB4416:0.0303,UltB4415:0.04205)0.817.39:0.0
1259)0.987.78:0.04464)0.940.79:0.02431,(((UltB4504:0.04626,((UltDel86:0.0
9575,UltB4507:0.0338)0.799.67:0.0285,(((UltB4421:0.00372,(UltB9215:0.0524
6,UltB9259:0.0078)0.786.71:0.00331)0.994.75:0.03852,(UltB4419:0.01678,((U
ltB4418:0.01545,((UltSulf6:0.03972,(UltDel61:5.4E-
4,UltB4420:0.00746)0.773.68:0.00389)0.856.71:0.00739,(UltB9260:0.00764,Ul
tDesu8:0.01926)0.765.51:0.00364)0.870.65:0.00738)0.745.54:0.00391,DsfAnil
i:0.03179)0.783.74:0.00664)0.776.71:0.01097)0.748.49:0.00571,UltDel85:0.0
0728)0.908.96:0.02106)0.636.7:0.01551)0.772.69:0.00782,(((UltDe111:0.0074
2,UltDe112:5.5E-
4)0.966.84:0.03821,DrcBaars:0.06809)0.732.21:0.00668,(UltDel83:0.03756,((
UltDe144:0.06745,(Ult17233:0.01365,(((Ult17227:0.0103,(Ult17230:0.00379,(
(Ult17228:0.0037,Ult17229:5.5E-4)1.000.575:5.3E-
4,(Ult17231:0.00386,Ult17232:0.01112)0.644.10:0.01134)0.776.72:0.00344)0.
990.67:0.04397)0.881.95:0.01922,(Ult17222:0.01939,(UltGram6:0.03951,Ult17
223:0.0077)0.769.67:0.00394)0.863.94:0.01409)0.813.54:0.00965,(Ult17234:0
.01431,(Ult17226:0.02808,(Ult17225:0.01555,(Ult17224:0.02012,UltDe222:0.0
1456)0.990.68:0.03324)0.501.9:0.01218)0.797.50:0.00647)0.591.10:0.00768)0
.852.75:0.02236)0.928.66:0.04741)0.978.50:0.05299,(UltSyntr:0.0286,(UltB4
803:0.13803,UltAc745:0.0273)0.958.82:0.0354)0.875.89:0.01699)0.889.82:0.0
1489)0.750.41:0.00446)0.842.80:0.00996)0.867.78:0.01076,(((UltB4474:0.043
34,(UltDel88:0.04006,UltB4500:0.06767)0.791.56:0.01818)0.847.94:0.01802,(
(((PshgggY5:0.04952,UltB4424:0.02076)0.995.81:0.06424,(UltB4373:0.06744,(
((((UltDel63:0.01924,(DsvAlkal:0.04626,DetProte:0.02261)0.943.75:0.03293)
0.889.83:0.03025,UltB4372:0.04452)0.328.9:0.01523,((DlbMedit:5.4E-
4,UltB4368:0.02285)0.042.5:0.01186,(((UltB4365:0.02716,((DlbSpeci:0.00746
,UltB4369:5.4E-4)1.000.576:5.4E-
4,((UltDesu3:0.00742,(DlbElong:0.00358,(UltB4366:0.01893,UltB4367:0.00378
)0.523.6:0.00751)0.945.67:5.4E-

4)0.940.80:0.00745,((UltDel58:0.01495,UltDel59:0.03112)0.970.65:5.5E-
4,((UltB4362:0.03562,(UltB4370:0.0,UltB4371:0.0):0.0189)0.014.5:5.4E-
4,(UltB4363:0.0,Ult27566:0.0):0.0037)0.845.81:0.0037)0.035.8:5.4E-
4)0.838.70:0.00363)0.927.86:0.00753)0.913.90:5.3E-
4,(UltB4364:0.01109,DlbPropi:0.00396)0.249.12:0.00369)0.859.78:0.00714,Dl
bRhabd:0.00401)0.865.88:0.01121)0.942.77:0.02027)0.773.69:0.00487,(UltDel
62:0.03071,UltDel60:0.0085)0.931.79:0.02041)0.862.79:0.0182,(((((DftArcti
:5.4E-
4,DftPsych:0.00369)0.933.86:0.01162,(UltDel50:0.00369,UltDel51:5.5E-
4)0.948.83:0.01452)0.588.9:5.5E-
4,((UltB4354:0.0201,(((UltB4349:0.00745,((DesVacuo:0.04513,UltDel52:0.008
03)0.739.35:0.00324,((UltDel46:0.01223,DesSinga:0.00692)0.895.99:0.01236,
(UltB4343:0.00186,(UltB4344:5.2E-
4,(AtPYy085:0.05786,UltB4345:0.01521)0.070.5:0.01196)1.000.577:0.08874)0.
842.81:0.01355)0.850.85:0.00794)0.909.73:5.3E-
4)0.933.87:0.00739,(UltDesu2:0.01519,(UltB4351:0.01138,(DsfCorro:0.01145,
UltB4359:0.00744)0.853.66:0.00758)0.874.104:0.00753)0.849.74:5.4E-
4)0.903.80:0.00739,(UltDel47:5.5E-
4,(((UltDel54:0.015,(UltHydr5:0.00382,UltDesul:0.01113)0.561.12:0.00367)0
.778.68:5.5E-
4,(((UltB4347:0.02334,(UltBa765:0.04329,UltB4350:0.00484)0.873.86:0.01464
)0.751.38:0.00352,((((DspThioz:5.5E-
4,UltB4356:0.01907)0.812.51:0.00368,((UltB4358:0.0,UltSulfa:0.0):5.5E-
4,UltB4357:0.00368)0.950.78:5.5E-
4)0.922.112:0.01452,UltDel55:0.01048)0.881.96:0.00126,BacROME9:5.5E-
4)0.905.77:0.00744)0.550.4:5.4E-
4,(UltB4360:0.01902,(DspSulfe:0.00746,UltDel56:0.02288)0.639.14:0.00372)0
.612.13:5.5E-
4)0.883.97:0.00744)0.907.81:0.00752,(UltB4352:0.01889,UltB4348:0.00713)0.
904.84:5.5E-4)0.779.67:5.5E-4)0.918.85:5.4E-
4)0.944.63:0.01689)0.801.63:0.00142,((UltB4346:0.01521,(((UltDel57:0.0032
4,UltB4361:0.03699)0.951.75:0.02433,DsfCatec:5.4E-
4)0.827.57:0.00358,((OlvCras3:0.02295,UltB4353:0.01408)0.912.111:0.01394,
UltDel53:0.00756)0.859.79:0.00763)0.780.84:5.5E-
4)0.929.76:0.00768,(OlvAlga2:0.01895,(DlsGlyco:0.02279,UltB4355:0.00579)0
.751.39:0.00521)0.762.75:0.00529)0.936.77:0.00178)0.967.58:0.01559)0.885.
74:0.01455,(UltDel48:0.00364,UltDel49:0.01125)0.975.56:0.02918)0.808.57:0
.0166,UltB9221:0.08651)0.915.83:0.02794)0.894.85:0.01733)0.932.78:0.0258)
0.694.11:0.00312,(UltDel84:0.04356,(UltB4384:0.02217,(UltDel70:0.03153,(U
ltB4381:0.00971,UltB4802:0.02526)0.277.8:0.00697)0.962.76:0.03595)0.905.7
8:0.0275)0.711.21:0.00556)0.837.54:0.00986,(UltB4374:0.01977,(UltArcha:5.
4E-
4,UltB4375:0.00743)0.622.5:0.00304)0.994.76:0.03789)0.576.11:0.01281)0.95
6.73:0.02802,(((((UltB4287:0.03566,(UltB4288:0.02986,(Sgffff00:0.05422,(U
ltDel25:0.0387,((Sgffff02:0.0,ZnsYyyy0:0.0,Sgffff03:0.0):5.4E-
4,(((UltB4285:5.4E-
4,((UltDel17:0.00379,(BfhgggY3:0.01947,UltB4286:0.01553)0.741.47:0.00327)
0.578.6:0.0037,(UltB4283:5.4E-4,((UltDel15:0.0,UltDel16:0.0):5.4E-
4,((UncUncu4:0.0,UncUncu5:0.0):0.00365,(UltB4284:0.0,UncUncu7:0.0):5.4E-
4)0.882.87:0.01121)0.886.109:0.00737)0.994.77:0.02303)0.769.68:5.4E-
4)0.893.89:5.3E-
4,PshgggY3:0.0108)0.122.2:0.0037,(((((((UltDel28:0.01948,Otu00131:0.00356
)0.976.49:0.02371,(Otu00078:0.01443,((UltDel26:0.08672,(Otu00266:0.00385,
(Otu00128:0.01522,UltDel27:0.01543)0.710.20:0.00383)0.730.29:0.00334)0.97

4.44:0.02708,Otu00227:0.00814)0.250.8:0.00382)0.872.90:0.00842)0.798.58:0
.00701,(Otu00053:0.0114,Otu00029:0.0152)0.915.84:5.5E-
4)0.972.67:0.01904,((UncUncu6:0.01514,AtPYy084:0.01959)0.447.5:5.4E-
4,UltDel19:0.02672)0.893.90:0.00769)0.977.52:5.5E-
4,(UltDel24:0.0,UltDel18:0.0,Otu00210:0.0):5.5E-4)0.457.11:5.4E-
4,((UltDel20:0.0,UltDel21:0.0):5.4E-4,(UltDel22:5.3E-
4,UltDel23:0.00742)0.951.76:0.01119)0.822.46:0.00369)0.795.58:0.00382,Otu
00212:0.00371)0.771.80:0.00345)0.891.75:0.00811)0.890.91:5.4E-
4)0.794.57:0.00978)0.996.70:0.05277)0.749.70:0.00793)0.770.75:0.01042,Ult
B4289:0.05346)0.997.84:0.05764,(UltB4294:0.06341,(UltB4223:0.08226,((Ult2
7562:0.00512,Ult27563:0.01833)0.978.51:0.03152,(Ult27560:0.03297,(UltB432
3:0.00746,(UltB4324:0.0,Ult27557:0.0,Ult27561:0.0):5.5E-
4)0.910.85:0.01473)0.742.37:0.0115)0.967.59:0.05468)0.907.82:0.03676)0.80
9.56:0.02631)0.778.69:0.00852,(((((((DfvFerro:5.4E-
4,(DfvSpec9:0.02063,(DfvBizer:0.06616,DfvSenez:0.0198)0.491.5:0.01503)0.9
59.73:0.03165)0.947.66:0.03376,(UltDe140:0.26115,UltDe141:0.06937)0.206.6
:5.5E-
4)0.790.66:0.012,((((((UltOrg34:0.07103,(DfvLongu:0.00612,(DfvGraci:0.007
47,(DfvCapi2:5.5E-
4,DfvCapil:0.00369)0.771.81:0.00369)0.869.72:0.00889)0.912.112:0.02162)0.
919.108:0.02375,((DfvSpec5:0.00798,(DfvProfu:0.02615,((DfvHalop:0.00368,D
fvOxycl:5.5E-4)1.000.578:0.05311,(((DfvSpec6:0.00368,DfvDechl:5.5E-
4)0.769.69:0.00647,(DfvTunis:0.02058,DfvAespo:0.0233)0.192.7:0.00694)0.92
4.86:0.01963,(UltB4597:0.00632,UltDe139:0.00862)0.920.100:0.0157)0.739.36
:0.00518)0.418.7:0.00969)0.987.79:0.04463)0.105.9:0.01705,(DfvSalex:0.029
45,(((DfvFerri:5.4E-
4,((DfvSpec4:0.01084,DfvFrigi:0.012)0.453.9:0.01145,UdnBac20:0.00275)0.65
4.8:0.00121)0.777.70:0.00422,(DfvHydro:0.00127,DfvZoste:0.00273)0.966.85:
0.02382)0.897.76:0.01083,DfvBasti:5.4E-
4)0.877.99:0.02257)0.400.8:0.01195)0.939.67:5.4E-
4)0.888.97:0.01313,(DfvAmino:0.00368,DfvSpec7:5.5E-
4)0.991.68:0.03651)0.882.88:0.01619,(DfvIdaho:5.4E-
4,(UltB4598:0.019,DfvMexic:5.4E-
4)0.965.63:0.02301)0.946.75:0.01688)0.875.90:0.01399,((UltB4567:0.03501,(
UltB4563:5.4E-
4,UltB4564:0.01531)0.884.94:0.01271)0.844.82:0.01227,(UltB4565:0.03956,Ul
tB4566:0.05355)0.792.76:0.01407)0.944.64:0.02218)0.776.73:5.5E-
4,(((UltDe137:0.05547,((DfvGigan:0.01682,(DfvMarin:0.01818,DfvGabon:0.018
54)0.826.51:0.02049)0.937.77:0.02883,(DfvGigas:0.01492,DfvPaque:5.5E-
4)0.994.78:0.06296)0.799.68:0.01357)0.770.76:0.0067,((DfvVietn:0.03955,(U
ltB4593:0.02121,(UltB4590:0.01325,(UltB4591:0.01018,UltB4592:0.01835)0.82
4.53:0.00944)0.736.37:0.00793)0.899.91:0.01384)0.931.80:0.02383,((DfvInop
i:0.05585,(DfvPsych:0.01176,((DfvAcryl:0.0,DfvDesu2:0.0):5.5E-
4,UltB4589:0.08276)1.000.579:0.06726)0.950.79:0.03391)0.452.12:0.00808,((
((((((UncUnc23:0.03006,(UltDe136:0.02068,(UltDe134:0.01224,Otu00395:0.014
78)0.982.51:0.02579)0.272.8:5.3E-
4)0.978.52:0.02521,P0114612:0.00353)0.769.70:0.00396,((Kty00003:0.0,Kty00
004:0.0):0.03223,UltB4588:0.00314)0.871.88:0.00817)0.821.48:5.5E-
4,(((PshgggY6:0.00571,UltDe135:0.01427)0.862.80:0.01107,UncUnc24:0.05975)
0.902.94:0.02401,((((S0114732:0.0,PahggY10:0.0,PshgggY7:0.0):5.4E-
4,UltPro56:0.01503)0.811.51:0.00743,((PahggY11:0.01537,(UncUnc20:0.00373,
(UncUnc21:0.01108,UltDe133:5.4E-
4)0.905.79:0.01111)0.904.85:0.00759)0.777.71:0.00368,UncUnc22:0.00748)0.7
91.57:0.00367)1.000.580:5.5E-4,UltDes28:0.01126)1.000.581:5.4E-

4)0.798.59:0.00323)0.998.95:0.04044,UltDes29:0.0072)0.928.67:0.02094,((DfvCunea:0.01536,((((Otu00082:0.02715,Otu00188:0.00818)0.436.10:0.0078,((UltDe129:0.0,Otu00463:0.0):5.5E-4,UltDe130:0.00369)0.987.80:0.02784)0.882.89:0.01166,(UltDes23:0.021,(((UltDes26:0.01158,UltDe127:0.03619)0.854.77:0.00742,(UncUnc17:0.02329,BfhgggY6:0.01931)0.750.42:0.00389)0.906.99:0.00774,((UltDes25:0.0105,(BfhgggY5:0.0038,(Cuiiii12:0.03138,((UncUnc18:5.5E-4,UncUnc19:5.4E-4)0.985.59:0.03114,PahggYy7:0.01054)0.147.7:0.00491)0.738.31:5.5E-4)0.866.80:0.01016)0.792.77:0.00584,UltDe132:0.02354)0.767.59:0.00392)0.715.17:0.00191)0.937.78:0.01644)0.796.51:0.0044,((Otu00366:0.01989,(((UltDes24:5.4E-4,(UltB4587:0.01917,DfvSpec3:0.00741)0.949.80:0.01523)0.790.67:0.00367,((PahggYy8:0.0,PahggYy9:0.0):0.03116,(PahggYy6:0.01121,UltB4586:5.5E-4)0.919.109:0.00742)0.084.7:5.4E-4)0.860.57:0.00756,UltDe131:0.01143)0.836.56:0.00723)0.864.79:0.00783,UltDes27:0.01138)0.921.97:5.5E-4)0.858.79:0.01123)0.865.89:0.01132,UltDe128:0.03712)0.816.57:0.00951)0.636.8:0.00404,DfvLitor:0.05225)0.997.85:0.05816,((((((DfvLongr:0.00759,((DfvVulga:0.03643,BacS9552:0.02372)0.962.77:0.02384,(DfvVulg2:0.0,Otu00151:0.0):5.4E-4)0.410.9:0.00373)0.768.55:0.00636,(UltDe138:0.00711,UncUnc26:0.00798)0.991.69:0.03144)0.210.12:0.00649,(((UltPro55:5.5E-4,Otu00189:0.00369)0.913.91:0.01496,(UltDe119:0.00296,((UltDe120:0.0,Otu00279:0.0):0.02396,UncUnc11:0.00723)0.905.80:0.01716)0.937.79:0.0201)0.851.79:0.00908,(((UncUnc10:0.00689,UltDes16:0.01516)0.902.95:5.4E-4,(UltB4548:0.01515,AtPYy086:0.14727)0.949.81:5.5E-4)0.861.84:0.01209,(UltRum18:0.03551,(UltB4545:0.00368,((UltDes14:0.0,UltB4546:0.0):5.5E-4,UltDes13:0.00371)0.987.81:5.5E-4)0.985.60:0.04252)0.965.64:0.02977)0.905.81:0.01597)0.899.92:0.01855)0.891.76:0.01199,(UltB4547:0.02845,((((BrpSpeci:5.5E-4,UltB4526:0.00369)0.472.9:5.5E-4,((UltB4528:0.0037,(UltB4529:0.00741,(UltB4530:0.0,UltB4527:0.0):5.5E-4)0.044.5:5.4E-4)0.918.86:0.00754,(BacteNe3:0.01907,UltB4531:0.01508)0.863.95:5.4E-4)0.851.80:0.00372)0.794.58:0.00376,((UltB4523:0.0,UltB4524:0.0,UltPro53:0.0,Otu00056:0.0):0.00755,(UltB4525:0.00744,((((UltB4544:0.01937,(((UltB4534:0.0,UltB4537:0.0,UltB4538:0.0,UltB4543:0.0,UltB4542:0.0,UltB4535:0.0):5.5E-4,((UltB4539:0.01126,UltB4536:0.04363)0.275.9:0.00369,UltB4541:0.02264)0.897.77:5.4E-4)0.291.9:5.5E-4,UltB4540:0.0151)0.984.54:0.02358)0.761.59:0.00385,DlZntaZn:0.0037)0.883.98:0.00781,(UltDe117:0.0037,Otu00132:0.00375)0.884.95:0.00763)0.867.79:0.00772,(UltDe118:0.00751,(UltDe116:0.01164,(UltPro54:5.4E-4,(UltDes15:0.0037,(UltDe115:0.00369,Otu00126:5.5E-4)0.770.77:5.5E-4)0.916.97:0.01491)0.484.7:0.00749)0.856.72:0.00762)0.773.70:0.00371)0.750.43:0.00376)0.789.59:0.00389)0.770.78:0.00375)0.825.54:0.00415,((DfvSpec2:0.01004,(UltB4532:0.05097,UltB4533:0.02109)0.852.76:0.01439)0.876.71:0.0121,(BacNLA44:0.00363,(BacNLA46:0.00368,(UltDes12:5.5E-4,((BacNLA43:0.0,BacNLA45:0.0,DfvDesul:0.0):5.5E-4,BacNLA47:0.00425)0.391.10:5.5E-4)0.294.6:5.5E-4)0.602.8:5.4E-4)0.936.78:0.01607)0.897.78:5.4E-4)0.855.78:0.01016)0.892.77:0.00783)0.713.25:5.5E-4,((LawIntra:0.04432,(UltB4561:0.05957,((UltB4558:5.5E-4,(((UltB4553:0.0,UltB4560:0.0,UltB4559:0.0,UltB4556:0.0,UltB4555:0.0,Ult

B4557:0.0):5.5E-4,((UltB4550:0.02643,UltB4562:0.03874)0.955.58:5.4E-
4,UltB4549:0.00369)0.461.7:5.5E-4)0.000.580:5.5E-
4,(UltB4552:0.00369,UltB4554:0.01492)0.959.74:5.5E-4)0.626.14:5.5E-
4)0.271.5:5.4E-4,UltB4551:5.5E-4)0.967.60:5.4E-
4)0.670.11:0.00832)0.984.55:0.03975,(((UncUnc14:0.01519,(UltDes20:5.4E-
4,UltDe121:0.053)0.109.8:0.00371)0.776.74:0.00631,UltDe122:0.00532)0.964.
74:0.0332,((UncUnc15:0.01634,(((Otu00754:0.01475,(UltB4585:0.01697,(UltDe
125:0.01938,Otu01770:0.02009)0.906.100:0.02717)0.892.78:0.02769)0.919.110
:0.0246,((UncUnc16:0.05505,(UltDe124:0.01075,((UltDe126:0.0,Otu00448:0.0)
:0.02344,Z0114650:0.01297)0.940.81:0.02519)0.793.63:0.01131)0.959.75:0.02
815,(UltDe123:0.03982,(UltDes22:0.01042,(Try00000:0.01529,Otu00623:0.0277
8)0.983.48:0.0297)0.763.75:0.0075)0.876.72:0.01807)0.814.41:0.00793)0.748
.50:0.00547,UltDes21:0.00439)0.882.90:0.02469)0.978.53:0.04004,BfhgggY7:0
.02597)0.955.59:0.03267)0.118.5:0.0045)0.943.76:0.02884)0.939.68:0.01598,
(UltB4596:0.0485,(UltB4594:0.00217,UncUnc25:0.03761)0.999.117:0.07071)0.7
45.55:0.0054)0.735.33:0.00419)0.444.19:0.01732)0.786.72:0.00808)0.310.7:0
.00679)0.744.45:0.01078,((DmcOrale:0.01837,((DmcBacul:0.00373,((BacEnr56:
5.5E-4,(DmcSpeci:0.01938,DmcMaces:0.00369)0.881.97:5.5E-4)0.755.54:5.5E-
4,DmcTherm:0.04363)0.776.75:5.3E-4)0.891.77:5.4E-
4,(DmcEscam:0.01147,(UltB4609:5.3E-
4,UltB4608:0.00727)0.524.7:0.00383)0.905.82:0.01407)0.532.11:0.00764)0.98
6.54:0.03988,((DfvAlkal:0.02679,((UltB4605:0.05889,(DfvPutea:5.5E-
4,UltB4604:0.04758)0.924.87:0.02845)0.911.100:0.02591,((UltB4600:0.03224,
(((((UltB4599:0.03241,(DfvCarbi:0.00371,DfvMarra:5.5E-
4)0.830.55:0.00365)0.418.8:5.4E-
4,DfvFruct:0.02281)0.895.100:0.00757,DfvMagne:0.00361)0.745.56:0.00377,Df
vAlcoh:0.00763)0.834.50:0.00917,UltB4603:0.06009)0.873.87:0.01102)0.072.4
:0.01162,(UltB4602:0.00909,DfvSulfo:0.01443)0.928.68:0.02422)0.722.18:0.0
146)0.969.54:0.03349)0.807.47:0.01439,(DfvAfric:0.00268,DfvSpec8:0.03734)
0.958.83:0.02627)0.478.8:0.01602)0.890.92:0.02435)0.888.98:0.03507)0.711.
22:0.02094)0.907.83:0.01639,(DfhRetba:5.4E-
4,DfhRetb2:0.0118)0.923.115:0.03373)0.892.79:0.01993,((UltDe143:0.04775,(
UltOrg35:0.05346,(DfhUtahe:0.00368,DvrHalop:0.01507)0.831.52:0.00768)0.74
9.71:0.00631)0.491.6:0.02144,((DvbBacte:0.02925,((DncAutot:0.02503,DncSub
ma:0.01517)0.962.78:0.03046,(DthOkina:0.00486,DthNapht:0.01852)0.992.67:0
.04708)0.812.52:0.01567)0.568.8:0.01775,((DfpDelic:0.0,DfpThiod:0.0):5.3E
-4,(DnvSpeci:5.5E-
4,DnvHydro:0.01124)0.942.78:0.01888)0.977.53:0.04299)0.878.80:0.01973)0.7
01.19:0.00761)0.688.12:0.01927,(UltB4606:0.00433,(DnrCoope:5.3E-
4,DnrLacus:0.0316)0.952.63:0.01473)0.910.86:0.02192)0.962.79:0.03796,((Ul
tB4458:0.04987,(UltB4454:0.03413,(UltB4455:0.06506,(UltB4456:0.06937,Ult2
4678:0.01715)0.382.10:0.01665)0.720.20:0.00783)0.985.61:0.05227)0.936.79:
0.03292,((((((UltB4213:0.04899,(UltB4217:0.02754,UltB4219:0.04405)0.804.
48:0.01829)0.826.52:0.01954,(UltCyst3:0.0148,UltB4209:5.5E-
4)0.994.79:0.05143)0.761.60:0.00649,((((AnmDehal:0.00529,(UltB4214:5.4E-
4,UltB4215:0.01497)0.990.69:0.03537)0.913.92:0.01538,(UltB4220:0.10316,Ul
tB4218:0.02716)0.207.3:0.00675)0.849.75:0.01123,UltB4221:0.02555)0.000.58
1:5.4E-
4,UltB4216:0.02205)0.876.73:0.01656,(((UltB4201:0.0436,(((UltB4211:0.0364
8,(MxcVire2:5.5E-
4,MxcVires:0.00362)0.867.80:0.00813)0.757.48:0.00344,UltB4208:0.03987)0.7
61.61:0.00386,(((CysMinus:0.0,UltCysto:0.0,UltB4199:0.0,HyaMinut:0.0,StiK
oree:0.0,UltCyst4:0.0):5.5E-4,(((CysGraci:5.4E-
4,(UltB4210:0.00369,UltCyst2:5.4E-

4)0.960.80:0.01502)0.876.74:0.00719,UltB4202:0.03502)0.370.8:5.5E-
4,MxcSpeci:0.0037)0.891.78:5.4E-4)0.768.56:5.5E-
4,((CorCora2:0.0,CorCoral:0.0):5.5E-
4,StiAuran:0.01124)0.810.59:0.00369)0.912.113:0.00737)0.000.582:5.3E-
4)0.000.583:5.3E-
4,UltB4200:0.00369)0.861.85:0.00926,((CysBadiu:0.00683,MltLiche:0.01632)0
.893.91:0.01526,UltB4212:0.05785)0.765.52:0.00901)0.981.61:0.0298)0.753.6
8:0.00425)0.866.81:0.01327,(UltB4247:0.06129,UltDel91:0.14683)0.799.69:0.
02314)0.969.55:0.03402,((Ult27573:0.08407,((UltHydr4:0.03719,Ult27572:0.1
3773)0.990.70:0.09624,((UltMyxo3:0.10302,UltB4506:0.0164)0.902.96:0.03038
,(UltB4266:0.06359,(UltB4503:0.10575,UltDe108:0.03468)0.554.12:0.00524)0.
897.79:0.02974)0.223.4:0.02696)0.719.16:0.01578)0.756.62:0.00888,UltDel87
:0.01742)0.982.52:0.03468)0.800.55:5.4E-
4,(UltB4422:0.01872,(UncUncu9:0.00895,UltB4423:0.03917)0.923.116:0.02765)
0.954.73:0.03021)0.776.76:0.0044,(((((UltB4205:0.02782,Ult16215:0.03075)0
.756.63:0.00699,(UltB4203:0.07187,UltB4204:0.05624)0.948.84:0.03574)0.885
.75:0.02033,(UltB4206:0.05665,UltOrg31:0.06622)0.492.13:0.01147)0.922.113
:0.02723,((UltB4242:0.0681,(UltGam58:0.0705,(UltPolya:0.04457,(((EngSalin
:0.0109,(UltDelt4:0.02337,(MxbcSYR2:0.01164,UltB4240:0.00381)0.806.43:0.0
0373)0.759.77:7.0E-
4)0.817.40:0.00883,PctPacif:0.01345)0.901.85:0.01186,(UltDelt5:0.05656,(U
ltEnhyg:0.06171,((NnnExed2:5.5E-
4,NnnExede:0.08664)0.990.71:0.0452,UltB4241:0.06258)0.795.59:0.01102)0.78
2.77:0.00871)0.800.56:0.00916)0.783.75:0.00919)0.616.9:0.01637)0.817.41:0
.0146)0.931.81:0.03258,(((((HngOchra:0.01258,(HngTepid:0.01693,KofFlava:0
.00985)0.417.13:0.00445)0.918.87:0.01974,(UltB4239:0.02364,(UltB3271:0.02
638,UltB4232:0.01351)0.921.98:0.02371)0.414.9:0.01496)0.917.84:0.01145,((
(((((UltB4235:0.04456,UltB4236:0.01564)0.132.11:0.01967,(UltrSo55:0.00765
,UltB4237:0.02971)0.373.11:0.007)0.404.11:5.3E-
4,(UltB4234:0.01695,UltB4233:0.00214)0.928.69:0.0222)0.964.75:0.03428,Ult
B4238:0.0067)0.955.60:0.03711,UltSlu20:0.01798)0.891.79:0.02418,(UltB4230
:0.01598,(UltB4274:0.02007,(UltB4229:0.01908,UltB4228:5.5E-
4)0.921.99:0.02036)0.959.76:0.02595)0.845.82:0.01216)0.424.12:0.00779,(Ul
tB4231:0.04487,((UltMyxoc:0.00351,UltB4224:0.00771)0.889.84:0.01741,(Ult1
4254:0.1141,(UltB4225:0.01864,UltB4226:0.05885)0.896.74:0.03597)0.384.11:
5.4E-4)0.982.53:0.03472)0.709.15:0.00245)0.908.97:0.01572)0.153.7:5.5E-
4,(UltDelt7:0.04061,(UltDelt3:0.02264,(UltB4227:0.00727,UltB4243:0.01538)
0.989.62:0.0346)0.286.7:0.01157)0.763.76:0.00664)0.282.6:0.01123,(UltB424
4:0.05533,(UltB4245:0.00487,UltAnae4:0.06645)0.934.76:0.03897)0.892.80:0.
02396)0.867.81:0.03007)0.909.74:0.02742)0.936.80:0.01946,((((UltB4517:0.0
5732,(UltOrg33:0.04319,UltDes31:0.06816)0.395.11:0.01991)0.793.64:0.01117
,Ult16214:0.03458)0.845.83:0.01426,(((UltB4515:0.00758,UltB4521:0.01138)0
.991.70:0.03674,(UltB4518:0.04143,UdnBac19:0.02063)0.640.11:0.00249)0.860
.58:0.01156,(((UltB4514:0.03886,UltB4520:0.01261)0.177.10:0.01284,UltB451
6:0.03998)0.222.4:0.00728,(UltB4519:0.05041,UltB4522:0.02804)0.957.64:0.0
2591)0.838.71:0.00935)0.918.88:0.01905)0.880.91:0.01774,(((((UltDel12:5.5
E-
4,UltDel13:0.02299)0.985.62:0.05569,((UltB4269:0.03904,UltB4268:0.00507)0
.998.96:0.06861,(UltB4267:0.03392,UltB4273:0.04704)0.682.16:0.00606)0.946
.76:0.03487)0.291.10:0.02426,(UltB4272:0.09319,UltB4207:0.08088)0.846.91:
0.02649)0.892.81:0.02346,UltPro47:0.07689)0.846.92:0.01133,(((((UltB4249:
0.00983,UltPoly3:0.0394)0.929.77:0.01836,((((UltB4250:0.00753,(UltDelt8:0
.02175,BysCruen:0.02188)0.748.51:0.00685)0.896.75:0.01507,(SorCellu:0.035
74,UltSoran:0.03063)0.229.7:0.01287)0.970.66:5.5E-

4,(UltB4255:0.02367,PtsFlava:0.01116)0.032.3:0.00369)0.934.77:0.02049,Cho
Apicu:0.0068)0.710.21:0.00619)0.820.40:0.01578,(UltDelt9:0.01627,(((UltDe
l10:0.0074,(UltPoly4:0.02519,UltB4263:0.03992)0.456.10:0.00595)0.763.77:0
.00707,UltB4261:0.05419)0.708.23:0.00375,(((UltB4258:0.00383,(UltB4265:0.
02334,UltB4262:0.00342)0.950.80:0.01556)0.462.12:0.00375,(UltB4260:0.0148
4,UltB4259:0.01913)0.754.55:0.00481)0.844.83:0.01183,UltB4264:0.04923)0.9
10.87:0.01722)0.958.84:0.0323)0.852.77:0.02814)0.794.59:0.01275,((UltB425
6:0.06907,UltB4282:0.0306)0.150.5:5.3E-
4,(((UltB4254:0.02889,(UltPoly2:0.02567,(UltB4251:0.0066,Ult11740:0.05049
)0.373.12:0.0148)0.937.80:0.02891)0.737.50:0.0062,((UltPro45:0.01982,UltB
4252:0.06717)0.784.67:0.01373,UltB4253:0.0173)0.311.6:0.01186)0.981.62:0.
03419,(UltDel11:0.11094,(UltPoly5:0.00697,(UltB4270:0.00744,UltB4271:5.5E
-
4)0.973.52:0.03301)0.923.117:0.02075)0.660.10:0.00658)0.725.26:0.00395)0.
867.82:0.01532)0.811.52:0.013,(Ult16884:0.07974,UltB4257:5.4E-
4)0.961.69:0.03408)0.942.79:0.02511)0.835.65:0.01143)0.749.72:0.00348)0.8
90.93:0.0081)0.842.82:0.00192)0.937.81:0.02935)0.330.8:0.00844,(UltDelt6:
0.04005,(UltDel97:0.06626,UltB4457:0.03362)0.998.97:0.09546)0.367.13:0.02
175)0.819.50:0.01005)0.882.91:0.01141,((UltOrg32:0.07813,(UltB4248:0.0529
,UltB4376:0.08764)0.851.81:0.01835)0.105.10:0.00919,(UltB4414:0.02567,(Ul
tPro52:0.0197,(DmnTiedj:0.00839,(UltB4497:0.01677,DmnLimim:0.02824)0.303.
10:0.00631)0.702.12:0.0111)0.924.88:0.02543)0.841.64:0.01084)0.864.80:0.0
1407)0.725.27:0.00335)0.879.89:0.00825)0.758.68:0.00386)0.848.70:0.00694,
(((UltB4413:0.07374,(UltDe110:0.02206,UltDe109:0.00894)0.931.82:0.03087)0
.484.8:0.02358,(UltB4499:0.05558,UltB4498:0.03012)0.836.57:0.0153)0.838.7
2:0.01351,((UltrSo57:0.12643,(UltB4471:0.01379,(((((UltB4464:5.5E-
4,UltB4466:5.5E-
4)0.967.61:0.02505,((DlvAdipi:0.02109,UltB4460:0.04256)0.833.71:0.01485,(
((DldAmnig:5.5E-
4,((UltB4463:0.00402,(UltB4461:0.01436,(SynWolin:0.02308,UltB4462:0.01004
)0.745.57:0.0039)0.952.64:0.01882)0.904.86:0.01416,UltB4468:0.01819)0.739
.37:0.01831)0.860.59:0.01589,(UltB4469:0.06188,(SynFumar:5.5E-
4,UltB4467:5.5E-
4)0.753.69:0.0038)0.935.80:0.01579)0.779.68:0.00646,(UltB4465:0.0,SynSulf
a:0.0):0.01784)0.672.13:0.00298)0.815.55:0.01033)0.815.56:0.01187,(DcmHyd
ro:0.04827,UltDel98:0.03859)0.830.56:0.01254)0.829.62:5.4E-
4,(UltB4459:0.10843,(TrlNorve:0.0539,DgbAlkan:0.03009)0.825.55:0.01696)0.
475.12:0.00474)0.899.93:0.02335,UltB4470:0.04237)0.892.82:0.02495)0.364.1
0:0.01807)0.903.81:0.02819,UltB4472:0.01847)0.976.50:0.03335)0.731.24:0.0
0652)0.754.56:0.00649)0.797.51:0.01035,(((UltB4613:0.07283,UltDe145:0.119
52)0.991.71:0.07789,(UltB4275:0.08887,(((UltB4331:0.09868,((UltB4336:0.09
605,OlvIlva3:0.02059)0.804.49:0.01156,((UltBdel3:0.12685,(((BdlBact2:0.0,
BdlBacte:0.0):0.02148,(BdlSpec2:0.05019,UltB4328:0.02517)0.729.20:0.00423
)0.775.71:0.01044,(UltB4327:0.03568,(UltB4329:5.3E-
4,(BdlSpec3:0.05274,UltB4330:0.00731)0.810.60:0.01145)0.970.67:0.03605)0.
668.18:0.02695)0.884.96:0.0292)0.847.95:0.02693,(UltPro50:0.0502,(UltB433
5:0.0145,UltBdel4:0.0225)0.998.98:0.05217)0.859.80:0.0113)0.923.118:0.023
18)0.892.83:0.01934)0.765.53:0.01423,UltB4332:0.02848)0.977.54:0.04946,(U
ltB4334:0.10974,((OlvIlva2:0.13068,UltDel45:0.12175)0.770.79:0.03775,UltB
4333:0.07054)0.798.60:0.02711)0.768.57:0.00951)0.927.87:0.04088)0.734.42:
0.0131)0.841.65:0.0149,(((UltDe103:0.0238,((((UltB4478:0.00598,(SnpGenti:
5.5E-4,(SnpBuswe:5.5E-
4,UltDe100:0.01496)0.917.85:0.00736)0.423.10:0.00951)0.979.63:0.03078,((U
ltDe102:0.04113,UltSynt2:0.00986)0.834.51:0.01103,(((((UltB4491:0.00782,U

ltB4484:0.03349)0.631.11:0.0057,(SmiPropi:0.00454,((UltB4486:0.0,UltB4487
:0.0):0.01061,UltDe101:0.00833)0.941.73:0.01561)0.749.73:0.00494)0.702.13
:0.00166,(UltB4485:0.0037,UltB4488:0.01894)0.767.60:0.00374)0.877.100:0.0
0766,UltB4489:0.02731)0.748.52:0.00371,(UltB4481:0.00373,(UltB4479:0.0189
7,UltB4480:0.0111)0.862.81:5.3E-
4)0.938.51:0.01136)0.713.26:0.00251)0.855.79:0.01187)0.674.15:0.00893,(Ul
tB4496:5.5E-4,(UltB4322:0.00372,Ult27564:0.00372)0.635.7:5.5E-
4)0.962.80:5.4E-
4)0.875.91:0.01248,(UltPl521:0.07198,Ult27565:0.04919)0.575.4:0.01339)0.9
71.57:0.03698)0.742.38:0.00417,UltB4494:0.02253)0.867.83:0.01358,((UltB44
90:0.05208,UltB4492:0.04201)0.752.63:0.0107,(UltB4337:0.1181,(UltB4194:0.
01465,(UltrSo53:0.1527,UltDelt2:0.23511)0.955.61:0.08023)0.938.52:0.03385
)0.936.81:0.03309)0.195.5:0.01286)0.832.60:0.01054)0.933.88:5.4E-
4)0.986.55:0.03545,(UltB4475:0.03417,(UltB4476:0.04757,UltDel99:0.02712)0
.750.44:0.00925)0.968.67:0.03049)0.397.9:0.0042,UltB4477:0.05143)0.783.76
:0.0076,((((DeuHalop:0.03392,DeuSpeci:0.02668)0.987.82:0.05228,(BfhgggY4:
0.04468,(UltB4411:0.02969,DfsSapov:0.0202)0.836.58:0.01764)0.783.77:0.011
54)0.929.78:0.02417,((((UltDel82:0.02864,(DsfVacuo:0.0059,(DsfAutot:0.011
24,UltDel80:5.4E-
4)0.894.86:0.02112)0.862.82:0.01507)0.886.110:0.01628,(UltB4409:5.3E-
4,(((UltB4407:0.01176,((DlpJoerg:0.02643,(UltDel79:0.01223,((DbcSpeci:0.0
0369,DbcVibri:5.5E-4)0.905.83:5.4E-4,((DbcPostg:0.00369,UltDesu6:5.5E-
4)0.910.88:5.3E-4,(((UltB4401:0.0,UltB4402:0.0,DbcCurva:0.0):5.5E-
4,DbcSpec2:0.0037)1.000.582:5.4E-4,(DbcHalot:5.5E-
4,DbcLatus:0.00369)0.822.47:0.00365)0.961.70:0.01505)0.889.85:0.00738)0.9
55.62:0.01836)0.835.66:0.00772)0.945.68:5.4E-4,(((DfgTolue:5.5E-
4,UltB4403:5.5E-4)1.000.583:5.1E-4,(UltSulf5:5.4E-
4,DfgSpeci:0.02283)1.000.584:0.00317)0.973.53:0.01935,(UltB4405:0.01866,(
DblToluo:5.4E-4,(UltB4406:0.0111,(UltB4192:5.3E-
4,UltB4404:0.02278)0.420.13:0.00386)0.974.45:0.02705)0.830.57:0.00406)0.8
62.83:0.00786)0.179.4:0.00372)0.435.11:0.00646)0.706.20:5.5E-
4,UltB4408:0.01896)0.919.111:0.01157,UltDesu7:0.02627)0.376.5:0.00472)0.8
94.87:0.01463)0.908.98:0.02252,(UltDel81:0.05588,(UltB4410:0.0601,UltB439
9:0.11502)0.801.64:0.0302)0.669.14:0.00924)0.875.92:0.01942,(DfrConse:0.0
4324,((((UltDel78:0.00745,UltB4390:5.4E-
4)0.960.81:0.02647,(UltB4379:5.4E-
4,UltB4392:0.01882)0.987.83:0.03667)0.732.22:0.01397,(DfmMagnu:0.01338,((
DfmIshim:0.01381,(UltB4391:0.01741,UltAc876:0.01848)1.000.585:0.07125)0.8
46.93:0.02163,((UltB4398:0.00301,(((P0114622:0.0,PahggYy5:0.0):5.4E-
4,UltB4397:0.01133)0.777.72:0.00374,PshgggY4:0.01133)0.751.40:0.00444)1.0
00.586:0.06641,(Ult25770:0.00864,(DlcBiacu:0.01088,DlcMulti:0.01464)0.307
.5:0.00466)0.707.17:0.00252)0.850.86:0.0184)0.491.7:0.01196)0.835.67:0.01
38)0.863.96:0.01112,((((UltB4396:0.00397,DffFasti:0.01905)0.458.17:0.0062
4,(DffGelid:5.3E-
4,DffHanse:0.01639)0.997.86:0.04037)0.461.8:0.00777,((((((((UltDel72:0.01
09,DetProt2:0.03151)0.756.64:0.00448,((Ult27574:0.01901,(UltDel65:0.02349
,(UltDel66:0.03091,((OlvCras4:0.00371,(UltDel64:0.00371,UltB4807:0.00746)
0.806.44:0.00371)0.634.9:5.5E-4,UltB4377:5.5E-4)0.984.56:5.4E-
4)0.149.9:0.01122)0.776.77:0.01129)0.867.84:5.5E-
4,(UltB4378:0.00379,UltDel71:0.0193)0.753.70:0.00363)0.867.85:0.00758)0.7
88.64:0.00382,(UltB4382:0.00749,UltDel73:0.00366)0.784.68:0.00379)0.795.6
0:0.00373,UltB4383:5.4E-
4)0.870.66:0.00764,UltB4417:0.0314)0.000.584:5.3E-4,(UltDel74:5.5E-
4,(UltDel75:0.0408,UltDel76:0.04608)0.716.20:0.01801)0.901.86:0.01436)0.8

84.97:0.00767,(UltB4412:0.05209,UltDel77:0.00478)0.828.50:0.00747)0.746.4
9:0.00481,(((UltB4385:0.02237,((UltDel67:0.0,UltDel68:0.0):5.5E-
4,UltB4380:0.00743)0.967.62:0.02231)0.993.59:5.4E-
4,(AgnButyr:0.00904,(((UltB4386:0.0,UltB4389:0.0):0.00816,(DsnSpeci:0.026
7,(OlvCras5:0.01946,(UltDel69:0.04371,DlcOleov:0.02031)0.385.14:0.004)0.4
68.13:0.00408)0.904.87:0.01262)0.789.60:0.0088,(OlvAlga3:5.5E-
4,(UltDesu4:0.00332,(PshggY05:0.03092,DlrOvata:5.3E-
4)0.082.8:0.0037)0.986.56:0.02353)0.918.89:0.01725)0.816.58:0.00849)0.919
.112:0.01203)0.905.84:0.01197,((DfcAliph:0.01501,UltB4388:0.01953)0.886.1
11:0.0078,(UltB4395:5.4E-4,((DsdSpong:5.5E-
4,((DlgOcean:0.00371,UltDesu5:0.00748)0.795.61:5.4E-4,UltB4394:5.5E-
4)0.934.78:0.00368)1.000.587:5.5E-
4,DlgFragi:0.00741)1.000.588:0.03519)0.925.102:0.01184)0.859.81:5.4E-
4)0.922.114:0.01456)0.779.69:0.0045)0.726.20:0.00156,((DshButyr:0.03942,U
ltB4387:0.0164)0.875.93:0.02011,(DsfIndol:0.04076,(OlvIlva4:0.02747,UltB4
393:0.00357)0.709.16:0.01311)0.987.84:5.4E-
4)0.946.77:0.00182)0.879.90:0.01043)0.551.10:0.0094)0.495.8:0.00413)0.860
.60:0.01155)0.856.73:0.014,(UltB4400:0.02274,DtfOlefi:0.02411)0.859.82:0.
01221)0.858.80:0.01682)0.945.69:0.02847)0.487.13:0.00164)0.879.91:0.0081)
0.766.62:0.00645)0.907.84:0.02064)0.378.10:0.01032)0.819.51:0.01488,(((((
(((((Ult13303:0.04174,(UltGem45:0.01893,UltGem46:0.00859)0.969.56:0.03102
)0.718.19:0.00555,(BacEll13:0.03164,Ult13302:0.02849)0.994.80:0.0579)0.97
5.57:0.03457,((Ult13313:0.00245,(Ult13305:5.4E-
4,Ult13304:0.00738)0.995.82:0.03265)0.952.65:0.02093,(((UltGem47:0.01169,
BacEll14:0.01504)0.774.78:0.00371,((UltrS158:0.02368,((Ult13301:0.04135,(
((UltGem43:5.5E-
4,(Ult13299:0.02915,(Ult13297:0.01753,Ult13298:0.04203)0.025.3:0.00669)0.
977.55:0.02899)0.252.5:0.02162,(Ult13293:0.01035,(Ult13295:0.00383,Ult132
94:0.00383)0.856.74:0.00176)0.851.82:0.01549)0.999.118:0.04852,Ult13300:5
.3E-
4)0.720.21:0.00287)0.985.63:0.02751,Ult13306:0.00388)0.737.51:0.00283)0.9
08.99:0.01189,Ult13307:0.04352)0.852.78:0.00738)0.833.72:0.00951,(UltGem4
4:0.03145,((UltGem55:5.3E-4,((((BacEll15:0.00366,Udntddd3:5.5E-
4)0.898.102:0.00817,(Ult13344:5.3E-4,(Ult13343:5.5E-4,(Ult13341:5.5E-
4,(UltGem68:0.0037,(Ult13342:0.00369,UltGem69:5.5E-4)0.410.10:5.5E-
4)0.827.58:0.00368)0.433.14:0.00752)0.984.57:0.01507)1.000.589:0.06701)0.
987.85:5.2E-4,(Ult13323:5.5E-4,(Ult13314:5.5E-4,((((UltGem51:5.5E-
4,UltGem52:0.0036)0.824.54:0.00354,((Ult13315:0.0,UltrS160:0.0,UltGem54:0
.0,Ult13317:0.0,Ult13319:0.0):5.5E-
4,(Ult13321:0.01088,Ult13320:0.00738)0.982.54:5.1E-4)1.000.590:5.4E-
4)0.938.53:0.01103,(UltGem49:0.00682,(Ult13316:0.02583,UltGem53:0.04029)0
.997.87:0.06261)0.763.78:5.5E-4)0.863.97:5.5E-4,UltGem56:5.5E-
4)0.118.6:5.5E-4)0.610.7:5.4E-
4)0.933.89:0.00276)0.924.89:0.01156,((UltGem57:0.0,UltGem58:0.0):0.00348,
Ult13324:0.00774)0.171.11:0.00371)0.894.88:5.5E-
4)0.775.72:0.00317,Ult13322:0.00818)0.985.64:0.03936)0.909.75:0.02187)0.8
57.85:0.01238)0.768.58:0.00596)0.751.41:0.0089,(((((Ult13308:5.3E-
4,((Ult13310:0.0,Ult13311:0.0):5.5E-
4,Ult13312:0.06073)0.875.94:0.00716)0.736.38:0.00356,Ult13309:0.00765)0.9
79.64:0.01904,UltGem48:5.4E-
4)0.990.72:0.03584,(Ult13387:0.00317,(((Ult13384:5.5E-
4,UltGem77:0.0037)0.839.69:0.00369,(Ult13385:5.5E-4,Ult13386:5.5E-
4)0.484.9:5.5E-4)0.158.6:5.5E-
4,Ult13388:0.00741)0.995.83:0.03227)0.905.85:0.01678)0.871.89:0.01721,(((

(((UltGem67:0.02082,((UltrS159:0.01706,Ult13327:0.0115)0.260.7:0.01495,(U
lt13326:0.01857,(Ult13325:0.00377,(UltGem50:0.0036,UltGem59:0.02348)0.773
.71:0.00387)0.867.86:0.00986)0.787.59:0.01115)0.766.63:0.00688)0.877.101:
0.01025,((((Ult13361:0.03469,DenBac28:0.01304)0.725.28:0.01002,(UltGem71:
0.01691,((Ult13358:0.01894,UltrS165:0.00719)0.834.52:5.4E-
4,((((Ult13351:0.0,Ult13352:0.0,Ult13396:0.0):5.5E-
4,Ult13356:0.0037)0.866.82:0.00755,(Ult13357:0.01895,((Ult13353:0.01502,(
(Ult13394:0.01493,Ult13395:5.5E-
4)0.865.90:0.00369,(Ult13349:0.0,Ult13355:0.0,Ult13392:0.0,Ult13393:0.0):
5.5E-4)0.940.82:5.5E-4)0.855.80:0.0037,Ult13350:5.5E-4)0.902.97:5.4E-
4)0.813.55:0.00734)0.909.76:0.01168,Ult13354:0.01511)0.762.76:0.00389)0.9
27.88:0.01442)0.871.90:0.01257)0.763.79:0.01104,(Ult13345:0.02738,(Ult133
40:0.02863,(((Ult13360:0.01989,Ult13359:0.04046)0.862.84:0.01898,Ult13346
:0.06157)0.269.7:5.4E-
4,Ult13362:0.01949)0.942.80:0.01974)0.820.41:0.00852)0.730.30:0.01786)0.8
63.98:0.01657,(((Ult13269:5.3E-
4,UltGem26:0.01488)0.664.10:0.02215,(((Ult13265:0.00371,Ult13266:5.5E-
4)0.747.47:0.0047,(Ult13264:5.4E-
4,UltB4196:0.04318)0.854.78:0.01023)0.935.81:0.02051,((UltGem27:0.0,UltGe
m28:0.0,UltGem29:0.0):0.02952,(Ult13268:0.00936,(Ult13267:5.5E-
4,UltGem25:0.01126)0.785.69:0.00574)0.999.119:0.05783)0.615.14:0.00958)0.
808.58:0.01024)0.923.119:0.01634,((Ult13270:0.0,Ult13271:0.0):0.01758,((U
lt13347:0.0,Ult13348:0.0):0.02305,(UltGem31:0.0083,Ult13272:0.02722)0.579
.12:0.00807)0.890.94:0.01459)0.850.87:0.00891)0.896.76:0.017)0.772.70:0.0
0955)0.870.67:0.0077,(UltGem39:0.00783,(UltGem74:0.01883,UltrS162:5.4E-
4)0.959.77:0.01548)0.746.50:0.0033)0.756.65:0.00369,((((Ult13338:0.01829,
UltrS161:0.02577)0.950.81:0.02951,((Ult13371:0.03663,(Ult13366:5.5E-
4,((Ult13365:0.01515,(Ult13364:0.00369,Ult13369:0.01132)0.440.7:5.3E-
4)0.950.82:0.01468,(Ult13370:0.04397,(Ult13367:0.00374,Ult13368:0.00374)0
.791.58:0.00683)0.774.79:0.00217)0.795.62:0.00647)0.724.21:0.00655)0.867.
87:0.01661,(Ult13373:0.03493,((Ult13374:0.01932,(UltrS163:0.03551,Ult1336
3:0.04205)0.371.11:0.00763)0.916.98:0.02027,Ult13372:0.01571)0.322.11:0.0
0976)0.875.95:0.02072)0.856.75:0.01558)0.910.89:0.02014,(((UltGem66:0.013
4,Ult13275:0.03094)0.912.114:0.01854,Ult13339:0.0045)0.838.73:0.01368,(Ul
tGem73:0.01211,(((((UltGem41:0.00531,(UltGem78:0.0,Ult13391:0.0):0.02995)
0.959.78:0.02328,(Ult13375:0.00951,(Ult13328:0.01997,Ult13390:0.0395)0.91
3.93:0.022)0.422.10:0.00482)0.207.4:0.0074,UltrS164:0.01695)0.877.102:0.0
1701,UltGem72:0.01018)0.627.5:0.00206,(Ult13334:0.02316,(((Ult13337:0.035
54,((((Ult13330:0.00372,Ult13332:5.5E-4)0.703.22:5.3E-
4,GmnAuran:0.00742)0.795.63:0.0045,((Ult13331:0.01105,Ult13336:5.3E-
4)0.708.24:0.00747,(Ult13318:0.00742,Ult13335:5.3E-
4)0.803.48:0.00399)0.633.6:0.00268)0.718.20:0.00784,UltGem60:0.04301)0.75
5.55:0.00399)0.721.17:0.00331,(UltGem61:0.02008,((UltGem64:0.00369,UltGem
65:0.00369)0.905.86:5.4E-
4,((Ult13333:0.0,UltGem70:0.0):0.0115,UltGem63:0.01917)0.723.24:0.00732)0
.951.77:0.02262)0.811.53:0.01154)0.997.88:5.0E-
4,(Ult13329:0.01514,UltGem62:0.01891)0.999.120:5.4E-
4)0.008.3:0.01315)0.878.81:0.01381)0.821.49:0.00887)0.458.18:0.01031)0.74
7.48:0.00202)0.312.10:0.01244,(Ult13381:0.0,Ult13382:0.0):0.01575)0.754.5
7:0.00355)0.901.87:0.00777,(Ult13274:0.08318,((UltGem37:0.00741,(Ult13289
:0.01174,UltGem40:0.01568)0.780.85:0.00364)0.772.71:0.00381,UltGem38:0.00
356)0.956.74:0.0189)0.834.53:5.4E-
4)0.756.66:0.00421,((((((Ult13383:0.15097,(Ult13296:0.13032,UltGem30:0.11
392)0.777.73:0.08159)0.968.68:0.09079,Ult13378:0.0022)0.949.82:5.5E-

4,((Ult13380:0.01048,UltGem76:0.01623)0.861.86:0.00809,((((Ult13285:0.021
76,((Ult13288:0.03418,(UltrS157:0.0225,(Ult13279:0.0,Ult13280:0.0):0.0137
7)0.941.74:0.02263)0.895.101:0.01432,((UltGem34:5.4E-
4,((Ult13277:0.02119,Ult13283:0.02125)0.571.7:0.01091,(UltGem35:0.02619,(
Ult13282:0.00644,(Ult13278:0.00335,Ult13281:5.5E-4)0.903.82:5.3E-
4)0.940.83:0.02433)0.948.85:0.02838)0.927.89:0.02097)0.940.84:0.01334,(Ul
t13276:0.00353,Ult13284:0.02784)0.314.7:0.00675)0.711.23:0.00471)0.647.6:
0.00976)0.787.60:0.00811,(Ult13287:0.03696,(Ult13286:0.10562,UltGem36:0.0
124)0.785.70:0.01378)0.635.8:0.01158)0.981.63:0.04217,Ult13273:0.05775)0.
952.66:0.03268,Ult13379:5.4E-
4)0.841.66:0.00859)0.852.79:0.00817)0.447.6:0.01176,((UltGem42:0.04624,(U
lt13290:0.06462,Ult13291:0.04686)0.958.85:0.03855)0.843.61:0.03764,((Ult1
3292:0.0,Ult13389:0.0):0.04899,(UltGem32:0.05331,UltGem33:0.02686)0.940.8
5:0.0498)0.945.70:0.04684)0.986.57:0.07115)0.756.67:0.00959,(UltFor13:0.0
1108,(Ult13376:5.4E-
4,Ult13377:0.00707)0.999.121:0.06922)0.960.82:0.03536)0.352.8:0.00278,(((
Ult13257:5.5E-
4,Ult13258:0.00368)0.987.86:0.05013,(Ult13259:0.0312,((Ult13249:0.00374,(
UltGemm9:5.4E-4,((((((UltGem14:5.5E-
4,((Ult13254:0.0037,Ult13256:0.02667)0.782.78:0.00369,((Ult13241:0.0,Ult1
3242:0.0,Ult13244:0.0,Ult13245:0.0,Ult13255:0.0):5.5E-
4,Ult13243:0.00369)0.839.70:0.00369)0.884.98:5.5E-4)0.937.82:5.5E-
4,(UltGem12:0.01126,UltGem13:0.00372)0.866.83:0.0074)0.780.86:0.00744,(Ul
tGem11:0.00368,(Ult13248:0.0,UltGem20:0.0):5.5E-4)1.000.591:5.5E-
4)0.832.61:0.00364,Ult13246:0.00742)0.307.6:5.4E-4,UltGem10:5.4E-
4)0.997.89:0.00172,Ult13247:0.03444)0.996.71:0.00203,(Ult13240:0.0,UltAc1
53:0.0):5.5E-
4)0.923.120:0.00746)0.936.82:0.01118)0.860.61:0.00939,((((Ult13252:5.5E-
4,(Ult13250:5.4E-
4,Ult13251:0.00722)0.878.82:0.00727)0.828.51:0.0037,UltGem16:5.5E-
4)0.688.13:5.3E-
4,Ult13253:0.00371)0.838.74:0.00724,((UltGem15:0.00796,UltGem23:0.00326)0
.842.83:0.00759,(((UltGem24:0.00205,UltGem17:0.01094)0.928.70:0.00197,(Ul
tGem19:5.3E-4,UltGem22:0.00743)0.580.8:0.00371)0.920.101:5.5E-
4,UltGem18:0.01114)0.835.68:0.00777)0.734.43:0.00767)0.897.80:0.01468)0.9
29.79:0.02045)0.658.10:0.01616)0.903.83:0.01924,(UltGem75:0.00812,((Ult13
262:0.0,Ult13263:0.0):0.06631,(UltrS155:0.00375,(UltrS156:0.01118,(Ult132
61:5.5E-4,Ult13260:5.5E-4)0.981.64:5.5E-4)0.942.81:0.01112)0.319.11:5.5E-
4)1.000.592:0.06961)0.509.7:0.00503)0.920.102:0.01741)0.777.74:0.00382)0.
913.94:0.01921)0.864.81:0.02435)0.994.81:0.06406,((Ult13474:0.05175,((((U
lt13458:0.00374,(UltActi9:0.00585,Ult13457:0.00578)0.976.51:0.02296)0.910
.90:0.01157,(Ult13473:0.01152,Ult13459:0.01136)0.841.67:0.00739)0.613.13:
0.01028,(Ult13460:0.02055,(((Ult13462:0.03446,((((Ult13464:5.5E-
4,Ult13465:0.01868)0.924.90:0.00731,((Ult13472:0.02277,(Ult13469:0.01144,
Ult13468:0.00721)0.900.77:5.5E-4)0.605.13:5.4E-4,Ult13467:5.5E-
4)1.000.593:5.4E-
4)0.062.6:0.00738,Ult13466:0.01904)0.781.62:0.00377,Ult13463:5.4E-
4)0.779.70:0.00475)0.043.7:0.00707,(Ult13470:0.01113,Ult13471:0.00101)0.8
71.91:0.0028)0.769.71:0.00358,Ult13461:0.0154)0.947.67:0.01849)0.822.48:5
.4E-
4)0.087.7:0.01096,(UltAct10:0.0111,(((UltGe114:0.00818,Ult13455:0.00306)0
.998.99:0.04059,Ult13454:0.0187)0.460.8:5.4E-
4,(Ult13453:0.01151,Ult13456:0.036)0.796.52:0.01179)0.948.86:5.4E-
4)0.504.7:0.01366)0.861.87:0.01342)0.750.45:0.00527,(((((UltrS168:0.01142

,((UltGe111:0.00754,UltGe112:0.00747)0.879.92:0.00796,(UltGe108:0.02307,(
UltGe113:5.4E-4,(Ult13452:5.5E-4,(Ult13451:5.5E-4,Ult13450:5.5E-
4)0.743.53:5.5E-4)0.941.75:0.0075)0.935.82:5.5E-
4)0.861.88:0.00719)0.819.52:0.00737)0.000.585:5.3E-
4,((Ult13399:0.00378,UltGem88:0.00371)0.933.90:0.01199,((((Ult13418:0.007
52,(UltrS167:0.00369,(Ult13417:5.5E-
4,(UltPro60:0.0,Ult13398:0.0,UltGem89:0.0):5.5E-4)0.841.68:5.5E-
4)0.804.50:0.00373)0.945.71:0.00206,Ult13416:0.0148)0.957.65:0.00185,(Ult
13413:5.5E-4,(Ult13414:0.00371,Ult13397:0.00372)0.710.22:5.5E-
4)0.947.68:5.5E-
4)0.770.80:0.00373,Ult13407:0.00761)0.777.75:0.00327)0.896.77:0.00774)0.7
46.51:0.00376,(((UltGem92:0.01774,((((((UltGem99:0.05967,UltGem98:0.01002
)0.123.6:0.01472,(UltFib66:0.01456,Ult13434:0.01224)0.957.66:0.02882)0.71
1.24:0.00414,(Ult13426:0.02388,UltGe102:0.04138)0.814.42:0.00636)0.897.81
:0.01109,((((UltGe103:0.01918,(UltGem93:0.01954,((((Ult13431:0.00753,Ult1
3430:5.5E-4)0.906.101:0.0075,(UltGem94:5.5E-
4,UltGem95:0.0037)1.000.594:5.4E-4)0.247.3:0.00748,UltGem96:5.4E-
4)0.960.83:0.01135,(UltNitr9:5.5E-
4,(Ult13427:0.00371,Ult13428:0.01122)0.874.105:5.5E-4)0.934.79:5.5E-
4)0.755.56:0.00339)0.911.101:0.016)0.877.103:0.01198,(Ult13405:0.00702,Ul
t13410:0.00701)0.847.96:0.00112)0.757.49:0.00329,Ult13409:0.00774)0.942.8
2:0.01585,(UltGem97:0.01847,(Ult13432:5.5E-
4,Ult13433:0.03698)0.766.64:0.0044)0.302.5:0.00376)0.815.57:0.00442)0.208
.6:5.5E-
4,UltGem91:0.01895)0.879.93:0.01309,Ult13429:0.01689)0.820.42:0.00849)0.8
96.78:0.0125,((Ult13449:0.04929,((Ult13437:0.02763,(Ult13444:0.01172,(Ult
13443:0.01434,(UltActi8:0.00353,Ult13445:0.01164)0.002.4:0.00374)0.480.9:
5.3E-
4)0.948.87:0.01565)0.623.8:0.00398,(Ult13439:0.05385,UltGe109:0.02429)0.8
70.68:0.01404)0.768.59:0.0048)0.899.94:0.01458,(((((((Ult13424:0.00751,((
UltGem86:5.5E-4,((((((Ult13406:0.0151,Ult13425:5.5E-
4)0.923.121:0.0075,UltGe115:0.02307)0.767.61:5.4E-4,UltGem85:5.4E-
4)0.842.84:0.00371,Ult13403:0.01123)0.914.98:5.4E-
4,(Ult13415:0.00747,UltGem87:0.00743)0.791.59:5.5E-4)1.000.595:5.1E-
4,((((UltGem83:0.00698,(Ult13402:5.5E-
4,UltGem79:0.0037)0.912.115:0.00209)0.029.5:8.5E-
4,((Ult13400:0.0,UltGem80:0.0):5.4E-
4,(Ult13408:0.0,Ult13412:0.0):0.00372)0.922.115:0.00247)0.922.116:0.00246
,Ult13404:0.00745)0.600.10:5.4E-4,UltrS166:0.0037)0.912.116:5.5E-
4)0.931.83:5.5E-4)0.797.52:5.4E-
4,(Ult13401:0.0,UltGem81:0.0):0.00372)0.895.102:0.00371)0.824.55:5.4E-
4,UltGem90:5.5E-4)0.992.68:5.3E-
4,Ult13423:0.01109)0.870.69:0.00755,(Ult13435:5.5E-
4,UltGe100:0.01126)0.991.72:0.01937)0.786.73:0.00359,UltGe101:5.4E-
4)0.760.65:0.00328,(Ult13436:5.5E-
4,UltActi6:0.00744)0.988.69:0.0196)0.771.82:0.00565,UltGe104:0.05756)0.75
4.58:0.00618)0.926.97:5.4E-
4)0.764.73:0.00767,((Ult13421:0.00372,(Ult13420:0.00377,(Ult13419:0.00375
,Ult13422:0.00377)0.782.79:0.00373)0.815.58:0.00378)0.767.62:0.00358,(Ult
Gem84:5.4E-4,((UltGem82:0.0,UltPro59:0.0):5.5E-
4,Ult13411:0.01535)0.667.17:0.00371)0.981.65:0.01944)0.856.76:0.00787)0.8
47.97:0.00717)0.768.60:0.00418,((UltGe105:0.0038,(UltGe106:0.0,UltGe107:0
.0):0.00748)0.997.90:0.04,(Ult13446:0.02074,Ult13447:0.03357)0.986.58:0.0
4369)0.880.92:5.4E-

4)0.820.43:0.00759,((((UltActi7:0.0144,(Ult13442:0.04074,Ult13438:0.05563
)0.866.84:0.0204)0.449.21:0.01275,(Ult13440:5.4E-
4,Ult13441:0.02252)0.181.3:0.00455)0.921.100:0.01911,UltGe110:0.01977)0.7
42.39:0.00334,Ult13448:0.04122)0.912.117:0.01562)0.987.87:0.04111)0.830.5
8:0.0091)0.886.112:0.01754,((((Ult13478:0.00958,Ult13477:0.03043)0.986.59
:0.05157,(Ult13475:0.02235,Ult13476:0.00836)0.723.25:0.01099)0.880.93:0.0
3898,((Ult13493:0.07101,(Ult13494:0.04605,Ult13489:0.02554)0.512.5:0.0024
1)0.772.72:0.00499,((((Ult13481:0.0093,(Ult13479:5.4E-
4,Ult13480:0.01116)0.857.86:0.00971)0.880.94:0.01154,((Ult13483:0.01525,U
ltAct13:0.02397)0.935.83:0.02561,(Ult13482:0.04148,((UltAct12:0.14326,Ult
Act19:0.02696)0.839.71:0.02325,UltAct11:0.02492)0.778.70:0.01065)0.410.11
:0.0142)0.607.7:5.3E-
4)0.885.76:0.016,(Ult13488:0.0363,((Ult13676:0.01854,((Ult13486:0.07152,(
Ult13484:5.4E-
4,Ult13485:0.00732)0.699.21:0.02354)0.930.72:0.03079,Ult28950:0.03242)0.7
45.58:0.00481)0.885.77:0.01351,((Ult13487:0.01116,Ult10681:0.08844)0.722.
19:0.01039,UltGe116:0.01323)0.780.87:0.00425)0.746.52:0.00467)0.893.92:0.
01664)0.962.81:0.02623,((Ult13491:0.01588,Ult13490:0.0233)0.832.62:0.0073
8,(Ult13492:0.01116,UltPro64:0.03574)0.726.21:0.0042)0.897.82:0.00879)0.7
10.23:0.0019)0.719.17:0.0069)0.933.91:0.02605,Ult13495:0.0331)0.896.79:0.
02167)0.873.88:0.03003,(((Ult13564:0.01575,(UltrS174:0.07985,(Ult13652:0.
02298,(((Ult13650:0.00359,Ult13651:0.00752)0.877.104:0.00778,(Ult13648:0.
00375,Ult13649:0.00742)0.758.69:0.00337)0.939.69:0.01514,(UltFor18:0.0147
6,UltFor17:0.00364)0.923.122:0.01134)0.741.48:0.0033)0.911.102:0.03111)0.
778.71:0.06023)0.863.99:0.02597,((Ult13563:0.03499,((Ult13561:0.02976,(Ul
t13559:0.00866,((((Ult13555:0.04781,(UltGe126:0.00839,(UltGe128:0.03631,U
ltGe127:0.02054)0.906.102:0.01806)0.885.78:0.01465)0.544.7:0.01303,((Ultr
S169:0.06329,UltrS170:0.02175)0.920.103:5.5E-
4,(((UltGe124:0.0,UltGe125:0.0):5.5E-
4,(Ult13558:0.00987,Ult13557:0.01531)0.889.86:0.0101)0.975.58:0.04031,(((
((Ult13537:0.00196,(Ult13539:0.00368,((Ult13543:0.0,Ult13544:0.0):5.5E-
4,(Ult13541:0.00371,Ult13538:0.00745)0.829.63:5.5E-4)0.943.77:5.5E-
4)0.981.66:0.01877)0.941.76:0.00184,Ult13540:0.00754)0.849.76:0.00802,(Ul
t13547:0.02801,(Ult13542:0.01527,((Ult13553:0.0,Ult13545:0.0,Ult13546:0.0
):5.5E-
4,Ult13552:0.01127)0.791.60:0.00389)0.762.77:0.00344)0.895.103:0.01349)0.
974.46:0.03091,(Ult13554:0.00352,(UltGe123:0.01521,(Ult13549:0.00582,Ult1
3556:0.03973)0.784.69:0.0056)0.765.54:0.00395)0.857.87:0.00982)0.381.15:0
.00483,Ult13551:0.01187)0.971.58:0.03419)0.660.11:0.0135)0.895.104:0.0185
4)0.680.16:0.00942,((Ult13570:5.4E-
4,((UltGe131:0.0078,(UltGe130:0.01127,(UltGe132:0.00721,(Ult13568:0.01494
,(UltrS171:5.3E-4,Ult13567:0.00732)0.918.90:5.4E-
4)0.990.73:0.02713)0.915.85:0.01157)0.745.59:0.00309)0.996.72:0.03988,(Ul
t13572:0.03469,((Ult13582:0.00365,(Ult13573:5.5E-
4,Ult13580:0.00364)0.855.81:5.5E-
4)0.983.49:0.02304,(Ult13585:0.03221,((((Ult13575:0.01956,UltGe135:0.0149
9)0.944.65:0.0162,(Ult13579:0.01642,(Ult13576:0.03395,(Ult13577:5.5E-
4,Ult13578:0.01905)0.858.81:5.4E-
4)0.994.82:0.04326)0.671.10:0.00352)0.855.82:0.01656,(UltGe134:0.0481,Ult
13574:5.4E-
4)0.939.70:0.02707)0.968.69:0.03214,(((Ult13571:0.00957,(UltrS172:5.5E-
4,UltrS173:0.00365)0.932.79:0.01799)0.976.52:0.0229,UltGe137:0.01155)0.87
8.83:0.01336,UltGe133:0.00629)0.925.103:0.02096)0.500.10:0.00172)0.962.82
:0.02053)0.950.83:5.4E-

4)0.953.66:0.01873)0.738.32:0.00384)0.833.73:0.00366,Ult13569:5.4E-
4)0.993.60:0.03939)0.922.117:0.02817,Ult13560:0.05905)0.683.19:0.01613)0.
880.95:0.01677)0.780.88:0.00691,Ult13562:0.0119)0.738.33:0.00578)0.706.21
:0.01144,((UltGe129:0.03729,(Ult13565:0.01452,Ult13566:0.00463)0.909.77:0
.02128)0.425.6:0.01897,((Ult13584:5.5E-4,(UltGe136:5.3E-
4,(Ult13548:0.00364,Ult13583:5.5E-
4)0.995.84:0.03001)0.893.93:0.00819)0.782.80:0.00559,Ult13581:0.00549)1.0
00.596:0.08525)0.713.27:0.00667)0.760.66:0.0087)0.970.68:0.0532,(((Ult135
34:0.02297,Ult13535:0.00812)0.845.84:0.01572,((Ult13533:0.05566,(((Ult135
10:0.04726,(UltGe117:0.04077,(UltGe119:0.0675,((Ult13508:0.00367,Ult13509
:0.00368)0.948.88:0.02497,(UltGe118:5.5E-4,(Ult13506:5.4E-
4,Ult13507:0.01109)0.975.59:0.01889)0.128.3:0.00724)0.287.7:0.00669)0.850
.88:0.01394)0.862.85:0.01484)0.876.75:0.01851,(Ult13511:0.07869,(((Ult134
97:0.02246,Ult13500:0.01216)0.816.59:0.00913,(Ult13496:0.07793,(Ult13499:
5.4E-
4,(Ult13498:0.01137,(UltFor14:0.01681,Ult13504:0.05387)0.704.15:0.00186)0
.591.11:0.00376)0.794.60:0.0017)0.775.73:0.0164)0.980.49:0.03507,((Ult135
03:0.0,Ult13502:0.0):5.4E-
4,Ult13501:0.00365)0.822.49:0.00966)0.817.42:0.01905)0.799.70:0.0129)0.92
7.90:0.02778,(((((Ult13522:0.04421,Ult13521:0.03206)0.992.69:0.06185,(Ult
Ge120:0.00746,Ult13516:0.01896)0.847.98:0.01003)0.704.16:0.00274,(((Ult13
515:0.01161,Ult13529:0.07608)0.793.65:0.01296,(Ult13518:0.02337,((Ult1352
6:0.0,Ult13527:0.0):5.5E-4,(Ult13505:5.4E-
4,(Ult13525:0.00366,Ult13528:0.00368)0.925.104:0.00734)0.958.86:0.01082)0
.943.78:0.02402)0.915.86:0.02402)0.748.53:0.00827,(Ult13512:0.01123,(Ult1
3513:0.01141,Ult13514:0.0074)0.906.103:0.01661)0.826.53:0.01594)0.683.20:
0.00949)0.762.78:0.00906,(UltGe121:0.02388,((Ult13520:0.01326,UltGe122:0.
00972)0.977.56:0.03936,(Ult13517:0.06123,Ult13519:0.04517)0.657.10:0.0135
7)0.825.56:0.01947)0.784.70:0.01153)0.940.86:0.02846,(Ult13524:0.05327,((
Ult13523:0.02294,Ult13532:0.02503)0.702.14:0.0044,(Ult13531:0.02791,Ult13
530:0.05285)0.845.85:0.01117)0.926.98:0.02625)0.933.92:0.03115)0.863.100:
0.01685)0.524.8:5.4E-
4)0.734.44:0.01288,Ult13536:0.04078)0.919.113:0.03184)0.870.70:0.03863,((
((Ult13647:0.0438,Ult13646:0.03805)0.991.73:0.0702,((Ult13661:0.0388,(((U
lt13656:0.0093,(Ult13657:0.01087,UltGa301:5.4E-
4)0.876.76:0.01408)0.882.92:0.01383,(Ult13655:0.03509,(Ult13653:0.00364,U
lt13654:0.00367)0.980.50:5.4E-
4)0.953.67:0.02411)0.168.7:0.00536,((((((UltFor20:0.00351,((Ult13674:0.00
757,UltPro63:0.00347)0.946.78:0.01837,Ult13673:0.02249)0.879.94:0.01325)0
.780.89:0.00375,(UltFor19:0.0,UltFor21:0.0):5.4E-
4)0.804.51:0.00957,(UltDe227:0.03146,(UltAct17:0.01595,(UltSpon4:0.00723,
UltSpon5:0.0113)0.926.99:0.01792)0.406.8:0.00945)0.764.74:0.0075)0.200.7:
0.003,Ult13658:0.0431)0.502.5:0.00646,Ult13663:0.02293)0.201.7:0.00367,((
Ult13668:0.01922,((Ult13672:0.01539,UltGe155:0.01118)0.959.79:0.02729,((U
ltAct18:0.01925,(Ult13669:0.00363,Ult13675:5.5E-
4)0.919.114:0.01114)0.811.54:0.00626,(Ult13671:0.0074,(((Ult13666:0.00725
,Ult13667:0.00748)0.744.46:0.00433,(Ult13664:5.0E-
4,Ult13665:0.00735)0.956.75:0.01447)0.346.12:0.0073,Ult13670:0.00675)0.75
9.78:0.0043)0.788.65:0.00511)0.649.10:5.3E-
4)0.933.93:0.02546)0.970.69:0.03026,Ult13662:0.01936)0.736.39:0.00373)0.8
68.66:0.01257)0.940.87:0.02594)0.737.52:0.00525,(Ult13659:0.04664,Ult1366
0:0.05934)0.911.103:0.02062)0.943.79:0.03457)0.246.10:0.00842,(Ult13642:0
.03419,(Ult13644:5.4E-4,(Ult13643:0.01262,(Ult13641:5.5E-
4,((Ult13640:0.01932,Ult13645:0.01995)0.736.40:0.00332,((UltGe154:5.5E-

4,UdnBac59:0.01908)0.872.91:5.4E-
4,UltGe153:0.01106)0.898.103:0.01132)0.993.61:0.03227)0.949.83:0.02142)0.
871.92:0.01191)0.835.69:0.00872)0.989.63:0.04361)0.888.99:0.02633,((Ult13
605:0.01004,Ult13604:0.02903)0.931.84:0.02311,(((UltOr236:0.06862,Ult1360
3:0.04276)0.777.76:0.00981,(Ult13602:0.00907,((Ult13597:0.01258,(Ult13598
:0.0144,Ult13599:0.00837)0.457.12:0.00734)0.948.89:0.02347,(Ult13601:0.00
716,(UdnBac57:0.01647,Ult13600:0.04375)0.872.92:0.01619)0.768.61:0.00798)
0.422.11:0.01269)0.897.83:0.01821)0.876.77:0.01424,((((((UltGe138:0.02023
,UltGe139:0.01448)0.908.100:0.01128,((Ult13592:0.01382,(Ult13637:0.03239,
(UltGe152:0.04102,(UltPro62:0.00355,((UltAct16:0.0,Ult13636:0.0):5.4E-
4,UdnBac58:0.01847)0.472.10:0.00725)0.734.45:0.00556)0.378.11:0.01145)0.8
84.99:0.01683)0.875.96:0.01248,(UltGe140:0.01782,Ult13593:0.00883)0.588.1
0:0.01108)0.905.87:0.01309)0.856.77:5.5E-
4,((((Ult13590:0.00363,UltMa103:0.04346)0.932.80:0.00206,(Ult13587:5.3E-
4,(Ult13586:0.01146,Ult13588:0.01132)0.444.20:0.00712)0.930.73:0.01062)0.
807.48:0.0018,UltAct14:0.01503)0.929.80:0.01218,Ult13589:0.03426)0.748.54
:0.00364)0.763.80:0.0043,((Ult13591:5.4E-
4,((Ult13622:0.01864,(Ult13596:0.01075,((Ult13594:5.5E-
4,UltGe141:0.00364)0.887.89:0.01037,(UltPro61:5.5E-
4,Ult13623:0.00363)0.939.71:0.0158)0.264.4:0.00401)0.810.61:0.0114)0.764.
75:0.00359,((UltFor15:0.01195,(UltGe145:0.0231,Ult13595:0.01097)0.911.104
:0.0145)0.796.53:0.0074,Ult13621:0.03116)0.719.18:0.00369)0.895.105:0.007
48)0.882.93:5.4E-
4,((UltGe142:0.0,UltGe143:0.0):0.0052,UltGe144:0.03274)0.993.62:0.04552)0
.868.67:0.01046)0.913.95:0.02093,(((Ult13615:5.4E-
4,((Ult13614:0.01913,Ult13613:5.4E-
4)0.885.79:0.00744,UltGe149:0.00375)0.937.83:0.01129)0.000.586:5.4E-
4,(Ult13616:0.02726,UltGe150:0.00517)0.895.106:0.01015)0.996.73:0.05257,(
(((Ult13624:5.5E-
4,Ult13638:0.00368)0.965.65:0.01796,((Ult13611:0.00342,Ult13639:0.03194)0
.999.122:0.05398,(Ult13612:0.03751,UltOr237:0.03276)0.802.53:0.0191)0.994
.83:5.4E-
4)0.673.8:0.0158,((UltGe147:0.03032,UltGe148:0.0043)0.987.88:0.02761,(((U
lt13607:0.00368,((UltGe146:0.0131,UltAct15:0.03912)0.774.80:0.00657,Ult13
608:0.01528)0.890.95:0.00753)0.088.8:5.4E-
4,Ult13606:0.02281)0.825.57:0.00652,(Ult13609:0.00363,Ult13610:0.02315)0.
771.83:0.00463)0.861.89:0.0076)0.843.62:0.01055)0.252.6:0.01118,(Ult13619
:0.01838,(Ult13618:5.5E-
4,(UltGe151:0.00738,Ult13617:0.03113)0.853.67:0.01118)0.922.118:0.01644)0
.932.81:0.02235)0.369.9:0.01713)0.782.81:0.00905)0.718.21:0.008,((Ult1362
9:0.05162,((Ult13620:0.02199,(Ult13633:0.02097,((Ult13631:0.01745,(Ult136
30:0.0079,Ult13632:0.01572)0.861.90:0.00895)0.893.94:0.01854,UltFor16:0.0
2237)0.935.84:0.02576)0.898.104:0.01674)0.808.59:0.00773,(Ult13627:0.0204
2,(Ult13628:0.01829,Ult13634:0.04181)0.806.45:0.01476)0.904.88:0.0183)0.3
60.11:0.00379)0.826.54:0.00921,((Ult13625:0.0376,Ult13626:0.01828)0.390.1
1:0.0097,(UltOr235:0.06509,(Ult13635:0.00505,UltDe157:0.01423)0.943.80:0.
02879)0.932.82:0.02978)0.777.77:0.01047)0.881.98:0.01718)0.830.59:0.01117
)0.936.83:0.02808)0.857.88:0.035)0.560.7:0.02143)0.916.99:0.03831)0.925.1
05:0.04528)0.956.76:0.04736,(Ult15498:0.24413,(UltDel30:0.07734,(UltB4293
:0.06195,(UltB4292:0.00379,(UltDel29:0.0121,(UltB4291:0.02349,(UltB4290:0
.00748,UltMar38:0.00746)0.841.69:0.00783)0.864.82:0.01115)0.946.79:0.0405
6)0.983.50:0.06947)0.864.83:0.0342)0.974.47:0.08019)0.396.18:0.01214)0.91
3.96:0.03807,(((Ult16373:0.01488,(AdbBac13:0.01917,(Ult16367:0.01108,((((
UltAc827:0.02696,((((Ult16397:0.06654,((Ult16394:0.00369,(Ult16398:0.0,Ul

t16404:0.0,Ult16406:0.0,Ult16411:0.0):5.5E-
4)0.854.79:0.0037,((Ult16390:0.0,Ult16391:0.0,Ult16392:0.0,Ult16393:0.0,U
ltHol35:0.0,Ult16396:0.0,Ult16399:0.0,Ult16401:0.0,Ult16402:0.0,Ult16403:
0.0,Ult16405:0.0,Ult16407:0.0,Ult16408:0.0,Ult16410:0.0,Ult16409:0.0):5.5
E-4,(Ult16395:0.02265,Ult16412:0.00369)0.717.24:5.5E-4)0.952.67:5.4E-
4)0.751.42:0.00329)0.977.57:0.02759,(Ult16368:0.00745,Ult16381:0.03198)0.
854.80:0.01149)0.986.60:5.5E-
4,((((UltAc836:0.02309,Ult16383:0.00369)0.883.99:0.01091,(Ult16382:0.0149
4,(Ult16374:0.01912,(Ult16380:0.00369,Ult16389:5.5E-4)0.992.70:5.3E-
4)0.910.91:0.0158)0.284.4:0.01147)0.867.88:0.01169,((Ult16384:0.00362,(Ho
lFoeti:5.5E-
4,UltDe163:0.00371)0.991.74:0.02352)0.877.105:0.00763,((UltAc828:0.01126,
UltHol34:0.00373)0.889.87:0.00764,(UltAc840:0.01159,(UltAc837:0.00375,Ult
Ac838:0.00747)0.930.74:0.0115)0.858.82:0.00759)0.784.71:0.00363)0.996.74:
5.5E-4)0.968.70:0.0071,((Ult16387:0.01925,Ult16388:0.01519)0.984.58:5.4E-
4,(Ult16385:0.0,Ult16386:0.0,UltAc839:0.0):5.4E-4)0.732.23:5.5E-
4)0.937.84:0.00739)0.927.91:0.00739,(((Ult16365:0.00369,Ult16415:0.00369)
0.923.123:5.5E-
4,(Ult16371:0.0,Ult16372:0.0,Ult16378:0.0,Ult16413:0.0):5.5E-
4)0.000.587:5.5E-
4,(((Ult16369:0.0,UltEps38:0.0):0.00751,Ult16416:0.00369)0.837.55:5.5E-
4,(Ult16414:0.00369,UltGeoth:0.00369)0.622.6:5.5E-4)0.000.588:5.5E-
4)0.921.101:5.5E-4)0.674.16:5.4E-4)0.851.83:0.00363,(Ult16366:5.5E-
4,Ult16370:0.00369)0.901.88:5.4E-4)0.932.83:0.00735,UltAc826:5.4E-
4)0.269.8:5.5E-4,(((UltAc832:0.03744,(UltAc830:5.5E-
4,(UltAc829:0.002,UltAc831:0.01481)0.942.83:0.00185)0.916.100:0.02621)0.9
92.71:0.06854,(UltAc834:0.02297,UltAc835:5.5E-
4)0.504.8:0.00983)0.265.4:0.00323,UltAc833:0.00649)0.995.85:0.03114)0.873
.89:0.00756)0.914.99:0.01147)0.628.3:5.3E-
4)0.972.68:0.0608,((Ult16379:0.08587,Ult16539:0.03854)0.980.51:0.07016,Ul
tB4786:0.13107)0.846.94:0.02681)0.815.59:0.01898,((((Ult15680:0.1177,(Ult
B2844:0.10099,(((((((UltB2196:0.0428,UltB2439:0.05132)0.765.55:0.01348,(U
ltB1574:0.02603,UltB1753:0.06218)0.787.61:0.01737)0.923.124:0.02917,(((Ul
tB1565:5.5E-
4,UltB1566:0.00364)1.000.597:0.07552,((((UltB1742:0.023,((ThrAroma:0.0074
1,((AzcSpeci:5.4E-4,(UltrB176:0.00744,Otu00449:5.4E-
4)0.996.75:0.01901)0.911.105:0.00745,UltB2488:0.00748)0.553.10:5.4E-
4)0.966.86:0.01504,ThrTerpe:0.00372)0.782.82:0.00379)0.803.49:0.0037,((Ul
trBe53:0.00369,(((UltB1824:0.00369,(((((UltrSoi8:0.0037,(UltB1831:0.0037,
(((IroBact3:0.00367,(((UltB1862:0.0,UltB1864:0.0,UltB1894:0.0):5.5E-
4,(UltB1863:0.00369,(((((UltrBe77:0.00369,((UltB1868:0.0074,UltB1869:0.01
567)0.883.100:0.00773,(UltAquab:0.00751,UltB1893:5.5E-4)0.728.33:5.5E-
4)0.567.6:5.5E-4)0.241.8:5.5E-4,UltB1872:0.00741)0.000.589:5.5E-
4,(UltB1876:0.05386,UltB1867:0.02741)0.754.59:0.00462)0.451.14:5.5E-
4,UltB1849:0.00369)0.000.590:5.5E-4,UltB1875:0.00369)0.064.7:5.5E-
4)0.000.591:5.5E-4)0.198.11:5.5E-
4,(UltB1865:0.00738,(UltB1871:0.0074,UltB1866:0.00742)0.350.8:5.5E-
4)0.834.54:0.00368)0.117.6:5.5E-4)0.281.5:5.5E-
4,(UltB1870:0.01867,UltB1861:5.5E-
4)0.828.52:0.00365)0.840.75:0.00466,(((UltBur12:0.0,UltBur13:0.0,UltBur15
:0.0,UltB1891:0.0,UltB1892:0.0):5.5E-4,((UltBur16:0.00369,(UltB1885:5.5E-
4,(UltB1883:0.00743,UltB1884:0.01243)0.125.8:5.4E-
4)0.940.88:0.00824)0.218.5:5.4E-4,UltBur14:5.5E-4)0.643.6:5.5E-
4)0.984.59:5.4E-4,((AqbParvu:0.0,UltB1877:0.0):5.5E-

4,UltrBe74:0.00741)0.836.59:0.00363)0.825.58:0.00823)0.986.61:5.3E-
4)0.243.6:5.5E-4)0.003.4:5.5E-4,UltB1873:0.00742)0.482.12:5.5E-
4,BrhBacte:0.00369)0.450.11:5.5E-4,UltBur11:5.5E-4)0.000.592:5.5E-
4,UltB1878:0.00369)0.380.9:5.5E-4)0.826.55:5.4E-
4,((((UltB1457:0.0037,(UltB1919:5.5E-
4,(UltB1838:0.01497,(UltB1854:0.0037,UltB1927:0.00745)0.861.91:5.5E-
4)0.852.80:0.00368)0.779.71:0.0037)0.820.44:0.0037,((UltB1833:0.0,UltB185
7:0.0,MthFulvu:0.0):5.5E-
4,((UltrB103:0.00744,MthSpeci:0.0037)0.424.13:5.5E-
4,UltB1834:0.00376)0.904.89:5.5E-4)0.792.78:5.4E-4)0.099.3:5.5E-
4,(((UltB1835:0.0,UltB1859:0.0):5.5E-4,(UltrBe71:0.00741,((IroBac10:5.5E-
4,(UltrBe81:0.00369,UltrBe82:5.5E-
4)0.923.125:0.00743)0.819.53:0.00748,UltB1858:0.00742)0.919.115:5.4E-
4)0.181.4:5.5E-4)0.756.68:5.4E-
4,((AqbCommu:0.0,UltB1879:0.0):0.0037,(UltB1890:0.0,UltrBe78:0.0):5.4E-
4)0.860.62:0.0037)0.788.66:0.00368)0.405.16:0.00368,(UltrBe68:0.0,UltRhiz
o:0.0):5.4E-4)0.946.80:0.01122)0.911.106:0.00892,(UltB1815:5.4E-
4,(RhfSpeci:5.4E-
4,((((((UltCurvi:0.00369,(UltB1821:0.00741,UltB1822:0.03307)0.341.12:5.5E
-4)0.249.13:5.5E-4,(CurLance:0.0,UltB1823:0.0,UltCurv2:0.0):5.5E-
4)0.185.8:5.5E-
4,UltB1819:0.00741)0.833.74:0.00445,(((UltAcid6:0.00369,UltB1480:0.01499)
0.936.84:5.5E-
4,(AcdFacil:0.0,AcdDela4:0.0,UltB1483:0.0,UltOrg11:0.0,UltB1567:0.0):5.5E
-4)0.778.72:5.5E-4,UltB1481:0.0037)0.857.89:0.0037)0.625.8:5.5E-
4,(((((CurDelic:0.00368,(UltB1575:0.02278,(UltB1713:5.4E-
4,((((IroBacte:5.4E-
4,UltCom18:0.01112)0.817.43:0.0036,((((((IroBact5:5.5E-
4,((UltB1712:0.00369,((UltB1548:0.0,UltB1586:0.0):5.5E-
4,(UltrBe72:0.01135,((MthSubsa:0.0151,(UltVerm4:0.01125,(UltVerm2:0.01469
,(UltVerm3:0.00691,(UltVerm5:5.4E-4,(VerEise2:0.0148,(VerEisen:5.5E-
4,VerEise3:5.5E-4)0.623.9:5.5E-
4)0.969.57:0.01168)0.779.72:0.00446)0.540.9:0.0079)0.755.57:0.00458)0.354
.8:0.00384)0.854.81:5.3E-4,UltVermi:5.5E-
4)0.931.85:0.00775)0.660.12:5.5E-4)0.339.12:5.4E-
4)0.843.63:0.00383,(((UltB1739:0.00371,((UltB1738:0.0,UltrBe47:0.0,UltrBe
48:0.0):5.5E-
4,UltB1536:0.01885)0.811.55:0.00369)0.809.57:0.0037,UltB1486:5.5E-
4)0.837.56:0.0037,(IroBact2:0.00369,((AcdDefl3:0.0,AcdDefl2:0.0,AcdDeflu:
0.0,AcdSpec2:0.0,UltB1474:0.0,IroBact7:0.0,IroBact6:0.0,IroBact8:0.0,AcdS
pec3:0.0,AcdSpec4:0.0,UltB1488:0.0,UltAcid4:0.0,UltB1563:0.0,UltB1576:0.0
,UltB1594:0.0,UltAcid7:0.0):5.5E-
4,(((UltB1484:0.02655,((UdnBact6:0.10473,(((AcdDelaf:0.0,AcdDela2:0.0,Acd
Dela3:0.0):5.5E-4,(UltB1561:0.00369,(UltB1740:0.01134,(((((UltB1582:5.5E-
4,((UltB1551:5.5E-4,UltrBe30:5.5E-4)0.715.18:5.5E-
4,(UltB1552:0.0,BacteRJ1:0.0,CmdBact2:0.0,SmpPsyc2:0.0,CmdBact3:0.0,Ult31
918:0.0):5.5E-4)0.000.593:5.5E-4)0.838.75:5.5E-
4,((UltB2365:0.00369,((((UltB1721:0.0,UltrBe43:0.0,UltB1724:0.0,BeaProt3:
0.0,LepDisc2:0.0,LepDisc3:0.0):5.4E-
4,UltB2249:0.00361)0.132.12:0.00369,(AqsSpeci:0.0,UltB1579:0.0,UltB1580:0
.0):0.00362)1.000.598:5.5E-4,((SmpPsych:0.0,UltB1602:0.0):5.5E-
4,UltrBe33:5.5E-4)0.821.50:5.5E-4)0.629.14:5.4E-
4)0.873.90:0.00369,UltB1603:0.0037)0.863.101:5.5E-4)1.000.599:5.4E-
4,((UltB1578:5.5E-4,UltBurk5:5.5E-4)0.765.56:5.4E-

4,(UltB1528:0.0037,UltB1604:5.5E-
4)0.864.84:0.00371)0.798.61:0.00362)0.132.13:0.00369,UltB1813:5.5E-
4)0.811.56:0.00362,(((UltB1675:0.23371,UltB1596:0.01152)0.070.6:5.4E-
4,UltB1581:0.01515)0.393.8:0.00734,(IdeAzoti:5.5E-
4,UltB1922:0.00369)0.772.73:0.00458)0.860.63:0.00811)0.764.76:0.00414)0.7
84.72:0.00413)0.310.8:5.5E-4)0.378.12:5.4E-
4,(UltAcido:0.0037,UltB1421:0.00196)1.000.600:0.00173)0.915.87:5.5E-
4)0.922.119:0.00194,UltB1485:0.01882)0.009.5:5.5E-
4)0.957.67:0.00191,((UltPro14:5.5E-
4,((UltB1730:0.00369,UltB1728:0.00369)0.000.594:5.5E-
4,((UltB1550:0.0,UltrBe45:0.0,UltrBe46:0.0,UltB1729:0.0,UltB1842:0.0):5.5
E-4,(((UltB1774:0.01109,(UltB1747:5.5E-4,UltB1492:0.00367)0.373.13:5.5E-
4)0.790.68:0.00398,UltB1549:0.0193)0.776.78:0.00394,UltrBe44:0.00369)0.20
5.7:5.5E-4)0.000.595:5.5E-4)0.220.7:5.5E-
4)0.921.102:0.0079,UltB1525:5.4E-4)0.922.120:0.00787)0.623.10:5.4E-
4,IroBact4:0.00369)0.213.6:5.5E-4)0.000.596:5.5E-4)0.437.12:5.5E-
4)0.000.597:5.5E-4)0.343.5:5.5E-4)0.000.598:5.5E-
4,UltB1725:0.00744)0.417.14:5.4E-4,UltAcid2:5.5E-4)0.076.4:5.5E-
4,UltB1727:0.0074)0.033.1:5.4E-4,UltB1487:5.5E-4)0.159.4:5.3E-
4,UltB1660:5.5E-4)0.431.13:5.4E-4)0.803.50:5.4E-
4,((((UltB1407:0.02941,(DelSpeci:0.02704,UltB1600:0.01828)0.392.7:0.00548
)0.448.9:0.01335,AntBact7:5.4E-
4)0.850.89:0.00357,((((((UltAcid3:0.0,UltB1526:0.0,UltBurk9:0.0,UltB1736:
0.0,UltB1737:0.0):5.5E-4,((((((UltB1470:0.0,UltB1776:0.0):5.5E-
4,((UltB1781:0.00369,(UltB1584:0.02651,(((UltCom20:5.5E-
4,(((UltB1772:0.00369,((((((UltrBe59:5.5E-
4,((UltB1755:0.00369,((UltrBe49:0.04484,UltB1748:0.00368)0.000.599:5.5E-
4,UltB1771:0.00742)0.285.7:5.5E-4)0.449.22:5.5E-
4,UltrBe51:0.00369)0.532.12:5.5E-4)0.000.600:5.5E-
4,(UltB1719:0.0,UltB1750:0.0,UltBanis:0.0,UltrBe57:0.0,RhfFerri:0.0,UltB1
775:0.0,UltCom21:0.0,UltB1777:0.0,UltrBe60:0.0,UltB1779:0.0,UltB1745:0.0,
AntBact9:0.0,UltB1749:0.0,UltrBe62:0.0):5.5E-4)0.485.13:5.5E-
4,UltB1773:0.0037)0.217.8:5.5E-4,UltB1744:0.00746)0.000.601:5.5E-
4,UltrBe58:0.0074)0.000.602:5.5E-4,UltrBe27:0.00369)0.000.603:5.5E-
4)0.365.8:5.5E-4,UltB1530:0.00369)0.458.19:5.5E-4,AntBac10:5.5E-
4)0.375.11:5.1E-4)0.266.7:5.5E-
4,UltrBe50:0.0074)0.833.75:0.0038,BeaProt4:5.5E-4)0.402.8:5.5E-
4)0.410.12:5.5E-4)0.000.604:5.5E-4,UltB1537:5.5E-4)0.415.10:5.5E-
4)0.344.10:5.5E-
4,UltAnta5:0.00734)0.842.85:0.00406,UltB1689:0.0037)0.120.7:5.5E-
4,UltB1471:0.00369)0.424.14:5.5E-4,UltB1473:0.00369)0.427.9:5.5E-
4)0.000.605:5.5E-4,LepMobil:0.00369)0.109.9:5.5E-
4,((UltB1449:0.01515,(UltB1764:0.0,UltB1784:0.0):0.00369)0.926.100:5.4E-
4,UltrBe54:0.00369)0.844.84:0.00369)0.263.6:5.5E-
4,AcdFaci2:0.01115)0.078.8:5.5E-4,UltrBe21:0.01116)0.305.7:5.5E-
4)0.491.8:5.5E-4,(((GlcBacte:0.0,UltCom23:0.0,PlrSpec2:0.0):5.5E-
4,AcdSpe12:0.00365)0.965.66:0.01115,(UltrBe85:5.5E-
4,SphSpeci:0.01134)0.324.6:5.5E-
4)0.937.85:0.00743)0.909.78:0.00807)0.809.58:0.00369,((UltB1741:0.01511,(
(UltB1692:0.01528,XxxChlor:0.01152)0.777.78:0.00569,((UltrBe17:0.01152,(U
ltCom16:0.00754,(((UltB1610:0.00727,(UltB1623:0.00369,(HdgSpec5:5.5E-
4,(((((((BeaPro20:0.00747,(UltPro15:0.00369,((UltSynth:5.5E-
4,UltB1939:5.5E-4)0.394.11:5.5E-
4,((TpdIgnav:0.00371,TpdAquat:0.00371)0.789.61:0.00373,(TepXavie:0.0,UltH

ydr3:0.0):0.00751)0.800.57:0.00372)0.821.51:0.00367)0.372.9:5.5E-
4)0.949.84:0.00748,(UltB1606:5.5E-
4,((UltB1607:0.0,UltB1622:0.0,UltrBe35:0.0,UltB1638:0.0):5.5E-
4,(UltB1400:0.00743,UltB1608:5.5E-4)0.609.14:5.5E-4)0.804.52:5.5E-
4)0.862.86:5.5E-
4)0.878.84:0.00357,(UltB1636:0.0036,(((UltB1531:0.0,HdgSpeci:0.0,HdgSpec2
:0.0,HdgFlava:0.0,HdgBisan:0.0,HdgSpec4:0.0,HdgSpec6:0.0,HdgPalle:0.0):5.
5E-4,UltB1621:0.00749)0.722.20:5.5E-
4,(UltrBe34:0.00372,((UltB1448:0.00369,UltHydro:0.0037)0.688.14:5.5E-
4,UltB1631:5.5E-4)1.000.601:5.4E-4)0.589.12:0.0037)0.583.7:5.5E-
4)0.150.6:0.0037)1.000.602:5.4E-4,(UltrBe25:5.5E-
4,UltB1639:0.00369)0.502.6:5.4E-
4)0.922.121:0.00337,((UltB1627:0.011,UltB1632:5.4E-
4)0.043.8:0.00398,UltB1643:0.00738)0.422.12:0.00729)1.000.603:5.4E-
4,(((((UltB1620:0.0,HdgAtypi:0.0,ArnBacte:0.0,GroBiofi:0.0,UltB1640:0.0):
5.5E-
4,((((UltB1529:0.00369,((UltB1451:0.0,UltB1555:0.0,UltB1618:0.0,UltrBe36:
0.0):5.5E-4,(UltB1619:0.0115,UdnBact7:0.00369)0.574.9:5.5E-
4)0.359.6:5.5E-4)0.830.60:0.0038,(UltMalik:0.01531,HdgSpec7:5.5E-
4)0.863.102:0.00376)0.534.10:5.5E-4,UltB1642:0.00743)0.000.606:5.5E-
4,UltB1633:0.02713)0.492.14:5.5E-4)0.000.607:5.5E-4,(HdgSpec9:5.5E-
4,UltB1641:0.00199)1.000.604:0.00169)0.000.608:5.5E-
4,HdgSpe10:0.00371)0.409.7:5.4E-
4,((UltB1630:0.01892,(MacBipun:0.00343,UltB1625:0.0201)0.876.78:0.00777)0
.948.90:5.3E-4,(UltB1624:0.00369,(MalGrano:5.5E-4,PsdSp153:5.5E-
4)0.000.609:5.5E-4)0.274.5:5.5E-4)0.930.75:0.00368)0.486.11:5.4E-
4)0.862.87:0.00379,(((AntBact8:5.5E-4,UltB1782:5.5E-
4)0.940.89:0.00749,((HdgTaeni:0.0,UltB1629:0.0):0.00369,HdgSpec8:5.5E-
4)0.619.5:5.4E-
4)0.797.53:0.00369,UltHydr2:0.01537)0.777.79:0.00369)0.228.9:5.5E-
4)0.149.10:5.4E-4)0.917.86:5.4E-4)0.177.11:0.00748,((((UltB1609:5.5E-
4,(HdgInter:5.4E-
4,UltB1637:0.0037)0.931.86:0.00745)0.844.85:0.00369,HdgSpec3:5.4E-
4)0.834.55:0.0037,HdgFlav2:5.5E-
4)0.931.87:0.0112,(UltB1626:0.00374,(BacBH982:0.0,UltB1628:0.0):0.0075)0.
946.81:5.5E-
4)0.839.72:0.00779)0.895.107:0.01181,UltB1656:0.01895)0.538.8:5.3E-
4)0.765.57:0.00383)0.874.106:0.00977,UltB1799:0.00944)0.903.84:0.01198)0.
787.62:0.00562)0.900.78:5.5E-
4,(LepSpec2:0.00373,(UltrBe87:0.0,UltB1920:0.0):0.00749)0.896.80:0.00741)
0.514.9:0.0077)0.977.58:5.4E-4)0.845.86:0.00425)0.572.7:5.5E-
4)0.189.6:5.5E-
4,(UltB1462:0.0,UltB1479:0.0,UltB1562:0.0,UltrBe41:0.0,BeaProt9:0.0,UltB1
818:0.0):5.5E-4)0.249.14:5.5E-
4,UltB1805:0.00371)0.891.80:0.00397,((((((((VarDokdo:0.0072,((((((UltB1
685:0.01945,AcdKonja:0.01499)0.809.59:5.5E-
4,(((UltXylop:0.0,AcdSpec7:0.0,UltB1534:0.0,UltB1705:0.0,UltB1714:0.0):5.
5E-4,(((SmpMetam:0.0,SmpMeta2:0.0):0.00369,((UltB1489:5.5E-
4,(UltB1687:0.00369,((AcdCattl:0.0,AcdOryza:0.0,AcdAvena:0.0):5.5E-
4,(((UltB1752:0.0037,((UltB1757:0.0,UltB1763:0.0,UltB1765:0.0):5.5E-
4,(UltB1758:0.00369,((UltB1766:0.00369,((UltrBe56:0.0,UltB1746:0.0):5.5E-
4,UltB1768:5.5E-4)0.242.7:5.5E-
4)0.820.45:0.0039,UltB1785:0.03461)0.764.77:5.5E-4)0.103.5:5.5E-
4)0.587.10:5.5E-4)0.917.87:0.0079,(BeaProt5:0.0,CurPutat:0.0):5.5E-

4)0.939.72:0.00791,VarSpec4:5.5E-4)0.849.77:0.00393)0.000.610:5.5E-
4)0.863.103:0.00393)0.850.90:0.00392,UltB1826:0.01893)0.612.14:5.5E-
4)0.163.6:5.5E-4,AcdKonj2:0.00368)0.500.11:5.5E-4)0.000.611:5.5E-
4,RamTatao:0.00369)0.000.612:5.5E-4)0.210.13:5.5E-
4,((UltB1930:0.0,UltrBe95:0.0):5.4E-
4,UltrBe80:0.01501)0.836.60:0.00369)0.477.7:5.5E-
4,(XylAmpel:0.0037,(UltB1482:0.00369,((AcdSpec8:0.0,UltB1605:0.0,UltB1706
:0.0):5.5E-4,UltB1688:5.5E-4)0.327.8:5.5E-4)0.541.4:5.5E-
4)0.845.87:0.0037)0.537.10:5.4E-4,(UltB1839:5.5E-
4,(UltB1682:0.00367,(UltB1956:0.00371,((UltB1559:0.0,RamSpeci:0.0,UltRaml
i:0.0,UltB1683:0.0,UltB1699:0.0,UltB1827:0.0):5.5E-4,((UltB1698:5.5E-
4,UltrSoi7:0.0074)0.830.61:0.00369,UltB1681:0.00369)0.660.13:5.5E-
4)0.396.19:5.5E-4)0.848.71:5.5E-
4)0.862.88:0.00367)0.875.97:0.00367)0.446.8:5.4E-
4,VarSpeci:0.00727)0.821.52:0.00381,(((BacSV8XX:5.5E-
4,XnhAzovo:0.00369)0.823.51:5.4E-
4,(AcdSpec6:0.00724,((UltB1802:0.0,UltB1820:0.0):5.5E-
4,CmdBact4:0.00369)0.958.87:0.0111)0.918.91:5.4E-
4)0.608.16:0.0037,(UltrBe66:0.00745,(RamHench:5.5E-
4,UltB1686:0.00369)0.771.84:5.4E-4)0.838.76:0.00368)0.542.9:5.3E-
4)0.873.91:0.00743)0.955.63:5.5E-4,(UltBurk8:0.0,AcdAven2:0.0):5.5E-
4)1.000.605:5.3E-4,((UltB1657:0.0037,((XnhSpeci:0.0,VarSpec5:0.0):5.5E-
4,UltB1691:0.00372)0.305.8:5.5E-
4)0.850.91:0.00362,((UltB1553:0.01525,UltB1693:5.4E-
4)0.839.73:0.0037,HylGraci:0.00755)1.000.606:5.4E-
4)0.157.10:0.0037)0.855.83:0.00373,((AcdSpec5:0.0,XnhAerol:0.0,OttThioo:0
.0,BacteHW1:0.0,UltCom17:0.0):5.5E-
4,(BeaProt2:0.00742,UltB1690:0.00742)0.953.68:5.5E-4)0.000.613:5.5E-
4)0.257.5:5.4E-
4,(UltrBe28:0.0,UltVario:0.0):0.0037)0.905.88:0.0077,UltVari4:5.5E-
4)0.390.12:5.4E-
4,UltVari2:0.01503)0.952.68:0.00508,((BactFA15:0.01501,((UltrSoi3:0.0,Ult
rSoi4:0.0,UltrSoi6:0.0):5.5E-4,UltrSoi5:0.00369)0.995.86:5.5E-
4)0.645.10:0.00739,(UltB1476:0.00369,(VarParad:0.0,UltVari3:0.0):5.5E-
4)1.000.607:5.5E-
4)0.921.103:0.0047)0.944.66:0.00555,(((UltB1694:0.0,VarPara2:0.0):5.5E-
4,(((UltCom25:0.00369,UltrBe63:0.01115)0.651.18:5.5E-
4,((UltCom24:0.0,BeaProt7:0.0,PlrJejue:0.0):5.5E-
4,((UltB1790:0.0037,UltB1794:5.5E-
4)0.880.96:0.00362,((UltB1478:0.0,UltB1756:0.0,AntBac11:0.0,PlrNapht:0.0,
UltB1795:0.0,PlrSpec3:0.0):5.4E-
4,UltB1796:0.00362)0.592.7:0.0075)1.000.608:5.4E-4)0.730.31:5.5E-
4)0.539.2:5.5E-
4,AntBac13:0.00368)0.834.56:0.00369)0.847.99:0.00369,(((UltCom14:5.5E-
4,(((UltB1469:5.5E-4,((UltB1791:0.01862,UltB1798:0.00371)0.862.89:5.5E-
4,(UltB1589:0.0,UltB1786:0.0,UltB1787:0.0,BeaProt6:0.0,PlrSpeci:0.0,UltB1
792:0.0,PlrRhizo:0.0,UltB1797:0.0):5.5E-4)0.931.88:5.5E-4)0.943.81:5.5E-
4,(UltB1793:0.00744,(PlrVacuo:5.5E-
4,AntBac12:0.0111)0.862.90:0.00738)0.733.31:5.5E-
4)0.789.62:0.00366,(UltB1450:0.0,UltB1743:0.0):0.01132)0.774.81:0.00369)0
.940.90:0.00741,((((UltCom15:0.0,UltVari6:0.0,UltB1696:0.0,UltrRape:0.0):
5.5E-4,VarSpec2:5.5E-4)0.000.614:5.5E-
4,(UltB1697:0.00369,(UltB1702:0.0037,UltVari5:0.00741)0.719.19:5.5E-
4)0.912.118:5.5E-4)0.715.19:5.5E-4,UltB1695:0.00369)0.094.4:5.5E-

4)0.124.7:5.4E-4,VarSpec3:5.5E-4)0.476.12:5.5E-
4)0.914.100:0.01091)0.497.2:0.00772)0.998.100:5.5E-4,UltB1806:5.4E-
4)0.841.70:0.00378)0.782.83:0.00406,((((UltB1886:0.0,BeaPro12:0.0,AqbHong
k:0.0):5.5E-4,UltB1887:5.5E-4)0.858.83:5.5E-
4,((UltrB101:0.00757,(PlnBrach:0.00112,UltB1948:0.01476)0.864.85:0.0108)0
.767.63:0.00382,(UltB1597:0.00784,(UltBurk2:0.00734,UltLautr:0.01172)0.97
8.54:0.02431)0.744.47:0.00339)0.927.92:0.01135)0.823.52:0.00368,((((UltB1
898:0.0,BeaPro17:0.0):0.0037,(UltrBe38:5.3E-
4,((UltrBe91:0.0037,AqnTerti:5.4E-
4)0.783.78:0.00371,((((UltB1947:0.01888,((UltUromy:5.5E-
4,(CldSpeci:0.00745,PheBacte:0.0037)0.333.6:5.5E-
4)0.819.54:0.00368,(UltB1803:0.01128,((((UltSlud4:5.5E-
4,(UltrBe99:0.00213,((((BacEnri4:0.01122,(((UltrBe84:5.3E-
4,((UltB1847:0.01142,(UltB1936:5.4E-
4,(((UltComa5:0.00374,UltCom22:0.00745)0.974.48:0.01513,UltB1828:0.01136)
0.825.59:5.4E-4,UltB1925:5.4E-4)0.927.93:0.00366)0.762.79:5.4E-
4)0.829.64:0.00366,(UltB1949:0.00369,(((BacEnri3:0.00372,UltB1926:0.00738
)0.909.79:0.00213,UltrBe93:0.00691)0.895.108:0.00212,LepSpec5:0.02697)0.7
77.80:5.4E-4)0.547.7:5.3E-
4)0.934.80:0.00788)0.797.54:0.00394,(UltGa207:0.01124,UltGreen:5.5E-
4)0.781.63:0.0037)0.843.64:0.0038,((UltB1684:0.01911,((((GroBiof2:0.0,Ult
B1801:0.0):5.4E-4,(UltCom28:0.01551,(UltB1924:0.00368,UltrBe90:5.5E-
4)0.568.9:5.5E-4)0.103.6:0.00382)0.809.60:0.00381,UltB1475:5.5E-
4)0.014.6:5.5E-4,UltAlpha:5.5E-4)0.842.86:0.00384)1.000.609:5.5E-
4,UltB1800:0.0036)0.897.84:0.01181)1.000.610:5.4E-4)0.414.10:5.5E-
4,UltB1953:0.0233)0.885.80:0.00882,UltB1860:0.01088)0.745.60:0.00822,UltB
1950:0.01882)0.897.85:0.00369)0.918.92:0.00266)0.261.6:5.5E-
4,(AzhdLat2:5.5E-4,AzhdLata:5.5E-
4)0.874.107:0.0037)0.863.104:0.00405,(UltB1458:0.01514,(UltB1942:0.01122,
UltrBe98:0.00369)0.572.8:5.5E-4)0.854.82:0.00369)0.533.7:5.5E-
4,(UltB1937:0.0,UltB1938:0.0,LepSpec4:0.0):5.5E-4)0.173.5:5.4E-
4)0.601.6:5.4E-4)0.273.8:0.00403)0.768.62:5.5E-
4,AzhAustr:0.00369)0.364.11:5.4E-4,(UltB1940:0.0,UltB1943:0.0):5.5E-
4)0.635.9:5.5E-
4,(UltB1941:0.00371,UltCom26:0.01127)0.784.73:0.00369)0.797.55:0.00404)0.
786.74:0.00403)0.252.7:5.4E-4)0.938.54:0.00485,(((BeaPro19:5.5E-
4,UltrB100:0.00369)0.901.89:0.00754,(((UltB1899:0.00749,MthPetro:0.00369)
0.801.65:5.5E-4,MthPetr2:5.5E-4)0.888.100:5.4E-4,(((LamHyali:5.4E-
4,UltB1403:0.01493)0.946.82:0.01474,MthSpec2:0.00203)0.954.74:0.00187,(Gr
oBiof3:0.00368,(((UltB1853:0.00369,BeaPro10:0.00378)0.897.86:5.5E-
4,(UltB1851:0.00369,(((UltB1846:0.0,UltB1836:0.0,UltB1837:0.0,UltB1855:0.
0):5.5E-4,AqbSpeci:0.00372)1.000.611:5.5E-4,(UltB1850:5.5E-
4,(((UltB1760:0.01137,UltB1829:0.00751)0.785.71:0.00369,((UltB1840:0.0,Ul
tB1856:0.0,UltB1929:0.0,UltB1933:0.0):5.4E-4,(UltMethy:5.5E-
4,UltB1888:0.0037)0.834.57:0.0037)0.986.62:5.5E-
4)0.922.122:0.0024,UltB1852:0.00251)0.922.123:0.00242)0.454.15:0.0037)0.8
12.53:0.00366)0.801.66:5.5E-4)0.000.615:5.5E-4,MthAquat:5.5E-
4)0.905.89:5.3E-4)0.714.23:5.5E-
4)0.866.85:0.00369)0.780.90:0.00367)0.887.90:0.00748,((UltB1778:0.00368,(
(UltB1443:0.01501,(UltB1522:0.00752,(((((((((UltB1405:0.01124,CmmSpec5:0
.00201)0.753.71:0.00197,(UltB1434:0.00369,UltB1435:5.5E-
4)0.861.92:0.00369)0.916.101:5.4E-
4,((UltB1454:0.0,CmmKerst:0.0,CmmSpec4:0.0,CmmKers2:0.0,UltB1433:0.0,CmmS
pec6:0.0,UltB1546:0.0,UltB1560:0.0):5.5E-

4,UltB1543:0.00369)0.000.616:5.5E-4)0.000.617:5.5E-
4,(UltB1453:0.00369,(UltAnaer:0.0074,UltB1440:0.00369)0.000.618:5.5E-
4)0.279.4:5.5E-4)0.943.82:5.4E-4,(GieAnulu:5.5E-
4,GieVoron:0.00369)0.927.94:0.00749)0.784.74:5.4E-
4,((((UltB1455:0.0,UltB1414:0.0,UltB1429:0.0):5.5E-4,((UltB1430:5.5E-
4,UltB1570:0.01116)0.836.61:0.0037,UltB1426:0.00369)0.757.50:5.5E-
4)0.896.81:5.5E-4,UltB1420:0.01909)0.935.85:5.4E-
4,((UltB1463:0.00371,((UltB1427:0.0,UltB1437:0.0):5.5E-
4,(UltB1431:0.00369,UltB1601:0.00369)0.911.107:5.5E-4)0.888.101:5.5E-
4)1.000.612:5.5E-4,(UltB1428:0.0037,(UltB2195:0.00754,UltB1438:5.5E-
4)0.788.67:5.5E-
4)0.919.116:0.00735)0.353.6:0.00727)0.911.108:0.00725)0.844.86:0.00354,((
(AqbFonti:0.0,UltB1882:0.0,UltB1935:0.0):5.5E-
4,UltB1881:0.00369)1.000.613:5.4E-
4,(UltB1439:0.00738,((CmmAqua2:0.0,CmmAqua3:0.0):5.5E-
4,UltrBe16:0.00369)0.908.101:5.5E-
4)0.878.85:0.00358)0.154.5:0.00367)1.000.614:5.5E-
4,((UltB1404:0.01115,(((UltB1710:0.0,UltB1711:0.0):5.5E-
4,UltB1825:0.0037)0.965.67:5.4E-
4,UltrBe92:0.01125)0.824.56:0.00369)0.866.86:5.5E-
4,((UltB1591:0.0,UltB1707:0.0,UltB1726:0.0):5.5E-
4,(UltB1709:0.0037,((UltB1811:0.0,UltB1812:0.0):5.5E-
4,UltB1918:0.0037)0.786.75:0.00371)0.794.61:5.4E-4)0.759.79:5.5E-
4)0.908.102:5.5E-
4)0.894.89:0.00715,((((UltB1497:0.07381,UltB1494:0.03454)0.931.89:5.5E-
4,UltB1495:0.01116)0.812.54:0.00367,(((AcdSpec9:5.5E-
4,(UltB1468:0.00369,UltB1496:0.01487)1.000.615:0.00207)1.000.616:0.00159,
(((((((UltB1401:0.0037,UltBurk7:5.5E-
4)0.846.95:0.00369,(((((((Udntfde2:0.01118,(UltSludg:0.00369,((UltB1441:0
.0,DiaOryza:0.0,BeaPro18:0.0):5.5E-
4,(IdeSpeci:0.00369,(((((((UltComa6:5.5E-
4,UltBrach:0.02487)0.000.619:5.5E-4,UltComa7:0.0037)0.440.8:5.5E-
4,UltrBe31:0.00368)0.484.10:5.5E-4,UltB1442:5.5E-4)0.452.13:5.5E-
4,CmmSpe11:0.0037)0.136.7:5.5E-
4,XnpSilur:0.0037)0.852.81:0.00388,UltrBe86:0.00744)0.191.7:5.5E-
4)0.108.5:5.5E-4)0.253.10:5.4E-4)0.833.76:0.00388)0.213.7:5.4E-
4,(UltB1520:5.4E-4,(UltB1672:5.4E-
4,(((((UltB1399:0.0,BacteRA1:0.0,UltB1517:0.0,AlhSpeci:0.0,UltB1518:0.0,U
ltrBe32:0.0,UltB1669:0.0):5.5E-4,(UltB1516:5.5E-
4,UltB1670:0.00188)1.000.617:0.00179)0.000.620:5.4E-
4,(((((((UltB1921:0.00369,UltB1571:0.02273)0.999.123:5.5E-
4,IdeDechl:5.5E-4)0.000.621:5.5E-4,UltrBe88:5.5E-4)0.777.81:5.5E-
4,(UltrBe73:5.4E-
4,UltrBe94:0.00744)0.842.87:0.0037)0.796.54:0.00373,UltBurkh:0.00749)0.92
2.124:0.00247,UltB1814:0.00245)0.922.125:0.00245,AlhDenit:0.00741)0.861.9
3:5.5E-4)0.823.53:5.5E-
4,((UltB1402:0.01151,UltrBe29:0.0115)0.756.69:0.00365,(UltB1422:0.01533,(
(UltTepi2:0.0,UltTepid:0.0):0.01133,TpdTherm:0.00372)0.782.84:0.00376)0.7
82.85:0.00369)0.793.66:0.00372)0.844.87:5.4E-
4,UltCom11:0.01921)0.805.56:0.00361)0.398.8:0.0037)0.841.71:0.00363)0.166
.8:5.3E-4,UltB1668:0.00369)0.480.10:5.5E-
4,UltB1493:0.00371)0.484.11:5.5E-
4,(UltB1397:0.0,UltB1502:0.0,DiaSpeci:0.0,AcdSpe10:0.0,UltrBe26:0.0,UltB1
593:0.0,UltB1585:0.0,UltB1612:0.0,UltB1658:0.0,UltSlud2:0.0,UltB1661:0.0,

UltB1663:0.0,UltB1673:0.0,UltB1674:0.0,UltB1808:0.0,UltrBe67:0.0):5.5E-4)0.000.622:5.5E-4,UltB1666:0.01114)0.476.13:5.5E-4,UltB1667:0.00369)0.479.12:5.5E-4)0.472.11:5.5E-4,UltB1538:0.00369)0.480.11:5.5E-4,BrcChiro:0.00369)0.328.10:5.4E-4,UltB1807:5.5E-4)0.243.7:5.5E-4,((BactRM10:0.00369,((CmmTerri:5.5E-4,UltB1564:0.00369)0.818.39:5.5E-4,CmmTerr2:5.5E-4)0.801.67:5.5E-4)0.992.72:5.3E-4,(UltComa9:0.02297,UltB1436:5.4E-4)0.424.15:0.00369)0.849.78:0.00369)0.030.3:5.5E-4,(UltB1410:0.26972,UltB1465:5.3E-4)0.826.56:0.00363)0.000.623:5.5E-4,UltB1634:0.00741)0.301.6:5.5E-4)0.097.7:5.2E-4,UltB1521:5.5E-4)0.247.4:5.5E-4)0.494.6:5.5E-4,((((UltB1425:0.00368,UltB1424:5.5E-4)0.877.106:5.5E-4,(CmmCompo:0.0,UltB1415:0.0,CmmDeni2:0.0,CmmDenit:0.0,CmmSpec3:0.0,CmmNitra:0.0,UltB1432:0.0,CmmSpe10:0.0,UltB1513:0.0,UltB1514:0.0,UltB1587:0.0):5.5E-4)0.729.21:5.5E-4,(UltB1423:0.00367,((UltrBe15:0.0,UltCom12:0.0):5.3E-4,CmmBadia:0.01123)0.102.5:0.00369)1.000.618:5.4E-4)0.915.88:5.4E-4,(((((CmmSpeci:0.0037,(((UltB1406:0.0,UltB1409:0.0,UltComam:0.0,CmmTest2:0.0,CmmTest4:0.0,UltComa2:0.0,CmmSpe12:0.0,CmmSpe13:0.0,UltB1761:0.0):5.5E-4,(BclSpeci:0.01496,UltB1416:5.5E-4)0.840.76:5.5E-4)0.748.55:5.5E-4,(UltB1718:0.00368,UltB1412:0.01493)0.575.5:5.5E-4)0.808.60:5.5E-4)0.750.46:5.5E-4,(UltrBe20:5.5E-4,UltB1464:0.01492)0.840.77:0.00367)0.814.43:5.5E-4,UltB1411:0.00362)1.000.619:5.5E-4,(UltDelft:0.0,CmmTesto:0.0,UltB1413:0.0,CmmTest3:0.0):0.00362)0.141.5:0.00361,BactSM26:0.00362)1.000.620:5.4E-4)0.956.77:0.00726)0.914.101:5.5E-4)0.344.11:0.00376,UltB1889:0.00366)0.972.69:5.5E-4)0.783.79:0.00378)0.921.104:0.00216,UltrBe65:0.01092)0.926.101:0.00189)0.118.7:5.4E-4,(((UltB1583:0.0495,(UltB1592:0.02516,(UltrBe19:0.03551,UltDe142:0.02852)0.675.8:0.02204)0.594.9:0.01562)0.851.84:0.01307,UltB1510:0.02079)0.740.49:0.00594,((UltB1532:0.00741,(((UltB1459:0.0,UltB1460:0.0,UltB1466:0.0,UltB1467:0.0,BrcDeni3:0.0,BrcDeni4:0.0,BrcDeni5:0.0,UltB1393:0.0,UltB1396:0.0,BrcSpeci:0.0,UltB1394:0.0,UltB1395:0.0,UltB1398:0.0,UltB1509:0.0,UltB1511:0.0,UltB1512:0.0,UltB1533:0.0,UltB1554:0.0,UltB1572:0.0,UltB1615:0.0,UltB1616:0.0,UltB1617:0.0,UltB1934:0.0,Ult27544:0.0):5.5E-4,((UltB1392:0.00369,(UltB1452:0.00369,(UltB1613:0.00742,(BrcDenit:0.03741,BrcDeni6:0.00772)0.885.81:5.5E-4)0.442.13:5.5E-4)0.409.8:5.5E-4)0.440.9:5.5E-4,UltB1614:0.00369)0.469.11:5.5E-4)0.397.10:5.5E-4,BrcDeni2:0.00368)0.020.6:5.5E-4)0.847.100:0.004,UltB1524:0.00749)0.348.10:5.5E-4)0.812.55:0.00397)0.818.40:5.4E-4)0.948.91:0.00613)0.948.92:0.00692,(((UltB1507:0.00361,(UltB1843:0.00361,(UltB1946:0.00372,(UltB1931:5.5E-4,UltB1945:5.5E-4)0.967.63:0.01126)1.000.621:5.5E-4)0.931.90:0.01128)0.984.60:5.4E-4,((Udntdde2:0.01148,RubBenzo:0.01179)0.771.85:0.00368,((((BeaProt8:0.0037,(RubGelat:0.0,RubGela2:0.0):5.5E-4)0.862.91:0.0037,((SmpSpeci:0.01552,UltB1783:5.5E-4)0.904.90:0.00769,(UltB1896:5.5E-4,((UltB1932:0.00368,(UltCom27:5.5E-4,LepSpec3:0.0037)0.169.4:5.5E-4)0.922.126:0.00237,(UltrB102:5.3E-4,(((UltrBe96:5.5E-4,UltB1897:0.00362)0.201.8:0.00369,(LepGinse:5.3E-4,UltB1900:0.0037)1.000.622:5.4E-4)0.834.58:0.00379,UltB1577:0.00745)0.256.5:5.4E-4)0.922.127:0.00262)0.922.128:0.00261)0.863.105:0.00386)0.267.8:5.4E-

4)0.328.11:5.5E-4,CmdBacte:5.5E-4)0.275.10:5.3E-
4,UltB1508:0.00369)0.847.101:0.00399)0.611.7:5.4E-
4)0.795.64:0.00399,UltSlud3:0.00747)0.773.72:0.00401)0.762.80:5.5E-
4)0.898.105:0.01077)0.820.46:0.00545)0.820.47:0.01096)0.910.92:0.01078)0.
457.13:5.4E-4)0.867.89:5.5E-4,(((((UltrBe52:5.5E-
4,(UltB1780:0.00369,UltB1557:0.0164)0.484.12:5.5E-4)0.000.624:5.5E-
4,UltB1767:5.5E-4)0.284.5:5.5E-
4,RhfFerme:0.0037)0.892.84:0.00403,(((((DelTsuru:5.5E-4,(UltDelf2:5.5E-
4,(UltDelf7:5.5E-4,CaeKoree:0.00651)0.238.8:5.5E-4)0.000.625:5.5E-
4)0.531.4:5.5E-4,UltDelf3:0.00371)0.769.72:5.5E-
4,(UltB1504:0.00369,(((UltB1500:0.01119,(UltB1501:5.5E-
4,DelSpec2:0.01932)0.815.60:5.3E-4)0.197.8:5.5E-4,UltDelf6:5.5E-
4)0.472.12:5.5E-
4,(UltrBe22:0.0,DelAcido:0.0,UltB1503:0.0,RhbSphae:0.0,DelAcid2:0.0,UltDe
lf4:0.0,UltrBe23:0.0,UltB1506:0.0):5.5E-4)0.470.4:5.5E-4)0.466.11:5.5E-
4)0.590.8:5.3E-4,(((LepCholo:0.0,LepDisco:0.0):0.00372,(UltB1832:5.5E-
4,((UltrBe75:0.0,AqbCitra:0.0,UltrBe76:0.0):5.5E-
4,UltB1880:0.00742)0.981.67:0.01509)0.791.61:0.00374)0.905.90:0.00755,(Ra
pRhiz2:5.4E-4,((((MitChito:0.01867,(RosDepo2:5.5E-
4,RosDepol:0.0037)0.887.91:0.00717)0.860.64:5.4E-
4,(UltB1914:0.0037,((UltB1902:0.00369,(((UltB1923:5.5E-
4,(UltB1754:0.00369,((UltB1547:0.0,UltB1770:0.0):5.5E-
4,UltrBe55:0.00371)0.806.46:0.00368)0.809.61:0.00372)0.844.88:0.00368,(((
UltB1952:0.00745,(((AntBac15:0.0,UltB1903:0.0,UltB1904:0.0,PelSacch:0.0,P
elSacc2:0.0,PelSacc3:0.0,PelSacc4:0.0):5.5E-4,((((UltB1665:5.5E-
4,((UltB1751:0.00742,(UltB1408:0.0,UltB1708:0.0):0.00369)0.000.626:5.5E-
4,((UltB1417:0.0,UltB1664:0.0,UltB1731:0.0,UltB1733:0.0,UltB1734:0.0,UltB
1735:0.0):5.5E-
4,(((((UltB1906:0.00371,(((UltB1418:0.0,UltB1545:0.0):0.00369,(UltB1419:0
.0,CmmOdont:0.0):5.5E-4)0.721.18:5.4E-4,(CmmSpec2:5.5E-
4,(((UltB1444:0.0,UltB1461:0.0,CmmSpec8:0.0,CmmSpec7:0.0,BeaProte:0.0,Acd
Spe11:0.0,UltB1523:0.0,UltB1573:0.0,UltB1611:0.0,UltB1722:0.0,UltB1723:0.
0,UltB1732:0.0):5.5E-4,UltrBe42:0.00369)0.834.59:0.00369,UltrBe24:5.4E-
4)0.848.72:0.0037)0.859.83:0.00369)0.881.99:0.00751)0.881.100:0.00751,(Ul
tB1911:0.0037,UltB1905:5.5E-4)0.765.58:5.5E-
4)0.825.60:0.00369,(BeaPro13:0.0,BeaPro14:0.0):5.5E-
4)0.806.47:0.0037,(AcdSpeci:5.4E-
4,UltrBe64:0.00747)0.784.75:0.00371)0.791.62:0.00369,(UltB1659:0.00375,(U
ltB1810:0.01138,((UltB1635:0.00378,((BacCYCU0:0.0,CmmSpec9:0.0,UltB1498:0
.0,UltB1499:0.0,UltB1917:0.0,LepSpeci:0.0):5.3E-4,(PsvInter:5.5E-
4,UltB1662:0.00739)0.825.61:0.00369)0.783.80:0.00369)0.786.76:0.00369,((U
ltB1817:0.03451,(UltB1519:6.3E-
4,UltB1809:0.04709)0.962.83:0.0034)0.480.12:5.4E-
4,(UltB1544:0.0,UltB1816:0.0):5.5E-4)0.684.12:5.5E-
4)0.770.81:0.00403)0.896.82:0.00826)0.743.54:0.00409)0.775.74:0.00408)0.0
00.627:5.5E-4)0.351.11:5.3E-4)0.000.628:5.5E-
4,UltCom19:0.00368)0.510.7:5.5E-4,(CmmKoree:0.00747,(UltComa3:5.5E-
4,(BacteRM4:0.00369,(UltComa4:0.0,UltBur10:0.0):5.5E-4)0.756.70:5.5E-
4)0.945.72:5.5E-
4)0.877.107:0.00366)0.850.92:0.00398,((((BeaPro15:0.0,PelAquat:0.0,UltB19
07:0.0,UltB1909:0.0,UltB1910:0.0,IroBact9:0.0):5.5E-
4,(BeaPro11:0.0037,((BrkSpeci:0.0,PdnBacte:0.0):0.00368,PsdSp155:0.01133)
0.787.63:0.00375)0.904.91:5.5E-4)0.737.53:5.4E-4,UltB1908:5.5E-
4)0.922.129:5.5E-4,UltPelom:0.01114)0.829.65:0.00368)0.024.6:5.4E-

4)0.847.102:0.00397,UltGa206:5.3E-4)0.864.86:0.00418)0.310.9:5.5E-
4,(AcdValer:0.0,UltrBe79:0.0,PauToxi2:0.0,PauToxin:0.0,PsdSp154:0.0,UltB1
915:0.0,UltB1916:0.0):5.5E-4)0.021.3:5.5E-
4,UltrBe97:0.00369)0.314.8:5.4E-
4)0.850.93:0.00418,(UltB1830:0.00758,UltrBe69:0.00368)0.899.95:0.00752)0.
117.7:5.4E-4)0.397.11:5.5E-
4,(UltrBe70:0.0,AntBac14:0.0,UltB1901:0.0,GroBiof4:0.0,UltB1912:0.0,MitCh
it2:0.0):5.5E-4)0.000.629:5.5E-4)0.231.9:5.5E-4)0.610.8:5.5E-
4,BeaPro16:0.02265)0.835.70:0.0041,(UltB1704:0.0112,(((UltB1472:0.0,UltB1
515:0.0,UltrBe40:0.0,UltB1703:0.0):5.5E-4,(UltB1588:5.5E-
4,((UltB1804:0.0159,AquFonta:5.5E-4)0.176.12:5.5E-
4,(((UltrBe89:0.0037,UltGa205:5.5E-4)1.000.623:5.4E-
4,UltrBe39:0.00362)0.051.4:0.00369,(((UltB1677:0.0,UltB1676:0.0,UltB1678:
0.0,UltB1680:0.0):5.5E-
4,(UltB1701:0.00369,UltB1679:0.00741)0.696.15:5.5E-4)0.814.44:5.4E-
4,UltB1700:0.01124)0.889.88:0.00361)0.998.101:5.1E-
4)0.936.85:0.00392)0.320.4:5.5E-4)0.158.7:5.4E-
4,UltB1477:0.00369)0.595.6:5.5E-4)0.843.65:0.00392)0.451.15:5.3E-
4)0.313.7:5.3E-4)0.819.55:0.00421)0.965.68:5.5E-
4,UltB1505:0.07821)0.867.90:0.00839)0.845.88:5.4E-
4,((UltB1556:0.00369,UltB1769:5.5E-
4)0.779.73:0.00372,UltrBe61:0.00748)0.777.82:0.0037)0.905.91:0.00937)0.84
0.78:0.01024)0.998.102:5.3E-
4,((UltBa751:0.04112,(UltB1954:0.04189,UltB1965:0.05087)0.720.22:0.00941)
0.503.5:0.01957,(BacUWNR1:0.31143,((UltB2142:0.13711,UltB2151:0.00833)0.6
94.12:0.00266,((OxlBacte:0.00714,(((MssNiast:0.0,MssAurea:0.0,UltB2120:0.
0):5.5E-4,(UltOxal2:0.00746,UdntBeta:0.01113)0.784.76:5.5E-
4)0.966.87:5.3E-4,(((UltB2121:0.01966,((JanSpec4:5.5E-
4,((((UltB2135:0.00373,UltB2145:0.00368)0.911.109:0.00744,(UltrB121:0.0,O
xlBact3:0.0):0.00742)0.747.49:5.5E-4,OxlBact2:5.5E-4)0.936.86:5.5E-
4,((MssSpeci:0.0,UltrB120:0.0,UltB2137:0.0,UltB2138:0.0):0.00374,RaiTrout
:0.01875)0.409.9:0.00369)0.922.130:0.00738)0.658.11:0.00374,(TelChiti:0.0
1138,((MssAlbid:5.4E-4,(MsslDura:5.4E-
4,(UltrRap2:0.0,UltMass2:0.0):0.00369)0.907.85:0.00744)0.837.57:5.4E-
4,(UltB2113:0.00369,(((UltB2107:0.0,UltB2109:0.0):5.5E-4,UltB2112:5.5E-
4)1.000.624:5.5E-4,(UltB2108:0.00369,UltB2106:0.01128)0.681.11:5.5E-
4)0.786.77:5.5E-
4)0.944.67:0.01128)0.354.9:0.00372)0.792.79:0.00376)0.844.89:0.00704)0.68
3.21:0.00754,(((MssSpec2:0.0,UltB2114:0.0,UltrB118:0.0,MssAeril:0.0,MssSp
ec3:0.0,JanSpeci:0.0):5.5E-
4,(UltrB117:0.00369,UltB2111:0.0037)0.902.98:5.5E-4)0.915.89:5.5E-
4,UltrB119:0.00368)0.868.68:5.4E-
4)0.904.92:0.01059,((UltNaxi2:0.00753,(NaxHaem2:5.5E-4,(OxaSpeci:5.5E-
4,(NaxHaema:0.0,UltB2117:0.0):5.5E-4)0.716.21:5.5E-4)0.905.92:5.5E-
4)1.000.625:5.4E-
4,(((UltB2115:0.0,NaxAlkal:0.0,MssTimon:0.0,MssSpec4:0.0,UltB2118:0.0,Mss
Brevi:0.0,UltB2122:0.0,MssSpec5:0.0):5.5E-4,(UltNaxib:5.5E-
4,(MslSpeci:0.00369,(TelMixta:0.00742,(TelMixt2:0.0,UltB2187:0.0):5.5E-
4)0.871.93:0.00369)0.708.25:5.5E-4)0.000.630:5.5E-4)1.000.626:5.4E-
4,((((UltB2130:5.5E-4,(UltB2129:5.5E-4,UltB2190:5.5E-4)0.000.631:5.5E-
4)0.000.632:5.5E-
4,((UltB2110:0.0,MsslCf00:0.0,MssTimo3:0.0,MssTimo4:0.0,UltB2119:0.0,OxaS
pec2:0.0,JanSpec2:0.0,UltB2124:0.0,UltB2127:0.0,AntBac16:0.0,UltB2189:0.0
):5.5E-4,(MssTimo2:0.00369,UltOxalo:0.00369)0.883.101:5.5E-

4)0.000.633:5.5E-4)0.000.634:5.5E-
4,(UltB2128:0.00369,(UltMassi:0.00385,UltB2123:5.5E-4)0.720.23:5.5E-
4)0.000.635:5.5E-4)0.806.48:5.5E-4,((UltB2146:5.5E-
4,UltB2147:0.00368)0.887.92:0.0036,((JanSpec3:5.5E-
4,(AntBac17:0.00742,UltB2132:5.5E-4)0.505.3:5.5E-
4)0.877.108:0.00358,(UltrSo11:0.0037,(UltB2131:0.0,UltB2133:0.0):0.00747)
1.000.627:5.5E-4)0.853.68:0.00736)1.000.628:5.4E-
4)0.892.85:0.00735)0.123.7:0.00738)0.896.83:0.00704)0.921.105:5.5E-
4)0.778.73:0.00391)0.488.11:0.00724,(((UltB2158:0.02648,((UltB2143:0.0439
7,UltB2157:0.05894)0.979.65:0.02767,(UltB2152:0.06078,UltB2144:0.0934)0.8
24.57:0.00958)0.745.61:0.00336)0.372.10:5.5E-
4,UltB2153:0.01867)0.807.49:0.00349,((UltrB122:0.00367,UltB2150:0.02254)0
.121.5:5.4E-
4,(((UltB2134:0.0,UltB2136:0.0,AntBac18:0.0,JanAgari:0.0,UltB2154:0.0,Ult
B2155:0.0,JanSpec5:0.0,UltB2161:0.0,BacTHCL2:0.0,UltB2209:0.0):5.5E-
4,(((UltB2194:0.00369,(UltB2139:0.00369,((UltB2141:0.02677,UltB2149:0.054
92)0.884.100:5.4E-4,((UltB2159:0.0,UltB2192:0.0):5.5E-
4,(UltB2156:0.00369,UltB2160:0.00369)0.244.5:5.5E-
4)0.922.131:0.00369)0.192.8:5.5E-4)0.484.13:5.5E-4)0.000.636:5.5E-
4,((UltB2116:0.0,UltB2126:0.0):5.4E-
4,UltB2188:0.00752)0.847.103:0.00369)0.478.9:5.5E-
4,UltB2193:0.00369)0.487.14:5.5E-4)0.419.7:5.5E-
4,((UltB2191:0.03895,UltB2140:0.00746)0.302.6:0.00369,UltB2148:0.01869)0.
822.50:5.5E-4)0.976.53:5.5E-4)0.752.64:5.5E-4)1.000.629:5.5E-
4)0.905.93:0.0033)0.822.51:0.02126)0.787.64:0.01701)0.954.75:0.02506)0.70
1.20:0.00192,(((UltB1951:0.04159,BrhBact2:0.00271)0.985.65:0.03702,(((Ult
B1913:0.00868,UltrBe83:0.00639)0.945.73:0.02038,(UltB1848:5.4E-
4,BacEnri2:0.01096)0.880.97:0.01709)0.808.61:0.00918,(((UltB1845:0.00369,
(SclTher2:0.0,SclTherm:0.0,BeaPro21:0.0,SclAquat:0.0):5.5E-
4)0.874.108:0.00754,(ThmSpeci:0.00767,(ThmSpec2:0.01148,(UltB1966:0.00381
,(ThmSpec3:5.5E-4,ThmSpec4:5.5E-
4)0.776.79:0.00364)0.901.90:0.01167)0.853.69:0.00785)0.887.93:0.01153)0.8
58.84:0.01573,(UltB1968:0.00516,(ThmCupri:0.01126,UltPro19:0.02367)0.955.
64:0.01917)0.922.132:0.0149)0.000.637:5.4E-
4)0.946.83:0.01526)0.988.70:5.4E-
4,(((UltB1490:0.00741,UltB1491:0.00752)0.961.71:0.01897,(UltB1671:0.01601
,(UltCom10:5.4E-
4,UltBurk4:0.00721)0.995.87:0.04123)0.857.90:0.01148)0.915.90:0.005,(UltB
1649:0.00362,((UltComa8:0.0,UltPro18:0.0):5.4E-
4,(((UltrBe37:0.00369,(UltB1445:0.0,UltB1645:0.0,UltB1644:0.0,UltB1646:0.
0,UltB1647:0.0,UltB1651:0.0,UltB1652:0.0,UltBurk6:0.0,UltB1715:0.0,UltB17
17:0.0):0.0037)0.914.102:5.5E-4,UltB1653:5.5E-4)1.000.630:5.5E-
4,UltB1650:0.1217)0.823.54:0.0036)0.875.98:0.00753)0.937.86:0.01421)0.908
.103:0.00344)0.880.98:0.00843)0.717.25:0.00711)0.830.62:0.00938,(UltB1895
:0.03141,(UltB2197:0.02415,(UltB1383:0.03482,UltB2440:0.06516)0.837.58:0.
03026)0.913.97:0.02294)0.722.21:0.00522)0.896.84:0.0115)0.308.8:0.00168,(
((Ult31662:0.00371,Ult31663:0.00743)0.975.60:0.03485,((Ult27538:0.03066,U
lt27540:0.01789)0.751.43:0.00556,(Ult27529:0.02803,(Ult27527:0.01783,Ult2
7528:0.03475)0.946.84:0.02228)0.909.80:0.01567)0.921.106:0.01728)0.958.88
:0.02265,((((((((RlsEutro:5.5E-
4,(((((((CprTaiw3:0.00746,CprSpeci:0.00371)0.769.73:0.00341,(CprTaiwa:0.
00372,(CprBasil:5.5E-
4,((CprBasi2:0.0,UltB2239:0.0,CprNeca3:0.0,CprNeca4:0.0,CprRespi:0.0,CprR
esp2:0.0,UltB2240:0.0):5.5E-

4,(CprNeca2:0.00379,RlsSpec4:0.00369)0.276.9:5.5E-4)0.374.10:5.5E-
4)0.902.99:0.00775)0.785.72:0.0041)0.966.88:0.01957,CprNecat:0.00714)0.77
6.80:5.4E-4,CprTaiw2:5.5E-
4)0.943.83:0.01151,CprResp3:0.03915)0.957.68:5.5E-4,RlsSpec3:5.5E-
4)0.864.87:0.00365,((UltRals6:0.0,RlsMetal:0.0,CprMetal:0.0):5.4E-
4,(CprCampi:0.0,CprPaucu:0.0):0.0036)0.152.9:0.0037)1.000.631:5.4E-
4,UltB2238:0.01122)0.464.10:0.00731)0.970.70:0.01846,(((((UltB2243:0.0,Ul
tB2244:0.0,UltB2241:0.0,PlcSpeci:0.0):5.5E-4,(((UltrB131:5.5E-
4,UltB2246:0.00369)0.911.110:0.01123,UltB2245:0.00765)0.277.9:0.00365,Plc
Neces:0.0111)0.889.89:5.5E-
4)0.720.24:0.00264,((UltB1759:0.03555,UltB1716:0.00359)0.929.81:0.0114,Ul
tB1569:5.4E-
4)0.962.84:0.02027)0.987.89:0.03157,UltB2247:0.00967)0.809.62:0.01423,Ult
B1654:0.01716)0.531.5:0.01058)0.762.81:0.00492,UltB2544:0.00779)0.844.90:
0.00731,((BeaPro29:0.01144,((((UltB2542:0.0,UltB2541:0.0):5.3E-
4,(UltB2512:5.5E-4,(((DecAgita:0.0,UltB2513:0.0):5.5E-
4,(UltB2521:0.01777,(BacEnr52:0.00375,(DecSpeci:0.02263,UltB2509:5.5E-
4)0.765.59:0.00371)0.245.6:0.00762)0.776.81:0.00555)0.931.91:0.00218,UltB
2517:0.02289)0.986.63:0.00164)0.917.88:0.00732)0.898.106:0.00739,(AzvSpec
i:0.0037,(((Ult27541:0.01134,(UltrB180:0.00742,(UltB2507:5.5E-
4,Ult27542:5.5E-4)0.944.68:5.4E-4)0.428.12:0.00369)0.958.89:5.4E-
4,((DecSpec2:0.0,DecHorte:0.0,Ult27543:0.0):5.5E-4,UltB2514:5.5E-
4)0.000.638:5.5E-4)0.811.57:5.3E-
4,UltB2508:0.00369)0.783.81:0.00371)0.803.51:0.00379)0.798.62:0.00368,((U
ltB2526:0.01529,((UltB2522:5.4E-
4,(UltB2527:0.0115,UltB2505:0.01924)0.763.81:0.00345)0.855.84:0.00368,Azn
Caeni:5.5E-4)0.134.10:5.5E-
4)0.937.87:0.00752,(UltB2518:0.03887,(((BeaPro30:5.5E-
4,MtlUnive:0.00371)0.783.82:0.00374,(UltB2489:0.01131,UltB2483:0.00371)0.
763.82:0.00362)0.795.65:0.00363,(UltB2548:5.5E-4,(UltB2486:5.5E-
4,(((UltB2485:0.01529,(((AzcAnaer:0.0,UltB2484:0.0,UltrB178:0.0):5.5E-
4,AzcToluv:5.5E-4)0.629.15:5.5E-4,AzcSpec3:0.015)0.177.12:5.4E-
4)0.809.63:0.00387,((PshYyy12:0.0,PshgggY2:0.0):5.5E-
4,PshggY02:0.0345)0.975.61:0.01893)0.000.639:5.5E-4,(AzcSpec2:5.4E-
4,AzcToluc:0.00369)0.781.64:0.00371)0.599.7:5.4E-4)0.551.11:5.4E-
4)0.683.22:5.5E-4)0.917.89:0.01142)0.879.95:5.5E-
4)0.764.78:0.00379)0.887.94:0.00763)0.761.62:0.00361,UltRho13:0.00749)0.7
73.73:0.00374)0.881.101:5.5E-
4,((UltB2525:0.0,UltrB184:0.0,Otu00052:0.0):0.00367,(UltrB185:0.01122,Z01
14630:0.00369)0.867.91:5.5E-
4)0.999.124:0.04337)0.849.79:0.0119,(((AtPYy082:0.01218,((((((UltrB174:5.
4E-4,(UltrB173:0.01099,Otu00113:0.01111)0.848.73:5.4E-
4)0.849.80:0.01112,(Otu00172:0.02405,(Otu00684:0.02983,(Otu00233:0.02277,
UltrB175:0.03007)0.732.24:0.00483)0.617.7:0.00193)0.897.87:0.01548)0.903.
85:0.01246,((Otu00083:0.01262,S0114582:0.05091)0.657.11:0.00265,Otu00202:
0.00364)0.753.72:0.00373)0.616.10:0.00381,Otu00506:0.00771)0.853.70:0.008
28,UltrB183:0.01485)0.728.34:0.01252,UltrB182:0.0258)0.675.9:0.00835)0.55
9.8:5.5E-
4,Otu00269:0.02593)0.756.71:0.00756,((UltB2524:0.0,UltrB181:0.0):5.5E-
4,EchggYyy:0.04429)0.889.90:0.01035)0.859.84:0.00768)0.850.94:0.00788,(Ul
tB2445:0.02315,(((PahggYy2:0.00371,(UltB2528:0.00373,UltrB186:0.00371)0.8
86.113:0.00761)0.980.52:0.00936,((UltPro29:0.00752,((Otu00139:0.01448,(Ot
u00051:0.01206,Otu00055:0.02806)0.955.65:0.0321)0.901.91:5.3E-
4,Otu00073:0.01117)0.751.44:5.4E-4)0.809.64:5.5E-

4,(UltB2529:0.00772,(UltB2530:5.5E-
4,UltB2534:0.00369)0.937.88:0.01518)0.168.8:0.00754)0.966.89:0.00214)0.96
7.64:0.03604,(CanProca:0.00355,CanProc2:0.00387)0.986.64:0.04563)0.888.10
2:0.01661)0.934.81:0.02322)0.808.62:0.00795,((((((CanGlome:0.0205,(BtSmbO
f0:0.00769,(CanTrem2:0.01344,CanTremb:0.00617)0.934.82:0.03139)1.000.632:
0.15417)0.814.45:0.02501,(UltB2443:5.5E-
4,UltB2444:0.00368)1.000.633:0.14145)0.892.86:0.02589,((((((((BrkMulti:5
.4E-4,((BrkGlat3:0.0,BrkGlat4:0.0,BrkGlat5:0.0):5.5E-
4,(((BrkSpe45:0.0,BrkSpe46:0.0,BrkMult2:0.0):5.4E-
4,((BrkSarti:0.00369,((((BrkSpe22:0.0,UltrB114:0.0):5.5E-
4,UltBur23:0.00369)0.939.73:0.00747,(BrkSpe29:0.00363,((BrkSpe33:0.00738,
(BrkSpe32:5.5E-4,(UltBur24:0.00369,(((PsdSp156:5.5E-
4,BrkTerri:0.01641)0.320.5:5.5E-
4,(BrkTerr2:0.0,BrkSpe25:0.0,BrkBryop:0.0,BrkSpe28:0.0,BrkSpe35:0.0,BrkPh
ena:0.0,BrkSpe47:0.0):5.5E-4)0.246.11:5.5E-
4,UltB2083:0.00369)0.463.9:5.5E-4)0.023.3:5.5E-4)0.462.13:5.5E-
4)0.862.92:0.00396,((UltrB115:5.5E-4,BrkPhyt2:0.00293)1.000.634:7.3E-
4,((BrkPheno:0.0,BrkCepa2:0.0,BrkXenov:0.0,BrkGrami:0.0,BrkSpe30:0.0,BrkS
pe31:0.0,UltB2082:0.0,UltEubac:0.0,BrkSpe26:0.0,BrkCaled:0.0,BrkPhyto:0.0
,UltBur28:0.0):5.5E-4,BrkSpe27:0.00372)0.584.9:5.4E-4)0.844.91:5.3E-
4)0.144.7:0.00402)0.999.125:5.5E-
4)0.829.66:0.00402,(UltB2079:0.0,UltB2084:0.0):5.5E-4)0.120.8:5.5E-
4)0.827.59:0.00398,((BrkSpe42:0.0,BrkSpe43:0.0,BrkSordi:0.0,UltB2086:0.0)
:5.5E-4,(BrkGlath:0.00369,BrkGlat2:0.00369)0.678.15:5.5E-4)0.492.15:5.5E-
4)0.745.62:0.00819)0.911.111:0.00811,BrkSpe49:0.00368)0.090.8:5.4E-
4)0.879.96:0.004)0.795.66:0.00395,((BrkGladi:0.0,BrkGlad2:0.0,BrkPlant:0.
0,BrkGlad3:0.0,BrkPlan2:0.0):0.0037,(((UltB2093:0.0074,UltB2094:0.00737)0
.800.58:0.00367,((UltB2092:0.00369,((UltB2091:5.5E-
4,(BrkCepa3:0.0,BrkCepa5:0.0,BrkCepa7:0.0,BrkSpe52:0.0):0.00219)1.000.635
:0.0015,((BrkSpe50:0.00373,((((UltrSo10:0.0111,(CanBurk2:0.00748,CanBurk3
:0.01138)0.804.53:0.00365)0.855.85:5.5E-
4,(BrkSpe37:0.0,BrkSpe40:0.0,BrkSymb2:0.0):5.5E-4)0.688.15:5.5E-
4,UltBur25:0.00367)0.938.55:0.0074,((BrkSpe34:0.0,BrkSpe36:0.0,BrkSymbi:0
.0,UltB2085:0.0,BrkSpe41:0.0):5.5E-4,CanBurkh:0.00366)0.802.54:5.5E-
4)0.943.84:0.01181)0.868.69:0.00783,(BrkRhizo:0.00369,(BrkSpe38:0.0,BrkSp
e39:0.0):5.5E-4)0.881.102:0.00751)0.803.52:0.00383)0.115.3:5.5E-
4)0.337.6:5.5E-
4,(BrkMalle:0.0,BrkCepa4:0.0,UltB2095:0.0,BrkCepa6:0.0,UltB2096:0.0):5.5E
-4)0.582.9:5.5E-4)0.021.4:5.5E-4,(UltB2097:0.01121,(BrkSpe51:5.4E-
4,(((BrkSpe20:0.0,BrkSpe21:0.0,BrkPhyma:0.0,BrkSpe24:0.0,BrkHospi:0.0,Brk
Spe23:0.0,BrkCaryo:0.0):5.5E-
4,(UltB2090:0.00369,((BrkCarib:0.0,BrkTerra:0.0):0.00369,(BrkSpe48:0.0080
1,((BrkSpec6:0.0,BrkSpec7:0.0):0.01099,(((((BrkSpe18:5.4E-
4,(UltBur22:5.5E-4,(UltB2078:0.00369,UltRume8:5.5E-
4)0.841.72:0.00368)0.936.87:0.00718)0.449.23:0.00721,(BrkSpec9:0.0,BrkSpe
19:0.0):0.01124)0.784.77:5.4E-4,((UltPro22:0.0,BrkSpec8:0.0):5.5E-
4,((BrkSpec3:0.0,BrkNodos:0.0,UltB2076:0.0,BrkMimos:0.0,BrkSpec5:0.0,BrkS
ilva:0.0,BrkSpe44:0.0):5.4E-4,(BrkSpe15:5.5E-4,(BrkSpe14:5.5E-
4,(((BrkUnama:5.5E-
4,BrkSpec4:0.00744)0.926.102:0.00696,((BrkTrop4:0.01873,(BrkTropi:0.0,Brk
Trop3:0.0):5.5E-4)1.000.636:5.5E-
4,BrkTrop2:0.03063)0.897.88:0.00206)0.898.107:0.00209,UltrSoi9:5.4E-
4)0.925.106:0.00764)0.212.8:5.5E-
4)0.914.103:0.00761)0.922.133:0.00757)0.216.7:5.4E-4)0.395.12:5.4E-

4,BrkSpe10:0.00369)0.779.74:0.00724,(BrkKurur:0.0,BrkSpe11:0.0,BrkTuber:0
.0,BrkSpe12:0.0,BrkSpe13:0.0,BrkSpe16:0.0,BrkSpe17:0.0):5.5E-
4)0.541.5:0.01202)0.949.85:0.01704)0.888.103:0.01124)0.611.8:5.4E-
4)0.155.8:5.5E-4)0.607.8:5.5E-
4,BrkHosp2:0.01116)0.860.65:0.00376)0.895.109:5.5E-
4)0.944.69:0.00761)0.797.56:0.00389)0.882.94:0.00793)0.889.91:0.00878,((U
ltB2088:5.4E-
4,(ThmCupr2:0.08831,UltB2326:0.0252)0.816.60:0.00978)0.771.86:0.00373,Brk
Spe53:0.00369)0.881.103:0.0075)0.777.83:0.00428,(((UltB2515:0.00743,(((Ul
tB2520:5.5E-4,(ThbclQ00:0.00369,(BacROME3:0.0,BeaPro38:0.0):5.5E-
4)0.694.13:5.5E-4)0.606.11:5.4E-4,(UltB2504:5.5E-
4,UltB2523:0.00368)0.972.70:0.01123)0.945.74:0.01088,QuaAustr:0.01494)0.9
14.104:5.3E-
4)0.813.56:0.01157,((UltB1595:0.03115,(UltB2362:0.01883,FrbLimne:5.5E-
4)0.731.25:0.00314)0.764.79:0.00743,(UltB2506:5.5E-
4,UltB2516:0.00368)1.000.637:0.03926)0.793.67:5.5E-4)0.666.17:5.5E-
4,(UltB2519:5.4E-4,((AzcIndig:0.0,UltrB177:0.0):0.00761,(ThrButan:5.5E-
4,ThrLinal:5.5E-
4)0.785.73:0.00364)0.973.54:0.01532)0.938.56:0.00748)0.927.95:0.00751)0.6
50.12:5.5E-4,UltB2359:0.03494)0.533.8:5.4E-4,((BrkAndr2:5.5E-
4,BrkAndro:5.5E-
4)0.973.55:0.01927,((UltrSo17:0.00319,UltB2304:0.01569)0.174.7:0.00538,((
(BacTG149:0.0037,((UltB2302:5.4E-
4,UltB2301:0.02723)0.793.68:0.00369,UltB2068:0.00744)0.465.11:5.4E-
4)0.939.74:0.01143,UltB2307:0.00761)0.722.22:0.00233,UltB2303:0.00873)0.9
01.92:0.01411)0.874.109:0.01239)0.940.91:0.01508)0.126.7:0.00597,UltB2434
:0.01858)0.893.95:0.0115,((((UltB2538:0.01118,(UltB2543:5.5E-
4,((UltB2426:0.0,UltB2536:0.0):5.5E-
4,(UltB2537:0.00745,UltB2535:0.00369)0.729.22:5.5E-4)0.931.92:5.5E-
4)0.947.69:5.5E-4)0.542.10:0.00365,((AzpRestr:0.0,UltB2540:0.0):5.4E-
4,(DchSpeci:0.01914,(UltB2510:5.5E-
4,UltGa209:0.0037)0.965.69:0.01529)0.733.32:0.00356)0.982.55:5.4E-
4)0.927.96:0.0074,(UltB2511:0.01121,((RhcTenui:0.00366,(RhcPurpu:5.5E-
4,(UltRho22:0.00749,((SteDenit:5.4E-
4,UltRhod8:0.00369)0.986.65:0.01924,(BeaPro23:0.01136,(UltB2480:0.0,UltB2
481:0.0):0.00752)0.769.74:0.00367)0.784.78:0.00379)0.783.83:0.00369)0.782
.86:0.00381)0.773.74:0.00373,RhcTenu2:0.00758)0.894.90:0.00768)0.645.11:5
.5E-4)0.949.86:0.00195,UltB2539:0.0148)0.938.57:0.00191)0.315.11:5.4E-
4,((Otu00246:0.02589,(((UltB2501:0.00364,(BeaPro37:5.5E-
4,UltB2500:0.00369)1.000.638:5.3E-4)0.236.4:0.00795,(UltB2502:5.5E-
4,UltB2503:0.02334)0.748.56:0.00336)0.903.86:0.02361,(ChiTaiwa:0.02413,Ch
iKoree:0.03128)0.577.6:0.02037)0.878.86:0.01757)0.867.92:0.0144,(UltrB187
:0.03481,(PrpLimic:0.01942,((((((UltrB163:0.00375,((UltB2431:0.0,UltB2432
:0.0):5.5E-
4,UltB1598:0.02294)0.802.55:0.00749)0.874.110:0.00755,UltRhod9:0.00756)0.
912.119:0.01136,(UltB1599:0.01111,(((UltB2437:5.5E-4,(UltB2428:5.5E-
4,UltB2435:0.00369)0.202.10:5.5E-4)0.876.79:5.4E-
4,(UltB2427:0.01136,(UltB1382:0.01114,UltGalli:5.5E-4)0.880.99:5.4E-
4)0.920.104:0.01103)0.543.11:0.00764,(GllSpeci:0.00749,UltB2436:0.01515)0
.848.74:0.007)0.893.96:0.01154)0.887.95:5.5E-4)1.000.639:5.5E-
4,(((((UltB2216:5.5E-4,((UltB1527:0.02861,(OxaFormi:5.5E-
4,OxaForm2:0.00373)0.999.126:0.04185)0.799.71:0.00647,(UltB2125:0.00382,U
ltB2180:0.02312)0.771.87:0.00357)0.999.127:5.4E-
4)0.829.67:0.00367,(((UltB2204:0.0,OxlBact5:0.0):5.5E-

4,((((((UltB2164:0.00384,UltB2223:0.00741)0.904.93:0.0132,(UltB2165:0.007
44,(UltB2163:0.00741,(AqsArcti:5.5E-4,UltB2162:0.00369)0.366.8:5.5E-
4)0.790.69:0.00371)0.820.48:0.00778)0.886.114:0.01303,(((UltB2166:5.5E-
4,UltB2205:5.5E-4)0.879.97:0.00369,((AntBac19:0.0,AntBac20:0.0):5.5E-
4,UltB2219:0.00369)0.093.8:5.5E-4)0.842.88:0.00368,UltB2213:5.3E-
4)0.940.92:5.3E-4)0.913.98:0.00425,(UltB2212:5.4E-
4,(UltrB124:0.00777,UltB2211:0.00736)0.322.12:0.00376)0.898.108:0.00422)0
.907.86:0.00989,((((LimSpeci:0.0,UltB2099:0.0,UltB2100:0.0):0.0037,UltB21
01:5.4E-
4)0.897.89:0.00755,(((UltB1985:0.02689,((SutParvi:0.00546,((UltB1981:0.00
746,((UltB1982:5.5E-4,UltB1980:5.5E-
4)0.843.66:0.00368,(UltB1978:0.00369,(UltB1979:0.00369,Ult20652:0.00369)0
.108.6:5.5E-4)0.895.110:5.5E-
4)0.882.95:0.00747)0.939.75:0.0144,UltB1983:0.00998)0.792.80:0.00502)0.94
0.93:0.01504,((UltB1971:5.5E-
4,((UltB1970:0.03856,(((UltB1972:0.0,UltB1973:0.0,UltB1976:0.0,UltB1977:0
.0,UltB7033:0.0,Ult20651:0.0):5.5E-
4,(UltB1969:0.00369,UltB1975:0.00369)0.711.25:5.5E-4)0.928.71:5.5E-
4,(GutMetag:0.0,UltB1974:0.0):0.02709)0.000.640:5.5E-4)0.000.641:5.4E-
4,BrhBact3:0.035)0.627.6:0.00366)0.979.66:0.01997,((UltB1987:0.00369,UltB
2198:0.00371)0.914.105:0.01978,(PrsSpeci:0.01464,UltB1994:0.03665)0.835.7
1:0.02386)0.944.70:0.02483)0.770.82:0.00355)0.869.73:5.5E-
4)0.951.78:0.01824,(BeaPro22:0.03218,(UltB1984:0.02762,(UltB2026:0.01005,
(UltB2025:0.02575,(UltB2022:0.02068,((UltrB107:0.01296,(UltB2021:5.4E-
4,Ult32111:0.00369)0.893.97:0.01006)0.851.85:0.00818,((UltrB108:0.04424,(
UltB2023:0.00273,UltB2024:0.00872)0.774.82:0.00422)0.820.49:0.00682,(UltB
2018:0.02342,(UltB2019:0.00353,(UltB2020:0.00745,UltrB106:5.3E-
4)0.838.77:0.0078)0.777.84:0.00415)0.935.86:0.01593)0.796.55:0.00699)0.95
7.69:0.01576)0.572.9:5.4E-
4)0.900.79:0.01551)0.909.81:0.01772)0.483.15:0.0069)0.851.86:0.0101)0.799
.72:0.00823,(((((((UltB2012:0.00741,UltB2003:0.05481)1.000.640:5.5E-
4,((UltB2000:0.0,UltB2005:0.0,UltB2008:0.0,UltB2009:0.0,UltB2010:0.0,UltB
2013:0.0,UltB2014:0.0,UltB2015:0.0):5.5E-
4,(UltB2001:0.00369,UltB2004:0.0029)1.000.641:7.8E-4)0.497.3:5.4E-
4)0.236.5:5.5E-4,UltB2002:5.5E-4)0.211.9:5.5E-4,(UltB2011:5.5E-
4,(UltB2016:0.00742,UltB2017:0.00739)0.727.26:5.5E-4)0.225.5:5.5E-
4)1.000.642:5.4E-
4,(UltB1999:0.00376,UltB2006:0.0228)0.901.93:0.00324)0.880.100:0.02445,((
(UltB1998:0.0,Ult17543:0.0):5.5E-
4,UltB1997:0.00369)0.846.96:0.01423,(UltB1995:0.00364,UltB1996:0.00376)0.
999.128:0.06674)0.844.92:0.02263)0.953.69:0.03259,((UltB1986:0.01942,UltB
1993:0.01107)0.749.74:0.00367,(UltB1928:0.06134,(SutSpeci:0.00746,((UltB1
990:0.01578,UltB1989:0.02198)0.424.16:0.00729,(UltB1991:0.00784,UltB1992:
0.06803)1.000.643:5.4E-4)0.908.104:0.00283)0.993.63:5.4E-
4)0.498.7:0.0145)0.820.50:0.01429)0.665.18:0.00307)0.899.96:0.01099)0.774
.83:0.00364,(((UltrB132:0.00379,UltB2253:0.02575)0.887.96:0.01237,(((((Ul
tB2105:0.02734,((UltB2250:0.0071,(UltB1540:5.4E-
4,UltLaut2:0.01111)0.913.99:0.01161)0.947.70:0.01521,(UltrSo13:0.01891,(U
ltB2104:5.5E-
4,UltB2103:0.01145)0.868.70:0.00799)0.739.38:0.00408)0.770.83:0.00359)0.7
44.48:0.00369,UltB2252:0.00793)0.000.642:5.4E-
4,UltB2081:0.01954)0.871.94:0.00859,(UltB2098:0.01923,(UltOxal6:0.00369,U
ltrB129:5.5E-
4)0.993.64:0.03849)0.868.71:0.00979)0.884.101:0.01361,(UltB2545:0.01963,(

UltB2255:0.016,(UltB2028:0.00746,(UltB2027:5.5E-
4,UltB2029:0.0037)0.697.9:5.5E-
4)0.922.134:0.01535)0.632.8:0.00792)0.973.56:5.4E-
4)0.777.85:0.00598)0.743.55:0.01023,UltB2210:0.04371)0.860.66:5.4E-
4)0.897.90:0.00781)0.778.74:0.00385,(UltB2215:5.4E-
4,PcmLemoi:0.01519)0.925.107:0.00743)0.433.15:5.4E-4)0.229.8:5.5E-
4,UltB2062:0.03913)0.973.57:5.4E-
4)0.431.14:0.00386,(UltB2221:0.01126,(((((UltRals7:5.5E-
4,(UltB2227:0.02319,((UltB2206:0.0,UltRhoda:0.0):5.5E-
4,UltB2207:0.00369)0.950.84:5.5E-4)0.832.63:0.00369)0.504.9:5.5E-
4,((((((UltB2203:0.0,UltB2236:0.0):5.5E-
4,((UltRals4:0.00305,UltRals5:5.5E-4)0.951.79:6.3E-
4,(RlsMann2:0.0,RlsManni:0.0,RlsSola2:0.0,RlsSolan:0.0,RlsSola3:0.0):0.00
744)0.661.12:5.4E-4)0.000.643:5.5E-4,UltRals3:5.5E-4)0.000.644:5.5E-
4,UltB2232:0.00369)0.000.645:5.5E-
4,(UltB2233:0.01875,UltB2234:0.00741)0.632.9:5.5E-4)0.933.94:5.5E-
4,(UltB2235:0.04268,(UltB2230:0.00368,((UltB2229:5.5E-
4,(UltrB112:0.0,RlsSpec2:0.0,RlsSpeci:0.0,BeaPro28:0.0,UltRals2:0.0,UltBu
r26:0.0):5.5E-4)0.758.70:5.5E-
4,(UltRalst:0.00368,UltB2231:0.0306)0.426.18:5.5E-4)0.795.67:5.4E-
4)0.919.117:0.00738)1.000.644:5.5E-4)0.916.102:0.00367)0.914.106:5.4E-
4,(BrkSpe55:0.00744,UltB2254:0.02804)0.919.118:0.01158)0.826.57:0.0074,((
(((UltrB125:5.4E-4,(((UltB2226:5.5E-4,(ClmFung2:0.00369,ClmArena:5.5E-
4)0.860.67:0.0037)0.929.82:0.00754,(ClmFungi:0.00369,(ClmSpec2:0.0,ClmSpe
ci:0.0):5.5E-4)0.612.15:5.5E-
4)0.771.88:0.00369,UltB2178:0.00747)0.789.63:0.0037)0.797.57:0.00369,BeaP
ro27:0.0075)0.766.65:0.0039,((UltB2202:0.00369,UltB2220:5.5E-
4)0.900.80:5.4E-4,(BeaPro26:0.00743,(UltB2167:5.5E-
4,UltOxal4:0.00369)0.768.63:5.5E-
4)0.919.119:0.01111)0.484.14:0.00753)0.781.65:0.00352,UltrSo12:0.00745)0.
778.75:5.4E-4,((UltB2327:0.00367,(UltB2218:5.5E-4,((OxlBact4:5.5E-
4,(UltrB130:0.00753,UltB2214:0.00745)0.658.12:5.5E-
4)0.831.53:0.00368,UltB2222:0.02653)0.859.85:5.5E-4)0.898.109:5.4E-
4)0.914.107:5.5E-4,(((((OxaForm4:0.00743,(OxaForm3:5.5E-
4,((UltB2224:0.0,UltOxal5:0.0):5.5E-
4,(UltB2179:0.00745,UltB2225:0.0037)0.289.4:5.5E-
4)0.936.88:0.0037)0.034.7:5.5E-
4)0.909.82:0.00742,(UltB2237:0.00741,UltB2208:5.3E-
4)0.495.9:0.00369)0.944.71:5.4E-4,UltPro23:5.4E-4)1.000.645:5.4E-
4,(HerAutot:0.01537,(UltHerb2:0.00368,((HerSerop:0.0,HerSero2:0.0):0.0037
,(UltrB128:0.0,UltHerb3:0.0):5.5E-4)0.000.646:5.0E-4)0.260.8:5.4E-
4)0.859.86:0.0036)0.000.647:0.00369,((UltB1539:5.5E-4,UltB2181:5.5E-
4)0.000.648:5.4E-4,UltOxal7:0.02302)1.000.646:5.4E-
4)0.862.93:0.00359)0.844.93:0.00365)1.000.647:5.4E-
4)0.771.89:0.00352,(UltB2217:5.4E-4,(HrmFonti:5.5E-
4,UltB2182:0.00743)0.859.87:0.00361)0.168.9:0.0037)1.000.648:5.4E-
4)0.872.93:5.4E-
4)0.888.104:0.00334,((OxcFlavu:0.0,OxcFaeci:0.0):0.0037,HerSpec4:5.5E-
4)1.000.649:5.3E-
4)0.357.10:0.00808,UltB2087:0.02287)0.834.60:0.0162)0.777.86:0.00349,((Ul
tRho21:0.00764,(UltRho19:0.00371,(UltRho20:0.00372,UltrB179:0.00369)0.698
.19:5.5E-
4)0.932.84:0.01146)0.829.68:0.00741,(((UltB2424:0.0,UltB2425:0.0):5.5E-
4,UltrB162:0.00741)0.969.58:5.5E-

4,(UltB2423:0.01848,(UltB2433:0.02269,((UltB2201:0.0,UltB2420:0.0):5.5E-
4,UltB2421:5.5E-4)0.983.51:5.3E-
4)0.913.100:0.00404)0.931.93:0.00406)0.870.71:0.00754)0.918.93:0.01144)0.
933.95:0.01112,(UltB2533:5.4E-
4,(((((UltrB156:0.01954,((((UltB2430:0.00375,(UltrB135:0.00369,UltB2267:0
.0037)0.963.68:0.0152)0.952.69:0.01532,UltB2547:0.00378)0.915.91:0.01209,
UltB2429:0.00696)0.915.92:0.0123,GllFerru:0.01937)0.890.96:0.01185)0.755.
58:0.01514,(((UltrB153:5.5E-
4,((UltB2476:0.05595,(UltB2389:0.00369,UltB2392:5.5E-
4)0.762.82:0.00365)0.905.94:0.01136,(UltB2400:0.00745,((((UltB2399:0.0038
9,(UltB2395:0.01101,(UltB2401:5.4E-4,(UltB2397:5.5E-4,UltB2398:5.5E-
4)0.925.108:0.00368)0.898.110:5.4E-
4)0.507.7:0.00742)0.791.63:0.0035,((UltB2396:5.5E-
4,UltB2438:0.00369)0.851.87:0.00369,(MtpSpeci:0.00369,UltMeth3:5.5E-
4)0.920.105:0.00746)0.765.60:5.4E-4)0.478.10:5.4E-
4,UltrSo21:0.03501)0.402.9:0.00368,(((((UltB2409:0.00371,((MtpLeis2:0.0,U
ltB2405:0.0,UltB2406:0.0,UltB2407:0.0,UltB2408:0.0):5.5E-
4,MtpLeisi:0.0037)0.777.87:0.0037)0.793.69:0.00369,MttMobil:5.5E-
4)0.918.94:0.0075,(UltrB154:0.03113,(UltB2402:0.00735,BeaPro31:0.0199)0.4
06.9:0.0114)0.822.52:5.5E-4)0.998.103:5.3E-
4,(UltB2403:0.0,UltB2404:0.0):5.5E-4)0.399.11:5.5E-
4,IroBac11:0.00368)0.748.57:5.5E-4)0.923.126:0.00735)0.291.11:5.4E-
4)0.951.80:0.01851)0.893.98:0.00766,(((MtvrMays:0.0027,(MtbFlage:0.00466,
MtbPrate:0.02188)0.438.11:0.00754)0.757.51:0.00468,UltrB155:0.01519)0.882
.96:0.0076,(UltrB160:0.0103,(UltMeth4:0.00748,UltPro28:0.00371)0.878.87:0
.0104)0.765.61:0.00573)0.852.82:0.0073)0.826.58:0.00697,UltB1558:0.02014)
0.783.84:0.01457)0.844.94:5.4E-
4,UltrB159:0.00759)0.789.64:0.00368,((UltB2416:0.0145,(UltrB140:0.01838,(
((DerGummo:0.01425,(UltB2080:0.0114,UltCom29:0.01529)0.858.85:0.00993)0.8
57.91:0.00992,NtpSpeci:5.4E-
4)0.829.69:0.00369,((UltB2317:0.00754,(NtpSpec2:5.5E-
4,NtpSpec3:0.01121)0.812.56:0.00369)0.785.74:0.00371,(NtpMulti:5.5E-
4,NtpSpec4:5.5E-4)0.873.92:5.5E-4)0.517.4:5.3E-
4)0.848.75:0.00805)0.639.15:0.00765)0.840.79:0.00831,((UltrBe18:0.01127,(
(UltrB157:0.0312,(UltB2410:5.5E-
4,UltB2069:0.04286)0.804.54:0.00366)0.584.10:5.4E-
4,UltrB158:0.00369)0.795.68:0.00376)0.871.95:0.00765,((((((UltB1648:5.5E-
4,UltOrg14:0.01498)1.000.650:5.4E-4,((UltB2393:0.0,UltB2390:0.0):5.5E-
4,UltB2391:0.00369)0.805.57:0.00366)0.447.7:0.0037,(UltrB152:0.0,UltB2386
:0.0,UltB2387:0.0):0.0113)0.977.59:0.01974,((UltB1655:0.0,UltB2394:0.0,Ul
tB2388:0.0,UltB4483:0.0):0.00318,UltB4482:0.01555)0.870.72:0.00762)0.380.
10:0.00368,((((UdbPigru:5.4E-4,(UltB2383:0.01562,((UdbSpeci:5.4E-
4,(UltB2172:5.5E-4,UltB2173:5.5E-
4)0.835.72:0.00369)0.930.76:0.00747,((UltB2170:0.0,BeaPro25:0.0,UltB2174:
0.0,UltB2175:0.0):5.5E-
4,(UltB2168:0.00369,UltB2171:0.00369)0.650.13:5.5E-4)0.916.103:5.5E-
4)0.781.66:0.00336)0.891.81:0.01176)0.266.8:0.01125,(UltB2169:0.00369,Ult
rB123:5.5E-4)0.996.76:5.4E-
4)0.892.87:0.00749,(((UltrB127:0.00744,(HerSpec2:5.5E-
4,((HerHutti:0.0,HerSpeci:0.0,HerRubri:0.0):5.5E-
4,UltHerba:0.00369)0.423.11:5.5E-4)0.836.62:0.00368)0.882.97:5.5E-
4,HerSpec3:5.5E-4)0.489.10:5.4E-
4,((((((UltB2177:0.0,UltB2185:0.0,HrmArsen:0.0,HrmArse2:0.0,HrmGlaci:0.0)
:5.5E-4,((UltOrg12:0.00783,UltB2186:0.00743)0.418.9:5.5E-

4,UltB2183:0.00369)0.331.8:5.5E-4)0.194.7:5.5E-
4,UltrB126:0.00369)1.000.651:5.4E-
4,UltB2184:0.05089)0.907.87:0.0077,((UltB2228:0.01568,UltB2415:0.0192)0.7
50.47:0.00365,UltB2176:0.02314)0.879.98:0.00781)0.996.77:5.4E-
4,UltOxal3:0.01542)0.819.56:0.00387)0.941.77:0.01187)0.751.45:0.00391,Ult
Derxi:0.01122)0.788.68:0.00432)0.772.74:0.00339,((UltB2411:0.01565,(UltB2
442:0.00668,((VogIndig:0.01933,(AqsSerpe:5.5E-
4,UltB2471:0.01175)0.962.85:0.01529)0.750.48:0.00426,((UltB2276:0.0075,Ul
tB2275:5.5E-
4)0.917.90:0.01436,(UltB2277:0.0144,ThbAquae:0.0085)0.728.35:0.01137)0.83
0.63:0.00777)0.851.88:0.0075)0.889.92:5.5E-
4)0.870.73:0.00748,((((UltB2412:0.01418,ThbSpeci:0.03097)0.937.89:0.026,U
ltB2418:0.0281)0.118.8:0.00851,((ThbPlumb:5.5E-
4,UltB2414:0.00369)0.990.74:0.03159,(((ChmViola:0.00385,((UltB2465:0.0669
3,UltB2466:5.4E-4)0.946.85:0.01553,((((GulMobil:0.01127,(AqtMagnu:5.5E-
4,(AqtDenit:5.5E-
4,AqtSpeci:0.00371)0.862.94:0.0037)0.792.81:0.00369)0.785.75:0.00415,ChmS
pec2:0.00328)0.784.79:0.00747,ChmSpeci:0.0108)0.875.99:5.4E-
4,PsgSubfl:0.0039)0.457.14:0.0037)0.584.11:5.4E-
4)0.602.9:0.00763,UltB2467:0.00703)0.877.109:5.5E-
4,(PalYongn:0.00751,UltB2468:0.00372)0.793.70:0.00396)0.894.91:0.0122)0.9
08.105:0.01671)0.687.15:0.00954,((((((UltB2319:0.00735,UltXanth:0.01935)0
.998.104:0.03717,(UltB2102:0.02285,(((UltB2325:0.04272,(((UltrB139:0.0089
6,(UltrSo15:0.02649,(UltCom13:0.01801,(UltrSo14:0.04013,UltB2286:0.0116)0
.118.9:0.00388)0.924.91:0.01563)0.920.106:0.01743)0.892.88:0.01773,(UltPr
o21:0.01371,(UltrSo16:0.00753,UltB2298:0.00373)0.847.104:0.01097)0.421.9:
0.00614)0.932.85:0.02518,(UltOrg13:0.04202,((UltB2295:0.01122,((((UltB228
7:0.00369,UltBur27:5.5E-
4)0.916.104:0.01166,(UltB2288:0.01137,UltB2294:0.01937)0.911.112:0.0113)0
.879.99:0.00768,((((((UltSlud5:0.00755,UltrB137:0.01524)0.778.76:0.00363,
UltrB136:0.0037)0.040.3:5.4E-
4,UltrB138:0.01125)0.760.67:0.00366,((UltB2282:5.5E-
4,(UltB2285:0.01119,(((UltB2283:0.01517,UltB2284:5.5E-
4)0.899.97:0.0075,UltB2279:0.01516)0.773.75:0.00367,UltB2293:0.01122)0.61
6.11:5.3E-
4)0.931.94:0.01125)0.945.75:0.01124,((UltB1841:0.00359,UltB2296:0.00764)0
.781.67:0.00374,(UltB2280:5.4E-
4,UltB2281:0.01501)0.893.99:0.00762)0.880.101:0.00758)0.781.68:0.00395)0.
902.100:0.01071,(UltB2292:0.00687,(UltB2291:0.00332,(UltB2289:0.00748,Ult
B2290:0.0037)0.896.85:0.00823)0.760.68:0.0041)0.840.80:0.00774)0.829.70:5
.5E-4,UltB2297:0.01146)0.868.72:0.00737)0.931.95:0.01163,Ult13852:5.3E-
4)0.797.58:0.00374)0.808.63:0.00388,(UltPro24:0.03422,(UltB2299:0.00703,(
PshgggYy:0.0,UltB2300:0.0):0.01578)0.055.2:0.00742)0.756.72:0.00519)0.637
.8:0.00256)0.963.69:0.03472)0.912.120:0.0236)0.807.50:0.01187,((UltPro25:
5.4E-
4,((((UltNitro:0.01361,UltB2333:0.02277)0.548.10:0.01406,(UltB2334:0.0073
6,UltB2328:0.00381)0.871.96:0.0132)0.004.7:0.00178,((UltB2336:0.00375,Ult
B2335:0.01918)0.755.59:0.00355,((UltrB145:0.00937,(((UltB2329:0.00383,((U
ltB2331:0.0,UltrB144:0.0):0.01844,(UltNitr3:0.0085,(UltB2337:0.01539,UltB
2332:0.00744)0.806.49:0.00669)0.761.63:0.0078)0.766.66:0.00469)0.838.78:0
.01145,((UltMeth2:0.0,UltB2339:0.0):5.4E-
4,UltrSo19:0.01538)0.905.95:5.4E-4)0.799.73:0.00564,(UltB2330:5.5E-
4,UltB2338:5.5E-
4)0.874.111:0.00936)0.934.83:0.01968)0.958.90:0.01761,UltNitr2:5.5E-

4)0.955.66:0.01537)0.065.11:0.01206)0.964.76:0.03676,(UltB2324:5.5E-
4,Ult15744:0.00367)1.000.652:0.07208)0.738.34:0.0038)0.788.69:0.00695,(Ul
tB2310:0.07557,UltrB143:5.4E-4)0.000.649:5.4E-
4)0.917.91:0.01967)0.901.94:0.02094,(UltB2323:0.01116,(UltB1762:0.04065,U
ltrB142:0.02427)0.000.650:0.00703)0.874.112:0.01612)0.617.8:0.00897)0.844
.95:0.01178)0.755.60:0.00378,((((NtsEutro:0.00752,NtsEurop:0.00373)0.758.
71:0.00381,(NtsHalop:0.0024,NrsMobil:0.04028)0.503.6:0.01407)0.934.84:0.0
1556,(UltB2314:0.03172,(NtsCommu:0.01154,NtsNitro:0.0074)0.972.71:0.02393
)0.795.69:0.00799)0.850.95:0.00792,((NtsUreae:0.01936,UltB2316:0.01512)0.
121.6:0.00737,((((UltB2312:0.01935,(NtsSpeci:5.5E-
4,UltB2315:0.01121)0.000.651:0.00365)0.743.56:0.00427,((UltB2311:0.01103,
NtsOligo:0.00401)0.902.101:0.01585,Otu00453:0.04498)0.912.121:0.01642)0.6
04.11:0.0033,UltB2313:0.00759)0.852.83:0.00749,(NtsAestu:0.0,Udntfde3:0.0
):0.00378)0.283.5:5.4E-
4)0.821.53:0.0108)0.990.75:0.02756)0.764.80:0.00709,(UltB2318:0.04001,((U
ltB2305:5.3E-
4,(UltB2308:0.02431,UltrSo18:0.02824)0.915.93:0.01849)0.505.4:0.00362,Ult
B2306:5.4E-
4)0.929.83:0.01764)0.781.69:0.00666)0.959.80:0.02068,(DenBact2:0.0582,(Ul
tB2464:5.4E-4,((LrbHong2:5.5E-4,LrbHongk:5.5E-
4)0.909.83:0.01808,(McrAerod:0.01079,(McrAero2:0.0,McrAero3:0.0):0.00517)
0.855.86:0.01307)0.864.88:0.02338)0.875.100:0.00719)0.839.74:0.00828)0.63
9.16:0.00762,((((((((ChbTaina:0.01551,((IodFluvi:0.0074,IodSpeci:5.5E-
4)1.000.653:0.0531,(BeaPro33:5.4E-
4,ChnAquat:0.02278)0.830.64:0.02317)0.961.72:5.5E-
4)0.893.100:0.01654,((SilTerra:5.4E-4,ChtShina:0.00747)0.186.5:5.3E-
4,(NsrBacte:0.02215,(AndChiti:0.00909,(ForCitri:0.02983,(UltB2475:0.00766
,((DeeRivul:0.0,UltB2473:0.0,UltB2474:0.0):5.5E-
4,BeaPro34:0.01904)0.760.69:0.00349)0.998.105:0.04992)0.841.73:0.01382)0.
861.94:0.01867)0.742.40:0.00456)0.898.111:0.01913)0.971.59:0.03755,LeeOry
za:0.02555)0.940.94:0.03316,(UltB2472:0.0444,((UltB2274:5.7E-
4,(DenBact4:0.00368,(DenBact3:0.00368,DenBacte:5.5E-4)0.509.8:5.5E-
4)0.766.67:0.0145)0.819.57:0.02172,((UltB2256:5.5E-4,(((ThbThiop:5.5E-
4,UltThiob:0.00741)0.827.60:0.00365,(((ThbDenit:0.0,UltB2264:0.0):5.5E-
4,(UltB2258:0.0076,UltB2259:0.0037)0.568.10:5.5E-4)0.374.11:5.5E-
4,(UltB2262:5.5E-
4,(UltrB133:0.01136,UltB2263:0.00356)0.895.111:0.00759)0.918.95:0.00744)1
.000.654:5.4E-4)0.768.64:0.00757,(ThbThio2:0.01507,ThbDeni2:5.4E-
4)0.955.67:0.01131)0.644.11:5.3E-
4)0.988.71:0.00194,UltB2257:0.00188)0.961.73:0.02566)0.872.94:0.01218)0.8
32.64:0.01252)0.513.6:0.01025,((UltB2463:0.00369,VogSpeci:5.5E-
4)0.974.49:0.01931,(UltrB169:0.00744,VogPerlu:5.5E-4)0.971.60:5.5E-
4)0.881.104:0.01249)0.926.103:0.01921,SpiWinog:0.03105)0.870.74:0.01292,(
(NssSpec7:5.3E-
4,((AlyCrass:0.0,AlyFilif:0.0):0.00376,(((((KnlKinga:0.0109,UltB2453:0.02
748)0.966.90:5.1E-
4,((SmnMuell:0.0037,((NssSpec4:0.00372,(BacteNew:0.00371,UrubSuis:0.00753
)0.785.76:0.00377)0.764.81:0.00371,(((UltB2452:0.00373,(ConStee4:0.00745,
(ConStee2:0.00369,(ConSteed:0.0,ConStee3:0.0):5.5E-4)0.740.50:5.5E-
4)0.894.92:0.00751)0.901.95:0.00421,((UltB2200:0.00749,NssSpec3:0.00745)0
.619.6:5.4E-
4,((UltB2454:0.01128,(NssSpec2:0.0,KnlPotus:0.0):0.00742)0.250.9:5.4E-
4,UltB2455:0.00738)0.830.65:0.00366)0.951.81:0.01429)0.899.98:0.00414,((K
nlDenit:5.5E-4,(BrgDenit:0.00745,UltB2451:0.0037)0.789.65:5.4E-

4)1.000.655:5.5E-4,(((NssWeave:0.0,NssAnima:0.0,NssSpec5:0.0):5.4E-
4,(NssDenti:0.00369,((((UltB2449:5.5E-4,(UltB2446:0.01112,(NssMeni3:5.5E-
4,(UltNeis3:5.5E-4,(UltNeis2:5.5E-4,UltNeiss:5.5E-4)0.872.95:5.5E-
4)0.956.78:0.0074)0.624.14:5.2E-
4)0.857.92:0.00366)0.868.73:0.00366,(((UltB2450:0.0,NssMucos:0.0):5.5E-
4,BacteNe2:0.0151)0.726.22:5.5E-4,UltB2447:5.5E-4)0.876.80:5.5E-
4)0.712.19:5.4E-
4,((NssMenin:0.0,NssMeni2:0.0):0.00752,NssLacta:0.00751)0.763.83:0.0037)0
.878.88:0.00744,((NssBacil:5.4E-
4,NssSpeci:0.01127)0.951.82:0.01134,UltNeis4:0.00752)0.768.65:0.00368)0.8
74.113:0.00746)0.885.82:0.00743)0.821.54:0.00365,NssCanis:5.4E-
4)0.857.93:0.00366)0.888.105:0.00751)0.883.102:0.00753)0.902.102:0.00736)
0.764.82:5.4E-
4,((ConKuhn2:0.00375,UltB2448:0.00751)0.870.75:0.00747,ConKuhni:0.00376)0
.873.93:0.00753)0.852.84:5.4E-
4)0.975.62:0.01752,(((StnAceti:0.0,Otu00307:0.0):0.02274,UltrB168:0.02912
)0.986.66:0.03301,((((UltB2462:5.4E-
4,VitSterc:0.00377)0.978.55:0.01911,((UltB2461:0.01875,(UltB2459:0.03982,
UltB2460:0.00313)0.977.60:0.02878)0.990.76:0.03255,((UltrB167:0.0,BeaPro3
2:0.0,UltSimon:0.0):0.00368,(UltrB165:5.5E-
4,UltrB166:0.00369)0.925.109:0.00763)0.775.75:0.00335)0.872.96:0.0081)0.7
73.76:0.00357,UltB2457:5.4E-4)0.910.93:0.01206)0.864.89:5.4E-
4)0.883.103:0.00745,(KnlSpeci:0.0,UltB2456:0.0):0.00852)0.640.12:5.4E-
4,(NssSpec6:5.4E-4,((NssZoode:0.0,NssAnim2:0.0):5.5E-
4,UltB2458:0.00741)0.934.85:0.00744)0.983.52:0.01794)0.955.68:0.01505)0.9
73.58:0.02668)0.808.64:0.01324,(UltB2309:0.0152,Ult16633:0.00755)0.964.77
:0.02179)0.780.91:0.01198)0.882.98:0.01093,UltB2419:0.04226)0.668.19:0.00
143,((UltB2417:0.01645,((UltB2251:0.02503,UltB2413:0.02918)0.802.56:0.013
62,UltrB164:0.04357)0.691.13:0.00778)0.131.5:0.01427,(((((UltAlca8:5.5E-
4,(UltBur20:0.00763,UltBur21:0.01191)0.921.107:0.01154)0.919.120:0.01118,
(AcgBacte:5.5E-4,UltAlca9:0.00373)0.937.90:5.3E-
4)0.937.91:0.01139,UltBur19:0.00374)0.785.77:0.00707,((((UltrB172:0.03976
,(UltrB171:0.02459,(UltB2469:5.3E-
4,UltrB170:0.01076)0.772.75:0.01498)0.988.72:0.05244)0.979.67:0.05103,(Ul
tNeis5:0.14252,BacEnri7:0.08258)0.506.10:0.0158)0.592.8:0.01421,AqsPutri:
0.05556)0.399.12:0.00988,UltAlca7:0.01089)0.857.94:0.01323)0.977.61:0.030
36,(UltAlc10:0.01933,(((AclSpec5:0.02358,((((((((UltB2042:0.0,CasSpeci:0.
0):5.5E-4,UltB2043:0.0474)0.827.61:0.00365,CasDenit:5.5E-
4)0.000.652:5.3E-
4,CasSpec2:0.00744)0.939.76:0.01459,(((UltrB111:0.0072,(UltB2045:0.00714,
UltB2067:5.4E-
4)0.476.14:0.00744)0.885.83:0.00778,((((((AdvSpec2:0.0,PigKulla:0.0,UltB2
049:0.0):5.5E-4,PigDaegu:0.00747)1.000.656:5.4E-4,(TetMimig:5.5E-
4,UltB2048:0.04252)0.792.82:0.00357)0.422.13:0.00369,(UltB2051:5.5E-
4,(UltB2050:0.00375,UltB2070:0.00367)0.886.115:0.00749)0.844.96:0.00361)1
.000.657:5.3E-4,(AclSpeci:0.0037,AdvSpeci:5.5E-4)0.675.10:5.4E-
4)0.891.82:0.00742,((BorSpeci:5.5E-4,((BeaPro24:0.0,KerGyior:0.0):5.5E-
4,(UltB2047:0.00746,(((UltB2065:5.5E-
4,UltAlc11:0.02694)0.895.112:0.00745,UltB2066:0.00745)0.784.80:0.00371,((
BorParap:0.0,BorPetri:0.0):5.5E-
4,((((UltB2046:0.00929,(KinBlast:0.00552,KinCrith:0.02918)0.930.77:0.0152
3)0.719.20:0.00188,UltBorde:0.06578)0.683.23:5.5E-
4,AclSpe10:0.00376)0.033.2:5.5E-4,(((BorSpec2:5.5E-
4,UltB2061:0.0037)0.845.89:0.00368,(AclSpe11:5.5E-

4,((((AchXylo4:0.0037,(AchXylo2:0.0116,((AchSpani:0.0,UltB2064:0.0,UltB20
71:0.0):5.5E-
4,(((AclSpec7:0.00247,UltB2053:0.01952)1.000.658:0.00126,(UltB2054:0.0948
3,UltB2059:0.0021)0.133.6:0.00161)0.816.61:5.5E-4,AchPiech:5.5E-
4)0.000.653:5.4E-4)0.960.84:5.5E-4)0.846.97:0.00377)0.179.5:5.5E-
4,UltB2060:0.00369)0.500.12:5.5E-4,UltB2063:5.5E-4)0.532.13:5.5E-
4,(UltB2056:0.0,UltB2055:0.0,AchXylo3:0.0,UltB2058:0.0,AclSpec8:0.0,UltB2
057:0.0,AclSpec9:0.0,UltAchro:0.0,AchSpec2:0.0,AchXylo5:0.0,BorTrema:0.0)
:5.5E-4)0.531.6:5.5E-4)0.496.5:5.3E-4)0.773.77:5.5E-
4,UltrB110:0.01517)0.918.96:0.00757)0.599.8:5.4E-4)0.488.12:5.4E-
4)0.916.105:0.00844)0.043.9:5.5E-4)0.962.86:0.00842)0.822.53:5.5E-
4,UltB2052:0.00368)0.968.71:0.01226)0.000.654:5.5E-
4)0.767.64:0.00452,(BrlOedip:0.01547,UltB2032:0.02371)0.935.87:0.01907)0.
404.12:0.00817)0.766.68:0.00445,((AcgBact3:5.4E-
4,(PusSpeci:0.00746,AcgBact2:5.4E-4)0.919.121:0.00753)0.891.83:5.5E-
4,(UltrB109:0.01528,(PlsEurop:0.0151,UltB2041:5.4E-
4)0.894.93:0.00751)0.869.74:0.00744)0.591.12:0.00744)0.986.67:5.4E-
4,((((AclFaec4:5.5E-4,((AclFaec3:0.0,UltB2039:0.0,UltB2040:0.0):5.5E-
4,(AclSpec3:0.00369,UltB2038:0.00369)0.659.14:5.5E-4)0.753.73:5.5E-
4)0.423.12:5.5E-4,RhbSpha2:5.5E-
4)0.985.66:0.01492,((AclFaeca:0.01139,AclFaec2:0.00376)0.989.64:0.02334,(
AclSpec4:0.00373,AcgBact4:0.01131)0.867.93:0.00749)0.930.78:5.4E-
4)0.827.62:0.00368,(((TayEqui2:0.0,TayEquig:0.0):0.01123,TayAsini:5.5E-
4)0.943.85:0.01123,(UltB2033:0.01157,(AclSpec2:5.4E-
4,UltB2035:0.0033)0.956.79:0.01963)0.913.101:0.01513)0.725.29:5.4E-
4)0.877.110:0.00368)0.954.76:5.5E-
4,(UltB2034:0.01944,AclSpec6:0.00369)0.605.14:0.00762)0.882.99:0.00845)0.
838.79:0.00788,((((OliUreol:0.02226,OliUreth:0.00856)0.979.68:0.04183,Ult
B2044:0.03955)0.933.96:0.02628,(UltB1094:0.0,UltAlcal:0.0):0.00888)0.945.
76:0.01785,(PusNoert:0.00371,UltCompo:0.01516)0.868.74:0.00756)0.732.25:0
.00393)0.865.91:0.01125,(UltBur18:0.02224,(UltB2036:0.00339,UltB2037:5.4E
-
4)0.813.57:0.02098)0.778.77:0.01231)0.831.54:0.00925)0.873.94:0.00854)0.9
48.93:0.02442)0.959.81:5.5E-
4)0.760.70:0.00442)0.915.94:0.01754)0.563.8:0.00391)0.997.91:5.4E-
4)0.890.97:0.00752)0.775.76:0.00383)0.753.74:0.00394)0.938.58:0.01176,(Pr
pDicar:5.5E-
4,(UltB2531:0.00378,(UltB2532:0.00359,PrpPelop:0.01526)0.869.75:0.00762)0
.868.75:0.0114)0.721.19:0.01111)0.956.80:5.4E-4)0.638.9:5.4E-
4)0.898.112:0.00774)1.000.659:0.00122)0.948.94:0.00175)0.933.97:0.01211)0
.867.94:0.00381)0.362.10:5.2E-4,(UltB2487:0.00745,(((BrkNodo2:5.5E-
4,UltB2077:0.00369)0.973.59:0.01133,((UltrB161:0.00735,BacEnri6:0.00376)0
.674.17:0.00739,UltB2422:0.0111)0.825.62:5.4E-
4)0.819.58:0.00369,((UltB2089:0.00369,(PndPulmo:5.5E-
4,((PndNorim:0.0,PndSpeci:0.0,UltrB116:0.0,UltPando:0.0):5.5E-
4,PndSpec2:0.00369)0.838.80:0.00369)0.913.102:0.00744)0.730.32:5.4E-
4,BrkSpe54:5.5E-4)0.834.61:5.5E-
4)0.901.96:0.00745)0.783.85:0.00372)0.959.82:5.5E-
4,((((((UltB2368:0.01501,UltB2384:5.5E-
4)0.406.10:0.0075,((((DenBact5:0.0,DenBact6:0.0):5.5E-
4,UltB2369:0.0037)0.939.77:0.00746,UltRhod7:0.00746)0.754.60:5.4E-
4,UltB2373:5.3E-4)0.801.68:0.00368)1.000.660:5.4E-
4,(UltrB150:0.00361,UltrB151:0.00383)0.838.81:0.0075)0.983.53:0.00148,(Ul
tB2372:0.02323,UltB2366:5.4E-

4)0.968.72:0.01484)0.931.96:0.00229,(UltB2382:5.4E-
4,UltB2441:0.04434)0.424.17:0.00728)0.903.87:0.00757,(((UltB2381:0.00381,
((UltPro27:0.0037,(DntOestr:0.01895,(UltB2370:0.00746,UltrB149:5.4E-
4)0.907.88:0.00749)0.936.89:5.4E-
4)0.866.87:0.0037,((((UltB2376:0.00593,(UltB2374:0.00378,(UltB2375:0.0234
,UltRhod2:5.5E-
4)0.785.78:0.00368)0.770.84:0.00592)0.993.65:0.03176,(UltB2378:5.5E-
4,((UltRhod3:5.4E-4,UltB2379:0.00719)0.907.89:0.0109,(UltRhod4:5.5E-
4,UltB2380:0.00368)0.930.79:0.002)0.919.122:0.00197)0.836.63:0.00967)0.84
4.97:0.00926,(UltB2371:5.4E-
4,(UltB2377:0.01134,UltRhod5:0.00749)0.790.70:0.00372)0.878.89:0.01233)0.
248.8:5.5E-4,(UltB2367:5.4E-
4,UltB2385:0.00744)0.971.61:0.01691)0.854.83:0.00509)0.143.7:5.3E-
4)0.754.61:5.5E-
4,UltB2358:0.03525)0.818.41:0.00367,UltRhod6:0.00742)0.948.95:5.5E-
4)0.936.90:0.0151)0.734.46:0.00392,((UltB2491:0.04459,(((UltB2498:0.0,Zgl
Oryza:0.0):5.5E-4,(((UltB2268:5.5E-4,UltB2497:5.5E-4)1.000.661:5.5E-
4,((ZglResin:0.0,ZglSpeci:0.0,UltB2493:0.0):5.5E-4,UltB2495:5.5E-
4)0.936.91:0.01116)0.246.12:0.01134,(((ThrSpec3:5.5E-
4,UltRho18:0.00369)0.963.70:0.01134,(UltRho16:0.0074,((UltB1541:0.0,UltSl
ud6:0.0,UltRho14:0.0,UltB2499:0.0):5.5E-4,UltB2494:5.5E-4)0.518.11:5.5E-
4)0.524.9:5.4E-
4)0.867.95:0.00388,(UltB2492:0.0,UltB2496:0.0,UltRho17:0.0):5.4E-
4)0.908.106:0.0078)0.972.72:5.4E-4)0.291.12:5.4E-
4,UltRho15:0.00369)0.800.59:0.00774)0.677.12:0.0135,(((UltB2356:0.03071,(
UltrB148:0.01902,(((ThcSubte:0.02275,(UltB2030:0.0036,UltB2031:0.02305)0.
975.63:0.02712)0.841.74:0.00972,(UltB2354:0.01101,UltB2355:0.01604)0.960.
85:0.02054)0.729.23:0.00352,UltRhodo:0.01961)0.871.97:0.01181)0.923.127:5
.4E-
4)0.793.71:0.02143,UltB2357:0.01214)0.968.73:0.02576,((UltB2321:0.01907,(
((((UltB2349:0.00761,((UltB2348:5.5E-
4,UltB2350:0.0113)0.873.95:0.00756,(UltB2342:0.01537,((UltB2343:0.0,UltB2
344:0.0):5.4E-4,(((UltrB147:5.4E-4,(UltrB146:0.01122,UltB2341:5.5E-
4)0.928.72:0.00743)0.922.135:0.00246,UltB2347:0.00246)0.922.136:0.00246,(
UltBur29:0.02702,(UltB2345:5.5E-
4,UltB2346:0.00369)1.000.662:0.02725)0.113.9:5.5E-
4)0.832.65:0.00369)0.908.107:0.01134)0.905.96:0.01144)0.861.95:0.00756)0.
828.53:0.00739,UltrRap3:0.0155)0.991.75:0.03246,UltB2322:0.00719)0.929.84
:0.01439,((((ThrSpec2:0.0,UltB2478:0.0,UltRho10:0.0,ThrMeche:0.0,ThrSpeci
:0.0,Ult27545:0.0):5.5E-
4,(UltRho12:0.00369,((UltB2364:0.0,UltB2477:0.0,ThrAmino:0.0):5.5E-
4,((ThrArom2:0.0,ThrArom3:0.0):0.00369,ThrSelen:0.01116)0.759.80:5.4E-
4)0.847.105:0.00369)0.901.97:5.5E-4)0.000.655:5.5E-
4,(UltRho11:0.00376,ThrChlor:0.00375)0.695.15:5.5E-4)0.947.71:5.3E-
4,((((UltB2272:0.03143,((HdpHalor:5.5E-4,(HdpTherm:0.00744,HdpHirsc:5.5E-
4)0.840.81:0.00368)0.963.71:0.02754,(PetSucci:5.5E-4,(UltB2269:5.5E-
4,UltB2270:5.5E-
4)0.806.50:0.00696)0.588.11:0.01256)0.926.104:0.02315)0.830.66:0.00827,Az
cCommu:0.00746)0.161.1:5.5E-4,(((Ult31917:0.01132,(UltB2479:5.4E-
4,((BacEnri5:0.00866,UltB2075:0.01028)0.873.96:0.01185,((UltrB113:0.00764
,UltB2072:0.02311)0.865.92:5.4E-
4,(UltB1720:0.01975,(UltB2073:0.00782,UltB2074:0.00343)0.982.56:0.0286)0.
806.51:0.00682)0.975.64:0.02599)0.694.14:0.01693)0.814.46:0.00349)0.773.7
8:0.00369,BeaPro35:0.0037)0.846.98:0.00776,(UltrB134:0.02214,(UltB2248:0.

01884,UltB2265:5.4E-
4)0.888.106:0.02664)0.976.54:0.03302)0.777.88:0.00354)0.925.110:0.00723,(
BeaPro36:0.01564,(UltB2490:5.5E-
4,UliGangw:0.01626)0.985.67:0.0353)0.736.41:0.00341)0.731.26:5.4E-
4)0.951.83:0.01598)0.447.8:0.00359,(UltB2273:5.4E-
4,UltB2278:0.21335)0.985.68:0.03403)0.960.86:0.02012)0.861.96:0.00763,(Ul
tB2320:0.00785,(((UltrSo20:0.04265,(UltB2353:0.03235,UltB2352:0.02265)0.4
00.9:0.01653)0.810.62:0.0122,UltPro70:0.01205)0.798.63:0.00704,UltB2351:0
.00768)0.994.84:0.03285)0.804.55:0.00679)0.893.101:0.01134)0.387.10:5.4E-
4)0.939.78:0.01205)0.790.71:5.4E-
4)0.937.92:0.02111,(Ult16375:0.04991,Ult16377:0.01885)0.977.62:0.03588)0.
604.12:0.00822)0.769.75:0.0067)0.831.55:0.01889,((UltB2199:0.0504,(UltB22
42:0.09795,(Ult11344:0.08205,Ult11737:0.07629)0.922.137:0.04278)0.183.9:0
.01973)0.688.16:0.0095,Ult18710:0.0797)0.678.16:0.02384)0.277.10:0.00302,
(UltB1535:0.01227,UltB2266:0.04158)0.943.86:0.02689)0.949.87:0.02781,Ult1
2388:0.05852)0.755.61:0.01912)0.422.14:5.4E-
4)0.799.74:0.01543,(Ult13902:0.08184,UltGa228:0.10466)0.867.96:0.03061)0.
455.17:5.4E-
4,(UltB4011:0.09635,(UltB4607:0.09645,(UltB4580:0.0579,((UncUnc13:0.02015
,(((UltRum19:0.05483,(UltB4582:0.03426,UltB4581:0.00481)0.941.78:0.03504)
0.987.90:0.05835,((UltB4579:0.01956,(UltB4575:0.04455,(UltB4568:0.01074,(
UltB4572:0.00635,(UltB4571:0.00376,((UltB4574:5.4E-
4,UltB4573:0.00746)0.868.76:0.00718,(UltB4569:0.0,UltB4570:0.0):0.01906)0
.323.6:5.4E-
4)0.887.97:0.0102)0.786.78:0.00652)0.883.104:0.00775)0.138.9:5.3E-
4)0.688.17:5.4E-
4,(UltB4578:0.044,(UltB4576:0.013,UltB4577:0.0501)0.939.79:0.03043)0.870.
76:0.01895)0.975.65:0.03934)0.767.65:0.01346,((UltDes19:0.00825,(Otu00309
:0.04428,Otu00621:0.01122)0.882.100:0.0173)0.901.98:0.01343,(UltB4584:0.0
231,(UltB4583:0.00743,UltDes18:0.00371)0.754.62:0.00425)0.983.54:0.03218)
0.855.87:0.01407)0.492.16:0.01618)0.919.123:0.02369,(UltDes17:0.02171,Unc
Unc12:0.01803)0.447.9:0.00773)0.960.87:0.05141)0.951.84:0.05655)0.783.86:
0.02652)0.708.26:0.01924)0.957.70:0.03472,((((UltBa931:0.06503,(UltBa309:
0.00977,UltBa890:0.03046)0.316.5:0.01542)0.748.58:0.00496,((UltGa114:0.02
862,((((UltAlkan:0.00371,(UltBa902:0.00425,(UltBa887:0.00421,(UltAci38:5.
5E-
4,UltAlka2:0.00369)0.905.97:0.01092)0.768.66:0.00759)0.908.108:0.01112)0.
882.101:0.00766,((UltB1082:0.0,AlsIllin:0.0):5.5E-
4,UltPseu9:0.00369)0.356.11:5.5E-4)1.000.663:5.4E-
4,AlsHongk:0.00354)0.703.23:0.00362,(AntBact4:0.00775,UltB1084:0.00351)0.
787.65:0.00389)0.798.64:0.00677)0.736.42:0.00376,(((UltB1088:0.0112,(UltB
1085:0.00369,UltGa115:0.00369)0.808.65:5.4E-
4)0.747.50:0.00354,(UltGa113:0.01931,(UltB1086:5.4E-
4,UltB1091:0.04787)0.921.108:0.00746)1.000.664:5.5E-
4)0.804.56:0.00964,((PerPisci:5.4E-
4,((UltB1090:0.00369,UltB1089:0.00743)0.885.84:0.00743,(UltB1092:5.5E-
4,UltB1093:0.00368)0.911.113:5.4E-
4)0.441.13:0.00748)0.973.60:0.01948,UltB1087:0.01591)0.740.51:0.00602)0.9
78.56:0.03142)0.967.65:0.02687)0.957.71:0.02925,((((PsdSpe94:0.02019,((Ul
tB1246:0.03606,(UltGa177:0.03032,UltB1247:0.01894)0.780.92:0.0103)0.934.8
6:0.03081,UltB1253:0.04788)0.919.124:0.02781)0.078.9:0.00491,UltB1296:0.2
7683)0.867.97:0.01933,KlbSpeci:0.03045)0.917.92:0.0258,((((GmmPro22:0.009
92,UltGam65:0.03822)0.962.87:0.02694,((UltB1150:0.01454,((UltB1146:0.0037
6,(UltBa842:0.0038,UltGa160:0.00367)0.899.99:0.00754)0.718.22:0.00509,(Ul

tGa159:0.00839,UltGa165:0.02244)0.643.7:0.0187)0.882.102:0.01498)0.634.10
:0.00795,(UltGa163:0.02678,((((UltB1147:5.5E-4,UltB1568:5.5E-
4)0.908.109:5.5E-
4,(((UltB1148:0.00367,(UltB1050:0.01904,(UltB1152:0.00369,UltCell2:0.0232
)0.754.63:5.4E-4)0.835.73:0.01125)1.000.665:5.5E-
4,UltGa162:0.00369)0.484.15:5.5E-4,(UltGa161:0.0037,UltB1151:5.5E-
4)0.866.88:0.0037)0.934.87:0.00749)0.906.104:5.5E-
4,UltB1590:0.06065)0.459.9:0.00715,UltGa164:0.0075)0.926.105:0.0113)0.463
.10:5.4E-
4)0.947.72:0.01772)0.753.75:0.00659,((UdnBac12:0.02464,((((((MaiGamm5:0.
00877,(UltGa133:0.00373,(UltMar22:0.03137,UltGa134:0.01503)0.301.7:5.4E-
4)0.852.85:0.01022)0.681.12:0.01155,((UltMar21:0.0037,((UltGa132:0.01885,
UltB1108:5.5E-4)0.411.9:0.0037,(UltB1109:0.01508,UltB1149:5.3E-
4)0.883.105:0.00741)0.781.70:0.00374)0.444.21:5.4E-
4,(UltGa142:0.01742,UltB1122:0.03059)0.638.10:0.02232)0.933.98:0.01784)0.
838.82:0.00967,(UltB1104:0.0284,((UltB1105:0.0113,(UltOrga7:5.4E-
4,((UltB1099:0.0,UltPro11:0.0,UltB1100:0.0):0.01131,(UltB1103:0.00372,(Ul
tB1101:0.00766,UltGa121:0.0037)0.460.9:5.5E-4)0.290.9:5.5E-
4)0.822.54:0.0038)0.840.82:0.00382)0.391.11:5.4E-
4,(UltGa120:0.00367,UltGa125:0.02354)0.954.77:0.01528)0.148.3:0.00267)0.9
68.74:0.02183)0.832.66:0.00744,(((UltGa119:0.00175,(((((UltB1102:0.00736,
UltB1106:5.5E-4)0.948.96:0.01505,(UltGa126:5.3E-
4,UltGa127:0.01516)0.731.27:0.00356)0.167.7:5.5E-
4,(UltB1107:0.00832,UltB1390:0.02222)0.797.59:0.00811)0.882.103:0.00892,(
(UltGa168:0.01887,UltB1155:0.00761)0.391.12:0.00404,(UltB1153:0.01105,(Ul
tB1154:0.01624,UltGa167:0.01151)0.845.90:0.00831)0.479.13:0.00427)0.937.9
3:0.02295)0.857.95:0.00848,(UltGa122:0.00536,(UltB1096:0.00842,(UltGa124:
5.5E-4,UltGa131:5.5E-
4)0.892.89:0.01277)0.843.67:0.01189)0.295.15:0.00372)0.893.102:0.01073)0.
935.88:0.00217,((UltGa137:5.3E-
4,((MaiGamm3:0.0,MaiGamm4:0.0):0.00757,(UltB1098:0.0076,UltB1097:0.0076)0
.795.70:0.0036)0.767.66:0.00376)0.913.103:0.00748,(UltGa117:0.03113,((Ult
Ga136:5.5E-4,((((UltGa123:0.01588,UltGa130:0.02733)0.487.15:5.4E-
4,((UltOrga8:0.0,UltPro12:0.0,GmmPro30:0.0):0.00369,(UltB1111:0.00744,Hai
Rubra:5.4E-4)0.327.9:5.5E-
4)0.832.67:0.01054)0.378.13:0.0076,(UltGa128:0.00751,UltGa135:0.00751)0.7
78.78:0.00359)0.773.79:0.00382,HaiSalex:0.00371)0.789.66:0.0037)0.902.103
:0.0075,UltGa129:0.00374)0.792.83:0.00365)0.622.7:5.4E-
4)0.785.79:0.00385)0.770.85:0.00702,UltGa118:0.09791)0.724.22:0.00417)0.9
13.104:0.0124,(((UltOrg10:0.0072,(UltB1114:0.00373,((UltOrga9:0.02724,Ult
B1113:5.4E-4)0.847.106:0.00368,UltB1112:5.5E-
4)0.914.108:0.0075)0.863.106:5.4E-
4)0.980.53:0.03933,(UltGa138:0.01107,(UltB1116:0.01542,UltB1115:5.3E-
4)0.625.9:5.4E-
4)0.893.103:0.01892)0.979.69:0.03656,UltB1110:0.01097)0.744.49:0.00467)0.
869.76:0.00816,((AcmMinus:0.05633,(UltGa139:0.06517,(UltGa166:0.03531,Ult
Mar23:0.02882)0.533.9:0.00912)0.839.75:0.01438)0.923.128:0.02391,((UltPse
10:0.0,UltCh475:0.0):0.04417,(UltB1117:0.02788,(UltB1118:0.02108,(UltB111
9:0.00936,UltB1120:0.00586)0.544.8:0.00361)0.732.26:0.00375)0.964.78:0.03
001)0.855.88:0.00784)0.048.4:5.5E-
4)0.851.89:0.00994,((((RhnChiro:0.0,UltBa437:0.0):0.00666,((RhnhSoli:0.01
599,UltBa441:0.03565)0.979.70:0.02752,((((UltGam33:0.0,UltBa436:0.0):5.5E
-4,UltBa435:0.01485)0.913.105:0.0033,(RhnSpeci:5.5E-
4,RhnTangs:0.00372)0.997.92:5.4E-

4)0.877.111:0.01217,((((UltBa440:0.0274,(GmmProt9:0.00684,((UltBa886:0.02
682,UltGam35:5.5E-4)0.823.55:0.00369,(GmmPro10:0.0,RhnSpec2:0.0):5.5E-
4)0.932.86:0.00241)0.058.7:0.002)0.767.67:0.00364,(RhnPacif:5.5E-
4,UltBa439:5.5E-
4)0.779.75:0.00371)0.907.90:0.00344,((MaiBacte:0.0,RhnBalti:0.0):5.5E-
4,(UltGam34:0.0,GmmProt8:0.0):0.01923)1.000.666:5.4E-
4)0.709.17:0.01272,(RhnPerlu:0.00578,(AliFetal:0.00369,((AliAestu:0.0,Ali
Jeotg:0.0):5.5E-4,(AliSpeci:0.0078,UltBa438:0.00741)0.815.61:5.5E-
4)0.622.8:5.4E-
4)0.760.71:0.00551)0.880.102:0.02029)0.733.33:0.00967)0.874.114:0.011)0.7
44.50:0.00437)0.942.84:0.02389,((AlkAmylo:0.0,AlkDelam:0.0):0.00369,AlkCo
lla:5.1E-4)0.979.71:5.5E-
4)0.970.71:0.02718,UltB2952:0.04894)0.726.23:0.00508)0.794.62:0.0093)0.88
5.85:0.01727,((((UltGam94:0.00784,(GmmPro24:0.02357,UltBa848:0.01093)0.32
7.10:0.00376)0.964.79:0.03632,((UltMar16:0.02171,((MaiGamm2:0.02003,MaiGa
mma:0.00468)0.730.33:0.01423,((UltBa844:0.03316,UltGam92:5.3E-
4)0.894.94:0.01255,UltPlanc:0.01908)0.654.9:0.00579)0.845.91:0.00955)0.99
5.88:0.05554,(MaiGamm9:0.03264,(UltB2949:0.0037,UltB2950:0.02345)0.719.21
:0.00384)0.943.87:0.03021)0.893.104:0.02191)0.887.98:0.01998,(UltGa154:0.
03077,(UltGa170:0.03721,UltGa169:0.01901)0.217.9:0.00873)0.897.91:0.01491
)0.926.106:0.02254,((((MaiMeta2:0.00748,(((((UltB1167:0.01137,(((((UltAlc
a6:0.00371,(AlxSpec7:0.0,UltB1166:0.0,AlxSpec8:0.0):5.5E-
4)0.959.83:0.00192,UltB1168:0.01865)0.966.91:0.00195,(AlxSpec5:0.0,AlxDie
se:0.0,AlxSpec6:0.0):5.5E-4)0.341.13:5.5E-
4,(UltAlca5:0.04331,(UltB1161:0.15269,UltB1160:0.0178)0.327.11:0.00437)0.
819.59:5.5E-4)0.796.56:0.00541,(UltB1164:5.5E-
4,UltB1165:0.00744)0.947.73:0.01478)0.884.102:0.01049)0.757.52:0.00498,(U
ltB1163:0.03646,UltGa173:0.03441)0.430.10:0.01138)0.878.90:0.00988,((AlxS
pec4:0.0,UltAlca3:0.0):5.5E-
4,(UltB1158:0.00369,(AlxSpec2:0.00366,((UltB1156:0.03924,((UltGa171:0.0,A
lxBorku:0.0):5.5E-4,(UltB1157:0.00369,UltAlca2:0.0037)0.675.11:5.5E-
4)1.000.667:5.5E-
4)0.431.15:0.00739,((AlxHongd:0.0,UltB1159:0.0,UltAlca4:0.0):5.5E-
4,AlxSpec3:0.00371)0.939.80:5.4E-4)0.804.57:0.00741)1.000.668:5.4E-
4)0.706.22:5.3E-4)0.994.85:5.3E-4)0.827.63:0.0036,AlxSpeci:5.4E-
4)0.972.73:0.02588,(UltGa172:0.02737,UltB1162:0.03594)0.667.18:0.01918)0.
795.71:5.3E-
4)0.763.84:0.00412,((MrpAlkal:0.01463,((MrpInsul:0.02271,MrpMinut:5.5E-
4)0.991.76:0.02249,(GmmPro18:5.5E-4,MrpSpeci:5.5E-
4)0.774.84:0.00433)0.168.10:0.0076)0.844.98:0.01555,(HldBacte:0.05846,((C
anPort3:0.04449,(CanPort2:0.00787,(PrmEndo9:0.01817,(CanPorti:0.01896,Por
Endos:0.00877)0.990.77:0.04563)0.869.77:0.01648)0.608.17:0.01009)0.996.78
:0.05929,(ZymPalma:5.4E-4,(HltAlkal:5.3E-
4,(CarNigri:0.01055,((HlmIlici:0.03766,((KusSpeci:0.01511,(KusAuran:0.007
24,(((KusAvice:0.0,HlmSpe32:0.0):5.5E-
4,KusSpec2:0.00757)0.835.74:0.00375,UltHalo5:0.00373)0.905.98:5.5E-
4)0.911.114:0.01533)0.912.122:0.01192,((((((((HlmGudao:0.0073,((HlmElong:
0.00372,HlmSinai:5.5E-4)0.800.60:0.00381,(HlmSabkh:0.00369,HlmAlmer:5.5E-
4)0.858.86:0.00737)0.828.54:0.00751)0.903.88:5.4E-
4,(HlmAnti2:0.0,HlmAntic:0.0):0.01536)0.716.22:5.5E-4,HlmSpe20:5.3E-
4)1.000.669:5.5E-4,(ModSpeci:0.01523,HlmCerin:5.4E-
4)0.830.67:0.00365)0.478.11:0.00371,(((HlmShen2:0.0,HlmSheng:0.0):5.4E-
4,(BacteWE5:0.00369,(UltBa775:0.0,HlmCampi:0.0):5.5E-
4)0.868.77:0.0037)0.932.87:0.00251,((HlmSpe17:0.00749,HlmSpe29:0.01129)0.

722.23:5.5E-4,(HlmAlime:0.0,HlmSpe21:0.0,HlmSalin:0.0,HlmNitro:0.0):5.5E-
4)0.932.88:0.00242)0.932.89:0.00241)1.000.670:5.5E-
4,((HlmNitr2:0.0,HlmKoree:0.0):5.5E-
4,(HlmMaura:0.0112,HlmBeime:0.01134)0.759.81:5.4E-4)0.898.113:5.5E-
4)0.823.56:0.00368,(((HlmSubgl:0.00377,(HlmSpe12:0.03625,HlmHalod:0.01533
)0.714.24:0.00402)0.768.67:0.0037,(HlmHaloc:0.00776,(HlmKorle:0.01123,(((
ChrSpeci:5.4E-
4,ChrSpec3:0.01123)0.659.15:0.00637,(HlmKribb:0.03425,(CobMarin:0.0,CobSp
eci:0.0):0.01936)0.448.10:0.00951)0.777.89:0.00484,HlmSpe28:5.3E-
4)0.796.57:5.4E-
4)0.926.107:0.01136)0.863.107:0.00747)0.804.58:0.00373,((HlmSpe13:0.0,Hlm
Sali3:0.0,HlmSali2:0.0,HlmSmyrn:0.0):5.5E-
4,((UltBa771:0.18541,(((HlmVaria:5.5E-
4,(GmmPro17:0.0,HlmVari3:0.0):0.00362)0.800.61:0.00748,(((UltHalom:0.0036
8,HlmVenus:0.01115)0.363.12:5.5E-
4,((UltBa769:0.0,UltOrga3:0.0,HlmSpeci:0.0,HlmSpec4:0.0,HlmMerid:0.0,HlmS
pec5:0.0,HlmHydro:0.0,HlmSpe10:0.0):5.5E-
4,(UltBa768:0.00369,UltOrga6:0.00369)0.923.129:5.5E-4)0.874.115:5.5E-
4)0.979.72:5.5E-4,HlmSpec2:0.02623)0.814.47:0.00352)1.000.671:5.4E-
4,((((UltBa770:0.0,HlmSpec7:0.0,HlmSpec6:0.0,HlmSpec8:0.0,HlmNeptu:0.0,Hl
mSpec9:0.0,HlmSulfi:0.0,HlmSpe11:0.0,UltHalo2:0.0,PsdFluo2:0.0):5.5E-
4,(((UltBa773:0.00741,((((UltBa774:0.0,HlmDesid:0.0,HlmNitri:0.0,UltHalo3
:0.0,HlmKenye:0.0,HlmCampa:0.0,HlmSpe22:0.0,HlmDenit:0.0,HlmSacch:0.0,Hlm
Spe23:0.0,HlmSpe24:0.0,HlmSpe25:0.0,HlmSalif:0.0,UltBa782:0.0,UltHalo4:0.
0):5.5E-
4,((UltBa776:0.00755,UltBa777:0.01134)0.762.83:0.00362,((((((HlmCupid:0.
00374,HlmCamp2:0.0074)0.316.6:5.5E-4,HlmSpe16:0.00369)0.582.10:5.5E-
4,HlmSpe26:5.5E-4)0.637.9:5.5E-4,HlmPacif:5.5E-4)0.000.656:5.5E-
4,(HlmPante:0.00371,((HlmSpe19:0.0,HlmMural:0.0):5.5E-
4,HlmDaqin:0.00372)0.381.16:5.4E-4)0.868.78:0.00374)0.458.20:5.5E-
4,UltBa781:0.00368)0.473.9:5.5E-
4,(Bact75C4:0.00369,HlmSpe18:0.01116)0.773.80:0.00369)0.359.7:5.5E-
4)0.000.657:5.5E-4)0.455.18:5.3E-4,(HlmSpe15:0.02951,UltBa778:5.5E-
4)0.833.77:0.00473)0.152.10:5.3E-
4,HlmMagad:0.01124)0.829.71:0.00377)0.124.8:5.5E-
4,UltBa772:0.00369)0.335.9:5.5E-4,(HlmGomse:5.3E-
4,((HlmJangg:0.0,HlmSubte:0.0,HlmVari2:0.0):0.00247,HlmArcis:0.00247)0.93
7.94:0.00247)0.832.68:0.00369)0.539.3:5.4E-4)0.000.658:5.5E-
4,HlmSpec3:5.5E-4)0.458.21:5.5E-4,(HlmSpe27:0.00372,HlmTaean:5.3E-
4)0.865.93:0.0037)0.890.98:5.4E-4)0.887.99:5.5E-
4)0.865.94:0.00716,((HlmFonti:0.0,HlmAlim2:0.0):0.00361,HlmHalop:0.07906)
0.997.93:5.5E-4)0.533.10:5.5E-4)0.458.22:5.4E-4)0.653.8:5.4E-
4)0.962.88:0.01542,(((ChrIsra2:0.0,ChrSalex:0.0,ChrThail:0.0):5.4E-
4,(ChrIsrae:5.5E-
4,(UltBa780:0.01527,((HlmSpe30:0.0,HrrSpeci:0.0,HlmSpe31:0.0):5.5E-
4,(UltBa779:0.00741,HrrSpec2:0.00746)0.081.9:5.5E-
4)0.747.51:0.00351)0.980.54:0.02319)0.850.96:5.3E-4)0.979.73:5.5E-
4,(ChrMaris:0.0037,(ChrSpec2:0.0,ChrNigra:0.0):5.5E-
4)0.852.86:0.00365)0.863.108:0.00782)0.752.65:0.00351)0.716.23:0.00254)0.
957.72:0.02041,ModTunis:0.00828)0.981.68:0.02718)0.802.57:0.00908)0.976.5
5:0.01531)0.964.80:0.02931)0.801.69:0.01057)0.583.8:0.01782)0.928.73:0.02
01)0.969.59:0.03048,((BalAlpic:0.03667,((((PdsJapon:0.0551,(((((UltBa787:
0.00369,(((MrrLitor:0.00368,((MrrSedim:0.01263,MrrSpec3:0.02643)0.429.9:0
.00527,(MrrStan2:5.5E-

4,MrrStani:0.03925)0.917.93:0.01172)0.862.95:0.01368)0.757.53:0.00364,(Hl
mSpe33:0.01921,((((UltBa796:0.03968,((MnmSpeci:0.0,MnmSpec3:0.0):5.5E-
4,(((UltBa794:0.0,MnmAreni:0.0,MnmSpec2:0.0,MnmArct2:0.0):5.5E-
4,(MnmPolar:0.00369,(MnmPrimo:5.5E-4,(MnmArcti:0.0,ArctiSe2:0.0):5.5E-
4)0.694.15:5.5E-4)0.905.99:0.00749)0.906.105:0.00742,(MnmSpec4:5.5E-
4,(MnmDokdo:0.00744,MnmUshua:0.00755)0.370.9:5.5E-
4)0.914.109:0.00751)0.560.8:5.5E-4)1.000.672:5.4E-
4)0.843.68:0.00372,((((MnmBasal:0.0,MnmCommu:0.0):0.00369,MnmAquim:5.5E-
4)0.891.84:0.00748,MnmSpec6:0.00748)0.777.90:0.00367,(MnmnVaga:0.0,UltMar
i8:0.0):5.4E-
4)0.818.42:0.00371)0.765.62:0.00365,(((UltBa847:0.01526,UltBa846:5.3E-
4)0.848.76:0.00746,(UltGam93:0.0271,(UltBa845:5.3E-
4,(UltGam61:0.00376,UltMar17:0.00365)0.946.86:0.0113)0.763.85:0.00355)0.8
68.79:0.01176)0.993.66:0.03562,UltMari9:0.0074)0.864.90:0.00762)0.933.99:
0.01499,(MnmMedit:0.0,MnmSpec5:0.0):5.4E-
4)0.976.56:0.03211)0.904.94:0.01569)0.782.87:0.00398,UltBa786:0.0074)0.27
3.9:5.5E-4)0.247.5:5.4E-
4,UltBa792:0.00369)0.770.86:0.00748,(MaiMetag:0.03873,UltBa788:5.5E-
4)0.981.69:0.04269)0.858.87:0.01952,(NptSpeci:5.5E-
4,(BacteIS6:0.02383,NptCaesa:0.00305)0.950.85:0.01584)0.881.105:0.01187)0
.835.75:0.01269,((ProSymb4:0.04743,((MrrJanna:0.00366,UltBa793:0.00828)0.
848.77:0.01072,((MrrNitra:0.00363,(UltGam60:5.5E-
4,(MrrSpec4:0.00369,MrrRhizo:5.5E-
4)0.857.96:0.00369)0.811.58:0.00382)0.887.100:0.01125,((UltBa789:0.00744,
(((UltBa790:0.00744,MrrSpec2:5.3E-
4)0.865.95:0.00369,(MrrHalop:0.0,MrrGeorg:0.0):5.4E-
4)0.915.95:0.00744,UltMari6:0.00369)0.158.8:5.4E-
4)0.941.79:0.01121,UltBa791:5.5E-
4)0.919.125:0.01166)0.911.115:0.01378)0.749.75:0.00378)0.730.34:0.00782,(
AlrBact3:5.4E-
4,(NtnLacis:0.00915,OcpSpec2:0.12109)0.817.44:0.00983)0.458.23:0.00544)0.
706.23:0.01049)0.892.90:0.01959)0.955.69:0.02892,((NepJapon:0.01322,(GmmP
ro19:0.00368,NepNapht:5.4E-
4)0.694.16:0.00595)0.789.67:0.00776,(UltGam59:0.0051,(UltB4801:0.00208,(U
ltMari5:5.5E-
4,UltOlei2:0.02681)0.892.91:0.00909)0.911.116:0.01159)0.739.39:0.00396)0.
683.24:0.00126)0.978.57:0.0374,(ProSymb3:0.0229,UltAnta2:5.5E-
4)0.870.77:0.0166)0.246.13:0.00967,(OcpSpeci:5.4E-4,(AmpAtlan:5.5E-
4,(UltBa785:0.01128,AmpJapon:5.4E-
4)0.851.90:0.00369)0.795.72:0.00711)0.878.91:0.01042)0.859.88:0.01352)0.3
21.7:0.01115,(((OcpMulti:0.01277,(UltBa802:0.01596,UltBa803:0.03543)0.987
.91:0.03692)0.836.64:0.01069,(OcpBeije:5.5E-
4,(OcpMaris:0.00366,UltOcea2:0.00375)0.950.86:0.01126)0.797.60:0.00374)0.
813.58:0.00692,(((((((UltBa510:0.01657,((UltBa511:0.01166,ThlGangh:0.011
22)0.780.93:0.00381,ThlAgari:0.03163)0.889.93:0.01582)0.926.108:0.01913,(
ThlSedim:5.5E-
4,ThlLoyan:0.02288)0.260.9:0.00761)0.762.84:0.00364,UltGam44:0.00375)0.92
4.92:0.01141,(((((UltBa513:0.00741,(UltGam43:0.01116,(ThlVirid:0.0,ThlHal
io:0.0):5.5E-
4)0.883.106:0.0076)0.760.72:0.00364,(UltBa738:0.00371,((ColPolar:0.0,ColS
pec2:0.0):5.4E-4,((ColSpec3:0.0,ColSpec4:0.0):5.5E-
4,UltBa508:0.00369)0.988.73:0.01519)0.888.107:0.00743)0.989.65:0.02334)0.
870.78:0.00759,(HdrtVent:0.01126,UltBa514:0.00747)0.762.85:0.00365)0.873.
97:0.00773,(((UltB1041:0.00719,((UltBa509:5.4E-

4,(UltBa501:0.00365,UltGam39:0.00373)0.971.62:0.01496)0.479.14:0.00743,(U
ltBa506:5.5E-
4,((UltBa503:0.0038,((UltGam40:0.0,UltGam42:0.0,ColPsyc2:0.0,ColPsyc3:0.0
,UltBa504:0.0):5.4E-
4,(ColPsych:0.00374,ArctiSea:0.00369)0.799.75:0.00372)0.793.72:0.00365)0.
931.97:0.01128,(ColPsyc4:5.5E-4,((UltBa505:0.00369,(UltGam41:5.5E-
4,ColRosse:5.5E-4)0.882.104:5.5E-4)0.152.11:5.5E-
4,UltBa507:0.00371)0.951.85:0.00752)0.855.89:0.00728)0.935.89:5.5E-
4)0.808.66:0.00368)0.906.106:5.3E-
4)0.947.74:0.01859,UltBa500:0.03062)0.507.8:5.4E-
4,(UltColwe:0.02285,ColSpeci:5.4E-
4)0.687.16:0.00365)0.767.68:0.00353)0.942.85:0.01117,GmmPro12:5.4E-
4)0.870.79:0.00731)0.803.53:0.00718,(((UltBa497:5.5E-
4,(IdmLoih2:0.0,IdmLoihi:0.0):0.0037)0.920.107:5.4E-4,((IdmSpec6:5.5E-
4,(IdmSpec5:5.5E-
4,BacteSL4:0.03101)0.613.14:0.00739)0.996.79:0.03201,((PsnSalin:5.5E-
4,(((PsnSpeci:0.0,PsnTaina:0.0):0.00367,IdmSpec2:0.01133)0.800.62:0.00376
,(PsnDongh:0.00744,IdmSpec3:0.01499)0.225.6:5.3E-
4)0.910.94:0.00742)0.952.70:5.5E-
4,((IdmSpeci:0.00744,(PsnSedim:0.0,PsnTaiwa:0.0):0.00371)0.782.88:0.00485
,(PsnSpec2:0.01035,IdmSpec4:0.0126)0.809.65:0.0077)0.936.92:0.01423)0.825
.63:0.00744)0.935.90:0.01537)0.284.6:0.00696,(IdmBalti:5.5E-
4,((IdmLoih3:0.0,IdmSeos2:0.0):0.01103,(IdmSeosi:0.0,MrcSpeci:0.0):5.5E-
4)0.875.101:0.00718)0.929.85:5.3E-
4)1.000.673:0.05759)0.891.85:0.01523,(UltGam95:0.01123,(UltBa851:0.02307,
OlpMessi:0.00755)0.793.73:0.00364)1.000.674:5.5E-
4)0.981.70:0.00158,(UltGa153:5.3E-
4,(UltBa850:0.00391,(UltBa849:0.01153,UltBurk3:0.01536)0.748.59:0.00319)0
.985.69:0.0229)0.973.61:0.02275)0.964.81:0.00201,(((CanEndo3:0.0,EnmbnO22
:0.0):0.00376,(CanEndob:0.00742,CanEndo2:5.5E-
4)0.992.73:0.03187)0.755.62:0.00367,UltGa152:0.01136)0.879.100:0.00763)0.
977.63:0.01944)0.861.97:0.00805)0.993.67:0.03161)0.322.13:5.4E-
4,((((((((((UltB2992:0.02414,UltB2993:0.03559)0.881.106:0.01536,(((((Ul
tB2990:0.00364,(SedSelen:5.5E-4,UltB2989:0.00741)1.000.675:5.4E-
4)0.907.91:0.01608,UltB2936:0.05234)0.776.82:0.00776,((UltB2933:0.011,(Ul
tB2934:5.5E-
4,UltB2935:0.00745)0.957.73:0.03008)0.950.87:0.02589,(UltGa218:0.02303,En
mbnO26:0.00803)0.795.73:0.00723)0.760.73:0.00506)0.821.55:0.00851,UltOrg1
7:0.03964)0.404.13:0.0105,UltGa278:0.0188)0.880.103:0.00495,(((((UltB2847
:0.03484,(BggtAlba:0.07955,(AcbFerr4:0.00226,IrxAcido:0.00162)0.846.99:0.
0173)0.933.100:0.02444)0.746.53:0.01268,((UltB2755:0.0,UltB2862:0.0):0.00
702,UltB2848:0.03595)0.859.89:0.00892)0.946.87:0.02351,(((UltB2852:0.0068
7,(UltGam75:0.02234,(UltGa243:0.00742,UltGa244:0.00369)0.842.89:5.3E-
4)0.972.74:0.02328)0.989.66:0.03335,UltB2942:0.03124)0.789.68:0.00748,(Ul
tB2849:0.05128,((UltB2955:0.00765,(UltB2954:0.02834,UltB2953:0.01216)0.99
6.80:0.05209)0.607.9:0.01617,UltGa260:0.03625)0.756.73:0.00463)0.495.10:0
.01552)0.879.101:0.01273)0.905.100:0.01313,(UltGa266:0.02172,(UltGa261:0.
02133,(UltB2956:5.5E-
4,Ult27555:0.03432)0.991.77:0.03443)0.924.93:0.02004)0.812.57:5.4E-
4)0.989.67:0.03326,UltGa275:0.03716)0.773.81:0.003)0.336.10:0.01368)0.738
.35:0.00397,(UltGa281:0.01107,((UltBa873:0.0434,GmmPro32:0.02528)0.892.92
:0.01904,(GraAntar:0.03188,((SpnMarin:0.0,GmmPro31:0.0):5.3E-
4,CllSpeci:0.00713)0.976.57:0.03064)0.897.92:0.01419)0.584.12:5.3E-
4)0.777.91:0.01577)0.831.56:0.0078,((UltB2994:0.02513,(UltGa268:0.00871,U

ltGa276:0.05282)0.869.78:0.0173)0.766.69:0.00699,(((((UltB2998:0.02355,Ul
tB2999:0.05874)0.926.109:0.02339,(UltEcto2:0.02805,(((((UltB3007:0.02933,
(UltGa242:0.0032,(UltB2838:0.01535,(UltB2839:0.0,UltChro2:0.0):5.5E-
4)0.492.17:5.4E-4)0.901.99:0.0239)0.974.50:0.0324,((UltB2972:5.5E-
4,(UltrSo27:0.00374,UltB1961:0.0191)0.773.82:0.00366)0.761.64:0.00369,Ult
B2973:5.4E-4)0.948.97:5.4E-4)0.140.3:0.00738,(((TlhThioc:5.3E-
4,((UltB2938:0.01153,(UltB2937:0.03821,(UltGa216:0.02606,UltB2988:0.04234
)0.880.104:0.01932)0.639.17:0.01092)0.888.108:0.01514,SlfBact2:0.0149)0.4
53.10:0.01232)0.892.93:0.01146,(UltB2846:0.04453,UltGa282:0.02408)0.718.2
3:0.0035)0.948.98:0.01603,((UltB2995:0.01073,(UltB2997:5.5E-
4,(UltGa279:0.01888,(UltB2996:0.00743,OlvAlgar:5.4E-4)0.996.81:5.5E-
4)0.342.9:0.00405)0.916.106:0.015)0.990.78:0.03174,(UltDechl:0.0402,(UltB
2736:0.01291,(UltGa253:0.02867,(UltB2915:0.0037,UltB2916:5.5E-
4)0.719.22:0.00276)0.996.82:0.05969)0.856.78:0.01136)0.865.96:0.01155)0.4
18.10:5.4E-
4)0.901.100:0.01292)0.841.75:0.00801,(UltB2974:0.0209,(UltGa116:0.01326,(
UltPse11:0.00878,GmmPro29:0.01908)0.750.49:0.00508)0.954.78:0.02902)0.809
.66:0.00959)0.849.81:0.01289,(UltB2864:0.0233,(UltB2930:0.01167,UltB2866:
0.02723)0.763.86:0.00333)0.965.70:0.02355)0.757.54:0.00349)0.868.80:0.012
6)0.406.11:0.00457,(Ult26399:0.05463,(UltB2829:0.06541,(UltB2828:0.04802,
UltB2840:0.07238)0.771.90:0.00944)0.836.65:0.02576)0.840.83:0.01656)0.595
.7:0.00891,((UltB2865:0.0034,UltB2863:0.01543)0.857.97:0.00778,(((AklMobi
l:0.0,AkmHalod:0.0):0.02414,ThnNitra:0.02395)0.861.98:0.01119,(UltB2991:0
.01919,EnmbnO24:0.00734)0.895.113:0.01154)0.856.79:0.00774)0.877.112:0.00
81)0.706.24:0.00272,UltB2758:0.03262)0.856.80:0.00889)0.909.84:0.01309)0.
869.79:0.00916,(((UltBa874:0.03904,TrmTepid:0.07852)0.929.86:0.03715,(Enm
bnO25:0.01587,Ult13067:0.07974)0.985.70:0.03713)0.766.70:0.01139,(((NatAc
eti:0.02332,(UltB2826:0.03903,(MtmKenye:0.03279,UltB2827:0.0087)0.746.54:
0.00815)0.874.116:0.02235)0.966.92:0.03634,((((UltB2970:0.05494,(UltB2880
:0.01412,UltB9935:0.01238)0.848.78:0.01637)0.889.94:0.01971,(MtntrE10:0.0
16,((MtcCapsu:5.4E-
4,((MtdTepid:0.00402,(MtdGraci:0.0037,MtdSzege:0.00764)0.899.100:0.01128)
0.995.89:0.03648,MtcCaps2:0.01535)0.825.64:0.01141)0.885.86:0.01482,((Ult
B2823:0.01069,UltB2943:0.05089)0.842.90:0.01385,UltMeth6:0.04245)0.507.9:
0.00948)0.966.93:0.03067)0.899.101:0.01784)0.451.16:0.00987,(ThbProsp:0.0
4325,SlfBacte:0.01369)0.796.58:0.00896)0.764.83:0.01061,((((((((UltGa256
:0.00538,(UltGa258:0.03757,UltB2860:0.01443)0.693.17:0.00776)0.904.95:0.0
1762,(UltGa255:0.04023,Ult16042:0.03741)0.075.3:0.00519)0.659.16:0.00951,
(((UltB2914:5.5E-4,((UltB2910:0.00738,UltB2911:5.4E-
4)0.747.52:0.00743,(UltB2912:0.01095,UltB2913:0.00199)0.916.107:0.00196)0
.999.129:5.4E-
4)0.898.114:0.02139,(UltB2904:0.04296,((Ult16048:0.02492,(UltB2908:0.0386
1,UltB2907:0.02187)0.892.94:0.0315)0.484.16:0.02515,((Ult16376:0.03523,Ul
tB2892:0.01679)0.748.60:0.00471,(((UltB2896:0.01525,(UltB2906:0.00756,(Ul
t16893:0.0538,UltB2859:0.01367)0.763.87:0.00414)0.969.60:0.01558)0.949.88
:5.4E-
4,((((UltB2899:0.02676,UltB2898:0.00677)0.896.86:0.0169,(((UltPse47:5.5E-
4,UltB2902:0.00742)0.846.100:0.00701,(UltB2905:0.00375,(UltB2894:0.00369,
UltB2895:5.5E-
4)0.808.67:0.00373)0.784.81:0.00425)0.874.117:0.00764,((UltB2900:0.00371,
UltB2901:0.00371)0.806.52:0.00394,(UltPse46:0.01214,(UltB2897:5.5E-
4,UltB2903:0.00741)0.978.58:0.0237)0.820.51:0.00704)0.779.76:0.00409)0.73
9.40:0.00397)0.456.11:0.00387,UltB2893:0.02669)0.666.18:5.3E-
4,UltB2909:0.01025)0.275.11:0.00843)0.788.70:0.014,UltGa251:0.01278)0.297

.4:0.00433)0.745.63:0.0067)0.748.61:0.01695)0.866.89:0.04407)0.427.10:0.0
1621,((((StdDenit:5.3E-
4,(UltB2884:0.00746,(((UltB2891:0.01514,((UltChro5:0.0,UltB2890:0.0,UltXa
n22:0.0):5.5E-4,(Ult16433:0.00369,Ult16432:0.02277)0.894.95:5.5E-
4)0.978.59:5.5E-
4)0.936.93:0.00746,(UltPse45:0.01504,(UltB2882:0.01502,UltXan20:5.4E-
4)0.550.5:0.00695)0.914.110:5.4E-
4)0.807.51:0.00375,UltB2886:0.01512)0.783.87:0.00371)0.926.110:0.0035)0.2
83.6:0.00938,(UltB2883:0.02207,UltGa250:0.03392)0.677.13:0.01538)0.722.24
:0.00568,UltB2885:0.01835)0.930.80:5.4E-
4,(((UltB2888:0.02258,UltB2889:0.02003)0.969.61:0.02804,(UltB2887:5.5E-
4,(UltrSo25:0.00374,UltGa249:0.00371)0.797.61:0.00373)0.893.105:0.01049)0
.969.62:5.4E-
4,UltXan21:0.00797)0.968.75:0.01559)0.429.10:0.01428)0.948.99:0.02832)0.8
84.103:0.01399,(UltB2857:0.06224,((UltB2932:0.03519,(UltGa257:0.02223,Ult
B2918:0.01493)0.738.36:0.00528)0.759.82:0.0037,(UltB2751:0.05806,UltB2931
:0.04333)0.958.91:0.03662)0.787.66:0.00368)0.000.659:5.4E-
4)0.759.83:0.00449,((UltB2925:0.01129,(UltB2929:0.00841,(UltB1391:0.01527
,UltGa204:0.00997)0.716.24:7.5E-4)0.874.118:0.01135)0.592.9:5.5E-
4,((((UltB2921:0.01526,UltB2923:0.00754)0.764.84:0.00368,((UltB2926:0.007
44,(UltB2920:0.00747,UltB2922:5.5E-4)0.731.28:5.4E-
4)0.867.98:0.00367,((UltB2924:5.5E-
4,UltB4805:0.01925)0.964.82:0.01543,UltB2927:0.00749)0.852.87:5.4E-
4)0.805.58:0.0037)0.785.80:0.004,UltGa259:0.015)0.399.13:0.00357,(UltB288
1:0.02325,UltB2928:0.02812)0.743.57:0.00405)0.939.81:0.01492)0.098.1:0.00
6)0.000.660:5.5E-
4,SlfBact3:0.068)0.347.7:0.01179,(((((((UltBeggi:0.0774,(MhmLacus:0.04719
,UltGa246:0.05672)0.520.6:0.02067)0.737.54:0.00384,((UltGa102:0.03017,((B
erMaris:0.0067,(UltBa798:0.02961,(UltOleis:0.01893,(UltBa797:0.02812,SpoN
orve:0.00332)0.907.92:0.01276)0.000.661:0.00375)0.733.34:0.01873)1.000.67
6:0.05079,UltGa101:0.04177)0.000.662:5.3E-
4)0.990.79:0.04227,(UltGa265:0.08506,((UltB2819:0.03631,(UltB2820:0.03578
,(UltB2822:0.01991,(UltB2818:0.0482,(UltB2817:0.01727,(CoxBurne:0.01638,B
acEnri9:0.02459)0.390.13:0.005)0.879.102:0.0105)0.809.67:0.0069)0.591.13:
5.5E-
4)0.811.59:0.00845)0.350.9:0.00638,(UltGa226:0.05071,UltGam91:0.0633)0.78
1.71:0.01214)0.410.13:0.00734)0.070.7:0.00478)0.923.130:0.01848)0.670.12:
5.5E-4,((Ult16043:0.05111,(UltrSo26:0.01494,(UltGa254:5.5E-
4,UltBur17:0.06947)0.811.60:5.3E-
4)0.939.82:0.02214)0.958.92:0.04092,UltPro31:0.07867)0.741.49:0.00768)0.8
10.63:0.00457,(UltGa220:0.04201,UltB2951:0.0711)0.713.28:0.00574)0.779.77
:0.00445,(((UltGa252:0.04946,(UltB2821:0.07736,UltGa236:0.00414)0.915.96:
0.02622)0.922.138:0.02293,UltB2919:0.00705)0.653.9:0.0116,UltB2917:0.0080
4)0.974.51:0.03138)0.834.62:0.00736,((Ult16631:0.0,Ult16632:0.0):0.04722,
(((UltB2853:0.01024,Ult17397:0.01655)0.872.97:0.01684,UltGa245:0.12552)0.
209.9:0.00697,(UltB2854:0.00665,(UltGa219:0.03435,UltB2760:0.03403)0.967.
66:0.03798)0.413.13:0.01316)0.906.107:0.02499)0.838.83:0.0133)0.849.82:0.
00828,(((UltOrg15:0.01604,UltB2858:0.01508)0.920.108:0.02257,((((UltB2957
:0.01091,UltB2958:0.04028)0.763.88:0.00608,(UltB2959:0.0043,UltB2960:0.02
67)0.758.72:0.00531)0.918.97:0.01999,(((UltB2962:0.01478,(UltGa267:0.0113
9,UltB2961:0.02709)0.971.63:5.4E-
4)0.957.74:0.01892,UltB2963:0.00767)0.910.95:5.4E-
4,UltPro33:0.0186)0.748.62:0.02055)0.952.71:0.03436,(UltB2855:0.05155,Ult
B2969:0.05335)0.778.79:0.01071)0.913.106:0.01937)0.728.36:0.01636,UltB283

3:0.04114)0.892.95:0.02565)0.907.93:5.4E-
4)0.894.96:0.00798,(OlvCrass:0.03722,(SulCaldi:0.06026,UltB2747:0.02283)0
.942.86:0.02784)0.862.96:0.0121)0.744.51:0.00357,((((UltB2841:0.0302,(((U
ltChrom:0.0,UltChro3:0.0):5.5E-
4,UltChro4:0.00369)0.856.81:0.0171,(UltB2824:5.4E-
4,UltB4790:0.03123)0.998.106:0.05334)0.886.116:0.02348)0.412.8:0.01484,Ul
tB2850:0.01491)0.928.74:0.02188,((HlrAbdel:0.01475,(HlrNeutr:0.01855,HlrH
alop:0.00459)0.985.71:0.03713)0.957.75:0.02194,(UltEcto3:0.07818,((UltB28
51:5.4E-
4,((TlvHalop:0.00582,((TlvSpec2:0.00373,TlvNitra:0.01127)0.748.63:0.00372
,(TlvSpeci:0.00526,TlvVersu:0.0189)0.922.139:0.01478)0.880.105:0.01325)0.
938.59:0.02094,(TlvNitr2:0.00412,TlvParad:0.0229)0.799.76:0.0152)0.929.87
:0.0198)0.970.72:0.01513,(((ThdSibir:0.02735,((EctMongo:0.02785,(PhpBact2
:0.008,PhpBacte:0.04067)0.802.58:0.00741)0.786.79:0.00355,(EcrMarin:0.007
43,EcrVaria:5.4E-
4)0.755.63:0.00398)0.837.59:0.00813)0.981.71:0.02435,(TlvDenit:0.00609,Tl
vThioc:0.00602)0.887.101:0.01075)0.772.76:0.00336,EcrMobil:0.03626)0.781.
72:0.00423)0.962.89:5.4E-
4)0.865.97:0.01581)0.875.102:0.01354)0.880.106:0.01639,(UltB2757:0.01223,
(UltB2725:0.01636,UltB2843:0.06276)0.900.81:0.01565)0.881.107:0.01169)0.7
82.89:0.00635)0.909.85:0.00826)0.904.96:5.4E-
4)0.823.57:0.0046,(((UltEctot:0.01089,AqlAsiat:5.4E-
4)0.984.61:0.03804,(NrsOcean:0.00766,NrsOcea2:0.03213)0.980.55:0.03536)0.
850.97:0.01122,(UltB2947:0.03635,(((UltB2948:0.03352,(UltB2825:0.00415,(N
ccMobil:0.05582,UltGa238:8.4E-
4)0.600.11:0.01807)0.936.94:0.02266)0.587.11:0.01237,((GmmPro38:0.02953,(
(OlvIlvae:5.5E-4,GmmPro39:0.01129)0.201.9:5.5E-
4,(OlvCras2:0.01507,OlvLoisa:0.01599)0.773.83:0.00765)0.852.88:0.00985)0.
568.11:0.00829,((TflMobi2:5.5E-
4,(TccPfenn:0.0037,TflMobil:0.01545)0.819.60:0.00355)0.945.77:0.02703,(((
((TlcHalop:0.00745,LmpPurpu:0.00377)0.899.102:0.01115,(UltGa264:0.00379,(
HcrSalex:0.00765,HcrRoseu:0.01931)0.849.83:0.00742)0.482.13:0.01162)0.801
.70:5.3E-4,(TlcMarin:0.00768,((((LmpRoseo:0.02283,UltB2940:5.4E-
4)0.960.88:0.01899,((TbcTruep:0.01666,(TcpImhof:0.01025,(TcpLitor:0.02273
,((TcpRosea:0.0,TcpRoseo:0.0):5.5E-
4,(TcpMarin:0.0,TcpMachi:0.0):0.00743)0.592.10:5.5E-
4)0.864.91:0.01359)0.540.10:0.01797)0.862.97:0.01223,((UltB2946:0.02272,U
ltB2945:0.02173)0.964.83:0.02633,(TdcElega:0.01174,TctViol3:0.02731)0.354
.10:5.5E-
4)0.052.7:0.01772)0.853.71:0.00918)0.892.96:0.00374,(MrhSpeci:0.00753,(((
(UltGa263:0.01047,((((((AcrRenuk:0.00372,AcrWarmi:0.00754)0.936.95:0.0114
4,(AcrVinos:0.0,AcrMinut:0.0):5.4E-4)0.857.98:0.00369,AcrPhaeo:5.4E-
4)0.765.63:0.00386,CrmSpeci:0.08221)0.766.71:0.00354,((TctViol2:0.01926,(
TctGelat:5.5E-4,UltB2939:0.01132)0.271.6:5.5E-
4)0.798.65:0.00367,TctViola:0.03156)0.772.77:0.00401)0.699.22:0.00379,(Ul
tChro6:5.5E-4,CrmOkeni:5.5E-
4)0.987.92:0.04023)0.853.72:0.02069)0.895.114:0.01234,((TmpPedio:0.05274,
(TrhMinor:0.00428,(TrhManni:0.00698,TphMangr:0.02783)0.627.7:0.00369)0.80
7.52:0.01104)0.913.107:0.02129,TrhBheem:5.3E-
4)0.878.92:0.00784)0.550.6:0.00507,((TrhDrew2:0.0,TrhDrews:0.0):0.0037,Tr
hKakin:5.5E-
4)0.873.98:0.01039)0.904.97:0.01516,(MrhBheem:0.01128,MrhGraci:5.5E-
4)0.850.98:0.00883)0.893.106:0.00998)0.852.89:0.00704)0.886.117:0.00109,U
ltB2941:5.4E-

4)0.952.72:0.01506)0.800.63:0.00339)0.713.29:0.00453,UltGa262:0.04307)0.1
12.4:0.00696,(RhaMarin:0.02093,TrvWinog:0.0109)0.930.81:0.02286)0.177.13:
0.00952)0.933.101:0.02493)0.582.11:0.00687)0.919.126:0.01778,(UltB2944:0.
0048,UltB4793:0.03632)0.879.103:0.01096)0.741.50:0.00315)0.823.58:0.00794
)0.868.81:0.00787)0.933.102:0.01108)0.688.18:5.4E-
4)0.907.94:0.01225,((((UltB2964:0.03365,UltB2965:0.05225)0.984.62:0.07366
,(UltB2966:0.06804,UltB2971:0.05068)0.197.9:0.02501)0.921.109:0.04186,(((
UltB2716:0.00223,IgtLarva:0.00518)0.943.88:0.03759,(UltB2717:0.04445,((Ig
tSpeci:5.5E-4,WohChiti:0.00741)0.973.62:0.01775,(UltB2714:5.5E-
4,UltB2715:0.0074)0.749.76:0.00484)0.277.11:0.00455)0.628.4:0.02392)0.980
.56:0.05518,(((((((((TheFusca:0.0,TheFusc2:0.0):0.01467,(((((PsdPicto:5.
5E-4,StrAcida:0.00369)0.360.12:5.5E-
4,(UltSten4:0.02728,(UltrSo23:0.00376,(UltB2612:0.0,StrSpec5:0.0):5.5E-
4)0.769.76:0.00336)0.892.97:0.00781)0.987.93:0.02375,((UltB2625:0.0201,((
(XntCampe:5.5E-4,UltB2626:5.5E-4)0.838.84:0.00364,(XntAxon2:5.5E-
4,XntAxono:0.03387)0.769.77:5.5E-
4)0.764.85:0.00361,((PsxBroeg:0.00369,PsxSuwon:5.5E-4)0.920.109:5.5E-
4,(PsxTaiwa:0.00829,((UltB2623:5.5E-
4,UltB2624:0.00368)0.942.87:0.01085,((((XntAlbi2:0.0,XntTrans:0.0):5.5E-
4,(XntSpec2:0.01121,XntAlbil:5.5E-4)0.334.9:5.5E-4)0.996.83:5.5E-
4,UltB2627:0.03062)0.975.66:0.01536,PsxKoree:0.01529)0.211.10:5.4E-
4)0.893.107:0.01147)0.843.69:0.007)0.883.107:0.00746)0.833.78:0.00701)0.9
09.86:0.01463,((UltB2621:0.01487,((UltGa210:0.0,PsxJapon:0.0,PsxMexic:0.0
,PsxSpec2:0.0):0.01113,UltB2622:0.01188)0.964.84:0.0242)0.818.43:0.01548,
(((UltXant2:0.0112,(PsxSache:0.0,PsxDokdo:0.0):5.4E-4)0.913.108:5.5E-
4,(UltB2620:0.0058,(PsxSpeci:0.00726,(PsxSpadi:5.4E-
4,(PsxKalam:0.03106,UltXant3:5.4E-4)0.842.91:0.00367)0.921.110:5.3E-
4)0.905.101:0.01022)0.862.98:0.00993)0.869.80:0.00751,UltrSo24:0.01961)0.
953.70:5.4E-
4)0.914.111:0.01537)0.850.99:0.00757)0.744.52:0.00287,((((UltB2610:5.5E-
4,(UltB2618:0.00744,((XntSpeci:0.12068,((UltB2604:0.0,StrMal10:0.0,StrMal
11:0.0):5.4E-4,UltB2611:0.0075)0.715.20:5.5E-
4)0.860.68:0.0072,UltrSo22:5.4E-4)0.845.92:0.00371)0.889.95:5.4E-
4)0.199.4:5.4E-4,(StrSpec6:0.0151,((UltSten3:0.02681,UltSten2:5.5E-
4)0.824.58:5.3E-4,((((UltB2601:0.0,StrMalt2:0.0):5.5E-
4,(StrSpec4:0.0037,UltB2617:0.00369)0.702.15:5.5E-4)0.000.663:5.5E-
4,StrMalt7:5.5E-4)0.716.25:5.4E-
4,(((UltB2603:0.0,StrMalt8:0.0,PsdGenic:0.0):5.5E-
4,(UltB2605:0.01117,((StrSpec2:0.0,StrSpec3:0.0):0.01557,StrMalt9:0.03071
)0.773.84:0.00361)0.246.14:5.5E-4)0.805.59:5.4E-
4,((PsdBoreo:0.01903,(StrMalt3:0.00369,(PsdBetel:5.5E-
4,(UltB2606:0.0074,UltB2608:0.00369)0.861.99:5.5E-4)0.735.34:5.5E-
4)0.772.78:0.00522)0.885.87:0.01056,((StrMalto:0.0,StrMalt4:0.0,StrMalt5:
0.0,StrMalt6:0.0):5.4E-4,(StrSpeci:5.5E-
4,UltB2607:0.11018)0.894.97:0.00749)0.100.5:5.5E-
4)0.884.104:0.00752)0.818.44:0.00372)0.922.140:0.00748)0.392.8:5.4E-
4)0.856.82:0.00374)0.779.78:0.00429,(UltSteno:0.02646,(((UltB2613:0.00374
,(UltRume9:0.01132,(UltB2614:0.00742,UltB2615:0.0037)0.885.88:5.3E-
4)0.885.89:0.0075)0.952.73:5.5E-
4,UltB2616:0.00725)0.851.91:0.00723,StrKoree:5.4E-
4)0.920.110:0.01193)0.494.7:0.00751)0.775.77:0.01114,(UltB2609:5.4E-
4,((UltB2619:0.0,StrMal12:0.0,StrRhizo:0.0):5.5E-
4,(StrMal13:0.03051,StrSpec7:5.5E-4)0.821.56:5.5E-
4)0.962.90:0.00742)0.814.48:5.3E-

4)0.910.96:0.01171)0.860.69:0.01598,((UltXant8:0.01525,UltXant9:0.04652)0
.994.86:0.06374,(XllFast2:5.5E-
4,(XllFasti:0.0,XllFast3:0.0,UltXylel:0.0):0.00364)0.137.6:0.0107)0.713.3
0:0.00653)0.913.109:0.01991)0.980.57:0.03971,((UltB2711:0.01442,((((UltB2
640:0.01601,UltB2641:0.04464)0.866.90:0.01776,(UltXant7:0.02782,(UltGa211
:0.00369,(UltXant4:0.0,UltXant5:0.0,UltXant6:0.0):5.5E-
4)0.747.53:0.00386)0.988.74:0.04032)0.726.24:0.00999,(SnnLenta:0.00849,Ul
tB2709:0.03885)0.912.123:0.01697)0.429.11:0.00629,Ult14474:0.06352)0.163.
7:0.00327)0.817.45:0.01053,((((UltB2644:0.01138,((LutMarin:5.5E-
4,LutCompo:0.00369)0.972.75:0.01508,UltB2665:0.0037)0.875.103:0.00774)0.8
58.88:0.00754,(((((UltAcid8:5.5E-4,((UltB2628:0.0,UltB2662:0.0):5.5E-
4,((UltTher2:5.5E-
4,UltLysob:0.00369)0.869.81:0.0037,UltGa213:0.00369)0.726.25:5.5E-
4)0.883.108:5.5E-4)0.907.95:5.4E-
4,UltXan14:0.01508)0.919.127:0.00744,(UltB2646:0.00742,((Udntddde:0.0,Lut
Aquat:0.0):5.5E-4,(UltB2602:0.0037,UltB2642:0.00745)0.842.92:5.5E-
4)0.397.12:5.5E-4)0.027.5:5.4E-4)0.860.70:0.00366,(UltXan13:5.5E-
4,UltB2643:0.01122)0.439.10:5.5E-
4)0.947.75:0.00737,(((UltB2668:0.0263,UltB2710:0.01258)0.784.82:0.00759,U
ltB2658:0.014)0.782.90:0.01234,((((UltB2667:0.02303,(LysConcr:5.3E-
4,LysSpong:0.00369)0.937.95:5.3E-
4)0.773.85:0.00271,(LysSpec3:0.00743,((((UltB2669:0.00742,((LysAnti2:0.0,
LysAntib:0.0):5.5E-4,(LysSpec5:0.00369,LysCapsi:5.5E-
4)0.839.76:0.00379)0.856.83:0.00378)0.272.9:5.4E-
4,UltB2856:0.00369)0.954.79:5.5E-4,((LysNiast:5.5E-
4,LysSpec4:0.01505)0.805.60:0.00369,LysSpec6:5.4E-
4)0.893.108:0.0074)0.913.110:0.0115,(LysSpec2:0.0037,LysDaeje:5.4E-
4)0.821.57:0.00365)1.000.677:5.4E-
4)0.891.86:0.01009)0.838.85:0.00945,(LysOryza:5.5E-4,(LysYangp:5.4E-
4,((LysNiabe:0.0,UltLyso2:0.0):5.4E-4,(LysKoree:0.00753,LysSpeci:5.5E-
4)0.832.69:0.00369)0.972.76:0.01121)0.975.67:0.01506)0.791.64:0.00598)0.7
98.66:0.00609,(TheHaemo:0.00746,(TheHydro:0.01549,(((((UltB2654:0.0,UltB2
664:0.0):5.4E-
4,(UltB2653:0.0037,((UltTher5:0.00749,UltTher6:0.00373)0.795.74:0.00371,(
UltB2645:0.01527,LutAestu:0.00743)0.136.8:5.3E-
4)0.815.62:0.00372)0.813.59:0.00372)0.900.82:0.0074,UltB2648:0.09606)0.58
5.4:5.4E-4,((UltB2651:5.5E-
4,UltB2652:0.12342)0.813.60:0.00364,((UdnBac10:5.4E-4,(UltB2647:5.4E-
4,(UltB2649:0.00372,UltB2650:0.00369)0.952.74:0.01898)0.787.67:0.01926)0.
744.53:0.00371,(((((LutSpeci:5.5E-
4,LutSpec2:0.00369)0.904.98:0.00746,(UltTherm:0.00747,UltTher4:0.00374)0.
801.71:0.00366)0.784.83:5.4E-4,UltB2656:0.00368)1.000.678:5.5E-
4,(UltB2666:0.00373,XntSpec3:0.00757)0.826.59:0.00366)0.092.7:0.00373,(Ul
tB2655:0.0,UltB2657:0.0):5.4E-
4)0.840.84:0.0037)0.903.89:0.00761)0.346.13:5.5E-
4)0.735.35:0.00357,UltTher3:0.01147)0.758.73:0.00377)0.727.27:0.00399)0.5
88.12:0.00366)0.442.14:0.01179)0.966.94:5.5E-4)0.442.15:5.4E-
4)0.882.105:0.00945,((XnmBacte:0.00792,UltB2663:0.0307)0.831.57:0.0167,((
(UltSten5:0.04126,LysXimon:0.02355)0.374.12:0.01315,(UltB2861:0.03643,Ult
Pro32:0.01887)0.996.84:0.04964)0.735.36:5.4E-
4,(UltB2697:0.06788,((UltB2632:0.0,UltB2633:0.0):5.4E-
4,(UltXan10:0.01567,UltB2631:0.0118)0.813.61:0.00298)0.372.11:0.00389)0.7
89.69:0.01223)0.606.12:0.00796)0.943.89:0.03283)0.285.8:5.4E-
4,((UltPse44:0.01742,(((AreMalth:0.0,UltB2630:0.0):5.5E-4,AsrCompo:5.5E-

4)0.818.45:0.00368,(AreDongh:5.5E-4,Udntfde4:5.5E-4)0.631.12:5.4E-
4)0.841.76:0.00932)0.777.92:0.00568,((UltB2686:0.04265,(((UltXan16:0.0160
2,((UltB2696:0.0272,(UltB2712:0.02257,(UltDokdo:5.4E-4,(UltDokd2:5.5E-
4,((DokGinse:0.0034,(UltB2629:0.00368,UltGa212:0.00373)0.994.87:0.02759)0
.942.88:0.01592,DokFugit:0.00353)0.841.77:0.00777)0.896.87:0.01091)0.656.
13:0.00914)0.949.89:0.01924)0.751.46:0.00356,(UltB2699:0.03115,((LysTaiwa
:0.03027,(UltB2698:0.02665,UltXan17:0.0247)0.952.75:0.0234)0.815.63:0.009
38,DokKoree:5.3E-
4)0.959.84:0.02635)0.932.90:0.02103)0.736.43:0.00418)0.858.89:0.01345,(Ba
cCYCU2:0.02893,((UltGa214:5.5E-
4,UltB2707:0.01901)0.991.78:0.02728,UltB2659:0.0077)0.771.91:0.00741)0.82
9.72:0.01778)0.681.13:0.00287,UltB2702:0.03511)0.308.9:0.01096)0.878.93:0
.01211,(((UltPro30:0.00775,((UltB1962:0.0,UltB2687:0.0):5.5E-
4,UltB1963:5.5E-
4)0.935.91:0.01577)0.864.92:0.02052,(GmmPro37:0.01855,UltXan15:0.01646)0.
781.73:0.00821)0.913.111:0.01868,(UltB2637:0.02557,(((UltB2638:0.02326,Ul
tB2639:0.00387)0.896.88:0.00195,(UltXan11:0.00759,((UltB2634:0.01493,UltB
2635:5.5E-4)0.944.72:0.01097,AreOryzi:0.00715)0.919.128:5.4E-
4)0.923.131:0.007)0.879.104:0.00196,(UltB2636:0.0,UltXan12:0.0):5.5E-
4)0.855.90:0.01118)0.918.98:0.01981)0.825.65:0.00768)0.877.113:0.01084)0.
871.98:0.01053)0.765.64:0.00835)0.360.13:0.01783)0.859.90:5.4E-
4,(UltB2670:0.02295,(UltB2661:0.00369,UltB2660:5.5E-
4)0.951.86:0.01901)0.419.8:0.00765)0.830.68:0.00752,((UltB2684:0.02376,Ul
tB2685:0.01055)0.961.74:0.02297,(AqmVorai:5.4E-
4,UltB2708:0.01502)0.882.106:0.00963)0.901.101:0.01417)0.773.86:0.00368,(
(((((((AcbFerro:0.0,AcbFerr2:0.0):5.5E-
4,(AcbThioo:0.0,UltGa241:0.0):0.0075)0.887.102:0.00723,UltB2834:0.02662)0
.928.75:5.5E-4,(AcbFerr3:5.5E-
4,UltB2837:0.00369)0.835.76:0.0037)0.955.70:5.4E-
4,UltAcidi:0.00738)0.390.14:0.00882,(UltB2835:5.5E-
4,(ThbSpec2:0.06981,((UltAcid9:0.00361,(AcbThio2:5.5E-
4,(AcbCald2:0.00369,AcbCaldu:5.5E-
4)0.928.76:0.01103)0.251.7:0.00754)0.759.84:0.00386,UltB2836:5.3E-
4)0.804.59:0.00858)0.931.98:0.01831)0.911.117:0.01789)0.988.75:0.0617,(Ul
tBa932:0.04027,UltB2674:0.0561)0.794.63:0.02053)0.782.91:0.02393,((((DyeG
inse:0.00753,((UltB2695:5.5E-4,UltB2703:0.0074)0.765.65:5.4E-
4,((UltB2713:0.01407,UltB2704:0.00416)0.877.114:0.00424,(UltB2694:0.01488
,UltB2261:0.05165)0.817.46:5.5E-
4)0.975.68:0.01912)0.989.68:0.02714)0.897.93:5.5E-
4,((UltFrate:0.00371,(UltB2691:5.4E-
4,((((LtbSpeci:0.00377,(RhtSpeci:5.3E-
4,RhtThioo:0.00745)0.910.97:0.0075)0.849.84:0.0075,UltB2689:0.01561)0.848
.79:0.00712,(UltB1964:5.5E-4,UltB2681:5.5E-
4)0.935.92:0.00206)0.409.10:5.2E-
4,((((((UltB2690:0.0,UltB2677:0.0,UltB2682:0.0):0.00752,RhtGinse:5.4E-
4)0.908.110:0.00739,(UltB2673:0.01137,UltB2675:0.00754)0.998.107:5.4E-
4)0.263.7:0.00742,((UltB2672:0.00725,(UltB2678:0.0,UltB2679:0.0):0.01122)
0.930.82:5.4E-
4,(RhtTerra:0.0037,((UltB2680:0.0,UltB2683:0.0,RhtSpath:0.0):5.5E-
4,RhtLinda:5.5E-4)0.952.76:0.00753)0.233.7:5.4E-4)0.677.14:5.3E-
4)0.924.94:0.0074,(UltB2688:5.5E-4,(UltB2692:0.0,UltB2693:0.0):5.5E-
4)0.839.77:0.00369)0.560.9:5.5E-
4,UltB2676:0.00741)0.918.99:0.00696)0.933.103:0.00201)0.923.132:0.00736)0
.607.10:5.4E-

4,LtbRhizo:0.01522)0.887.103:0.0073)0.880.107:0.01519,(FulvSoli:0.01118,(
(DyeYeoju:0.01453,(UltB2671:0.01484,(UltB2705:0.02338,UltB2706:0.02421)0.
811.61:0.01253)0.642.10:0.01519)0.752.66:0.00451,(DyeTerra:0.00758,DyeSpe
c2:0.00765)0.861.100:0.00762)0.842.93:0.00721)0.916.108:5.5E-
4)0.889.96:0.00736,((FrtAuran:0.0,FrtAura2:0.0,DyelSoli:0.0,DyeSpeci:0.0,
DyeKoree:0.0):5.5E-4,(FrtAura3:0.01118,PahggYy3:0.00369)0.956.81:5.5E-
4)0.976.58:5.3E-
4)0.872.98:0.01253)0.501.10:0.01095,(UltB2700:0.10611,UltB2701:5.4E-
4)0.997.94:0.0447)0.797.62:0.00705)0.950.88:0.01196,(MtrTherm:0.06604,(Mt
sSpeci:0.01362,MtsCrime:0.02125)0.540.11:0.00882)0.923.133:0.02098)0.620.
9:5.5E-
4,(UltB2987:0.08066,(UltB2724:0.03151,(HlhKelly:0.0348,((TllHalop:0.02321
,(HlhHalop:0.00745,UdnBac11:5.5E-
4)0.752.67:0.0086)0.992.74:0.04159,(HlhNeapo:0.03316,UltB2718:0.03037)0.9
49.90:0.03164)0.065.12:5.5E-
4)0.962.91:0.03485)0.764.86:0.02145)0.222.5:0.02024)0.931.99:0.0263,(((Ul
tB2271:0.02143,(UltB2879:0.0686,(Ult27551:5.3E-
4,(UltB2877:0.02396,(UltB2878:0.00369,Ult27552:5.4E-
4)0.849.85:0.00772)0.904.99:0.01123)0.763.89:0.00829)0.734.47:0.01022)0.9
98.108:0.0969,(UltB2830:0.03172,(SlhSpeci:0.00902,SlhHydro:0.03893)0.961.
75:0.0351)0.966.95:0.05386)0.535.11:0.01308,(UltrB141:0.22785,((UltB2723:
0.00732,(UltB2722:0.04287,ThvSulfu:9.5E-
4)0.957.76:0.00304)0.974.52:0.05527,((UltB2550:0.0,ThfTepid:0.0,UltB2719:
0.0):0.01119,(UltB2721:0.02558,UltB2720:0.01353)0.255.2:0.02178)0.988.76:
0.07958)0.763.90:0.0249)0.874.119:0.02541)0.816.62:0.01445)0.091.3:0.0038
6,((((((UltB2872:0.0,UltB2873:0.0):0.00368,(UltB2871:0.00989,(SoliSoli:0.
0072,SinFlavu:0.01181)0.956.82:0.01942)0.868.82:0.01)0.882.107:0.0074,Ult
Ga247:5.3E-
4)0.986.68:0.05226,(UltB2876:0.04834,(UltGa248:0.01915,UltB2874:0.06154)0
.283.7:0.01138)0.167.8:0.01079)0.966.96:0.04236,(((UltXan19:0.02844,(Ultr
Rap4:5.5E-
4,(AlbDiffi:0.09128,(UltB2870:0.00372,(NevRamos:0.00371,((UltB2867:5.4E-
4,UltB2868:0.03843)0.897.94:0.00732,UltB2869:0.00747)0.769.78:0.0036)0.79
2.84:0.00363)0.998.109:5.4E-
4)0.948.100:0.03444)0.968.76:0.02929)0.961.76:0.03311,PncPersp:0.01826)0.
938.60:5.5E-
4,HdcEffus:0.05125)0.169.5:0.01174)0.520.7:0.01871,(UltB1095:0.09917,UltB
2875:0.04036)0.939.83:0.05294)0.915.97:0.04128)0.832.70:0.0139)0.738.37:0
.01929)0.876.81:0.0188,((ThhAlkal:0.01888,ThhHalop:0.03479)0.998.110:0.06
675,(UltChro7:5.4E-
4,(UltB2756:0.03577,UltB3006:0.04494)0.477.8:0.0076)0.710.24:0.00632)0.22
8.10:0.0118)0.965.71:5.4E-
4)0.935.93:0.01968,(UltB1121:0.02082,((DasMarin:0.028,((UltGa140:0.00421,
UltMar24:0.08423)0.821.58:0.03104,UltGa141:0.02247)0.954.80:5.1E-
4)0.777.93:0.02273,MaiGamm6:0.00867)0.965.72:0.03616)0.983.55:0.03859)0.8
92.98:5.4E-4,((((((MtgSulfi:0.0111,((MtgMari2:0.0,MtgThala:0.0):5.5E-
4,(MtgMarin:0.0,MtgAmini:0.0):5.5E-4)0.908.111:5.5E-
4)0.770.87:0.0037,((MtgMurat:0.01504,UltB2738:5.5E-
4)0.868.83:0.00737,((UltB2737:0.0,MtgAlcal:0.0):5.3E-
4,(UltMeth5:0.01919,UltGa239:0.00761)0.772.79:0.00353)0.942.89:0.01561)0.
876.82:0.00765)0.830.69:0.00692,(((UltGa217:5.3E-
4,(((UltBa795:0.0,UltB2744:0.0):5.5E-4,UltB2745:0.00367)0.994.88:5.4E-
4,UltB2743:0.02686)0.838.86:0.00367)1.000.679:5.4E-
4,(UltB2742:0.00216,(UltB2741:0.00369,UltB2746:5.5E-

4)0.936.96:0.00201)0.907.96:0.00687)0.857.99:0.01606,UltB2740:0.03108)0.3
97.13:0.004)0.990.80:5.4E-
4,(UltB2739:0.07623,((UltGa215:0.06004,(ZooGang2:5.5E-
4,ZooGangh:0.00368)0.951.87:0.03244)0.953.71:0.0411,(UltGam64:0.03758,Ult
Ga100:0.10149)0.701.21:0.00898)0.948.101:0.03941)0.298.7:0.01085)0.913.11
2:0.02607,((UltB2986:0.02453,LeuMucor:0.02623)0.596.6:0.01115,(((((ThxNiv
ea:0.02325,(UltThio2:5.5E-
4,UltB3002:0.0074)0.973.63:0.02347)0.933.104:0.01576,ThxDisci:0.01132)0.8
47.107:0.00741,ThxI0000:0.00386)0.906.108:0.01737,(ThxFlexi:0.02692,((Ult
B3003:0.07796,(UltB3000:0.00369,UltB3001:0.00767)0.942.90:5.5E-
4)0.982.57:0.02696,UltGa280:0.0242)0.903.90:0.01267)0.742.41:0.00691)0.99
3.68:0.04463,(UltB3004:0.00708,UltB3005:0.05997)0.910.98:0.01848)0.557.10
:0.00975)0.974.53:0.03257)0.830.70:0.01169,((UltB2750:0.00801,(CycPuget:0
.01532,UltB2749:0.00372)0.944.73:0.01914)0.787.68:0.00791,(UltBa783:0.061
88,MlcBacte:0.01597)0.866.91:0.0292)0.831.58:0.01167)0.870.80:0.01136,(Ul
tB2968:0.03802,((((PisSalmo:0.07477,(UltBa852:0.13617,(((BggSpeci:0.05022
,((UltGa234:0.07722,(((UltB2793:0.0652,(UltB2794:0.14829,UltDelta:0.08106
)0.813.62:0.05854)0.487.16:0.02234,UltB2814:0.09543)0.543.12:0.01419,((((
(LgnGeest:0.04137,((((((((UltB2783:7.6E-
4,(UltB2782:0.009,(UltB2780:0.0038,UltB2781:0.00855)0.966.97:0.02186)0.93
7.96:0.01785)0.996.85:0.04506,(UltGa221:0.1037,UltB2771:0.03437)0.772.80:
0.01126)0.839.78:0.01085,((Ult13849:0.01016,(TtlMacea:0.00942,(LgnIsrae:0
.01146,((LgnWalte:0.01543,UltLegi3:0.01922)0.880.108:0.01903,UltB2770:5.4
E-
4)0.000.664:0.01225)0.775.78:0.01225)0.874.120:0.01453)0.694.17:0.00963,(
LgnOakri:0.044,UltB2775:0.02135)0.828.55:0.0106)0.747.54:0.00413)0.742.42
:0.00566,UltB2776:0.02693)0.881.108:0.01026,(((((Ult27553:0.05692,(LgnGre
si:5.5E-
4,LgnBusan:0.00746)0.769.79:0.00393)0.249.15:0.00367,LgnBelia:0.00765)0.8
62.99:0.01069,LgnLansi:0.03944)0.911.118:5.3E-
4,(TtlMicda:0.01565,UltB2777:0.01528)0.741.51:0.00375)0.861.101:0.00755,U
ltB2772:0.01556)0.751.47:0.00389)0.885.90:0.00744,(((UltB2768:0.01931,(((
(((((LgnGrati:0.00373,(FluBoze2:5.5E-4,(FluBozem:0.0,LgnSanti:0.0):5.5E-
4)0.698.20:5.5E-
4)0.908.112:0.00355,((UltB1384:0.01123,(LgnWadsw:0.00375,(LgnSteig:5.5E-
4,FluGorma:5.5E-4)0.803.54:0.00373)0.792.85:0.00373)0.998.111:5.5E-
4,(LgnCherr:0.0,LgnParis:0.0,LgnAnisa:0.0,LgnSaint:0.0,LgnLongb:0.0):5.5E
-4)1.000.680:5.4E-
4)0.897.95:0.00756,((LgnDranc:0.00459,(LgnTucso:0.00359,UltB2761:0.00766)
0.809.68:0.00995)0.890.99:0.00437,(LgnYabuu:0.01174,(LgnPneum:0.00328,Lgn
Speci:0.02771)0.868.84:0.00788)0.940.95:0.01502)0.756.74:0.00387)0.790.72
:0.00357,UltLegi2:0.0114)0.807.53:0.00397,(LgnFallo:5.4E-
4,(UltB2763:0.00247,(LgnWorsl:5.5E-4,(LgnQuate:0.00372,LgnMorav:5.5E-
4)0.864.93:0.00373)0.924.95:0.00247)0.924.96:0.00248)0.795.75:0.00373)0.8
44.99:5.4E-
4,UltB2764:0.02703)0.931.100:0.01649,(LgnShake:0.01611,(LgnLytic:0.0189,L
glAmoeb:5.5E-
4)0.687.17:0.01257)0.342.10:0.01278)0.768.68:0.00243,UltLegio:0.01917)0.7
56.75:0.00474)0.755.64:0.00374,(LgnDroza:5.4E-
4,(LgnDonal:0.0154,LgnLondi:0.00773)0.445.14:0.01138)0.924.97:0.01117)0.7
64.87:0.00368,UltB2767:0.00753)0.858.90:0.00742)0.871.99:0.0073,(((UltB27
69:5.4E-4,LgnFairf:0.019)0.959.85:0.01115,UltB2773:0.04852)0.872.99:5.4E-
4,((UltB2766:0.00832,UltB2765:0.02246)0.416.19:0.00386,(LgnRubri:0.0037,L
gnTauri:5.5E-

4)0.948.102:0.0148)0.870.81:0.00797)0.775.79:0.00758)0.054.1:5.3E-
4,(LgnBrune:0.01564,((UltB2774:0.03534,LgnQuinl:0.0145)0.750.50:0.00496,(
LgnBirmi:0.01466,(LgnAdela:0.01993,(LgnJames:0.01643,(LgnJorda:0.01947,(P
ahggYy4:0.07839,(UltB2778:0.04532,Ult16634:0.09276)0.825.66:0.01707)0.841
.78:0.0211)0.829.73:0.01453)0.746.55:0.01454)0.834.63:0.01879)0.936.97:5.
4E-
4)0.762.86:0.00337)0.959.86:0.01534)0.764.88:0.00746,(UltXan18:0.01017,Ul
tGa222:0.07771)0.695.16:0.02309)0.808.68:0.0134)0.679.18:0.02766,((UltB27
84:0.09031,UltB2786:0.13584)0.853.73:0.03195,UltB2779:0.0275)0.000.665:0.
01024)0.912.124:0.03708,(UltEpsil:0.05915,(UltB2785:0.10337,UltGa223:0.02
81)0.905.102:0.03213)0.788.71:0.0189)0.727.28:0.02708,(UltB3583:0.07835,(
UltB2816:0.07251,(EubaFro2:5.4E-
4,(RicSymbi:0.00378,(RicGryll:0.002,UltB2815:0.00695)0.897.96:0.00211)0.9
49.91:0.01913)0.991.79:0.08038)0.936.98:0.05525)0.945.78:0.05217)0.784.84
:0.0164,((UltB2813:0.03025,BacEnri8:0.0228)0.889.97:0.02263,((UltGa231:0.
02425,((UltLegi4:5.4E-
4,UltB2811:0.08139)0.992.75:0.05176,UltGa232:0.00591)0.481.12:0.00912)0.8
60.71:0.02265,((UltB2812:0.00458,(UltB2805:0.05343,(UltB2804:0.04488,((((
UltB2803:5.3E-
4,((UltCoxie:0.01766,UltAqui3:0.01385)0.894.98:0.01237,UltB2806:0.01551)0
.800.64:0.00766)0.844.100:0.00393,(UltAquic:0.02921,UltB2807:0.03342)0.85
2.90:0.01268)0.761.65:0.00428,((((UltCoxi2:0.01941,UltCoxi3:0.02831)0.915
.98:0.01592,AqcSipho:0.0038)0.620.10:0.00389,UltAqui2:0.04041)0.883.109:5
.4E-
4,AgltSoil:0.01877)0.858.91:0.01182)0.887.104:0.01258,((AqcLusit:0.05426,
UltB2808:0.03188)0.827.64:0.01954,UltB2802:0.02019)0.037.2:0.0041)0.923.1
34:0.02608)0.808.69:0.01631)0.980.58:0.05046)0.714.25:0.02137,UltB2809:0.
0623)0.399.14:0.01661)0.301.8:0.02173)0.936.99:0.02596)0.718.24:0.01029)0
.279.5:0.01351)0.594.10:0.01733,((UltB2791:0.01994,UltB2792:0.05115)0.131
.6:0.02147,(((UltB2787:0.02514,(UltGa224:0.04489,UltB2788:0.01253)0.892.9
9:0.01941)0.962.92:0.03753,(Ult16428:0.02867,(UltB2789:0.01608,UltB2790:0
.03782)0.833.79:0.01584)0.888.109:0.03036)0.000.666:0.01272,(UltGa225:0.0
154,UltB2798:0.12572)0.254.5:0.0181)0.853.74:0.02235)0.844.101:0.02139)0.
664.11:0.0098)0.710.25:0.02258,((((((BfhgggYy:0.01762,UltCoxi4:0.01703)0.
429.12:0.00612,(Otu00319:0.02512,Otu00597:0.01781)0.811.62:0.00943)0.175.
4:0.00725,EchggYy2:0.00334)0.996.86:0.06217,UltB1958:0.0758)0.870.82:0.01
772,(UltGa230:0.04261,UltB2800:0.08581)0.961.77:0.04136)0.857.100:0.013,(
UltB2801:0.03943,(UltB2759:0.03565,UltGa233:0.07043)0.698.21:0.00783)0.78
7.69:0.01097)0.245.7:0.00909)0.824.59:0.01721,(UltB2795:0.01383,UltB4081:
0.17964)0.989.69:0.07499)0.733.35:0.0103)0.080.4:0.02045)0.710.26:0.00805
,(UltEps36:0.08905,((UltPro40:0.15026,(GntGnemo:0.13522,((AslNidus:0.0667
8,(ArrExcel:0.04966,(GyrAmeri:0.01503,((LacFerna:0.0117,((AisPlant:0.0183
7,PlaRigid:0.09011)0.754.64:0.00469,BrsSchre:5.4E-
4)0.941.80:0.01967)0.868.85:0.00762,((CocTrilo:0.0,MyrFragr:0.0,ChsMulti:
0.0,CinCamph:0.0,HdsArbor:0.0,EupBenne:0.0,AscRubri:0.0,DapMicra:0.0):5.5
E-4,((((OrzSati2:0.00375,Ze0aMays:5.5E-4)0.373.14:5.4E-
4,TasInsip:0.00755)0.908.113:0.00368,((((LinUsita:0.00376,GerHimal:0.0152
5)0.767.69:0.00376,OenBerte:5.5E-
4)0.850.100:0.00376,(((AkeQuina:0.01926,XnrSimpl:0.01547)0.768.69:0.00374
,(NelNucif:5.5E-
4,((CcsMelo2:0.19885,(HnrAfric:0.02639,AcoGrami:0.07928)0.799.77:0.0257)0
.803.55:0.01064,SoaLyco2:0.00397)0.843.70:0.00445)1.000.681:5.4E-
4)0.577.7:5.5E-
4,(PtrCanes:0.0,DctSpeci:0.0,SngCanad:0.0,MimGutta:0.0):5.5E-

4)0.233.8:5.4E-4)1.000.682:5.4E-
4,(AssOffic:0.0,CrlPalma:0.0,TofCalyc:0.0):0.00368)0.194.8:0.00378)1.000.
683:5.4E-
4,(BxsSpeci:0.00376,((HorFlori:0.00374,((AneCalif:0.0,SauCernu:0.0):5.4E-
4,(CnnOdora:0.01511,(AsmCanad:0.01136,TmnMoore:0.0038)0.787.70:0.00757)0.
854.84:5.4E-4)0.859.91:0.00372)0.799.78:5.4E-
4,PmsBoldu:0.0114)0.454.16:0.00372)0.986.69:5.4E-4)0.485.14:5.4E-
4)0.459.10:5.4E-4)0.630.10:5.5E-
4)0.965.73:0.04102)0.988.77:0.05126)0.000.667:5.4E-
4,(HprLucid:0.00725,ChaVulga:0.12894)0.568.12:0.01673)0.854.85:0.06434)0.
999.130:0.1962)0.841.79:0.03744,((((((UltB4189:0.07402,LebAcidi:0.01544)
0.931.101:0.0233,(CmbHydro:0.00416,(CmbMedia:0.0037,(CmbProfu:0.00369,Ult
Eps33:5.5E-4)0.431.16:5.5E-
4)0.847.108:0.00712)0.806.53:0.00882)0.951.88:0.02467,(UltPro44:0.02759,(
NtlLitho:0.01558,NtlProfu:0.00357)0.913.113:0.01266)0.717.26:0.00573)0.85
3.75:0.01797,UltB4190:0.01166)0.943.90:0.02566,UltEps34:0.00608)0.982.58:
0.04538,(((((UltB4152:0.00392,((((((UltB4153:0.03595,(UltB4154:5.3E-
4,(UltB4155:0.01651,EpbMetag:0.05963)0.854.86:0.01027)0.447.10:0.00429)0.
783.88:0.00406,UltB4156:0.01526)0.613.15:0.00314,UltEps27:0.00474)0.928.7
7:0.01925,(UltEps25:0.04297,(UltB4150:0.0,UltB4151:0.0):0.00331)0.996.87:
0.04738)0.886.118:0.01595,((UltB4148:0.01876,UltB4147:0.0376)0.383.9:0.00
698,(UltEps23:0.01548,UltEps24:0.01171)0.840.85:0.01046)0.761.66:0.00158)
0.790.73:0.00676,(UltB4149:5.4E-4,(SlvLitho:5.4E-
4,(UltEps18:0.00384,(((UltMar37:5.5E-4,UltEps22:0.00368)0.957.77:5.3E-
4,(((UltB4144:0.0075,UltB9222:0.04811)0.829.74:0.00741,(UltB4142:0.01496,
(((UltB4140:0.0,UltEps20:0.0):5.5E-4,UltB4141:0.00741)1.000.684:5.4E-
4,(UltPro43:5.4E-4,(UltPro42:0.0113,UltB4146:5.4E-
4)0.390.15:0.00744)0.725.30:0.00364)0.804.60:0.0152)0.911.119:5.3E-
4)0.797.63:5.3E-4,(UltEps19:0.00375,(UltB4143:5.5E-
4,UltEps21:0.00369)0.807.54:0.00367)0.904.100:0.00753)0.928.78:0.00743)0.
832.71:0.00742,(UltB4138:5.5E-4,UltB4139:5.5E-4)1.000.685:5.5E-
4)0.777.94:0.00352)0.967.67:0.01515)0.901.102:0.0074)0.752.68:0.00376)0.9
53.72:0.01539)0.772.81:0.00403,(UltEps26:0.0,UltB4157:0.0):0.02732)0.325.
10:0.0068,UltB4145:0.02955)0.985.72:0.04472,(((UltB4092:0.10461,(((((UltE
psi9:0.0,UltB4108:0.0):5.4E-4,((UltB4110:0.0,UltEpsi8:0.0):5.5E-
4,UltB4113:5.5E-
4)0.875.104:0.0037)0.795.76:0.00369,(((UltB4111:0.00368,UltB4107:5.5E-
4)0.992.76:0.02317,UltB4112:0.00354)0.785.81:0.00387,UltB4109:5.5E-
4)0.809.69:0.0037)0.761.67:0.00356,UltEpsi7:0.00383)0.964.85:0.0392,UltB4
114:0.07129)0.354.11:0.01296)0.896.89:0.02196,(UltB4115:0.20223,((((UltB4
100:0.01152,(UltB4103:0.01542,UltB4101:0.00731)0.553.11:0.00757)0.949.92:
0.02203,((UltEpsi3:0.03049,UltB4099:0.00477)0.821.59:0.01007,((SfmDenit:0
.00968,UltB4096:0.00965)0.778.80:0.00331,((UltEpsi4:0.01139,UltB4097:0.01
166)0.875.105:0.01343,(((SfmAutot:0.00753,SfmParal:0.01528)0.749.77:0.003
62,UltB4098:0.02324)0.892.100:0.00762,UltB4104:0.00374)0.789.70:0.00778)0
.904.101:0.01359)0.985.73:0.02749)0.516.6:0.00535)0.927.97:0.02639,UltB40
95:0.11951)0.741.52:0.00335,((UltB4105:0.03975,UltEpsi5:0.01978)0.755.65:
0.00379,(UltB4106:0.05093,UltEpsi6:0.0111)0.999.131:0.07886)0.771.92:0.00
403)0.722.25:0.00394)0.867.99:0.0092)0.817.47:0.01203,((((UltEpsi2:0.1290
9,((((WllSucci:0.06669,(HlbCanad:5.2E-
4,(HlbEquor:0.0037,(HlbTrog4:0.00369,(HlbTrogo:0.0,HlbTrog3:0.0):5.5E-
4)0.397.14:5.5E-
4)0.865.98:0.0037)0.991.80:0.00417)0.982.59:0.00356,(((HlbApode:5.4E-
4,HlbMesoc:0.00746)0.846.101:0.0037,((HlbGanma:0.0,UltHeli2:0.0):5.5E-

4,(UltHelic:0.00741,UltB4090:0.00369)0.698.22:5.5E-4)0.742.43:5.5E-
4)0.855.91:0.00369,(HlbRoden:0.0,UltB4091:0.0):5.4E-
4)0.787.71:0.00372)0.793.74:0.00377,((((HlbPullo:5.5E-
4,(HlbPull2:0.0,HlbPamet:0.0):5.5E-
4)0.911.120:0.00757,HlbMacac:0.02329)0.898.115:5.5E-
4,(HlbPylo3:0.00367,(HlbPylo2:0.00367,(HlbSpe10:0.00368,HlbPylor:0.00739)
0.711.26:5.5E-4)0.608.18:5.5E-
4)0.931.102:0.01089)0.331.9:0.00372,(((HlbTrog2:5.4E-
4,((UltB4088:0.0,UltB4087:0.0,HlbHepat:0.0,Ult17542:0.0):0.01142,HlbSpe12
:0.0076)0.767.70:0.00359)0.916.109:0.01169,((HlbSpec5:0.0114,(HlbBili4:0.
00372,(HlbCani2:5.5E-4,HlbCanis:5.5E-4)0.872.100:5.5E-
4)0.756.76:0.00355)0.740.52:0.00404,(HlbSpec7:0.01755,(HlbSpeci:0.00369,(
HlbCina2:0.00369,(HlbSpec4:0.00364,(HlbCinae:5.4E-
4,(HlbSpec3:0.01911,((HlbBilis:0.0,HlbBili2:0.0,FleRappi:0.0,HlbBili3:0.0
):5.5E-4,HlbSpec2:0.00748)0.732.27:5.5E-
4)0.833.80:0.00361)0.082.9:0.00372)1.000.686:5.2E-4)0.924.98:5.5E-
4)0.948.103:0.02175)0.843.71:0.01161)0.839.79:0.00746)0.958.93:0.01593,((
(((HlbFenne:5.5E-4,HlbSpec8:0.00369)0.948.104:5.5E-
4,((UltB4089:0.00369,HlbAurat:0.00375)0.916.110:0.01189,CanHelic:0.01128)
0.243.8:0.00735)0.799.79:0.00722,(HlbMarmo:0.02028,(HlbMurid:0.00308,((Hl
bTyphl:5.5E-
4,Ult17541:0.015)0.979.74:0.01868,(HlbMasto:0.00607,HlbSpec6:0.00607)0.84
6.102:5.3E-
4)0.930.83:0.01537)0.777.95:0.01042)0.903.91:0.01672)0.912.125:0.0116,((U
ltHeli3:0.02636,(HlbFelis:5.5E-4,(HlbBizzo:5.3E-
4,HlbBacul:0.01119)0.816.63:0.00741)0.782.92:0.00667)0.885.91:0.01461,(Hl
bSpe11:0.00678,(HlbFeli2:5.6E-
4,HlbcSuis:0.03103)0.845.93:0.01257)0.821.60:0.0123)0.734.48:0.00281)0.89
4.99:0.00755,HlbCetor:0.00369)1.000.687:5.4E-
4)0.774.85:0.00337)0.792.86:0.00403)0.847.109:0.00366,(HlbSpec9:5.4E-
4,(HlbWingh:5.5E-4,HlbVulpe:0.02276)0.823.59:0.00368)0.997.95:5.4E-
4)0.956.83:0.03267)0.829.75:0.01803,((UltB4124:0.03616,(((ArbMytil:0.0112
4,ArbHalop:0.0117)0.887.105:0.0107,(ArbSpeci:0.00269,(((UltB4122:5.4E-
4,UltEps14:0.02306)0.802.59:0.00406,(((ArbSpec2:0.0,UltB4125:0.0):5.5E-
4,(UltB4126:0.0076,UltB4127:0.01132)0.861.102:0.00744)0.784.85:0.00566,Ul
tB4136:0.03549)0.262.7:0.00599)0.873.99:0.01184,UltB4137:0.024)0.650.14:5
.4E-
4)0.950.89:0.01978)0.792.87:0.00784,(((((UltArcob:0.04837,(ArbSpec3:0.011
22,((UltB4135:0.02265,(ArbButzl:5.5E-4,UltB4128:0.01496)0.356.12:5.5E-
4)0.885.92:5.4E-
4,(((UltB4131:0.00367,(((UltB4130:0.00371,ArbCryae:0.00744)0.787.72:0.003
7,((((UltAnae3:0.0,UltEps16:0.0):5.5E-
4,ArbCibar:0.00371)0.784.86:0.00373,((ArbSkirr:0.0,UltB4133:0.0):5.5E-
4,UltB4134:0.07406)0.927.98:0.01157)0.782.93:0.00377,ArbSpec4:5.4E-
4)0.854.87:0.00374)0.942.91:5.4E-4,UltB4132:5.5E-4)0.629.16:5.5E-
4)0.662.10:5.3E-
4,PshgggY0:0.00371)0.888.110:0.00783,(UncUncu2:0.00748,UltEps15:5.5E-
4)0.851.92:0.00717)0.411.10:0.00377)0.829.76:0.00745)0.866.92:0.00908)0.1
16.6:0.0043,UltEps17:0.04342)0.800.65:0.01915,UltB4102:0.04388)0.912.126:
5.4E-4,UltB4123:5.3E-
4)0.885.93:0.01237,UltB4129:0.03819)0.883.110:0.0129)0.918.100:0.01595)0.
258.8:0.01119,(UltB4177:0.07049,UltB4094:0.08698)0.514.10:0.0234)0.544.9:
0.00964)0.809.70:0.01686,UltB4093:0.10182)0.886.119:0.02214,((((UltB4185:
0.02244,(((CmplLari:5.5E-

4,((((CmpSpec4:0.00901,CmpSpec6:0.027)0.942.92:0.02285,CmpSpec3:5.4E-
4)0.697.10:5.4E-
4,((UltB4171:0.00367,((CmpHelv2:0.0,CmpHelv3:0.0,UltB4167:0.0,CmpUpsal:0.
0,UltB4172:0.0,CmpJeju2:0.0):5.5E-4,CmpJejun:0.02261)0.983.56:5.4E-
4)0.170.6:0.00371,(((CmpCanad:0.03923,CmpCunic:0.0036)0.839.80:0.00754,Cm
pSpec5:0.01124)0.766.72:5.5E-4,CmpHelve:5.5E-4)0.757.55:5.4E-
4)0.919.129:0.00698)0.401.10:0.00743,((UltB4173:0.03574,(((((CmpHomin:0.0
551,(BcrUreol:0.00727,UltCampy:0.01145)0.961.78:0.02698)0.740.53:0.01856,
((UltB4163:5.5E-4,(CmpSputo:5.5E-
4,UltB4162:0.03048)0.834.64:0.00358)0.993.69:0.05431,UltB4164:0.07112)0.7
33.36:0.00575)0.802.60:0.01033,((CmpShowa:0.0,CmpRectu:0.0,CmpRect2:0.0):
0.00547,CmpGraci:0.00568)0.455.19:0.00809)0.327.12:0.00919,UltB4165:0.018
44)0.576.12:5.3E-4,(UltB4168:0.01858,(UltB4169:0.0037,UltB4170:5.5E-
4)0.876.83:0.00818)0.780.94:0.01104)0.782.94:0.00778)0.956.84:0.01557,((C
mpConci:5.4E-4,(UltB4161:5.5E-
4,(CmpSpeci:0.00369,(CmpMuco2:0.0,CmpMucos:0.0):5.5E-
4)0.867.100:0.00369)0.871.100:0.00369)0.890.100:0.00745,((CmpCurv2:5.5E-
4,CmpCurvu:0.00369)0.864.94:5.4E-
4,(((CmpHyoi2:0.0,CmpHyoi3:0.0,CmpHyoi4:0.0):0.00369,BrpHamps:5.5E-
4)0.905.103:0.00741,((CmpSpec2:0.00369,((CmpFetus:0.0,CmpFetu3:0.0,CmpFet
u4:0.0):5.5E-4,CmpFetu2:5.5E-4)0.893.109:5.4E-
4)0.983.57:0.01518,(CmpHyoin:5.4E-
4,(CmpLanie:0.0,UltB4166:0.0):0.0037)0.835.77:0.00369)0.362.11:5.4E-
4)0.940.96:0.01123)0.440.10:0.00372)0.765.66:0.00383)0.966.98:0.02282)0.9
00.83:0.01156)0.998.112:0.04471,((UltEps30:5.5E-
4,((UltEps29:0.0,Otu00026:0.0):0.01133,(UncUncu3:5.5E-4,Kty00002:5.5E-
4)0.767.71:5.4E-
4)0.932.91:0.0034)0.747.55:0.0046,(UltEps28:0.01972,UltB4181:0.0193)0.781
.74:0.01131)0.956.85:0.02335)0.825.67:0.00914,UltB4182:0.02299)0.979.75:0
.04107)0.864.95:0.02132,((UltB4183:0.04702,(UltB4184:0.0,SllArcac:0.0):0.
01691)0.670.13:0.01577,((((SllHalor:5.4E-
4,UltB4179:0.00745)0.914.112:0.00741,(UltB4180:5.5E-
4,(CmpSpec7:0.00372,UltB8947:0.00369)0.765.67:5.5E-4)0.903.92:5.4E-
4)0.999.132:5.4E-4,(SllCavol:0.01121,(SllDeley:5.4E-
4,(SllBarn2:0.00367,SllBarne:5.5E-4)0.911.121:0.00737)0.974.54:5.3E-
4)0.998.113:0.03977)0.355.13:0.00337,UltB4178:5.5E-
4)0.952.77:0.03704)0.959.87:0.03327)0.957.78:0.02798,(HdmTherm:5.4E-
4,((HdmSpeci:0.00823,(UltB4188:0.0089,(NrtTerga:0.03179,UltEps32:0.06325)
0.643.8:0.01688)0.992.77:0.04422)0.814.49:0.01585,(UltEps31:5.5E-
4,UltB4187:0.00388)0.952.78:0.02731)0.865.99:0.01738)0.913.114:0.02267)0.
796.59:0.01463,((UltB4117:0.03653,((UltB4119:0.0,UltB4121:0.0):0.16224,((
UltEps11:0.02111,Otu00171:0.02875)0.793.75:0.01935,((Tryyy091:0.0433,(Ult
Eps10:0.01128,UltPro41:0.01541)0.743.58:0.00423)0.877.115:0.01217,(UltB41
16:5.4E-4,(UltEps12:0.00367,(UltEps13:0.00746,Otu00086:5.5E-
4)0.905.104:0.01141)0.989.70:0.02723)0.941.81:0.02234)0.748.64:0.03724)0.
617.9:0.02961)0.918.101:0.04395)0.868.86:0.06426,UltB4118:0.07958)0.995.9
0:0.12201)0.720.25:0.00696)0.853.76:0.01391)0.919.130:0.0224)0.832.72:0.0
1856,((UltB4160:0.01137,((UltB4176:0.05351,UltB4186:0.04839)0.966.99:0.04
696,(UltB4175:0.02789,UltB4174:0.0204)0.926.111:0.02778)0.952.79:0.03324)
0.823.60:0.02453,(NtfSalsu:0.04642,(UltB4158:0.00472,(UltB4159:0.04083,Ul
tEps35:0.03643)0.982.60:0.03383)0.817.48:0.01724)0.896.90:0.02608)0.926.1
12:0.0379)0.972.77:0.04438)0.909.87:0.0281,(TrcMican:0.04903,UltB4191:0.0
3135)0.759.85:0.01257)0.981.72:0.07036)0.927.99:0.04552)0.909.88:0.05178)
0.822.55:0.01716,((((UltGa235:0.10268,((UltB2845:0.11623,UltRick2:0.18564

)0.780.95:0.02679,(((UltBa841:0.00931,UltMar13:0.01794)0.983.58:0.04896,(
((UltBa826:0.00378,(UltMar12:0.00746,(UltBa827:5.5E-
4,((UltGam85:0.0,UltBa825:0.0,SarClust:0.0,UltGam86:0.0,UltBa828:0.0):5.5
E-4,UltBa824:0.00369)0.737.55:5.5E-4)0.906.109:5.4E-
4)0.854.88:0.0074)0.964.86:0.02584,((UltBa823:0.00501,UltBa834:0.00665)0.
887.106:0.01298,((UltBa836:0.01525,UltBa837:0.00357)0.958.94:0.01855,(Ult
Gam87:5.5E-4,(UltBa831:5.4E-
4,(UltBa829:0.00369,((UltGam88:0.0,UltBa830:0.0,UltGam89:0.0):5.5E-
4,UltBa832:0.00369)0.101.2:5.5E-
4)0.856.84:0.00368)0.927.100:0.01124)0.854.89:0.00946)0.897.97:0.01664)0.
846.103:0.00876)0.881.109:0.02017,(UltBa833:5.5E-4,(UltBa840:5.4E-
4,(UltBa838:5.4E-
4,UltBa839:0.01522)0.981.73:0.01516)0.998.114:0.03088)0.991.81:0.06594)0.
948.105:0.03801)0.892.101:0.03301,(UltMar14:0.0148,UltMar15:0.02508)0.875
.106:0.02615)0.894.100:0.02566)0.748.65:0.01655)0.187.6:0.00109,(UltBa835
:0.04238,(((UltB3792:0.01578,((UltB3777:0.00781,(UltB3793:0.00721,(UltB37
73:0.00342,(UltB3772:0.0037,((UltB3774:5.5E-4,((UltB3769:5.4E-
4,UltB3770:0.00362)0.143.8:0.0037,(UltB3771:0.00371,UltB3776:0.00371)0.83
2.73:0.00362)1.000.688:5.5E-4)0.803.56:5.5E-
4,UltB3775:0.0037)0.854.90:0.00371)1.000.689:5.4E-
4)0.390.16:0.00375)0.769.80:0.00362)0.381.17:0.00373,(UltB3794:0.01128,Ul
tB3779:5.4E-4)0.853.77:5.4E-4)0.891.87:0.00972)0.325.11:5.5E-
4,((((((UltB3795:0.02291,(UltB3804:0.00368,(UltB3808:5.5E-
4,(UltB3807:0.00369,UltB3753:0.00372)0.765.68:5.5E-4)1.000.690:5.5E-
4)0.802.61:0.00742)0.970.73:5.4E-
4,UltAl117:0.00366)0.937.97:0.01128,(UltB3760:0.00367,((UltB3798:5.5E-
4,UltB3805:0.00369)0.792.88:5.4E-
4,(((UltAl114:0.0,UltB3800:0.0,UltB3801:0.0):5.5E-
4,(UltAl116:0.00361,(UltB3797:5.5E-
4,UltB3802:0.00361)0.617.10:0.00744)1.000.691:5.4E-4)0.707.18:5.5E-
4,UltB3743:0.00369)0.873.100:0.00369)0.876.84:0.00753)0.890.101:0.00759)0
.764.89:0.0038,(UltB3796:5.5E-
4,UltAl115:0.01501)0.321.8:0.00368)0.857.101:0.00812,((UltB3781:5.4E-
4,(UltB3784:0.00371,((UltB3778:0.00745,UltB3780:0.01126)0.888.111:5.3E-
4,((((UltB3782:0.0037,(((UltB3790:5.5E-4,(UltB3788:0.00369,UltB3789:5.5E-
4)0.869.82:0.0037)0.928.79:0.00246,UltB3791:0.00245)0.928.80:0.00245,UltB
3787:0.00373)0.903.93:0.00751)0.831.59:5.5E-4,UltB3783:5.5E-
4)0.770.88:5.4E-
4,UltB3786:0.01508)0.772.82:0.00371,UltB3768:0.01915)0.788.72:0.00369)0.7
67.72:0.00371)0.782.95:0.0037)0.772.83:0.00365,UltB3785:0.00378)0.905.105
:0.01072)0.863.109:0.00751,(UltAl113:0.01466,((((UltB3761:5.5E-
4,(UltB3763:0.00371,UltB3764:0.00369)0.744.54:5.5E-4)0.847.110:5.5E-
4,(UltB3766:0.00369,UltB3765:0.00746)0.803.57:0.00367)0.919.131:0.01119,(
UltB3731:5.5E-4,(UltB3762:0.00743,((UltB3738:0.00737,UltB3767:5.3E-
4)0.201.10:0.00369,((((UltB3749:0.0,UltB3755:0.0):0.0037,UltB3734:5.4E-
4)0.859.92:0.00368,((UltB3750:0.0,UltB3739:0.0):5.5E-
4,((UltB3730:0.00371,(UltB3735:0.0,UltB3754:0.0,UltB3806:0.0):5.5E-
4)0.835.78:0.00369,((UltB3732:0.0,UltB3737:0.0):5.5E-
4,UltB3733:0.019)0.843.72:0.00369)0.698.23:5.5E-4)0.893.110:5.5E-
4)0.330.9:5.4E-
4,((UltB3748:0.0,UltB3736:0.0,UltB3740:0.0,UltB3742:0.0,UltB3799:0.0):0.0
037,(((UltB3758:5.5E-
4,((UltB3741:0.0,UltAl111:0.0,UltMar34:0.0,UltB3745:0.0,UltB3756:0.0,UltB
3757:0.0,UltB3759:0.0):5.5E-

4,(UltAl112:0.00369,UltMar33:0.02283)1.000.692:5.5E-4)0.730.35:5.5E-
4)0.907.97:0.00741,UltB3803:0.01496)0.747.56:5.5E-
4,(UltB3747:0.0,UltAl110:0.0,UltB3746:0.0,UltB3751:0.0,UltB3752:0.0):5.5E
-4)0.823.61:5.5E-4)0.948.106:0.00752)0.821.61:0.00364)1.000.693:5.5E-
4)1.000.694:5.4E-
4)0.957.79:0.00314)0.984.63:0.01915,UltMar35:0.01544)0.493.12:5.3E-
4)0.943.91:0.01127)0.867.101:0.00779)0.819.61:0.01556,((UltB3809:0.00368,
(UltB3810:0.0,UltB3811:0.0):5.5E-
4)0.938.61:0.02695,(UltB3814:0.03423,(UltB3812:0.00323,UltB3813:5.4E-
4)0.970.74:0.04477)0.875.107:0.02647)0.998.115:0.08911)0.993.70:0.0754)0.
984.64:0.06037)0.704.17:0.00749,(Ult13182:0.05343,UltOr227:0.08943)0.999.
133:0.1745)0.966.100:0.05153,((UltMar11:0.00135,((UltBa820:0.00369,(UltGa
m79:0.0,UltGam82:0.0):5.5E-4)0.482.14:5.4E-4,((UltGam81:5.4E-
4,(Ult12914:0.02744,(((UltBa817:0.01516,((UltGam78:0.00374,UltBa819:0.003
72)0.803.58:0.00367,((UltGam74:0.0,UltGam76:0.0,VesSpeci:0.0):5.4E-
4,((((UltGam70:0.03385,(UltGam68:0.02641,UltGam71:0.02679)0.814.50:0.0068
8)0.910.99:5.3E-
4,(((UltGam67:0.03044,UltB9262:0.01425)0.815.64:0.00412,UltBa814:0.00699)
0.930.84:0.00109,(UltGam72:0.0,UltGam73:0.0,UltGam69:0.0,UltSUP05:0.0,Ult
Gam80:0.0):5.5E-4)0.520.8:5.4E-4)0.894.101:0.00734,(EnmbnO19:5.4E-
4,(ThiBacte:5.4E-4,EnmbnO21:0.01499)0.886.120:0.00725)0.839.81:5.4E-
4)0.499.7:0.00742,UltBa816:0.00365)0.958.95:5.4E-4)0.872.101:5.5E-
4)0.865.100:0.00739)0.702.16:5.5E-4,((UltMar10:0.0037,(CalFaust:5.5E-
4,EnmbnO20:0.00372)0.809.71:0.0037)0.689.16:5.5E-4,UltBa818:5.5E-
4)0.538.9:5.4E-
4)0.975.69:0.00154,CalMagni:0.01493)0.929.88:0.002)0.980.59:0.02361)0.698
.24:0.00368,UltBa821:0.00369)0.793.76:0.00369)0.835.79:0.00661)0.813.63:0
.00761,((((UltB2831:0.01567,(UltB2832:0.02692,UltGa240:5.4E-
4)0.955.71:0.02294)0.995.91:0.05032,(UltBa822:0.10816,(((UltGam84:0.08523
,(UltB2470:0.0853,UltB2733:0.06001)0.877.116:0.02522)0.949.93:0.04908,(Ul
tGa273:0.00916,(UltGa274:0.00568,UltB2985:0.04353)0.995.92:0.06606)0.643.
9:0.0109)0.799.80:0.01119,UltB2984:0.0411)0.970.75:0.03002)0.663.15:5.4E-
4)0.893.111:0.00869,(((((UltB1069:0.03283,(MxlMorax:5.5E-
4,UltB1068:0.00763)0.836.66:0.01127)0.999.134:0.06019,((((UltB1038:0.0103
6,(UltB1035:0.04494,(UltB1027:0.00869,UltB1037:0.0025)0.768.70:0.01001)0.
757.56:0.00525)1.000.695:0.04112,((((((MxlCavia:5.5E-
4,(((MxlBovis:0.0,MxlMora2:0.0):5.5E-
4,(UltMora4:0.0075,(MxlBovoc:0.0,MxlCapra:0.0,MxlLacun:0.0):5.5E-
4)0.900.84:0.00355)0.463.11:0.00754,(MxlCunic:5.4E-
4,((UltB1074:0.0037,MxlMora3:5.5E-4)0.908.114:0.00749,(((MxlCanis:5.5E-
4,MxlSpec2:5.5E-4)0.840.86:0.00386,UltB1072:0.00369)0.282.7:5.5E-
4,MxlPlura:5.5E-4)0.999.135:5.5E-
4)0.422.15:0.00774)0.952.80:0.01584)0.756.77:0.00402)0.963.72:0.01214,Mxl
Branh:0.01562)0.944.74:5.4E-
4,UltMora5:0.01489)0.799.81:0.00786,(((UltB1023:0.00369,(UltB1016:0.00301
,UltB2555:0.04894)0.949.94:0.02018)0.486.12:0.00397,(((UltB1075:0.01502,(
UltB1025:5.5E-4,(UltB1026:0.00369,UltB1034:0.0037)0.680.17:5.5E-
4)0.861.103:5.3E-
4)0.908.115:0.00341,((UltB1017:0.0,UltB1018:0.0):0.01487,(UltB1051:0.0,Ul
tB1052:0.0):0.00773)0.645.12:0.00751)1.000.696:5.3E-
4,(UltB1019:0.00369,((UltB1029:0.0,UltB1031:0.0):5.5E-
4,((UltB1030:0.00743,((UltB1028:0.0,UltB1032:0.0):0.0013,UltB1024:0.06076
)0.995.93:0.00257)0.525.10:5.5E-4,UltB1033:0.00369)0.412.9:5.5E-
4)0.140.4:5.5E-4)0.126.8:5.4E-

4)0.789.71:0.00386)0.987.94:0.02848,UltB1021:0.01188)0.458.24:0.00433)0.8
39.82:0.01123,((((MxlBoevr:0.02798,(UltB1070:0.0162,(UltBa928:0.03024,(((
MrxBact2:5.5E-4,((UltEnhy2:5.5E-
4,((UltB1063:0.01486,UltB1065:0.01108)0.774.86:0.0037,(UltB1064:0.01135,U
ltB1066:0.01106)0.272.10:5.4E-4)0.555.8:5.5E-4)0.000.668:5.5E-
4,UltEnhyd:5.5E-4)0.614.8:5.5E-4)0.583.9:5.5E-
4,MxlSpeci:0.00366)0.349.5:5.5E-4,UltB1067:0.00365)0.657.12:5.5E-
4)0.956.86:0.02692)0.984.65:0.02934)0.875.108:0.01155,UltB1073:0.02346)0.
910.100:0.01535,(PsbMenin:0.01539,((PsbCeler:0.03534,(UltPsych:5.5E-
4,((((PsbSpec2:0.0,PsbSpeci:0.0,UltB1043:0.0,PsbPisci:0.0,PsbPacif:0.0):5
.5E-4,PsbSpec8:0.0902)0.897.98:0.00738,UltB1059:5.4E-
4)0.914.113:0.00741,PsbFulvi:0.00371)0.677.15:5.4E-
4)0.822.56:0.00373)0.853.78:5.4E-
4,(((((PsbSpec7:0.01116,((PsbSpec3:0.0,PsbSpec4:0.0,UltB1047:0.0,PsbSpec6
:0.0):5.5E-4,UltB1071:0.00369)0.757.57:5.5E-
4)0.922.141:0.01148,(((UltB1048:0.0,PsbSpec5:0.0,PsbCryoh:0.0,AntBact3:0.
0,PsbFrigi:0.0):5.5E-4,(UltGa111:5.5E-4,AntBact2:5.5E-4)0.764.90:5.3E-
4)0.727.29:5.5E-
4,PsbImmob:0.00369)0.788.73:0.00367)0.939.84:0.016,PsbPheny:0.01119)0.800
.66:0.01134,((PsbSpe12:0.0305,((UltB1056:0.07327,UltPro10:0.01943)0.739.4
1:0.00341,(UltB1057:5.5E-
4,((PsbSpe13:0.0,PsbPulmo:0.0,UltB1058:0.0,PsbSpe14:0.0):5.5E-
4,(GmmPro28:0.01615,PsbPulm2:0.00369)0.449.24:5.5E-4)0.000.669:5.5E-
4)0.591.14:5.5E-4)0.014.7:5.3E-
4)0.904.102:0.00791,(((PsbMarin:0.0037,(PsbMari3:0.00368,((UltGa112:0.018
81,PsbJeotg:5.5E-
4)0.904.103:0.00749,((UltB1053:0.00373,(PsbSalsu:0.00369,(PsbMarit:5.5E-
4,(UltGa110:0.0,PsbSpe10:0.0,PsbMari2:0.0,PsbPsych:0.0,PsbHalop:0.0,UltB1
054:0.0,UltB1055:0.0):5.5E-4)0.344.12:5.5E-4)0.205.8:5.5E-
4)0.325.12:5.5E-4,PsbSpec9:0.00747)0.462.14:5.5E-4)0.201.11:5.5E-
4)0.558.9:5.5E-4)0.829.77:0.00815,PsbSpe11:0.17148)0.963.73:5.4E-
4,((UltB1039:0.01118,PsbAlim2:5.5E-
4)0.811.63:0.00368,(PchMarin:0.00369,(UltB1045:5.5E-
4,(UltB1046:0.00369,(UltB1042:0.00741,UltB1044:0.00369)0.001.6:5.5E-
4)0.229.9:5.5E-4)0.705.19:5.3E-
4)0.793.77:0.00373)0.771.93:0.00373)0.812.58:5.4E-4)0.875.109:5.5E-
4)0.826.60:0.00729,(((PsbAlime:0.00744,PsbSpe15:5.4E-
4)0.905.106:0.0075,(UltB1060:0.01953,UltB1061:0.01194)0.755.66:0.00314)0.
919.132:5.4E-
4,(PsbLutip:0.00361,UltB1062:0.03553)0.866.93:0.00745)0.842.94:0.0075)0.9
03.94:0.01098)0.045.6:0.00367)0.898.116:0.00793)0.799.82:5.3E-
4,(MxlCuni2:0.00351,MxlOblon:0.04035)0.514.11:0.00775)0.960.89:0.01662)0.
873.101:0.00875,MxlLinco:0.00833)0.280.5:5.5E-
4)0.813.64:0.00967,(UltB1020:0.00374,((UltB1014:0.0,UltB1015:0.0,UltB1040
:0.0):5.5E-4,(UltB1013:0.00369,UltB1012:0.02294)0.893.112:5.4E-
4)0.814.51:0.00372)0.861.104:0.01022)0.857.102:0.01029,Udntfded:0.03437)0
.217.10:0.00659)0.938.62:0.03068,(UltB1081:0.02004,(((UltB1078:0.0,UltB10
80:0.0):5.5E-
4,UltB1079:0.0074)0.845.94:0.0086,(UltB1076:0.0187,(UltB1036:0.00369,UltB
1077:0.0037)0.984.66:5.4E-
4)0.898.117:0.01412)0.952.81:0.02971)0.788.74:0.0126)0.857.103:0.02299,((
(UltBa972:0.0038,((UltBa888:0.0,UltBa980:0.0):0.00369,(UltBa927:0.00369,(
((((((AciUrsin:0.0,UltGa106:0.0,AciSpe13:0.0):0.01127,UltBa905:0.01517)0.
690.19:5.4E-4,((AciSpec4:5.3E-

4,((((AciSpe12:0.0,UltBa901:0.0,UltAcin4:0.0,UltBa930:0.0):5.5E-
4,(UltBa993:0.00369,((UltBa990:0.00373,(AciTown2:5.5E-
4,(AciSpe45:0.0,AciSpe46:0.0,UltBa992:0.0):5.5E-4)0.903.95:5.5E-
4)0.858.92:5.5E-4,UltBa991:0.01129)0.840.87:0.00369)0.734.49:5.5E-
4)1.000.697:5.3E-4,(AciTowne:5.5E-4,((AciCal11:5.5E-
4,((AciLwof2:0.00368,((((UltBa916:0.0,AciSpe29:0.0,UltBa947:0.0,AciSpe30:
0.0,AciLwof3:0.0,AciLwof4:0.0,AciSpe31:0.0,UltBa948:0.0,AciSpe32:0.0,AciL
wof5:0.0,UltBa977:0.0,UltBa994:0.0,UltBa995:0.0,UltBa996:0.0,AciSpe47:0.0
,AciLwof7:0.0):5.5E-
4,(UltBa978:0.0074,(UltBa997:0.00742,UltBa914:0.01117)0.402.10:5.5E-
4)0.916.111:5.5E-4)0.000.670:5.5E-
4,(UltAci36:0.00751,UltAci14:0.00369)0.939.85:5.5E-4)0.000.671:5.5E-
4,((UltBa917:0.00369,(UltB1010:0.01869,UltBa952:0.00716)0.919.133:5.4E-
4)0.766.73:5.5E-4,AciLwof6:5.5E-4)0.000.672:5.5E-4)0.867.102:5.5E-
4)0.988.78:5.0E-4,AciLwoff:0.02225)0.852.91:0.00361)1.000.698:5.4E-
4,((AciSpe11:0.0,UltBa899:0.0):5.5E-
4,UltBa900:0.00369)0.846.104:0.00362)0.205.9:0.00362)0.747.57:0.0035)0.88
0.109:0.00726,((ProSymb5:5.5E-4,UltBa999:0.00753)0.888.112:5.5E-
4,(UltAci15:0.00745,(((UltBa949:0.0,UltBa950:0.0,UltBa951:0.0):5.5E-
4,(AciSpe33:0.00746,UltB1001:0.00741)0.791.65:5.5E-4)0.835.80:5.5E-
4,UltBa953:0.01908)0.840.88:5.4E-
4)0.502.7:0.0037)0.931.103:0.00743)0.879.105:5.4E-
4)0.915.99:0.00726,((UltBa976:0.01495,(((((AciVene2:0.0,AciVenet:0.0,AciS
pe14:0.0):5.5E-
4,(UltAcin5:0.00746,(UltBa903:0.00368,AciCal12:0.02663)1.000.699:5.5E-
4)0.743.59:5.5E-4)0.793.78:5.4E-
4,((UltBa896:0.04077,UltB1000:0.01273)0.709.18:0.00238,AciCal10:0.00376)0
.817.49:0.00374)0.917.94:5.3E-
4,AciXiame:0.027)0.868.87:0.00725,(UltGa105:5.5E-
4,((AciRadi2:0.0,UltBa895:0.0,AciRadi3:0.0,UltBa897:0.0,AciRadi4:0.0):5.5
E-4,AciRadi5:0.00369)0.745.64:5.5E-4)0.951.89:5.4E-
4)0.922.142:0.00746)0.861.105:5.4E-
4,((AciSpeci:0.00375,(AciCalc8:0.06361,((((AciBayl4:0.0,AcinSoli:0.0,Acin
Sol2:0.0,AcinSol3:0.0):5.5E-4,((AciSpec5:0.00367,(AciSpec6:5.5E-
4,(AciBayl2:0.0,AciBayl3:0.0):5.5E-4)0.901.103:5.5E-4)0.794.64:5.5E-
4,AciBayly:0.01123)0.840.89:0.00368)0.941.82:0.00747,AciSpec3:0.00741)0.7
98.67:5.5E-4,(UltGa104:5.5E-
4,(AciCalc5:0.0,AciCalc2:0.0,AciCalco:0.0,AciCalc3:0.0,AciCalc4:0.0,AciSp
ec2:0.0,UltAcine:0.0,AciCalc6:0.0,AciCalc7:0.0,AciGenom:0.0,AciCalc9:0.0,
UltAcin2:0.0):5.5E-4)0.891.88:5.4E-4)0.996.88:5.5E-
4)0.817.50:0.00484)0.887.107:0.01,UltBa891:0.00373)0.773.87:0.00365)0.891
.89:5.4E-
4)0.936.100:0.00722)0.890.102:0.00663,((((((AciSpec8:0.03494,UltBa894:0.0
1114)0.162.6:5.5E-4,AciCal13:0.00369)0.000.673:5.5E-4,(((AciSpe21:5.5E-
4,((UltBa925:0.0,UltGa108:0.0,AciParvu:0.0,AciSpe22:0.0,AciSpe23:0.0,UltB
a940:0.0,AciTjer2:0.0,AciTjern:0.0,UltBa944:0.0,AciHaemo:0.0,AciSpe34:0.0
,AciSpe35:0.0,UltAci16:0.0,UltAci17:0.0,AciSpe44:0.0,AciCal16:0.0):5.5E-
4,((AciTjer3:0.0,AciTjer4:0.0):5.5E-4,(AciHaem3:0.00407,AciJuni5:5.5E-
4)0.662.11:5.5E-4)0.000.674:5.5E-4)0.196.10:5.5E-4)0.238.9:5.4E-
4,(AciTando:5.4E-
4,UltAci12:0.02285)0.847.111:0.00368)0.887.108:0.00879,(UdnBact3:0.01525,
UltBa989:5.5E-4)0.776.83:0.00377)0.888.113:0.00883)0.819.62:5.5E-
4,AciBauma:5.5E-4)0.458.25:5.3E-
4,(AciBaum3:0.0,UltBa909:0.0,UltBa912:0.0):5.5E-4)0.563.9:5.4E-

4,AciBaum2:0.01496)0.830.71:0.00298)0.809.72:0.00263,(UltBa904:0.01946,UltBa981:0.00869)0.765.69:0.00394)0.884.105:5.5E-4,UltBa889:0.00737)0.243.9:5.5E-4,UltBa971:0.00366)0.000.675:5.5E-4)0.000.676:5.5E-4)0.789.72:5.5E-4)0.371.12:5.5E-4,(UltBa926:0.00735,((((AciJuni2:0.01492,UltBa964:0.00696)0.660.14:5.4E-4,UltBa906:0.00369)0.739.42:5.4E-4,((UltMorax:0.00364,(UltAci10:5.5E-4,AciGuil2:0.0021)0.618.6:0.00104)1.000.700:5.3E-4,((((AciSpe19:5.5E-4,(((((AciSpe27:5.5E-4,(AciCal15:0.0037,AerPunct:0.00743)0.819.63:5.5E-4)0.567.7:5.5E-4,(((AciJuni7:0.01895,UltBa913:0.00721)0.916.112:5.4E-4,(AciSpe10:0.0046,UltB1083:0.04008)0.907.98:0.01476)0.792.89:5.3E-4,UltBa933:5.4E-4)0.831.60:0.00369)0.867.103:0.00369,(((UltAci35:0.00371,UltAcin7:0.00378)0.000.677:5.5E-4,((UltBa967:5.5E-4,((UltAci32:5.5E-4,AciJuni3:0.0872)0.818.46:0.00368,(UltAcin3:0.00368,(UltBa910:0.00369,((((((UltB1006:0.00368,AciSpe36:0.03191)0.500.13:5.5E-4,UltBa911:0.00369)0.000.678:5.5E-4,UltMora3:0.00369)0.234.7:5.5E-4,(UltBa941:5.5E-4,((UltAci29:5.5E-4,((((((UltBa960:0.00368,((UltBa923:5.5E-4,((((((UltAcin8:0.00369,((((UltGa109:5.5E-4,(((UltBa965:0.01981,(UltAci20:0.01503,UltAci26:0.02721)0.737.56:0.00371)0.996.89:0.00332,UltAci23:5.5E-4)1.000.701:5.1E-4,UltBa956:0.00774)0.246.15:5.5E-4)0.417.15:5.4E-4,UltBa962:0.00372)0.000.679:5.5E-4,Ult24937:0.0037)0.464.11:5.5E-4,(UltGa107:0.0,UltBa924:0.0,UltBa935:0.0,GmmPro27:0.0,UltBa958:0.0,UltBa959:0.0,UltBa961:0.0,UltAci22:0.0,UltBa966:0.0,UltAci33:0.0,AciSpe49:0.0,UltB1003:0.0,UltAci37:0.0):5.5E-4)0.495.11:5.4E-4)0.000.680:5.5E-4,(((UltBa955:5.5E-4,((UltAcin9:5.5E-4,((AciSpe39:5.5E-4,(UltAci27:0.01294,UltAci28:0.02356)0.769.81:0.00637)0.396.20:5.5E-4,(UltMar20:0.0,AciSpe38:0.0,UltAci25:0.0,UltAci24:0.0,UltB1011:0.0):5.5E-4)0.456.12:5.5E-4)0.163.8:5.5E-4,UltBa921:0.00369)0.807.55:0.00396)0.197.10:0.00401,AciSpe26:5.5E-4)0.908.116:0.0027,UltBa920:0.00236)0.908.117:0.0027)0.510.8:5.5E-4,(UltAci30:5.4E-4,(UltBa939:0.00728,AciJohn2:0.01163)0.930.85:5.4E-4)0.897.99:0.00384)0.549.13:5.5E-4,(UltAci21:0.0,UltAci31:0.0):5.5E-4)0.277.12:5.5E-4,UltBa957:0.03908)0.331.10:5.4E-4,UltBa918:0.00369)0.441.14:5.4E-4)0.000.681:5.5E-4,AciJohn3:5.5E-4)0.101.3:5.5E-4)0.108.7:5.3E-4,(UltBa942:0.0428,(UltBa945:0.02272,((BacEnric:0.0074,AciSchin:0.00369)0.639.18:5.5E-4,(UltBa943:0.0,AciSchi2:0.0,AciJohns:0.0,AciSchi3:0.0):5.5E-4)0.723.26:5.5E-4)0.893.113:5.5E-4)0.829.78:0.00364)0.196.11:5.5E-4,(UltBa963:0.02265,UltBa998:0.0037)0.797.64:0.00367)0.130.7:5.5E-4,UltAci18:0.00371)0.210.14:5.5E-4,((UltBa919:0.0424,UltB1004:0.05042)0.743.60:0.00383,((AciHaem2:0.0,AciBouv2:0.0,UltBa968:0.0,UltBa970:0.0,UltBa986:0.0,UltB1005:0.0,UltB1007:0.0,UltB1009:0.0):5.5E-4,(UltBa969:0.00369,(AciBouve:0.00371,UltBa987:0.00369)0.299.13:5.5E-4)0.000.682:5.5E-4)0.992.78:5.3E-4)0.864.96:0.00359)0.850.101:0.00462,((((UltBa946:0.00358,UltRume7:0.00385)0.416.20:0.00372,AciSpe24:0.01514)0.877.117:5.4E-4,(UltBa922:0.0,AciSpe25:0.0,MrxBacte:0.0):5.5E-4)0.660.15:5.5E-4,UltB1002:0.00373)0.989.71:0.01948)0.673.9:5.5E-4)0.005.3:5.4E-4,AciSpe48:5.5E-4)0.829.79:0.00467)0.918.102:0.0095)0.120.9:5.5E-4,UltBa979:0.00366)0.214.7:5.5E-4,AciSpec9:0.0075)0.000.683:5.5E-

4,AciSpe28:5.5E-4)0.184.4:5.5E-4)0.487.17:5.5E-4)0.000.684:5.5E-
4)0.141.6:5.5E-4)0.000.685:5.5E-4,UltBa988:5.5E-4)0.489.11:5.5E-
4)0.000.686:5.5E-4,(AciGuill:0.00746,(AciGerne:0.0,AciGrimo:0.0):5.5E-
4)0.000.687:5.1E-4)0.493.13:5.5E-4)0.000.688:5.5E-
4,(AciSpec7:0.0,UltBa892:0.0,UltBa893:0.0,UltBa907:0.0,UltBa908:0.0,UltAc
in6:0.0,AciJunii:0.0,UltBa934:0.0,AciSpe15:0.0,AciSpe16:0.0,AciSpe17:0.0,
AciSpe18:0.0,UltBa936:0.0,AciSpe20:0.0,GmmPro26:0.0,UltAci13:0.0,AciJuni4
:0.0,AciSpe37:0.0,AciJuni6:0.0,UltBa982:0.0,UltAci34:0.0,UltBa983:0.0,Aci
Spe40:0.0,UltBa984:0.0,AciSpe41:0.0,UltBa985:0.0,AciSpe43:0.0,AciGuil3:0.
0,AciSpe50:0.0,UltB1008:0.0):5.5E-4)0.281.6:5.5E-
4,AciSpe42:0.01125)0.000.689:5.5E-4)0.143.9:5.5E-4,UltMora2:5.5E-
4)0.149.11:5.5E-4,UltBa954:0.00367)0.343.6:5.5E-
4,(UltBa898:0.00208,UltAci11:0.01472)0.911.122:0.00181)0.022.2:5.5E-
4)0.525.11:5.2E-
4)0.802.62:0.00468,UltBa937:0.00739)0.030.4:0.0045)0.884.106:5.4E-
4)0.754.65:0.00385,(UltBa915:0.07401,(UltBa938:0.04056,(UltBa974:5.4E-
4,UltBa975:0.02358)0.918.103:0.02082)0.444.22:0.00904)0.714.26:0.00495)0.
999.136:0.05198)0.992.79:0.04622,((UltB2799:0.04572,((UltB2796:0.01075,(F
anHongk:0.01762,CdbTaeni:0.03691)0.619.7:0.01129)0.998.116:0.07641,(UltGa
227:5.4E-4,(FrcTular:0.01871,FrcPhilo:5.4E-
4)0.835.81:0.00733)0.944.75:0.02881)0.813.65:0.02167)0.992.80:0.05705,Ult
Gam83:0.01613)0.244.6:0.00351)0.776.84:0.00363)0.876.85:0.01105,(UltB2967
:0.10264,((UltB1022:0.01901,((UltB2568:0.0074,((UltB2574:0.0037,(UltB2571
:0.00369,UltB2572:5.5E-4)0.184.5:5.5E-
4)0.848.80:0.00371,(UltB2589:0.00368,(((UltB2590:0.0037,((UltB2560:0.0,Ul
tB2584:0.0,UltB2593:0.0):5.5E-
4,(UltB2581:0.00757,(UltB2587:0.0074,(UltB2559:5.5E-
4,UltB2578:0.00369)0.494.8:5.5E-4)0.824.60:0.00369)0.559.9:5.5E-
4)0.472.13:5.5E-
4)0.800.67:0.00377,(UltB2561:0.00738,(UltB2579:0.00378,UltB2582:0.01124)0
.796.60:0.00769)0.000.690:5.4E-4)0.998.117:5.5E-4,(((UltB2567:5.4E-
4,(UltB2558:0.0201,UltB2564:0.01525)0.392.9:0.0113)0.792.90:0.00711,(UltB
2566:0.01459,(UltB2562:5.5E-
4,UltB2563:0.00371)0.888.114:0.01048)0.767.73:0.00537)0.796.61:0.00377,Ul
tB2569:5.4E-4)0.856.85:0.00369)0.003.5:5.5E-4)0.000.691:5.5E-
4)0.266.9:5.3E-4)0.664.12:5.4E-4,(((((UltB2556:0.00741,UltB2570:5.5E-
4)0.820.52:0.00366,(UltB2583:0.00369,(UltB2557:5.5E-
4,(UltB2586:0.00378,UltB2577:0.00369)0.317.11:5.5E-4)0.285.9:5.5E-
4)1.000.702:5.4E-
4)0.298.8:0.00378,((UltB2592:0.00756,(UltB2565:0.00737,UltB2573:5.5E-
4)0.947.76:0.01133)0.768.71:0.00364,(UltB2588:0.00369,UltB2591:5.5E-
4)0.416.21:5.5E-4)0.569.11:5.5E-
4)0.929.89:0.00758,(UltB2585:0.01118,UltB2576:5.4E-4)0.733.37:5.4E-
4)0.990.81:0.02374,(UltB2580:5.4E-
4,UltB2575:0.03549)0.466.12:0.00689)0.878.94:5.5E-
4)0.982.61:0.02978)0.943.92:0.03068,((((UltB2595:0.00737,(UdnProte:5.5E-
4,CrdHomin:5.5E-4)0.874.121:5.5E-4)0.875.110:0.01301,(CrdValva:5.5E-
4,UltB2594:0.03081)0.499.8:0.0097)0.806.54:0.02074,(UltB2553:0.06612,(Dcl
Nodos:0.0631,SttOrnit:0.00979)0.321.9:0.00847)0.694.18:0.02077)0.864.97:0
.0247,(UltB2552:0.00368,UltB2554:5.5E-
4)0.970.76:0.03968)0.924.99:0.03235,UltB2551:0.01979)0.776.85:0.01978)0.9
23.135:0.03074)0.819.64:0.02323)0.539.4:0.00588)0.849.86:0.00899)0.899.10
3:0.01501)0.836.67:0.01382,UltGa277:0.02764)0.784.87:0.00986)0.836.68:0.0
1244)0.837.60:0.0075)0.787.73:0.00488,(((MhsFibra:0.02318,MtcTherm:0.0544

)0.757.58:0.008,((((UltMeth9:0.03034,((UltGa270:0.01123,UltB2982:0.01582)
0.973.64:0.02791,(MaoHadal:0.01932,(UltGa272:0.01933,UltGa271:0.00369)0.8
41.80:0.00841)0.703.24:0.00326)0.906.110:0.01274)0.678.17:0.00435,((UltB2
978:0.00376,((UltMeth8:0.0,UltB2977:0.0):5.3E-
4,UltB2983:0.04328)0.708.27:0.00365)0.762.87:0.00694,(MlsDiffi:0.02115,(C
rePolys:0.00766,UltB2976:0.03266)0.267.9:0.02312)0.754.66:0.02234)0.794.6
5:0.00974)0.819.65:0.00972,(UltB2981:0.01465,UltMet10:0.01187)0.849.87:0.
01215)0.756.78:0.00561,(((((UltMet11:0.01603,(((MhcAlcal:0.0,MhcJapan:0.0
,UltMeth7:0.0):0.00883,(MhcBurya:0.00473,MhcKenye:0.01051)0.809.73:0.0101
4)0.788.75:0.01983,((MhcAgile:0.0,MhcAlbum:0.0):0.00762,MhcAgil2:0.01931)
0.816.64:0.01742)0.378.14:0.01008)0.355.14:0.01571,((((MhbMarin:0.00403,(
UltGam77:0.04331,(MhbLuteu:0.00369,UltB2979:5.5E-4)0.866.94:5.4E-
4)0.723.27:0.00705)0.495.12:0.0076,MhbWhitt:0.0188)0.737.57:0.00413,MhsLa
cus:0.00377)0.780.96:0.00479,(UltB2980:0.00714,MlmAuran:0.00949)0.050.7:0
.00597)0.311.7:0.0095)0.755.67:6.8E-
4,(MlmRubra:0.00731,UltGa269:0.00783)0.872.102:0.00742)0.924.100:0.01162,
(MlmMetha:5.5E-4,MlmSpeci:5.5E-
4)0.778.81:0.00396)0.757.59:0.00395,UltB2975:0.02802)0.865.101:0.0109)0.7
75.80:0.00442)0.874.122:0.01484,MhpHanso:0.0126)0.978.60:0.02357)0.857.10
4:0.00985,(((UltBa812:0.00363,UltBa813:5.5E-
4)0.734.50:0.0055,(UltBa807:0.00358,(UltBa806:0.00504,((UltBa804:0.0,UltB
a805:0.0):0.01274,UltBa808:0.02656)0.943.93:0.03268)0.881.110:0.01869)0.9
97.96:0.0626)0.989.72:0.03743,(UltBa810:0.05376,(((HdrtVen2:0.02016,((Thp
Thyas:5.5E-4,(TlmAerop:0.00741,TlmMicro:5.5E-
4)0.843.73:0.00367)0.985.74:0.02956,(UltB2728:0.01752,(ThpChile:0.00752,(
(((UltB2730:0.00716,UltB2729:0.02361)0.864.98:0.00793,(UltB2727:0.01509,(
UltB2726:0.00758,ThpFrisi:0.00376)0.783.89:0.0037)0.446.9:5.5E-
4)0.921.111:0.00743,(ThpArcti:0.0,ThpPsych:0.0):5.5E-4)1.000.703:5.5E-
4,((ThpTherm:0.0092,(UltB2731:0.00592,(ThpHalop:0.01112,(HdvMarin:0.0,Thp
Kuene:0.0):5.4E-
4)0.732.28:0.00915)0.773.88:0.00741)0.930.86:0.01755,UltB2732:0.01924)0.7
38.38:0.00417)0.954.81:0.01964)0.933.105:0.01744)0.868.88:0.01185)0.964.8
7:0.03717)0.952.82:0.02726,UltGa229:0.03966)0.973.65:0.03424,((UltBa811:0
.00538,KngKoree:0.0344)0.206.7:0.01172,(UltGam66:0.01953,GmmPro23:0.06817
)0.931.104:0.03084)0.781.75:0.02373)0.391.13:5.5E-
4)0.796.62:0.01998)0.861.106:0.01412)0.733.38:0.00356,((((((((UltB1134:0.
04319,(UltThala:0.00819,(OcbKrieg:0.0072,(UltBa800:5.5E-
4,UltGam62:0.0037)0.901.104:5.3E-
4)0.907.99:0.0149)0.310.10:0.00693)0.213.8:0.00826,(ThsSpeci:5.4E-
4,UltBa801:0.07498)0.765.70:0.00398)0.890.103:0.01257,(RobSpeci:0.03195,U
ltGam63:0.01937)0.760.74:0.00571)0.847.112:0.00782,(UltB1124:0.03377,UltB
1125:0.06204)0.843.74:0.01113)0.936.101:0.01854,((UltBa863:0.04469,((((Mr
cSpec4:0.0037,((MrcSpec5:0.0,UltGam96:0.0,UltBa856:0.0):5.5E-
4,(MrcSpec3:0.00768,(((MrcMobil:0.0037,((MrcBryoz:0.00368,(UltBa855:0.007
43,(((MrcLitor:0.00744,(MrcMobi2:0.0037,MrcZheji:5.5E-4)0.851.93:5.5E-
4)0.959.88:0.00489,(MrcDaepo:5.4E-4,((MrcKoree:0.00747,(MrcSpe14:5.5E-
4,UltBa862:0.00367)0.922.143:5.5E-
4)0.874.123:0.0082,((MrcLipo2:0.0,UltBa860:0.0,MrcSpe11:0.0):0.01493,(Ult
Ba857:0.01571,(MrcLito2:5.5E-4,(MrcSpe12:5.5E-
4,(MrcMarit:0.00743,MrcSqual:0.00749)0.017.5:5.3E-
4)0.839.83:0.0037)0.754.67:0.00337)0.884.107:0.01072)0.391.14:0.00156)0.6
32.10:0.00771)0.959.89:0.00514)0.959.90:0.00513,(MrcSpe13:5.4E-
4,(MrcHydro:5.5E-4,((MrcSpe15:0.0,RhdBacte:0.0,GmmPro25:0.0):5.5E-
4,(UltGam97:0.00369,UltBa861:0.00369)0.943.94:5.5E-4)0.888.115:5.5E-

4)0.838.87:0.00367)0.426.19:0.00368)0.661.13:5.3E-4)0.437.13:5.5E-
4)0.000.692:5.5E-
4,(MrcGudao:0.0,MrcLipol:0.0,UltBa858:0.0,MrcSpe10:0.0,UltBa859:0.0,MrcGo
seo:0.0):5.5E-4)0.477.9:5.4E-4)0.000.693:5.5E-
4,MrcSalic:0.0037)0.915.100:0.00765,((MrcPelag:5.3E-
4,MrcSzuts:0.01885)0.817.51:0.00365,(MrcSpec6:5.5E-
4,UltMar19:0.0112)0.993.71:5.5E-4)0.585.5:0.00753)0.830.72:5.4E-
4)0.550.7:5.5E-4)0.371.13:5.4E-4)0.461.9:5.5E-
4,(UltGa103:0.03144,(UltMar18:0.00753,(MrcSalsu:5.5E-
4,(MrcSpec7:0.01098,((UltBa854:0.0,MrcSpec8:0.0,MrcSpec9:0.0):5.5E-
4,(UltBa853:0.0,UltArcti:0.0):0.0037)0.720.26:5.5E-
4)0.854.91:0.00364)0.878.95:0.00735)0.858.93:0.00753)0.860.72:0.0074)0.89
9.104:0.00786,(MrcLutao:0.01517,MrcTaiwa:5.5E-
4)0.779.79:0.00366)0.871.101:0.00783,MrcZhanj:0.00375)0.835.82:0.00814)0.
955.72:0.02052,(UltBa877:0.03147,(UltBa876:5.3E-
4,((SlcMaras:0.00369,UltPseu8:5.5E-
4)0.942.93:0.01618,(UltBa875:0.0038,(PsdHalop:0.00369,(PsdSpec2:0.00368,H
lsDenit:0.00369)0.749.78:5.4E-
4)0.968.77:0.01503)0.818.47:0.00651)0.995.94:0.04057)0.878.96:0.01536)0.8
23.62:0.00746)0.773.89:0.00615)0.293.7:0.01098,((((((UltB1389:5.4E-
4,(UltGa203:0.01559,UltrBe14:0.01152)0.918.104:0.01532)0.987.95:0.03067,(
(((UltB1377:0.01966,(PsdSp148:5.4E-4,(PsdSp149:5.5E-
4,UltB1376:0.00742)0.926.113:0.01119)0.133.7:0.00355)0.984.67:0.02447,(Ul
tB1374:5.5E-
4,UltB1375:0.01521)0.819.66:0.00665)0.935.94:0.01574,((((PsdSpe99:5.4E-
4,PsdGuine:0.0037)0.916.113:0.00745,(PsdSp100:0.0037,PsdSp131:5.4E-
4)0.900.85:0.00743)0.480.13:5.5E-4,(((PsdmPeli:5.5E-
4,(UltB1349:0.00369,(PsdAngui:0.0,UltB1309:0.0,PsdSpe98:0.0,PsdAngu2:0.0)
:5.5E-4)0.294.7:5.5E-4)0.930.87:0.00737,(AgrAgile:5.5E-
4,UltB1366:0.0037)0.420.14:5.3E-
4)0.782.96:0.00365,(((UltB1370:0.0037,((PsdDenit:0.0037,(PsdPertu:0.00369
,UltB1369:0.00374)0.913.115:0.00751)0.347.8:5.5E-4,PsdXiame:5.5E-
4)0.881.111:0.0037)0.544.10:5.4E-4,PsdSp157:5.5E-4)0.894.102:5.3E-
4,(UltB1371:0.00705,((((PsdSp142:0.0,AntSeawa:0.0,ArcSeawa:0.0):5.5E-
4,PsdXinji:5.5E-4)0.849.88:5.5E-
4,(PsdSp141:0.00749,PsdSpeci:0.00744)0.636.9:5.5E-4)0.781.76:5.3E-
4,((PsdSp143:0.01494,(PsdSp144:0.00371,PsdSabul:5.5E-4)0.499.9:5.5E-
4)0.927.101:0.00723,UltGa196:0.00684)0.276.10:5.4E-
4)0.767.74:0.0077)0.724.23:0.0118)0.961.79:0.0188)0.876.86:0.0074)0.983.5
9:0.01912,((((UltB1348:0.00369,(PsdSp127:0.00368,(((((((UltB1347:0.01318,
UltPse39:0.00375)0.806.55:0.00436,UltGa193:0.00369)0.473.10:5.5E-
4,UltPse38:5.5E-4)0.126.9:5.5E-
4,(PsdSpe97:0.0,PsdMend2:0.0,PsdSp123:0.0,ElbRive3:0.0,PsdPseu2:0.0,PsdSp
128:0.0,PsdPseu4:0.0,PsdSp129:0.0,UltB1350:0.0,PsdMend3:0.0,PsdSp132:0.0,
UltB1351:0.0,PsdSp133:0.0,PsdPseu5:0.0,PsdPseu6:0.0):5.5E-4)0.349.6:5.5E-
4,PsdNitr5:0.00369)0.347.9:5.5E-4,PsdSp125:5.5E-4)0.078.10:5.5E-
4,PsdSp124:5.5E-4)0.932.92:0.00864)0.000.694:5.4E-4)0.452.14:5.5E-
4,PsdSp126:5.5E-4)0.323.7:5.5E-4,PsdPseu3:0.00369)0.454.17:5.5E-
4,PsdPseud:0.00738)0.596.7:5.4E-4)0.513.7:5.4E-
4)0.907.100:0.01546,((((((PsdSpe11:0.0,PsdSynxa:0.0,PsdPalle:0.0,PsdTola3
:0.0,UltB1275:0.0):5.5E-4,(((UltB1237:0.00745,((PsdFluo8:5.5E-
4,(PsdFluo3:0.00739,((PsdLiba2:5.4E-
4,(PsdLiban:0.01927,PsdLiba3:0.02298)0.846.105:0.00744)0.821.62:0.00374,P
sdFlu11:5.4E-4)0.826.61:0.00371)0.030.5:5.5E-

4)0.847.113:0.0037,((UltB1286:0.00738,(PsdFlu16:5.5E-4,((PsdFluo9:5.5E-
4,(UltGa175:0.0077,((((PsdFluo4:5.5E-
4,((PsdFlu15:0.00814,PsdSpe17:0.00543)0.008.4:5.5E-
4,BacL194W:0.00405)0.463.12:5.5E-4)0.210.15:5.5E-
4,PsdSpe14:0.02268)0.219.6:5.5E-
4,(PsdVeron:0.00245,UltB1279:0.00245)0.919.134:0.00245)0.328.12:5.5E-
4,PsdFlu10:5.5E-4)0.256.6:5.5E-4)0.000.695:5.5E-4)0.000.696:5.5E-
4,(BacL188S:0.0,BacteFK2:0.0,PsdFluo6:0.0,UltPse13:0.0,PsdFluo7:0.0,PsdSp
e19:0.0,PsdFlu12:0.0,UltB1236:0.0,PsdSpe26:0.0,UltB1263:0.0,UltGa183:0.0)
:5.5E-4)0.416.22:5.4E-4)0.056.7:5.5E-4)0.729.24:5.5E-
4,PsdFlu13:0.03567)0.475.13:5.3E-4)0.858.94:0.00458)0.245.8:5.5E-
4,UltB1235:0.00369)0.402.11:5.5E-4,(PsdMucid:5.5E-
4,PsdSpec5:0.01869)0.539.5:5.4E-4)0.312.11:5.5E-4)0.468.14:5.3E-
4,(((((((UltB1280:5.5E-
4,(UltGa181:0.00201,UltB1285:0.01088)0.922.144:0.00198)0.836.69:0.00368,(
((UltB1201:5.3E-
4,(UltB1202:0.03967,UltB1231:0.06044)0.272.11:0.01074)0.886.121:0.00723,(
((PsdTrivi:0.00368,((PsdSpe10:0.0,UltB1222:0.0,UltB1223:0.0,UltB1227:0.0,
PsdSpe16:0.0,UltB1169:0.0,UltB1170:0.0,UltB1180:0.0,UltB1198:0.0,UltB1204
:0.0,UltGa174:0.0,UltPro13:0.0,UltB1230:0.0,PsdSpe18:0.0,PsdReact:0.0,Psd
Spe20:0.0,PsdFlu14:0.0,PsdPuti2:0.0,PsdFlu17:0.0,PsdTola4:0.0,PsdTola5:0.
0,PsdSpe24:0.0,PsdSpe25:0.0,UltB1278:0.0,UltB1284:0.0,UltB2596:0.0):5.5E-
4,((((((UltB1218:0.0564,((UltB1173:0.10566,BacTHCL9:0.07593)0.836.70:0.01
445,UltB1190:0.05987)0.737.58:0.00855)0.311.8:0.00827,(UltB1178:0.05183,(
(UltB1191:0.05402,(UltB1193:0.04495,UltB1192:0.05225)0.249.16:0.00778)0.7
65.71:0.00724,(UltB1194:0.04116,UltB1203:0.04115)0.914.114:0.01454)0.872.
103:0.01065)0.699.23:0.0041)0.154.6:5.5E-
4,((UltB1216:0.03463,UltB1217:0.05107)0.918.105:0.01432,(((((UltB1187:0.0
8007,(UltB1214:0.10376,UltB1177:0.04318)0.345.9:0.02056)0.864.99:0.02536,
UltB1215:0.04767)0.816.65:0.01141,((UltB1186:0.11617,(UltB1213:0.04942,Ul
tB1188:0.10354)0.347.10:0.0229)0.745.65:0.0066,UltB1181:0.05208)0.705.20:
0.00706)0.821.63:0.00742,(UltB1172:0.05891,UltB1174:0.07336)0.763.91:0.01
104)0.349.7:0.00395,(BacTHCL1:0.08011,BacL200B:0.04357)0.836.71:0.00857)0
.905.107:0.00907)0.691.14:5.5E-
4)0.762.88:0.00334,(UltB1220:0.02653,UltB1228:0.04707)0.852.92:0.00991)0.
803.59:0.00333,((UltB1179:0.03015,UltB1189:0.03578)0.046.6:0.00944,(UltB1
196:0.09127,UltB1239:0.03347)0.243.10:0.01106)0.758.74:0.00177)0.484.17:5
.5E-4,UltB1226:5.5E-4)0.393.9:5.5E-4)0.000.697:5.5E-4)0.000.698:5.5E-
4,UltB1197:0.01124)0.271.7:5.5E-4,UltB1199:0.00369)0.459.11:5.5E-
4)0.055.3:5.5E-
4,(UltB1241:0.00219,UltB1205:0.03427)0.990.82:0.00162)0.144.8:5.5E-
4)0.404.14:5.4E-
4,(((UltB1232:0.11863,UltB1200:0.05628)0.812.59:0.00695,UltB2600:0.01528)
0.741.53:0.0036,UltB1171:0.01895)0.760.75:0.00363)0.124.9:5.4E-
4,(UltB1221:0.03013,PsdSpe31:0.02265)0.931.105:5.4E-4)0.160.6:5.5E-
4,((PsdTolaa:0.03128,UltB1242:0.02662)0.939.86:5.5E-
4,(UltB1219:0.02543,(UltB1224:0.07194,UltB1195:0.01518)0.941.83:0.01632)0
.160.7:0.00434)0.870.83:0.00667)0.365.9:5.5E-
4,UltB1233:0.02615)0.174.8:5.4E-
4,((PsdSpe12:0.00367,((UltB1176:0.0037,((AntBact5:5.5E-
4,(UltGa176:0.00368,((PsdCedri:0.0,PsdSpe23:0.0):5.5E-
4,UltPse15:0.00369)0.465.12:5.5E-
4)0.940.97:0.00745)0.972.78:0.00195,PsdFlu18:0.01859)0.972.79:0.0019)0.23
4.8:5.5E-4,((PsdTola2:0.00368,UltB1238:0.01896)0.945.79:5.5E-

4,((UltB1243:0.0037,PsdSpe22:5.5E-4)0.848.81:5.5E-
4,(UltB1175:0.00369,UltB1185:0.00369)0.667.19:5.5E-4)0.746.56:5.5E-
4)0.862.100:5.5E-4)0.884.108:5.5E-4)0.599.9:5.4E-
4,(UltB1182:0.02629,(((UltBa307:5.4E-
4,(UltBa306:0.0271,UltBa512:0.01778)0.962.93:0.02677)0.405.17:0.00369,Ult
Gam45:0.0192)0.963.74:0.01522,UltB1183:5.5E-
4)0.981.74:0.00203)0.989.73:0.00175)0.065.13:5.5E-
4)0.923.136:0.00348)0.846.106:5.4E-
4,(PsdSpe36:0.12451,UltB1225:0.15873)0.372.12:0.00378)0.911.123:0.01686,(
PsdDurif:5.5E-4,(AzmAgili:0.01925,((PsdLute2:0.0,PsdLuteo:0.0):5.5E-
4,UltB1367:0.00369)0.894.103:0.00752)0.224.6:0.00378)0.892.102:0.00888)0.
785.82:0.00521,(((UltGa179:0.01516,(UltB1308:0.01503,(PsdBorbo:0.0,PsdBor
b2:0.0,PsdBorb3:0.0):5.5E-4)0.182.6:5.4E-
4)0.850.102:0.00694,(((((PsdSpe52:5.5E-4,((((PsdOryzi:5.5E-
4,((PsdFlu25:0.01497,UltB1293:5.4E-4)0.171.12:5.5E-
4,UltBa815:0.01503)0.409.11:5.5E-4)0.000.699:5.5E-
4,(PsdFlu20:0.0,PsdFlu24:0.0):5.5E-4)0.427.11:5.4E-
4,(PsdSpe93:0.0111,(PsdSpe48:5.5E-4,((PsdSpe83:5.5E-4,(PsdSpe86:5.5E-
4,PsdSpe89:5.5E-4)0.000.700:5.5E-4)0.000.701:5.5E-
4,(UltB1229:0.00741,(UltB1266:0.0,PsdFlu23:0.0,PsdSpe81:0.0,PsdSpe84:0.0,
PsdSpe85:0.0,UltPse32:0.0):5.5E-4)0.841.81:5.5E-4)0.747.58:5.5E-
4)0.879.106:5.4E-4)0.819.67:0.00366)0.111.5:5.1E-
4,(UltB1304:0.00371,(((((UltB1207:0.05753,UltB1210:0.07503)0.840.90:0.010
42,(UltB1206:0.03883,UltB1252:0.0154)0.731.29:0.00326)1.000.704:5.3E-
4,((UltB1272:0.002,PsdSpec7:0.0185)0.977.64:0.00185,(UltB1269:5.5E-
4,((UltB1049:0.0,PsdSpec6:0.0,PsdPsych:0.0,PsdFluo5:0.0,PsdFragi:0.0,PsdS
pec8:0.0,UltB1211:0.0,UltB1212:0.0,UltPse14:0.0,PsdLunde:0.0,UltB1257:0.0
,UltB1255:0.0,UltB1256:0.0,UltB1258:0.0,UltB1260:0.0,UltB1268:0.0,UltB127
0:0.0,PsdSpe35:0.0):5.5E-
4,(UltB1209:0.00741,UltB1208:0.00742)0.681.14:5.5E-4)0.747.59:5.5E-
4)0.418.11:5.5E-4)0.824.61:5.5E-
4)0.933.106:0.00726,(((PsdSpe13:0.0,UltB1267:0.0,UltB1273:0.0,UltB1274:0.
0,PsdSyri2:0.0,UdnBact4:0.0,UltB1276:0.0,UltB1288:0.0,PsdSyri4:0.0,PsdSyr
i5:0.0,PsdFicus:0.0,PsdSyri6:0.0,PsdSpe42:0.0,UltB1289:0.0):5.5E-
4,UltPse31:0.00742)0.958.96:5.5E-4,PsdSyrin:0.00362)0.648.14:5.5E-
4)0.914.115:0.00228,(PsdSyri3:5.5E-4,PsdSyri7:5.5E-
4)0.914.116:0.00261)0.914.117:0.00242)0.797.65:0.00366)0.997.97:5.5E-
4)0.843.75:0.00361,(((PsdSpe64:0.0,PsdSpe69:0.0,PsdSpe73:0.0,PsdMonte:0.0
,UltB1307:0.0):5.5E-
4,((PsdSpe71:0.00401,BacWMB17:0.01804)0.377.10:0.00444,((((PsdAgari:0.0,P
sdAgar2:0.0,PsdAgar3:0.0):0.00753,(PsdAsple:5.3E-
4,(((UltB1298:0.00743,((UltGa188:0.0,PsdGrami:0.0,PsdRhizo:0.0,PsdLutea:0
.0,PsdGram2:0.0,PsdViri2:0.0,PsdVirid:0.0,PsdViri3:0.0,PsdViri4:0.0,PsdSp
e96:0.0):5.5E-4,(PsdSpe59:0.0,PsdSpe95:0.0):0.00369)0.868.89:5.5E-
4)0.933.107:0.00743,PsdSpe58:5.4E-4)0.945.80:0.00752,PsdGinge:5.4E-
4)0.840.91:0.00369)0.031.3:5.4E-
4)0.774.87:0.00369,(UltGa187:0.01187,(((((((((PsdPuti4:0.0,PsdFilis:0.0,P
sdSpe54:0.0,PsdSpe55:0.0,PsdPuti9:0.0,PsdSpe61:0.0,UltB1300:0.0,UltB1299:
0.0,PsdCich2:0.0,PsdCicho:0.0):5.5E-
4,((PsdSpe60:0.02448,(PsdPuti7:0.00416,UltB1295:0.0757)0.756.79:0.00329)0
.653.10:5.5E-4,ElbRive2:0.01115)0.362.12:5.5E-4)0.401.11:5.5E-
4,PsdPuti8:0.0037)0.262.8:5.5E-
4,PsdPuti6:0.00366)0.914.118:0.00276,PsdPleco:0.00228)0.914.119:0.00276,P
sdJapon:0.01123)0.296.7:5.5E-4,UltB1262:0.00369)0.205.10:5.4E-

4,PsdSpe62:5.5E-4)0.840.92:5.4E-
4,(PsdStut6:0.00717,((PsdStut4:0.05584,UltB1386:0.01141)0.896.91:5.5E-
4,(((((PsdStu10:0.00252,(PsdStut9:0.01508,UltB1346:5.5E-
4)0.912.127:0.00242)0.912.128:0.0025,PsdFrag2:0.00371)0.421.10:5.5E-
4,(PsdStut3:0.0,PsdStut2:0.0,UltPse35:0.0,PsdStut5:0.0,PsdSp117:0.0,UltB1
343:0.0,PsdSp119:0.0,PsdSp122:0.0,UltB2546:0.0):5.5E-4)0.594.11:5.5E-
4,UltB1342:5.5E-4)0.973.66:5.5E-4,(((UltB1385:0.01951,(PsdBalea:5.5E-
4,(UltB1387:0.0114,(PsdSp152:0.00737,(UltB1388:0.00367,(UltPse42:0.0,PsdS
p151:0.0):5.5E-4)0.420.15:5.5E-
4)0.768.72:0.00348)0.990.83:0.0231)0.933.108:0.01144)0.471.12:0.00358,((P
sdStu13:5.5E-4,PsdStu14:5.5E-
4)0.966.101:0.01097,(((PsdStut8:0.0,PsdSp121:0.0):5.5E-
4,(MetExtor:0.01506,PsdSp120:0.00743)0.786.80:5.5E-4)0.000.702:5.5E-
4,(UltB1341:0.00369,(((UltB1250:0.0,UltB1265:0.0,PsdStu11:0.0,PsdChlo7:0.
0,UltPse36:0.0,PsdStu12:0.0,UltB1345:0.0):5.5E-4,PsdStut7:5.5E-
4)0.745.66:5.5E-4,UltB1344:0.01123)0.826.62:0.00369)0.664.13:5.5E-
4)0.926.114:5.5E-4)0.753.76:5.4E-4)0.892.103:5.5E-
4,PsdSp118:0.02696)0.886.122:0.00706)0.831.61:0.00365)0.920.111:5.5E-
4)0.939.87:0.01132)0.407.11:5.3E-
4)0.852.93:0.00719)0.864.100:0.00689,(((PsdSpe82:0.0076,(((PsdPlec2:0.0,P
sdMosse:0.0,PsdMoss2:0.0,UltB1303:0.0,PsdSpe75:0.0):5.5E-4,UltGa189:5.5E-
4)0.016.2:5.3E-4,(PsdSpe65:0.0,PsdSpe66:0.0):0.00369)0.125.9:7.3E-
4)0.030.6:5.9E-
4,((UltB1297:0.02621,(UltPse29:0.00372,((PsdFlu19:0.0,UltB1305:0.0,PsdSpe
80:0.0):5.5E-4,UltShee2:5.5E-4)0.853.79:5.5E-4)0.992.81:5.5E-
4)0.847.114:0.00364,(PsdSpe46:0.00369,((PsdChlor:0.0,PsdChlo2:0.0,PsdChlo
3:0.0,PsdChlo4:0.0,PsdChlo5:0.0,PsdChlo6:0.0,BrkCepac:0.0,BactePK2:0.0,Ul
tGa186:0.0,PsdSpe53:0.0,UltGa190:0.0,GmmPro35:0.0,PdmBacte:0.0):5.5E-
4,UltGa191:5.5E-4)0.000.703:5.5E-4)0.987.96:5.4E-
4)0.823.63:0.00365)1.000.705:9.7E-4,((((PsdMedit:0.00369,((UltPse22:5.5E-
4,((UltPse16:5.5E-
4,((((((((((PsdBrenn:0.0,PsdSpe15:0.0,UltB1184:0.0,UltSheep:0.0,UltB1259
:0.0,BacTLCL7:0.0,UltGa185:0.0,UltB1381:0.0):5.5E-
4,(UltB1234:0.00369,(PsdCanna:0.00739,(UltPse20:5.5E-
4,(PsdSpe38:0.00708,(PsdFlu21:0.00742,((UltB1245:0.00368,((((((PsdSpe50:
0.02672,((BactWMC9:0.00752,BacWMC11:0.02223)0.778.82:0.0043,(PsdSpe88:0.0
0368,(UltB1287:0.00739,(((UltPse33:0.00368,(UltB1306:0.00745,(PsdSpe92:5.
4E-4,UltB2599:0.01554)0.846.107:0.00383)0.315.12:5.5E-4)0.384.12:5.5E-
4,PsdSpe87:0.00369)0.638.11:5.5E-
4,(PsdSpe49:0.0,PsdPut10:0.0,PsdPut20:0.0,Udntdede:0.0,PsdPut21:0.0,PsdSp
e90:0.0,PsdPut22:0.0,PsdPut23:0.0):5.5E-4)0.562.13:5.5E-4)0.173.6:5.4E-
4)0.189.7:5.5E-4)0.124.10:5.5E-4)1.000.706:5.4E-
4,((PsdMand2:0.06064,UltPse27:0.01603)0.737.59:0.00264,UltB1281:5.4E-
4)0.443.7:0.00365)0.850.103:0.00369,(UltPse26:0.0148,PsdFlu22:0.00753)0.8
99.105:5.5E-4)0.425.7:5.5E-4,PsdSpe41:0.00369)0.425.8:5.5E-
4,(UltPse18:5.5E-4,(UltPse23:5.5E-4,(UltPse17:5.5E-
4,UltPse19:0.01416)0.843.76:5.4E-4)0.808.70:5.4E-4)0.768.73:5.3E-
4)0.445.15:5.5E-4,PsdSpe30:5.5E-4)0.437.14:5.5E-
4,(PsdSpe29:0.0,PsdMargi:0.0,PsdMande:0.0,PsdSpe39:0.0,UltPse24:0.0,PsdSp
e45:0.0,PsdFlu26:0.0,PsdSpe91:0.0):5.5E-4)0.447.11:5.5E-4)0.016.3:5.5E-
4,PsdPut19:0.00368)0.118.10:5.4E-4)0.936.102:5.5E-
4)0.864.101:0.00765)0.527.8:5.5E-4)0.458.26:5.5E-4)0.247.6:5.5E-
4)0.000.704:5.5E-4,UltB1244:0.00369)0.427.12:5.5E-
4,UltB1248:0.00368)0.496.6:5.4E-

4,UltB1271:0.01109)0.839.84:0.00409,AntBact6:0.0037)0.476.15:5.5E-
4,(PsdSpe44:5.5E-4,(UltGa180:0.00369,UltrSoil:0.00369)0.831.62:5.5E-
4)0.000.705:5.5E-4)0.000.706:5.5E-4,PsdSpe78:0.0074)0.460.10:5.5E-
4,PsdSpe79:0.00369)0.462.15:5.5E-4,(UltB1277:0.02268,PsdSpe21:5.5E-
4)0.000.707:5.5E-4)0.464.12:5.5E-
4,(UltB1291:0.00743,UltBa885:0.00369)0.000.708:5.5E-4)0.457.15:5.5E-
4)0.000.709:5.5E-
4,(UltPse21:0.0,GmmPro34:0.0,PsdSpe28:0.0,PsdSpe32:0.0,PsdSpe33:0.0,PsdSp
e40:0.0,UltGa182:0.0,UltrSoi2:0.0,UltB1290:0.0,PsdSpe47:0.0,PsdBrass:0.0,
PsdSpe51:0.0,PsdSpe77:0.0,PsdSpe76:0.0,RapRhizo:0.0,UltB2597:0.0,UltB2598
:0.0):5.5E-4)0.432.16:5.4E-4)0.221.7:5.5E-4,PsdSpe43:5.5E-
4)0.838.88:0.00405)0.139.9:5.5E-4,(PsdThive:0.0,PsdSpe34:0.0):5.5E-
4)0.233.9:5.5E-
4,PsdSpec9:0.00369)0.915.101:0.00265,((UltB1240:0.0,UltB1282:0.0):5.5E-
4,UltB1251:5.5E-
4)0.898.118:0.00171)0.879.107:0.0077)0.741.54:0.00261)0.829.80:0.00216)0.
932.93:5.4E-
4)0.810.64:0.00418,PsdPut12:0.0019)0.789.73:0.00475)0.047.4:5.1E-
4,((PsdSpe74:5.5E-
4,(((PsdPut14:0.0,PsdPut17:0.0):0.00369,(((PsdSpe70:0.01004,UltB1294:0.00
742)0.106.8:5.5E-4,(PsdPlec3:5.5E-
4,(UltB1249:0.0,UltGa178:0.0,PsdPuti3:0.0,UltB1283:0.0,UltGa184:0.0,UltB1
292:0.0,PsdSpe67:0.0,PsdSpe63:0.0,PsdPut11:0.0,PsdPut13:0.0,PsdPlec4:0.0,
PsdPut16:0.0,PsdSpe72:0.0,UltB1302:0.0,PsdFulva:0.0,PsdPut18:0.0,PsdFulv2
:0.0,UltGa192:0.0,PsdSp130:0.0,PsdArge2:0.0,PsdArgen:0.0,PsdFlave:0.0,Udn
Bact5:0.0,PsdSp134:0.0,PsdSp150:0.0):5.5E-4)0.000.710:5.5E-
4)0.392.10:5.5E-4,(UltPse30:5.5E-
4,PsdMont2:0.002)1.000.707:0.00169)0.000.711:5.5E-4)0.423.13:5.5E-
4,UltB1301:0.00369)0.425.9:5.5E-4)0.403.10:5.5E-
4,PsdPut15:0.00742)0.596.8:5.5E-
4)0.836.72:0.00479,UltPse28:0.00765)0.355.15:5.4E-
4,(UltB1264:0.0,UltB1378:0.0):5.5E-
4)0.803.60:0.00149)0.847.115:0.00696,((AzmMacro:0.01768,((AzmInsi2:0.0073
3,AzmInsig:5.5E-4)0.901.105:0.01679,(Bact52N3:0.01475,(PsdSp147:5.4E-
4,((UltB1368:0.0,PsdSp146:0.0,PsdSp145:0.0,UltB1373:0.0):5.5E-
4,UltB1372:0.00742)0.976.59:0.01515)0.350.10:0.00444)0.931.106:0.02137)0.
388.9:0.00448)0.880.110:0.00899,((PsdAzoti:0.0,PsdSp138:0.0):0.01129,(Azt
Nigri:0.00734,((AztSalin:5.5E-
4,((UltB1363:0.0,AztChro4:0.0):0.0037,((AztChroo:0.0,AztBeije:0.0):5.5E-
4,(UltPse41:0.00369,((((((UltB1352:0.0,UltB1353:0.0,UltB1354:0.0):5.5E-
4,(UltB1356:0.01472,UltB1357:0.00199)0.941.84:0.00193)0.790.74:5.5E-
4,UltB1355:0.00744)0.882.108:0.00751,(UltGa195:0.00723,(PsdJinju:5.5E-
4,PsdTherm:0.01509)0.932.94:5.4E-
4)0.849.89:0.00732)0.891.90:0.00766,(((((((UltB1330:0.03038,(((PsdSp110:5
.5E-
4,PsdCitro:0.02272)0.853.80:0.00369,(PsdNitr4:0.0121,(UltB1327:0.00748,Ps
dNitr3:0.07829)0.203.7:0.00782)0.646.12:5.5E-4)0.366.9:5.5E-
4,UltB1325:0.00369)0.000.712:5.5E-4)0.807.56:5.5E-
4,PsdSp104:0.0074)0.000.713:5.5E-4,PsdSp107:0.00369)0.000.714:5.5E-
4,(UltB1329:0.02665,UltB1328:0.04794)0.751.48:0.00374)0.448.11:5.5E-
4,(PsdCitr2:0.0,PsdSp108:0.0,PsdNitro:0.0,PsdNitr2:0.0,UltB1326:0.0,PsdSt
utz:0.0,UltB1331:0.0,PsdSp109:0.0,PsdSp111:0.0,PsdSp112:0.0):5.5E-
4)0.395.13:5.5E-4,BacFCC42:0.00369)0.348.11:5.5E-
4,UltB1332:0.02281)0.900.86:0.00923)0.770.89:0.00452,UltB1358:0.00746)0.4

96.7:5.4E-4)0.260.10:5.5E-4)0.385.15:5.5E-
4)0.852.94:0.00455)0.566.9:5.4E-
4,((AztChro2:0.0,AztChro3:0.0):0.0037,UltPse40:5.4E-
4)0.927.102:0.01125)0.657.13:0.00459)0.788.76:0.00474)0.374.13:5.3E-
4)0.880.111:0.00818)0.486.13:5.4E-4)0.926.115:0.01594)0.727.30:5.3E-
4)0.228.11:0.0057,(PsdTuomu:0.0,SrpFlexi:0.0):0.00748)0.839.85:0.00884,(P
sdSp139:0.00744,((PsdOryz2:0.0,PsdSp140:0.0,PsdOryz3:0.0,UltB1364:0.0,Ult
B1379:0.0):5.5E-4,UltB1365:0.00369)0.964.88:5.3E-
4)0.920.112:0.01485)0.351.12:0.01119,(UltB2734:0.0046,UltB2735:0.00648)0.
999.137:0.06236)0.967.68:0.03115,((((UltGa144:0.00281,UltGa145:0.02418)0.
954.82:0.01956,((UltBa843:0.02812,(((GmmPro36:0.01156,(UltrBe12:0.00367,(
(UltGa199:0.0,UltPse43:0.0):5.5E-4,UltGa200:5.5E-4)1.000.708:5.5E-
4)0.988.79:0.0356)0.848.82:0.01644,(UltGa201:0.02958,(UltGa197:0.00374,(U
ltGa202:0.06609,(UltGa198:0.00369,UltrBe13:5.5E-4)0.655.20:5.4E-
4)0.732.29:0.01513)0.998.118:0.05473)0.517.5:0.00799)0.973.67:0.03905,(Ul
tB4787:0.04861,(((((PsdResi3:0.0,UltB1340:0.0,PsdSp136:0.0,PsdSp137:0.0):
5.5E-
4,((UltB1362:0.00369,(UltB1361:0.00369,(UltB1359:0.00369,(PsdAlca4:0.0036
9,((UltGa194:5.5E-4,UltB1360:5.5E-4)0.387.11:5.5E-
4,PsdResi2:0.02677)0.243.11:5.5E-4)0.464.13:5.5E-4)0.476.16:5.5E-
4)0.510.9:5.5E-4)0.000.715:5.5E-4,(((AztTropi:5.5E-
4,(AzrPaspa:0.0,AztVinel:0.0):0.01127)0.867.104:5.4E-
4,PsdSp135:0.00743)0.112.5:0.0037,UltB1254:0.00741)0.822.57:5.4E-
4)0.656.14:5.5E-4)0.397.15:5.5E-4,(PsdOtiti:0.00371,PsdSp106:5.3E-
4)0.866.95:0.00369)0.131.7:5.3E-
4,(((UltB1310:0.00368,UltB1311:0.01121)0.156.8:5.5E-
4,((UltB1312:0.0,UltB1313:0.0,UltB1380:0.0):5.5E-
4,UltB1314:0.00369)0.906.111:5.5E-4)0.795.77:5.5E-
4,UltB1315:0.01113)0.944.76:0.01118)0.242.8:5.4E-
4,((PsdSp103:0.01494,((((((PsdAeru8:0.02666,((UltB1333:5.5E-
4,(((UltB1339:0.00369,((((PsdSp116:0.00369,PsdResin:0.01141)0.109.10:5.5E
-4,UltB1335:0.00741)0.528.9:5.5E-
4,(PsdSp114:0.0,UltB1336:0.0,UltB1337:0.0,PsdAlcal:0.0,PsdSp115:0.0):5.5E
-4)0.550.8:5.5E-4,PsdAlca2:5.5E-4)0.504.10:5.5E-4)0.893.114:5.5E-
4,UltB1338:0.0589)0.933.109:0.00743,(PsdAer17:5.4E-
4,PsdAlca3:0.00738)0.314.9:0.00741)1.000.709:5.5E-4)0.820.53:5.4E-
4,(PsdSp113:5.5E-
4,PsdMendo:0.01126)0.949.95:0.01127)0.916.114:0.00749)0.669.15:5.4E-
4,PsdIndic:0.01115)0.468.15:5.5E-
4,(PsdAer15:0.04306,UltBa356:0.01484)0.875.111:5.4E-4)0.498.8:5.5E-
4,(UltBa734:0.0,UltBa735:0.0,UltBa883:0.0,UltBa884:0.0,PsdAerug:0.0,UltB1
334:0.0,UltB1316:0.0,PsdSp101:0.0,PsdAeru2:0.0,UltB1317:0.0,PsdAeru3:0.0,
PsdAeru4:0.0,PsdAeru6:0.0,UltB1318:0.0,PsdAeru7:0.0,PsdAeru9:0.0,PsdAer10
:0.0,PsdAer12:0.0,PsdAer13:0.0,PsdAer14:0.0,UltB1324:0.0,PsdAer16:0.0):5.
5E-4)0.328.13:5.5E-4,PsdSp102:0.01114)0.042.6:5.4E-
4,(PsdAeru5:0.01156,UltPse34:0.00975)0.781.77:0.00495)0.050.8:5.5E-
4,PsdAer11:5.5E-4)0.318.10:5.5E-4)0.526.9:5.5E-
4,(UltB1321:0.04308,((UltB1320:0.06325,UltB1322:0.16071)0.828.56:0.01532,
(UltB1319:0.02703,UltB1323:0.03391)0.736.44:0.00438)0.799.83:0.00372)0.83
0.73:5.4E-
4)0.835.83:0.00359)0.831.63:0.00706)0.918.106:0.02258)0.894.104:0.02353)0
.771.94:0.0274,UltGa147:0.03421)0.798.68:0.02091)0.771.95:5.4E-
4,(UltGa146:5.4E-
4,(UltGa300:0.06577,((UltSulfu:0.0,UltGa143:0.0,UltAlcan:0.0,UltPse12:0.0

):0.01208,UltB1123:0.01064)0.901.106:0.01534)0.606.13:0.01619)0.874.124:0
.01555)0.933.110:0.01509,((((UltB1126:0.0,UltB1127:0.0):0.0037,PrcHydro:5
.5E-4)0.856.86:5.4E-
4,(UltMar25:0.02309,UltB1128:0.01116)0.273.10:0.00362)0.957.80:0.02467,(U
ltGa151:0.02875,((UltGa149:5.5E-
4,((UltGa150:0.01447,(UltB1131:0.00339,MaiGamm8:0.00779)0.132.14:0.0037)0
.862.101:5.4E-4,UltB1132:5.4E-
4)0.430.11:0.00365)0.781.78:0.00539,((UltB1129:0.02579,(UltB1130:0.065,Ul
tGa148:0.00642)0.301.9:0.00732)0.663.16:0.00127,(MaiGamm7:5.5E-
4,UltMar26:0.00369)0.849.90:5.4E-
4)0.791.66:0.00567)0.202.11:0.00953)0.980.60:0.02921)0.843.77:0.00951)0.8
53.81:0.0117)0.629.17:5.5E-4)0.885.94:0.00909,(((MicSpeci:5.4E-
4,(((((MicDongh:0.01135,((ProteM37:0.0,MicEpial:0.0):5.5E-
4,MicVaria:5.5E-4)0.885.95:5.5E-4)1.000.710:5.4E-
4,(MicHydro:0.01124,(SmsLipol:5.5E-4,MicHydr2:5.5E-4)0.842.95:5.5E-
4)0.834.65:0.00362)0.442.16:0.00369,((BactQM16:0.0,PsdSpec3:0.0,MicSpec2:
0.0,MicMarit:0.0):5.4E-4,MicTherm:0.00369)1.000.711:5.5E-
4)0.867.105:0.00202,MicAgari:0.00206)0.915.102:0.00697,(MicCeler:0.0,MicH
alop:0.0):5.4E-4)0.912.129:0.00741)0.988.80:5.4E-
4,((UltAnta4:0.02503,((OleAntar:0.0,UltBa799:0.0):5.5E-
4,UltAnta3:0.00369)0.929.90:0.01856)0.946.88:0.02347,UltGa155:0.0483)0.70
9.19:0.02372)0.983.60:0.04079,((((ReiMarin:0.01686,GmmPro20:0.01048)0.737
.60:0.00887,(ReiBland:0.03356,GmmPro21:0.01612)0.849.91:0.01116)0.951.90:
0.02437,(SacSalsu:0.01509,(SacImpat:0.0,UltBa809:0.0):5.4E-
4)0.921.112:0.01388)0.424.18:0.00412,((((LtrLipol:0.01033,UltGam90:0.0130
5)0.999.138:0.06608,(UltB2797:0.0542,UltB2842:0.06052)0.649.11:0.03112)0.
696.16:0.00328,((IntBacte:0.03169,UltBa872:0.01549)0.586.14:0.01643,(((Ul
tBa870:0.05596,UltBa871:0.00942)0.916.115:0.02083,(UltBa869:0.00788,UltGa
m99:0.01115)0.932.95:0.01566)0.570.6:0.00682,(UltBa867:0.01555,((UltSpong
:0.01723,((UltBa866:0.02394,UltGam98:0.03778)0.902.104:0.0202,(UltBa868:0
.02193,(UltBa864:0.0037,UltBa865:5.0E-
4)0.890.104:0.01333)0.278.9:0.00603)0.875.112:0.01572)0.826.63:0.00833,En
zElysi:0.00755)0.261.7:5.3E-
4)0.778.83:0.00593)0.911.124:0.02256)0.957.81:0.034)0.817.52:0.01785,UltG
a156:0.06982)0.783.90:0.01217)0.961.80:0.02908)0.772.84:0.01009)0.981.75:
0.02351,((((((UltB1143:0.00746,((Udntdded:0.0,UltB1141:0.0,CllSpec2:0.0):
5.4E-4,(CllFulvu:5.4E-4,(CllGanda:0.02338,(CllSpec3:5.5E-
4,UltCellv:0.00369)0.482.15:5.4E-
4)0.964.89:0.01121)0.927.103:0.00742)0.771.96:0.00371)0.880.112:0.01278,(
CllJapon:0.00709,UltB1144:0.03206)0.857.105:0.01334)0.951.91:0.02081,(Bac
tQM42:0.00746,PsdSpec4:0.01118)0.000.716:5.3E-4)0.715.21:5.5E-
4,(((UltB1140:0.04361,(UltBa882:0.02116,(UltBa881:0.04745,((UltBa878:0.0,
UltBa879:0.0):5.5E-
4,UltBa880:0.00369)0.765.72:0.00501)0.983.61:0.04876)0.996.90:0.06698)0.3
78.15:0.00592,(MncKoree:0.00432,(MncAgari:0.0038,MncSpeci:0.00745)0.896.9
2:0.01092)0.959.91:0.02569)0.947.77:0.01826,(MncSpec2:0.02223,UltB1142:0.
02271)0.863.110:0.00873)0.975.70:5.4E-
4)0.913.116:0.01143,(((UltB1135:0.01598,(SchDegra:0.02613,UltB1138:0.0358
8)0.736.45:0.01417)0.947.78:0.02865,(((EnmbRT12:0.0,SymbiLP1:0.0):0.00385
,EnmbRT18:0.00361)1.000.712:0.0556,(((TerTurne:0.00717,SymbiLP2:0.0402)0.
761.68:5.3E-
4,((SimSpeci:0.01148,SimAgari:0.02751)0.944.77:0.01992,(GmmPro33:0.00261,
((UltB1136:0.0153,UltB1137:0.01947)0.960.90:0.02626,UltGa157:0.03838)0.67
6.10:0.00618)0.905.108:0.0126)0.854.92:0.00767)0.975.71:0.01705,UltGa158:

0.01069)0.000.717:5.4E-
4)0.477.10:0.01878)0.824.62:0.0141,(MaiMeta3:0.02131,UltB1139:0.04119)0.8
83.111:0.03673)0.753.77:0.00655)0.944.78:0.0145,((UltAntar:0.04209,(UltBa
516:0.10565,(UltBa521:0.02904,(((((((PolarSea:0.00447,MaiBact2:0.01044)0.
940.98:0.01508,(GlaSpec2:0.0017,(BowDenit:0.00801,(UltMari3:0.00324,(UltB
a518:0.01544,(AlmMacl3:0.01474,AldBacte:5.4E-
4)0.256.7:0.00368)0.400.10:5.5E-
4)0.880.113:0.01513)0.949.96:0.01837)0.929.91:0.00149)1.000.713:5.4E-
4,(AesSpeci:0.0,GlaLipol:0.0):0.02402)0.917.95:0.01002,(AesSalex:0.00757,
(GlaPunic:0.01117,UltGam49:0.00805)0.993.72:0.03723)0.915.103:0.01454)0.8
51.94:0.00529,((AlmSpec5:0.03137,UltGam50:0.05492)0.837.61:0.0132,(((SnmC
hung:5.5E-4,AlmHispa:5.5E-
4)0.837.62:0.00369,UltAlte2:0.00369)0.385.16:5.4E-
4,(AlmMacl2:0.0,UltAlter:0.0):5.5E-4)0.781.79:5.4E-
4)0.907.101:0.00219)0.901.107:0.0021,(AlmTagae:5.5E-
4,((AlmSimid:0.03523,UltGam48:0.01146)0.770.90:0.00353,(((((UltProt8:0.0,
AlmSpec3:0.0,AlmSpeci:0.0,AlmMacle:0.0,UltAlte3:0.0,UltGam47:0.0):5.5E-
4,(((UltBa515:0.00859,AlmSpec2:0.08928)0.734.51:0.00258,(AesHalop:5.4E-
4,((UltBa522:0.00752,(UltBa519:0.0112,(UltGlac2:0.00406,(UltGlac3:0.0,Ult
Alte4:0.0,UltBa520:0.0,UltGlac5:0.0):5.5E-4)0.839.86:5.5E-
4)0.965.74:0.02097)0.718.25:5.5E-
4,UltGam51:0.01126)0.844.102:0.00406)0.838.89:0.00402)0.552.10:5.3E-
4,UltBa517:0.00741)0.408.9:5.5E-4)0.000.718:5.5E-
4,UltGam46:0.00369)0.116.7:5.5E-4,(UltMari2:0.0037,(AlmSpec4:5.5E-
4,(PdlSpeci:0.00742,AlmAlvin:0.00369)0.703.25:5.5E-4)0.719.23:5.4E-
4)0.860.73:0.00369)0.127.3:5.5E-
4,(AlmGenov:0.0,AlmLitor:0.0):0.00744)0.423.14:5.5E-4)0.220.8:5.5E-
4)0.755.68:5.4E-4)0.904.104:5.4E-
4,(UltBa523:0.01127,(((GlaSpeci:0.0,PdlAtlan:0.0):0.00745,AntBacte:5.5E-
4)0.973.68:0.0232,(GlaPalli:0.00797,(UltGlac4:5.5E-
4,GlaNitra:0.0037)0.182.7:5.5E-
4)0.943.95:0.0208)0.738.39:0.00379)0.926.116:0.01196)0.895.115:0.01569)0.
884.109:0.02434)0.363.13:0.01625)0.744.55:0.02165,(UltB1145:0.05166,((Hah
Chej2:5.5E-
4,HahCheju:0.00369)0.941.85:0.01767,(HahGangh:0.01638,HahAntar:0.06913)0.
803.61:0.01392)0.870.84:0.01365)0.948.107:0.03834)0.987.97:0.05466)0.744.
56:5.5E-
4)0.967.69:0.01527)0.909.89:0.01005)0.880.114:0.01076)0.911.125:0.01386)0
.000.719:0.00351)0.771.97:0.00593,((((((ScvBacte:0.04018,((UltBa697:0.014
48,UltBa698:0.03887)0.994.89:0.05191,(UltBa699:0.0,UltBa700:0.0,UltRumin:
0.0):0.00673)0.230.6:0.00165)0.853.82:0.02393,((UltRume3:0.0,RmnBacte:0.0
):5.4E-4,((UltBa696:0.0,RumAmylo:0.0):5.5E-
4,UltRume2:0.02245)0.977.65:0.01492)0.857.106:0.01316)0.718.26:0.01268,(S
cmAmylo:0.14294,((AnaSucci:0.06201,(((((UltSucci:0.01788,UltBa688:0.02638
)0.755.69:0.01242,UltSucc2:0.01987)0.878.97:0.01475,(UltRumen:0.0037,(Suc
Dextr:0.00719,UltBa687:0.02345)0.910.101:5.4E-
4)0.793.79:0.00372)0.692.10:0.00372,UltBa686:5.4E-
4)0.956.87:0.03894,((UltBa689:0.03243,(((UltBa690:0.0,UltBa692:0.0):5.5E-
4,UltBa693:5.5E-4)0.985.75:5.4E-
4,UltBa691:0.04784)0.168.11:0.00327)0.846.108:0.02025,(UltBa695:0.06656,S
ccHippe:0.03376)0.575.6:0.01162)0.948.108:0.04982)0.703.26:0.02331)0.866.
96:0.01791,(UltBa694:0.06444,AnaThoma:0.02103)0.113.10:0.01209)0.976.60:0
.05776)0.882.109:0.02716)0.765.73:0.00727,(PrmEndos:0.05155,((((EnbBac11:
0.00744,EnbBac12:0.00369)0.940.99:5.5E-

4,(UltGam15:0.00769,((UltGam13:5.5E-4,((UltGamm9:5.5E-
4,UltGamm8:0.00199)1.000.714:0.00166,(((UltGam16:0.0037,UltGam29:0.00371)
0.579.13:5.5E-4,UltGam11:0.00368)0.882.110:5.5E-
4,((UltGam17:0.0,UltGam10:0.0,UltBa339:0.0,UltGamm6:0.0,UltGam18:0.0,UltG
am19:0.0,UltGam20:0.0,UltGam21:0.0,UltGam22:0.0,UltGam23:0.0):5.5E-
4,(UltGam24:0.01119,UltGamm7:0.00742)0.706.25:5.5E-4)0.000.720:5.5E-
4)0.933.111:5.5E-4)0.891.91:5.3E-4)0.899.106:0.00715,(UltGam14:5.5E-
4,UltGam12:0.01117)0.845.95:0.00369)0.770.91:5.3E-
4)0.679.19:0.00761)0.770.92:0.00377,((((OrbSpeci:5.4E-
4,GmmProt7:0.01123)0.783.91:0.00763,ScnSymb3:0.0321)0.761.69:0.0036,(UltG
am26:5.5E-4,(UltGam25:5.5E-
4,UltGam27:0.00369)0.843.78:0.0037)1.000.715:5.4E-
4)0.745.67:0.00366,(PsrBact6:0.00367,(UltGam28:0.00369,UltGam30:5.5E-
4)0.991.82:5.4E-
4)0.983.62:0.0195)0.873.102:0.00767)0.757.60:0.00479,(UltBa340:0.00501,Ul
tGam31:0.07649)0.029.6:0.00554)0.812.60:0.01744)0.987.98:0.05332)0.816.66
:0.01086,(((((UltBa341:0.04722,(((ChelOri2:0.01579,(PsrBact2:0.0,PsrBact3
:0.0):0.01626)0.000.721:0.00684,((PhoUteri:0.00367,PasSkyen:5.5E-
4)0.979.76:0.04507,(((((PasSpec2:0.01524,LonKoala:0.01511)0.820.54:5.4E-
4,(((ActSucc2:0.01124,(UltBa316:0.00369,AggSegni:5.5E-4)0.951.92:5.5E-
4)0.885.96:0.01128,(UltBa315:0.00749,((HaeHaemo:0.0,HaeHaem2:0.0,HaeHaem3
:0.0,UltBa314:0.0,HaeSpec2:0.0,HaeInfl2:0.0,HaeSpec3:0.0,HaeInfl3:0.0,Hae
Infl4:0.0,HaeInfl5:0.0):5.5E-4,(((UltHaemo:0.0,HaeQuent:0.0):5.5E-
4,HaeInflu:0.0037)0.840.93:0.00369,UltBa313:0.01122)0.911.126:5.5E-
4)0.506.11:5.5E-4)1.000.716:5.5E-4)0.845.96:0.00367,HaeSpeci:5.3E-
4)0.777.96:0.00364)0.881.112:0.00756,(PasBetty:0.01122,(ActMuris:0.00748,
(ManSpec3:5.5E-4,(PasSpeci:0.00369,ManGluco:0.0074)0.484.18:5.5E-
4)0.799.84:0.00373)0.801.72:0.0037)0.821.64:5.4E-4)0.955.73:5.4E-
4,((ActArthr:5.5E-
4,((ActHomin:0.0,ActPleur:0.0,ActEquul:0.0,PasCabal:0.0):5.5E-
4,((ActSpec2:0.00367,((ActCaps4:0.0,ActSpeci:0.0):5.3E-
4,PasLanga:0.0074)0.696.17:0.00744)1.000.717:5.4E-
4,ActUreae:0.0037)0.880.115:5.5E-4)0.000.722:5.5E-4)1.000.718:5.5E-
4,HaeDucre:0.00366)0.395.14:0.01111)0.788.77:0.00793,((((((UltBa338:0.015
04,ActScoti:5.4E-
4)0.986.70:0.01917,((GalMelop:0.0,GalTreha:0.0,GalTreh2:0.0):0.01902,UltA
ctin:0.01898)0.253.11:5.5E-
4)0.884.110:0.01,((BsrHudso:0.01148,(((HisSomn2:0.0,HisSomni:0.0,HisSomn3
:0.0,HisSomn4:0.0):0.03221,(GalGenom:0.0151,GalAnati:5.5E-
4)0.967.70:0.02421)0.829.81:0.00735,(((PsrBact5:0.00738,(AviGall2:5.4E-
4,(AviSpeci:0.00372,(AviPara2:5.5E-
4,AviParag:0.00743)0.785.83:0.00557)0.798.69:0.00558)0.923.137:0.01172)0.
912.130:0.01203,(PsrBact4:5.4E-4,(VolPsit2:5.5E-4,VolPsitt:5.5E-
4)0.954.83:0.01138)0.919.135:0.01137)0.727.31:0.00349,((HaeSpec4:0.0,UltB
a321:0.0):0.00751,UltBa320:0.01144)0.964.90:0.01964)0.873.103:0.0079)0.85
9.93:0.00768)0.847.116:0.00989,((((((HaePara2:0.00247,((HaeParas:0.0,HaeP
ara3:0.0):5.5E-
4,(ActIndo3:0.0,ActIndo4:0.0):0.0037)0.925.111:0.00246)0.925.112:0.00246,
(ActIndo2:0.0,ActIndol:0.0):5.3E-
4)0.785.84:0.0037,(((GalGroup:0.0037,(AviEndoc:5.5E-
4,AviAvium:0.00737)0.871.102:0.01146)0.931.107:0.01135,((ActPorc2:0.0,Act
Porc3:0.0,UltBa327:0.0):5.5E-4,(ActPleu2:5.5E-4,HaeParah:5.5E-
4)0.923.138:0.00369)0.902.105:5.4E-
4)0.487.18:0.00369,(ActMinor:0.0,UltBa325:0.0):5.4E-

4)0.881.113:0.00749)0.900.87:0.00751,(ActPorci:0.0,UltBa326:0.0):5.5E-
4)0.994.90:5.4E-
4,ActMino2:0.00368)0.892.104:0.00748,(((GalGeno2:0.0,GalSalpi:0.0):0.0112
1,((((UltBa324:0.00369,UltBa334:5.5E-
4)0.844.103:0.00369,(PasAero2:0.0,ActPorc4:0.0):5.5E-4)0.187.7:5.5E-
4,(ActRossi:5.5E-
4,ActRoss2:0.00198)1.000.719:0.00172)0.903.96:0.0037,(((UltBa323:0.03511,
HaeHaem4:5.5E-
4)0.896.93:0.00748,(UltBa331:0.00714,UltBa332:0.01485)0.904.105:5.4E-
4)0.536.12:5.5E-
4,(UltBa317:0.0,BsgTaxo8:0.0,UltBa335:0.0,UltBa336:0.0,ProSymb2:0.0,UltBa
337:0.0):5.5E-4)0.764.91:5.3E-
4)0.774.88:0.00383)0.203.8:0.00377,ActDelph:0.00735)0.930.88:5.4E-
4)0.846.109:0.00326)0.896.94:0.00718)0.856.87:0.0116,(PasAerog:0.0,UltBa3
18:0.0):0.00995)0.880.116:0.00946,((((((ActCaps3:0.0037,(ActCapsu:0.00366
,(HaeFelis:5.5E-4,((PasMulto:0.0,PasCanis:0.0):0.0037,(BsgTaxo2:5.5E-
4,(NicSemol:0.01115,(((((((ManGran2:0.0,ManGranu:0.0):0.00369,(ActSucci:0
.00746,((UltHaem2:0.00369,(AggAphro:5.5E-
4,((BsgTaxo7:0.00373,(UltBa322:0.0,BsgTaxo6:0.0):5.5E-4)0.000.723:5.3E-
4,((ActSemin:5.5E-
4,PasMairi:0.00749)0.975.72:0.01911,(AggActin:0.00369,AggActi2:5.5E-
4)0.968.78:0.01912)0.768.74:0.00376)0.851.95:0.00369)0.739.43:5.3E-
4)1.000.720:5.4E-
4,(BsgTaxo3:0.01516,PasSpec4:0.00781)0.469.12:0.00759)0.918.107:0.00341)0
.285.10:5.5E-4)0.834.66:0.00368,(UltBa333:0.0,HaePittm:0.0):5.3E-
4)0.121.7:5.4E-4,(BsgTaxon:0.0037,((PasPneum:0.0,PasPneu3:0.0):5.5E-
4,PasPneu2:5.5E-4)0.800.68:5.4E-
4)0.950.90:0.0075)0.850.104:0.00367,(ManSpeci:0.0037,(ManSpec2:0.00372,(M
anVarig:0.0,PasHaemo:0.0):5.5E-
4)0.783.92:0.0037)0.794.66:0.00371)0.080.5:5.4E-
4,ManSucci:0.01897)0.908.118:0.00769,AviSpec2:0.00736)0.330.10:0.00372)0.
879.108:5.5E-4)0.367.14:5.4E-
4)0.855.92:0.00938)0.421.11:0.00946)1.000.721:5.5E-
4)0.939.88:0.00741,ActCaps2:5.4E-
4)0.924.101:0.00747,PasSpec3:0.00746)0.876.87:5.3E-4,(ManHaemo:5.5E-
4,(ManRumi2:0.0,ManRumin:0.0):0.00744)0.831.64:0.00752)0.874.125:0.00725,
((UltBa319:0.0,UltBa330:0.0):5.5E-
4,(UltBa329:0.00369,UltBa328:0.00742)0.676.11:5.5E-4)0.951.93:5.5E-
4)0.790.75:0.01501,Psrlyyyy:0.00428)0.831.65:0.00759)0.122.3:5.5E-
4,(BsgTaxo9:0.08011,AviGalli:5.5E-
4)0.996.91:0.03527)0.807.57:0.01019)0.790.76:0.02353)0.970.77:0.05151)0.7
09.20:0.01013,(PasTestu:5.4E-4,((BsgTaxo5:0.00369,(PsrBacte:5.5E-
4,BsgTaxo4:5.5E-4)1.000.722:5.5E-
4)0.961.81:0.02258,ChelOris:0.00979)0.644.12:0.00984)0.812.61:0.01406)0.9
87.99:0.03782)0.342.11:0.00949,ThoAnoph:0.08062)0.000.724:0.01004,(((ScnS
ymb5:0.01878,ScnSymb6:5.5E-
4)0.951.94:0.03317,((ScnEndos:0.00742,CanHamil:5.5E-
4)0.962.94:0.02803,(CanRegie:0.00369,(CanRegi2:5.5E-
4,ScnSymb4:0.00369)1.000.723:5.4E-
4)0.876.88:0.01709)0.645.13:0.01427)0.782.97:0.01359,((ArsEndo4:0.03344,C
anRiesi:0.11489)0.955.74:0.04518,(BchAphid:0.04493,((MycSymbi:0.03724,((S
cnEndo4:0.02239,(((EnmbnO13:0.01301,EnmbnO14:0.03459)0.999.139:0.07726,((
EnmbnOf9:0.0072,EnmbnO10:0.04397)0.860.74:0.00846,((EnmbnO11:0.03552,Enmb
nO12:0.01516)0.972.80:0.0244,(EnmbnOf8:5.4E-4,(EnmbnOf7:5.4E-

4,EnmbnOf6:0.02333)0.920.113:0.00742)0.929.92:0.01829)0.158.9:0.00772)0.9
52.83:0.03185)0.984.68:0.04555,(UltEnt15:0.06773,(((EnmbnO16:0.01935,(Ult
Ba354:0.015,(UltBa355:0.00369,UltBa736:5.5E-4)0.893.115:5.5E-
4)0.832.74:0.00773)0.762.89:0.00359,(EnmbnO15:0.01952,(CanBloc2:0.01556,C
anBloch:0.01157)0.840.94:0.00712)0.850.105:0.00765)0.634.11:0.00224,(UltB
a357:0.02756,(((WggGloss:0.03586,CanCurcu:0.01551)0.100.6:0.00338,(((ScnE
ndo8:0.03352,(EnbBact6:0.02896,ScnEndo7:0.04712)0.622.9:0.00402)0.917.96:
0.01318,(UltSodal:0.01341,(GmSmbOf0:0.03644,ScnEndo5:0.03721)0.956.88:0.0
3547)0.443.8:0.01365)0.778.84:0.00712,(ScnEndo3:0.01132,(((CanBau10:0.022
28,(CanBau13:0.00426,(CanBau12:5.5E-
4,CanBau11:0.02691)0.933.112:0.01075)0.476.17:0.00764)0.812.62:0.00834,((
((CanBaum6:0.00757,CanBau14:0.01915)0.874.126:0.00767,(CanBaum7:0.01157,(
(CanBaum3:5.5E-
4,(CanBaum2:0.01128,(CanBaum4:0.00371,CanBaum5:0.00746)0.882.111:0.00751)
0.777.97:0.0037)0.897.100:5.5E-
4,CanBauma:0.01504)0.962.95:0.01914)0.878.98:0.01142)0.742.44:0.00565,Can
Baum8:0.02124)0.986.71:0.03763,CanBaum9:0.01734)0.896.95:0.01817)0.741.55
:0.00318,(CanBau18:0.01928,(CanBau16:0.01753,(CanBau15:0.01115,CanBau17:0
.01951)0.780.97:0.00769)0.859.94:0.01307)0.992.82:0.03756)0.940.100:0.015
98)0.769.82:0.00395)0.744.57:0.00377)0.931.108:0.01677,EnmbnO17:0.04357)0
.693.18:0.01133)0.840.95:0.00937)0.395.15:0.00831)0.439.11:0.01034)0.000.
725:5.5E-
4)0.851.96:0.01443,(((BchAphi5:0.01551,((BchAphi2:0.00374,(BchAphi3:5.4E-
4,(BchAphi6:0.02401,BchAphi7:0.02997)0.918.108:0.03007)0.526.10:0.01479)0
.108.8:5.5E-
4,BchAphi4:0.01494)0.948.109:0.02741)0.966.102:0.02693,IshSymb3:0.02893)0
.854.93:0.0081,((IshSymbi:5.4E-
4,CanIshik:0.01886)0.973.69:0.02418,(IshSymb2:0.01668,(EnbBac16:0.02194,(
EnbBac15:0.01011,(EnbBac13:0.01954,EnbBac14:0.01144)0.856.88:0.00857)0.51
1.9:0.00394)0.827.65:0.01005)0.766.74:0.00752)0.907.102:0.01481)0.929.93:
0.0214)0.247.7:0.01166)0.923.139:0.02658,((ArsEndo3:0.0,ScnEndo2:0.0):0.0
2715,YsymbOf0:0.00343)0.932.96:0.02414)0.789.74:0.00904)0.726.26:0.00452)
0.949.97:0.03695)0.672.14:5.5E-
4)0.938.63:0.02305,UltBa342:0.0642)0.674.18:5.3E-
4,((UltRume6:0.15455,(UltRume4:0.09936,UltRume5:5.5E-
4)0.912.131:0.04581)0.953.73:0.05769,(UltMari4:0.0919,(UltBa703:0.00363,U
ltBa702:5.4E-
4)0.985.76:0.0622)0.286.8:0.02977)0.877.118:0.02663)0.747.60:0.00499)0.95
6.89:0.02491,(((((((ZobDenit:0.01122,ZobTaiwa:5.5E-
4)0.875.113:0.00769,((OceSpeci:0.00365,(UltOcean:0.00368,((OceBauma:0.0,O
ceSpec2:0.0,OceSpec3:0.0):5.5E-4,OceDoudo:0.00369)0.477.11:5.5E-
4)0.744.58:5.5E-
4)0.769.83:0.00377,(OcnDongh:0.01522,OcnLitor:0.00366)0.923.140:0.01136)0
.764.92:0.00375)0.785.85:0.00638,(UltBa763:0.03054,(((FerSpec2:5.5E-
4,FerSenti:0.00752)0.957.82:5.5E-
4,((UltBa496:0.01897,(FerBalea:0.00367,(FerSpeci:5.5E-
4,FrrBacte:0.00369)1.000.724:5.3E-4)0.996.92:0.0235)0.777.98:5.4E-
4,FerMarin:5.5E-
4)0.914.120:0.00744)0.374.14:0.00731,(FerSpec3:0.01502,UltBa495:0.00371)0
.878.99:0.00785)0.970.78:0.03355)0.913.117:0.02419)0.143.10:5.4E-
4,(ColSpec5:0.02004,(SrrSpec8:0.01497,ShwSpeci:5.4E-
4)0.986.72:0.03742)0.612.16:0.01733)0.959.92:0.02381,((((((ShwSurug:0.015
49,(ShwHaned:0.01499,(UltBa733:0.00369,(ShwHane2:5.5E-
4,(ShwHane3:0.0,ShwHane4:0.0):5.5E-4)0.796.63:5.5E-4)0.900.88:5.4E-

4)0.771.98:0.00345)0.928.81:0.01163,(ShwSedi2:0.0,ShwSedim:0.0):5.4E-
4)0.986.73:5.3E-4,((ShwAtlan:0.0,ShwWoody:0.0):5.4E-
4,(ShwSpe23:0.00749,((ShwSpe22:5.5E-
4,((((ShwColwe:0.0,ShwGelid:0.0,ShwAbyss:0.0):5.4E-4,(ShwColw2:5.5E-
4,ShwSpe17:5.5E-4)0.844.104:0.00369)0.831.66:0.00369,(ShwFidel:5.5E-
4,((ShwMaris:0.0,ShwSpe19:0.0,ShwWaksm:0.0,PhagSwp3:0.0,ShwKair2:0.0,ShwK
aire:0.0):5.5E-4,((ShwLoihi:0.01927,ShwSpe20:0.03585)0.004.8:5.4E-
4,ShwSpe21:0.01484)0.472.14:5.5E-4)0.000.726:5.5E-4)0.367.15:5.3E-
4)0.877.119:0.00375,ShwSpe18:0.00369)0.157.11:5.5E-
4)0.946.89:0.00501,(ShwBenth:0.00374,(((ShwViola:0.0,ShwSpe24:0.0,ShwPiez
o:0.0):5.5E-4,(ShwPeale:0.0037,ShwBent2:0.01117)0.886.123:5.2E-
4)0.782.98:5.3E-4,UltShew2:0.00756)0.905.109:5.4E-
4)0.926.117:0.00462)0.946.90:0.00502)0.086.8:5.4E-
4)0.827.66:0.00369)0.818.48:0.00752,(((((CelDiazo:0.02049,AlrBacte:0.0238
8)0.973.70:0.03813,(UltBa714:0.01921,((((PsyOssib:0.00698,PsySpeci:0.0021
3)0.886.124:0.00205,((PsyJapon:0.00747,(PsyAntar:0.0037,(UltGam38:5.5E-
4,PsyIngra:5.5E-4)0.926.118:5.5E-4)0.127.4:5.3E-
4)0.919.136:0.00742,(((PsyAgari:0.00373,PsyHeite:0.01168)0.788.78:0.00369
,(UltBa498:5.5E-4,(UltBa499:0.00761,PsyArcti:0.0037)0.295.16:5.4E-
4)0.837.63:0.00378)0.523.7:5.5E-4,PsyKaiko:5.5E-4)0.096.1:5.5E-
4)0.072.5:5.5E-
4)0.866.97:0.0089,PsyHadal:0.01386)0.648.15:0.00787,BaropWHB:0.02851)0.97
5.73:0.03623)0.835.84:0.0161)0.815.65:0.01242,((UltBa502:0.0152,GmmPro16:
0.0114)0.866.98:0.01001,((AlgMarin:0.0188,((((PdlAntar:0.01796,UltBa528:0
.04715)0.226.8:0.00871,(((((PdlSpec9:0.00369,AlmSpec7:0.0037)0.763.92:5.5
E-4,(PdlSpe12:0.03873,PdlSpec3:0.00368)0.915.104:5.5E-4)0.000.727:5.5E-
4,((PdlSpec5:5.5E-4,UltGam53:0.00369)0.000.728:5.5E-
4,(PdlSpec4:0.0,UltPseu2:0.0,UltPseu3:0.0,PdlHalop:0.0,PdlArcti:0.0,GmmPr
o14:0.0):5.5E-4)0.000.729:5.5E-4)0.954.84:5.4E-
4,(PdlCitre:0.00369,((PdlDenit:0.0,PdlSpe20:0.0):0.01506,(PdlPheno:0.0073
4,(PdlSpec2:0.0,AlrBact2:0.0):5.5E-4)0.873.104:5.3E-
4)0.756.80:0.00738)0.996.93:5.5E-4)0.738.40:5.3E-4,(PdlSpe11:5.4E-
4,(PdlRuthe:0.0037,(PdlPisc2:0.0,PdlPisci:0.0,PdlPisc3:0.0,MaiBact3:0.0,P
dlPisc4:0.0,PdlRubra:0.0,PdlByuns:0.0):5.5E-
4)0.854.94:0.00366)0.170.7:0.0037)1.000.725:5.4E-
4)0.921.113:0.00335,((((UltPseu5:0.00369,((PdlSpe13:0.00743,(UltBa527:0.0
0227,PdlSpec8:0.04743)0.892.105:0.00155)0.485.15:5.5E-
4,PdlPorph:0.00369)0.000.730:5.5E-4)0.401.12:5.5E-
4,(UltOrga2:0.01122,(UltBa526:0.03877,UltGam52:5.5E-
4)0.836.73:0.00367)0.488.13:5.5E-4)0.438.12:5.5E-
4,(PdrBacte:0.0,UltPseud:0.0,PdlSpe10:0.0,PdlMarin:0.0,PolarSe2:0.0,UltPs
eu4:0.0,PdlSpe15:0.0,BacteX12:0.0,PdlSpe16:0.0,PdlSpe17:0.0,PdlSpe18:0.0,
PdlSpe19:0.0,UltPseu6:0.0,UltPseu7:0.0):5.5E-4)0.411.11:5.4E-
4,(PdlSpe14:0.00369,((PdlUlvae:0.01124,((PdlSpec6:0.0,PdlUlva2:0.0,PdlSpe
c7:0.0):5.5E-4,PdlTunic:0.00741)0.409.12:5.5E-4)0.985.77:5.5E-
4,UltBa529:0.04)0.483.16:0.01514)0.872.104:5.4E-4)0.941.86:5.4E-
4)0.794.67:5.5E-4,((UltAlte5:5.5E-4,(AgcBact2:0.00741,GmmPro15:5.5E-
4)0.992.83:0.02745)0.901.108:0.03194,PdlRuth2:0.3906)0.728.37:0.00739)0.9
51.95:0.02398)0.806.56:0.01634,(PrlAlkal:0.0596,(UltBa739:5.5E-
4,((MrlSpeci:0.0,MrlAbyss:0.0,MrlVisco:0.0,MrlMarin:0.0):5.5E-
4,(MrlSpec2:0.01115,MrlSpec3:0.00369)0.632.11:5.5E-4)0.978.61:5.4E-
4)0.843.79:0.01097)0.979.77:0.03567)0.484.19:0.01211)0.893.116:0.00916)0.
739.44:0.00315,(AgaAlbus:0.0038,(UltAgari:0.01919,UltBa524:5.5E-
4)0.941.87:0.01504)0.945.81:0.01607)0.979.78:0.02267,((ShwOlley:0.01816,(

ShwDongh:0.00741,(AldBact4:5.5E-
4,(ShwSpe12:0.0,ShwJapon:0.0,ShwSpe13:0.0,ShwSpe14:0.0):5.5E-
4)0.827.67:5.5E-
4)0.875.114:0.00881)0.858.95:0.01593,(((PrfSedim:0.01522,(ShwSpong:0.0075
2,(ShwSpec4:0.00751,ShwIrcin:0.0037)0.759.86:0.00376)0.770.93:0.00365)0.7
50.51:0.00557,UltBa679:0.03986)0.767.75:0.00553,((ShwSpec5:0.01132,(((((S
hwSpec3:0.01118,ShwFrig4:0.00743)0.470.5:5.5E-4,(ShwFrig5:5.5E-
4,((ShwFrig2:0.00547,ShwFrigi:0.01128)0.735.37:0.00547,ShwFrig3:0.10634)0
.863.111:5.4E-4)0.784.88:5.5E-4)0.397.16:5.4E-
4,ShwSpec2:0.00734)0.836.74:0.00362,(AldBact2:0.00373,(AldBact3:5.5E-
4,(ShwBasal:5.5E-4,ShwGaetb:0.00373)0.724.24:5.5E-4)0.711.27:5.5E-
4)0.782.99:5.4E-4)0.773.90:0.00361,((UltBa726:0.01534,((ShwPutr2:5.5E-
4,(UltBa723:0.00369,(((((UltBa717:5.5E-
4,UltBa721:0.0074)0.847.117:0.00369,ShwSacch:0.01135)0.000.731:5.5E-
4,((UltBa718:0.04782,(UltBa729:0.0,UltBa730:0.0):5.4E-4)0.547.8:5.5E-
4,ShwOneid:0.00721)0.879.109:0.00727)0.252.8:5.5E-
4,UltBa722:0.00369)0.478.12:5.5E-
4,(UltBa628:0.0,UltBa715:0.0,UltBa716:0.0,ShwPutr3:0.0,ShwPutr4:0.0,ShwSp
ec6:0.0,UltBa724:0.0,UltBa725:0.0,UltBa727:0.0,UltBa728:0.0,ShwAlgae:0.0)
:5.5E-4)0.479.15:5.5E-4)0.249.17:5.5E-4)0.778.85:5.4E-
4,(UltBa719:0.01521,(UltBa625:0.00368,(UltBa626:0.0,UltBa627:0.0):5.5E-
4)0.742.45:0.0037)0.883.112:0.00746)0.762.90:0.00368)0.884.111:0.00775,(S
hwSpe11:0.00725,((UltBa720:0.0,ShwBalti:0.0,ShwSpe10:0.0,ShwBalt2:0.0):5.
5E-
4,(ShwPutre:0.00743,((((((ShwPutr5:0.0024,ShwPutr6:0.00372)1.000.726:0.00
131,UltShewa:5.5E-4)0.450.12:5.3E-4,(ShwSpe15:5.4E-
4,ShwSpe16:0.02687)0.804.61:0.00367)0.879.110:0.00369,ShwMarin:0.00742)0.
879.111:5.3E-
4,ShwAmazo:0.01113)0.745.68:0.00751,(UltBa732:0.02665,((UltBa731:0.0,ShwD
ecol:0.0,ShwSpec7:0.0,ShwSpec8:0.0):5.5E-
4,ShwSpec9:0.00742)0.999.140:5.5E-
4)0.780.98:0.00353)0.781.80:0.00384)0.756.81:5.5E-4)0.901.109:5.5E-
4)0.752.69:0.00358)0.907.103:0.00738)0.776.86:0.00372)0.790.77:0.00368,((
ShwDeni2:0.0,ShwDenit:0.0,ShwGaet2:0.0):5.5E-
4,ShwLivin:0.01526)0.944.79:0.01139)0.855.93:5.5E-
4)0.820.55:0.01)0.648.16:0.0078)0.913.118:5.5E-
4)0.872.105:0.00789,ShwSpe25:0.0111)0.162.7:0.00401,((UltBa494:0.00752,((
(SlnCost3:0.00369,((SlnCost2:0.0,SlnProte:0.0):5.5E-
4,(SlnCosti:0.00369,SlnSiame:0.00743)0.910.102:5.5E-4)0.930.89:5.2E-
4)0.931.109:0.01126,(((((PhcSpec7:0.0,PhcSpec8:0.0,PhcRosen:0.0):5.5E-
4,PhcLutim:5.5E-4)0.918.109:0.00357,(PhcGangh:5.5E-
4,(PhcSpe11:0.10583,PhcHalot:0.00358)1.000.727:5.4E-4)1.000.728:5.3E-
4)0.776.87:0.00353,VbnBact6:0.13941)0.443.9:5.4E-
4,GmmPro13:0.03521)0.571.8:0.00799)0.772.85:0.00392,(VbnBact7:5.5E-
4,(((PhcDams2:0.0,BacteST7:0.0):5.5E-
4,PhcDamse:0.00744)0.952.84:0.01108,VbnBact8:0.01503)0.835.85:5.4E-
4)0.783.93:0.00367)0.935.95:0.01584,((UltBa492:0.0,UltBa490:0.0,UltBa491:
0.0):0.0075,UltBa493:5.5E-
4)0.959.93:0.01925)0.908.119:0.0119)0.898.119:5.4E-
4,((((EnvNigri:0.00369,((EnvCalvi:0.0,EnvNorve:0.0,PhcSpe10:0.0):0.0037,(
((GriHolli:0.01107,(GriSpeci:5.4E-
4,(PhcSpec9:0.0,EnvSpeci:0.0):0.00742)0.502.8:0.00719)0.935.96:5.5E-
4,UltBa489:5.5E-4)0.682.17:5.4E-
4,UltBa487:0.00369)0.861.107:0.0037)0.007.5:5.5E-

4)0.924.102:0.00256,EnvCoral:0.00142)0.893.117:0.01082,(UltBa488:0.00309,
((((((PhcSwing:0.0,UltBa480:0.0,PhcPhosp:0.0,PhcAquim:0.0,UltBa483:0.0,Ph
cLeiog:0.0,PhcSpec3:0.0,PhcLeio2:0.0,UltBa486:0.0):5.5E-4,UltBa485:5.5E-
4)0.873.105:5.4E-
4,(PhcSpec2:0.00698,((PhcSpeci:0.0,PhcAplys:0.0,PhcProf2:0.0,PhcProfu:0.0
,PhcProf3:0.0,PhcProf4:0.0,HpmSpeci:0.0,PhcIndic:0.0,UltBa760:0.0):5.5E-
4,UltBa479:0.0037)0.912.132:0.00209)0.912.133:0.00207)0.704.18:5.5E-
4,(UltBa484:0.01543,UltBa481:0.06102)0.768.75:0.00363)0.850.106:0.0037,Ul
tBa482:5.5E-
4)0.771.99:0.00368,(PhcLipol:0.00755,UltGam37:0.00372)0.888.116:0.00763)0
.885.97:0.00981)0.277.13:0.00984)0.800.69:0.0032,(UltBa525:0.01519,(FerKy
ona:0.01555,((FerFutts:0.0,UltFerri:0.0):5.5E-
4,UltBa740:0.0037)0.866.99:0.00762)0.848.83:0.00782)0.875.115:0.01004)0.9
21.114:0.0096)0.957.83:0.01548)0.944.80:5.4E-
4)0.884.112:0.0136,(AlmSpec6:0.04931,(AgcSagam:0.02093,(PdlSpe21:0.02387,
AgcBacte:0.02286)0.784.89:0.00705)0.981.76:0.03969)0.820.56:0.01388)0.846
.110:0.01365,((((((((((KlbPne30:0.01533,(ButGavin:5.5E-
4,(((LecAdec3:5.5E-4,((((((UltBa120:0.01508,(ErwAmylo:5.5E-
4,(ErwSpec2:5.5E-
4,((UltBa116:0.00371,EnrSpe22:0.00361)0.910.103:0.00725,((((UltCitr3:0.00
376,(UltBa112:0.00752,UltBa360:0.00688)0.143.11:5.3E-4)0.759.87:5.4E-
4,((UltCitr4:5.5E-4,(((ErwTasm2:0.0,ErwTasm3:0.0):5.4E-
4,((ErwAmyl2:0.00741,(ErwPyrif:5.5E-
4,((PecCypr3:0.0,ErwAmyl5:0.0,ErwAmyl4:0.0,ErwAmyl3:0.0):5.5E-
4,(UltBa191:0.00369,PecCypr2:0.00408)0.484.20:5.5E-4)0.374.15:5.5E-
4)0.586.15:5.5E-4)0.922.145:0.00279,((PntSpec8:0.0,PecCypri:0.0):5.5E-
4,(PahggYy0:5.5E-
4,PahggYyy:0.00369)0.835.86:0.0037)0.926.119:0.00229)0.922.146:0.00279)0.
863.112:0.00406,(((SalBongo:0.01125,(((EschCo11:5.5E-
4,UltCrono:0.00818)0.406.12:5.5E-4,EnbBacte:5.5E-4)0.302.7:5.5E-
4,(EscSpeci:0.0,UltEsche:0.0,EnrCowan:0.0,EnrSpec7:0.0,CroMuytj:0.0,CroSa
ka2:0.0,CroMuyt2:0.0,EnbBact8:0.0):5.5E-4)0.572.10:5.5E-
4)0.823.64:0.00405,(UltBa113:5.5E-
4,((CitSpeci:0.00369,(UltBa297:0.00369,((UltGamm3:0.0,EncGroup:0.0,UltCit
r5:0.0,UltBa111:0.0,CitAmalo:0.0,UltBa119:0.0,CitFarm3:0.0,CitFarme:0.0,C
itFarm2:0.0,UltCitro:0.0,UltCitr2:0.0,UltProte:0.0,UltEnt12:0.0,UltBa165:
0.0,EnrGergo:0.0,UltBa170:0.0):5.5E-4,EnrSpe23:5.5E-4)0.000.732:5.5E-
4)0.386.9:5.5E-4)0.000.733:5.5E-4,UltBa115:0.01504)0.536.13:5.5E-
4)0.000.734:5.5E-4)0.507.10:5.5E-4,UltEnte8:5.5E-4)0.000.735:5.5E-
4)0.812.63:5.5E-4)0.062.7:5.5E-4,((EnrSpec3:5.5E-
4,(UltEnte5:0.0,UltGamm2:0.0):0.00369)0.954.85:5.4E-
4,(((EnrAerog:0.0,EnrOryza:0.0,EnrSpec9:0.0):5.5E-
4,(EnrRadic:0.19154,(UltEnte6:5.5E-
4,(UltBa133:0.00751,UltBa131:0.05202)0.875.116:0.00739)0.833.81:0.00365)0
.948.110:5.4E-4)0.911.127:5.4E-
4,UltKlebs:0.00742)0.253.12:0.00368)0.900.89:0.00739)0.245.9:5.4E-
4)0.305.9:5.5E-4,(((UltBa117:0.0,CitRoden:0.0):5.5E-
4,UltBa118:0.02268)0.532.14:5.5E-
4,CitFarm4:0.01101)0.828.57:0.00362)0.145.3:5.4E-
4,BacNLA22:0.00361)0.079.4:5.5E-
4)0.951.96:0.00877)0.833.82:0.00433)0.250.10:5.5E-
4)0.848.84:0.00431,(((((UltBa122:0.01133,((PntPunct:0.0,PntPunc2:0.0,PntT
err2:0.0,PntTerr3:0.0,TatPtyse:0.0):5.5E-4,TatMorb3:5.5E-4)0.730.36:5.1E-
4)1.000.729:5.1E-4,(TatMorb2:0.0,TatMorbi:0.0):5.5E-

4)1.000.730:0.00318,((EnrSpec5:0.0,EnrSpec6:0.0):0.01127,PntTerre:0.01018
)0.047.5:5.5E-4)0.403.11:5.4E-
4,(ErwSpec3:0.0,ErwTasma:0.0,EnrSpec8:0.0,PntCitre:0.0,TatSaani:0.0):5.5E
-4)0.235.11:5.3E-4,ErwSpec4:0.00369)0.688.19:5.5E-
4)0.861.108:0.00419,(((UltBa241:0.01102,((EnrClo11:5.5E-4,(UltBa227:5.5E-
4,Otu00016:0.01132)0.907.104:0.00743)0.848.85:0.00368,((EnrSpe25:0.04935,
UltBa141:0.02383)0.675.12:0.00263,((UltBa196:5.5E-
4,(((UltErwin:0.00381,((((PntSpe12:0.0,PntStew5:0.0,KluSpec2:0.0,KluSpeci
:0.0,UltBa195:0.0,EnbBac10:0.0,PntAgg18:0.0,UltBa140:0.0,KlbGranu:0.0,Klb
Gran2:0.0):5.5E-4,((UltBa194:0.00369,HumanGut:0.0216)0.108.9:5.5E-
4,UltBa189:0.00369)0.481.13:5.5E-4)0.412.10:5.5E-
4,UltBa186:0.00739)0.280.6:5.5E-4,UltBa185:5.5E-4)0.303.11:5.4E-
4)0.867.106:0.00415,((((EnrHorma:0.03045,(EnrCloa9:0.02324,EnrClo13:0.012
51)0.747.61:0.00422)0.784.90:0.00423,((((UltBa192:0.0,ErwRhapo:0.0):5.5E-
4,((((CanStam4:0.00924,(CanStam5:0.00954,CanStam6:0.00547)0.359.8:0.00375
)0.750.52:0.00565,(CanStam3:0.02348,ScnSymbi:0.05914)0.870.85:0.01618)0.3
94.12:0.00743,(CanStam2:0.0117,CanStamm:0.01127)0.991.83:0.03231)0.898.12
0:5.4E-4,KlbPne10:0.01248)0.819.68:0.00413)0.123.8:5.5E-4,ErwRhap2:5.5E-
4)0.814.52:0.00409,((KlbOxyt5:0.00734,(EnrCloa8:0.01113,(EnrCloa4:0.03159
,(KlbPne19:0.00779,(EnrLudw2:0.01542,KlbPne18:0.0032)0.704.19:0.00368)0.7
57.61:0.00365)0.735.38:0.00323)0.407.12:5.4E-
4)0.854.95:0.00368,(KlbPne21:5.5E-4,(((UltBa240:5.5E-
4,(UltBa172:0.00368,(KlbPne29:0.0164,KlbPne20:0.00744)0.241.9:5.5E-
4)0.000.736:5.5E-4)0.203.9:5.5E-4,EnrSpe26:0.00743)0.132.15:5.5E-
4,UltKleb5:0.00368)0.116.8:5.5E-4)0.915.105:5.5E-
4)0.838.90:0.00443)1.000.731:5.4E-
4)0.187.8:0.00109,EnrHorm2:0.00369)0.000.737:5.6E-
4,KlbSpec2:0.0074)0.048.5:6.0E-4)0.007.6:5.4E-4,(((UltBa173:5.5E-
4,LecAdec2:0.00369)0.883.113:0.00368,(((((PntAggl2:5.5E-
4,((EnrLudwi:5.5E-
4,(((UltrBet4:0.01914,(((KlbPne27:0.0157,(KlbPne28:0.01818,KlbPne25:0.009
28)0.763.93:0.00409)0.791.67:0.00591,(KlbOxyto:0.02755,(EnrCanc4:0.01838,
(((EnrAero4:0.00675,KlbPne23:0.04711)0.794.68:0.00745,EnrCloa7:0.01175)0.
766.75:0.0037,KlbPne26:0.05552)0.771.100:0.00384)0.832.75:0.00101)0.866.1
00:0.0031)0.447.12:5.4E-
4,KlbPne22:0.026)0.750.53:0.00581)0.792.91:0.00595,((EnrCloa6:5.5E-
4,(EnrSpe14:0.00248,((UltBa238:0.00748,GmmProt5:0.01105)0.886.125:5.3E-
4,ButAgre3:5.5E-4)0.934.88:0.00245)0.139.10:7.1E-
4)1.000.732:0.00173,(((((((UltBa151:0.0,UltBa288:0.0,UltBa155:0.0,UltBa15
0:0.0,UltBa154:0.0,UltBa146:0.0,UltBa147:0.0,UltBa158:0.0,UltrBet9:0.0,Ul
tBa149:0.0):5.5E-
4,((EnrSpe21:0.00368,(EnhBacte:0.00369,(((UltBa235:0.00369,((KlbPneu4:0.0
0264,UltBa142:0.00236)0.926.120:0.00264,((((UltBa754:0.01119,((EnrCanc2:0
.00372,((((KlbPneu3:5.5E-4,((EnrSpe16:5.5E-
4,(UltBa137:0.0074,(EnrClo12:0.00386,AceSpeci:0.01323)0.579.14:5.5E-
4)0.468.16:5.5E-4)0.205.11:5.5E-4,UltGamm4:0.00367)0.274.6:5.5E-
4)0.531.7:5.5E-4,UltBa167:5.5E-4)0.221.8:5.5E-4,EnrHorm7:5.5E-
4)0.491.9:5.5E-4,KlbPne16:5.5E-4)0.316.7:5.5E-4)0.000.738:5.5E-
4,((PntSpeci:0.10711,KlbSpe10:0.10888)0.774.89:0.01003,UltGamm5:5.4E-
4)0.817.53:0.00368)0.000.739:5.5E-4)0.490.4:5.5E-
4,KlbPneu9:0.00368)0.343.7:5.5E-
4,(UltrBet3:0.00372,((EnrCanc5:0.0,UltrBeta:0.0,UltrBet5:0.0,UltBa148:0.0
):5.5E-4,((UltBa153:0.0,UltrBet6:0.0):5.5E-
4,UltBa152:0.00373)1.000.733:0.00198)0.000.740:0.00103)1.000.734:6.9E-

4)0.413.14:5.5E-4,UltStaph:0.00372)0.000.741:5.5E-4)0.611.9:5.5E-
4)0.477.12:5.5E-4,KlbPne12:5.5E-4)0.000.742:5.5E-
4,KluAscor:0.00369)0.000.743:5.5E-4)0.000.744:5.5E-4)0.000.745:5.5E-
4,(EnrClo10:0.0,BacNLA21:0.0):5.5E-4)0.452.15:5.5E-4)0.000.746:5.5E-
4,(EnrCloa2:0.0,ErwPers2:0.0,ErwSpec5:0.0,ErwPersi:0.0,EnrTuric:0.0,UltBa
181:0.0,EnmbnOf0:0.0,EnrKobei:0.0,PntAgg16:0.0,EnbBact9:0.0,EnrSpe29:0.0,
UltBa176:0.0,EnrHorm5:0.0,EnrSpe27:0.0,UltEnt13:0.0,EnrHorm6:0.0,EnrSpe20
:0.0,EnrSpe19:0.0,EnrAsbu5:0.0,UltBa168:0.0,EnrAero2:0.0,EnmbnOf2:0.0,Enr
Spe12:0.0,EnbBact3:0.0,EnrCance:0.0,ButAgres:0.0,UltBa166:0.0,EnrAsbu2:0.
0,EnrCloa5:0.0,UltEnt11:0.0,EnrCloa3:0.0,PntAgglo:0.0,EnrAsbu3:0.0,ProSym
bi:0.0,AciRadio:0.0,EnrSpe13:0.0,UltBa164:0.0,EnrHorm3:0.0,EnrAero3:0.0,R
hoPalus:0.0,UltBa163:0.0,UltBa171:0.0,EnrSpe18:0.0,GmmProte:0.0,EnrSpe24:
0.0,PntEndop:0.0,UltBa169:0.0,EnrAero5:0.0,BacCCBAU:0.0,UltCitr6:0.0,BacN
LA20:0.0,KluAsco2:0.0,UltBa237:0.0,KluInte2:0.0,KluInte3:0.0,KluInter:0.0
,UltBa236:0.0,ButSpec2:0.0,ButAgre2:0.0,BacteLMG:0.0,ButNoack:0.0,ButSpec
3:0.0,ButWarmb:0.0,EnrSpe31:0.0,UltBa197:0.0,EnrSpe35:0.0,GmmProt4:0.0,En
rSpe34:0.0,EnrSpe30:0.0,EnrAmnig:0.0,RaoOrnit:0.0,EnrSpe11:0.0,UltBa159:0
.0,UltBa102:0.0,KlbSpec4:0.0,SwnFeca2:0.0,KlbSpec6:0.0,KlbPne14:0.0,KlbPn
e15:0.0,KlbPne13:0.0,KlbPneum:0.0,UltBa144:0.0,KlbPneu2:0.0,KlbPne11:0.0,
KlbPne17:0.0,UltBa135:0.0,UltBa138:0.0,KlbPneu6:0.0,RaoTerri:0.0,EnrAero6
:0.0,EnrSpe33:0.0,UltBa233:0.0,UltBa139:0.0,EnmbnOf5:0.0,UltBa293:0.0,Ult
Ba295:0.0,UltEnt14:0.0,UltBa350:0.0,XenBovi3:0.0,UltBa752:0.0):5.5E-
4)0.000.747:5.5E-4,UltBa136:0.00369)0.217.11:5.5E-
4,KlbSpec5:0.00369)0.148.4:5.5E-4,UltErwi2:0.00369)0.284.7:5.5E-
4,KlbPneu8:0.01897)0.000.748:5.5E-4)0.000.749:5.5E-4)0.637.10:5.5E-
4,(LecAdeca:0.00367,(KlbPneu5:0.01844,KlbPne24:0.01141)0.766.76:0.00455)0
.820.57:0.0045)0.430.12:5.4E-4)0.448.12:5.4E-
4,KlbSpec3:0.00367)0.275.12:5.4E-4)0.000.750:5.4E-
4,UltBa226:0.0037)0.284.8:5.3E-4,UltBa162:0.00371)0.267.10:5.5E-
4,EnrClo14:0.00368)0.000.751:5.5E-
4,(EnrSpe17:0.04008,UltBa156:0.01381)0.935.97:0.01318)0.946.91:7.3E-
4)0.000.752:5.4E-4,UltBa143:5.5E-4)0.000.753:0.01663)0.068.6:5.3E-
4)0.188.5:0.00152,EscVulne:0.0037)0.046.7:0.00175)0.007.7:5.4E-
4)0.386.10:5.4E-4)0.469.13:5.4E-4,(EnrCanc6:0.00253,EnrSpe15:5.5E-
4)0.135.8:0.00114)0.649.12:5.5E-
4,((EnrCanc3:0.00368,PntDisp5:0.00743)1.000.735:0.00317,EnmbnOf3:5.5E-
4)0.300.11:5.1E-4)0.416.23:5.4E-4)0.041.6:5.4E-
4,((UltBa209:0.09505,((UltEnt10:0.00371,(CitGille:0.00369,(CitSpec3:0.030
93,UltBa224:0.01157)0.756.82:0.00352)0.195.6:5.2E-
4)0.994.91:0.00227,CitSpec2:0.0419)0.156.9:5.1E-
4)0.999.141:0.00101,((AchXylos:0.00733,(EnbBact2:0.00738,(SalSubte:5.5E-
4,((EscHerma:0.0,TraSpeci:0.0,TraGuam2:0.0,TraGuam3:0.0):5.5E-
4,(UltProt2:0.00369,(TraOdont:0.0,UltBa129:0.0,TraGuame:0.0,UltBa130:0.0)
:0.0037)0.706.26:5.5E-4)0.724.25:5.5E-4)0.857.107:5.5E-4)0.692.11:5.3E-
4)0.843.80:0.00364,((((((UltBa285:0.0075,(UltBa287:5.5E-
4,((UltBa280:0.0,UltBa284:0.0,UltBa283:0.0,UltBa286:0.0,UltBa282:0.0):5.5
E-4,(UltBa204:5.4E-4,(UltBa134:5.5E-
4,(UltBa225:0.0,UltBa220:0.0):0.00738)0.457.16:0.00369)1.000.736:5.1E-
4)0.437.15:5.1E-
4)0.155.9:0.0012)0.998.119:0.00143,((UltBa973:0.01116,(((((UltBa221:0.003
7,((UltBa215:0.00369,(((UltBa199:0.00369,(BacNLA23:0.0,CitFreu3:0.0):5.5E
-
4)0.842.96:0.00369,(UltBa114:0.00369,(UltCitr6:0.04123,KlbSpec9:0.00368)0
.000.754:5.5E-4)0.365.10:5.5E-4)0.334.10:5.5E-4,(UltBa214:5.4E-

4,(UltBa212:0.01099,UltBa213:5.5E-
4)0.961.82:0.01482)0.814.53:0.00369)0.457.17:5.5E-4)0.000.755:5.5E-
4,(UltBa223:0.04278,(UltBa202:0.02709,UltBa364:0.00781)0.924.103:0.00229)
0.993.73:0.00114)0.000.756:5.4E-4)0.370.10:5.5E-
4,(UltBa217:0.0,CitGill2:0.0,UltBa216:0.0,CitFreu4:0.0,UltBa201:0.0,UltBa
219:0.0,UltBa198:0.0,CitFreun:0.0,UltBa210:0.0,UltBa200:0.0,EnrSpe32:0.0,
UltBa128:0.0):5.5E-4)0.358.8:5.5E-4,(UltBa218:0.0074,UltBa759:5.5E-
4)0.851.97:0.00369)0.427.13:5.4E-4,(UltCitr7:5.4E-
4,UltBa308:0.00369)0.847.118:0.00369)0.431.17:5.5E-
4,SelBacte:0.01115)0.393.10:5.5E-4)0.000.757:5.5E-
4,CitFreu5:0.00369)0.404.15:5.5E-4)0.358.9:5.5E-
4,UltBa208:0.01131)0.053.3:5.5E-4,UltBa203:0.0074)0.165.4:5.4E-
4,CitFreu2:0.00367)0.156.10:5.4E-4,UltBa222:5.5E-4)0.082.10:5.5E-
4,Ult24812:5.5E-4)0.136.9:5.5E-4)0.531.8:5.4E-
4)0.802.63:0.0061)0.349.8:5.4E-4,UltBa174:0.01068)0.551.12:5.4E-
4,((UltBa229:0.00368,((UltBa231:0.00371,((((((EnrSpec2:0.00743,KlbOxyt7:5
.4E-
4)0.856.89:0.00366,((((SalEnte2:0.0,EnrCloac:0.0,KlbOxyt2:0.0,UltBa230:0.
0,KlbOxy11:0.0,KlbOxy10:0.0,BacNLA24:0.0):5.5E-
4,((UltBa123:0.00369,(((UltBa125:0.00369,(((UdnThrip:5.5E-
4,PntAgg17:0.01172)0.827.68:0.00694,YokRegen:0.00718)0.939.89:5.5E-
4,UltBa127:0.0037)0.028.5:5.5E-4)0.977.66:5.5E-
4,(EnrSpec4:0.0,EnrAsbur:0.0,EnrSpeci:0.0,UltBa126:0.0,UltBa124:0.0,SalEn
te4:0.0,SalEnte3:0.0,UltBa132:0.0,KlbSpec7:0.0):5.5E-4)0.732.30:5.5E-
4,PhyDiazo:0.00369)0.155.10:5.4E-
4)0.860.75:0.0037,((KlbOxyt3:0.00591,KlbOxyt4:0.00364)0.799.85:0.0058,((K
lbOxyt6:0.0142,(UltKleb2:0.01214,(EnrAsbu4:0.00737,(KlbPneu7:5.5E-
4,EnrHorm4:5.5E-4)0.804.62:5.5E-
4)0.916.116:0.00139)0.897.101:0.00276)0.680.18:5.4E-
4,KlbOxyt8:0.00404)0.850.107:0.00404)0.461.10:5.5E-4)0.348.12:5.5E-
4)0.000.758:5.4E-
4,(UltBa232:0.00209,UltBa157:0.00692)0.857.108:0.00207)0.000.759:5.5E-
4,UltKleb3:0.00369)0.373.15:5.5E-4)0.000.760:5.5E-
4,KlbSpec8:0.0037)0.455.20:5.5E-4,UltBa175:0.05462)0.332.7:5.5E-
4,PntDisp4:0.0036)0.682.18:5.5E-4,UltBa121:0.00369)0.000.761:5.5E-
4)0.214.8:5.5E-4,UltKleb4:5.5E-4)0.442.17:5.5E-4)0.554.13:5.3E-
4,KlbOxyt9:0.01937)0.271.8:0.00459)0.836.75:0.00505)0.000.762:5.5E-
4,KlbSinga:0.00362)0.866.101:0.00559,(EnrSpe10:0.00369,UltBa737:5.5E-
4)0.932.97:0.00741)0.017.6:5.4E-
4)0.791.68:0.00564)0.787.74:0.00559,(EnlEndo2:0.0037,EnlEndos:0.00373)0.9
04.106:0.0075)0.065.14:5.5E-4,GmmProt3:0.00369)0.382.11:5.5E-
4,EnpBacte:0.00365)0.882.112:0.00453,UdnThri2:5.6E-
4)0.935.98:0.01,(ButSpeci:0.0,RaoPlan2:0.0,RaoPlan3:0.0,UltBa278:0.0):5.5
E-4)0.001.7:5.5E-4,UltBa279:0.00735)0.826.64:0.00507,(((XenNema5:5.5E-
4,(UltBa267:0.00744,((SrrSpec3:0.01153,(((((UltBa249:0.00369,((UltBa101:0
.0,SrrMarc2:0.0,UltBa251:0.0,SrrMarc5:0.0,SrrMarc7:0.0,SrrMarc8:0.0,UltBa
253:0.0,SrrSpec4:0.0,SrrSpeci:0.0,SrrSpec2:0.0):5.5E-
4,(((((((SodGloss:5.4E-
4,((PrmEndo4:0.02379,SdlSymbi:0.02396)0.753.78:0.00422,((ScnEndo6:0.0194,
UltSoda2:0.02329)0.772.86:0.00443,PrmEndo8:0.0219)0.375.12:0.00752)0.778.
86:0.00272)0.890.105:5.4E-
4,(BiosTofi:0.00387,PrmEnd10:0.00728)0.142.9:0.0037)0.875.117:0.00656,(Ye
rSpec2:0.01166,UltProt6:0.03962)1.000.737:5.5E-
4)0.015.6:0.00374,((PrmEndo5:0.02641,(PrmEndo7:0.00367,(EnmbnO18:0.02161,

(SitZeama:0.13358,PrmEndo6:5.4E-
4)0.250.11:0.00809)0.760.76:0.00463)0.792.92:0.00493)0.813.66:0.00274,Ult
Ba358:0.00241)0.798.70:0.00287)0.782.100:0.00406,(ScnEndo9:0.01947,ScnEnd
10:0.03619)0.836.76:0.00727)0.890.106:0.00833,AquHaywa:5.5E-
4)0.823.65:0.00407,SrrMarc4:5.5E-4)0.633.7:5.4E-4)0.075.4:5.4E-
4)0.059.9:5.5E-4,UdnBact2:0.00369)0.267.11:5.5E-
4,SrrMarce:0.00369)0.413.15:5.5E-4,SrrMarc3:0.00368)0.075.5:5.5E-
4,SrrMarc6:0.03063)0.323.8:5.5E-4)0.771.101:5.4E-4,(((EscAlbe2:5.5E-
4,((UltBa145:0.00209,UltBact9:0.01088)0.904.107:0.00193,((UltBac93:5.4E-
4,UltBac77:0.01516)0.828.58:0.00368,(BacNLA14:5.5E-4,((UltBac22:5.5E-
4,((((((UltBacte:5.5E-
4,UltBac79:0.03367)0.837.64:0.00358,(((UltBac13:0.00753,UltBa205:0.02715)
0.768.76:0.00364,((((BacNLA10:0.00365,((UltBa312:0.0037,((UltBac67:0.0036
9,((UltBa311:0.00369,((UltBac43:0.00369,(((((ShiFlexn:0.00742,UltBac21:0.
00247)1.000.738:0.00121,(EscFergu:0.00742,(((UltBac84:0.0144,(Ult17362:0.
11267,(UltBac34:5.3E-
4,(UltBac32:0.07839,UltBac35:0.03131)0.959.94:0.02754)0.998.120:0.05143)0
.111.6:5.5E-4)0.392.11:5.4E-4,UltBact3:0.00369)0.428.13:5.5E-
4,(UltBac50:0.00943,(UltBac49:0.02282,UltBac39:0.03491)0.682.19:0.01038)0
.782.101:0.00534)0.841.82:0.00467)0.354.12:5.5E-4)0.000.763:5.4E-
4,((((EschCol6:5.5E-4,((((UltBa299:0.00368,(((((((UltBac88:5.5E-
4,((EschCol7:5.5E-
4,((UltBac56:0.00371,DfvSpeci:0.04989)0.934.89:0.00242,(UltBac48:0.00815,
BacNLAEz:0.00366)0.932.98:0.00269)0.930.90:0.00291)0.699.24:5.3E-
4,UltEnte2:5.5E-4)0.451.17:5.5E-4)0.000.764:5.5E-
4,(UltBac64:0.0,UltBac62:0.0,UltBac59:0.0,UltBac60:0.0,UltBac61:0.0):5.5E
-4)0.000.765:5.5E-4,UltBac66:0.00369)0.000.766:5.5E-
4,UltBac95:0.00369)0.000.767:5.5E-4,(UltBac65:5.5E-
4,UltShig2:0.00382)0.840.96:0.00369)0.000.768:5.5E-
4,UltBac68:0.0037)0.000.769:5.5E-
4,((UltBac89:0.0,UltBac90:0.0,UltBac42:0.0,UltBac29:0.0,UltBac31:0.0,UltB
ac91:0.0,UltBac46:0.0,EschCol2:0.0,UltBac40:0.0,UltBact5:0.0,SwnFecal:0.0
,UltBac18:0.0,UltBac53:0.0,UltBac33:0.0,UltBac58:0.0,BacNLA13:0.0,CroSaka
z:0.0,UltBac37:0.0,UltBac38:0.0,UltBact6:0.0,EschCol8:0.0,UltBact7:0.0,Es
cFerg2:0.0,UltBac12:0.0,UltBac19:0.0,UltBac80:0.0,BacNLAE5:0.0,UltBac81:0
.0,EschCol4:0.0,UltBact4:0.0,UltBac72:0.0,UltGamma:0.0,UltBac87:0.0,BacNL
A16:0.0,BacNLA17:0.0,EschCol3:0.0,UltBac96:0.0,UltBac11:0.0,UltEnter:0.0,
ShiSonne:0.0,UltBac51:0.0,UltBac41:0.0,UltBac52:0.0,UltBac54:0.0,BacNLAE2
:0.0,EschCol9:0.0,BacNLAE7:0.0,BacNLAE9:0.0,UltBac24:0.0,UltBac28:0.0,Ult
Bac27:0.0,UltBa298:0.0,UltBa302:0.0,UltBa290:0.0,UltBa301:0.0,UltBa292:0.
0,UltBa756:0.0,UltBa757:0.0,UltBa758:0.0,Ult24981:0.0):5.5E-
4,((((UltBac85:0.00732,UltBac78:0.04945)0.930.91:0.00359,(UltBac15:0.0422
,UltBac10:0.03154)0.434.7:0.00758)1.000.739:5.5E-
4,(UltEnte3:0.00691,(UltBa291:0.07154,UltBac44:5.4E-
4)0.887.109:0.00214)0.889.98:0.00214)0.073.4:5.5E-
4,(UltBac14:0.01572,(UltBac57:0.01294,UltBac16:0.05662)0.304.8:0.00641)0.
864.102:0.00794)0.368.7:5.5E-4)0.000.770:5.3E-4)0.638.12:5.5E-
4)0.000.771:5.4E-4,EschCo10:0.0074)0.000.772:5.5E-
4,UltBac55:0.00369)0.000.773:5.5E-
4,(UltBac70:0.0,UltBac76:0.0):0.00369)0.411.12:5.5E-4)0.065.15:5.5E-
4,(UltBac30:0.0261,UltBac73:0.04233)0.743.61:0.00455)0.262.9:5.5E-
4,BacNLA15:5.5E-4)0.156.11:5.5E-4,UltBac82:5.5E-4)0.000.774:5.5E-
4)0.642.11:5.5E-4,BacNLA12:5.5E-4)0.000.775:5.4E-
4,(UltBac92:0.01482,UltBa300:0.00715)0.674.19:5.4E-4)0.000.776:5.5E-

4)0.463.13:5.4E-4,(UltBact2:5.3E-
4,Ult21326:0.00369)0.853.83:0.00368)0.000.777:5.5E-4)0.000.778:5.5E-
4,UltBac97:0.00369)0.468.17:5.5E-4)0.195.7:5.5E-4,BacNLA19:5.5E-
4)0.000.779:5.5E-4)0.373.16:5.5E-4,UltBac99:0.00372)0.387.12:5.5E-
4)0.071.7:5.5E-4,EschCol5:0.00735)0.000.780:5.5E-4,EschColi:5.5E-
4)0.000.781:5.4E-4,UltBac94:0.00369)0.396.21:5.5E-4)0.464.14:5.5E-
4,(UltBac71:0.01863,UltBact8:5.5E-4)0.841.83:0.00361)0.102.6:5.5E-
4)0.000.782:5.4E-4,(UltBac83:5.5E-4,(BacNLAE3:5.5E-4,BacNLAE8:5.5E-
4)1.000.740:0.00316)0.999.142:5.1E-4)0.000.783:5.5E-4,(BacNLA18:5.5E-
4,BacNLA11:5.5E-4)0.914.121:0.00366)0.534.11:5.5E-
4,(UltBac20:0.00374,((UltBa104:5.5E-
4,UltBa108:0.00366)0.844.105:0.00352,((UltBa109:5.5E-
4,(UltBa107:0.00366,(UltBa105:0.01112,((UltEnte7:5.5E-
4,(UltBa160:0.00371,(UltBa228:0.00369,UltBa361:5.5E-
4)1.000.741:0.00219)0.129.7:7.7E-4)1.000.742:7.1E-4,UltBa103:5.5E-
4)0.523.8:5.5E-4)0.110.7:5.5E-4)0.866.102:5.5E-4)0.757.62:5.5E-
4,(((UltBac36:0.0037,UltBa161:0.00369)0.504.11:5.5E-
4,UltBa106:0.00734)0.813.67:5.5E-4,UltBa110:0.00722)0.059.10:5.5E-
4)0.914.122:5.5E-4)0.780.99:0.00339)0.803.62:0.00339)0.032.4:5.5E-
4,(UltBac86:0.01017,(UltBac17:0.02296,(UltBac75:0.04698,UltBac74:0.04376)
0.810.65:0.00765)0.782.102:0.00562)0.773.91:0.00575)0.000.784:5.5E-
4)0.531.9:5.5E-4,(BacNLAE6:5.1E-4,(UltBac69:0.04216,BacNLAE4:5.5E-
4)0.848.86:5.4E-4)0.926.121:0.0036)0.091.4:5.4E-4)0.000.785:5.5E-
4)0.357.11:5.4E-4)0.679.20:5.5E-4)0.189.8:5.5E-
4,(UltBac63:0.00413,UltShige:0.08301)0.780.100:0.00349)0.924.104:0.00686,
((UltBa234:5.5E-
4,(((((((PntDispe:0.0,CroSaka5:0.0,UltEnte9:0.0,CroTuri3:0.0,CroTuric:0.0
):5.5E-4,((((((UltPant4:0.0,UltPant5:0.0,UltPant3:0.0):5.5E-
4,SymbiOf0:0.00719)0.977.67:0.01888,(UltBa178:0.01139,EnbBact7:0.01118)0.
264.5:0.00364)0.587.12:5.4E-
4,(PntDisp2:0.00369,(UltBa180:0.00369,((UltBa182:0.00694,((((UltBa362:0.0
074,((UltBa248:5.5E-4,(UltBa245:0.02265,(UltBa244:0.00371,(SrrFicar:5.5E-
4,(ErwTolet:0.00367,UltBa243:0.00369)0.795.78:0.00382)0.388.10:5.5E-
4)0.209.10:5.5E-4)0.435.12:5.5E-4)0.000.786:5.5E-4,UltBa242:5.5E-
4)0.000.787:5.4E-4)0.409.13:5.5E-
4,((UltBa294:0.00369,PntAnana:0.0037)0.254.6:5.4E-
4,UltBa363:0.00369)0.858.96:0.00369)0.366.10:5.5E-
4,(UltBa246:0.00691,PsdFluor:0.00209)0.867.107:0.00206)0.299.14:5.4E-
4,BrnQuerc:0.00361)0.896.96:0.00222)0.881.114:0.00223,(PntStewa:0.00369,(
(PntSpe14:0.0,FlaSpeci:0.0,PntSpe10:0.0,PntDisp3:0.0,ErwSpeci:0.0,EnhBact
2:0.0,PntAnan3:0.0,PntAnan4:0.0,PntAnan5:0.0,PntAnan2:0.0,PntSpec7:0.0,Ul
tPant2:0.0,PntStew2:0.0,PntStew3:0.0,PntStew4:0.0):5.5E-4,PntSpec9:5.5E-
4)0.000.788:5.5E-4)0.000.789:5.5E-4)0.592.11:5.5E-4)0.389.8:5.5E-
4)0.000.790:5.5E-4)0.170.8:5.5E-4,EnrPyrin:0.00369)0.045.7:5.4E-
4,(EnbBact4:0.01136,EnbBact5:0.00368)0.777.99:0.00375)0.871.103:0.00441,(
((BactE440:0.0,EnrPulv2:0.0,EnrPulve:0.0):5.5E-
4,((EnrHelv2:0.01117,EnrHelve:5.5E-
4)0.847.119:0.00369,PntOleae:0.00369)0.657.14:5.5E-4)0.757.63:5.5E-
4,UltBa190:0.0037)0.839.87:0.00369)0.149.12:5.3E-4)0.002.5:5.4E-
4,CroSaka8:5.5E-
4)0.799.86:0.00507,(UltBac47:0.07042,UltBa250:0.00947)0.832.76:0.00985)0.
769.84:0.0038,UltBa179:0.02279)0.193.5:5.5E-4,(CroSaka3:5.5E-
4,(ErwYyyyy:0.01167,((EscAlbe3:0.0,EscAlber:0.0):0.00744,((CroSaka6:0.0,C
roSaka7:0.0,CroSaka4:0.0,CroMalon:0.0):5.5E-

4,(EscBlat2:0.0,EscBlatt:0.0,UltEnte4:0.0):0.0037)0.509.9:5.4E-
4)0.871.104:0.00757)0.339.13:0.00371)1.000.743:0.00378)0.436.11:5.5E-
4,UltBa281:0.00373)0.439.12:5.5E-4)0.000.791:5.4E-
4,(PntSpe13:0.0,CroDubl2:0.0,CroDubli:0.0,CroTuri2:0.0,KluGeorg:0.0,UltBa
100:0.0):5.5E-4)0.949.98:5.4E-
4)0.834.67:0.00372)0.845.97:0.0048)0.000.792:5.4E-
4)0.800.70:0.0048,(UltBa409:0.01084,(((UltBa393:0.01083,(UltBa376:5.5E-
4,(UltBa390:0.1663,UltBa391:0.01114)0.754.68:0.0037)0.933.113:0.00196)0.9
29.94:0.00199,(((UltBa377:5.5E-
4,((((((UltBa585:0.00369,((UltBa550:0.00369,((UltBa303:0.0,UltBa373:0.0,U
ltBa374:0.0,UltBa375:0.0,UltBa380:0.0,UltBa382:0.0,UltBa384:0.0,UltBa385:
0.0,UltBa387:0.0,UltBa389:0.0,UltBa392:0.0,UltBa394:0.0,UltBa395:0.0,UltB
a398:0.0,UltBa407:0.0,UltBa400:0.0,UltBa401:0.0,UltBa402:0.0,UltBa411:0.0
,UltBa541:0.0,UltBa549:0.0,UltBa581:0.0,UltBa582:0.0,UltBa584:0.0,UltBa61
0:0.0,UltBa673:0.0,UltBa674:0.0):5.5E-
4,((((((UltBa260:0.00739,(HafSpeci:5.5E-
4,(((BrnQuer2:0.00371,(BrnQuer3:5.5E-4,EnrSpe36:5.5E-4)0.765.74:5.5E-
4)0.839.88:0.00369,(((UltSerra:0.0,SrrFonti:0.0,UltRahn7:0.0,RahSpeci:0.0
,BacSV26I:0.0,RahAqua4:0.0,UltRahn3:0.0,UltRahn5:0.0,UltRahn6:0.0,RahAqua
3:0.0,UltBa271:0.0,YerRucke:0.0,YerSpeci:0.0,SrrPlym2:0.0):5.5E-
4,(((UltBa258:0.00368,(UltProt4:0.00369,((RahAqua2:5.5E-
4,UltBa266:0.00714)0.830.74:5.5E-4,(((((UltBa263:5.5E-4,UltRahne:5.5E-
4)0.913.119:0.00368,(((((((Ult24843:0.01123,Ult24982:5.4E-
4)1.000.744:0.05165,UltBa269:0.00224)0.994.92:0.00153,(((UltrBet7:5.5E-
4,(((BudAquat:5.5E-
4,UltBa353:0.00369)0.905.110:0.00742,(((PntAggl8:0.05386,UltSerr2:0.09539
)0.939.90:0.02366,PsdPutid:0.00744)0.866.103:0.00181,SrrProte:0.00751)0.8
97.102:0.0025)0.459.12:5.4E-
4,(UltArani:0.0,SrrSpec5:0.0,UltProt3:0.0,SrrQuin2:0.0,SrrProt2:0.0,UltBa
259:0.0,SrrGrime:0.0,SrrSpec6:0.0,SrrSpec7:0.0,EwiAmer2:0.0,RahAquat:0.0,
UltBa265:0.0,UltBa270:0.0,YerEnter:0.0,YerAlek3:0.0,YerAleks:0.0,YerAlek2
:0.0,YerKrist:0.0,YerPesti:0.0,YerSimil:0.0):5.5E-4)0.438.13:5.5E-
4)0.000.793:5.4E-4,UltBa262:0.01874)0.000.794:5.5E-
4,(((SrrRubi2:0.0,SrrRubid:0.0,UltBa252:0.0,SodGlos2:0.0):5.5E-
4,(UltBa247:0.00367,SrrRubi3:0.07586)0.393.11:5.0E-
4)0.931.110:0.00856,(SrrFica2:0.0,SrrFica3:0.0,SrrEntom:0.0,SrrOdori:0.0)
:5.4E-4)0.925.113:0.00855)0.800.71:5.5E-4)0.378.16:5.5E-
4,(UltBa264:0.01492,YerFrede:5.5E-4)0.596.9:5.5E-4)0.092.8:5.5E-
4,UltBa261:0.01116)0.167.9:5.5E-
4,(GmmProt6:0.03064,ScnSymb2:0.01104)0.783.94:5.5E-4)0.142.10:5.5E-
4,SrrLique:5.5E-4)0.159.5:5.5E-4,UltBa268:5.5E-4)0.438.14:5.5E-
4)0.188.6:5.4E-4,(SrrPlymu:0.00365,SrrQuini:5.5E-
4)0.920.114:0.00735)0.930.92:0.00895,(RahAqua7:0.00246,(RahAqua5:5.5E-
4,RahAqua6:0.00742)0.920.115:0.00245)0.920.116:0.00246)0.000.795:5.5E-
4,YerRuck2:0.0037)0.441.15:5.5E-4)0.000.796:5.5E-4)0.392.12:5.5E-
4)0.388.11:5.5E-4,RahAqua8:0.00374)0.076.5:5.5E-
4,EwiAmeri:0.0074)0.442.18:5.5E-4)0.000.797:5.5E-
4,UltBa257:0.00369)0.000.798:5.5E-4)0.438.15:5.5E-
4,(UltRahn4:0.00741,UltRahn2:5.5E-4)0.840.97:0.00369)0.375.13:5.5E-
4)0.395.16:5.5E-4)0.889.99:0.0043,(((UltBa366:5.5E-
4,UltBa405:0.06393)0.963.75:0.01584,UltBa406:5.4E-4)1.000.745:5.4E-
4,UltBa274:0.00365)0.133.8:0.00367)0.956.90:5.5E-
4,UltBa410:0.0074)0.246.16:5.5E-4,UltBa381:0.0037)0.000.799:5.5E-
4,(UltBa671:0.00369,((UltBa370:0.0,UltBa583:0.0):5.5E-

4,((UltBa372:0.00369,UltBa586:5.5E-
4)0.851.98:0.00369,(UltBa383:0.00369,UltBa367:0.00371)0.000.800:5.5E-
4)0.856.90:0.00369)0.147.8:5.4E-4)0.844.106:0.00369)0.000.801:5.4E-
4,(UltBac98:0.0,UltBa396:0.0,UltBa399:0.0,UltBa762:0.0):0.0037)0.451.18:5
.5E-4)0.000.802:5.5E-4)0.451.19:5.5E-4,UltBa412:5.5E-4)0.118.11:5.5E-
4)0.385.17:5.5E-4,UltBa710:0.0074)0.000.803:5.5E-
4,UltBa408:0.00369)0.282.8:5.5E-4,UltBa371:0.00739)0.000.804:5.3E-
4,(UltBa277:0.00369,(UltBa388:0.01117,UltBa404:0.01506)0.566.10:5.5E-
4)0.842.97:0.00369)0.400.11:5.5E-4,UltBa672:0.00368)0.148.5:5.5E-
4)0.269.9:5.5E-4,UltBa378:0.03004)0.418.12:5.5E-
4,(UltBa369:0.01104,(UltBa368:0.01361,(UltBa379:0.0398,UltBa386:0.05767)0
.181.5:0.00438)0.771.102:0.00857)0.939.91:5.4E-4)0.604.13:5.5E-
4)0.012.3:5.5E-4,UltBa397:0.01874)0.526.11:5.5E-
4)0.928.82:0.00718)0.994.93:5.4E-
4,((BacYSS20:0.00767,((((((EdwTard2:0.0,EdwHoshi:0.0,EdwIctal:0.0,EdwTard
a:0.0):5.5E-
4,UltBa254:0.00741)0.952.85:0.01516,(((UltPecto:0.0,UltPect2:0.0,UltPect3
:0.0):5.5E-
4,(CanRohrk:0.00363,UltBa256:0.03051)0.758.75:0.00374)0.984.69:5.5E-
4,((PecCacti:0.00368,((BrnnAln2:0.0,PecCaro2:0.0,PecCaro3:0.0,UltBa255:0.
0,PecCarot:0.0,PecBeta2:0.0,PecBetav:0.0,PecBeta3:0.0,PecAtro2:0.0,PecAtr
o3:0.0,PecAtros:0.0,PecWasa2:0.0,PecWasab:0.0):5.5E-
4,PecCaro4:0.01116)0.998.121:5.5E-4)0.833.83:0.00745,((((ErwChry3:5.5E-
4,((BrnRubr3:0.0,BrnRubri:0.0,BrnRubr2:0.0):0.00369,BrnSalic:0.01523)0.76
7.76:0.00373)0.000.805:5.5E-
4,(BrnNigri:0.0,ErwChry2:0.0,DicDada2:0.0,DicDiant:0.0,DicPara2:0.0,DicPa
rad:0.0,DicDadan:0.0,ErwChrys:0.0,DickZea2:0.0,DickZeae:0.0):5.5E-
4)0.000.806:5.5E-4,DicChrys:0.00369)0.986.74:5.3E-
4,((UltBa289:0.00739,UltBa296:0.00365)0.678.18:5.5E-
4,UltBa305:0.0185)0.831.67:0.0036)0.804.63:5.4E-
4)0.970.79:0.01104)0.748.66:0.00356)0.969.63:0.0224,UltBa359:0.00739)0.92
0.117:5.3E-4,PraFonti:0.00369)0.809.74:5.4E-
4,((EtrDtb11:0.01528,((UltBa272:0.0,UltrBet8:0.0,UltBa273:0.0,UltrBe10:0.
0,UltrBet2:0.0):5.5E-
4,((UltBa276:0.0,HafAlvei:0.0,ObeProte:0.0,ObeProt2:0.0,UltBa304:0.0):5.5
E-4,(UltBa275:0.00369,UltBa365:0.00369)0.912.134:5.5E-4)0.758.76:5.5E-
4)0.949.99:0.01529)0.875.118:0.00742,((LemGrimo:5.4E-
4,(UltBa351:0.00372,UltBa352:0.01125)0.901.110:0.00744)0.869.83:0.00369,(
((((((UltBa349:0.0,PrnRusti:0.0):5.5E-
4,((PrnAlcal:0.00197,PrnSpec2:0.01478)0.926.122:0.00194,(((PrnSpeci:0.008
14,AchSpeci:0.0117)0.513.8:5.5E-4,UltBa348:0.0037)0.411.13:5.5E-
4,UltBa347:0.00369)0.473.11:5.5E-4)0.000.807:5.5E-4)0.519.8:5.4E-
4,((BacNLA39:5.5E-4,((MorMorga:0.0,MorMorg2:0.0,PhhLumi8:0.0):5.5E-
4,(UltrBe11:5.5E-4,MorSpeci:0.01119)0.753.79:5.5E-4)0.883.114:5.5E-
4)0.874.127:0.00751,(MorPsych:5.5E-
4,UltMorga:0.00371)0.805.61:0.00363)0.962.96:0.01513)0.305.10:5.5E-
4,PrnRettg:0.00738)0.559.10:5.3E-4,((SwnManu2:0.02719,((((UltProt5:5.5E-
4,(CanArsen:0.01109,(ArsSymbi:0.01558,EubaFrom:0.02725)0.387.13:0.0072)0.
882.113:5.5E-4)0.887.110:5.5E-4,((UltGam32:0.00371,UltBa343:5.5E-
4)0.850.108:0.00369,CanPhlom:0.02271)1.000.746:5.5E-4)0.951.97:5.5E-
4,ArsEndo2:0.01101)0.786.81:0.00359,ArsEndos:0.00375)0.855.94:0.00735)0.9
17.97:0.01104,((((BacNLA31:5.4E-4,(BacNLA28:0.00368,((BacNLA32:5.5E-
4,((((BacNLA38:0.00369,PrtMira2:0.02669)0.724.26:5.5E-
4,(BacNLA25:0.0,BacNLA26:0.0,UltBa344:0.0,BacNLA35:0.0,BacNLA36:0.0,PrtPe

nne:0.0):5.5E-4)0.000.808:5.5E-4,(PrtMirab:5.5E-4,SwnManur:5.5E-4)0.000.809:5.5E-4)0.000.810:5.5E-4,(PrtPenn2:5.5E-4,((SwnFeca3:0.0,UltBa345:0.0,PrtHause:0.0,PrtVulga:0.0):0.00369,((BacNLA30:0.00354,BacNLA34:5.5E-4)0.825.68:0.00385,(BacNLA29:5.5E-4,(BacNLA33:5.5E-4,BacNLA27:5.5E-4)0.416.24:5.5E-4)0.858.97:0.00388)0.501.11:5.5E-4)0.412.11:5.5E-4)0.000.811:5.5E-4)0.150.7:5.5E-4)0.000.812:5.5E-4,BacNLA37:0.00368)0.119.6:5.5E-4)0.847.120:0.00401)0.967.71:0.02583,((XenHomin:0.00745,(XenNema4:5.4E-4,(XenBovie:0.0,XenBovi2:0.0):0.0037)0.815.66:0.00375)0.833.84:0.00356,((XenStoc2:5.5E-4,XenStock:0.00368)0.889.100:0.00739,((((XenSpec2:0.03471,XenKozod:0.01108)0.860.76:5.4E-4,XenIndic:0.00372)0.896.97:0.00752,(((XenBuda2:0.0,XenBudap:0.0):0.0037,((((XenNema2:0.0,XenJapon:0.0):5.5E-4,(XenJapo2:0.00746,XenJapo3:5.5E-4)0.845.98:0.0037)1.000.747:5.3E-4,((XenNemat:0.0,XenSpeci:0.0):5.5E-4,(XenNema3:5.5E-4,(XenDouce:0.0,XenRoman:0.0):0.00209)1.000.748:0.0016)0.858.98:0.00362)0.758.77:0.00744,(XenKoppe:5.5E-4,(XenCaba2:0.0,XenCaban:0.0):0.0037)1.000.749:5.4E-4)0.866.104:0.00362)0.776.88:5.5E-4,(XenBedd2:0.0,XenBeddi:0.0,XenSzent:0.0):5.5E-4)0.996.94:5.4E-4)0.823.66:0.00368,(((XenEhler:0.0037,XenGriff:5.4E-4)0.863.113:0.0037,(XenInnex:0.00746,((PhhSpec3:0.015,((PhhLumi5:0.0,PhhSpeci:0.0,PhhTemp3:0.0):5.5E-4,((((PhhLumi6:0.0,PhhLumi7:0.0):5.5E-4,(PhhLumin:5.5E-4,(PhhLumi4:0.0037,(PhhLumi2:0.0037,PhhLumi3:5.5E-4)0.879.112:0.00371)0.245.10:5.4E-4)0.841.84:0.0037)0.847.121:0.0037,(PhhAsym3:5.5E-4,(PhhAsym2:5.5E-4,PhhAsymb:0.00369)0.840.98:0.00369)0.708.28:5.5E-4)0.935.99:0.00746,(PhhTempe:0.0,PhhTemp2:0.0):0.00744)0.822.58:5.5E-4)0.906.112:5.4E-4)0.238.10:0.00369,(PhhSpec2:0.0,PhhTemp4:0.0):5.4E-4)0.809.75:0.00396)0.152.12:5.5E-4)0.599.10:5.4E-4,(XenPoina:0.0,XenPoin2:0.0,XenMiran:0.0):5.5E-4)0.592.12:5.1E-4)0.865.102:5.4E-4)0.725.31:5.5E-4)0.833.85:0.0171)0.824.63:0.00753,UltBa346:0.00369)0.273.11:5.4E-4,(PrnStua2:0.0,PrnStuar:0.0):5.5E-4)0.934.90:5.4E-4)0.943.96:0.0072)0.823.67:0.00403,MoeWisco:0.00369)0.183.10:5.4E-4,(PrnHeimb:0.0,UltProvi:0.0):0.00742)0.960.91:0.0123)0.374.16:5.3E-4)0.962.97:0.01669)0.293.8:0.00396)0.933.114:0.0122,(EnbBac17:5.4E-4,UltBa193:0.04388)0.847.122:5.4E-4)0.851.99:0.00768)0.843.81:0.00481)0.204.5:5.3E-4,(((ErwPapay:0.0037,SamEryth:5.5E-4)0.832.77:0.00353,(((PntSpec2:5.5E-4,(PsdFlect:0.0037,((UltBa207:0.00751,RaoOrni2:0.00434)0.466.13:5.5E-4,(UltBa206:0.0,RaoOrni3:0.0,BacSV6XV:0.0,RaoPlant:0.0,UltBa239:0.0):5.5E-4)0.411.14:5.5E-4)0.852.95:0.00418)0.446.10:0.00425,(((PntAggl6:5.5E-4,((((PntSpec3:0.03464,EnrSpe28:0.04292)0.887.111:5.5E-4,(PntAgg13:5.5E-4,PntSpec6:0.00369)0.000.813:5.5E-4)0.000.814:5.5E-4,(ErwMallo:0.0,PntAggl3:0.0,UltPanto:0.0,PntAgg10:0.0,PntAgg15:0.0,PntAgg11:0.0,PntAgg12:0.0,PntSpec4:0.0,PntAggl5:0.0,UltBa177:0.0,PntAgg14:0.0,GmmProt2:0.0,PntSpec5:0.0,UdnBacte:0.0,PntAggl7:0.0):5.5E-4)0.000.815:5.5E-4,(ErwTrach:0.00369,ErwPsidi:0.00369)0.657.15:5.5E-4)0.870.86:5.5E-4)0.334.11:5.4E-4,(PntAggl4:5.5E-4,PntAggl9:5.5E-4)0.939.92:5.5E-4)0.958.97:0.00192,UltPant6:0.01479)0.937.98:0.00188)0.760.77:5.4E-4,(((PntCede2:0.0,PntCeden:0.0,ErwSpec6:0.0,ErwAmyl6:0.0):5.5E-

4,MrgVenez:5.5E-4)0.984.70:5.4E-4,((UltBa310:0.00742,(UltBa184:5.5E-
4,UltBa183:5.5E-4)0.779.80:5.5E-
4)0.955.75:0.00743,(BactIps7:0.00368,((UltBa187:0.0,UltBa188:0.0):0.00369
,PntSpe11:0.01124)0.423.15:5.5E-4)0.797.66:5.5E-
4)0.841.85:0.00367)0.857.109:0.00366)1.000.750:5.5E-
4)0.192.9:0.00845,BrnnAlni:0.01509)0.792.93:0.00436)0.683.25:0.0024,(SrrM
arc9:0.0056,(UltBa675:0.00752,(((UltBa635:0.01156,UltBa645:0.05648)0.448.
13:0.01077,(((UltBa532:0.03013,(UltBa530:0.0622,UltBa531:0.05767)0.927.10
4:0.02185)0.870.87:0.00743,UltBa642:0.02695)0.865.103:0.00731,((UltBa617:
0.0148,(UltBa428:0.01898,UltBa651:0.02262)0.863.114:0.01114)0.720.27:5.5E
-4,UltBa621:0.06386)0.390.17:5.3E-4)1.000.751:5.3E-
4)0.791.69:0.00361,((UltBa568:0.03425,(UltBa415:5.5E-
4,(UltBa418:0.02655,(UltBa600:5.5E-4,(UltBa599:5.5E-
4,UltBa417:0.00745)0.853.84:0.00369)0.847.123:5.4E-
4)0.880.117:0.00367)0.909.90:0.01154)0.760.78:0.00329,((UltBa644:0.0148,(
UltBa567:0.02223,(UltBa576:0.02269,UltBa657:0.03442)0.972.81:0.00215)0.97
8.62:0.00164)0.811.64:0.00361,((((UltBa661:0.00367,((UltBa633:0.00368,(((
(UltAerom:5.5E-
4,(((UltBa664:0.05749,(UltBa622:0.01956,UltBa623:0.04767)0.814.54:0.00895
)0.775.81:0.0056,((((((UltBa634:0.00375,(UltBa659:0.0038,(UltBa534:0.0042
9,(UltBa554:0.01854,(UltBa619:0.08389,(UltBa430:0.10165,UltBa553:0.03467)
0.837.65:0.05279)0.031.4:0.03587)0.986.75:0.05579)0.993.74:0.04304)0.960.
92:0.02629)0.785.86:0.00362,((BacNLA40:5.5E-
4,((UltBa660:0.00369,((UltBa639:0.00742,(((((((UltBa577:0.01473,UltBa652:
0.00197)0.955.76:0.00194,(((UltBa537:0.0037,(UltBa603:0.02806,UltBa713:5.
5E-4)0.906.113:0.00776)0.781.81:0.00385,UltBa433:0.0074)0.148.6:5.3E-
4,UltBa431:0.00368)0.491.10:5.5E-4)0.000.816:5.5E-
4,(((UltBa425:0.0,UltBa564:0.0,UltBa604:0.0,UltBa638:0.0,UltBa761:0.0):5.
5E-
4,UltBa640:0.00369)0.920.118:0.0036,((((UltBa545:0.0,UltBa678:0.0):5.5E-
4,UltBa562:0.00369)0.710.27:5.5E-
4,UltBa555:0.01878)0.920.119:0.0036,(UltBa676:0.0113,((UltBa413:0.0,UltBa
449:0.0,UltBa561:0.0,UltBa712:0.0):5.5E-
4,(UltBa559:0.00369,UltBa590:0.00369)0.686.13:5.5E-4)0.741.56:5.4E-
4)1.000.752:5.3E-4)0.105.11:0.00369)0.999.143:5.4E-4)0.417.16:5.2E-
4,AerHydr4:0.01124)0.000.817:5.4E-4,(((((UltBa620:0.05769,UltBa608:5.5E-
4)0.779.81:0.00368,UltBa643:0.11898)0.782.103:0.00383,((UltBa539:5.5E-
4,(((UltBa656:0.0,UltBa666:0.0):5.5E-
4,UltBa665:0.00742)0.833.86:0.00369,(UltBa421:0.00172,UltBa601:0.01143)0.
920.120:0.00237)0.259.5:5.5E-4)0.314.10:5.5E-4,UltBa578:5.5E-
4)0.851.100:0.00386)0.078.11:5.5E-4,UltBa533:0.00369)0.000.818:5.4E-
4,(AerSpec2:0.01498,(UltBa538:0.00741,(AerSpeci:0.0,AerSobri:0.0,AerSobr2
:0.0):5.5E-4)0.965.75:0.00197)0.960.93:0.00195)0.000.819:5.5E-
4)0.564.6:5.5E-4,UltBa615:0.0037)0.000.820:5.5E-
4,UltBa655:0.00369)0.000.821:5.5E-4)0.468.18:5.4E-
4,(UltBa403:0.0,UltBa426:0.0,UltBa427:0.0,UltBa414:0.0,UltBa422:0.0,UltBa
423:0.0,UltBa424:0.0,UltBa429:0.0,UltBa432:0.0,UltBa434:0.0,UltBa451:0.0,
UltBa535:0.0,UltBa536:0.0,UltBa540:0.0,UltBa543:0.0,UltBa544:0.0,UltBa546
:0.0,UltBa548:0.0,AerVeron:0.0,AerHydr2:0.0,UltBa551:0.0,UltBa557:0.0,Ult
Ba558:0.0,AerJanda:0.0,UltBa566:0.0,UltBa569:0.0,UltBa570:0.0,UltBa572:0.
0,UltBa573:0.0,UltBa587:0.0,UltBa588:0.0,UltBa589:0.0,UltBa602:0.0,UltBa6
05:0.0,UltBa606:0.0,UltBa612:0.0,UltBa613:0.0,UltBa609:0.0,UltBa611:0.0,U
ltBa616:0.0,UltBa624:0.0,UltBa629:0.0,UltBa630:0.0,UltBa632:0.0,AerHydr3:
0.0,AerSalmo:0.0,AerSpec3:0.0,AerHydr5:0.0,UltBa636:0.0,AerHydr6:0.0,AerH

ydr7:0.0,UltBa646:0.0,UltBa648:0.0,UltBa649:0.0,UltBa653:0.0,AerSpec5:0.0
,UltBa662:0.0,BacNLA41:0.0,UltBa663:0.0,UltBa667:0.0,UltBa668:0.0,UltBa66
9:0.0,UltBa670:0.0,UltBa677:0.0,UltBa680:0.0,UltProt9:0.0,UltBa706:0.0,Ul
tBa707:0.0,UltBa708:0.0,UltBa709:0.0,UltBa711:0.0):5.5E-4)0.141.7:5.5E-
4)0.470.6:5.5E-4,UltBa650:0.00369)0.434.8:5.5E-4)0.000.822:5.5E-
4,AerHydro:5.5E-4)0.855.95:5.5E-4)0.000.823:5.5E-
4,(UltGam55:0.00193,AerSpec4:5.5E-4)1.000.753:0.00174)0.000.824:5.5E-
4,UltBa618:0.00369)0.000.825:5.5E-
4,(UltBa575:0.00369,(UltBa574:0.0196,UltBa614:0.01512)0.897.103:5.4E-
4)0.827.69:0.00384)0.500.14:5.5E-
4,(UltBa563:0.00208,UltBa658:0.02231)0.966.103:0.00175)0.000.826:5.5E-
4)0.121.8:5.5E-
4,((UltGam54:0.01654,(UltBa416:0.00643,UltBa580:0.03384)0.874.128:0.00868
)0.737.61:0.0012,(UltBa450:0.0,UltBa565:0.0):0.00241)0.908.120:0.00254)0.
457.18:5.5E-4)0.000.827:5.3E-4,UltBa547:0.00369)0.000.828:5.5E-
4,BacNLA42:0.00387)0.610.9:5.5E-4,(UltBa637:5.4E-
4,(UltBa420:0.00736,(UltBa641:0.00741,(UltBa560:5.5E-
4,((UltBa552:0.03039,UltBa631:5.4E-
4)0.430.13:0.00369,(UltBa654:0.00742,UltBa556:5.4E-
4)0.761.70:0.00361)0.794.69:0.00375)0.790.78:0.00367)0.918.110:5.4E-
4)0.189.9:0.00367)0.766.77:0.00363)0.000.829:5.5E-4)0.000.830:5.5E-
4,UltBa647:0.00371)0.332.8:5.5E-4)0.100.7:5.5E-4,UltBa419:5.5E-
4)0.150.8:5.5E-
4,(UltBa579:0.00368,(UltBa607:0.00368,(((UltBa684:0.00372,(UltBa685:0.003
87,(UltGam56:0.00372,(UltBa682:5.5E-4,(UltBa704:0.00374,(TolAuens:5.5E-
4,UltGam57:0.00404)0.794.70:0.00406)0.787.75:0.00409)0.783.95:0.0041)0.80
8.71:0.00408)0.791.70:0.00408)0.980.61:5.4E-
4,UltBa705:0.02235)0.904.108:0.01608,(AerSimi2:0.0,AerSimia:0.0):0.00744)
0.575.7:5.5E-4)0.817.54:5.4E-4)0.854.96:0.00365)0.356.13:5.5E-
4,(ElbRiver:0.0037,(UltBa542:0.01106,UltBa571:5.5E-4)0.184.6:5.3E-
4)0.850.109:0.00364)0.095.5:5.4E-4)0.113.11:5.5E-4)0.389.9:5.5E-
4)0.888.117:0.01551)0.850.110:0.01295)0.870.88:0.01177)0.871.105:0.01409,
(UltBa681:0.0,UltBa683:0.0):0.00745)0.758.78:0.00757)0.802.64:0.01033,(Ul
tBa784:0.09654,((((VirFort2:0.0,UltVibri:0.0,VirSpe27:0.0,VirSpe28:0.0):5
.5E-4,(((((VirCrass:5.5E-
4,((VirSpeci:0.0,ExiAuran:0.0,VirSpec4:0.0,VirCycli:0.0,VirSpec7:0.0,VirS
ple3:0.0,UltMarin:0.0):5.5E-
4,((UltBa442:0.00374,VbnBacte:0.01523)0.000.831:5.5E-4,(VirKanal:5.5E-
4,(UltBa443:0.01502,(((VirSpe34:0.0,VirSpe35:0.0,VirSpe36:0.0,VirSplen:0.
0,VirSple2:0.0,GmmPro11:0.0):5.5E-
4,((AlvSalmo:0.00758,(AlvSpeci:0.00369,((AlvLoge2:0.0,AlvLogei:0.0,UltBa4
61:0.0,AlvLoge3:0.0,AlvSpec2:0.0,AlvSpec3:0.0,UltBa463:0.0,AlvSalm2:0.0,V
irSpec8:0.0,AlvFisch:0.0,VirFisc2:0.0):5.5E-
4,((VirFisch:0.00369,(UltBa460:0.0074,UltBa462:0.05321)0.761.71:0.00383)0
.133.9:5.5E-4,VirSpe39:0.00369)0.515.8:5.5E-4)0.407.13:5.5E-
4)0.439.13:5.4E-4)0.848.87:0.00382,VirSpec5:5.5E-4)0.377.11:5.5E-
4)0.238.11:5.5E-4,VirSpec6:5.5E-4)0.542.11:5.3E-
4)0.886.126:0.00804)0.920.121:5.3E-4)0.298.9:5.5E-4)0.000.832:5.5E-
4)0.000.833:5.5E-4,VirSpec2:0.00369)0.279.6:5.5E-
4,VirSple4:0.00367)0.584.13:5.5E-4,((((MucBacte:5.5E-4,VirAgari:5.5E-
4)0.863.115:0.00151,((VirSpe37:0.0,VirPecte:0.0):5.4E-4,(VirHispa:5.5E-
4,VirHarv5:5.5E-
4)0.838.91:0.00366)0.874.129:0.01029)0.897.104:0.01397,((((LisAngui:0.0,U
ltBa452:0.0,UltBa454:0.0,UltBa455:0.0,UltBa447:0.0,UltBa448:0.0,LisAngu2:

0.0,UltBa596:0.0,UltBa597:0.0):5.5E-
4,(((UltBa453:0.01501,(UltBa592:0.02634,UltBa595:0.11673)0.642.12:0.01539
)0.993.75:5.4E-4,UltBa456:0.00369)0.000.834:5.5E-
4,UltBa598:0.01115)0.163.9:5.5E-4)0.416.25:5.5E-
4,(UltBa591:0.0,UltBa593:0.0,UltBa594:0.0):0.0037)0.497.4:5.4E-
4,UltBa445:0.01879)0.962.98:0.01619)0.766.78:0.00541,VirLentu:5.5E-
4)0.761.72:0.00391)0.967.72:0.01778,((BacteK20:0.01098,((UltVibr3:0.01126
,(UltProt7:0.0,LobstGut:0.0,VirRumoi:0.0):5.5E-4)0.540.12:5.4E-
4,(VirSpe41:0.00367,(UltBa465:5.5E-4,(UltBa464:0.00369,VirSpe40:5.5E-
4)0.945.82:0.0112)0.760.79:0.00742)1.000.754:5.4E-4)0.987.100:5.5E-
4)0.914.123:0.01068,(AlvWodan:5.5E-
4,(UltBa446:0.04021,VirPorte:0.01315)0.701.22:0.01851)0.923.141:0.01096)0
.829.82:5.5E-4)0.218.6:5.4E-4)0.924.105:5.4E-
4,((((VirPara6:0.00727,(((UltBa466:0.0,VirForti:0.0):5.5E-
4,VirSpe56:0.01896)0.959.95:0.01131,(VirVulni:5.5E-
4,(((VirParah:0.01323,((PhcSpec4:0.0,PhcSpec5:0.0):0.01127,PhcSpec6:0.003
7)0.915.106:0.0111)0.772.87:5.5E-4,UltGam36:5.5E-4)0.526.12:5.4E-
4,(VirSpe38:0.0,VirSpe54:0.0,VirSpec9:0.0,VirSpe10:0.0,VirRotif:0.0,VirSp
e11:0.0,VirHarve:0.0,VirMytil:0.0,VirDiabo:0.0,VirPara2:0.0,VirHarv4:0.0,
VbnBact4:0.0,VirNatri:0.0,VirSpe19:0.0,VirDiab2:0.0,VirNatr2:0.0,VirSpe20
:0.0,VirSpe32:0.0,VirCoral:0.0,VirCora3:0.0):5.5E-4)0.183.11:5.5E-
4)0.000.835:5.5E-4)0.577.8:5.5E-4)0.124.11:5.4E-4,(VirHarv2:5.5E-
4,(VirAlgi2:5.5E-4,(VirAlgin:5.5E-4,VbnBact2:5.5E-4)0.352.9:5.5E-
4)0.698.25:5.5E-4)0.923.142:0.00361)0.886.127:5.4E-
4,(((UltBa473:0.17348,VirSpe18:0.08546)0.851.101:5.4E-
4,(((UltBa474:0.01115,(UltBa471:5.5E-
4,UltBa470:0.0074)0.852.96:0.00369)0.907.105:5.5E-
4,(VirSpe52:0.0,VirDiazo:0.0):5.5E-4)0.723.28:5.5E-
4,(UltBa472:0.00714,UltBa475:0.03825)0.910.104:5.3E-4)1.000.755:5.5E-
4)0.727.32:0.00345,(UltBa444:5.5E-4,VirSpe12:5.5E-4)0.979.79:5.5E-
4)0.854.97:0.00349)0.845.99:0.00339,((VirSpe33:0.00208,VirSpe30:0.0424)0.
998.122:0.00167,((((((VirSpe22:5.5E-
4,VirSpe29:0.0228)0.836.77:0.00369,((((VbnBact5:0.04277,(VirShilo:0.00381
,((((VirHangz:0.0,VirSpe50:0.0,VirSpe31:0.0):5.5E-4,(VirSpe55:5.5E-
4,(VirPonti:0.0,VirSpe13:0.0,VirSpe14:0.0):5.5E-
4)0.931.111:0.0037)0.293.9:5.4E-
4,VbnBact3:0.01922)0.918.111:0.00713,(((((VirSpec3:5.5E-
4,VirTapet:0.00369)0.291.13:0.00368,PhbStein:0.03568)0.971.64:5.4E-
4,(((VirOrien:0.00746,VirOrie2:5.4E-
4)0.887.112:0.00756,(UltVibr4:0.00375,UltBa476:0.26501)0.654.10:0.0038)0.
808.72:0.00366,(VirSpe26:5.4E-4,((VirSpe47:0.0,VirSpe23:0.0):5.4E-
4,(VirSpe44:0.00371,(VirPaci2:0.0,VirPacin:0.0):5.5E-
4)0.867.108:0.0037)0.856.91:0.00362)0.240.10:0.0037)1.000.756:5.4E-
4)0.736.46:5.5E-4,(VirAest2:5.5E-4,(VirMedi2:0.0,VirMedit:0.0):5.5E-
4)0.000.836:5.5E-4)0.773.92:5.3E-
4,VirAestu:0.00371)0.919.137:0.00206)0.105.12:0.00215)0.301.10:0.00371)0.
990.84:5.4E-
4,(VirVuln4:0.0,VirVuln5:0.0,VirVuln6:0.0,VirVuln7:0.0,VirVuln8:0.0):5.5E
-4)1.000.757:5.3E-4,((VirSpe43:5.4E-4,(VirNeon2:0.00369,(VirRarus:5.5E-
4,(VirPenae:0.0,VirGall2:0.0,VirGalli:0.0,VirSuper:0.0,VirInusi:0.0,VirIn
us2:0.0,VirBreog:0.0,VirHalio:0.0,VirHali2:0.0,VirHali3:0.0,VirNeona:0.0)
:5.5E-4)0.695.17:5.5E-4)0.922.147:0.00743)1.000.758:5.4E-
4,((((UltBa478:0.00369,VirNatr3:0.00369)0.902.106:5.5E-
4,(VirCinci:0.0,VirMetsc:0.0):5.5E-4)0.949.100:5.5E-4,(VirMangr:5.5E-

4,(VirRuber:0.00741,(VirSpe42:0.0,VirRhizo:0.0):5.5E-
4)0.834.68:0.00369)0.935.100:0.00746)0.851.102:0.00369,(VirFluvi:0.0,AllE
nter:0.0,VirFluv2:0.0):5.5E-
4)0.882.114:0.00361)0.133.10:0.00369)0.855.96:0.00366,((((VirNavar:0.0,Vi
rVuln2:0.0,VirPomme:0.0,VirVuln3:0.0):0.00367,((UltBa457:0.00735,(VirChol
4:0.0114,((VirChol2:0.0,VirChole:0.0,VirChol3:0.0,UltBa469:0.0):5.5E-
4,(UltBa467:5.5E-
4,UltBa468:0.00742)0.823.68:0.00369)0.886.128:0.00751)0.925.114:0.01119)0
.927.105:5.5E-
4,((VirSpe45:0.0,VirSpe46:0.0):0.01891,UltBa477:0.08434)0.742.46:0.00388)
0.317.12:0.00374)0.785.87:0.00382,VirSpe15:0.02741)0.884.113:5.5E-
4,VirSpe51:0.01515)0.822.59:0.00727)0.712.20:5.5E-4)0.421.12:5.5E-
4,VirCora2:0.00369)0.468.19:5.5E-4,VirIchth:0.00368)0.451.20:5.5E-
4,(VirNeptu:0.0,VirSpe48:0.0,VirSpe49:0.0,VirIcht2:0.0,VirSpe53:0.0,UltVi
br5:0.0,VirSpe17:0.0,VirSpe16:0.0,VirHarv3:0.0,VirRoti2:0.0,VirCampb:0.0,
VirPara3:0.0,VirAlfac:0.0,VirSpe21:0.0,UltBa458:0.0,VirSpe24:0.0,ViriXuii
:0.0,VirSpe25:0.0,UltVibr2:0.0,VirPara5:0.0,UltBa459:0.0,VirHepat:0.0):5.
5E-4)0.420.16:5.4E-4,VirPara4:0.00368)0.494.9:5.3E-4)0.984.71:5.4E-
4)0.866.105:0.01017)0.662.12:0.0065,(PhcSpe12:0.15985,(VirAerog:0.04245,P
sdSp105:0.32341)0.993.76:0.1099)0.904.109:0.03134)0.858.99:0.01184)0.902.
107:0.02301)0.773.93:0.00788)0.910.105:0.02443)0.709.21:0.01298)0.064.8:0
.00333)0.907.106:0.0157,((UltPse37:0.36052,UltrS298:0.30234)0.870.89:0.05
377,((((UltBa746:0.02446,(UltB3687:0.09232,(((UltBa749:5.2E-
4,UltBa747:0.02272)0.778.87:0.0037,UltBa750:0.017)0.785.88:0.00433,UltBa7
48:0.01104)0.539.6:0.01146)0.852.97:0.01374)0.850.111:0.01333,(UltBa755:0
.00814,(EnmbnOf4:0.04774,(UltBa743:0.0148,UltBa744:5.5E-
4)0.901.111:0.01262)0.752.70:0.00546)0.349.9:0.0128)0.923.143:0.02353,Ult
Ba766:0.03385)0.865.104:0.01508,UltBa211:0.09003)0.763.94:0.00909)0.737.6
2:0.00471)0.811.65:0.01334)0.897.105:0.0216)0.912.135:0.03531)0.743.62:0.
00689,(UltB4325:0.08749,((UltDel38:0.05402,(BacEnr19:0.0351,UltrSo56:0.06
537)0.887.113:0.039)0.973.71:0.05926,(Ult27537:0.0724,(Ult27486:0.05867,U
lt27548:0.11793)0.616.12:0.01809)0.959.96:0.05982)0.767.77:0.01911)0.927.
106:0.04587)0.895.116:0.01754)0.765.75:0.00696)0.854.98:0.01037,(Ult17357
:0.047,(UltOr277:5.5E-4,(UltOr278:5.5E-
4,UltOr279:0.00371)0.999.144:0.05691)0.995.95:0.05982)0.977.68:0.04634)0.
866.106:0.00886,(((((((Ult12857:0.02502,(Ult12854:0.01888,(Ult12855:0.00
787,Ult12856:0.01118)0.939.93:0.01782)0.711.28:0.00588)0.998.123:0.08886,
((Ult12781:5.5E-
4,(Ult12780:0.01078,Ult12782:0.02432)0.915.107:0.01573)0.799.87:5.4E-
4,(Ult12783:0.01107,Ult12784:0.05211)0.971.65:0.04203)0.992.84:0.07715)0.
894.105:0.02836,(((UltActi5:0.03488,(Ult12721:0.10024,(Ult12723:0.08866,U
lt12722:0.01049)0.828.59:0.02289)0.583.10:0.01104)0.998.124:0.09562,((Ult
12733:0.03948,((Ult12732:0.02214,Ult12731:0.02483)0.734.52:0.01124,(Ult12
734:0.00442,((Ult12736:0.02004,Ult12737:0.01109)0.916.117:0.02597,Ult1273
5:0.04209)0.174.9:0.02066)0.903.97:0.02339)0.743.63:0.011)0.908.121:0.021
55,((Ult12730:0.00764,UltCan55:0.00739)0.975.74:0.03279,((Ult12728:0.0178
9,Ult12729:0.02499)0.972.82:0.03137,(UltCan54:0.01715,(Ult12727:0.01228,(
Ult12724:0.01548,(Ult12726:0.01824,Ult12725:0.032)0.757.64:0.00577)0.939.
94:0.01864)0.885.98:0.01297)0.702.17:0.00928)0.842.98:0.01256)0.904.110:0
.01923)0.943.97:0.03774)0.841.86:0.02182,((Ult12629:0.11789,(Ult12415:0.1
0491,(((((UltCan74:0.04909,UltOr200:5.4E-
4)0.891.92:0.01041,UltOr201:0.00465)0.292.5:0.00673,((Ult12861:0.01507,Ul
t12862:0.00787)0.944.81:0.01791,(Ult12863:0.00964,Ult12864:0.02119)0.573.
8:0.01121)0.861.109:0.01116)0.979.80:0.02896,Ult12866:0.00574)0.264.6:0.0

172,Ult12865:0.0209)0.991.84:0.10701)0.301.11:0.03381)0.950.91:0.04577,((
(Ult12769:0.03931,UltrS152:0.03669)0.885.99:0.02118,((Ult12767:0.00971,Ul
t12768:0.02922)0.900.90:0.02032,((Ult12764:0.02157,UltCan58:0.00978)0.995
.96:0.05598,(Ult12765:0.02527,((Ult12763:0.00697,Ult12766:0.0197)0.724.27
:0.00379,((Ult12751:0.0146,(Ult12760:0.01071,(Ult12759:0.01899,Ult12761:0
.02479)0.739.45:0.00372)0.972.83:0.01971)0.844.107:5.4E-
4,(Ult12762:0.0115,(((StmClon3:0.00329,Ult12753:0.02363)0.871.106:0.00808
,(Ult12754:0.01595,Ult12758:0.02758)0.949.101:0.0194)0.779.82:0.00373,(((
Ult12749:0.01169,(Ult12748:5.5E-4,(Ult12747:0.01496,UltCan57:5.5E-
4)0.864.103:0.00369)0.784.91:0.00342)0.907.107:0.01231,(Ult12752:5.5E-
4,Ult12756:0.00369)0.888.118:0.01093)0.207.5:0.00769,(Ult12755:5.5E-
4,(Ult12750:0.01148,Ult12757:0.00354)0.893.118:0.00767)0.916.118:0.01082)
0.992.85:5.5E-
4)0.881.115:0.00763)0.886.129:0.00781)0.879.113:0.0083)0.881.116:0.01372)
0.743.64:0.00769)0.326.6:0.01659)0.885.100:0.02436)0.978.63:0.03764,((Ult
Pl552:0.01918,(Ult12770:0.02307,(Ult12771:0.01548,Ult12772:0.02795)0.962.
99:0.02896)0.790.79:0.01824)1.000.759:0.11416,(Ult12774:0.02928,Ult12773:
0.02755)0.960.94:0.03231)0.249.18:0.02554)0.736.47:0.0056)0.944.82:0.0242
4)0.709.22:0.01161)0.925.115:0.02458,(Ult16430:0.11112,(Ult12842:0.02291,
Ult12843:0.03699)0.980.62:0.06418)0.944.83:0.04131)0.817.55:0.01031,(((((
(((UdnBac68:0.00369,Ult16742:5.5E-
4)1.000.760:0.03081,Ult16735:0.00375)0.770.94:0.00372,((Ult16748:0.03555,
((Ult16743:5.4E-4,(Ult16730:5.5E-
4,((Ult16734:0.00373,UltNit88:0.00372)0.819.69:0.00369,Ult16728:0.00748)0
.784.92:5.5E-4)0.833.87:0.00369)0.782.104:0.00367,((Ult16738:5.4E-
4,(UltNit89:0.01149,Ult16737:0.01533)0.863.116:0.00727)0.774.90:0.00363,(
Ult16736:5.5E-4,UltNit91:5.5E-
4)0.973.72:0.01902)0.771.103:0.00383)0.414.11:0.00344)0.883.115:0.0078,((
Ult16731:0.00344,Ult16732:0.00399)0.457.19:0.00748,(Ult16729:0.00743,Ult1
6733:0.02311)0.904.111:5.4E-
4)0.865.105:0.00714)0.761.73:0.00362)0.750.54:0.00398,((Ult16751:0.01252,
(Ult16749:0.03878,Ult16750:0.01254)0.926.123:0.0242)0.955.77:0.02797,(Ult
16744:0.04932,(Ult16745:0.01124,(Ult16746:0.0,Ult16747:0.0):5.4E-
4)0.988.81:0.04506)0.394.13:0.00692)0.896.98:0.01399)0.702.18:0.01696,((U
lt16741:0.04373,(Ult16757:0.06085,Ult16758:0.03511)0.988.82:0.06602)0.559
.11:0.02502,(Ult16740:0.03178,(UltNit90:0.03019,Ult16739:0.04682)0.218.7:
0.00885)0.458.27:0.00586)0.862.102:0.01657)0.750.55:0.00901,((Ult16752:0.
0,Ult16753:0.0,Ult16754:0.0):0.06615,((UltThe10:0.0212,UltTher9:0.00567)0
.879.114:0.01423,((UltNit72:0.0175,(((Ult16694:0.0,UltNit87:0.0):5.4E-
4,Ult16695:0.01121)1.000.761:0.05277,((Ult16691:0.0,Ult16692:0.0,UltNit78
:0.0):5.5E-
4,(Ult16690:0.01125,Ult16693:0.0273)0.838.92:0.0076)0.824.64:0.00918)0.93
9.95:0.02186)0.859.95:0.01096,((UltNit53:0.00372,UltNit52:0.00749)0.920.1
22:0.01118,(UltNit51:0.00378,UltNit50:0.03102)0.845.100:5.4E-
4)0.781.82:0.00624)0.064.9:0.01273)0.955.78:0.04038)0.810.66:0.02782)0.98
0.63:0.05837,(((((((((Ult16711:0.0,Ult16713:0.0,Ult16726:0.0):0.0037,Ult
16712:5.5E-4)0.824.65:0.00368,Ult16721:0.03854)1.000.762:5.5E-
4,(Ult16714:0.0,Ult16715:0.0):5.5E-4)0.946.92:5.5E-
4,Ult16716:0.00738)0.758.79:0.01041,TrfThiop:0.04498)0.255.3:0.01173,(Ult
Nit85:5.4E-
4,(((NrpSpec6:0.0,NrpSpec7:0.0):0.03103,Ult16709:0.00743)0.229.10:5.4E-
4,(((TrfIslan:0.00776,(Ult16708:5.5E-
4,Ult16710:0.00372)0.754.69:0.00353)0.982.62:0.01924,UltThe13:0.00369)0.4
73.12:5.5E-

4,TrfAggre:0.01509)0.935.101:0.01121)0.803.63:0.00742)0.870.90:0.01211)0.780.101:0.00434,(Ult16717:5.5E-4,Ult16718:0.01963)0.814.55:0.00631)0.780.102:0.00837,TrfHydro:0.02459)0.988.83:0.06283,(Ult16759:0.14918,((((UltNit83:0.0,UltNit84:0.0):0.02389,(CanMagne:5.5E-4,UltNit82:0.0074)0.947.79:0.03532)0.666.19:0.03043,(Ult16723:0.07481,(Ult16725:0.03863,(Ult16722:0.03195,Ult16724:0.03292)0.841.87:0.01716)0.809.76:0.01986)0.961.83:0.04818)0.958.98:0.05486,(((((((UltNit80:0.00369,(Ult16702:5.5E-4,(Ult16701:0.00369,Ult16703:0.00374)0.720.28:5.5E-4)0.945.83:5.0E-4)1.000.763:5.4E-4,(Ult16704:0.00378,(UltNit79:0.0164,((Ult16696:5.4E-4,(Ult16700:0.00354,Ult16699:0.04875)0.881.117:0.01541)0.769.85:0.00372,(Ult16698:0.00745,Ult16697:5.5E-4)0.786.82:0.00366)0.951.98:0.02113)0.917.98:0.01652)0.762.91:0.00359)0.999.145:0.03953,(Ult16705:0.00369,UltNit81:5.5E-4)0.987.101:5.3E-4)0.300.12:0.00956,Ult16706:0.01824)0.855.97:0.02047,(UltNit70:0.01589,UltNit71:0.01538)0.976.61:0.04505)0.892.106:0.025,((((Ult16719:0.02999,((Ult16667:0.0,Ult16668:0.0,Ult16669:0.0):0.00368,Ult16670:5.5E-4)0.956.91:0.03144)0.939.96:0.03427,((((((((Ult16687:0.01546,(Ult16688:0.01134,Ult16727:5.4E-4)0.956.92:0.01923)0.683.26:0.00334,Ult16681:0.03617)0.948.111:0.01593,UltNit76:0.01125)0.958.99:0.0195,(Ult16683:0.00371,(Ult16682:0.00746,Ult16684:5.4E-4)0.764.93:0.00371)0.932.99:0.01164)0.776.89:0.00376,Ult16685:0.0315)0.775.82:0.00658,(Ult16679:0.06693,Ult16686:0.02709)0.831.68:0.01331)0.889.101:0.01341,Ult16680:0.03952)0.957.84:0.02055,((((Ult16677:0.0043,Ult16676:0.03964)0.674.20:0.01045,(Ult16678:0.03999,UltNit77:0.01633)0.641.11:0.01531)0.664.14:0.01073,(Ult16674:0.04768,UltNit75:5.5E-4)0.952.86:0.02197)0.922.148:0.01969,Ult16675:0.02376)0.594.12:0.00342)0.760.80:0.00431,(Ult16673:0.05274,(Ult16671:5.3E-4,Ult16672:0.12123)0.945.84:0.01566)0.517.6:0.01144)0.863.117:0.01656)0.149.13:0.00912,(Ult16689:0.05023,(Ult16666:0.03459,((Ult16662:0.0,Ult16664:0.0,Ult16665:0.0):5.5E-4,Ult16663:0.00369)0.832.78:0.00373)0.969.64:5.5E-4)0.871.107:0.01884)0.959.97:0.02974,(Ult16661:0.02973,((((Ult16646:0.01911,((((Ult16655:0.00133,(Ult16653:0.01181,(UltNit62:0.02283,((UltNit68:0.03108,(Ult16659:0.00746,(Ult16658:0.00376,UltNit69:0.02641)0.776.90:0.00357)0.883.116:0.00711)0.923.144:5.5E-4,((Ult16657:0.01124,(UltNit63:0.00369,(UltNit60:0.01927,Ult16651:0.01154)0.749.79:0.00348)0.861.110:5.4E-4)0.796.64:0.00743,(Ult16650:0.00375,Ult16656:0.01525)0.769.86:0.00366)0.962.100:5.4E-4)0.784.93:0.00547)0.788.79:0.00551)0.688.20:0.0145)0.417.17:0.01042,((((UltMagn6:0.0152,Ult16652:0.00742)0.938.64:0.01129,StmClon6:5.5E-4)0.961.84:0.01506,((UltrS296:8.1E-4,UltrS297:0.01705)0.893.119:0.00331,(UltrS294:0.02571,UltrS295:0.09515)0.424.19:0.01369)1.000.764:5.4E-4)0.832.79:0.00424,Ult16654:5.4E-4)0.865.106:0.00912)0.879.115:0.01151,(((UltNit57:0.01491,UltNit58:5.5E-4)0.935.102:0.01541,(UltThe11:0.01959,UltThe12:0.00355)0.905.111:0.01159)0.904.112:0.01164,(UltNit56:0.01574,UltNit61:0.01553)0.725.32:0.00325)0.740.54:0.00392)0.800.72:0.00848,(Ult16647:0.02359,(Ult16649:0.00396,(Ult16648:0.03954,(Ult16644:0.00378,((Ult16642:5.4E-4,(Ult16643:0.01122,UltNit49:0.01122)0.751.49:0.00371)0.787.76:0.00371,UltAc875:0.02322)0.775.83:0.00365)0.927.107:0.01439)0.749.80:0.00493)0.929.

95:0.01556)0.814.56:0.00734)0.903.98:0.01652)0.742.47:0.00513,((UltNit67:
0.00743,(UltNit66:0.03321,(Ult16645:0.0074,UltNit54:5.5E-
4)0.932.100:0.01866)0.427.14:0.007)0.794.71:0.00774,(UltNit64:0.00323,Ult
Nit65:0.03179)0.947.80:0.01606)0.459.13:0.00273)0.902.108:0.019,(UltTher8
:0.01678,(UltNit55:0.0195,UltNit59:0.02877)0.943.98:0.02231)0.936.103:5.4
E-
4)0.876.89:0.02571,(UdnNitro:0.06762,UltNit86:0.05434)0.704.20:0.00756)0.
878.100:0.01633)0.687.18:0.00505)0.937.99:0.02503)0.785.89:0.01414,((UltN
it73:0.01483,Ult16760:0.07094)0.690.20:0.01149,(UltNit74:0.05697,Ult16660
:0.0309)0.861.111:0.01438)0.802.65:0.00886)0.350.11:0.00903)0.252.9:0.010
59)0.871.108:0.03665)0.745.69:0.01556)0.991.85:0.07239,(((((Ult11166:0.01
582,Ult11167:0.05653)0.367.16:0.0092,(Ult11168:0.01956,(Ult11169:0.02336,
(Ult11171:0.06569,Ult11170:0.04031)0.453.11:0.01708)0.974.55:0.03305)0.89
6.99:0.01912)0.947.81:0.06367,(UltPl516:0.15034,Ult11172:0.10742)0.945.85
:0.0635)0.948.112:0.05637,((((Ult11175:0.01436,(Ult11173:0.02687,Ult11174
:0.05784)0.848.88:0.02908)0.981.77:0.04566,Ult11176:0.02534)0.056.8:0.007
88,Ult11177:0.00135)0.999.146:0.132,UltB4222:0.14455)0.895.117:0.04789)0.
943.99:0.04182,((((UltPl475:0.11423,Ult11215:0.12103)0.754.70:0.01673,(((
(Ult15694:0.08538,(((Ult11228:0.13722,(Ult11193:0.01943,Ult11194:0.05728)
0.973.73:0.08117)0.953.74:0.06814,UltPl540:0.04004)0.804.64:0.02424,(((Ul
tPl539:0.05563,Ult11242:0.06982)0.988.84:0.07927,(UltPl537:0.02446,(UltPl
538:0.03598,((Ult11240:0.03922,Ult11241:0.01292)0.397.17:0.01188,(Ult1123
9:0.02371,Ult11238:0.04083)0.298.10:0.00598)0.837.66:0.01553)0.670.14:0.0
2182)0.679.21:0.02366)0.831.69:0.01941,(((((UltPl467:0.03144,UltPl468:0.0
6096)0.985.78:0.05851,Ult11130:0.03441)0.958.100:0.04578,((Ult11119:0.034
6,((Ult11117:0.02971,Ult11118:0.00561)0.992.86:0.05807,(Ult11116:0.03828,
(UltPl464:0.0,UltPl465:0.0):8.9E-
4)0.881.118:0.0296)0.752.71:0.03237)0.783.96:0.01272,(Ult11120:0.04901,(U
lt11121:0.028,(UltPl466:0.01966,Ult11122:0.01933)0.751.50:0.0045)0.780.10
3:0.00923)0.883.117:0.01469)0.776.91:0.01075)0.861.112:0.0238,(Ult11131:0
.09495,((Ult11123:5.4E-
4,Ult11124:0.0345)0.948.113:0.02785,(Ult11125:0.01193,((Ult11128:0.03137,
(Ult11126:0.00703,Ult11129:0.00688)0.861.113:5.4E-
4)0.634.12:0.00852,Ult11127:0.00703)0.916.119:0.01517)0.928.83:0.02947)0.
933.115:0.0347)0.847.124:0.02238)0.885.101:0.02857,Ult11243:0.05206)0.734
.53:0.00967)0.454.18:0.02303)0.547.9:0.01114)0.718.27:0.01564,(((Ult11012
:0.06517,((UltPl429:0.06775,((Ult11068:0.05657,Ult11069:0.0339)0.974.56:0
.04284,(((Ult11070:0.09449,Ult11072:0.02573)0.566.11:0.02061,Ult11071:0.0
4589)0.837.67:0.01487,((Ult11073:5.5E-
4,Ult11075:0.07198)0.955.79:0.02118,Ult11074:0.00994)0.911.128:0.02636)0.
883.118:0.02021)0.956.93:0.02489)0.145.4:0.01196,((Ult11008:0.0,Ult11009:
0.0):0.0344,(Ult11010:0.00374,Ult11011:0.00369)0.822.60:0.01534)0.999.147
:0.07226)0.669.16:0.01015)0.382.12:0.01377,(((Ult11156:0.03341,UltPl501:0
.05994)0.290.10:0.0238,(Ult11157:0.06391,((Ult11137:0.00411,(((((((UltPl4
85:0.00369,UltPl492:0.00738)0.070.8:5.4E-
4,((((((UltPl482:0.00367,(UltPl480:0.0074,UltPl479:0.00368)0.785.90:0.003
68)0.866.107:5.5E-4,(UltPl481:0.0,UltPl483:0.0):5.5E-4)0.914.124:5.5E-
4,(Ult11134:0.00742,(UltPl476:5.5E-4,(UltPl477:0.00368,UltPl478:5.5E-
4)0.844.108:0.00367)0.722.26:5.5E-
4)0.831.70:0.00367)0.986.76:0.01897,((UltPl487:5.4E-
4,(UltPl490:0.01495,UltPl488:5.4E-
4)0.673.10:0.00367)0.823.69:0.00367,(UltPl491:0.0,UltPl484:0.0):5.4E-
4)0.980.64:0.00197)0.981.78:0.00189,(UltAnae9:0.0,UltPl486:0.0):0.00368)0
.053.4:5.3E-4,UltPl489:5.5E-

4)0.829.83:0.00368)0.770.95:0.00373,CanScali:0.01525)0.426.20:0.00763,Ult
11135:0.00354)1.000.765:5.4E-
4,UltCan30:0.00369)0.955.80:0.01502,(Ult11136:0.00741,(UltPl493:5.5E-
4,(UltPl494:0.0074,UdnBac41:0.00368)0.753.80:5.5E-4)0.940.101:5.4E-
4)0.640.13:0.00378)0.465.13:0.00379,((CanScal3:0.01861,UltCan31:5.5E-
4)0.530.7:0.00392,(AnbAmmon:5.4E-
4,UltPl495:0.01185)0.833.88:0.007)0.996.95:0.03255)0.607.11:0.00341)0.995
.97:0.06719,((AnbAmmo2:0.02669,(((UltPl497:0.02197,(CanBroca:0.00363,(Ult
11150:0.03474,(Ult11151:0.00367,UltPl496:0.00368)0.882.115:5.3E-
4)0.932.101:0.01128)0.901.112:0.0143)0.887.114:0.01415,((((UltPl500:5.5E-
4,(UltPl498:0.00736,Ult11152:0.02255)0.557.11:5.5E-4)0.828.60:5.4E-
4,UltPl499:0.00741)0.961.85:0.01119,Ult11153:0.00368)0.062.8:5.4E-
4,CanBroc2:0.0074)0.896.100:0.01252)0.990.85:0.03512,(CanKuene:0.00738,Ul
t11154:0.00368)0.902.109:5.5E-
4)0.556.9:0.00436)0.445.16:0.00821,(((Ult11146:0.0,Ult11148:0.0):5.5E-
4,Ult11147:5.5E-4)0.758.80:0.00351,((Ult11149:0.02539,(CanAnamm:5.5E-
4,((Ult11143:0.0,Ult11144:0.0,Ult11145:0.0):0.03583,Ult11142:0.01382)0.62
0.11:0.00909)0.898.121:0.02262)0.549.14:0.01948,(Ult11141:5.4E-
4,((Ult11138:0.0,Ult11139:0.0,Ult11140:0.0):0.00563,CanJette:0.01039)0.88
6.130:0.01029)0.882.116:0.01612)0.772.88:0.00722)0.884.114:0.01486)0.798.
71:0.01204)0.959.98:0.0443)0.925.116:0.03761)0.911.129:0.03012,Ult11155:0
.02028)0.939.97:0.03937)0.881.119:0.01452,(Ult11114:0.11809,(((((Ult11049
:0.01132,Ult11050:0.05369)0.655.21:0.04213,(Ult11051:0.11998,UltPl436:0.0
3461)0.896.101:0.04939)0.933.116:0.04578,((((Ult11046:0.01581,(Ult11047:0
.0193,Ult11048:0.02015)0.733.39:0.00314)0.801.73:0.00626,(((UltPl435:0.00
69,UltPl434:0.08508)0.294.8:0.00741,(Ult11041:0.03384,(Ult11039:0.01285,U
lt11040:0.01479)0.976.62:0.0435)0.807.58:0.02476)0.941.88:0.0274,Ult11042
:0.04376)0.923.145:0.02262)0.119.7:0.00431,(Ult11045:0.03041,(Ult11038:0.
00717,(Ult11029:0.00457,((Ult11028:0.00599,((Ult11027:0.00382,((UltPl431:
0.0,UltPl432:0.0):0.01938,Ult11025:5.4E-
4)0.999.148:0.02734)0.774.91:0.00357,Ult11026:5.3E-
4)0.995.98:0.052)0.956.94:0.02643,(((((UltB9870:0.03441,Ult11020:0.00523)
0.978.64:0.02711,Ult11024:0.01124)0.869.84:0.00851,((((((Ult11016:0.01953
,Ult11030:5.5E-4)0.947.82:0.01142,(Ult11014:0.03479,(Ult11017:5.5E-
4,Ult11013:0.02754)0.458.28:5.4E-
4)0.606.14:0.00723)0.744.59:0.00348,(Ult11015:5.4E-
4,Ult11018:0.01106)0.774.92:0.00366)0.880.118:0.00714,(Ult11019:0.00371,(
Ult11021:0.03153,(UltPl430:0.00797,UltPl433:0.02346)0.895.118:0.01163)0.7
46.57:0.00406)0.770.96:0.0038)0.941.89:0.01111,Ult11022:0.00775)0.558.10:
5.3E-
4,Ult11023:0.01089)0.551.13:0.00727)0.753.81:0.00272,((Ult11035:0.03157,(
Ult11036:0.0112,Ult11037:0.00371)0.975.75:0.02721)0.870.91:0.01144,(UltCr
at4:0.02291,((Ult11031:0.0,Ult11033:0.0):5.4E-
4,Ult11032:0.01526)0.920.123:0.01526)0.420.17:5.4E-
4)0.972.84:0.01948)0.858.100:0.00727,Ult11034:0.01171)0.874.130:0.01291)0
.898.122:0.01751)0.877.120:0.02298)0.987.102:0.04799)0.850.112:0.0167)0.6
56.15:5.5E-
4,(Ult11043:0.03321,Ult11044:0.04434)0.916.120:0.02925)0.969.65:0.04704)0
.716.26:0.02049,((Ult11115:0.05891,UltPl463:0.0451)0.996.96:0.09167,UltOr
143:0.0467)0.838.93:0.02587)0.751.51:0.00529,((((UltPl449:0.0148,(UltPl45
2:0.04777,(Ult11080:0.03161,Ult11082:0.0513)0.121.9:0.01262)0.835.87:0.01
792)0.841.88:0.01339,((UltPl453:0.04786,(UltPl445:0.0668,UltPl446:0.00829
)0.998.125:0.08142)0.273.12:5.4E-
4,Ult11083:0.04687)0.948.114:0.0319)0.952.87:5.4E-

4,((((UltPl443:0.00379,((UdnPlan2:5.4E-
4,UdnMari2:0.01099)0.428.14:0.00714,(Ult11077:0.01469,Ult11076:5.3E-
4)0.963.76:0.01931)0.928.84:0.0153)0.910.106:5.4E-
4,(UltPl444:0.01588,Ult11078:0.07183)0.988.85:0.05688)0.342.12:0.01566,Ul
tPl442:0.01106)0.988.86:0.03549,(UltPl447:0.02976,(UltPl448:0.03082,(Ult1
1081:0.02168,(UltPl451:0.06322,UltPl450:0.00832)0.424.20:0.0088)0.962.101
:0.02227)0.889.102:0.02183)0.912.136:0.01844)0.859.96:0.01421)0.937.100:0
.0211,((Ult11109:0.0045,UltPl461:0.00292)1.000.766:0.11772,((UltPl456:0.0
3206,((((Ult11079:0.07001,UltPl460:0.0256)0.954.86:0.03408,(Ult11107:0.01
09,UltPl459:0.03607)0.992.87:0.04989)0.490.5:0.00955,Ult11102:0.03176)0.9
29.96:0.02374,((((UltPl457:0.03984,Ult11103:0.01915)0.855.98:0.01209,(Ult
Pl458:0.04005,(Ult11106:0.01756,(Ult11104:0.00585,Ult11105:0.02918)0.137.
7:0.00403)0.993.77:0.04778)0.783.97:0.00683)0.771.104:0.00947,Ult11101:0.
02919)0.771.105:0.01008,Ult11108:0.05841)0.184.7:0.01247)0.930.93:0.01762
)0.825.69:0.00754,(Ult11100:0.0546,((UdnBac38:0.03565,UltPl455:0.01181)0.
710.28:0.00789,(Ult11086:0.02443,(((Ult11093:0.00795,(Ult11089:0.01524,(U
lt11087:5.5E-4,Ult11088:0.00375)0.956.95:5.4E-
4)0.028.6:0.01129)0.939.98:0.0175,(Ult11097:0.04446,((Ult11098:0.01241,Ul
t11099:0.00681)0.924.106:0.02019,((Ult11090:0.00828,Ult11091:0.02301)0.86
3.118:0.0099,((Ult11094:0.0,Ult11095:0.0):0.03489,Ult11096:0.00886)0.772.
89:0.01008)0.878.101:0.01289)0.794.72:0.00896)0.907.108:0.01576)0.956.96:
0.02409,(UltPl454:0.03521,(Ult11085:0.03169,(Ult11084:5.5E-
4,Ult11092:5.5E-
4)0.939.99:0.02087)0.289.5:0.00861)0.913.120:0.01663)0.763.95:0.00704)0.8
94.106:0.01428)0.759.88:0.00471)0.871.109:0.01087)0.478.13:5.5E-
4)0.373.17:0.01002)0.992.88:0.04961)0.933.117:0.0258)0.783.98:0.00881)0.8
19.70:0.00994)0.961.86:0.03974,(((Ult11197:0.03466,((Ult11200:5.5E-
4,Ult11201:0.00369)0.947.83:0.02845,(Ult11199:0.05973,(Ult11198:0.01042,(
Ult11196:0.02601,Ult11195:0.04319)0.890.107:0.01601)0.822.61:0.01451)0.40
3.12:0.01168)0.935.103:0.04214)0.973.74:0.04485,((Ult11232:0.0648,UltPl53
4:0.05574)0.983.63:0.05908,(Ult11237:0.04793,(Ult11236:0.03064,UltPl536:0
.04085)0.967.73:0.04644)0.985.79:0.06192)0.693.19:0.00495)0.904.113:0.022
24,((((((UltPl528:0.0853,(Ult11211:0.02616,(Ult11210:0.05061,UltPl527:0.0
2198)0.964.91:0.0416)0.765.76:0.01193)0.958.101:0.04121,Ult11233:0.07903)
0.810.67:0.012,((((((((UltrS119:0.03886,(UltrS120:0.02356,UltPl525:5.5E-
4)0.218.8:0.00991)0.967.74:0.0288,(Ult11206:0.04331,(Ult11204:0.01141,Ult
11205:0.01928)0.882.117:0.01705)0.842.99:0.0131)0.955.81:0.03075,(Ult1120
9:0.0684,UltPl526:0.02191)0.271.9:0.01075)0.703.27:7.3E-
4,(Ult11207:0.01428,Ult11208:0.04525)0.948.115:0.02456)0.895.119:0.02185,
(UdnBac42:0.05968,(UltPl522:0.0266,(Ult11248:0.05281,UltPl523:0.01174)0.7
33.40:0.01059)0.937.101:0.02875)0.683.27:0.00766)0.866.108:0.01312,UltPl5
24:0.03281)0.860.77:0.00965,(Ult11203:0.01511,UltPl520:5.4E-
4)0.992.89:0.02719)0.730.37:5.4E-
4,UltPl519:0.04818)0.848.89:0.01168)0.897.106:0.01739,Ult11202:0.09581)0.
791.71:0.00919,((Ult11159:0.07429,UltPl506:0.0534)0.934.91:0.04317,(Ult11
216:0.04426,UltPl530:0.04313)0.994.94:0.08387)0.839.89:0.01945)0.481.14:0
.00204,((UltPl505:0.08328,(UltPl504:0.03162,(UltPl503:0.04985,(Ult11158:0
.00961,UltPl502:0.02899)0.564.7:0.01605)0.394.14:0.00442)0.991.86:0.09038
)0.795.79:0.03414,((Ult11231:0.09129,Ult11230:0.04676)0.913.121:0.03838,(
(Ult11190:0.05629,Ult11191:0.08924)0.948.116:0.04257,(UltPl529:0.08369,(U
lt11212:0.05117,Ult11213:0.02893)0.910.107:0.03723)0.982.63:0.06153)0.901
.113:0.02588)0.661.14:5.4E-
4)0.912.137:0.02604)0.925.117:0.0138)0.726.27:0.00568)0.751.52:0.00704,((
(Ult11110:0.02632,Ult11111:0.00473)0.976.63:0.04729,((Ult11112:0.02991,Ul

t11113:0.04443)0.863.119:0.01798,(UltPl462:0.02891,UltMar51:0.15014)0.937
.102:0.04215)0.865.107:0.02436)1.000.767:0.09386,(UltPl541:0.05153,(UltPl
542:0.00662,((Ult11246:0.04518,Ult11244:0.03919)0.070.9:0.01406,(Ult11245
:0.01137,Ult11247:0.01533)0.885.102:0.01291)0.786.83:0.00893)0.817.56:0.0
1121)0.960.95:0.03516)0.827.70:0.01228)0.429.13:0.00357)0.922.149:0.01621
,(((Ult11222:0.04065,(Ult11221:0.09068,(UltPl531:5.4E-4,(((Ult11224:5.5E-
4,((Ult11223:0.0,Ult11226:0.0):5.5E-
4,Ult11220:0.00744)0.845.101:0.00337)0.892.107:0.01415,(Ult11219:0.02289,
Ult11225:0.02548)0.557.12:0.00935)0.819.71:0.01376,(Ult11217:0.01154,Ult1
1218:0.02321)0.779.83:0.00576)0.926.124:0.02501)0.815.67:0.01047)0.863.12
0:0.02347)0.995.99:0.14715,(Ult11227:0.03777,(UltPl532:0.06081,UltPl533:0
.05686)0.932.102:0.05114)0.972.85:0.0976)0.956.97:0.07567,(((((Ult11059:0
.03942,PnmctA20:5.4E-
4)0.000.837:0.00647,(UltPl440:0.02393,Ult11060:0.05984)0.493.14:0.03037)0
.989.74:0.09047,((Ult11054:0.02579,((Ult11055:0.02303,Ult11056:5.5E-
4)0.972.86:0.03603,(Ult11053:0.0114,(Ult11052:0.00361,UltPl437:0.00382)0.
890.108:0.01598)0.791.72:0.02119)0.881.120:0.02553)0.579.15:0.0149,(UltPl
439:0.0512,(Ult11057:0.01664,((UltMar50:0.01535,UltPl438:0.00365)0.953.75
:0.01671,Ult11058:0.00647)0.343.8:0.00361)0.887.115:0.02283)0.907.109:0.0
3427)0.945.86:0.05593)0.990.86:0.08331,((Ult11067:0.06289,Ult11066:0.0683
5)0.981.79:0.06761,(UltPl441:0.07118,(Ult11064:0.09583,(Ult11063:0.0164,(
UltrS118:0.03462,Ult11062:0.02629)0.730.38:0.00559)0.967.75:0.04712)0.884
.115:0.02673)0.070.10:0.02779)0.902.110:0.03765)0.762.92:0.01186,(((UdnBa
c40:0.12473,(Ult11132:0.01784,UltPl469:0.03119)0.870.92:0.03616)0.852.98:
0.03194,(Ult11187:0.05925,(Ult11186:0.04433,Ult11188:0.0569)0.865.108:0.0
2165)0.997.98:0.07259)0.795.80:0.01385,((Ult11189:0.1824,(Ult11214:0.0832
8,Ult11192:0.06784)0.337.7:0.02283)0.864.104:0.02428,((UltrS121:0.1143,Ul
t10546:0.10292)0.803.64:0.01756,(Ult11235:0.0503,(Ult11234:0.01492,UltPl5
35:5.4E-
4)0.975.76:0.059)1.000.768:0.12936)0.573.9:0.02433)0.770.97:0.01235)0.843
.82:0.02463)0.760.81:0.00738)0.911.130:5.5E-
4)0.775.84:0.02053,(((Ult11061:0.09777,Ult29006:0.27113)0.939.100:0.06929
,(((Ult11007:0.05245,(Ult11006:0.055,(UltOr142:0.03613,(Ult11005:0.01889,
Ult11004:0.00387)0.850.113:0.02037)0.349.10:0.02986)0.879.116:0.027)0.993
.78:0.0864,((UltPl360:0.16341,(((((Ult10942:0.05607,(((((((UltPl401:0.096
25,((Ult10933:0.03292,(Ult10935:0.00364,(PnmGMD14:0.03189,Ult10934:0.0245
1)0.767.78:0.00891)0.774.93:0.00734)0.883.119:0.02904,(UltOr127:0.05722,(
UltPl400:0.03245,(Ult10936:0.03681,UltPl399:0.04459)0.820.58:0.01458)0.23
3.10:0.01153)0.930.94:0.03511)0.881.121:0.02592)0.882.118:0.02438,UdnBac3
7:0.01784)0.917.99:0.02907,UltPl402:0.07716)0.973.75:0.0446,(Ult10958:0.0
1516,Ult10959:5.4E-
4)0.991.87:0.06262)0.870.93:0.02416,Ult10941:0.04004)0.987.103:0.05413,((
((Ult10955:0.06183,(Ult10956:0.04835,(IroBac15:0.04813,Ult10954:0.03231)0
.957.85:0.03629)0.799.88:0.01763)0.922.150:0.0317,(UltPl409:0.03106,Ult10
957:5.4E-
4)0.965.76:0.047)0.945.87:0.04437,(Ult10939:0.03575,UltPl405:0.05201)0.87
6.90:0.02633)0.481.15:0.01926,Ult10940:0.05502)0.897.107:5.5E-
4)0.964.92:0.04136,(Ult10937:0.09328,((Ult10938:0.0348,Ult10943:0.11462)0
.936.104:0.03543,(UltPl403:0.08313,(PnmGMD15:0.0449,UltPl404:0.07678)0.54
0.13:0.02566)0.828.61:0.02275)0.187.9:0.00746)0.423.16:0.0407)0.738.41:0.
01312)0.528.10:0.01318,(((((Ult10907:0.00368,UltPl396:5.5E-
4)0.953.76:0.02616,(Ult10908:0.04355,Ult10909:0.00472)0.906.114:0.01625)0
.954.87:0.02809,(UltOr125:0.01935,UltOr124:0.01127)0.828.62:0.01102)0.738
.42:5.4E-

4,((Ult10905:0.06491,UltOr126:0.0206)0.723.29:0.00398,Ult10906:0.02265)0.
959.99:0.02384)0.984.72:0.0424,((((Ult10920:0.0108,UltMar49:0.00436)0.969
.66:0.0259,(Ult10976:0.10255,(((Ult10930:0.05681,(Ult10893:0.0,Ult10894:0
.0):0.06117)0.719.24:0.00594,(((Ult10891:5.5E-
4,Ult10892:0.00741)0.758.81:0.00867,Ult10901:0.13029)0.911.131:0.02684,(U
ltPl391:0.07044,((Ult10897:5.4E-
4,(Ult10890:0.00749,(UltPl389:0.04738,(Ult10889:0.00383,UltPl387:0.01518)
0.780.104:0.00743)0.033.3:5.5E-
4)0.930.95:0.00756)0.792.94:0.00588,((((UltPl392:0.03539,(Ult10898:0.0125
8,(UltPl390:0.00486,Ult10899:0.0141)0.430.14:0.00732)0.846.111:0.01097)0.
937.103:0.01989,(Ult10900:0.00619,((Ult10903:0.01106,((Ult10904:0.01134,(
UdnBac35:0.01457,(UltPl393:0.02298,Ult10902:0.02555)0.982.64:0.03204)0.92
0.124:0.01763)0.936.105:0.01167,(Ult10896:5.5E-4,UltPl394:5.5E-
4)0.922.151:5.4E-4)0.858.101:5.4E-
4)0.941.90:0.01863,UltPl395:0.02821)0.823.70:0.00914)0.727.33:0.00889)0.8
57.110:0.01519,Ult10895:0.02664)0.157.12:0.01229,UltPl388:0.01619)0.414.1
2:0.00834)0.886.131:0.00905)0.456.13:5.4E-
4)0.988.87:0.02947)0.591.15:0.0062,((((Ult10923:0.02321,UltPl398:0.02325)
0.760.82:0.00367,UltrS116:0.0152)0.723.30:0.0022,Ult10926:0.02825)0.767.7
9:0.01731,(Ult10927:0.03514,((Ult10925:0.01158,(Ult10928:0.01519,Ult10922
:0.03133)0.869.85:0.00751)0.766.79:0.00387,(Ult10924:5.3E-
4,Ult10929:0.0071)0.791.73:0.01129)0.911.132:0.01762)0.751.53:0.00589)0.9
96.97:0.04184)0.910.108:0.02282)0.843.83:0.01632)0.876.91:0.01624,(Ult109
21:0.0366,(UdnBac36:0.01168,Ult10919:0.019)0.985.80:0.03565)0.866.109:0.0
1027)0.920.125:0.02276,((UltCrat3:0.04054,(Ult10917:0.07125,(Ult10911:5.3
E-4,(Ult10910:5.4E-
4,Ult10912:0.01129)0.998.126:0.03982)0.996.98:0.05535)0.243.12:0.00748)0.
682.20:0.01026,(((Ult10915:0.00368,Ult10916:5.5E-
4)0.999.149:0.09989,(Ult10913:0.01841,Ult10914:0.00424)0.757.65:0.02897)0
.907.110:0.03754,(Ult10918:0.10335,UltPl397:0.04985)0.301.12:0.03245)0.79
4.73:0.0169)0.872.106:0.01252)0.874.131:0.02067)0.656.16:0.02013)0.953.77
:0.04182,((MaiMet15:0.05504,((UltOr131:0.05067,(UltOr130:0.04334,(UltOr12
8:0.0264,UltPl418:0.07329)0.955.82:0.0499)0.611.10:0.02001)0.925.118:0.02
898,((UltOr129:0.05548,(Ult10975:0.04398,(UltFor12:0.02143,Ult10978:0.065
56)0.556.10:0.01836)0.832.80:0.01492)0.784.94:0.00958,(UltPl419:0.04116,U
ltPl420:0.06751)0.115.4:0.0205)0.783.99:0.01349)0.871.110:0.02948)0.590.9
:0.02347,((Ult10932:0.01138,Ult10931:0.00354)0.998.127:0.06691,(((Ult1097
0:0.02558,UltrS117:0.05688)0.214.9:0.00455,((Ult10971:0.00366,Ult10972:0.
0272)0.908.122:0.0183,Ult10973:0.06411)0.799.89:0.01167)0.863.121:0.01807
,((((Ult10977:0.02674,(Ult10966:0.05283,UltPl417:0.0374)0.915.108:0.02031
)0.720.29:0.0042,(Ult10968:0.01931,Ult10967:0.01907)0.902.111:0.0138)0.81
0.68:0.00892,(Ult10969:0.03425,(Ult10974:0.04643,(Ult10964:0.05263,Ult109
65:0.08524)0.537.11:0.01102)0.952.88:0.03423)0.846.112:0.01644)0.856.92:0
.01041,((PhiMikre:0.14095,UltPl416:0.05063)0.173.7:0.01518,(UltPl415:0.06
383,((Ult10962:0.00379,Ult10963:0.00361)0.997.99:0.06832,((UltPl413:0.066
96,UltPl414:0.03171)0.952.89:0.03083,(Ult10961:5.4E-
4,((UltPl411:0.00583,(Ult10960:0.01526,UltPl410:0.00363)0.777.100:0.00604
)0.964.93:0.01921,UltPl412:0.00358)0.979.81:0.0196)0.985.81:0.03511)0.737
.63:0.0047)0.797.67:0.01948)0.981.80:0.05076)0.898.123:0.01974)0.821.65:0
.00905)0.928.85:0.02597)0.192.10:5.4E-
4)0.143.12:0.02988)0.926.125:0.03554,(((Ult10946:0.10023,((Ult10952:0.024
68,Ult10953:0.05915)0.968.79:0.03119,Ult10947:0.02965)0.052.8:0.00889)0.4
26.21:0.01301,((Ult10948:0.05031,(Ult10944:0.03467,Ult10945:0.04261)0.987
.104:0.0425)0.469.14:5.5E-

4,(Ult10949:0.07694,(Ult10950:0.01306,Ult10951:0.00582)0.997.100:0.11053)
0.988.88:0.06098)0.952.90:0.03343)0.926.126:0.01737,(UltPl408:0.03086,(Ul
tPl406:0.04287,UltPl407:0.04677)0.876.92:0.01399)0.723.31:0.00465)0.913.1
22:0.03039)0.841.89:0.04697,(((UltPl382:0.04152,(UltPlan9:0.13529,UltFor4
5:0.07838)0.957.86:0.07584)0.447.13:0.03809,((((((UltPl376:0.03218,(((Ult
10793:0.02766,Ult10804:0.03255)0.909.91:0.01779,((Ult10871:0.0074,UltPl38
0:5.4E-
4)0.941.91:0.01941,Ult10805:0.03484)0.669.17:0.0139)0.730.39:0.00435,Ult1
0792:0.02717)0.756.83:0.00389)0.679.22:0.00689,Ult10802:0.05801)0.772.90:
5.4E-
4,(UltrS112:0.00341,Ult10779:0.05658)0.973.76:0.01929)0.550.9:0.00956,((U
lt10790:0.05272,Ult10801:0.04773)0.559.12:0.02481,UltrS111:0.0067)0.887.1
16:0.02105)0.443.10:0.00871,((Ult10799:0.00602,UltrS110:0.00514)0.962.102
:0.01927,((((((Ult10816:5.4E-
4,(Ult10820:0.015,Ult10823:0.01541)0.846.113:0.01137)0.915.109:0.01091,(U
lt10815:0.03175,(Ult10827:5.3E-
4,Ult10884:0.00349)0.975.77:0.03162)0.778.88:0.0117)0.308.10:5.4E-
4,Ult10825:5.4E-4)0.971.66:5.5E-4,((((Ult10880:0.00367,Ult10881:5.5E-
4)0.999.150:0.06544,(Ult10886:0.00691,(UltPl381:0.0199,Ult10887:0.00402)0
.922.152:0.01708)0.915.110:0.01536)0.943.100:0.02369,(((UltEub17:5.5E-
4,(Ult10817:0.00757,Ult10818:0.01915)0.770.98:0.00359)0.954.88:0.01751,(U
lt10819:0.02497,(Ult10812:0.01209,(UltrS114:0.04108,Ult10885:0.00343)0.90
6.115:0.01192)0.897.108:0.01713)0.906.116:0.01622)0.228.12:5.5E-
4,(Ult10840:0.00802,Ult10813:0.03548)0.890.109:0.01111)0.737.64:0.00322)0
.882.119:0.01129,(Ult10814:0.0305,(Ult10810:5.5E-
4,Ult10811:0.00725)0.991.88:0.05653)0.763.96:0.00843)0.564.8:0.01803)0.96
7.76:0.02268,((UltPl377:0.05397,(Ult10822:5.4E-
4,Ult10821:0.05753)0.902.112:0.03072)0.996.99:5.4E-
4,((Ult10865:0.02308,(PmcBact2:0.03606,Ult10848:5.4E-
4)0.968.80:0.02775)0.897.109:0.01174,((((Ult10806:0.02972,(Ult10870:0.026
92,((Ult10873:0.0,Ult10874:0.0):5.5E-
4,(Ult10872:0.00369,Ult10869:0.00369)0.662.13:5.5E-4)0.487.19:5.3E-
4)0.757.66:0.00538)0.647.7:0.01556,(Ult10847:0.00403,(UltrS113:0.03456,(U
lt10846:0.00374,((Ult10850:0.03832,Ult10851:0.01378)0.630.11:0.01432,(Ult
10875:0.00369,Ult10878:5.5E-
4)0.849.92:0.01149)0.794.74:0.00888)0.843.84:0.01308)0.680.19:0.01277)0.8
27.71:0.00841)0.120.10:0.00274,(((Ult10862:0.01875,Ult10863:5.4E-
4)0.971.67:0.02789,(Ult10808:0.01124,(PmcBacte:0.00742,Ult10809:5.5E-
4)0.759.89:0.0037)0.904.114:0.01192)0.899.107:0.01487,Ult10807:0.02303)0.
983.64:5.4E-4)0.917.100:0.01124,((((((Ult10853:5.5E-4,((UltPl379:5.5E-
4,Ult10852:0.00742)0.675.13:5.5E-
4,Ult10857:0.01506)0.865.109:0.00369)0.801.74:5.4E-
4,Ult10854:0.01916)0.967.77:0.01502,Ult10856:0.00746)0.777.101:0.00367,Ul
t10855:5.5E-
4)0.931.112:0.01851,(Ult10861:0.00407,Ult10864:0.00337)0.915.111:0.0207)0
.889.103:0.02,((Ult10845:0.04369,(Ult10866:5.4E-
4,(((Ult10877:0.00844,((UltEub18:0.0,Ult10876:0.0):0.0456,Ult10883:0.0344
4)0.758.82:0.00915)0.982.65:0.03205,((((Ult10842:0.00386,Ult10843:0.0073)
0.994.95:0.03506,(UltPl378:5.4E-
4,(UltPl372:0.01214,UltPl373:0.0114)0.999.151:0.04754)0.987.105:0.03533)0
.716.27:5.4E-4,(Ult10833:0.00752,((Ult10835:5.4E-
4,TchVagin:0.02269)0.983.65:0.01506,(Ult10836:5.5E-
4,(Ult10830:0.00694,(Ult10829:5.4E-
4,(Ult10828:0.00367,((Ult10834:0.00383,Ult10832:0.03225)0.780.105:0.0036,

Ult10831:0.01119)0.943.101:5.4E-
4)0.807.59:0.0075)0.906.117:0.00204)0.907.111:0.00213)0.958.102:5.4E-
4)0.925.119:0.00751)0.807.60:5.4E-4)0.957.87:0.01112,Ult10867:5.4E-
4)0.917.101:0.01492)0.286.9:0.00758,(Ult10844:0.02714,Ult10868:5.4E-
4)0.982.66:0.02274)0.944.84:5.4E-4)0.817.57:0.00365)0.829.84:5.4E-
4,(Ult10849:0.01548,((Ult10826:0.00743,Ult10860:5.5E-
4)0.814.57:0.00369,(Ult10858:0.0037,(Ult10824:5.5E-
4,Ult10859:0.00369)0.901.114:0.00743)0.800.73:0.00372)0.753.82:0.00367)0.
919.138:0.01153)0.536.14:0.00441)0.933.118:0.02686)0.805.62:5.4E-
4)0.973.77:0.01509)0.864.105:5.4E-
4)0.963.77:0.01508,(Ult10841:0.0074,Ult10882:5.5E-
4)0.914.125:0.00759)0.763.97:5.3E-
4,(Ult10803:0.01292,Ult10879:0.04978)0.928.86:0.02663)0.978.65:0.02412)0.
756.84:0.00333)0.662.14:0.0096,((((((Ult10837:0.01886,Ult10839:0.024)0.94
2.94:0.02026,(Ult10782:0.05004,Ult10838:0.01869)0.866.110:0.01628)0.902.1
13:0.01568,(Ult10781:0.03202,Ult10783:0.03984)0.908.123:0.01544)0.923.146
:0.01563,(Ult10789:0.06403,((Ult10787:0.02867,(Ult10785:0.00674,((UltPl37
4:0.0,UltPl375:0.0):0.00716,Ult10786:0.00776)0.647.8:0.00478)0.319.12:0.0
1081)0.970.80:0.03357,(UltrS108:0.01508,(Ult10780:0.02338,(Ult10778:0.037
17,Ult10788:0.02227)0.891.93:0.02844)0.941.92:0.0386)0.966.104:0.03382)0.
511.10:0.00485)0.603.14:0.02896)0.515.9:5.5E-
4,Ult10784:0.04974)0.972.87:0.0234,((((Ult10795:0.0,Ult10794:0.0):5.5E-
4,Ult10796:0.00369)0.772.91:5.5E-
4,(Ult10797:0.00719,(UltrS109:0.01509,Ult10798:5.5E-4)0.927.108:5.3E-
4)0.907.112:0.00721)0.958.103:0.01106,(Ult10791:0.02862,Ult10800:0.02948)
0.798.72:0.01786)0.894.107:5.3E-
4)0.749.81:0.00441)0.980.65:0.0726)0.991.89:0.09384,(UltrS115:5.5E-
4,((UltPl385:0.00743,((UltPl383:0.0,UltPl386:0.0):5.5E-
4,UltPl384:0.00367)0.889.104:5.3E-
4)0.912.138:0.01838,Ult10888:0.04115)0.976.64:0.02798)0.993.79:0.08786)0.
878.102:0.04021)0.953.78:0.04561)0.817.58:0.03378,((((((((UltPl424:0.0343
,Ult10980:0.03161)0.620.12:0.01177,UltPl426:0.06911)0.010.6:0.003,((((Ult
Or133:0.00368,((UltPl425:0.0317,(UltOr137:0.00369,UltOr136:5.5E-
4)0.932.103:0.02417)0.286.10:0.01263,(UltOr141:0.05257,(UltOr138:0.03113,
UltOr139:5.4E-
4)0.956.98:0.02383)0.914.126:0.02338)0.870.94:0.01508)0.917.102:0.01493,U
ltOr134:0.00412)0.924.107:0.01939,((Ult10987:0.04832,UltOr132:0.03432)0.2
82.9:0.01098,(UltOr135:0.01319,UltOr140:0.03118)0.948.117:0.02888)0.710.2
9:0.0032)0.858.102:0.01112,Ult10979:0.05892)0.687.19:0.01184)0.621.11:0.0
1763,UltOr305:0.11879)0.831.71:0.01075,((Ult10661:0.12899,((Ult10685:0.00
378,(UltPl354:0.00754,Ult10684:0.0074)0.773.94:0.00372)0.994.96:0.0682,((
(Ult10686:0.02924,(Ult10687:0.07307,UltPl356:0.05137)0.786.84:0.00765)0.9
31.113:0.02465,(UltPl355:0.07435,Ult10688:0.05296)0.873.106:0.01589)0.977
.69:0.03932,((((Ult10665:0.04513,((Ult10677:5.4E-
4,(Ult10675:0.04007,(Ult10676:0.01132,(Ult10674:5.5E-4,Ult10678:5.5E-
4)0.877.121:5.1E-
4)0.881.122:0.00773)0.742.48:0.00343)0.955.83:0.03228,((Ult10680:0.02287,
Ult10679:0.05131)0.906.118:0.01621,((UltPl352:0.00369,(Ult10673:0.0048,(U
lt10672:0.01249,((Ult10669:0.01109,Ult10670:5.4E-
4)0.961.87:0.02149,Ult10671:0.02494)0.639.19:0.00869)0.957.88:0.02522)0.8
92.108:0.01028)0.865.110:0.01304,UltPl353:0.02711)0.887.117:0.01957)0.630
.12:0.00519)0.992.90:0.04673)0.663.17:0.01241,(UltOr112:0.04546,Ult10666:
0.02799)0.810.69:0.00877)0.962.103:0.02835,(((((((Ult10682:0.00371,Ult106
83:0.0037)0.961.88:0.0293,Ult10656:0.03936)0.661.15:0.01525,(Ult10667:0.0

3125,Ult10668:0.03294)0.638.13:0.0077)0.281.7:0.00116,Ult10664:0.00338)0.
995.100:0.0429,(((UltPl350:0.00794,(Ult10658:5.5E-
4,(UltPl351:0.01956,(Ult10657:0.00736,(Ult10659:0.0,Ult10660:0.0):5.5E-
4)0.763.98:0.00371)0.793.80:0.00367)0.963.78:0.02698)0.978.66:0.02872,Ult
10662:0.01078)0.915.112:0.0158,((((((Ult10651:0.0,Ult10652:0.0):0.00723,U
lt10653:5.4E-4)0.889.105:0.01468,(Ult10655:0.00736,Ult10654:5.5E-
4)0.869.86:0.01173)0.100.8:0.0075,Ult10567:0.07352)0.916.121:0.01888,UltO
r119:0.03073)0.463.14:0.00398,UdnBac34:0.03049)0.790.80:0.00785)0.725.33:
5.2E-4)0.916.122:0.01596,Ult10663:0.01539)0.365.11:5.5E-
4,((UltOr117:0.02423,(UltOr115:0.06036,(UltOr116:0.05132,(UltOr113:0.0537
8,UltOr114:0.02142)0.946.93:0.03277)0.390.18:0.0132)0.788.80:0.02306)0.73
4.54:0.00225,UltOr118:0.03539)0.864.106:0.00762)0.359.9:0.00742)0.805.63:
0.01452,(Ult10649:0.01674,(((((Ult10690:0.01504,Ult10691:0.00408)0.990.87
:0.05978,Ult11250:0.11745)0.817.59:0.02039,(Ult10689:0.00902,(UltPl357:0.
03681,UltPl358:0.03531)0.363.14:0.00894)1.000.769:0.10646)0.311.9:5.5E-
4,(Ult10599:0.09416,(Ult10648:0.12716,Ult10650:0.0658)0.953.79:0.0531)0.9
27.109:0.02863)0.756.85:0.00746,(((UltPl349:0.04379,((UltPl347:0.03909,(U
lt10585:0.02453,(Ult10590:0.07176,Ult10602:0.0302)0.919.139:0.02332)0.726
.28:0.00469)0.897.110:0.01129,(((((((UltPl345:0.01132,(Ult10632:5.4E-
4,Ult10633:0.00745)0.770.99:0.00365)0.894.108:0.00754,Ult10644:0.00746)0.
807.61:0.00363,((Ult10635:0.00744,(Ult10636:0.00716,Ult10638:0.00717)0.92
9.97:5.4E-4)0.798.73:0.00369,Ult10639:5.5E-4)0.562.14:5.4E-
4)0.831.72:0.00365,(((((Ult10640:5.4E-4,(Ult10642:0.00745,Ult10641:5.5E-
4)0.852.99:0.0037)0.903.99:0.00725,((UltPl346:5.4E-
4,Ult10626:0.02281)0.416.26:0.00738,((Ult10621:5.5E-
4,((Ult10629:0.00367,Ult11249:0.00752)0.773.95:0.00376,(((Ult10624:5.3E-
4,Ult10625:0.01488)0.940.102:0.01519,(Ult10619:0.00743,(Ult10618:5.4E-
4,((Ult10616:5.5E-4,(Ult10614:5.5E-
4,Ult10615:0.0037)0.835.88:0.00369)0.860.78:0.00369,Ult10617:5.5E-
4)0.938.65:0.00747)0.799.90:0.00371)0.788.81:0.00385)0.771.106:0.00356,(U
lt10631:0.00741,((Ult10620:0.0,Ult10628:0.0):5.5E-
4,(Ult10623:0.00741,Ult10622:0.00369)0.752.72:5.5E-4)0.672.15:5.5E-
4)0.816.67:0.00369)0.272.12:5.5E-
4)0.856.93:0.0037)0.820.59:0.00369,Ult10627:5.4E-
4)0.937.104:0.01133)0.242.9:5.4E-4)0.929.98:0.01094,Ult10630:5.4E-
4)0.753.83:0.00367,Ult10634:0.0113)0.932.104:0.01125,Ult10637:5.4E-
4)1.000.770:5.4E-
4)0.490.6:0.0074,((Ult10596:0.01535,(Ult10578:0.01137,Ult10579:0.00744)0.
952.91:0.01531)0.355.16:5.5E-
4,(((Ult10570:0.03089,(Ult10574:0.00787,(Ult10575:0.00356,(Ult10572:0.003
8,(Ult10571:0.00747,Ult10573:0.00368)0.760.83:0.00368)1.000.771:0.05435)0
.367.17:0.00351)0.977.70:0.0281)0.802.66:0.01103,Ult10576:0.01288)0.443.1
1:0.00777,Ult10577:0.01901)0.805.64:0.01484)0.910.109:0.01086)0.000.838:5
.5E-
4,Ult10580:0.04799)0.474.14:0.00201,(Ult10643:0.0232,((Ult10584:0.00766,(
UltPl344:0.02477,(UltPl342:0.00739,((Ult10581:0.0037,(Ult10582:0.00381,Ul
t10583:0.02658)0.766.80:0.0037)0.902.114:0.00744,UltPl343:0.00744)0.911.1
33:5.5E-
4)0.897.111:0.01365)0.967.78:0.02459)0.978.67:0.0265,((Ult10588:0.02777,(
Ult10589:0.01657,Ult10603:0.04127)0.849.93:0.01025)0.893.120:0.0122,Ult10
587:0.01922)0.398.9:0.00352)0.760.84:0.00416)0.913.123:0.01321)0.861.114:
0.00749)0.664.15:0.00168)0.868.90:0.00759,((Ult10613:5.4E-
4,(Ult10594:0.02737,Ult10606:0.03618)0.804.65:0.01121)0.889.106:0.01068,(
((((Ult10569:0.03179,((Ult10601:0.03692,(Ult10566:0.03066,(Ult10562:0.014

46,(Ult10561:0.00368,(Ult10559:0.00369,Ult10560:0.01117)0.953.80:5.4E-
4)0.778.89:0.0082)0.821.66:0.00788)0.904.115:0.01345)0.864.107:0.00987,(U
lt10563:0.02713,(Ult10564:0.01336,(Ult10565:0.00764,Ult10592:0.01622)0.81
8.49:0.01024)0.749.82:0.00574)0.756.86:0.0037)0.895.120:0.0121)0.803.65:0
.00688,((Ult10554:0.01133,((Ult10557:0.00749,Ult10558:0.01126)0.947.84:0.
01568,(Ult10555:0.01468,Ult10556:0.01439)0.743.65:0.00552)0.902.115:0.011
25)0.880.119:0.00765,(((Ult10548:5.5E-
4,(Ult10551:0.02653,Ult10549:0.00369)0.846.114:5.3E-
4)0.838.94:0.0037,(Ult10547:0.00369,Ult10552:5.5E-4)0.657.16:5.4E-
4)0.882.120:0.00749,(Ult10550:0.01104,Ult10553:5.4E-
4)0.863.122:0.00722)0.768.77:0.00367)0.877.122:0.00795)0.760.85:0.00357,U
lt10568:0.01555)0.868.91:0.00864,(Ult10600:0.01061,(Ult10610:0.00564,((Ul
t10611:0.01314,Ult10612:0.00949)0.751.54:0.00373,(Ult10609:0.00948,(Ult10
607:5.5E-
4,Ult10608:0.00743)0.770.100:0.00546)0.937.105:0.01136)0.763.99:0.00374)0
.793.81:0.00642)0.479.16:0.0071)0.875.119:0.01245,(Ult10604:0.02413,Ult10
605:0.03158)0.200.8:0.00741)0.744.60:0.00553)0.740.55:0.00493)0.772.92:0.
00367,((Ult10595:0.03888,((Ult10645:0.00485,Ult10646:0.02661)0.952.92:0.0
1914,(Ult10597:0.01377,Ult10598:0.01938)0.739.46:0.00466)0.148.7:0.0038)0
.868.92:0.00839,(UltOr111:0.01823,(((Ult10593:0.03419,UltPl348:0.05658)0.
902.116:0.01807,(Ult10586:0.04298,Ult10591:0.04621)0.876.93:0.01402)0.080
.6:0.00312,Ult10647:0.02277)0.894.109:0.02058)0.315.13:0.00118)0.742.49:0
.00346)0.763.100:0.00499)0.944.85:0.01837)0.954.89:0.03001)0.896.102:0.02
503)0.952.93:0.03891)0.413.16:0.0174)0.816.68:0.01744,UltPl427:0.10782)0.
853.85:0.01699)0.928.87:0.03414,(Ult10995:0.06091,((Ult10998:0.02427,Ult1
1000:0.03987)0.877.123:0.01537,((((Ult10994:0.14173,(Ult10999:0.11533,(Ul
t10986:0.06655,(Ult10996:0.02274,Ult10997:0.01161)0.969.67:0.04893)0.976.
65:0.05082)0.661.16:0.0276)0.879.117:0.02913,(Ult10991:0.02496,(Ult10990:
0.03781,Ult10992:0.02878)0.813.68:0.01106)0.877.124:0.0144)0.759.90:0.006
37,Ult10993:0.05729)0.679.23:0.00394,(((Ult10989:0.02531,((Ult10985:0.022
52,UltOr490:0.09487)0.837.68:0.0203,(Ult10983:0.03094,Ult10984:0.01175)0.
989.75:0.0515)0.781.83:0.0127)0.293.10:0.00385,(Ult10988:0.01845,Ult11003
:0.02417)0.851.103:0.00981)0.876.94:0.00954,((Ult10981:0.01572,Ult10982:0
.02755)0.749.83:0.00308,(Ult11002:0.02806,Ult11001:0.04505)0.902.117:0.01
559)0.897.112:0.01103)0.920.126:0.01228)0.750.56:0.00467)0.836.78:0.0162)
0.905.112:0.02594)0.948.118:0.02987,(UltPl428:0.01487,(Ult11251:0.00508,U
lt11252:0.01002)1.000.772:0.12741)0.734.55:0.02831)0.888.119:0.02347,(Ult
Pl423:0.07919,((((((Ult10764:0.05781,Ult10765:0.01859)0.738.43:0.01133,(
(Ult10763:0.06215,(UltPl370:5.4E-
4,(Ult10761:0.00755,Ult10762:0.0037)0.794.75:0.00374)0.813.69:5.5E-
4)0.980.66:0.03369,(Ult10766:0.02148,UltPl371:0.01381)0.932.105:0.01806)0
.798.74:0.00929)0.981.81:0.05458,(Ult10768:0.04425,Ult10769:0.02891)0.976
.66:0.05891)0.221.9:0.04195,(((Ult10743:0.07155,(Ult10742:0.04315,(Ult107
41:0.04361,UltPl363:0.03106)0.896.103:0.02085)0.970.81:0.04389)0.961.89:0
.03625,(Ult10746:0.0586,((Ult10747:0.09875,(((((UltPl364:0.00374,UltPl365
:5.5E-
4)0.965.77:0.03228,(Ult10740:0.26887,Ult10748:0.02733)0.500.15:0.03352)0.
906.119:0.02045,((UltPl368:0.04014,Ult10753:0.01784)0.884.116:0.01401,(((
UltPl362:0.03154,(((Ult10733:0.03563,Ult10735:0.04397)0.884.117:0.01704,U
lt10749:0.03782)0.755.70:0.00476,(((Ult10731:0.02659,(Ult10732:0.02446,Ul
t10736:0.03285)0.389.10:0.00892)0.758.83:0.00466,(Ult10752:0.04864,UltPl3
67:0.036)0.249.19:0.00757)0.743.66:0.00495,(Ult10750:0.00825,Ult10751:0.0
0845)0.923.147:0.01647)0.748.67:0.00396)0.859.97:0.00903)0.730.40:0.00272
,Ult10734:0.01979)0.638.14:0.00853,UltPl366:0.044)0.493.15:0.00811)0.146.

8:5.4E-4)0.801.75:0.01885,(Ult10738:5.5E-4,(Ult10737:5.5E-
4,Ult10739:0.00744)0.717.27:0.00368)0.999.152:0.08339)0.342.13:0.01065,((
Ult10758:0.05302,(Ult10756:0.02969,Ult10757:0.03438)0.840.99:0.01622)0.94
5.88:0.03302,(Ult10754:0.05021,Ult10755:0.03834)0.864.108:0.01653)0.863.1
23:0.01577)0.876.95:0.02544)0.101.4:0.01294,(UltPl369:0.04029,(Ult10759:5
.5E-
4,Ult10760:0.00368)0.997.101:0.04873)0.722.27:0.02216)0.814.58:0.01316)0.
672.16:0.01842)0.335.10:5.5E-
4,(Ult10744:0.00945,Ult10745:0.04654)0.995.101:0.06839)0.967.79:0.04439)0
.745.70:0.00739,(Ult10730:0.08491,Ult10767:0.04973)0.813.70:0.01234)0.876
.96:0.02253,(Ult10710:0.04072,(((UltPl359:0.02618,(UltOr120:0.05522,UltOr
121:0.07375)0.784.95:0.00916)0.743.67:0.00385,(((Ult10699:0.06816,(Ult106
98:0.01838,Ult10706:0.02684)0.808.73:0.01247)0.200.9:0.00793,(Ult10705:0.
01583,(Ult10704:0.03194,((Ult10700:0.01922,Ult10707:0.01553)0.966.105:0.0
2352,UltDe148:0.02434)0.735.39:0.00491)0.886.132:0.01035)0.732.31:0.00126
)0.878.103:0.01134,((Ult10703:0.00893,(Ult10701:5.5E-4,Ult10702:5.5E-
4)0.797.68:0.00639)0.742.50:0.00715,(Ult10696:0.0532,(Ult10697:0.03184,(U
lt10692:0.07042,(Ult10694:0.01896,(Ult10693:0.0403,Ult10695:0.02339)0.146
.9:0.0074)0.584.14:0.01416)0.165.5:0.01381)0.682.21:0.01801)0.750.57:0.01
163)0.870.95:0.01393)0.872.107:0.00806)0.878.104:0.01232,((Ult10708:5.4E-
4,Ult10709:0.01545)0.979.82:0.02259,(UltOr122:0.04169,Ult10711:0.0128)0.4
85.16:0.00411)0.864.109:0.01052)0.814.59:0.01094)0.988.89:0.05322)0.931.1
14:0.02946,(Ult10775:0.04915,((((Ult10729:0.05422,((Ult10717:0.00745,Ult1
0716:0.01533)0.991.90:0.03496,(((Ult10728:0.05435,(((UltOr123:0.03538,Ult
10726:0.01619)0.856.94:0.01114,(Ult10727:0.03449,Ult10725:0.02698)0.043.1
0:5.5E-
4)0.846.115:0.01033,(Ult10718:0.0202,(Ult10723:0.01376,Ult10724:0.0265)0.
944.86:0.02281)0.879.118:0.01242)0.394.15:0.00749)0.769.87:0.00524,(Ult10
720:0.02654,(Ult10719:0.03014,Ult10721:0.00962)0.876.97:0.01624)0.759.91:
0.00412)0.844.109:0.00765,((Ult10715:0.0464,(Ult10712:0.02356,Ult10722:0.
00492)0.922.153:0.01767)0.230.7:0.00274,((UltPl361:0.03282,Ult10713:0.016
86)0.470.7:0.00513,Ult10714:0.02055)0.745.71:0.00414)0.919.140:0.01329)0.
303.12:0.00111)0.977.71:0.04259)0.962.104:0.03574,UltPl421:0.0535)0.747.6
2:0.00366,UltPl422:0.04131)0.902.118:0.01583,(((Ult11254:0.0163,(Ult10770
:5.5E-4,Ult10771:5.5E-4)0.794.76:5.3E-
4)0.970.82:0.02926,(Ult10772:0.01731,(Ult10773:0.06102,(Ult10774:0.00143,
Ult11253:0.00598)0.948.119:0.03983)0.890.110:0.02171)0.918.112:0.01997)0.
928.88:0.01905,(Ult10776:0.03375,Ult10777:0.06523)0.848.90:0.01674)0.930.
96:0.0227)0.718.28:0.00289)0.925.120:0.02139)0.871.111:0.01521)0.653.11:0
.01156)0.760.86:0.01126)0.882.121:0.04964)0.833.89:0.03879,(UltPl334:0.11
246,(((Ult10481:0.01459,(((((Ult10491:0.00452,(Ult10529:0.04595,UltEndo5:
0.04516)0.957.89:0.03393)0.432.17:5.5E-
4,(((Ult10515:0.00746,((((((Ult10497:0.0,Ult10498:0.0):0.00751,Ult10496:
0.00358)0.955.84:0.01573,(Ult10499:0.00699,(IsoSpec3:5.4E-
4,PnmctSt9:0.01481)0.918.113:0.01523)0.730.41:0.00387)0.890.111:0.02198,(
Ult10503:0.02044,(UltEndo6:0.00637,Ult10524:0.01626)0.955.85:0.03008)0.47
0.8:0.01913)0.625.10:0.02398,(((IssphSt2:5.4E-
4,Ult10502:0.03081)0.858.103:0.01912,(AgltSoi4:0.03028,IsoSpec2:0.02253)0
.977.72:0.04722)0.193.6:5.4E-
4,((Ult10505:0.00409,(Ult10504:0.00653,UltPl329:0.03392)0.900.91:0.01741)
0.830.75:0.00703,Ult10506:0.00372)0.893.121:0.01101)0.762.93:0.00673)0.79
0.81:0.0115,Ult10514:0.01467)0.128.4:5.5E-
4,((((Ult10510:0.00378,((Ult10508:0.01098,Ult10509:0.0227)0.936.106:5.5E
-4,Ult10507:0.01105)0.643.10:0.00742)0.862.103:5.4E-

4,Ult10513:0.01073)0.477.13:0.00734,Ult10512:0.01161)0.828.63:0.00695,(Ul
t10511:5.4E-4,Ult10517:0.01511)0.834.69:5.4E-
4)0.717.28:0.00732,((Ult10516:0.02599,UltPl330:0.02608)0.902.119:0.02194,
UltIsos2:0.01086)0.893.122:5.5E-
4)0.886.133:0.0073)0.773.96:0.00361)0.947.85:0.01588,(((SnlAcidi:5.4E-
4,Ult10492:0.04376)0.989.76:0.03741,(UltPl327:0.00711,(IsoSpeci:0.03111,U
ltPl328:0.00404)0.756.87:0.00407)0.789.75:0.01083)0.295.17:0.01035,((IsoP
alli:0.05471,(NosLimi4:0.02331,(Ult10500:0.03026,Ult10501:5.5E-
4)0.727.34:0.00344)0.986.77:0.04451)0.886.134:0.01829,(UltIsos3:0.08337,(
Ult10490:0.00324,Ult10489:0.03546)0.935.104:0.025)0.148.8:0.00902)0.777.1
02:0.00702)0.869.87:0.01091)0.941.93:0.01901,(((NosLimic:0.02733,Ult10495
:0.02997)0.876.98:0.01781,((NosLimi3:0.00367,((Ult10523:0.01204,Ult10522:
0.02132)0.387.14:0.00869,(UltrS106:0.03961,(IssphStr:0.02742,((PnmctS26:0
.0131,PnmctSt8:0.0311)0.921.115:0.01824,(Ult10493:0.016,UltPl331:0.02576)
0.871.112:0.01027)0.795.81:5.4E-4)0.933.119:0.01231)0.996.100:5.5E-
4)0.846.116:0.00295)1.000.773:5.3E-
4,(Ult10521:0.00378,(Ult10518:0.00377,(NosLimi2:0.0073,(Ult10519:5.5E-
4,Ult10520:0.0148)0.845.102:0.00751)0.781.84:0.00375)0.752.73:0.00369)0.7
80.106:0.00343)0.969.68:0.0142)0.629.18:5.5E-4,(UltEub16:5.5E-
4,Ult10494:0.00354)0.976.67:0.01199)0.329.11:0.00597)0.783.100:0.00463)0.
930.97:0.01933,((Ult10530:0.02408,(Ult10531:0.00536,Ult10532:0.002)0.869.
88:0.02647)1.000.774:0.08143,((Ult10527:0.04355,Ult10528:5.4E-
4)0.996.101:0.03245,(BacElli2:0.0023,((((Ult10479:0.00259,Ult10478:0.0655
9)0.999.153:0.00115,Ult10486:0.00738)0.900.92:5.4E-
4,((Ult10485:0.01091,UltEub15:0.00706)0.928.89:5.4E-
4,UltIsosp:0.019)0.505.5:0.0074)0.754.71:0.00363,Ult10480:5.3E-
4)0.895.121:0.00879)0.488.14:0.00666)0.047.6:5.3E-
4)0.885.103:0.01438)0.784.96:0.00721,(Ult10488:0.02235,((Ult10487:0.01117
,(UltPl326:0.00313,((UdnBac33:0.03055,(UltEndo7:0.0344,(((UltrS107:0.0102
5,(Ult10526:0.00264,UltSlu28:0.05051)0.999.154:0.04718)0.102.7:0.0095,Ult
Pl333:0.0792)0.774.94:0.00675,Ult10525:0.01157)0.105.13:5.4E-
4)0.955.86:0.01538)0.826.65:0.00781,BacElli3:0.02277)0.949.102:0.0248)0.9
18.114:0.01604)0.021.5:5.5E-
4,UltPl332:0.0659)0.953.81:0.02782)0.956.99:0.02019)0.746.58:0.00407,(Ult
10483:0.00739,(Ult10482:0.01119,Ult10484:0.0038)0.958.104:0.01539)0.849.9
4:0.00788)0.764.94:0.00802)1.000.775:0.09449,((Ult10457:0.0606,(Ult10464:
0.08644,(((Ult10452:0.08021,Ult10447:0.02944)0.734.56:0.03802,Ult10451:0.
04731)0.262.10:0.00725,(((Ult10448:0.06627,(Ult10455:0.04514,Ult10454:0.0
6764)0.782.105:0.01032)0.500.16:0.01256,((Ult10449:0.02151,(Ult10450:0.14
067,Ult10456:0.06849)0.215.6:0.02492)0.937.106:0.04457,(Ult10445:0.00375,
Ult10446:0.00742)0.921.116:0.02078)0.786.85:0.0141)0.640.14:0.01499,(UltP
l324:0.09042,Ult10453:0.03382)0.983.66:0.0559)0.738.44:0.0095)0.916.123:0
.03636)0.243.13:0.01422)0.950.92:0.03806,(((Ult10413:0.04863,(Ult10411:0.
03352,((Ult10436:0.07628,Ult10419:0.04242)0.910.110:0.02621,((((((Ult103
31:0.01835,((Ult10350:0.07593,(Ult10458:0.06528,Ult10459:0.02556)0.983.67
:0.05163)0.788.82:0.01142,UltPl312:0.0521)0.883.120:0.017)0.131.8:0.00395
,(((Ult10393:0.05049,Ult10376:0.05387)0.897.113:0.02635,((Ult10460:0.0400
7,((UltCrat2:0.05725,Ult10375:0.03616)0.848.91:0.01862,Ult10461:0.11314)0
.777.103:0.02276)0.693.20:0.02603,(Ult10373:0.0372,Ult10374:0.02274)0.985
.82:0.04393)0.944.87:5.5E-
4)0.766.81:0.00768,(((((UltPl290:0.00789,((Ult10372:0.00979,UltTher7:0.00
918)0.528.11:0.00801,(Ult10371:0.03008,(UltPl303:0.00433,UltGemm6:0.01453
)0.903.100:0.01932)0.907.113:0.02036)0.959.100:0.02798)0.653.12:0.00753,(
(((Ult10357:0.04553,UltPl291:0.04105)0.700.26:0.00549,(((UltPl316:0.0,Ult

Pl317:0.0):0.06883,(Ult10366:0.01654,UltPl302:0.06026)0.633.8:0.03161)0.9
90.88:0.07366,((Ult10353:0.01249,Ult10368:0.08356)0.667.20:0.02937,(((((U
ltPl283:0.0744,(((Ult10264:0.04147,UltGemm5:0.02337)0.748.68:0.00417,(Ult
10269:0.02818,Ult10268:0.01223)0.738.45:0.00271)0.841.90:0.0078,((Ult1026
5:0.01415,(Ult10266:0.0,Ult10267:0.0):0.00969)0.925.121:0.01865,((Ult1027
1:0.00745,UltrSo97:5.5E-
4)0.839.90:0.00373,Ult10270:0.01898)0.948.120:5.4E-
4)0.867.109:0.00737)0.812.64:0.0117)0.728.38:0.01767,(ZavFormo:5.5E-
4,GemmaSt4:0.02273)0.908.124:0.02291)0.844.110:0.01698,((Ult10263:0.02482
,(Ult10248:5.4E-4,((UltPl278:0.0189,Ult10250:5.5E-
4)0.986.78:0.04862,Ult10249:0.04422)0.849.95:0.01884)0.830.76:0.02683)0.8
98.124:0.03259,(((Ult10275:0.01146,Ult10276:0.00359)0.965.78:0.03093,(Ult
10274:0.02448,(Ult10273:0.01362,UltPl284:0.00522)0.412.12:0.00756)0.912.1
39:0.01531)0.919.141:0.01731,(Ult10272:0.05091,Ult10277:0.09443)0.360.14:
0.00857)0.746.59:0.00573)0.923.148:0.03868)0.922.154:0.03585,(((((Ult1021
4:0.01925,(((Bac2BP58:0.0,UltEub13:0.0):5.5E-4,UltEub14:5.5E-
4)0.996.102:0.03101,((Ult10199:5.4E-
4,(((Ult10201:0.0037,(Ult10200:0.00747,Ult10203:0.01883)0.109.11:5.5E-
4)0.907.114:0.00752,((Ult10198:0.00745,UltrSo95:5.4E-
4)0.969.69:0.01852,((GemmaStr:5.4E-
4,GmtObscu:0.01466)0.880.120:0.0076,(GemmaSt2:5.5E-
4,GmtObsc2:0.019)0.870.96:0.00698)0.497.5:0.00375)0.831.73:5.4E-
4)0.918.115:0.00748,Ult10204:5.4E-
4)0.896.104:0.00739)0.971.68:0.01498,((((Ult10211:0.00368,Ult10212:0.0037
2)0.777.104:0.00564,((Ult10205:5.5E-
4,Ult10206:0.00743)0.783.101:0.00371,(UltGemm2:0.01546,((GemmaSt3:0.0268,
Ult10210:5.5E-4)0.894.110:0.0109,(UltGemma:0.01088,(Ult10208:5.5E-
4,(Ult10209:0.01119,Ult10207:0.02282)0.302.8:5.5E-4)0.885.104:5.4E-
4)0.673.11:0.00749)0.932.106:0.0208)0.782.106:0.00972)0.773.97:0.00557)0.
653.13:0.0075,Ult10202:0.00556)0.759.92:0.00557,Ult10213:0.00747)0.939.10
1:0.01125)0.861.115:5.5E-
4)0.848.92:0.00744)0.956.100:0.02339,(Ult10255:0.0,Ult10256:0.0):0.01564)
0.894.111:0.01904,(Ult10254:0.01863,(Ult10251:0.03439,(UltPl279:0.0075,Ul
t10252:0.01947)0.873.107:0.01375)0.931.115:0.01668)0.950.93:0.02407)0.489
.12:5.5E-
4,((((Ult10223:0.00371,(Ult10225:0.00371,Ult10226:0.00371)0.785.91:0.0036
8)0.802.67:0.00367,Ult10224:5.5E-
4)0.999.155:0.04588,(UltrSo96:0.01021,(Ult10227:0.01879,Ult10228:5.5E-
4)0.985.83:0.0284)0.667.21:0.00773)0.857.111:0.0089,(Ult10262:0.03482,Ult
Pl282:5.5E-4)0.878.105:0.00756)0.940.103:0.01523)0.858.104:5.5E-
4,(((Ult10261:0.07199,(((UltPl285:0.02697,(((Ult10282:0.00381,Ult10281:0.
01919)0.821.67:0.00703,((Ult10280:0.01787,(Ult10278:0.0112,Ult10279:5.5E-
4)0.960.96:0.02281)0.872.108:0.01411,(Ult10283:0.0,Ult10284:0.0):5.4E-
4)0.807.62:0.0077)0.984.73:0.03583,(((UltPl311:0.03907,Ult10430:0.03957)0
.936.107:0.03642,((Ult10285:0.05546,(Ult10425:0.04002,Ult10426:0.02045)0.
183.12:5.5E-
4)0.926.127:0.01584,Ult10427:0.02915)0.729.25:0.00548)0.789.76:0.01264,((
Ult10429:0.01259,(Ult10292:0.02889,(UltPl286:0.04755,Ult10286:0.03505)0.9
09.92:0.02339)0.881.123:0.01591)0.845.103:0.01202,(Ult10428:0.01278,(((Ul
t10287:0.01296,Ult10290:0.02657)0.783.102:0.00732,(Ult10288:0.0,Ult10289:
0.0):5.4E-
4)0.917.103:0.01124,Ult10291:0.00376)0.986.79:0.03073)0.631.13:0.00437)0.
794.77:0.01347)0.918.116:0.02497)0.587.13:0.02522)0.662.15:0.00139,(Ult10
257:0.02966,UltPl280:0.03112)0.347.11:0.01738)0.952.94:0.03374,(UltPl281:

0.03385,Ult10260:0.02246)0.891.94:0.02376)0.766.82:0.00373)0.813.71:0.007
63,((Ult10259:0.01052,((UltGemm3:0.00369,UltPiete:5.5E-
4)0.932.107:0.01661,Ult10258:0.02667)0.687.20:0.00768)0.878.106:0.01225,U
ltGemm4:0.00847)0.941.94:0.01677)0.874.132:0.00796,((Ult10253:0.03107,(Ul
t10229:0.01714,(Ult10232:0.04203,((((Ult10216:5.3E-
4,Ult10218:0.0746)0.975.78:0.02288,((Ult10219:0.03161,UltPl276:0.06193)0.
904.116:0.02202,Ult10215:0.03001)0.909.93:0.01972)0.675.14:0.00146,Ult102
17:0.08046)0.209.11:0.01049,(Ult10221:0.0152,(Ult10222:0.00767,(Ult10220:
0.01493,BacEnr37:5.4E-
4)0.389.11:0.00737)0.969.70:0.01933)0.763.101:0.00383)0.938.66:0.02096)0.
882.122:0.01193)0.800.74:0.00991)0.336.11:0.0038,(Ult10230:0.01127,Ult102
31:0.01547)0.968.81:0.02632)0.901.115:0.01492)0.861.116:0.00912)0.692.12:
0.00345)0.963.79:0.03132,((UltSlu26:0.02791,UltSlu27:0.02509)0.972.88:0.0
3098,(Ult10378:0.02136,Ult10354:0.10005)0.740.56:0.00532)0.957.90:0.02787
)0.909.94:0.02193)0.761.74:0.0117)0.537.12:0.01092)0.686.14:0.00206,((Ult
10365:0.02027,(Ult10363:0.04075,(Ult10364:0.03822,(Ult10362:0.05172,(Ult1
0360:0.01446,Ult10361:0.04242)0.173.8:0.00865)0.847.125:0.02927)0.592.13:
0.03643)0.731.30:0.01564)0.585.6:0.01536,(((Ult10437:0.02458,(UltPl318:0.
00744,UltPl319:5.5E-
4)0.893.123:0.01502)0.905.113:0.02137,UltrS104:0.0603)0.740.57:0.00806,Ud
nBac30:0.0281)0.792.95:0.00818)0.872.109:0.01624)0.969.71:0.02224,(((((((
UdnBac32:0.0114,(Ult10356:5.3E-
4,UltPl299:0.02723)0.931.116:0.01134)0.874.133:0.01144,((UltrS103:0.05678
,(Ult10442:0.05081,(UltrS102:0.0564,UltPl315:0.03742)0.585.7:0.02936)0.74
7.63:0.01026)0.844.111:0.01786,(Ult10405:0.03393,Ult10404:0.00945)0.934.9
2:0.03331)0.781.85:0.02183)0.751.55:0.00485,(Ult10304:0.01907,Ult10305:0.
02341)0.957.91:0.02263)0.743.68:0.00631,UltPl287:0.07152)0.916.124:0.0172
4,(Ult10379:0.04491,(Ult10307:0.03176,(UltrSo99:0.02239,(Ult10313:0.02263
,(((((((Ult10308:0.00373,Ult10312:0.00368)0.327.13:5.5E-
4,(Ult10309:0.0,Ult10318:0.0):5.5E-4)0.522.3:5.5E-4,Ult10314:5.5E-
4)0.549.15:5.5E-4,Ult10316:5.5E-4)0.314.11:5.5E-
4,Ult10311:0.01493)0.079.5:5.5E-4,Ult10310:0.00738)0.211.11:5.5E-
4,Ult10317:5.5E-4)0.789.77:5.5E-
4,Ult10315:0.011)0.737.65:0.00446)0.988.90:0.03616)0.877.125:0.01371)0.74
0.58:0.00612)0.903.101:0.01801)0.710.30:0.00389,((((UltPl310:0.04146,(Ult
10434:0.00233,(Ult10432:0.01141,Ult10433:5.4E-
4)0.869.89:0.0089)0.911.134:0.02355)0.676.12:0.00651,Ult10422:0.04837)0.4
85.17:0.01266,(((((Ult10407:0.0208,(Ult10359:0.01418,UltPl301:0.01335)0.7
47.64:0.00629)0.734.57:0.0045,(Ult10345:0.00809,Ult10341:0.03267)0.298.11
:0.00807)0.793.82:0.00799,(UltPl298:0.03012,UltPl309:0.04226)0.856.95:0.0
0995)0.544.11:0.00377,((UltPl300:0.01096,PttBacte:0.02397)0.759.93:0.0152
8,(Ult10340:0.02139,Ult10406:0.03556)0.464.15:0.0141)0.834.70:0.01468)0.7
97.69:0.00639,((Ult10431:0.02715,(Ult10342:5.5E-4,UltPl313:5.5E-
4)0.958.105:5.4E-
4)0.922.155:0.01143,((Ult10435:0.00396,UltPl314:0.00792)0.999.156:0.05123
,(((((UltrS105:0.01821,(Ult10462:0.00768,Ult10463:0.00727)0.859.98:0.0088
3)0.965.79:0.02413,((Ult10396:0.04242,(Ult10397:5.3E-
4,Ult10398:0.00742)0.959.101:0.02163)0.706.27:0.00399,(UltPl305:0.03225,(
Ult10412:0.01545,Ult10418:0.05246)0.893.124:0.01703)0.919.142:0.01744)0.8
63.124:0.01258)0.756.88:0.006,(UltPl306:0.00717,((((Ult10402:0.015,(UltPl
308:0.01518,Ult10400:0.02329)0.729.26:0.00367)0.789.78:0.00371,Ult31658:0
.0037)0.987.106:5.5E-
4,(Ult10403:0.0194,Ult10401:0.00373)0.131.9:0.00759)0.942.95:0.01508,(Ult
10399:5.5E-4,UltPl307:0.00369)0.730.42:5.4E-4)0.906.120:5.5E-

4)0.922.156:0.01972)0.863.125:0.01312,(Ult10369:0.03266,(Ult10349:0.0298,
((((UdnBac31:0.02527,Ult10424:0.02088)0.843.85:0.02793,(Ult10443:5.3E-
4,Ult10444:0.02677)0.933.120:0.03642)0.963.80:0.0315,Ult10423:0.01424)0.7
02.19:0.00958,Ult10329:0.02829)0.861.117:0.01781)0.474.15:0.01096)0.866.1
11:0.01281)0.644.13:0.00413,(Ult10348:5.4E-
4,Ult10395:0.04353)0.986.80:0.03958)0.202.12:0.00921)0.759.94:0.00745)0.8
84.118:0.00262)0.824.66:0.00148)0.801.76:0.0036,(Ult10439:0.03201,(UltPl3
22:0.02676,(UltPl320:5.5E-
4,UltPl321:0.00742)0.976.68:0.02521)0.865.111:0.014)0.929.99:0.02179)0.95
4.90:0.01468)1.000.776:5.4E-
4,((UltPl289:0.00226,(UltPl288:0.02573,(UltrSo98:0.01514,(Ult10300:0.0,Ul
t10301:0.0):5.4E-
4)0.851.104:0.01035)0.817.60:0.0125)0.967.80:0.03691,((Ult10334:0.04972,(
(Ult10330:0.02456,(Ult10306:0.05997,(Ult10420:0.03242,Ult10440:0.04369)0
.786.86:0.01123)0.379.8:0.01121)0.870.97:0.01108,(Ult10302:0.00574,(Ult10
408:0.04388,Ult10441:0.01414)0.794.78:0.00799)0.882.123:0.01081)0.929.100
:0.01195,(Ult10421:0.04811,(Ult10438:0.05838,Ult10346:0.01567)0.861.118:0
.01107)0.603.15:5.4E-
4)0.861.119:0.01039)0.731.31:0.00454,Ult10339:0.03134)0.879.119:0.01644)0
.849.96:0.02612)0.812.65:0.00353)0.681.15:5.5E-
4)0.735.40:0.00571,(Ult10370:0.01558,Ult10380:0.02372)0.876.99:0.01798)0.
813.72:0.01417,Ult10355:0.04569)0.918.117:0.01994,(Ult10377:0.06649,((Ult
10344:0.0,Ult10352:0.0):0.01627,Ult10351:0.011)0.957.92:0.028)0.430.15:0.
00496)0.797.70:0.00805)0.745.72:0.00547)0.860.79:0.01236,Ult10394:0.02994
)0.884.119:0.0143,(((Ult10388:0.02698,((Ult10386:0.05026,Ult10385:0.046)0
.455.21:0.01055,(Ult10384:0.05406,Ult10387:0.01605)0.353.7:0.01117)0.879.
120:0.02479)0.896.105:0.02294,(((((UltPl264:0.05334,(((((Ult10190:0.0186,
((Ult10156:5.5E-4,Ult10157:5.5E-4)0.906.121:0.01108,(Ult10153:5.5E-
4,(Ult10155:0.01504,Ult10154:5.5E-4)0.961.90:0.01131)0.610.10:5.3E-
4)1.000.777:0.0731)0.908.125:0.02488,((UltPl269:0.05151,((UltPl270:5.5E-
4,UltPl271:0.0074)0.974.57:0.03564,((Ult10186:0.0,Ult10187:0.0):5.3E-
4,(Ult10189:0.01138,(Ult10188:5.3E-
4,UltPl268:0.01495)0.809.77:0.00758)0.528.12:0.01504)0.957.93:0.02982)0.4
85.18:0.00882)0.224.7:0.01764,(UltPl275:0.00783,((Ult10196:5.4E-
4,UltVerru:0.03992)0.952.95:5.5E-
4,((((Ult10193:0.01877,((Ult10191:0.00745,UltPl273:5.5E-4)0.740.59:5.4E-
4,(Ult10192:0.00386,ScsPalud:0.01929)0.247.8:0.00363)0.953.82:5.4E-
4)0.882.124:0.01133,UltPl272:0.01181)0.203.10:0.00711,PnsSpec9:5.5E-
4)0.905.114:0.01419,(UltrSo94:0.01379,(UltPl274:0.00498,(Ult10194:0.00369
,Ult10195:5.5E-
4)0.872.110:0.0101)0.964.94:0.01975)0.903.102:0.01559)0.441.16:0.00967)0.
886.135:0.0147)0.987.107:0.03701)0.825.70:0.01312)0.664.16:5.4E-
4,(((PnsSpec8:0.01511,(Ult10184:0.00743,UltPl266:0.00749)0.853.86:0.00747
)0.900.93:0.01191,(UltPl267:0.01744,(UltrSo93:0.01303,Ult10185:0.0179)0.6
36.10:0.00801)0.897.114:0.01294)0.848.93:0.00832,(Ult10183:0.0431,((Ult10
181:0.0,Ult10182:0.0):0.01523,(Ult10180:0.00874,(Ult10179:0.00745,UltPl26
5:5.5E-
4)0.975.79:0.02752)0.912.140:0.02389)0.742.51:0.02109)0.880.121:0.022)0.9
37.107:0.02109)0.850.114:0.01388,((Ult10094:0.0232,(Ult10092:0.00373,(Ult
10093:0.00374,(UltPl232:0.03384,Ult10091:0.02124)0.739.47:0.00179)0.772.9
3:0.00373)0.844.112:0.02049)1.000.778:0.14126,(((((UltPl226:0.02025,Ult10
087:0.01555)0.999.157:0.07519,(UltPl204:5.4E-
4,(Ult10062:0.01142,Ult10063:0.01142)0.907.115:0.01899)0.841.91:0.02025)0
.855.99:0.02873,(PnsSpec6:0.08813,(((Ult10026:0.02338,((Ult10046:0.01109,

(((Ult10048:0.00736,(Ult10042:0.00728,Ult10043:0.01907)0.866.112:0.00755)
0.005.4:5.5E-4,(Ult10044:0.0,Ult10045:0.0,UltrB189:0.0):5.5E-
4)0.384.13:5.5E-4,Ult10047:0.00747)0.908.126:5.5E-
4)0.918.118:0.01118,((Ult10049:0.0,UltPl200:0.0):0.00373,(Ult10050:0.0113
,(Ult10051:0.0,Ult10052:0.0,Ult10053:0.0):0.00373)0.760.87:0.00372)0.870.
98:0.00745)0.906.122:0.01262)0.328.14:0.00664,((((((Ult10036:0.00369,Ult1
0037:5.5E-
4)0.900.94:0.00743,(((Ult10034:0.0,Ult10035:0.0,Ult10041:0.0,UltGa289:0.0
):5.5E-4,(Ult10033:0.00369,Ult10040:0.00369)0.758.84:5.5E-
4)0.954.91:5.5E-
4,((Ult10057:0.00322,Ult10056:0.00808)0.887.118:0.00795,(Ult10031:0.00759
,(Ult10028:0.00371,((Ult10029:0.0,Ult10032:0.0,Ult10064:0.0):5.5E-
4,(Ult10027:0.01501,Ult10030:0.00369)0.502.9:5.5E-4)0.712.21:5.5E-
4)0.795.82:0.00372)0.749.84:0.00324)0.875.120:0.00805)0.384.14:0.00749)0.
900.95:5.4E-
4,(UltPl197:0.00747,(Ult10038:0.0037,UltPl198:0.00744)0.772.94:0.00374)0.
783.103:0.00366)0.949.103:0.00744,Ult10055:0.01124)0.786.87:5.4E-
4,Ult10054:5.4E-4)0.887.119:5.5E-4,((Ult10039:5.4E-
4,UltPl199:0.00371)0.920.127:0.01158,(((Ult10025:0.00542,((UltPl195:0.003
69,((Ult10021:0.0,Ult10023:0.0):5.5E-
4,(UltPl196:0.00369,Ult10022:0.00745)0.372.13:5.5E-4)0.610.11:5.5E-
4)1.000.779:5.5E-
4,Ult10024:0.03932)0.952.96:0.01454)0.907.116:0.01489,UltPl194:0.00737)0.
950.94:0.0196,((Ult10018:5.5E-4,Ult10019:0.00369)0.549.16:5.5E-
4,(Ult10020:0.00374,(Ult10017:0.00372,(Ult10015:0.00371,Ult10016:0.00373)
0.880.122:0.00755)0.890.112:0.00756)0.885.105:0.00749)0.209.12:5.4E-
4)0.330.11:0.00749)0.914.127:0.01156)0.774.95:0.00461)0.000.839:0.00346,(
(UltPl202:0.04409,Ult10059:5.5E-4)1.000.780:0.0532,((UltPl201:5.4E-
4,Ult10058:0.00747)0.977.73:0.022,(Ult10061:5.5E-
4,(UltPl203:0.0075,Ult10060:0.00749)0.766.83:0.00362)0.951.99:0.01712)0.0
70.11:0.00199)0.859.99:0.03491)0.957.94:0.04672)0.863.126:0.02488)0.731.3
2:0.00902,((UltPl227:0.01418,(PnsBrasi:0.02657,(Ult10100:5.5E-
4,Ult10101:0.00365)0.998.128:0.0628)0.913.124:0.03115)0.847.126:0.01578,U
lt10088:0.01942)0.973.78:0.03522)0.830.77:0.01606,(UltPl205:0.05134,(UltP
l231:0.02247,((Ult10089:0.00366,UltPl229:0.00375)0.876.100:0.01156,((UltP
l230:0.01141,Ult10090:0.00759)0.880.123:0.01325,UltPl228:0.0101)0.415.11:
0.00767)0.761.75:0.00977)0.943.102:0.02861)0.886.136:0.0207)0.929.101:0.0
3899)0.844.113:0.01776)0.052.9:0.00756,(((((Ult10116:0.00368,Ult10117:5.5
E-
4)0.985.84:0.06045,(Ult10115:0.00166,UltPl237:0.01374)0.564.9:0.03442)0.9
97.102:0.07004,((Ult10168:0.00367,((UltPl257:5.5E-4,(UltPl256:5.5E-
4,(UltPl258:0.00369,UltPl255:0.00369)0.882.125:5.5E-4)0.724.28:5.5E-
4)0.895.122:5.3E-4,(Ult10170:0.04429,Ult10169:0.07377)0.965.80:5.4E-
4)0.779.84:0.00373)0.786.88:0.00601,UltPl259:0.02963)0.810.70:0.016)0.987
.108:0.04627,((((Ult10150:0.02775,((Ult10149:0.00345,(Ult10148:5.4E-
4,(Ult10147:0.00763,UltPl246:0.00364)0.664.17:0.01141)0.986.81:0.03162)0.
971.69:0.01978,(UltrSo92:0.02686,(((Ult10151:0.00391,(Ult10140:0.0319,Ult
10152:0.01155)0.773.98:0.00361)0.983.68:0.02398,(UltPl248:0.02415,(((Ult1
0141:0.0,Ult10146:0.0):5.4E-
4,(Ult10143:0.0143,(Ult10126:0.01571,Ult10142:0.00717)0.749.85:0.00584)0.
898.125:0.01067)0.802.68:0.00369,(Ult10145:0.00369,Ult10144:5.5E-
4)0.302.9:5.4E-
4)0.970.83:0.02012)0.743.69:0.0027)0.771.107:0.00412,(AgltSoi3:0.01912,Ul
t10158:5.3E-4)0.976.69:0.01923)0.516.7:0.00708)0.886.137:5.4E-

4)0.859.100:0.00799)0.896.106:0.01197,((Ult10177:0.03195,(Ult10166:0.0111
,(((UltPl251:0.0,UltPl252:0.0):0.01363,UltPl253:0.01472)0.569.12:0.01125,
UltPl254:0.02306)0.839.91:0.01401)0.857.112:0.00916)0.907.117:0.01269,Ult
Pl247:0.06389)0.693.21:0.00184)0.802.69:0.00729,(((Ult10171:0.03594,UltPl
260:0.03347)0.880.124:0.01568,(((Ult10097:0.01165,(Ult10095:0.00366,Ult10
096:0.0075)0.976.70:0.03205)0.960.97:0.02902,(UltOr108:0.03277,Ult10161:0
.03831)0.614.9:0.01221)0.736.48:0.00478,(Ult10098:0.02487,UltPl233:0.0504
8)0.822.62:0.01274)0.905.115:0.01724)0.894.112:0.01799,(((((UltPl238:0.06
377,Ult10118:0.07079)0.840.100:0.01731,(Ult10119:0.0261,Ult10120:0.01389)
0.877.126:0.01272)0.879.121:0.01519,(((Ult10163:0.01838,(Ult10162:5.3E-
4,UltOr109:0.00752)0.753.84:0.00585)0.849.97:0.01081,(Ult10167:0.04035,(U
ltPl250:5.4E-4,(PnsMaris:5.4E-4,(PnsSpec7:5.5E-4,Ult10165:5.5E-
4)0.946.94:0.01127)0.956.101:0.01109)0.988.91:0.04752)0.926.128:0.02906)0
.703.28:0.01259,(Ult10164:0.00582,(((Ult10172:0.0,UltPl261:0.0):5.4E-
4,Ult10173:0.00742)0.948.121:0.02226,(Ult10175:5.5E-4,(Ult10174:5.4E-
4,UltPl262:0.0037)0.913.125:0.01468)0.043.11:0.01709)0.881.124:0.01542)0.
762.94:0.00589)0.883.121:0.0142)0.593.7:0.01228,(UltPl234:0.01603,(Ult101
78:0.05113,(UltPl235:0.05346,Ult10176:0.03055)0.723.32:0.01499)0.763.102:
0.00552)0.793.83:0.00643)0.689.17:0.00322,(UltPl263:0.03266,(Ult10159:0.0
2108,Ult10160:0.02006)0.276.11:0.00608)0.906.123:0.01479)0.850.115:0.0116
3)0.788.83:0.01337)0.797.71:0.00683,((((Ult10111:0.02983,((((Ult10104:0.0
,Ult10105:0.0):5.5E-
4,(((Ult10106:0.01139,Ult10110:0.01105)0.767.80:0.00372,(Ult10109:0.01453
,Ult10108:0.0366)0.731.33:0.00281)0.320.6:5.5E-
4,Ult10103:0.01115)0.034.8:5.5E-4)0.226.9:5.5E-
4,Ult10107:0.00739)0.437.16:5.5E-
4,Ult10102:0.00366)0.791.74:0.00627)0.450.13:0.01538,((UltPl236:0.00768,U
lt10112:0.01118)0.850.116:0.01285,UltCrate:0.0369)0.749.86:0.00542)0.753.
85:0.023,((Ult10114:0.01137,Ult10113:5.3E-4)0.709.23:5.4E-
4,(Ult31660:0.01544,Ult27166:0.09433)0.960.98:0.05162)0.923.149:0.04209)0
.963.81:0.03432,((UltPl245:0.03542,((Ult10133:5.3E-
4,(Ult10134:0.03534,(Ult10131:5.5E-4,Ult10132:0.00744)0.997.103:5.5E-
4)0.669.18:0.01113)0.979.83:0.02596,(Ult10138:0.02342,Ult10137:0.01969)0.
951.100:0.02532)0.394.16:0.00382)0.868.93:0.00861,((Ult10125:0.00746,(Ult
Pl243:0.00744,(Ult10127:5.4E-
4,(UltPl244:0.00372,Ult10130:0.01138)0.769.88:0.00372)0.859.101:0.0037)0.
378.17:5.4E-4)0.769.89:0.00541,((UltB9681:0.01153,Ult10139:5.3E-
4)0.764.95:0.00544,(UltPl240:5.4E-4,(((Ult10122:0.0,Ult10123:0.0):5.5E-
4,(UltPl242:0.01124,(UltPl241:0.01124,(Ult10128:0.00369,Ult10129:0.00742)
0.901.116:5.4E-4)0.558.11:0.00368)0.790.82:5.4E-
4)0.908.127:0.0033,(Ult10121:0.00798,Ult10124:0.01109)0.224.8:0.0075)1.00
0.781:5.3E-
4)0.885.106:0.00928)0.736.49:0.00758)0.826.66:0.00883)0.900.96:0.01625)0.
745.73:0.00525)0.872.111:0.00892)0.524.10:5.5E-
4,(((Ult10136:0.01164,Ult10135:0.02348)0.988.92:0.04665,UltPl249:0.03688)
0.855.100:0.01842,((UltPl239:0.01749,((Ult10077:0.00373,UltPl218:0.02289)
0.742.52:0.00285,(((Ult10078:0.0,Ult10080:0.0):5.5E-
4,(Ult10076:0.00368,Ult10075:0.03506)0.613.16:5.3E-4)0.949.104:5.4E-
4,(UltPl216:5.3E-4,((UltPl213:5.5E-4,((Ult10079:0.0,UltPl214:0.0):5.5E-
4,UltPl217:0.00369)0.843.86:0.00371)0.853.87:0.0037,UltPl215:5.3E-
4)0.923.150:0.00747)0.987.109:0.01888)0.487.20:0.00838)1.000.782:0.07171)
0.802.70:0.02466,((UltPl211:0.02685,((UltPl210:0.00754,(Ult10074:0.00742,
(UltPl209:5.5E-
4,Ult10073:0.00738)0.830.78:0.00757)0.771.108:0.00391)0.798.75:0.00372,Ul

tPl212:0.00377)0.778.90:0.0045)0.890.113:0.01217,(((((Ult10067:0.01155,Ul
t10070:0.01829)0.872.112:0.01435,(PnsSpeci:0.02362,(UltPl207:0.00744,Ult1
0068:0.00765)0.734.58:0.00353)0.906.124:0.01804)0.517.7:0.009,(Ult10069:0
.00374,(UltPl206:0.01522,(Ult10065:0.00349,Ult10066:0.01177)0.919.143:0.0
117)0.870.99:0.00765)0.979.84:5.5E-
4)0.247.9:0.00553,(((UltPl225:0.01454,(((((Ult10081:0.02745,(UltPl222:0.0
2064,(UltPl220:0.00731,UltPl221:0.01161)0.794.79:0.00829)0.893.125:0.0173
8)0.939.102:0.01745,(PnsSpec4:0.00944,UltPl219:0.01005)0.741.57:0.00425)0
.874.134:0.01608,(UltPl224:0.01903,(Ult10085:0.01533,Ult10086:0.01941)0.9
98.129:0.06527)0.701.23:0.00568)0.425.10:0.00836,(PnsSpec5:5.3E-
4,(Ult10082:5.5E-
4,UltPl223:0.02302)0.969.72:0.01508)0.825.71:0.01021)0.937.108:0.03417,Ul
trSo91:0.06375)0.882.126:0.03018)0.967.81:0.04626,(Ult10083:0.02844,Ult10
084:0.01177)0.972.89:0.05047)0.778.91:0.01089,(UltPl208:0.01807,(Ult10071
:0.06183,Ult10072:5.5E-
4)0.702.20:0.00964)0.849.98:0.01427)0.924.108:0.01548)0.884.120:0.02986,(
PnsSpec2:0.00993,PnsSpec3:0.01298)1.000.783:0.09457)0.936.108:0.03066)0.9
01.117:0.01825)0.843.87:0.01092)0.900.97:0.01245)0.933.121:0.01556)0.812.
66:0.02432)0.956.102:0.04782,(((UltPl119:0.02144,UltB9908:0.15852)0.834.7
1:0.01763,(((((UltOr105:0.0192,(((PtcYyyyy:0.08158,((((((UltB9927:0.00743
,(UltB9939:0.00738,((UltB9923:0.00735,(UltB9922:0.01944,((UltB9919:0.0036
6,(UltB9921:0.00369,UltB9920:5.5E-
4)0.803.66:0.00374)0.905.116:0.01141,(UltB9918:0.01495,UltPl178:5.4E-
4)0.818.50:0.0076)0.874.135:0.01117)0.988.93:0.03227)0.933.122:0.01905,(U
ltB9874:0.00873,UltB9937:0.03555)0.649.13:0.01951)0.267.12:0.00406)0.880.
125:5.4E-4)0.877.127:0.0037,UltB9940:0.00369)0.694.19:5.5E-
4,(UltPl183:0.0,UltB9933:0.0,UltB9938:0.0):5.5E-4)0.819.72:5.4E-
4,((((UltB9924:0.02311,(UltB9930:0.01933,UltPl179:0.01513)0.181.6:0.00368
)0.935.105:5.4E-
4,UltB9931:0.00726)0.331.11:0.00374,UltPl180:0.00728)0.758.85:0.00393,(((
(UltB9800:0.01579,UltB9832:0.02374)0.897.115:0.01131,(UltB9929:5.4E-
4,((UltPl181:0.0037,(UltB9925:0.00753,(UltB9941:5.5E-
4,UltPl182:0.0076)0.910.111:5.4E-4)0.173.9:0.00375)1.000.784:5.4E-
4,(UltB9932:0.00374,UltB9928:0.00374)0.434.9:5.5E-
4)0.927.110:0.00372)0.888.120:5.4E-
4)0.888.121:0.00723,((((Ult10003:0.01185,((UltOr107:0.01138,(Ult10002:5.4
E-4,UltB9716:0.03138)0.769.90:0.00384)0.794.80:0.0033,Ult10001:5.3E-
4)0.918.119:0.01518)0.929.102:0.02027,(UltPl137:0.01012,UltB9823:0.03712)
0.214.10:0.00747)0.788.84:0.00615,(UltB9822:0.00384,UltB9834:0.04012)0.68
1.16:0.00764)0.946.95:0.00194,(UltB9833:0.00384,(UltB9967:0.04559,UltB999
9:0.03283)0.782.107:0.00669)0.928.90:0.00173)0.977.74:0.01852)0.947.86:5.
5E-
4,UltB9926:0.00757)0.894.113:0.00739)0.777.105:0.00363)0.764.96:0.00417,(
(UltB9964:0.01579,(UltB9965:0.02671,UltB9966:0.02371)0.867.110:0.01234)0.
374.17:0.00788,UltB9917:0.06162)0.745.74:0.00895)0.895.123:0.01108,(((Ult
Pl173:0.01312,(UltB9889:0.02299,UltB9890:0.0074)0.872.113:0.00764)0.775.8
5:0.00565,((UltB9872:0.01243,UltPl124:0.04885)0.842.100:0.01748,(((((UltP
l171:0.0115,((UltOr106:0.0,UltrSo86:0.0):0.00335,(UltFor11:5.4E-
4,(UltB9873:0.02769,UltB9887:0.0115)0.869.90:0.01111)0.733.41:0.00764)0.8
88.122:0.00805)0.827.72:0.00727,UltB9892:0.00732)0.577.9:5.5E-
4,UltB9891:0.01143)0.650.15:0.00358,UltB9893:0.00898)0.788.85:0.00621,(Ul
tPl188:0.01545,(UltB9968:0.00785,UltB9969:0.02267)0.870.100:0.01277)0.785
.92:0.0067)0.723.33:0.00596)0.825.72:0.0101)0.459.14:0.00421,((((UltB9884
:0.02968,UltB9885:0.02095)0.819.73:0.01166,(UltPl172:0.08618,(UltB9881:0.

02052,(UltB9883:0.01955,UltB9882:0.02385)0.863.127:0.02004)0.493.16:0.018
68)0.981.82:0.04348)0.931.117:0.01789,((UltB9683:0.031,UltrSo85:0.04498)0
.123.9:0.0081,(((UltB9880:0.05049,(UltB9878:5.5E-4,UltB9879:5.5E-
4)0.990.89:0.04409)0.897.116:0.01967,(UltB9953:0.00997,(UltB9954:5.4E-
4,UltB9955:0.0151)0.879.122:0.00891)0.962.105:0.02033)0.583.11:5.4E-
4,((UltB9876:0.00772,(UltSlu24:5.5E-
4,UltB9877:0.00745)0.744.61:0.00351)0.744.62:0.00584,(UltrSo84:0.02306,Ul
tB9875:0.0077)0.887.120:0.01325)0.973.79:0.03067)0.486.14:0.02211)0.902.1
20:0.01443)0.949.105:0.02528,(UltB9886:0.05293,UltB9871:0.00403)0.154.7:0
.00378)0.832.81:0.0069)0.857.113:0.00848)0.748.69:0.00344)0.839.92:0.0077
6,UltSlu25:0.03185)0.827.73:5.5E-
4,(UltB9952:0.02266,((UltBlas2:0.02541,((((((UltB9947:0.01861,(UltB9945:0
.00368,UltB9946:5.5E-4)0.953.83:5.5E-
4)0.469.15:0.01313,((UltB9950:0.00935,UltB9951:0.00564)0.889.107:0.00757,
((UltB9948:5.5E-
4,UltB9949:0.02245)0.798.76:0.00548,UltPl186:0.01328)0.755.71:0.0036)0.83
6.79:0.00715)0.889.108:0.00961,(UltB9943:0.0,UltB9944:0.0):5.5E-
4)0.493.17:0.00363,((UltOr104:0.00369,UltB9942:0.00374)0.907.118:0.00751,
MaiMet13:5.4E-4)0.745.75:5.5E-
4)0.991.91:0.02637,UltPl187:0.00772)0.477.14:0.00756,MaiMet14:0.00369)0.9
17.104:0.0182)0.919.144:0.02067,((UltB9798:0.01923,(UltB9797:0.01121,UltP
l118:0.00377)0.184.8:5.5E-
4)0.418.13:0.02348,(UltB9826:0.00674,UltB9827:0.00488)0.994.97:0.04806)0.
823.71:0.0129)0.803.67:0.00945)0.755.72:0.0044)0.871.113:0.01351)0.207.6:
5.3E-
4,(UltPl169:0.01149,UltPl170:0.00725)0.951.101:0.01516)0.920.128:0.01468,
((((((UltB9819:0.01471,UltPl128:0.00425)0.952.97:0.0166,((UltPl131:0.003
95,UltPl130:0.03114)0.989.77:0.03883,UltB9806:0.03171)0.724.29:0.00401)0.
917.105:0.01379,(UltB9836:0.02289,UltB9961:0.0247)0.743.70:0.00885)0.806.
57:5.5E-
4,(UltB9808:0.03096,((((Ult10012:0.01714,Ult10014:0.06798)0.210.16:0.0154
7,(Ult10013:0.0,Ult10011:0.0):0.01868)0.342.14:0.00851,UdnPlanc:5.4E-
4)0.996.103:0.0437,((((UltPl184:0.01961,UltPl185:0.00753)0.987.110:0.0313
2,UltPl120:5.3E-
4)0.686.15:0.00369,UltB9801:0.0114)0.955.87:0.02436,(UltPl122:0.01512,(Ul
tB9802:0.00375,UltPl121:0.01926)0.388.12:5.4E-
4)0.923.151:0.01982)0.888.123:0.01298)0.742.53:0.0038)0.755.73:0.00537)0.
912.141:0.0117,((((UltB9818:0.01154,(UltB9817:0.0041,(UltPl100:0.00776,(U
ltB9811:0.0217,UltB9812:0.00366)0.962.106:0.02178)0.542.12:0.00876)0.870.
101:0.01252)0.846.117:0.00888,(((UltPl129:0.03973,UltPl132:0.03854)0.644.
14:0.00358,UltB9820:0.07784)0.613.17:0.00706,UltB9868:0.02926)0.864.110:0
.00962)0.746.60:0.00442,UltB9807:0.02727)0.978.68:5.4E-
4,(UltPla99:0.0038,UltB9813:0.01139)0.961.91:0.01968)0.858.105:0.0083)0.9
26.129:5.5E-
4,(UltB9805:0.01926,(((UltB9960:0.02613,(UltB9958:0.0,UltB9959:0.0):0.067
91)0.924.109:0.02167,(((UltPl193:0.04607,UltPl192:0.03273)0.826.67:0.0123
5,((UltOr102:0.00349,UltOr103:0.01569)0.873.108:0.01773,((((UltB9864:0.03
59,UltB9865:0.04217)0.879.123:0.02641,((UltB9861:0.00721,(((UltB9860:5.4E
-
4,UltB9863:0.03499)0.798.77:0.00374,((UltB9859:0.05118,(UltB9857:0.00369,
UltB9858:5.5E-4)0.896.107:5.4E-
4)0.699.25:0.01784,UltRu267:0.11658)0.967.82:0.04018)0.124.12:5.4E-
4,(UltB9847:0.00738,((UltB9848:0.0,UltB9852:0.0,UltB9851:0.0,UltB9853:0.0
):5.5E-4,((UltB9849:5.5E-

4,UltB9854:0.00369)0.834.72:0.00369,UltB9850:0.00369)0.877.128:5.5E-
4)0.972.90:5.5E-
4)0.964.95:0.02262)0.413.17:0.00751)0.898.126:0.02009,UltB9862:0.02822)0.
991.92:0.0534)0.968.82:0.03386,((Otu00918:0.01743,(UltB9856:0.03629,Otu00
491:0.02935)0.924.110:0.02286)0.946.96:0.03264,(UltB9866:0.05419,UltB9867
:0.14867)0.939.103:0.05102)0.186.6:0.01232)0.698.26:0.00507,((PahgYy40:0.
01134,(Bfhggg41:0.02272,(UltPl167:0.00404,(UltPl165:0.04716,((PahggY42:5.
5E-4,S0114252:5.5E-
4)0.980.67:0.02364,UltPl166:0.01461)0.408.10:0.00382)0.776.92:0.0039)0.88
8.124:0.01125)0.909.95:0.01182)0.876.101:0.01498,(UltPl168:0.0321,((UltPl
164:0.0,Otu01326:0.0):0.03344,((Otu00451:0.01708,(UltPl158:5.3E-
4,(((Kty00023:0.01135,Otu00130:0.0037)0.779.85:0.00372,((UncUn117:0.0037,
((((((UltPl151:0.00739,UltPl152:5.5E-
4)0.867.111:0.00368,((UltPl159:0.0,Otu00255:0.0):5.4E-4,((UltPl160:5.5E-
4,Otu00917:0.02751)0.585.8:0.00792,(UncUn120:0.00377,(UncUn118:0.00744,Un
cUn119:5.5E-
4)0.958.106:0.01522)0.965.81:0.02321)0.429.14:0.01128)0.818.51:0.00351)0.
894.114:5.4E-4,UltPl154:0.01138)0.824.67:0.00739,((Try00028:5.5E-
4,(Tryy0094:0.02752,UltPl150:0.01907)0.962.107:5.5E-4)0.938.67:5.4E-
4,UltPl153:0.01135)0.860.80:0.00374)0.988.94:5.4E-
4,Tryy0095:0.00741)0.849.99:0.00374,((((UltPl145:0.01133,((UltPl144:0.007
76,UltPl143:0.00718)0.554.14:0.00374,(OphioYyy:0.01073,UltPl146:0.02322)0
.974.58:5.5E-4)0.723.34:5.4E-
4)0.935.106:0.00757,(UltPl147:0.02759,UltB9846:0.02308)0.646.13:5.4E-
4)0.780.107:0.00381,UltPl148:0.0114)0.868.94:0.00748,UltPl149:0.01152)0.9
56.103:0.0193)0.007.8:5.4E-4)0.845.104:0.00373,UltPl155:5.5E-
4)0.926.130:0.00751)0.805.65:5.5E-
4,(UltPl156:0.0075,UltPl157:0.00751)0.918.120:0.01134)0.971.70:0.01147)0.
867.112:0.01121)0.883.122:0.01094,(UltPl161:0.05359,(UltPl162:0.02108,Ult
Pl163:0.00596)0.927.111:0.01759)0.641.12:0.0095)0.906.125:0.01958)0.911.1
35:0.01635)0.852.100:0.02133)0.159.6:0.00803)0.925.122:0.03048)0.848.94:0
.01755)0.941.95:0.03151,(UltPl127:0.04559,(Ult10009:0.03032,Ult10010:0.00
515)0.986.82:0.03909)0.925.123:0.02705)0.845.105:0.01045)0.970.84:5.4E-
4,(((((UltB9971:0.0152,(UltB9970:0.00515,UltrSo87:0.02633)0.085.5:0.00691
)0.979.85:0.02843,(UltB9814:0.03333,(UltPl189:0.01062,(UltrSo89:0.00795,(
UltB9972:5.4E-
4,UltrSo88:0.01529)0.962.108:0.01522)0.082.11:0.00531)0.992.91:0.0391)0.8
47.127:0.01456)0.871.114:0.01217,((Ult10006:0.0,Ult10007:0.0):0.01743,Ult
10008:0.02723)0.967.83:0.02691)0.735.41:5.5E-
4,((UltPl177:0.01667,UltB9915:0.05346)0.692.13:0.01158,UltB9830:0.01745)0
.901.118:0.01348)1.000.785:5.5E-
4,((((UltB9835:0.04237,UltrSo83:0.04073)0.890.114:0.02373,(UltB9914:0.026
45,UltPl175:0.01287)0.780.108:0.00527)0.690.21:0.00789,UltPl176:0.01909)0
.801.77:0.00777,((((((UltB9991:0.00376,(UltB9993:0.02718,UltB9821:0.0399
2)0.845.106:5.5E-
4)0.820.60:0.00377,(((UltPl135:0.0,UltPl136:0.0):0.00932,(UltB9829:0.0134
2,UltB9828:0.02604)0.399.15:0.00735)0.820.61:0.0057,UdnBac28:0.03565)0.87
1.115:5.4E-4)1.000.786:5.3E-
4,((UltrSo90:0.02276,(UltB9985:0.02702,UltB9994:0.01195)0.855.101:0.01125
)0.496.8:5.4E-
4,UltB9992:0.03203)0.369.10:0.00737)0.903.103:0.00338,(UltB9979:0.02306,(
((UltB9987:0.00727,(UltB9982:0.00381,UltB9983:0.01135)0.928.91:5.5E-
4)0.788.86:0.00607,(UltB9986:0.02343,(UltB9984:0.01582,UltB9990:0.01142)0
.834.73:0.00753)0.823.72:0.0064)0.787.77:0.00604,(UltB9980:0.03588,((UltB

9981:0.02299,UltB9989:0.01471)0.578.7:5.5E-
4,UltB9988:0.00768)0.887.121:5.4E-
4)0.771.109:0.00363)0.971.71:0.01892)0.825.73:5.4E-
4)0.854.99:0.00726,(((UltB9975:5.5E-
4,(UltB9973:0.01926,UltB9974:0.01123)0.979.86:0.02326)0.983.69:0.02522,((
UltB9962:0.05183,(UltB9997:0.01585,UltB9998:0.03551)0.972.91:0.03382)0.66
0.16:0.02173,UltB9934:0.08555)0.751.56:0.00522)0.673.12:0.01301,(UltB9963
:0.04794,UltB9978:0.02321)0.799.91:5.4E-
4)0.803.68:0.00698)0.758.86:0.00406,UltB9810:0.01536)0.877.129:0.00794,((
(UltB9824:0.00369,UltB9825:5.5E-
4)0.999.158:0.08199,Ult10000:0.0275)0.846.118:0.02846,(UltB9816:0.0315,Ul
tB9831:0.03518)0.939.104:0.02601)0.784.97:0.01068)0.617.11:5.4E-
4)0.381.18:5.5E-
4)0.990.90:0.01441)0.770.101:0.00356)0.786.89:0.00359)0.993.80:0.02084,((
UltPl126:0.0216,(UltPl125:0.03004,(UltB9809:0.02574,UltOr101:0.01225)0.84
4.114:0.0115)0.845.107:0.00831)0.749.87:0.00499,(UltPl133:0.02248,(UltPl1
34:0.03368,(UltB9956:0.00543,UltB9957:0.01786)0.944.88:0.02646)0.646.14:0
.00581)0.762.95:0.00951)0.787.78:0.00784)0.732.32:0.00428)0.814.60:0.0094
,(Ult10005:0.09035,(UltB9916:0.10156,UltB9815:0.02746)0.612.17:0.01514)0.
908.128:0.02392)0.759.95:0.00551,((((((UltB9688:0.02562,(((UltB9708:0.011
84,UltB9709:0.00754)0.965.82:0.03168,(UltB9706:5.5E-
4,(UltB9705:0.06545,(UltB9707:0.02618,(UltPla98:0.00398,UltPla97:0.01958)
0.694.20:0.00128)0.418.14:0.00722)0.514.12:0.00652)0.906.126:0.01827)0.86
0.81:0.01572,(UltB9907:5.5E-
4,UltB9906:0.01122)0.997.104:0.05174)0.602.10:0.01061)0.163.10:0.01531,((
(UltB9571:0.0179,(UltB9569:0.00378,UltB9570:0.0114)0.966.106:0.0326)0.969
.73:0.03554,((((((UltB9900:0.00368,(UltB9894:5.5E-
4,((UltB9898:0.00741,UltB9897:0.02282)0.984.74:5.3E-4,UltB9896:5.5E-
4)0.455.22:5.5E-4)0.000.840:5.5E-4)0.459.15:5.5E-
4,UltB9899:0.0074)0.886.138:5.3E-
4,UltB9895:0.00752)0.135.9:0.00376,(UltB9901:0.0037,UltB9902:0.01537)0.76
9.91:0.00399)0.957.95:0.02571,(((UltB9904:0.01753,UltB9905:0.03292)0.946.
97:0.02693,BacEnr36:0.01978)0.685.16:0.01111,UltB9903:5.4E-
4)0.976.71:0.02693)0.967.84:0.02866,((UltPla19:0.0281,((((((UltB9553:0.0
1714,AgltSoi2:0.06728)0.657.17:0.01251,(((UltB9554:0.01536,(((((UltB9566:
0.00729,UltB9567:0.0039)1.000.787:0.0616,UltB9542:5.4E-
4)0.752.74:0.00914,(UltPlan8:0.01481,((((((UltB9551:0.00854,(UltB9546:0.0
148,(UltB9548:0.00367,(UltB9549:0.0,UltB9550:0.0):0.00375)0.830.79:0.0102
5)0.690.22:0.01263)0.689.18:5.4E-
4,(UltPlan6:0.01278,UltPla10:0.02579)0.882.127:0.01154)0.926.131:0.00987,
UltPlan7:5.4E-
4)0.899.108:0.01108,(UltPlan5:0.01386,(UltB9544:0.00535,UltB9545:0.00589)
0.177.14:0.00731)0.851.105:0.0095)0.780.109:0.00764,UltPlan4:0.02647)0.75
5.74:0.00439,(UltB9543:0.00755,(UltB9541:0.0151,UltPlan3:0.00739)0.867.11
3:0.00765)0.874.136:0.00739)0.063.6:5.5E-
4)0.856.96:0.02142)0.882.128:0.01165,UltB9547:0.00966)0.951.102:0.02113,U
ltPla12:5.4E-
4)0.918.121:0.01141)0.881.125:0.01102,(UltB9552:0.00739,UltPla11:0.00369)
0.915.113:5.4E-
4)0.730.43:0.00295,UltB9558:0.02394)0.933.123:0.02439)0.660.17:0.01393,(U
ltB9556:0.00407,(UltB9555:0.00324,UltPla13:0.00421)0.338.5:0.00775)0.985.
85:0.03148)0.480.14:5.4E-4,(UltB9563:5.5E-
4,((UltB9560:0.00746,(UltB9561:0.0,UltB9562:0.0):5.5E-
4)0.874.137:0.01118,((UltB9557:0.0,UltB9564:0.0):0.00333,(UltPla14:0.0036

9,UltPla15:5.5E-4)1.000.788:5.4E-
4)0.797.72:0.01157)0.898.127:0.00763)0.965.83:0.01529)0.928.92:0.01997,Pi
rStale:0.01421)0.880.126:0.0197,UltB9559:0.01168)0.403.13:0.0032,(UltAc71
0:0.08383,(UltB9775:0.02953,UltPl559:0.08975)0.978.69:0.06339)0.827.74:0.
03721)0.220.9:0.02734)0.942.96:0.02846,(UltPirel:0.02696,UltB9568:0.02397
)0.829.85:0.01863)0.323.9:0.00445)0.880.127:0.01604,(UltB9575:0.00968,Ult
Pla18:0.01753)0.976.72:0.0386)0.389.12:0.00913)0.454.19:0.01398,((UltPla8
5:0.0208,(((UltB9702:0.05588,(UltHydr6:0.00369,(UltB9692:5.5E-
4,(UltPla86:0.0037,UltB9936:0.07376)0.786.90:0.00369)0.829.86:5.4E-
4)0.823.73:0.00973)0.958.107:0.02398,(UltB9691:0.00807,(UltB9689:5.5E-
4,UltB9690:0.01522)0.775.86:0.00811)0.969.74:0.0243)0.872.114:0.01159,Ult
Pla87:0.01411)0.959.102:0.0281)0.859.102:0.01803,(UltB9659:0.04364,(UltB9
657:0.01544,UltB9658:0.01584)0.863.128:0.01814)0.947.87:0.03084)0.406.13:
0.01068)0.749.88:0.01425)0.053.5:0.01265,(UltB9717:0.00862,(UltB9714:0.00
464,UltB9713:0.01064)0.978.70:0.02368)0.886.139:0.01414)0.982.67:5.5E-
4,((UltPl101:0.04631,UltB9718:0.02806)0.237.10:0.01192,(UltB9711:0.01163,
(UltB9712:0.02714,(UltB9684:0.00613,UltB9710:0.02088)0.712.22:0.01185)0.8
51.106:0.01132)0.725.34:0.00808)0.863.129:5.4E-4)0.003.6:5.3E-
4,((((UltB9703:0.01531,UltB9704:5.5E-
4)0.971.72:0.02677,UltB9715:0.00949)0.802.71:0.01413,(UltB9694:0.03575,Ul
tPla91:0.03154)0.890.115:0.01138)0.942.97:5.3E-
4,((((((UltPla82:0.0198,UltB9701:0.01345)0.765.77:8.4E-
4,(UltB9678:0.0133,UltPla81:0.05358)0.869.91:0.01128)0.855.102:0.00892,(U
ltPla42:0.03888,(UltPla37:0.00831,(UltPara2:0.0639,UltPire4:5.4E-
4)1.000.789:0.05257)0.745.76:0.01056)0.975.80:0.03942)0.000.841:0.00549,(
(Ult10099:0.02741,(UltB9585:0.01689,UltB9586:0.01485)0.946.98:0.02548)0.9
53.84:0.02603,((UltB9623:0.00495,(UltPla36:0.02382,UltB9622:5.4E-
4)0.788.87:0.00596)0.996.104:0.04354,((UltB9618:0.03804,((UltB9615:0.0074
6,(UltB9617:0.01132,UltPla34:0.01927)0.862.104:0.00774)0.738.46:0.00449,U
ltB9616:0.01917)0.923.152:0.02218)0.862.105:0.0177,((UltB9620:0.05002,Ult
B9621:0.0476)0.738.47:0.00374,(UltB9619:5.5E-
4,UltPla35:0.00369)0.966.107:0.01691)0.752.75:0.00634)0.969.75:0.02674)0.
816.69:0.0096)0.898.128:0.01355)0.870.102:0.0135,(((UltPla79:0.02103,UltP
la80:0.06806)0.985.86:0.05658,(UltB9693:0.0041,UltPla89:0.03112)0.883.123
:0.01445)0.549.17:0.01336,((((((((PirSpeci:0.05621,(UltPla29:0.00406,Ult
B9682:0.02776)0.925.124:0.02695)0.827.75:0.01706,(((UltPla27:0.01265,((Ul
tB9603:5.4E-
4,((UltOrg99:0.00836,UltOrg98:0.01056)0.372.14:0.00394,(PnmctStr:0.004,Ul
tB9604:0.0149)0.566.12:0.00448)0.926.132:0.01434)0.851.107:0.00982,UltPir
e3:0.00533)0.965.84:0.02445)0.854.100:0.01058,((UltPla26:0.01875,BacEnr34
:0.00369)0.696.18:5.5E-4,(BacEnr32:5.5E-
4,(UltB9602:0.01493,BacEnr33:0.00369)0.606.15:5.4E-
4)0.892.109:0.00742)0.982.68:0.03222)0.823.74:0.00839,UltPla31:0.01929)0.
730.44:0.00465)0.895.124:0.01549,(UltPla28:0.01211,(UltB9605:0.02277,UltP
la30:5.4E-
4)0.405.18:0.00688)0.857.114:0.01479)0.876.102:0.0185,((UltB9597:0.0,UltB
9598:0.0):0.00746,UltPla24:0.00376)0.985.87:0.03383)0.694.21:0.00248,(Ult
B9599:0.01476,(UltPla25:0.00371,UltB9600:0.00371)0.999.159:5.5E-
4)0.990.91:0.04586)0.976.73:0.03445,UltB9596:0.00756)0.874.138:0.01175,(U
ltB9595:0.01881,((UltPla23:0.00739,(UltPla22:5.5E-4,(UltB9589:5.5E-
4,UltB9587:0.0112)0.924.111:0.00748)0.902.121:5.5E-
4)0.843.88:0.00743,(UltB9593:0.01117,((UltB9590:0.0,UltB9591:0.0):5.5E-
4,UltB9592:5.5E-4)0.896.108:5.5E-4)0.806.58:0.00364)1.000.790:5.3E-
4)0.755.75:0.00368)0.874.139:0.01264,(((((UltB9679:0.01744,(UltPire7:0.01

071,UltPla83:5.5E-
4)0.657.18:0.01271)0.975.81:0.0456,(((UltB9698:0.00363,UltB9699:5.5E-
4)0.981.83:0.03867,(((UltB9695:0.00369,UltPla92:0.02299)0.861.120:0.00768
,(UltB9697:0.04482,((UltPla93:0.02781,UltPla94:9.5E-
4)0.837.69:0.00168,(UltPla96:0.00761,(UltB9700:0.01148,UltPla95:0.00757)0
.752.76:0.00384)0.976.74:0.0247)0.859.103:0.01288)0.747.65:0.0037)0.820.6
2:0.00753,UltB9696:0.02386)0.966.108:0.03564)0.866.113:0.01647,((UltB9580
:0.01516,(UltB9582:0.00682,(UltB9584:0.0039,(UltB9579:5.4E-
4,(UltB9581:5.5E-
4,UltB9583:0.0037)0.922.157:0.00353)0.425.11:0.00371)0.891.95:0.00164)0.9
02.122:0.00238)0.787.79:0.00685,(UltB9576:0.07648,(UltB9578:0.0076,(UltPl
a21:0.0189,UltB9577:0.00801)0.868.95:0.00795)0.686.16:8.7E-
4)0.788.88:0.00838)0.972.92:0.02572)0.566.13:0.01094)0.907.119:0.02485,Ul
tB9588:0.10877)0.711.29:0.00561,(UltB9565:0.03189,(UltB9613:5.4E-
4,((UltPla33:0.01153,UltB9614:0.0035)0.982.69:0.01944,(UltB9611:0.00369,U
ltB9612:0.00369)0.478.14:5.3E-
4)0.852.101:0.01508)0.993.81:0.0484)0.906.127:0.01714)0.831.74:0.01021,(U
ltB9608:0.02265,(UltB9609:0.0,UltB9606:0.0,UltB9607:0.0):5.3E-
4)0.892.110:0.02063)0.932.108:0.02568)0.733.42:0.00622,UltB9601:0.05181)0
.926.133:0.01868)0.852.102:0.0185)0.594.13:5.4E-
4,((((((UltPl114:0.01066,UltB9765:0.03954)0.805.66:5.5E-
4,(((UltB9772:0.0,UltB9773:0.0):5.5E-4,(UltB9769:5.5E-
4,UltB9771:0.00188)1.000.791:0.0018)0.867.114:0.00802,(UltB9774:0.02366,(
UltB9764:0.01155,(UltB9770:0.02332,UltPl113:0.00771)0.729.27:0.00355)0.83
3.90:0.00736)0.906.128:0.01156)0.759.96:0.00385)0.779.86:0.00399,(UltB976
3:0.0076,UltB9762:0.03595)0.481.16:0.00409)0.975.82:0.03319,UltB9783:0.05
013)0.898.129:0.02032,(((UltB9677:0.0216,((((UltPla76:0.0233,((RprSpec4:5
.4E-4,((PnmctSt3:5.4E-
4,((RprSpeci:0.0,RprSpec2:0.0):0.00369,RprSpec3:5.5E-
4)0.985.88:0.01503)0.820.63:0.00369,PirSpec5:0.01127)0.623.11:5.5E-
4)0.974.59:0.00182,((PirSpec4:0.0,RprBalti:0.0):5.5E-
4,((UltB9662:0.00369,UltB9663:5.5E-4)0.989.78:0.0192,PirSpec3:5.5E-
4)0.859.104:0.00369)0.974.60:0.002)0.970.85:0.02299)0.104.9:0.00756,((Ult
B9673:0.07592,(PnmctSt7:5.4E-4,((PirSpec8:5.5E-
4,(UltPla77:0.027,PnmctSt6:0.04034)0.134.11:0.01152)0.942.98:0.02701,(Pir
Spec7:5.4E-4,RprSpec6:0.00371)0.848.95:0.00368)0.899.109:5.4E-
4)0.748.70:0.00175)0.968.83:0.02486,((UltPla75:0.01103,(((UltB9669:0.0113
7,UltB9672:0.00758)0.780.110:0.00355,(UltB9671:5.3E-
4,UltB9670:0.02308)0.443.12:0.00371)0.966.109:5.4E-4,(((((UltB9668:5.5E-
4,(UltB9667:0.00744,PnmctSt4:0.0037)0.839.93:5.5E-4)0.000.842:5.5E-
4,((UltPla70:0.00361,(UltPla69:0.01123,(UltB9665:5.4E-
4,UltB9666:0.00361)0.868.96:0.00744)0.777.106:0.00375)1.000.792:5.4E-
4,UltPla71:5.5E-4)0.000.843:5.5E-4)0.876.103:5.5E-
4,PirSpec6:0.00368)0.891.96:5.3E-
4,UltPla72:0.01121)0.815.68:0.00366,(UltPla73:0.0189,RprSpec5:5.4E-
4)0.640.15:5.4E-4)0.989.79:0.0188)0.948.122:0.01113)0.761.76:5.5E-
4,(UltPla74:0.02702,PnmctSt5:0.01515)0.957.96:5.4E-
4)0.897.117:0.00749)0.663.18:5.4E-
4)0.932.109:0.02125,UltB9664:0.02511)0.977.75:0.03108,((UltB9594:0.0,UltB
9676:0.0):5.4E-4,(PirSpec9:0.02296,(UltB9674:0.0,UltB9675:0.0):5.4E-
4)0.431.18:0.00727)0.987.111:0.03762)0.879.124:0.0163)0.945.89:0.01519,Ul
tPla32:0.02817)0.666.20:5.3E-4,(((((UltPire6:5.5E-
4,((UltB9632:0.03118,UltPla44:5.4E-
4)0.980.68:0.0277,(UltB9631:0.01496,UltPire5:5.5E-

4)0.841.92:0.00757)0.927.112:0.01889)0.918.122:0.02085,(UltPla41:0.01324,
(UltPla39:0.01718,((((UltB9627:5.5E-
4,(UdnMarin:0.00741,(UltB9626:0.00374,UltPla38:0.00372)0.814.61:0.00373)0
.825.74:5.4E-4)0.916.125:5.4E-
4,UltB9625:0.00746)0.819.74:0.0037,(UltPla40:0.01928,(UltPla56:0.01499,(U
ltB9624:0.00377,(UltB9629:0.00387,UltB9628:0.03498)0.529.8:0.00762)0.906.
129:0.01119)0.124.13:5.4E-4)0.442.19:5.4E-
4)0.854.101:0.0037,UltB9630:5.5E-
4)0.776.93:0.00191)0.697.11:0.01568)0.957.97:0.0255)0.919.145:0.02084,(((
(UltB9636:0.0,UltPla55:0.0):0.00368,UltB9637:5.4E-
4)0.967.85:0.02153,((UltB9635:0.00991,UltPla54:0.02491)0.984.75:0.04034,(
BacEnr35:0.03208,((UltPla50:0.00362,UltPla51:0.01133)0.995.102:0.03965,(U
ltPla52:0.00225,UltPla53:0.06522)0.834.74:0.00953)0.823.75:0.0082)0.970.8
6:0.02586)0.599.11:0.00662)0.868.97:0.00992,(((UltPla47:0.01929,(UltPla46
:0.00717,((UltPla43:0.02685,UltB9633:0.00793)0.802.72:0.00842,UltPla45:0.
0073)0.765.78:0.01138)0.791.75:5.5E-
4)0.821.68:0.00692,UltB9634:0.00811)0.926.134:0.02075,(UltPla48:0.03328,(
UltPla49:0.0,UltMar48:0.0):0.00661)0.964.96:0.02611)0.889.109:0.01501)0.7
72.95:0.0066)0.933.124:0.0205,(PirSpe10:0.02413,UltPla78:0.01156)0.998.13
0:0.06707)0.446.11:0.0123,(((((UltPla64:0.04035,(UltB9652:5.5E-
4,UltB9686:0.00369)0.814.62:0.00806)0.932.110:0.01984,((UltB9655:0.02457,
UltB9654:0.0224)0.878.107:0.01188,(((UltB9653:0.01922,(UltB9650:0.03034,U
ltPla88:0.01831)0.922.158:0.01935)0.158.10:0.00783,UltPla65:5.4E-
4)0.937.109:0.01573,(UltPla66:5.5E-
4,((UltB9651:0.03588,(UltB9656:0.01009,UltB9660:0.0538)0.760.88:0.00485)0
.764.97:0.00777,UltPla67:0.01086)0.330.12:5.4E-
4)0.890.116:0.01107)0.926.135:0.01493)0.805.67:5.4E-
4)0.889.110:0.01189,(((UltB9647:0.00744,((UltB9646:0.0191,(UltB9644:0.018
66,(UltPla61:0.01124,UltB9645:0.00369)0.894.115:5.5E-
4)0.874.140:0.00769)0.362.13:0.0074,UltB9648:0.00346)1.000.793:5.5E-
4)0.782.108:0.00433,UltPla60:0.00679)0.935.107:0.01941,(UltPla63:0.03986,
(UltB9649:0.00369,UltPla62:0.0191)0.038.5:5.5E-
4)0.445.17:0.01416)0.671.11:0.00463)0.801.78:0.0065,UltPla90:0.00508)0.86
2.106:0.01266,(UltPla68:0.01697,(UltB9661:0.02239,UltB9685:0.03102)0.892.
111:0.02155)0.970.87:0.03347)0.909.96:0.01438)0.862.107:0.01041)0.887.122
:0.00855)0.750.58:0.00361,(((((UltPl174:0.00716,(UltB9912:5.5E-
4,UltB9913:0.0037)0.906.130:5.4E-
4)0.964.97:0.02891,(UltB9911:0.01814,(UltB9909:0.0037,UltB9910:0.00745)0.
763.103:0.00492)0.944.89:0.03248)0.975.83:0.0406,UltPla84:0.01883)0.824.6
8:0.01182,(UltB9610:0.05968,UltB9680:0.02247)0.947.88:0.02012)0.702.21:0.
00305,(UltB9995:5.5E-
4,UltB9996:0.00368)1.000.794:0.04086)0.752.77:0.0039)0.992.92:0.02368)0.9
07.120:0.00723)0.951.103:0.00706)0.921.117:0.01784,(((UltB9643:0.02312,((
UltB9639:0.02388,((UltPla57:0.01849,UltB9641:0.08445)0.988.95:0.0423,UltB
9640:0.04022)0.867.115:5.3E-4)0.842.101:0.01568,(UltB9638:5.5E-
4,UltOr100:0.00747)0.736.50:0.00317)0.997.105:0.05922)0.900.98:0.0181,(Ul
tB9888:0.05225,((UltPl117:0.05613,(UltB9803:0.0158,(UltB9804:5.4E-
4,UltPl123:0.00369)0.769.92:0.00745)0.994.98:0.06518)0.796.65:0.02885,Ult
10004:0.10333)0.763.104:0.01883)0.903.104:0.02587)0.292.6:5.4E-
4,(((((UltB9642:0.0308,((UltB9752:0.00369,UltPl110:5.5E-
4)0.749.89:0.00278,((UltB9754:0.01104,((UltB9753:0.01813,(UltB9781:0.0181
9,UltB9782:0.0288)0.823.76:0.0075)0.753.86:0.00492,((((UltB9722:0.0076,(U
ltB9746:0.01135,(UltB9723:5.4E-4,(UltPire8:0.0115,((UltB9720:5.4E-
4,((UltB9719:0.0,UltPl103:0.0):0.00371,UltB9721:0.00374)0.904.117:0.00752

)0.801.79:0.00365,UltPl102:5.4E-
4)0.938.68:0.01555)0.858.106:0.00758)0.946.99:0.01131)0.759.97:0.00358)0.
771.110:0.00374,((UltB9739:0.00371,UltB9740:0.00371)0.770.102:0.00365,(((
UltB9737:0.02311,UltB9744:5.4E-
4)0.824.69:0.00365,(((UltB9733:0.01493,((UltB9726:0.01106,(UltB9724:0.007
57,UltB9725:0.01126)0.411.15:0.00749)0.892.112:5.4E-
4,UltB9734:0.00369)0.925.125:5.4E-
4)0.820.64:0.01124,((UltB9728:0.00371,(UltB9738:0.00747,(UltB9745:5.5E-
4,(UltB9730:5.5E-4,UltSlu23:0.01116)0.832.82:0.00369)0.196.12:5.4E-
4)0.901.119:0.00743)0.814.63:0.00368,(((UltB9735:0.01479,UltB9727:0.00775
)0.408.11:0.00744,(UltB9732:0.00379,UltB9731:0.01506)0.838.95:0.00345)1.0
00.795:5.5E-4,(UltB9742:5.5E-
4,(UltB9736:0.0037,UltB9743:0.00369)0.687.21:5.5E-4)0.617.12:5.5E-
4)0.949.106:0.00742)1.000.796:5.3E-
4)0.820.65:0.00366,(UltB9741:0.00369,UltPl104:0.02708)0.822.63:5.1E-
4)0.716.28:5.3E-
4)0.795.83:0.00372,UltB9729:0.01889)0.770.103:0.00367)0.879.125:0.00755)0
.953.85:0.01512,(UltB9841:0.03148,((UltB9792:0.02719,UltPl139:0.01928)0.7
47.66:0.00375,((((UltPl140:0.01976,(UltB9795:0.03665,UltB9794:0.03055)0.8
67.116:0.01623)0.606.16:0.01659,(UltB9757:0.00379,(UltB9687:0.04913,UltB9
756:0.04401)0.848.96:0.0135)0.697.12:0.00284)0.857.115:0.01426,UltB9793:0
.03287)0.852.103:0.00979,((UltB9842:0.01533,(UltB9843:0.02683,(UltB9844:0
.05279,(UltB9840:5.5E-
4,UltPl141:0.00367)0.779.87:0.00356)0.411.16:0.00407)0.869.92:0.00797)0.4
41.17:0.00347,(UltB9796:0.018,((UltB9839:0.01662,UltPl138:0.01451)0.289.6
:0.01008,(UltB9838:0.01556,UltB9837:0.01153)0.848.97:0.01226)0.788.89:0.0
111)0.772.96:0.0065)0.787.80:0.00406)0.798.78:0.00372)0.943.103:5.5E-
4)0.939.105:0.01875)0.737.66:0.00371,((UltPl109:0.01489,UltB9845:0.0527)0
.795.84:0.00661,((UltB9789:0.01146,(((UltB9791:0.0196,(UltB9787:0.0113,(U
ltB9755:0.00369,UltPl111:5.5E-
4)0.880.128:0.00794)0.762.96:0.00352)0.769.93:0.00353,(UltPl112:0.00379,U
ltB9786:0.02739)0.494.10:0.00392)0.820.66:5.5E-
4,(UltB9785:0.01366,(UltPl116:0.01386,UltB9784:0.02919)0.840.101:0.01217)
0.618.7:0.0128)0.987.112:0.01805)0.290.11:5.4E-
4,(UltB9788:0.03142,UltB9790:0.00823)0.912.142:0.01524)0.927.113:0.01521)
0.891.97:0.01014)0.873.109:0.00784)0.861.121:0.00812)0.924.112:0.01786,((
UltB9780:0.00745,UltPl115:0.00745)0.793.84:0.00395,(((UltB9751:5.4E-
4,UltPl108:0.00746)0.843.89:0.00924,(UltPl105:0.00813,(UltB9747:0.01023,(
UltB9748:0.09133,(UltPl106:0.0,UltB9749:0.0,UltPl107:0.0):5.5E-
4)0.903.105:0.01008)0.836.80:0.00956)0.965.85:0.023)0.981.84:0.03551,(Ult
Pire9:0.02236,(((UltB9779:0.03094,UltB9778:5.3E-
4)0.644.15:0.00745,((PirSpe11:0.01092,UltB9777:0.00399)0.869.93:0.01505,U
ltB9776:0.00342)0.998.131:5.5E-
4)0.875.121:0.0096,(((UltB9758:0.0,UltB9759:0.0):0.0041,(UltB9760:0.00407
,UltB9761:0.01861)0.864.111:0.0075)0.827.76:0.00681,(UltB9766:0.00757,(Ul
tB9767:0.01515,UltB9768:0.02016)0.744.63:5.5E-4)0.889.111:5.4E-
4)0.874.141:0.01403)0.766.84:0.00522)0.865.112:0.00952)0.739.48:0.00394)0
.961.92:0.02298)0.858.107:0.00955)0.962.109:0.02464)0.102.8:0.00747,(UltB
9799:0.01624,(UltPla58:0.01854,UltPla59:5.5E-
4)1.000.797:0.06254)0.862.108:0.01555)0.793.85:0.00857,((BlpMarin:5.5E-
4,UltBlast:0.00369)0.943.104:0.0156,(PirSpec2:5.5E-
4,PnmctSt2:0.00744)0.964.98:0.0194)0.836.81:0.00948)0.684.13:0.00754,(((U
ltB9573:0.00427,UltB9574:0.03125)0.949.107:0.0177,(UltPire2:0.01059,UltPl
a17:0.0242)0.724.30:0.00202)0.948.123:0.03479,(UltB9572:0.01151,UltPla16:

0.01162)1.000.798:0.11241)0.772.97:0.02845)0.754.72:0.00434,(UltPla20:0.0
8968,(UltB9976:0.02731,((UltPl190:0.01847,UltPl191:0.00479)0.945.90:0.031
63,((UltAmino:0.03634,(UltRu268:0.0275,UltB9977:0.0159)0.958.108:0.04173)
0.998.132:0.082,(S0114393:0.03269,PahggY43:0.00227)0.471.13:0.00496)0.725
.35:0.01341)0.965.86:0.04117)0.935.108:0.03536)0.968.84:0.0464)0.939.106:
0.01628)0.816.70:0.01165)0.862.109:0.01546)0.943.105:0.02679)0.963.82:0.0
3678,Ult10197:0.03158)0.913.126:0.03519)0.651.19:0.02655,(Ult10543:0.0583
7,(((Ult10542:0.0,Ult10541:0.0):5.5E-
4,Ult10540:0.00359)0.961.93:0.04014,((Ult10536:0.03971,(Ult10535:0.04,(Ul
t10533:0.03505,Ult10534:5.5E-
4)0.933.125:0.03509)0.957.98:0.0383)0.975.84:0.05545,(((((UltPl338:0.0308
1,UltPl339:0.03577)0.950.95:0.03156,(UltPl335:0.04723,(Ult10537:0.05257,U
ltPl336:0.00899)0.934.93:0.02554)0.683.28:0.00874)0.662.16:0.01063,(Ult10
538:0.00375,UltPl337:0.0037)0.965.87:0.02017)0.715.22:5.5E-
4,Ult10545:0.02622)0.481.17:0.00922,(Ult10539:0.01663,(Ult10544:0.0096,(U
ltPl340:0.0,UltPl341:0.0):0.01359)0.997.106:0.07046)0.966.110:0.03535)0.6
69.19:0.01408)0.908.129:0.02954)0.936.109:0.04554)0.889.112:0.03427)0.909
.97:0.03774,((Ult10477:0.04612,(Ult10476:0.0301,(Ult10475:0.04009,(((Ult1
0470:5.5E-4,Ult10471:5.5E-
4)0.911.136:0.01383,(((Ult10469:0.01536,(Ult10468:0.00368,(Ult10472:0.0,U
lt10473:0.0):0.01128)0.744.64:0.00354)0.965.88:0.01544,Ult10465:0.00755)0
.708.29:0.00364,(Ult10466:0.0,Ult10467:0.0):5.4E-
4)0.762.97:0.00115)0.168.12:0.00373,Ult10474:0.01818)0.862.110:0.01318)0.
941.96:0.03816)0.867.117:0.02772)0.946.100:0.045,(((UltPl293:0.0038,(Ult1
0328:0.01938,UltPl292:0.05558)0.909.98:0.01554)0.911.137:0.01167,UltPl294
:0.00345)0.979.87:0.04194,(((((Ult10323:0.02589,Ult10325:0.02292)0.644.16
:0.01257,(((Ult10319:0.0,Ult10320:0.0):5.5E-
4,(Ult10322:0.02701,Ult10343:0.09369)0.089.4:5.5E-
4)0.765.79:0.00557,Ult10321:0.02267)0.786.91:0.00764)0.929.103:0.01606,Ul
t10324:5.4E-
4)1.000.799:0.06553,((((Ult10299:0.00377,Ult10295:0.04378)0.634.13:0.0077
6,Ult10298:0.01154)0.879.126:5.4E-
4,(Ult10296:0.0,Ult10297:0.0):0.01073)0.932.111:0.01114,(((Ult10333:0.021
26,(Ult10327:0.08162,(Ult10335:0.0493,Ult10326:0.00729)0.195.8:0.00564)0.
854.102:5.3E-
4)0.187.10:0.01188,Ult10336:0.04765)0.919.146:0.01787,((Ult10358:0.023,(U
lt10303:0.0298,(Ult10293:0.00369,Ult10294:5.5E-
4)0.978.71:0.03885)0.955.88:0.02866)0.085.6:5.3E-
4,(UltPl297:0.00744,((Ult10332:0.0,UltPl295:0.0):0.00744,UltPl296:0.00371
)0.037.3:5.5E-4)0.924.113:0.00746)0.987.113:5.4E-
4)0.902.123:0.00749)0.913.127:5.5E-
4)0.714.27:0.0083,(Ult10337:0.02041,Ult10338:0.04004)0.441.18:0.00265)0.9
11.138:0.0239)0.873.110:0.02344)0.551.14:0.01671)0.810.71:0.02404,Ult1036
7:0.02222)0.820.67:0.01628)0.866.114:0.01731,((Ult10391:0.01888,Ult10392:
0.03644)0.917.106:0.02565,(Ult10390:0.04098,UltPl323:0.06937)0.910.112:0.
02441)0.852.104:0.01561)0.813.73:0.01262)0.909.99:0.0178,(((Ult10382:0.02
515,Ult10381:0.01056)0.756.89:0.00423,(UltPl304:0.04935,UdnBac29:0.00406)
0.932.112:0.02139)0.789.79:0.00834,Ult10383:0.01713)0.425.12:0.00499)0.86
0.82:5.3E-
4,UltrS101:0.0371)0.931.118:0.01937,((UltrS100:0.04115,(Ult10347:5.3E-
4,Ult10414:0.0233)0.960.99:0.03004)0.858.108:0.01322,(Ult10389:0.06951,(U
lt10409:0.04806,Ult10410:0.0359)0.840.102:0.02709)0.735.42:0.00348)0.731.
34:0.0041)0.973.80:0.03179)0.740.60:0.00701)0.765.80:0.01458)0.570.7:0.01
082,Ult31659:0.05309)0.822.64:5.4E-

4,((Ult10415:0.0,Ult10416:0.0):0.07371,(Ult10417:0.04589,UltPl325:0.07224
)0.760.89:0.04178)0.903.106:0.0293)0.747.67:0.00464)0.826.68:0.0145)0.348
.13:0.01655,(Ult10247:5.5E-
4,(Ult10244:0.01702,((Ult10245:0.00365,Ult10246:5.5E-
4)0.942.99:0.02101,(((Ult10236:0.00359,(((Ult10239:0.0,UltPl277:0.0):5.5E
-4,(Ult10237:5.5E-4,Ult10238:0.00734)0.830.80:0.0036)0.804.66:5.5E-
4,(Ult10241:5.5E-4,(Ult10235:5.5E-
4,(Ult10234:0.00736,Ult10233:0.01906)0.785.93:0.00357)0.984.76:0.01461)0.
909.100:0.0072)0.808.74:0.00367)0.551.15:0.00356,(Ult10240:5.5E-
4,Ult10242:0.00739)0.911.139:0.01141)0.930.98:0.01604,Ult10243:0.018)0.46
8.20:0.00919)0.738.48:0.00719)1.000.800:0.08029)0.990.92:0.07432)0.921.11
8:0.04584)0.955.89:0.06055)0.842.102:0.02883)0.272.13:0.02915,(UltDe114:0
.18342,(Ult25871:0.05568,((UltSulf8:0.0159,(SfbAcido:0.02911,UltSulfo:0.0
3501)0.530.8:0.01721)0.923.153:0.02342,(SfbBenef:5.4E-
4,((SfbSibir:0.00373,(((Ult25867:0.00386,Ult25870:0.05806)0.766.85:0.0036
5,Ult25866:5.4E-4)0.919.147:5.5E-
4,(Ult25868:0.00354,Ult25869:0.00782)0.972.93:0.01965)0.862.111:0.00725)0
.911.140:5.3E-
4,SfbTherm:0.00752)0.994.99:0.03187)0.942.100:0.02619)0.542.13:0.00238)0.
991.93:0.13077)0.976.75:0.12205)0.651.20:0.00493)0.733.43:0.00747)0.890.1
17:0.02874)0.762.98:0.0158)0.925.126:0.02242,((((((UltDefe2:0.1127,(UltG
a208:0.09331,Ult12953:0.09368)0.853.88:0.0529)0.605.15:0.04328,(((((((Ult
12941:0.0,Ult12942:0.0):0.0312,Ult12943:0.02427)0.974.61:0.03735,(UltOr20
5:5.4E-
4,UltCan76:0.04942)0.964.99:0.02863)0.177.15:0.01214,(((UltCald6:0.04359,
(Ult12950:0.0473,Ult12956:0.05052)0.867.118:0.01833)0.199.5:0.01222,((((U
lt12948:5.4E-4,(Ult12947:5.5E-
4,UltCald7:0.01125)0.996.105:0.03508)0.713.31:0.00955,(UltB4794:0.03269,(
Ult12949:0.01168,UltCan78:0.01129)0.876.104:0.01452)0.983.70:0.04279)0.93
0.99:0.02144,((UdnBac54:0.10695,(Ult12967:0.0361,Ult12966:0.01215)0.548.1
1:0.01751)0.978.72:0.03895,(Ult12965:0.01884,(((Ult12957:5.5E-
4,(UltCald8:0.00369,(UltCald9:5.5E-4,UltCal10:5.5E-4)0.751.57:5.5E-
4)0.833.91:0.00367)0.990.93:0.04105,UltCal12:0.03937)0.042.7:0.0104,((((U
lt12961:0.01548,(Ult12959:0.01519,Ult12964:0.01521)0.900.99:0.01123)0.765
.81:0.00471,(((Ult12954:0.02679,Ult12955:0.01327)0.956.104:0.02236,Ult129
62:0.00814)0.015.7:0.00561,Ult12960:0.03427)0.829.87:0.01048)0.238.12:5.3
E-
4,UltDefe3:0.0214)0.296.8:0.00759,(Ult12958:0.04129,UltCal11:0.03531)0.72
3.35:0.01206)0.823.77:5.4E-
4)0.821.69:0.01035)0.722.28:0.0044)0.987.114:0.04454)0.521.5:0.01057,(Ult
Cald3:0.03203,(UltCald4:0.03575,UltCald5:0.01051)0.733.44:0.004)0.881.126
:0.0082)0.680.20:5.4E-
4)0.868.98:0.02022,(Ult12968:0.04752,((((UltChl52:0.04076,(UltB9459:0.037
11,UltChl46:0.03064)0.725.36:0.00437)0.945.91:0.01608,(UltB9458:0.01053,(
(UltB9460:0.05147,(UltChl47:0.0243,UltB9448:0.01631)0.778.92:0.006)0.856.
97:0.00836,((UltChl53:0.036,(UltChl49:0.01129,((UltB9453:0.00751,UltB9454
:0.01141)0.987.115:0.0271,(((UltB9445:0.01185,UltChl51:0.03635)0.751.58:0
.00323,(((UltB9444:0.02241,(UltB9442:0.0037,UltB9443:0.00371)0.843.90:5.5
E-
4)0.032.5:0.00374,UltB9455:0.02773)0.758.87:0.00533,UltAl197:0.02289)0.80
5.68:0.00593)0.771.111:0.00364,UltB9446:0.00386)0.964.100:0.01541)0.882.1
29:5.4E-
4)0.773.99:0.00376)0.775.87:0.00372,((UltChl48:0.00766,(UltB9452:0.01741,
UltChl50:0.02702)0.943.106:0.02293)0.760.90:0.0036,UltB9449:0.00375)0.888

.125:0.0075)0.922.159:5.4E-
4)0.929.104:0.01232)0.747.68:0.00352)0.875.122:0.00784,(UltB9447:0.05459,
(UltB9456:5.5E-
4,UltB9457:0.00737)0.866.115:0.01555)0.875.123:0.01237)0.774.96:0.00569,(
UltB9450:5.5E-4,UltB9451:0.01504)0.810.72:8.4E-
4)0.997.107:0.09652)0.968.85:0.04904)0.885.107:0.02273)0.770.104:0.0117,(
((Ult12944:0.02149,(UltCan75:0.01432,Ult12946:0.0791)0.884.121:0.01803)0.
858.109:0.01105,(Ult12945:0.01871,UltDefer:0.05064)0.988.96:0.05079)0.787
.81:0.00914,(UltOr203:0.0,UltOr204:0.0):0.02744)0.642.13:0.01028)0.970.88
:0.04461,(((Ult12997:0.01092,(UltB4796:0.03706,(Ult12998:0.01895,(Ult1299
9:0.00728,Ult13002:0.18279)0.783.104:0.01288)0.819.75:0.0174)0.768.78:0.0
1328)0.629.19:0.0127,(Ult12996:0.01361,(StmClon5:0.01087,(Ult12994:5.5E-
4,Ult12995:0.01505)0.990.94:5.4E-
4)0.976.76:0.03524)0.892.113:0.0186)0.745.77:0.00613,(UltOr209:0.14499,Ul
t13000:0.01529)0.437.17:0.00995)0.980.69:0.04678)0.821.70:0.02392,(Ult132
28:0.11622,((UltCan93:0.00423,Ult13181:0.00308)0.999.160:0.16072,(Ult1318
0:0.04564,Ult13179:0.07622)0.924.114:0.05025)0.876.105:0.04041)0.958.109:
0.07646)0.489.13:0.025)0.719.25:0.01911,((UltOr193:0.06299,((Ult12988:0.0
4152,(UdnBac55:0.01467,(((Ult12987:0.03325,UltOr208:0.02906)0.863.130:0.0
1905,(((Ult12983:0.0,Ult12982:0.0):0.01568,(UltDefe6:0.00873,Ult12984:0.0
2225)0.688.21:0.00748)0.636.11:0.00479,Ult12985:0.01431)0.843.91:0.0135)0
.338.6:0.01513,Ult12986:0.01468)0.988.97:0.05409)0.964.101:0.03621)0.923.
154:0.02535,(CltSpeci:0.00361,((Ult12992:0.00771,((Ult12989:0.01933,Ult12
990:0.00346)0.982.70:0.04096,(((Ult12975:0.00765,(Ult12973:0.02217,Ult129
74:0.01939)0.831.75:0.00848)0.899.110:0.01215,((StmClon4:0.0,Ult12977:0.0
):5.5E-4,(Ult12969:0.07062,(((Ult12971:5.4E-
4,(Ult12978:0.01522,Ult12972:5.5E-
4)0.844.115:0.00368)0.807.63:0.00373,UltDe155:0.00369)0.762.99:0.00372,(U
lt12976:0.01863,((UltDefe5:0.00369,(Ult12970:0.00533,UltDefe4:0.0788)0.84
7.128:0.00979)0.782.109:0.0037,Ult12979:5.3E-
4)0.847.129:0.00367)0.513.9:5.3E-
4)1.000.801:0.00231)0.931.119:0.00135)0.904.118:0.01455)0.890.118:0.01746
,(UltOr207:0.03172,(((Ult12991:0.03722,UltDefe8:0.04057)0.894.116:0.01573
,(Ult12981:0.0305,Ult12980:0.03797)0.953.86:0.03279)0.568.13:0.00485,UltD
e156:0.00444)0.847.130:0.02229)0.884.122:0.01942)0.900.100:0.02527)0.662.
17:0.01357)0.773.100:0.00712,CltAbyss:0.00749)0.353.8:5.4E-
4)0.927.114:0.02026)0.394.17:0.01116)0.969.76:0.03639,(((((Ult12713:0.065
47,Ult12714:0.04744)0.935.109:0.02875,(((((Ult12709:0.022,(Ult12707:0.012
97,UltOr196:0.05645)0.488.15:0.00759)0.770.105:0.00413,Ult12712:0.06601)0
.165.6:0.0043,((((Ult12700:0.01009,(UltrS151:0.03036,Ult12710:0.03947)0.8
98.130:0.01577)0.740.61:0.00479,Ult12706:0.00789)0.857.116:0.00869,Ult127
05:0.0463)0.728.39:0.00979,((Ult12704:5.5E-
4,Ult12708:0.01121)0.877.130:0.00722,(Ult12702:0.00372,Ult12703:0.02272)0
.894.117:5.4E-
4)0.646.15:0.00457)0.908.130:0.01272)0.885.108:0.01769,((Ult12711:0.01134
,((Ult12696:0.01108,Ult12697:0.01571)0.991.94:0.03915,(UltOr197:0.00795,U
ltOr198:0.00766)0.994.100:0.04039)0.336.12:0.00762)0.925.127:0.01808,((Ul
t12699:0.00588,Ult12698:0.04215)0.742.54:0.01154,Ult12701:0.02466)0.776.9
4:0.00636)0.757.67:0.00358)0.889.113:0.01184,(UltOr194:0.023,UltOr195:0.0
1594)0.161.2:5.5E-
4)0.889.114:0.02635)0.930.100:0.0308,((Ult17011:0.10961,((Ult12718:0.0062
,Ult12719:0.02599)0.301.13:0.00389,(Ult12716:0.01294,Ult12717:0.03336)0.6
90.23:0.00786)0.979.88:0.05466)0.745.78:0.01117,Ult13024:0.17207)0.820.68
:0.01695)0.829.88:0.01277,((Ult12940:0.10493,(Ult12938:0.01967,(UltEub23:

0.01899,Ult12939:0.01544)0.746.61:0.00358)0.760.91:0.01141)0.970.89:0.055
98,(Ult12951:0.15449,Ult12952:0.09365)0.553.12:0.04112)0.895.125:0.03623)
0.541.6:5.4E-
4,((UltGem21:0.07199,((UltB9468:0.04517,(UltCaldi:0.02796,(UltB9478:0.042
9,((UltCald2:0.02505,((UltB9471:0.0074,(UltB9469:0.00739,UltB9470:0.01511
)0.872.115:0.00745)1.000.802:5.4E-
4,UltB9472:0.00354)0.744.65:0.00543)0.493.18:0.01206,(UltB9473:0.01626,(U
ltB9475:5.4E-
4,UltB9474:0.02634)0.044.6:0.02291)0.963.83:0.02495)0.703.29:0.00267)0.91
4.128:0.01495)0.872.116:0.01599)0.760.92:0.00605,((UltB9466:0.0348,(UltB9
467:0.01711,UltDe146:0.00934)0.556.11:0.00588)1.000.803:0.06227,(UltB9476
:0.00772,UltB9477:0.0147)0.908.131:0.017)0.839.94:0.00963)0.775.88:0.0114
)1.000.804:0.13412,((Ult12715:0.05628,Ult17359:0.12438)0.844.116:0.02084,
((UltFirm4:5.4E-4,(UltDefe7:5.4E-
4,UltDef14:0.0561)0.654.11:0.00381)0.998.133:0.10584,Ult12993:0.06946)0.9
21.119:0.04502)0.409.14:5.3E-
4)0.944.90:0.0622)0.867.119:0.01232)0.000.844:5.4E-
4)0.852.105:0.01226,((Ult12570:0.09281,Ult21905:0.05998)0.843.92:0.02178,
(((((UltMar75:0.02086,((Ult12884:0.01093,((Ult12888:0.01071,Ult12887:0.01
624)0.889.115:0.01124,((Ult12886:0.00366,(Ult12885:0.00368,Ult12889:0.003
68)0.797.73:0.00368)0.908.132:0.01169,((Ult12891:0.01505,Ult12890:0.02018
)0.989.80:0.05661,(Ult12892:0.01432,UltFib65:0.02829)0.995.103:0.06617)0.
652.11:0.03683)0.633.9:5.3E-
4)0.834.75:0.00716)0.896.109:0.0201,(Ult12893:0.02722,Ult12894:0.11285)0.
837.70:0.03478)0.789.80:0.01462)0.986.83:0.05688,(Ult12899:0.02282,(Ult12
896:0.03215,(Ult12895:0.01136,(Ult12897:0.00359,Ult12898:0.00757)0.751.59
:0.00372)0.809.78:0.00693)0.922.160:0.02674)0.843.93:0.01639)0.971.73:0.0
519,((Ult12937:0.02881,(Ult12936:0.01571,(UltMa101:5.5E-
4,(UltMa100:0.03811,UltMa102:0.05166)0.675.15:5.5E-
4)0.807.64:0.01122)0.994.101:0.07932)0.996.106:0.07151,Ult12934:0.13285)0
.667.22:0.01022)0.892.114:0.02781,(UltOr202:0.07967,(Ult12935:0.05419,((U
ltCan77:5.4E-
4,UltOr206:0.05985)0.989.81:0.06483,UltCan79:0.08669)0.939.107:0.04335)0.
734.59:0.03298)0.763.105:0.01861)0.741.58:0.00656,(((Ult17392:0.0,Ult1739
3:0.0):0.00786,((Ult17394:0.0,Ult17395:0.0):0.02723,UltAna11:0.00689)0.88
8.126:5.5E-
4)0.877.131:0.01904,((Ult17388:0.02785,(Ult17387:0.01085,(Ult17386:0.0142
3,(Ult17384:0.00805,Ult17385:0.00721)0.737.67:0.00502)0.917.107:0.01273)0
.869.94:0.01497)0.960.100:0.03189,(Ult17391:0.00614,(Ult17389:5.4E-
4,Ult17390:0.01104)0.999.161:0.07027)0.878.108:0.0184)0.221.10:0.01358)0.
999.162:0.08026)0.794.81:0.00944)0.118.12:0.01574)0.806.59:0.01618,(UltOr
g73:0.13564,(UltB5257:0.08031,(UltB5259:0.00453,(UltMet15:0.05268,UltB526
0:0.01138)0.581.11:0.0107)0.992.93:0.08805)0.819.76:0.02614)0.925.128:0.0
3614)0.146.10:0.0142,((UltCa130:0.01165,(Ult17203:5.4E-
4,Ult17204:0.00368)0.964.102:0.04065)0.999.163:0.11683,((UltB5258:0.14992
,(UltB5043:0.05264,UltB5044:0.05737)0.996.107:0.11109)0.860.83:0.03255,((
(Ult17468:0.07014,((Ult17466:0.09546,(Ult17469:0.00435,UltCa155:0.00303)0
.990.95:0.06608)0.748.71:0.00998,((Ult17467:0.11886,UltrS307:0.12799)0.87
1.116:0.0401,UltCa154:0.10809)0.893.126:0.03128)0.585.9:0.00191)0.920.129
:0.02776,((UltrS312:0.07002,(Ult17491:0.08622,(UltCa158:0.04933,(((UltOr2
88:0.03009,(UltrS311:0.00509,Ult17490:0.07193)0.857.117:0.01731)0.754.73:
0.00594,(UltOr287:0.03777,(Ult17489:0.06418,(Ult17487:0.00373,Ult17488:0.
03136)0.692.14:0.00578)0.766.86:0.01968)0.916.126:0.02232)0.295.18:0.0100
2,(Ult17486:0.05955,Ult17485:0.02449)0.684.14:0.00413)0.473.13:0.01285)0.

686.17:0.00952)0.363.15:0.00733)0.955.90:0.05163,((UltB4779:0.08097,(UltB
4777:5.5E-
4,UltB4778:0.00738)0.729.28:0.00764)0.997.108:0.09786,(((Ult17484:0.03274
,(Ult17483:0.02513,Ult17482:0.10657)0.875.124:0.0164)0.995.104:0.0672,(Ul
t17481:0.02981,(UltCa156:0.01695,UltCa157:0.02586)0.295.19:0.01218)0.899.
111:0.01748)0.537.13:5.3E-
4,((((Ult17476:0.00356,Ult17477:0.01134)0.930.101:0.01145,((Ult17471:0.00
382,(Ult17472:0.0074,(Ult17470:0.0,Ult17473:0.0):5.5E-
4)0.784.98:0.00356)0.970.90:0.01523,(Ult17474:0.00692,(Ult17475:0.02069,U
ltrS308:0.04019)0.175.5:0.01491)0.582.12:5.4E-
4)0.758.88:0.00379)0.775.89:0.00362,(UltrS309:0.01538,UltrS310:0.00779)0.
771.112:0.00355)0.175.6:0.00423,((Ult17479:0.00755,(Ult17478:0.00375,Ult1
7480:0.01893)0.909.101:0.01165)0.743.71:0.0061,Pshggg37:0.04609)0.564.10:
0.01364)0.868.99:0.01123)0.962.110:0.05831)0.687.22:0.02306)0.919.148:0.0
318)0.222.6:5.5E-
4,(Ult17456:0.08717,((((UltAc878:0.018,(Ult17459:0.01144,(UltOr285:0.0140
4,(Ult17457:5.4E-
4,Ult17458:0.01468)0.000.845:0.00901)0.980.70:0.03679)0.948.124:0.03087)0
.962.111:0.03423,((((UltrS306:0.03551,Ult17462:0.01908)0.906.131:5.4E-
4,Ult17461:0.01909)0.855.103:0.01045,(UltB4754:0.0206,UltrS305:0.04629)0.
474.16:0.0115)0.938.69:0.02069,(Ult17460:0.02382,(Ult17454:5.5E-
4,((Ult17453:0.0,Ult17451:0.0,Ult17452:0.0,Ult17455:0.0):5.4E-
4,Ult31825:0.01124)0.538.10:0.00364)1.000.805:0.11611)0.038.6:0.00963)0.9
00.101:0.02381)0.883.124:0.0185,Ult17463:0.02942)0.861.122:0.01483,UltCa1
53:0.02771)0.865.113:0.02799)0.996.108:0.07695)0.927.115:0.03209)0.810.73
:0.01182)0.805.69:0.02344)0.411.17:0.01487,((((((((Ult21899:0.04299,UltB2
482:0.06611)0.377.12:0.00921,(((UltSynt3:5.5E-
4,(UltB4510:0.02403,UltB4509:0.02384)0.946.101:0.01914)0.786.92:0.00458,(
SnhAroma:0.0162,((UltB4508:0.0,UltB4513:0.0):5.5E-4,UltB4512:5.5E-
4)0.840.103:0.00659)0.622.10:0.00755)0.698.27:0.00427,UltB4511:0.11081)0.
492.18:0.01312)0.916.127:0.02904,((Ult13192:0.06334,(Ult13190:0.06551,Ult
13191:0.0801)0.918.123:0.04744)0.999.164:0.13638,((Ult13015:0.02039,(Ult1
3010:0.05438,(((Ult13003:0.01878,(Ult13016:0.0037,UltAci43:0.00745)0.867.
120:5.4E-
4)0.925.129:0.0151,((Ult13004:0.01464,Ult13005:0.00424)0.914.129:0.01535,
(Ult13007:0.02737,Ult13006:0.03546)0.849.100:0.00842)0.854.103:0.01104)0.
906.132:0.01468,(Ult13008:0.02235,Ult13009:0.01243)0.875.125:0.01171)0.85
3.89:0.00908)0.818.52:0.01051)0.816.71:0.01203,(((Ult13011:0.00372,Ult130
12:5.5E-
4)0.915.114:0.01766,(UltGa296:0.03215,Ult13013:0.03235)0.896.110:0.0179)0
.995.105:0.04942,(Ult13014:0.05707,Ult13017:0.02217)0.660.18:0.02416)0.75
7.68:0.00742)0.998.134:0.11753)0.690.24:0.02621)0.648.17:0.01507,(((UltDe
166:0.0163,UltDe167:0.0181)1.000.806:0.11145,((((Ult32043:0.11649,(Ult320
42:0.01115,(Ult32040:0.00909,Ult32041:0.02223)0.665.19:0.00919)0.949.108:
0.06657)0.964.103:0.08599,((Ult32038:0.08026,((UltTer28:0.02354,(Ult32008
:0.05598,((Ult32011:0.04448,((((UltEnd36:0.01029,(PCyyy000:0.0185,(Otu007
43:0.01537,P0114412:0.00378)0.628.5:5.5E-
4)0.615.15:0.01132)0.749.90:5.5E-
4,(((((UncUn248:0.0,UncUn249:0.0):0.00295,Otu00123:0.01212)0.977.76:0.027
63,(UltEnd34:0.00847,(((UltEnd37:0.0,Otu00450:0.0):0.01367,Otu00068:0.017
53)0.971.74:0.02812,(UltEnd38:5.5E-
4,Otu00289:0.00742)0.840.104:0.01388)0.718.29:0.01241)0.842.103:0.01248)0
.890.119:0.01297,((Otu00090:0.00273,Otu00316:0.02395)0.949.109:0.01608,((
UltTer27:0.031,Sgfffff7:5.4E-

4)0.425.13:0.00503,(UltEnd42:0.02637,Otu00252:0.03318)0.957.99:0.02422)0.
898.131:0.01427)0.324.7:5.4E-
4)0.886.140:0.01091,(((UltTer19:0.01533,UltTer20:0.01142)0.909.102:0.0111
8,((UltEnd43:0.11275,(UltEnd41:0.01502,(UltTer26:0.01905,Otu00013:0.00712
)0.911.141:5.4E-
4)0.910.113:0.02385)0.755.76:0.01189,((UltTerm8:0.00369,(UltEnd31:0.00369
,((UltTerm7:0.05746,UltTerm6:0.00742)0.879.127:0.01535,((CDvEvE00:0.00743
,Hmyy0037:0.01489)0.859.105:5.4E-
4,(UltTerm4:0.0037,((Otu00011:0.00369,(UltEnd24:5.5E-
4,(UltTerm3:0.01116,(UltTerm2:0.00752,UltTermi:0.01135)0.958.110:0.01523)
0.846.119:5.5E-4)0.324.8:5.5E-4)0.882.130:5.4E-
4,UltTerm5:0.01902)0.830.81:0.0037)0.219.7:5.3E-
4)0.868.100:0.00363)1.000.807:5.5E-4)0.993.82:5.5E-
4)0.993.83:0.019,(((((Kty00026:0.03231,(UltTer15:0.01137,((UltTer13:0.003
72,UltTer12:0.00746)0.791.76:0.00365,(((UltTer14:0.0038,UltEnd27:0.01144)
0.797.74:0.00364,UltEnd26:0.00745)0.335.11:5.5E-
4,UltEnd25:0.0074)1.000.808:5.4E-
4)0.772.98:0.00379)0.821.71:0.00546)0.873.111:5.4E-
4,(((MaswggYy:0.0,MaswgY18:0.0,MaswgY19:0.0,MaswgY20:0.0):0.04792,(Otu000
12:5.5E-
4,(CDEvvE00:0.00746,UltTer24:0.03113)0.752.78:0.00371)0.746.62:0.00534)0.
865.114:0.01378,(UltEnd28:0.01246,(UltTer10:5.5E-
4,(UltTerm9:0.00754,((Ult32006:0.0,Otu00198:0.0):0.01474,(UltEnd29:0.0112
5,UltTer11:0.01514)0.835.89:0.00723)0.890.120:5.5E-
4)0.495.13:0.00371)0.806.60:0.00646)0.895.126:0.01692)0.377.13:0.00776)0.
934.94:0.00524,(UltTer22:0.01126,UltTer18:0.04776)0.857.118:0.00757)0.822
.65:0.00374,(((UltEnd40:5.5E-
4,UltEnd39:0.00743)0.856.98:0.00369,UltTer21:0.03041)0.933.126:5.5E-
4,(Otu00020:0.00364,Otu00190:0.02332)0.914.130:0.01146)0.823.78:0.00365)0
.929.105:5.0E-4,UltEnd30:5.4E-4)0.950.96:5.4E-
4)0.637.11:0.00374)0.783.105:0.00388)0.738.49:0.00381,(((((Otu00176:0.008
66,(((ZnvYyy08:0.0188,((Sgfffff6:0.03882,(UltEnd21:0.00744,(UltEnd22:0.0,
UltEnd23:0.0):5.5E-4)0.499.10:5.3E-
4)0.842.104:0.00366,(((ZnvYyy05:0.00749,(ZnvYyy07:0.01117,ZnvYyy06:5.5E-
4)0.772.99:0.00372)0.778.93:0.00369,(Sgffff16:0.0037,(ZnvYyyy3:0.0,ZnvYyy
y2:0.0):0.00745)0.559.13:5.3E-4)0.836.82:0.00369,(Otu00075:5.5E-
4,(ZnGrG002:0.05616,UltEnd20:0.0076)0.776.95:0.00344)0.750.59:5.4E-
4)0.563.10:5.4E-4)0.145.5:5.5E-4)0.957.100:5.4E-
4,(UltEndom:0.0,ZnCTTCTC:0.0):0.00738)0.847.131:0.01113,((UltEnd32:0.0074
2,UltEnd33:5.5E-4)0.783.106:0.00371,((UltTer17:0.0,Otu00007:0.0):5.4E-
4,Otu00099:0.02666)0.815.69:0.00745)0.763.106:0.00498)0.738.50:0.01261)0.
798.79:0.00918,((Hmyyy062:5.5E-
4,Hmyyy063:0.00369)0.798.80:0.00439,((Hmyy0036:5.4E-4,(Hmyy0034:5.5E-
4,Hmyy0035:5.5E-
4)0.912.143:0.00744)0.976.77:0.0306,(UltTer25:0.06741,(((Kty00025:0.0,Kty
y0026:0.0,Kty00027:0.0):0.01488,KtYyyyy3:0.03995)0.958.111:0.06117,((Hmyy
0026:5.5E-
4,Hmyy0029:0.00369)0.963.84:0.05013,(Hmyy0030:0.00936,((Hmyy0027:5.4E-
4,(Hmyy0033:5.5E-
4,(Hmyy0032:0.01494,(Hmyy0028:0.01362,HmZZZZ07:0.02672)0.745.79:0.00379)0
.098.2:5.4E-
4)0.987.116:0.01703)0.966.111:0.0111,(UltTer23:0.00366,Hmyy0031:5.5E-
4)0.972.94:5.4E-
4)0.995.106:0.04539)0.412.13:0.03063)0.958.112:0.06109)0.873.112:0.0365)0

.894.118:0.02177)0.725.37:0.00437)0.980.71:0.0248)0.269.10:0.00382,UltTer
16:0.01585)0.828.64:0.01155,Otu00296:0.01844)0.951.104:0.0198,UltEnd35:0.
00547)0.714.28:0.00557)0.908.133:0.0076)0.459.16:5.4E-
4)0.964.104:0.02877,Ult32007:0.02697)0.400.12:0.01136,UltRu560:0.03075)0.
962.112:0.02677)0.274.7:0.007,(Ult32009:0.04207,Ult32010:0.02525)0.829.89
:0.01258)0.901.120:0.02833)0.428.15:0.0342)0.934.95:0.04438,((Ult32012:0.
14759,((((Ult32019:0.05794,UltTer32:0.04107)0.842.105:0.01062,(Ult32020:0
.04066,(Ult32018:0.11139,(((Ult32016:0.00755,Ult32015:0.01524)0.892.115:0
.01145,(UltTer31:0.03077,(Ult32014:0.02226,(UltAc944:0.08106,(Ult32013:0.
01524,(UltTer29:0.004,UltTer30:0.0352)0.394.18:0.0076)0.710.31:0.00298)0.
809.79:0.0096)0.703.30:0.01602)0.995.107:0.05459)0.743.72:0.00356,Ult3201
7:0.00786)0.926.136:0.02896)0.866.116:0.02752)0.594.14:0.00758)0.890.121:
0.02491,(Ult32035:0.03076,UltTer35:0.03755)0.861.123:0.01906)0.929.106:0.
03881,(Ult32027:0.13483,(UltB4780:0.0523,(Ult32024:0.07797,(UltThe39:0.02
079,(UltCl397:0.00966,(Ult32023:0.05855,(Ult32021:0.0112,Ult32022:0.01542
)0.903.107:0.01252)0.783.107:0.00877)0.997.109:0.06521)0.889.116:0.03539)
0.829.90:0.02276)0.953.87:0.0399)0.837.71:0.01947)0.969.77:0.06255)0.150.
9:0.00462,((((Ult32064:0.08132,(UltTer37:0.12484,(Ult32039:0.01875,UltTer
36:5.3E-
4)0.519.9:0.02099)0.973.81:0.04891)0.699.26:0.0356,((UltTer38:0.03663,(Ul
t32046:0.03947,(Ult32045:0.01346,Ult32047:0.03451)0.038.7:0.00387)0.850.1
17:0.01431)0.953.88:0.04501,(((Ult32071:0.02336,Ult32074:0.03597)0.805.70
:0.00798,((UltArch4:0.02666,(Ult32073:0.02707,((Ult32070:0.00879,(Ult3206
5:0.02955,(Ult32072:0.01621,UltrS461:0.0228)0.534.12:0.00543)0.847.132:0.
01635)0.719.26:0.00237,(Ult32075:0.05674,Ult32066:0.00801)0.889.117:0.011
02)0.920.130:0.00761)0.908.134:5.4E-
4)0.697.13:0.0077,(UltTer40:0.0511,(UltrS460:0.01934,Ult32067:0.02306)0.5
64.11:0.0038)0.889.118:0.01341)0.864.112:0.01085)0.862.112:0.01092,(Ult32
068:0.004,Ult32069:0.00728)0.928.93:0.02044)0.959.103:0.03283)0.428.16:0.
00903)0.822.66:0.01548,(UltAc949:5.4E-
4,((((UltrS459:0.01137,(Ult32060:0.01142,(Ult32061:0.0728,UltTer39:0.0166
5)0.724.31:0.00213)0.767.81:0.00383)0.909.103:0.01741,Ult32063:0.03011)0.
842.106:0.01886,(Ult32062:0.02317,(Ult32055:0.00747,((Ult32051:5.5E-
4,(Ult32050:0.00369,Ult32052:0.01136)0.736.51:5.4E-4)0.819.77:5.4E-
4,((Ult32049:0.01896,Ult32053:0.00395)0.134.12:0.01894,((UltrS457:0.00768
,(Ult32048:0.01534,Ult15449:0.07085)0.830.82:0.00763)0.835.90:0.00694,Ult
32054:0.01594)0.949.110:0.0196)0.501.12:5.4E-
4)0.926.137:0.01133)0.737.68:0.00375)0.751.60:0.00592)0.464.16:0.00918,(U
lt32059:0.01678,((AcuYJF13:5.3E-4,(Ult32056:5.5E-
4,UltrS458:0.00369)0.893.127:0.0071)0.531.10:0.00467,(Ult32057:5.5E-
4,Ult32058:0.01119)0.859.106:0.01001)0.852.106:0.01301)0.942.101:0.02432)
0.737.69:0.0045)0.969.78:0.02714)0.658.13:0.02599,Ult32044:0.1368)0.751.6
1:0.0063)0.781.86:0.02286)0.399.16:0.02161)0.896.111:0.03328,(Ult32036:0.
00422,Ult32037:0.02237)0.972.95:0.04805)0.836.83:0.02564)0.994.102:0.1177
1,(UltDe234:0.13342,(Ult32078:0.0562,Ult32077:0.06055)0.993.84:0.09486)0.
163.11:0.01133)0.455.23:0.01822,Ult32076:0.06234)0.837.72:0.02642)0.726.2
9:0.02512,(CanPisci:0.17052,(((((UltChla9:0.08785,(CblSequa:0.0754,EstLau
sa:0.03961)0.809.80:0.01989)0.915.115:0.02489,(Ult12533:0.16997,((((Ult12
534:0.00861,((UltChl55:0.0527,(Ult12532:0.03208,(NchSpeci:0.01107,NchHart
m:5.4E-
4)0.942.102:0.02266)0.383.10:0.00892)0.845.108:0.0094,UltPara5:0.02682)0.
852.107:0.00781)0.749.91:0.0047,((PmdAcant:0.01952,UltPara4:0.01518)0.920
.131:0.01541,(EnmbnO29:0.00745,PhdBacte:0.03216)0.905.117:0.01336)0.811.6
6:0.00899)0.846.120:0.01138,((CanProto:0.01546,PtdNaegl:0.00748)0.961.94:

0.02472,(UltPara3:0.0185,(CanProt2:0.00728,UltrS147:0.03828)0.367.18:0.00
522)0.861.124:0.00838)0.152.13:0.00439)0.814.64:0.00648,(Ult12538:0.05456
,(Ult12537:0.05881,(WadSpeci:0.01085,(WadChon2:0.01872,WadChond:5.5E-
4)0.912.144:0.01193)0.973.82:0.0378)0.762.100:0.01223)0.887.123:0.01758)0
.320.7:0.00584)0.688.22:0.00535)0.740.62:0.00445,UltChla8:0.05229)0.898.1
32:0.01101,((((UltChla6:0.04139,UltChla7:0.05968)0.893.128:0.0218,ClsBact
3:0.08488)0.839.95:0.01576,(UltChl57:0.06576,(ChySpeci:0.03244,((ChydSuis
:0.01902,(ChyTrac2:0.00374,(ChyTrach:5.5E-4,ChyTrac3:5.5E-
4)0.950.97:5.5E-
4)0.852.108:0.00792)0.866.117:0.0128,(CdpPecor:0.01939,(CdpPsitt:0.0076,C
dpPneum:0.01906)0.833.92:0.00769)0.416.27:0.01223)0.964.105:0.04702)0.446
.12:0.01993)0.992.94:0.05911)0.655.22:0.00527,((Ult12539:0.09897,UltChla5
:0.0946)0.849.101:0.02855,((((UltCan32:0.09139,(UltCan33:0.01733,(CanRhab
2:0.00769,CanRhabd:0.01234)0.983.71:0.03156)0.000.846:0.00396)0.544.12:0.
01567,(UltChla2:0.01881,ClsBacte:5.4E-
4)0.740.63:0.00433)0.933.127:0.02853,((Ult12530:0.01319,(UltChla3:0.02304
,RbdBacte:0.05101)0.797.75:0.00817)0.990.96:0.04498,(ClsSymbi:0.0188,(Can
Frit2:0.00745,CanFrits:0.0037)0.741.59:0.00413)0.994.103:0.04957)0.199.6:
0.00752)0.722.29:0.01447,(UltChla4:5.4E-
4,(SmkNegev:0.03135,ClsBact2:0.00376)0.790.83:0.00744)0.924.115:0.02119)0
.827.77:0.01722)0.885.109:0.02113)0.629.20:0.00111)0.902.124:0.02498,((Cl
sBact4:0.01336,((UltChl56:0.06598,(Ult12535:0.02543,(Ult12536:0.02079,Cls
Bact6:0.02368)0.966.112:0.03333)0.808.75:0.01031)0.768.79:0.00846,ClsBact
5:0.059)0.728.40:0.00965)0.999.165:0.08902,Ult12531:0.04161)0.696.19:0.00
953)0.893.129:0.0322)0.983.72:0.07948)0.930.102:0.04283)0.843.94:0.01454,
(((UltDef15:0.18101,(UltrdL20:0.09568,(((Ult31182:0.11104,Ult31175:0.0558
)0.759.98:0.01019,(((((Ult31183:0.02499,Ult31184:0.01124)0.999.166:0.0635
2,(Ult31181:0.07362,(Ult31179:0.00714,Ult31180:0.03702)0.983.73:0.04262)0
.045.8:5.5E-
4)0.822.67:0.01172,(Ult31177:0.10223,(Ult31173:0.10009,(Ult31170:0.0203,(
Ult31171:0.02383,Ult31172:0.02822)0.778.94:0.00713)0.701.24:0.01348)0.614
.10:0.02493)0.928.94:0.03241)0.855.104:0.0158,(Ult31176:0.05959,(Ult31178
:0.06265,Ult31186:0.02578)0.811.67:0.02724)0.848.98:0.02197)0.379.9:0.012
3,(Ult31174:0.05534,Ult31185:0.07981)0.843.95:0.01902)0.735.43:0.00405)0.
967.86:0.03485,(Ult31193:0.16063,(Ult31192:0.14754,(UltOr481:0.10052,(Ult
De233:0.06056,(Ult31191:0.09,(((Ult31188:0.01148,Ult31189:0.00376)0.891.9
8:0.00776,((Otu00164:5.4E-
4,(UltCa380:0.00511,Ult31187:0.03095)0.405.19:0.01395)0.920.132:0.01869,(
UltCa379:0.00748,((Ult31190:5.4E-
4,Hmyyy029:0.03151)0.830.83:0.00372,UltCa381:0.00374)0.639.20:5.5E-
4)0.779.88:0.01088)0.863.131:5.5E-
4)0.759.99:0.00716,UltCa382:0.03015)0.956.105:0.05099)0.907.121:0.03706)0
.209.13:0.01546)0.198.12:0.01684)0.867.121:0.03765)0.785.94:0.04629)0.087
.8:0.00192)0.936.110:0.04196)0.493.19:0.03155,((((((Ult17364:0.00374,((Ul
t17361:0.02288,(Ult17366:0.00743,(Ult17363:0.0,Ult17367:0.0):5.5E-
4)0.920.133:5.5E-4)0.850.118:0.00713,Ult17365:5.1E-
4)0.783.108:0.0037)0.824.70:0.00675,Ult17368:0.00454)0.934.96:0.04617,(Ba
cLinco:0.02181,Ult17369:0.02699)0.549.18:0.01933)0.978.73:0.05643,((UltDe
f10:0.03886,((UltDef11:0.03109,(UltDef12:0.05984,Otu00275:0.05528)0.605.1
6:0.03764)0.897.118:0.02726,UncUn126:0.01004)0.730.45:0.00436)0.708.30:0.
02055,Ult17370:0.05076)0.946.102:0.04625)0.915.116:0.03959,((DisSelen:0.0
3035,GvbThiop:8.7E-
4)0.998.135:0.08169,(DnbAceti:0.04686,Ult17371:0.0683)0.973.83:0.05506)0.
413.18:0.01003)0.843.96:0.027,((Ult17381:0.01917,((Ult17378:0.0,CdrNitro:

0.0,Ult17380:0.0):0.00341,(Ult17376:0.00369,Ult17377:5.5E-
4)1.000.809:5.5E-
4)0.750.60:0.01575)0.963.85:0.03527,((Ult17372:0.01993,Ult17373:0.00687)0
.059.11:0.01226,(((Ult17374:0.00745,Ult17383:5.5E-
4)0.908.135:0.0074,(Ult17375:0.00757,UltDef13:5.5E-4)0.578.8:5.5E-
4)0.949.111:0.02225,((DrrTherm:0.00752,(DrrSpeci:5.5E-4,Ult17382:5.5E-
4)0.589.13:5.5E-
4)0.889.119:0.01659,(DrrAbyss:0.01935,DrrAutot:0.00748)0.073.5:0.01429)0.
991.95:0.03896)0.829.91:0.01175)0.846.121:0.02449)0.935.110:0.04183)0.975
.85:0.07513)0.924.116:0.0394,(((Ult12648:0.07979,((((Ult12679:0.01399,(((
Ult12680:0.01517,Ult12682:0.0408)0.921.120:0.01894,Ult12681:0.02823)0.000
.847:5.3E-
4,Ult12683:0.06163)0.667.23:0.00961)0.884.123:0.01495,(UltVe277:0.06118,(
Ult12685:0.05244,(UltVe276:0.03865,Ult12684:0.02201)0.752.79:0.02056)0.93
6.111:0.03538)0.460.11:0.00969)1.000.810:0.08606,((((Ult12633:0.0604,((Ul
t12634:0.05078,Ult12635:0.10237)0.998.136:0.12896,((Ult12632:0.0649,(Ult1
2630:0.0388,Ult12631:0.01882)0.963.86:0.05243)0.783.109:0.02544,(UltVe273
:0.13496,(UltCan47:0.03542,UltVe272:0.07514)0.937.110:0.04536)0.787.82:0.
02671)0.891.99:0.05086)0.843.97:0.05599)0.677.16:0.01901,Ult12678:0.1059)
0.831.76:0.02336,((((Ult12622:0.08321,Ult12621:0.04722)0.803.69:0.0524,((
UltVe271:0.06075,(UltVe270:0.01532,(Ult12604:0.02321,Ult12603:0.04403)0.6
71.12:0.00536)0.867.122:0.02713)0.939.108:0.03749,((((Ult12595:0.02433,((
UltrS149:0.02963,(UdnBac50:0.02288,UltOr190:0.02767)0.855.105:0.01747)0.9
23.155:0.02682,(((((Ult12586:0.00368,Ult12587:0.00369)0.825.75:5.4E-
4,(UltVe269:0.01681,(Ult12593:0.05688,Ult12588:0.01453)0.188.7:0.01019)0.
434.10:0.00608)0.824.71:5.4E-
4,((Ult12591:0.01763,(Ult12589:0.03012,Ult12590:0.06242)0.242.10:0.00985)
0.930.103:0.02208,(Ult12592:0.06381,Ult12594:0.05291)0.940.104:0.03078)0.
744.66:0.01469)0.785.95:0.02182,MixCultu:0.03523)0.968.86:0.03424)0.792.9
6:0.01209)0.949.112:0.03842,Ult12602:0.02465)0.479.17:0.0219,Ult12605:0.0
3267)0.875.126:0.03686,(Ult12601:0.0743,(Ult12599:0.02666,((Ult12598:0.00
752,(Ult12596:5.4E-4,Ult12600:0.0478)0.898.133:0.00744)0.857.119:5.5E-
4,Ult12597:0.00783)0.858.110:0.01613)0.875.127:0.01731)0.509.10:0.02961)0
.619.8:0.02479)0.942.103:0.04787)0.938.70:0.06835,(((Ult12617:0.03401,Ult
Can45:0.05372)0.975.86:0.04126,(Ult12611:0.04037,UltCan44:0.01933)0.102.9
:0.00969)0.513.10:5.4E-
4,((Ult12610:0.0136,(Ult12608:0.00404,((Ult12607:0.0037,(UltCan43:0.00378
,Ult12609:0.02297)0.790.84:0.00365)1.000.811:5.5E-
4,Ult31860:0.1031)0.184.9:0.00324)0.929.107:0.02655)0.997.110:0.07355,(((
Ult12612:0.00476,Ult12613:0.02193)0.969.79:0.05039,((UltrS150:0.14925,Ult
12616:0.03324)0.753.87:0.03028,Ult12615:0.09775)0.829.92:0.02916)0.778.95
:0.01296,(Ult12618:0.05994,Ult12619:0.10593)0.858.111:0.04945)0.780.111:0
.0239)0.872.117:0.02877)0.599.12:0.00906)0.290.12:0.03237,(Ult12620:0.113
06,(UltCan46:0.14601,((Ult12624:0.03083,((UdnBac51:0.02813,((Ult12625:0.0
3972,Ult12628:0.17366)0.829.93:0.01772,Ult12623:0.03044)0.823.79:0.01207)
0.719.27:0.00825,Ult12626:0.06884)0.116.9:0.01372)0.901.121:0.02168,Ult12
627:0.06674)0.191.8:0.02411)0.990.97:0.09483)0.870.103:0.04224)0.973.84:0
.05294)0.872.118:0.01458,(((((UltCan51:0.08929,((UltOr191:0.02043,(Ult126
44:0.04559,((UltCan49:0.02619,UltCan50:0.01352)0.983.74:0.04968,(Ult12645
:0.0489,((Ult12638:0.03062,UltVe274:0.03811)0.879.128:0.02841,(((Ult12640
:0.0,Ult12641:0.0,Ult12642:0.0):0.01802,UltBani3:0.0055)0.978.74:0.03177,
(Ult12637:0.01896,(UltCan48:0.03386,((Ult12636:0.01867,UltPl551:0.0043)0.
097.8:0.01122,Ult12639:0.10668)0.866.118:0.01732)0.831.77:0.01056)0.000.8
48:0.00157)0.792.97:0.01413)0.876.106:0.02395)0.471.14:0.0056)0.690.25:0.

00864)0.922.161:0.0269)0.858.112:0.01365,(Ult12643:5.4E-
4,UltVe275:0.00724)0.792.98:0.00679)0.998.137:0.0983)0.129.8:0.01529,((Ul
t12662:0.09772,(Ult12649:0.06728,(Ult12650:0.05635,(Ult12651:0.01493,Ult1
2652:0.00788)0.865.115:0.01765)0.289.7:0.01623)0.939.109:0.03559)0.583.12
:0.0047,(Ult12647:0.02807,Ult12646:0.05555)0.955.91:0.03704)0.891.100:0.0
2373)0.868.101:0.01314,((Ult12663:0.0508,(Ult12658:0.04183,(Ult12661:0.04
887,(UltOr192:5.3E-
4,(Ult12657:0.01345,(Ult12655:0.01745,Ult12656:0.01315)0.938.71:0.01562)0
.915.117:0.01289)0.175.7:0.00352)0.933.128:0.02696)0.944.91:0.02149)0.663
.19:0.00327,(Ult12687:0.04842,Ult12686:0.04981)0.992.95:0.07147)0.880.129
:0.02032)0.760.93:0.00894,(((Ult12664:0.03914,Ult12665:0.03948)0.763.107:
0.00637,((Ult12666:0.08042,UltCan53:0.01021)0.802.73:0.01088,Ult12667:0.0
0582)0.871.117:0.00829)0.940.105:0.02562,(((Ult12677:0.06893,UltCan52:0.0
6479)0.861.125:0.0209,Ult12669:0.00852)0.975.87:0.03315,Ult12668:0.0317)0
.869.95:0.01652)0.973.85:0.03819)0.879.129:0.02059,(((Ult12654:0.02444,Ul
t12653:0.0368)0.991.96:0.07964,(((Ult12674:0.02288,((((Ult12671:0.0164,Ul
t12675:0.01845)0.380.11:0.00334,Ult12676:0.03043)0.895.127:0.00836,(UdnBa
c52:0.011,Ult12672:0.01951)0.690.26:0.00381)0.790.85:5.5E-
4,Ult12670:0.02319)0.638.15:0.00375)0.873.113:5.5E-
4,Ult12673:0.01115)0.968.87:0.03975,(Ult12659:0.03632,Ult12660:0.08084)0.
971.75:0.06308)0.600.12:0.01488)0.934.97:0.03569,(UltDe150:0.09527,CddDiv
i2:0.16551)0.405.20:0.01456)0.904.119:0.03056)0.919.149:0.02484)0.743.73:
5.4E-
4)0.964.106:0.04537,((UltCan41:0.06911,(Ult12555:0.02827,UltPl550:0.0419)
0.985.89:0.06915)0.499.11:0.03096,((Ult12552:0.00913,Ult12553:0.01766)0.7
31.35:0.0122,(((((((UltVe267:0.01874,Ult12546:0.04924)0.932.113:0.01587,(
(Ult12545:5.5E-
4,(UltVe266:0.00982,Ult12544:0.05173)0.918.124:0.01718)0.815.70:0.00745,U
ltAna10:0.00746)0.898.134:0.0091)0.746.63:0.00486,(Ult12548:0.06497,UltCa
n34:0.04973)0.758.89:0.00999)0.176.13:0.01034,UltCan35:0.03004)0.930.104:
0.02549,(Ult12547:0.00455,(((UltCan36:0.01258,UltCan37:0.00646)0.861.126:
0.01766,(Ult12551:0.01146,(Ult12549:0.021,UltVe268:0.05422)0.482.16:0.018
88)0.831.78:0.01884)0.847.133:0.01254,(Ult12550:0.01148,(UltrS148:0.01139
,UltCan38:0.0076)0.744.67:0.00348)0.754.74:0.00401)0.711.30:0.00285)0.716
.29:0.00473)0.909.104:0.02045,UltCan39:0.02142)0.977.77:0.04166,(Ult12554
:0.0459,UltPl549:0.03277)0.934.98:0.03005)0.179.6:0.03054)0.952.98:0.0654
4)0.685.17:0.05837)0.916.128:0.02933)0.959.104:0.02905,((Ult16763:0.01187
,(((Ult16765:0.00603,((Ult16762:0.01997,Ult16764:0.02284)0.970.91:0.02629
,((Ult16770:0.0,Ult16773:0.0):5.4E-
4,((Ult16766:0.02266,(Ult16767:0.03031,UltNit92:0.00436)0.752.80:0.0073)0
.921.121:0.02842,(Ult16772:0.00367,(UltNit93:5.5E-
4,(Ult16771:0.0074,(Ult16768:0.00746,Ult16769:0.01504)0.920.134:5.4E-
4)0.837.73:0.0111)0.915.118:0.00718)0.812.67:0.00725)0.936.112:5.4E-
4)0.778.96:0.00868)0.818.53:0.01036)0.735.44:0.01767,Ult16774:0.00565)0.8
61.127:0.0123,Ult16761:0.00965)0.919.150:0.03631)1.000.812:0.11177,((Ult1
6640:0.04936,(Ult16639:0.05537,(Ult16637:0.0156,(Ult16638:0.01125,Ult1664
1:5.5E-
4)0.995.108:0.04524)0.803.70:0.02175)0.448.14:0.02263)0.952.99:0.05187,((
Ult16635:0.16564,((Ult16593:0.04814,(UltNit46:0.0687,(Ult16591:0.02531,Ul
t16592:0.04628)0.852.109:0.01973)0.916.129:0.04064)0.892.116:0.03461,((Ul
t16597:5.3E-
4,UltNit47:0.01494)0.966.113:0.04618,((((Ult16568:0.00766,(UltNit32:0.015
33,UltNit33:0.00762)0.749.92:0.00333)0.935.111:0.01517,UltNit34:0.00384)0
.709.24:0.00261,Ult16567:0.0322)0.948.125:0.03514,((Ult16589:0.02742,((((

(UltNit37:0.01482,(Ult16571:0.01509,(UltNit36:0.03532,Ult16576:0.01148)0.
750.61:0.0034)0.879.130:5.5E-
4)0.827.78:0.00757,(((Ult16577:0.00751,Ult16578:0.00743)0.886.141:0.00735
,((Ult16572:0.00373,Ult16575:0.01912)0.787.83:0.00366,((Ult16573:5.5E-
4,UltNit38:0.00746)0.442.20:0.00368,(Ult16574:5.5E-
4,(Udntded2:0.01127,(Ult16569:5.5E-4,Ult16570:5.5E-4)0.793.86:5.5E-
4)0.741.60:0.00369)0.998.138:5.4E-4)0.824.72:0.00365)0.379.10:5.5E-
4)0.965.89:5.5E-
4,UltNit35:0.02286)0.327.14:0.00713)0.759.100:0.00428,(Udntfd11:0.01503,(
Udntded3:5.5E-
4,Udntfd10:0.14912)0.564.12:0.0038)0.987.117:0.02788)0.807.65:0.00726,Ult
16579:0.0316)0.764.98:0.00428,((((Ult16586:0.02273,(Ult16588:0.00375,((Ul
tNit43:0.00373,UltNit44:5.5E-
4)0.947.89:0.01568,(UltNit42:0.01913,((Ult16580:5.5E-4,Ult16581:5.5E-
4)0.913.128:5.5E-4,((UltNit39:0.00371,UltNit40:5.5E-
4)0.942.104:0.01125,(UltNit41:0.0037,(Ult16583:0.00799,Ult16582:0.01152)0
.890.122:5.4E-4)0.920.135:0.00745)0.266.10:5.5E-
4)0.782.110:0.00392)0.863.132:0.00776)0.900.102:0.01126)0.769.94:5.4E-
4)0.765.82:0.00754,UltNit45:0.00353)0.876.107:0.00764,Ult16587:0.0076)0.7
63.108:0.00529,(Ult16584:0.01278,Ult16585:0.03732)0.253.13:0.00907)0.958.
113:0.0217)0.405.21:0.0038)0.972.96:0.04302,(((UltNit31:0.02752,(Ult16566
:0.02808,(UdnBac65:0.01795,UdnBac66:0.01396)0.975.88:0.03905)0.845.109:0.
01349)0.928.95:0.01619,((((Ult16536:5.4E-
4,((Ult16558:0.00975,(Ult16538:0.00461,(UltNit26:0.01103,(Ult16557:0.0036
9,((NrpSpec4:0.0037,(Ult16552:5.5E-
4,(((NrpSpec2:0.00369,(Ult16553:0.0,Ult16554:0.0,Ult16555:0.0,Ult16556:0.
0):5.5E-4)0.000.849:5.5E-4,CanNitro:5.5E-4)0.000.850:5.5E-
4,NrpSpec3:5.5E-4)0.756.90:5.5E-4)0.782.111:5.5E-
4)0.937.111:0.00742,(NrpSpec5:0.00741,UltNit25:5.5E-4)0.400.13:5.5E-
4)0.771.113:0.00371)0.884.124:0.00775)0.859.107:0.01071)0.979.89:0.02903)
0.790.86:0.02241,(Ult16560:0.00741,(Ult16534:0.01117,Ult16559:5.5E-
4)0.923.156:5.5E-
4)0.664.18:0.00569)0.374.18:0.00546)0.951.105:0.01501,Ult16561:5.5E-
4)0.849.102:0.00969,(((Ult16565:5.5E-
4,UltrS293:0.00368)0.999.167:0.0409,(UltNit29:0.00742,(UltNit17:0.00269,U
ltNit28:0.02308)0.831.79:0.00139)0.873.114:0.01158)0.913.129:0.01197,((Ul
t16551:0.01778,(((Ult16547:0.01126,((UltNit23:0.01911,(Ult16535:0.00721,U
lt16546:0.02702)0.902.125:5.4E-4)0.887.124:5.4E-4,UltNit19:5.5E-
4)0.810.74:5.4E-
4)0.802.74:0.0058,((UltNit22:0.03089,((((Ult16545:0.00745,((Ult16544:0.00
747,((UltNit18:5.5E-4,UltNit15:0.0037)0.801.80:5.4E-
4,UltNit16:0.0075)0.834.76:0.00371)0.709.25:5.4E-4,UltNit24:5.5E-
4)0.839.96:0.0037)0.927.116:5.5E-4,UltGree6:5.5E-4)0.976.78:5.5E-
4,(((UltGree5:5.5E-4,(Ult16548:0.00746,Ult16549:0.00372)0.723.36:5.5E-
4)0.734.60:5.1E-4,UltGree4:0.00372)0.867.123:0.00373,UltNit21:5.5E-
4)0.828.65:0.00372)0.407.14:0.0038,(Ult16523:0.0,UltNit12:0.0):0.027)0.88
3.125:5.5E-4)0.488.16:0.00375,(FreSedi2:0.00371,Ult16550:5.4E-
4)0.782.112:0.00347)0.789.81:0.0079)0.894.119:0.01324,(((((((Ult16524:0.0
0753,Ult16528:0.00764)0.882.131:0.00739,((Ult16529:0.0,Ult16527:0.0):5.5E
-4,(Ult16533:0.00368,UltNit14:5.5E-
4)0.854.104:0.00369)0.859.108:0.0037)0.296.9:5.5E-4,(NrpMosco:5.4E-
4,((Ult16531:5.5E-4,(UltrS292:0.02662,Ult16530:0.00368)0.771.114:5.5E-
4)0.699.27:5.5E-
4,Ult16532:0.01124)0.906.133:0.00739)0.849.103:0.00367)0.894.120:0.00738,

(Ult16526:5.5E-
4,((Ult16540:0.0,Ult16541:0.0,NrpSpeci:0.0,Ult16525:0.0):5.5E-
4,(Ult16542:0.00744,Ult16543:0.00369)0.728.41:5.5E-4)0.756.91:5.5E-
4)0.909.105:5.4E-
4)0.419.9:0.0037,UltNit13:0.00379)0.961.95:0.01118,(UltNit11:0.01896,(Ult
16537:5.5E-
4,(UltNit10:0.00372,Ult16520:0.00368)0.907.122:0.00746)0.834.77:0.00367)0
.345.10:5.4E-4)0.750.62:0.00747,(Ult16522:0.0,Ult16521:0.0):5.5E-
4)0.929.108:0.0115)0.747.69:0.00481)0.506.12:0.01205,UltNit30:0.01757)0.9
16.130:0.01707)0.062.9:0.00307)0.872.119:0.01004,((UltNit27:0.00369,Ult16
563:5.5E-
4)0.980.72:0.02344,Ult16564:0.00757)0.862.113:0.00775)0.206.8:5.4E-
4)0.768.80:0.00506,Ult16562:0.00596)0.497.6:0.01615)0.945.92:0.04369)0.93
0.105:0.03778)0.903.108:0.03318)0.608.19:0.01626)0.919.151:0.04224,(Ult16
636:0.02951,(((Ult16602:0.01512,(((Ult16601:5.4E-4,(Ult16604:5.5E-
4,UltPro84:5.5E-
4)0.992.96:0.01508)0.834.78:0.00367,(Ult16598:0.00368,(Ult16627:0.00369,(
(LppSpeci:0.0,Ult16599:0.0,Ult16628:0.0):5.5E-
4,(UltLep16:0.00369,(Ult16629:0.0037,UltLep18:0.0074)0.000.851:5.5E-
4)0.648.18:5.5E-4)0.000.852:5.5E-4)0.590.10:5.5E-4)0.722.30:5.4E-
4)0.849.104:0.00376,(UltLep17:0.00719,(((LppFerr2:0.00374,Ult16612:0.0111
4)0.399.17:0.00369,((Ult16622:0.0227,(Ult16620:0.00368,(Ult16624:0.00743,
(((LppFerr3:0.00373,Ult16625:0.0074)0.286.11:5.5E-
4,Ult16623:0.00369)0.494.11:5.5E-4,(Ult16619:0.0,Ult16621:0.0):5.5E-
4)0.208.7:5.5E-4)0.377.14:5.5E-4)0.188.8:5.5E-4)0.286.12:5.5E-
4,Ult16618:5.5E-4)0.903.109:5.5E-4)0.954.92:0.01114,Ult16603:5.5E-
4)0.883.126:0.00712)0.916.131:5.4E-4)0.708.31:0.00371)0.395.17:5.5E-
4,((((((UltLep21:0.0037,UltNit48:0.0037)0.084.8:5.5E-
4,(UltLep20:0.00373,(Ult16605:0.00722,UltLep19:5.4E-
4)0.866.119:0.00727)0.789.82:0.00369)0.956.106:0.01125,Ult16609:5.4E-
4)0.839.97:0.00365,Ult16608:5.3E-
4)0.813.74:0.00989,((((Ult16607:0.0,Ult16613:0.0):5.5E-4,Ult16616:5.5E-
4)0.795.85:0.00376,(((Ult16614:5.5E-4,((Ult16611:0.0,LppFerro:0.0):5.5E-
4,UdnBac67:0.015)0.000.853:5.5E-4)0.892.117:5.5E-
4,Ult16610:0.00367)0.772.100:5.5E-
4,Ult16615:0.01501)0.785.96:0.00364)0.879.131:0.011,Ult16606:0.01623)0.65
0.16:0.00499)0.993.85:0.04469,Ult16617:0.00367)0.785.97:0.00377)0.949.113
:0.00321,(Ult16600:5.5E-4,Ult16626:0.00742)1.000.813:5.5E-
4)1.000.814:0.20849)0.575.8:0.03221)0.977.78:0.07597)0.188.9:0.01075)0.94
0.106:0.03742)0.697.14:0.00316,((Ult12529:0.06966,Ult12528:0.09786)0.996.
109:0.10515,(Ult12294:0.29015,(UltB9462:0.05394,(UltB9463:0.0581,UltB9464
:0.03155)0.897.119:0.03548)0.990.98:0.13092)0.752.81:0.05613)0.450.14:5.5
E-
4)0.891.101:0.01882)0.711.31:0.00669)0.440.11:0.0057,(((((((UltB5034:0.02
151,(UltB5033:0.03493,UltSp220:0.0782)0.779.89:0.0232)0.982.71:0.06839,(U
ltB5039:0.13322,Ult12584:0.13551)0.883.127:0.03611)0.944.92:0.05,(UltB504
2:0.01701,(UltSp227:0.04017,(UltB5040:0.03485,(UltB5041:5.4E-
4,UltSp226:0.03171)0.439.14:0.02262)0.978.75:0.05061)0.914.131:0.03328)0.
926.138:0.04491)0.968.88:0.06292,(UltBa764:0.05388,Ult32223:0.09051)0.920
.136:0.05467)0.937.112:0.03868,((Ult16775:0.0624,(Ult16596:0.01402,(Ult16
594:0.02777,Ult16595:0.01184)0.979.90:0.05691)0.984.77:0.05746)0.824.73:0
.02908,(Ult13201:0.09697,((Ult12577:0.03221,(Ult12576:0.01456,Ult12578:5.
3E-
4)0.941.97:0.05184)0.920.137:0.04859,(Ult16797:0.10609,(Ult16794:0.02733,

(Ult16793:0.01484,(Ult16792:5.4E-
4,(Ult16789:0.0151,(Ult16790:0.01881,Ult16791:5.3E-4)0.222.7:5.4E-
4)0.958.114:0.01125)0.847.134:0.01169)0.608.20:0.01074)0.909.106:0.04364)
0.948.126:0.05703)0.834.79:0.03631)0.403.14:0.0279)0.888.127:0.03134)0.18
8.10:0.00339,((Ult13001:0.10831,((Ult13185:0.05842,Ult13186:0.04112)0.998
.139:0.1086,(Ult13189:0.08122,(Ult13187:0.1837,Ult13188:0.04649)0.748.72:
0.03281)0.932.114:0.06573)0.878.109:0.04232)0.745.80:0.00811,(Ult13195:0.
29058,Ult13145:0.05792)0.769.95:0.02743)0.984.78:0.05762)0.000.854:0.0038
7,((((((Ult12582:0.07919,Ult12688:0.11297)0.886.142:0.03717,((Ult17218:0.
08466,((Ult16477:0.04533,Ult16478:0.00356)0.955.92:0.02546,(Ult16475:0.00
749,(Ult16474:5.5E-
4,((Ult16472:0.02091,Ult16473:0.02049)0.989.82:0.04215,Ult16476:0.00998)0
.780.112:0.00494)0.795.86:0.00367)0.747.70:0.00618)0.966.114:0.04808)0.87
2.120:0.02844,((UltB9540:0.03549,Ult31347:0.10175)0.888.128:0.02543,((Ult
16897:0.0036,(Ult16902:0.01122,(((Ult16903:0.0,Ult16907:0.0):5.3E-
4,Ult16904:0.01122)0.781.87:0.0037,((Ult16899:0.0074,Ult16905:0.00369)0.5
02.10:5.4E-4,((((Ult16898:5.5E-
4,(Ult16901:0.01201,Ult16906:0.01475)0.380.12:0.00754)0.921.122:0.00285,N
cBacter:0.00785)0.875.128:0.00756,Ult16896:0.00405)0.764.99:0.00371,Ult16
900:0.02318)0.735.45:0.00335)0.805.71:0.00365)0.813.75:0.00367)0.899.112:
5.5E-
4)0.925.130:0.02416,((UltGram4:0.01989,(Ult16908:0.01128,((UltFirm9:0.008
08,(BacEnr40:0.00395,NcBacte2:0.00379)0.800.75:0.00716)0.904.120:0.01967,
(UltFirm7:0.00368,UltFirm8:5.5E-
4)0.856.99:0.01073)0.886.143:0.01931)0.825.76:0.01763)0.963.87:0.03335,(U
lt16909:0.04542,(Ult11255:0.00744,(Ult16918:0.11361,(((Ult16916:0.0265,((
UdnBac69:0.00746,((UltAquif:0.0,Ult16912:0.0,UltGram5:0.0):0.01406,(Ult16
913:0.0,Ult16914:0.0):0.00971)0.919.152:0.01421)0.745.81:0.00483,(Ult1691
5:0.00752,UltFir10:0.01134)0.793.87:0.00646)0.650.17:0.00912)0.894.121:0.
01483,UltrdLo8:0.00373)0.887.125:0.00743,Ult16917:5.3E-
4)0.907.123:0.01196)0.351.13:0.00335)0.992.97:0.05544)0.851.108:0.01889)0
.851.109:0.021)0.943.107:0.02631)0.825.77:0.01629)0.826.69:0.01123)0.774.
97:0.02182,(UltDe218:0.05971,(UltB9539:0.05411,(UltB9538:0.01405,(UltB953
6:0.03315,UltB9537:0.09003)0.557.13:0.003)0.815.71:0.0119)0.998.140:0.059
53)0.857.120:0.01826)0.272.14:0.01013,((((UltB9534:0.02671,UltB9533:0.060
4)0.798.81:0.02822,(UltB9535:0.07053,(UltB9532:0.02815,((UltB9530:0.00374
,UltB9531:0.00367)0.944.93:0.01817,(UltB9528:0.01644,UltB9529:0.02277)0.8
84.125:0.01475)0.929.109:0.02332)0.841.93:0.01508)0.742.55:0.00684)0.996.
110:0.05842,(((Ult12691:0.04935,(Ult12689:0.01068,Ult12690:0.01614)0.850.
119:0.01955)1.000.815:0.10166,(Ult12695:0.08268,(Ult12543:0.06873,(Ult125
42:0.03819,(UltVe264:0.01572,(UltVe265:0.03899,Ult12541:0.03852)0.730.46:
0.01496)0.780.113:0.00905)0.861.128:0.0196)0.945.93:0.0394)0.871.118:0.02
496)0.847.135:0.01889,((Ult12692:0.03573,Ult12693:0.07119)0.952.100:0.043
06,((Ult12694:0.00179,Ult16720:0.03298)0.930.106:0.04594,(Ult12573:0.0423
5,(Ult12571:0.0,Ult12572:0.0,Ult12574:0.0):0.01898)0.989.83:0.05469)0.966
.115:0.04825)0.684.15:0.00834)0.871.119:0.02291)0.900.103:0.02253,(((Ult1
6836:5.4E-4,(((Ult16849:5.4E-
4,(Ult16848:0.01066,UltAct22:0.04093)0.356.14:0.00471)0.990.99:0.03363,((
((UltCa101:5.5E-4,((Ult16859:0.03891,((Ult16869:5.5E-
4,(Ult16861:0.00742,(Ult16858:0.01122,((UltCa100:0.00744,(Ult16852:0.0037
1,((Ult16853:5.5E-4,Ult16855:0.00743)0.962.113:0.01124,(((Ult16856:5.3E-
4,Ult16850:0.02281)0.448.15:0.00378,Ult16854:0.00368)0.876.108:5.4E-
4,Ult16851:0.00369)0.507.11:5.5E-
4)0.816.72:0.00379)0.808.76:0.00379)0.313.8:5.4E-

4,Ult16862:0.01132)0.819.78:0.00378)0.429.15:5.4E-4)0.451.21:5.5E-
4)0.471.15:5.5E-4,Ult16860:0.00742)0.962.114:5.5E-
4)0.787.84:0.00762,((Ult16865:0.00368,Ult16866:0.00749)0.893.130:0.00749,
(Ult16864:5.5E-4,Ult16868:0.00369)0.426.22:5.4E-
4)0.826.70:0.00401)0.237.11:5.5E-4)1.000.816:6.0E-
4,(Ult12606:0.05107,Ult27570:0.05737)0.935.112:0.03419)0.939.110:0.00359,
(Ult16857:0.0037,Ult16863:5.5E-
4)0.885.110:0.00755)0.770.106:0.00569,(Ult16846:0.01111,((Ult16802:0.0,Ul
t16832:0.0,Ult16879:0.0):0.00369,(((((Ult16840:0.0,Ult16842:0.0,Ult16845:
0.0,Ult16877:0.0,Ult16882:0.0):5.5E-
4,(Ult16881:0.00369,Ult16871:0.00743)0.727.35:5.5E-4)0.991.97:5.3E-
4,Ult16841:0.00746)0.777.107:0.00369,((((Ult16867:0.0188,Ult31512:5.5E-
4)0.980.73:0.01887,(Ult16811:5.4E-
4,Ult16812:0.02706)0.945.94:0.01497)0.873.115:5.5E-
4,(Ult16837:0.00361,Ult16838:0.0077)0.955.93:0.01558)0.778.97:5.4E-
4,(((((Ult16828:0.0074,Ult16829:5.5E-
4)0.997.111:0.03986,((Ult16806:0.01502,((Ult16805:0.0,Ult16822:0.0,Ult168
44:0.0,Ult16872:0.0,Ult16873:0.0):5.5E-4,(Ult16826:0.0187,Ult16823:5.5E-
4)0.849.105:0.00367)0.772.101:5.5E-
4)0.914.132:0.00346,(Ult16827:0.00751,((((((Ult16830:0.03378,Ult16878:0.0
4394)0.629.21:0.00451,Ult16820:0.04526)0.776.96:0.00693,Ult16819:0.01459)
0.955.94:0.01998,(((Ult16815:0.0135,Ult16816:0.00975)0.673.13:0.0118,(Ult
16814:0.01172,Ult16817:0.00726)0.740.64:0.00546)0.467.12:0.00444,Ult16818
:0.00923)0.976.79:0.02608)0.662.18:0.00415,UltCan99:0.0164)0.986.84:0.030
49,(Ult16825:5.4E-
4,Ult16824:0.03555)0.857.121:0.0076)0.851.110:0.00741)1.000.817:5.4E-
4)0.790.87:0.01648)0.414.13:0.00332,Ult16831:0.01047)0.818.54:0.00863,(Ul
t16803:0.00369,Ult16847:0.00371)0.774.98:0.00356)0.796.66:0.00383,(Ult168
39:5.5E-4,((Ult16804:5.4E-
4,((((Ult16798:0.0,Ult16799:0.0,Ult16800:0.0,Ult16801:0.0,Ult16833:0.0,Ul
t16834:0.0,UltPro85:0.0,Ult16880:0.0):5.5E-4,Ult16807:5.5E-
4)0.859.109:5.5E-4,(Ult16874:0.00369,Ult16808:0.0226)0.720.30:5.5E-
4)0.998.141:5.4E-
4,((Ult16875:0.02274,((Ult16810:0.00376,Ult16809:0.0392)0.768.81:0.00367,
(UltCan98:0.0,Ult16883:0.0):5.5E-4)0.658.14:5.4E-
4)0.969.80:0.00199,Ult16821:0.01879)0.965.90:0.0019)0.824.74:0.00753)0.93
6.113:0.01136,Ult16813:0.01145)0.888.129:5.4E-4)0.689.19:5.5E-
4)0.910.114:0.00746)0.123.10:5.5E-4)0.904.121:5.4E-
4,(Ult16870:0.03339,Ult16876:0.03338)0.919.153:0.01952)0.791.77:0.00364)0
.047.7:5.4E-
4)0.943.108:0.01776)0.679.24:0.01159)0.937.113:0.02261,Ult16835:0.05876)0
.729.29:0.00401)0.996.111:0.08812,((Ult17184:0.01983,Ult17185:0.00294)0.9
86.85:0.05899,(UltDe215:0.00369,UltDe216:0.0112)0.790.88:0.01413)0.962.11
5:0.0525)0.854.105:0.03023,(((DcgTurgi:5.5E-4,(Ult32130:5.4E-
4,UltHyd29:0.06054)0.994.104:0.02665)0.981.85:0.07622,((Ult31346:0.01797,
(Ult31344:0.00716,Ult31345:0.01973)0.906.134:0.02682)0.998.142:0.13318,(U
lt31348:0.01674,(Ult31349:0.01525,Ult31350:0.01135)0.471.16:0.00726)0.933
.129:0.04396)0.750.63:0.0264)0.910.115:0.05149,((((UltThe66:0.02918,(Ult3
2222:0.00522,(BacteEM3:0.00582,Ult32221:0.00592)0.918.125:0.0175)0.956.10
7:0.03533)0.995.109:0.06554,(Ult32220:0.09004,(Ult32219:0.07399,Ult32226:
0.04002)0.921.123:0.03123)0.283.8:0.01974)0.739.49:0.01978,((UltThe67:0.0
4397,(Ult32224:0.0187,Ult32225:0.16344)0.374.19:0.00787)0.975.89:0.05324,
((Ult32216:5.4E-
4,Ult32217:0.0038)0.715.23:0.00633,((ThglsSt4:0.00369,Ult32215:5.5E-

4)0.901.122:5.4E-
4,(Ult32214:0.00387,(Ult32212:0.00739,(((Ult32208:0.0,Ult32209:0.0,Ult322
11:0.0,Ult32213:0.0):5.5E-4,Ult32210:0.02264)0.813.76:5.4E-
4,Ult32218:0.03845)0.934.99:0.01145)0.952.101:0.01552)0.711.32:0.00715)0.
834.80:0.01638)0.972.97:0.0403)0.172.9:0.02442)0.994.105:0.11134,((UltCan
91:0.17174,((Ult13155:0.00317,Ult13156:0.00813)0.000.855:5.4E-
4,((Ult13153:0.00377,(Ult13154:0.0,Ult13157:0.0):5.5E-
4)0.985.90:0.07799,(Ult13158:0.02056,Ult13159:0.03323)1.000.818:0.19804)0
.512.6:0.04398)0.978.76:0.12941)0.897.120:0.04974,((Ult13165:0.06715,UltC
an92:0.12877)0.893.131:0.04727,((Ult13163:0.00724,Ult13164:0.01947)0.950.
98:0.04491,((Ult13160:0.00369,Ult13161:5.4E-
4)0.713.32:0.01117,Ult13162:0.21218)0.632.12:0.0385)0.932.115:0.05794)0.9
52.102:0.06898)0.923.157:0.04415)0.779.90:0.02131)0.890.123:0.0271)0.896.
112:0.03006)0.738.51:0.00463)0.955.95:0.02987,((((((((UltCan65:0.01012,Ul
tCan66:0.02152)0.973.86:0.04252,((Ult12565:0.10427,UltCan67:0.06191)0.770
.107:0.01064,((UltCan64:0.0278,UltVe278:0.01622)0.965.91:0.02923,((Ult128
21:0.07307,((((UltOr199:0.03624,(Ult12841:0.04142,(Ult12839:0.03038,Ult12
840:0.0836)0.738.52:0.01674)0.890.124:0.02244)0.872.121:0.02325,(((((((Ul
t12794:0.06569,((((Ult12791:0.00759,Ult12795:0.00757)0.845.110:0.00717,(U
lt12804:0.03999,Ult12805:0.0047)0.911.142:0.01499)0.762.101:0.00387,(((((
(Ult12792:0.03085,Ult12793:0.04837)0.732.33:0.00375,Ult12796:0.01212)0.76
1.77:0.00303,(Ult12802:0.01157,Ult12809:0.00745)0.870.104:0.00775)0.879.1
32:0.00728,((((Ult12810:5.4E-4,(UltCan61:5.5E-
4,Ult12808:0.00747)0.934.100:0.00746)0.828.66:0.0037,Ult12801:5.5E-
4)0.857.122:5.5E-4,Ult12803:0.01133)0.847.136:0.00372,(UltAci42:5.4E-
4,(Ult12797:0.00374,Ult12798:0.00372)0.795.87:0.00373)0.861.129:0.00372)0
.915.119:5.3E-
4)0.777.108:0.00758,(Ult12799:0.0061,(Ult12807:0.00754,Ult12812:0.03168)0
.883.128:0.01063)0.791.78:0.00651)0.833.93:0.00704,Ult12806:0.00397)0.938
.72:0.01159)0.762.102:0.00392,Ult12800:0.00757)0.294.9:0.00382)0.267.13:0
.00454,(Ult12788:0.00362,Ult12811:0.0196)0.921.124:0.01462)0.786.93:0.008
49,(Ult12813:0.01172,((((((UltPl553:0.00389,Ult12789:0.03146)0.981.86:5.4
E-4,UltrS153:0.00748)0.336.13:5.3E-
4,Ult12790:0.00738)0.488.17:0.00896,Ult12786:0.0304)0.860.84:0.01564,Ult1
2787:0.02109)0.449.25:0.01093,Ult12785:0.01118)0.865.116:0.01268)0.631.14
:0.01171)0.929.110:0.01686,(UltCan60:0.03406,(Ult15394:0.0197,(Ult12814:0
.00743,UltGemm7:0.01159)0.991.98:0.0425)0.741.61:0.01395)0.792.99:0.00993
)0.907.124:0.02262,Ult12815:0.02974)0.992.98:0.06412,(UltrS154:0.01647,(U
lt12819:0.01052,Ult12820:0.07697)0.787.85:0.01607)0.838.96:0.01445)0.845.
111:0.0142,(Ult12817:0.00681,(Ult12816:0.02321,Ult12818:0.01669)0.816.73:
0.00784)0.910.116:0.01987)0.985.91:0.04126)0.849.106:0.01315,((Ult12837:0
.00361,UltCan63:5.4E-
4)0.994.106:0.06022,((Ult12831:0.00379,(Ult12829:0.01895,((Ult12828:0.005
56,Ult12832:0.00961)0.228.13:0.00745,Ult12830:0.02503)0.792.100:0.00964)0
.750.64:0.00448)0.898.135:0.01701,((Ult12835:0.00514,Ult12836:0.02174)0.7
40.65:0.00769,(Ult12834:0.01919,Ult12833:0.0123)0.851.111:0.00863)0.823.8
0:0.00965)0.966.116:0.03738)0.950.99:0.03297)0.399.18:0.01028,Ult12838:0.
02899)0.878.110:0.02224)0.448.16:0.03193,((Ult12826:0.03469,((UltCan62:0.
02878,(Ult12824:0.00785,(Ult12823:5.5E-
4,Ult12822:0.00369)0.984.79:0.02309)0.869.96:0.01773)0.809.81:0.02016,Ult
12825:0.03396)0.707.19:0.0122)0.351.14:0.00947,Ult12827:0.03581)0.869.97:
0.01638)0.918.126:0.02635)0.822.68:0.00826)0.838.97:0.01013)0.931.120:0.0
2453,(UltB4846:0.19273,Ult12860:0.06315)0.859.110:0.03419)0.858.113:5.5E-
4,((UltCan72:5.3E-

4,(UltCan71:0.02813,UltCan73:0.03222)0.911.143:0.01521)0.989.84:0.04779,(
(Ult12779:0.02968,Ult12778:0.05821)0.860.85:0.01789,(UltCan59:0.05283,(Ul
t12775:0.01892,(Ult12776:0.0,Ult12777:0.0):5.5E-
4)0.990.100:0.05539)0.779.91:0.00946)0.899.113:0.01953)0.958.115:0.02832)
0.753.88:0.00593,((Ult12852:0.02698,(Ult12853:5.3E-
4,UltrdLo2:0.01846)0.413.19:0.01763)0.965.92:0.04388,((UltCan69:0.02642,(
UltCan68:0.03172,(Ult12849:0.00993,Ult12850:0.02502)0.951.106:0.02715)0.2
25.7:0.00632)0.028.7:0.01109,((Ult12847:0.02692,Ult12848:0.0203)0.245.11:
0.01404,(Ult12851:0.0383,(UltCan70:0.06174,(Ult12846:0.01276,(Ult12844:0.
00793,Ult12845:0.02726)0.870.105:0.02034)0.905.118:0.01908)0.899.114:0.01
93)0.691.15:0.00405)0.632.13:0.01878)0.886.144:0.0285)0.915.120:0.03181)0
.971.76:0.03444,(((((Ult13139:0.11445,(Ult13137:0.04959,Ult13138:0.01671)
0.830.84:0.01903)0.935.113:0.04468,(Ult13133:0.03996,(Ult13135:0.00364,Ul
t13136:5.5E-
4)0.982.72:0.04254)0.738.53:0.00912)0.767.82:0.02213,Ult13134:0.03098)0.9
70.92:0.04248,((Ult12858:0.04807,Ult12859:0.05814)0.942.105:0.04783,(UltO
r289:0.17455,(Ult17396:0.16737,(Ult16895:0.09312,(Ult11229:0.11001,Ult297
05:0.34741)0.973.87:0.11145)0.710.32:0.02115)0.727.36:0.01369)0.733.45:0.
01991)0.957.101:0.04647)0.727.37:0.00386,((Ult13022:0.00215,Ult13023:0.01
318)0.984.80:0.04588,(Ult13020:0.01922,Ult13021:0.0046)0.898.136:0.02795)
0.985.92:0.05232)0.923.158:0.02075)0.755.77:0.00409,((UdnBac56:0.11269,(U
ltDe214:0.07684,(Ult12579:0.05605,(Ult12580:0.00334,Ult12581:0.02329)0.88
1.127:0.02516)0.968.89:0.04521)0.845.112:0.02223)0.945.95:0.04011,(UltFir
m5:0.02732,UltFirm6:0.03831)1.000.819:0.10681)0.798.82:0.01353)0.811.68:0
.0047,(UltPl543:0.24892,(Ult12558:0.00368,Ult12559:5.5E-
4)0.943.109:0.04879)0.868.102:0.05154)0.000.856:5.5E-
4)0.752.82:0.00402,(((Ult13200:0.09711,(Ult13198:0.03972,Ult13199:0.06315
)0.992.99:0.08058)0.220.10:0.0254,(Ult12585:0.14779,UltDe217:0.14528)0.73
6.52:0.06121)0.695.18:5.5E-
4,((Ult13143:0.0063,Ult13144:0.02094)1.000.820:0.16563,(Ult16784:0.09036,
(((Ult17206:0.03527,Ult17205:0.03648)1.000.821:0.12571,(Ult16780:0.01835,
Ult16781:0.01653)0.997.112:0.0845)0.491.11:0.0115,(Ult16779:0.06265,(Ult1
6778:0.00805,(Ult16776:0.00375,Ult16777:0.00364)0.981.87:0.05372)0.977.79
:0.05722)0.702.22:0.02964)0.576.13:0.0143)0.784.99:0.01209)0.757.69:0.005
84)0.978.77:0.03512)0.936.114:0.02235)0.828.67:0.01363)0.938.73:0.0247,((
(UltDe165:0.10616,(Ult16795:0.05567,Ult16796:0.02455)0.993.86:0.10782)0.9
74.62:0.06638,(((Ult13183:0.21618,(Ult12933:0.04348,(Ult12932:0.03862,(Ul
t12928:0.01279,(Ult12930:0.00372,(Ult12929:5.5E-
4,Ult12931:0.00369)0.652.12:5.3E-
4)0.722.31:0.00268)0.971.77:0.06034)0.301.14:0.02605)0.728.42:0.04801)0.1
06.9:0.04582,(MaiMet20:0.10842,(((((((UltMar92:5.5E-
4,((UltMar95:0.0,UltMar97:0.0,UltMar98:0.0,UltMar99:0.0):5.5E-
4,UltMar96:0.00741)0.729.30:5.5E-4)0.948.127:5.4E-
4,(UltMar91:0.00752,UltMar93:0.03026)0.773.101:0.00408)0.939.111:0.03586,
((((MaiMet17:0.03214,Ult12867:0.0277)0.563.11:0.01613,((Ult12878:0.01994,
((UltMar64:0.00368,UltMar65:0.00371)0.768.82:0.00589,((((Ult12868:0.03604
,UltSAR42:0.02587)0.678.19:0.01732,((Ult12871:0.01122,(Ult12869:0.01111,(
Ult12870:0.00402,UltB4776:0.01508)0.379.11:0.00749)0.736.53:0.00418)0.818
.55:0.00376,(MaiMet18:0.01943,UltFib64:0.00764)0.915.121:0.01152)0.570.8:
5.4E-
4)0.907.125:0.01492,(((Ult12874:0.01858,Ult12879:0.00483)0.950.100:0.0185
8,Ult12873:0.0151)0.730.47:0.00373,Ult12872:0.00751)0.778.98:0.00352)0.75
3.89:0.00552,(Ult12877:0.01449,(Ult12875:0.0116,Ult12876:0.0073)0.669.20:
0.00748)0.894.122:0.01532)0.054.2:0.00888)0.906.135:0.01651)0.875.129:0.0

1688,((Ult12883:5.4E-
4,((Ult12880:0.00373,(MaiMet19:0.0037,(Ult12881:0.0037,UltMar72:0.01127)0
.102.10:5.5E-4)0.821.72:0.00371)0.785.98:5.5E-
4,UltMar74:0.03131)0.802.75:0.00367)0.844.117:0.00957,(Ult12882:0.01187,U
ltMar73:0.07083)0.759.101:0.00555)0.974.63:0.02616)0.930.107:5.3E-
4)0.954.93:0.03143,((UltMar71:5.5E-
4,UltMar70:0.00369)0.922.162:0.01777,(UltMar66:0.01559,(UltMar67:0.0,UltM
ar68:0.0,UltMar69:0.0):5.4E-
4)0.763.109:0.005)0.535.12:0.00437)0.998.143:0.05185,UltMar94:5.3E-
4)0.989.85:0.05902)0.947.90:0.03793,(((Ult12923:0.01495,(UltSAR43:0.00742
,(MaiMet23:0.01119,MaiMet24:5.5E-4)0.812.68:0.00369)0.442.21:5.4E-
4)0.610.12:0.01412,(Ult12922:0.01507,(((Ult12918:0.0038,((UltMar85:0.0,Ul
tMar86:0.0,UltMar89:0.0,UltMar90:0.0):5.4E-
4,(UltMar87:0.05295,UltMar88:5.4E-
4)0.739.50:0.00368)0.957.102:0.01143)0.780.114:0.00364,((((Ult12921:0.014
1,(MaiMet22:5.5E-
4,Ult12920:0.00369)0.859.111:0.00955)0.970.93:0.02273,(UltDe154:0.00743,(
(UltMar83:0.0,UltMar84:0.0):5.5E-
4,(UltMar82:0.00369,UltMar81:0.00369)0.685.18:5.5E-4)0.930.108:5.5E-
4)0.806.61:5.5E-
4)0.626.15:0.00373,Ult12919:0.01917)0.773.102:0.00372,UltMar80:0.00371)0.
887.126:0.0075)0.861.130:5.5E-
4,(Ult12917:0.00817,(Ult12916:0.00449,(UltMar78:5.5E-4,UltMar79:5.5E-
4)0.875.130:0.01053)0.864.113:0.01186)0.783.110:0.01038)0.862.114:0.01128
)0.996.112:0.05251)0.214.11:0.01052,(UltDe153:0.01622,(Ult12913:5.5E-
4,Ult12915:0.00745)0.735.46:0.00271)0.994.107:0.04969)0.911.144:0.02666)0
.827.79:0.01575,((Ult12926:0.09577,(Ult12925:0.0493,(UdnBac53:0.06426,Ult
12927:0.02137)0.993.87:0.06327)0.862.115:0.02452)0.581.12:0.01482,(Ult129
11:0.00369,Ult12912:5.5E-
4)0.942.106:0.02852)0.732.34:0.00611)0.767.83:0.0175,(Ult12900:5.4E-
4,((UltMar77:0.0,UltMar76:0.0):0.00742,(MaiMet21:0.01,(((Ult12902:0.0,Ult
12904:0.0):5.5E-4,Ult12905:0.01119)0.929.111:5.5E-
4,(Ult12901:0.00371,Ult12903:0.01508)0.768.83:0.00719)0.867.124:0.00977)0
.864.114:0.01021)0.957.103:0.03534)1.000.822:0.15656)0.786.94:0.014,(Ult1
2924:0.05296,(Ult12910:0.03776,(Ult12909:0.01902,(Ult12908:0.01171,(Ult12
906:0.01498,Ult12907:0.00775)0.764.100:0.00355)0.857.123:0.01241)0.545.13
:0.02383)0.836.84:0.01824)0.719.28:0.01158)0.887.127:0.04023)0.969.81:0.0
6217)0.830.85:0.01744,(Ult12575:0.19548,(Ult13184:0.17596,Ult12583:0.1316
8)0.165.7:0.01784)0.870.106:0.0482)0.621.12:0.01253)0.953.89:0.05442,((Ul
t17209:0.01533,(Ult17210:0.02493,(Ult17207:0.02515,Ult17208:0.00929)0.832
.83:0.01191)0.032.6:0.01013)0.697.15:0.02791,(Ult17212:0.0561,Ult17213:0.
11135)0.949.114:0.04535)0.988.98:0.07179)0.705.21:0.00437)0.932.116:0.017
76,((((((UltFirm3:0.08135,(UltB5256:0.17369,((Ult31581:0.01123,Ult31582:
0.0156)0.088.9:0.00439,(Ult31583:0.06952,((((Ult31568:0.0037,UltEnd18:0.0
0747)0.915.122:0.02751,(((Ult31567:0.10165,((Ult31570:0.02132,Ult31571:0.
01008)0.860.86:0.01402,(UltrS455:5.5E-
4,Ult31572:0.04369)0.915.123:0.02064)0.681.17:0.01834)0.726.30:0.0053,(Ul
t31574:0.04264,(UltEnd19:0.00771,(UltOr485:0.01131,Ult31573:5.3E-
4)0.831.80:0.00731)0.966.117:0.03321)0.958.116:0.03785)0.745.82:0.00587,(
UltCarn3:0.02178,(Ult31565:0.01743,((Ult31562:0.01505,Ult31563:5.4E-
4)0.959.105:0.02864,(Ult31566:0.0464,Ult31564:0.02321)0.850.120:0.02618)0
.010.7:0.0172)0.864.115:0.01504)0.943.110:0.02161)0.932.117:0.02708)0.915
.124:0.03168,((Ult31577:0.01153,(Ult31575:0.00369,(Ult31576:0.0,Ult31584:
0.0):5.5E-

4)0.970.94:0.0334)0.562.15:0.0179,(Ult31578:0.23708,(UltrS456:0.02164,(Ul
t31569:0.02518,UltrS454:0.07075)0.876.109:0.02176)0.871.120:0.02296)0.756
.92:0.01259)0.876.110:0.01948)0.485.19:0.01551,(Ult31579:0.0,Ult31580:0.0
):0.04019)0.987.118:0.05976)0.537.14:0.01183)0.998.144:0.16035)0.914.133:
0.07384)0.900.104:0.05409,(UltB9526:0.09939,((UltB9527:0.08216,UltDe147:0
.06694)0.961.96:0.07539,(UltB9524:0.03078,UltB9525:0.07789)0.923.159:0.04
413)0.816.74:0.02371)0.964.107:0.06189)0.825.78:0.04478,UltB4713:0.27938)
0.703.31:0.01928,(((((Ult32028:5.5E-
4,Ult32029:0.01134)0.940.107:0.01104,(Ult32031:5.5E-
4,(Ult32030:0.00742,((Ult32032:0.0,Ult32034:0.0):0.00366,(Ult32033:5.4E-
4,(Otu00490:0.03474,(UltTer34:0.00761,Otu00219:0.00754)0.714.29:0.00395)0
.991.99:0.03467)0.998.145:0.05695)0.765.83:5.4E-
4)0.808.77:0.00367)0.845.113:5.5E-
4)0.942.107:0.01852,UltTer33:0.04448)0.407.15:0.01211,EluMinut:5.4E-
4)1.000.823:0.14087,(((((LpsSpec2:0.0,LpsSpeci:0.0,LpsInter:0.0):0.00381,
((UltB5063:0.03391,(UltB5062:0.02952,((UltB5061:5.4E-4,UltSp240:5.5E-
4)0.983.75:0.04694,(LpsBifle:0.00369,((LpsBifl2:0.0,LpsGenom:0.0,LpsBifl3
:0.0,LpsWolba:0.0):5.5E-4,LpsBifl4:5.5E-4)0.754.75:5.4E-
4)0.866.120:0.0204)0.990.101:0.05309)0.773.103:0.00831)0.895.128:0.0134,(
UltSp243:0.04791,(UltSp242:0.06831,(UltB5067:0.0309,((UltB5064:5.5E-
4,(UltB5065:0.0,UltB5066:0.0):5.5E-
4)0.971.78:0.03426,(UltB5068:0.02309,UltSp241:0.04239)0.083.4:0.0151)0.72
5.38:0.0052)0.598.8:0.00692)0.929.112:0.02723)0.821.73:0.01263)0.994.108:
0.05151)0.724.32:0.00852,((LpsBroom:0.0,LpsInada:0.0):0.028,LpsLicer:0.00
737)0.193.7:0.00396)0.988.99:0.09246,(((LpnIllin:0.05624,((UltB5071:0.005
74,(UltB5070:0.0526,UltB5069:0.02365)0.648.19:0.01112)0.984.81:0.04079,(U
ltSp244:0.07746,UltB5072:0.0312)0.883.129:0.0235)0.797.76:0.01507)0.985.9
3:0.04633,UltB5073:0.02326)0.865.117:0.0261,(UltSp245:0.0,UltSp246:0.0):0
.02959)0.901.123:0.04225)0.964.108:0.07245,(((UltB5058:0.00664,(UltB5059:
0.00347,UltB5060:0.00395)0.971.79:0.06098)0.997.113:0.08391,(((UltB5075:0
.04381,UltB5076:0.05668)0.828.68:0.04489,(UltB5077:0.1364,(TurParva:0.016
11,UltB5074:0.03215)0.981.88:0.09151)0.751.62:0.0351)0.952.103:0.04598,(U
ltOr228:0.1788,(Ult13193:0.03327,Ult13194:0.05692)0.963.88:0.10212)0.971.
80:0.07079)0.879.133:0.02323)0.908.136:0.04044,(((((UltrSo58:0.17636,(Ult
B5080:0.0,UltB5078:0.0,UltB5079:0.0,UltB5082:0.0):5.3E-
4)0.940.108:0.02268,(((UltB5088:0.0231,(UltLep15:0.01434,UltSp249:0.00458
)0.936.115:0.02325)0.963.89:0.02136,UltSp247:0.00374)0.797.77:0.00378,Ult
B5086:0.04361)0.872.122:5.5E-
4)0.744.68:0.00423,((UltB5085:0.00315,UltB5087:0.04876)0.848.99:0.00815,(
(UltSp248:0.01192,UltLep14:0.02699)0.600.13:0.00789,((UltB5083:0.00411,(U
ltLep13:0.04459,UltB5084:0.01576)0.865.118:0.01515)0.838.98:0.0089,(BacEn
r20:0.0,UltB5081:0.0):0.03604)0.837.74:0.00983)0.754.76:0.00419)0.787.86:
0.0114)0.960.101:0.0394,(UltB5091:0.0,UltB5089:0.0,UltB5090:0.0):0.05378)
0.358.10:0.01465,UltB4806:0.06834)0.970.95:0.06616)0.676.13:0.00625)0.430
.16:0.01132)0.855.106:0.02085)0.751.63:0.00705,(((Ult28896:0.04949,(Ult31
586:0.01833,(Ult31585:0.03166,((Ult31587:0.0,UltCa409:0.0):0.00518,(((Ult
31588:0.0,Ult31589:0.0):0.01644,UltCa410:0.01559)0.905.119:0.01412,Ult315
90:0.00912)0.758.90:0.00505)0.834.81:0.00733)0.621.13:5.3E-
4)0.951.107:0.03478)0.991.100:0.05761,((Ult16885:0.08493,((UltrS389:0.040
24,(Ult29497:0.04567,(Ult29495:5.4E-
4,(UltCh561:0.02367,Ult29496:0.0115)0.059.12:0.00738)0.574.10:0.01927)0.8
27.80:0.0152)0.989.86:0.07531,(Ult29491:0.02747,(Ult29492:5.5E-
4,Ult29493:0.01122)0.816.75:0.0073)0.969.82:0.05337)0.856.100:0.03116)0.9
17.108:0.03093,((CddDiv10:0.00361,Ult31425:5.5E-

4)0.997.114:0.09198,((UltCa400:0.07168,(Ult31520:0.01927,UltCa399:0.0306)
0.918.127:0.04197)0.899.115:0.02071,(((Ult31469:0.06052,(((Ult31462:0.0,U
lt31463:0.0):0.0329,Ult31466:0.01159)0.223.5:0.0102,(((Ult31465:0.0,Ult31
467:0.0,Ult31468:0.0):5.3E-
4,Ult27477:0.07455)0.809.82:0.00364,Ult31464:5.4E-
4)0.888.130:0.02308)0.920.138:0.03054)0.822.69:0.01977,(Ult31423:0.0406,(
(Ult31436:0.00326,((Ult31432:0.0,Ult31434:0.0):0.00382,Ult31424:0.05349)0
.822.70:0.01204)0.873.116:0.01298,(((((((Ult31447:0.07303,Ult31446:0.0423
4)0.938.74:0.03786,((UltCa392:0.0,UltOr484:0.0):0.02239,Ult31448:0.03794)
0.934.101:0.02662)0.847.137:0.01355,(Ult31429:0.01532,(Ult31427:5.4E-
4,Ult31428:0.03115)0.168.13:0.00736)0.762.103:0.00809)0.734.61:0.00372,((
Ult31435:0.0,Ult31437:0.0):0.01448,UltCa388:0.00593)0.298.12:0.00601)0.39
9.19:5.4E-
4,Ult31438:0.01137)0.941.98:0.01113,(((UltCa391:0.00899,Ult31439:0.05335)
0.812.69:0.0211,(Ult31444:0.02513,(Ult31442:0.00808,Ult31441:0.02295)0.43
5.13:0.00377)0.824.75:0.00948)0.913.130:5.3E-
4,(Ult31440:0.00857,((Ult31431:5.5E-4,(Ult31426:5.5E-
4,((UltCa387:0.0,Ult31430:0.0,UltCa389:0.0):5.5E-
4,Ult31461:0.00369)0.000.857:5.5E-4)0.000.858:5.5E-4)0.945.96:5.5E-
4,Ult31433:0.00371)0.947.91:0.01402)0.368.8:0.00368)0.920.139:0.00834)0.6
93.22:5.5E-
4,(UltCa390:0.0459,(Ult31443:0.02694,Ult31445:0.0179)0.889.120:0.0112)0.7
09.26:0.00262)0.904.122:0.01496)0.538.11:0.01486)0.986.86:0.06658)0.810.7
5:0.02127,(((UltCa393:0.02313,Ult31453:5.4E-
4)0.888.131:0.00743,((Ult31455:5.4E-
4,(Ult31460:0.02715,(Ult31452:0.00708,Ult31459:0.01949)0.892.118:0.01565)
0.948.128:5.4E-
4)0.806.62:0.00744,((Ult31458:0.01128,Ult31457:0.02297)0.887.128:5.4E-
4,Ult31456:0.02664)0.914.134:0.01486)0.218.9:5.4E-
4)0.779.92:0.00819,Ult31454:0.02726)0.990.102:0.07242)0.586.16:0.02108)0.
924.117:0.02824)0.621.14:0.0228)0.829.94:0.01563)0.932.118:0.04065,((((Ul
t31372:0.03723,(Ult31371:0.01426,Ult31373:0.03417)0.618.8:0.00702)0.998.1
46:0.06994,(((Ult31361:0.00744,Ult31362:5.4E-4)0.528.13:5.5E-
4,((Ult31370:0.01522,Ult31366:0.04459)0.835.91:0.01257,((((Ult31365:0.012
68,(UltrS447:0.01521,UltrS449:0.03594)0.701.25:0.00386)0.997.115:0.03178,
Ult31364:0.00226)0.882.132:0.00883,Ult31363:5.3E-
4)0.900.105:0.00809,(UltrS450:0.03583,(UltB1789:0.02275,UltEu134:0.01184)
0.456.14:0.00336)0.833.94:0.0074)0.251.8:0.00323)0.963.90:0.01586)0.889.1
21:0.01824,(Ult31368:0.0152,(Ult31367:5.4E-
4,(Ult31369:0.01157,UltrS448:0.01158)0.757.70:0.0035)0.905.120:0.01531)0.
973.88:0.02576)0.440.12:5.5E-
4)0.370.11:0.03734,(((Ult31400:0.09486,UltrS451:0.01629)0.980.74:0.05105,
(((Ult31397:0.03573,((((Ult31391:0.02069,(((Ult31381:0.00943,Ult31382:0.0
26)0.988.100:0.0337,(Ult31394:0.03907,(Ult31380:0.06144,(Ult31385:0.01333
,Ult31386:0.05048)0.890.125:0.01686)0.753.90:0.01012)0.829.95:0.01149)0.9
68.90:0.02434,(Ult31383:0.05148,Ult31393:0.03042)0.788.90:0.00495)0.721.2
0:0.00535)0.775.90:0.01308,UltOr483:0.04)0.913.131:0.01602,(Ult31378:0.02
721,(Ult31392:0.00751,(Ult31377:0.02839,(Ult31374:0.01192,UltCra13:0.0037
5)0.985.94:0.02882)0.730.48:0.00207)0.780.115:0.00376)0.777.109:0.00431)0
.146.11:5.3E-
4,(Ult31399:0.05903,(Ult31395:0.00737,Ult31396:0.00368)0.737.70:0.002)0.9
64.109:0.03208)0.139.11:0.0167)0.833.95:0.01554,((Ult31389:0.03073,(UltCa
385:0.00368,(Ult31390:0.03523,((Ult31387:0.0,UltCa384:0.0):5.4E-
4,Ult31388:0.01521)0.746.64:0.00374)0.914.135:0.01131)0.916.132:0.01764)0

.885.111:0.01203,(Ult31375:0.00746,Ult31376:0.01113)0.974.64:0.03057)0.00
0.859:5.4E-
4)0.000.860:0.01389,(Ult31384:0.00303,Ult31379:0.07089)0.454.20:0.01584)0
.977.80:0.04184)0.772.102:0.01736,Ult31398:0.00754)0.968.91:0.06143)0.295
.20:0.03814,((((Ult31547:0.01104,(((Ult31525:0.01515,Ult31528:0.03614)0.9
80.75:0.02725,Ult31527:5.3E-
4)0.855.107:0.00751,Ult31526:0.02694)0.977.81:0.02242)0.000.861:5.4E-
4,((((Ult31545:0.06065,((Ult31541:0.03806,Ult31544:0.07567)0.787.87:0.009
73,Ult31546:0.00744)0.889.122:0.02226)0.065.16:0.01089,Ult31556:0.02635)0
.897.121:0.01233,(((Ult31549:0.01516,Ult31550:0.01543)0.964.110:0.02248,U
lt31548:0.03401)0.838.99:0.01031,((Ult31555:0.00367,(Ult31551:0.00736,(Ul
t31552:5.5E-4,(Ult31553:0.01129,Ult31554:0.00366)0.754.77:5.5E-
4)0.980.76:5.5E-4)0.982.73:0.01495)0.683.29:5.4E-
4,(UltCa406:0.00943,UltCa407:0.08062)0.889.123:0.02989)0.760.94:0.00362)0
.745.83:0.00374)0.958.117:0.01484,(((Ult31538:0.01921,(Ult31539:0.01485,U
lt31540:0.01537)0.932.119:0.01559)0.961.97:0.0191,((Ult31529:0.00699,(Ult
31561:0.03144,(((Ult31542:0.0,Ult31543:0.0):5.3E-
4,UltCa405:0.01863)1.000.824:0.03516,Ult31558:0.01901)0.803.71:0.00701)0.
829.96:0.00753)0.974.65:0.02295,((Ult31533:0.04191,(Ult31534:0.02094,(Ult
Ca401:0.01764,((UltCa408:0.05318,Ult31531:0.04349)0.628.6:0.02844,Ult3153
2:0.06043)0.804.67:0.00966)0.979.91:0.03578)0.188.11:0.00702)0.928.96:0.0
1751,Ult31530:0.01415)0.890.126:0.01496)0.750.65:5.4E-
4)0.864.116:0.01112,Ult31557:0.03903)0.000.862:5.4E-
4)0.901.124:0.00728)0.996.113:5.5E-
4,(Ult31537:0.03597,((Ult31535:0.07827,(UltCa402:5.5E-
4,UltCa403:0.00366)0.909.107:0.01464)0.000.863:0.01177,(UltCa404:0.02998,
Ult31536:0.06975)0.158.11:0.01746)0.928.97:0.03631)0.978.78:0.03924)0.829
.97:0.01201,(Ult31560:0.025,(Ult31559:0.00355,UltPl567:0.0038)0.996.114:0
.06775)0.851.112:0.02308)0.997.116:0.10761)0.942.108:0.05752)0.644.17:0.0
2279)0.790.89:0.01054,(((((Ult16518:0.01645,(Ult16517:0.01889,(Ult16516:5
.5E-
4,Ult16515:0.01877)0.515.10:0.00751)0.850.121:0.01055)0.879.134:0.03038,(
(UltMa106:0.02077,Ult16514:0.00214)0.915.125:0.02702,((Ult16513:0.01115,(
UltAc870:0.00758,UltAc871:0.00985)0.779.93:0.00495)0.860.87:0.01053,(UltA
c872:0.00399,UltAc873:0.06131)0.886.145:0.01738)0.918.128:0.02263)0.908.1
37:0.03721)0.940.109:0.06289,(UltAnap2:0.06363,((NrkRisti:0.00734,(UltAna
pl:0.01177,(UltB3951:0.03128,UltB3952:5.5E-
4)0.881.128:0.01949)0.983.76:0.0333)0.431.19:0.01522,UltNeori:5.4E-
4)0.999.168:0.1163)0.961.98:0.07323)0.879.135:0.04218,(((FirBacte:5.4E-
4,((CcpSacch:0.0,UltThe44:0.0):5.5E-
4,SpdBacte:0.07639)0.906.136:0.00714)0.974.66:0.0386,((UdnAnae2:6.5E-
4,Ult32128:0.01513)0.623.12:0.00818,(TnrCellu:0.02308,(Ult25104:5.4E-
4,(((CcpOwens:0.0037,((AaoTherm:0.0,CcpKrono:0.0):0.00369,(CcpSpeci:5.5E-
4,CcpSpec2:5.5E-4)0.904.123:5.5E-4)0.524.11:5.4E-
4)0.900.106:0.00749,UdnAnaer:0.01127)0.791.79:5.4E-4,CcpAceti:5.3E-
4)0.824.76:0.00368)0.994.109:0.03976)0.435.14:0.00971)0.774.99:0.00988)0.
953.90:0.04692,((HppMarit:0.01675,(DsuKamch:0.00362,UltDes30:5.5E-
4)1.000.825:0.15423)0.933.130:0.05257,Ult17360:0.16694)0.880.130:0.03767)
0.431.20:0.03629)0.331.12:0.03071,((((Ult17333:0.13691,(((Ult17289:0.0478
1,(Ult17241:0.06598,Ult17338:0.12243)0.672.17:0.0236)0.979.92:0.0477,((((
(((UltCa137:0.03202,((Ult17317:0.08562,Ult17318:0.06541)0.916.133:0.05501
,(Ult17331:0.1483,Ult17332:0.0767)0.158.12:0.03453)0.701.26:0.02622)0.785
.99:0.01269,(((((Ult17261:0.04209,Ult17274:0.04681)0.780.116:0.01618,(Ult
De226:0.01576,(Ult17286:0.03784,(Ult17284:0.04006,Ult17283:0.04557)0.954.

94:0.03265)0.880.131:0.02246)0.764.101:0.00584)0.743.74:0.00606,((((((Ult
17301:0.05072,(UltrS303:0.03365,UltPro86:0.03681)0.923.160:0.02625)0.898.
137:0.02158,(Ult17302:0.04772,Ult17269:0.04065)0.740.66:0.0097)0.879.136:
0.01631,(Ult17268:0.0459,((Ult17296:0.04799,UltCa141:0.01041)0.934.102:0.
02042,((Ult17272:0.02676,(Ult17271:0.02112,Ult17270:0.0185)0.731.36:0.006
49)0.850.122:0.01037,Ult17273:0.01425)0.957.104:0.01977)0.384.15:5.4E-
4)0.801.81:0.00587)0.406.14:0.00136,Ult17324:0.05001)0.978.79:0.02305,(Ul
t17308:0.07059,(Ult17262:0.07243,(Ult17245:0.09121,Ult17244:0.01169)0.909
.108:0.02318)0.390.19:0.00627)0.863.133:0.01257)0.912.145:5.5E-
4,(((Ult17293:0.02406,Ult17294:0.02441)0.968.92:0.03247,(Ult17295:0.03993
,((UltCa135:0.03191,UltCa136:0.05718)0.906.137:0.02125,(Ult17264:0.06102,
Ult17263:0.04064)0.739.51:0.00617)0.920.140:0.01902)0.579.16:0.01486)0.71
2.23:0.00699,(((UltDe223:0.04444,(Ult17253:0.07045,Ult17254:0.0565)0.938.
75:0.03487)0.825.79:0.01301,((Ult17242:0.05412,(Ult17356:0.04976,((UltCa1
32:0.05018,(Ult17246:0.04264,Ult17247:0.05964)0.678.20:0.00544)0.796.67:0
.0127,UltrS302:0.07022)0.475.14:0.00574)0.731.37:0.01651)0.839.98:0.01293
,((UltCa133:0.05133,(Ult17250:0.06213,(UltCa131:0.0572,((Ult17239:0.02728
,(Ult17240:0.01738,Ult17237:0.05115)0.720.31:0.00976)0.243.14:5.4E-
4,(Ult17236:0.01143,Ult17238:0.01133)0.875.131:0.01108)0.753.91:0.00846)0
.904.124:0.02395)0.654.12:0.0217)0.649.14:0.02556,(Ult17243:0.03315,Ult17
248:0.07072)0.833.96:0.0242)0.958.118:5.4E-
4)0.894.123:0.01707)0.826.71:0.00815,(UltOr275:0.03719,(Ult17252:0.12651,
(Ult17249:0.05404,Ult17251:0.04801)0.845.114:0.0188)0.668.20:0.00585)0.89
9.116:0.02106)0.885.112:0.01454)0.923.161:0.01467)0.750.66:0.01634)0.931.
121:0.01737,((Ult17306:0.09827,Ult17307:0.03619)0.944.94:0.03453,((UltCa1
42:0.04919,UltCa143:0.05611)0.895.129:0.02628,(((Ult17315:0.05831,((Ult17
313:0.01201,Ult17314:0.003)0.993.88:0.04826,(UltrS304:0.00145,(Ult17311:0
.01416,Ult17312:0.02669)0.520.9:0.0127)0.673.14:0.01439)0.814.65:0.01386)
0.641.13:0.01139,UltVe279:0.04324)0.955.96:0.03425,((Ult17256:0.02607,((U
lt17255:0.01632,UltCa134:0.0188)0.974.67:0.0508,Ult17257:0.03246)0.372.15
:0.01627)0.934.103:0.04046,Ult17304:0.0952)0.835.92:0.01865)0.826.72:0.01
327)0.754.78:0.00821)0.856.101:0.01483)0.321.10:0.0115,(((Ult17265:0.0648
5,Ult17354:0.02851)0.918.129:0.03696,(Ult17266:0.04438,(Ult17258:5.3E-
4,(Ult17259:0.02719,Ult17260:0.00727)0.663.20:0.00762)0.998.147:0.05571)0
.392.13:0.00599)0.959.106:0.03635,(Ult17277:0.02931,(((Ult17305:0.06589,U
lt17309:0.08997)0.759.102:0.01437,VrcBact6:0.0698)0.410.14:0.01885,Ult173
16:0.09566)0.747.71:0.00964)0.791.80:0.01383)0.676.14:0.02691)0.060.4:5.4
E-4)0.803.72:0.00759,((UltCa140:0.01955,((UltDe224:0.02316,UltDe225:5.5E-
4)0.975.90:5.3E-
4,(Ult17291:0.01076,Ult17292:0.03243)0.828.69:0.00385)0.940.110:0.02565)0
.993.89:0.04464,(((((Ult17278:0.0333,(Ult17282:0.04774,Ult17281:0.02866)0
.809.83:0.01387)0.875.132:0.01419,(UltCa138:0.01334,(DetProt3:0.02118,Ult
17279:0.03417)0.934.104:0.01973)0.807.66:0.01037)0.823.81:0.01947,(UdnBac
70:0.11004,Ult17310:0.00261)0.773.104:0.00622)0.094.5:0.0105,Ult17303:0.0
4786)0.883.130:0.0114,(((Ult17299:0.01568,(Ult17300:0.0343,(Ult17297:0.00
369,Ult17298:5.5E-
4)0.961.99:0.02448)0.260.11:0.0139)0.928.98:0.0249,Ult17288:0.0907)0.742.
56:0.02567,(Ult17287:0.04427,(((Ult17322:0.01554,(Ult17321:0.01993,UltCa1
45:0.0579)0.930.109:0.01977)0.872.123:0.01208,(Ult17320:0.02736,UltCa144:
0.00372)0.957.105:0.02367)0.838.100:0.00979,(UltCa139:0.07166,(UltOr276:0
.03659,(Ult17280:0.05414,(UltAc877:0.02433,(Ult17275:0.00746,Ult17276:0.0
0757)0.766.87:0.00732)0.969.83:0.03417)0.731.38:0.00758)0.648.20:0.01159)
0.870.107:0.01929)0.575.9:0.00635)0.560.10:0.00995)0.858.114:0.01005)0.80
2.76:5.5E-

4)0.923.162:0.01619)0.353.9:0.00982,(Ult17323:0.05044,Ult17319:0.06213)0.
938.76:0.02936)0.798.83:0.01562,(Ult17285:0.00331,Ult17355:0.00407)0.996.
115:0.04894)0.726.31:0.00754,Ult17267:0.05295)0.853.90:0.02011,Ult17290:0
.05705)0.913.132:0.03714)0.759.103:0.01768,((Ult17340:0.07401,(((Ult17337
:0.02836,((((Ult17351:0.08628,(Ult17352:0.08713,UltCa151:0.04494)0.000.86
4:0.01526)0.734.62:0.0408,((Ult17350:0.03101,UltCa150:0.01258)0.995.110:0
.08284,(UltCa149:0.06701,(((Ult17346:0.0,Ult17347:0.0):0.03256,((Ult17344
:0.07401,Ult17348:0.08357)0.183.13:0.02792,Ult17345:0.07036)0.134.13:0.01
507)0.595.8:0.01356,Ult17349:0.08521)0.871.121:0.03097)0.889.124:0.03227)
0.908.138:0.0462)0.789.83:0.0152,(Ult17334:0.09155,Ult17343:0.06503)0.914
.136:0.03008)0.417.18:0.00784,(Ult17336:0.03945,Ult17335:0.06058)0.880.13
2:0.02983)0.934.105:0.03095)0.718.30:0.00383,(Ult17330:0.02648,((UltCa147
:0.02108,(UltCa146:0.01598,((Ult17326:0.01494,Ult17327:5.4E-
4)0.891.102:0.01302,Ult17325:0.01382)0.087.9:0.00442)0.656.17:0.01198)0.7
81.88:0.026,(Ult17328:0.02241,Ult17329:0.05681)0.533.11:0.00888)0.866.121
:0.02047)0.937.114:0.02788)0.727.38:0.02296,(Ult17342:0.04198,(Ult17341:0
.08217,UltCa148:0.08594)0.241.10:0.02267)0.905.121:0.03963)0.647.9:0.0174
6)0.917.109:0.02531,(Ult17339:0.08476,Ult17353:0.11271)0.720.32:0.01453)0
.727.39:0.02381)0.826.73:0.032)0.990.103:0.07039,(Ult32306:0.1808,(((UltB
4755:0.004,UltB4756:0.00344)0.986.87:0.05928,((UltB4757:0.04241,UltCand2:
0.04235)0.940.111:0.0306,((UltB4775:0.0299,(UltB4762:0.01439,(UltB4761:0.
03326,UltB4758:0.06367)0.754.79:0.00926)0.983.77:0.03355)0.288.10:0.00289
,((((UltB4763:0.0186,(UltB4759:0.00747,UltB4760:5.5E-
4)0.932.120:0.01696)0.740.67:0.00295,(UltB4773:0.00821,UltB4774:0.01546)0
.982.74:0.027)0.865.119:0.00792,((UltCand3:0.03489,((UltB4764:0.0,UltB476
5:0.0):0.04838,UltB4770:0.03017)0.672.18:0.01015)0.746.65:0.00491,((UltB4
766:0.05615,UltB4768:0.02581)0.884.126:0.01722,(UltB4767:0.02395,(UltB477
2:0.00726,UltB4771:0.01168)0.882.133:0.01207)0.769.96:0.01164)0.726.32:0.
00422)0.881.129:0.01049)0.793.88:0.0082,UltB4769:0.0281)0.903.110:0.01352
)0.750.67:0.00776)0.684.16:0.00394)0.974.68:0.09132,((((Ult32303:0.02802,
Ult32304:0.04601)0.979.93:0.07165,Ult32305:0.09618)0.831.81:0.02669,(Ult3
2307:0.19624,UltCa416:0.15758)0.693.23:0.03125)0.977.82:0.09918,(Ult32302
:0.07716,((Ult32300:0.07307,((Ult32298:0.01435,(UltOr492:0.00744,Ult32299
:0.0037)0.748.73:0.00496)0.933.131:0.02972,((Ult32294:5.5E-
4,UltrdKB1:0.01123)0.924.118:0.03079,(Ult32296:0.01025,(Ult32295:0.00431,
Ult32297:0.00312)0.247.10:0.00613)0.900.107:0.02475)0.827.81:0.02454)0.73
6.54:0.01562)0.920.141:0.0179,Ult32301:0.03965)0.680.21:0.01223)0.988.101
:0.15101)0.330.13:0.0517)0.933.132:0.06038)0.922.163:0.03448)0.860.88:0.0
1871,((Ult30162:0.13879,((VlcMedia:0.07007,(((MnhHydro:0.04792,((Ult31341
:0.00528,Ult31342:0.00216)0.985.95:0.05914,(UltDei15:0.03164,((MeeChlia:0
.01092,(((((Ult31336:0.01122,Ult31337:5.3E-
4)0.914.137:0.00745,Ult31335:0.01118)1.000.826:5.4E-
4,(((MeeSpec2:0.00743,TmuSpe18:0.00391)0.495.14:5.5E-4,MeeCerbe:5.5E-
4)0.004.9:5.5E-4,(Ult31338:5.5E-4,(MeeTaiwa:0.04599,MeeSpeci:5.5E-
4)0.886.146:5.4E-
4)0.858.115:0.00392)0.882.134:0.00349)0.325.13:0.00771,(UltMeiot:0.04758,
((UltDei14:0.04708,(UltThe33:5.5E-4,Ult31339:0.03093)0.736.55:8.3E-
4)0.969.84:0.02184,UltThe34:0.01198)0.787.88:0.00711)0.938.77:0.01669)0.9
54.95:0.02537,(MeeTimid:0.00915,UltMeio2:0.00205)0.783.111:0.01123)0.829.
98:0.01477)0.985.96:0.04832,(MeeSilva:0.01862,Ult31340:0.02678)0.958.119:
0.03473)0.899.117:0.02309)0.693.24:0.02051)0.943.111:0.02826)0.370.12:0.0
1766,(UltProk8:0.03415,(Ult31333:5.5E-4,Ult31334:0.00371)0.861.131:5.4E-
4)0.946.103:0.03348)0.926.139:0.03443,(TpcBacte:0.05727,(TmuOshim:0.01815
,(((TmuTherm:5.5E-4,(((TmuKawar:0.00371,((TmuSpe12:5.4E-

4,(TmuSpe11:0.00742,(((Ult31326:0.0,TmuSpec9:0.0):5.5E-
4,(Ult31327:0.00369,Ult31325:0.0074)0.742.57:5.5E-4)0.954.96:5.4E-
4,((TmuBrock:0.0,TmuSpe10:0.0):0.00373,TmuSpe16:0.0037)0.918.130:0.00747)
0.837.75:0.00744)0.933.133:5.4E-4)0.832.84:0.00369,Ult31328:5.4E-
4)0.928.99:0.01133)0.866.122:0.00749,TmuSpe17:5.4E-
4)0.767.84:0.00404,((TmuSpe13:0.00372,(Ult31330:0.00749,((((((TmuScot2:0.
00266,TmuSpec8:0.04852)0.937.115:0.01617,(TmuScot3:0.00369,(TmuSpec5:5.5E
-4,(((TmuSpec6:0.00194,TmuSpec7:0.03495)0.992.100:0.00184,UltThe26:5.5E-
4)0.000.865:5.5E-4,((TmuScoto:0.0037,Ult31323:0.00741)1.000.827:5.5E-
4,(UltThe29:0.00369,(TmuSpec3:5.5E-
4,Ult31324:0.00742)0.838.101:0.00368)0.615.16:5.5E-4)0.000.866:5.5E-
4)0.709.27:5.5E-4)0.773.105:5.5E-4)0.847.138:0.00368)0.012.4:5.5E-
4,(((TmuSpeci:0.00366,(TmuSpec2:5.5E-
4,UltThe25:0.00369)0.779.94:0.00377)0.918.131:0.01174,(TmuYunna:0.01499,T
muSpec4:5.5E-
4)0.337.8:0.00727)0.016.4:0.00372,TmuAntra:0.01099)0.881.130:5.3E-
4)0.957.106:0.00738,(UltEu133:0.00743,(Ult31332:5.5E-
4,UltThe27:0.00369)0.773.106:5.5E-4)0.847.139:0.00369)0.801.82:5.5E-
4,TmuSpe15:5.5E-4)0.307.7:5.4E-
4,((Ult31331:0.0,UltThe31:0.0):0.0037,((((UltThe32:0.0,UltThe82:0.0):5.5E
-4,(Ult32263:0.02651,TmuSpe14:0.00368)0.785.100:5.5E-4)0.000.867:5.5E-
4,(Ult32274:0.0037,UltThe30:0.0074)0.853.91:5.5E-4)0.885.113:5.5E-
4,Ult32273:0.00364)0.402.12:5.5E-
4)0.920.142:0.00736)0.764.102:0.00368)0.811.69:0.00362)1.000.828:5.5E-
4,(Ult31329:0.00742,UltThe28:0.00751)0.899.118:0.00329)0.523.9:0.00745)0.
848.100:0.00711)0.712.24:0.0035,TmuTher2:0.02425)0.972.98:0.03512,(UltArc
h3:0.09689,TmuFilif:0.00363)0.766.88:0.01939)0.858.116:0.03308)0.978.80:0
.05612)0.853.92:0.01543)0.535.13:0.00157)0.925.131:0.03954,(Ult31301:0.12
758,(((((UltThe23:0.00805,(MaiMet39:0.00968,UltDei13:0.0253)0.993.90:0.03
875)0.771.115:0.00313,((Ult31312:0.03057,(Ult31322:0.1027,Ult31313:0.0046
7)0.524.12:0.01963)0.816.76:0.01209,((Ult31307:0.0182,((((Ult31310:0.0074
4,(UltEnd17:5.5E-
4,(Ult31306:0.00369,(UltDei12:0.0,Ult31309:0.0):0.00371)0.729.31:5.5E-
4)0.568.14:5.4E-4)0.890.127:7.5E-
4,UltDei11:0.06962)0.999.169:0.00277,Ult31305:0.00391)0.982.75:0.01919,Ul
t31308:5.3E-
4)0.789.84:0.00107)0.770.108:0.00976,((UltThe24:0.01104,Ult31321:5.4E-
4)1.000.829:0.08632,(TueRadio:5.5E-
4,Ult31311:0.01532)0.841.94:0.00759)0.226.10:0.00536)0.998.148:0.0485)0.9
76.80:0.02618)0.836.85:5.3E-
4,((Ult31318:0.01783,Ult31319:0.01716)0.018.2:0.00763,((Ult31315:0.009,Ul
t31316:0.00983)0.973.89:0.02457,(Ult31314:0.00948,Ult31317:0.00545)0.916.
134:0.01633)0.626.16:0.01145)0.865.120:0.01319)0.645.14:0.00277,Ult31320:
0.02831)0.997.117:0.06379,(((Ult31302:0.0,Ult31303:0.0):0.03979,(Ult31304
:0.04863,UltThe22:0.04424)0.499.12:0.01985)0.932.121:0.02653,(((DioPapag:
0.00368,Ult31300:5.4E-
4)0.989.87:0.02696,(((DioPimen:0.04673,(((Ult31293:0.07139,(UltDein9:0.00
182,(Ult31298:0.00632,(DioYavap:0.02397,Ult31299:0.01344)0.862.116:0.0249
9)0.935.114:0.03221)0.968.93:0.04908)0.698.28:0.02685,DioGeoth:0.00704)0.
806.63:0.00981,(((((((((Ult31276:0.01775,Ult31283:0.01779)0.572.11:0.003
66,(Ult31294:0.02299,(Ult31275:0.01241,(DioNavaj:0.01547,Ult31277:0.01943
)0.884.127:0.01408)0.873.117:0.01193)0.932.122:0.01894)0.583.13:0.00393,(
(UltDein3:0.02324,(((Ult31296:0.0147,(DioRadi3:0.02241,DioSpe13:0.02945)0
.185.9:0.00693)0.931.122:0.02665,DioPisci:0.00471)0.941.99:0.01905,DioPro

te:0.01494)0.861.132:0.01195)0.765.84:0.00444,(Ult31292:0.01968,(DioSpec8
:0.0238,((((Ult31279:0.02656,((DioGrand:0.00369,((DioSpec4:0.0,Ult31280:0
.0):5.5E-4,DioSpec5:5.5E-4)0.913.133:5.5E-4)0.909.109:5.5E-
4,(DioYunwe:0.0142,DioSpec2:0.01795)0.855.108:0.01392)0.955.97:5.5E-
4)0.865.121:0.01365,(DioSonor:0.03945,((DioFrige:0.00527,DioMarmo:0.01059
)0.855.109:0.016,((DioAquat:0.00368,(Ult31271:5.5E-
4,DioRadio:0.00369)0.333.7:5.5E-4)0.770.109:0.0037,(DioSpeci:5.3E-
4,UltDeino:0.0037)0.917.110:0.00758)0.688.23:0.0033)0.960.102:0.02624)0.8
32.85:0.01333)0.876.111:0.01515,(DioDeser:0.01884,(Ult31281:5.5E-
4,((DioHohok:0.0,DioSpec6:0.0,DioAqua3:0.0):5.5E-
4,DioSpec7:0.01127)0.734.63:5.5E-4)0.888.132:5.3E-4)0.336.14:5.4E-
4)0.874.142:0.01112,DioIndic:0.01879)0.941.100:0.01708)0.830.86:0.01049)0
.906.138:0.01656)0.825.80:0.00896)0.941.101:0.02001,(DioRadi2:0.0085,Ult3
1282:0.01432)0.764.103:0.004)0.748.74:0.00324,(UltDein4:0.01024,DioFicus:
0.01859)0.760.95:0.00534)0.863.134:0.01132,(((((DioAltit:5.5E-
4,(DioAquir:5.5E-4,DioSpe14:5.5E-
4)0.917.111:0.0148)0.901.125:0.01538,DioClaud:0.00754)0.822.71:0.00665,(D
ioRadi4:0.00368,BacEll28:0.00755)0.943.112:0.00997)0.377.15:0.01537,(UltD
ein7:0.03113,Ult31297:0.01827)0.885.114:0.02076)0.969.85:0.02792,(DioAqua
2:0.02445,Ult31278:0.03371)0.823.82:0.00942)0.918.132:0.01595)0.801.83:0.
00953,(DioGobie:0.00457,DioSpe12:0.01044)0.866.123:0.00842)0.828.70:0.012
78,(DioRetic:0.0324,(Ult31290:0.04537,(Ult31287:0.00895,(Ult31288:0.03119
,(((Ult31285:0.00347,Ult31289:0.0078)0.938.78:0.01528,Ult31286:0.00549)0.
599.13:0.01014,(Ult31284:5.4E-4,DioAethe:0.02711)0.330.14:5.4E-
4)0.641.14:0.00497)0.892.119:0.01347)0.890.128:0.02191)0.964.111:0.03916)
0.681.18:0.0077)0.296.10:0.00819,(UltDein2:0.00511,DioSpec3:0.0232)0.977.
83:0.03849)0.936.116:0.02852,(((((UltDein6:0.00628,(DioSpe11:0.02883,(Ult
31291:0.01229,(DioApach:0.0315,DioAeriu:0.00396)0.034.9:0.00379)0.934.106
:0.01875)0.842.107:0.01033)0.943.113:0.01866,((UltEnd15:0.0,UltEnd16:0.0)
:0.03324,((Ult31274:5.4E-4,(Ult31273:5.3E-
4,Ult31272:0.0113)0.823.83:0.00369)1.000.830:0.02735,DioHopie:5.5E-
4)0.742.58:0.00242)0.956.108:0.01711)0.736.56:0.00406,(DioSpe10:0.0037,Ul
tDein5:5.5E-
4)0.774.100:0.0035)0.918.133:0.0222,DioMurra:0.01044)0.873.118:0.01967,Di
oSpec9:0.0209)0.899.119:0.02895)0.106.10:5.4E-
4)0.878.111:0.0192)0.745.84:0.00893,(DioMisas:5.5E-
4,DioRoseu:0.00367)0.998.149:0.06582)0.836.86:0.01443,((DioSpe17:0.02396,
(DioSpe15:0.01125,(UltDein8:0.00369,DioMaric:5.5E-4)0.945.97:5.5E-
4)0.997.118:0.05057)0.828.71:0.01126,UltDei10:0.06397)0.781.89:0.00846)0.
699.28:0.00417)0.838.102:0.01736,((DioSpe18:0.08456,(DioPerar:5.4E-
4,(Ult31295:0.01829,DioAlpin:0.02191)0.997.119:0.04539)0.952.104:0.03483)
0.645.15:0.02516,DioSpe16:0.00423)0.898.138:0.02666)0.974.69:0.04071)0.86
0.89:0.02341)0.659.17:0.05069)0.770.110:0.01632)0.944.95:0.04711)0.893.13
2:0.04283,(Ult13196:0.19892,CddDiv12:0.15656)0.813.77:0.04346)0.883.131:0
.04236)0.880.133:0.02468,(((Ult32101:5.4E-
4,(Ult32100:0.01557,((Ult32102:0.00366,UltCal27:0.00378)0.998.150:0.0359,
UltCal26:0.00698)0.780.117:5.5E-
4)0.408.12:0.01622)0.999.170:0.15105,((Ult32097:0.06931,(Ult32094:0.01461
,(Ult32095:5.5E-4,Ult32096:0.01116)0.881.131:5.4E-
4)0.853.93:0.01781)0.866.124:0.01527,(CduExile:0.0307,(UltCal25:0.03391,(
Ult32098:0.01764,Ult32099:0.04825)0.975.91:0.04368)0.991.101:0.06567)0.53
3.12:0.00874)0.945.98:0.06626)0.976.81:0.08861,((((Ult29488:0.00719,((Ult
Ac942:0.00364,((UltrS388:0.0,Ult29482:0.0,Ult29484:0.0):5.5E-
4,Ult29483:0.00743)0.921.125:0.00725)0.924.119:5.4E-

4,((((Ult29478:0.00367,Ult29479:0.00735)0.858.117:5.4E-
4,(((Ult29467:0.03056,Ult29461:0.00227)0.750.68:0.00149,((((((Ult29470:0.
01028,(Ult29463:0.00375,Ult29489:0.04655)0.763.110:0.00364)0.883.132:0.00
437,Ult29485:0.0074)0.914.138:5.4E-
4,(Ult29464:0.0,Ult29468:0.0,Ult29469:0.0):5.4E-
4)0.977.84:0.01864,Ult29487:0.00704)0.902.126:5.4E-
4,((Ult29462:0.00366,Ult29486:5.5E-
4)0.774.101:0.00367,Ult29465:0.00994)0.796.68:0.00478)0.654.13:5.5E-
4,(Ult29472:5.5E-
4,(Ult29458:0.05168,Ult29466:0.01484)0.858.118:0.00745)0.588.13:5.4E-
4)0.928.100:5.4E-4)0.900.108:0.00771,(Ult29471:5.5E-4,(Ult29459:5.4E-
4,Ult29460:0.00707)0.420.18:0.00704)0.753.92:0.00321)0.771.116:0.00742)0.
898.139:0.00915,Ult29481:0.00911)0.243.15:0.00345,(Ult29473:0.10527,Ult29
480:0.01262)0.654.14:0.00437)0.237.12:0.00958)0.867.125:0.00755)0.938.79:
0.02446,((UltCh559:0.00356,(Ult29477:0.03897,UltCh560:0.00763)0.782.113:0
.00352)0.881.132:0.00887,(UltOr390:0.10673,((Ult29474:0.00778,Ult29475:5.
5E-
4)0.846.122:0.01087,(UltThe21:0.0418,(UltFi101:0.00673,Ult29476:0.03571)0
.903.111:0.02015)0.773.107:0.00878)0.885.115:0.01382)0.789.85:0.00697)0.8
35.93:0.0089)0.923.163:0.03589,(Ult24036:0.13589,(Ult23384:0.29823,(Ult24
399:0.11032,Ult24400:0.07112)0.620.13:0.03524)0.965.93:0.08682)0.883.133:
0.0409)0.960.103:0.05586,((((Ult28893:0.064,(Ult28892:0.04338,(Ult28894:0
.0,Ult28895:0.0):5.4E-
4)0.775.91:0.0126)1.000.831:0.169,((Ult28538:0.05636,((UltCh341:0.03435,(
UltB4010:0.09733,(((Ult28521:0.00272,Ult28522:0.00114)0.982.76:0.03563,((
((((((Ult28525:5.5E-
4,Ult28526:0.00741)0.966.118:0.01894,(Ult28518:0.00739,Ult28519:5.5E-
4)0.940.112:0.0186)0.754.80:0.00776,(Ult28530:0.01762,(Ult28535:0.03785,U
ltCh342:0.07755)0.852.110:0.01896)0.872.124:0.01504)0.874.143:0.00863,(Ul
t28523:0.02325,(Ult28524:5.5E-
4,Ult28540:0.00742)0.987.119:0.02877)0.723.37:0.00332)0.760.96:0.00366,Ul
tSlu42:0.03179)0.882.135:0.01583,Ult28517:0.03984)0.781.90:0.00496,(Ult28
520:0.02126,Ult28527:0.04406)0.261.8:0.01491)0.765.85:5.5E-
4,((((Ult28531:0.00792,(Ult28532:0.00368,Ult28533:5.5E-
4)1.000.832:0.04416)0.730.49:0.00226,(Ult28534:0.00401,UltPl561:0.01921)0
.892.120:0.00857)0.896.113:0.0125,Ult28536:0.02888)0.887.129:0.01463,Ult2
8528:0.00772)0.867.126:0.00864)0.926.140:0.01676)0.110.8:0.00304,(UltrS36
1:0.01356,Ult28529:0.01945)0.757.71:0.00573)0.697.16:0.00387)0.124.14:0.0
1942)0.985.97:0.08037,Ult28537:0.03656)0.937.116:0.04951)0.902.127:0.0390
9,(((Ult28543:0.05316,(Ult28541:0.04191,(UltCh343:5.5E-
4,Ult28544:0.02257)0.834.82:0.01344)0.310.11:0.00664)0.948.129:0.02016,Ul
t28542:0.01542)0.834.83:0.01059,UltCh344:0.02467)0.719.29:0.01253)0.921.1
26:0.0414)0.839.99:0.025,(((((Ult28612:0.08588,(((((((Ult28237:0.0285,Ult
28238:0.0416)0.909.110:0.02824,(((Ult28509:0.00774,Ult28510:0.01552)0.713
.33:0.00424,(Ult28514:0.03157,Ult28515:0.01029)0.928.101:0.02507)0.930.11
0:0.01981,((Ult28512:0.0037,Ult28513:5.4E-
4)0.866.125:0.01306,Ult28511:0.04748)0.705.22:0.00896)0.998.151:0.07164)0
.810.76:0.02279,(Ult28245:0.03673,(UltCh340:0.03418,Ult28516:0.04462)0.93
3.134:0.03176)0.000.868:0.00562)0.868.103:0.02069,(((Ult28235:0.00414,(Ul
t28234:0.01549,Ult28236:0.06165)0.724.33:0.00722)0.959.107:0.02993,((((((
UltCh339:0.06124,(Ult28503:0.01849,(Ult28505:0.01197,(Ult28504:5.4E-
4,(UltrS359:0.0116,UltrS360:0.00388)0.635.10:0.01223)0.928.102:0.0154)0.7
35.47:0.00433)0.813.78:0.00983)0.772.103:0.00812,(Ult28502:0.00691,(UltCh
337:0.00704,UltCh338:0.01999)0.959.108:0.03028)0.874.144:0.01416)0.678.21

:0.01535,(Ult28508:0.05368,(((Ult28495:0.03783,Ult28496:0.00215)0.936.117
:0.0199,((Ult28493:0.00997,(Ult28491:0.0219,Ult28492:0.00135)0.965.94:0.0
213)0.947.92:0.02125,((Ult28487:0.02422,(Ult28498:0.04075,(Ult28488:0.019
4,Ult28497:0.00377)0.893.133:0.02011)0.049.2:0.01905)0.852.111:5.4E-
4,((UltEu115:5.5E-
4,Ult28490:0.015)0.993.91:0.03553,Ult28489:0.01474)0.640.16:0.01252)0.657
.19:0.01285)0.732.35:0.00398)0.751.64:0.00595,(Ult28501:0.01792,(Ult28494
:0.08485,(UltCh336:0.0261,(Ult28499:0.00738,Ult28500:5.5E-
4)0.991.102:0.04927)0.830.87:0.01298)0.791.81:0.01219)0.789.86:0.00853)0.
889.125:0.01484)0.940.113:0.03325)0.644.18:0.01673,(Ult28506:0.05172,Ult2
8507:0.01781)0.940.114:0.03803)0.997.120:0.08866,(Ult29001:0.03883,(Ult29
002:0.00399,Ult29003:0.0331)0.856.102:0.03082)0.990.104:0.08166)0.719.30:
0.02023,(((((Ult28177:0.01176,Ult28179:0.02296)0.734.64:0.00392,(Ult28178
:0.01164,UltCh236:0.03631)0.949.115:0.01975)0.871.122:0.00774,(((((((Ult2
8194:0.00375,Ult28195:0.01522)0.871.123:5.5E-
4,(Ult28182:0.00756,((Ult28176:0.02348,(Ult28192:0.012,Ult28193:0.01928)0
.739.52:0.00336)0.500.17:0.00792,UltCh247:0.01146)0.745.85:0.0036)0.475.1
5:0.00373)0.844.118:0.0071,(((((((UltCh241:0.00371,((UltCh253:0.00371,((U
ltOr367:5.5E-4,(UltOr366:0.01879,UltCh570:0.01126)0.882.136:5.3E-
4)1.000.833:5.4E-4,UltCh245:0.03074)0.832.86:0.00371)0.312.12:5.5E-
4,UltCh244:0.00371)0.000.869:5.5E-4)0.568.15:5.5E-4,UltCh243:5.5E-
4)0.582.13:5.5E-4,UltCh240:5.5E-4)0.982.77:5.5E-
4,UltCh242:0.01502)0.981.89:0.02347,(Ult28191:0.02751,(UltCh246:0.01513,U
ltOr372:5.5E-
4)0.765.86:0.00359)0.986.88:0.02379)0.898.140:0.01117,((UltCh249:0.00369,
(((UltOr369:0.0,UltOr370:0.0):5.4E-
4,UltOr371:0.01887)0.998.152:0.03605,(UdnGree2:0.00373,Ult28186:0.00758)0
.975.92:0.02784)0.878.112:0.01119)0.286.13:5.4E-
4,UltCh250:0.00371)0.830.88:0.00374)0.919.154:5.5E-
4,((UltCh238:0.008,((Ult28196:0.02489,((UltCh255:0.04656,UltCh239:0.02341
)0.733.46:0.00553,UltOr365:0.05139)0.516.8:0.0084)0.871.124:0.01599,((Ult
28184:0.01142,(Ult28183:5.5E-
4,Ult28185:0.00371)0.769.97:0.00366)0.979.94:0.02281,((Ult28181:0.00763,(
UltOr364:5.5E-
4,UltCh237:0.02302)0.348.14:0.00372)0.945.99:0.01842,(Ult28180:0.0158,(Ul
tOr359:0.04377,((UltOr358:5.4E-
4,UltOr361:0.00752)0.779.95:0.00415,(((UltOr362:0.0,UltOr363:0.0):0.00817
,UltOr368:0.02248)0.403.15:0.00789,(UltOr357:5.5E-
4,UltOr360:0.00372)0.760.97:0.00404)0.789.87:0.0069)0.779.96:0.00404)0.50
2.11:0.00369)0.875.133:5.5E-
4)0.814.66:0.00897)0.586.17:0.00733)0.748.75:0.00473)0.449.26:0.00446,Ult
Ch248:0.01995)0.439.15:0.01477)0.206.9:0.00785)0.818.56:0.01203,((UltCh25
1:0.00745,(UltCh252:5.5E-4,UltCh254:5.5E-4)0.668.21:5.4E-
4)0.911.145:0.01118,Ult28175:0.05667)0.502.12:5.4E-
4)0.865.122:0.00748,(Ult28188:0.0,Ult28189:0.0):0.00374)0.908.139:5.5E-
4,(Ult28190:0.0037,Ult28187:0.01918)0.854.106:0.01094)0.837.76:0.01456,Ul
tCh235:0.00965)0.909.111:0.01417)0.874.145:0.00827,(((((UltCh569:0.05663,
(UltCh256:5.5E-4,UltOr373:0.00371)0.876.112:5.5E-
4)0.269.11:0.00381,UltCh257:0.0034)0.962.116:0.0268,(UltCh258:0.03137,(Ul
tCh262:0.03622,(UltCh260:0.03971,(UltCh259:0.01139,UltCh261:0.01914)0.879
.137:5.4E-4)0.846.123:0.01129)0.199.7:5.4E-
4)0.662.19:0.01355)0.868.104:0.01331,UltOr374:0.00977)0.922.164:0.01411,U
lt28197:0.03528)0.733.47:0.0038)0.968.94:0.02704,((((((UltCh119:0.08372,(
Ult27887:0.0,UltCh118:0.0):0.00953)0.986.89:0.04165,((Ult27881:0.03118,(U

lt27892:0.00376,((UltCh117:0.0,UltCh123:0.0):0.00755,((Ult27891:0.00912,(
UltCh124:0.01718,Ult27893:0.02509)0.083.5:0.00389)0.912.146:0.01322,(Ult2
7894:0.01519,Ult27895:5.5E-4)0.754.81:0.00721)0.932.123:5.4E-
4)0.894.124:0.0075)0.761.78:0.00378)0.838.103:0.00676,((Ult27885:0.00476,
Ult27886:0.04771)0.976.82:0.03182,((Ult27882:0.00773,Ult27883:0.01099)0.8
61.133:0.00957,Ult27884:0.071)0.337.9:0.01011)0.977.85:0.02928)0.905.122:
0.01575)0.272.15:5.4E-
4,((UltCh150:0.00368,(UltMa112:0.01516,(Ult27946:0.00378,Ult27947:0.02695
)0.775.92:0.00372)0.872.125:0.00743)0.929.113:0.01153,((Ult27888:0.0229,U
lt27948:0.02308)0.913.134:0.01134,((Ult27942:0.00377,((Ult27945:0.00739,U
ltOr303:0.0114)0.999.171:0.04808,((Ult27944:5.5E-
4,(Ult27941:0.0037,Ult27943:5.5E-
4)0.920.143:0.00743)0.914.139:0.00742,Ult27940:5.4E-4)1.000.834:5.4E-
4)0.762.104:0.00361)1.000.835:0.05136,(((UltB4785:0.0,Ult27949:0.0):5.8E-
4,Ult27950:0.04787)0.966.119:0.02762,Ult27951:0.03569)0.787.89:0.01492)0.
262.11:5.4E-
4)0.786.95:0.004)0.895.130:0.00757)0.870.108:0.01461,(((Ult28394:0.12069,
((UltArch2:0.01706,(Ult28364:0.0099,Ult28365:0.02578)0.966.120:0.02807)0.
915.126:0.0242,((((((Ult28391:0.0,Ult28392:0.0):5.5E-
4,Ult28393:0.00369)0.829.99:0.01166,(Ult28389:0.00369,Ult28390:0.00373)0.
876.113:0.01254)0.987.120:0.03277,((((Ult28356:0.08265,((Ult28355:0.00747
,UltCh305:0.00381)0.916.135:0.01231,(Ult28354:0.053,(Ult28353:5.4E-
4,(Ult28351:0.00576,(Ult28350:5.5E-
4,Ult28352:0.01124)0.588.14:0.01013)0.937.117:0.01838)0.568.16:0.0115)0.8
34.84:0.00774)0.969.86:0.03106)0.515.11:5.4E-
4,(((Ult28383:0.0,Ult28384:0.0):0.01076,((Ult28358:0.00369,(Ult28359:0.0,
Ult28381:0.0):5.5E-
4)0.922.165:0.00466,((Ult28382:0.0,Ult28385:0.0,Ult28387:0.0,Ult32116:0.0
):5.5E-
4,Ult28360:0.06077)0.899.120:0.00374)0.908.140:0.01258)0.477.15:0.00403,(
Ult28386:0.01294,((Ult28380:0.00939,((Ult28362:0.0,Ult28379:0.0):5.5E-
4,Ult28378:0.00742)0.794.82:0.00569)0.775.93:0.02405,((UltCh306:5.4E-
4,((Ult28388:5.5E-4,(Ult28361:5.5E-
4,Ult28376:0.00369)0.865.123:0.00369)0.913.135:0.00752,(Ult28374:0.00383,
Ult28375:0.01895)0.784.100:0.00354)0.785.101:0.00373)0.774.102:0.00409,(U
lt28377:0.0077,UltrS356:0.01947)0.401.13:0.00741)0.805.72:0.00949)0.919.1
55:0.02551)0.679.25:0.00934)0.936.118:0.01966)0.940.115:0.02871,(((Ult283
44:0.00414,Ult28345:0.01088)0.995.111:0.06912,Ult28357:0.04339)0.259.6:0.
01262,(((Ult28363:0.0,Ult28368:0.0):0.01272,((Ult28369:0.0,Ult28372:0.0):
5.5E-
4,Ult28370:0.00368)0.980.77:0.03017)0.797.78:0.01068,((Ult28371:0.00797,(
Ult28367:0.01159,Ult28373:0.02366)0.737.71:0.0031)0.870.109:0.01773,Ult28
366:0.06314)0.799.92:0.01065)0.944.96:0.02004)0.735.48:0.00445)0.915.127:
0.01984,UltrS355:0.02357)0.462.16:0.00302)0.886.147:0.01804,Ult28347:0.06
328)0.427.15:0.00601,((((((UltCh299:0.01249,UltOr375:0.03251)0.522.4:0.01
784,(Ult28340:0.03279,UltCh301:0.04476)0.419.10:0.00775)0.830.89:0.01155,
(Ult28341:0.0128,Ult28343:0.06458)0.969.87:0.03012)0.771.117:0.00413,(Ult
28346:0.016,(UltCh303:0.04224,Ult28348:0.02489)0.450.15:0.00508)0.887.130
:0.01254)0.880.134:5.3E-
4,((Ult28349:0.02301,((UltCh300:0.01531,UltCh304:0.00767)0.305.11:0.00368
,Ult28342:0.01499)0.882.137:5.5E-
4)0.895.131:0.00754,(UltCh298:0.03345,UltCh302:0.02473)0.704.21:0.00248)0
.224.9:0.00372)0.913.136:0.02448,(UltOr376:0.04506,UltOr377:0.02815)0.915
.128:0.02604)0.880.135:0.02497)0.901.126:0.01673)0.540.14:0.00298)0.991.1

03:0.04837,(((Ult28273:0.00714,(Ult28271:0.00371,Ult28272:0.05148)0.838.1
04:5.5E-4)0.984.82:0.05555,(Ult28274:0.02695,Ult28275:5.4E-
4)0.963.91:0.03618)0.963.92:0.03633,((Ult28281:0.00475,(Ult28316:0.07715,
UltCh287:0.03849)0.995.112:0.06208)0.885.116:0.01443,((((UltCh144:0.03151
,Ult28338:5.4E-
4)0.763.111:0.00375,((((Ult28270:0.03027,(Ult28285:0.01107,(Ult28284:0.00
754,Ult28283:0.01517)0.845.115:5.5E-
4)0.969.88:0.02478)0.302.10:0.0075,Ult28269:0.01239)0.921.127:0.0142,((Ul
t28262:0.0,Ult28261:0.0):5.4E-
4,Ult28263:0.01963)0.781.91:0.00399)0.930.111:0.01159,((Ult28260:0.00765,
Ult28267:0.00765)0.777.110:0.00375,(Ult28268:0.00379,(((Ult28259:0.00377,
UltCh282:0.03534)0.999.172:5.5E-4,Ult28256:0.00378)0.643.11:5.4E-
4,(Ult28266:0.01936,((UltCh283:0.00377,UltCh285:0.00771)0.788.91:0.00369,
((Ult28264:0.02302,(Ult28258:0.01572,Ult28339:0.00754)0.062.10:0.00358)0.
877.132:5.5E-4,Ult28257:0.00376)0.836.87:0.00371)0.739.53:5.4E-
4)0.842.108:0.00378)0.855.110:0.00379)0.312.13:5.5E-
4)0.775.94:0.00365)0.812.70:0.00666)0.934.107:0.01164,(((Ult28279:0.02508
,Ult28278:0.04582)0.328.15:0.01066,(Ult28280:5.3E-
4,((Ult28276:0.0548,Ult28282:0.02121)0.827.82:0.0099,Ult28277:0.00406)0.4
11.18:0.0075)0.791.82:0.00324)0.000.870:5.4E-
4,(((UltCh284:0.01568,Ult28265:0.01258)0.875.134:0.01443,(((Ult28325:0.01
253,((UltrS353:0.0229,UltrS354:5.5E-
4)0.972.99:0.04169,Ult28326:0.04543)0.814.67:0.01626)0.964.112:0.02515,((
(UltCh294:0.02918,Ult28327:0.07163)0.715.24:0.02344,UltCh295:0.07055)0.64
1.15:0.01514,((Ult28323:0.01225,Ult28324:0.04194)0.889.126:0.01774,((Ult2
8322:0.03322,(UltCh290:0.0,UltCh293:0.0):0.01161)0.588.15:0.00388,((UltCh
291:0.0,UltCh289:0.0):0.00776,UltCh292:0.04174)0.649.15:0.00334)1.000.836
:0.05297)0.140.5:0.00883)0.562.16:5.4E-
4)0.913.137:0.02236,(UltCh296:0.01886,(Ult28329:0.02265,((Ult28318:0.0,Ul
t28319:0.0,Ult28320:0.0):5.5E-4,Ult28317:0.00369)0.991.104:5.5E-
4)0.987.121:0.03905)0.903.112:0.02168)0.894.125:0.0182)0.944.97:0.02568,(
(Ult28313:0.02524,(((Ult28307:0.0,Ult28309:0.0):0.01563,((Ult28304:0.0037
4,Ult28305:0.00739)0.754.82:0.00376,((Ult28310:0.00369,Ult28311:5.5E-
4)0.779.97:0.00438,(Ult28312:0.03988,(Ult28306:0.0,Ult28333:0.0):0.01246)
0.397.18:0.00706)0.777.111:0.0069)0.840.105:0.0073)0.925.132:0.02786,((((
Ult28330:0.00379,(Ult27393:5.4E-
4,Ult27394:0.01093)0.518.12:0.00718)0.877.133:0.00734,(((Ult28301:0.00369
,Ult28293:0.00369)0.671.13:5.5E-4,Ult28303:5.5E-4)0.000.871:5.5E-
4,(Ult28287:0.0,Ult28289:0.0,Ult28290:0.0,Ult28291:0.0,Ult28292:0.0,Ult28
294:0.0,Ult28295:0.0,Ult28296:0.0,Ult28302:0.0,Ult28331:0.0,Ult28334:0.0,
Ult28337:0.0):5.5E-4)0.912.147:5.5E-4)0.453.12:5.5E-
4,Ult28286:0.0037)0.961.100:0.01129,(Ult28298:0.00369,(((Ult28288:0.02126
,UltB2363:0.04795)0.968.95:0.02988,Ult28336:0.02273)0.867.127:5.4E-
4,(Ult28297:0.0,Ult28299:0.0,Ult28300:0.0,Ult28332:0.0):5.4E-
4)0.826.74:0.00369)0.860.90:5.4E-4)0.838.105:5.5E-
4)0.934.108:0.03452)0.929.114:0.02349,UltCal17:0.03042)0.968.96:0.0393)0.
851.113:0.01584)0.772.104:0.00383)0.997.121:5.5E-
4,(UltCh286:0.01315,(Ult28315:0.03623,Ult28314:0.02687)0.652.13:0.01129)0
.821.74:0.00571)0.926.141:0.0125)0.697.17:0.00541)0.848.101:0.01333)0.953
.91:0.02409,((UltCh212:0.02056,(UltCh211:0.04323,UltOr316:0.01327)0.919.1
56:0.02946)0.876.114:0.01947,(Ult28154:0.02457,(Ult28152:0.01126,Ult28153
:5.5E-
4)0.963.93:0.02901)0.879.138:0.01565)0.757.72:0.00486)0.972.100:0.03141)0
.526.13:0.01391,((((((UltCh143:0.0224,UltCh142:0.07572)0.897.122:0.01811

,(UltCh145:0.01538,((((Ult27905:0.05901,Ult27927:0.05793)0.824.77:0.01812
,(Ult27930:0.02291,Ult27929:5.4E-
4)0.793.89:0.00526)0.787.90:0.00513,(((Ult27922:0.0,Ult27923:0.0,Ult27924
:0.0):5.3E-
4,(Ult27925:0.00365,(Ult27880:0.00512,UltCh122:0.01418)0.979.95:0.0266)0.
797.79:0.00377)0.873.119:0.0087,((Ult27926:0.0118,Ult27931:0.03954)0.791.
83:0.00854,(UltCh109:0.05686,(Ult27392:0.01209,((Ult27391:0.02717,Ult2739
0:0.00385)1.000.837:5.3E-4,((Ult27734:0.01733,(Ult27389:5.3E-
4,UltChl68:0.01503)0.256.8:0.00569)0.139.12:0.00366,((UltChl67:0.02084,((
UltOr298:0.0,UltOr299:0.0):5.4E-
4,UltChl69:0.00742)0.791.84:0.00596)0.591.16:0.00791,(UltChl66:0.02029,Ul
tOr300:0.07173)0.511.11:5.5E-
4)0.951.108:0.01561)0.860.91:0.00518)0.934.109:0.02265)0.939.112:0.02439)
0.863.135:0.01123)0.753.93:0.00555)0.847.140:0.00749)0.744.69:0.00442,Ult
27928:5.3E-
4)0.884.128:0.01148)0.854.107:0.00994)0.755.78:0.00422,((((UltCh125:0.046
5,((UltCh126:0.01677,(Ult27630:5.4E-
4,(Ult27898:0.01898,Ult27899:0.00377)0.908.141:0.01106)0.841.95:0.00589)0
.885.117:0.01168,(((Ult27933:0.00953,Ult27932:0.02952)0.889.127:0.01208,(
(Ult27900:0.09546,((Ult27901:0.01553,(Ult27903:0.00368,Ult27904:5.4E-
4)0.937.118:0.01509)0.875.135:0.00846,(Ult27902:0.01039,UltCh127:0.03696)
0.846.124:0.00804)0.861.134:0.01191)0.837.77:0.01164,Ult27889:0.02871)0.7
67.85:0.00514)0.654.15:0.00123,(Ult27896:0.02311,Ult27897:5.4E-
4)0.777.112:0.00741)0.760.98:0.01259)0.741.62:0.00686)0.866.126:0.01111,(
Ult27890:0.02688,((Ult27631:0.00369,Ult27879:5.5E-
4)0.941.102:0.01765,(UltCh120:0.01113,UltCh121:0.02374)0.764.104:0.00557)
0.866.127:0.01443)0.572.12:0.00788)0.841.96:0.00887,((UltCh141:0.00772,((
(((Ult27915:0.00213,(Ult27917:0.00763,UltMa111:0.01544)0.848.102:0.00699)
0.919.157:0.00203,(UltHyd11:0.00369,UltCh136:5.5E-4)1.000.838:5.5E-
4)0.663.21:0.00369,(((UltCh140:0.00743,UltCh138:5.5E-
4)0.857.124:0.00369,Ult27918:0.01125)0.902.128:5.5E-
4,(UltCh137:0.0,UltCh139:0.0):5.5E-4)1.000.839:5.4E-
4)0.778.99:0.0037,UltCh135:0.01515)0.405.22:0.00371,(Ult27914:0.00744,Ult
27916:0.0112)0.922.166:5.4E-
4)0.969.89:0.02286)0.863.136:0.00799,(Ult27877:0.03546,(UltCh134:0.04755,
(UltCh133:0.04285,((((Ult27910:0.03709,((Ult27919:0.0,Ult27920:0.0):0.031
71,Ult27921:0.02745)0.992.101:0.05912)0.750.69:0.01831,(Ult27906:0.03929,
((Ult27907:0.0,Ult27908:0.0):0.06028,(UltCh128:0.01011,UltCh129:0.03492)0
.853.94:0.01495)0.880.136:0.01623)0.890.129:5.5E-
4)0.866.128:0.01779,(UltCh130:5.4E-
4,UltCh131:0.01879)0.998.153:0.04547)0.842.109:0.01255,(Ult27909:0.03356,
UltCh132:0.06651)0.862.117:0.02142)0.223.6:0.00807)0.966.121:5.4E-
4)0.879.139:0.00998)0.949.116:0.016)0.909.112:0.01514)0.860.92:0.01438,((
(Ult27938:5.5E-
4,UltCh146:0.01117)0.935.115:0.02046,(UltCh147:0.01029,UltCh148:0.00518)0
.936.119:0.02232)0.970.96:0.03116,(Ult27878:0.04068,((Ult27841:0.0,UltCh1
05:0.0):0.05357,Ult27939:0.04928)0.256.9:0.01372)0.827.83:0.01306)0.871.1
25:0.01267)0.852.112:0.00996)0.857.125:0.00762,(UltHyd12:5.4E-
4,((((Ult27937:0.01531,UltHyd13:0.01532)0.837.78:0.00749,UltCh149:0.0229)
0.755.79:0.00371,(Ult27934:0.00747,Ult27935:0.01117)0.756.93:5.4E-
4)0.767.86:0.00373,Ult27936:0.02302)0.275.13:0.00369)0.925.133:0.01396)0.
842.110:0.01158,(Ult27912:0.00369,(Ult27911:5.5E-4,Ult27913:5.5E-
4)0.630.13:5.5E-
4)0.930.112:0.01842)0.782.114:0.00566,(UltOr379:0.03446,(Ult28546:0.08507

,Ult28547:0.01063)0.829.100:0.01323)0.993.92:0.04248)0.745.86:0.00387,(((
(Ult27245:0.01869,Ult27709:0.03354)0.823.84:0.02588,((Ult27221:0.05702,((
Ult27689:0.01287,(Ult13085:0.00754,Ult27244:0.01115)0.985.98:0.03772)0.69
5.19:0.00474,(Ult27688:0.00449,((Ult27691:0.01906,(Ult27721:0.00972,Ult27
725:0.02265)0.958.120:0.02183)0.465.14:0.00342,(((Ult27155:5.5E-
4,(Ult27070:0.05315,Ult27154:0.03519)0.825.81:0.01272)0.981.90:0.02747,(U
lt27147:0.01877,Ult27146:5.3E-
4)0.806.64:0.00373)0.855.111:0.00222,Ult27690:0.03414)0.689.20:0.00729)0.
941.103:0.02139)0.916.136:0.01832)0.622.11:0.01096)0.291.14:5.4E-
4,Ult27710:0.01708)0.578.9:0.01893)0.852.113:0.01493,Ult27568:0.08029)0.1
38.10:0.02935,(Ult27367:0.01511,(Ult25888:0.03836,((((((Ult27137:0.00365,
(((Ult27133:0.0,Ult27138:0.0,Ult27695:0.0):5.5E-
4,((Ult27142:0.00369,(Ult27134:5.4E-4,((Ult27193:5.5E-
4,((((Ult27167:0.00369,(Ult27172:0.0037,(UltChl61:0.00369,(UltEu108:0.061
18,((Ult27194:0.00369,((Ult27173:0.0,Ult27222:0.0):5.5E-
4,Ult27176:0.00384)0.173.10:5.5E-4)0.816.77:0.00375,(Ult27215:5.5E-
4,((Ult27182:0.00369,Ult27354:0.00741)0.829.101:5.5E-
4,(((Ult27353:0.00369,(Ult27099:0.00696,(BacEnr50:0.0,Ult27192:0.0,Ult272
87:0.0):0.0021)0.909.113:0.00208)0.059.13:5.5E-
4,Ult27178:0.00369)0.723.38:5.5E-
4,(Ult27123:0.0,Ult27125:0.0,Ult27153:0.0,Ult27174:0.0,Ult27175:0.0,Ult27
179:0.0,Ult27180:0.0,Ult27185:0.0,Ult27187:0.0,Ult27189:0.0,Ult27190:0.0,
Ult27504:0.0,Ult27532:0.0):5.5E-4)0.000.872:5.5E-4)0.880.137:5.5E-
4)0.986.90:5.5E-
4)0.777.113:0.00386)0.800.76:0.00373)0.823.85:0.00382)0.220.11:5.5E-
4)0.000.873:5.5E-4,Ult27195:0.00369)0.000.874:5.5E-
4,Ult27196:0.0037)0.524.13:5.5E-
4,(Ult27118:0.0,Ult27144:0.0,Ult27162:0.0,Ult27181:0.0,Ult27191:0.0,Ult27
288:0.0,Ult27289:0.0):5.5E-4)0.298.13:5.4E-
4)0.945.100:0.00756,(Ult27170:0.00744,Ult27135:5.5E-
4)0.856.103:0.0037)0.365.12:5.5E-4)0.851.114:0.00376)0.246.17:5.5E-
4,Ult27132:0.00369)0.455.24:5.5E-4)0.204.6:5.5E-
4,Ult27136:0.00369)1.000.840:5.5E-
4)0.456.15:0.00376,((Ult27213:0.00717,Ult27214:0.00413)0.436.12:0.00753,(
(Ult27197:0.00369,Ult27124:5.5E-4)0.460.12:5.5E-
4,Ult27119:0.0037)0.931.123:0.00334)1.000.841:5.5E-4)0.852.114:5.4E-
4,((Ult27116:0.0,Ult27139:0.0,Ult27140:0.0,Ult27184:0.0,Ult27186:0.0):0.0
0371,Ult27183:0.00371)0.785.102:0.00371)0.295.21:5.2E-4,UltLongi:5.4E-
4)0.573.10:5.3E-
4,((Ult27711:0.03677,Ult27712:0.007)0.987.122:0.04508,((((UltAna18:5.5E-
4,UltEu109:0.0037)0.953.92:0.00744,(Ult27131:5.5E-4,Ult27141:5.5E-
4)0.965.95:0.0113)0.191.9:5.3E-
4,Ult27226:0.00369)0.855.112:0.0037,(((Ult27227:0.0,Ult27730:0.0):0.01076
,(Ult27117:0.00697,Ult27085:0.01563)0.211.12:0.00371)0.871.126:5.5E-
4,((Ult27115:0.01105,(Ult27509:0.01272,Ult27130:0.00628)0.772.105:0.00402
)0.900.109:0.01115,((Ult27228:0.0,Ult27533:0.0):0.00783,((((Ult27361:0.00
747,(Ult27079:0.04178,(Ult27497:0.02855,Ult27498:0.01436)0.760.99:0.01821
)0.851.115:0.01128)0.434.11:0.00369,(Ult27301:0.00372,((((((((((Ult27535:
5.5E-4,Ult27683:0.00208)1.000.842:0.0016,(Ult27256:0.02657,Ult27675:5.5E-
4)0.837.79:5.4E-4)0.896.114:5.5E-4,((Ult27122:0.0037,Ult27255:5.3E-
4)0.824.78:0.00369,Ult27266:0.01118)0.804.68:5.4E-4)0.000.875:5.3E-
4,(Ult27208:0.00369,Ult27305:0.00369)0.699.29:5.5E-4)0.000.876:5.5E-
4,Ult27330:5.5E-4)0.000.877:5.5E-
4,(Ult27714:0.00369,Ult27151:0.00369)0.907.126:5.5E-4)0.000.878:5.5E-

4,(Ult27679:0.00369,(Ult27524:5.5E-4,Ult27263:0.0074)0.594.15:5.5E-4)0.000.879:5.5E-4)0.000.880:5.5E-4,((Ult27309:0.00369,Ult27320:0.00369)0.931.124:5.5E-4,((Ult27120:0.0,Ult27128:0.0,Ult27148:0.0,Ult27143:0.0,Ult27145:0.0,Ult27149:0.0,Ult27152:0.0,Ult27168:0.0,Ult27177:0.0,Ult27205:0.0,Ult27207:0.0,Ult27210:0.0,Ult27212:0.0,Ult27223:0.0,Ult27234:0.0,Ult27242:0.0,Ult27246:0.0,Ult27235:0.0,Ult27236:0.0,Ult27237:0.0,Ult27238:0.0,Ult27239:0.0,UltChl65:0.0,Ult27251:0.0,Ult27252:0.0,Ult27253:0.0,Ult27254:0.0,UdnBac75:0.0,Ult27260:0.0,Ult27262:0.0,Ult27269:0.0,Ult27276:0.0,Ult27277:0.0,Ult27278:0.0,Ult27279:0.0,Ult27281:0.0,Ult27272:0.0,Ult27273:0.0,Ult27274:0.0,Ult27283:0.0,Ult27284:0.0,Ult27285:0.0,Ult27286:0.0,Ult27291:0.0,Ult27292:0.0,Ult27295:0.0,Ult27293:0.0,Ult27296:0.0,Ult27297:0.0,Ult27300:0.0,Ult27302:0.0,Ult27303:0.0,Ult27304:0.0,Ult27306:0.0,Ult27308:0.0,Ult27310:0.0,Ult27312:0.0,Ult27314:0.0,Ult27318:0.0,Ult27319:0.0,Ult27321:0.0,Ult27322:0.0,Ult27323:0.0,Ult27324:0.0,Ult27325:0.0,Ult27327:0.0,Ult27359:0.0,Ult27360:0.0,Ult27365:0.0,Ult27492:0.0,Ult27493:0.0,Ult27494:0.0,Ult27501:0.0,Ult27505:0.0,Ult27506:0.0,Ult27512:0.0,Ult27516:0.0,Ult27519:0.0,Ult27520:0.0,Ult27521:0.0,Ult27530:0.0,Ult27531:0.0,Ult27666:0.0,Ult27667:0.0,Ult27668:0.0,Ult27669:0.0,Ult27670:0.0,Ult27672:0.0,Ult27673:0.0,Ult27674:0.0,Ult27677:0.0,Ult27678:0.0,Ult27680:0.0,Ult27681:0.0,Ult27682:0.0,Ult27684:0.0,Ult27685:0.0,Ult27686:0.0,Ult27692:0.0,Ult27693:0.0,Ult27696:0.0,Ult27697:0.0,Ult27698:0.0,Ult27699:0.0,Ult27700:0.0,Ult27704:0.0,Ult27705:0.0,Ult27707:0.0,Ult27713:0.0,Ult27716:0.0,Ult27717:0.0,Ult27718:0.0,Ult27719:0.0,Ult27720:0.0,Ult27726:0.0,Ult27727:0.0,Ult27729:0.0,Ult27732:0.0):5.5E-4,((Ult27264:0.01494,Ult27363:5.5E-4)0.954.97:0.01119,(Ult27258:5.5E-4,Ult27271:0.00317)0.990.105:5.1E-4)0.875.136:5.5E-4)0.000.881:5.5E-4)0.000.882:5.5E-4)0.000.883:5.5E-4,((Ult27329:5.5E-4,Ult27518:5.5E-4)0.000.884:5.5E-4,((Ult27316:0.00741,(Ult27261:0.00714,Ult27313:0.00372)0.002.6:5.4E-4)0.912.148:5.4E-4,((Ult27259:5.5E-4,Ult27280:0.00297)1.000.843:7.1E-4,((Ult27490:0.00369,(Ult27671:0.00371,(Ult27150:0.0,Ult27209:0.0,Ult27211:0.0,Ult27241:0.0,Ult27243:0.0,Ult27268:0.0,Ult27267:0.0,Ult27270:0.0,Ult27311:0.0,Ult27326:0.0,Ult27522:0.0,Ult27723:0.0):5.5E-4)0.071.8:5.5E-4)0.896.115:0.00361,(Ult27203:0.00361,Ult27503:5.4E-4)0.402.13:0.00369)1.000.844:5.3E-4)0.911.146:5.0E-4)0.000.885:5.5E-4)0.000.886:5.5E-4)0.000.887:5.5E-4,(((((((Ult27275:0.01111,(Ult27121:0.00751,Ult27250:0.00375)0.867.128:0.00725)0.927.117:5.5E-4,Ult27703:0.00371)0.789.88:5.5E-4,Ult27169:5.5E-4)0.000.888:5.5E-4,(((Ult27495:0.0074,(Ult27282:5.4E-4,((Ult27478:0.00366,Ult27489:0.00373)0.953.93:0.01123,(Ult27340:0.00747,(Ult27514:0.00367,(Ult27487:0.01827,(Ult27507:5.4E-4,Ult27536:0.00741)0.812.71:0.0058)0.787.91:0.00587)0.888.133:0.00751)0.764.105:0.00364)0.979.96:5.4E-4)0.867.129:5.5E-4)0.828.72:0.00369,Ult27499:5.4E-4)0.841.97:0.00369,Ult27701:0.0037)0.718.31:5.5E-4)0.000.889:5.5E-4,Ult27315:5.5E-4)0.000.890:5.5E-4,(((Ult27298:5.5E-4,Ult27508:0.01086)0.996.116:5.5E-4,Ult27706:0.00369)0.477.16:5.5E-4,Ult27240:0.00369)0.000.891:5.5E-4)0.000.892:5.5E-4,(((Ult27307:5.5E-4,Ult27294:0.00371)0.761.79:5.5E-4,(((Ult27114:0.0,Ult27206:0.0,Ult27249:0.0,Ult27299:0.0,Ult27513:0.0,Ult27517:0.0,Ult27676:0.0,Ult27702:0.0,Ult27728:0.0):5.5E-4,Ult27491:0.00369)0.819.79:0.00369,Ult27722:0.00369)0.933.135:5.5E-4)0.000.893:5.5E-4,Ult27523:0.00368)0.000.894:5.5E-4)0.000.895:5.5E-4)0.914.140:5.5E-4)0.744.70:5.5E-4)0.933.136:0.01129,((((UltChl62:5.4E-

4,((Ult27233:0.00361,UltChl63:0.00761)0.990.106:0.03181,(Ult27232:5.5E-
4,(UltChl64:0.01508,Ult27328:0.00369)0.535.14:5.5E-4)0.995.113:5.4E-
4)0.834.85:0.00368)1.000.845:5.5E-4,((Ult27733:0.02679,Ult27358:5.4E-
4)0.612.18:0.00748,(Ult27229:5.5E-
4,(Ult27230:0.0,Ult27231:0.0,Ult27715:0.0):5.5E-
4)0.783.112:0.00379)0.783.113:0.0036)0.862.118:0.00742,((Ult27496:0.00368
,(((Ult27225:0.00368,(((Ult27336:0.00369,(Ult27086:0.0,Ult27202:0.0):0.00
369)0.898.141:5.5E-
4,(Ult27343:0.00368,((Ult27129:0.00371,((Ult27126:0.0,Ult27346:0.0,Ult273
47:0.0,Ult27731:0.0):5.5E-4,Ult27257:5.5E-4)0.709.28:5.5E-
4)0.826.75:0.0037,Ult27334:0.00371)0.387.15:5.5E-4)0.871.127:5.5E-
4)0.000.896:5.5E-
4,(Ult27216:0.0,Ult27217:0.0,Ult27224:0.0,Ult27331:0.0,UltAna19:0.0,Ult27
333:0.0,Ult27335:0.0,Ult27337:0.0,Ult27344:0.0,Ult27348:0.0,Ult27349:0.0,
Ult27350:0.0,Ult27351:0.0,Ult27352:0.0,Ult27500:0.0,Ult27687:0.0,Ult27694
:0.0):5.5E-4)0.899.121:5.3E-4)0.938.80:5.5E-
4,Ult27345:0.01902)0.920.144:0.0034,(Ult27356:0.01478,Ult27364:0.00777)0.
615.17:0.00742)1.000.846:5.3E-4)0.706.28:5.4E-4,Ult27332:5.5E-
4)0.952.105:5.5E-
4)0.888.134:0.00742,Ult27317:0.00372)0.801.84:0.00375)0.788.92:0.00369,(U
lt27342:5.5E-4,(Ult27362:0.00741,Ult27724:0.0037)0.521.6:5.5E-
4)0.770.111:0.00376)0.896.116:0.01129)0.801.85:0.00967)0.731.39:0.00218)0
.768.84:5.5E-4)0.917.112:0.00728)1.000.847:5.4E-
4)0.890.130:0.00843,(((Ult27127:0.00744,((Ult27064:0.00369,Ult27665:0.011
16)0.904.125:5.4E-4,(((Ult27062:0.00714,Ult27074:5.5E-4)0.894.126:5.5E-
4,Ult27098:0.00369)0.620.14:5.5E-
4,(Ult27090:0.0,Ult27096:0.0,Ult27102:0.0,Ult27061:0.0,Ult27069:0.0,Ult27
071:0.0,Ult27049:0.0,Ult27051:0.0,Ult27072:0.0,Ult27073:0.0,Ult27106:0.0,
Ult27111:0.0,Ult27165:0.0,Ult27290:0.0,Ult27534:0.0,Ult27539:0.0):5.5E-
4)0.000.897:5.5E-4)0.764.106:5.4E-
4)0.792.101:0.00455,(Ult27502:0.02335,(Ult27050:0.00371,(Ult27112:5.5E-
4,Ult27248:0.00369)0.498.9:5.5E-
4)0.794.83:0.00365)0.775.95:0.00295)0.737.72:0.00359,(Ult27113:0.00856,((
Ult27247:5.4E-4,(Ult27198:0.00376,(Ult27200:5.5E-
4,(((((Ult27089:0.0,Ult27092:0.0,Ult27093:0.0,Ult27095:0.0,Ult27083:0.0,U
lt27087:0.0,Ult27097:0.0,Ult27100:0.0,Ult27101:0.0,Ult27103:0.0,Ult27059:
0.0,Ult27068:0.0,Ult27052:0.0,Ult27053:0.0,Ult27104:0.0,Ult27105:0.0,Ult2
7110:0.0,Ult27157:0.0,Ult27158:0.0,Ult27159:0.0,Ult27160:0.0,Ult27163:0.0
,Ult27171:0.0):5.5E-
4,(((Ult27060:0.00368,(Ult27091:0.00369,(((Ult27084:0.0038,Ult27055:5.5E-
4)1.000.848:5.4E-
4,((((Ult27058:0.0,Ult27063:0.0,Ult27066:0.0,Ult27054:0.0,Ult27057:0.0,Ul
t27156:0.0,Ult27199:0.0):5.5E-
4,(((Ult27065:0.00369,Ult27109:0.00379)0.000.898:5.5E-
4,Ult27664:0.00369)0.409.15:5.5E-4,Ult27088:0.00369)0.625.11:5.5E-
4)0.366.11:5.5E-4,Ult27056:0.00368)0.885.118:5.5E-
4,Ult27188:0.01119)0.827.84:0.00366)0.192.11:0.00374,Ult27107:0.00376)1.0
00.849:5.5E-4)0.008.5:5.5E-4)0.000.899:5.5E-
4,Ult27067:0.00369)0.479.18:5.5E-4,(Ult27355:0.00369,Ult27368:5.5E-
4)0.853.95:0.00369)0.625.12:5.5E-4)0.000.900:5.5E-4,Ult27164:5.5E-
4)0.625.13:5.5E-4,Ult27108:5.5E-4)0.507.12:5.5E-
4,(Ult27094:0.00243,Ult27161:5.5E-
4)1.000.850:0.00125)0.880.138:0.00373)0.800.77:0.00376)0.925.134:0.00755)
0.699.30:0.00534,(Ult27201:0.0,Ult27204:0.0):0.01476)0.910.117:0.01394)0.

509.11:0.00374)0.766.89:0.00385)0.929.115:0.02191)0.838.106:0.01416)0.772
.106:0.01023)0.984.83:0.04961)0.757.73:0.00443)0.898.142:0.01752,((((UltC
h107:0.00377,(((DenBac29:0.0,DenBac30:0.0,DenBac31:0.0,DenBac32:0.0,DenBa
c33:0.0,DenBac34:0.0,DenBac35:0.0,DenBac37:0.0):5.5E-
4,DenBac36:0.00368)0.994.110:5.5E-4,(Ult27839:0.00371,(Ult27838:5.5E-
4,Ult27837:0.03925)0.894.127:0.00744)0.921.128:0.00745)0.952.106:0.01501)
0.929.116:0.0113,((UltChl93:0.00369,(UltCh106:0.00369,Ult27842:5.5E-
4)0.995.114:5.5E-
4)0.890.131:0.01005,Ult27840:0.04311)0.744.71:0.0053)0.977.86:0.03151,(((
Ult28328:0.05286,(Ult27737:0.05016,(Ult27735:0.03658,Ult27736:0.03361)0.7
65.87:0.00849)0.798.84:0.02357)0.869.98:0.01768,(((((UltrS345:0.015,(Ultr
S344:0.01079,UltrS346:0.00418)0.778.100:0.00998)0.804.69:0.00559,(UltCh10
4:0.02289,(Ult27833:0.00368,Ult27834:5.5E-4)0.946.104:5.4E-
4)0.793.90:0.01127)0.758.91:0.0031,(((Ult27810:0.01119,Ult27811:5.5E-
4)0.933.137:5.4E-4,((Ult27802:0.01493,Ult27829:5.5E-
4)0.615.18:0.01139,((Ult27791:0.00369,(Ult27800:0.0,Ult27801:0.0):5.5E-
4)1.000.851:5.4E-
4,UltSlu35:0.02683)0.877.134:0.00753)0.388.13:0.00371)0.965.96:0.0195,((U
ltrS341:0.01458,Ult27836:0.00807)0.855.113:0.00749,((UltChl94:5.5E-
4,UltrS340:0.00369)0.969.90:0.01453,((Ult27792:0.00369,Ult27809:5.5E-
4)0.923.164:5.4E-
4,((UltChl96:0.0,UltChl97:0.0):0.00369,(Ult27804:0.00369,(Ult27803:5.5E-
4,((Ult27795:0.00369,((((Ult27793:0.00369,(Ult27794:0.0,UltAna22:0.0):5.5
E-
4)0.865.124:0.00762,((Ult27786:0.0,Ult27808:0.0,Ult27830:0.0):0.01108,(((
Ult27787:0.00368,(UltrS342:0.00369,Ult27807:0.00369)0.294.10:5.5E-
4)0.000.901:5.5E-4,(Ult27788:0.0,Ult27789:0.0,Ult27805:0.0):5.5E-
4)0.724.34:5.5E-4,UltrS343:0.0037)0.925.135:5.4E-
4)0.865.125:0.00731)0.882.138:0.00757,(Ult27790:0.00369,Ult27806:0.00369)
0.763.112:5.5E-
4)0.942.109:0.00744,(((Ult27784:0.0,Ult27785:0.0,Ult27796:0.0):5.5E-
4,(Ult27799:0.00203,Ult27797:0.01092)0.930.113:0.00198)0.758.92:5.4E-
4,Ult27798:5.5E-4)0.709.29:5.4E-4)0.846.125:0.00369)0.806.65:5.4E-
4,UltChl95:0.01124)0.915.129:0.00747)0.910.118:0.00745)0.993.93:5.4E-
4)0.366.12:0.00369)0.949.117:0.01102)0.620.15:5.4E-
4)0.776.97:0.00398)0.913.138:0.0121)0.847.141:0.0113,((Ult27814:0.0075,((
((((UltCh102:0.01493,(Ult27823:0.00371,UltDe231:0.0037)0.871.128:5.3E-
4)0.979.97:0.01908,UltCh103:0.00386)0.792.102:0.00356,Ult27824:5.4E-
4)0.787.92:0.00626,Ult27822:0.01249)0.202.13:0.00742,(Ult27825:0.0037,(Ul
t27828:0.00743,(Ult27826:0.00744,(Ult27827:0.00369,Ult27835:0.00369)0.000
.902:5.5E-4)0.218.10:5.5E-
4)0.776.98:0.0037)0.873.120:0.01067)0.944.98:0.01581,(Ult27812:5.5E-
4,Ult27831:0.00369)0.775.96:0.00379)0.776.99:0.00356)0.897.123:0.00748,Ul
t27815:5.4E-4)0.951.109:0.0183)0.721.21:5.5E-
4,(Ult27821:0.0085,((UltChl98:0.00756,(UltChl99:0.00745,UltCh100:5.5E-
4)0.751.65:0.00369)0.944.99:0.0184,(((Ult27813:0.00354,UltCh101:0.01149)0
.870.110:0.00768,(Ult27819:0.0,Ult27820:0.0):5.5E-
4)0.782.115:0.00369,(Ult27816:0.0,Ult27817:0.0,Ult27818:0.0):5.4E-
4)0.871.129:0.00868)0.803.73:0.01141)0.881.133:0.0105)0.833.97:0.00747)0.
937.119:0.02482,(Ult27739:0.01931,((((((UltChl87:0.01195,(Ult27639:0.0157
5,Ult27738:0.00301)0.844.119:0.00774)0.894.128:0.01087,(Ult27638:0.0325,(
(Ult27635:0.01946,Ult27640:0.01155)0.921.129:0.01546,((Ult27634:0.01553,U
lt27740:0.01591)0.668.22:0.00301,((Ult27636:0.01007,UltChl86:0.02656)0.91
8.134:0.01456,Ult27637:0.00356)0.790.90:0.00497)0.814.68:0.01247)0.767.87

:0.00664)0.719.31:0.0043)0.836.88:0.01041,((((UltChl89:0.02016,Ult27656:0
.00656)0.951.110:0.02576,(Ult27655:0.04806,(UltChl88:0.03391,(Ult27641:0.
00393,Ult27643:0.03966)0.814.69:0.00995)0.619.9:0.01463)0.563.12:0.00598)
0.508.8:0.00481,(Ult27654:0.02837,Ult27644:0.01092)0.399.20:0.01076)0.695
.20:0.00535,(Ult27653:0.02417,Ult27645:0.01635)0.777.114:0.00745)0.968.97
:0.02659)0.994.111:0.04194,(((((Ult27438:0.00921,(BactK4b6:0.00783,UltEu1
11:0.02748)0.959.109:0.02373)0.827.85:0.01043,(((UltEu113:0.0351,(((Ult27
624:0.02525,(UltChl83:0.02548,((Ult27620:0.00822,(Ult27621:0.03003,Ult276
22:0.00451)0.649.16:5.5E-
4)0.842.111:0.01354,(((Ult27616:0.0,Ult27617:0.0):5.5E-
4,Ult27623:0.00369)0.916.137:0.01528,(UltChl82:0.00432,(UltChl81:5.4E-
4,(Ult27618:0.02321,Ult27619:0.01112)0.851.116:5.4E-
4)0.866.129:0.00723)0.725.39:0.00407)0.827.86:0.00958)0.838.107:0.01051)0
.994.112:0.06378)0.441.19:0.02274,(UltBani6:0.04549,Ult27628:0.05641)0.11
0.9:0.01665)0.852.115:0.0187,(UltChl90:0.02965,(Ult27657:0.02267,Ult27658
:0.06728)0.823.86:0.03115)0.891.103:0.033)0.831.82:0.01694)0.366.13:0.005
28,(UltChl74:0.03864,(Ult27443:0.00474,(Ult27444:0.00732,Ult27782:5.4E-
4)0.908.142:0.01834)0.522.5:0.01217)0.496.9:0.00423)0.911.147:0.01293,(((
((Ult27437:0.0,UltChl71:0.0):0.00411,(Ult27422:5.5E-
4,((((((Ult27416:5.3E-
4,UltChl70:0.0037)0.922.167:0.00242,((Ult27415:0.0,Ult27417:0.0,Ult27418:
0.0,Ult27430:0.0,Ult27763:0.0,UltAna21:0.0):5.4E-
4,Ult27765:0.0112)0.922.168:0.0025)0.922.169:0.00241,Ult27419:5.3E-
4)0.333.8:0.00371,(Ult27764:0.0037,((Ult27400:0.0,Ult27420:0.0,Ult27421:0
.0,Ult27424:0.0,Ult27431:0.0,Ult27752:0.0):5.5E-4,Ult27526:5.5E-
4)0.771.118:5.5E-4)0.803.74:0.00366)1.000.852:5.5E-
4,((Ult27357:0.01119,((Ult27411:0.0,Ult27412:0.0,Ult27754:0.0):5.5E-
4,(Ult27423:0.00741,(Ult27414:0.00299,Ult27426:5.5E-4)0.957.107:6.9E-
4)0.854.108:5.5E-4)0.788.93:5.5E-4)0.843.98:0.00774,((Ult27413:5.5E-
4,((Ult27399:0.0,Ult27401:0.0,Ult27405:0.0,Ult27409:0.0,Ult27410:0.0,Ult2
7427:0.0,Ult27428:0.0,Ult27525:0.0,Ult27761:0.0,Ult27762:0.0):5.5E-
4,Ult27404:5.5E-4)0.725.40:5.5E-4)0.736.57:5.4E-
4,((((Ult27402:0.0,Ult27406:0.0,Ult27429:0.0,Ult27432:0.0,Ult27433:0.0,Ul
t27434:0.0,Ult27435:0.0,Ult27436:0.0,Ult27757:0.0,Ult27759:0.0,Ult27760:0
.0):5.5E-4,Ult27403:5.5E-4)0.818.57:5.5E-
4,(Ult27408:0.00369,Ult27407:0.00368)0.588.16:5.5E-4)0.718.32:5.4E-
4,Ult27753:0.01499)0.814.70:0.00368)0.950.101:0.0149)0.552.11:0.00754)0.8
89.128:0.00337,Ult27425:0.00747)0.882.139:0.00747)0.857.126:0.00703)0.763
.113:0.00904,Ult27440:0.02133)0.254.7:0.00406,UltEu110:0.00605)0.870.111:
0.00896,(Ult27439:0.0,UltChl72:0.0):5.5E-
4)0.931.125:0.00746)0.155.11:5.3E-
4)0.790.91:0.00398,(((Ult27366:0.03952,((Ult27370:0.01121,(Ult27482:0.007
48,Ult27515:0.01126)0.757.74:0.0036)0.779.98:0.00374,(Ult27384:0.00369,((
((Ult27075:0.0,Ult27076:0.0,Ult27077:0.0,Ult27080:0.0,Ult27265:0.0,Ult273
72:0.0,Ult27371:0.0,Ult27373:0.0,Ult27374:0.0,Ult27375:0.0,Ult27376:0.0,U
lt27377:0.0,Ult27378:0.0,Ult27380:0.0,Ult27381:0.0,Ult27383:0.0,Ult27385:
0.0,Ult27388:0.0,Ult27741:0.0,Ult27743:0.0,Ult27744:0.0,Ult27745:0.0,Ult2
7746:0.0,Ult27747:0.0,Ult27748:0.0,Ult27749:0.0,Ult27750:0.0,Ult27751:0.0
,Ult27758:0.0):5.5E-4,Ult27742:5.5E-4)0.000.903:5.5E-
4,(Ult27386:0.00369,((Ult27379:0.0,Ult27382:0.0):5.5E-
4,Ult27556:0.00369)0.831.83:0.00369)0.704.22:5.5E-4)0.000.904:5.5E-
4,(Ult27369:0.00369,(Ult27078:0.00371,Ult27081:0.01908)0.962.117:0.01504)
0.723.39:5.5E-4)0.904.126:5.4E-4)0.849.107:0.00369)0.909.114:5.5E-
4)0.844.120:0.00753,((Ult27625:0.0,Ult27626:0.0,Ult27627:0.0):0.03579,Ult

27783:0.00762)0.928.103:0.01582)0.856.104:0.01048,(((((Ult27387:0.0,Ult27
660:0.0,Ult27661:0.0):0.01528,Ult27395:0.00757)0.907.127:0.0113,(Ult27460
:0.00536,Ult27461:0.02205)0.960.104:0.01899)0.737.73:0.00363,((Ult27467:0
.00372,Ult27468:0.00367)0.993.94:0.03081,((Ult27510:0.01886,(Ult27456:0.0
0369,(Ult27457:0.0,Ult27458:0.0):5.5E-4)0.888.135:5.5E-
4)0.870.112:0.00758,(Ult27453:0.01146,(Ult27454:0.0,Ult27455:0.0):0.00746
)0.838.108:0.00764)0.935.116:0.01601)0.850.123:0.0078)0.779.99:0.0072,(Ul
t27777:0.01933,(Ult27776:0.00745,((LnlArvor:0.00372,UltChl92:5.5E-
4)0.961.101:0.01135,((((Ult27770:0.00369,(Ult27773:0.00196,Ult27778:0.018
56)0.979.98:0.0019)0.861.135:5.5E-
4,(Ult27767:0.0,Ult27768:0.0,Ult27775:0.0,Ult27771:0.0):5.5E-
4)0.000.905:5.5E-4,((Ult27772:0.00369,Ult27779:0.01116)0.957.108:5.5E-
4,((Ult27766:0.0074,Ult27780:0.00369)0.629.22:5.5E-
4,(Ult27769:0.00369,Ult27774:0.00369)0.881.134:5.5E-4)0.000.906:5.5E-
4)0.747.72:5.5E-4)0.938.81:5.5E-4,Ult27459:0.00369)0.813.79:5.5E-
4)0.877.135:0.00741)0.832.87:0.00724)0.981.91:0.02455)0.755.80:0.00581)0.
780.118:0.00566)0.790.92:0.00392,((((MeaSeque:0.03558,(((Ult27851:0.00755
,Ult27854:0.00755)0.788.94:0.00365,(UltCh112:0.00753,Ult27853:0.00372)0.9
99.173:5.4E-
4)0.817.61:0.00757,Ult27852:0.0075)0.977.87:0.03151)0.397.19:0.01717,((Ul
t27082:0.04058,(Ult27866:0.01658,(Ult27867:0.07006,UltCh113:5.3E-
4)0.137.8:0.00992)0.588.17:0.0128)0.932.124:0.02901,(((((Ult27856:0.0,Ult
27857:0.0,Ult27859:0.0,Ult27861:0.0):5.5E-
4,Ult27860:0.00821)0.993.95:0.03036,(Ult27858:0.00742,Ult27865:5.4E-
4)0.858.119:0.00751)0.880.139:0.01188,((Ult27855:0.01934,(Ult27862:0.0118
4,Ult27863:0.01507)0.755.81:0.01137)0.623.13:5.3E-
4,(((Ult27848:0.00762,Ult27849:0.0232)0.766.90:0.00335,(Ult27847:0.00372,
UltCh110:0.00372)0.968.98:0.01559)0.740.68:0.00412,((UltChl91:0.0,UltCh11
1:0.0):5.5E-
4,Ult27850:0.00371)0.860.93:0.00751)0.355.17:0.01147)0.791.85:0.01527)0.8
14.71:0.00691,Ult27864:0.02384)0.369.11:0.00612)0.840.106:0.0094)0.913.13
9:5.5E-
4,((((Ult27466:0.03706,(Ult27662:0.02111,(Ult27469:0.03925,Ult27663:0.023
91)0.775.97:0.01193)0.900.110:0.01884)0.836.89:0.01033,((Ult27397:0.0,Ult
27398:0.0):5.5E-4,(Ult27396:0.00369,(Ult27442:0.00741,(Ult27441:5.5E-
4,Ult27576:0.00369)0.101.5:5.5E-4)0.924.120:0.00743)0.527.9:5.5E-
4)1.000.853:5.3E-
4)0.936.120:0.01122,((Ult27756:0.06982,(Ult27450:0.02298,Ult27462:0.06972
)0.837.80:0.0168)0.555.9:0.00919,(Ult27451:0.03058,Ult27452:5.4E-
4)0.979.99:0.02291)0.770.112:0.00353)0.897.124:5.5E-
4,(((((Ult27448:0.0,Ult27449:0.0):5.5E-4,Ult27447:0.01516)0.840.107:7.8E-
4,(Ult27445:0.00832,(Ult27446:0.01904,LplTardi:0.00455)0.379.12:0.00366)0
.785.103:0.00603)0.358.11:0.00375,Ult27781:0.01054)0.965.97:0.02725,((Ult
Chl59:0.0651,UltChl75:0.00405)0.973.90:0.02531,(UltChl84:0.02642,((Ult275
79:0.0,Ult27580:0.0):0.01995,UltChl79:0.0113)0.931.126:0.02555)0.907.128:
0.01841)0.720.33:0.00803)0.858.120:0.01329)0.796.69:0.01093)0.115.5:0.003
63,(Ult27465:5.5E-
4,(Ult27463:0.00746,(BacEnr51:0.00369,(Ult27464:0.0,Ult27755:0.0):5.5E-
4)0.022.3:5.3E-
4)0.829.102:0.00368)1.000.854:0.03508)0.852.116:0.00743)0.860.94:0.00836,
(((Ult27577:0.03942,((UltChl73:0.04852,(UltChl80:0.03159,Ult27659:0.0167)
0.068.7:0.00654)0.886.148:0.018,Ult27629:0.02309)0.035.9:5.4E-
4)0.909.115:0.00986,((Ult27647:0.09067,((Ult27341:0.00734,(Ult27651:5.5E-
4,((Ult27649:0.0,Ult27650:0.0):0.00369,Ult27652:0.0112)0.593.8:5.5E-

4)0.995.115:5.3E-
4)0.947.93:0.02751,(UltAna20:0.02814,(Ult27646:0.00904,((AaeTherm:5.5E-
4,Ult27648:0.00372)0.795.88:0.00565,AaeTher2:0.06152)0.207.7:0.00528)0.95
7.109:0.02857)0.956.109:0.03031)0.415.12:0.0111)0.971.81:0.03203,(Ult2763
3:0.06412,(UltChl76:0.05354,(Ult27632:0.04586,UltChl85:0.01553)0.155.12:0
.00745)0.403.16:0.00701)0.981.92:0.03797)0.917.113:0.01949)0.664.19:5.4E-
4,(Ult27218:0.01964,(Ult27615:0.01588,(((((ChfGenom:0.06562,(UltRu551:0.0
4888,Ult27610:0.04606)0.870.113:0.02672)0.928.104:0.02908,((Ult27613:0.0,
Ult27614:0.0):0.00858,((Ult27338:0.01118,((((Ult27609:5.5E-
4,((Ult27219:0.0,Ult27582:0.0,Ult27584:0.0,Ult27585:0.0,Ult27586:0.0,Ult2
7588:0.0,Ult27589:0.0,Ult27590:0.0,Ult27591:0.0,Ult27593:0.0,Ult27594:0.0
,Ult27595:0.0,Ult27596:0.0,Ult27597:0.0,Ult27599:0.0,Ult27600:0.0,Ult2760
1:0.0,Ult27604:0.0,Ult27605:0.0,Ult27606:0.0):5.5E-
4,((Ult27608:0.00368,Ult27592:0.0265)0.916.138:5.5E-
4,(Ult27339:0.00748,Ult27587:0.00369)0.908.143:5.5E-4)0.000.907:5.5E-
4)0.000.908:5.5E-4)0.000.909:5.5E-4,Ult27602:5.5E-4)0.000.910:5.5E-
4,((Ult27598:0.01472,(Ult27603:0.0037,Ult27220:0.01121)0.849.108:0.00196)
0.942.110:0.00194,Ult27607:0.00369)0.880.140:5.5E-4)0.799.93:5.4E-
4,Ult27583:0.00368)0.728.43:5.5E-
4)0.743.75:0.00391,Ult27581:0.07966)0.944.100:0.01802)0.779.100:0.00543)0
.843.99:0.00988,UltEu112:0.00645)0.871.130:0.00859,Ult27612:0.00414)0.672
.19:0.0026,Ult27611:0.00471)0.813.80:0.01756)0.984.84:0.06006)0.996.117:0
.06163)0.859.112:0.01161)0.692.15:0.00705)0.854.109:5.5E-
4,(Ult27868:0.03372,UdnGreen:0.04262)0.856.105:0.01324)0.948.130:0.01526,
((Ult27846:0.01161,(Ult27843:0.01497,(Ult27844:0.00373,Ult27845:5.5E-
4)0.918.135:5.5E-
4)0.924.121:0.01529)0.958.121:0.01968,UltCh108:0.00387)0.000.911:5.4E-
4)0.876.115:0.0108)0.798.85:0.00684)0.862.119:0.02106)0.246.18:0.00568,((
(((UltCh345:0.03198,(Ult28548:0.0452,Ult28549:0.02714)0.839.100:0.01766)0
.978.81:0.05167,((Ult27980:0.04905,(Ult27977:0.01955,(Ult27978:0.004,Ult2
7979:5.5E-
4)0.929.117:0.01275)0.710.33:0.00498)0.961.102:0.03149,((UltCh155:0.01127
,((Ult27973:0.00372,UltSlu36:5.5E-
4)0.928.105:0.0075,Ult27974:0.00747)0.983.78:5.4E-
4)0.981.93:0.0298,((Ult27976:5.5E-
4,UltrS347:0.00745)0.745.87:0.00369,(Ult27983:0.00984,(Ult27982:0.02034,U
lt27975:0.02503)0.251.9:0.00581)0.887.131:0.00931)0.877.136:0.00922)0.292
.7:0.00435)0.901.127:0.03703)0.837.81:0.02692,(UltCrat8:0.09868,Ult27984:
0.03772)0.946.105:0.04337)0.867.130:0.02023,(((Ult27963:0.02788,(Ult27955
:0.03152,(Ult27956:0.00765,(((Ult27961:0.00741,Ult27962:5.4E-
4)0.912.149:0.01507,BenMine3:0.02915)0.903.113:0.01583,(((UltChl78:0.0400
6,(Ult27957:0.02416,UltCh154:0.03365)0.534.13:0.01018)0.435.15:0.01074,((
Ult27969:0.00386,((Ult27966:5.3E-
4,Ult27967:0.01558)0.947.94:0.01139,((Ult27968:0.0037,(UltCh152:0.02751,(
Ult27970:0.00368,UltCh153:5.5E-4)0.588.18:5.5E-
4)0.788.95:0.00369)0.813.81:0.01025,(Ult27958:0.02431,(Ult27959:0.01112,(
UltCh151:0.04469,Ult27960:0.0146)0.339.14:0.0116)0.642.14:0.00301)0.723.4
0:0.01137)0.760.100:9.2E-4)0.955.98:0.01135)1.000.855:5.5E-
4,(Ult27971:0.01134,Ult27972:0.00378)0.806.66:0.00342)0.769.98:0.00424)0.
798.86:0.00709,(Ult27952:0.07492,(Ult27953:0.02235,Ult27954:0.01088)0.751
.66:0.00499)0.917.114:0.01669)0.906.139:0.00122)0.922.170:0.00303)0.073.6
:5.4E-4)0.940.116:0.01206)0.823.87:5.5E-
4,(Ult27964:0.00779,Ult27965:0.01562)0.898.143:0.01105)0.806.67:0.01002,(
UltCh156:0.02314,UltCh157:0.04171)0.696.20:0.01478)0.885.119:0.02096)0.87

3.121:5.4E-
4,(Ult27981:0.0427,(Ult27986:0.02629,Ult27985:0.03681)0.734.65:0.00533)0.
861.136:0.00914)0.989.88:0.02929)0.787.93:0.00781)0.826.76:0.01126)0.893.
134:0.02113)0.900.111:0.02801)0.815.72:0.01132,((((Ult28597:0.02225,((Ult
28599:0.04335,Ult28600:0.01298)0.991.105:0.03939,UltCh351:0.0385)0.000.91
2:0.00268)0.921.130:0.02311,(UltAc941:0.00955,UltCa159:0.01794)0.895.132:
0.01417)0.885.120:0.01855,Ult28598:0.01156)0.995.116:0.04805,(((Ult28594:
0.02241,(Ult28595:0.0,Ult28596:0.0):0.0411)0.210.17:0.00778,(Ult28592:0.0
6216,Ult28591:5.5E-4)0.858.121:0.00553)0.847.142:5.5E-
4,Ult28593:0.01677)0.981.94:0.03526)0.741.63:0.00521)0.888.136:0.02353)0.
927.118:0.02312,(((UltCh514:0.00351,UltCh515:0.00765)1.000.856:0.1043,((U
lt28617:0.03347,UltCh355:0.1289)0.909.116:0.03129,((Ult28616:0.01509,(Ult
Ch354:0.02247,(Ult28614:0.00838,Ult28615:0.01817)0.722.32:0.00727)0.874.1
46:0.01328)0.963.94:0.03368,((Ult28239:0.0034,Ult28240:0.01542)0.993.96:0
.05203,(Ult28241:0.027,Ult28242:0.00404)0.959.110:0.02569)0.597.11:0.0104
9)0.911.148:0.02238)0.883.134:0.02191)0.806.68:0.01788,(Ult27876:0.0803,(
Ult29307:0.03771,Ult29306:0.03601)0.998.154:0.07922)0.413.20:0.01428)0.43
4.12:0.01379)0.704.23:5.4E-
4,(UltB4784:0.13733,(Ult28449:0.16656,(Ult28448:5.5E-4,(Ult28447:5.4E-
4,Ult27554:0.04277)0.762.105:0.00356)0.783.114:0.03807)0.977.88:0.08423)0
.241.11:0.02907)0.727.40:0.00548,(Ult28611:0.05054,UltCh353:0.03735)0.970
.97:0.04163)0.538.12:0.02268)0.906.140:0.0214,(((((((Ult28248:0.05248,(Ul
t28247:0.01475,(Ult28246:0.02433,UltCh276:5.5E-
4)0.829.103:0.01314)0.850.124:0.01328)0.792.103:0.00948,UltCh281:0.01637)
0.829.104:0.0115,(((((Ult28604:0.03665,(Ult28602:0.04609,Ult28603:0.00282
)0.878.113:0.01615)0.942.111:0.02405,((Ult28609:5.4E-
4,Ult28610:0.03136)0.981.95:0.03607,(Ult28608:0.02817,(UltCh352:0.02415,(
Ult28606:0.01931,Ult28607:0.00732)0.928.106:0.0163)0.701.27:0.00289)0.636
.12:0.01223)0.980.78:0.03346)0.231.10:0.01142,(Ult28605:0.07234,(Ult28252
:0.02271,(Ult28243:0.04988,(Ult27875:5.5E-4,Ult28244:5.5E-
4)0.960.105:0.03728)0.989.89:0.06303)0.861.137:0.02637)0.742.59:0.02156)0
.513.11:5.4E-
4,(((Ult28222:0.00378,UltCh272:0.00742)0.942.112:0.01909,(Ult28221:0.1504
7,UltCh271:0.0322)0.939.113:0.04178)0.209.14:0.00749,((Ult28210:5.4E-
4,((((UltCh267:0.00972,UltCh270:0.01303)0.564.13:0.00378,Ult28217:0.00532
)0.877.137:0.00974,Ult28216:5.4E-4)0.767.88:0.00372,(Ult28213:5.4E-
4,Ult28223:0.00747)0.895.133:0.00746)0.888.137:0.00746)0.902.129:0.00747,
(((Ult28209:5.4E-
4,(UltCh268:0.00745,(Ult13232:0.01101,(Ult28211:0.00397,Ult28212:0.01878)
0.760.101:0.00748)0.769.99:0.00401)0.791.86:0.0037)0.530.9:5.4E-
4,(Ult28219:0.00369,((Ult28218:5.5E-4,((Ult28215:0.01497,Ult28220:5.5E-
4)0.894.129:0.01131,UltCh269:0.01118)0.323.10:5.5E-
4)0.856.106:0.00359,((Ult28208:5.5E-
4,UltCh266:0.00369)0.777.115:0.00378,(UltCh273:0.07218,UltCh274:5.5E-
4)0.984.85:0.01871)0.223.7:0.00371)1.000.857:5.5E-
4)0.858.122:0.00367)0.828.73:0.00365,Ult28214:0.01886)0.482.17:5.5E-
4)0.937.120:5.4E-
4)0.975.93:0.01865)0.814.72:0.0106,(Ult28601:0.04444,((UltCh278:0.02549,U
ltCh279:0.00533)0.553.13:0.00761,(Ult28250:0.03641,(UltCh280:0.01961,(Ult
28254:0.0179,(Ult28255:0.0265,(Ult28251:0.02059,Ult28253:0.03202)0.735.49
:0.00662)0.360.15:0.00425)0.617.13:5.4E-
4)0.777.116:0.00361)0.877.138:0.00838)0.763.114:0.00718)0.947.95:0.02222)
0.723.41:5.3E-
4)0.904.127:0.01552,((Ult28485:0.01784,Ult28486:0.02099)0.904.128:0.03186

,(DhlSpeci:0.02335,DhlSpec2:0.02333)0.973.91:0.04635)0.922.171:0.03487)0.
491.12:0.00798,((((Ult28458:5.5E-
4,(Ult28456:0.00369,Ult28457:0.00743)0.416.28:5.5E-4)0.959.111:5.5E-
4,(Ult28455:0.00371,UltB1446:0.0189)0.447.14:0.00369)0.979.100:0.02647,(U
lt28459:0.0263,(Ult28453:5.4E-
4,Ult28454:0.00716)0.918.136:0.02312)0.912.150:0.02086)0.838.109:0.01295,
((((Ult28481:0.01889,((Ult28482:0.01202,(Ult28476:5.4E-4,((Ult28473:5.4E-
4,UltB4783:0.09361)0.913.140:0.01119,((UltB4781:5.5E-
4,((Ult28471:0.0,Ult28474:0.0,Ult28475:0.0):5.5E-
4,Ult28472:0.00369)0.724.35:5.5E-4)0.767.89:5.5E-4,Ult28470:5.5E-
4)0.958.122:5.3E-
4)0.817.62:0.00364)0.997.122:0.03199)0.794.84:0.00602,((((Ult28466:0.0189
1,((Ult28469:0.0191,Ult28483:0.01131)0.904.129:0.01132,Ult28480:0.01492)0
.256.10:5.3E-4)0.819.80:0.00368,UltCh335:5.4E-4)0.959.112:5.4E-
4,UltCh334:0.01887)0.738.54:0.00449,Ult28477:0.01053)0.575.10:0.00387)0.7
65.88:0.00461)0.149.14:0.00351,Ult28484:0.04496)0.754.83:0.00399,((Ult284
68:0.00357,(Ult28464:0.00734,((Ult28461:0.0074,Ult28462:5.5E-
4)0.847.143:0.00369,(Ult28463:5.5E-
4,(Ult28460:0.00742,Ult28467:0.00369)0.931.127:5.5E-4)0.958.123:5.5E-
4)1.000.858:5.4E-4)0.794.85:0.0114)0.964.113:0.0192,(UltCh332:5.4E-
4,(UltB4782:0.00365,Ult28465:0.01123)0.299.15:0.01129)0.989.90:0.02676)0.
896.117:0.01136)0.768.85:0.00367,(UltCh333:0.04897,(Ult28478:0.03602,Ult2
8479:0.04165)0.882.140:0.02208)0.761.80:0.00887)0.976.83:0.02385)0.968.99
:0.02293)0.832.88:5.3E-
4,((Ult28207:0.03185,(((Ult28205:0.01142,Ult28206:0.00756)0.775.98:0.0036
4,(Ult28199:0.0037,(((Ult28198:0.00749,Ult28201:0.01124)1.000.859:5.4E-
4,(UltCh263:0.00384,UltCh264:0.01514)0.438.16:0.00754)0.780.119:0.00347,U
lt28200:0.01126)0.783.115:0.00373)0.900.112:0.00748)0.742.60:0.00382,((Ul
tCh265:0.00635,Ult28204:0.03239)0.906.141:0.01929,(Ult28202:5.3E-
4,Ult28203:0.05026)0.988.102:0.03478)0.913.141:0.01807)0.921.131:0.01585)
0.992.102:0.04126,Ult28613:0.05642)0.840.108:5.5E-
4)0.878.114:0.00989,Ult28227:0.04606)0.793.91:0.00739)0.762.106:0.00908,(
(((Ult28014:0.07402,(Ult28012:0.00421,Ult28013:0.007)0.950.102:0.04761)0.
953.94:0.06835,Ult29182:0.26444)0.284.9:0.03066,(Ult28155:0.03057,Ult2815
6:0.0662)0.763.115:0.02326)0.974.70:0.05984,((((Ult28146:0.04314,(Ult2814
5:5.3E-4,(((Ult28143:0.10278,((Ult28141:5.3E-
4,Ult28142:0.00368)0.936.121:0.03317,(((UltrS351:0.00358,(UltrS352:0.0151
7,Ult28135:0.00385)0.788.96:0.00397)0.774.103:0.0167,(Ult28136:0.02791,(U
lt28137:0.01256,Ult28138:0.02741)0.946.106:0.03617)0.862.120:0.02623)0.79
8.87:0.01633,(Ult28139:5.4E-
4,Ult28140:0.01091)1.000.860:0.08594)0.449.27:0.01124)0.929.118:0.05074)0
.323.11:0.01781,UltCh208:0.05858)0.817.63:0.02738,(Ult28144:0.04448,UltPl
560:0.05707)0.906.142:0.02681)0.878.115:0.02052)0.964.114:0.02993)0.850.1
25:0.0169,((Ult28151:0.00236,(Ult28150:0.0078,(Ult28148:0.00773,(Ult28147
:0.02039,Ult28149:0.06241)0.023.4:0.00835)0.805.73:0.00703)0.924.122:0.01
642)0.993.97:0.05204,(UltCh209:5.4E-
4,UltCh210:0.0564)0.957.110:0.02535)0.489.14:0.01577)0.884.129:0.01441,((
((Ult28015:0.08261,(UltCh161:0.04788,(UltrS348:0.02221,(UltCh160:0.01391,
UltAna23:0.06039)0.959.113:0.04641)0.837.82:0.03336)0.956.110:0.05115)0.8
48.103:0.02051,(((Ult28002:0.04507,((Ult28000:0.01503,Ult28001:5.5E-
4)0.849.109:0.01813,(Ult27999:0.01196,(Ult27997:5.5E-4,(Ult27996:5.5E-
4,Ult27998:0.03018)0.975.94:0.01657)0.867.131:0.02126)0.832.89:0.02339)0.
855.114:0.01911)0.972.101:0.03759,((UltCh159:0.01785,(Ult27990:0.03383,Ul
t27991:0.04319)0.815.73:0.01457)0.995.117:0.06433,((((Ult27988:0.00541,(U

ltCh158:0.01905,(Ult27987:0.00747,Ult27989:0.00763)0.000.913:5.1E-
4)0.968.100:0.02139)0.930.114:0.0158,(Ult27993:5.5E-
4,Ult27994:0.00369)0.917.115:0.01341)0.704.24:0.0034,Ult27992:0.01749)0.2
70.4:0.00348,UltCh162:0.06656)0.807.67:0.01057)0.726.33:0.00403)0.748.76:
0.01366,(((UltOr309:0.00394,UltOr310:0.02338)0.943.114:0.03624,(UltCh180:
0.03298,Ult28071:0.0031)0.921.132:0.03092)0.989.91:0.06426,(Ult28004:0.05
765,(Ult28003:0.01811,(UltSlu37:0.02358,UltSlu38:0.01118)0.861.138:0.0097
)0.917.116:0.02853)0.872.126:0.0326)0.958.124:0.04267)0.856.107:0.02272)0
.759.104:0.00969,(Ult28019:0.02103,((Ult28020:0.01127,(Ult28022:0.00309,U
lt13230:0.02027)0.940.117:0.01219)0.746.66:0.00368,Ult28021:5.4E-
4)0.807.68:0.01901)0.998.155:0.11128)0.964.115:0.04555,((((UltCh179:0.091
51,(Ult28011:0.09443,(UltB1447:5.5E-
4,UltCh165:0.01888)0.836.90:0.01625)0.920.145:0.02755)0.906.143:0.02702,(
((((Ult28009:0.02805,UltOr304:0.04634)0.969.91:0.04142,UltOr306:0.03698)0
.162.8:0.00829,Ult28010:0.03911)0.775.99:0.02371,(UltCh166:0.13781,((Ult2
8058:0.05831,((UltCh177:0.01883,(Ult28065:0.07812,(Ult28069:0.09785,(UltC
h175:0.08643,((Ult28063:0.0114,Ult28064:5.4E-
4)0.907.129:0.02744,(Ult28068:0.06229,(Ult28067:0.02437,(Ult28066:0.00917
,UltCh176:0.03885)0.812.72:0.00915)0.627.8:0.0146)0.747.73:0.02008)0.517.
8:0.014)0.745.88:0.01269)0.184.10:0.02189)0.846.126:0.03774)0.980.79:0.08
085,UltOr307:0.06762)0.905.123:0.03974)0.858.123:0.01902,(((Ult28060:0.00
422,((Ult28059:0.00343,(UltrS350:5.1E-
4,UltCh173:0.00367)0.796.70:0.00392)0.436.13:0.00367,UltCh174:5.5E-
4)0.803.75:0.00663)0.986.91:0.07147,(Ult28061:0.01604,Ult28062:0.01567)0.
937.121:0.04381)0.994.113:0.09056,((Ult28056:0.00757,(Ult13229:0.00383,Ul
t28057:0.04571)0.980.80:0.03151)0.995.118:0.09951,(UltCh172:0.03064,(Ult2
8054:5.5E-
4,Ult28055:0.01927)0.780.120:0.01256)0.979.101:0.05611)0.880.141:0.03067)
0.081.10:0.00774)0.935.117:0.04459)0.923.165:0.04334)0.855.115:0.02587,(U
lt28008:0.01341,(Ult28005:5.4E-
4,(UltCh163:0.01535,(Ult28006:0.02178,Ult28007:0.02175)0.751.67:0.00689)0
.958.125:0.01554)0.811.70:0.00963)0.993.98:0.0553)0.394.19:0.00153)0.896.
118:0.01896,Ult28023:0.11541)0.769.100:5.4E-
4,((UltThe17:0.08449,(Ult28018:0.04629,(Ult28017:0.01587,Ult28016:0.04182
)0.842.112:0.03337)1.000.861:0.10003)0.942.113:0.0428,(UltCh164:0.05368,(
Ult28070:0.05024,(UltOr308:0.06425,UltCh178:0.04104)0.872.127:0.02401)0.9
44.101:0.03615)0.919.158:0.03101)0.841.98:0.01488)0.870.114:0.02058)0.830
.90:0.01595)0.756.94:0.00595,((UltChl58:0.08782,((((((Ult28125:0.07949,Ul
t28124:0.04958)0.042.8:0.00593,(((Ult28104:0.00775,(((UltCh203:0.06743,(U
lt28111:0.09626,(Ult28106:0.04106,(Ult28117:5.4E-
4,Ult28118:0.14332)0.995.119:0.08434)0.365.13:0.02851)0.140.6:0.0062)0.90
7.130:0.0171,((Ult28105:0.02998,(((Ult28108:0.02016,Ult28109:0.01203)0.68
8.24:0.0114,Ult28103:0.04174)0.297.5:0.0074,Ult28110:0.02123)0.027.6:0.00
609)0.732.36:0.00734,((Ult28119:0.0,Ult28120:0.0):0.0188,Ult28121:0.02653
)0.983.79:0.03589)0.759.105:0.00753)0.807.69:0.00961,Ult28107:0.06195)0.7
63.116:0.00675)0.814.73:0.01593,((Ult28116:0.02918,(UltCh202:0.05126,((Ul
t28112:0.0,Ult28114:0.0,Ult28115:0.0):5.5E-
4,Ult28113:0.02257)0.850.126:0.01577)0.960.106:0.04362)0.992.103:0.05977,
Ult28122:0.04444)0.774.104:0.0118)0.895.134:0.02751,Ult28102:0.03276)0.97
0.98:0.05172)0.401.14:0.01582,Ult28123:0.06753)0.990.107:0.06513,(Ult2813
2:0.0166,Ult28133:0.01797)0.989.92:0.05777)0.518.13:0.0135,(((((UltCh186:
0.05058,(Ult28096:0.04828,(Ult28084:0.04414,(Ult13234:0.00751,(Ult28086:0
.01891,Ult28085:0.0038)0.904.130:0.01116)0.595.9:5.4E-
4)0.972.102:0.04392)0.070.12:0.01912)0.802.77:0.00893,Ult28088:0.05082)0.

892.121:0.01249,((((Ult28100:0.02434,(Ult28093:0.02223,((Ult28089:0.0,Ult
28092:0.0):0.00325,(Ult28090:0.0,Ult28091:0.0):5.4E-
4)0.450.16:0.0126)0.913.142:0.01695)0.705.23:0.0016,((UltCh194:0.0323,(Ul
tCh193:0.01024,Ult28097:0.02958)0.933.138:0.0224)0.698.29:0.00471,(((UltC
h190:0.0,UltOr311:0.0,UltCh192:0.0):5.5E-4,UltCh191:5.5E-
4)0.911.149:0.01795,((Ult28101:0.01153,UltCh183:0.07157)0.855.116:0.01442
,((UltCh199:0.0,UltCh201:0.0):5.4E-
4,UltCh200:0.011)1.000.862:0.04534)0.932.125:5.5E-
4)0.908.144:0.01295)0.985.99:0.02943)0.723.42:5.5E-
4,(((UltCh188:0.0248,UltCh195:0.02269)0.291.15:0.0048,(UltCh288:0.01957,(
UltCh184:0.01373,UltCh185:0.0229)0.743.76:0.00601)0.899.122:0.01862)0.786
.96:0.00736,(((UltCh181:6.3E-
4,UltCh182:0.01074)0.929.119:0.02267,(((UltCh196:0.0,UltCh197:0.0):0.0332
6,Ult28098:0.01302)0.972.103:0.03497,(Ult28087:0.00373,Ult28099:0.00366)0
.891.104:0.0146)0.763.117:0.00679)0.834.86:0.00926,(UltOr312:0.03066,(Ult
13233:0.08905,UltCh198:0.01211)0.756.95:0.00629)0.668.23:5.4E-
4)0.942.114:0.01359)0.831.84:0.00117)0.946.107:0.01961,UltCh189:0.02246)0
.840.109:0.00792)0.235.12:0.00556,((Ult28094:0.01754,Ult28095:0.03889)0.9
84.86:0.05542,UltCh187:0.06993)0.706.29:0.01572)0.784.101:0.01069,UltSlu4
1:0.10263)0.931.128:0.02609)0.884.130:0.02226,((((UltCh207:5.4E-
4,UltOr314:0.01109)0.971.82:0.04014,(UltCh205:0.01732,(UltOr313:5.5E-
4,UltCh206:0.00369)0.749.93:0.00159)0.805.74:0.01779)0.831.85:0.02014,(Ul
tCh204:0.01494,Ult28134:0.06645)0.849.110:0.03731)0.995.120:0.07942,(Ult2
8539:0.03994,((Ult28130:5.4E-4,(Ult28129:5.4E-
4,(Ult28126:0.0,Ult28127:0.0):0.00748)0.427.16:0.00735)0.909.117:0.0182,(
Ult28128:0.00406,Ult28131:0.01931)0.982.78:0.03479)0.949.118:0.02975)0.98
7.123:0.04449)0.788.97:0.01157)0.703.32:0.01887)0.744.72:0.03476,((Ult280
53:0.03478,(Ult28052:0.03412,UltCh171:0.04861)0.885.121:0.02121)0.998.156
:0.08692,((((((Ult28045:0.11198,Ult28051:0.02069)0.645.16:0.03268,(((Ult2
8044:5.4E-
4,(Ult28043:0.00365,UltSlu40:0.00754)0.930.115:0.01119)0.851.117:0.00803,
(Ult13231:0.03355,(Ult28042:0.09207,(Ult28041:0.00661,UltCh170:0.02062)0.
661.17:0.00883)0.686.18:0.00246)0.890.132:0.01058)0.917.117:0.03415,(Ult2
8028:0.11606,(UltAnt11:0.0212,Ult28027:0.0364)0.874.147:0.03694)0.976.84:
0.05699)0.878.116:0.02298)0.775.100:0.01033,((((UltCh167:0.02201,Ult28024
:0.01031)0.990.108:0.0864,(UltCh168:0.07079,Ult28026:0.12466)0.716.30:0.0
4072)0.379.13:0.02397,(Ult28040:0.05007,(Ult28038:0.03945,Ult28039:5.5E-
4)0.983.80:0.05848)0.864.117:0.02945)0.844.121:0.02785,(Ult28025:0.09575,
(UltSlu39:0.09643,(Ult28048:5.5E-
4,Ult28049:0.00368)0.986.92:0.07138)0.886.149:0.03291)0.865.126:0.02521)0
.443.13:0.00254)0.906.144:0.02207,(Ult28046:0.03392,Ult28047:0.02295)1.00
0.863:0.08377)0.700.27:5.4E-
4,Ult28050:0.1158)0.887.132:0.03195,(((UltCal16:5.4E-4,(Ult28033:5.4E-
4,Ult28034:0.01903)0.819.81:0.00369)0.727.41:0.00521,(Ult28029:0.00742,(U
lt28031:0.00369,(Ult28030:0.0227,Ult28032:0.00743)0.760.102:5.4E-
4)0.986.93:5.5E-
4)0.768.86:0.01814)0.987.124:0.05109,(UltAc393:0.06872,((Ult28036:0.00718
,Ult28037:5.4E-4)0.776.100:0.01798,((Ult28035:5.5E-
4,UltCh169:0.00369)0.955.99:0.03081,(UltGa297:0.04517,UltrS349:0.00422)0.
983.81:0.04396)0.911.150:0.0293)0.907.131:0.03157)0.829.105:0.02217)0.957
.111:0.03317)0.714.30:0.00696)0.935.118:0.04783)0.910.119:0.02643)0.981.9
6:0.03518)0.400.14:0.01842)0.859.113:0.01861,(((((((Ult29266:5.3E-
4,(Ult29267:0.00377,(Ult29249:5.4E-
4,UltCh504:0.03515)0.608.21:0.00727)0.886.150:0.00749)0.973.92:0.01519,((

(UltCh503:5.4E-4,(((Ult29247:0.01156,Ult29246:5.5E-
4)0.927.119:0.00159,(Ult29245:0.00388,(Ult29248:0.01519,(((UltCh505:0.022
94,Ult29299:0.02724)0.752.83:0.00423,Ult29244:5.4E-
4)0.907.132:0.00746,(Ult29239:0.01987,(Ult29255:0.01167,UltCh508:0.02283)
0.807.70:0.0077)0.779.101:0.00419)0.852.117:5.5E-
4)0.256.11:0.00392)0.906.145:0.00234)0.930.116:0.01158,(Ult29236:0.0037,U
lt29238:5.4E-4)0.837.83:0.00369)0.522.6:5.4E-4)0.621.15:5.4E-
4,(Ult29235:5.4E-
4,(Ult29265:0.03482,(Ult29243:0.00366,(Ult29237:0.00369,(((Ult29234:5.5E-
4,Ult29242:5.5E-4)0.677.17:5.5E-
4,((Ult29231:0.00458,Ult29233:0.05182)0.911.151:0.01442,(Ult29252:0.0037,
(Ult29270:5.5E-4,(((Ult29254:0.00383,(Ult29253:5.5E-4,UltCh506:5.5E-
4)0.901.128:0.0112)0.775.101:0.00756,(Ult29251:0.00371,Ult29250:0.01133)0
.979.102:5.3E-
4)0.912.151:0.00187,UltCh507:0.00191)0.922.172:0.01085)0.851.118:0.00722)
0.854.110:5.5E-4)0.903.114:0.00741)0.594.16:5.4E-
4,(Ult29240:0.00247,Ult29241:0.00247)0.924.123:0.00246)0.957.112:0.01121)
0.193.8:5.4E-4)0.822.72:0.00365)0.044.7:5.4E-
4)0.901.129:0.00737)0.898.144:0.00781,Ult29232:5.3E-
4)0.795.89:0.00393)0.886.151:0.00738,(Ult29269:0.01562,((UltB4795:0.00755
,(Ult29262:0.01566,Ult29263:0.0154)0.920.146:0.01178)0.866.130:0.00777,(U
lt29264:0.01767,(((Ult29256:0.0043,Ult29257:0.01083)0.438.17:0.0099,((Ult
29258:0.00387,(Ult29261:5.4E-4,(Ult29260:5.3E-
4,Ult29259:0.02742)0.214.12:0.0074)0.977.89:0.02318)0.831.86:0.01513,UltG
ram9:0.00792)0.402.14:0.01319)0.970.99:0.03289,Ult29268:0.00897)0.838.110
:0.02692)0.767.90:0.00613)0.922.173:0.01153)0.664.20:5.5E-
4)0.955.100:0.03316,Ult29271:0.01463)0.895.135:0.0327,((Ult29298:0.04226,
((Ult29291:0.01576,(Ult29295:0.03246,(Ult29296:0.01533,Ult29297:0.01542)0
.997.123:0.08393)0.834.87:0.03891)0.748.77:0.00775,((Ult29288:0.0,Ult2928
9:0.0,Ult29294:0.0):0.00714,((Ult29292:0.0,Ult29293:0.0):0.02366,Ult29290
:0.01158)0.209.15:5.5E-
4)0.843.100:0.01916)0.997.124:0.09904)0.955.101:0.06533,(Ult28396:0.0904,
((UltAna26:5.5E-
4,UltCh509:0.00369)0.942.115:0.01138,(Ult29287:0.03958,(UltCh510:0.00715,
(Ult29283:0.01454,(Ult29286:0.03566,(Ult29284:0.0,Ult29285:0.0):0.00373)0
.755.82:0.00451)0.925.136:0.01537)0.791.87:0.00436)0.743.77:0.00394)0.988
.103:5.5E-
4)0.905.124:0.04231)0.760.103:0.01844)0.958.126:0.05461,((UltEnd12:0.0107
5,((UltCh310:0.07316,UltCh311:0.09237)0.889.129:0.04052,(UltCh309:0.0232,
(Ult28395:5.5E-
4,(UltCh307:0.03642,UltCh308:0.02149)0.998.157:0.09162)0.996.118:0.09177)
0.985.100:0.05768)0.654.16:0.02569)1.000.864:0.18747,(UltCh297:0.03514,((
((UltCh321:0.00366,((((Ult28403:0.05069,Ult28405:0.02351)0.739.54:0.00681
,UltOr378:0.03759)0.864.118:0.01635,((UltCh331:5.4E-
4,UltCh330:0.03098)0.986.94:0.02312,(((((((Ult28438:0.0,Ult28439:0.0,Ult2
8440:0.0):0.00753,Ult28437:0.00377)0.870.115:5.4E-
4,(((Ult28441:0.0,Ult28442:0.0):5.5E-
4,Ult28443:0.0075)0.899.123:0.01464,(UltCh315:5.5E-4,(UltCh313:5.4E-
4,UltCh314:0.01141)0.811.71:0.0035)1.000.865:0.12997)0.718.33:0.00444)0.8
68.105:0.01705,((UltCal18:0.00434,UltCal19:0.02113)0.971.83:0.03982,(UltC
al23:0.0231,CdlAerop:5.3E-
4)0.987.125:0.04352)0.761.81:0.01241)0.043.12:0.01716,(Ult28411:0.0429,(U
lt28412:0.0087,(Ult28409:0.00747,((Ult28410:0.0,UltCh324:0.0):5.5E-
4,UltAna24:0.00371)0.985.101:5.5E-

4)0.961.103:0.03147)0.948.131:0.02669)0.923.166:0.03453)0.771.119:0.0119,
((Ult28450:0.0,Ult28451:0.0):0.1135,(Ult28413:5.3E-
4,(Ult28415:0.01514,Ult28414:5.3E-
4)0.947.96:0.01106)0.870.116:0.01993)0.852.118:0.02)0.761.82:0.00377,((Ul
t28436:0.02203,(Ult28435:0.07591,((UltrS358:0.00325,(Ult28431:5.4E-
4,Ult28432:0.0171)0.840.110:0.0141)1.000.866:0.14057,(Ult28434:0.07262,Ul
t28446:0.09623)0.410.15:0.03699)0.946.108:0.04228)0.031.5:5.4E-
4)0.877.139:0.01272,(((((Ult28400:0.04693,(Ult28398:0.04168,Ult28399:0.00
99)0.916.139:0.02741)0.875.137:0.0233,((Ult28444:0.02838,(UltCal22:0.0078
3,(Ult28445:0.01552,ChfBacte:0.01161)0.908.145:0.01561)0.734.66:0.00351)0
.926.142:0.02079,((Ult28433:0.01971,UltCh329:0.01184)0.987.126:0.03982,Ul
t28416:0.02841)0.854.111:0.01588)0.819.82:0.01261)0.782.116:0.0095,(Ult28
430:0.02784,UltCh328:0.028)0.960.107:0.02655)0.822.73:0.01458,((UltCh325:
5.4E-4,Ult28423:0.01516)0.880.142:0.00761,(UltCh326:5.4E-
4,((Ult28424:0.00391,Ult28425:0.00733)0.974.71:0.02795,((UltrS357:0.00453
,(Ult28419:0.01619,(UltCh327:0.01167,(((Ult28422:0.00754,(Ult28420:0.0233
4,Ult28421:0.01541)0.881.135:0.01119)0.752.84:0.00379,(UltCal20:0.01171,U
ltCal21:0.02757)0.941.104:0.01521)0.769.101:0.00382,(Ult28418:5.4E-
4,(Ult28427:5.5E-
4,Ult28428:0.00369)0.946.109:0.01115)0.903.115:0.01133)0.754.84:0.00338)0
.990.109:0.02517)0.677.18:0.00232)0.950.103:0.01637,Ult28426:0.01215)0.68
9.21:0.00338)0.921.133:0.01565)0.764.107:0.00355)0.971.84:0.02033)0.745.8
9:0.00733,(UltCh323:0.02533,(UltCh316:0.02696,Ult28429:0.06662)0.549.19:0
.01895)0.825.82:0.01078)0.728.44:0.00418)0.925.137:0.01103)0.694.22:5.4E-
4)0.891.105:0.01221)0.718.34:0.00715,((UltCh312:0.00709,(Ult28401:0.00917
,Ult28402:0.03562)0.983.82:0.03733)0.972.104:0.02619,(Ult28404:0.03641,Ul
t28407:0.02888)0.726.34:0.00416)0.254.8:0.01012)0.906.146:0.01554)0.729.3
2:0.00339,UltCh322:0.01158)0.817.64:0.00669,(UltCh320:0.03946,Ult28408:0.
01922)0.804.70:0.00281)0.787.94:0.00228,(Ult28406:0.03492,(UltCh319:0.035
65,(UltCh317:5.5E-
4,UltCh318:0.00368)0.964.116:0.03491)0.995.121:0.06462)0.871.131:0.01779)
0.941.105:0.02942)0.820.69:0.03551)0.868.106:0.05995)0.115.6:0.0104,(((Ul
t28940:0.01481,Ult28941:0.00799)0.549.20:0.01134,(UltThe18:0.00686,(UltCh
436:0.00486,Ult28942:0.0518)0.759.106:0.01106)0.445.18:0.02123)1.000.867:
0.13643,(UltCh114:0.04252,((Ult27871:0.01467,((Ult27873:0.01546,(Ult27874
:0.0197,(UltOr301:0.00764,((UltOr302:0.0,UltCh116:0.0):5.5E-
4,UltCh115:0.00745)0.843.101:0.00735)0.966.122:0.02021)0.820.70:0.00732)0
.932.126:0.01976,(Ult27869:0.0503,Ult27870:0.01065)0.847.144:0.01192)0.89
7.125:0.02117)0.043.13:0.0109,Ult27872:0.02541)0.948.132:0.03398)0.911.15
2:0.03061)0.932.127:0.04193)0.677.19:5.5E-
4)0.793.92:0.01374,(((UltCh275:0.05425,(Ult32230:0.08758,((Ult28224:0.0,U
lt28225:0.0):0.00479,Ult28226:0.01061)0.915.130:0.03525)0.942.116:0.04051
)0.934.110:0.03295,((((((((ClfBacte:0.04059,((((((((((UltCh548:0.44116,
UltOr387:5.4E-
4)0.972.105:0.04575,(UltCh547:0.03794,HtlBact2:0.04601)0.432.18:0.01289)0
.839.101:0.02409,UltOr388:0.02649)0.431.21:0.01402,(UltCh542:0.03509,(Ult
Ch543:0.03541,(Ult29389:0.02076,Ult29388:0.03786)0.283.9:0.01196)0.177.16
:0.01389)0.984.87:0.0469)0.981.97:0.05308,((UltCh540:0.00376,UltCh541:0.0
2663)0.985.102:0.03662,((((UltCh537:0.02632,UltCh536:0.0368)0.472.15:0.01
205,UltCh539:0.04614)0.687.23:0.01025,((UltCh533:5.5E-
4,UltCh534:0.00369)0.991.106:0.04177,UltCh538:0.03855)0.743.78:0.00478)0.
942.117:0.02114,UltCh535:0.03733)0.938.82:0.02956)0.141.8:0.01346)0.770.1
13:0.00884,((Ult29390:0.0,Ult29391:0.0):0.05931,(UltCh549:5.5E-
4,UltOr389:0.00368)0.969.92:0.02846)0.841.99:0.01055)0.896.119:0.018,((Ul

t29395:0.00888,((Ult29396:0.088,Ult29397:0.03985)0.823.88:0.05958,(Ult293
92:0.04014,Ult29394:0.04523)0.027.7:0.01146)0.754.85:0.01802)0.916.140:0.
02404,Ult29393:0.04667)0.980.81:0.0395)0.897.126:0.01736,(UltCh532:0.0154
8,(OslTrich:5.3E-
4,OslSpeci:0.00714)0.947.97:0.02498)0.958.127:0.03348)0.897.127:0.02621,(
UltCh531:0.05375,(((ChxAggre:0.00796,Ult29385:0.04612)0.881.136:0.01819,C
hxSpeci:0.02196)0.905.125:0.02991,(ChxAuran:0.0026,(Ult29386:0.03771,Ult2
9384:0.06493)0.952.107:0.02267)0.755.83:0.01178)0.987.127:0.06308)0.662.2
0:0.03135)0.050.9:5.4E-
4,(UltCh530:0.03209,Ult29387:0.08216)0.792.104:0.01364)0.754.86:0.02204,(
(HtlBacte:0.01014,UltCh546:0.06237)0.988.104:0.06505,(UltCh544:0.03214,Ul
tCh545:0.14631)0.537.15:0.02342)0.591.17:0.03139)0.748.78:0.00944)0.950.1
04:0.03487,((Ult29365:0.01095,(Ult29311:5.5E-
4,Ult29366:0.00367)0.983.83:5.4E-
4)1.000.868:0.16246,(((Ult29378:0.01518,Ult29381:0.00375)0.879.140:0.0095
3,(Ult29377:0.0136,(Ult29376:0.00739,Ult29380:5.3E-
4)0.881.137:0.01047)0.752.85:0.00426)0.737.74:0.00461,(Ult29379:0.03765,(
Ult29382:0.00698,Ult29383:0.0044)1.000.869:0.07612)0.179.7:5.4E-
4)0.412.14:0.03503)0.992.104:0.09034)0.905.126:0.03434,Ult29398:0.07948)0
.883.135:0.03243,((Ult29370:0.10498,(Ult29371:0.09204,(Ult29369:0.04412,(
UltrS384:0.04775,Ult29368:0.0137)0.930.117:0.03981)0.961.104:0.04785)0.32
2.14:0.01778)0.651.21:0.02741,((Ult29358:0.0198,(Ult29310:0.12965,(((Ult2
9373:0.0,Ult29374:0.0):0.00369,Ult29375:5.4E-4)0.991.107:5.3E-
4,(((((UltCh528:0.00759,(UltCh526:0.00367,UltCh527:0.00375)0.479.19:5.4E-
4)0.890.133:5.5E-4,(UltCh519:0.02704,((UltCh522:5.5E-
4,((UltCh525:0.02343,(UltCh523:5.5E-
4,UltCh520:0.00759)0.821.75:0.00374)0.947.98:5.5E-
4,UltCh524:0.00369)0.569.13:5.5E-4)0.356.15:5.5E-4,UltCh518:5.5E-
4)0.917.118:5.5E-
4)0.797.80:0.00693)0.756.96:0.00957,(RsfCaste:0.02181,UltCh529:0.03795)0.
417.19:0.00703)0.926.143:0.02769,(RsfSpeci:5.5E-
4,Ult29372:0.00742)0.866.131:0.01415)0.776.101:0.01135,UltCh521:0.1897)0.
598.9:0.0086)0.997.125:0.12814)0.939.114:0.07257)0.861.139:0.02503,(((Ult
Rosei:0.03305,UltCh517:0.03373)0.999.174:0.15467,((Ult29367:0.06412,(Ult2
9362:0.24843,((Ult29359:0.02687,Ult29360:5.4E-
4)0.999.175:0.07936,(Ult29363:0.01459,(Ult29364:0.01982,Ult29361:0.12579)
0.861.140:0.02279)0.832.90:0.02881)0.746.67:0.01034)0.830.91:0.02669)0.76
0.104:0.03045,((Ult29355:0.09521,((((Ult29351:0.01133,Ult27550:0.17031)0.
969.93:0.05037,(EikeType:0.02742,UltCh516:0.0192)0.912.152:5.5E-
4)0.962.118:0.01911,Ult29350:0.00811)0.751.68:0.00679,ChfBact4:0.0127)0.9
97.126:0.04933)0.133.11:5.3E-4,(((Ult29356:5.4E-
4,((Ult29340:0.00388,((Ult29341:5.4E-4,(UltrS383:0.01521,Ult29342:5.4E-
4)0.952.108:0.00747)0.849.111:0.00724,(Ult29338:5.4E-4,(Ult29337:5.5E-
4,Ult29339:0.01127)0.828.74:0.00369)0.902.130:5.4E-
4)0.959.114:0.01524)0.747.74:0.00747,(((((Ult29333:0.01284,(Ult29329:0.00
727,((((Ult29314:0.0,Ult29317:0.0,Ult29318:0.0):0.01139,((Ult29332:5.4E-
4,(Ult29328:5.5E-
4,(Ult29322:0.00528,Ult29327:0.01052)0.970.100:0.01909)0.897.128:0.01509)
0.949.119:0.01528,(Ult29316:0.0076,Ult29330:0.01128)0.948.133:0.0192)0.73
7.75:0.00454)0.852.119:0.00788,Ult29335:0.00748)0.940.118:0.0113,Ult29331
:0.00371)0.879.141:5.3E-
4)0.790.93:0.01316)0.812.73:0.00823,(UltrS381:5.4E-
4,Ult29336:0.01095)0.517.9:0.00751)0.919.159:0.01137,Ult29334:5.5E-
4)0.370.13:0.00374,(((Ult29313:0.00368,(Ult29321:0.0,Ult29323:0.0):5.5E-

4)0.920.147:0.01163,(Ult29324:0.00367,UltrS382:0.00377)0.910.120:0.01155)
0.908.146:0.01168,(Ult29315:0.00368,(Ult29320:0.0,Ult29325:0.0):5.5E-
4)0.893.135:5.4E-
4)0.865.127:0.00755)0.897.129:0.01072,(Ult29319:0.00752,(Ult29326:0.00376
,Ult29343:0.01129)0.886.152:0.00752)0.875.138:5.5E-
4)0.861.141:0.02445)0.996.119:0.06534)0.878.117:0.03267,(Ult29352:0.05518
,(Ult29353:0.00377,Ult29354:0.02303)0.265.5:0.00365)0.893.136:0.01654)0.5
43.13:0.01292,(((Ult29349:0.04801,(Ult29312:0.00289,Ult29348:0.00466)0.86
8.107:0.01779)0.969.94:0.03846,Ult29344:0.00349)0.859.114:0.02497,(Ult293
45:0.01169,(Ult29346:5.5E-
4,Ult29347:0.10254)0.942.118:0.02314)0.732.37:0.00289)0.919.160:0.03909)0
.926.144:0.03248)0.941.106:0.04363)0.924.124:0.04903)0.694.23:0.01545,(Gr
eNonSu:0.09092,Ult29357:0.13374)0.561.13:0.01542)0.614.11:0.01362)0.972.1
06:0.07409)0.994.114:0.10015)0.938.83:0.06191,((Ult29211:0.10051,(Ult2807
9:0.18392,(Ult28078:0.03825,Ult28077:0.06929)0.632.14:0.01091)0.957.113:0
.05958)0.860.95:0.04104,((Ult29399:0.07666,(UltCh550:0.055,(HrpAuran:0.00
34,HrpGeyse:5.5E-
4)0.966.123:0.07163)0.912.153:0.05667)0.984.88:0.09467,((Ult29410:0.09233
,(Ult29411:0.1032,(Ult29409:0.02819,(Ult29406:0.02497,(Ult29407:0.07608,U
lt29408:5.4E-
4)0.925.138:0.02611)0.859.115:0.02911)0.993.99:0.08849)0.834.88:0.03823)0
.689.22:0.01008,(Ult29405:0.09687,(((Ult29403:0.03323,Ult29404:0.07282)0.
400.15:0.00928,(UltrS385:0.03663,Ult29400:0.14536)0.763.118:0.03791)0.822
.74:5.5E-
4,((Ult29402:0.02347,UltEnd13:0.0075)0.979.103:0.05183,Ult29401:0.1069)0.
392.14:0.01631)0.861.142:0.03465)0.834.89:0.04734)0.983.84:0.1003)0.787.9
5:0.03882)0.556.12:0.04417)0.840.111:0.03596,(((((Ult29198:0.10165,(((Ult
rS377:0.01173,UltCa160:0.04092)0.971.85:0.0321,(Ult29188:0.0,Ult29196:0.0
):5.5E-4)0.426.23:5.5E-
4,Ult29187:0.03888)0.000.914:0.01293)0.831.87:0.00624,((Ult29189:0.10522,
UltrS375:0.06174)1.000.870:0.17832,(Ult29190:0.09025,(UltrS376:0.00375,(U
lt29191:0.01068,((Ult29197:0.03049,(Ult29194:0.00731,(Ult29192:5.5E-
4,Ult29193:0.00369)0.957.114:0.01577)0.899.124:0.01258)0.397.20:0.00427,U
lt29195:0.02591)0.798.88:0.00844)0.816.78:0.00854)0.821.76:0.01067)0.412.
15:0.0061)0.690.27:5.4E-
4)1.000.871:0.13676,(((Ult29436:0.00744,(Ult29437:0.0,UltCh555:0.0):5.4E-
4)0.949.120:0.03922,(UltCh554:0.06148,UltEnd14:0.02863)0.934.111:0.03674)
0.993.100:0.08455,(((((Ult29435:0.0182,(Ult29434:0.02328,((Ult29432:0.003
67,UltCh552:5.5E-
4)0.998.158:0.04777,(UltCh553:0.00369,Ult29433:0.0037)0.944.102:5.4E-
4)0.928.107:0.01531)0.904.131:0.0184)0.909.118:0.01822,Ult29426:0.02676)0
.484.21:0.02117,(((UltCh551:0.04976,Ult29427:0.02328)0.353.10:0.02232,(Ul
t29430:0.06219,(Ult29420:0.0193,(((((Ult29413:0.00742,(Ult29412:5.5E-
4,Ult29417:0.00369)0.314.12:5.3E-
4)0.804.71:0.0071,(Ult29416:0.00736,(Ult29414:0.00369,Ult29415:5.5E-
4)0.774.105:5.5E-4)0.970.101:5.4E-
4)0.989.93:0.03792,(Ult29418:0.00646,(Ult29419:0.00992,UltrS386:0.02103)0
.883.136:0.01895)0.677.20:0.02141)0.857.127:0.0083,(Ult29421:5.5E-
4,(Ult29423:0.01894,Ult29422:0.00369)0.840.112:5.5E-4)0.633.10:5.4E-
4)0.848.104:0.01089,UltThe19:0.01597)0.964.117:0.02994)0.903.116:0.01793)
0.872.128:0.01826)0.875.139:5.3E-
4,(Ult29424:0.00595,(Ult29431:0.03096,Ult29425:5.5E-
4)0.416.29:0.0097)0.884.131:0.00918)0.780.121:0.01157)0.863.137:0.01323,(
Ult29428:0.0082,Ult29429:0.0112)0.955.102:0.03256)0.791.88:0.01167,(UltrS

387:0.10612,((UltCh558:0.06356,(ShaTherm:0.01458,(Ult29445:0.03046,(TemBa
cte:0.02318,((TbmRoseu:0.0,UltThe20:0.0):0.00371,Ult29444:0.00749)0.986.9
5:0.02451)0.958.128:5.4E-
4)0.997.127:0.07431)0.841.100:0.01569)0.840.113:0.01756,((Ult29441:0.0310
2,((Ult29438:0.00678,Ult29439:0.0258)0.950.105:0.03239,Ult29440:0.01758)0
.797.81:0.01241)0.733.48:0.01259,(Ult29442:0.07302,((UltCh556:0.0,UltSpha
e:0.0,UltAl201:0.0):0.04938,(Ult29443:5.5E-4,UltCh557:5.5E-
4)0.816.79:0.01786)0.880.143:0.02569)0.691.16:0.03155)0.977.90:0.05666)0.
887.133:0.03396)0.912.154:0.04021)0.000.915:0.00518)0.917.119:0.03787)0.2
82.10:0.02388,(((Ult31359:0.03076,(Ult31360:5.4E-
4,(Ult31357:0.04053,((Ult31356:5.4E-
4,(Ult31355:0.00916,UltCa383:0.02652)0.988.105:0.03518)0.257.6:0.00306,Ul
t31358:0.05417)0.398.10:0.00459)0.750.70:0.00376)0.893.137:0.02592)0.938.
84:0.06261,(Ult29450:0.09898,(Ult29184:0.3064,Ult29449:0.07446)0.911.153:
0.09106)0.950.106:0.07804)0.782.117:0.07461,(Ult29448:0.11873,(Ult29446:0
.05893,Ult29447:0.02815)0.844.122:0.04056)0.912.155:0.05022)0.889.130:0.0
2886)0.738.55:0.00677,((Ult28900:0.08332,UltrS379:0.12872)0.845.116:0.026
69,((Ult29209:0.0318,Ult29210:0.00822)0.986.96:0.06184,(Ult29200:0.07959,
(Ult29199:0.05705,Ult29202:0.06198)0.611.11:0.02076)0.911.154:0.03711)0.8
57.128:0.04229)0.907.133:0.03289)0.464.17:0.0118)0.850.127:0.02082,((((Ul
t29015:0.03057,((Ult29011:5.5E-
4,(Ult29010:0.00372,Ult29014:0.01902)0.298.14:0.00372)0.924.125:0.00719,(
(Ult29016:0.01915,(Ult29012:0.00754,Ult29013:0.02342)0.855.117:0.00759)0.
518.14:0.00374,Ult29009:0.02685)0.805.75:5.3E-
4)0.928.108:0.02741)0.888.138:0.01617,((Ult29008:0.01908,((UltCh473:0.013
03,(UltCh472:0.01472,(Ult29017:0.02002,UltCh471:0.0029)0.843.102:0.01325)
0.943.115:0.02884)0.941.107:0.02901,(Ult29020:0.0469,(UltGa303:5.5E-
4,UltCh474:0.00748)0.913.143:0.01802)0.797.82:0.00823)0.756.97:0.00628)0.
906.147:0.01487,Ult29019:0.02182)0.681.19:0.01004)0.995.122:0.06747,((((U
lt29453:0.02413,Ult29454:0.02455)1.000.872:0.09036,(TeoTerre:0.05905,Ult2
9452:0.06057)0.890.134:0.01919)0.600.14:0.00507,Ult29457:0.07063)0.839.10
2:0.02078,(Ult29455:0.00361,Ult29456:5.5E-
4)0.969.95:0.05193)0.979.104:0.05831)0.355.18:0.00325,((Ult28977:0.07735,
((Ult28998:0.12131,(((((Ult28994:0.0037,Ult28995:5.5E-
4)0.824.79:0.00373,(Ult28993:0.01512,(UltrS367:0.00372,(Ult28985:5.4E-
4,(Ult28986:0.00375,((Ult28997:0.07469,(Ult28990:0.0037,(UltCh465:0.0,Ult
28996:0.0):5.5E-4)0.993.101:5.4E-
4)0.074.3:0.00368,UltrS366:0.00372)0.929.120:0.0115)0.776.102:0.00369)0.8
05.76:0.0037)0.677.21:5.4E-4)0.976.85:0.01539)0.748.79:5.5E-
4,((UltCh466:0.0,UltCh467:0.0):0.01153,((Ult28989:0.01537,Ult28988:0.0036
4)0.746.68:0.00378,Ult28992:0.03879)0.851.119:0.00787)0.828.75:0.00808)0.
908.147:0.0141,Ult28991:0.00843)0.958.129:0.04424,(Ult28987:0.04473,UltrS
368:0.10896)0.070.13:0.01875)0.851.120:0.02585)0.976.86:0.05063,(((Ult289
74:0.01939,Ult28975:0.07321)0.932.128:0.04044,(((UltrS365:5.4E-
4,((Ult28981:0.01327,((Ult28980:0.01235,(Ult28978:0.01006,Ult28979:0.0275
6)0.830.92:5.3E-
4)0.904.132:0.01368,Ult28984:0.03637)0.820.71:0.01086)0.929.121:0.01924,U
lt28982:0.02344)0.479.20:0.00746)0.965.98:0.02824,Ult28983:0.02429)0.911.
155:0.02585,(Ult28966:0.06625,(Ult28967:0.03198,(Ult28973:0.04296,(Ult289
63:0.0131,((((UltCh459:0.01538,((Ult28964:0.00373,(Ult28968:0.0,Ult28969:
0.0):0.00752)0.898.145:0.00752,UltCh458:5.5E-4)0.773.108:5.5E-
4)0.801.86:0.00371,(Ult28970:5.5E-
4,(UltCh460:0.00375,(Ult28972:0.01135,UltCh464:0.00753)0.769.102:0.00374)
0.808.78:0.00373)0.782.118:5.4E-

4)0.782.119:0.00382,(UltCh463:0.00374,(Ult28965:0.0189,(UltCh461:0.00373,
UltCh462:0.00748)0.967.87:5.3E-
4)0.918.137:0.01544)0.774.106:0.00371)0.897.130:0.00876,(UltCh457:0.00768
,Ult28971:0.0048)0.137.9:0.00626)0.768.87:0.00647)0.512.7:0.01316)0.595.1
0:0.01935)0.952.109:0.04763)0.943.116:0.0363)0.358.12:0.01094)0.764.108:0
.02222,Ult28976:0.11008)0.767.91:0.01047)0.747.75:0.0101)0.503.7:0.00508,
(UltCh468:0.02616,(UltCh469:0.07195,Ult28999:0.01715)0.700.28:0.00639)0.9
96.120:0.06342)0.905.127:0.02349)0.905.128:0.02526)0.814.74:0.01424,(((Ul
t29308:0.03383,(UltCh513:0.07191,Ult29309:0.08592)0.831.88:0.03482)0.495.
15:0.03083,(((Ult29305:0.05476,(Ult29451:0.11574,(UltCh512:0.06133,(Ult29
302:0.04064,(Ult29301:0.01677,Ult29300:0.05447)0.388.14:0.01192)0.806.69:
0.013)0.982.79:0.07545)0.915.131:0.04329)0.764.109:0.00867,Ult29304:0.056
62)0.819.83:0.01547,(Ult29000:0.07909,Ult29303:0.07172)0.863.138:0.02543)
0.462.17:0.02407)0.663.22:0.02704,(((((((((UltCh500:5.5E-
4,Ult29216:0.01118)0.929.122:0.00744,UltrS380:0.00369)0.778.101:5.5E-
4,(((Ult29212:0.00934,Ult29213:0.02221)0.940.119:0.01782,Ult29214:0.05997
)0.883.137:5.4E-
4,Ult29215:0.00742)0.882.141:0.00737)0.827.87:0.0096,(((UltCa161:0.0074,U
lt29219:5.5E-4)0.767.92:5.5E-
4,(Ult29218:0.01143,UltCh501:0.0075)0.767.93:0.0038)0.874.148:0.0119,Ult2
9217:0.05609)0.453.13:5.5E-
4)0.997.128:0.05053,((Ult29021:0.06455,(Ult28953:0.02255,Ult28954:0.03829
)0.972.107:0.04036)0.932.129:0.02812,(((UltCh493:0.0726,((Ult29205:0.0451
,Ult29204:0.00756)0.865.128:0.028,(UltCh492:0.03115,(UltCh491:0.01928,Ult
Ch494:0.10582)0.808.79:0.01024)0.752.86:0.01439)0.827.88:0.02186)0.865.12
9:0.02066,((Ult29007:0.01511,(UltCh496:0.00768,(Ult29206:5.4E-
4,UltCh495:0.01117)0.217.12:0.00366)0.876.116:0.01164)0.958.130:0.02606,(
UltCh497:0.01544,UltrS378:0.058)0.147.9:0.00819)0.851.121:0.01468)0.958.1
31:0.03314,UltCh498:0.07497)0.750.71:0.00468)0.926.145:0.01677)0.840.114:
5.4E-
4,((Ult29222:0.01447,(Ult29221:0.03649,Ult29220:0.03632)0.869.99:0.01322)
0.981.98:0.03809,(Ult29224:0.0207,Ult29223:0.05729)0.488.18:0.00881)0.791
.89:0.00701)0.921.134:0.01945,((Ult28891:0.04544,Ult29227:0.07346)0.771.1
20:0.02823,((((((UltCh400:0.021,Ult28890:0.05853)0.861.143:0.02801,(Ult28
887:5.3E-
4,(Ult28888:0.01782,Ult13236:0.04726)0.423.17:0.01282)0.775.102:0.00507)0
.713.34:0.00391,(((Ult28872:0.0,Ult28873:0.0):0.02723,(Ult28871:0.02415,(
Ult28869:5.5E-
4,Ult28870:0.01129)0.757.75:0.00291)0.934.112:0.01749)0.938.85:0.01719,(U
lt28874:0.01498,((((Ult28882:0.0,Ult28883:0.0):5.4E-
4,(Ult28877:0.03941,Ult28875:0.02309)0.899.125:5.4E-
4)0.835.94:0.00366,((Ult28876:0.0,Ult28878:0.0):5.5E-
4,Ult28881:0.00369)0.871.132:5.5E-
4)0.924.126:0.01121,(Ult28880:0.01537,Ult28879:0.01135)0.732.38:0.00373)0
.923.167:0.01187)0.688.25:8.7E-
4)0.966.124:0.0198)0.823.89:0.00797,(Ult28889:0.01421,(Ult28886:0.01136,U
lt28885:0.00381)0.918.138:0.01238)0.420.19:0.00591)0.963.95:0.03286,(UltA
na25:0.01614,(Ult29207:0.01267,(UltCh499:0.02307,Ult29208:0.00771)0.978.8
2:0.02649)0.543.14:0.00744)0.756.98:0.00642)0.795.90:0.02212,((Ult28903:0
.00744,(Ult28922:5.5E-
4,(Ult28901:0.00369,Ult28902:0.00369)0.882.142:5.5E-4)0.948.134:5.4E-
4)0.910.121:0.01172,(((Ult28908:0.0157,((Ult28916:0.00799,(UltCh401:0.004
19,Ult28917:0.01876)0.820.72:0.00763)0.892.122:0.01153,(Ult28913:0.0112,(
(Ult28914:0.00209,((Ult28912:0.01138,((Ult28910:0.0,Ult28911:0.0):0.00743

,UltrS363:0.00741)0.526.14:5.4E-
4)0.797.83:0.00532,Ult28918:0.01745)0.684.17:0.00756)0.794.86:0.00534,Ult
13238:0.00743)0.242.11:5.4E-
4)0.770.114:0.0031)0.875.140:0.00765)0.670.15:0.00368,(Ult28904:0.0077,(U
lt28905:0.0,Ult28906:0.0):0.01929)0.756.99:0.00398)0.742.61:0.00277,((Ult
28915:0.0037,(Ult28920:5.4E-
4,(Ult28909:0.01122,(Ult28907:0.00367,Ult28921:5.5E-
4)0.883.138:0.01135)0.919.161:5.4E-4)0.960.108:0.00744)0.060.5:5.4E-
4,(Ult28919:0.0,UltCh402:0.0):0.00369)0.764.110:0.00417)0.835.95:0.00766)
0.945.101:0.02637)0.783.116:0.01106)0.861.144:0.01677)0.868.108:0.01793,U
lt28452:0.03461)0.324.9:0.01487,(Ult29226:0.03752,(Ult29225:0.03193,UltCh
502:0.03618)0.947.99:0.02333)0.986.97:0.05189)0.216.8:0.01427,(((((((UltC
h423:0.01933,Ult28933:0.01949)0.826.77:0.00752,((UltCh419:0.0,UltCh420:0.
0):0.01139,(UltCh421:0.01141,UltCh422:0.01138)0.726.35:0.00376)0.857.129:
0.00758)0.997.129:0.0382,(UltCh431:0.05121,((UltCh434:0.01128,UltCh435:0.
0213)0.406.15:0.00631,((Ult28937:0.0048,(UltCh433:0.02295,UltCh432:0.0568
2)0.982.80:0.05155)0.329.12:0.01588,(MaiMet28:0.01692,(Ult28936:0.04059,(
UltCh430:0.05669,UltCh429:0.00946)0.298.15:0.00822)0.906.148:0.03019)0.80
8.80:0.01376)0.411.19:0.01736)0.923.168:0.02214)0.565.9:0.0043)0.781.92:0
.00786,((Ult28935:0.02775,(Ult28934:0.03625,UltCh424:0.00309)0.978.83:0.0
2841)0.770.115:0.01126,(Ult28939:0.0586,(UltCh427:0.02558,(UltCh428:0.048
36,(UltCh425:5.5E-4,UltCh426:5.3E-
4)0.885.122:0.01212)0.667.24:0.00787)0.860.96:0.02478)0.849.112:0.01548)0
.833.98:0.00915)0.994.115:0.04072,(((((UltCh417:0.0,UltCh418:0.0):5.4E-
4,Ult28932:0.01115)0.992.105:0.07757,((Ult28938:0.17709,(Ult28931:8.3E-
4,(MaiMet27:0.00368,Ult28930:5.4E-
4)0.868.109:0.02654)0.994.116:0.11493)0.851.122:0.06335,UltCh416:0.09696)
0.889.131:0.03515)0.674.21:0.00873,((((Ult28928:0.08978,((Ult28927:0.0417
8,UltCh413:0.05057)0.977.91:0.04364,((CytSpe14:0.03573,(CpoSpeci:0.00406,
(CpoSpec2:0.00753,CpoSpec3:0.00367)0.114.3:0.00339)0.808.81:0.01638)0.994
.117:0.12261,(UdnEuba9:0.18651,(Ult32311:0.01264,BactEM19:0.00623)0.992.1
06:0.1138)0.009.6:0.02588)0.926.146:0.06274)0.005.5:0.00606)0.923.169:0.0
2144,((UltCh409:0.1056,(UltCh408:0.05854,(Ult28924:0.01596,(UltCh407:0.00
909,(UltCh405:0.019,((Ult28923:5.5E-
4,UltCh403:0.01117)0.975.95:0.015,(UltCh404:0.01138,UltCh406:5.4E-
4)0.557.14:0.00739)0.535.15:5.5E-
4)0.692.16:0.00211)0.463.15:0.00514)0.933.139:0.0247)0.865.130:0.01265)0.
839.103:0.00802,(UltCh415:0.01933,(UltCh414:0.02598,Ult28929:0.0089)0.488
.19:0.0154)1.000.873:0.07657)0.735.50:0.00445)0.792.105:0.00868,((UltCh41
0:0.00371,(UltCh411:0.00368,UltCh412:0.00372)0.789.89:0.00371)0.996.121:0
.05862,(UltSpon6:5.4E-
4,(Ult28925:0.00747,Ult28926:0.02195)0.386.11:0.01261)0.916.141:0.02413)0
.903.117:0.01773)0.973.93:0.05277,(UltCra10:0.00893,UltCrat9:0.00224)1.00
0.874:0.12541)0.859.116:0.03047)0.987.128:0.051,(((UdnEuba7:0.01496,(((Ul
tCh450:0.12275,(Ult28943:0.01448,(UltCh439:0.00406,(UltCh438:0.00376,(Ult
Ch440:0.0151,UltCh437:0.02697)0.863.139:0.0075)0.814.75:0.00717)0.360.16:
0.0092)0.909.119:0.02247)0.855.118:0.01705,(UdnEuba6:5.4E-
4,UltCh441:0.01493)0.952.110:0.02687)0.947.100:0.02485,(UltCh442:0.03553,
(((UltCh448:0.01057,UltCh449:0.00894)0.978.84:0.02722,((UltrS364:0.01951,
(UltCh443:0.05977,((Ult28948:0.02485,(UltCh445:5.5E-
4,(UltCh447:0.01517,UltCh446:0.00748)0.767.94:0.0037)0.878.118:5.4E-
4)0.795.91:0.00378,UltCh444:0.01527)0.872.129:5.4E-
4)0.939.115:0.01816)0.336.15:0.00375,(Ult28947:0.00368,(Ult28946:0.01155,
(Ult28944:0.00371,Ult28945:0.0037)0.930.118:0.01122)0.931.129:0.01136)0.9

07.134:0.01449)0.970.102:5.4E-4)0.496.10:0.00984,(UltCh451:5.4E-
4,(Ult28949:0.01909,UltCh452:5.5E-
4)0.887.134:0.01128)0.995.123:0.02714)0.763.119:0.00658)0.904.133:0.01675
)0.618.9:0.00852)0.963.96:0.04069,Ult28951:0.03744)0.910.122:0.02583,Ult2
8858:0.06921)0.880.144:0.02039)0.810.77:0.00996)0.720.34:0.00466,((((UltC
h453:0.07094,Ult28952:0.07483)0.907.135:0.04301,(Ult28962:0.06344,UltCh45
6:0.06517)0.793.93:0.01794)0.599.14:0.01599,(Ult28960:0.04712,Ult28961:0.
01113)0.825.83:0.01997)0.691.17:0.0179,((Ult28956:0.06679,Ult28959:0.0609
7)0.936.122:0.0358,(UltEu120:0.03962,(Ult28958:0.02627,Ult28957:0.00853)0
.884.132:0.02519)0.423.18:0.00808)0.750.72:0.00853)0.892.123:0.01955)0.73
3.49:0.0092,(((Ult28847:0.01498,Ult28848:0.01602)0.998.159:0.06183,(((Ult
28852:0.04892,(((Ult28844:0.00516,Ult28845:0.02576)0.999.176:0.09178,((((
(Ult28836:0.02682,(Ult28837:0.03846,UltCh386:0.01088)0.599.15:0.00499)0.8
85.123:0.01236,(UltCh387:0.04085,UltCh388:0.0407)0.424.21:0.01125)0.855.1
19:0.00856,(UltCh389:0.01479,Ult28846:0.0245)0.467.13:0.00719)0.182.8:0.0
037,(Ult28840:0.00826,(Ult28838:0.01508,Ult28839:0.00372)0.981.99:0.02254
)0.434.13:0.00735)0.802.78:0.01006,(Ult28843:0.0612,(Ult28841:5.3E-
4,Ult28842:0.03464)0.951.111:0.03775)0.827.89:0.03278)0.591.18:0.02235)0.
855.120:0.01718,((UltOr383:0.02842,((((Ult28830:0.02315,((Ult28828:0.0156
1,Ult28827:0.01546)0.924.127:0.0192,(Ult28834:0.11386,(Ult28823:0.04018,(
UltCh382:0.06033,((Ult28809:0.01521,(Ult28810:0.00733,(UltCh379:0.00747,(
Ult28807:0.00745,Ult28808:0.01125)0.739.55:0.00373)0.874.149:0.01159)0.65
0.18:0.01171)0.952.111:0.02324,(((((Ult28812:0.00712,Ult28820:0.004)0.765
.89:0.00744,(((Ult28819:0.03611,Ult28818:0.01954)0.771.121:0.00304,(Ult28
816:0.00739,Ult28817:0.0075)0.775.103:0.00376)0.894.130:0.00757,(Ult28813
:0.01916,(Ult28822:0.00371,Ult28821:0.00371)0.785.104:0.00359)0.783.117:0
.00384)0.668.24:5.3E-
4)0.907.136:0.00345,(UltCh383:0.01843,(UltCh380:0.01132,UltCh381:0.01516)
0.941.108:0.01432)0.766.91:0.00529)0.764.111:0.00363,Ult28811:0.01904)0.5
44.13:0.00382,(Ult28814:5.3E-
4,Ult28815:0.00742)0.992.107:0.02714)0.772.107:0.00384)0.772.108:0.00508)
0.828.76:0.03031)0.905.129:0.02107)0.323.12:0.0055)0.765.90:0.00444)0.225
.8:0.01128,Ult28829:0.06024)0.882.143:0.01504,(Ult28825:0.0141,(Ult28824:
0.01119,Ult28826:0.00396)0.462.18:0.00937)0.999.177:0.07359)0.869.100:5.3
E-
4,((Ult28832:0.01242,Ult28833:0.03851)0.974.72:0.03605,(UltCh384:0.04369,
Ult28831:0.01956)0.938.86:0.02898)0.936.123:0.03086)0.944.103:0.0301)0.98
5.103:0.04089,(Ult28835:0.01935,UltCh385:0.02394)0.986.98:0.04969)0.878.1
19:0.02035)0.960.109:0.03879)0.781.93:0.01067,((Ult28854:0.06131,((Ult288
49:0.01509,UltCh390:0.00384)0.979.105:0.02598,(UltCh392:0.05293,(Ult28850
:0.03925,(UltCh391:0.02033,Ult28851:0.05372)0.765.91:0.0136)0.768.88:0.00
889)0.216.9:0.00411)0.670.16:0.00799)0.807.71:0.01056,(Ult28853:0.08055,U
ltCh393:0.12017)0.802.79:8.1E-
4)0.910.123:0.01571)0.919.162:0.02323,(((Ult32114:0.0,Ult32113:0.0):0.007
51,Ult32115:0.02421)0.997.130:0.09619,(((Ult28082:0.0,Ult28080:0.0):0.065
51,(Ult28083:0.02996,Ult28081:0.07344)0.959.115:0.05995)0.989.94:0.09996,
(Ult28233:0.05285,Ult28232:5.4E-
4)0.963.97:0.06753)0.758.93:0.04224)0.841.101:0.02517)0.743.79:0.00939)0.
739.56:0.00559,(((Ult28868:0.01969,(UltCh399:0.04138,(((UltCh397:0.02807,
UltCh398:0.02284)0.877.140:0.01135,(Ult28866:0.05364,((Ult28864:0.06129,U
lt28863:0.01131)0.390.20:5.5E-
4,Ult28865:0.01507)0.828.77:0.00675)0.735.51:0.00399)0.942.119:0.0159,(Ul
tDeha8:0.01541,Ult28867:0.00747)0.973.94:0.02417)0.748.80:0.0037)0.518.15
:0.01568)0.954.98:0.03664,(Ult28659:0.15485,(UltCh396:0.03353,UltOr384:0.

02769)0.988.106:0.08193)0.704.25:0.02613)0.778.102:0.00695,((Ult28860:0.0
4362,((((UltCh394:0.03088,Ult28855:0.01203)0.845.117:0.0118,Ult28856:0.03
974)0.489.15:0.00584,(UltCh395:0.02999,Ult13235:0.02558)0.477.17:0.01439)
0.755.84:0.00584,((Ult28708:0.013,(Ult13166:0.02468,(Ult28707:5.4E-
4,UltCh369:0.01487)0.981.100:0.04316)0.986.99:0.04854)0.885.124:0.01952,(
(((Ult28727:0.01572,Ult28709:0.03141)0.853.96:0.00772,((Ult28792:0.01148,
Ult28793:0.06024)0.844.123:0.00753,(((Ult28794:0.01307,Ult28795:0.01754)0
.377.16:0.00352,UltCh375:0.02133)0.779.102:0.0045,Ult28791:0.01093)0.901.
130:0.01275)0.961.105:0.01593)0.805.77:0.00677,((Ult28737:0.03895,((Ult28
806:0.02909,(Ult28732:0.01748,Ult28734:0.02563)0.423.19:0.01116)0.865.131
:0.00902,(UltCh372:0.01844,(Ult28712:0.04937,((Ult28733:0.0219,Ult28710:0
.085)0.792.106:0.02147,(Ult28731:0.05207,UltCh370:0.02648)0.860.97:0.0126
1)0.791.90:5.5E-4)0.037.4:0.00751)0.718.35:5.4E-
4)0.795.92:0.00442)0.796.71:0.00367,(((((((((((Ult28665:0.00372,(UltCh36
1:0.00748,(UltCh362:5.4E-
4,UltEu117:0.01896)0.886.153:0.00742)0.757.76:0.0037)0.862.121:5.4E-
4,(Ult28671:0.0,Ult28672:0.0):0.03912)0.891.106:0.0141,(((Ult28668:0.0036
9,((Ult28667:0.00719,Ult28670:0.02307)0.782.120:0.00747,Ult28669:0.02652)
0.835.96:5.2E-
4)0.762.107:0.0038,Ult28666:0.01129)0.770.116:0.00364,(Ult28803:0.03089,(
(Ult28802:0.04481,((Ult28799:0.00362,(UltCh376:5.5E-
4,Ult28798:0.0559)0.910.124:0.00757)0.893.138:0.01233,(Ult28800:0.00763,U
lt28801:0.01143)0.735.52:0.00274)0.965.99:0.03839)0.906.149:0.02076,(Ult2
8711:0.03924,UltOr382:0.031)0.277.14:0.00492)0.416.30:0.01379)0.734.67:0.
00371)0.789.90:0.00553)0.880.145:0.01046,(Ult28694:0.02142,(Ult28692:0.00
598,Ult28693:0.00893)0.714.31:0.00754)0.872.130:0.00913)0.765.92:0.0036,(
(Ult28770:0.01855,(Ult28771:0.01903,(Ult28769:0.01133,(Ult28768:5.5E-
4,Ult28767:0.00369)0.976.87:0.01518)0.781.94:0.00378)0.744.73:0.00521)0.9
20.148:0.01427,(Ult28663:0.05452,(Ult28673:0.01025,Ult28751:0.03665)0.500
.18:0.01118)0.833.99:0.0095)0.847.145:5.4E-
4)0.810.78:0.00369,((((((Ult28766:0.03616,Ult28775:0.01123)0.470.9:0.0109
2,((Ult28772:0.0236,(UltCh377:0.09554,UltCh378:0.00471)0.909.120:0.01521)
0.911.156:0.01538,(((Ult28662:0.01265,UltCh360:0.02657)0.984.89:0.03079,U
ltCh374:0.01923)0.749.94:0.0039,Ult28788:0.02301)0.846.127:0.0071)0.891.1
07:0.01111)0.843.103:5.4E-
4,(Ult28748:0.00744,Ult28790:0.0229)0.769.103:0.00374)0.788.98:0.00369,((
(((Ult28759:0.0,Ult28760:0.0,Ult28762:0.0,Ult28764:0.0):5.3E-
4,((Ult28761:5.4E-4,((Ult28756:5.5E-
4,(Ult28754:0.00368,(Ult28755:0.00369,Ult28753:0.01504)0.802.80:5.5E-
4)0.758.94:5.4E-4)0.944.104:5.4E-
4,Ult28763:0.0074)0.920.149:0.00742)0.924.128:0.00209,Ult28758:0.00694)0.
925.139:0.00207)0.959.116:0.01119,Ult28757:5.3E-
4)0.850.128:0.00369,(((((Ult28723:0.02241,((Ult28713:0.0,Ult28714:0.0,Ult
28715:0.0):0.00368,Ult28716:5.5E-
4)0.999.178:0.04462)0.895.136:0.01694,(Ult28717:0.03008,(Ult28719:0.01435
,(Ult28718:0.00755,Ult28797:0.00731)0.774.107:0.00485)0.743.80:0.0042)0.0
52.10:0.01098)0.594.17:5.5E-
4,Ult28724:0.01808)0.912.156:0.01861,Ult28789:0.02301)0.751.69:0.00366,(U
lt28774:0.01897,Ult28787:0.01896)0.864.119:0.00749)0.771.122:0.00371)0.85
1.123:5.5E-
4,Ult28725:0.02288)0.899.126:0.00746)0.775.104:0.00377,(((Ult28804:0.0710
5,((((Ult28782:0.01002,Ult28785:0.00996)0.885.125:0.00994,(Ult28780:0.011
27,(Ult28779:0.00745,((Ult28776:0.01901,Ult28777:0.00366)0.892.124:0.0075
3,Ult28778:0.01503)0.750.73:0.00371)0.773.109:0.00366)0.809.84:0.00381)0.

396.22:0.00371,((Ult28783:0.02292,Ult28784:0.01527)0.877.141:0.01133,Ult2
8781:0.01108)0.937.122:5.4E-
4)0.921.135:0.01107,Ult28786:0.00747)0.735.53:0.00374)0.965.100:0.01917,U
lt28646:0.03511)0.750.74:0.00353,(((Ult28645:0.00745,(Ult28624:0.02691,((
Ult28623:0.00369,((Ult28625:5.4E-4,(Ult28620:0.00741,(Ult28626:5.5E-
4,((Ult28618:0.00714,(Ult28619:0.00345,Ult28621:0.004)0.551.16:0.00752)0.
912.157:5.3E-4,Ult28622:5.5E-4)0.040.4:5.5E-4)0.909.121:5.4E-
4)0.854.112:0.00369)0.915.132:0.00737,((Ult28627:0.01114,((((Ult28644:0.0
0369,Ult28643:0.01498)0.805.78:0.00368,((Ult28641:0.0,Ult28642:0.0):5.5E-
4,(Ult28638:0.00745,Ult28639:5.5E-4)0.532.15:5.5E-4)0.363.16:5.5E-
4)0.414.14:5.5E-
4,Ult28640:0.0037)0.852.120:0.00372,(Ult28637:0.0037,(Ult28632:0.01122,(U
lt28631:0.00371,Ult28796:5.5E-
4)0.804.72:0.0037)0.764.112:0.00372)0.782.121:0.0037)0.882.144:5.4E-
4)0.892.125:0.00748,(Ult28635:0.02745,((Ult28628:0.0,Ult28629:0.0,Ult2863
0:0.0):0.02363,(Ult28634:0.01947,Ult28633:0.02301)0.724.36:0.00383)0.850.
129:0.00763)0.833.100:0.00692)0.898.146:0.00766)0.920.150:5.3E-
4)0.182.9:5.4E-4,Ult28636:0.00369)0.907.137:5.4E-
4)0.983.85:0.02344)0.894.131:0.00781,UltEu116:0.00752)0.877.142:0.00749,(
Ult28664:0.04402,Ult28749:0.04413)0.855.121:0.01271)0.729.33:0.00383)0.86
0.98:0.00766)0.785.105:0.00367,(Ult28739:0.02398,Ult28740:0.02398)0.742.6
2:0.00258)0.963.98:5.4E-
4)0.754.87:0.00375,((Ult28674:0.01482,Ult28726:0.02332)0.417.20:0.00771,U
lt28736:0.03463)0.761.83:0.00431)0.783.118:0.00375,(Ult28765:0.02678,((Ul
t28695:0.02392,Ult28696:0.0296)0.834.90:0.00873,(((UltCh373:0.01814,(Ult2
8746:0.00787,Ult28747:0.00332)0.964.118:0.02709)0.939.116:0.01894,((UltCh
365:0.00373,UltCh366:0.01122)0.879.142:0.01014,(Ult28722:0.01123,Ult28735
:0.03104)0.867.132:0.01013)0.845.118:0.00944)0.421.13:0.00719,(UltEu118:0
.01177,(Ult28691:0.03071,Ult28697:0.00439)0.407.16:0.00375)0.957.115:5.5E
-4)0.264.7:0.0089)0.903.118:0.01265)0.659.18:5.5E-
4)0.823.90:0.00375,(Ult28773:0.01121,(Ult28738:0.01959,UltDeha7:0.02359)0
.833.101:0.00706)0.112.6:5.4E-4)0.897.131:5.5E-
4,((UltCh357:0.08119,(((Ult28652:0.01862,(((Ult28660:0.0152,Ult28661:0.02
307)0.920.151:0.01498,(UltCh358:0.04076,(UltCh359:0.03215,Ult28650:0.0535
6)0.077.8:0.00424)0.683.30:0.01449)0.451.22:5.5E-
4,(((Ult28655:0.00822,(Ult28654:0.02793,(UltDehal:0.02349,Ult28653:0.0562
1)0.961.106:0.0252)0.961.107:0.02621)0.810.79:0.0118,Ult28656:0.01998)0.8
56.108:0.00932,(Ult28657:0.02063,Ult28658:0.03833)0.363.17:0.00687)0.747.
76:0.01611)0.901.131:0.01603)0.890.135:0.01461,(Ult28651:0.01413,(Ult2864
8:0.01867,Ult28649:5.4E-
4)0.991.108:0.03321)0.881.138:0.01367)0.374.20:0.00846,(UltCh356:0.08043,
(Ult13030:0.06075,Ult27832:0.06968)0.840.115:0.02408)0.808.82:0.01217)0.7
89.91:0.00826)0.517.10:0.0139,Ult28706:0.0807)0.083.6:0.00393)0.907.138:0
.00742,(UltDeha6:0.04816,UltCh367:0.03902)0.783.119:5.4E-
4)0.799.94:0.00377,(Ult28744:0.05148,((Ult28743:0.00752,Ult28745:0.01133)
0.933.140:0.01527,((Ult28741:0.00372,Ult28742:0.00931)0.780.122:0.00451,U
lt28690:0.04)0.772.109:0.00385)0.770.117:0.00591)0.757.77:0.0062)0.758.95
:0.00373)0.930.119:0.01191)0.953.95:0.01988,((Ult28728:0.02796,(((((DhbCh
lor:0.03405,(Udntfd17:0.0132,Ult28704:0.00975)0.995.124:0.06188)0.919.163
:0.03562,(Ult28705:0.05533,(((Ult28698:0.00747,((Ult28700:0.01567,Ult2869
9:0.03278)0.992.108:5.4E-4,(UltDeha3:5.5E-4,Udntddd7:5.5E-
4)0.610.13:5.5E-4)0.534.14:5.5E-
4)1.000.875:0.00218,(Ult28701:0.0074,((DhlSpec3:0.0,UltDeha4:0.0):5.5E-
4,UltDeha5:0.00369)0.714.32:5.5E-

4)1.000.876:0.00152)0.999.179:0.06318,(Ult28703:0.01268,(Ult28702:0.0289,
(DhgLykan:0.00368,Udntddd8:5.3E-
4)0.734.68:0.00387)0.948.135:0.02461)0.261.9:0.01576)0.930.120:0.02573)0.
718.36:0.00418)0.939.117:0.02772,(Ult28750:0.05705,Ult28647:0.06675)0.861
.145:0.01912)0.715.25:0.00234,(((((Ult28682:5.5E-
4,Ult28683:0.00375)0.790.94:0.00634,(Ult28680:0.01134,Ult28681:0.00742)0.
777.117:0.0051)0.952.112:0.02873,((Ult28678:0.03948,((UltCh363:0.03191,Ul
t28675:0.01911)0.746.69:0.00346,((Ult28677:0.00377,UltDeha2:0.00364)0.816
.80:0.00683,Ult28676:0.02797)0.217.13:0.00797)0.968.101:0.02857)0.781.95:
0.01593,Ult28679:0.0195)0.894.132:0.01757)0.950.107:0.02645,(Ult28684:0.0
4036,((Ult28689:0.01238,(Ult28687:0.01207,Ult28688:0.0347)0.460.13:0.0056
9)0.873.122:0.0091,(Ult28685:0.02805,Ult28686:0.02196)0.687.24:0.00382)0.
764.113:0.00423)0.876.117:0.01404)0.896.120:0.01419,(Ult28752:0.0378,((Ul
tCh368:0.0391,Ult28805:0.07649)0.786.97:0.0103,(Ult28720:5.4E-
4,Ult28721:0.00334)1.000.877:0.06108)0.383.11:0.00777)0.829.106:0.01066)0
.804.73:0.00815)0.864.120:0.00838,(Ult23537:0.02688,UltCh364:0.03228)0.99
8.160:0.04741)0.735.54:0.0029)0.854.113:0.01141,((UltCh371:0.03991,UltEu1
19:0.06184)0.819.84:0.00127,((Ult28730:0.05952,Ult28729:0.02481)0.966.125
:0.03638,(((Ult29203:0.08397,(UltB9869:0.13217,UltCh489:0.0212)0.582.14:0
.03315)0.914.141:0.03845,(Ult13851:0.01459,UltCh490:0.03798)0.773.110:0.0
0807)0.715.26:0.01231,(UltCh488:0.04289,UltCh487:0.02845)0.985.104:0.0522
9)0.993.102:0.06335)0.826.78:0.0141)0.855.122:0.01016)0.944.105:0.01958)0
.920.152:0.0172)0.948.136:0.0277)0.952.113:5.4E-
4)0.197.11:0.00499,(Ult28859:0.04335,Ult28861:0.04989)0.857.130:0.012)0.8
70.117:0.01292)0.110.10:0.00484)0.947.101:0.03393)0.861.146:0.01314)0.877
.143:0.02296)0.908.148:0.02168)0.658.15:0.0066,((Ult28898:0.01671,Ult2889
9:0.01061)0.961.108:0.0324,(Ult28862:0.05941,UltCh476:0.15336)0.911.157:0
.03613)0.782.122:0.02806)0.948.137:0.03206)0.704.26:0.00222,((Ult28076:0.
02893,(Ult28075:0.09041,(Ult28074:5.5E-4,(Ult28072:5.4E-
4,Ult28073:0.01121)0.998.161:0.03885)0.857.131:0.03795)0.941.109:0.03678)
0.954.99:0.03292,(UltEu114:0.04794,(Ult28229:0.04188,(Ult28228:0.0471,(Ul
t28550:0.02796,Ult28551:0.03654)1.000.878:0.08105)0.831.89:0.01632)0.193.
9:0.02144)0.886.154:0.01839)0.751.70:0.00579)0.861.147:0.01632)0.877.144:
0.02084)0.091.5:5.4E-4,(((((((UltOr329:0.0,UltOr331:0.0):5.5E-
4,(UltOr328:0.01848,UltOr330:0.04422)0.766.92:0.00475)0.081.11:5.4E-
4,UltOr332:0.00369)0.932.130:0.01535,(UltOr334:0.04368,((UltCh215:0.0,Ult
Ch218:0.0,UltOr333:0.0):5.5E-
4,(UltCh216:0.0382,UltCh217:0.01089)0.863.140:5.5E-
4)0.741.64:0.00361)0.967.88:0.02386)0.850.130:0.01243,(((UltOr322:5.3E-
4,(UltOr321:0.01605,((UltCh214:5.5E-4,UltCh213:5.5E-
4)0.972.108:0.02237,(UltOr320:0.01818,(UltOr319:0.00742,(UltOr317:5.5E-
4,UltOr318:0.00369)0.837.84:5.5E-4)0.966.126:0.01398)0.853.97:5.5E-
4)0.885.126:0.01412)0.961.109:0.024)0.756.100:0.00695,UltOr323:0.00798)0.
992.109:0.06002,(UltOr315:0.06216,(UltOr326:0.01125,(UltOr324:5.5E-
4,(UltOr327:0.00746,UltOr325:0.00369)0.556.13:5.4E-4)0.930.121:5.4E-
4)0.836.91:0.0125)0.855.123:0.03219)0.898.147:0.01969)0.890.136:0.01389,(
UltCh234:0.04865,((Ult28174:0.03048,((Ult28170:0.00369,Ult28173:5.5E-
4)0.851.124:0.00719,(TlnMetha:0.01889,((Ult28167:0.0,Ult28168:0.0,Ult2816
9:0.0,Ult28171:0.0):5.5E-4,Ult28172:5.5E-4)0.889.132:5.5E-
4)0.763.120:0.00405)0.972.109:0.04068)0.899.127:0.02327,(((((UltOr345:0.0
5617,((UltCh233:0.0221,(Ult28165:0.05866,((UltCh229:0.0,UltCh230:0.0):0.0
3564,UltCh232:0.01688)0.792.107:0.01549)0.574.11:0.00263)0.590.11:0.00809
,(UltCh231:0.0313,Ult28164:0.04988)0.806.70:0.0127)0.899.128:0.01711)0.60
3.16:0.0163,((((UltOr335:0.0,UltOr336:0.0,UltOr337:0.0,UltOr339:0.0):5.5E

-4,UltOr338:0.03838)0.967.89:0.03338,(UltOr355:5.4E-
4,(UltOr353:0.03177,(UltCh225:5.3E-4,(UltOr352:5.5E-
4,(UltOr348:0.00369,(UltOr349:0.02322,((UltOr351:0.0,UltOr354:0.0):0.0112
1,(UltCh226:5.5E-
4,(((UltCh224:0.00368,((UltOr347:0.01536,UltOr350:0.02328)0.851.125:0.007
37,UltCh227:0.0037)0.680.22:5.5E-4)0.323.13:5.5E-4,UltCh228:5.5E-
4)0.471.17:5.5E-4,UltCh223:5.5E-4)0.425.14:5.4E-4)0.851.126:5.5E-
4)0.768.89:0.00373)0.784.102:0.00375)0.368.9:5.4E-
4)0.921.136:0.00372)0.683.31:5.4E-
4)0.187.11:0.00371)0.996.122:0.06421)0.868.110:0.0127,((UltOr346:0.0141,(
UltCh221:5.5E-
4,UltCh222:0.0113)0.833.102:0.00946)0.970.103:0.03269,((Ult28161:0.03027,
(Ult28159:0.01819,Ult28160:0.00997)0.979.106:0.03324)0.721.22:5.3E-
4,Ult28162:0.01295)0.982.81:0.03433)0.723.43:0.00445)0.866.132:0.01209)0.
959.117:5.4E-
4,(UltOr356:0.07337,(UltOr344:0.03865,(UltCh219:0.03065,(UltCh220:5.4E-
4,((UltOr340:0.0,UltOr341:0.0):0.00964,(UltOr342:5.4E-
4,UltOr343:0.00743)0.944.106:0.01835)0.918.139:0.01414)0.740.69:0.00396)0
.995.125:0.06223)0.982.82:0.05117)0.854.114:0.01902)0.913.144:0.02004,Ult
28163:0.03839)0.967.90:0.03768,Ult28157:0.04714)0.876.118:0.01589)0.581.1
3:0.01008)0.729.34:0.00547)0.998.162:0.07866,((((Ult32079:0.0,Ult32080:0.
0):0.05493,Ult32081:0.01007)1.000.879:0.14332,((Ult32235:0.00713,(UltFi10
8:5.5E-
4,(UltFi107:0.00343,Ult32234:0.01556)0.964.119:0.01974)0.871.133:0.00784)
0.986.100:0.04847,((Ult32231:0.00369,(Ult32232:5.5E-
4,(Ult32243:0.00741,Ult28231:0.0074)0.675.16:5.5E-4)0.740.70:5.0E-
4)0.980.82:0.03673,((Ult32237:0.00384,(Ult32236:0.01813,(Ult32238:0.00767
,Ult32239:0.02777)0.997.131:0.04017)0.859.117:0.00913)0.953.96:0.02647,(U
lt32240:0.02166,(Ult32242:0.04749,Ult32241:0.02544)0.499.13:0.00811)0.964
.120:0.03354)0.959.118:0.03328)0.745.90:0.00847)0.845.119:0.03243)0.796.7
2:0.03563,(((Ult32315:0.00245,UltAc950:0.01667)0.999.180:0.12212,(Ult3231
0:0.00167,(CddDiv13:0.07397,(Ult32309:0.02886,Ult32308:0.04734)0.845.120:
0.01387)0.916.142:0.02127)0.998.163:0.10233)0.711.33:0.02433,(UltChl77:0.
09778,(Ult28230:0.05632,Ult32233:0.03931)0.971.86:0.05539)0.437.18:0.0203
)0.739.57:0.00894)0.855.124:0.02395)0.381.19:0.01867)0.947.102:0.03768)0.
800.78:0.0185)0.834.91:0.02109)0.768.90:0.0117)0.145.6:0.0057)0.943.117:0
.02709,(((((UltB8533:0.04674,Ult27484:0.0575)0.999.181:0.17318,((Ult11184
:0.07274,UltPl518:0.06099)0.345.11:0.01572,(Ult11185:0.16807,((UltAnta9:0
.11245,((UltAnt10:0.0437,Ult11182:0.09739)0.769.104:0.01159,(UltPl517:0.0
391,Ult11183:5.4E-
4)0.968.102:0.03501)0.301.15:0.00458)0.881.139:0.01749,(Ult11181:0.03671,
(Ult11180:0.00342,(Ult11178:0.00492,Ult11179:0.01042)0.999.182:0.05781)0.
722.33:0.00282)0.946.110:0.02568)0.811.72:0.024)0.775.105:0.02367)0.991.1
09:0.08285)0.957.116:0.08087,((UltRum15:0.05453,(((UltRum13:0.00748,((Ult
Al139:0.05335,(UltAl135:0.0538,(UltAl138:0.04809,((((UltAl136:0.01928,(Ot
u00344:0.01507,(UltAl132:0.00743,(UltAl129:0.00164,UltAl130:0.01037)0.822
.75:0.01472)0.291.16:5.5E-
4)0.750.75:0.00386)0.451.23:0.01048,UltAl137:0.01855)0.440.13:0.01,(UltAl
131:0.06608,UltAl134:0.01004)0.907.139:0.01475)0.776.103:0.00789,UltAl133
:0.03603)0.693.25:0.00272)0.901.132:0.01898)0.471.18:0.01428)0.973.95:0.0
3081,(Hmyyy056:0.02344,(UltAl141:0.01521,(UltAl140:0.02347,(S0114132:0.01
133,UltAl142:0.02365)0.849.113:0.0076)0.864.121:5.4E-
4)0.923.170:0.01159)0.708.32:0.00383)0.751.71:0.00391)0.881.140:0.00749,(
UltRum14:5.5E-4,UltB3911:0.00744)0.158.13:5.4E-

4)0.715.27:0.00296,UdnRumen:0.02008)0.924.129:0.03008)1.000.880:0.13514,(
(((UltB9490:0.04794,(UltB9489:0.01577,UltFib20:0.02807)0.932.131:0.04118)
0.885.127:0.03274,((UltCan29:0.00796,((AtPYy119:0.01715,AtPYy120:0.01799)
0.132.16:0.0083,((UltFib18:0.00364,UltFib19:5.5E-
4)0.965.101:0.02228,(Otu00608:0.0439,UltCan28:0.08381)0.135.10:0.00421)0.
853.98:0.01631)0.831.90:0.0341)1.000.881:0.17838,Ult13197:0.10178)0.116.1
0:0.02325)0.931.130:0.04072,(((UltOrg97:0.08045,(UltOrg66:0.01548,UltB948
8:0.0471)0.707.20:0.00769)0.912.158:0.03954,((((UltB9484:0.03763,UltB9485
:0.02732)0.073.7:0.00982,((UltB9481:0.02482,(UltB9479:5.5E-
4,UltB9480:0.02266)0.909.122:0.01798)0.827.90:0.01216,(UltB9486:0.04385,(
(((((((UltFib10:0.01095,(((UltFib15:0.00732,(UltFibro:0.00363,UltFib14:5
.5E-4)0.834.92:0.00363)0.912.159:5.5E-4,UltFib17:5.5E-4)0.979.107:5.4E-
4,UltFibr7:0.01468)0.816.81:0.00358)0.360.17:5.4E-4,(UltFibr6:5.5E-
4,UltFi127:0.00364)0.971.87:5.4E-4)0.987.129:5.4E-
4,(UltFibr5:0.01845,UltFib16:5.5E-
4)0.506.13:0.00722)0.747.77:0.00349,(UltFibr2:5.5E-
4,UltFibr4:0.00731)0.942.120:5.4E-
4)0.439.16:0.00716,(UltFib11:0.00742,UltFib13:0.03057)0.952.114:5.4E-
4)0.883.139:0.00711,(((UltCand8:0.00741,UltCan10:0.00762)0.598.10:0.00299
,((UltCand9:5.5E-
4,UltCa470:0.0037)0.834.93:0.00695,(UltFibr9:0.0262,UltFi153:5.4E-
4)0.967.91:0.01945)0.626.17:0.00793)0.950.108:0.01581,UltFibr8:5.4E-
4)0.816.82:0.00361)0.299.16:5.5E-4,(UltFib12:0.01847,UltFibr3:5.5E-
4)0.965.102:0.01109)0.863.141:0.01076,Tryyy095:0.04165)0.143.13:0.00832,(
(UltCan11:5.5E-
4,(UltCa434:0.00741,(UltCan13:0.0,UltCan12:0.0):0.02064)0.055.4:5.4E-
4)0.977.92:0.02055,(UltCan15:0.02273,((UltCan16:0.0,UltCa474:0.0):0.00438
,(UltCan14:0.01133,UltCan17:0.00749)0.773.111:0.00311)0.680.23:0.01931)0.
725.41:0.00479)0.240.11:0.00377)1.000.882:0.08433)0.721.23:0.00564)0.905.
130:0.01741)0.947.103:0.0317,(UltB9483:0.05697,(((UltCan23:0.01793,(((Ult
Can18:0.04365,UltCan19:0.04258)0.627.9:0.0209,UltB9482:0.07892)0.399.21:5
.5E-4,(UltCan21:0.01986,(UltCan20:5.5E-
4,UltCa475:0.00369)0.813.82:0.00691)0.979.108:0.02418)0.716.31:0.00866)0.
313.9:0.00406,(UltCan22:0.0175,UltCan24:0.14251)0.545.14:0.00479)0.976.88
:0.03135,((UltCan25:0.0238,UltCan26:0.00695)0.946.111:0.03046,(UltCan27:0
.05219,UltOrg96:0.02921)0.947.104:0.0252)0.869.101:0.01661)0.599.16:0.007
8)0.374.21:0.00556)0.000.916:0.01577,UltB9487:0.03472)0.687.25:0.03506)0.
933.141:0.06378,(UltB9504:0.08633,((((((UltFib39:0.0113,UltFib40:0.00366
)0.753.94:0.00406,((UltFib37:5.4E-4,(((UltFib32:0.00378,(UltFib30:5.5E-
4,(UltFib33:0.00367,(UltFib31:0.00745,(UltFib27:5.5E-4,(UltFib28:5.5E-
4,(UltFib36:0.00367,(UltFib34:5.5E-4,UltFib29:5.5E-
4)0.859.118:0.00465)0.448.17:5.5E-4)0.229.11:5.4E-
4)0.791.91:0.00467)0.806.71:0.0047)0.253.14:5.5E-4)0.948.138:5.4E-
4)0.893.139:0.00715,UltFib41:5.4E-
4)0.852.121:0.00367,((UltFib25:0.00741,UltFib26:0.00758)0.758.96:0.00364,
UltFib38:0.01505)0.775.106:0.00373)0.895.137:5.4E-
4)0.836.92:0.00673,((UltFib62:0.01679,((UltFib60:0.00334,((UltFib59:0.003
7,(UltFib63:0.00368,UltFib58:0.01127)0.808.83:0.00373)0.911.158:5.4E-
4,UltFib61:0.00695)0.920.153:0.01516)0.333.9:0.00673,UltFib57:0.00133)0.3
75.14:0.00922)0.993.103:0.04182,UltFib35:0.00368)0.779.103:0.00379)0.841.
102:0.00727)0.743.81:0.00511,(((UltFib53:5.4E-
4,(UltFib52:0.03128,UltFib51:5.4E-
4)0.891.108:0.01081)0.838.111:0.00921,(UltFib54:0.01502,UltFib55:0.02571)
0.774.108:0.0079)0.410.16:0.00791,(((((UltFib49:0.01109,(UltFib50:5.5E-

4,(UltFib48:5.3E-
4,UltFib47:0.00746)0.821.77:0.00367)0.851.127:0.00768)0.980.83:0.02615,((
UltFib43:5.5E-
4,UltFib42:0.00369)0.923.171:0.01461,UltFib45:0.01633)0.328.16:0.00589)0.
780.123:0.00693,UltFib46:0.01544)0.870.118:0.00725,UltFib44:5.5E-
4)0.940.120:0.01883,(UltFi174:0.00366,UltFib56:5.5E-
4)0.970.104:0.02375)0.848.105:0.00978)0.214.13:0.00838)1.000.883:0.16022,
(((((UltB9497:0.0,UltB9495:0.0):0.01468,(UltB9496:0.01121,((UltB9498:0.0,
Ult20597:0.0):5.5E-4,UltB9499:0.00743)0.107.13:5.5E-
4)0.850.131:0.00942)0.882.145:0.01364,(((FibSpeci:5.5E-4,UltB9493:5.5E-
4)0.993.104:5.3E-4,(UltB9494:0.0039,(UltRu266:0.01931,(UltRu634:5.5E-
4,(FibSucc7:0.00371,FibSucc6:5.5E-4)0.968.103:0.01136)0.366.14:5.5E-
4)0.155.13:0.00356)1.000.884:0.04099)0.982.83:0.02306,(FibSucc5:0.00384,(
(UltRu265:0.00734,(FibSucci:0.02009,((FibSucc2:5.5E-
4,FibSucc4:0.00367)0.873.123:5.5E-
4,((FibSucc3:0.0,UltRu263:0.0):0.0037,((UltB9491:0.0,UltRu616:0.0):5.5E-
4,(UltRu264:0.0037,UdnRum22:5.5E-4)0.541.7:5.5E-4)0.937.123:5.5E-
4)0.844.124:0.0037)0.868.111:0.00752)0.900.113:0.01174)0.993.105:0.02772,
UltB9492:5.4E-
4)0.962.119:0.0152)0.741.65:0.0036)0.863.142:0.01016)0.501.13:0.01749,Fib
Spec2:0.04076)0.820.73:0.01622,((UltFib21:5.3E-
4,(Ult45978:0.00369,(UltFib22:5.5E-
4,(Ult37024:0.00369,UltFib23:0.0037)0.767.95:5.5E-4)0.631.15:5.5E-
4)0.781.96:0.00362)0.993.106:0.06197,((EchggY10:0.0,EchggYy0:0.0):0.00722
,(Bfhggg37:0.10005,((BfhYyy22:0.0,Bfhggg35:0.0,Bfhggg36:0.0,BfhYyy23:0.0,
Bfhggg38:0.0,Bfhggg39:0.0):5.5E-4,Bfhggg40:0.00369)0.312.14:5.4E-
4)0.731.40:0.00412)0.947.105:0.03856)0.862.122:0.03697)0.724.37:0.00593)0
.843.104:0.02517,((UltRu631:5.5E-
4,UltB9503:0.00368)0.767.96:0.00911,(FibIntes:5.5E-
4,((UltB9501:0.0,UltB9502:0.0):0.0191,UltB9500:0.01903)0.191.10:0.00748)0
.895.138:0.02217)0.940.121:0.04639)0.931.131:0.04436,((Ult46176:0.0,UltFi
b24:0.0):0.01703,(UltB9505:0.04535,UltFi179:0.02762)0.669.21:0.02284)0.89
2.126:0.03323)0.382.13:0.03687,Ult46175:0.0408)0.923.172:0.05728)0.992.11
0:0.12685)0.838.112:0.0517)0.883.140:0.03876,(((UltB9411:0.17431,(UltB922
8:0.10686,(UltB9229:0.03251,((((PldPhaeo:5.4E-
4,ClrFerro:0.0037)1.000.885:5.4E-
4,(((((((ClrPhae3:0.00372,ClrLimic:0.01154)0.780.124:0.00377,(UltChl11:0.
00368,(UltB9251:5.5E-4,(ClrLimi4:0.0037,UltChl10:0.00369)0.757.78:5.5E-
4)0.816.83:5.5E-
4)0.812.74:0.00373)0.811.73:0.00374,(UltChl13:0.01137,(ClrChlor:0.02351,U
ltB9237:0.00777)0.770.118:0.00347)0.777.118:0.00376)0.855.125:5.5E-
4,(PthVibr3:0.01059,((ClrPhae2:0.0,ClrLuteo:0.0):5.5E-
4,((UltB9212:0.0,UltB9214:0.0,UltB9219:0.0,UltB9245:0.0):0.01128,(((UltB9
246:0.00739,UltB9235:0.02301)0.629.23:5.5E-
4,(UltB9210:0.0,UltB9213:0.0,UltB9218:0.0,UltB9216:0.0,UltB9227:0.0,UltB9
234:0.0,UltB9239:0.0,UltB9241:0.0,UltB9243:0.0,UltB9247:0.0,UltB9252:0.0)
:5.5E-4)0.379.14:5.5E-4,UltB9160:0.00369)0.615.19:5.3E-
4)0.934.113:0.00757)0.781.97:0.00345)0.917.120:0.01133)0.773.112:0.00717,
(((UltB9250:0.01559,(UltB9220:0.01128,((UltB9211:0.0,UltB9208:0.0,ClrPhae
o:0.0,UltB9217:0.0,UltB9226:0.0,UltB9236:0.0,UltB9240:0.0,UltB9242:0.0,Ul
tB9465:0.0):5.5E-4,(UltB9249:0.00369,(UltB9209:5.5E-4,UltB9244:5.5E-
4)0.845.121:0.00369)0.865.132:5.5E-4)0.882.146:5.4E-
4)0.842.113:0.00745)0.949.121:0.01593,((PthAest4:0.0,UltChlo3:0.0):0.0152
6,((UltB9223:0.0035,(UltB9225:0.01115,(PthAest2:5.5E-

4,PthAest3:0.00368)0.778.103:0.00389)0.360.18:0.0075)1.000.886:5.4E-
4,((PthAestu:5.4E-
4,PthIndic:0.0037)0.897.132:0.01041,(UltB9224:0.01998,((PthSpec2:5.5E-
4,(PthVibr2:0.0,UltB9248:0.0):5.5E-4)0.820.74:0.00397,(UltChlo2:5.5E-
4,(PthSpeci:0.0,PthVibri:0.0):5.5E-
4)0.790.95:0.00356)0.771.123:0.00381)0.767.97:0.0057)0.880.146:0.01045)0.
779.104:0.00379)0.766.93:0.00353)0.961.110:0.01582,UltB9233:0.00381)0.905
.131:0.01403)0.675.17:0.01323,((ClcMaces:5.4E-
4,(ClrLimi3:0.00366,((UltChlo4:0.0,ClcParvu:0.0,ClrSpeci:0.0,ClcParv2:0.0
):5.4E-4,UltChlo9:0.00743)0.851.128:0.01139)0.999.183:5.5E-
4)0.831.91:0.00372,(((UltB9238:0.0,UltChlo8:0.0):0.0037,((((((ClrPhae4:5
.5E-4,UltChlo6:5.5E-4)0.000.917:5.5E-4,(UltChlo5:0.0,ClcLimna:0.0):5.5E-
4)0.786.98:5.5E-4,ClcSpeci:0.00369)0.499.14:5.5E-
4,(UltChlo7:0.01093,UltChl12:0.03633)0.981.101:5.4E-
4)0.798.89:0.00367,ClrTepid:0.00378)0.800.79:0.00497,(ClcTepid:0.00885,Cl
rLimi2:0.0141)0.509.12:0.00389)0.786.99:0.00631,ClrLute2:0.00746)0.643.12
:5.4E-4)0.820.75:0.0037,(ClcThio2:5.5E-4,ClcThios:0.00368)0.273.13:5.4E-
4)0.904.134:0.00744)0.895.139:5.5E-
4)0.864.122:0.01119,UltB9232:0.04461)0.767.98:0.0033)0.382.14:0.00795,((U
ltChl14:0.07051,(ClhThal2:5.5E-4,ClhThala:5.5E-
4)0.931.132:0.02136)0.290.13:0.00657,((UltB9258:0.01146,(UltChl15:0.01365
,(UltB9256:0.00369,UltB9257:5.5E-
4)0.779.105:0.00507)0.437.19:0.00603)0.563.13:0.01842,((UltB9254:5.5E-
4,(UltB9253:0.00367,UltB9255:5.5E-
4)0.884.133:0.01893)0.852.122:0.01504,UltChl16:0.07511)0.728.45:0.03277)0
.991.110:0.0744)1.000.887:0.09936)0.950.109:0.02062,(UltB9231:0.05315,Ult
B9230:0.06055)0.755.85:0.00815)0.706.30:0.00727)0.880.147:0.0787)0.949.12
2:0.09898)0.414.15:0.04175,(UltB9187:0.26599,(UltB9431:0.08995,(UdnBac27:
0.05029,(UltB9432:0.02058,(DenBac27:0.05811,(UltB9429:0.01371,UltB9430:0.
0014)0.960.110:0.04915)0.961.111:0.05635)0.881.141:0.03151)0.815.74:0.027
36)0.088.10:0.01849)0.787.96:0.03077)0.283.10:0.02532,((((((UltB9308:0.06
235,(UltOrg94:0.0999,((UltB9316:0.04672,((UltB9304:0.01554,UltChl31:0.034
37)0.584.15:0.00971,(UltChl26:0.04347,UltChl30:0.03034)0.929.123:0.01955)
0.858.124:0.00992)0.840.116:0.0092,(UltB9326:0.00281,((UltB9323:0.0369,Ul
tChl32:0.09627)0.925.140:0.04171,UltB9327:0.03777)0.851.129:0.02156)0.935
.119:0.02581)0.756.101:0.00694)0.763.121:5.6E-
4)0.233.11:0.0083,(UltB9303:0.01128,UltChl25:0.01134)0.759.107:0.0043)0.9
30.122:0.01768,((((UltChl27:0.01151,(((UncUn115:0.01118,UltB9311:5.5E-
4)0.977.93:0.02538,(UncUn116:0.00741,UltChl29:0.01525)0.762.108:0.00521)0
.263.8:0.00367,UltChl28:0.01569)0.874.150:0.01092)0.798.90:0.00317,(Otu00
543:0.03525,UltB9310:0.01569)0.721.24:0.0041)0.996.123:0.04201,(UltB9309:
0.03985,(((UltB9302:0.08184,UltB9307:0.0271)0.424.22:0.02176,((UltB9313:0
.05699,(UltB9315:0.0195,(UltB9312:0.03126,UltB9314:0.01619)0.865.133:0.01
296)0.932.132:0.03066)0.810.80:0.03108,UltB9325:0.02222)0.438.18:0.0245)0
.658.16:0.00632,((UltSAR40:0.03692,UltB9301:0.04468)0.862.123:0.0291,((Ul
tB9297:0.00754,UltB9298:0.00738)0.926.147:0.01485,(UltB9299:5.5E-
4,UltB9300:5.5E-4)0.970.105:5.4E-
4)0.998.164:0.07504)0.654.17:0.02173)0.735.55:0.00964)0.755.86:0.00752)0.
925.141:0.024,UltB9324:0.11098)0.550.10:0.00461)0.942.121:0.01772,(((UltB
9321:0.01018,UltB9322:0.00526)0.994.118:0.04425,(((UltSlu22:0.0423,(((Ult
B9329:0.04107,(UltB9330:0.03162,(UltB9331:5.5E-
4,UltB9332:0.01118)0.953.97:0.03062)0.921.137:0.02606)0.978.85:0.04148,Ud
nCyto3:0.01405)0.861.148:0.01337,UltB9333:0.00923)0.923.173:0.03184)0.925
.142:0.03268,UltB9328:0.0644)0.909.123:0.03206,((UltB9317:0.0108,UltB9318

:0.02425)0.994.119:0.07895,(UdnCyto2:0.00589,(UltB9320:0.011,UltB9319:0.0
2188)0.386.12:0.01611)0.984.90:0.06057)0.472.16:0.0303)0.824.80:0.01685)0
.726.36:0.00699,(UltB9305:0.0696,UltB9306:0.05432)0.995.126:0.05458)0.772
.110:0.00817)0.973.96:0.04242,(((UltCyt13:0.04164,UltB9196:0.06792)0.969.
96:0.04314,(UltOrg90:0.03423,((((UltB9191:0.01585,(SstLonga:0.02516,(UltB
9190:0.01527,(UltB9189:0.07483,(UltB9188:0.00374,(UltHalop:0.01121,SnbRub
er:5.4E-4)0.974.73:0.01511)0.952.115:5.4E-
4)0.861.149:0.01101)0.999.184:0.06066)0.732.39:0.00604)0.976.89:0.03103,U
ltB9192:0.07772)0.686.19:0.00505,(UltB9194:0.06113,UltB9197:0.01881)0.950
.110:0.03816)0.404.16:0.02287,((UltCyt12:0.02804,((UltrSo81:0.06517,(RdtM
ari2:0.01119,RdtMarin:5.5E-
4)0.994.120:0.04824)0.817.65:0.01401,UltB9193:0.04138)0.772.111:0.00719)0
.896.121:0.01243,(UltMar47:5.4E-4,(UltB1960:5.5E-
4,UltB9195:0.00369)0.891.109:0.00726)0.827.91:0.01558)0.871.134:0.00993)0
.039.5:5.4E-
4)0.743.82:0.00593)0.888.139:0.03636,(((((BacEnr31:0.00603,(UltSph32:0.08
843,(GrcTropi:0.00846,(UltB9199:0.03048,(UltB9198:5.4E-
4,(BnlVulga:0.01344,UltChit2:0.00789)0.827.92:0.01299)0.982.84:0.04104)0.
932.133:0.02168)0.485.20:0.00781)0.495.16:5.4E-
4)0.909.124:0.02237,UltOrg91:0.03045)0.901.133:0.02066,(UltOrg92:0.12543,
((UltB9205:0.01669,(UltB9204:0.0299,UltB9206:0.01912)0.924.130:0.03156)0.
948.139:0.03326,(UltB9202:0.04605,(UltB9207:0.07038,UltB9203:0.0503)0.487
.21:0.00407)0.893.140:0.02127)0.920.154:0.03167)0.862.124:0.02161)0.850.1
32:0.01356,UltB9201:0.04354)0.291.17:0.01171,UltB9200:0.04148)0.995.127:0
.10066)0.966.127:0.06707)0.248.9:5.5E-4,((((UltB9419:5.5E-
4,(UltB9418:0.03073,UltChl42:0.00779)0.249.20:0.00792)0.979.109:0.0494,((
UltB9381:0.03762,((((UltB9397:0.0237,UltB9413:0.06326)0.942.122:0.02,UltB
9410:0.01084)0.750.76:0.00435,((((((UltB9399:0.00369,UltChl37:5.5E-
4)0.934.114:0.01126,((UltB9402:0.02655,(UltB9401:0.00322,(UltB9400:0.0036
9,UltChl39:5.4E-4)0.982.85:0.01967)0.422.16:0.00376)0.719.32:5.3E-
4,UltChl38:0.01907)0.818.58:0.00373)0.768.91:0.00373,((((UltB9395:0.01134
,UltB9406:0.01142)0.950.111:0.0155,(UltB9377:0.02312,((UltB9392:0.00742,U
ltB9393:0.00372)0.955.103:0.01508,(UltB9394:0.01502,(UltB9390:5.4E-
4,UltB9391:0.00737)0.670.17:0.00743)0.762.109:0.00384)0.743.83:0.00347)0.
932.134:0.01146)0.824.81:0.00357,((UltB9415:0.01128,UltB9416:0.00751)0.91
7.121:0.01627,UltB9417:0.04118)0.372.16:0.0149)0.996.124:5.5E-
4,(UltOrg95:0.06824,(UltB9384:0.02049,UltB9385:0.01942)0.909.125:0.02228)
0.774.109:0.01014)0.945.102:0.01123)0.794.87:0.00374,(UltB9409:0.00761,(U
ltB9407:0.01595,UltB9408:0.04315)0.755.87:0.0085)0.862.125:0.00749)0.743.
84:0.00275,(UltB9405:0.03328,(UltB9404:0.00897,UltB9403:0.02588)0.895.140
:0.01645)0.953.98:0.02096)1.000.888:5.4E-
4,((((UltChl41:0.0559,(UltB9396:0.03308,UdnEuba2:0.02274)0.860.99:0.01227
)0.745.91:0.00492,(UltrdLow:0.03117,UltB9412:0.06526)0.880.148:5.4E-
4)0.926.148:0.01169,(((UltrdSB1:0.07478,(UltChl36:0.05204,((UltB9378:0.05
715,UltB9398:0.05974)0.305.12:0.01187,((DenBac17:0.0,DenBac21:0.0):5.4E-
4,((((DenBact9:5.5E-4,((DenBac23:0.00743,DenBac10:0.00743)0.950.112:5.5E-
4,((DenBac12:0.0,DenBac14:0.0,DenBac15:0.0,DenBac16:0.0,DenBac18:0.0,DenB
ac22:0.0):5.5E-4,DenBac20:0.00369)0.000.918:5.5E-4)0.000.919:5.5E-
4)0.716.32:5.5E-4,(DenBact8:0.0,DenBac13:0.0):0.00368)0.440.14:5.4E-
4,DenBac19:5.5E-4)0.305.13:5.5E-4,DenBac11:5.5E-
4)0.993.107:0.00319)0.999.185:0.07263)0.288.11:0.00541)0.895.141:0.01611)
0.806.72:0.00904,(((((UltB4792:0.00709,UltB9383:0.02069)0.925.143:0.01872
,((UltB9362:0.00356,((UltB9359:0.0,UltB9360:0.0):5.4E-
4,(((UltB9356:0.0,UltB9357:0.0,UltB9366:0.0,UltB9365:0.0,UltB9363:0.0,Ult

B9364:0.0):0.01151,(UltB9367:0.01114,UltB9368:5.5E-
4)0.768.92:0.00363)0.888.140:0.01145,UltB9358:0.00736)0.744.74:5.5E-
4)0.794.88:0.0038)0.927.120:0.01189,(UltB9369:0.01931,UltB9380:0.02332)0.
729.35:0.00355)0.613.18:0.00717)0.781.98:0.00786,(UltB9379:0.00809,(((Ult
B9371:0.0,UltB9373:0.0):5.5E-
4,UltB9372:0.00369)0.946.112:0.0156,(UltB9370:0.01128,UltChl35:5.5E-
4)0.833.103:0.00735)0.947.106:0.016)0.750.77:0.00328)0.944.107:0.01879,(U
ltB9382:0.00443,UltB9389:0.0107)0.745.92:0.01082)0.748.81:0.00501,UltB937
6:0.02016)0.935.120:0.01646)0.784.103:5.5E-4,(UltB9387:5.4E-
4,(UltB9388:0.00369,UltB9386:0.01498)0.791.92:0.00369)1.000.889:0.04651)0
.759.108:0.00736)0.896.122:0.01081,(UltChl40:0.04531,UltB9414:0.00853)0.7
15.28:0.00264)0.808.84:0.00426)0.971.88:0.02342,UltB9375:0.03556)0.799.95
:0.007)0.916.143:0.02253,UltB9374:0.03522)0.069.5:0.0043)0.956.111:0.0300
8,(UltB9420:0.031,Ult28249:0.06696)0.944.108:0.03924)0.878.120:0.02095)0.
696.21:0.01101,(((UltB9342:0.04501,(UltB9340:0.00917,UltB9341:0.03532)0.9
23.174:0.02801)0.720.35:0.02118,(UltB9343:0.00527,(UltB9344:0.02943,UltB9
345:0.01202)0.952.116:0.02652)0.705.24:0.00376)0.851.130:0.01267,(UltChl3
3:0.02324,(UltB9346:0.01502,UltrSo82:0.02702)0.527.10:5.4E-
4)0.846.128:0.0102)0.954.100:0.03046)0.928.109:0.02098,((((UltB9338:0.028
95,(((UltB9335:0.0141,UltB9334:0.00944)0.946.113:0.0187,UltB9337:0.0075)0
.142.11:0.00746,UltB9336:5.4E-
4)0.961.112:0.04714)0.997.132:0.0968,(((UltChl22:0.05587,(UltB9286:0.0533
,(UltChl21:0.02937,(UltB9288:0.05892,UltB9287:0.02621)0.000.920:5.4E-
4)0.546.12:0.00963)0.916.144:0.02176)0.802.81:0.00947,((UltB9283:0.0911,U
ltChl20:0.02732)0.920.155:0.02479,(((UltB9284:0.01533,(UltB9279:0.00741,(
UltB9280:5.5E-4,UltB9285:5.5E-4)0.843.105:5.5E-
4)0.779.106:0.00365)0.772.112:0.00379,UltB9282:5.5E-
4)0.761.84:0.00331,UltB9281:0.00781)0.987.130:0.03849)0.855.126:0.01421)0
.929.124:0.02908,((((((UltB9276:0.04538,UltB9274:0.06361)0.758.97:0.00866
,UdnBac26:0.02879)0.313.10:0.00985,(((UltB9269:0.00368,(UltChl17:0.00372,
((UltB9264:0.0,UltB9265:0.0,UltB9266:0.0,UltB9268:0.0):5.5E-
4,UltB9267:5.5E-4)0.782.123:5.5E-
4)0.785.106:0.00376)0.874.151:0.00996,UltB9273:0.0391)0.746.70:0.00571,((
UltB9270:0.0,UltB9271:0.0):5.3E-
4,UltB9272:0.0113)0.926.149:0.01127)0.880.149:0.01355)0.958.132:0.0265,(U
ltB9275:0.06354,UltOrg93:0.01311)0.960.111:0.03533)0.917.122:0.01718,((Ul
tB9289:0.0,UltB9290:0.0,UltB9291:0.0,UltB9292:0.0):0.06045,(UltB9296:0.02
5,(UltChl19:0.03092,(UltAnae8:0.0098,UltChl18:0.04719)0.211.13:0.00813)0.
885.128:0.01858)0.911.159:0.01822)0.727.42:0.00524)0.874.152:0.01779,(Ult
B9294:0.02453,(UltB9295:0.05422,(UltB9277:0.00374,UltB9278:0.00743)0.902.
131:0.01149)0.837.85:0.01051)0.765.93:0.0105)0.802.82:0.02735)0.898.148:0
.02904)0.566.14:0.01208,(UltB9339:0.05833,(UltB9350:0.02173,((UltB9354:5.
5E-
4,UltB9355:0.00369)0.724.38:0.00821,((UltB9349:0.00655,(UltB9347:0.02755,
UltB9348:5.5E-
4)0.949.123:0.02902)0.995.128:0.06579,((UltB9351:0.00715,UltB9352:0.00429
)0.865.134:0.00955,(UltChl34:0.01887,UltB9353:0.01019)0.480.15:0.00372)0.
926.150:0.02908)0.462.19:0.02144)0.958.133:0.06561)1.000.890:0.11636)0.86
6.133:0.02)0.928.110:5.3E-4,(((((DenBac26:5.4E-
4,UltB9425:0.01528)0.950.113:0.02626,((UltB9423:5.5E-
4,UltB9424:0.01519)1.000.891:0.07243,(((DenBac24:0.0,UltChl43:0.0):5.5E-
4,DenBac25:0.02694)0.981.102:0.02405,(UltB9421:0.00736,UltB9422:0.01204)0
.851.131:0.00795)0.653.14:0.00413)0.588.19:0.01362)0.994.121:0.06071,(Ult
B9428:0.05522,((UltB9426:0.01132,UltB9427:0.00381)0.863.143:0.01804,UdnEu

ba3:0.08548)0.434.14:0.00985)0.415.13:0.01418)0.987.131:0.06821,(UltB9439
:0.43685,(UltB9441:0.04992,(UltB9440:0.03872,((UltChl45:0.01162,UltChl44:
0.02771)0.784.104:0.00584,((UltB9433:0.00727,UltB9434:5.4E-
4)0.779.107:0.0043,((UltB9436:0.07585,(UltB9437:0.04791,(UltB9438:0.02781
,UltGree3:0.08971)0.949.124:0.0318)0.496.11:0.01234)0.608.22:0.01284,UltB
9435:0.01747)0.929.125:0.0232)0.869.102:0.00843)0.962.120:0.03882)0.946.1
14:0.0412)0.134.14:5.1E-
4)0.846.129:0.03565)0.849.114:0.02234,((UltCh277:0.0226,(UltB9461:0.02888
,UltChl54:0.06068)0.910.125:0.02893)0.986.101:0.07944,(UltB9293:0.00547,(
UltChl23:0.01131,UltChl24:0.01952)0.904.135:0.01816)0.986.102:0.08394)0.6
16.13:0.03503)0.842.114:0.02206)0.505.6:0.00805)0.963.99:0.03115)0.704.27
:0.0244)0.873.124:0.01315)0.323.14:5.1E-
4)0.690.28:0.03316)0.774.110:0.01097,(((((((((((((Ult31103:0.02258,(Ult
31100:0.0113,(((((Ult31075:0.03544,(Ult31080:0.01142,(Otu00576:5.4E-
4,((Ult31083:0.01112,Ult31085:0.01598)0.699.31:0.01134,(((UltCy239:0.0075
8,Ult31082:0.01496)0.901.134:5.5E-
4,(Ult31081:0.02693,((UltCy241:0.0355,Otu00530:0.01201)0.740.71:0.01127,U
ltCy240:0.01091)0.762.110:0.00421)0.774.111:0.00374)0.831.92:0.00374,Ult3
1084:0.01523)0.702.23:5.5E-
4)0.818.59:0.00707)0.878.121:0.00747)0.742.63:0.00361)0.862.126:0.00771,(
(Ult31077:0.00754,Ult31099:0.02769)0.474.17:0.00778,((UltRu556:0.03144,(
Ult31073:5.5E-4,(Ult31071:5.5E-
4,Ult31072:0.00374)0.839.104:0.00371)0.850.133:0.00884)0.663.23:0.01834,(
(Ult31090:0.0,Ult31091:0.0):0.01151,(((Ult31095:0.0119,(Ult31093:0.00751,
(Ult31092:0.01918,Ult31094:0.01519)0.761.85:0.00381)0.916.145:0.0117)0.59
4.18:0.00359,(Ult31097:0.02845,Ult31096:0.02194)0.798.91:0.00578)0.660.19
:0.00401,((Ult31086:0.0,Ult31088:0.0,Ult31089:0.0):5.5E-
4,Ult31087:0.02277)0.939.118:0.01132)0.729.36:0.00383)0.956.112:0.02384)0
.904.136:5.5E-
4)0.868.112:0.00818,(Ult31079:0.0212,Ult31098:0.04543)0.985.105:0.03734)0
.735.56:0.00296)0.901.135:0.01155,(Ult31074:0.01105,(Ult31076:0.00825,Ult
31102:0.01501)0.043.14:0.0075)0.740.72:0.00425)0.882.147:0.00767,(Ult3110
1:0.00808,Ult31105:0.03195)0.821.78:0.00714)0.563.14:5.4E-
4,Ult31104:0.00716)0.988.107:0.02722,Ult31106:0.01154)0.766.94:0.00349)0.
753.95:0.00844)0.771.124:0.00941,Ult31078:0.12299)0.982.86:0.05131,(Ult31
108:0.00808,((Ult31109:0.02246,Ult31107:0.01365)0.966.128:0.02735,(Otu040
68:0.08428,((UltRu557:0.03059,Ult31111:0.00379)0.936.124:0.02579,(RmnBact
6:0.0168,Ult23528:0.05261)0.601.7:0.01564)0.860.100:0.01063)0.741.66:0.00
547)0.496.12:0.00742)0.812.75:0.01336)0.910.126:0.02653,Ult31110:0.07431)
0.983.86:0.04002,((Ult31114:0.04703,((Ult31116:0.04265,(UltCyan2:0.02839,
Ult31117:0.01877)0.603.17:0.01025)0.916.146:0.0286,(Ult31115:0.03504,(Ult
31112:5.5E-
4,Ult31113:0.00368)0.999.186:0.0718)0.712.25:0.00447)0.586.18:0.01774)0.9
74.74:0.05931,((Ult31144:0.00748,Ult31145:0.00749)0.974.75:0.03485,(((Ult
31160:0.02661,((((Ult31159:0.02757,Ult31165:0.07753)0.881.142:0.01595,((U
lt31156:0.01908,Ult31157:0.01934)0.980.84:0.02345,Ult31158:5.4E-
4)0.787.97:0.0043)0.868.113:0.01075,Ult31150:0.0231)0.935.121:0.01588,(Ul
t31151:0.02466,((Ult31142:0.01601,Ult31143:0.01852)0.476.18:0.00361,((Ult
31141:0.00739,(Ult31139:5.5E-
4,Ult31140:0.0037)0.951.112:0.01543)0.919.164:0.0114,(UltAc943:0.00757,(U
lt31138:5.4E-
4,Ult31137:0.01885)0.652.14:0.0075)0.861.150:0.00766)0.852.123:0.00824)0.
981.103:0.02716)0.741.67:0.00313)0.720.36:0.00432)0.867.133:0.00813,(Ult3
1161:0.0308,((Ult31164:0.02162,(Ult31166:0.01854,(Ult31162:5.5E-

4,Ult31163:0.00744)0.951.113:0.03074)0.903.119:0.02887)0.860.101:0.01683,
((Ult31152:0.0037,Ult31153:5.4E-4)0.830.93:0.00369,(Ult31154:5.5E-
4,Ult31155:5.5E-4)0.923.175:5.5E-
4)0.961.113:0.02039)0.054.3:0.0062)0.856.109:0.00957)0.854.115:0.01049,((
UltCy245:0.00777,(UltCy246:0.00831,(Ult31146:0.0,Ult31147:0.0):0.03525)0.
984.91:0.03078)0.094.6:5.4E-
4,(Ult31148:0.02047,Ult31149:0.06473)0.535.16:0.02316)0.939.119:0.01584)0
.901.136:0.02333)0.911.160:0.0435)0.743.85:0.00637)0.884.134:0.02175,(Ult
31125:0.07097,(Ult31119:0.03582,((((Ult31118:0.01776,(Ult31121:0.00362,Ul
t31122:0.01147)0.971.89:0.02373)0.829.107:0.01005,Ult31124:0.03797)0.740.
73:0.00569,(Ult31120:5.4E-
4,(UltrS441:0.01521,UltCy243:0.00747)0.867.134:0.00747)0.921.138:0.01588)
0.828.78:0.01005,(Ult31123:0.08217,UltCy242:0.00742)0.858.125:0.01482)0.1
91.11:0.01282)0.873.125:0.01966)0.931.133:0.03509)0.798.92:5.4E-
4,Ult31130:0.04054)0.746.71:0.00348,(Ult31129:0.02652,(Ult31127:0.04086,U
lt31128:0.0202)0.986.103:0.04139)0.736.58:0.0053)0.832.91:0.01307,((UltB1
788:0.12325,Ult31131:0.09164)0.967.92:0.07882,(((OlrAcumi:5.5E-
4,(UltCya61:0.01931,(Ult30927:0.0,UltCy165:0.0):5.5E-
4)0.690.29:0.00371)0.994.122:0.04574,(UltOr479:0.0522,((((((((UltCya97:0.
04173,(CilMagnu:0.01404,SriZimba:0.02922)0.988.108:0.05031)0.928.111:0.03
619,(((Ult30986:0.00717,Ult30987:5.4E-
4)0.889.133:0.0214,(CcdSpec6:0.01727,(CcdSpec5:0.02365,((UltChr10:0.0,Ult
Chr11:0.0):5.5E-4,CcdSpec4:5.5E-
4)0.855.127:0.0122)0.504.12:0.01616)0.994.123:0.06495)0.912.160:0.02472,(
LynWolle:0.04938,(UltCy210:0.04948,UltCy226:0.05033)0.754.88:0.02489)0.89
3.141:0.02845)0.656.18:0.00819)0.872.131:0.01442,((((Ult30869:0.0,Ult3087
0:0.0):0.0229,((Ult30868:0.01165,UdnCyano:0.01517)0.956.113:0.0286,(UltCy
118:0.0118,(((UltCy115:5.5E-
4,(UltCy116:0.00369,UltCy137:0.00369)0.666.21:5.5E-4)0.910.127:5.3E-
4,(UltCy117:5.5E-4,UltCy119:0.00369)0.858.126:0.0037)0.969.97:6.0E-
4,(UltCy112:0.03491,(UltCy114:5.5E-
4,(UltCy136:0.02661,UltCy113:0.00369)0.687.26:5.5E-4)0.994.124:5.5E-
4)0.957.117:0.02295)0.888.141:0.01931)0.907.140:0.01972)0.767.99:0.00628)
0.969.98:0.02089,((UltCy183:0.09229,((PlkSpeci:5.4E-
4,(PlkMouge:0.01135,(PlkPseu2:0.01158,(Ult30908:0.00369,(Ult30909:0.00369
,PlkPseud:5.5E-4)0.204.7:5.5E-
4)0.862.127:0.00752)0.920.156:0.01157)0.773.113:0.00369)0.804.74:5.4E-
4,(PlkSpec2:0.01092,(OlrSpec2:0.00295,(PlkPseu3:0.01127,((PlkAgar2:0.0,Pl
kAgard:0.0):5.5E-4,Ult30910:0.00369)0.979.110:5.4E-
4)0.418.15:0.00836)0.990.110:0.02891)0.857.132:0.00821)0.875.141:0.02609)
0.940.122:0.02959,(((Ult30921:0.05717,(((((UltCy159:5.5E-
4,(UltCy160:0.00369,(MisAntar:0.00369,(UltAnt24:0.0037,(UltAnt23:5.5E-
4,((Ult30920:0.0,UltCy161:0.0):5.5E-
4,(PmiAutum:0.00749,Ult30919:0.00369)0.523.10:5.5E-4)0.000.921:5.5E-
4)0.511.12:5.5E-4)0.000.922:5.4E-4)0.853.99:0.00372)0.444.23:5.5E-
4)0.598.11:5.5E-4,Ult30918:5.5E-
4)0.963.100:0.01132,UltrS439:0.00369)0.548.12:5.5E-
4,(UltCya96:0.0,MisVagin:0.0,Ult30917:0.0):5.5E-4)0.873.126:5.5E-
4,Ult30915:0.00741)0.988.109:5.4E-
4)0.970.106:0.03692,(Ult30923:0.01505,OlrLutea:5.5E-
4)0.922.174:0.02592)0.963.101:0.03232,Ult30916:0.03853)0.740.74:0.01171)0
.893.142:5.4E-
4)0.847.146:0.01051,((UltCy202:0.03445,(((ClxSpec7:0.0,ClxSpec8:0.0):0.00
798,Ult30977:0.01501)0.977.94:0.03213,(((ClxSpec6:0.00743,(Ult30973:0.003

69,(Ult30974:0.0,Ult30975:0.0):5.5E-4)0.999.187:5.5E-
4)0.976.90:0.02678,((UltCy195:0.0,UltCy201:0.0):0.031,Ult30976:0.00747)0.
956.114:5.5E-
4)0.965.103:0.02329,(RivSpec3:0.00258,((Ult30971:0.01918,(Ult30969:5.4E-
4,(ClxSpec5:0.01902,Ult30972:5.5E-
4)0.853.100:0.00369)0.774.112:0.00369)0.837.86:0.00364,(ClxDeser:5.4E-
4,(Ult30970:0.00387,(ClxSpec4:5.5E-4,(ClxSpec3:0.00743,RivSpec2:5.5E-
4)0.964.121:0.01127)0.933.142:0.01127)0.982.87:0.02325)0.971.90:5.5E-
4)0.964.122:0.02442)0.931.134:0.01717)0.000.923:5.4E-
4)0.919.165:0.02661)0.960.112:0.03371,(((((UltCy205:0.0541,ScyHofma:0.024
08)0.786.100:0.00858,BslRober:0.00689)0.975.96:0.0202,(BslBrome:0.00373,(
BslTerre:0.00595,(UltCy206:0.0,UltCy207:0.0):0.02702)0.783.120:0.00568)0.
691.18:5.3E-4)0.809.85:0.00749,((SgmCyano:5.3E-
4,Ult30960:0.00751)0.929.126:0.01539,(UltCy203:5.5E-
4,UltCy204:0.01115)0.987.132:0.0278)0.938.87:0.0196)0.980.85:0.02127,((Ul
tCy200:0.0037,(UltCy199:0.00375,UltCy198:0.00742)0.286.14:5.5E-
4)0.902.132:0.01549,((ScySpec3:0.01107,(ScySpec2:5.5E-
4,ScySpeci:0.02687)0.966.129:0.02066)0.996.125:0.05515,(Ult31035:0.0318,(
UltBrasi:0.00719,UltrS440:0.06238)0.863.144:0.01421)0.875.142:0.01912)0.5
00.19:0.01728)0.790.96:0.00792)0.914.142:0.01874)0.869.103:0.01661)0.421.
14:0.01062)0.784.105:0.01692,((((((AhiMaxim:0.0,AhiPlat2:0.0):5.4E-
4,AhiPlate:0.01092)0.855.128:0.01143,((Ult30912:0.0,Ult30913:0.0,LynAestu
:0.0,Ult30911:0.0):0.0038,(UltCy156:0.0484,LynHiero:5.5E-
4)0.952.117:0.0191)0.923.176:0.01174)0.839.105:0.00743,((UltCy167:0.0159,
UltCy168:0.02708)0.990.111:0.03232,(UltCy155:0.01667,((UltCy152:0.0,UltCy
153:0.0):0.00914,UltCy154:0.0185)0.992.111:0.03987)0.841.103:0.01159)0.74
7.78:0.00385)0.764.114:0.00396,(((UltCy162:0.01132,(Ult30924:0.02729,PmiS
pe10:0.00371)0.761.86:0.00381)0.894.133:0.02214,Ult31034:0.06587)0.915.13
3:0.02191,((Ult31033:0.02041,(Ult30926:0.0167,(((UltAnt19:0.01101,UltAnt2
6:5.4E-
4)0.950.114:0.02445,(Ult30925:0.01313,TicSocia:0.02734)0.944.109:0.02409)
0.818.60:0.01549,(CyumPL00:0.01949,((OssCyan4:0.01086,(OlrSpec3:5.3E-
4,PmiAmbig:0.01124)0.617.14:0.00423)0.806.73:0.00719,UltCy163:0.01525)0.8
28.79:5.4E-
4)0.985.106:0.04403)0.859.119:0.01508)0.882.148:0.01571)0.873.127:0.01634
,PmiUncin:0.02327)0.502.13:0.0101)0.805.79:0.00875)0.940.123:0.01791,((((
((((Ult31057:0.021,(UltCy231:0.01205,UltCy232:0.02239)0.901.137:0.01521)0
.922.175:0.01337,(Ult30968:0.03545,Ult31023:5.4E-
4)0.440.15:0.0064)0.812.76:5.4E-4,((((HdlLyngb:5.5E-4,(TcdThieb:5.4E-
4,(TcdEryth:0.01897,((OlrSanc2:5.5E-
4,OlrSanct:0.0037)0.951.114:0.01526,(BlhSpeci:0.00368,OlrNigro:5.4E-
4)0.872.132:0.0076)0.854.116:0.00739)0.368.10:0.00368)0.984.92:0.01892)0.
999.188:0.06893,((BacEnr54:0.0,Ult30894:0.0):0.10688,((((((ApzGraci:0.00
384,(((AabSpeci:0.00371,((AabFlosa:0.01119,((AabCrass:5.4E-
4,(AabCirc2:0.0037,(AabAffin:0.00369,(AabEllip:0.0,UltCy180:0.0):5.5E-
4)0.865.135:5.5E-
4)0.970.107:0.01121)0.903.120:0.00742,AabCirc3:0.00369)0.931.135:5.4E-
4)0.488.20:0.00367,UltAnaba:5.3E-
4)0.967.93:0.01519)0.905.132:0.01089,(ApzFlos5:0.00792,(UltCy181:0.00369,
((ApzGrac2:0.00757,(((ApzFlos3:5.5E-
4,ApzFlos4:0.01494)0.821.79:0.00369,(ApzFlosa:5.5E-4,ApzFlos2:5.5E-
4)0.770.119:5.5E-4)0.790.97:0.00371,(AabPlanc:5.4E-
4,(AabCirci:0.00369,AabPlank:0.00372)0.809.86:0.0037)0.783.121:0.00371)0.
842.115:0.00725)0.923.177:5.5E-4,ApzSpeci:5.5E-4)0.725.42:5.3E-

4)0.751.72:0.00325)0.747.79:0.00751)0.853.101:5.4E-4,(AabSpec2:5.5E-
4,(AabSpec3:0.00369,AabSpec4:5.5E-
4)0.940.124:0.00742)0.844.125:0.00371)0.795.93:0.00368)0.893.143:0.01281,
((AabFlos2:0.00365,AabSolit:5.5E-4)0.833.104:0.01023,((AabSpec9:5.4E-
4,((ApzIssa2:0.0,ApzIssat:0.0,AabSpe10:0.0):0.00184,AabaeCf0:0.01489)0.69
0.30:0.00165)0.899.129:0.01389,(Ult30950:0.01568,(NooSpec7:5.4E-
4,(AabAzoti:0.00772,(NooMusco:0.0,NooSpec8:0.0):0.0115)0.871.135:0.00736)
0.983.87:0.02694)0.912.161:0.01335)0.964.123:0.03077)0.122.4:0.01427)0.92
5.144:0.02153,((((CppRaci2:0.0,CppRacib:0.0,RphBrook:0.0):0.01333,(AabSpe
11:0.06642,AabSpe12:0.02616)0.801.87:0.013)0.958.134:0.02439,AabSpe13:0.0
2165)0.912.162:0.0192,((AabOscil:5.3E-
4,AabaeCf2:0.02707)0.763.122:0.00368,(XxxNosto:5.4E-
4,(AabFlos3:0.02728,(AabAphan:0.00372,AabKisse:0.0037)0.827.93:0.00732)0.
983.88:0.02326)0.781.99:0.00373)0.901.138:0.01715)0.876.119:0.01579)0.838
.113:0.00898,((NooCalci:5.4E-
4,(NooSpec3:0.00339,NooElgon:0.03655)0.897.133:0.01176)0.892.127:0.0075,N
ooSpec4:0.00371)0.970.108:0.01926)0.728.46:0.00425,NooSpe16:0.00792)0.888
.142:0.01469,(((NooSpe10:0.0291,NooSpec9:5.4E-
4)0.579.17:0.01711,((ClxBrevi:5.3E-4,(FisSpec2:0.01057,((NshSpeci:5.4E-
4,((WesSpeci:0.0,WesProli:0.0):0.00369,HpsHiber:0.00371)0.823.91:0.00371)
0.855.129:0.00746,FisSpec3:5.3E-
4)0.785.107:0.00485)0.989.95:0.0314)0.754.89:0.00468,ClxSpeci:0.01075)0.9
46.115:0.01955)0.795.94:0.00905,(((Ult30953:5.4E-4,((NooSpe36:5.5E-
4,(NooComm5:5.5E-4,NooPunct:0.00317)0.350.12:5.1E-
4)0.802.83:0.00373,(NooSpe32:5.5E-
4,(UdnCyan4:0.02296,((NooSpe31:0.0,NooSpe33:0.0,NooComm6:0.0,UltNosto:0.0
,UltNost5:0.0):5.5E-4,(((((Ult30954:5.4E-
4,(NooSpe37:0.0,NooSpe40:0.0):0.00369)1.000.892:5.5E-
4,((NooSpe28:0.00378,(NooSpe29:0.00365,Ult30952:0.00381)0.761.87:0.00373)
0.972.110:0.0153,((((NooSpe41:0.00731,((NooSpe42:0.0,NooSpe43:0.0):0.0040
7,(((NooSpe24:0.0,NooSpe25:0.0):5.5E-
4,(NooComm2:0.0037,Ult30957:0.0037)0.421.15:5.4E-4)0.962.121:5.3E-
4,(Ult30956:0.0037,(Ult30951:0.00369,NooSpe22:5.5E-4)0.540.15:5.4E-
4)0.898.149:0.00738)0.345.12:0.00741)0.334.12:0.00738)0.922.176:5.5E-
4,(GtcEchin:0.0,Ult30958:0.0):0.00369)1.000.893:5.5E-
4,((NooSpe20:0.0,NooSpe38:0.0,NooSpe39:0.0):0.00372,(NooSpe26:0.0,NooSpe2
7:0.0):5.4E-
4)0.863.145:0.00364)0.445.19:0.00707,(NooPisci:0.00808,NooSpe14:0.00694)0
.889.134:0.01153)0.908.149:5.4E-
4)0.523.11:0.00754)0.832.92:0.00358,NooSpe34:5.4E-
4)0.886.155:0.00359,((NooSpe30:0.0,NooSpe35:0.0):5.5E-
4,UltNost4:0.00369)1.000.894:5.4E-
4)0.296.11:0.00366,(UltAnt25:0.00741,(NooSpe21:0.0,NooSphae:0.0,NooCommu:
0.0,NooComm3:0.0,NooComm4:0.0):5.5E-4)0.865.136:0.00362)1.000.895:5.4E-
4)0.766.95:5.4E-4)1.000.896:5.1E-
4)0.970.109:0.00314)0.891.110:0.00728)0.349.11:0.0081,((AabSpec5:0.0,AabS
pec6:0.0,AabSpec8:0.0):5.4E-
4,AabSpec7:0.0037)0.824.82:0.00705)0.910.128:0.0119,(StoOcell:0.00493,(Pn
eSpeci:0.0145,(UltCy208:0.02212,(MgdRepen:0.0114,SypSpeci:5.5E-
4)0.940.125:0.01722)0.847.147:0.01394)0.942.123:0.01815)0.848.106:0.00996
)0.781.100:0.00783)0.926.151:0.01771)0.683.32:0.00404,(((CyuBECID:0.0151,
(AabSpe16:0.0,AabFlos4:0.0):0.00771)0.871.136:0.00781,(((((NooSpe11:0.015
09,(((CydSpeci:5.4E-4,(CluSpeci:5.5E-4,(UltCy196:0.00369,UltCy197:5.5E-
4)0.849.115:0.0037)0.919.166:0.00746)0.826.79:0.00369,NooSpec2:0.00742)0.

678.22:5.3E-
4,(Ult30943:0.0,NooSpe19:0.0):0.01515)0.908.150:0.00723)0.888.143:5.4E-
4,(NooSpeci:5.5E-4,PudSpeci:0.0075)0.930.123:0.01124)0.855.130:5.5E-
4,TlxSpec2:5.5E-4)0.934.115:5.5E-
4,NooSpe18:0.01124)0.828.80:0.00372,(((((UltNost3:0.00369,UltNost2:5.5E-
4)0.904.137:5.5E-4,(NooSpe23:5.5E-
4,Ult30955:0.00369)0.943.118:0.00745)0.795.95:0.00352,(AabSpe14:0.00749,A
abSpe15:5.5E-
4)0.956.115:0.01514)0.794.89:0.00366,(TlxSpec3:0.00105,Ult30967:0.01922)0
.937.124:0.00307)0.733.50:0.00758,((NooMusc2:0.00751,NoocCf00:0.00373)0.9
33.143:0.0027,(NooCarne:0.0,TlxSpeci:0.0):0.00222)0.749.95:0.0022)1.000.8
97:5.3E-
4)0.745.93:0.00339)0.784.106:0.00389,((((CydStagn:0.00369,((NoaCyano:0.00
37,((((NooSpe12:0.0037,NooSpe13:5.5E-
4)0.914.143:0.00746,((AabFlos6:0.00369,Ult30949:0.01118)0.902.133:5.5E-
4,Ult30948:5.5E-4)0.858.127:5.5E-4)0.966.130:0.01123,NooSpe44:5.4E-
4)0.797.84:0.00372,Ult30947:0.00376)0.906.150:0.00757)0.083.7:5.5E-
4,Ult30959:5.5E-4)0.848.107:0.00373)0.763.123:5.4E-
4,(TihDolio:0.01902,(NooSpec5:0.0151,(AabaLaxa:0.0112,(NooSpec6:0.0,AabVa
ria:0.0,AabFlos5:0.0):5.5E-4)0.769.105:5.5E-4)0.674.22:5.5E-
4)0.983.89:0.01912)0.761.88:0.00367,(NooSpe15:0.01856,(AbpCircu:0.0,NooSp
e17:0.0):0.00938)0.951.115:0.01885)0.905.133:0.01163,(((((NooEllip:0.0109
,(((FisSpec4:0.01446,Ult31036:0.08885)0.996.126:0.07437,((CglSpeci:0.0599
6,(CglFrits:0.02627,Ult30964:0.00937)0.541.8:0.00928)0.958.135:0.03036,(C
lxSpe10:5.4E-
4,(UltCy193:0.01962,(UltCy192:0.0039,(UltCy191:0.00742,(UltCy194:5.5E-
4,UltCy190:0.00367)0.384.16:5.5E-
4)0.868.114:0.00736)0.925.145:0.01549)0.984.93:0.02753)0.927.121:0.02897)
0.833.105:0.01263)0.859.120:0.01318,((Ult30965:0.01112,RivSpeci:5.4E-
4)0.992.112:0.02726,ClxSpec2:5.4E-
4)0.968.104:0.02331)0.744.75:0.00398)0.808.85:0.00726,Ult30966:0.06677)0.
735.57:0.00842,Ult14251:0.10929)0.339.15:5.4E-
4,((Ult30946:0.00952,((NodHarve:5.5E-
4,Ult30944:0.00369)0.932.135:0.00685,(NodSpumi:5.4E-4,(NodSpum2:5.4E-
4,(((NodSpha4:0.0,Ult30945:0.0,ApzSpec2:0.0):5.5E-
4,(NodSpha3:0.0037,UltCy182:0.0037)0.770.120:5.5E-4)0.913.145:5.5E-
4,((NodSpha2:0.0,NodSphae:0.0):0.00753,(NodSpum3:0.00376,NodSpum4:0.0037)
0.795.96:0.00372)0.885.129:5.5E-
4)0.865.137:0.0037)0.916.147:0.00745)0.887.135:0.00229)0.938.88:5.4E-
4)0.802.84:0.00488,((AabBergi:0.00384,(UmzNatan:0.01882,(ApzOval2:0.0,Apz
Ovali:0.0):5.5E-
4)0.957.118:0.01518)0.094.7:0.00874,(Udntddd9:0.00405,(NodHarv2:0.01111,(
(AbpElenk:0.0,AbpSpeci:0.0,AbpElen2:0.0):5.4E-
4,AbpElen3:0.00332)0.357.12:0.00761)0.811.74:0.00772)0.993.108:0.02887)0.
745.94:0.00117)0.980.86:0.02143)0.970.110:0.01882,((AabSpe17:0.00842,((Hp
sSpeci:0.02238,(((FisMusci:0.0,MglLamin:0.0):0.00721,(Ult30962:0.05443,Ul
t30963:0.02738)0.978.86:0.03579)0.817.66:0.00824,(((UltCy188:0.00243,UltC
y189:0.00246)0.914.144:0.00244,(UltCy187:5.5E-4,(UltCy186:5.5E-
4,UltCy185:0.00741)0.748.82:5.5E-4)0.829.108:5.5E-
4)0.864.123:0.00709,((FlnTher5:0.0,FisSpeci:0.0):5.5E-
4,((CcdSpec3:0.0,UltCy184:0.0):0.00371,Ult30961:0.0037)0.179.8:5.5E-
4)0.772.113:0.00407)0.456.16:0.00375)0.864.124:0.00932)0.979.111:0.02685,
CydSpec2:0.0132)0.885.130:0.01435)0.829.109:0.00691,(AabCylin:5.4E-
4,AabCyli2:0.00369)0.973.97:0.01546)0.938.89:5.5E-

4)0.949.125:0.01835)0.797.85:5.4E-
4)0.785.108:0.00439)0.915.134:0.01983)0.835.97:0.02639)0.781.101:0.0102,(
(Ult30980:0.02403,(UltCy164:0.03444,Ult30997:0.0442)0.704.28:0.01665)0.86
7.135:0.01464,(UltCy209:5.5E-
4,UltCy211:0.00369)0.932.136:0.01701)0.886.156:0.01536)0.864.125:0.01391,
(Ult30978:0.01808,Ult30979:0.04859)0.863.146:0.00923)0.725.43:5.5E-
4)0.951.116:0.01474,(Ult31009:0.03534,(Ult31055:0.01489,(Ult31049:0.08354
,((Ult31050:0.0,Ult31053:0.0):5.4E-
4,(Ult31048:0.01523,(Ult31051:0.00369,Ult31052:5.5E-4)0.983.90:5.4E-
4)0.905.134:0.01121)0.761.89:0.0044)0.896.123:0.01515)0.755.88:0.01154)0.
755.89:0.00341)0.809.87:0.00418,Ult31008:0.00749)0.000.924:0.00152,(UltCy
230:0.01261,(UltCy229:0.00227,Ult31056:0.02493)0.824.83:0.00818)0.993.109
:0.04304)0.661.18:0.00351,(((((Ult31010:5.5E-4,Ult31011:5.5E-
4)0.916.148:0.01103,(CcdSpec9:0.01141,(Ult31007:0.00876,((Ult31005:0.0036
9,Ult31006:5.5E-
4)0.872.133:0.01036,(UltCy221:0.00369,(UltCy220:0.01224,UltCy222:0.01115)
0.068.8:5.5E-
4)0.774.113:0.0051)0.931.136:0.01945)0.854.117:0.01315)0.856.110:5.4E-
4)0.819.85:0.00615,(Ult31030:0.00755,(((Ult31018:0.00737,(((Ult31015:0.00
586,Ult31016:0.0185)0.795.97:0.0058,Ult31020:0.01908)0.973.98:5.5E-
4,((Ult31019:0.0,Ult31022:0.0,Ult31013:0.0,Ult31014:0.0,Ult31012:0.0,Ult3
1017:0.0):5.5E-4,(Ult31021:0.0074,Ult31024:5.5E-4)0.939.120:5.5E-
4)0.913.146:5.5E-4)0.959.119:5.4E-
4)0.976.91:0.01865,Ult31028:0.00376)0.436.14:0.00729,(((Ult31025:0.0,Ult3
1026:0.0):5.5E-4,Ult31027:5.5E-4)0.909.126:5.5E-
4,((Ult31032:0.00753,UltCy225:0.01137)0.789.92:0.00366,(Ult31031:5.5E-
4,(Ult31029:0.00369,UltCy224:0.00369)0.861.151:5.5E-4)0.789.93:5.5E-
4)0.844.126:0.0037)0.951.117:5.4E-
4)0.976.92:0.01892)0.927.122:0.01267)0.763.124:0.00398,((Ult31002:0.00645
,(CyumOU20:5.5E-4,(Ult31000:5.3E-
4,Ult31001:0.01535)0.910.129:0.00742)0.988.110:0.04361)0.971.91:0.0392,(U
ltCy213:0.00995,Ult31004:0.02993)0.607.12:0.01173)0.657.20:0.00136)0.950.
115:5.4E-
4,(UltCy223:0.05187,((Ult30988:0.01707,((((((Ult30989:0.01286,Ult31054:0.
02253)0.985.107:0.03341,((Ult30996:5.3E-
4,(Ult30995:0.0189,Ult30994:0.03172)0.836.93:0.00857)0.904.138:0.0131,Ult
30993:0.0136)0.482.18:0.00393)0.887.136:0.00911,(Ult30991:5.3E-
4,(Ult30992:5.5E-
4,Ult30990:0.00372)0.866.134:0.0037)0.795.98:0.00403)0.795.99:0.00775,Ccd
Spec7:0.02829)0.745.95:0.00841,((Ult30981:0.0,Ult30982:0.0,Ult30983:0.0):
0.01408,(Ult30984:0.0,Ult30985:0.0):0.02626)0.928.112:0.01982)0.961.114:0
.02221,((CcdSpec8:0.02349,UltCy212:0.00701)0.998.165:0.04047,Ult30998:5.4
E-
4)0.847.148:0.00946)0.886.157:0.01328)0.850.134:0.00868,(CceCyano:0.01119
,(Ult30999:0.03553,(UllTherm:0.0074,CgcSider:5.5E-
4)0.630.14:0.00759)0.968.105:5.5E-
4)0.937.125:0.01627)0.869.104:0.01435)0.765.94:0.00463)0.943.119:0.01635)
0.907.141:0.01403,((((GtlSpeci:0.01333,((PmiSpec8:5.3E-
4,PmiSpec9:0.00369)0.956.116:0.01902,GtlSpec2:0.01337)0.653.15:0.00441)0.
998.166:0.05203,((((Ult30808:5.5E-
4,Ult30809:0.00369)0.936.125:0.02777,((Ult30930:0.0,Ult30931:0.0):0.02204
,(((EuhNatro:5.5E-
4,(UltOr480:0.00749,((UltCy170:0.00369,(UltCy171:0.0019,UltCy169:0.02655)
0.988.111:0.00192)0.377.17:5.5E-4,UltCy172:5.5E-4)0.899.130:5.4E-

4)0.919.167:0.00747)0.592.14:5.4E-4,(((Ult30929:5.5E-
4,UltCy173:0.01519)0.922.177:5.5E-
4,Ult30928:0.01109)0.869.105:0.00393,(DcsSalin:0.00369,CyhSpec8:5.5E-
4)0.915.135:0.00418)0.939.121:0.01408)0.522.7:0.00368,HthSpeci:5.5E-
4)0.910.130:0.02159)0.996.127:0.06005)0.950.116:0.03625,((Ult30811:0.0493
8,(Ult30810:0.03089,LgrMBIC2:0.02294)0.792.108:0.01056)0.769.106:0.00711,
(((Cyum6220:0.02236,(((LpgSpec7:0.00345,LpgSpec6:0.02388)0.659.19:0.00789
,FlnCyano:5.3E-4)0.848.108:0.00898,(LgrMBIC1:0.00369,OssCyano:5.5E-
4)0.875.143:0.00994)0.907.142:0.01528)0.912.163:0.01636,((Ult30807:0.0201
,CyumOSC0:0.01507)0.619.10:0.00842,((FlnCyan4:0.01734,(FlnCyan3:0.00761,(
Ult30799:0.05251,((((UltOr477:0.0,UltOr478:0.0):0.02717,Ult30804:0.01507)
0.641.16:0.00312,(UdnBac85:0.01185,((((((Ult30798:0.01153,((Ult30796:0.00
741,LpgSpec2:0.00369)0.866.135:5.5E-
4,Ult30797:0.01889)0.761.90:0.00361)0.852.124:0.00733,(Ult30802:0.00377,U
lt30803:0.00749)0.923.178:5.4E-
4)0.858.128:0.0073,(Ult30800:0.00769,(LpgSpeci:0.00762,(SiuLaxis:0.02749,
PmiSpec2:0.00608)0.770.121:0.00561)0.874.153:0.00789)0.772.114:0.00368)0.
842.116:0.00745,(Ult30801:0.01958,LpgSpec3:5.4E-4)0.665.20:5.4E-
4)0.869.106:0.00905,(OlrSpeci:0.02547,UltCya60:0.02087)0.441.20:0.00378)0
.887.137:0.00986,PmiSpeci:5.4E-
4)0.935.122:0.02013)0.914.145:0.01797)0.887.138:0.01403,((UltCya63:5.3E-
4,(UltCya62:0.03993,UltCya64:0.01621)0.868.115:0.01594)0.923.179:0.01755,
UltCya65:0.03941)0.803.76:0.00722)0.518.16:5.5E-
4)0.366.15:0.01666)0.970.111:0.03355)0.837.87:0.00932,(PmiSpec3:0.02012,P
miSpec4:0.01151)0.827.94:0.00745)0.749.96:0.00454)0.629.24:0.00481)0.900.
114:0.01881,(PmiSpec6:0.04193,(CyumSC10:0.02329,FlnCyan2:0.00778)0.855.13
1:0.01254)0.639.21:0.01627)0.923.180:0.02298)0.554.15:0.00576)0.796.73:0.
00871,HmrSpeci:0.02312)0.897.134:0.01337)0.399.22:0.0153,(((((PurSpec6:0.
06085,UltCy174:0.02655)0.181.7:0.0062,(UltCy175:0.02327,(SiuSubs3:0.02829
,SiuSubs2:0.04532)0.461.11:0.0143)0.882.149:0.01848)0.937.126:0.01954,(Cc
occCf0:0.11094,Ult30933:0.01053)0.407.17:0.00694)0.819.86:0.00982,(((((((
((PeeCyano:0.06544,SycSpe29:0.06816)0.693.26:0.01977,(((Ult30849:0.0209,(
UltAnt15:0.02756,(UltCya99:0.00398,(UltCya98:0.0037,(Ult30848:0.01133,Cpn
Subgl:0.0037)0.372.17:5.4E-
4)0.785.109:0.00346)0.833.106:0.0122)0.990.112:0.05665)0.981.104:0.04523,
(UltCya56:0.05187,UltCya59:0.03219)0.914.146:0.02936)0.756.102:0.00554,Ul
tCy138:0.03276)0.982.88:5.4E-
4)0.334.13:0.017,((UltCy142:0.01939,UltCy141:0.01719)0.817.67:0.01917,(Ul
trS438:0.03768,(UltCy139:0.04078,PeeCyan2:0.05428)0.761.91:0.0168)0.854.1
18:0.01587)0.766.96:0.01046)0.952.118:0.02115,((((Ult30814:0.02835,((LpgS
pe15:0.00356,(LpgSpe14:0.00752,(UltCy120:0.0074,(UltCy121:5.5E-
4,UltCy122:0.00369)0.740.75:5.5E-4)0.961.115:0.01126)1.000.898:5.5E-
4)0.858.129:0.02175,UltCy217:0.0942)0.869.107:0.01998)0.247.11:0.0145,(((
(UltCy140:0.0388,(CcoSpec2:0.00726,CcoSpec3:0.02878)0.997.133:0.06312)0.8
63.147:0.02304,(UltCy133:5.4E-
4,UltCy134:0.0151)0.970.112:0.03048)0.581.14:0.01368,(UltCy135:0.00368,(L
pgAntar:5.4E-4,(UltCy132:0.00369,(UltAnt20:5.5E-
4,UltAnt21:0.00369)0.225.9:5.5E-
4)0.866.136:0.00369)0.793.94:0.00372)0.980.87:0.03457)0.878.122:0.02765,(
ClxSpec9:0.0215,(Ult30873:0.01098,UltCy123:0.02468)0.962.122:0.02792)0.73
5.58:0.01245)0.709.30:0.00652)0.279.7:0.00765,Ult30834:0.04399)0.879.143:
0.01982,((UltMa132:0.05341,(CyumWH7B:0.0264,(UltCy125:0.02004,(Ult30874:0
.01053,UltCy124:0.0044)0.219.8:0.00366)0.988.112:0.0361)0.735.59:0.00442)
0.700.29:0.00451,(UltCy127:0.04143,(Ult30875:0.03537,(UltAnt18:5.4E-

4,(UltCy126:0.0037,UltAnt17:0.00371)0.808.86:0.00367)0.804.75:0.00794)0.7
22.34:0.00762)0.999.189:0.06026)0.773.114:0.00826)0.762.111:0.00489)0.761
.92:0.00535,(((SiuSpeci:0.02511,SiuSubsa:0.02343)0.987.133:0.04299,(UltCy
128:0.07581,UltCy129:0.01029)0.855.132:0.01664)0.715.29:0.01479,(Ult30837
:0.03203,(Ult30881:0.00741,Ult30880:5.5E-
4)0.821.80:0.01082)0.871.137:0.02102)0.972.111:0.03528)0.780.125:0.00981,
((Ult30871:0.0,Ult30872:0.0):0.04292,UltCya76:0.05403)0.764.115:0.01201)0
.906.151:0.02155,(SycSpe28:0.01513,((SycSpe26:0.00364,((Ult30906:0.00999,
SycSpe25:0.01312)0.308.11:0.02314,(PmiSpec7:0.00893,Ult30905:0.02217)0.91
4.147:0.02128)0.908.151:0.02448)0.172.10:0.00398,SycSpe27:0.00365)0.984.9
4:0.04098)0.907.143:0.02359)0.221.11:0.01055,((UltPro96:0.01591,(UltPro95
:0.02286,UltSyn40:0.00757)0.727.43:0.00296)0.992.113:0.04822,((UltPro94:5
.5E-4,(UltPro93:0.0037,UltPro92:0.01498)0.817.68:5.5E-4)0.302.11:5.4E-
4,(UltProc9:5.4E-
4,UltMyc26:0.01887)0.852.125:0.00365)0.905.135:0.01596)0.927.123:0.02455)
0.837.88:0.0122,((((SiuSpec2:0.01305,Ult30934:0.01059)0.992.114:0.04823,(
(((((UltCy150:0.00366,Ult30900:0.02322)0.831.93:0.00698,(Ult30899:0.01458
,UltCy148:0.00422)0.792.109:0.00751)0.751.73:0.00414,UdnCyan3:5.4E-
4)0.820.76:0.02874,Ult30914:0.11318)0.790.98:0.02598,UltPro99:0.02283)0.8
43.106:0.00885,CcdSpec2:0.03238)0.841.104:0.00836)0.926.152:0.0124,(((((U
dnCyan2:0.00986,(((MiyWesen:5.5E-
4,(MiyAerug:0.00369,MiyVirid:0.01499)0.707.21:5.4E-4)0.000.925:5.5E-
4,Ult30896:5.5E-4)0.852.126:0.00366,(MiyAeru4:5.5E-4,(MiyAeru7:5.5E-
4,((MiyAeru5:0.0562,MiyAeru2:0.015)0.903.121:5.5E-
4,((MiySpeci:0.0189,MiyAeru3:0.00716)0.907.144:5.3E-
4,(MiyAeru6:0.0077,MiyWese2:0.00714)0.929.127:5.5E-4)0.000.926:5.5E-
4)0.826.80:5.5E-4)0.000.927:5.5E-4)1.000.899:5.4E-
4)0.756.103:0.01766)0.998.167:0.07518,(RcsSpeci:0.09851,CcoSpeci:0.01819)
0.742.64:0.02135)0.855.133:0.02818,(CyhSpec3:0.01834,Ult30897:0.005)0.913
.147:0.02668)0.728.47:0.01635,CyhSpec2:0.04334)0.881.143:0.02019,((RldGib
ba:0.02791,(((UltMa133:0.0401,(CyhSpec5:0.01124,GlhSpeci:5.3E-
4)0.937.127:0.01645)0.490.7:0.00716,((SymbiOf2:0.00358,(CyhSpec6:0.01511,
CyhSpec7:5.3E-4)0.940.126:0.01946)0.859.121:0.01939,AhpSpeci:5.4E-
4)0.908.152:0.01042)0.850.135:0.00832,(AhhSacru:0.02471,(CyhSpec4:0.00554
,UltCy151:0.00948)0.594.19:0.01223)0.906.152:0.01357)0.926.153:0.01545)0.
729.37:0.00406,((GlhMembr:0.04251,(UltCy145:0.0427,UltCy146:0.00917)0.959
.120:0.02967)0.849.116:0.01193,((MerGlauc:0.05159,(((SssSpec2:0.0,SssSpec
3:0.0):0.03923,SnoRosea:0.0495)0.812.77:0.0083,(WorNaege:0.01594,SssSpec4
:0.02807)0.706.31:7.9E-
4)0.897.135:0.01339)0.835.98:0.0102,(((UltCy108:0.04271,UltCy107:0.01721)
0.875.144:0.01696,(((((LpgSpe12:0.02681,(Ult30836:0.05222,(LpgSpe10:0.015
37,Ult30835:0.02038)0.478.15:0.00204)0.905.136:0.0237)0.972.112:0.03767,(
(CfLepto2:0.00699,((UltCy106:0.03559,((UltCya89:5.5E-4,(UltCya87:5.5E-
4,((UltCya88:0.00369,UltCya85:0.01873)0.685.19:5.5E-
4,UltCya86:0.0074)0.710.34:5.5E-4)0.735.60:5.5E-4)0.879.144:5.5E-
4,UltCya84:0.01491)0.989.96:5.4E-4)0.899.131:0.02701,UltCya90:5.4E-
4)0.769.107:0.00423)0.849.117:0.01285,((PmiPris2:0.01493,Ult30853:5.4E-
4)0.859.122:0.01245,((UltAnt16:0.01125,PmiPris4:5.4E-
4)0.949.126:0.02197,(UltCy101:0.01515,PmiPris3:0.00749)0.765.95:0.00543)0
.181.8:0.01145)0.988.113:0.04185)0.316.8:0.00931)0.706.32:0.0048,(UltCya8
3:0.0038,(Ult30844:0.0095,(CfLeptol:0.02508,UltCya82:0.00722)0.881.144:0.
0147)0.967.94:0.02607)0.807.72:0.00646)0.975.97:0.02391,FlnTherm:0.07174)
0.890.137:5.3E-
4,((UltAnt14:0.02445,(Ult30840:0.055,(Ult30842:0.02255,(Ult30841:0.00954,

((Ult30838:0.0,Ult30839:0.0):0.00498,UltCya77:0.02733)0.766.97:0.0049)0.7
76.104:5.4E-
4)0.779.108:0.01934)0.834.94:0.01604)0.850.136:0.01899,((Ult30847:5.4E-
4,(UltAnt12:0.0075,UltAnt13:0.00371)0.928.113:0.01129)0.816.84:0.00369,(U
ltCya92:0.0,UltCya93:0.0):5.4E-
4)0.875.145:0.01009)0.843.107:0.01085)0.903.122:0.01244)0.737.76:0.00329,
((UltCya91:0.0345,(UltCy109:0.00369,UltCy110:5.5E-
4)0.854.119:0.01199)0.882.150:0.01728,((Ult30862:0.00365,Ult30861:5.4E-
4)0.995.129:0.03356,(Ult30845:0.05407,AhaAfric:0.02618)0.778.104:0.00676)
0.860.102:0.01388)0.838.114:0.0112)0.965.104:0.02652)0.641.17:0.00146)0.7
91.93:0.01187)0.864.126:0.01327)0.859.123:0.0257)0.297.6:5.4E-
4,(((Ult30902:0.0,Ult30903:0.0):0.02901,Ult30904:0.02134)0.853.102:0.0145
,(CcoTurgi:0.0459,((((XnsSpec2:0.00761,(Ult30891:5.5E-
4,(Ult30889:0.0075,Ult30890:0.00745)0.980.88:0.01914)0.965.105:0.01543)0.
794.90:0.00609,Ult30892:0.00939)0.862.128:0.00887,(Ult30895:0.00746,Ult30
893:0.00374)0.743.86:0.00217)0.995.130:0.03726,((LpgSpe16:0.0439,(UltCy14
3:0.01924,((Ult30887:0.01137,Ult30888:0.00381)0.880.150:0.01216,((CcdSpec
i:0.02069,(XnsSpeci:0.00424,((SaeSpeci:0.02247,PurSpec4:0.00964)0.856.111
:0.01032,((DeoSpec2:0.0,DeoSpeci:0.0):0.01502,(DmrSpeci:0.02058,(Ult30886
:0.01154,(MxsSpeci:5.3E-
4,PurSpec5:0.0072)0.855.134:0.00737)0.809.88:0.00659)0.640.17:0.0041)0.91
5.136:0.01642)0.850.137:0.0081)0.853.103:0.01037)0.935.123:5.4E-
4,((PurSpeci:0.01176,(PurSpec3:5.5E-
4,PurSpec2:0.00372)0.987.134:0.01966)0.764.116:0.00341,Ult30885:0.00374)0
.966.131:0.01814)0.755.90:0.00465)0.930.124:0.01461)0.889.135:0.01608)0.8
46.130:0.02012,(((CoySpeci:0.04509,(GcpSpeci:0.01069,Ult30901:0.01321)0.8
86.158:0.01911)0.887.139:0.01644,(SaeCyano:5.5E-
4,UllCyano:0.0037)0.951.118:0.02233)0.856.112:0.01322,(UltCy144:0.00385,(
XnsSpec3:0.00735,XnsSpec4:5.5E-
4)0.896.124:0.01109)0.934.116:0.01401)0.791.94:0.00786)0.728.48:0.00609)0
.733.51:0.00671)0.928.114:0.01676)0.780.126:0.00719)0.865.138:0.00895)0.8
67.136:0.01302)0.841.105:0.01016,((UltCy176:0.01263,SiuSpec3:0.01132)0.98
8.114:0.03503,((((OlrKawam:0.00397,OlrPrinc:0.01498)0.543.15:0.00878,(Ult
Cy227:5.3E-
4,UltCy228:0.03973)0.801.88:0.00663)0.942.124:0.01931,Ult30907:0.08448)0.
164.12:5.3E-
4,((UltAnt22:0.06361,((((MisSpec2:0.0,MisSpeci:0.0):0.02268,(UltCya94:0.0
1468,(PmiMurr2:0.00371,PmiMurra:5.5E-
4)0.891.111:0.01028)0.760.105:0.00251)0.805.80:0.01783,((((PmaCyano:5.4E-
4,((Ult31037:0.0,Ult31038:0.0):0.05272,Ult30942:0.0154)0.389.13:0.01607)0
.400.16:0.02596,(((Ult30935:0.0,Ult30938:0.0):5.5E-
4,(Ult30936:0.02577,Ult30937:0.05461)0.744.76:0.00508)0.975.98:0.01113,(M
isChth3:5.5E-4,(MisChth2:5.5E-4,UltCy179:5.5E-
4)0.913.148:0.00367)0.554.16:5.4E-
4)0.879.145:0.01755)0.916.149:0.02674,(Ult30939:0.00369,MisPalud:5.5E-
4)0.877.145:0.01139)0.760.106:0.00463,(Ult30941:0.00968,(Ult30940:0.00995
,(MisSpec3:0.02808,UltCy166:0.05267)0.758.98:0.01635)0.444.24:0.01887)0.8
90.138:0.0103)0.823.92:5.4E-
4)0.802.85:0.00759,(UltCy233:0.01322,((Ult31059:0.00368,Ult31060:5.4E-
4)0.989.97:0.03139,((SylSpec4:0.0,SylSpec5:0.0):0.02623,(SylSpec3:0.00679
,(SylAtlan:0.00251,(SylSpeci:0.0,SylSpec2:0.0):6.1E-
4)0.382.15:0.01777)0.862.129:0.01538)0.784.107:0.00979)0.890.139:0.01392)
0.853.104:0.00802)0.842.117:0.01109)0.562.17:0.0082,(SczSpeci:0.05067,(Ul
tLyngb:0.00367,(Ult31058:0.00368,LynSpeci:5.5E-4)1.000.900:5.5E-

4)0.967.95:0.04561)0.789.94:0.03534)0.883.141:0.01589)0.829.110:0.00756)0
.927.124:0.01585)0.973.99:5.4E-
4)0.801.89:0.00429,((HthSpec2:0.07596,(HspSpeci:0.0152,HspTapet:5.4E-
4)0.853.105:0.0151)0.733.52:0.00582,(LyngbCf0:0.02399,(UltCya95:0.06168,U
ltCy157:0.02825)0.631.16:0.00994)0.857.133:0.0159)0.682.22:0.00709)0.921.
139:0.01637)0.776.105:0.00777)0.848.109:0.01871)0.831.94:0.01293,((((Ult3
1039:0.00365,(Ult31040:5.5E-
4,(Ult31041:0.00742,Ult31042:0.00371)0.862.130:5.5E-4)0.992.115:5.4E-
4)0.820.77:0.01164,(Ult31047:0.03033,(Ult31045:0.01133,(Ult31046:5.5E-
4,(Ult31044:0.00741,Ult31043:0.00369)0.787.98:5.5E-4)0.975.99:5.5E-
4)0.988.115:0.04452)0.616.14:0.01226)0.958.136:0.03696,(UltCy158:0.08712,
((MisChtho:0.06821,((UltCy178:0.02728,UltCy177:0.00858)0.999.190:0.08092,
((OlrCoral:0.0041,UltOscil:0.01512)0.988.116:0.04367,((((OlrSpon3:0.02303
,OlrSpon4:5.5E-
4)0.820.78:0.00857,OlrSpong:0.0036)0.091.6:0.00633,OlrSpon5:0.0133)0.793.
95:0.00583,OlrSpon2:5.5E-4)0.983.91:0.02381)0.033.4:5.3E-4)0.240.12:5.5E-
4)0.678.23:0.02906,Ult30898:0.07056)0.797.86:0.01394)0.850.138:0.02207)0.
693.27:0.01583,PmiSpe11:0.0517)0.446.13:0.01503)0.457.20:0.00911,(Ult3087
9:0.05911,((UltCya57:0.04177,UltCya58:0.02077)0.993.110:0.0598,(Ult30876:
0.02518,Ult30877:0.00655)0.904.139:0.01997)0.735.61:0.01121)0.839.106:0.0
1101)0.946.116:0.0276,(((((OssCyan3:0.04809,SycSpe23:0.03128)0.896.125:0.
02774,Ult30932:0.04189)0.559.14:0.02199,Ult30688:0.03154)0.831.95:0.01078
,(Ult30884:0.01749,((Ult31003:0.02939,(UltCy219:0.02118,UltCy218:0.01886)
0.676.15:0.00109)0.730.50:0.00822,(Ult30687:0.02632,((UltCya74:0.01214,(A
aySpeci:0.00727,AaySpec2:0.03179)0.972.113:0.02397)0.947.107:0.02516,(Ult
30833:0.01613,(CyhSpeci:0.01741,(Ult30878:0.02594,(UltCya75:0.02657,(AayM
arin:0.02322,(UltAcar2:0.02612,UltAcary:5.4E-
4)0.917.123:0.02141)0.884.135:0.02044)0.826.81:0.01348)0.902.134:0.01661)
0.645.17:0.00643)0.618.10:0.00578)0.919.168:0.01756)0.694.24:5.5E-
4)0.282.11:0.01375)0.871.138:0.01758)0.746.72:0.00467,(UltCya50:0.0548,(U
ltCy216:0.00914,(UltCy214:0.00783,UltCy215:0.02318)0.787.99:0.00578)0.927
.125:0.03556)0.890.140:0.02597)0.902.135:0.02004)0.921.140:0.02215,((SycS
pe22:0.03592,(SycSpe21:5.4E-
4,((Ult30831:0.0,Ult30832:0.0):0.00362,(UltCya73:0.00747,UltSyn39:0.00367
)0.880.151:0.0076)0.943.120:0.01124)0.863.148:0.02542)0.996.128:0.06009,(
(((PudSpec5:5.4E-
4,FlnCyan5:0.03567)0.978.87:0.05958,((PudSpec2:0.00578,Ult31064:0.15735)0
.497.7:0.03116,((((UltAnt28:5.3E-
4,UltAnt27:0.07436)0.871.139:0.02555,(CyuSpec3:0.03955,UltCy234:0.01248)0
.905.137:0.01976)0.654.18:0.01144,PmiMucic:0.0244)0.967.96:0.03834,UltCy2
36:0.0407)0.793.96:0.01664)0.952.119:0.04474)0.834.95:0.01981,((((UltSyn3
8:0.06089,((((Ult30789:0.0,Ult30790:0.0):0.0207,(Ult30791:0.00504,(Ult307
93:0.0087,Ult30792:0.01027)0.899.132:0.01932)0.790.99:0.00964)0.904.140:0
.02083,((SycSpe13:5.4E-
4,SckStr02:0.03983)0.861.152:0.0077,((SycNidul:0.0,SycSpe12:0.0):5.5E-
4,(((UltSyn20:5.4E-
4,((Ult30749:0.0,UdnBac84:0.0):0.00373,((((UltCya51:0.01121,((Ult30700:5.
5E-
4,((Ult30764:0.0,UltSynec:0.0,UltSyne3:0.0,Ult30690:0.0,UltSyne5:0.0,UltS
yne6:0.0,UltSyne7:0.0,UltSyne8:0.0,Ult30691:0.0,UltSyn11:0.0,UltSyn13:0.0
,Ult30693:0.0,Ult30698:0.0,Ult30697:0.0,Ult30696:0.0,Ult30701:0.0,Ult3070
3:0.0,Ult30705:0.0,Ult30706:0.0,Ult30707:0.0):5.5E-
4,((UltMa127:0.00369,UltMa128:5.5E-4)0.923.181:0.00751,(Ult30702:5.5E-
4,(Ult30695:0.03274,UltMa126:5.5E-4)0.829.111:0.00392)0.000.928:5.5E-

4)0.376.6:5.5E-4)0.218.11:5.5E-4)0.124.15:5.4E-
4,UltSyne9:0.00369)0.851.132:0.00396)0.373.18:5.5E-
4,((((UltSyne4:0.0,UltSyn15:0.0,UltSyn18:0.0,Ult30692:0.0,Ult30710:0.0,Ul
t30777:0.0):5.4E-4,(((Ult30767:5.4E-
4,Ult30689:0.00363)0.426.24:0.00371,UltSyn17:0.00363)1.000.901:5.4E-
4,(UltSyne2:0.0,UltSyn10:0.0,Ult30699:0.0):5.4E-
4)0.921.141:0.0037)1.000.902:5.4E-4,(UltSyn19:5.5E-4,(Ult30766:5.4E-
4,(((UltProc7:0.00374,((Ult30742:0.03927,(Ult30721:0.00368,(((Ult30716:0.
00369,((Ult30753:0.00742,(UltProc2:0.00369,(Ult30730:0.0,Ult30737:0.0,Ult
30765:0.0,UltProc3:0.0,UltProc5:0.0):5.5E-4)0.254.9:5.5E-
4)0.914.148:0.0035,(Ult30736:0.00399,UltSyn12:0.01112)0.343.9:0.00746)0.1
51.6:5.4E-4)0.894.134:5.5E-4,(((Ult30711:0.01099,Ult30750:5.5E-
4)0.969.99:0.01873,Ult30757:0.00369)1.000.903:5.5E-
4,(Ult30712:0.0,Ult30714:0.0,Ult30715:0.0,Ult30717:0.0,Ult30718:0.0,Ult30
719:0.0,Ult30720:0.0,Ult30725:0.0,Ult30726:0.0,Ult30729:0.0,Ult30731:0.0,
Ult30727:0.0,Ult30728:0.0,Ult30732:0.0,Ult30733:0.0,Ult30734:0.0,Ult30738
:0.0,Ult30740:0.0,Ult30739:0.0,Ult30741:0.0,Ult30743:0.0,Ult30745:0.0,Ult
30746:0.0,Ult30747:0.0,Ult30751:0.0,Ult30752:0.0,Ult30754:0.0,Ult30755:0.
0,Ult30756:0.0,Ult30759:0.0,Ult30760:0.0,Ult30761:0.0,Ult30762:0.0,Ult307
63:0.0,UltProc8:0.0,UltProch:0.0,UltProc6:0.0):5.5E-4)0.780.127:5.5E-
4)0.781.102:5.5E-4,Ult30713:5.5E-4)0.332.9:5.4E-4)0.980.89:5.5E-
4)0.841.106:0.00367,Ult30758:0.00376)0.786.101:0.00372)0.760.107:0.00394,
Pr0Marin:0.00352)0.844.127:0.01135,(Pr0Mari2:0.00367,UltProc4:0.00374)0.0
00.929:5.3E-
4)0.538.13:0.01119)0.826.82:0.00369)0.854.120:0.00364)0.409.16:0.00372,Ul
tSyn16:0.00363)0.938.90:5.3E-4)0.152.14:5.4E-4,SycSpeci:5.5E-
4)0.903.123:5.4E-4,((((UltSyn21:0.02354,(CybSpec6:5.3E-
4,(PuiChro3:0.01167,(PuiChro2:0.00372,PuiChrom:0.00374)0.847.149:0.00741)
0.984.95:0.02405)0.877.146:0.0159)0.707.22:5.5E-
4,(((Ult30735:0.00397,(((((Ult30779:0.00555,(Ult30723:0.00552,(SycSpec5:5
.5E-
4,UltCya54:0.00733)0.548.13:0.01056)0.870.119:0.00986)0.763.125:0.00624,S
ycSpec8:0.00383)0.810.81:0.01136,(((SckStr00:0.00745,(MiyHolsa:0.00389,Cy
bSpec5:5.5E-4)0.866.137:0.00379)0.540.16:5.5E-
4,(BacEnr53:0.0,SycSpe11:0.0):5.5E-4)0.443.14:5.5E-
4,(SycSpec9:0.00247,Ult30782:0.00247)0.936.126:0.00247)0.916.150:0.00365)
1.000.904:5.4E-4,CybSpec4:5.4E-4)0.907.145:0.00361,((CybSpec3:5.3E-
4,((((SycSpec6:0.00603,((Ult30724:0.0112,((UltSyn36:0.01124,(UltSyn26:0.0
037,((SycSpec4:0.0,UltSyn23:0.0,UltSyn24:0.0,UltSyn35:0.0):5.5E-
4,UltSyn25:0.00369)0.268.6:5.5E-
4)0.804.76:0.00374)0.768.93:0.00372,(UltSyn28:0.00739,(((UltSyn30:0.00369
,SycSpec7:0.00369)0.888.144:5.5E-
4,(UltSyn34:0.00369,UltSyn33:0.00369)0.693.28:5.5E-4)0.000.930:5.5E-
4,(UltSyn29:0.0,UltSyn31:0.0,UltSyn32:0.0):5.5E-4)0.941.110:5.4E-
4)0.720.37:0.00752)0.959.121:0.01936)0.892.128:5.5E-
4,Ult30771:0.00743)0.924.131:0.01045)0.788.99:0.00601,Ult30781:0.00749)0.
096.2:5.4E-
4,(UltChro8:0.0,UltChroo:0.0,UltChro9:0.0):0.01136)0.784.108:0.00372,(((S
ycSpe14:0.0,SycSpe15:0.0):5.4E-
4,((CybSpec7:0.01751,UltCya55:0.01884)0.788.100:0.00705,(CybSpec2:0.0,Syc
Spec2:0.0,Ult30780:0.0):0.0113)0.496.13:5.4E-
4)0.875.146:0.00369,SycSpec3:5.1E-
4)0.912.164:0.00748)0.787.100:0.00376)0.844.128:0.00374,(((Ult30786:0.011
14,Ult30787:5.5E-

4)0.436.15:0.00369,((SycSpe10:0.0,UltSyn37:0.0,Ult30788:0.0):5.5E-
4,(Ult30784:0.00369,Ult30785:0.00369)0.869.108:5.5E-4)0.816.85:5.5E-
4)0.943.121:5.4E-4,Ult30783:0.07454)0.845.122:0.00715)1.000.905:5.5E-
4)0.883.142:0.01578)0.925.146:0.01283,(Ult30704:0.00746,(Ult30769:0.0,Ult
30770:0.0):5.5E-
4)0.752.87:0.00282)0.776.106:0.00411,(CybSpeci:0.0,Ult30768:0.0):0.00709)
0.733.53:0.00835)0.904.141:0.01461,(((Ult30744:0.0,UltSyn22:0.0):5.5E-
4,UltCya53:0.00369)0.970.113:0.01695,((UltSyn14:0.0,Ult30774:0.0,Ult30775
:0.0,Ult30773:0.0,Ult30776:0.0,UltCya52:0.0,UltMa131:0.0):0.00745,Ult3077
8:5.5E-
4)0.822.76:0.00638)0.748.83:0.00435)0.977.95:0.03026,(UltSyn27:5.5E-
4,Ult30772:0.00369)0.988.117:0.03506)0.710.35:0.00534)0.809.89:0.00371)0.
806.74:0.00373)0.762.112:0.00337,(UdnBac83:0.01094,Ult30709:0.00566)0.785
.110:0.00637)0.771.125:0.00372,(Ult30694:0.01505,((UltMa130:0.0,Ult30708:
0.0):5.5E-
4,UltMa129:0.00369)0.866.138:0.00777)0.846.131:0.00779)0.972.114:0.0159)0
.459.17:0.00363)0.908.153:0.02175)0.989.98:0.05452,(SycElong:0.00802,(Miy
Elabe:0.04659,MiyHols2:0.03222)0.995.131:0.06166)0.909.127:0.02495)0.703.
33:0.01418)0.908.154:0.02278,Ult30846:0.12052)0.918.140:0.02893,(((Ult308
65:0.05005,((((Ult30863:0.00626,Ult30864:0.02546)0.969.100:0.034,((Ult308
83:0.00959,(UltCy111:0.01691,Ult30867:0.02322)0.989.99:0.03653)0.926.154:
0.01979,((LpgFrig2:0.02664,Ult30882:0.0326)0.772.115:0.00782,((Ult30866:0
.03537,(UltCya79:0.03093,(LpgSpe17:0.02133,LpgSpe18:0.01776)0.927.126:0.0
1815)0.201.12:0.00385)0.800.80:0.01021,((UltCya78:0.04323,(LpgSpe11:0.022
93,(LpgFrigi:0.01097,UltCya81:0.02413)0.954.101:0.02163)0.893.144:5.4E-
4)0.740.76:0.01025,((Ult30843:0.00345,(OssCyan2:0.01939,UltCya80:5.4E-
4)0.888.145:0.0078)0.766.98:0.0047,(Udntfd18:0.01468,LpgSpe13:0.03244)0.8
86.159:0.01163)0.841.107:0.01201)0.816.86:0.01009)0.885.131:0.00812)0.772
.116:0.00209)0.870.120:0.00685)0.977.96:0.03804,(Ult30860:0.01494,((UltCy
103:0.00719,UltCy104:5.4E-
4)0.956.117:0.03897,(PehSpec2:0.01987,((Ult30859:0.03115,UltCy105:5.3E-
4)0.354.13:0.02114,LpgFoveo:0.01354)0.863.149:0.01266)0.333.10:0.00764)0.
970.114:0.02971)0.942.125:0.02499)0.845.123:0.01461,(UltCy131:5.4E-
4,(((FlnTher3:0.00223,FlnTher2:0.02702)0.983.92:0.00162,FlnTher4:5.5E-
4)0.462.20:5.3E-
4,UltCy130:0.00373)0.992.116:0.02735)0.930.125:0.01983)0.780.128:0.02591)
0.966.132:0.04932,(Ult30858:0.03545,(Ult30856:0.0372,((Ult30857:0.01497,U
ltCy102:5.5E-
4)0.999.191:0.09548,(Ult30854:0.01927,Ult30855:0.04318)0.982.89:0.06739)0
.067.3:0.01643)0.776.107:0.02025)0.707.23:0.01033)0.822.77:0.01295,(Ult30
822:0.07636,(LmtSpeci:5.5E-
4,((Ult30818:0.0,Ult30819:0.0,Ult30820:0.0,Ult30817:0.0):5.5E-
4,Ult30821:5.5E-
4)0.824.84:0.00709)0.962.123:0.06327)0.984.96:0.06019)0.214.14:0.01112)0.
837.89:0.02272,((Ult31062:0.00919,(Ult31063:0.00829,(UltCy235:0.0161,Ult3
1061:0.01041)0.764.117:0.00389)0.496.14:0.00983)0.825.84:0.01752,(UltCy23
7:0.00681,(Ult31066:0.03678,Ult31065:5.4E-
4)0.869.109:0.02088)0.999.192:0.0962)0.960.113:0.04605)0.347.12:0.03029)0
.838.115:0.01618,(((RuiLacun:0.01885,(UltPro97:5.5E-4,UltPro98:5.5E-
4)0.875.147:0.01392)0.959.122:0.04239,(Ult30795:0.07939,((PcxHoll2:0.0,Pc
xHolla:0.0):0.05726,Ult30794:0.00984)0.854.121:0.03583)0.357.13:0.02313)0
.755.91:0.01074,(((Ult31067:5.5E-4,((((PudSpec3:5.5E-
4,AhaGygax:0.00369)0.899.133:5.5E-4,(PudSpec4:5.5E-
4,AabSpiro:0.00369)0.947.108:0.01123)0.815.75:0.00548,((Ult31069:5.5E-

4,Ult31070:0.00369)0.938.91:0.01363,(Ult31068:0.00375,UltAnt29:0.00372)0.
801.90:0.00548)0.848.110:0.00778)0.884.136:0.00872,UltCy238:0.00461)0.428
.17:0.03587)1.000.906:0.11836,((GbtViola:0.00366,UltGloeo:0.00375)0.996.1
29:0.07717,((((Ult30830:0.01472,(UltCya72:0.01508,Ult30829:0.00374)0.980.
90:0.03803)0.976.93:0.04497,(Ult30827:0.00369,Ult30828:5.5E-
4)0.994.125:0.06916)0.941.111:0.04045,(Ult30824:5.5E-
4,Ult30825:0.00369)0.986.104:5.4E-
4)0.102.11:0.00471,(Ult30826:0.00968,(SycSpe19:0.02681,(Ult30823:0.01509,
SycSpe18:0.01179)0.954.102:0.02764)0.941.112:0.03173)0.769.108:0.00863)0.
989.100:0.05791)0.645.18:0.01678)0.729.38:0.00658,SycSpe20:0.06288)0.921.
142:0.02863)0.943.122:0.03083)0.000.931:0.00572)0.150.10:5.4E-
4)0.879.146:0.01387)0.646.16:0.00688)0.833.107:0.01614,((((((CnzMerol:0.0
326,(GldMaxim:0.01582,UltCyani:0.01606)0.685.20:0.0084)1.000.907:0.16754,
(CdmSpec2:0.01479,CdmSpeci:0.01614)0.798.93:0.0121)0.169.6:0.01667,CdmSpe
c3:0.03427)0.717.29:0.0153,(((((((GuiTheta:0.00781,((UltCya34:0.00398,(Ul
t30591:0.00718,UltPho30:5.5E-
4)0.590.12:0.00745)0.761.93:0.00376,(DnpNorve:0.00376,(Ult30592:0.00766,U
ltCya33:0.01145)0.780.129:0.00348)0.867.137:0.00724)0.966.133:0.0153)0.75
8.99:0.00391,(CpmOvata:0.01575,((UltPho28:0.01542,(CpmParam:0.03205,UltPh
o29:0.01905)0.896.126:0.01182)0.758.100:0.00446,((UltCya31:0.01471,(Ult30
589:0.00787,UltEuk11:0.01494)0.730.51:0.00574)0.661.19:0.00293,(UltCya32:
0.02201,((UltPho27:0.0,Ult30590:0.0):0.00689,UltPho26:0.02858)0.302.12:5.
4E-
4)0.875.148:0.00799)0.866.139:0.01099)0.861.153:0.0109)0.928.115:0.01602)
0.870.121:5.4E-4,(PoeSulca:5.5E-
4,UdnCrypt:0.0191)0.914.149:0.01048)0.721.25:0.0039,(RmsSalin:0.00797,Pyr
Salin:0.03546)0.879.147:0.01234)0.938.92:0.01177,(Ult30593:0.00983,(HemVi
res:0.00903,(CmoSpeci:0.0037,CmoPlaco:0.00372)0.800.81:0.00606)0.285.11:0
.00367)0.866.140:5.4E-
4)0.990.113:0.03934,((PavGyran:0.00394,PavLuthe:0.01109)1.000.908:0.13013
,(UltEuk10:0.06362,(Ult30550:0.01939,(((KrmMicr2:0.00349,KrmMicru:0.00392
)0.998.168:0.12102,(GymMikim:0.09211,KrnBrevi:0.09206)1.000.909:0.1556)0.
889.136:0.0388,(UltOr453:0.00463,(((((((Ult30553:0.00743,((Ult30588:0.092
28,Ult30576:0.01027)0.799.96:0.00888,UltOr451:0.00756)0.759.109:0.00363)0
.136.10:5.4E-
4,UltCya28:0.02664)0.817.69:0.00366,(((UltHapto:0.0,Ult30560:0.0,Ult30570
:0.0,UltOr452:0.0,Ult30581:0.0,Ult30585:0.0,Ult30587:0.0):5.4E-
4,(((Ult30545:0.01131,(Ult30544:0.00752,UltPho22:0.00369)0.910.131:0.0114
2)0.922.178:0.01139,Ult30578:0.01124)0.782.124:0.00368,(Ult30577:5.4E-
4,(Ult30552:0.00739,((Ult30547:0.0,Ult30546:0.0,Ult30564:0.0,Ult30565:0.0
):0.01505,Ult30554:0.01124)0.920.157:5.5E-
4)0.578.10:0.00746)0.805.81:0.00364)1.000.910:5.5E-4)0.726.37:5.4E-
4,(((Ult30555:5.4E-
4,((DnpMitr4:0.05682,UltPho25:0.01089)0.693.29:0.00804,Ult30580:0.00359)1
.000.911:5.4E-4)1.000.912:5.5E-
4,(Ult30583:0.03557,Ult30579:0.00757)0.787.101:0.00353)0.114.4:0.00369,((
DnpMitr3:0.00369,(((Ult30566:0.0,Ult30568:0.0,Ult30571:0.0,Ult30573:0.0,U
lt30574:0.0):5.5E-4,Ult30575:5.5E-
4)0.801.91:0.0037,((Ult30558:0.00745,(Ult30559:0.00371,Ult30569:5.5E-
4)0.964.124:0.01943)0.922.179:0.01151,(Ult30556:0.01123,Ult30561:0.00369)
0.971.92:5.4E-4)0.811.75:0.0037)0.808.87:0.00372)0.984.97:5.4E-
4,(UltMa119:0.0112,Ult30582:5.4E-
4)0.761.94:0.00745)0.766.99:0.00358)1.000.913:5.5E-4)0.882.151:5.5E-
4)0.816.87:5.3E-4,(DnpMitra:0.00718,(((Ult30584:5.5E-

4,UltCya30:0.00747)0.908.155:0.00749,(Ult30572:5.4E-4,((((EmiHuxle:5.5E-
4,UltPho23:0.0037)0.798.94:0.00616,(OcrSpeci:0.01623,(Ult30551:0.0144,((D
npMitr2:0.0,Ult30563:0.0):0.00372,UltCya27:0.04856)0.767.100:0.00459)0.79
3.97:0.0114)0.886.160:0.01205)0.239.9:0.00761,(IscSpeci:0.01458,(Ult30548
:0.01139,Ult30549:0.00363)0.894.135:0.01204)0.349.12:0.00841)0.076.6:0.00
899,CshSpeci:0.00942)0.839.107:0.01081)0.790.100:0.00373)0.911.161:0.0021
5,(UdnEuka2:0.00369,(UltMa120:0.0037,(Ult30557:0.0037,(UltCya29:0.00746,U
lt30562:5.3E-
4)0.773.115:0.00371)0.808.88:0.0037)0.776.108:0.00376)0.097.9:0.00207)0.9
59.123:0.00668)0.944.110:5.4E-
4)0.471.19:0.00367,Ult30586:0.00367)1.000.914:5.4E-
4,((Ult30543:0.0,Ult30567:0.0):5.5E-
4,(UltMa122:0.00369,(UltMa121:0.0037,CshPolyl:0.00369)0.144.9:5.5E-
4)0.725.44:5.5E-4)0.914.150:5.4E-4)0.835.99:0.00366,UltPho24:5.4E-
4)0.886.161:0.01027)0.200.10:0.01204)0.736.59:0.00593)0.933.144:0.02802)0
.605.17:0.01455)0.825.85:0.00911)0.895.142:0.01602,((CmgCaeru:0.05283,(Ul
tCya41:0.02321,(ErhCarne:0.01515,RooParvu:0.01634)0.756.104:0.00341)0.743
.87:0.00321)0.846.132:0.00767,((((((NemTortu:0.02154,(TorViola:0.04283,(
UltCya39:0.0112,UltCya40:5.5E-
4)0.963.102:0.02694)0.858.130:0.01308)0.933.145:0.02297,UltOr450:0.07255)
0.764.118:0.00616,(UltParal:0.03557,(PamPalma:0.02589,PemAnnul:0.01432)0.
884.137:0.01404)0.896.127:0.01624)0.747.80:0.01093,((CodOrnat:0.0191,CteR
icht:0.0152)0.865.139:0.01097,(BngSubsi:0.01195,(RrsMarin:0.02666,StyAlsi
d:0.00413)0.879.148:0.01503)0.216.10:0.01496)0.964.125:0.03167)0.487.22:5
.4E-
4,((FliSangu:0.0183,PouAerug:0.02846)0.872.134:0.01553,RllViola:0.05221)0
.903.124:0.0129)0.535.17:0.01124,(((((((PtoSubco:0.02665,(PtoLelia:0.012
34,(GdlAcero:0.02778,(CpeImple:0.01414,(GlmPrist:0.01302,(GlmCrina:0.0052
9,(GlmSerru:0.01513,GlmLatif:5.5E-
4)0.784.109:0.00588)0.207.8:0.00805)0.917.124:0.01605)0.761.95:0.00637)0.
734.69:0.00448)0.386.13:0.00605)0.911.162:0.01196,PtaLucid:0.01503)0.933.
146:5.4E-
4,(PteMelan:0.00993,PteCapil:0.01422)0.872.135:0.00985)0.929.128:0.01158,
(GcrTenui:0.03925,GrrSjoes:0.01308)0.989.101:0.04857)0.000.932:5.5E-
4,((AhfPlica:0.01609,(BonAspar:0.03317,UltCya38:0.04057)0.249.21:0.01548)
0.802.86:0.01111,(HyeSpeci:0.02769,(((PtiPulch:0.0276,(ScoSpeci:0.02426,S
coStege:0.01439)0.942.126:0.02254)0.649.17:0.01746,(((FalHarve:0.00707,((
((CahPikea:0.00157,CphGunni:0.01487)0.946.117:0.00218,CahColla:0.02744)0.
783.122:0.0039,(HetPappe:0.01877,(AghHalli:0.02666,((((UltCya36:5.3E-
4,CenClavu:0.03543)0.840.117:0.00367,(AmlSpiro:0.00186,Ult30601:0.02312)0
.980.91:0.00199)0.812.78:0.00372,UltCya37:0.02285)0.988.118:5.5E-
4,((UltOr455:0.07929,UltOr454:5.4E-
4)0.991.111:0.02807,(CeaDiaph:0.01059,(Ult30600:0.0244,Ult30599:0.02635)0
.500.20:0.00771)0.755.92:0.00382)0.904.142:0.0238)0.936.127:0.01532)0.540
.17:0.00375)0.778.105:0.00407)0.781.103:0.00364,(AipMoore:0.02326,DpsPtil
o:0.0114)0.751.74:0.00358)0.922.180:0.01169)0.896.128:0.01146,(NplDensa:0
.00859,(SesInter:0.00864,SesVirid:0.00671)0.999.193:0.04043)0.465.15:0.01
395)0.096.3:5.3E-
4,((EptArtic:0.00365,CruElisi:0.04861)0.851.133:0.00787,((Ult30595:0.0446
9,EptFergu:0.01513)0.889.137:0.01145,(EptFormo:0.00736,EptMolle:0.01532)0
.873.128:0.00821)0.742.65:0.00301)0.760.108:0.01167)0.892.129:0.02205)0.6
89.23:5.5E-
4,(PoyHarve:0.02758,UltCya35:0.01934)0.839.108:0.01164)0.721.26:0.01501)0
.537.16:0.0075)0.837.90:0.01834)0.925.147:0.01125,((Ult30597:0.04878,PocC

arti:0.02179)0.849.118:0.01507,(ChuCrisp:0.0452,Ult30598:0.05341)0.812.79
:0.01004)0.756.105:0.0056)0.773.116:0.00392,(((DixGrise:0.09492,GlpVacuo:
0.03358)0.992.117:0.06453,((Ult30607:0.05704,((Ult30542:0.01846,(CdmCalda
:5.3E-
4,GldSulph:0.00369)0.770.122:0.00415)0.991.112:0.0543,(CdmCald2:0.07279,G
loWittr:0.05169)0.606.17:0.02913)0.886.162:0.03098)0.813.83:0.01351,Ult30
602:0.04164)0.765.96:0.01155)0.851.134:0.01628,(Ult30596:0.11208,Ult30604
:0.02917)0.943.123:0.0327)0.714.33:0.00395)0.709.31:0.00771,((CiaOffic:0.
01774,Ult30606:0.043)0.842.118:0.0095,((UltOr462:0.0226,(Ult30605:0.00455
,UltOr461:0.00287)0.822.78:0.01106)0.693.30:0.00323,(UltOr460:0.03293,((U
ltPho31:0.01766,UltOr459:0.01673)0.823.93:0.01041,((Ult30603:5.4E-
4,UltOr456:0.05987)0.822.79:0.00721,UltOr458:0.01534)0.841.108:0.01098)0.
554.17:0.00646)0.776.109:0.01701)0.837.91:0.00863)0.982.90:0.0239)0.842.1
19:0.00758)0.745.96:0.01066,(PryPurpu:5.4E-
4,(PryLeuco:0.03135,(BanAtrop:0.0037,BanFusco:0.01892)0.778.106:0.00377)0
.909.128:0.00741)0.998.169:0.05226)0.502.14:0.01039)0.878.123:0.00994)0.7
43.88:0.0087)0.960.114:0.03935,(((GccNosto:5.4E-
4,(((UltDefe9:0.73759,EchgYyyy:2.44468)0.000.933:0.10089,(PsdSpe27:0.9938
6,PyoAsple:0.07523)0.966.134:0.19947)0.950.117:0.23175,((HevHaina:0.0,Ult
30632:0.0):0.08288,Ult30631:0.04173)1.000.915:0.43161)0.900.115:0.12941)0
.586.19:0.00374,(CynGloeo:0.02266,(CypPara2:5.5E-
4,CypParad:0.00746)0.878.124:0.01691)0.994.126:0.03647)0.990.114:0.04878,
(((Ult30850:5.5E-
4,Ult30851:0.00368)1.000.916:0.09407,(UltCy100:0.1369,Ult30852:0.01508)0.
385.18:0.0177)0.971.93:0.04876,((((Ult30677:0.03884,(Ult30675:0.00703,(Ul
tPho49:0.00742,Ult30676:5.5E-
4)0.800.82:0.01221)0.995.132:0.08129)0.998.170:0.08186,((CchSpeci:0.01855
,(BigNatan:0.01551,CchRepta:0.01485)0.850.139:0.0264)1.000.917:0.13513,((
(Ult30674:0.01076,((Ult30670:0.00373,Ult30671:0.00367)0.855.135:0.00823,(
Ult30672:0.03965,(Ult30673:0.0,UltPho48:0.0):0.02422)0.060.6:0.00386)0.95
2.120:0.03314)0.998.171:0.10376,(Ult30594:0.12746,PycProva:0.13893)0.826.
83:0.02955)0.910.132:0.03865,((((((UdnRhodo:0.00851,(((Ult30537:0.0268,((
UltPho19:0.00382,(UltPho18:0.0235,Ult30682:0.01126)0.887.140:0.01147)0.85
7.134:0.00779,(UltEuka8:0.01558,(UltPho20:5.5E-
4,(Ult30535:0.03812,(UltMa118:0.0155,Ult30534:0.01551)0.794.91:0.00914)0.
995.133:0.03806)0.860.103:0.00718)0.743.89:0.00363)0.947.109:0.01371)0.97
5.100:5.5E-4,((Ult30532:0.01746,(Ult30533:5.5E-
4,Ult30722:0.02666)0.991.113:0.03487)0.147.10:0.00683,(Ult30539:0.02322,(
UltPho17:5.3E-
4,UltOr449:0.00365)0.997.134:0.04511)0.902.136:0.0134)0.911.163:0.01532)0
.595.11:0.00787,(UltEuka7:0.02038,Ult30536:0.00834)0.843.108:0.0116)0.862
.131:0.00801)0.724.39:0.00878,(UltEuka9:0.01758,Ult30538:0.08476)0.914.15
1:0.02161)0.521.7:0.0242,(Ult30540:5.5E-
4,UltRho48:0.00368)0.993.111:0.06299)0.948.140:0.04675,((HcdSpeci:0.12609
,(PohZopf2:0.00535,PohZopfi:0.01355)0.996.130:0.15324)0.476.19:0.0352,((U
lt30647:0.00933,(((Ult30649:0.00737,(((UltPho44:0.00422,Ult30650:0.0148)0
.773.117:0.0037,UltMa125:0.00368)0.759.110:0.00373,MnoSquam:0.00426)0.840
.118:0.00698)0.883.143:0.00752,(UltOr463:0.08033,(Ult30648:0.00365,(MmsSp
eci:0.0,UltPho46:0.0):5.5E-4)1.000.918:5.5E-
4)0.840.119:0.0037)0.742.66:0.00373,UltPho45:0.00361)0.976.94:0.03025)0.8
92.130:0.02907,((((UltMa124:0.01121,((((Ult30643:5.4E-
4,Ult30644:0.00704)0.951.119:0.01519,Ult30645:0.00322)0.785.111:0.00409,(
OstTauri:5.5E-4,Ult30642:0.01894)0.970.115:0.0151)0.850.140:5.5E-
4,UltPho43:0.00367)0.780.130:0.00364)0.875.149:0.00741,UltMa123:0.00372)0

.949.127:0.01492,(MaiMet37:0.01113,UltPho42:5.4E-
4)0.778.107:0.00367)0.818.61:0.00698,(UltPrasi:0.00364,Ult30646:5.4E-
4)0.890.141:0.0116)0.808.89:0.01464)1.000.919:0.1629)0.911.164:0.04369)0.
832.93:0.01925,(((((Ult30514:0.00758,(((Ult30501:0.00369,((((Ult30494:0.0
1114,Ult30506:0.01117)0.706.33:5.5E-4,Ult30504:0.00369)0.000.934:5.5E-
4,Ult30518:0.00369)0.000.935:5.5E-4,Ult30517:0.00369)0.000.936:5.5E-
4)0.979.112:5.5E-4,(((((Ult30502:0.0037,Ult30497:0.0037)0.724.40:5.5E-
4,(Ult30496:0.0,Ult30510:0.0,Ult30511:0.0,UltCya23:0.0,UltCya24:0.0):5.5E
-4)0.000.937:5.5E-4,Ult30508:0.00369)0.000.938:5.5E-
4,(Ult30490:0.00743,(((Ult30503:0.0,Ult30498:0.0):5.5E-
4,(((Ult30493:0.00369,Ult30515:0.00369)0.823.94:0.00368,((((Ult30492:0.00
742,Ult30513:5.5E-4)0.818.62:0.00369,Ult30491:0.01117)0.671.14:5.5E-
4,(Ult30489:0.0037,Ult30512:0.00369)0.690.31:5.5E-4)0.000.939:5.5E-
4,(Ult30495:0.0,Ult30499:0.0):5.5E-4)0.984.98:5.5E-
4)0.432.19:0.00369,Ult30516:0.02667)0.886.163:5.4E-4)0.684.18:5.4E-
4,Ult30509:0.00369)0.879.149:0.00369)0.927.127:5.4E-4)0.984.99:5.5E-
4,((Ult30500:0.11628,Ult30507:0.02868)0.567.8:0.00183,Ult30505:0.03438)0.
876.120:5.3E-4)0.926.155:0.00366)0.882.152:5.4E-4,(((Ult30519:5.4E-
4,Ult30520:0.15024)0.943.124:0.01467,Ult30521:5.5E-
4)0.468.21:0.01181,(UltOr448:0.0378,Ult30522:0.01579)0.901.139:0.02069)0.
938.93:0.01996)0.181.9:0.00361)0.914.152:0.0174,UltCya25:0.00651)0.960.11
5:0.02959,((((Ult30486:0.01042,(Ult30485:0.0158,Ult30484:0.00361)0.804.77
:0.01336)1.000.920:0.10195,((((Ult30487:0.00369,UltDia17:5.5E-
4)0.918.141:0.01139,(MaiMet36:5.4E-4,(UltCya21:5.4E-4,(UltDia18:5.5E-
4,UltPho16:0.00369)0.981.105:0.01495)0.950.118:0.01492)0.879.150:0.01142)
0.958.137:0.01912,UltMa117:0.06662)0.730.52:0.00444,Ult30488:0.00739)0.83
2.94:0.01083)0.867.138:0.0149,((((Ult30462:0.00633,UltrS437:7.9E-
4)0.914.153:0.01115,(Ult30461:0.01886,(UltCya14:5.5E-
4,UltCya20:0.02254)0.882.153:0.01571)0.427.17:0.00731)0.668.25:5.3E-
4,(SeaMicro:0.01481,(((Ult30448:0.03123,UltDiat7:0.01507)0.908.156:5.5E-
4,((((UltDia16:0.02499,(UltPhot2:0.02473,Ult30425:0.01422)0.852.127:0.010
42)0.785.112:0.00699,(Ult30460:0.00331,((RzsSeti2:0.0,RzsSetig:0.0):0.003
72,UltPho12:0.00745)0.781.104:0.00412)0.911.165:0.01552)0.774.114:0.00413
,(((DckUlvac:0.0,Ult30432:0.0,UltEuka5:0.0):5.4E-
4,(BcpSpeci:0.01174,(UltDia11:0.00373,(CymSubt2:5.5E-4,CymSubtu:5.5E-
4)0.908.157:0.00738)0.865.140:0.00711)0.412.16:0.00732)0.775.107:0.00698,
((((UltCya15:0.00373,(EucAntar:0.00371,UdnEukar:0.00744)0.768.94:0.00373)
0.382.16:0.00331,(UltOr442:0.00803,(UltEuka3:0.0,UltEuka4:0.0):0.01101)0.
460.14:0.00751)0.997.135:5.5E-
4,(((CaoSocia:0.00743,(CaoSpeci:0.00207,UltOr444:0.00209)0.925.148:0.0069
5)0.820.79:5.4E-4,(Ult30465:0.00369,Ult30475:0.00369)0.709.32:5.5E-
4)0.000.940:5.5E-4,(Ult30466:0.0,UltEuka2:0.0,Ult30476:0.0):5.5E-
4)0.841.109:5.5E-
4)0.805.82:0.00372,Ult30455:0.00749)0.779.109:0.00371)0.761.96:0.00397)0.
885.132:0.00737,(((((((AsiRalfs:0.0,UltDiat9:0.0,Ult30454:0.0):5.5E-
4,Ult30453:0.0037)0.797.87:0.00372,UltDia10:0.00746)0.815.76:0.00367,Ult3
0451:0.00741)0.401.15:5.4E-
4,(FreSedi3:0.01107,((((UdnMari3:0.0,UdnMari4:0.0):5.5E-
4,NllStell:0.00753)0.840.120:0.00368,(((((TalPseud:0.0,Ult30472:0.0):0.00
749,(UltPho14:0.0112,Ult30467:5.5E-4)0.220.12:5.5E-4)0.232.8:5.5E-
4,UltDia15:0.00744)0.405.23:5.5E-
4,(SklPseud:0.0,TalGrav2:0.0,UltOr445:0.0,TalWeiss:0.0,Ult30469:0.0):5.5E
-4)0.372.18:5.5E-4,Ult30468:0.00369)0.442.22:5.5E-
4)0.922.181:0.00538,(((LauAnnul:5.5E-4,Ult30477:5.5E-4)0.517.11:5.5E-

4,PodStell:0.0037)0.182.10:5.5E-4,DtnConfe:0.0037)0.846.133:5.3E-
4)0.012.5:0.0037)0.800.83:0.0038)0.917.125:5.4E-
4,(((((AheSept2:0.00372,AheSepte:5.5E-4)1.000.921:5.4E-
4,((((UltCya10:5.5E-4,((Ult30417:0.0,Ult30418:0.0,UltCya12:0.0):5.5E-
4,Ult30419:0.00369)0.719.33:5.5E-4)0.776.110:5.5E-4,UltCyan9:5.5E-
4)0.850.141:0.00364,(UltCya13:0.00369,(UltOr438:0.01137,UltPhot3:0.01141)
0.790.101:0.00365)0.848.111:5.5E-4)0.869.110:0.00357,(FglPinna:5.5E-
4,FglZeill:0.01493)1.000.922:5.4E-
4)0.420.20:0.00365)0.791.95:0.00357,(DphSpeci:0.0113,(((Ult30459:0.00744,
((UltDia14:5.4E-4,(MlrCf000:5.5E-
4,UltDia13:0.00369)0.944.111:0.00746)0.799.97:0.00369,((AulAmbig:0.0,AulG
ranu:0.0):5.5E-
4,Ult30458:0.00369)0.825.86:0.00371)0.898.150:0.00745)0.903.125:5.4E-
4,PiaSulca:5.5E-
4)0.799.98:0.00372,UltOr426:0.00747)0.789.95:0.00365)0.881.145:0.00759)0.
794.92:0.00368,((UltOr439:0.00363,UltPhot4:0.01124)0.798.95:0.00377,(HasC
ruci:0.00369,(((UltEukar:5.5E-
4,UltDiat8:0.01906)0.826.84:0.00369,(GsgFasci:0.0074,(UltOr424:0.01116,((
((UltMic13:0.00369,((((((Ult30434:5.4E-
4,UltOr432:0.01511)0.783.123:0.0037,UltOr446:0.00372)0.829.112:0.00369,((
(UltOr425:0.00745,Ult30443:0.00743)0.978.88:5.5E-4,(UltOr440:5.4E-
4,((UltDiat5:5.5E-
4,(((PayTric2:0.0,PayTrico:0.0):0.00722,PgmInter:0.01506)0.895.143:5.4E-
4,((UltOr428:0.0,UltOr429:0.0):5.5E-
4,UltOr430:0.03248)0.840.121:0.00389)0.327.15:5.5E-4)0.518.17:5.4E-
4,(UltOr427:0.0076,Ult30427:0.0076)0.769.109:0.00361)0.861.154:0.00382)0.
306.5:0.00389)0.819.87:0.0038,((UltPhoto:0.01132,UltCyan7:0.01512)0.851.1
35:0.0075,Ult30430:5.4E-4)0.848.112:0.00368)0.298.16:5.5E-
4)0.132.17:5.5E-4,((UltDiat4:0.00369,UltCyan4:0.00377)0.129.9:5.5E-
4,UltOr431:0.00741)0.856.113:0.00369)0.494.12:5.4E-
4,(Ult30478:0.01868,Ult30450:0.01482)0.859.124:5.5E-4)0.453.14:5.4E-
4,((((NavPelli:5.5E-4,DitBrigh:0.01122)0.986.105:5.5E-4,(UltOr422:5.5E-
4,UltOr423:0.03051)0.461.12:0.00738)0.745.97:0.00367,Ult30431:0.0037)0.80
2.87:0.00368,Ult30420:0.00371)0.786.102:0.00368)0.021.6:5.3E-
4,(((Ult30452:0.00367,(UltCya16:5.5E-4,UltCya18:0.00369)0.846.134:5.5E-
4)0.787.102:0.00365,CyaSolea:0.01135)0.790.102:0.00373,(UltPho10:0.00744,
UltOr441:0.00369)0.799.99:5.4E-
4)0.856.114:0.00367)0.862.132:0.00386)0.149.15:5.3E-
4,(Ult30422:0.0037,(Ult30423:0.0,Ult30424:0.0,Ult30480:0.0):5.5E-
4)0.851.136:0.00369)0.240.13:5.2E-
4,(UltCyan5:0.0074,(UltCyan6:0.00369,Ult30421:0.00741)0.093.9:5.5E-
4)0.857.135:0.00369)0.363.18:5.5E-
4,(Ult30428:0.02251,Ult30429:0.04385)0.943.125:5.5E-4)0.692.17:5.4E-
4)0.428.18:0.00775)0.562.18:5.5E-4)0.439.17:5.5E-
4,(HasWawri:0.0,AeiGlac2:0.0,AeiGlaci:0.0):5.5E-4)0.000.941:5.5E-
4)0.458.29:5.4E-4)0.765.97:5.4E-
4)0.795.100:0.00313,(Ult30433:0.07821,Ult30470:0.0522)0.905.138:0.02653)1
.000.923:5.4E-4)0.443.15:0.00354,((UltPhot9:0.0037,((UltOr436:5.5E-
4,UltOr435:0.00741)0.987.135:5.5E-
4,(((Ult30441:0.00368,FrlCylin:0.01141)0.793.98:0.00377,(UltPhot6:0.01101
,(UltPhot7:0.0,UltPhot8:0.0):5.5E-4)0.842.120:0.00364)0.863.150:5.4E-
4,((((((EnnClone:5.3E-
4,UltPho11:0.01098)0.980.92:0.02774,((Ult30446:5.5E-
4,(UltCya11:0.00745,(SyeHyper:5.5E-

4,(Ult30444:0.0,Ult30445:0.0):0.00369)0.553.14:5.4E-
4)0.860.104:0.00369)0.912.165:0.00347,Ult30447:5.4E-
4)0.460.15:0.01182)0.630.15:0.01174,((Ult30439:5.5E-
4,(PmtPandu:0.0037,UltOr434:5.5E-
4)0.815.77:0.00369)0.938.94:0.00748,((Ult30435:0.0,ExtCribr:0.0):5.5E-
4,Ult30436:5.5E-4)0.845.124:5.4E-4)0.928.116:0.00348)0.821.81:5.5E-
4,((Ult30457:5.4E-4,((Ult30456:0.0,Ult30473:0.0):5.5E-
4,Ult30474:0.00369)0.837.92:0.00369)0.894.136:0.0114,Ult30438:0.02327)0.8
56.115:0.00745)0.390.21:5.5E-
4,(((StuConst:0.01137,UltOr447:0.00749)0.795.101:0.00374,((UltCyan8:0.003
64,UltDiat3:5.5E-
4)0.368.11:0.00369,(Ult30471:0.0,Ult30479:0.0):0.00741)0.924.132:5.5E-
4)0.899.134:5.5E-4,(UltOr433:0.0,Ult30440:0.0):5.5E-4)0.877.147:5.4E-
4)0.926.156:5.5E-4,(CylClost:0.00362,((((BlrPaxil:0.0,NtzClost:0.0):5.5E-
4,(UltPhot5:0.00369,Ult30437:0.00741)0.923.182:5.5E-4)0.000.942:5.5E-
4,UltOr443:5.5E-4)0.712.26:5.5E-4,((Ult30442:0.01125,UltOr437:5.4E-
4)0.950.119:0.01126,((HasOstre:0.0,OdnSinen:0.0):5.5E-
4,AhtBrevi:0.01113)0.808.90:0.00367)0.987.136:5.5E-4)0.931.137:5.4E-
4)0.860.105:0.0036)0.839.109:0.00359)0.822.80:0.00356)0.806.75:0.00356)0.
791.96:0.00357,(Ult30464:0.00369,((Ult30426:0.01139,Ult30463:0.00367)0.89
8.151:0.00756,(UltCya17:5.5E-
4,UltDiato:0.00743)0.827.95:0.00369)0.478.16:5.4E-4)0.983.93:5.4E-
4)0.859.125:0.0035)0.996.131:5.4E-
4)0.836.94:0.00343)0.843.109:0.00345,(((UltDiat2:0.02692,(UltPho13:0.0488
3,Ult30449:0.00395)0.869.111:0.01949)0.151.7:0.00385,UltDiat6:0.05653)0.8
62.133:5.3E-
4,((StxNippo:0.01522,TalNorde:0.00362)0.795.102:0.0038,TalGravi:5.4E-
4)0.743.90:0.00369)0.995.134:5.4E-
4)0.779.110:0.00348)0.861.155:0.00664)0.843.110:0.00746,((CsnRadia:0.0115
6,((PobAlata:0.0,PobInerm:0.0):0.01503,(UltDia12:0.01515,PobIndic:0.00373
)0.758.101:5.3E-
4)0.956.118:0.01949)0.763.126:0.00712,CotPenna:0.0392)0.000.943:5.4E-
4)0.860.106:0.00887)0.000.944:0.0033,((EccSilic:0.01438,PylLitto:0.01238)
0.741.68:0.00569,(FucVesic:0.01089,Ult30529:0.04556)0.225.10:0.01521)0.99
8.172:0.05349)0.932.137:0.01839)0.925.149:0.01891,((Ult30530:0.00808,Ult3
0531:0.01882)0.970.116:0.02955,(((Ult30523:0.03999,(Ult30524:5.5E-
4,UltCya26:0.06086)0.457.21:0.0037)0.860.107:0.00791,Ult30528:0.0266)0.85
2.128:0.0095,((Ult30527:0.01112,Ult30525:5.5E-
4)0.944.112:0.02095,Ult30526:0.02963)0.662.21:0.01207)0.898.152:0.02059)0
.904.143:0.01722)0.902.137:0.01657,(UltPho21:0.11787,(UltCya22:0.00377,Fb
rJapon:0.10706)0.862.134:0.01683)0.768.95:0.00831)0.893.145:0.01435)0.758
.102:0.00637,((AueLagun:0.00353,(Ult30483:0.04841,UltEuka6:0.07976)0.556.
14:0.01527)0.235.13:0.01247,(UltCya19:0.02312,(Ult30482:0.01254,(Ult30481
:5.5E-4,UltPho15:5.5E-
4)0.798.96:0.00626)0.195.9:0.00695)0.962.124:0.03324)0.995.135:0.06132)0.
979.113:0.04272)0.710.36:0.00834)0.792.110:0.01275)0.804.78:0.01525,(((Tc
nAbrup:0.13818,(((((((LncTript:0.05403,((((LncStein:0.01292,LncFusif:0.05
337)0.488.21:0.00646,LnclOvum:0.01644)0.943.126:0.02198,LnclAcus:0.02277)
0.815.78:0.01053,LncSpiro:0.01871)0.985.108:0.05358)0.962.125:0.04932,Eug
Limno:0.02691)0.757.79:0.02491,((PcuAppla:0.02397,(PcuRanul:0.02491,(PcuH
amel:5.4E-
4,PcuSegre:0.01112)0.488.22:0.00975)0.479.21:0.00754)0.900.116:0.01501,(P
cuSimil:0.02291,(PcuGranu:5.5E-4,((PcuParvu:0.02676,PcuPusil:5.5E-
4)0.978.89:0.03129,PcuSkuja:0.01566)0.844.129:0.01082)0.746.73:0.00382)0.

994.127:0.03535)0.852.129:0.00946)0.748.84:0.00487,(PcuAcumi:0.03309,(Dcp
Adunc:0.05819,DcpSpath:0.03871)0.764.119:0.01617)0.809.90:0.02303)0.371.1
4:0.00612,(PcuTriqu:0.0747,PcuWarsz:0.03441)0.424.23:0.01678)0.821.82:0.0
1057,(PcuPleur:0.07435,(PcuOrbi2:0.00696,PcuOrbic:0.01191)0.971.94:0.0391
2)0.492.19:0.01717)0.933.147:0.02166,(((EugVirid:0.01878,((EugDese2:0.007
98,EugDeses:0.01465)0.957.119:0.0192,((EugGymno:0.03566,(EugCant2:0.00382
,(EugCanta:0.00762,EugCant3:0.00747)0.910.133:0.01136)0.953.99:0.01916)0.
821.83:0.00987,EugClara:0.02631)0.794.93:0.01346)0.639.22:5.4E-
4)0.402.15:0.00827,((((EugLacin:5.5E-
4,EugSpeci:0.01124)0.947.110:0.02293,EugSocia:0.04958)0.906.153:0.01577,(
((((EugClava:0.02812,(CliCalvu:0.04295,(CliVesic:0.00614,CliMucro:0.01297
)0.325.14:0.00978)0.963.103:0.03245)0.819.88:0.01347,((SmmAcumi:5.5E-
4,(SmmOvali:0.00744,SmmCosta:0.00374)0.570.9:0.0037)0.980.93:0.03267,(Eug
Cauda:0.02399,EugCaud2:0.00787)0.928.117:0.02316)0.853.106:0.01704)0.028.
8:5.5E-
4,(MonAenig:0.05361,(EugAnab2:0.02637,EugAnaba:0.01)0.842.121:0.01876)0.6
97.18:0.01998)0.907.146:0.0156,(((CpgSkuja:0.01376,(CpgPigra:0.0039,CpgPi
gr2:0.03532)0.434.15:0.00586)0.984.100:0.03852,(MonOvata:5.4E-
4,MonPyrum:0.0113)0.919.169:0.01555)0.580.9:0.00422,MonPseud:0.00849)0.86
4.127:0.01068)0.644.19:5.4E-
4,((((EugChad2:0.01499,((EugStel2:0.00863,(EugTrist:0.01124,EugStell:5.5E
-
4)0.984.101:0.02657)0.918.142:0.02316,EugGranu:0.08763)0.850.142:0.01243)
0.752.88:0.00441,(EugChade:0.01982,EugTris2:0.03423)0.864.128:0.01109)0.7
70.123:0.00623,(EugSpec2:0.03741,(EugMuta2:0.01098,EugMutab:5.4E-
4)0.994.128:0.05476)0.817.70:0.03106)0.871.140:0.01713,EugSpec3:0.03071)0
.825.87:0.00793)0.886.164:0.01033)0.731.41:0.00327,((EugSplen:0.07004,((T
cnEllip:0.1207,(TcnSimil:0.03549,(TcnVolvo:0.00363,(TcnOblon:5.5E-
4,TcnVolv2:0.00742)0.999.194:5.5E-
4)0.910.134:0.02525)0.980.94:0.06111)0.952.121:0.04528,(EugGeni3:0.01942,
(EugGeni2:0.017,EugGenic:0.01449)0.954.103:0.02486)0.970.117:0.03533)0.69
1.19:0.01208)0.899.135:0.01866,(EugAdhae:0.01355,((EugCarte:0.04649,(EugL
onga:0.09603,(EugAgili:0.02819,((EugAgil4:0.03432,(EugAgil2:0.02638,EugAg
il3:0.04407)0.797.88:0.01295)0.772.117:0.00935,EugGraci:0.05517)0.144.10:
0.0061)0.825.88:0.01411)0.930.126:0.02512)0.729.39:0.00509,((TcnLefev:5.4
E-
4,(TcnBerna:0.06989,TcnPlanc:0.00352)0.946.118:0.01871)0.998.173:0.08881,
(TcnRugul:5.5E-
4,TcnVolv3:0.04963)1.000.924:0.12045)0.885.133:0.02293)0.875.150:0.00929)
0.948.141:0.02304)0.777.119:0.00772)0.746.74:0.00409)0.862.135:0.0082,(Eu
gPseud:0.02963,((TcnHispi:0.02693,(TcnEchin:0.07484,TcnZoren:0.03987)0.85
0.143:0.05384)0.995.136:0.09605,EugProxi:0.10895)0.301.16:0.03067)0.559.1
5:0.00563)0.938.95:0.03238)0.773.118:0.03733)0.910.135:0.03875,(EutBraar:
0.0868,EtpVirid:0.16876)0.532.16:0.01281)0.888.146:0.02983,EutPomqu:0.032
09)0.904.144:0.03549)0.787.103:0.0096,(((Ult30684:0.0,Ult30685:0.0,Ult306
86:0.0):0.0791,((((NphPyrif:0.04801,(NphOliva:0.02713,UltPho40:5.3E-
4)1.000.925:0.0999)0.906.154:0.02678,(ChkAtmop:0.03245,MeiVirid:0.0255)0.
930.127:0.02424)0.718.37:0.00368,((((((LsrTerre:0.07804,((OltVirid:0.1177
6,((PsuAkine:0.05642,(Ult30681:0.02369,(Ult30680:0.01578,(Ult30679:0.0074
8,UltPho50:0.01127)0.762.113:0.003)0.886.165:0.01145)0.972.115:0.04124)0.
965.106:0.03898,(PlyMirum:0.20733,((UltDunal:0.04306,CadAppla:0.05986)0.4
83.17:0.02691,(CadMoewu:0.03846,PlyOvifo:0.06126)0.864.129:0.02169)0.916.
151:0.0407)0.912.166:0.02722)0.724.41:0.00833)0.967.97:0.03291,(UltOr476:
0.02246,ScfDubia:0.03626)0.965.107:0.04727)0.110.11:0.01029)0.897.136:0.0

293,((UltPho47:0.11159,((CadReinh:0.00431,VlvCarte:0.03044)0.988.119:0.06
704,(Ult30653:0.06144,(SceObliq:0.05493,Ult30652:0.10397)0.948.142:0.0593
2)0.632.15:0.02722)0.350.13:0.01832)0.865.141:0.02165,(OedCardi:0.19826,S
gcHelve:0.04463)0.953.100:0.04205)0.870.122:0.02266)0.760.109:0.02263,(Ul
tCh567:0.02588,(((Ult30637:0.00374,Ult30638:5.5E-
4)0.781.105:0.00595,(Ult30636:0.05059,PioEukar:0.02672)0.425.15:0.00696)0
.862.136:0.02002,((AuxSpeci:0.04512,(AuxProto:0.00477,PohWicke:0.01015)0.
554.18:0.00716)0.991.114:0.04718,((CseAcicu:0.03537,PcoKessl:0.0607)0.998
.174:0.09794,((Ch0Vulga:5.5E-4,(Ch0Sorok:5.4E-
4,UltCya45:0.00742)0.782.125:0.00752)1.000.926:5.3E-
4,(UltCya46:0.08369,UltCya47:0.00423)0.836.95:0.00272)0.489.16:0.01252)0.
846.135:0.01571)0.823.95:0.01576)0.975.101:0.04236)0.817.71:0.01307)0.157
.13:0.00623,UltCh568:0.08045)0.741.69:0.00409,(OocSolit:0.03046,(XxxChlo3
:5.4E-4,(UltProk7:0.01517,(Ult30639:5.5E-
4,(PdhSpeci:0.0037,XxxChlo2:0.00372)0.771.126:5.5E-4)0.930.128:5.5E-
4)0.987.137:0.01904)0.872.136:0.00839)0.373.19:0.00437)0.839.110:0.00879,
((UltPho41:0.05442,(((UltCh563:0.03076,(UltCh566:0.01117,(UltCh564:0.0111
4,UltCh565:5.5E-4)0.809.91:0.00367)0.854.122:5.4E-
4)0.998.175:0.0506,((UltPho39:5.4E-4,(UltPho38:5.5E-
4,UltCh562:0.0037)0.909.129:0.00337)0.499.15:0.01044,(CcxGlaro:5.4E-
4,ClySpeci:0.0471)0.998.176:0.04455)0.931.138:0.02011)0.852.130:0.01673,(
Ult30635:0.13088,(Ult30633:0.00302,Ult30634:0.0082)0.912.167:0.04999)0.09
3.10:0.01937)0.966.135:0.04341)0.833.108:0.01336,((Ult30640:0.01176,(UltC
ya48:0.01473,UltEu131:0.00858)0.771.127:0.0069)0.982.91:0.06004,Ult30641:
0.09814)0.601.8:0.02208)0.822.81:0.01166)0.753.96:0.00454)0.818.63:0.0106
2,((PdiMinor:0.08554,Ult30541:0.09138)0.889.138:0.03387,(((CleOrbic:0.036
47,((MeoCalda:0.01186,HnrAfri2:0.22388)0.878.125:0.03399,(SigMaxim:0.0208
1,(UltCya42:0.02322,UltCya43:0.00336)0.864.130:0.01135)0.863.151:0.0156)0
.818.64:0.0098)0.767.101:0.00628,(CpdGlobo:0.02473,((Ult30628:0.00369,Ult
30629:5.5E-4)0.857.136:5.4E-4,((BtmPomif:5.4E-
4,(((UltPho36:0.01075,SatPunct:0.01638)0.477.18:0.00621,(UltPho37:0.03146
,ZygCircu:0.02705)0.871.141:0.01439)0.429.16:0.00823,(KlsFlacc:0.09968,Et
nFimbr:0.10923)0.860.108:0.05957)0.739.58:0.00725)0.801.92:5.4E-
4,(((((((OroMinor:0.00714,(PlaRugel:0.0059,PegonX00:0.01835)0.868.116:0.0
0536)0.949.128:6.0E-
4,OenElata:0.00369)0.820.80:0.00369,((CniAmeri:0.00753,EpfVirgi:0.01916)0
.971.95:0.01914,(((MocForst:0.0,RiiSoroc:0.0):5.5E-
4,(AieEvect:0.00362,(((TeuLacun:0.00744,((CusGrono:0.01512,((PflBiflo:0.0
4347,(ScdVerti:0.06107,((((EphDista:5.4E-
4,(GntLeybo:0.0113,WelMirab:0.00367)0.757.80:0.00363)0.942.127:0.02539,((
AusSpica:0.02878,CepHarri:0.0174)0.883.144:0.00136,((((CyeFortu:5.5E-
4,CunLance:0.01496)0.837.93:0.00368,((TaiCrypt:0.00591,ToeNucif:0.01443)0
.796.74:0.00585,(Ult30616:0.03532,(MibDecus:5.5E-
4,TtcArtic:0.0037)0.547.10:5.4E-
4)0.909.130:0.00746)0.767.102:0.00371)0.976.95:5.4E-
4,(AibPyram:0.02705,WidNodif:0.0076)0.753.97:0.01118)0.911.166:0.01666,(T
axWalli:0.031,PtxChien:5.4E-
4)0.897.137:0.0095)0.818.65:0.00245)0.990.115:0.02937)0.773.119:0.00575,(
MchTetra:0.03083,((AfrFalca:0.0,NageNagi:0.0):5.4E-
4,(LagFrank:0.01831,AopPanch:0.01014)0.788.101:0.00528)0.788.102:0.00744)
0.820.81:0.00667)0.879.151:0.00843,(ArrArauc:0.0286,LphLaxif:0.01082)0.16
7.10:0.00327)0.918.143:0.01269)0.832.95:0.00787)0.751.75:0.00424,(((PvcLy
ell:5.5E-
4,SmhCirci:0.00747)0.796.75:0.0037,(((PiuSativ:0.01143,CicAriet:0.00351)0

.871.142:0.00752,(MedTrunc:5.5E-4,MedTrun2:0.03046)0.916.152:5.5E-
4)0.274.8:0.00748,TfmSubte:0.015)0.870.123:0.00759)0.789.96:0.00373,(((Ae
aPhyll:0.03028,LygJapon:0.00887)0.804.79:0.01155,(((CibGlauc:0.01116,(((A
diPedat:5.4E-
4,VttLinea:0.0112)0.441.21:0.0037,AdiCapil:0.01497)0.781.106:0.00392,(Pt0
Ensif:0.00369,MaeMutic:5.5E-
4)0.873.129:0.00741)0.648.21:0.00749)0.952.122:5.4E-
4,(AslNidu2:0.01933,DavFejee:0.00762)0.869.112:0.00726)0.576.14:0.00763,(
GleDicar:0.00755,HmpSpeci:0.00756)0.767.103:0.00365)0.917.126:0.01267)0.7
74.115:0.00651,(SeeUligi:0.04193,(SeeApoda:0.02238,SeeUncin:0.03668)0.815
.79:0.01175)0.874.154:0.01357)0.869.113:0.00744)1.000.927:5.5E-
4)0.765.98:0.00372)0.468.22:0.00403,(Pe0Neesi:0.02293,((FosPusil:0.00373,
Pe0Epiph:0.00373)0.757.81:0.00318,HnpFlabe:0.01564)0.381.20:0.00377)0.797
.89:5.4E-4)0.871.143:5.3E-4)0.010.8:5.4E-
4,(SscNobil:0.01514,((BazTrilo:5.4E-
4,NowCurvi:0.00743)0.831.96:0.00369,(((CagMuell:0.00371,SonHyali:0.0037)0
.808.91:0.0037,BleTrich:0.01883)0.802.88:5.5E-4,SniNemor:5.5E-
4)0.928.118:5.3E-4)0.800.84:0.0037)0.766.100:0.0037)1.000.928:5.4E-
4,(HapMnioi:0.01128,PtuPulch:0.00378)0.810.82:0.00355)0.460.16:0.00388)1.
000.929:5.4E-
4)0.854.123:0.00387,((((((((Ult30612:0.00196,TacCaeru:0.0188)0.963.104:0
.00193,((((RcnComm4:0.08861,((GcnMax02:0.08166,(SoaLyco5:0.04618,SoaLyco4
:0.07568)0.787.104:0.01429)0.840.122:0.0101,RcnComm5:0.00727)0.844.130:0.
00741)0.770.124:0.00391,(PutAgent:0.00749,((SoaLyco3:0.0418,RcnComm2:0.07
977)0.873.130:0.01393,(((CyrCocci:0.05279,CyiRuber:0.08049)0.759.111:0.00
843,((CcsMelo3:0.0699,RcnComm3:0.07283)0.515.12:0.01479,RcnCommu:0.02019)
0.774.116:0.00654)0.069.6:0.00723,((MloNudif:0.00763,CusEurop:5.4E-
4)0.506.14:0.00369,Ult30610:5.4E-4)1.000.930:5.4E-
4)0.850.144:0.00346)0.878.126:5.5E-4)0.516.9:5.5E-4)0.383.12:5.5E-
4,(AgiEnvir:0.0,VtsVinif:0.0,SoaTuber:0.0,Ult30623:0.0):5.5E-
4)0.370.14:5.5E-4,CcsSativ:0.01878)0.000.945:5.5E-4)0.343.10:5.4E-
4,(SoaLyco8:0.02274,(PosTrich:0.01136,SoaLyco7:0.0113)0.448.18:0.00371)0.
806.76:5.4E-4)0.080.7:5.5E-
4,(SvlAemul:0.01474,(Ult30613:0.00369,JasNudif:0.01883)0.948.143:0.00198)
0.947.111:0.00193)0.000.946:5.5E-4,CacPapay:0.00739)0.000.947:5.5E-
4,DauCarot:0.00369)0.455.25:5.5E-4,(Ult30622:5.5E-
4,SoaLyco6:0.02275)0.835.100:0.00369)0.479.22:5.5E-
4,PomCommu:0.00369)0.504.13:5.5E-4,(BssNapus:5.5E-
4,AeuCampy:0.01116)0.832.96:0.00369)0.830.94:0.00386)0.832.97:5.4E-
4)0.458.30:5.5E-4)0.224.10:5.5E-4,Ult30614:5.5E-4)0.150.11:5.3E-
4,(((PsoVulga:0.00372,AacHypog:0.00368)0.272.16:5.5E-
4,(LotJapon:0.00369,GcnMax00:0.00744)0.908.158:5.5E-4)0.987.138:5.5E-
4,Ult30608:0.03847)0.830.95:0.00367)0.563.15:5.4E-4,(((((PpvSomni:5.4E-
4,(((CljBiddl:0.0,LcjClype:0.0):0.00379,RadCompl:0.01124)0.924.133:0.0112
5,(Ult30630:0.00741,(AhoFormo:0.00369,MgcAenig:5.5E-4)0.874.155:5.5E-
4)0.974.76:0.0151)0.931.139:5.3E-
4)0.924.134:0.00369,((CcaTaitu:0.0,SgrEriop:0.0):5.5E-
4,((Ult30609:0.07839,((EquHyema:0.00751,(MtzConju:0.01842,(AuaMirab:0.018
82,RcrLatif:5.4E-
4)0.263.9:0.01027)0.785.113:0.00534)0.880.152:0.00748,(Ult30615:5.4E-
4,((UltPho33:0.00744,(Ult30624:0.00745,(((NotLongi:0.0,UltPho35:0.0):5.5E
-
4,((UltPho34:0.00739,(Ult30617:0.00369,((OrzSati6:0.00369,(((FesArund:0.0
2784,Ult30625:0.00368)0.558.12:5.4E-

4,(Ult30621:0.0,OrzSati5:0.0,Ze0aMay4:0.0,Ult30627:0.0,Ult30626:0.0):5.5E
-4)0.137.10:5.5E-
4,(Ze0aMay3:0.02669,OrzSati4:0.00744)0.790.103:0.00367)0.365.14:5.5E-
4)0.107.14:5.3E-4,Ze0aMay2:0.01498)0.819.89:0.00386)0.000.948:5.5E-
4)0.000.949:5.5E-4,UltPho32:5.5E-4)0.422.17:5.5E-4)0.000.950:5.5E-
4,PinSquam:0.00744)0.390.22:5.5E-4)0.201.13:5.5E-4)0.699.32:5.4E-
4,Ult30618:0.06898)0.814.76:0.00381)0.210.18:5.5E-
4)0.392.15:0.0038)0.697.19:5.4E-
4,((((HntZeyla:0.00375,PsiNudum:0.00749)0.900.117:0.00747,IstMelan:0.0191
5)0.765.99:5.5E-
4,(HprLuci2:0.01091,(AcoCalam:0.01158,TakCerat:0.00718)0.271.10:0.0037)0.
866.141:5.5E-
4)0.846.136:0.00372,(Ult30620:0.06034,Ult30619:0.06521)0.962.126:0.03072)
0.881.146:5.5E-4)0.621.16:5.4E-4)0.000.951:5.5E-4)0.473.14:5.4E-
4,(DctSpec2:0.00369,TmnMoor2:0.015)0.809.92:0.00369)0.430.17:5.5E-
4,(Ult30611:0.0037,LmnMinor:0.0037)0.810.83:0.00369)0.105.14:5.5E-
4,NymOdora:0.0037)0.852.131:0.00386)0.785.114:0.00398,DirScopa:0.00735)0.
061.6:0.0039)0.922.182:0.01165)0.920.158:0.02403)0.833.109:0.01582)0.878.
127:0.01805,(MiaYamam:0.17015,(UltCya44:0.01404,NieSpeci:0.01781)0.933.14
8:0.02688)0.758.103:0.00739)0.945.103:0.0279)0.753.98:0.0104)0.799.100:0.
01265)0.821.84:0.01401,((((UltOr464:0.01356,(Ult30654:0.00792,Ult30655:0.
00704)0.776.111:0.0058)0.935.124:0.04071,(PyaParke:0.01596,EnnClon2:0.051
37)0.970.118:0.04809)0.796.76:0.01887,((UltCya49:0.04519,(MnxSpeci:0.0278
,UltPho51:0.0277)0.883.145:0.02067)1.000.931:0.12093,Ult30678:0.13154)0.6
16.15:0.008)0.842.122:0.02736,((BryPlumo:0.01265,(CdiFragi:0.00815,(CdiAr
abi:0.01584,CdiSimul:0.01433)0.496.15:0.00757)0.974.77:0.03791)0.951.120:
0.0265,((((RpcPhoen:0.03708,RpsJaven:0.00647)0.991.115:0.03988,(FaiPetio:
0.0124,(UltOr475:0.01907,(RplCrass:0.00722,RppProfu:0.02692)0.879.152:0.0
0738)0.933.149:0.01399)0.080.8:0.00346)0.799.101:0.01107,(Ult30669:0.0180
4,(CueSertu:0.02299,(CulAmbig:0.00516,Ult30668:0.02141)0.997.136:0.06527)
0.976.96:0.04215)0.752.89:0.00655)1.000.932:0.11005,(((Ult30663:0.03214,(
(Ult30662:0.04698,(Ult30661:0.00733,(OsrSpeci:0.01911,UltOr467:0.02811)0
.752.90:0.00334)0.956.119:5.4E-
4)0.805.83:0.00358,(((Ult30659:0.0092,Ult30660:0.0276)0.212.9:0.00407,Ult
30658:0.01167)0.812.80:0.00709,(Ult30656:0.01474,(UltOr468:0.00951,(UltOr
466:0.02157,UltOr469:0.04241)0.732.40:0.01147)0.774.117:0.00582)0.108.10:
0.00368)0.850.145:5.5E-4)0.854.124:5.4E-
4,UltOr465:0.01137)0.988.120:0.02352)0.717.30:0.00205,((Ult30667:0.01116,
(UltOr457:0.03167,UltOr472:0.04026)0.731.42:0.00279)0.781.107:0.00393,(Ul
tOr473:0.01085,UltOr474:0.01149)0.525.12:0.0037)0.873.131:0.01178)0.568.1
7:0.01269,((AvrLacer:0.01009,AvrNigri:0.01736)0.998.177:0.06409,(UltOr471
:0.03442,(Ult30666:0.05252,(UltOr470:0.01856,(Ult30664:0.00373,Ult30665:0
.00366)0.994.129:5.4E-
4)0.820.82:0.01665)0.992.118:0.06464)0.928.119:0.02907)0.918.144:0.01971)
0.931.140:0.02793)0.781.108:0.01181)0.981.106:0.05966)0.872.137:0.02379)0
.862.137:0.02077)0.949.129:0.03085)0.622.12:0.00265)0.926.157:0.02379)0.8
14.77:0.01622,((Ult30813:0.00979,(Ult30812:0.01613,UltCya66:0.02743)0.908
.159:0.01419)0.992.119:0.08506,(LgrCyano:0.06377,((Ult30816:0.01498,UltCy
a71:0.05009)0.052.11:0.01177,(((((UltCya67:0.0,UltCya68:0.0,UltCya69:0.0)
:5.4E-
4,(PceSpeci:0.00377,((SycSpe16:0.00717,LpgSpec9:0.02318)0.946.119:5.4E-
4,PehSpeci:0.01137)0.792.111:0.0077)0.919.170:0.01135)0.696.22:5.4E-
4,LpgSpec8:0.01922)0.920.159:0.01456,(PmiPrist:5.4E-
4,((SycSpe17:0.00328,Ult30815:0.03183)0.902.138:0.01413,(PmiSpec5:0.02644

,UltCya70:0.02613)0.601.9:0.00757)0.884.138:0.01361)0.896.129:0.01267)0.7
68.96:0.00905,((Ult30806:0.0039,((LpgSpec4:5.5E-4,LpgSpec5:5.5E-
4)0.766.101:0.00346,UltLep22:0.0358)0.857.137:0.00774)0.784.110:0.00659,U
lt30805:0.00851)1.000.933:0.05128)0.783.124:0.01799)0.572.13:0.03305)0.87
7.148:0.0342)0.888.147:0.03462)0.918.145:0.02357)0.986.106:0.05678)0.782.
126:0.03191)0.773.120:0.0081,(Ult31126:0.09206,MaiMet38:0.13073)0.781.109
:0.03884)0.664.21:5.5E-
4,((Ult31134:0.07766,(Ult31132:0.01662,Ult31133:0.01801)0.977.97:0.06435)
0.960.116:0.06327,((Ult31135:0.04356,UltGra10:0.00499)0.996.132:0.14437,(
UltCy244:0.05255,Ult31136:0.03683)0.884.139:0.06135)0.957.120:0.06673)0.9
07.147:0.0358)0.998.178:0.08347,(((((Ult30165:0.13442,(Ult30163:0.09711,U
lt30164:0.08475)0.935.125:0.06412)0.997.137:0.14075,((UltCa331:0.12129,Ul
tB2810:0.25051)0.889.139:0.0496,(((Ult30130:0.16788,Ult30131:0.16598)0.39
1.15:0.00958,(Ult30132:0.16777,UltEps50:0.07375)0.994.130:0.13566)0.930.1
29:0.04877,((Ult30138:0.10021,(Ult30129:0.16468,(Ult30167:0.12788,Ult3016
6:0.083)0.995.137:0.13163)0.861.156:0.05196)0.904.145:0.05945,(((Ult30135
:0.12674,Ult30136:0.06749)0.964.126:0.05878,(Ult30137:0.11713,(UltEps49:0
.15561,UltrS408:0.15197)0.726.38:0.01985)0.424.24:0.01406)0.121.10:0.0168
5,((Ult30133:0.13089,(UltBani7:0.14072,UltEu130:0.09458)0.923.183:0.05385
)0.858.131:0.03216,Ult30134:0.11848)0.842.123:0.03529)0.755.93:0.02537)0.
945.104:0.05684)0.834.96:0.01702)0.605.18:0.00921)0.878.128:0.03599,(((((
Ult29770:5.4E-
4,Ult29771:0.01853)0.977.98:0.07597,(Ult29772:0.06279,Ult29773:0.08065)0.
423.20:0.02793)0.840.123:0.0389,(Ult29769:0.11701,((Ult29766:0.03709,(Ult
Ca240:5.4E-
4,(UltCa238:0.00356,UltCa239:0.00761)0.997.138:0.04789)1.000.934:0.10173)
0.923.184:0.03811,(Ult29767:0.07473,(Ult29768:0.23563,(((Ult29764:0.02758
,((Ult29762:0.00416,Ult29763:0.00706)0.273.14:0.01202,(UltCa236:0.03146,U
ltCa235:0.01694)0.521.8:0.01678)0.759.112:0.00831)0.980.95:0.04069,(Ult29
765:0.06599,UltCa237:0.05438)0.302.13:0.02257)0.842.124:0.02021,((UltCa24
8:0.07254,(UltCa241:0.01739,(UltCa243:0.04363,UltCa242:0.0154)0.351.15:0.
00646)0.991.116:0.06107)0.878.129:0.02283,((((UltCa229:0.01538,UltCa233:0
.04001)0.840.124:0.00771,((UltCa231:0.02249,UltCa232:0.00805)0.771.128:0.
00388,(UltCa230:0.00808,Ult29761:0.01853)0.774.118:0.00378)0.738.56:0.003
27)0.974.78:0.02758,(UltCa234:0.04002,((UltRu552:0.00463,((Ult29759:0.017
18,(Ult29758:0.00519,UltRu553:0.04683)0.887.141:0.02554)0.986.107:0.05914
,(((Otu00408:0.01507,(UltCa224:0.01518,Otu00609:0.01513)0.854.125:5.4E-
4)0.952.123:0.01506,UltCa223:0.01508)0.653.16:5.5E-
4,Ult29757:0.00738)0.914.154:0.02381)0.531.11:0.0297)0.983.94:0.0394,(Ult
Ca246:0.0232,UltCa247:0.01519)0.970.119:0.02723)0.848.113:0.00861)0.798.9
7:0.00672)0.753.99:0.00688,(UltCa244:0.03547,((UdnBac76:0.03995,(Ult29760
:0.06772,(UltCa228:0.06329,UltCa227:0.01551)0.588.20:0.01869)0.850.146:0.
01653)0.912.168:0.01919,(UltCa245:0.01597,(UltCa225:0.02258,UltCa226:5.4E
-
4)0.999.195:0.05521)0.683.33:0.00869)0.954.104:0.02552)0.426.25:0.01016)0
.893.146:0.02271)0.841.110:0.0153)0.842.125:0.0246)0.053.6:0.01986)0.739.
59:0.03728)0.957.121:0.08037)0.941.113:0.0656)0.997.139:0.11551,((((Ult30
007:0.1415,(Ult30006:0.00591,Ult30005:0.01288)0.999.196:0.18237)0.885.134
:0.06279,(Ult29654:0.20211,(Ult30008:0.08146,UltCa311:0.16394)0.838.116:0
.04739)0.798.98:0.01896)0.952.124:0.05939,(((Ult29893:0.16264,(UltCa265:0
.01683,UltCa266:0.0226)0.978.90:0.09398)0.963.105:0.08591,(UltPro91:0.064
37,Ult30140:0.20942)0.988.121:0.13376)0.761.97:0.03962,((UltCa214:0.25261
,Ult30141:0.06833)0.979.114:0.13175,((((Ult29889:0.07135,(UltCyan3:0.3005
2,((((((Ult29793:0.05889,UltEps42:0.04744)0.823.96:0.01555,(Ult29787:0.06

129,(((((Ult29790:0.01748,Ult29791:0.02477)0.504.14:0.01309,((Ult29806:0.
04989,MaiMet30:0.01617)0.833.110:0.0148,((UltCa251:0.05379,Ult29805:0.015
29)0.754.90:0.00582,((UltCa249:0.00536,((Ult29804:0.01813,Ult29807:0.0554
1)0.891.112:0.01541,(Ult29797:0.00748,UltEps48:0.01912)0.993.112:0.04239)
0.372.19:0.00407)0.861.157:0.00801,UltCa250:0.02504)0.846.137:0.01136)0.4
89.17:0.01176)0.873.132:0.01436)0.872.138:0.01537,(Ult29794:0.02555,Ult29
792:0.07326)0.349.13:0.01769)0.854.126:0.01838,((Ult29795:0.09181,Ult2979
8:0.03413)0.744.77:0.00498,(UltEps43:0.0074,(UltEps44:0.0,Ult29803:0.0):5
.5E-
4)0.999.197:0.05979)0.725.45:0.00382)0.942.128:0.01979,((((UltEps45:0.100
71,((((((Ult29780:0.005,((Ult29777:5.5E-
4,Ult29783:0.01123)0.866.142:0.01022,((UltEps39:5.5E-4,UltEps40:5.5E-
4)0.840.125:5.5E-4,(Ult29776:5.5E-4,(Ult29775:5.5E-
4,Ult29774:0.00369)0.858.132:0.0037)0.884.140:0.0037)0.868.117:0.00873)0.
738.57:0.01166)0.890.142:0.01083,(Ult29778:0.02443,Ult29796:0.06158)0.418
.16:0.00313)0.964.127:0.01772,Ult29781:0.0241)0.592.15:0.0043,Ult29782:0.
03432)0.793.99:0.0099,(((UdnProt2:0.02622,Ult29788:0.06475)0.826.85:0.015
56,(((Ult29784:0.03305,UltProk5:0.02812)0.983.95:0.04183,(Ult29785:0.0696
4,Ult29786:0.02712)0.660.20:0.02962)0.763.127:0.00709,Ult29789:0.02831)0.
579.18:0.01271)0.724.42:0.00474,Ult29779:0.03236)0.932.138:0.01576)0.850.
147:5.4E-
4,(UltEps41:0.01186,UltEps47:0.03238)0.891.113:0.01999)0.941.114:0.01812)
0.685.21:0.01002,((Ult29799:0.03491,Ult29800:0.05258)0.651.22:0.01446,((U
lt29802:0.0754,UltEps46:0.03334)0.810.84:0.008,Ult29801:0.01376)0.756.106
:0.0056)0.967.98:0.02129)0.575.11:0.0011,(Ult29808:0.00374,Ult29809:0.003
66)0.992.120:0.03611)0.931.141:0.02085,UltPro88:0.04208)0.811.76:0.01822)
0.542.14:0.01837)0.320.8:0.01367)0.983.96:0.06874,UltPro89:0.06036)0.923.
185:0.04843,Ult29813:0.08599)0.829.113:0.04057,UltCa252:0.19931)0.150.12:
0.03543,(Ult29810:0.14195,(Ult29811:0.04853,Ult29812:0.06441)0.920.160:0.
05064)0.565.10:0.03771)0.995.138:0.17291)0.683.34:0.05483)0.783.125:0.013
89,((((Ult29814:0.12637,(((Ult29827:0.16049,(UltMa115:0.04426,UltMa114:0.
00828)0.994.131:0.07917)0.699.33:0.0066,Ult29815:0.09402)0.845.125:0.0161
6,((Ult29899:0.04603,(Ult29900:0.03595,(Ult29901:0.07029,(((Ult29897:0.10
003,(MaiMet31:0.05157,Ult29894:0.06937)0.798.99:0.02112)0.411.20:0.02773,
Ult29896:0.00612)0.982.92:0.05492,Ult29895:0.03869)0.941.115:0.0485)0.904
.146:0.03526)0.676.16:0.03125)0.402.16:0.04084,Ult29898:0.04794)0.985.109
:0.06549)0.806.77:0.01362)0.941.116:0.03436,(Ult29891:0.06698,Ult29890:0.
10552)0.931.142:0.03914)0.036.6:5.4E-
4,(((Ult29838:0.05593,(Ult29836:0.00736,Ult29837:5.5E-
4)0.974.79:0.05835)0.994.132:0.0873,(Ult29830:0.06039,((Ult29835:0.05912,
(Ult29833:0.02267,(Ult29831:0.0116,Ult29832:0.00328)0.976.97:0.03324)0.00
2.7:0.01528)0.775.108:0.01559,((Ult29829:0.05732,Ult29834:0.03449)0.750.7
8:0.0077,((UltOr404:0.03302,Ult29828:0.04862)0.936.128:0.03724,Ult29839:0
.03697)0.738.58:0.00892)0.378.18:0.01292)0.795.103:0.01602)0.720.38:0.006
21)0.905.139:0.05114,(Ult29842:0.06878,(UltCa256:0.02359,((UltOr407:0.048
18,Ult29841:0.0406)0.879.153:0.02359,((UltOr405:0.0,UltOr406:0.0):0.03172
,Ult29840:0.02393)0.961.116:0.0367)0.000.952:0.00665)0.904.147:0.05152)0.
000.953:0.02499)0.888.148:0.03612)0.521.9:0.04465,((Ult29892:0.15872,(((U
lt29880:0.01842,Ult29881:0.01712)0.981.107:0.06226,(Ult29882:5.4E-
4,(Ult29879:0.02411,(Ult29883:0.07121,Ult29878:0.00813)0.808.92:0.00701)0
.976.98:0.03149)0.311.10:0.01482)0.933.150:0.03765,((((UltCa261:0.02193,(
(Ult29871:0.01014,((Ult29870:0.00411,(UltOr412:0.01141,(Ult30412:0.03549,
(UltCa263:0.07704,UltCa262:0.05008)0.886.166:0.02521)0.822.82:0.01164)0.9
18.146:0.01577)0.885.135:0.00862,UltMa116:0.02197)0.756.107:0.00496)0.718

.38:0.01116,Ult29872:0.00909)0.765.100:0.0084)0.796.77:0.00823,(Ult29873:
0.0824,Ult29876:0.0093)0.000.954:0.01114)0.474.18:5.3E-
4,(Ult29875:0.09331,Ult29874:0.01343)0.902.139:0.01975)0.837.94:0.0157,Ul
t29877:0.02572)0.900.118:0.02842)0.963.106:0.07645)0.770.125:0.03347,(((U
lt29843:0.01954,(UltOr409:0.02588,(UltOr410:0.10303,UltOr408:0.04922)0.88
1.147:0.01704)0.976.99:0.03164)0.740.77:0.00398,(Ult29844:0.04331,Ult2984
5:0.00857)0.882.154:0.01412)0.365.15:0.00794,(Ult29826:0.10547,(Ult29824:
0.04961,Ult29825:0.0239)0.942.129:0.06087)0.997.140:0.10457)0.961.117:0.0
5157)0.833.111:0.01876)0.940.127:0.04193)0.966.136:0.04275,(UltCa267:0.19
234,((Ult29868:0.01456,Ult29869:5.6E-
4)1.000.935:0.14601,(UltCa254:0.13092,UltCa255:0.15013)0.275.14:0.03576)0
.912.169:0.05609)0.802.89:0.03089)0.928.120:0.03302,((Ult29817:0.10299,((
(UltB4120:0.02315,Ult29816:5.5E-
4)0.944.113:0.02828,(UltCa253:0.05355,(Ult29818:0.11368,(UltPro90:0.01759
,UltProk6:0.07333)0.887.142:0.03525)0.265.6:0.00567)0.874.156:0.02022)0.9
51.121:0.051,(UltOr403:0.05753,(Ult29819:0.05322,Ult29820:0.07429)0.705.2
5:0.01501)0.926.158:0.0404)0.916.153:0.04984)0.997.141:0.09865,(((Ult2982
3:0.07517,((Ult29856:0.05077,(Ult29853:0.00829,(Ult29855:0.0597,Ult29854:
0.03466)0.911.167:0.03063)0.961.118:0.05825)0.984.102:0.08342,((((UltCa25
9:0.09446,(((Ult29867:0.07433,(UltCa260:0.02237,Ult29866:0.044)1.000.936:
0.10844)0.623.14:0.00502,Ult29864:0.14482)0.919.171:0.04199,Ult29865:0.03
091)0.295.22:0.03818)0.714.34:0.03102,(UltCa258:0.04254,((Ult29859:0.0436
7,Ult29861:0.12138)0.933.151:0.05431,((UltCa257:5.3E-
4,(Ult29857:0.04467,Ult29858:0.07496)0.677.22:0.02692)0.940.128:0.04631,U
lt29860:0.0493)0.242.12:0.0178)0.965.108:0.05955)0.490.8:0.03415)0.758.10
4:0.02558,(((Ult29851:0.04098,Ult29852:0.00833)0.995.139:0.07009,((Ult298
48:0.03673,Ult29849:0.0374)0.210.19:0.00989,(Ult29863:0.08013,(Ult29862:0
.03101,UltrS398:0.02188)0.983.97:0.06351)0.917.127:0.04031)0.855.136:0.01
466)0.668.26:0.01165,Ult29850:0.05598)0.924.135:5.4E-
4)0.942.130:0.0327,(UltOr411:0.06158,(Ult29846:0.06357,Ult29847:0.01696)0
.952.125:0.03109)0.749.97:0.01121)0.959.124:0.0771)0.992.121:0.09643)0.98
7.139:0.0872,(Ult30139:0.17149,((Ult29886:0.03065,(Ult29885:0.01706,(Ult2
9884:0.04704,Ult29887:0.02528)0.747.81:0.00566)0.740.78:0.00431)0.786.103
:0.01489,(Ult29888:0.06506,UltCa264:0.05545)0.827.96:0.03186)0.976.100:0.
06026)0.912.170:0.039)0.702.24:0.01294,(Ult29821:0.09328,Ult29822:0.16095
)0.885.136:0.05427)0.951.122:0.0434)0.707.24:0.00478)0.734.70:0.00961)0.7
45.98:0.01096)0.757.82:0.00941)0.840.126:0.01306,(((((UltDe232:0.08335,(U
ltOr402:0.03939,UltCa222:0.00471)0.985.110:0.08384)0.998.179:0.12517,(((U
lt29753:0.01847,Ult29755:5.4E-
4)0.943.127:0.02996,(Ult29756:0.02633,Ult29754:0.05587)0.580.10:0.00424)0
.981.108:0.08171,((Ult29744:0.0388,((Ult29743:0.03984,Ult29746:0.03052)0.
961.119:0.02404,(((Ult29749:0.0166,Ult29747:0.06509)0.895.144:0.02189,(Ul
t29750:0.00792,(Ult29748:0.01462,Ult29751:5.4E-
4)0.849.119:0.00672)0.770.126:0.00777)0.746.75:0.00641,Ult29752:0.03376)0
.944.114:0.0218)0.760.110:5.4E-
4)0.107.15:0.00616,Ult29745:0.00501)0.891.114:0.03921)0.918.147:0.05405)0
.992.122:0.16995,(((Ult30128:0.23419,(Ult30126:0.12404,Ult30127:0.05253)0
.992.123:0.13496)0.913.149:0.05878,((UltOr419:0.17336,((((Ult30093:0.0555
7,(Ult30103:0.16282,Ult30084:0.10928)0.385.19:0.02695)0.715.30:0.00627,((
UltCa321:0.07187,Ult30072:0.07524)0.756.108:0.01175,(((Ult30087:0.06023,U
lt30089:0.20383)0.182.11:0.02047,((((Ult30094:0.0448,Ult30095:0.05898)0.9
80.96:0.0599,((Ult30102:0.11208,(Ult30099:0.09312,Ult30117:0.08499)0.888.
149:0.05058)0.886.167:0.04065,(Ult30101:0.19268,Ult30100:0.19526)0.899.13
6:0.05867)0.888.150:0.03848)0.680.24:0.01089,UltOr418:0.16003)0.212.10:0.

01473,((UltCa323:0.0,UltCa322:0.0):0.00364,(Ult30098:0.00736,Ult30097:0.0
0366)1.000.937:5.5E-
4)1.000.938:0.12879)0.952.126:0.04938)0.802.90:0.01033,(Ult30088:0.04288,
(Ult30071:0.10668,(Ult30085:0.00384,Ult30086:0.01217)0.804.80:0.01095)0.9
33.152:0.02241)0.847.150:0.01592)0.780.131:0.00824)0.904.148:0.01952)0.77
8.108:0.01428,((Ult30079:0.09178,((UltCa320:0.05072,Ult30096:0.20411)0.89
6.130:0.04296,(Ult30078:0.15154,(Ult30076:0.07283,Ult30077:0.05505)0.942.
131:0.06421)0.866.143:5.5E-
4)0.918.148:0.02605)0.852.132:0.01554,((Ult30074:0.07181,(Ult30080:0.0601
5,(Ult30081:0.05875,(Ult30082:0.01023,Ult30083:0.00468)0.934.117:0.03722)
0.919.172:0.04422)0.989.102:0.06508)0.817.72:0.01955,(Ult30075:0.03972,Ul
t30073:0.01887)0.738.59:0.00437)0.185.10:0.00423)0.650.19:0.00624)0.959.1
25:0.04963,((UltrS407:0.06848,(Ult30090:0.02499,Ult30091:0.03432)0.930.13
0:0.04357)0.999.198:0.1126,Ult30092:0.0633)0.845.126:0.02112)0.738.60:0.0
343)0.957.122:0.07665,(((Ult30067:0.1402,((((((Ult30033:0.1816,(UltOr416:
0.1208,((((Ult30011:0.01487,Ult30012:5.5E-
4)0.995.140:0.04097,(UdnBac80:0.00488,(Ult30010:0.06468,UltOr417:0.02163)
0.956.120:0.02791)0.733.54:0.00505)0.946.120:0.02177,MaiMet34:0.07076)0.7
92.112:0.01466,(Ult30009:0.0796,Ult30013:0.04241)0.739.60:0.00451)0.899.1
37:0.02789)0.950.120:0.05387)0.952.127:0.04267,Ult30043:0.03896)0.876.121
:0.02386,(Ult30039:0.17095,Ult30053:0.13775)0.853.107:0.03779)0.937.128:0
.03922,(((Ult30034:0.09088,((((((Ult30055:0.04655,(UltCa313:0.0654,Ult300
52:0.07164)0.915.137:0.03599)0.906.155:0.02003,(Ult30054:0.05711,UltrS405
:0.03007)0.771.129:0.00719)0.425.16:0.01072,(Ult30057:0.02606,(UltCa314:0
.05587,Ult30058:0.03955)0.955.104:0.03778)0.516.10:0.0181)0.793.100:0.030
63,(Ult28321:0.03426,(Ult30017:5.3E-
4,Ult30018:0.02244)1.000.939:0.1099)0.741.70:0.0196)0.945.105:0.04446,Ult
Ca315:0.03065)0.903.126:0.02269,Ult30051:0.06646)0.860.109:0.01975)0.438.
19:0.00582,(Ult30046:0.06067,Ult30045:0.0461)0.887.143:0.03081)0.428.19:0
.00903,((((Ult30042:0.07912,Ult30066:0.1579)0.870.124:0.036,(((Ult30059:0
.01944,UltCa316:0.05399)0.882.155:0.04036,(UltCa317:0.03833,UltCa318:0.09
43)0.836.96:0.01914)0.962.127:0.04428,(((Ult30040:0.09158,(Ult30037:0.047
43,MaiMet35:0.17952)0.889.140:0.04243)0.831.97:0.04856,(((((Ult30016:0.07
757,Ult30015:0.04211)0.931.143:0.03869,((Ult30031:0.0358,(Ult30032:0.0663
2,(Ult30030:0.02101,UltrS401:0.00988)0.942.132:0.03827)0.963.107:0.04962)
0.274.9:0.01684,(Ult30029:0.09199,(((((Ult30022:0.01509,((Ult30021:0.0141
4,Ult30020:0.04234)0.960.117:0.04517,Ult30023:0.04179)0.943.128:0.02989)0
.860.110:0.02365,Ult30026:0.06323)0.766.102:0.03896,Ult30027:0.07074)0.84
1.111:0.01945,Ult30028:0.04995)0.768.97:0.01509,(Ult30024:0.00368,Ult3002
5:5.5E-
4)0.997.142:0.07272)0.796.78:0.01351)0.951.123:0.04384)0.883.146:0.03485)
0.472.17:0.02752,(Ult30041:0.00727,UltrS403:5.5E-
4)0.993.113:0.08086)0.482.19:0.0196,((UltrS402:0.08132,(UltCa312:0.13428,
Ult30014:0.05977)0.199.8:0.05034)0.042.9:0.04443,Ult30019:0.11784)0.902.1
40:0.04735)0.846.138:0.02327,Ult30038:0.04539)0.883.147:0.02401)0.913.150
:0.04341,(Ult30035:0.09006,Ult30036:0.10846)0.979.115:0.07886)0.547.11:0.
00188)0.694.25:0.01768)0.771.130:0.01032,(Ult30044:0.04814,(((Ult30061:0.
09812,Ult30060:0.05471)0.936.129:0.03839,(Ult30049:0.00736,(Ult30047:0.0,
Ult30048:0.0,Ult30050:0.0):5.5E-
4)1.000.940:0.10835)0.715.31:0.00824,(Ult30062:0.10023,(UltrS406:0.04222,
UltrS404:0.12127)0.701.28:0.02577)0.914.155:0.04337)0.812.81:0.01655)0.87
8.130:0.01726)0.230.8:0.0045,Ult30056:0.07938)0.943.129:0.03025)0.844.131
:0.01324)0.747.82:0.01212,(Ult30064:0.07812,(Ult30113:0.13085,(UltCa324:0
.09876,(Ult30111:0.1151,Ult30112:0.10823)0.648.22:0.00952)0.914.156:0.048

07)0.913.151:0.05858)0.906.156:0.04698)0.927.128:0.06512,(Ult30065:0.1197
4,(Ult30063:0.10413,(Ult30069:0.10313,UltCa319:0.07457)0.823.97:0.03759)0
.785.115:0.031)0.937.129:0.03587)0.792.113:0.01811)0.906.157:0.04194,Ult3
0070:0.18191)0.022.4:0.01799,((Ult30068:0.08293,Ult30118:0.16807)0.752.91
:0.02973,(UltCa206:0.20972,((Ult30121:0.04418,(Ult30119:0.0194,Ult30120:0
.00355)0.927.129:0.05301)0.472.18:0.02249,(Ult30124:0.16559,(Ult30122:0.0
2178,Ult30123:0.02589)0.959.126:0.09984)0.966.137:0.10556)0.998.180:0.213
44)0.899.138:0.08672)0.834.97:0.01994)0.896.131:0.03535)0.293.11:0.00893)
0.922.183:0.04963,(Ult30114:0.20247,((UltAna39:0.06578,(((UltCa310:0.0238
4,(UltCa308:0.0397,(Ult30002:0.06075,(UltCa307:0.00371,UltCa309:5.5E-
4)0.755.94:5.5E-
4)0.737.77:0.00343)0.905.140:0.01622)0.872.139:0.01588,UltOr415:0.04106)0
.856.116:0.022,Ult30003:0.00807)0.941.117:0.05834)1.000.941:0.21334,((((U
ltCa305:0.05491,UltCa306:0.04014)0.952.128:0.05718,((UltCa328:0.00734,Ult
Ca329:0.01896)0.980.97:0.07289,(Ult29999:0.05539,((Ult29995:5.4E-
4,(UltCa303:0.01173,Ult29994:0.01928)0.952.129:0.01524)0.979.116:0.06255,
(((UltAna38:0.1231,UltrS400:0.09478)0.905.141:0.03899,(UltCa304:0.03202,U
lt29996:0.03956)0.421.16:0.01411)0.787.105:0.01651,(Ult29997:0.00367,Ult2
9998:5.5E-
4)0.983.98:0.04611)0.930.131:0.03606)0.994.133:0.07997)0.864.131:0.04547)
0.931.144:0.06146)0.962.128:0.08154,(Ult30000:5.4E-
4,Ult30001:0.03097)0.994.134:0.08563)0.276.12:0.02244,Ult30004:0.17911)0.
864.132:0.04185)0.316.9:0.01215)0.919.173:0.04715)0.264.8:0.02238)0.697.2
0:0.01037,(Ult30116:0.17919,(Ult30115:0.04038,UltOr420:0.11974)0.507.13:0
.03442)1.000.942:0.16582)0.812.82:0.01689,(((((Ult29991:0.18031,(((UdnBac
78:0.12654,(Ult29965:0.14612,Ult29968:0.11894)0.799.102:0.03651)0.953.101
:0.06811,((Ult29990:0.08153,(Ult29988:0.02431,Ult29989:0.03344)0.868.118:
0.03285)0.993.114:0.09772,((Ult29954:0.14307,UltPl566:0.31396)0.808.93:0.
05725,(Ult29955:0.06956,Ult29956:0.13524)0.955.105:0.06573)0.501.14:0.028
22)0.668.27:0.02874)0.934.118:0.04485,((Ult29964:0.09976,(UdnBac77:0.0504
1,(Ult29962:0.01167,Ult29963:0.05819)0.900.119:0.02537)0.996.133:0.11091)
0.939.122:0.04667,((Ult29977:0.05045,((UdnBac79:0.0217,(Ult29980:0.05609,
(Ult29978:0.07059,(Ult29975:0.06951,Ult29976:0.00506)0.588.21:0.01736)0.3
55.19:0.01125)0.829.114:0.01337)0.848.114:0.01824,(Ult29974:0.04068,Ult29
979:0.02703)0.947.112:0.02783)0.435.16:0.0157)0.875.151:0.02314,((Ult2997
3:0.02912,(Ult29970:0.00742,(Ult29971:0.0,Ult29972:0.0):5.4E-
4)0.921.143:0.03726)0.807.73:0.03952,Ult29981:0.13814)0.456.17:0.02262)0.
959.127:0.04836)0.877.149:0.01983)0.648.23:0.00272)0.895.145:0.02211,((Ul
t29969:0.17585,(Ult29933:0.06806,Ult29934:0.05944)0.985.111:0.07741)0.816
.88:0.02283,(UltOr414:0.15173,(((Ult29961:0.09113,(Ult29992:0.00549,Ult29
993:0.01726)0.997.143:0.12126)0.840.127:0.03333,(UltOr413:0.08255,UltCand
4:0.15401)0.725.46:0.03545)0.838.117:0.03363,(Ult29948:0.11401,((Ult29950
:0.04122,(Ult29949:0.02714,UltCa299:0.03969)0.957.123:0.03597)0.736.60:0.
00632,((Ult29947:0.02705,((Ult29944:0.0,Ult29945:0.0):5.5E-
4,Ult29946:0.00743)0.968.106:0.03316)0.921.144:0.0245,((Ult29942:0.03602,
UltCa298:0.11549)0.430.18:0.01676,(((UltCa291:0.06187,(UltAna37:0.00871,(
((((Ult29936:0.02649,Ult29937:0.0222)0.868.119:0.00943,UltCa284:0.00376)0
.609.15:0.00367,UltCa292:0.00746)1.000.943:5.4E-
4,(Ult29935:0.015,(UltCa285:0.00742,(UltCa286:0.00369,UltCa290:0.01126)0.
780.132:0.0037)0.786.104:5.4E-
4)0.827.97:0.00357)0.739.61:0.00754,Ult29938:0.01901)0.716.33:0.00283)0.3
40.13:0.01154)0.663.24:0.01283,((UltCa294:0.06569,((((Ult29940:5.5E-
4,UltCa287:0.01504)0.955.106:0.01542,(UltCa289:0.01144,UltCa288:0.02298)0
.728.49:0.00344)0.963.108:0.03523,Ult29939:0.06618)0.332.10:0.01148,Ult29

951:0.11449)0.776.112:0.03185)0.922.184:5.5E-
4,(Ult29941:0.06973,UltCa293:0.04181)0.919.174:0.03739)0.873.133:0.01411)
0.828.81:0.01,(((Ult29943:0.06642,(UltCa296:0.00499,(UltCa297:0.04826,(Ul
tCa300:0.08817,MaiMet32:0.02955)0.841.112:0.02028)0.964.128:0.03869)0.795
.104:0.01812)0.239.10:0.01809,UltCa295:0.11184)0.918.149:0.04028,Ult29952
:0.05435)0.784.111:0.01617)0.927.130:0.02644)0.909.131:0.02517)0.902.141:
0.02886)0.843.111:0.02702)0.898.153:0.03652)0.956.121:0.05244)0.543.16:0.
01612)0.734.71:0.01349)0.907.148:0.01792,(((UltAna36:0.13918,((MaiMet33:0
.15606,(Ult29966:0.01887,Ult29967:0.06926)0.990.116:0.09778)0.711.34:0.04
095,(Ult29932:0.07789,(Ult29982:0.02981,Ult29983:0.03985)0.985.112:0.0756
5)0.906.158:0.06228)0.839.111:0.04028)0.342.15:0.02941,((((UltCa282:0.045
3,(UltCa280:0.06549,((UltCa277:0.00387,(UltCa279:5.4E-
4,UltCa281:0.01101)0.947.113:0.01505)0.976.101:0.0192,(UltCa278:0.0012,Ul
t29931:0.02682)0.935.126:0.00287)0.932.139:0.02321)0.955.107:0.03981)0.81
8.66:0.03544,((((Ult29953:0.03407,UltCa301:0.04005)1.000.944:0.21496,(Ult
29921:0.07738,Ult29922:0.06197)0.902.142:0.06999)0.429.17:0.05842,UltCa27
3:0.02465)0.908.160:0.05046,(Ult29917:0.10246,(Ult29916:0.02793,(Ult29915
:0.10786,((Ult29920:0.04276,(UltCa271:0.06936,(Ult29914:0.01111,(Ult29918
:0.04726,Ult29919:5.4E-
4)0.106.11:0.02808)0.783.126:0.03103)0.930.132:0.02951)0.887.144:0.03026,
UltCa272:0.0558)0.778.109:0.01082)0.778.110:0.0105)0.893.147:0.02299)0.08
3.8:0.02852)0.929.129:0.04661)0.312.15:0.00954,UltCa283:0.10522)0.855.137
:0.01582,(Ult29960:0.20032,((Ult29930:0.07217,((((UltPl565:0.0747,(UltCa2
75:0.05176,(UltCa274:0.08224,(Ult29923:5.3E-
4,Ult29924:0.01879)0.895.146:0.02038)0.810.85:0.01536)0.862.138:0.03025)0
.667.25:0.03321,(Ult29926:0.01241,Ult29927:0.0114)0.990.117:0.07955)0.784
.112:0.02487,(Ult29928:0.07721,(UltCa276:0.10531,Ult29925:0.01704)0.940.1
29:0.05022)0.390.23:0.03044)0.454.21:0.015,Ult29929:0.08425)0.929.130:0.0
3838)0.854.127:0.03478,(((UltCra12:0.20329,Ult29987:0.12596)0.406.16:0.06
666,((Ult29912:0.04485,(Ult29913:0.01522,UltCra11:0.00358)1.000.945:0.093
72)0.772.118:0.00942,(Ult29910:0.0783,Ult29911:0.05935)0.953.102:0.03691)
0.954.105:0.03477)0.000.955:5.4E-
4,(Ult29909:0.03458,((UltrS399:0.03574,(UltCa270:0.03588,UltEu129:0.03738
)0.876.122:0.01885)0.269.12:0.01008,(Ult29908:0.0786,(((Ult29903:0.0,Ult2
9902:0.0,Ult29904:0.0):0.01143,Ult29905:0.01116)0.974.80:0.03284,((UltCa2
68:5.5E-
4,UltCa269:0.01496)0.988.122:0.04264,(Ult29906:0.05716,Ult29907:0.0245)0.
334.14:0.00893)0.911.168:0.01766)0.952.130:0.03547)0.000.956:0.02474)0.82
7.98:0.0299)0.994.135:0.076)0.802.91:0.03078)0.818.67:0.02322)0.765.101:0
.01857)0.808.94:0.02713)0.765.102:0.01423,((UltB4193:0.03948,Ult29959:0.0
5392)0.937.130:0.05073,(Ult29958:0.01381,(UltCa302:0.03275,Ult29957:0.131
98)0.774.119:0.0433)0.984.103:0.07657)0.995.141:0.08435)0.382.17:0.00752)
0.940.130:0.05558,(Ult29985:0.0873,Ult29984:0.11997)0.999.199:0.1731)0.82
2.83:0.03837,UltOr421:0.2006)0.867.139:0.03063)0.969.101:0.03696)0.270.5:
0.00445)0.948.144:0.03008,((((Ult30291:0.08597,MldSoil2:0.12813)0.987.140
:0.08418,(((Ult30302:0.05493,((Ult30297:0.10664,(((Ult30299:0.1426,(((Ult
30281:0.06117,(UltrS428:0.03994,(Ult30279:0.03121,Ult30280:0.00813)0.835.
101:0.00766)0.901.140:0.02368)0.917.128:0.02489,((Ult30277:5.5E-
4,(Ult30276:0.01969,Ult30278:0.01122)0.972.116:0.0199)0.765.103:0.01043,U
lt30293:0.09713)0.843.112:0.02199)0.701.29:0.01074,((Ult29986:0.23889,Ult
30294:0.10091)0.961.120:0.08927,(Ult30282:0.03416,(Ult30284:0.07351,Ult30
283:0.0171)0.890.143:0.02434)0.556.15:0.02531)0.990.118:5.5E-
4)0.914.157:0.03491)0.428.20:0.0175,((((((UltrS417:0.12847,(Ult30237:0.03
43,(UltCa349:0.01668,Ult30238:0.05866)0.692.18:0.02304)0.945.106:0.03854)

0.911.169:0.0333,(((Ult30220:0.02817,(Ult30218:0.0548,Ult30219:0.0569)0.968.107:0.04679)0.946.121:0.03985,Ult30221:0.02535)0.689.24:0.03141,(UltCa347:0.05763,(Ult30269:0.062,(Ult30268:0.06888,(Ult30267:0.03651,UltCa353:0.06993)0.745.99:0.0329)0.918.150:0.05276)0.913.152:0.03991)0.877.150:0.03657)0.853.108:0.03566)0.691.20:0.033,(((Ult30243:0.06469,(((((Ult30260:0.02694,((UltrS415:0.02037,(((UltCa340:5.4E-
4,UltrS421:0.03486)0.880.153:0.00747,Ult30207:0.01895)0.757.83:0.00477,(Ult30213:0.00369,((Ult30226:0.01054,(Ult30223:0.00373,(((((Ult30182:0.0223,Ult30184:0.01396)0.936.130:0.01839,(Ult30185:5.4E-
4,Ult30225:0.03942)0.929.131:0.01532)0.844.132:0.00742,(Ult30187:0.01513,UltrS422:0.02296)0.862.139:0.00767)0.749.98:0.00355,Ult30183:0.00753)0.893.148:0.00754,Ult30224:0.00369)0.757.84:0.00366)0.986.108:0.02857)0.958.138:0.02029,UltrS418:0.01905)0.956.122:5.5E-
4)0.960.118:0.01787)0.497.8:0.00749)0.858.133:0.01386,((Ult30188:0.02837,Ult30190:0.019)0.869.114:0.01591,Ult30186:0.03521)0.274.10:0.00891)0.875.152:0.02019)0.862.140:0.01612,(((Ult30210:0.00555,Ult30211:0.0256)0.985.113:0.04028,(Ult30258:0.04527,Ult30247:0.04416)0.525.13:0.01059)0.876.123:0.01807,(((((Ult30233:0.03604,(Ult30231:5.5E-4,Ult30408:5.5E-
4)0.863.152:0.01111)0.910.136:0.01804,(Ult30232:0.03472,((((UltrS423:0.00312,Ult30234:0.02847)0.862.141:0.00772,(Ult30215:5.5E-
4,Ult30216:0.00367)0.936.131:0.01149)0.864.133:0.00745,(Ult30227:0.00739,Ult30228:5.4E-
4)0.923.186:0.01094)0.871.144:0.0078,((Ult30235:0.00369,Ult30236:0.01115)0.751.76:0.00416,(Ult30230:0.00299,Ult30229:0.0159)0.875.153:0.00799)0.847.151:0.00703)0.773.121:0.00363)0.755.95:0.00513)0.916.154:0.01912,(Ult30198:0.03096,Ult30199:0.06785)0.881.148:0.04111)0.883.148:0.01515,(UltCa352:0.0405,((Ult30175:0.01646,UltCa348:0.03411)0.482.20:0.00801,Ult30192:0.06429)0.846.139:0.01741)0.946.122:0.02509)0.791.97:0.00629,(Ult30212:0.02345,(UltrS419:0.03181,Ult30259:0.02731)0.927.131:0.01906)0.838.118:0.00733)0.851.137:0.00913)0.679.26:0.01104)0.872.140:0.00967,(Ult30174:0.00809,((UltCa338:0.04808,((Ult30180:0.03384,Ult30179:0.02304)0.958.139:0.03931,(UdnBac82:0.0198,Ult30181:0.03273)0.875.154:0.01992)0.607.13:0.01528)0.936.132:0.03711,(Ult30177:0.05603,Ult30253:0.07274)0.477.19:0.02866)0.757.85:0.01314)0.984.104:0.03449)0.849.120:5.5E-
4,((UltrS424:0.02893,(Ult30244:0.04931,(UltrS427:0.02678,Ult30274:0.06735)0.849.121:0.04108)0.890.144:0.02849)0.702.25:0.00284,((Ult30251:0.00266,(Ult30250:0.08278,(Ult30248:5.5E-
4,Ult30249:0.01874)0.789.97:0.02778)0.935.127:0.03456)0.991.117:0.03847,UltrS426:0.02608)0.854.128:0.01536)0.867.140:0.01027)0.904.149:0.00765,(((Ult30252:0.02917,(Ult30214:0.05181,(Ult30222:0.03222,UltEps51:0.02633)0.120.11:0.00907)0.765.104:0.01532)0.762.114:0.00713,(Ult30245:0.03867,(Ult30239:0.01859,UltrS425:0.05085)0.858.134:0.01214)0.907.149:0.0187)0.759.113:0.00409,(Ult30178:0.0431,(MldSoil3:0.06158,Ult30176:0.03904)0.499.16:0.02598)0.707.25:0.00736)0.871.145:0.01007)0.937.131:5.4E-
4)0.412.17:0.00855,((UltrS420:0.04049,(Ult30217:0.02743,UltCa346:0.0247)0.932.140:0.0334)0.878.131:0.02046,((Ult30189:0.05081,UltrS416:0.05463)0.920.161:0.02853,(Ult30240:0.0,Ult30241:0.0,Ult30242:0.0):0.05761)0.797.90:0.01015)0.866.144:0.0173)0.466.14:0.01507,((Ult30193:0.07103,((((Ult30201:0.00368,(UltCa345:5.5E-
4,(Ult30202:0.00739,UltCa344:0.01898)0.101.6:5.4E-
4)0.845.127:0.00368)0.984.105:5.5E-
4,(Ult30203:0.00367,Ult30209:0.00772)0.941.118:0.01113)0.977.99:0.03107,(((Ult30168:0.01077,Ult30410:0.04455)0.886.168:0.01214,((Ult30171:0.0076,Ult30173:0.02333)0.836.97:0.01,((((Ult30200:0.00767,Ult30197:0.01915)0.932

.141:0.01501,Ult30196:5.5E-
4)0.247.12:0.00741,UltCa343:0.00726)0.743.91:0.00357,(UltCa342:5.5E-
4,UltCa335:0.03053)0.920.162:0.02318)0.951.124:0.01683)0.911.170:0.01588)
0.938.96:0.01614,(Ult30172:0.01513,((Ult30206:0.0188,((((UltCa341:0.0,Ult
30194:0.0,Ult30195:0.0):5.4E-4,(Ult30170:0.01118,UltCa337:5.5E-
4)0.905.142:0.00741)0.974.81:0.01501,UltCa336:0.00368)0.714.35:5.5E-
4,(UltCa334:0.0,Ult30169:0.0):5.5E-
4)0.745.100:0.00377)0.966.138:0.0231,((UltrS413:0.00715,UltrS414:0.01534)
0.773.122:0.01133,(Ult30205:5.4E-
4,Ult30204:0.02268)0.915.138:0.01837)0.964.129:5.4E-
4)0.516.11:0.00765)0.966.139:0.02269)0.762.115:0.00439)0.932.142:0.02093,
Ult30246:0.03899)0.788.103:0.01657)0.784.113:0.013,(Ult30208:0.04627,(Ult
30191:0.01493,UltCa339:5.3E-
4)0.923.187:0.023)0.958.140:0.03184)0.849.122:0.01469)0.124.16:0.01394)0.
147.11:0.0156,UltCa354:0.1002)0.853.109:0.02312,(Ult30275:0.06446,((Ult30
300:0.02886,Ult30301:0.01874)1.000.946:0.11242,(Ult30257:0.04784,((Ult302
54:0.01516,Ult30255:0.02542)0.957.124:0.0288,(UltCa350:0.03408,Ult30256:0
.03733)0.201.14:0.00434)0.875.155:0.01909)0.993.115:0.04613)0.738.61:5.3E
-
4)0.905.143:0.03515)0.879.154:0.02438,((Ult30273:0.03838,(Ult30270:0.0250
2,(Ult30271:0.03195,Ult30272:0.04105)0.913.153:0.02247)0.977.100:0.04653)
0.924.136:0.03142,((Ult30266:5.5E-
4,Ult30409:0.00366)0.976.102:0.0447,((Ult30264:0.01691,((Ult30262:0.0112,
(UltCa351:5.4E-4,Ult30261:0.04812)0.984.106:0.01882)0.555.10:5.5E-
4,Ult30263:0.03064)0.882.156:0.01407)0.978.91:0.03291,Ult30265:0.01685)0.
926.159:0.03383)0.994.136:0.05873)0.711.35:0.00579)0.854.129:0.01782)0.85
6.117:0.02186,(Ult30296:0.02058,Ult30295:0.05671)1.000.947:0.0962)0.627.1
0:0.02604)0.637.12:0.01148,Ult30298:0.06023)0.929.132:0.03888)0.434.16:5.
5E-
4,((Sgffff15:0.02012,(HmZZZZ06:0.04384,HmZZZZ05:0.0515)0.994.137:0.06974)
0.934.119:0.0454,((Ult30305:0.04278,UltGa304:0.0397)0.913.154:0.02735,(((
((UltCa355:0.04381,((UltCa359:0.0,UltCa360:0.0):0.03682,(Ult30303:0.02116
,(UltCa356:5.4E-
4,Sgfffff5:0.00745)0.685.22:0.00188)0.721.27:0.01098)0.933.153:0.02579)0.
777.120:0.01129,(UltCa358:0.02911,(((UncUn221:0.01833,((UncUn220:0.00852,
(Ult30315:0.01495,Ult30314:0.00398)0.871.146:0.01485)0.923.188:0.02147,((
Ult30311:5.5E-
4,Ult30313:0.00739)0.989.103:0.03582,((Ult30307:0.0,Ult30308:0.0,Ult30309
:0.0):0.01842,Ult30310:0.00814)0.748.85:0.01124)0.880.154:0.01732)0.851.1
38:0.02529)0.893.149:0.04686,UncUn222:0.04906)0.185.11:0.03219,UltCa357:0
.00589)0.980.98:0.05909)0.980.99:0.0549)0.920.163:0.02704,(Ult30306:0.028
64,(UltCa361:0.02752,Ult30304:0.01115)0.952.131:0.02122)0.624.15:0.01095)
0.747.83:0.01595,((((((Ult30316:0.0214,(Ult30344:0.01924,(Ult30401:0.003
93,((Ult30403:0.00743,Ult30404:0.01498)0.928.121:0.01312,(UltCa378:0.0191
9,(Ult30402:0.01444,(Ult30405:0.01143,Ult30406:0.01126)0.875.156:0.01792)
0.954.106:0.02932)0.163.12:0.00538)0.893.150:0.01319)0.669.22:0.00338)0.8
84.141:0.02069)0.777.121:5.4E-
4,((Ult30400:0.03528,Ult30359:0.03692)0.870.125:0.02352,(((((UltCa367:0.0
2118,((Ult30357:0.01308,((UncUn223:0.01209,(Bfhggg86:0.00762,S0114502:5.5
E-
4)0.738.62:0.00279)0.000.957:0.00477,(UltCa369:0.03016,(((UltRu555:0.0238
7,(Ult30349:0.04588,(UltRu554:0.00431,(Ult30347:0.01639,Ult30348:0.02456)
0.612.19:0.02054)0.942.133:0.02947)0.458.31:0.01162)0.886.169:0.01821,((U
lt30355:5.5E-4,((Ult30353:5.5E-4,Ult30354:0.03363)1.000.948:5.3E-

4,(Ult30356:5.5E-
4,(Ult30351:0.0068,Ult30352:0.002)0.897.138:0.00199)0.459.18:0.00361)0.87
0.126:0.00352)0.793.101:5.5E-
4,Ult30350:0.01104)0.848.115:0.00741)0.807.74:0.01059,TmPhylu3:0.0199)0.9
34.120:0.02641)0.540.18:0.01494)0.937.132:0.02668)0.943.130:0.041,(Ult303
58:5.5E-4,Ult30411:0.01098)0.964.130:5.4E-
4)0.947.114:0.03376)0.782.127:0.02359,(UltCa365:0.04305,UltCa368:0.13294)
0.000.958:0.00266)0.744.78:0.03475,UltCa366:0.06079)0.840.128:0.02133,Ult
Ca377:0.11363)0.849.123:0.01539,(UltCa376:0.01536,(TmPhylu4:0.02924,(Ult3
0398:0.01518,Ult30399:0.01093)0.961.121:0.02267)0.802.92:0.00889)0.069.7:
0.00663)0.329.13:0.01271)0.828.82:0.00736)0.878.132:0.01097,Pshggg65:0.02
057)0.947.115:0.02086,(Ult30318:0.03272,Ult30362:0.02794)0.511.13:0.014)0
.975.102:5.5E-
4,(Ult30371:0.0207,((Ult30346:0.00976,(Ult30360:0.03887,Ult30361:0.00365)
0.451.24:0.00993)0.562.19:0.01857,((Ult30379:0.02291,(UltrS434:0.00362,(U
lt30394:0.00748,(Ult30378:0.01124,Ult30380:0.02676)0.854.130:0.00715)0.89
8.154:0.01132)0.917.129:0.01122)0.780.133:0.00544,(((UltrS433:0.00363,(((
Ult30343:0.00732,Ult30390:0.00365)0.883.149:0.00732,((Ult30388:0.01841,(U
ltCa374:0.0434,Ult30414:0.01335)0.980.100:0.03935)0.143.14:0.00802,Ult303
92:0.01855)0.959.128:5.4E-
4)0.788.104:0.00736,Ult30385:0.01479)0.964.131:5.4E-4)1.000.949:5.3E-
4,(((((UltrS432:5.5E-
4,(UltCa370:0.01474,Ult30387:0.00368)0.990.119:0.02247)0.712.27:0.01475,(
Ult30386:0.0186,Ult30391:5.4E-4)0.941.119:5.4E-
4)0.954.107:0.01103,(UltMyc24:0.0073,Ult30389:5.5E-
4)0.826.86:0.00362)0.033.5:5.4E-
4,UltrS431:0.02648)0.437.20:0.00362,Ult30393:5.4E-
4)0.844.133:0.00353)0.765.105:0.01108,((((TmPhylu2:0.0153,UltCa364:0.0191
1)0.900.120:0.01085,((((Ult30334:0.0,Ult30416:0.0):5.5E-
4,(Ult30415:0.01851,Ult30336:0.00362)0.940.131:5.5E-4)0.896.132:5.4E-
4,(Ult30331:0.01876,((Ult30332:5.5E-
4,Ult30333:0.01504)0.889.141:0.00722,Ult30335:0.00746)0.878.133:0.00723)0
.237.13:5.4E-4)0.637.13:5.3E-4,(((Ult30372:0.0,Ult30375:0.0):5.3E-
4,((Ult30376:0.02259,((Ult30384:0.02699,Ult30383:0.00741)0.969.102:0.0231
7,(UltCa375:0.00727,(((UltrS435:0.0186,(Ult30396:0.00364,(Ult30395:0.0592
4,UltCa372:0.01109)0.328.17:5.5E-4)0.915.139:0.00738)0.839.112:5.5E-
4,(Ult30397:0.00366,(UltCa373:0.0073,UltrS436:5.5E-
4)0.881.149:0.00731)0.895.147:0.00711)0.773.123:0.00369,((Ult30363:0.0036
8,(Ult30377:0.00731,(Ult30367:0.01107,UltCa371:0.0036)0.869.115:0.00735)0
.933.154:0.01106)0.751.77:0.00363,((Ult30364:0.00361,(((UltGa305:0.0,Ult3
0365:0.0,Ult30366:0.0):0.00379,(Ult30368:5.5E-
4,Ult30382:0.01465)0.807.75:0.00731)0.771.131:0.00348,(Ult30369:0.0,Ult30
370:0.0):5.5E-4)0.878.134:0.00745)0.971.96:0.01887,Ult30381:5.4E-
4)0.899.139:0.00725)0.922.185:0.01116)0.931.145:0.01114)0.756.109:0.00363
)0.785.116:0.00373)0.773.124:0.00362,Ult30374:5.5E-
4)0.850.148:0.00362)0.853.110:0.00362,UltrS430:5.3E-
4)0.902.143:0.00727)0.902.144:0.01059)0.254.10:5.4E-
4,((((Ult30329:0.01103,Ult30330:5.5E-4)0.860.111:0.00741,((UltCa363:5.5E-
4,((Ult30339:0.00363,Ult30340:0.00364)1.000.950:0.00161,Ult30345:0.05058)
0.999.200:0.00209)0.868.120:5.3E-4,(Ult30338:0.00206,(TmPhylum:5.4E-
4,(Ult30341:0.01094,(Ult30413:0.00723,(Ult30337:5.5E-
4,(CddDivi7:0.00733,Ult30407:0.00362)0.238.13:5.5E-4)0.958.141:5.5E-
4)0.888.151:5.4E-
4)0.968.108:0.01453)0.929.133:0.00185)0.931.146:0.01058)0.885.137:0.00742

)0.948.145:0.01525,Ult30373:0.01153)0.799.103:0.00399,Ult30328:0.00428)0.
946.123:0.01385)0.956.123:0.01539,Ult30342:0.02308)0.734.72:0.00348)0.914
.158:0.01291)0.852.133:0.01063)0.895.148:5.4E-
4)0.866.145:0.00904)0.828.83:5.4E-
4,Ult30317:0.02256)0.763.128:0.00555,(Ult30324:0.01399,((Ult30326:0.01892
,Ult30327:0.03982)0.944.115:0.03252,(Ult30322:0.03112,(UltCa362:0.0233,((
Ult30323:0.0266,(Ult30320:0.01883,(Ult30319:0.00362,Ult30321:0.00731)0.89
9.140:5.4E-
4)0.881.150:0.01275)0.934.121:0.02207,Ult30325:0.00592)0.749.99:0.00483)0
.855.138:0.00913)0.440.16:0.00809)0.655.23:0.01952)0.984.107:0.03795)0.86
0.112:0.01098)0.732.41:0.00478,CddDivi8:0.0441)0.775.109:0.00887)0.897.13
9:0.03211)0.947.116:0.05135)0.982.93:0.05311,((Ult30292:0.03597,(Ult30289
:0.00829,(((Ult30285:5.5E-
4,Ult30286:0.00368)0.788.105:0.00393,(Ult30287:0.00748,Ult30288:0.00368)0
.773.125:0.00351)1.000.951:0.06617,Ult30290:0.0348)0.644.20:0.00439)0.986
.109:0.06816)0.850.149:0.04181,UltrS429:0.05011)0.966.140:0.04524)0.621.1
7:0.02112)0.856.118:0.01744,(UltCa333:5.4E-
4,(Ult30159:0.03338,(Ult30158:5.5E-
4,Ult30157:0.00707)0.996.134:0.07446)0.948.146:0.04362)0.998.181:0.09159)
0.653.17:0.01723,((Ult30160:0.1247,(Ult29004:0.06234,(UltrS369:0.19501,Ul
t29005:0.12983)0.982.94:0.12163)1.000.952:0.24361)0.938.97:0.08876,((Ult3
0161:0.07506,UdnBac81:0.08109)0.963.109:0.07113,(UltrS412:0.08725,(((Ult3
0154:0.01695,(Ult30152:0.00895,Ult30153:0.04431)0.857.138:0.01376)0.605.1
9:0.01347,(Ult30155:0.00885,Ult30156:0.07116)0.941.120:0.03633)0.980.101:
0.05025,((UltrS411:0.06898,(Ult30151:0.02777,UltCa332:0.07039)0.481.18:5.
5E-
4)0.948.147:0.0309,(Ult30150:0.02795,(Ult30149:0.06366,(((UltrS410:5.5E-
4,(((Ult30143:0.0,Ult30144:0.0):0.0099,UltrS409:0.02679)0.751.78:0.00674,
(Ult30145:0.01233,Ult30146:0.01835)0.825.89:0.01564)0.861.158:0.01045)0.8
43.113:0.00746,Ult30142:0.04091)0.849.124:0.00763,(Ult30147:5.5E-
4,Ult30148:0.00369)1.000.953:5.5E-
4)0.937.133:0.02772)0.749.100:0.01956)0.970.120:0.04249)0.962.129:0.03374
)0.698.30:0.00611)0.928.122:0.04195)0.928.123:0.05751)0.645.19:0.01585)0.
953.103:0.0452)0.881.151:0.02273)0.930.133:0.03329,(((((Ult29585:0.01073,
UltPl562:5.4E-
4)0.991.118:0.09315,(((Ult29571:0.10176,(UltCa178:0.00744,UltCa177:5.5E-
4)0.999.201:0.10221)0.794.94:0.01498,(((UltAna32:0.08239,Ult29568:0.10482
)0.192.12:0.016,Ult29570:0.09448)0.827.99:0.0156,(UltOr392:0.08465,(Ult29
578:0.00576,Ult29579:0.00173)0.993.116:0.07363)0.851.139:0.01859)0.515.13
:0.02044)0.788.106:0.01264,((((Ult29573:0.02411,(Ult29572:0.01042,Ult2957
4:0.04659)0.398.11:0.02946)0.914.159:0.02992,(UltEu123:0.03754,(Ult29575:
0.02686,(Ult29576:0.0,Ult29577:0.0):0.00749)0.965.109:0.04892)0.490.9:0.0
2105)0.999.202:0.12037,(Ult29582:0.09749,(Ult29569:0.06055,(Ult29580:0.02
884,Ult29581:0.03233)0.980.102:0.05515)0.016.5:0.02269)0.533.13:0.03041)0
.714.36:0.00795,(UltCa179:0.05261,(Ult29584:0.05055,(Ult29583:0.0341,(Ult
Pl563:0.01236,(Ult29586:0.02916,Ult29587:0.03041)0.011.8:0.02144)0.991.11
9:0.06844)0.438.20:0.04189)0.964.132:0.04976)0.746.76:0.01132)0.828.84:0.
01798)0.800.85:0.03027)0.984.108:0.06561,((((Ult29668:0.06257,Ult29669:0.
11765)0.791.98:0.03026,(Ult29702:0.12953,(((UltCa188:0.14203,(Ult29656:0.
1468,(Ult29662:0.02597,Ult29663:0.04517)0.963.110:0.07815)0.924.137:0.084
81)0.979.117:0.11615,((Ult29700:0.12363,Ult29699:0.08623)0.928.124:0.0501
8,(Ult29701:0.14297,((Ult29698:0.10867,(((((UltCa199:0.02744,UltEu124:0.0
2996)0.802.93:0.01309,UltCa198:0.01465)0.728.50:0.00444,Ult29681:0.00388)
0.964.133:0.03206,((Ult29678:0.0048,(Ult29676:0.00713,((Ult29675:0.00367,

UltCa190:5.5E-4)0.982.95:0.01497,UltCa191:0.00371)0.907.150:5.4E-
4)0.989.104:0.04813)0.995.142:0.05566,(Ult29679:0.07199,((UltEu125:0.0038
1,(UltCa200:5.5E-
4,UltCa201:0.0074)0.888.152:0.00746)0.689.25:0.00559,((UltCa196:0.01986,U
ltCa195:0.01568)0.940.132:0.02318,(UltCa193:0.01525,UltCa194:5.5E-
4)0.931.147:0.02153)0.932.143:0.02403)0.882.157:0.01956)0.234.9:0.00916)0
.844.134:0.02175)0.287.8:0.00183,((Ult29677:5.3E-
4,UltCa192:0.0269)0.999.203:0.05332,(Ult29682:0.02332,(Ult29680:0.03828,U
ltCa197:0.0312)0.462.21:0.01258)0.426.26:0.00196)0.789.98:0.01893)1.000.9
54:0.1343)0.917.130:0.04564,((Ult29686:0.00295,Ult29687:0.01201)1.000.955
:0.13305,(((Ult29690:0.04669,Ult29684:0.12307)0.183.14:0.01528,(UltCa203:
0.04615,(Ult29689:0.06037,(UltCa204:0.09551,(Ult29691:0.00366,Ult29692:0.
01511)0.960.119:0.0581)0.977.101:0.08783)0.338.7:0.01789)0.860.113:0.0236
7)0.498.10:0.02042,(((Ult29683:0.07668,UltCa205:0.07123)0.922.186:0.03383
,(((Ult29697:0.01802,Ult29696:0.02512)0.994.138:0.08229,(Ult29693:0.04361
,Ult29694:0.03053)0.966.141:0.06741)0.831.98:0.02476,(Ult29688:0.07862,Ul
t29695:0.04413)0.742.67:0.01226)0.490.10:0.01195)0.733.55:0.00478,(Ult296
85:0.10105,UltCa202:0.07424)0.894.137:0.02359)0.941.121:0.03467)0.793.102
:0.01677)0.863.153:0.01632)0.943.131:0.05137)0.774.120:0.04585)0.918.151:
0.0423)0.490.11:0.01668,((((Ult29660:0.01463,(UltCa187:0.14906,(Ult29655:
0.0056,Ult29657:0.03372)0.599.17:0.0267)0.910.137:0.02781)0.864.134:0.033
14,(Ult29659:0.06769,Ult29661:0.07027)0.910.138:0.0353)0.531.12:0.01859,U
lt29658:0.0597)0.963.111:0.08747,(Ult29674:0.05029,Ult29673:0.0932)0.925.
150:0.0711)0.534.15:0.05907)0.934.122:0.04055)0.435.17:5.4E-
4)0.539.7:0.02498,(UltOr393:0.12867,(Ult29601:0.07352,(Ult29600:0.05956,(
Ult29599:0.04475,Ult29598:0.12259)0.859.126:0.03486)0.969.103:0.07375)0.9
59.129:0.07706)0.310.12:0.04281)0.678.24:0.02174,(Ult29664:0.17842,((Ult2
9672:0.11287,(UltCa189:0.06007,(Ult29670:0.03538,Ult29671:0.08521)0.785.1
17:0.01299)0.047.8:0.01829)0.979.118:0.07301,(Ult29597:0.13123,(((Ult2959
2:0.12457,UltCa181:0.0942)0.491.13:0.04346,(UltAna33:0.07681,Ult29593:0.1
7584)0.153.8:0.02746)0.813.84:0.02511,((Ult29596:0.00463,Ult29595:0.03016
)0.992.124:0.07913,Ult29594:0.04032)0.844.135:0.03573)0.916.155:0.04512)0
.739.62:0.01896)0.562.20:0.04042)0.713.35:0.02398)0.917.131:0.06395)0.893
.151:0.0506,((Ult29667:0.10402,(Ult29666:0.11477,(UltrS391:0.20622,Ult296
65:0.02812)0.857.139:5.4E-
4)0.972.117:0.07399)0.901.141:0.05794,(Ult29591:0.14171,(Ult29590:0.03602
,(Ult29589:0.03143,(Ult29588:0.0037,UltCa180:5.5E-
4)0.982.96:0.04761)0.951.125:0.04002)0.951.126:0.06062)0.983.99:0.07998)0
.000.959:6.7E-
4)0.966.142:0.06202,((Ult30106:0.08515,((Ult30109:0.06416,(Ult30108:0.095
26,Ult30110:0.10437)0.889.142:0.03935)0.755.96:0.02318,(Ult30107:0.10068,
(Ult30104:0.02134,Ult30105:0.02643)0.982.97:0.06768)0.666.22:0.06056)0.89
9.141:0.07888)1.000.956:0.24738,(((Ult29545:0.32081,((Ult29541:0.16151,Ul
t29542:0.10404)0.057.4:0.02366,(((((((Ult29508:0.01473,(Ult29507:0.04558
,Ult29510:0.06757)0.979.119:0.04897)0.862.142:0.01752,Ult29509:0.02772)0.
780.134:0.01002,(((((Ult29501:0.00734,(UltCa162:0.00262,Ult29502:0.03297)
0.990.120:0.04181)0.745.101:0.00357,(Ult29499:0.00365,Ult29500:5.5E-
4)1.000.957:5.4E-4)0.962.130:0.05731,((Ult29503:0.0,Ult29505:0.0):5.5E-
4,Ult29504:0.00364)0.999.204:0.10225)0.668.28:0.02565,Ult29506:0.02316)0.
873.134:0.03361,UltCa163:0.04318)0.076.7:0.02903)0.933.155:0.03521,((Ult2
9511:0.08292,Ult29512:0.0161)0.804.81:0.01167,UltCa164:0.12769)0.811.77:0
.03335)0.881.152:0.0216,((CddDivi6:0.13445,(Ult29516:0.08866,Ult29517:0.1
2257)0.892.131:0.03729)0.941.122:0.04237,(Ult29514:0.26257,Ult29518:0.041
68)0.109.12:0.01569)0.528.14:0.01034)0.971.97:0.04846,(Ult29515:0.0879,((

Ult29513:0.04964,UltrS390:0.18068)0.560.11:0.04514,(Ult29519:0.00245,Ult2
9520:0.01273)0.991.120:0.08028)0.462.22:0.01172)0.000.960:0.00711)0.072.6
:0.00801,((((UltCa173:0.05978,Ult29543:0.16919)0.974.82:0.07556,(UltCa172
:0.11311,UltCa171:0.02862)0.963.112:0.04791)0.345.13:0.00913,((((Ult29531
:0.05965,Ult29538:0.06251)0.649.18:0.0152,(((Ult29527:0.00364,Ult29528:5.
5E-
4)0.871.147:0.0136,(Ult29529:0.00787,Ult29530:0.01072)0.918.152:0.01712)0
.893.152:0.04758,(Ult29535:0.10256,(Ult29526:0.07863,(Ult29525:0.03153,((
Ult29523:0.0037,(UltCa165:0.01508,UltCa166:0.01128)0.776.113:0.00358)0.92
2.187:0.0071,Ult29524:5.4E-
4)0.376.7:0.01956)0.808.95:0.02876)0.977.102:0.06599)0.627.11:0.05262)0.7
20.39:0.03317)0.857.140:0.01096,(((UltCa169:5.5E-4,UltCa170:5.5E-
4)0.987.141:0.08071,Ult29536:0.11155)0.866.146:0.03138,(Ult29534:0.03936,
Ult29537:0.05128)0.743.92:0.00933)0.925.151:0.02532)0.866.147:0.02544,((U
lt29533:5.5E-
4,UltCa168:0.00365)0.813.85:0.01054,(Ult29532:0.03917,UltCa167:5.3E-
4)0.959.130:0.04064)1.000.958:0.14396)0.792.114:0.01324)0.320.9:0.00418,(
Ult29539:0.06004,Ult29540:5.4E-
4)1.000.959:0.15527)0.971.98:0.06941)0.797.91:0.02512,((Ult29522:0.14445,
Ult29521:0.06092)0.902.145:0.04085,Ult29544:0.20895)0.897.140:0.03962)0.9
23.189:0.13374)0.993.117:0.15038)0.869.116:0.05109,(Ult29715:0.32397,((((
(UltrS396:0.23224,Ult29703:0.20721)0.789.99:0.03513,Ult29612:0.25736)0.84
2.126:0.04788,(UltB4197:0.34189,Ult29704:0.1369)0.628.7:0.03239)0.069.8:0
.04185,(((Ult29617:0.06329,(Ult29620:0.13854,(Ult29618:0.03509,Ult29619:0
.08686)0.653.18:0.01168)0.630.16:0.03053)0.858.135:0.02946,(Ult29632:0.17
596,((Ult29637:0.11684,(Ult29630:0.03518,(Ult29628:0.05232,Ult29629:0.037
19)0.239.11:0.01584)0.926.160:0.0437)0.588.22:0.02149,((((Ult29625:0.0036
5,Ult29626:5.4E-
4)0.958.142:0.03934,((Ult29623:0.00772,Ult29624:0.00792)0.998.182:0.08067
,(Ult29636:0.06304,(Ult29635:0.06392,(UltrS393:0.00326,(Ult29633:0.02398,
Ult29634:0.03255)0.913.155:0.0202)0.109.13:0.01306)0.953.104:0.03394)0.58
0.11:0.01729)0.943.132:0.04301)0.259.7:0.00705,Ult29627:0.09722)0.557.15:
0.0261,((UltrS392:0.06486,(Ult29621:0.02656,Ult29622:0.03414)1.000.960:0.
12332)0.813.86:0.02257,Ult29631:0.10386)0.779.111:0.01296)0.933.156:0.042
21)0.231.11:0.01245)0.886.170:0.04816)0.978.92:0.06718,((Ult31167:0.23987
,((((((Ult29640:0.02899,UltrS395:0.14634)0.979.120:0.06487,Ult29639:5.4E
-
4)0.926.161:0.01882,Ult29638:0.00384)0.988.123:0.05098,(Ult29641:0.05708,
Ult29642:0.04576)0.367.19:0.00975)0.932.144:0.04714,((((Ult29644:0.07244,
(Ult29648:0.07223,Ult29649:0.03126)0.962.131:0.04711)0.644.21:0.04062,(Ul
t29647:0.11522,(UltCa185:0.04237,(Ult29646:0.08701,Ult29645:0.1181)0.230.
9:0.02467)0.976.103:0.04791)0.974.83:0.06681)0.859.127:0.03148,Ult29650:0
.04427)0.917.132:0.05268,Ult29643:0.04779)0.958.143:0.05976)0.833.112:0.0
2737,UltrS394:0.13695)0.838.119:0.02215,(((Ult29609:0.02245,(((UltCa182:0
.01032,((Ult29603:0.0495,Ult29606:0.09158)0.361.10:0.01807,Ult29607:0.051
3)0.216.11:0.01391)0.594.20:0.01561,(Ult29611:0.04647,(Ult29602:0.04436,U
lt29610:0.03611)0.545.15:0.00883)0.929.134:0.02115)0.884.142:0.01535,((Ul
tCa183:0.02201,UltCa184:0.02755)0.739.63:0.01475,(Ult29604:0.02025,Ult296
05:0.03609)0.982.98:0.0534)0.844.136:0.03041)0.976.104:0.05162)0.000.961:
0.00137,(UltPl564:0.33664,Ult29608:5.5E-
4)0.935.128:0.06162)0.997.144:0.15646,(Ult29616:0.09065,(Ult29615:0.0375,
(Ult29613:0.02663,Ult29614:0.00488)0.940.133:0.07431)0.984.109:0.12316)0.
976.105:0.10556)0.914.160:0.05508)0.595.12:0.0177)0.974.84:0.10621,(UltCa
186:0.21272,((((Ult29708:0.00539,(Ult29710:0.07365,((Ult29706:0.04967,Ult

29709:0.01965)0.788.107:0.01633,Ult29707:0.10681)0.680.25:0.009)0.754.91:
0.01427)0.980.103:0.10828,(Ult29712:0.07179,(UltCa207:0.00674,Ult29711:0.
02823)0.695.21:0.04935)1.000.961:0.17011)0.407.18:0.01954,(Ult29713:0.274
18,(UltAna40:0.10439,(UltCa327:0.07249,(UltCa325:0.07723,UltCa326:0.13042
)0.302.14:0.03832)0.835.102:0.03473)0.958.144:0.11205)0.891.115:0.07266)0
.870.127:0.02954,(Ult29714:0.02308,(UltCa208:0.02689,UltrS397:0.04308)0.8
49.125:0.03604)0.997.145:0.10748)0.838.120:0.02846)0.484.22:0.01441)0.927
.132:0.02721)0.288.12:0.01413)0.979.121:0.06898,((UltCa220:0.15197,((((Ul
tOr397:0.07615,Ult29727:0.08022)0.854.131:0.02384,(UltMa113:0.08282,(Ult2
9728:0.0807,(((UltOr395:0.00969,(UltOr396:0.01663,Ult29725:0.0226)0.550.1
1:0.00563)0.987.142:0.05889,((Ult29723:0.00867,((UltAna35:0.00227,Ult2972
4:0.00888)0.727.44:0.00813,(Ult29722:0.00367,(TlnMeth3:0.00368,Ult29721:5
.5E-4)0.855.139:5.5E-
4)0.998.183:0.04542)0.519.10:0.00704)0.854.132:0.00788,(UltCa212:0.02645,
UltCa213:5.3E-
4)0.966.143:0.01915)0.932.145:0.03862)0.949.130:0.04416,((Ult29726:0.0555
9,((UltAna34:0.01535,(Ult29716:0.03554,(UltCa210:0.01513,UltCa209:0.01909
)0.724.43:0.00727)0.740.79:0.00377)0.874.157:0.0098,(((Ult29719:0.01366,U
ltEu126:0.02078)0.882.158:0.01565,((UltEu128:0.06687,Ult29720:0.01421)0.1
31.10:0.02503,UltEu127:0.02696)0.885.138:0.01938)0.720.40:0.00436,(Ult297
18:0.02324,(Ult29717:0.01482,UltCa211:5.5E-
4)0.923.190:0.01502)0.938.98:0.02036)0.817.73:0.00785)0.862.143:0.01939)0
.846.140:0.02635,(Ult29729:0.14765,UltOr398:0.11808)0.928.125:0.06385)0.3
54.14:0.01116)0.809.93:0.04494)0.942.134:0.04182)0.354.15:0.00845)0.783.1
27:0.01482,Ult29742:0.11983)0.976.106:0.06568,(((Ult29736:0.09644,UltCa22
1:0.13165)0.147.12:0.02056,(((((UltOr400:0.08576,(UltOr401:0.10985,UltOr3
99:0.08301)0.817.74:0.02728)0.448.19:0.02432,Ult29733:0.01951)0.285.12:0.
03171,(Ult29730:0.08703,Ult29731:0.06882)0.201.15:0.01353)0.922.188:0.045
94,Ult29732:0.08546)0.513.12:0.03873,(Ult29734:0.11694,Ult29735:0.1852)0.
867.141:0.05685)0.906.159:0.04909)0.877.151:0.02425,(((Ult29740:0.04388,U
lt29741:0.04539)0.959.131:0.07323,(Ult29739:0.03405,(Ult29738:0.02194,Ult
29737:0.08897)0.765.106:0.01813)0.998.184:0.11178)0.829.115:0.02494,(UltC
a219:0.09778,(UltCa217:0.01349,UltCa218:0.09718)0.998.185:0.11945)0.874.1
58:0.04163)0.889.143:0.0344)0.191.12:0.00576)0.961.122:0.06442)0.293.12:0
.02148,(UltCa215:0.06607,UltCa216:0.09378)0.999.205:0.14926)0.952.132:0.0
4489)0.381.21:0.02601)0.692.19:0.00792)0.863.154:0.01197,(((UltCa330:0.19
309,(Ult29651:0.09322,Ult29653:0.03569)0.989.105:0.13487)0.529.9:0.03633,
(UltOr394:0.2719,((Ult29551:0.03139,(((Ult29550:0.07086,(UltAna28:0.00586
,UltAna29:0.01372)0.965.110:0.04184)0.983.100:0.05368,(((UltAna30:0.0956,
(Ult29556:0.01529,Ult29555:0.0407)0.649.19:0.0225)0.667.26:0.00786,(((Ult
29553:0.00793,Ult29554:0.09287)0.603.18:0.01501,((Ult29548:0.02611,UltDeh
a9:0.03909)0.951.127:0.03906,(UltOr391:0.04195,(Ult29549:0.0157,(Ult29547
:0.00372,(Ult29546:5.5E-4,UltAna27:0.00369)0.881.153:5.5E-
4)0.919.175:0.01281)0.883.150:0.01706)0.789.100:0.01066)0.491.14:0.01503)
0.806.78:0.00981,Ult29557:0.04662)0.869.117:0.00915)0.262.12:0.00935,((((
Ult29563:0.02293,Ult29562:5.5E-4)0.884.143:0.00783,((UltEu121:5.3E-
4,UltEu122:0.00371)0.780.135:0.00371,(Ult29564:5.5E-
4,Ult29565:0.0112)0.805.84:0.0037)0.748.86:0.00336)0.915.140:0.0152,(Ult2
9560:0.00389,((Ult29559:0.00698,((TlnMeth2:0.0,Ult29567:0.0):5.5E-
4,Ult29558:5.5E-
4)0.918.153:0.0159)0.999.206:0.05483,(UltAna31:0.00728,((UltCa174:0.0,Ult
29561:0.0):5.5E-4,UltCa175:0.0112)0.904.150:5.4E-
4)0.831.99:0.00764)0.791.99:0.00352)0.703.34:5.5E-
4)0.919.176:0.02227,Ult29566:0.16003)0.960.120:0.03589)0.453.15:0.00421)0

.809.94:0.01057,Ult29552:0.06496)0.887.145:0.0236)0.997.146:0.10193,UltCa
176:0.10978)0.650.20:0.02945)0.900.121:0.06673)0.934.123:0.06237,Ult30125
:0.33442)0.415.14:0.00274)0.810.86:0.02045)0.892.132:0.0333)0.874.159:0.0
296)0.721.28:0.0064,((((CanCars4:0.11146,((((CanCars2:0.02305,(PrmEndo2:5
.5E-
4,XsymbOf0:0.02701)0.765.107:0.00403)0.427.18:0.00743,CanCarso:0.03522)0.
951.128:0.01934,PrmEndo3:0.04914)0.818.68:0.00736,CanCars3:0.01242)0.708.
33:0.01393)0.881.154:0.12777,(BabBovis:0.58896,(PluFalci:0.04691,(PluBerg
h:0.0096,PluChaba:0.00562)0.885.139:0.0503)0.913.156:0.13073)0.967.99:0.2
5222)0.895.149:0.18654,((PnlTard2:0.04127,PnlPhia2:0.13158)1.000.962:0.98
936,UltB3946:0.6205)0.227.9:0.10725)0.459.19:0.14897,(UltSp225:0.25828,Ul
tB9179:0.3026)0.232.9:0.11831)0.069.9:0.1378)0.584.16:0.03098)0.908.161:0
.02715,UltCyano:0.1553)0.209.16:0.00931,((UltCan40:0.02391,(Ult12556:0.01
041,Ult12557:0.05944)0.922.189:0.03963)0.983.101:0.08916,((ExlTherm:0.052
87,((UltB5053:0.00342,(UltB5052:0.00372,((UltB5050:0.0,UltB5054:0.0):5.5E
-4,UltB5051:0.00371)0.025.4:5.5E-4)1.000.963:5.4E-
4)0.841.113:0.01163,(UltB5056:0.00394,(UltB5057:0.02737,UltB5055:5.4E-
4)0.835.103:0.00732)0.848.116:0.01541)0.995.143:0.12576)0.980.104:0.14316
,(UltSp235:0.12124,(BrpAalbo:0.02414,(BrpCorvi:0.02592,(BrpPilos:5.5E-
4,((BrpHamp2:0.0,BrpAlvin:0.0):0.00373,(BrpInnoc:0.0,BrpInno2:0.0):0.0037
1)0.612.20:0.00368)0.439.18:0.00135)0.905.144:0.02696)1.000.964:0.13896)0
.801.93:0.0705)0.955.108:0.09744)0.916.156:0.05068)0.850.150:0.0145,((Udn
Bac39:0.00697,(Ult11133:0.00746,UltPl474:5.5E-
4)0.751.79:0.00806)1.000.965:0.12824,((UltPl473:0.03501,(UltPl472:0.03488
,(UltPl470:0.0189,UltPl471:5.4E-
4)0.826.87:0.02271)0.986.110:0.08451)0.924.138:0.06085,(Ult11165:0.12846,
((UncUn122:5.4E-
4,(((PshggY80:0.01135,(UltPl510:0.00746,UltPl511:0.00372)0.788.108:0.0037
4)0.890.145:0.0075,UncUn124:5.4E-
4)0.845.128:0.0036,((UncUn123:0.00371,Ult11162:0.01136)1.000.966:5.4E-
4,(UncUn125:5.4E-4,(((UncUn121:0.0112,((Ult11161:5.4E-
4,UltPl508:0.01125)0.817.75:0.00366,(PahggY44:0.01127,((UltPl513:0.0038,(
(Otu00087:0.01469,(Otu00184:0.01049,(UltPl515:0.0128,Bacte090:0.02007)0.2
20.13:0.00525)0.953.105:0.01642)0.533.14:5.4E-4,(Ult11164:5.5E-
4,UltPl514:0.00711)0.703.35:0.00742)0.928.126:0.01166)0.802.94:0.01153,Ul
t11160:0.00348)0.995.144:5.5E-4)0.872.141:0.0113)0.992.125:5.4E-
4)0.898.155:5.5E-
4,UltPl507:0.00741)0.378.19:0.00369,(Ult11163:0.0,UltPl509:0.0):5.4E-
4)0.939.123:0.00741)0.818.69:0.0036)0.834.98:0.0114)0.918.154:5.5E-
4)0.946.124:8.2E-
4,UltPl512:0.03048)0.972.118:0.10646)0.981.109:0.11068)0.768.98:0.0443)0.
915.141:0.03916)0.950.121:0.02292)0.879.155:0.01822,(Ult12540:0.31974,(((
UltB5048:0.1028,(UltB5047:0.04309,CddDivis:0.01815)0.759.114:0.03911)0.49
8.11:0.04437,(UltB5046:0.02991,UltSp234:0.26578)0.137.11:0.0222)0.999.207
:0.13348,((UltB5254:0.05186,(UltB5255:0.02807,(UltB5253:0.00746,(UltB5252
:0.01497,UltOrg68:5.4E-
4)0.862.144:0.00743)0.998.186:0.05648)0.764.120:0.01152)0.948.148:0.07198
,((UltB5251:0.06453,UltGa287:0.14359)0.727.45:0.01502,(((((UltB5234:0.083
18,((((UltB5233:0.10975,UltB5240:0.08589)0.437.21:0.01861,UltB5241:0.0107
6)0.638.16:0.0205,UltB5236:0.05071)0.503.8:0.01405,(UltB5239:5.4E-
4,((UltSp253:0.0,UltB5238:0.0):5.5E-
4,UltB5237:0.00367)0.871.148:0.00367)0.880.155:0.01146)0.668.29:0.005)0.4
30.19:0.00835,((UltSp252:5.4E-
4,UltB9263:0.00367)0.945.107:0.02318,UltB5242:0.04587)0.866.148:0.01298)0

.882.159:0.01988,UltB5235:0.05471)0.654.19:0.01434,(UltrSo60:0.07969,(((U
ltB5203:0.03712,(((UltB5194:0.00744,UltB5193:0.01916)0.773.126:0.00631,(U
ltPlan2:0.02302,((UltB5195:0.01169,(((UltB5197:0.00745,UltB5198:0.00377)0
.807.76:0.00727,((UltB5204:0.00367,UltB5205:5.5E-
4)0.982.99:0.02294,(UltAnae5:0.00329,UltB5196:0.04408)0.942.135:0.01962)0
.774.121:5.5E-
4)0.802.95:0.0154,UltB5206:0.01528)0.885.140:0.01193)0.962.132:0.02526,((
(((UltB5134:0.0,UltB5186:0.0,UltB5222:0.0):5.5E-4,((UltB5138:5.5E-
4,UltB5143:0.00735)0.835.104:0.00366,(((UltB5120:0.01145,((((UltB5130:0.0
,UltB5132:0.0,UltB5119:0.0,UltB5118:0.0,UltB5121:0.0,UltB5124:0.0,UltB513
9:0.0,UltB5142:0.0,UltB5144:0.0,UltB5213:0.0,UltB5216:0.0,UltB5218:0.0,Ul
tB5221:0.0):5.5E-4,(UltB5141:0.0074,(UltB5217:5.5E-4,UltB5125:5.5E-
4)0.337.10:5.4E-4)0.000.962:5.3E-4)0.000.963:5.0E-
4,UltB5123:0.03868)0.352.10:5.3E-4,UltB5135:5.5E-4)0.000.964:5.5E-
4)0.000.965:5.5E-4,UltB5137:0.00744)0.460.17:5.4E-
4,(UltB5122:0.01438,UltB5133:0.01034)0.690.32:0.00133)0.550.12:5.5E-
4)0.825.90:5.5E-
4)0.724.44:0.00251,UltB5140:0.02432)1.000.967:0.00115,UltB5136:0.00373)0.
802.96:0.00365,((((UltB5226:5.5E-4,UltB5147:0.06867)0.869.118:5.5E-
4,((UltB5220:5.5E-
4,(UltB5224:0.00369,((UltB5131:0.00371,(((UltB5150:0.0,UltB5155:0.0):0.00
369,UltB5154:5.4E-4)0.834.99:0.00369,(UltB5219:5.5E-4,UltB5225:5.5E-
4)0.740.80:5.5E-4)0.790.104:0.0037)0.805.85:0.00372,(UltB5227:5.5E-
4,(UltB5146:0.00369,UltB5214:5.5E-4)0.886.171:5.5E-4)0.000.966:5.5E-
4)0.000.967:5.3E-4)0.000.968:5.5E-4)0.000.969:5.5E-
4,(UltB5149:0.0,UltB5151:0.0,CanCloac:0.0,UltB5223:0.0):5.5E-
4)0.728.51:5.5E-4)0.273.15:5.5E-
4,(UltB5152:0.00228,UltB5145:0.05445)0.998.187:0.00148)0.918.155:5.5E-
4,(UltB5215:0.03825,UltB5153:5.3E-4)0.833.113:0.00362)0.928.127:5.4E-
4)0.999.208:0.06704)0.392.16:5.5E-
4)0.722.35:0.00472)0.764.121:0.00671,(((UltB5190:0.00769,(UltB5191:0.0112
3,((UltB5187:0.0,UltB5188:0.0,UltB5229:0.0):5.5E-
4,(UltB5192:0.01142,UltB5189:0.00373)0.673.15:5.5E-4)0.913.157:5.5E-
4)0.751.80:0.00373)0.979.122:0.01896,((UltB5201:5.5E-
4,(UltB5202:0.03451,UltB5200:0.05911)0.982.100:5.3E-4)0.672.20:5.5E-
4,UltB5199:0.0037)0.896.133:0.01092)0.530.10:5.4E-
4,((UltB5129:0.00743,(((UltB5126:0.01153,UltB5127:0.02266)0.802.97:0.0113
1,(UltB5175:0.05128,((UltB5160:0.0,UltB5161:0.0,UltB5163:0.0,UltB5179:0.0
):5.4E-4,(UltB5170:0.0037,((UltB5159:0.0,UltB5162:0.0,UltB5184:0.0):5.5E-
4,(UltB5172:0.00371,UltB5164:0.00369)0.698.31:5.5E-4)0.733.56:5.5E-
4)0.868.121:0.0037)0.777.122:0.00385)0.740.81:0.00326)1.000.968:5.4E-
4,(((((UltB5169:5.5E-4,UltB5166:0.00369)0.114.5:5.5E-
4,(UltB5176:0.00369,UltB5171:0.00741)0.938.99:5.5E-4)0.000.970:5.5E-
4,UltB5182:5.5E-4)0.000.971:5.5E-
4,(UltB5165:0.00369,(UltB5148:0.0,UltB5158:0.0,UltB5167:0.0,UltB5168:0.0,
UltSp251:0.0,UltB5173:0.0,UltB5174:0.0,UltB5177:0.0,UltB5178:0.0,UltB5185
:0.0):5.5E-4)0.000.972:5.5E-4)0.613.19:5.5E-
4,((UltB5183:0.00419,UltB5181:0.03462)0.857.141:0.00794,UltB5180:0.00369)
0.761.98:5.5E-4)0.858.136:5.5E-
4)0.905.145:0.01139)0.985.114:0.03483,(UltB5157:0.012,(UltCand6:0.00373,(
UltB5156:0.0,UltB5228:0.0):0.01923)0.876.124:0.01533)0.987.143:0.03955)0.
840.129:0.01012)0.951.129:0.01645)0.357.14:0.0113)0.284.10:0.00716,((UltB
5230:0.0,UltB5231:0.0):5.5E-
4,UltB5232:0.00367)0.953.106:0.04075)0.984.110:0.05583,((UltrSo59:0.02481

,((UltB5209:0.00631,UltB5210:0.00513)0.980.105:0.02977,((UltB5208:5.5E-4,UltB5207:0.15652)0.987.144:0.04691,(UltB5211:5.5E-4,UltB5212:5.5E-4)0.905.146:0.01842)0.801.94:0.01223)0.632.16:0.01189)0.055.5:5.3E-4,StmClone:0.03118)1.000.969:0.11202)0.141.9:0.01428)0.993.118:0.05178)0.637.14:0.01085,((UltB5247:5.4E-4,(UltB5246:5.5E-4,(UltB5244:0.01122,UltB5245:0.01544)0.949.131:0.01548)0.999.209:0.03096)0.428.21:0.01609,(UltB5243:0.07955,(Cuiiiiii:0.01926,(UltB5248:0.00916,(UltB5250:0.05434,UltB5249:0.00553)0.631.17:0.01584)1.000.970:0.08378)0.190.10:0.00836)0.963.113:0.0491)0.999.210:0.09771)0.750.79:0.00946)0.971.99:0.08769)0.970.121:0.07842)0.863.155:0.03813)0.817.76:0.02836)0.565.11:0.01241)0.848.117:0.01273)0.738.63:0.00869)0.677.23:5.4E-4)0.639.23:0.00309,(Ult31628:0.07934,(Ult17465:0.05268,(UltOr286:0.16353,UltGa302:0.06189)0.837.95:0.05677)0.993.119:0.10676)0.794.95:0.04121)0.743.93:0.00614,(Ult16420:0.04252,((Ult16417:0.01406,Ult16418:0.04829)0.969.104:0.04153,((Ult16421:0.06076,(BacN2yML:0.05629,AcaPedis:0.03146)0.160.8:0.015)0.903.127:0.02217,(Ult16419:0.01484,UltAc841:0.04636)0.906.160:0.02082)0.331.13:0.00243)0.929.135:0.037)0.998.188:0.05798)0.071.9:5.4E-4)0.837.96:0.02223)0.927.133:0.03877)0.861.159:0.01351)0.778.111:0.00805)0.665.21:0.00654)0.905.147:0.02317)0.832.98:0.01512,(((UltRu374:0.06319,(UltLac38:0.04328,Otu01119:0.00566)0.965.111:0.048)0.953.107:0.05017,(UltClo80:0.12563,(Otu00265:0.06266,(UltLac42:0.05281,Otu00795:0.08517)0.622.13:0.02506)0.376.8:0.0221)0.846.141:0.02405)0.868.122:0.02428,((UltEub73:0.0433,UltLac41:0.04909)0.900.122:0.02148,((((((CsrSpe12:0.06009,UltEub71:0.03524)0.775.110:0.0073,((Ult21504:0.00505,Ult21503:0.08556)0.853.111:0.01134,(Ult27022:0.06202,(((AtPYy127:0.01878,CsrSpe11:5.3E-4)0.932.146:0.03989,(Ult21502:0.00905,((UltEub70:0.04897,Ult21501:0.02689)0.948.149:0.02524,(((UltEub69:0.00755,(Ult21499:0.00375,Ult21500:5.5E-4)0.580.12:5.5E-4)0.984.111:0.03708,(UltEub68:0.01198,(UltEub67:0.03255,UltEub66:0.00342)0.802.98:0.00777)0.888.153:0.01609)0.890.146:0.01253,UltEub65:0.00677)0.855.140:0.00942)0.925.152:0.01992)0.827.100:0.0194)0.892.133:0.03107,(Ult17838:0.07882,((((((Ult25196:5.4E-4,(Ult25195:0.00324,Ult20745:0.05876)0.808.96:0.00432)0.749.101:0.00443,((((Ult25188:0.01946,(Ult25192:0.0116,(Ult25190:0.05957,Ult25193:0.01241)0.173.11:0.00284)0.754.92:0.00435)0.759.115:0.00366,(Ult25191:0.03226,(Ult20746:5.5E-4,Ult25189:0.00375)0.951.130:0.02016)0.934.124:0.01664)0.908.162:0.01208,((Ult25186:0.00919,UltRu516:0.02068)0.604.14:0.01086,Ult25187:0.03812)0.709.33:0.00216)0.951.131:0.01742,(Ult25185:0.01602,((Ult25183:0.00749,Ult25184:0.00375)0.627.12:5.5E-4,(((Ult25178:0.0,Ult25182:0.0):5.5E-4,(Ult25179:0.00374,Ult25181:0.00752)0.635.11:5.5E-4)1.000.971:5.4E-4,Ult25180:0.02712)0.794.96:0.00369)0.895.150:0.01098)0.883.151:0.01068)0.925.153:0.0194,Ult25194:0.0422)0.462.23:0.01045)0.884.144:0.01062,(UltB8372:0.02324,((Ult25197:0.04015,(UltRu517:0.05038,Ult25198:0.00799)0.767.104:0.01509)0.959.132:0.02411,((Ult25203:0.04035,Ult25204:0.02326)0.464.18:0.00943,(Ult25202:0.02343,Ult25201:0.04318)0.892.134:0.0181)0.780.136:5.4E-4)0.904.151:0.01163)0.705.26:5.4E-4)0.921.145:0.01574,(Bfhggg77:0.01982,(Ult25205:0.01428,Ult25200:0.05695)0.813.87:0.01437)0.621.18:0.01183)0.823.98:0.01092,Ult25199:0.0156)0.842.127:0.01824,((((Ult25299:0.00374,(Ult25298:0.00376,(((Ult25289:0.02799,(Ult25290:0.02341,(Ult25288:0.04879,UltMoll5:0.00737)0.414.16:0.00822)0.772.119:0.00342)0.965.112:0.01996,((((Ult25296:5.3E-4,(Ult25291:0.00371,Ult25295:5.5E-4)0.360.19:0.00755)0.904.152:0.00756,(Ult25294:0.00759,(((UltErys2:0.0114

6,(Ult25207:5.3E-
4,Ult25206:0.02329)0.915.142:0.01155)0.750.80:0.00354,(Ult21890:0.04189,U
ltMoll6:0.03726)0.924.139:0.01998)0.895.151:0.00776,(Ult25293:0.00737,((U
lt25292:0.01972,(Ult25283:0.00753,Ult25284:0.00376)0.991.121:0.0314)0.721
.29:0.00352,(Ult25285:0.01567,(Ult25282:0.01925,Ult25286:5.4E-
4)0.473.15:0.00756)0.912.171:0.01146)0.892.135:0.00759)0.941.123:5.4E-
4)0.877.152:0.00758)0.890.147:0.00759)0.774.122:0.00373,Ult25297:0.01138)
0.857.142:5.4E-4,UltRu521:5.4E-
4)0.890.148:0.00754)0.775.111:0.00371,Ult25300:5.5E-4)0.514.13:5.4E-
4)0.887.146:0.00755)0.893.153:0.00764,Pshggg62:0.00376)0.770.127:0.00368,
(Ult25287:0.02022,((Ult25222:0.01903,(Ult25224:5.5E-
4,(Ult25225:0.0,Ult25226:0.0):5.5E-4)0.827.101:5.5E-
4)0.999.211:0.07241,(Ult25280:0.01324,(((Ult25250:0.00393,(Ult25247:0.027
97,Ult25248:0.01595)0.953.108:0.01939)0.798.100:5.5E-
4,((Ult25244:0.0,Ult25245:0.0):5.3E-
4,Ult25246:0.00754)0.903.128:0.0149)1.000.972:0.09136,Ult25281:0.00362)0.
905.148:0.01398)0.188.12:0.00405)0.918.156:0.01883)0.922.190:0.01515)0.76
0.111:0.00434,((PahggY86:0.02647,((((Ult25238:0.02744,Ult25239:5.5E-
4)0.875.157:0.02224,(((Ult25243:0.01805,(((Ult25242:0.0082,UltRu520:0.067
26)0.706.34:0.00332,(PahggY87:0.02305,UltMoll4:0.01218)0.956.124:0.02428)
0.845.129:0.01073,Ult25241:0.03309)0.971.100:0.02406)0.828.85:0.01,((Ult2
5232:5.4E-
4,(Ult25234:0.0,Ult25235:0.0):0.00374)0.896.134:0.01547,(Ult25237:0.0079,
Ult25236:0.01122)0.958.145:0.0243)0.714.37:0.01239)0.956.125:0.03074,(Ult
Ru519:0.02433,(Ult23515:5.4E-
4,Ult25233:0.00708)0.809.95:0.0147)0.914.161:0.02559)0.951.132:0.02977)0.
904.153:0.01333,((Ult25230:0.00403,Ult25229:0.01131)0.857.143:0.0076,(Ult
25223:0.02268,(Ult25227:0.0,Ult25228:0.0):0.04028)0.981.110:0.03564)0.940
.134:0.01607)0.757.86:0.00743,(Ult25240:0.04446,((((Ult25217:0.0,Ult25218
:0.0):5.5E-
4,(Ult25220:0.01142,(Ult25221:0.01165,Ult25231:0.0198)0.770.128:0.00371)0
.546.13:0.00761)0.862.145:0.00761,(Ult25215:0.00375,Ult25216:5.2E-
4)0.955.109:0.01569)0.764.122:0.00365,Ult25219:0.0077)0.887.147:0.00745)0
.037.5:5.4E-
4)0.914.162:0.01688)0.844.137:0.02228,((((((Ult25210:0.00359,((Ult25208:0
.0,Ult25209:0.0):5.4E-
4,UltRu518:0.03553)0.812.83:0.01165)0.958.146:0.01542,Ult25211:5.5E-
4)0.990.121:0.02809,(Ult25212:0.00375,(Ult25213:0.0,Ult25214:0.0):5.5E-
4)0.816.89:0.00728)0.791.100:0.0074,Ult25424:0.02864)0.993.120:0.04584,((
(Ult25279:0.02377,(((Ult25252:0.00753,(Ult25254:5.5E-
4,(Ult25253:0.00375,Ult25255:0.01139)1.000.973:5.4E-4)0.895.152:5.4E-
4)0.951.133:0.01152,(((Ult25261:0.00725,Ult25258:0.06046)0.907.151:5.4E-
4,(Ult25262:5.5E-
4,(Ult25257:0.00374,((Ult25256:0.0,Ult25259:0.0,Ult25260:0.0,Ult25264:0.0
,Ult25265:0.0):5.5E-4,Ult25263:5.5E-4)0.000.973:5.5E-4)0.497.9:5.5E-
4)0.895.153:5.5E-
4)0.893.154:0.01043,Ult25266:0.00609)0.813.88:0.00603)0.388.15:5.4E-
4,Ult24026:0.00748)0.976.107:0.02377)0.839.113:0.00785,((Ult25269:0.0329,
(Ult25268:0.0191,Ult25267:0.00413)0.918.157:0.01589)0.817.77:0.00777,(((U
lt25274:0.0,Ult25275:0.0):5.5E-
4,((((Ult25270:0.00762,Ult25423:0.03202)0.852.134:0.00757,(Ult25273:0.007
58,Ult25271:0.01922)0.764.123:0.00373)0.252.10:5.5E-4,Ult25272:5.3E-
4)0.907.152:0.00379,Ult25276:5.5E-4)0.344.13:5.4E-4)0.920.164:5.5E-
4,(Ult25278:0.03678,Ult25277:0.01582)0.845.130:0.00746)0.774.123:0.00414)

0.983.102:0.0242)0.775.112:0.00318,Ult25304:0.01146)0.944.116:0.02459)0.8
09.96:0.02323,(UltErysi:0.02214,(Ult25249:0.04148,(Ult25251:0.01643,Ult25
303:0.02854)0.733.57:0.0058)0.787.106:0.00965)0.562.21:0.01453)0.754.93:0
.00662)0.000.974:0.0035)0.937.134:0.03221)0.925.154:0.04191)0.996.135:0.1
0921)0.795.105:0.01529)0.848.118:0.0173)0.933.157:0.01513)0.958.147:0.021
57,((((Ult21515:0.04386,((Ult21513:0.00713,Ult21514:0.00789)0.996.136:0.0
3151,CsrColin:5.5E-4)0.014.8:0.00512)0.713.36:0.00448,(CsrPilif:5.4E-
4,UncUn136:0.05716)0.375.15:0.02625)0.875.158:0.01305,(((((Ult21510:5.4E-
4,Ult20683:0.01127)0.951.134:0.01144,Ult21509:0.01145)0.771.132:0.0036,Ps
hggg41:0.01924)0.762.116:0.00456,(Ult20536:0.05622,(Ult21511:0.00371,Ult2
1512:0.02305)0.923.191:5.5E-
4)0.989.106:0.02688)0.024.7:0.00291,(UltRu375:5.5E-
4,UltRu376:0.0037)0.998.189:0.03568)0.902.146:0.01541)0.873.135:0.014,(Ul
tClo77:0.00259,Ult22682:0.0546)0.990.122:0.05034)0.875.159:0.01418)0.895.
154:0.01616,(((Pshggg40:0.0349,UltEub47:0.03798)0.371.15:0.011,(UltEub45:
0.02377,((UltEub46:0.03842,UltFir20:0.01827)0.488.23:0.01092,(UltFir18:0.
05289,UltFir19:0.03268)0.814.78:0.02831)0.932.147:0.03097)0.971.101:0.037
6)0.833.114:0.01468,(UltCl151:0.05706,((UltEub60:0.01876,(UltClo64:0.0036
9,UltEub59:5.5E-4)0.968.109:5.4E-
4)1.000.974:0.06879,(UltEub57:0.04092,(UltEub53:0.00696,(UltEub56:0.01495
,(UltEub54:0.00743,UltEub55:5.5E-4)0.334.15:5.5E-4)0.870.128:5.4E-
4)0.792.115:0.01465)0.829.116:0.02219)0.394.20:0.00615)0.625.14:0.01057)0
.875.160:0.02068)0.468.23:0.01235,(((((OphiYyy3:0.01455,Otu00348:0.00835)
0.985.115:0.04742,((UltFir24:0.01119,UltFir25:5.5E-
4)0.872.142:0.01446,(UncUn135:0.02817,UltClo67:0.02158)0.990.123:0.04864)
0.874.160:0.02115)0.791.101:0.02174,(((CuiYyyy8:0.02501,((OphiYyy2:0.0351
3,Ult21497:5.4E-4)0.935.129:0.02109,(AtPYy122:0.03165,(((AtPYy121:5.4E-
4,AtPYy129:0.01107)0.934.125:0.02024,(AtPYyyy7:5.4E-
4,(AtPYyy12:0.01602,((AtPYyy11:0.0,AtPYyy10:0.0):0.02079,AtPYy128:0.02628
)0.581.15:0.02744)0.978.93:0.03918)0.231.12:0.02285)0.969.105:0.02476,AtP
Yyy13:0.0155)0.725.47:0.00377)0.975.103:0.02227)0.389.14:0.00409)0.929.13
6:0.026,AtPYy126:0.03428)0.827.102:5.5E-
4,(AtPYyy15:0.07398,((AtPYy123:0.01122,AtPYyy14:5.5E-
4)0.972.119:0.03295,(AtPYy124:0.017,AtPYy125:0.16625)0.833.115:0.03629)0.
715.32:0.0128)0.615.20:5.2E-
4)0.958.148:0.0282)0.762.117:0.00742,((((UltClo73:0.0075,(UltClo70:0.0192
7,(Ult21498:5.5E-
4,(UltClo71:0.01983,(UltClo68:0.02345,UltClo69:0.00397)0.746.77:0.00348)0
.885.141:0.00757)0.690.33:0.00763)0.868.123:0.00756)0.993.121:5.5E-
4,(UltClo72:0.0259,(UltEub74:0.02684,(Tryyy043:0.03324,UncUn137:0.02642)0
.956.126:0.03216)0.249.22:0.00604)0.610.14:0.01265)0.873.136:0.00771,((Ul
tEub62:0.02436,(UltEub63:0.01907,UltEub61:0.0171)0.674.23:0.00186)0.992.1
26:0.04131,((UltLac37:5.5E-4,(UltEub44:0.01123,(UltLac35:5.5E-
4,(Otu00544:0.00367,UltLac36:0.01887)1.000.975:5.5E-4)0.884.145:5.5E-
4)0.372.20:0.00714)0.991.122:0.04724,(UltEub80:0.02955,UltEub64:0.02719)0
.284.11:0.01444)0.815.80:0.01088)0.724.45:0.00286)0.939.124:0.01169,(((((
(UltEub72:0.018,(Ult21496:0.04156,Ult21505:0.02794)0.807.77:0.01052)0.892
.136:0.01668,UltClo66:0.00944)0.901.142:0.0174,((UltClo76:0.02768,Ult2150
8:0.01535)0.958.149:0.01675,UltEub58:0.02428)0.514.14:5.5E-
4)0.660.21:0.00625,Ult21506:0.05052)0.425.17:0.02115,((UncUn139:0.02466,(
(PshYyy13:0.0,Pshggg39:0.0):0.02542,UncUn138:0.03457)0.741.71:0.01133)0.7
99.104:0.01334,((UltEub82:0.01548,(UltEub84:0.01557,(UltEub83:5.3E-
4,UltLac43:0.00369)0.854.133:0.00742)0.839.114:0.00782)0.884.146:0.01226,
(UltClo75:0.01176,((UltEub77:5.5E-4,UltFir22:0.00367)0.990.124:5.4E-

4,((UltEub75:0.02697,(UltClo74:0.00383,(Ult21507:0.01892,UltEub76:5.4E-
4)0.868.124:0.01133)0.637.15:5.2E-4)0.948.150:0.01488,(UltEub79:5.5E-
4,UltEub78:0.00743)0.984.112:5.5E-
4)0.805.86:0.00359)0.858.137:0.00761)0.717.31:0.00302)0.870.129:0.0107)0.
801.95:0.01281)0.864.135:0.00856,((UltEub81:0.0221,(UltFir23:0.02215,((((
UltLac40:0.01095,UltEub49:0.02689)0.969.106:5.4E-
4,UltEub50:0.0037)0.290.14:0.0037,(UltEub51:5.5E-
4,(UltEub52:0.01079,Ult21495:0.02735)0.748.87:0.00458)0.998.190:0.03096)0
.821.85:5.4E-4,UltEub48:5.4E-
4)0.974.85:0.02578)0.795.106:0.00714)0.769.110:0.00686,(UltFir21:0.01429,
Otu00417:0.02246)0.821.86:0.00897)0.926.162:0.01906)0.878.135:5.4E-
4)0.763.129:0.00414)0.720.41:0.00236,UltClo65:0.09289)0.956.127:0.02073)0
.713.37:0.0037,((BacNL298:0.00724,BacNL299:0.01169)0.998.191:0.04661,(Otu
00501:0.04538,(UltLac39:0.02549,Ult21494:0.04559)0.946.125:0.02758)0.726.
39:0.00445)0.924.140:0.02881)0.817.78:0.0084)0.507.14:0.00794)0.942.136:0
.02014)0.789.101:0.01314,(((UltBegg2:0.06237,UltEub86:0.1351)0.949.132:0.
05607,(UltGa237:0.16221,((UltB4278:0.17074,(UltB3958:0.13304,UltrSo52:0.0
5302)0.692.20:0.03129)0.813.89:0.02071,(UltRicke:0.13877,((((UltB3831:0.0
8565,(MaiMeta7:0.09352,(((((((NovTarda:5.4E-
4,((UltB3624:0.02299,(((((UltB3584:0.0,UltB3621:0.0):5.3E-
4,(UltB3658:0.03997,(((UltB3703:0.02307,(AphPro44:0.0,AphPro45:0.0):0.007
56)0.769.111:0.00362,UltAl106:0.00747)0.277.15:5.5E-4,(AphPro43:5.4E-
4,((UltB3622:0.0,UltB3623:0.0):5.5E-4,(SpgSpec8:5.4E-
4,SpgSpec9:0.01869)0.925.155:0.01488)0.975.104:0.0152)0.846.142:0.00369)0
.791.102:0.00388)0.787.107:0.00352)0.953.109:0.01859,UltB3680:0.01213)0.4
54.22:0.00772,((((((UltB3721:0.00557,(UltB3590:5.4E-
4,UltB3722:0.00368)0.985.116:0.0346)0.864.136:0.0148,UltB3611:0.26876)0.7
78.112:0.01522,SpxGinse:0.00352)0.765.108:0.00382,(SpxWitfl:0.01109,(((Sp
xMacro:0.00745,UltB3594:0.01504)0.252.11:5.3E-
4,((SpgTerra:0.01521,UltB3592:0.00366)0.894.138:0.0075,(Bac08160:0.0,SpxT
aejo:0.0):5.3E-
4)0.856.119:0.00742)0.948.151:0.0113,(((SpgMacr2:0.0,SpxMacr2:0.0,SpgSpec
5:0.0):5.5E-4,(SpgSpec4:0.00369,UltB3593:0.0153)0.938.100:5.5E-
4)0.000.975:5.5E-4,(SpgMacro:0.00369,SpgSpec3:0.00369)0.661.20:5.5E-
4)0.788.109:5.4E-4)0.886.172:5.5E-
4)0.523.12:0.00744)0.769.112:0.00388,(SpxAlask:0.0,SpxSpeci:0.0):0.01517)
0.933.158:5.5E-
4,UltB3591:0.02673)0.891.116:0.02412)0.290.15:0.01079,(((UltParvi:0.02677
,SpxSpec2:5.4E-4)0.762.118:0.0075,(AphPro39:5.5E-
4,(UltB3575:0.03207,UltEryth:0.01571)0.344.14:0.00734)0.906.161:0.01134)0
.956.128:0.01523,(SpxBaekr:0.01131,(((((NovHassi:5.5E-
4,(((UltB3566:0.0037,((NovNitro:0.0,UltB3578:0.0):5.5E-
4,(((SpxLitor:0.0113,AntBac26:5.4E-
4)0.932.148:0.00747,(SpgSpec6:0.01504,(UltB3626:0.0067,(SpgmRosa:5.4E-
4,(UltB3587:0.01117,UltB3586:5.3E-
4)0.388.16:0.00367)0.997.147:0.04402)0.900.123:0.01719)0.803.77:5.4E-
4)0.787.108:0.00372,((NovAcidi:0.0,UltB3565:0.0):0.00369,(UltB3577:0.0111
7,(SpgCapsu:5.5E-4,SpgSpec2:5.5E-4)0.899.142:5.5E-
4)0.864.137:0.00373)0.465.16:5.5E-4)0.907.153:0.00754)0.238.14:5.5E-
4)0.913.158:0.00766,(BltNatat:0.00722,(UltSphi3:0.00756,((UltB3613:0.0037
7,AtrEpoxi:0.0154)0.772.120:0.00359,(UltPro37:0.00368,(UltB3617:5.5E-
4,(SpmBacte:0.00743,UltAl104:0.01121)0.819.90:5.5E-4)0.976.108:5.5E-
4)0.900.124:0.00759)0.868.125:0.00764)0.844.138:0.00722)0.927.134:5.4E-
4)0.827.103:0.00377,UltB3599:0.0151)0.770.129:5.4E-

4)0.851.140:0.00706,(SpgSuber:0.01917,(((SpbSpec4:0.01465,((UltSph15:0.00
375,(UltB3671:5.5E-4,(SpgSpe23:0.00371,(SpbXenop:5.5E-
4,(((((SpgSpe19:0.01089,(UltB3675:0.00331,AphPro46:0.00795)0.677.24:0.007
55)0.877.153:5.3E-4,SpbRhizo:5.5E-
4)0.916.157:0.00361,(SpgSpe24:0.0,Udntdddd:0.0,SpgSpe28:0.0):5.4E-
4)0.729.40:0.00743,(UltB3676:5.5E-
4,(UltB3677:0.00371,UltB3672:0.01495)0.156.12:5.5E-
4)0.916.158:0.00362)1.000.976:5.4E-4,UltSph16:0.00377)0.591.19:5.5E-
4)0.000.976:5.5E-4)0.475.16:5.4E-4)0.889.144:5.4E-
4)0.979.123:0.01516,(((SpbAmien:0.0,SpbSpec2:0.0):5.5E-
4,UltSph14:0.00369)1.000.977:5.4E-
4,(((((UltB3667:0.00369,((SpgSpe26:0.0,SpgSpe27:0.0):5.3E-
4,(SpbUmmar:5.4E-4,((SpbCloac:0.0,SpgSpe20:0.0):5.5E-
4,SpgSpe21:0.00746)0.845.131:0.00369)0.857.144:0.0037)0.831.100:0.0037)0.
950.122:5.4E-4,(UltB3666:0.00369,PsdAbiko:0.00371)0.704.29:5.5E-
4)0.000.977:5.5E-4,(SpgSpe25:0.0,SpbmOlei:0.0):5.5E-
4)0.919.177:0.00369,UltB3669:5.5E-4)0.068.9:5.4E-
4,UltB3668:0.0074)0.960.121:0.01125)0.845.132:0.00372)0.870.130:5.5E-
4)0.579.19:0.00749,(SpbSpec3:0.02636,(UltB3670:0.00295,(Bfhgg107:5.5E-
4,UltB3678:0.01135)0.972.120:0.01592)0.407.19:0.00376)0.839.115:5.5E-
4)0.958.150:0.01154,(Bac07282:0.01528,(UltB3665:0.0189,((SpbAroma:0.02739
,(UltB3673:5.3E-4,((UltB3674:0.01949,((SpbChlo2:0.0,SpbChlor:0.0):5.4E-
4,((SpgSpe15:0.0037,(SpbChung:0.0,SpgAgres:0.0):5.5E-
4)0.939.125:0.00746,(SpbChinh:0.0,SpgSpe16:0.0,SpgChlor:0.0):5.5E-
4)0.824.85:0.00369)0.859.128:0.01188)0.788.110:0.00896,(UltB3679:0.01363,
UltB3702:0.01341)0.690.34:0.01177)0.923.192:0.01351)0.991.123:0.02319)0.7
55.97:0.00361,((UltSph12:5.5E-4,((SpbSpeci:0.0037,UltSph13:5.5E-
4)0.934.126:5.5E-4,(SpgSpe22:0.0,SpbYanoi:0.0):5.5E-4)0.000.978:5.5E-
4)0.850.151:5.5E-4,UltAl107:0.00369)0.858.138:5.4E-4)0.984.113:5.1E-
4)0.895.155:0.01093)0.550.13:5.4E-
4)0.879.156:0.00743)0.765.109:0.00415)0.018.3:0.00375,UltNovos:0.01861)0.
439.19:0.00416,(UltB3597:0.01906,(((UltAnae2:0.0,UltB3585:0.0):5.5E-
4,(((UltNovo2:0.00373,UltB3598:0.00753)0.882.160:0.00756,UltAl101:0.00372
)0.805.87:0.00368,(SpgAroma:5.5E-4,SpgArom2:5.5E-
4)0.860.114:0.0037)0.777.123:5.4E-4)0.912.172:5.5E-
4,(UltB3576:0.0111,(SpgSubte:5.4E-
4,UltB3618:0.00377)0.870.131:0.007)0.923.193:5.4E-4)0.765.110:5.5E-
4)0.924.141:0.00744)0.913.159:5.4E-4,(UltB3568:5.5E-
4,UltB3569:0.01121)0.827.104:0.0037)0.820.83:5.5E-
4,((UltB3571:0.00376,(UltB3588:0.00377,(UltB3589:0.01123,((NovResi2:0.0,N
ovResin:0.0,UltNovo3:0.0,NovSpeci:0.0,NovNapht:0.0,NovPenta:0.0,SpgSpec7:
0.0,NovPanip:0.0,UltB3620:0.0):5.5E-4,UltB3619:0.0074)0.874.161:5.4E-
4)0.559.16:0.00373)0.930.134:0.01138)0.780.137:0.00373,(((SpgSpeci:0.0151
3,(UltB3567:0.00748,(SpgStyg2:0.0,SpgStygi:0.0):5.5E-
4)0.778.113:0.00364)0.896.135:0.0075,(((((UltB1957:0.00382,UltB3574:0.011
39)0.885.142:0.00772,UltB3580:0.00376)0.760.112:0.00376,UltB3579:0.00368)
0.886.173:0.00756,UltSphin:0.00369)0.398.12:5.4E-4,UltB3596:5.5E-
4)0.260.12:5.4E-4)0.574.12:5.4E-4,((UltB3573:0.00369,UltB3572:5.5E-
4)0.860.115:0.0037,(UltB3570:5.5E-4,NovLentu:0.0037)0.000.979:5.4E-
4)0.867.142:0.0037)0.911.171:0.00759)0.793.103:0.00373)0.456.18:0.00375)0
.783.128:5.5E-4)0.993.122:5.4E-
4)0.801.96:0.00357)0.869.119:0.00766,UltOrg30:0.01921)0.770.130:0.00368)0
.588.23:5.4E-
4,(UltB3582:0.09744,UltB3595:0.01945)0.758.105:0.00771)0.750.81:0.00381,(

((((AphPro37:0.00369,UltAl102:5.5E-4)0.900.125:5.4E-
4,(SpmBact2:0.01539,AphPro36:0.01535)0.744.79:0.00375)0.675.18:5.5E-
4,(AtrIndic:0.0041,((UltNovo4:0.0,UltLutib:0.0):0.03551,(ErySpeci:0.00777
,UltB3625:0.00726)0.947.117:0.01611)0.826.88:0.00723)0.968.110:0.02261)0.
757.87:0.01143,((AphPro41:0.00743,UltSphi5:5.5E-
4)0.958.151:0.01846,(((EryLitor:0.00355,((UltB3610:0.07811,(((UltB3606:0.
0,PphTepid:0.0):5.5E-
4,UltSphi4:0.00369)0.730.53:0.00245,((ErtRamos:0.0,ErtRamo2:0.0):5.5E-
4,(((((PphSangu:0.0,PphSpeci:0.0,PphNeust:0.0):5.5E-
4,(UltPorph:0.00369,UltB3607:0.00369)0.700.30:5.5E-4)0.988.124:5.5E-
4,(UltB1456:0.02232,UltB3608:0.01485)0.844.139:5.4E-
4)0.986.111:0.00209,(((UltB3609:0.00399,(ErySpec4:0.05093,(UltB3603:5.4E-
4,(UltAl105:0.00725,UltAlte7:0.01555)0.470.10:0.00747)0.621.19:0.01682)0.
500.21:0.00215)0.865.142:0.01001,UltB3616:0.01461)0.865.143:0.00992,UltPo
rp2:5.4E-
4)0.986.112:0.02328)0.924.142:0.00131,(UltB3602:0.00797,PphCrypt:0.01097)
0.757.88:0.00748)1.000.978:5.4E-4)0.743.94:0.00192)0.247.13:5.5E-
4)0.865.144:0.01385,(UltB3615:0.01191,(PphDokdo:0.00624,BltSpeci:0.01455)
0.785.118:0.00595)0.672.21:0.00771)0.750.82:0.00403)0.785.119:0.00381,(At
tSpeci:5.5E-4,(UltB3612:0.00368,(EryGaetb:0.00753,UltB3614:5.5E-
4)0.916.159:0.00751)0.691.21:5.4E-4)1.000.979:5.4E-
4)0.890.149:0.0075,(ErySpec6:0.00368,((AphPro42:0.0,UltOrg27:0.0):5.5E-
4,(UltB3605:0.00371,(UltB3604:5.5E-
4,((UltOrg29:0.00371,SpgPhyll:0.00369)0.947.118:5.5E-
4,(AphPro40:0.0,ErySpec5:0.0,UltOrg28:0.0):5.5E-4)0.608.23:5.5E-
4)0.779.112:5.5E-4)0.862.146:0.00369)1.000.980:5.5E-
4)0.876.125:0.00748)0.747.84:0.00511)0.926.163:0.01507)0.750.83:0.00339,(
ErySpec2:0.00762,((AphPro38:0.0,UltB3600:0.0):0.0149,(MucBact4:0.00402,((
EryLong2:0.0,EryLongu:0.0):5.4E-
4,ErySpec3:0.02297)0.473.16:0.01506)0.977.103:0.02716)0.955.110:5.5E-
4)0.971.102:0.01549)0.767.105:0.00387)0.830.96:0.01073,UltB3601:0.03051)0
.948.152:0.02202,(ErySeoha:5.5E-4,UltAl103:0.00369)0.800.86:5.4E-
4)0.809.97:0.00367,AtrLuteo:5.5E-
4)0.959.133:0.02939,(SanLimno:0.03627,((ZmmMobi3:0.0,ZmmMobil:0.0,ZmmMobi
2:0.0):0.0895,((UltB3723:0.0169,(UltB3724:0.0385,UltB3725:0.00773)0.882.1
61:0.01509)0.930.135:0.01919,((UltB3698:0.06325,UltB3697:0.03239)0.961.12
3:0.03513,(((UltB3720:0.02817,((SpgSpe12:0.01912,UltSph22:0.00381)0.860.1
16:5.5E-
4,UltB3663:0.0227)0.935.130:0.01522)0.725.48:0.00213,SndSibir:0.033)0.878
.136:0.00803,(((FlaSpec2:0.0,SpbFulig:0.0,SpgHerbi:0.0,SpgSpe18:0.0):0.00
381,SpgSpe17:0.00744)0.907.154:0.01131,(UltB3659:0.01236,(UltB3729:0.0485
1,(UltB3728:0.02298,(((UltSph20:0.00744,(((((SpgHaloa:0.0037,SpgSpe11:5.5
E-
4)0.924.143:0.01113,UltB3664:0.00401)0.267.14:0.00744,SpgWitti:0.0035)0.9
25.156:5.5E-4,SpgSpe13:5.4E-4)1.000.981:5.4E-
4,(SpgSpe10:0.01149,(SpgFenni:0.01758,UltB3660:0.0128)0.777.124:0.00731)0
.744.80:0.00747)0.925.157:0.00343)0.197.12:5.3E-
4,(UltB3657:0.00744,(UltB3656:0.00339,((UltB3638:0.004,(UltB3637:5.5E-
4,UltKais3:0.00719)0.015.8:0.00371)0.769.113:0.00374,(UltB3655:5.5E-
4,(UltB3636:0.00369,((UltKais8:0.0,UltB3635:0.0,UltKais5:0.0):5.5E-
4,UltKais6:0.00369)0.439.20:5.5E-
4)0.968.111:0.01127)0.864.138:0.00721)0.612.21:0.00753)1.000.982:5.5E-
4)0.825.91:0.00371)0.764.124:5.5E-4,((((((UltSph18:0.0074,(UltSph17:5.5E-
4,(UltB3726:0.00373,(UltSph23:0.00372,(UltB3681:0.00746,UltSph24:0.00374)

0.774.124:0.00368)0.798.101:0.00374)0.787.109:0.00371)0.878.137:0.00369)0
.651.23:5.4E-
4,(SpsnSoli:0.0,UltSphi6:0.0):0.01511)0.942.137:0.00743,(UltrSo42:5.4E-
4,(((UltrSo41:0.01328,UltB3727:0.00843)0.818.70:0.00833,(UltB3682:5.5E-
4,SpsSpeci:0.01119)0.724.46:5.4E-
4)0.453.16:0.00367,((((SpgKwang:0.00365,((UltB3692:0.0,UltB3690:0.0,UltB3
691:0.0,UltB3693:0.0,SpgSpe30:0.0):5.5E-
4,(UltB3694:0.00368,UltB3689:0.02615)0.972.121:5.5E-
4)0.869.120:0.00743)0.985.117:0.02272,SpgJapon:0.00399)0.483.18:0.00733,(
(((AntBac27:0.0,UltAnta7:0.0,SpgSpe38:0.0):5.4E-
4,SpgSpe41:0.01507)0.935.131:0.01101,(SpgAestu:0.01503,UltAl108:5.5E-
4)0.784.114:0.00396)0.441.22:0.00753,SpgSpe36:0.00708)0.886.174:5.4E-
4)0.929.137:0.00755,(UltB3683:0.01118,((((SpgMelon:0.0,SpgMelo2:0.0,SpgSp
e35:0.0):5.5E-
4,(SpgmMali:0.00369,((UltB3704:0.0,UltB3706:0.0,UltB3707:0.0,EnpBact2:0.0
,SpgInsul:0.0,SpgMelo3:0.0,UltB3718:0.0):5.4E-
4,((UltB3705:0.0,SpgDokdo:0.0):5.5E-4,(SpgMucos:5.4E-
4,(((UltAl109:0.0,SpgSpe39:0.0,SpgSpe40:0.0,UltB3717:0.0):5.5E-
4,SpgEchin:5.5E-4)1.000.983:5.5E-
4,UltB3715:0.01121)0.838.121:0.00361)0.417.21:0.00369)0.806.79:0.00362)0.
844.140:0.0037)0.574.13:5.5E-4)0.761.99:5.5E-
4,UltB3684:0.01503)0.997.148:5.4E-
4,(((((((UltB3716:0.00746,UltB3708:0.01125)0.778.114:0.00369,(UltB3713:0.
00369,UltB3714:0.0037)0.336.16:5.5E-
4)0.796.79:0.00365,SpgSpe37:0.04427)0.928.128:5.4E-
4,(UltCaul9:0.0151,(SpgSpe42:5.5E-4,(((SpgYunna:5.5E-
4,UltB3695:0.01125)1.000.984:5.4E-4,(SpgAdhae:5.5E-
4,UltB3709:0.00742)0.415.15:0.00369)0.816.90:0.00365,(UltSph19:0.0037,Ult
B3696:5.5E-4)0.975.105:0.01533)0.995.145:5.4E-4)0.931.148:5.4E-
4)0.893.155:0.00737)0.444.25:0.00746,((SpgRosei:0.01144,(SpgAbaci:0.00757
,(UltB3710:0.00747,UltB3711:0.00373)0.774.125:0.00365)0.776.114:0.00375)0
.890.150:0.00746,((((CauLeid2:0.0,SpgSpe34:0.0):5.5E-
4,CauLeidy:0.00369)0.853.112:0.00369,((SpgAzoti:0.0,SpgSpe31:0.0,SpgSpe32
:0.0,SpgSpe33:0.0):5.5E-
4,((SpgParap:0.00369,(SpgPauci:0.0,SpgSpe29:0.0,BacCAGY7:0.0,SpgPseud:0.0
,UltB3686:0.0):5.5E-4)0.908.163:0.00744,UltB3712:0.0037)0.831.101:5.4E-
4)0.767.106:5.4E-4)1.000.985:5.4E-
4,(UltB3627:0.00371,UltB3628:0.02282)0.849.126:0.01124)0.772.121:0.00361)
0.831.102:0.00365)1.000.986:5.5E-
4,((SpgPanni:0.0,UltB3688:0.0,SoaLycop:0.0):5.5E-
4,(SpgSanxa:0.01126,(SpgMollu:0.00369,((SpgDesic:0.0,BacF3200:0.0):5.5E-
4,UltB3699:0.00369)0.125.10:5.5E-4)0.869.121:0.00369)0.994.139:5.5E-
4)0.729.41:5.4E-4)0.997.149:5.4E-4,(SpgmSoli:0.015,UltB3700:5.4E-
4)0.756.110:0.00741)0.784.115:0.00363)0.737.78:0.00742)0.932.149:5.4E-
4)0.246.19:5.5E-4)0.832.99:0.00362)0.432.20:5.4E-
4)0.703.36:0.00338,(SpgKoree:5.5E-4,(UltB3701:5.4E-
4,UltB3581:0.02689)0.822.84:0.00369)0.931.149:0.01149)0.936.133:0.01804,(
(((SpmBact3:0.01126,UltKai13:5.4E-
4)0.938.101:0.00746,((((UltrSo40:0.02687,(UltB3643:0.01113,UltB3645:5.5E-
4)0.966.144:5.4E-4)0.876.126:0.00741,(UltB3644:5.4E-
4,(UltSph11:0.02701,(UltB3640:0.00743,((((UltB3649:5.4E-4,(UltB3634:5.4E-
4,((((((UltB3633:5.5E-
4,(UltKai12:0.0112,(UltPro39:0.0112,(UltB3646:0.00369,UltKai10:5.5E-
4)0.934.127:5.5E-4)0.937.135:0.01127)0.864.139:5.5E-

4)0.854.134:0.00367,UltSphi7:0.04269)0.670.18:5.5E-
4,UltB3631:0.01121)0.851.141:5.5E-
4,(UltB3632:0.0187,(UltSphi8:0.03853,UltSphi9:0.01121)0.817.79:0.00742)0.
856.120:5.3E-4)0.000.980:5.5E-
4,(UltKais2:0.0,UltKais4:0.0,UltKai11:0.0):5.5E-4)0.834.100:5.5E-
4,UltB3647:0.0037)0.918.158:0.00744)0.839.116:0.00369)0.859.129:0.0037,Ul
tB3652:0.0356)0.942.138:5.4E-
4,(UltB3650:0.01891,(UltB3641:0.00744,(UltB3642:5.5E-4,UltB3651:5.5E-
4)0.911.172:5.5E-4)0.761.100:5.5E-
4)0.789.102:0.00363)0.797.92:0.00746,SpgJasps:0.01125)0.921.146:5.5E-
4)0.848.119:0.00742)0.988.125:5.4E-4)0.246.20:5.4E-
4)0.892.137:0.00728,(SpgAstax:0.00734,((UltB3639:0.0,UltKais9:0.0):0.0037
,UltB3653:0.00371)0.775.113:0.00384)0.780.138:0.00749)0.918.159:5.5E-
4,(UltKais7:0.0,UltSph10:0.0):5.4E-4)0.139.13:5.4E-
4)0.781.110:0.00367,UltB3661:0.02299)0.764.125:0.00414,(UltB3654:0.00791,
(SpgSpe14:0.00751,UltB3662:0.00748)0.841.114:0.00713)0.885.143:0.01141)0.
860.117:0.00169)0.861.160:0.01313,(UltB3648:0.02118,UltB3719:0.04166)0.80
3.78:0.01213)0.942.139:0.01922)0.139.14:0.00772)0.818.71:0.00985)0.771.13
3:5.4E-4)0.931.150:0.0115)0.884.147:0.00776)0.728.52:5.4E-
4)0.966.145:0.02071)0.910.139:5.1E-
4)0.276.13:0.01001)0.878.138:0.0199)0.793.104:0.02576)0.712.28:0.00901)0.
720.42:0.02218,UltB3933:0.05365)0.376.9:0.00977,(UltB3830:0.05895,(UltB38
15:0.05484,(((UltSlu18:0.07839,UltB3827:0.07751)0.926.164:0.03191,UltB382
8:0.02135)0.738.64:0.01081,UltB3829:0.08736)0.926.165:0.02306)0.939.126:0
.0337)0.910.140:0.03381)0.918.160:0.02109,(((((((((UltSph25:0.10851,(UltB
3976:0.02034,UltB3977:0.01091)0.996.137:0.07445)0.814.79:0.01793,(((UltB3
984:0.05385,(UltCaedi:0.02761,(UltB3983:0.04509,CdbCaryo:0.02189)0.687.27
:0.01101)0.811.78:0.00954)0.837.97:0.01997,((UltB3982:0.00791,(UltB3980:5
.4E-
4,UltB3981:0.00717)0.892.138:0.01077)0.977.104:0.03863,UltB3969:0.12178)0
.364.12:5.4E-
4)0.832.100:0.02605,(UltB3968:0.0959,UltB4338:0.07943)0.826.89:0.03339)0.
000.981:0.01694)0.897.141:0.02791,(((CanOdyss:0.01559,(UltAl173:0.01937,(
UltAl172:0.01542,(UltAl171:0.01101,(UltAl169:0.02166,UltAl170:0.01295)0.3
44.15:0.02092)0.985.118:0.04654)0.928.129:0.02423)0.996.138:0.05843)0.921
.147:0.03121,(UltB3978:5.9E-
4,UltB3975:0.14808)0.792.116:0.02893)0.883.152:0.02733,BacEnr17:0.16341)0
.813.90:0.016)0.951.135:0.04163,UltB4021:0.16417)0.000.982:5.5E-
4,((UltB3964:0.11315,(UltAl162:0.07374,CanHepat:0.10803)0.922.191:0.05427
)0.907.155:0.05378,((((UltAl146:0.01659,(UltB3930:0.07421,(UltAl147:0.096
91,Otu00393:5.5E-
4)0.567.9:0.02557)0.070.14:0.01125)0.998.192:0.13404,(UltB3971:8.6E-
4,UltB3970:0.0103)0.996.139:0.11532)0.244.7:0.01844,(UltB4046:0.01181,((U
ltB4042:0.01222,((UltB4041:0.00729,(UltB4044:0.01505,(UltAl180:5.5E-
4,UltAl181:0.00761)0.886.175:5.4E-
4)0.987.145:0.02761)0.965.113:0.01956,UltB4296:5.4E-
4)0.875.161:0.01062)0.595.13:0.00774,(((UltB4045:5.5E-
4,UltAl182:0.00747)0.830.97:0.00369,UltrRap7:5.5E-4)0.208.8:5.4E-
4,UltB4043:0.00368)0.983.103:0.0243)0.860.118:0.01937)0.995.146:0.09015)0
.872.143:0.02516,(UltB3965:0.0165,(UltB3966:0.00458,UltB3967:0.04355)0.35
2.11:0.03215)1.000.987:0.19224)0.000.983:0.00217)0.715.33:0.00739)0.875.1
62:0.02817,(((UltB3929:0.03629,(UltB3927:0.00346,UltB3928:0.00401)0.918.1
61:0.03244)0.989.107:0.08659,(((((UltB3907:0.02115,((UltAl125:0.01257,(Ul
tAl127:0.04453,Otu00492:0.02002)0.842.128:0.01592)0.805.88:0.01003,(Otu01

733:0.00858,UncUncul:0.04409)0.918.162:0.01654)0.945.108:0.01719)0.590.13
:0.01194,((UltAl126:0.00523,((UltB3903:0.0,UltB3905:0.0):5.5E-
4,UltB3904:0.00369)0.963.114:0.03125)0.943.133:0.03497,(UltB3906:0.05635,
UltB3908:0.03301)0.505.7:0.01659)0.863.156:0.01865)0.811.79:0.01192,(UltB
3901:5.3E-
4,(UltB3900:0.05231,UltB3902:0.02762)0.940.135:0.02451)0.859.130:0.01121)
0.818.72:0.02678,((UltB3899:0.02439,UltRum11:0.05224)0.390.24:0.01089,(Ul
tRum12:0.06262,(UltB3898:0.02171,UltKopri:0.01786)0.859.131:0.01811)0.045
.9:0.00845)0.928.130:0.03206)0.872.144:0.03215,(Ult22622:0.06976,Ult18929
:0.06365)0.690.35:0.02988)0.962.133:0.06306)0.000.984:0.0193,(BfhgggY2:0.
09725,(UltB3953:0.13322,(UdnRume2:0.0097,((UltB3924:0.02794,S0114082:0.06
057)0.921.148:0.03499,(UltB3925:0.02057,UltB3926:0.05415)0.709.34:0.0121)
0.946.126:0.03787)0.922.192:0.05283)0.656.19:0.05238)0.159.7:0.0143)0.974
.86:0.06013)0.657.21:0.0134,((((Kty00000:0.06024,(((UltAl164:0.01132,Otu0
0458:0.00366)0.962.134:0.02544,((((Otu00568:0.04751,((UltAl163:0.00832,Ot
u00278:0.03086)0.879.157:0.00824,(Otu00766:0.01559,UltRum16:0.06585)0.770
.131:0.01323)0.803.79:5.4E-
4)0.596.10:0.01123,Otu00812:0.04508)0.701.30:0.00818,UltB3972:0.01034)0.9
17.133:0.01419,(UltAl165:0.06449,((UltB3973:0.0,Otu00850:0.0):0.02311,Otu
00196:0.05224)0.494.13:0.01452)0.676.17:0.00947)0.722.36:0.00576)0.691.22
:0.01421,(Otu00140:0.02459,((UltAl167:5.5E-
4,(UltAl168:0.0,Otu00851:0.0):5.5E-
4)0.987.146:0.02661,((UltAl166:0.00369,Otu00347:5.5E-
4)0.788.111:0.0083,UltB3974:0.10346)0.846.143:0.01313)0.371.16:0.00833)0.
759.116:0.01631)0.966.146:0.05098)0.992.127:0.08792,((UltAl160:0.0103,(Ul
tAl161:0.01809,Otu00308:0.00967)0.852.135:0.01256)0.941.124:0.02655,((Clv
BvvvB:0.01373,(UltAl159:0.02784,UltAl158:0.00734)0.365.16:0.01019)1.000.9
88:0.09336,((Otu00557:5.4E-
4,(UltAl152:0.0037,(((UltB3963:0.02676,((UltAl155:0.0,Otu00148:0.0):0.031
61,(Otu00165:0.01133,(((ChvBvvvB:0.00731,(UltB3962:0.01123,Otu00034:0.003
7)0.925.158:5.4E-
4)0.957.125:0.01926,(UltAl153:0.00737,(UltAl157:0.02318,((UltAl154:0.0040
2,Otu00071:0.01494)0.487.23:0.00362,UltAl156:0.0151)0.796.80:0.00404)0.91
6.160:5.4E-
4)0.487.24:0.00391)0.917.134:0.0043,Otu00211:0.01825)0.914.163:0.00378)0.
832.101:5.4E-
4)0.915.143:0.01654)0.849.127:0.00805,Otu00187:0.01116)0.964.134:0.02129,
(Otu01006:0.03879,(UltB3961:0.0062,(UltAl151:0.00883,Otu00261:0.02258)0.7
57.89:0.00986)0.778.115:0.00655)0.745.102:0.00577)0.835.105:0.00964)0.845
.133:5.4E-
4)0.789.103:0.00361,UltAl150:0.00379)0.990.125:0.0639)0.716.34:0.00629)0.
721.30:0.03693)0.912.173:0.04468,(UltB3944:0.0706,(MaiMeta8:0.11517,(((Ul
tB3939:5.4E-4,UltB3940:0.01896)0.798.102:5.5E-
4,(UltB3938:0.00363,(UltB3936:0.02296,UltB3937:0.01916)0.747.85:0.00362)0
.795.107:0.00746)0.948.153:0.02947,(UltB3942:0.01166,(UltB3943:0.00706,Ul
tB3941:0.00696)0.892.139:5.4E-
4)0.901.143:0.01807)0.663.25:0.02971)0.870.132:0.03572)0.961.124:0.04606)
0.833.116:0.01434,(UltAnap5:0.2017,(((UltB3897:0.05748,(((Ult14388:0.0,Ul
t14389:0.0,Ult14391:0.0):5.5E-
4,Ult14390:0.00367)0.980.106:0.06272,(((GltSacch:0.01507,((GltXylin:0.0,G
ltXyli2:0.0):0.00456,((GltHanse:0.0,GltMalti:0.0):5.3E-
4,((Udndddde:0.01157,GltRhaet:0.00753)0.877.154:0.00761,(AmeChian:5.5E-
4,GlbOrgan:0.0075)0.782.128:0.00379)0.450.17:0.00372)0.861.161:0.01078)0.
572.14:0.00764)0.853.113:0.00847,((((UltAcet6:0.01927,(AceLovan:5.4E-

4,(((((AcePaste:5.5E-4,(AcetOeni:0.00753,AceEstun:5.5E-4)0.342.16:5.3E-
4)0.974.87:5.5E-4,(AceNitro:0.00748,AceAceti:5.5E-
4)0.932.150:0.00745)0.630.17:0.00373,((UltB3836:0.00743,((UltB3837:0.0073
9,((AceSpec2:0.01917,UltB3838:0.01117)0.000.985:5.5E-4,(AceOrien:5.5E-
4,Otu00143:0.00371)0.853.114:0.0037)0.337.11:5.5E-4)0.000.986:5.5E-
4,(AcePast2:0.0,AcePast3:0.0,AcePast4:0.0):5.5E-4)0.000.987:5.3E-
4)0.313.11:5.5E-4,AceCerev:0.00742)0.574.14:5.4E-4)0.954.108:5.5E-
4,((AcePerox:0.0,AcePast5:0.0,AcePast6:0.0):0.01163,(GlnSpec2:5.4E-
4,(GlnSpeci:0.00194,((AcnMetha:0.02372,AceSpec3:0.00842)0.956.129:0.02352
,(((GlnFrate:0.0,GlnJapon:0.0):0.00739,(GlnCeri2:5.5E-
4,GlnCerin:0.00748)0.802.99:0.00387)0.931.151:0.01149,((UltAcet2:0.00742,
(ScrFlori:0.01523,(UltAceto:0.0076,(UltFirmi:0.00369,(UltAl123:5.5E-
4,UltAl124:5.5E-4)0.932.151:5.5E-
4)0.915.144:0.01126)0.764.126:0.00381)0.878.139:0.00752)0.869.122:0.00769
,((UltAcet3:0.00383,UltAcet4:0.01139)0.967.100:0.02008,UltAcet5:0.0369)0.
741.72:0.00329)0.798.103:0.00388)0.867.143:5.4E-
4)0.948.154:0.01512)0.922.193:0.00193)0.981.111:0.02422)0.822.85:0.00721)
0.928.131:0.01165,(AceFabar:0.0,AceLova2:0.0):5.4E-
4)0.782.129:0.00383)0.868.126:0.00787)0.875.163:0.00823,((UltAcet8:0.0,Ul
tB3839:0.0):0.02862,GrnBethe:0.01631)0.859.132:0.01143)0.899.143:0.00203,
(AsaKrung:0.00751,(AsaSiame:0.0037,Udntfde6:5.4E-4)0.071.10:5.5E-
4)0.930.136:0.01096)0.911.173:0.00188,(((((GltLique:0.00763,(GltSacc2:0.0
,GltSacc3:0.0):0.00763)0.784.116:0.00373,GltDiazo:5.4E-
4)0.767.107:0.00361,KozBalie:0.00385)0.308.12:0.01113,(CmlIntes:0.02973,(
UltGluco:0.01816,(UltAcet7:5.5E-
4,UltKozak:0.023)0.843.114:0.00854)0.732.42:0.01483)0.951.136:0.02673)0.7
04.30:5.4E-4,Otu00613:0.01915)0.810.87:0.00372)0.668.30:5.4E-
4)0.842.129:0.00957,UdnBac18:0.04728)0.941.125:0.05467)0.922.194:0.04344)
0.878.140:0.03277,(((UltB3832:5.4E-
4,(Ult16788:0.01876,(Ult16786:0.00715,(Ult16785:0.01557,Ult16787:0.01953)
0.906.162:0.01261)0.475.17:0.01792)1.000.989:0.10593)0.511.14:0.01579,(((
(UltAl119:0.05785,(AphPro47:0.00878,(UltB3820:0.06378,KorGwang:0.00732)0.
577.10:0.00699)0.851.142:0.01298)0.991.124:0.03567,(((UltAl178:0.07158,(U
ltAl122:0.02201,((UltB3561:0.0,SdnBact2:0.0):0.04869,(UltParv2:0.0037,Prb
Lavam:5.5E-4)0.999.212:5.4E-
4)0.987.147:0.05284)0.374.22:0.02506)0.810.88:0.01596,(UltB3833:0.04588,(
UltAl120:0.03642,UltAl121:0.00723)0.887.148:0.01536)0.889.145:0.01249)0.7
19.34:5.4E-
4,(RhhSalex:0.03394,(UltB3819:0.06028,((UltB3979:0.08888,(SntGloss:5.4E-
4,(UltB3835:0.01634,SntChine:0.02454)0.284.12:0.00677)0.917.135:0.03088)0
.922.195:0.03665,UltB3821:0.01936)0.804.82:0.00927)0.918.163:0.01818)0.92
4.144:0.02631)0.536.15:0.00797)0.681.20:5.4E-
4,(((UltB3826:0.0394,(((PvlBermu:0.03067,PvlLutao:0.01304)0.699.34:0.0035
7,((UltRhi23:0.02832,UltSlu12:0.00247)0.985.119:0.03296,((UltParvu:0.0,Ul
tPhaeo:0.0):0.01903,(UltAlp70:0.01885,UltAlp71:5.4E-
4)0.893.156:0.0077)0.732.43:0.00386)0.883.153:0.01563)0.983.104:0.05713,U
ltB4082:0.10371)0.802.100:0.01737)0.817.80:0.02318,((UltAlp64:0.03563,(((
Ult31169:0.10739,((UltB3541:0.02282,MegPerid:0.00776)0.904.154:0.03387,(U
ltPro20:0.01859,(UltSlu11:0.07369,UltAlp68:0.00614)0.768.99:0.01656)0.997
.150:0.05975)0.689.26:0.0321)0.739.64:0.00627,(MaiMeta4:0.01646,(RbrClift
:0.01969,UltAlp61:0.01529)0.768.100:0.00971)0.970.122:0.03053)0.784.117:0
.00688,(((((RhvTherm:0.00746,(((RhvSulfi:0.00369,((RhvSpeci:0.0,RhvSulf2:
0.0):5.5E-
4,((TclPacif:0.00745,(((((PhlCata2:0.0,PhlCatac:0.0):0.00755,(PhlLegum:5.

5E-4,PhlSpec4:5.5E-
4)0.790.105:0.00396)0.792.117:0.00405,((CohGelat:0.00367,UltB3056:5.4E-
4)0.984.114:0.01895,(UltB3080:0.00379,(((PleOryza:0.0,UltB3510:0.0):0.003
7,PleKoree:5.5E-
4)0.933.159:0.01139,SdnBacte:0.00751)0.875.164:0.00759)0.908.164:0.0075)0
.808.97:5.4E-
4)0.344.16:0.00824,((UltB3553:0.01497,((PhlTrifo:0.0,PhlSpec2:0.0,PhlSpec
i:0.0,PhlSpec3:0.0,PhlMyrs2:0.0,PhlMyrsi:0.0):5.5E-
4,(UltAlph5:0.00369,UltMeso2:0.00371)0.466.15:5.5E-4)0.321.11:5.4E-
4)0.913.160:7.3E-4,UltBrad2:0.01493)1.000.990:0.00288)0.998.193:5.5E-
4,(UltRhi14:0.00782,(UltMesor:5.3E-
4,UltB3074:0.00368)0.881.155:0.0076)0.783.129:0.00374)0.763.130:0.00415)0
.899.144:0.0083,((RhvStric:0.00372,RhvSpec2:5.5E-
4)0.803.80:0.00375,UltB3058:0.01898)0.771.134:0.00366)0.438.21:5.3E-
4)0.267.15:5.4E-4)1.000.991:5.5E-4,(((((AphProt8:5.4E-
4,UltB3143:0.00742)0.976.109:5.3E-
4,((((((RhrBac12:0.00369,(OccNanha:5.5E-
4,OccSpeci:0.01492)0.824.86:0.00369)0.430.20:5.5E-
4,(LtmSaema:0.0,OccPacif:0.0):5.5E-4)1.000.992:5.5E-
4,(UltMar31:0.0074,(OccMarin:5.5E-4,UltOrg24:0.01137)0.879.158:5.3E-
4)0.823.99:0.00741)0.748.88:0.00357,((OccBats2:0.0,OccBatse:0.0,TrpIsoal:
0.0,AphPro16:0.0):0.00361,PdgSpeci:5.4E-
4)0.296.12:0.00369)1.000.993:5.5E-
4,(UltB3166:0.0,UltB3168:0.0,UltB3142:0.0,JnnSeosi:0.0,PdgAquim:0.0):5.5E
-4)0.752.92:5.5E-
4,UltB3167:0.00369)0.834.101:0.00366)0.413.21:0.00369,(UltAlp46:0.0,UltAl
p51:0.0):0.00742)0.926.166:5.5E-4,RhvIodos:5.5E-4)0.786.105:5.4E-
4,UltAlp56:0.00369)0.820.84:0.00361)0.198.13:0.00838,(((((AphPro19:0.0079
9,RhbMaris:0.01315)0.909.132:0.0136,(BacteSL2:5.5E-
4,UltB3270:0.01101)0.650.21:0.01171)0.878.141:0.0116,UltB3235:0.00784)0.7
73.127:0.00334,((RslMahon:0.0076,WenMarin:0.01547)0.774.126:0.00371,((Jnn
Seoha:0.00371,(RhbAestu:0.00369,(JnnRubr2:0.0,JnnRubra:0.0):5.5E-
4)0.825.92:5.5E-
4)0.903.129:0.00752,UltAlp47:0.00374)0.791.103:0.00374)0.824.87:5.5E-
4)0.877.155:0.00748,((((((RhrBac10:0.0,RgrSpec4:0.0,MvtLitor:0.0,UltRose4
:0.0):5.3E-
4,UltAlp14:0.02281)0.951.137:0.01138,UltB3187:0.00375)0.796.81:0.00363,Ul
tB3185:5.4E-
4)0.739.65:0.00547,UltRho31:0.036)0.897.142:0.01432,(UltB3188:0.00716,(((
UltB3228:0.12686,UltRhi15:0.39258)0.928.132:0.05903,(UltB3251:0.06606,(((
UltB3825:0.04809,AmaTamwo:0.00589)0.900.126:0.01689,((CanEndow:0.04929,((
(((MesAlbi2:0.0,MesAlbiz:0.0):0.01102,(((UltB3063:0.00377,(UltB3064:0.019
16,MesSpec3:0.00376)0.776.115:0.00363)0.784.118:0.00381,(AphProt4:0.00763
,(PrhSpeci:5.5E-
4,((RhzBacte:0.0,UltB3068:0.0,UltB3069:0.0,UltB3070:0.0,MesSpec6:0.0,MesT
arim:0.0,MesMedi2:0.0):5.5E-
4,MesMedit:0.00369)0.817.81:0.00368)0.888.154:0.00755)0.874.162:0.00785)0
.789.104:0.00368,UltAlph4:0.01567)0.897.143:5.3E-
4)0.933.160:0.0084,UltB3264:0.03418)0.888.155:0.00854,(((UltB3066:0.0,Ult
B3078:0.0):5.5E-4,((UltB3060:0.00744,(UltrSo28:0.00741,MesAlhag:5.5E-
4)0.082.12:5.5E-
4)0.797.93:0.00373,(BacRBS36:0.00243,(UltOrg18:0.01137,((AntBac21:5.5E-
4,UltB3059:0.00369)0.876.127:0.0037,((HoePhoto:0.0,AhnSpeci:0.0,MesSpeci:
0.0):5.5E-4,(AphProt3:0.00747,HoeMarin:0.00369)0.776.116:5.5E-

4)0.853.115:5.1E-
4)0.901.144:0.00759)0.922.196:0.00247)0.922.197:0.00244)0.915.145:0.00751
)0.919.178:0.00742,((AmiAmino:0.0,RhiSpe42:0.0,MesSpec4:0.0):5.5E-
4,((UltB3062:0.00742,MesSpec5:5.5E-
4)0.926.167:0.00746,AmiLissa:0.00369)0.709.35:5.4E-4)0.914.164:5.4E-
4)0.540.19:5.5E-4)0.838.122:0.00372,AmiNiiga:5.5E-
4)0.834.102:0.00992)0.802.101:0.00874,(((NttBasal:0.01121,(AqrSpec2:5.3E-
4,(((MesSpec8:0.0,NttKimny:0.0,UltB3071:0.0,UltB3072:0.0,UltB3075:0.0):0.
00391,(AqrDeflu:5.5E-
4,((MesSpec2:0.0,AqrSpeci:0.0):0.0037,(DefLusat:5.5E-
4,UltB3067:0.00369)0.879.159:5.4E-
4)0.863.157:0.00735)0.205.12:0.01136)0.947.119:0.01904,(PdbDeflu:0.0115,A
phProt2:0.0115)0.212.11:0.00363)0.953.110:5.4E-
4)0.826.90:0.0037)0.795.108:5.5E-
4,(UltSlud7:0.01396,((((UltB3818:0.01959,(UltB3288:0.03021,((UltRhi31:5.5
E-
4,(MaiMeta6:0.01945,UltB3823:0.0234)0.957.126:0.02337)0.969.107:0.0198,((
(UltFores:0.02356,(UltAlp90:0.03494,UltB3824:5.5E-
4)0.953.111:0.01963)0.925.159:0.01965,UltB4070:5.4E-
4)0.811.80:0.00719,UltB3289:0.0155)0.751.81:0.00315)0.778.116:0.00573)0.4
75.18:0.01476)0.809.98:0.00727,(UltB3280:0.04711,UltMar32:5.4E-
4)0.261.10:0.01116)0.851.143:0.00746,(RhrBac14:0.01635,UltOrg26:0.04751)0
.853.116:0.01579)0.862.147:0.01012,UltB3447:0.01675)0.885.144:0.0115)0.79
1.104:0.00499)0.981.112:5.4E-4,(((MaiBact4:0.0,AhnKiele:0.0):5.3E-
4,(UltB3055:5.5E-4,UltB3073:0.02288)0.917.136:0.00745)0.059.14:5.5E-
4,UltAlp44:0.01893)0.965.114:0.01493)0.384.17:0.00459)0.519.11:0.00757)0.
775.114:0.0043,((UltB3180:0.01879,(((((((UltB3227:0.0,RhbBlast:0.0,UltB3
225:0.0):5.5E-4,BacEnr12:0.00369)0.939.127:5.5E-
4,(RblTherm:0.01144,UltB3126:0.0074)0.991.125:0.02737)0.450.18:0.00881,((
(((UltB3242:0.00369,((((CatNecta:0.0,RhbCaps2:0.0):5.5E-
4,((UltRho37:0.00743,UltB3220:5.4E-
4)0.676.18:0.00369,RhbSpec5:0.01124)0.882.162:5.5E-4)0.810.89:5.4E-
4,RhbCapsu:0.00371)0.927.135:0.00211,((AphPro20:0.0,UltB3244:0.0):5.5E-
4,UltB3014:0.03489)0.896.136:0.00693)0.923.194:0.00206)0.405.24:5.4E-
4,UltB3243:5.4E-4)0.938.102:5.4E-
4,(AntBac25:0.00375,(UltSlud8:0.00743,((((UltB3229:0.0,UltB3231:0.0):5.5E
-4,(Ult27547:0.00369,UltB3230:0.00369)0.644.22:5.5E-4)0.944.117:5.4E-
4,(UltB3258:5.4E-4,((UltAlp57:5.4E-
4,HmtMassi:0.0037)0.881.156:0.00362,((UltB3224:5.5E-
4,(((UltB3215:0.00381,UltB3222:0.01125)0.672.22:0.00761,(UltB3216:5.5E-
4,UltB3221:0.00746)0.893.157:0.00351)1.000.994:5.4E-
4,RhbSpec4:0.01124)0.629.25:5.5E-4)1.000.995:5.5E-
4,((UltB3219:0.00738,(UltB3218:0.00769,(((UltB3239:0.00673,UltB3233:0.012
96)0.938.103:0.01873,UltB3237:0.0051)0.798.104:0.00576,UltB3236:0.00717)0
.949.133:5.3E-4)0.783.130:0.01549)0.761.101:5.4E-
4,(RhbApigm:0.01121,AntBac24:5.4E-
4)0.944.118:0.00749)0.841.115:0.00361)0.415.16:0.00368)1.000.996:5.5E-
4)0.837.98:0.00371)0.861.162:0.00369,(RhbSpha3:0.0,RhbSpha4:0.0,RhbSpha5:
0.0):0.01519)0.021.7:5.4E-
4)0.810.90:0.0037)0.815.81:0.00371)0.968.112:0.01121,(UltB3238:0.0037,(Ul
tB3240:0.0114,UltRho36:0.00749)0.764.127:0.00363)0.924.145:5.5E-
4)0.790.106:0.00447,(UltB3266:0.02277,((UltB3265:0.00369,(UltAmari:5.4E-
4,((UltB3267:0.00798,RhvSpec3:0.02724)0.840.130:0.0111,FlvPelag:0.05865)0
.471.20:0.01681)0.901.145:0.00721)0.413.22:5.4E-4,AmaKapli:5.5E-

4)0.895.156:5.4E-
4)0.947.120:0.01856)0.482.21:0.00746)0.831.103:0.00669,(UltB3207:0.00355,
UltB3208:0.01524)0.924.146:0.01194)0.882.163:0.00783,((UltB3129:0.01609,(
(UltB3127:0.00342,UltB3128:5.5E-
4)0.932.152:0.02987,(RblAerol:0.00869,RblMesop:0.0063)0.113.12:0.01433)0.
760.113:0.00632)0.961.125:0.02238,((((PccKocur:0.0074,(PccAlcal:5.5E-
4,UltB3211:0.00369)0.939.128:5.4E-4)0.721.31:0.00751,(PccKoree:5.4E-
4,(PccSpe16:0.0037,UltB3210:0.0037)0.890.151:0.00746)0.790.107:0.00368)0.
932.153:5.4E-4,((PonLitor:5.5E-4,((AmaMacau:5.4E-
4,(UltB3259:0.00388,AmaVeron:0.0149)0.798.105:0.00368)0.979.124:0.015,Lok
Atril:5.4E-4)0.402.17:0.01105)0.818.73:0.01163,(UltB3130:5.5E-
4,AphPro17:0.00369)0.833.117:0.00723)0.641.18:0.00731)0.798.106:0.00846,(
UltRho32:0.00751,UltB3176:0.01508)0.718.39:0.00244)0.635.12:0.01244)0.706
.35:0.00363)0.946.127:0.01599,(((((((((IddBact2:0.00369,(RhrBact3:0.0036
9,RsvMucos:0.00369)0.328.18:5.5E-4)0.747.86:5.4E-
4,((((((((LokHong2:0.0,LokHongk:0.0,LokHong3:0.0):5.5E-
4,DSMBacte:0.00742)0.826.91:0.00371,((UltRho24:5.5E-
4,UltRho33:0.00368)0.787.110:0.00379,UltB3122:0.01911)0.789.105:0.0036)0.
774.127:0.00371,(LokSpeci:5.4E-4,(((RhrBac13:0.00741,(AphPro18:5.5E-
4,(DonEburn:0.00751,RsxHalod:5.4E-4)0.849.128:0.00369)0.602.11:5.4E-
4)0.845.134:0.0037,TlsHalod:5.5E-
4)0.837.99:0.0037,(UltAlp16:0.00368,(RgrLacus:0.0,SlbLacus:0.0):0.00752)0
.804.83:0.00375)0.752.93:5.3E-4)0.749.102:5.5E-4)0.804.84:5.3E-
4,((SlbSpec2:0.0,UltAlp18:0.0,RsvPacif:0.0):5.5E-
4,(RgrSpec2:0.05924,(UltAlp17:5.5E-
4,(UltB3139:0.00369,UltB3137:0.0037)0.747.87:5.5E-4)1.000.997:5.5E-
4)0.838.123:0.00368)0.928.133:0.00753)0.832.102:0.0037,(UltRho25:0.00751,
(UltB3147:5.5E-
4,UltB3194:0.0037)0.745.103:0.00374)0.886.176:0.0075)0.999.213:5.5E-
4,((UltOrg22:5.4E-4,(BacL1882:5.4E-4,(UltAlp40:0.00369,UltB3175:5.5E-
4)0.876.128:0.00369)0.862.148:0.00371)0.858.139:5.5E-
4,(UltAlp41:0.00366,UltB3158:0.01533)0.888.156:0.00751)0.704.31:0.00743)0
.800.87:0.00364,((UltAlp26:5.5E-4,(UltB3162:0.00369,UltB3157:5.5E-
4)0.866.149:0.00369)0.837.100:0.00369,((((PbcSpec2:0.0,UltB3192:0.0):5.5E
-4,(PbcDaep2:0.00369,(RhrBact6:0.0,UltB3196:0.0):0.00369)0.716.35:5.4E-
4)0.957.127:5.5E-4,(PbcDaepo:0.01118,(((RsbSpec3:0.0,UltB3148:0.0):5.5E-
4,(UltSalip:0.0037,(SlgMuco2:0.0,SlgMucos:0.0):5.5E-
4)0.872.145:0.0037)0.767.108:0.00378,UltB3149:0.01122)0.510.10:0.00752)0.
991.126:5.5E-4)0.840.131:0.00369,UltRoseo:0.01905)1.000.998:5.4E-
4)0.287.9:5.5E-4)0.859.133:0.00368,RhrBact5:0.0037)0.159.8:5.5E-
4,UltAlp45:0.01118)0.918.164:0.00743)0.827.105:0.00368,RsvHalot:5.3E-
4)0.292.8:5.5E-
4,((UltOrg20:0.01904,((SgtStel2:0.0,SgtStell:0.0,RsbSpec4:0.0,RgrMobil:0.
0,SlbSpeci:0.0,RgrAtlan:0.0,RhrBact2:0.0,RgrScott:0.0):5.5E-
4,RgrSpeci:0.00369)0.902.147:5.4E-
4)0.436.16:0.00369,((RgrAtla2:0.0,AphProt7:0.0):5.5E-4,RgrAtla3:5.5E-
4)0.958.152:5.4E-
4)0.942.140:0.00748)0.790.108:0.00373,(((UltAlph9:0.0,LokSalsi:0.0,LokSal
s2:0.0):5.4E-
4,LokAgnit:0.00742)0.789.106:0.00379,LokFryxe:0.0196)0.795.109:0.00365)0.
794.97:0.00373,(RhvMarin:5.5E-
4,RhvEuryh:0.00372)0.832.103:0.00369)0.338.8:5.4E-
4,(UltSinor:0.04501,((MhrTerri:0.00753,((((((((RdbBargu:0.0,RdbBogor:0.0
,UltAlp53:0.0):5.4E-4,RbcEkhon:0.0037)0.930.137:0.00747,(SdmQiaoh:5.5E-

4,((BacEnr11:5.5E-4,((RsxHalo2:0.0,RsxHalot:0.0):5.5E-
4,BacEnr10:0.00369)0.853.117:5.5E-
4)0.980.107:0.01912,UltPro34:0.04302)0.945.109:5.4E-
4)0.908.165:0.00744)0.768.101:5.4E-
4,UltAlp55:0.00744)0.888.157:0.00742,UltB3214:0.02692)0.765.111:5.4E-
4,BacCYCU3:5.5E-4)0.942.141:0.00741,(UltB3198:5.4E-
4,UltAlp54:0.02298)0.818.74:0.00369)0.689.27:5.3E-
4,((PccSulf2:0.0,PccSulfu:0.0):0.01124,PccSpec3:0.0037)0.889.146:5.5E-
4)0.930.138:5.5E-4,((((((PccSalip:0.00753,PccSpe18:0.00743)0.447.15:5.4E-
4,PccSpe15:0.00369)0.860.119:0.00369,(PccSerin:0.00369,(PccZeax2:0.0,PccZ
eaxa:0.0,PccHomie:0.0,UltB3205:0.0):5.5E-
4)0.911.174:0.00741)0.115.7:5.5E-4,(PccSpec2:0.01506,(RhbSpeci:5.3E-
4,RhbSpec2:0.00743)0.373.20:0.00369)0.958.153:5.4E-
4)0.911.175:0.00369,((PccSpe10:0.0,PccAestu:0.0,PccSpe14:0.0,PccMarin:0.0
):5.5E-
4,(UltB3201:0.00368,((MhrMarin:0.01877,UltSlu10:0.01865)0.770.132:0.00495
,(UltB3252:0.0,UltB3262:0.0):0.00369)0.281.8:5.5E-4)0.874.163:5.5E-
4)0.904.155:5.5E-4)0.839.117:5.4E-4,(AbdInexp:5.4E-
4,((((((UltB3226:0.0,UltSlud9:0.0):5.5E-
4,UltB3261:0.00366)0.875.165:0.0077,(((PccSpe13:5.5E-
4,((PccThioc:0.00758,(((PccSpec8:0.0,PccDenit:0.0,PccSpe17:0.0):5.5E-
4,(PccVers2:0.01865,(PccSpe19:0.00741,(PccBenga:0.0,PccVersu:0.0,PccSpec9
:0.0,PccDeni2:0.0,PccDeni3:0.0):5.5E-4)0.917.137:5.5E-
4)0.821.87:0.00366)0.807.78:0.0037,(((UltB3257:0.01121,UltB3203:0.00743)0
.761.102:5.5E-
4,(PccSpec5:0.0,PccAmin4:0.0,PccSpec7:0.0,PccHalop:0.0,UltPro35:0.0,PccSp
e20:0.0):5.5E-4)0.773.128:5.4E-
4,((UltB3202:0.00747,((PccSolve:0.0,PccSolv2:0.0):0.00752,PccAlken:5.4E-
4)0.798.107:0.00367)0.795.110:0.00374,UltB3209:5.5E-
4)0.838.124:0.0037)0.805.89:0.00375)0.801.97:0.00372)0.999.214:5.5E-
4,(PccSpe12:0.00744,UltB3206:5.4E-
4)0.174.10:0.0037)0.814.80:0.00363)0.759.117:0.00438,UltB3253:0.01436)0.5
54.19:0.00376,(UltB3234:0.0,UltB3232:0.0):0.01063)0.892.140:0.00829)0.769
.114:0.00361,CatSpeci:5.3E-4)0.897.144:5.4E-
4,((RhvKhole:0.0,RhbSpec6:0.0):0.0037,(RhbSpec7:5.5E-
4,(RhbVinay:0.00369,RhbVeldk:0.00745)0.756.111:5.5E-4)0.790.109:5.5E-
4)0.826.92:0.00716)0.412.18:0.0037,((RhbSpec8:5.5E-
4,UltB3248:0.00369)0.338.9:5.3E-4,(UltParac:0.0037,UltB3250:5.5E-
4)0.855.141:0.0037)0.790.110:0.0034)0.790.111:0.00398)0.900.127:0.0074)0.
826.93:0.00366)0.776.117:0.0037)0.805.90:0.00359,(UltB3249:5.5E-
4,(UltB3260:0.00369,(((((UltB3217:0.0,UltB3255:0.0,UltB3256:0.0):0.01403,
(UltB3241:0.01232,(RhbSpec3:0.00352,((PcccYeei:0.0,UltB3204:0.0):0.00753,
((PccCarot:0.0,PccSpeci:0.0):5.5E-
4,((UltB3245:0.0,UltB3246:0.0):0.00369,PccHaeun:0.00369)0.675.19:5.5E-
4)0.912.174:5.4E-4)1.000.999:5.5E-
4)0.771.135:0.01021)0.325.15:0.00396)0.782.130:0.00479,UltB3247:5.4E-
4)0.836.98:0.00369,((((UltB3199:0.0,PccAmino:0.0,PccSpec4:0.0,UltB3212:0.
0):5.5E-4,(PccSpec6:5.5E-
4,PccAmin3:0.01548)0.913.161:0.00765)0.000.988:5.5E-4,PccAmin2:5.5E-
4)0.520.10:5.5E-4,(UltB3200:0.00369,UltAlp58:5.5E-
4)0.868.127:0.00369)0.344.17:5.4E-4)0.779.113:0.00396,(UltRho35:5.4E-
4,(UltRho34:5.5E-4,((ProSW219:5.4E-4,(UltB3254:0.0117,(UdnBac16:5.4E-
4,UltB3223:0.00703)0.324.10:0.00373)0.837.101:0.00703)0.856.121:0.00733,R
hbChang:0.00768)0.445.20:0.00378)0.953.112:0.01096)0.907.156:0.0113)0.803

.81:0.00358)0.357.15:5.5E-4)1.000.1000:5.5E-
4)0.323.15:0.00796)0.835.106:0.00736)0.771.136:0.00381,AbmDongh:0.01904)0
.964.135:0.00192,UltAmar2:0.01897)0.913.162:0.00172,UltAmar3:0.01525)0.92
6.168:0.01183)0.778.117:0.00365,UltB3197:0.00746)0.875.166:0.01136)0.873.
137:5.4E-
4,(((UltB3174:0.00742,UltB9261:0.04855)0.865.145:0.00758,(((((UltB3161:0.
0,UltB3164:0.0):5.5E-
4,(UltAlp31:0.00369,(UltAlp48:0.01129,(UltAlp37:0.00369,(UltAlp49:0.00749
,(RsbSpec7:0.00743,(ArcSeaw2:0.0,UltB3191:0.0):5.3E-
4)0.810.91:0.00367)0.799.105:0.00374)0.407.20:5.5E-
4)0.927.136:0.00742)0.895.157:5.5E-4)0.868.128:5.4E-
4,UltAlp36:0.00367)0.912.175:0.00737,(((((UltB3169:0.01511,(AphProt6:0.0,
UltB3124:0.0):0.01527)0.916.161:0.01147,(UltMar30:0.0,UltRho29:0.0):5.3E-
4)0.910.141:0.00742,((UltB3171:0.0037,(LokKoree:0.01122,(UltB3173:0.01123
,UltB3172:0.00376)0.482.22:0.00371)0.873.138:5.5E-
4)0.919.179:0.00751,(((KetVulg2:5.5E-
4,(UltB3131:0.02299,KetVulga:0.00369)0.996.140:5.5E-
4)0.803.82:0.00588,UltB3183:0.01443)0.769.115:0.0059,(((RhrBact4:5.4E-
4,(UltB3134:0.0037,((((((((SltSpec6:5.5E-
4,(UltB3153:0.00369,SltSpec3:0.00743)0.703.37:5.5E-4)0.757.90:5.4E-
4,(UltSulf4:5.5E-4,((UltSulfi:0.0,UltSulf3:0.0):5.5E-4,UltSulf2:5.5E-
4)0.434.17:5.5E-
4)0.918.165:0.00743)0.944.119:0.00495,(((SltSpec2:0.0,UltAlp23:0.0,UltAlp
25:0.0,AphProt9:0.0,UltAlp27:0.0,SltDongh:0.0):5.5E-
4,SltMedit:0.00369)0.909.133:5.5E-4,(SltSpec7:0.00752,(((UdnBac15:5.4E-
4,(ArctiSe3:5.4E-
4,UltAlp28:0.00369)0.925.160:0.00743)0.944.120:0.00495,(UltB3125:0.00745,
((RsbDenit:5.5E-4,RsbDeni2:5.5E-
4)0.923.195:0.00737,((SltLitor:0.0,UdnBac14:0.0,SltSpeci:0.0,CfSulfit:0.0
):5.5E-4,AphPro10:0.0037)0.294.11:5.4E-
4)0.901.146:0.00742)0.944.121:0.0049)0.944.122:0.0049,(MaiBact7:0.0,AntSe
aw2:0.0,SltSpec4:0.0):5.4E-
4)0.892.141:0.00744)0.886.177:0.00743)0.944.123:0.00491)0.944.124:0.0049,
(OclIndol:0.0,OclIndo2:0.0,OclIndo3:0.0):5.5E-
4)0.938.104:0.00739,((((UltOrg21:0.0112,PllLitor:5.5E-
4)0.838.125:0.00369,((UltB3152:0.0,RsbSpec5:0.0,UltB3145:0.0):5.5E-
4,UltB3135:0.00369)0.983.105:5.3E-
4)0.918.166:0.00743,UltStale:0.00387)0.581.16:5.4E-
4,(SltGutti:0.0,SltSpec5:0.0):5.5E-4)0.590.14:5.4E-
4)0.921.149:0.00776,((MaiBact6:5.5E-
4,UltB3136:0.00369)0.944.125:0.00197,(UltB3133:0.00369,(UltB3151:0.01893,
(RsbLitor:0.0,RsbLito2:0.0):0.00742)0.120.12:5.5E-
4)0.944.126:0.00194)0.967.101:0.01481)0.598.12:5.5E-
4,(UltAlp15:0.0,RsbSpec6:0.0):5.4E-4)0.449.28:5.4E-4,((MaiBact8:5.5E-
4,(UltAnta6:0.0,AnrHelio:0.0):5.5E-
4)0.985.120:0.01511,((MaiAlpha:0.0,LeiAquim:0.0):5.5E-
4,LeiMethy:0.00369)0.996.141:5.4E-
4)0.929.138:0.00745)0.920.165:0.00777)0.521.10:5.3E-4)0.668.31:5.4E-
4,(((UltRho27:5.5E-4,(((YanPacif:0.00366,(RsvSpeci:5.5E-4,SlgSpeci:5.5E-
4)0.901.147:5.5E-4)0.900.128:5.5E-4,((RsvNubi2:0.0,RsvNubin:0.0):5.5E-
4,UltAlp50:0.00369)0.964.136:0.01124)0.808.98:0.00736,((CtrThioo:0.0,RsbS
pec2:0.0):5.5E-4,UltB3195:0.00741)1.000.1001:5.5E-
4)0.828.86:0.00364)0.898.156:0.00366,((PlgSpeci:0.0,PbcGalla:0.0,PbcSpeci
:0.0,MrvAlgic:0.0,MrvAlgi2:0.0,RgrSpec3:0.0,MrvAlgi3:0.0):5.5E-

4,(((UltAlp19:5.5E-4,(TroNapht:5.5E-4,(UltB3138:5.4E-
4,(UltAlp29:0.0037,((RgrPome2:0.0,RgrPomer:0.0,DonXiame:0.0,CtmSalin:0.0)
:5.5E-4,UltAlp20:5.5E-4)0.779.114:5.5E-
4)0.850.152:0.0037)0.858.140:0.00369)0.867.144:0.00371)0.909.134:0.00208,
(UltB3146:0.00741,(RsvToler:0.00741,IddBacte:5.5E-4)0.337.12:5.5E-
4)0.891.117:0.00694)0.911.176:0.00208,(PbcGall2:0.0,UltRose3:0.0):0.00369
)0.702.26:5.3E-4)0.918.167:5.4E-
4)0.919.180:0.00739,((UltB3132:0.00369,(TlbSpeci:5.4E-
4,TssSpeci:0.019)0.900.129:0.00743)0.732.44:5.4E-
4,(SltGutt2:0.0,PbcInhib:0.0,PbcGall3:0.0,PbcGall4:0.0):5.5E-
4)0.970.123:5.4E-
4)0.914.165:0.00738)0.928.134:0.0078,(UnwMarin:0.01508,(((UltB3179:0.0074
,((((UltRose2:0.01074,(RhrBacte:0.0,UltRho26:0.0,UltRuege:0.0):0.00845)0.
572.15:0.00752,(UltAlp30:0.00722,UltRho30:0.02181)0.836.99:5.5E-
4)0.902.148:0.00825,UltAlp22:5.5E-4)0.872.146:5.5E-
4,((UltRho23:0.0071,(((UltB3120:0.0,LokVestf:0.0):5.5E-
4,LokSpec2:0.00369)0.955.111:0.01138,LokMaric:0.00755)0.637.16:5.4E-
4)0.453.17:0.00583,((((LokRosea:0.0,UltAlp10:0.0):5.5E-
4,(SltPonti:0.00369,(UltB3150:0.0,UltMar29:0.0):5.5E-
4)0.926.169:0.00742)0.852.136:5.5E-4,((UltAlp12:5.4E-
4,UltAlp13:0.00743)0.882.164:0.00745,RhlBact2:0.01508)0.756.112:0.00371)0
.742.68:0.00327,UltAlp24:0.07134)0.154.8:0.00698)0.965.115:0.01803)0.546.
14:0.01489)0.945.110:5.5E-
4,RsvAestu:0.00741)0.899.145:0.00717,(((SltSpec8:0.01542,TltArena:0.01539
)0.904.156:0.0113,AphPro14:0.00756)0.872.147:0.00753,(UltB3177:0.02271,((
(((AphPro13:0.0,NerIgnav:0.0,NerIgna2:0.0):0.00369,(((UltAlp34:5.5E-
4,(OctSpec2:0.0,AphPro11:0.0,AphPro12:0.0):5.5E-4)0.982.101:5.5E-
4,((UltAlp38:0.0,UltB3189:0.0):5.3E-
4,RhlBacte:0.01505)0.916.162:0.00742)0.345.14:5.4E-
4,UltAlp60:0.03517)0.922.198:0.01118)1.000.1002:5.5E-
4,(UltB3165:0.0,UltOrg23:0.0):0.00746)0.797.94:0.00369,(UltAlp35:5.4E-
4,UltAlp33:0.03095)0.867.145:0.00716)0.840.132:5.5E-
4,((UltB3163:0.0,UltAlp43:0.0,UltAlp42:0.0):5.5E-
4,UltB3178:0.01124)0.776.118:0.01127)0.951.138:0.01504)0.400.17:5.3E-
4)0.919.181:0.00716)0.905.149:5.5E-4)0.907.157:0.00748)0.098.3:5.4E-
4)0.929.139:0.00773)0.469.16:5.4E-4)0.885.145:5.4E-
4,(UltB3121:0.00369,((UltB3154:0.0,UltB3155:0.0):5.5E-
4,UltB3156:0.00369)0.955.112:5.5E-
4)0.964.137:0.01502)0.916.163:0.01147,(UltArct2:0.00745,(((UltB3144:0.0,T
lbMedit:0.0):0.00742,((((((UltB3184:5.5E-4,((RstElong:5.5E-
4,((OccGran2:0.0,OccGranu:0.0):5.5E-
4,UltB3190:0.01501)1.000.1003:0.00219)1.000.1004:0.00149,MbsSalin:0.00741
)0.214.15:5.3E-4)1.000.1005:5.4E-
4,RhrBact9:0.00363)0.426.27:0.00369,RsbSpec8:0.00363)1.000.1006:5.1E-
4,((UltAlp32:0.0,RhrBact8:0.0):5.5E-
4,UltRho28:0.0037)0.867.146:0.00369)0.349.14:5.5E-
4,(UltAlp39:0.0,UltB3141:0.0,RsvCrass:0.0):5.4E-4)1.000.1007:5.3E-
4,((UltB3170:0.00751,(UltB3744:0.00752,(UltB3182:5.5E-
4,(UltB3181:0.0,UltAlp11:0.0,UltB3123:0.0):0.01917)0.791.105:0.00368)0.93
4.128:0.01141)0.871.149:0.00744,MmbAlkal:5.5E-
4)0.908.166:0.00741)0.330.15:0.00742)1.000.1008:5.1E-4,NauItali:5.5E-
4)0.793.105:0.0037)0.786.106:0.0037)0.116.11:5.4E-4)0.699.35:5.5E-
4,((TlbGelat:0.0,TlbGela2:0.0,TlbGela3:0.0):5.4E-
4,((((OctArcti:0.0,OctArct2:0.0):0.00369,OctSpeci:5.5E-4)0.956.130:5.3E-

4,(OctAntar:5.5E-
4,RsbSpeci:0.00742)0.763.131:0.00744)0.906.163:0.00986,((UltAlp52:0.00745
,(((((JnnHelgo:0.0,JnnPohan:0.0):0.01109,(TlbAestu:5.4E-
4,((UltShimi:0.01504,(RhrBact7:5.5E-4,(UltB3159:0.0,ShmMarin:0.0):5.5E-
4)0.582.15:5.5E-4)0.789.107:5.5E-4,(MaiArcti:5.3E-
4,UltB3160:0.02333)0.959.134:0.01896)0.983.106:0.02315)0.855.142:0.00764)
0.825.93:0.01147,MaiBact9:5.5E-4)0.828.87:0.01894,RsbPrion:5.5E-
4)0.957.128:0.0139,(UltB3268:0.02671,((((JnnDongh:5.5E-
4,(RssAnta2:0.0,RssAntar:0.0):0.015)0.410.17:0.00663,(UltAlp21:0.01122,Rh
rBac11:5.5E-4)0.823.100:0.00367)0.231.13:5.4E-
4,RgrSpec5:0.02355)0.902.149:5.4E-4,TtmOmpha:5.5E-4)0.213.9:5.4E-
4)0.919.182:0.00421)0.915.146:0.00429)0.510.11:5.4E-
4,(((AphPro15:0.0,TltSten2:0.0,TltSten3:0.0):5.4E-
4,(UltB3186:0.01503,TltSteno:5.5E-
4)0.451.25:0.00745)0.772.122:0.0037,(MucBact2:0.00737,UltMar28:0.03171)0.
865.146:0.00754)0.889.147:0.0074)0.929.140:0.00441)0.924.147:0.00443)0.84
5.135:0.00373)0.757.91:0.00362)0.869.123:0.00736,UltAlp59:0.00759)0.792.1
18:0.00349)0.748.89:5.5E-
4)0.860.120:0.00768)0.885.146:0.01061)0.451.26:5.4E-
4,((DinShib2:0.0,DinShiba:0.0):0.01902,TrnAlkan:0.0202)0.754.94:0.00435)0
.920.166:0.01414)0.773.129:0.00402)0.783.131:0.00356)0.826.94:0.00358)0.4
37.22:5.4E-
4)0.879.160:0.00737,((((PaeMaris:0.02336,UltB3213:0.03559)0.754.95:0.0035
9,RhvLacip:0.00753)0.771.137:0.00369,RhvImhof:0.00376)0.875.167:0.00748,(
UltB3269:0.05019,(RhvAdria:0.01513,RhvRobig:0.00382)0.719.35:0.01143)0.78
2.131:0.00398)0.885.147:0.0076)0.869.124:0.00851,(UltB3119:0.03156,(UltAl
p62:0.0304,UltB3263:0.0475)0.816.91:0.01012)0.738.65:0.00223)0.337.13:0.0
0368,((((UltAlp93:0.04598,UltB3512:0.00672)0.983.107:0.03089,((((((BrvDim
i2:0.00746,(UltRum10:0.00369,((BrvDimi3:0.0,BrvDimi4:0.0,BrvDimi5:0.0,Ult
Brev2:0.0,UltB3468:0.0):5.5E-4,UltB3458:0.00369)0.139.15:5.5E-
4)0.855.143:0.0037)0.942.142:5.4E-4,(UltB3465:5.4E-
4,(BrvDimi6:0.00742,((UltB3467:0.00384,((((BrvSpec5:0.0,BrvSpec6:0.0):5.4
E-4,(((UltB3461:0.00369,BrvSpec3:5.5E-4)0.028.9:5.5E-
4,(BrvSpec4:0.00369,UltB3462:0.03048)0.942.143:5.5E-
4)0.940.136:0.00743,(StpLongi:0.0,BrvSpec2:0.0,BrvInter:0.0):5.4E-
4)0.838.126:0.0037)0.839.118:0.00358,((CauSpec4:0.0037,BrvBacte:5.5E-
4)0.915.147:0.00746,((BrvnAlba:0.00371,(UltB3453:5.5E-
4,UltB3463:0.00369)0.814.81:5.5E-4)0.909.135:0.00751,((BrvKwang:5.5E-
4,UltBrev3:0.0037)0.033.6:5.3E-
4,(UltB3460:0.0037,(((UltB3455:0.0,AphPro35:0.0,CauSpec3:0.0):5.5E-
4,BrvSpeci:0.00369)0.923.196:0.00741,((BrvVaria:5.5E-
4,DriWater:0.0074)0.914.166:0.00744,(UltB3454:0.0,CauSpec2:0.0):0.00371)0
.157.14:5.5E-4)0.526.15:5.1E-
4)0.939.129:0.00746)0.761.103:0.00369)0.898.157:0.00752)1.000.1009:5.4E-
4)0.872.148:0.00742,(UltB3459:0.0037,BrvAveni:0.01524)1.000.1010:5.4E-
4)0.797.95:0.00349)0.992.128:0.02374,(BrvDimin:0.0037,(UltB3456:5.5E-
4,UltB3457:0.0037)0.650.22:5.3E-4)0.955.113:0.01123)0.887.149:5.4E-
4)0.940.137:0.01123)0.918.168:0.00722)0.433.16:0.01103,((UltCaul6:5.4E-
4,((AstTaihu:5.5E-4,AstBenev:5.5E-4)0.976.110:0.03666,(AstExce2:5.5E-
4,AstExcen:0.00369)0.744.81:0.00348)0.998.194:0.06839)0.754.96:0.02574,(U
ltB3466:0.01919,(UltB3490:0.02866,UltEndo2:0.01648)0.671.15:0.01919)0.928
.135:0.02371)0.601.10:0.0174)0.927.137:0.02352,((((UltAlp91:0.01155,((Ult
B3475:0.03115,UltPheny:5.3E-
4)0.969.108:0.01516,(UltB3474:0.01931,UltB3480:0.01124)0.885.148:0.00784)

0.850.153:0.0075)0.903.130:0.00725,(PhnCompo:0.01512,UltCaul8:0.00373)0.9
93.123:5.4E-
4)0.821.88:0.01521,(UltB3485:0.03571,(UltB3486:0.00768,(UltB3484:5.5E-
4,UltCaul7:0.01506)0.868.129:0.00743)0.951.139:0.01956)0.764.128:0.00497)
0.694.26:0.01205,(((AfpGeno3:5.3E-
4,(CauFusif:0.01148,((PhnConju:0.00749,(UltB3491:0.00366,(Udntfde5:5.5E-
4,(((UltB3470:0.0038,UltB3471:0.0019)1.000.1011:0.00178,(CauVibri:5.5E-
4,CauSegni:5.5E-4)0.917.138:5.5E-
4)0.985.121:0.01539,UltB3472:0.00369)0.859.134:5.5E-4)0.953.113:5.3E-
4)0.890.152:0.00741)0.873.139:0.00776,UltB3469:0.03191)0.751.82:0.00328)0
.978.94:0.01526)0.962.135:0.01507,((UltB3477:5.4E-
4,(UltB3476:0.00748,PhnImmob:0.00371)0.810.92:0.00367)0.986.113:0.02285,(
(((UltB3473:0.00745,(CauMirab:0.00744,((UltB3487:0.03631,(UltB3483:5.4E-
4,(UltB3489:0.00579,(UltB3482:0.02537,(UltB3481:5.5E-
4,UltB3630:0.00741)0.908.167:0.013)0.835.107:0.00759)0.896.137:0.00911)0.
331.14:0.0116)0.771.138:0.00353,PhnFalsu:0.00747)0.487.25:5.3E-
4)0.045.10:5.5E-4)0.001.8:5.4E-
4,(Ult14247:0.01958,UltCaul5:0.01163)0.772.123:0.00361)0.900.130:0.00759,
UltB3488:0.00369)0.445.21:5.3E-4,UltB3478:5.5E-4)0.991.127:5.4E-
4)0.951.140:0.01158)0.756.113:0.00384,UltB3479:0.00753)0.920.167:0.0163)0
.699.36:0.00371)0.950.123:0.01978,CanLiber:0.07642)0.753.100:0.0057,(((((
RhiSpe19:0.00372,((UltB3013:0.0,BacteFK8:0.0,RhiSpe23:0.0,RhiSpe24:0.0,Ul
tRhiz2:0.0):5.5E-4,RhiSpec6:0.00367)0.665.22:5.4E-4)0.896.138:5.5E-
4,(Ult13239:0.0229,(UltB3031:0.00371,(RhiSpe35:0.0,RhiSpe36:0.0,RhiSpe37:
0.0):5.5E-
4)0.780.139:0.00375)0.751.83:0.00361)0.892.142:0.00748,(RhiSpe18:5.5E-
4,((((RhiIndig:0.0,RhizEtli:0.0,RhiSpe13:0.0,RhiTrop4:0.0,RhiTrop5:0.0,Rh
iSpe15:0.0,RhiMult2:0.0,RhiMulti:0.0,RhiTrop2:0.0,RhiTropi:0.0,RhiRhizo:0
.0,RhiLegu2:0.0,RhiLegu3:0.0,RhiLegu4:0.0,RhiLegu5:0.0,RhiSpe20:0.0,RhiSp
e21:0.0,RhiSpe22:0.0,RhiLegu6:0.0):5.5E-4,RhiLegum:5.5E-4)0.000.989:5.5E-
4,(RhiTrop3:0.0074,RhiSpe16:0.01879)0.966.147:5.5E-4)0.000.990:5.5E-
4,RhiSpe14:5.5E-4)0.738.66:5.5E-
4)0.781.111:0.00371)0.872.149:0.01199,(UltB3047:0.04557,((BccBacte:0.0,St
pSpeci:0.0):5.5E-
4,(PdcGlaci:0.00748,OchGalli:0.00748)0.812.84:0.00367)0.840.133:0.01162)0
.802.102:0.00907)0.860.121:0.01184,(((UltB3039:5.4E-
4,((((OchSpec6:0.0,BacSV68A:0.0):5.5E-
4,((UltB3045:0.00372,((PdcSacch:0.0,PdcSpeci:0.0,UltB3046:0.0):5.5E-
4,PdcSacc2:5.5E-
4)0.768.102:0.00367)0.943.134:0.01122,DaeCaeni:0.00748)1.000.1012:5.5E-
4)0.883.154:5.5E-4,((BllAbort:5.5E-
4,((OchAnth3:0.0,UltOchr3:0.0):0.00371,((BllMelit:0.0,BllCanis:0.0,BllMel
i2:0.0,OchTriti:0.0,OchSpec2:0.0,OchAnthr:0.0,OchLupin:0.0,UltB3038:0.0,O
chGrign:0.0,OchSpec5:0.0,UltB3041:0.0):5.5E-
4,UltB3077:0.00369)0.029.7:5.5E-
4)0.866.150:0.0037)0.820.85:0.00369,BacSV682:0.00368)0.341.14:5.3E-
4)0.994.140:5.4E-4,((((OchSpeci:0.0,OchTrit2:0.0,OchTrit3:0.0):5.4E-
4,(OchAnth2:0.0,UltB3040:0.0,OchInter:0.0,UltB3043:0.0):0.00361)0.820.86:
0.00744,(OchAnth4:0.0,RhiSpe38:0.0):0.00362)1.000.1013:5.5E-
4,UltB3042:5.4E-
4)0.991.128:0.01533)0.819.91:0.00369)0.775.115:0.00369,(OchGrig2:5.4E-
4,((OchPseud:0.0,OchSpec3:0.0,UltOchr2:0.0,OchSpec4:0.0):5.4E-
4,UltB3044:0.0229)0.158.14:0.00743)0.899.146:0.00755)0.893.158:0.01196,((
(((BrtChome:0.0,WolMelop:0.0,BrtBovis:0.0,BrtCapre:0.0):0.00369,(BrtBirtl

:0.00374,(BrtCoope:0.00369,((BrtVins2:0.0,BrtTribo:0.0):5.5E-
4,((BrtClarr:0.0,BrtClar2:0.0):5.4E-4,(BrtBacil:0.01151,BrtQuint:5.5E-
4)0.946.128:0.00763)0.837.102:0.00377)0.290.16:5.3E-
4)0.801.98:0.00382)0.802.103:0.0038)0.380.13:5.4E-4,BrtVinso:5.5E-
4)0.910.142:0.00376,((UltRhi12:0.0,UltRhi13:0.0):0.01898,((UltRhi17:0.015
15,((UltRhi20:0.01862,UltRhi19:0.03163)0.768.103:0.00852,UltRhi18:5.4E-
4)0.865.147:0.00786)0.788.112:0.00887,AphPro23:0.06319)0.951.141:0.02618)
0.893.159:5.4E-4)0.964.138:5.5E-
4,((((((UltB3034:0.00369,ShnSpeci:0.00744)0.976.111:5.3E-
4,(UltB3035:0.0056,UltB3036:0.00367)0.784.119:0.00559)0.907.158:0.00207,(
UltB3032:5.4E-
4,((((BlaCapsu:0.01146,(((UltB3012:0.0,RhiMong2:0.0,RhiMongo:0.0,RhiYangl
:0.0,RhiSpec2:0.0,RhiSpec3:0.0):5.5E-4,RhiSpeci:5.5E-
4)0.922.199:0.00245,(UltB3079:0.03901,(((RhiSpec5:0.0,RhiAlami:0.0,RhiSpe
10:0.0,RhiSpec9:0.0,RhiSpe11:0.0):5.5E-
4,(RhiSpec7:0.00369,RhiSpe12:0.00369)0.907.159:5.5E-4)0.521.11:5.4E-
4,((((((SnrSpe10:0.0,UltB3037:0.0):5.5E-4,ShnKumme:5.5E-
4)0.891.118:0.00734,(UltB3048:0.00368,(UltRhiz5:0.0,ShnGranu:0.0,ZglRamig
:0.0,ShnSpec2:0.0):0.00746)1.000.1014:5.3E-
4)0.698.32:0.01118,(((AgrTume9:5.5E-
4,((RhiSpe40:0.0037,((UltB3028:0.0,UltB3027:0.0):5.5E-
4,UltB3029:0.01116)0.840.134:0.00369)0.781.112:5.5E-
4,(UltB3030:0.0074,BlaAggre:0.00741)0.647.10:5.5E-4)0.000.991:5.5E-
4)1.000.1015:5.4E-
4,(UltOchro:0.01131,(((AgrVitis:0.0,RhiVitis:0.0):0.00754,((RhiCellu:0.0,
UltB3015:0.0):0.00744,RhiGale2:5.3E-
4)0.758.106:0.00367)0.886.178:0.00751,(((AgrSpeci:0.0,AgrTumef:0.0,AgrSpe
c2:0.0,RhiSpe29:0.0,RhiSpe30:0.0,BacEU103:0.0,AgrTume3:0.0,UltB3026:0.0,R
hiSpe33:0.0):5.5E-4,(RhiSpe17:0.00368,AgrTume2:0.09515)1.000.1016:5.5E-
4)0.737.79:5.5E-4,(UltB3021:0.00367,UltB3022:0.0037)0.266.11:5.5E-
4)0.464.19:5.4E-
4)0.774.128:0.00365)0.829.117:0.00361)0.957.129:0.01491,(((AgrTume8:0.003
69,(RhiSpe34:0.0,RhiSelen:0.0,UltB3051:0.0):5.5E-
4)0.922.200:0.00246,(AphProte:0.00371,((UltB3050:0.0,UltB3052:0.0,UltB305
4:0.0):5.5E-4,UltB3053:0.00369)0.190.11:5.5E-
4)0.922.201:0.00246)0.922.202:0.00246,((((UltRhiz3:0.0112,RhiSpe28:5.5E-
4)0.848.120:0.00378,BacIrTJG:0.00369)0.452.16:5.5E-4,AgrTum10:5.5E-
4)0.607.14:5.5E-4,(AgrSpec5:0.00371,AgrSpec6:0.00715)0.882.165:5.5E-
4)0.558.13:5.4E-4)0.943.135:5.5E-4)0.897.145:0.00741)0.594.21:5.4E-
4,(((((SnrSpe12:0.00369,UltB3024:0.00742)0.683.35:5.5E-
4,((UltB3023:0.00746,ShnYamba:0.0037)0.326.7:5.5E-
4,UltB3049:0.0074)0.861.163:5.5E-4)0.000.992:5.0E-
4,(MesTians:0.02299,(OrzSativ:0.02677,(RhiSpe26:0.00369,(((AgrTume5:5.5E-
4,RhiHuaut:0.0037)0.000.993:5.5E-
4,(UltB3010:0.0,RhiGaleg:0.0,RhiSpe25:0.0,RhiLoess:0.0,UltRhiz4:0.0,AgrSp
ec4:0.0,RhizRubi:0.0,UltB3025:0.0):5.5E-4)0.000.994:5.5E-
4,(UltB3020:0.0037,RhiSpe32:0.00369)0.870.133:5.5E-4)1.000.1017:5.4E-
4)0.000.995:5.3E-4)0.277.16:0.00741)0.996.142:5.5E-4)0.862.149:5.5E-
4,(AgrYyyyy:0.0,AgrTume4:0.0,AgrSpec3:0.0,AgrTume6:0.0,RhiDaeje:0.0,SnrSp
e11:0.0):5.5E-4)0.718.40:5.4E-
4,(AztChro5:0.00744,AgrTume7:0.00744)0.766.103:5.5E-4)0.542.15:5.5E-
4)0.811.81:0.00747,(UltRhi22:5.0E-
4,((McplPeli:0.0,RhiUndic:0.0):0.0037,SnrSpe13:0.0037)0.795.111:5.3E-
4)0.807.79:5.3E-4)0.903.131:0.00741)0.847.152:5.5E-

4)0.922.203:0.00241)0.901.148:0.00228)0.975.106:0.01531,(SnrFred2:5.4E-
4,((RhiSpec8:0.00707,(UltRhiz6:0.01107,RhiSpe39:0.01583)0.483.19:0.00761)
0.141.10:5.5E-
4,(McpRamos:0.0,McpDimo2:0.0,McpDimor:0.0):0.00747)0.909.136:5.4E-
4)0.560.12:5.5E-4)0.898.158:5.4E-
4,EnfAdhae:0.05198)0.923.197:0.00709,(UltB3061:0.01569,UltB3118:0.02716)0
.477.20:0.01144)1.000.1018:5.4E-
4)0.914.167:0.00208)0.926.170:0.00692,UltAlph3:0.0113)0.509.13:5.4E-
4,(UltRhi10:5.5E-4,(UltRhiz8:5.5E-
4,(UltRhiz9:0.00374,UltRhi11:0.00373)0.802.104:0.00372)0.253.15:5.5E-
4)0.857.145:0.00363)0.837.103:0.00363,((UltBart2:0.0,UltRhiz7:0.0):5.5E-
4,UltBarto:5.5E-4)0.997.151:5.4E-
4)0.519.12:0.00735)0.917.139:0.01358)0.754.97:0.00526)0.971.103:0.02676)0
.927.138:0.02079)0.122.5:5.5E-
4,(UltAlp63:0.04303,UltB3193:0.11124)0.835.108:0.01881)0.726.40:0.00612,(
((PrhLitor:0.00616,(((UltB3564:0.03701,(((LbrAlexa:0.01483,((((PnnPhra2:0
.0,PnnPhrag:0.0):0.0037,((SppStell:0.00361,(UltB3451:0.0074,UltB3452:5.5E
-4)1.000.1019:5.3E-4)0.861.164:0.00742,(MucBact3:5.5E-
4,UltB3450:0.00747)0.871.150:0.00361)1.000.1020:5.5E-
4)0.864.140:0.0037,((LbrAggre:0.0,LbrAggr2:0.0):5.5E-
4,LbrnAlba:0.00373)0.219.9:5.5E-4)0.786.107:0.00374,BacteX20:5.5E-
4)0.000.996:5.4E-4)0.855.144:0.00758,(UltAlp88:0.01531,((UltB3432:5.4E-
4,BacEnr14:0.00749)0.968.113:0.01946,UltB3511:0.0235)0.851.144:0.00765)0.
754.98:0.0034)0.764.129:0.0042,((((UltRhi28:0.013,(PltYinch:0.0,PrhHirsc:
0.0):0.0489)0.808.99:0.00968,((((((AncOersk:5.5E-
4,(AncSpeci:0.0075,(MhdMulti:5.5E-4,(AncVacuo:0.0,AngTetra:0.0):5.5E-
4)0.872.150:5.4E-4)0.935.132:0.00747)0.916.164:5.0E-
4,(AncSpec2:0.01477,(UltB3439:5.4E-
4,(((UltB3437:0.0037,StaNovel:0.00745)0.793.106:0.00369,UltAncyl:5.5E-
4)0.029.8:5.5E-
4,UltB3438:0.01124)0.860.122:0.00368)0.970.124:0.01989)0.316.10:5.5E-
4)0.981.113:0.02409,UltB3364:0.00742)0.417.22:5.5E-4,UltAlph2:5.3E-
4)0.931.152:5.4E-
4,(UltB3441:0.01948,UltB3442:0.00728)0.995.147:0.03595)0.000.997:0.00461,
(AphPro30:0.04371,((UltBrady:0.02723,((((((UltB3297:0.00373,(UltRho39:0.
0,UltB3298:0.0):5.5E-4)0.775.116:0.00346,((UltB3302:5.4E-
4,((UltB3301:0.01116,((RhoPalu4:0.0,RhoPalu3:0.0):5.5E-4,RhoPalu2:5.5E-
4)0.836.100:5.5E-
4)0.753.101:0.00366,UltB3300:0.02342)0.783.132:0.0076)0.901.149:0.00712,R
hoPalu8:5.5E-
4)0.658.17:0.00756)0.770.133:0.00401,((RhoPalu5:0.0,RhoPalu6:0.0,RhoPalu7
:0.0):0.00379,UltB3308:0.01118)0.880.156:0.00749)0.882.166:0.00751,UltB32
96:5.5E-
4)0.942.144:0.01134,(UltB3309:0.0158,UltB3299:0.02398)0.737.80:0.00306)0.
878.142:0.00748,(((AfpGenos:0.0,UltAlp73:0.0):5.4E-
4,(UltB3304:0.00712,((AfpFeli2:0.0,AfpFelis:0.0,UltAlp72:0.0):0.00339,Ult
B3303:0.00403)0.120.13:0.00371)0.914.168:5.5E-4)0.958.154:5.4E-
4,(((UltB3312:5.5E-4,(((UltrRap5:0.00369,UltB3295:0.00742)0.819.92:5.5E-
4,((NtbAlkal:5.5E-4,(UltNitr4:0.00369,NtbWinog:0.00743)0.165.8:5.5E-
4)0.838.127:0.00369,UltB3314:0.00369)0.652.15:5.5E-4)0.000.998:5.5E-
4,(UltrSo30:0.0,BrdJapon:0.0):5.5E-4)0.746.78:5.5E-4)0.491.15:5.5E-
4,BrdSpec9:0.00365)0.461.13:5.5E-
4,((UltB3292:0.00361,(((AgmOligo:0.0,BrdSpec5:0.0,BrdSpec6:0.0,BrdSpec8:0
.0,BrdSpe10:0.0,AfpBroom:0.0,UltB3313:0.0,BrdSpe13:0.0,BrdSpe14:0.0,BrdEl

kan:0.0,BrdSpe15:0.0,BrdSpe17:0.0,UltB3365:0.0):5.5E-
4,(BrdSpe11:0.00369,BrdSpe16:0.00374)0.918.169:5.5E-4)0.744.82:5.5E-
4,(UltB3294:0.00369,(NtbHambu:0.0037,BrdSpe18:0.00751)0.801.99:0.00372)0.
663.26:5.5E-4)0.952.133:5.5E-4)0.856.122:0.00354,(((BrdSpec2:5.5E-
4,BrdSpeci:5.5E-
4)0.870.134:0.00372,((BrdSpec3:0.0,BrdSpec7:0.0,BrdSpe12:0.0):5.5E-
4,UltB3310:0.00369)0.681.21:5.5E-4)0.859.135:0.00363,(UltB3311:5.5E-
4,UltB3315:0.00747)1.000.1021:5.4E-4)0.845.136:0.00752)1.000.1022:5.4E-
4)0.932.154:0.0036)0.838.128:5.4E-
4)0.900.131:0.00717,(UltB3306:0.03057,(UltB3305:0.0,UltB3307:0.0):5.5E-
4)0.763.132:0.00366)0.866.151:0.00725)0.898.159:0.01057,(((UltB3338:5.4E-
4,UltB3362:0.00723)0.947.121:0.01515,(((((UltB3336:5.5E-
4,(((UltB3317:0.0,UltB3351:0.0):5.4E-
4,((((UltB3340:0.00237,UltB3341:0.01011)0.903.132:0.01016,(UltB3345:0.003
69,(UltB3344:0.0,UltB3554:0.0):5.5E-4)0.906.164:5.4E-
4)0.886.179:0.00763,(((PlbTaiwa:0.00748,((UltPro76:0.00357,(UltB3342:0.01
496,UltrRap6:0.00748)1.000.1023:5.5E-
4)0.904.157:0.01128,(UltB3346:0.0037,UltB3347:5.5E-
4)0.756.114:0.00357)1.000.1024:5.5E-
4)0.918.170:0.00997,((UltXan23:0.00373,((UltFore5:0.0,UltCaul4:0.0):5.4E-
4,(UltB3350:0.0,UltB3352:0.0):0.00372)0.800.88:0.00371)0.941.126:0.01164,
UltHyph2:0.00766)0.895.158:0.00443)0.906.165:0.00433,UltB3343:0.00375)0.8
74.164:0.00757)0.784.120:0.00374,UltB3349:0.0038)0.787.111:0.00367)0.659.
20:0.00204,(UltB3353:0.01144,Ult11739:0.03685)0.747.88:0.00948)0.786.108:
0.00537)0.768.104:0.00376,((UltB3331:0.0037,(UltB3360:0.00742,UltB3324:0.
00383)0.124.17:5.4E-
4)0.933.161:0.01201,((((UltB3546:0.01517,Ult27546:0.07185)0.887.150:0.016
23,RhpSpeci:0.0229)0.845.137:0.00775,(UltB3547:0.03416,UltB3333:0.01547)0
.629.26:0.00344)0.772.124:0.00422,(Ult15745:0.00739,(BlsSulfo:0.00375,(Bl
sViri2:0.03846,BlsVirid:5.5E-
4)0.775.117:0.00363)0.960.122:0.01524)0.868.130:0.00766)0.777.125:0.00403
)0.786.109:0.00356)0.882.167:0.00742,(UltB3339:0.0,UltB3356:0.0,UltB3357:
0.0):5.3E-
4)0.845.138:0.00364,((UltB3354:0.00371,UltAlp97:0.00748)0.797.96:0.0037,(
UltPro77:0.01132,UltB3348:0.01524)0.863.158:0.00747)1.000.1025:5.4E-
4)0.967.102:0.01501,((UltB3293:0.01158,(UltB3321:5.3E-
4,(UltAlp74:0.00373,(UltB3316:5.5E-4,((UltB3337:0.0,UltAlp75:0.0):5.5E-
4,UltHypho:0.00369)0.968.114:0.01133)0.900.132:0.00745)0.879.161:0.00746)
0.780.140:0.00362)0.832.104:0.00754,(UltrSo31:0.00379,((((((UltB3330:0.00
378,UltB3359:0.00754)0.787.112:0.00367,(UltB3326:5.4E-
4,(UltB3355:0.00754,(UltFore4:0.015,UltB3327:5.5E-
4)0.787.113:0.00368)0.783.133:0.00373)0.845.139:0.00368)0.685.23:5.5E-
4,UltB3328:5.5E-4)1.000.1026:5.4E-4,(((((UltB3358:5.4E-
4,(UltB3334:0.00371,(UltFore6:5.5E-4,(UltB3325:5.5E-
4,UltFore3:0.00369)0.746.79:5.5E-4)0.977.105:5.4E-
4)0.767.109:0.0037)0.000.999:5.5E-
4,((UltPro36:0.00365,((Ult15380:0.06781,Ult15379:0.01577)0.875.168:0.0156
6,Ult11738:0.03546)0.955.114:0.02052)0.855.145:0.00758,UltAlp77:5.5E-
4)0.950.124:0.01506)0.756.115:0.00358,(UltrSo32:0.01129,(((RhpRoseu:0.003
79,UltAlp76:0.01141)0.785.120:0.00365,(RhpElega:0.0075,RhpSeren:0.01497)0
.257.7:5.3E-4)0.770.134:0.00371,RhpSpec2:5.4E-4)0.363.19:5.5E-
4)0.834.103:0.00745)0.952.134:5.4E-
4,(AphPro24:0.0,AphPro25:0.0):0.01132)0.959.135:0.0114,((UltB3329:0.0,Ult
Caul3:0.0):5.5E-

4,((UltBrad3:0.00246,UltB3332:0.00246)0.922.204:0.00245,(UltCaulo:0.00657
,UltCaul2:0.0693)0.872.151:0.01272)0.895.159:5.5E-4)0.952.135:5.5E-
4)0.827.106:0.00365)0.788.113:0.0075,(UltRhi24:5.5E-
4,UltB3323:0.01512)0.817.82:0.00361)1.000.1027:5.5E-4,AphPro26:5.4E-
4)0.924.148:0.01143)0.310.13:0.00383)0.953.114:5.4E-
4)0.843.115:0.00721)0.385.20:5.5E-
4,(UltB3545:0.02702,((((((XnbAutot:0.0,XnbVisco:0.0):0.01142,(UltB3436:0.
00712,XnbSpeci:0.01114)0.916.165:5.4E-
4)0.860.123:0.00874,(((AzzCauli:0.0,AzzSpeci:0.0):0.00743,(AzzDoebe:0.007
43,AphPro29:5.4E-4)0.959.136:0.015)0.981.114:5.5E-
4,((XnbTaget:0.0,XnbAmino:0.0):5.5E-
4,XnbAgili:0.01126)0.801.100:0.00365)0.773.130:0.00637)0.988.126:0.02749,
(AmoCoral:0.01903,(UltB3057:0.01505,UltB3550:0.01126)0.756.116:0.00381)0.
776.119:0.00488)0.920.168:0.01797,UltB3361:5.4E-
4)0.930.139:0.01113,UltB3322:5.4E-4)0.088.11:5.3E-
4)0.939.130:0.00735)0.773.131:0.00385)0.220.14:0.00406)0.808.100:0.01055)
0.837.104:0.01812)0.968.115:0.02276,(((LabMethy:5.3E-4,((LabMiyag:5.4E-
4,(UltB3395:0.00359,UltB3396:0.02317)0.882.168:0.00762)0.808.101:0.00368,
LabNeptu:5.5E-
4)0.964.139:0.02309)0.998.195:0.0501,((((((BosEneae:0.0,BosMassi:0.0,BosS
peci:0.0):5.5E-4,AfpGeno2:0.00369)0.859.136:0.00968,(BosMinat:5.4E-
4,UltB3427:0.0037)0.789.108:0.00549)0.929.141:0.02409,(((UltB3407:0.00411
,(MetSpec8:5.5E-4,((UltB3397:0.0,UltB3398:0.0,UltB3405:0.0):5.5E-
4,UltB3406:0.00369)0.845.140:0.00369)0.936.134:0.01424)0.889.148:0.00416,
(UltB3404:0.00376,((((MetSpec5:5.4E-
4,UltB3403:0.00744)0.887.151:0.00746,(MetAdhae:0.00747,UltB3402:0.00747)0
.865.148:0.00757)0.765.112:0.00371,MetSpec6:5.4E-
4)0.927.139:0.01471,(((MetJeotg:0.0,MetSpec7:0.0):0.01107,((UltB3399:0.00
369,UltB3400:5.5E-
4)0.968.116:0.02344,(UltB3417:0.00287,UltB3418:0.01237)0.805.91:0.00807)0
.884.148:0.01216)0.860.124:0.00964,((((MetPersi:0.00369,(MetHispa:0.0,Ult
B3401:0.0,MetSpec4:0.0):5.5E-4)0.854.135:5.4E-
4,UltB3629:0.05597)0.900.133:0.0072,(MetPhyll:0.0037,((MlbBacte:0.0,MetSp
ec2:0.0,MetOryza:0.0,MetRadio:0.0,MetMesop:0.0,MetSpec3:0.0):5.5E-
4,Ult11282:0.00369)0.023.5:5.5E-4)0.917.140:5.5E-
4)0.893.160:0.01061,MetAerol:0.00441)0.845.141:0.01567)0.764.130:0.00438)
0.953.115:0.01942)0.869.125:0.00793)0.795.112:0.01477,MetOrgan:8.3E-
4)0.970.125:0.03107)0.653.19:0.00585,(((UltAlp87:5.4E-
4,(AphPro27:0.0094,(UltAlp86:0.01002,(SlrRosea:5.4E-
4,(UltB3431:0.00387,(UltB3430:5.5E-
4,UltBosea:0.01127)0.790.112:0.00355)0.967.103:0.02307)0.945.111:0.02169)
0.359.10:0.00733)0.872.152:0.00863)0.920.169:0.01148,((ChcAsacc:0.00379,(
ChcDaegu:0.00389,UltBeije:0.03167)0.781.113:0.00353)0.696.23:0.0063,(UltB
3434:0.01661,(UltEuba2:0.01235,UltB3433:0.01932)0.769.116:0.00694)0.861.1
65:0.00834)0.864.141:0.01198)0.757.92:5.5E-4,((UltB3319:5.5E-
4,UltB3429:0.00369)0.889.149:0.01127,((((MetIners:5.5E-
4,UltB3410:0.00369)0.048.6:5.3E-4,(((UltB3420:0.01338,(((UltMet14:5.4E-
4,McvSpeci:0.03912)0.375.16:0.00751,UltB3426:0.01546)0.178.6:0.00746,((Ul
tB3419:0.01554,((UltRhi25:0.0,UltRhi26:0.0):5.5E-
4,(MetSpe10:0.01432,((BlnSpeci:0.0,McvGuang:0.0):5.4E-
4,(UltB3423:0.00394,(UltB3421:5.5E-4,((((UltB3424:5.3E-
4,(BlnFlocc:0.00727,McvSubte:0.01106)0.925.161:5.4E-
4)0.775.118:0.00368,UltMet13:5.4E-4)0.860.125:5.4E-
4,UltB3425:0.04525)0.803.83:0.00761,UltB3422:0.00344)0.922.205:0.01146)0.

495.17:0.00745)0.831.104:0.00711)0.874.165:0.00962)0.957.130:0.01836)0.48
2.23:0.01149)0.771.139:0.00366,UltB3428:5.4E-4)0.876.129:5.4E-
4)0.861.166:0.01211)0.659.21:0.01261,(UltB3409:0.00375,((((MetExto2:0.0,M
etPodar:0.0,MetSuomi:0.0):5.5E-4,UltB3408:0.00369)1.000.1028:5.4E-
4,((MetPlata:5.5E-4,MetAquat:5.5E-
4)0.955.115:0.01816,(((MetVari2:0.0,MetVaria:0.0,UltB3412:0.0):0.00764,(M
etSpe11:5.5E-
4,MetNodul:0.00751)0.992.129:0.02341)0.653.20:0.00242,(MetRhodi:5.5E-
4,MetSalsu:5.5E-
4)0.770.135:0.00514)0.806.80:0.01587)0.852.137:0.0046)0.408.13:0.00741,Ul
tB3413:0.01926)0.919.183:0.01192)0.855.146:5.4E-
4)0.835.109:0.00713,UltB3411:0.00377)0.874.166:0.00746)0.894.139:5.4E-
4,MetSpec9:0.01525)0.941.127:0.01486,(UltrSo35:0.01154,(UltB3415:0.02319,
(UltrSo34:5.5E-4,UltB3414:0.00371)0.995.148:5.4E-
4)0.959.137:0.01915)0.918.171:0.01494)0.871.151:7.4E-
4)0.972.122:0.02028)0.968.117:0.02393)0.676.19:0.01223,(UltB3960:0.0852,U
ltPro78:0.06171)0.960.123:0.04681)0.896.139:0.02996)0.294.12:5.3E-
4,((UltB3318:0.01384,UltB3543:0.00924)0.825.94:0.01206,(UltB3544:0.02638,
((UltB3443:0.01481,(AphPro31:0.00576,UltB3542:0.0341)0.938.105:0.02724)0.
976.112:0.03011,(UltB3445:5.4E-
4,(UltB3320:0.03671,UltB3444:0.01216)0.890.153:0.01907)0.853.118:0.01279)
0.744.83:0.00747)0.919.184:0.02082)0.929.142:0.02877)0.893.161:0.02207)0.
780.141:5.3E-4,((UltB3385:0.03217,(MplCapsu:5.3E-4,(UltB3435:5.4E-
4,HanPlant:0.01142)0.920.170:0.00749)0.853.119:0.00761)0.837.105:0.00777,
(UltB3440:0.06218,(((MhtsYyyy:0.00392,BeiSpeci:0.02348)0.431.22:0.00737,U
ltB3387:0.00361)0.660.22:5.4E-
4,((((((UltB4753:0.08992,UltB3369:0.0082)0.678.25:0.00729,(UltAlp79:0.011
23,((BjrBacte:0.0,UltAlp80:0.0):0.00369,(UltAlp81:5.4E-
4,UltB3368:0.02707)0.469.17:0.00369)0.936.135:5.5E-
4)0.790.113:0.00366)0.785.121:0.00375,(UltB3383:5.4E-4,(MlpAcidi:5.4E-
4,(MhtHeyer:0.00722,(RdlSphag:0.0075,UltAlp83:0.00367)0.900.134:0.00784)0
.077.9:0.00372)0.848.121:0.00718)0.798.108:0.00374)0.873.140:0.00751,((((
BeiIndi2:0.0,BeiIndic:0.0,BeiDerxi:0.0):5.4E-4,((((((UltB3377:5.5E-
4,(((UltB3378:0.0,UltB3379:0.0):5.5E-
4,UltB3374:0.00743)0.799.106:0.00369,UltAlp84:0.00746)0.623.15:5.5E-
4)0.980.108:5.5E-4,((UltB3373:0.00433,(UltB3372:0.01125,UltB3376:5.4E-
4)0.849.129:0.00718)0.850.154:0.00807,UltB3371:0.01636)0.829.118:0.00714)
0.798.109:0.00365,UltB3375:0.00378)0.989.108:0.01964,(((UltB3394:0.00434,
UltrSo33:0.0111)0.995.149:0.03624,UltB3393:5.4E-
4)0.970.126:0.0153,(UltB3392:0.0032,((UltB3391:0.00396,(UltB3389:0.02729,
UltB3390:0.00379)0.840.135:0.00718)0.891.119:0.01897,BrdSpec4:0.15072)0.8
64.142:0.01699)0.870.135:0.00849)0.867.147:0.00754)0.853.120:0.00803,UltB
3363:0.02309)0.398.13:0.00761,(((UltB3382:0.01552,UltB3381:0.00708)0.762.
119:0.0075,UltAlp85:0.01091)0.739.66:5.4E-
4,UltB3370:0.00369)0.000.1000:0.0035)1.000.1029:5.3E-
4)0.924.149:0.00761,(MehSilve:5.5E-
4,MehPalus:0.01125)0.917.141:0.00744)0.615.21:5.4E-
4,(UltB3367:0.0037,(((UltB3380:0.00369,UltB3384:0.01131)0.682.23:5.4E-
4,UltAlp78:5.5E-4)0.905.150:5.3E-
4,((MhtSpec2:0.00741,(UltB3386:0.0075,((MhtSpeci:0.0,BacEnr13:0.0):5.5E-
4,TypeII00:5.5E-4)0.927.140:5.5E-4)0.435.18:5.4E-
4)0.805.92:0.00368,((MlnTrich:0.0154,MlnSpec2:5.5E-
4)0.787.114:0.00371,MlnSpeci:0.00372)0.799.107:0.00376)0.787.115:0.00369)
0.784.121:0.00365)0.815.82:0.00365)0.958.155:5.4E-

4)0.898.160:0.00754,(UltAlp82:0.01127,BeiFlumi:0.0037)0.896.140:5.3E-
4)0.101.7:0.00758,UltB3388:0.01133)0.588.24:5.3E-
4)0.905.151:0.01125)0.732.45:0.00371)0.888.158:0.00764)0.893.162:0.00701)
0.738.67:0.00313)0.940.138:0.01827,(UltAlp89:0.01191,(PdxnSoli:0.01147,((
((PdvSpeci:0.0,AphPro32:0.0,AphPro33:0.0):5.5E-
4,(AphPro34:0.00369,PdvJapon:5.5E-
4)0.996.143:0.02294)0.836.101:0.01892,(RbmHamel:0.00367,(UltB3449:5.5E-
4,RbmDenha:5.5E-4)0.725.49:5.5E-4)0.985.122:5.5E-
4)0.947.122:0.01306,(UltB3448:0.00941,NesExalb:0.01317)0.362.14:0.0037)0.
883.155:0.00954)0.740.82:0.00326)0.758.107:0.00756)0.803.84:5.5E-
4,(PrhConso:0.00355,(UltB3290:5.4E-
4,(UltB3291:0.03171,(UltSlu13:0.00589,UltMet12:0.01455)0.766.104:0.00611)
0.901.150:0.0115)0.970.127:0.01523)0.879.162:0.00763)0.786.110:0.00543)0.
888.159:0.00942,(((UltAlp96:0.05742,(UltB3563:5.4E-
4,(UltB3562:0.00745,UltFore7:0.00763)0.952.136:0.01928)0.907.160:0.02547)
0.938.106:0.02974,(UltB3446:0.00147,UltHyph3:0.0509)0.935.133:0.02267)0.7
39.67:0.00318,(((UltMeso3:0.01548,((CcbMarin:0.02571,(MaiBact5:0.00745,Ma
tMyrio:5.5E-
4)0.999.215:0.05886)0.565.12:0.01734,((UltDevos:0.01939,(UltB3095:0.02532
,(UltB3094:0.01306,(AphProt5:5.5E-4,DevSpeci:5.5E-
4)0.930.140:0.01735)0.688.26:0.00256)0.945.112:0.02095)0.891.120:0.01588,
(DevSpec2:0.01554,((UltB3110:0.03849,(((((DevoLimi:0.0,AntBac23:0.0):0.00
365,UltB3098:0.0075)0.873.141:0.00761,(UltrSo29:0.00374,UltB3097:0.019)0.
862.150:0.00747)0.955.116:0.01136,(CanDevos:0.00369,AntBac22:5.5E-
4)0.986.114:5.5E-4)0.724.47:0.01118,DevNeptu:5.3E-
4)0.820.87:0.01098)0.900.135:0.02121,((UltB3104:0.01483,(UltB3103:0.01162
,DevAlbog:0.01167)0.897.146:0.00111)0.872.153:0.01054,((((UltB3113:0.0074
8,(UltB3114:0.00372,UltB3112:0.01144)0.752.94:0.00374)0.899.147:0.00749,(
PrhMishu:5.5E-
4,(PrhEnhyd:0.00746,(((UltB3117:0.015,UltB3111:0.01109)0.912.176:5.5E-
4,(UltB3106:0.00369,((DevInsul:0.0,UltB3107:0.0,UltAlph8:0.0,UltB3366:0.0
):5.5E-4,UltB3109:0.00369)0.340.14:5.5E-
4)0.814.82:0.00358)0.743.95:0.00745,UltB3108:0.00358)1.000.1030:5.4E-
4)0.756.117:5.4E-4)0.934.129:5.3E-4)0.944.127:0.00752,(UltB9004:5.4E-
4,(UltB3115:0.00246,((DevChinh:0.0,DevGeoje:0.0):0.0037,UltB3116:5.3E-
4)0.922.206:0.00246)0.922.207:0.00246)0.734.73:5.3E-
4)0.941.128:0.00714,(UltB3096:0.00721,(((UltB3099:0.0,UltB3101:0.0):0.007
46,((DevCroci:0.0,UltB3100:0.0):0.00369,(DevSubae:5.5E-
4,(UltAlph7:0.00369,UltMar27:0.00741)0.705.27:5.5E-4)0.868.131:5.4E-
4)0.895.160:0.00757)0.926.171:0.01136,UltB3102:5.5E-
4)0.792.119:0.00395)0.795.113:0.01516)1.000.1031:5.4E-4)1.000.1032:5.4E-
4)0.760.114:0.00323)0.933.162:0.01157)0.841.116:5.5E-
4)0.871.152:0.01124)0.774.129:0.00383,((UltB3492:0.01338,((UltB3493:0.003
72,(PrhPneum:0.0,PrhSpec3:0.0):0.0308)0.896.141:0.00337,(((AphPro28:0.0,U
ltKaist:0.0):5.4E-
4,KaiAdipa:0.01134)0.926.172:0.00742,(KaiGranu:0.01496,(KaisSoli:5.5E-
4,(UltB3335:0.05219,(UltRhi27:5.5E-4,PrhSpec2:0.00371)0.573.11:5.5E-
4)0.887.152:0.01095)0.969.109:0.02301)0.139.16:5.5E-4)1.000.1033:5.5E-
4)0.462.24:0.00887)0.586.20:0.00751,(RdmGokar:0.0,RdmOrien:0.0):0.01025)0
.872.154:0.009)0.767.110:0.00317,((HpmZavar:0.01899,(UltB3552:0.00783,((U
ltAlp98:0.01135,((((UltRhi30:5.3E-4,UltB3557:0.00369)0.980.109:5.4E-
4,(UltB3556:0.01181,UltB3558:0.01502)0.709.36:0.01143)0.878.143:0.01103,U
ltB3551:0.01226)0.817.83:0.00742,(Ult27559:0.03661,(UltB3548:5.5E-
4,(UltB3105:5.4E-

4,UltB3549:0.00364)0.780.142:0.00744)0.757.93:0.00326)0.933.163:0.01166)0
.928.136:5.4E-
4)0.775.119:0.00506,(AnsBalti:0.0126,UltAl100:0.01426)0.943.136:0.0198)0.
842.130:0.01002)0.992.130:0.02749)0.911.177:5.5E-
4,((UltB3525:0.01539,((((((((((UltB3528:0.0,UltB3530:0.0):0.00684,UltB35
26:0.01597)0.564.14:0.00759,UltB3531:0.01098)0.077.10:5.4E-
4,UltrSo39:0.01109)0.934.130:0.01144,(UltSlu17:0.00373,UltB3539:0.00748)0
.797.97:0.00368)0.714.38:5.4E-4,UltSlu16:5.4E-
4)0.920.171:0.00369,(UltB3529:0.01916,UltB3533:0.01896)0.993.124:5.4E-
4)0.815.83:5.4E-4,UltB3532:5.5E-4)1.000.1034:5.3E-
4,((((FilInsig:0.01543,FilFusif:0.00374)0.880.157:0.0076,UltB3527:0.01122
)0.764.131:0.00375,(((UltB3284:5.5E-
4,BacEnr15:0.00743)0.842.131:0.00891,(UltB3287:0.00788,(UltB3286:0.02205,
(UltB3285:5.5E-4,UltGree2:5.5E-
4)0.915.148:0.01302)0.577.11:0.00885)0.899.148:0.01822)0.941.129:0.01871,
UltB3519:0.01565)0.751.84:0.0031)0.904.158:0.00497,((UltB3537:0.01501,(Ul
tHyph4:0.01508,((((PedManga:0.02212,(UltB3516:0.02332,HpmSulfo:0.02013)0.
256.12:0.00635)0.691.23:0.00456,(UltB3524:0.01499,(UltB3523:0.00743,UltHy
ph5:5.5E-4)0.681.22:5.5E-
4)0.917.142:0.01457)0.855.147:0.01039,UltB3517:0.02259)0.943.137:5.5E-
4,((HpmChlor:0.00743,(((HpmSpec2:0.0037,UltB3513:5.5E-4)0.964.140:5.5E-
4,(((((UltAlp94:0.01159,UltB3520:0.02352)0.890.154:0.01134,PedSpeci:0.007
53)0.781.114:0.00363,PedAustr:0.01127)0.932.155:0.00406,UltB3521:0.02634)
0.878.144:0.00387,UltB3522:0.01137)0.976.113:0.02312)0.941.130:0.01886,((
HpmFacil:5.4E-4,(UltB3515:0.0113,(UdnBac17:0.01119,(UltB3518:5.5E-
4,Ult14248:0.00744)0.839.119:0.00369)0.181.10:5.5E-
4)0.814.83:0.0037)0.839.120:0.00371,UltB3514:5.4E-
4)0.844.141:0.00369)0.514.15:5.5E-
4)0.819.93:0.00373,((UltB3534:0.0,UltB3536:0.0):0.00376,UltB3535:0.00706)
0.762.120:0.00354)0.749.103:0.00385)0.853.121:0.01058)0.062.11:0.00384)0.
839.121:5.3E-4,UltB3538:5.4E-
4)0.907.161:0.0034)0.935.134:0.01329)0.760.115:0.00337,(UltB3540:0.01891,
((UltB3281:0.00368,(UltB3283:0.00369,(UltRho38:5.5E-
4,UltrB105:0.01125)0.842.132:0.00369)0.182.12:5.5E-4)0.747.89:5.5E-
4,UltB3282:5.5E-
4)0.782.132:0.00368)0.763.133:0.00386)0.749.104:0.01052,(UltAlp99:0.01588
,(((UltRhi16:0.0074,UltMeso6:0.00741)0.904.159:5.3E-4,(((MesThiog:5.4E-
4,(MesSpe11:0.0037,(UltMeso4:0.01515,UltOrg19:0.01534)0.068.10:5.4E-
4)0.892.143:0.00723)0.937.136:0.0153,((EnfTera2:0.0,EnfTeran:0.0):0.0037,
(SnrSpec4:0.0,SnrSpec9:0.0,EnfFredi:0.0,MesSpec9:0.0):5.5E-
4)0.613.20:5.4E-4)0.918.172:5.5E-
4,(((((MesSpe10:0.01117,(MesSpec7:0.0,MesoLoti:0.0,MesOppor:0.0,MesSpe12:
0.0,MesSpe13:0.0,MesSpe14:0.0,MesHuaku:0.0):5.5E-4)0.951.142:5.5E-
4,MesoLot2:0.03473)0.833.118:0.00367,MesPluri:5.5E-
4)0.794.98:0.00371,UltEndol:5.5E-4)0.903.133:5.5E-
4,(AurSpeci:0.0037,(UltB3017:0.0037,((UltB3084:0.00369,((AurAltam:0.00364
,AurFrigi:0.00762)0.878.145:0.00763,UltB3087:0.00744)0.773.132:5.4E-
4)0.951.143:5.4E-4,(((UltB3085:0.0,UltB3093:0.0):0.00369,AurUreil:5.5E-
4)0.977.106:5.4E-4,(UltSino2:0.01825,(RhiSpe41:5.1E-
4,((((RhiSpec4:0.00729,UltB3011:5.4E-4)0.805.93:0.01523,(RhiSpe27:5.3E-
4,((MllMedit:0.0,RhiSpe31:0.0):0.01504,UltB3033:0.00394)0.781.115:0.01127
)0.776.120:0.00368)0.940.139:0.005,(((UltB3089:0.00741,(UltB3083:5.5E-
4,(UltB3086:0.00372,(AurCora2:0.0,AurCoral:0.0):0.0037)0.766.105:5.5E-
4)0.949.134:5.4E-4)0.722.37:0.00744,(UltB3090:0.0037,(UltB3091:5.5E-

4,(UltB3092:0.023,UltB3088:0.00369)0.972.123:5.5E-4)0.786.111:5.5E-
4)0.840.136:0.00365)1.000.1035:5.5E-4,RhiOryza:5.5E-
4)0.940.140:0.00491)0.938.107:0.00475,(UltB3019:0.01121,(UltB3016:5.5E-
4,UltB3018:0.00369)0.523.13:5.4E-
4)0.875.169:0.00759)0.284.13:0.00722)0.837.106:0.00933)0.933.164:0.01399)
0.824.88:0.00747)0.922.208:0.00746)0.727.46:5.4E-
4)0.921.150:0.01104)0.924.150:0.01095)0.638.17:0.01107)0.990.126:5.4E-
4,(UltB3082:5.5E-4,(UltB3081:5.5E-
4,UltB3065:0.01508)0.831.105:0.00369)0.952.137:0.00709)0.575.12:0.01162)0
.973.100:0.02286)0.135.11:5.4E-
4)0.695.22:0.00194,UltAlp95:0.00235)0.933.165:0.00691)0.856.123:0.00708)1
.000.1036:5.4E-
4)0.886.180:0.0071)0.873.142:0.00702,((NttSpeci:0.00363,(UltMeso7:0.00741
,ChlMulti:5.5E-
4)0.984.115:0.02324)0.276.14:0.01545,((AfiMarin:0.0,RhoJulia:0.0):0.04876
,((((((SnrSpec2:0.0,SnrSpeci:0.0,EnfMedic:0.0):5.5E-
4,(EnfMelil:0.0,EnfMeli2:0.0,SnrSpec3:0.0,EnfMeli3:0.0,SnrFredi:0.0,SnrSp
ec5:0.0,SnrSpec6:0.0,SnrSpec7:0.0,SnrSpec8:0.0):0.0037)0.960.124:0.01109,
UltMeso5:0.00719)0.922.209:5.3E-4,((UltB3076:0.01879,UltRhi21:5.5E-
4)0.895.161:0.00744,(UltB3559:0.03037,UltPhyll:0.01278)0.783.134:0.00707)
0.805.94:0.00369)0.434.18:5.5E-4,UltAlph6:0.01492)0.721.32:5.4E-
4,UdnBac13:0.01848)0.536.16:0.00761)0.623.16:0.01477)0.936.136:5.4E-
4)0.821.89:0.00576)0.877.156:0.0087)0.742.69:0.00382,(((WooMarit:0.0417,(
CauSpeci:0.02537,(OncAlexa:0.01936,UltAlp67:0.02796)0.382.18:0.01035)0.91
8.173:0.02736)0.903.134:0.01926,((UltOrg25:0.05644,(UltAlp66:0.02757,(Mcl
Salig:0.00534,(AphPro22:0.0075,(MclMaris:5.4E-
4,MclIndic:0.03521)0.901.151:0.0075)0.862.151:0.01027)0.905.152:0.01716)0
.806.81:0.01133)0.797.98:0.01933,(((UltB3277:0.02694,(HelBalne:0.02174,Ul
tB3276:0.03505)0.674.24:0.01704)0.120.14:0.01103,RbgAntar:0.02487)0.973.1
01:0.03999,(UltB3278:0.0191,UltB3817:0.12413)0.017.7:0.02832)0.811.82:0.0
1625)0.868.132:0.01572)0.736.61:0.005,((UltAlp69:0.06945,(HirSpeci:0.0205
,HirBalti:0.01281)0.864.143:0.01724)0.883.156:0.02624,(HpdBacte:0.04033,(
(UltB3273:5.4E-
4,(HenMarin:0.00764,AphPro21:0.0155)0.768.105:0.01134)0.985.123:0.0322,((
UltAlp65:0.00761,HpnOcean:0.01135)0.186.7:0.00373,(HpnSpeci:5.5E-
4,((HpnAdhae:0.00372,(HpnRosen:0.00743,HpnPolym:5.4E-
4)0.814.84:0.0037)0.776.121:0.00358,UltB3274:0.02737)0.863.159:0.00765)0.
888.160:5.3E-
4)0.896.142:0.01476)0.928.137:0.02178)0.841.117:0.01217)0.984.116:0.03557
)0.855.148:0.02335)0.978.95:0.01971)0.770.136:0.00611)0.845.142:0.01808,(
UltB1261:0.0396,UltB3275:0.0163)0.986.115:0.04893)0.940.141:0.02662)0.857
.146:0.01535,((AphPro48:0.06275,(((UltB3816:5.5E-
4,UltAl118:0.02655)0.975.107:0.0662,(UltB4084:0.07558,UltB4083:0.10741)0.
844.142:0.03251)0.788.114:0.04451,(UltB3685:0.06038,(UltB3822:0.01824,Mai
Meta5:0.01816)0.897.147:0.02249)0.783.135:0.01066)0.497.10:0.01127)0.833.
119:0.0187,UltMari7:0.11659)0.480.16:0.01481)0.508.9:0.00737)0.900.136:0.
01271,((UltAl179:0.07292,((((UltB4030:0.0037,(UltB4031:5.4E-
4,UltB4029:0.04799)0.960.125:0.01895)0.896.143:0.00732,(UltB4034:5.5E-
4,(UltB4033:0.00368,UltB4032:5.5E-4)0.947.123:0.00746)0.885.149:5.5E-
4)0.922.210:0.02486,(Ult15346:0.02597,(UltB4035:0.01625,UltB4037:0.05905)
0.822.86:0.01101)0.188.13:0.01091)0.890.155:0.0234,(UltB4036:0.05905,UltB
4073:0.07207)0.687.28:0.01354)0.982.102:0.04492)0.855.149:0.0201,((((UltB
4077:0.00652,UltAce10:0.05665)0.827.107:0.01179,(AphPro49:0.04978,(((UltB
3508:0.03567,(UltPro16:5.5E-

4,(((UltAlp92:0.01446,UltB3505:0.0042)0.368.12:0.00746,(UltB3506:0.01557,
UltB3507:0.01118)0.879.163:0.01096)0.898.161:0.00822,(UltRhi29:5.4E-
4,(UltSlu15:0.02225,UltSlu14:0.00957)0.880.158:0.00977)0.913.163:5.5E-
4)0.876.130:0.02624)0.926.173:0.04137)0.941.131:0.0404,((((UltB3497:0.022
24,((UltB3496:0.0205,(UltrSo37:0.00747,UltB3499:0.02333)0.897.148:0.01419
)0.506.15:5.4E-
4,((UltB3495:0.00949,UltrSo36:0.00565)0.649.20:0.00632,(UltB3494:0.01055,
UltB4002:0.00845)0.809.99:0.01664)0.891.121:0.01968)0.952.138:0.02462)0.9
23.198:0.02193,UltB3498:0.01247)0.815.84:0.0115,UltrSo38:0.01697)0.807.80
:0.00924,(UltB3500:0.03638,(UltB3504:0.04181,(UltB3501:0.01195,(UltB3502:
0.0,UltB3503:0.0):0.00317)0.762.121:0.00892)0.936.137:0.02286)0.904.160:0
.01543)0.845.143:0.01576)0.879.164:0.03095,((TstMobil:5.5E-
4,TstSpeci:5.5E-
4)0.984.117:0.04376,(UltB3935:0.07411,((UltB3997:0.01747,(RdsCente:0.0057
3,(AzlSpec5:0.00723,UltB3996:0.02813)0.947.124:0.02219)0.870.136:0.02622)
0.965.116:0.04067,UltB3993:0.05574)0.758.108:0.0125)0.753.102:0.02659)0.7
82.133:0.01621)0.749.105:0.00645)0.868.133:0.0126)0.852.138:0.0151,((((((
(((UltB4006:5.5E-
4,UltB4007:0.00369)0.760.116:0.00397,(UltAcet9:0.00387,(UltB4005:0.01143,
Ult11742:0.0038)0.856.124:0.00749)0.897.149:0.01134)0.958.156:0.02839,(Ul
tB4009:0.02163,(BacEllin:0.02698,UltB4008:0.03032)0.646.17:0.01641)0.925.
162:0.0239)0.962.136:0.02454,((((((SkeXinji:0.03182,UltSlu19:0.0519)0.692
.21:0.01483,((UltB4048:0.02898,(UltB4057:0.03449,UltB4058:0.02827)0.908.1
68:0.02315)0.887.153:0.02419,(UltB4003:0.06668,(UltB4056:0.06262,(UltB405
3:0.0113,(UltB4054:0.02603,UltB4055:0.00513)0.868.134:0.01493)0.936.138:0
.0261)0.721.33:0.00666)0.478.17:0.01311)0.748.90:0.01055)0.887.154:7.5E-
4,(UltB4039:0.05077,RhdBact2:0.04055)0.868.135:0.0178)0.483.20:5.5E-
4,(UltB4047:0.06113,UltB4004:0.03003)0.889.150:0.02527)0.930.141:0.02648,
((RsnGeno3:0.01142,((AzlCanad:5.4E-4,(AzlRugos:5.5E-
4,(AzlSpec4:0.01513,((AzlSpec3:0.0037,((AzlSpeci:0.0,AzllZeae:0.0):5.5E-
4,(AzlOryza:0.00743,AzlMelin:0.00369)0.708.34:5.5E-4)0.715.34:5.5E-
4)0.928.138:0.00193,(UltB3995:5.5E-4,AzlDoebe:5.5E-
4)0.940.142:0.0109)0.922.211:0.00204)0.798.110:0.00373)0.927.141:0.00752)
0.797.99:0.00377,((((AzlPicis:0.00745,UltB3994:0.00369)0.890.156:5.5E-
4,(AzlBrasi:0.01124,(Udntddd2:0.0,AzlBras2:0.0,AzlBras3:0.0):5.5E-
4)0.979.125:0.01505)0.652.16:0.00125,(AzlSpec2:0.00338,BacAEOC0:0.01989)0
.924.151:0.01536)0.940.143:0.01033,AzlLipof:0.00713)0.266.12:5.3E-
4)0.947.125:0.01958)0.980.110:0.02369,(EliTepid:0.07426,UltB4038:0.02678)
0.780.143:0.01777)0.268.7:5.5E-
4)0.951.144:0.0116,((StlHumos:0.02223,UltRho43:0.05686)0.889.151:0.01559,
(UltB4001:0.13198,(UltB3998:5.5E-
4,(SkeAerol:0.00101,(UltB3999:0.00807,UltB4000:0.01521)0.267.16:0.01494)0
.907.162:0.01834)0.781.116:0.01891)0.734.74:0.00962)0.866.152:0.01508)0.8
42.133:5.4E-
4)0.764.132:0.00371,(((((((UltRho42:0.00743,UltB4014:0.01138)0.748.91:0.0
0407,AzlSpec6:0.03154)0.188.14:0.00379,UltB4020:0.01123)0.753.103:0.00282
,((Ult14249:0.04994,((UltB4025:0.01525,UltB4026:0.00373)0.906.166:5.5E-
4,(UltNitr5:0.01128,UltB4027:0.03629)0.961.126:0.0236)0.929.143:0.02188)0
.594.22:0.00559,((UltB4023:0.00499,UltB4024:0.01019)0.965.117:0.02876,Ult
B4022:0.03856)0.776.122:0.00764)0.899.149:0.01311)0.760.117:0.00473,(UltB
4018:0.03302,((UltB4013:0.01027,(UltB4012:0.0,UltFor44:0.0):0.00492)0.999
.216:0.05238,(UltFore9:0.04861,UltB4015:5.5E-
4)0.757.94:0.00708)0.400.18:0.00752)0.929.144:0.01662)0.743.96:0.00393,(U
ltB4016:0.02278,(UltB4017:0.0371,UltB4019:0.02429)0.819.94:0.01491)0.854.

136:0.01403)0.878.146:0.01163,((((UltB1959:0.05572,(UltB3509:0.05131,(Ult
B3992:0.0378,UltMar36:0.04885)0.984.118:0.06029)0.430.21:0.0155)0.931.153
:0.02742,UltB4052:0.05894)0.778.118:5.4E-
4,((UltB4050:0.04378,(UltB3991:0.05686,((UltB3009:0.0037,(UltB3986:0.0,Ul
tB3988:0.0):5.5E-
4)0.799.108:0.01027,(UltB3989:0.01456,((UltB3140:0.0,UltB3990:0.0):0.0037
3,UltB3987:0.0114)0.832.105:0.00839)0.756.118:0.01443)0.825.95:0.01204)0.
837.107:0.02098)0.826.95:0.01241,((NisSalex:0.02391,((UltB4049:0.02707,(N
isDenit:0.01502,NisNitri:5.3E-
4)0.926.174:0.02897)0.904.161:0.03112,AphPro53:0.035)0.726.41:0.02293)0.7
38.68:0.01516,(UltAl183:0.0576,RhdBact3:0.0119)0.536.17:0.01752)0.796.82:
0.0113)0.957.131:0.02552)0.941.132:0.02911,(UltAl174:0.11182,((UltAl176:0
.09129,UltAl190:0.08933)0.668.32:0.00878,UltFore2:0.04651)0.914.169:0.027
49)0.599.18:0.00251)0.819.95:0.01389)0.513.13:0.0124)0.945.113:0.0205,(((
UltB3934:0.09924,((MgnCoccu:0.05243,(UltMagn4:0.0481,(UltMagn3:0.00363,Mg
nCocc3:5.4E-
4)0.979.126:0.04902)0.686.20:0.0108)0.858.141:0.0122,(MgnCocc2:0.0869,Ult
Magn5:0.07443)0.788.115:0.01279)0.899.150:0.02199)0.945.114:0.04026,((Ult
B4072:0.05634,(RhdBact5:0.00323,RhdBact4:0.00419)0.964.141:0.04383)0.948.
155:0.04292,(UltB4068:0.02276,((((UltAl189:0.0093,(UltB4066:0.00514,UltB4
065:0.01039)0.948.156:0.02139)0.852.139:0.0146,UltB4069:0.04664)0.875.170
:0.02212,UltB4067:0.04469)0.659.22:0.00269,KilLamin:0.07486)0.893.163:0.0
1468)0.851.145:0.01187)0.808.102:0.01216)0.231.14:0.01259,((UltAl143:0.06
343,TspSpeci:0.02496)0.968.118:0.04356,UltAl188:0.05736)0.692.22:0.00692)
0.961.127:0.02775)0.663.27:0.00475,((UltAl177:0.05493,UltB4078:0.06253)0.
839.122:0.01953,(UltB3945:0.05251,UltB4028:0.05012)0.775.120:0.0076)0.698
.33:0.01226)0.734.75:0.0074,((((((UltAl186:0.06672,(UltrB104:0.00802,((Ul
tAl184:0.04826,(UltAl185:0.05381,(UltB4061:0.02108,OlvLois2:0.02705)0.394
.21:0.0172)0.849.130:0.01724)0.781.117:0.01139,UltB4059:0.03813)0.885.150
:0.01773)0.867.148:0.013)0.849.131:0.02476,UltAl187:0.07352)0.829.119:0.0
2382,(UltB3915:0.0804,UltB4060:0.00463)0.890.157:5.4E-
4)0.878.147:0.0159,(((((((AphPro51:0.02753,Otu00547:0.09509)0.961.128:0.0
5491,UltAl144:0.10282)0.830.98:0.01759,((UltB4051:0.05881,(InqLimos:0.003
35,RdsSpeci:0.0117)0.916.166:0.0251)0.980.111:0.04148,((RdsRubru:0.01141,
(RdsPhoto:0.03119,RdsOryza:5.3E-
4)0.847.153:0.01966)0.984.119:0.03803,CnsBisan:0.03546)0.828.88:0.01425)0
.878.148:0.00885)0.748.92:0.0048,(UltB4086:0.07964,(((InsPereg:0.02453,Rs
rParvu:0.05518)0.861.167:0.01952,(MspIndic:0.0299,(NvsIters:0.01536,UltB3
914:0.01957)0.894.140:0.01422)0.889.152:0.01656)0.906.167:0.0183,(UltB327
9:0.00975,(TrsPusil:0.02757,UltB3912:0.01122)1.000.1037:0.08167)0.988.127
:0.04524)0.650.23:0.01537)0.944.128:5.4E-
4)0.952.139:0.01594,((UltB3916:0.03216,UltB3917:0.11325)0.869.126:0.01647
,AphPro55:0.01883)0.848.122:0.00784)0.888.161:0.01368,(((UltAl193:0.04439
,(UltB4063:0.04105,(UltB4062:0.02589,UltB4064:0.02243)0.778.119:0.00937)0
.787.116:0.00578)0.173.12:0.00855,(UltAl194:0.02519,(UltB4040:0.04482,(Ul
tAl191:0.00537,UltB4085:0.0135)0.974.88:0.04015)0.792.120:0.01928)0.831.1
06:5.4E-
4)0.861.168:0.01901,(UltB4071:0.05109,(RdrTruep:0.08305,(RspNavar:0.00876
,((RspGoens:0.01137,RspVisak:5.5E-4)0.806.82:5.3E-
4,(UltRho40:0.01144,RspMarin:0.01519)0.436.17:0.0037)0.763.134:0.00622)0.
933.166:0.0435)0.833.120:0.03929)0.835.110:0.0222)0.865.149:0.01859)0.098
.4:5.4E-
4,((UltGa284:0.09458,((((UltMagne:0.00369,ProteLS2:0.00382)0.987.148:5.4E
-4,((MagGryph:5.4E-4,AqsPolym:0.02326)0.992.131:5.4E-

4,((DcrSpeci:0.0,UltB3919:0.0):5.5E-
4,UltB3920:0.00739)0.577.12:0.00748)0.771.140:0.00364)0.926.175:0.02575,(
UltAl145:0.06834,(((MagMagne:0.0,MagSpec2:0.0):5.4E-
4,MagSpeci:0.00365)0.810.93:0.00727,(PhmChand:0.00753,PhmFulvu:0.02378)0.
932.156:0.01685)0.727.47:0.01236)0.429.18:0.01764)0.875.171:0.02261,((((U
ltB3923:0.02079,(UltB3922:0.02422,((UltRho41:0.00807,Otu01994:0.05368)0.9
67.104:0.03378,(TmtSiber:0.01814,Otu00934:0.02513)0.743.97:0.013)0.122.6:
9.0E-
4)0.830.99:0.00848)0.944.129:0.02401,UltMagn2:0.01016)0.378.20:0.00717,Ul
tB3921:0.02788)0.951.145:0.02659,UltB3918:0.02808)0.870.137:0.02032)0.971
.104:0.04499)0.870.138:0.02151,((((TspProfu:0.0,TspTepid:0.0):5.5E-
4,TspSpec2:0.00742)0.971.105:0.01501,(BactX301:5.4E-
4,(TspLucen:0.00369,(UltB3913:0.00741,AphPro52:0.00371)0.417.23:5.4E-
4)0.853.122:0.0037)0.895.162:5.5E-
4)1.000.1038:0.10099,UltB3959:0.06049)0.394.22:0.00809)0.854.137:0.01502)
0.894.141:0.02507)0.845.144:0.01184,AphPro54:0.02485)0.846.144:0.01613,((
(UltB4079:0.03312,(UltB4076:0.03756,(UltB4075:0.04423,(DflVanus:0.07114,U
ltB4074:0.02119)0.617.15:0.00685)0.790.114:0.01415)0.949.135:0.03022)0.90
9.137:0.02408,UltB4080:0.03688)0.624.16:0.01145,AphPro56:0.03798)0.769.11
7:0.00932)0.913.164:0.02103)0.855.150:0.01472,(Ult16590:0.10148,UltAl192:
0.05955)0.902.150:0.03335)0.889.153:0.01386)0.818.75:0.0075,((UltRho44:0.
00962,Ult27549:0.12066)0.968.119:0.03233,RvbSalin:0.0713)0.662.22:0.00151
)0.827.108:0.00953)0.968.120:0.0334)0.938.108:0.02496)0.826.96:0.01551,Ul
tB3834:0.15874)0.778.120:0.0109)0.962.137:0.02676,(((RctEndos:0.00826,((R
ctMonta:0.0037,(RctAustr:5.5E-4,RctSpeci:5.5E-4)0.904.162:5.5E-
4)0.625.15:5.5E-
4,RctSpec2:0.01504)0.757.95:0.00293)0.999.217:0.07423,(RctEndo2:0.01406,(
OrnTsuts:0.00393,OrnTsut2:0.00351)0.912.177:0.01695)0.947.126:0.02604)0.9
49.136:0.02709,((UltB3948:0.16182,UltB3957:5.5E-
4)0.998.196:0.07218,(((CanXenoh:0.16881,(UltB3956:0.03402,UltB3955:0.1069
2)0.537.17:0.01701)0.781.118:0.01173,((UltAnap4:0.04305,((CanNeoeh:0.0345
5,(UltAnap3:0.04111,(AnpMargi:0.02347,(AnpPhago:0.00318,AnpBovis:0.01569)
0.510.12:5.5E-
4)0.945.115:0.02966)0.847.154:0.01814)0.769.118:0.01603,(EhlRumin:0.00595
,((EhlEwing:0.0,EhlSpeci:0.0):5.4E-4,(UltEhrli:0.00371,(EhlCanis:5.5E-
4,(EhlChaff:0.0037,EhlMuris:0.0037)0.814.85:0.0037)0.797.100:0.00371)0.82
0.88:0.00371)0.770.137:0.00525)0.946.129:0.03224)0.791.106:0.01459)0.849.
132:0.03038,((WolEndos:0.01919,(IncSymbi:5.5E-
4,(((EnmbnO27:0.00741,(WolPipi2:5.5E-
4,(UltB3950:0.00741,(WolSpec2:0.09529,(WolSpeci:0.00371,WolSpec3:0.00375)
0.995.150:5.4E-4)0.898.162:0.00747)0.640.18:5.5E-4)0.636.13:5.5E-
4)0.842.134:0.00373,WolEndo3:5.4E-4)0.991.129:0.02324,IncSymb2:5.4E-
4)0.944.130:0.0151)0.876.131:5.4E-
4)0.999.218:0.00248,(((WolPipie:0.01125,((WolEndo2:0.03088,Otu00088:0.003
68)0.960.126:5.5E-4,UltB3949:5.5E-
4)0.888.162:0.00744)0.770.138:0.00399,WolSymbi:0.01103)0.664.22:0.00746,(
UltWolba:0.00748,UltWolb2:0.01133)1.000.1039:5.3E-
4)0.998.197:0.0011)0.977.107:0.04732)0.374.23:0.01731)0.894.142:0.02482,(
(UltrSo51:0.03017,UltAl175:0.1188)0.896.144:0.03429,UltB3985:0.16723)0.10
8.11:5.5E-
4)0.705.28:0.00766)0.430.22:0.00683)0.986.116:0.02774)0.497.11:5.3E-
4)0.257.8:5.4E-
4)0.192.13:0.01911)0.085.7:0.01573,((UltB4595:0.12545,(UltB3931:0.0607,(U
ltMorit:0.06489,UltB3932:0.0408)0.820.89:0.03608)0.773.133:0.01822)0.952.

140:0.04522,((MaiMeta9:0.12306,(UltDe113:0.07156,(UltMar39:0.02552,UltCandi:0.04128)0.753.104:0.03355)0.891.122:0.03385)0.950.125:0.06931,(UltB3910:0.08143,(UltAl128:0.02739,UltGa283:0.05517)0.983.108:0.064)0.905.153:0.04174)0.220.15:0.03269)0.255.4:0.0111)0.879.165:0.01512,(UltB3947:0.07369,(UltAl149:0.12712,(Sguffff:0.03164,UltAl148:0.1041)0.992.132:0.08678)0.932.157:0.04681)0.945.116:0.04956)0.821.90:0.01984)0.000.1001:5.5E-4)0.489.18:0.01273)0.937.137:0.04727)0.381.22:0.01567)0.728.53:0.0202,(Ult23518:0.0857,(Ult11299:0.09007,Ult11318:0.05556)0.986.117:0.06015)0.931.154:5.4E-4)0.837.108:0.01338)0.645.20:0.00384)0.777.126:0.00661)0.927.142:0.01306)0.781.119:0.00506)0.762.122:0.00522)0.780.144:0.00458,((((((Ult18103:0.03155,(Ult18105:0.01114,Ult19673:5.5E-4)0.773.134:0.00469)0.781.120:0.00459,(Ult18538:0.00721,(Ult18537:0.01117,Ult21338:0.0037)0.889.154:5.5E-4)0.970.128:0.02222)0.068.11:0.00333,(SrbOlear:0.03979,(CsrXyla3:5.5E-4,(UltLac31:0.01131,CsrJejue:5.5E-4)0.865.150:0.00369)0.763.135:0.0032)0.911.178:0.01385)0.752.95:0.01005,Ult18102:5.4E-4)0.989.109:0.01903,(Ult17545:5.4E-4,Ult17544:0.00369)0.981.115:0.00179)0.975.108:0.002,(Ult17546:0.0618,UltRu373:0.02592)0.883.157:0.01285)0.796.83:0.0037)0.879.166:0.00907)0.119.8:5.4E-4)0.767.111:5.4E-4)0.671.16:0.00471)0.750.84:0.00475)0.907.163:0.00196,Ult19417:0.01087)0.754.99:0.00197,(((Ult17871:0.0,Ult17872:0.0,Ult17873:0.0):5.5E-4,Ult23635:0.01887)0.985.124:0.0189,((((Ult19592:0.00369,(Ult17874:0.00369,Ult18696:0.01122)0.807.81:5.5E-4)0.775.121:5.5E-4,(Ult17842:0.00741,Ult17870:0.00369)0.927.143:5.5E-4)0.000.1002:5.5E-4,(((Ult17853:0.00743,(Ult17845:0.00716,Ult17852:0.0187)0.916.167:5.5E-4)0.912.178:5.5E-4,(Ult17867:0.0,Ult17869:0.0,Ult17844:0.0,Ult17846:0.0,Ult17847:0.0,Ult17848:0.0,Ult17851:0.0,Ult17875:0.0):5.5E-4)0.000.1003:5.5E-4,(Ult17868:0.0,Ult17849:0.0):0.00369)0.000.1004:5.5E-4)0.916.168:5.5E-4,Ult17850:0.01866)0.094.8:5.4E-4)0.915.149:0.00739)0.998.198:0.00219)0.584.17:9.7E-4,(((((Ult19643:0.01496,(Ult19642:5.4E-4,(Ult19489:0.00348,Ult24020:0.02739)0.955.117:0.01535)0.817.84:0.00367)0.746.80:5.5E-4,((Ult17782:0.01505,(Ult17783:0.01132,(((Ult17773:0.03054,Ult17779:0.00749)0.328.19:0.00368,((Ult17791:0.02285,(Ult17788:0.00365,Ult17790:0.00369)0.638.18:5.4E-4)0.679.27:0.00365,(Ult17774:5.5E-4,((Ult20203:0.00246,(Ult17777:5.5E-4,Ult18898:0.01504)0.926.176:0.00245)0.926.177:0.00246,HumanGu6:0.00369)0.698.34:5.5E-4)0.997.152:5.5E-4)0.865.151:0.00352)1.000.1040:5.4E-4,((Ult17785:0.00209,Ult20267:0.00693)0.916.169:0.00206,(((Ult17792:0.00369,Ult21297:0.01496)0.760.118:5.5E-4,UltLach6:0.00369)0.929.145:5.5E-4,(Ult17640:0.0,Ult17771:0.0,Ult17772:0.0,Ult17770:0.0,Ult17778:0.0,Ult17780:0.0,Ult17781:0.0,Ult17784:0.0,Ult17786:0.0,Ult17787:0.0,Ult17789:0.0,Ult17793:0.0,Ult20210:0.0,Ult22377:0.0):5.5E-4)0.869.127:5.5E-4)0.848.123:5.4E-4)0.827.109:0.00367)0.899.151:5.4E-4)0.869.128:0.00716,(((Ult17618:0.0,Ult20095:0.0):5.5E-4,Ult24104:0.00369)0.902.151:0.00764,(Ult17609:5.4E-4,(UltRu295:0.01433,(Ult17855:0.00369,Ult19438:5.5E-4)0.880.159:0.00993)0.919.185:0.01417)0.801.101:0.00755)0.880.160:0.00757)0.767.112:0.00357)0.762.123:0.00352,(((Ult19241:5.5E-4,(Ult19252:0.00209,(Ult19240:5.5E-

4,Ult20929:0.00741)0.914.170:0.00207)0.914.171:0.00694)0.973.102:0.01553,
(Ult17615:0.01166,(Ult17613:0.0,Ult17614:0.0):0.01152)0.851.146:0.00749)0
.872.155:0.00772,(((((UltRu293:0.00324,(RmnBact2:0.02675,UdnRum28:0.01242
)0.988.128:0.03282)0.836.102:0.01027,(Ult17753:5.5E-
4,Ult17754:0.01116)0.972.124:0.02741)0.803.85:0.01457,(Ult17828:0.00143,(
(((Ult17529:0.0,Ult17825:0.0,Ult20971:0.0):5.5E-
4,Ult17824:0.00369)0.981.116:0.01932,(Ult18039:5.5E-
4,Ult18038:0.00368)0.792.121:0.00375)0.801.102:0.00748,((Ult18028:0.00372
,(Ult18113:0.00372,Ult18114:5.5E-4)0.791.107:0.00371)0.854.138:5.5E-
4,(Ult18111:0.0,Ult18112:0.0):5.4E-4)0.965.118:5.4E-
4)0.872.156:0.01045)0.894.143:0.01515)0.061.7:5.3E-
4,(Ult17908:0.02247,(Ult17800:0.02253,Ult17799:5.5E-4)0.175.8:5.5E-
4)0.833.121:0.00698)0.924.152:0.014,Ult17819:0.01435)0.755.98:0.00522)0.7
71.141:0.00371)0.816.92:0.00358,(((Ult19650:0.0037,((CsrHathe:0.0,Ult1965
1:0.0,BacNL253:0.0):5.5E-
4,(BacNL238:0.01107,((BacNL225:0.00366,(BacNL241:0.0,BacNL245:0.0):5.3E-
4)0.876.132:0.00367,(BacNL226:5.5E-
4,(BacNL234:0.00368,((BacNL228:0.0,HumanG10:0.0,Ult19649:0.0,BacNL221:0.0
,BacNL224:0.0,BacNL233:0.0,BacNL237:0.0,BacNL250:0.0,Ult21330:0.0):5.5E-
4,((Ult19648:0.00369,(((BacNL235:5.5E-
4,BacNL247:0.00729)0.864.144:0.00361,((BacNL244:5.5E-4,(((BacNL248:5.5E-
4,(BacNL249:5.5E-4,(BacNL242:5.5E-4,BacNL243:5.5E-4)0.653.21:5.5E-
4)0.054.4:5.4E-4)0.942.145:5.5E-4,(BacNL236:5.4E-
4,BacNL240:0.00358)0.931.155:0.00721)0.820.90:0.00356,((BacNL230:5.5E-
4,((((BacNL227:0.00731,((BacNL222:5.5E-4,BacNL223:5.5E-4)0.522.8:5.3E-
4,BacNL231:0.00734)0.262.13:5.5E-4)0.858.142:0.00427,BacNL246:5.5E-
4)0.273.16:5.4E-4,BacNL229:5.5E-4)0.923.199:0.00421,BacNL232:5.5E-
4)0.322.15:5.4E-4)0.350.14:5.4E-
4,BacNL251:0.01454)0.851.147:0.00416)0.512.8:5.4E-4)0.227.10:5.5E-
4,BacNL252:5.5E-4)0.384.18:5.5E-4)0.443.16:5.4E-
4,Ult19817:0.01121)0.362.15:5.5E-4)0.000.1005:5.5E-4,BacNL239:5.5E-
4)0.135.12:5.5E-4)0.329.14:5.5E-4)0.218.12:5.5E-4)0.786.112:5.5E-
4)0.882.169:5.3E-4)0.876.133:0.00815)0.085.8:5.4E-
4)0.964.142:0.01265,Ult17876:5.4E-
4)0.394.23:0.00412,(((Ult17836:0.01027,(Ult17807:0.01569,((Ult17835:0.014
93,(Ult17834:0.0,Ult18400:0.0):5.5E-4)0.884.149:5.4E-
4,((Ult17830:0.00368,Ult17831:0.00737)0.800.89:0.00367,(Ult21333:0.00369,
(Ult17829:0.0,Ult17832:0.0,Ult17833:0.0):5.5E-4)0.134.15:5.5E-
4)0.887.155:0.0072)0.271.11:0.00374)0.813.91:0.00211)0.903.135:0.01039,((
Ult17705:0.00721,(Ult17706:5.5E-4,Ult19644:5.5E-4)0.918.174:5.5E-
4)0.906.168:0.01121,(Ult17707:0.00747,(Ult17627:0.00369,(Ult17625:0.00369
,Ult17626:5.5E-4)0.400.19:5.5E-
4)0.964.143:0.01928)0.761.104:0.00778)0.745.104:0.00418)0.857.147:0.00714
,(Ult17628:5.4E-
4,((((Ult18403:0.00405,(Ult18321:0.02759,UltRu294:0.01934)0.829.120:0.007
41)0.871.153:0.00722,((Ult18404:0.00369,Ult22393:5.5E-
4)0.776.123:0.00363,(((Ult18402:0.0,Ult22397:0.0):5.5E-
4,Ult22396:0.02287)0.898.163:0.0076,Ult17812:0.01176)0.756.119:0.00355)0.
906.169:0.0077)0.578.11:5.5E-4,Ult18405:5.4E-
4)0.908.169:0.0074,((Ult19637:0.00179,((((UltClo60:0.01213,(UncUn132:0.00
716,UncUn133:5.5E-
4)0.975.109:0.02286)0.800.90:0.00819,(UltClo54:0.01841,Ult21477:0.04153)0
.880.161:0.01532)0.893.164:0.01283,Ult19760:0.00758)0.867.149:0.00764,(((
((((Ult19640:0.00628,((Ult18104:0.0,UltClos6:0.0):0.00842,Ult19484:0.0143

8)0.543.17:0.00782)0.892.144:0.00455,(Ult17769:0.00377,((Ult17983:0.00366
,(((Ult17995:0.00383,((Ult17734:0.00747,Ult18007:0.00752)0.884.150:0.0073
7,(((Ult17993:0.0,Ult17997:0.0,Ult18003:0.0):5.5E-
4,((Ult17987:0.02677,Ult17992:0.01474)0.890.158:5.5E-
4,Ult17985:0.00369)0.435.19:5.5E-4)0.171.13:5.5E-
4,(Ult17998:0.00742,Ult17991:0.00745)0.483.21:5.5E-4)0.908.170:5.5E-
4)0.494.14:0.00374)0.867.150:0.00738,(Ult17978:0.00196,Ult17980:0.05955)1
.000.1041:0.00177)0.127.5:5.5E-
4,(Ult17976:0.0,Ult17977:0.0,Ult17982:0.0,Ult17981:0.0,Ult17989:0.0):5.5E
-4)0.550.14:5.5E-4)0.904.163:0.00739,(Ult17827:0.0,Ult17826:0.0):5.4E-
4)0.491.16:0.00757)0.897.150:0.01117)0.990.127:5.4E-
4,(Ult17621:0.00744,Pshggg38:0.00742)0.823.101:5.5E-
4)0.590.15:0.00371,(Ult19669:5.3E-4,Ult19633:0.00743)0.809.100:5.4E-
4)0.952.141:0.00196,((Ult19639:5.5E-
4,(Ult17620:0.00369,Ult21447:0.00369)0.646.18:5.5E-4)0.942.146:5.2E-
4,((Ult19638:0.0,Ult21023:0.0):5.5E-
4,Ult21329:0.04644)0.830.100:0.00363)0.961.129:0.00187)0.948.157:0.01472,
((Ult19635:0.0,Ult19636:0.0,UltLac16:0.0):5.5E-
4,Ult20931:0.01225)0.931.156:5.3E-
4)0.943.138:0.00751,((Ult18345:0.0099,Ult20098:0.02264)0.779.115:0.00557,
((Ult19221:0.02233,(Ult19330:0.01147,Ult19331:0.00932)0.878.149:0.01792)0
.736.62:0.00477,(Ult19818:0.00369,((Ult19596:0.02649,Ult19599:0.01098)0.8
69.129:5.5E-
4,(Ult19597:0.0,Ult19598:0.0,Ult19600:0.0,Ult19601:0.0,Ult19602:0.0,Ult19
603:0.0,Ult22674:0.0):5.5E-4)0.254.11:5.5E-4)0.999.219:5.4E-
4)0.786.113:0.00348)0.858.143:0.00742)0.951.146:5.4E-
4)0.885.151:0.00766)0.781.121:0.00247,Ult19641:5.4E-
4)0.909.138:0.00793)0.295.23:5.4E-4)0.536.18:5.5E-
4)0.836.103:0.00384)0.791.108:5.4E-
4)0.863.160:0.00716,((((Ult18954:0.02475,((Ult18952:5.4E-
4,(UltRu339:0.00596,Ult18953:0.00975)0.784.122:0.02311)0.861.169:0.00727,
UltRu338:0.00755)0.931.157:0.01551)0.862.152:0.00813,Ult17809:5.4E-
4)0.912.179:0.00778,Ult17808:5.4E-
4)0.846.145:0.00753,(((Ult17708:0.01518,Ult17709:5.4E-
4)0.625.16:0.00751,((UltLach4:0.00369,(Ult19672:0.01891,UltLach5:5.1E-
4)0.845.145:0.00369)0.677.25:5.4E-
4,(UltB7197:0.01439,(Ult17610:0.00369,Ult17611:5.5E-
4)0.733.58:0.0053)0.961.130:0.01863)0.900.137:0.00747)0.863.161:5.4E-
4,(Ult18412:0.0048,(Ult17612:0.01425,(Ult21283:5.5E-
4,Ult21284:0.00369)0.891.123:0.00882)0.881.157:0.01543)0.768.106:0.0061)0
.751.85:0.00757)0.917.143:0.01249)0.542.16:0.00357)0.726.42:0.00291)0.932
.158:0.0134)0.592.16:0.0013);
