# Supplementary material for: Differential Ecological Specificity of Protist and Bacterial Microbiomes across a Set of Termite Species
Source: Front Microbiol. 2017 Dec 19;8:2518. doi: 10.3389/fmicb.2017.02518 (PMC5742190; doi:10.3389/fmicb.2017.02518)
Supplement: Supplementary file 6 [file DataSheet6.PDF]

**MOTHUR command line for the analysis of the bacterial (16S rRNA gene sequences) dataset.**

mothur > make.contigs(file=stability.files,processors=12)
mothur > summary.seqs(fasta=current)
mothur > screen.seqs(fasta=current, group=current,maxambig=0,maxlength=275)
mothur > unique.seqs(fasta=current)
mothur > count.seqs(name=current,group=current)
mothur > summary.seqs(count=current,fasta=current)
mothur > align.seqs(fasta=current,reference=silva.v4.fasta)
mothur > summary.seqs(fasta=current,count=current)
mothur >
screen.seqs(fasta=current,count=current,summary=current,start=1968,end=11550,maxhomop=8)
mothur > summary.seqs(fasta=current,count=current)
mothur > filter.seqs(fasta=current,vertical=T,trump=.)
mothur > unique.seqs(fasta=current,count=current)
mothur > pre.cluster(fasta=current,count=current,diffs=2)
mothur > chimera.uchime(fasta=current,count=current,dereplicate=T)
mothur > remove.seqs(fasta=current,accnos=current)
mothur > summary.seqs(fasta=current,count=current)
mothur >
classify.seqs(fasta=current,count=current,reference=DictDb_v3.fasta,taxonomy=DictDb_v3.taxonomy,cutoff=80)
mothur > remove.lineage(fasta=current,count=current,taxonomy=current,taxon=Chloroplast-Mitochondria-unknown-Archaea-Eukaryota)
mothur > summary.seqs(fasta=current,count=current)
sub.sample(fasta=current,count=current,taxonomy=current,size=32824,persample=T)

Subsampled files were renamed after subsampling to not confuse them with previous files:
fasta-file: subsample32824.fasta
count-file: subsample32824.count_table
taxonomy-file: subsample32824.taxonomy

mothur >
cluster.split(fasta=subsample32824.fasta,count=subsample32824.fasta,taxonomy=subsample32824.taxonomy,splitmethod=classify,taxlevel=4,cutoff=0.15)
mothur > make.shared(list=current,count=current,label=0.03)
mothur > classify.otu(list=current,count=current,taxonomy=current,label=0.03)

Rarefaction Curves:

mothur >
rarefaction.single(shared=subsample32824.an.unique_list.shared,freq=1,label=0.03,calc=sobs,groupmode=F)

Weighted UniFrac Distance metric and community – distance based trees in the R-package pvclust:

./FastTree -nt -fastest subsample32824.fasta >Otuput.txt

mothur > unifrac.weighted(tree=Output.txt,count=subsample32824.count_table,distance=square)

In R:

```
read.table("Output.txt1.weighted.phylip.dist",header=TRUE)->data
pvclust(data,nboot=10000)
```

**MOTHUR command line for the analysis of the parabasalid (18S rRNA gene sequences) dataset.**

Note that the fastq – files were converted to fasta – files, trimmed from 250 bp to a length of 210 bp and then converted back to fastq – files for a proper alignment before proceeding:

[mothur > fastq.info(fastq=18S_Cd1_CTCGACTTATCT_CGTTACTA_L001_R1_001.fastq) . . . continue for all reads of each sample
mothur >
trim.seqs(fasta=18S_Cd1_CTCGACTTATCT_CGTTACTA_L001_R1_001.fasta,qfile=18S_Cd1_CTCGACTTATCT_CGTTACTA_L001_R2_001.qual,keepfirst=210,processors=12) … continue for all reads of each sample
mothur >
make.fastq(fasta=18S_Cd1_CTCGACTTATCT_CGTTACTA_L001_R1_001.trim.fasta,qfile=D1R1.trim.qual) … continue for all reads of each sample]

mothur > make.contigs(file=stability.files,processors=12)
mothur > summary.seqs(fasta=current,processors=12)
mothur > screen.seqs(fasta=current,group=current,maxambig=0,maxlength=240)
mothur > unique.seqs(fasta=current)
mothur > count.seqs(name=current,group=current)
mothur > summary.seqs(fasta=current,count=current)
mothur > align.seqs(fasta=current,reference=S12Alignment.fasta)
mothur > summary.seqs(fasta=current,count=current)
mothur >
screen.seqs(fasta=current,count=current,summary=current,start=132,end=494,maxhomop=8)
mothur > filter.seqs(fasta=current,vertical=T,trump=.)
mothur > unique.seqs(fasta=current,count=current)
mothur > summary.seqs(fasta=current,count=current)
mothur > pre.cluster(fasta=current,count=current,diffs=2)
mothur > chimera.uchime(fasta=current,count=current,dereplicate=T)
mothur > remove.seqs(fasta=current,accnos=current)
mothur > summary.seqs(fasta=current,count=current)
mothur >
classify.seqs(fasta=current,count=current,reference=S12Alignment.fasta,taxonomy=S12Taxonomy.taxonomy,cutoff=80)
mothur > remove.lineage(fasta=current,count=current,taxonomy=current,taxon=Chloroplast-Mitochondria-unknown-Archaea-Bacteria)
mothur > summary.seqs(fasta=current,count=current)
sub.sample(fasta=current,count=current,taxonomy=current,size=22825,persample=TRUE)

Subsampled files were renamed after subsampling to not confuse them with previous files:
fasta-file: subsample22825.fasta
count-file: subsample22825.count_table
taxonomy-file: subsample22825.taxonomy

mothur >
cluster.split(fasta=subsample22825.fasta,count=subsample22825.count_table,taxonomy=subsample22825.taxonomy,splitmethod=classify,taxlevel=4,cutoff=0.15)
mothur > make.shared(list=current,count=current,label=0.03)
mothur > classify.otu(count=current,taxonomy=current,label=0.03)

To generate the species-stackplot (Figure 3A) the step classify.seqs was modified, allowing for a cutoff of 60%:

mothur > classify.seqs(fasta=current,count=current,reference=S11Sequences.fas,taxonomy=S11Taxonomy.taxonomy,cutoff=60)

All previous commands were used as described above.

Rarefaction Curves:

mothur > rarefaction.single(shared=subsample22825.an.unique_list.shared,freq=1,label=0.03,calc=sobs,groupmode=F)

Weighted UniFrac Distance metric and community – distance based trees in the R-package pvclust:

./FastTree -nt -fastest subsample22825.fasta >Otuput.txt

mothur > unifrac.weighted(tree=Output.txt,count=subsample22825.count_table,distance=square)

In R:

read.table("Output.txt1.weighted.phylip.dist",header=TRUE)->data
pvclust(data,nboot=10000)

**MOTHUR command line for the analysis of the bacterial (16S rRNA gene sequences) dataset, including the outgroup *C. punctulatus*.**

Data from Dietrich et al. 2014 were downloaded from NCBI using the fastq-dump command, implemented in the SRA toolkit and converted to fasta files using the fastq.info command in MOTHUR. A group file for these sequences was created using the make.groups command in MOTHUR. The two datasets were merged after application of unique.seqs. Commands different from the protocol above are listed below.

Changes before merging the two datasets (command adapted for the sequences of Dietrich et al. 2014):
mothur > screen.seqs(fasta=stability.trim.contigs.fasta,group=stability.contigs.groups,maxambig=0,maxlength=475,minlength=200)

After merging the datasets and aligning to the silva.bacteria reference database the sequences were trimmed to get the overlap of our sequences and the ones from Dietrich et al 2014.:

mothur > chop.seqs(fasta=Merged_stability.trim.contigs.good.unique.align,countgaps=TRUE,numbases=23444)

mothur >
chop.seqs(fasta=Merged_stability.trim.contigs.good.unique.chop.fasta,countgaps=TRUE,numbases
=9575,keep=back)

mothur > screen.seqs(fasta=current,count=Merged_stability.trim.contigs.good.count_table,sum-
mary=current,start=1,end=9574,maxhomop=8)

The remove.groups command was used to isolate the data from *C. punctulaus*. Additionally, we removed the outliers Cd2, Rg2, Rg4 and Ps9 from the dataset. This command was used before the cluster.split.

mothur > remove.groups(fasta=Merged_stability.trim.contigs.good.unique.chop.chop.good.filter.u-
nique.precluster.pick.pick.fasta,count=Merged_stability.trim.contigs.good.unique.chop.chop.good.-
filter.unique.precluster.uchime.pick.pick.count_table,groups=16S_DK-16S_FK-16S_GK-
16S_PCR_NC-16S_PK-16S_SK-Adomesticus-Ameridionlis-Atrestus-Borientalis-Cniger-Cugaden-
sis-16S_G2-16S_G4-16S_P9-16S_D2-DPunctata-ECapucina-EChopardi-EFloridana-EPosticus-
GAssmilis-HMossambicus-HSjoestedti-IMarginipennis-MDawiniensis-MSubhyalinus-Macroter-
mesSp-MicrocerotermesSp-NCorniger-NJouteli-NTakasagoensis-OOrientalis.fatsa-Odontoter-
mesSp-OhpiotermesSp-PAngustipennis-PEphippiata-PanchloraSp-RMaderae-RSantonensis-
SEsakii-SLampyridiformis-SLateralis-SMacroptera-TrinervitermesSp-ZNevadensis)

The software package FastTree was used to generate a tree from the resulting fasta file, so it could be used as input for the weighted Unifrac analysis in Mothur.

./FastTree -nt
Merged_stability.trim.contigs.good.unique.chop.chop.good.filter.unique.precluster.pick.pick.pick.fa
sta >Output.txt

Note that we adjusted execution of the unifrac.weighted command by using the “subsample” and “iters” options to perform 1000 sequence resamplings for generation of unifrac distances.

mothur >
unifrac.weighted(tree=Output.txt,count=subsample3537.count_table,subsample,subsample=3537,ite
rs=1000,distance=square)

In R:

```
read.table("Output.txt1.weighted.ave.dist",header=TRUE)->data
clust <- pvclust(data,nboot=10000)
plot(clust,cex=0.8)
pdf(“Output.pdf”)
dev.off()
```
